# Idebenone-Activating Autophagic Degradation of *α*-Synuclein via Inhibition of AKT-mTOR Pathway in a SH-SY5Y-A53T Model of Parkinson's Disease: A Network Pharmacological Approach

**DOI:** 10.1155/2021/8548380

**Published:** 2021-09-16

**Authors:** Pei Kun He, Yu Yuan Gao, Feng-Juan Lyu, Jia Ning Chen, Yu Hu Zhang, Kun Nie, Qing Xi Zhang, Rui Huang, Qing Rui Duan, Man Li Guo, Zhi Hua Liu, He Ling Huang, Gui Xian Ma, Li Juan Wang, Li Min Wang

**Affiliations:** ^1^School of Medicine, South China University of Technology, Guangzhou 510006, China; ^2^Department of Neurology, Guangdong Provincial People's Hospital, Guangdong Academy of Medical Sciences, Guangdong Neuroscience Institute, No. 106 Zhongshan Second Road, Guangzhou 510080, China

## Abstract

**Background:**

Parkinson's disease (PD) is the second most common neurodegenerative disease worldwide, which currently lacks disease-modifying therapy to slow down its progression. Idebenone, a coenzyme Q10 (CQ10) analogue, is a well-known antioxidant and has been used to treat neurological disorders. However, the mechanism of Idebenone on PD has not been fully elucidated. This study aims to predict the potential targets of Idebenone and explore its therapeutic mechanism against PD.

**Method:**

We obtained potential therapeutic targets through database prediction, followed by Gene Ontology and Kyoto Encyclopedia of Genes and Genomes pathway enrichment analysis. Next, we constructed and analyzed a protein-protein interaction network (PPI) and a drug-target-pathway-disease network. A molecular docking test was conducted to identify the interactions between Idebenone and potential targets. Lastly, a PD cell line of SH-SY5Y overexpressing mutant *α*-synuclein was used to validate the molecular mechanism.

**Result:**

A total of 87 targets were identified based on network pharmacology. The enrichment analysis highlighted manipulation of MAP kinase activity and the PI3K-AKT signaling pathway as potential pharmacological targets for Idebenone against PD. Additionally, molecular docking showed that AKT and MAPK could bind tightly with Idebenone. In the cell model of PD, Idebenone activated autophagy and promoted *α*-synuclein degradation by suppressing the AKT/mTOR pathway. Pretreating cells with chloroquine (CQ) to block autophagic flux could diminish the pharmacological effect of Idebenone to clear *α*-synuclein.

**Conclusion:**

This study demonstrated that Idebenone exerts its anti-PD effects by enhancing autophagy and clearance of *α*-synuclein, thus providing a theoretical and experimental basis for Idebenone therapy against PD.

## 1. Introduction

Parkinson's disease (PD) is the second most common neurodegenerative disease, whose prevalence is growing faster than any other neurological disorder [1]. At present, pharmacological administration is still considered to be the major treatment modality, including levodopa preparation, dopamine agonists, and monoamine oxidase-B inhibitors [2]. Though these drugs can alleviate the symptoms of PD, none of them has proven effective in slowing the progress of PD in clinical practice [3]. Besides, symptomatic therapy not only has adverse reactions, such as impulse control disorders, hallucinations, and motor complications, but also has therapeutic effects of the fade with the duration of disease [4]. As a result, it is imperative to develop disease-modifying drug.

Idebenone, a coenzyme Q10 (CQ10) analogue with better pharmacological properties, contains a redox-active moiety, and thus it is able to transport electrons to protect cells against oxidative damage and help to preserve mitochondrial respiratory capacity [5]. Nevertheless, Idebenone may not act as a direct antioxidant according to its pharmacological kinetics and instead exerts cytoprotective effects via numerous physiological mechanisms, including inhibition of P52Shc, increasing the expression of Lin28A, and regulation of AKT [6]. Idebenone has been used to treat numerous neurological disorders, including Alzheimer's disease [7], Friedreich ataxia [8], and dementia [9]. As for Parkinson's disease, Idebenone could alleviate lipid peroxidation in a Parkinson's disease model induced by rotenone [10]. Furthermore, Idebenone has been shown to promote M2 macrophage polarization and acted as an anti-inflammatory agent in vivo and in vitro [11]. However, the pharmacological mechanism of Idebenone in PD remains unclear.

Misfolded *α*-synuclein is a major constituent of abnormal neuronal aggregates, known as Lewy bodies (LBs), which are a pathological hallmark of PD. In addition, the neurotoxic effect of *α*-synuclein has been suggested to play a central role in the pathogenesis of PD [12]. Therefore, therapeutic strategies targeting *α*-synucelin protein continue to gain attention [13, 14]. Macroautophagy (also named autophagy), characterized by autophagosomes with a bilayer membrane and the formation of autolysosomes [15], have been reported to take part in the clearance of *α*-synuclein [16, 17]. Numerous studies have demonstrated that activation of autophagy can decrease the content of *α*-synuclein protein and exert cytoprotective effects [18–22]. Idebenone was reported to regulate AKT [23], an upstream activator of the mechanistic target of rapamycin kinase (mTOR), and may further modulate autophagy. However, there has been little focus on the autophagy-associated effects of Idebenone.

In this study, we investigated the underlying therapeutic mechanism of Idebenone on PD using network pharmacology, a novel approach integrating network biology and polypharmacology, to further our understanding of the mechanisms of Idebenone treatment [24, 25]. Then, we conducted molecular experiments to verify the selected pharmacological target in an SH-SY5Y cell line overexpressing the *α*-synuclein A53T mutant (SH-SY5Y-A53T). We hypothesized that Idebenone could target AKT and decrease *α*-synuclein levels by enhancing autophagy through inhibition of the AKT/mTOR pathway. Our study provides new insight into the use of Idebenone as promising treatment option for patients with PD.

## 2. Materials and Methods

### 2.1. Screening Potential Targets for Idebenone against Parkinson's Disease

We obtained the chemical structure of Idebenone from ChemSpider (http://www.chemspider.com/) and then searched for its reported pharmacological targets through Swiss Target Prediction (http://www.swisstargetprediction.ch/) [26]. For disease-related genes, we conducted searches in various databases, including Online Mendelian Inheritance in Man (OMIN, https://omim.org/), DisGeNET (https://www.disgenet.org/) [27], and GeneCards v5.0 (https://www.genecards.org/), using the search terms “Parkinson's disease” or “Parkinson disease”. The potential target genes were predicted by an intersection between the primary targets of Idebenone and PD, which were presented by Venn diagram (http://bioinformatics.psb.ugent.be/webtools/Venn/).

### 2.2. Gene Ontology and Kyoto Encyclopedia of Genes and Genomes Enrichment Analysis

To further illustrate the biological processes of the targets systematically, we conducted Gene Ontology (GO) and Kyoto Encyclopedia of Genes and Genomes (KEGG) pathway enrichment analysis. We applied the R package, clusterProfiler [28], to conduct GO analysis with a *p* value less than 0.05. Meanwhile, the KEGG analysis was carried out using the online tool, Metascape (https://metascape.org/gp/index.html/) [29]. The top 20 terms identified from above analyses were visualized through R package, barplot. Further, we visualized and analyzed the pathways using R package of pathview [30]. The drug-target-pathway network was constructed in Cytoscape 3.7.2 [31].

### 2.3. Protein-Protein Interaction Network Construction and Analysis

We submitted the identified targets to the STRING database v11.0 (https://string-db.org/) [32] to obtain protein-protein interaction information. The confidence score was set to 0.7 or higher. We uploaded interaction data to Cytoscape 3.7.2 and calculated the topology score of the nodes. The genes whose degree value was twofold greater than the average degree score were regarded as hub genes.

### 2.4. Molecular Docking

We searched for X-ray crystal structures in the protein data bank (PDB, http://www.rcsb.org/) and saved it in the PDB file format. Then we removed water and small molecules in PyMOL (version 2.2, https://pymol.org/2/). We used Autodock v4.2.6 (http://autodock.scripps.edu/) to process the protein receptor files and ligand files, including adding hydrogens atoms, calculating Gasteiger charges, assigning AD4 type atoms, spreading charges deficit over all residue atoms, and setting rotatable bonds, and then converted them to a PDBQT format. We set a grid box to cover the active site and the molecular docking between Idebenone and hub genes using AutoDock's genetic algorithm. We chose the binding mode with lowest binding energy and visualized them with a 2D diagram using the Molecular Operating Environment (MOE, version 2010.12) software. In general, the binding energy ≤−5 kcal/mol indicates good binding activity [33].

### 2.5. Establishment of SH-SY5Y-A53T and Cell Culture

We purchased the SH-SY5Y cell line from the American type culture collection (ATCC, CRL-2266, USA). The cell line was transfected by lentivirus particles stably overexpressing SNCA (A53T) after puromycin-based screening. In the *α*-synuclein A53T mutant gene, the amino acid at position 53 was converted from alanine (A) to threonine (T) as a result of a missense mutation at position 209 from G to A [34]. Cells were cultured in Dulbecco's modified Eagle's medium (DMEM, GIBCO) containing 10% fetal bovine serum (FBS, GIBCO, Australia).

### 2.6. Administration of Drugs

Idebenone was purchased from a commercial reagent company (RHAWN, 58186-27-9), in the form of powder, stored at 4°C, and dissolved in DMSO at a storage concentration of 20 mM and stored at −20°C. The drug was diluted to the working concentration with fresh medium, and the drug was freshly prepared before each experiment.

### 2.7. Cell Viability Assay

Cell growth was detected by the Cell Counting kit 8 (CCK8, DojinDo, CK04, Japan). Cells were seeded in a 96-well plate with density reaching at least 2 × 104 cells per well. The cells were treated with Idebenone at different concentrations for 18 h. Then, 10 *μ*l of the CCK8 reagents was added to each well and the plate was incubated for 2 h at 37°C. The spectrophotometric absorbance of each sample was measured at 450 nm using a microplate reader (TECAN, INFINITE M PLEX). The cell viability was expressed as the percentage of the control group.

### 2.8. Regulation of Autophagy

In order to verify the autophagy-enhanced effects of Idebenone, chloroquine (CQ, MCE, HY-17583A) was used to inhibit autophagy as previously described [18].

### 2.9. Western Blotting Assay

Cells were lysed in ice-cold RIPA buffer (Beyotime, China) for 1 h, and then the lysates were centrifuged at 14000 × *g* for 10 min. The supernatant was collected, and the protein concentration was determined using a BCA assay (Beyotime, China). Protein lysates were loaded onto a 6–15% gel for sodium dodecyl sulfate-poly-acrylamide gel electrophoresis (SDS-PAGE) and transferred to polyvinylidene fluoride (PVDF) membranes (Millipore, USA). The membranes were blocked with 5% nonfat milk for 2 h at room temperature and incubated with the following primary antibodies overnight at 4°C: rabbit anti-*α*-synuclein (Abcam, ab138501), anti-*β*-actin (Cell Signaling Technology, 4970), SQSTM1/p62 (Abcam, 56416), AKT (Cell Signaling Technology, 4691), Phospho-AKT (Ser473, Cell Signaling Technology, 4060), mTOR (Affinity, AF6308), Phospho-mTOR (Ser2448, Affinity, AF3308), and mouse-anti-LC-3B (Cell Signaling Technology, 83506). The following day, the membranes were washed with 0.1% TBST for 15 minutes and incubated with HRP-conjugated secondary antibodies at room temperature for 1 h, including goat anti-rabbit IgG (Bioss, bs-0295G) and goat anti-mouse IgG (Bioss, bs-40296G). After that, the incubated membranes were washed three times with TBST buffer. Finally, the membranes were imaged with an enhanced chemiluminescence (ECL) detection reagent and captured by Bio-Rad ChemiDoc MP system. The bands were quantified by ImageJ software. All experiments were repeated in triplicate.

### 2.10. Statistical Analysis

GraphPad Prism 8.0 was used to perform statistical analysis. All data were expressed as mean ± standard deviation (SD). Student's *t*-test was used to compare two independent groups, while one-way analysis of variance (ANOVA) followed by Tukey's post hoc test was applied to compare multiple groups. A *p* value < 0.05 was considered to be statistically significant.

## 3. Results

### 3.1. Identification of Intersection Target Genes

**Figure 1:**
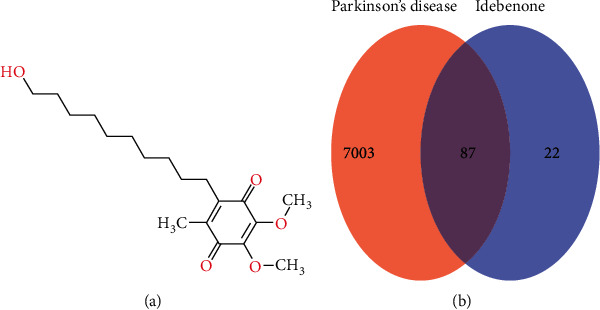
(a) Molecular structure of Idebenone. (b) Venn diagram of common targets of Idebenone for Parkinson's disease.

The structure of Idebenone is shown in Figure 1(a). A total of 109 predicted targets of Idebenone were obtained from the Swiss Target Prediction database (Table S1). A total of 7090 PD-relevant targets were identified using OMIN, DisGeNET, and GeneCards after deduplication (Table S2). A Venn diagram showing the common 87 targets between PD and Idebenone is shown in Figure 1(b), and the details of the 87 genes are presented in Supplementary Table S3.

### 3.2. GO and KEGG Pathway Enrichment Analysis

**Figure 2:**
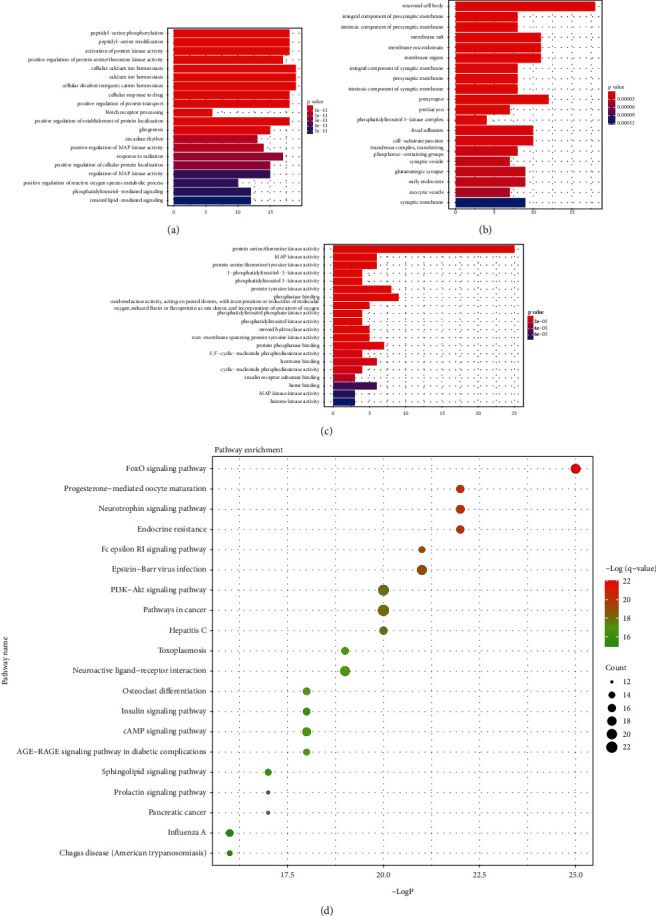
Enrichment analysis of potential targets of Idebenone for Parkinson's disease. (a) Top 20 Gene Ontology biological processes. (b) Top 20 Gene Ontology cellular components. (c) Top 20 Gene Ontology molecular functions. (d) Top 20 Kyoto Encyclopedia of Genes and Genomes (KEGG) pathways. The size of the bubble represents the number of targets in the pathway and the color represents the *q* value. The red represents a smaller *q* value.

To identify the function of the potential targets, we performed GO and KEGG pathway enrichment analysis of the 87 target genes. The GO analysis included biological process (BP), cellular component (CC), and molecular function (MF). The top 20 most significant terms are shown in Figures 2(a)–2(c) (Table S4). The main biological process included peptidyl-serine phosphorylation, positive regulation of protein serine/threonine kinase activity, calcium ion homeostasis, positive regulation of MAP kinase, and positive regulation of reactive oxygen species metabolic process. The cellular components involved include neuronal cell body, integral component of presynaptic membrane, membrane raft, presynaptic membrane, and perikaryon. The main molecular functions included protein serine/threonine kinase activity, MAP kinase activity, phosphatidylinositol 3-kinase activity, protein phosphatase binding, and insulin receptor substrate binding.

The top 20 KEGG pathways with the lowest *p* values are shown in the bubble gram (Figure 2(c), Table S4). The PD-relevant pathways involved were the FOXO signaling pathway, neurotrophin signaling pathway, PI3K-AKT signaling pathway, neuroactive ligand-receptor interaction, insulin signaling pathway, cAMP signaling pathway, and sphingolipid signaling pathway.

### 3.3. Protein-Protein Interaction Network Construction and Analysis

**Figure 3:**
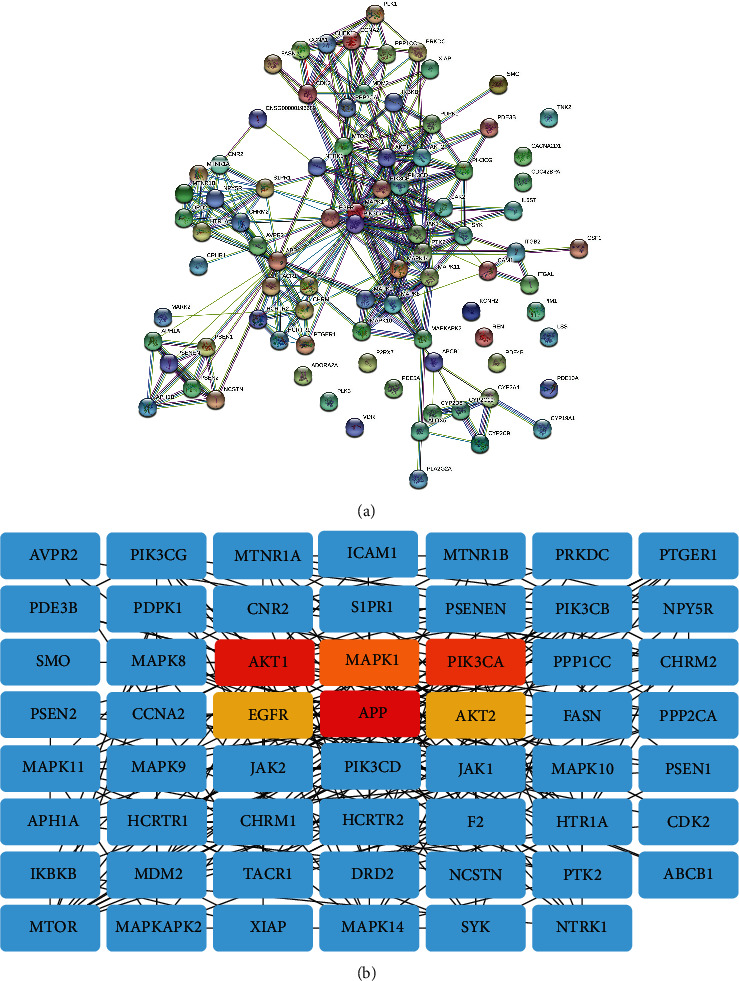
Network pharmacology analysis of the putative targets of Idebenone against Parkinson's disease. (a) The protein-protein interaction (PPI) network of the candidate protein targets of Idebenone. The nodes represent different proteins and the edges represent the association between them. The thickness of the interconnection between nodes indicates the strength of the evidence. (b) The hub genes (AKT1, MAPK1, PIK3CA, EGFR, APP, and AKT2) with a high degree value were screened from the PPI network analysis.

The 87 potential targets were uploaded to the PPI database to determine the interactions between them. After removing the free nodes, the PPI network consisted of 55 nodes and 281 edges. Thereafter, we identified 6 core genes according to the degree value, including APP, AKT1, PIK3CA, MAPK1, EGFR, and AKT2 (Figures 3(a)-3(b)). Details about the topological properties of the related genes are presented in Table S5.

### 3.4. Idebenone-Target-Pathway-PD Network Construction

**Figure 4:**
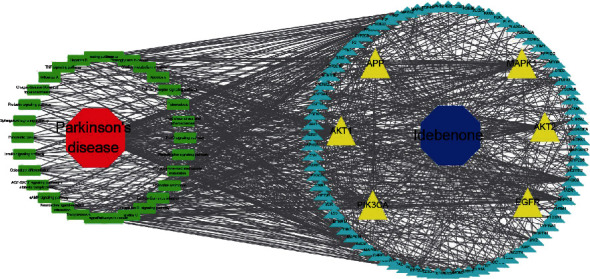
The drug-target-pathway-disease network diagram of Idebenone. The triangle nodes in yellow represent the hub genes while the rest represent other putative genes. The green rectangle nodes represent the top 20 related pathways in the KEGG enrichment analysis.

To comprehensively elucidate the underlying mechanism of Idebenone treatment in PD, we constructed an Idebenone-target-pathway-PD network using the Cytoscape software. The core genes are highlighted in yellow, while the pathways are highlighted in green (Figure 4).

### 3.5. Molecular Docking

**Figure 5:**
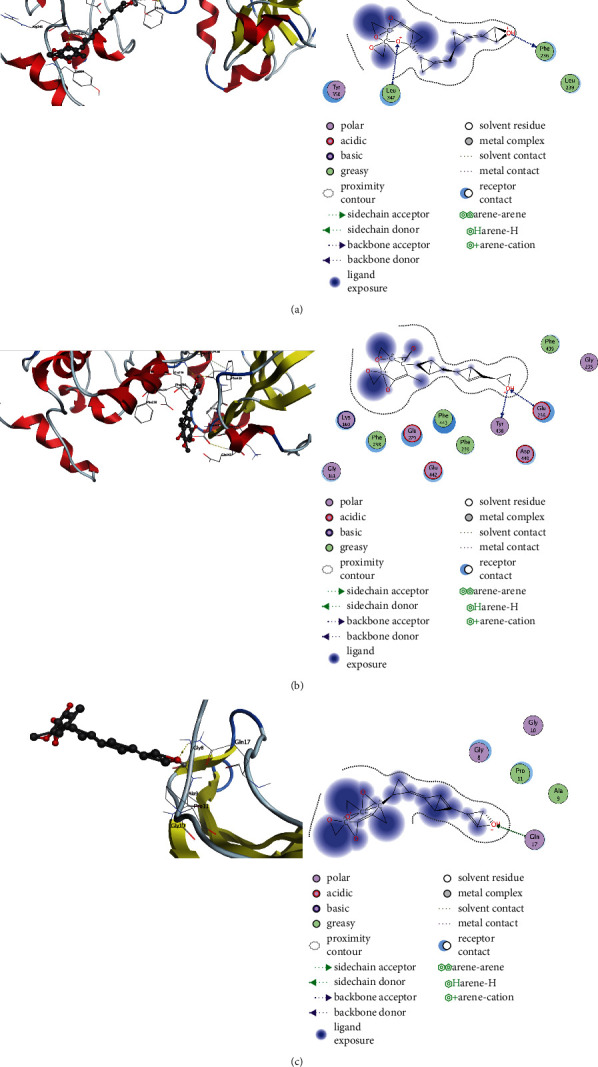
Molecular docking of Idebenone with AKT1, AKT2, and MAPK1.

Molecular docking was performed to investigate the interaction and potential binding mode between Idebenone and AKT1, AKT2, and MAPK1. These targets were chosen not only because they were hub genes in the PPI network, but also because they play an important role in autophagy-related pathway. The PDB ID of AKT1, AKT2, MAPK1 and their binding energy with Idebenone are shown in Table 1. We observed that Idebenone can form two hydrogen bonds with Phe236 and Leu347 of AKT1 (Figure 5(a)). Similarly, the docking results identified hydrogen bonds between Idebenone and Glu236 and Tyr438 of AKT2 (Figure 5(b)). Moreover, a hydrogen bond was formed between Idebenone with Gln17 in MAPK1 (Figure 5(c)).

### 3.6. Idebenone May Decrease Expression Level of *α*-Synuclein via Enhancing Autophagy

**Figure 6:**
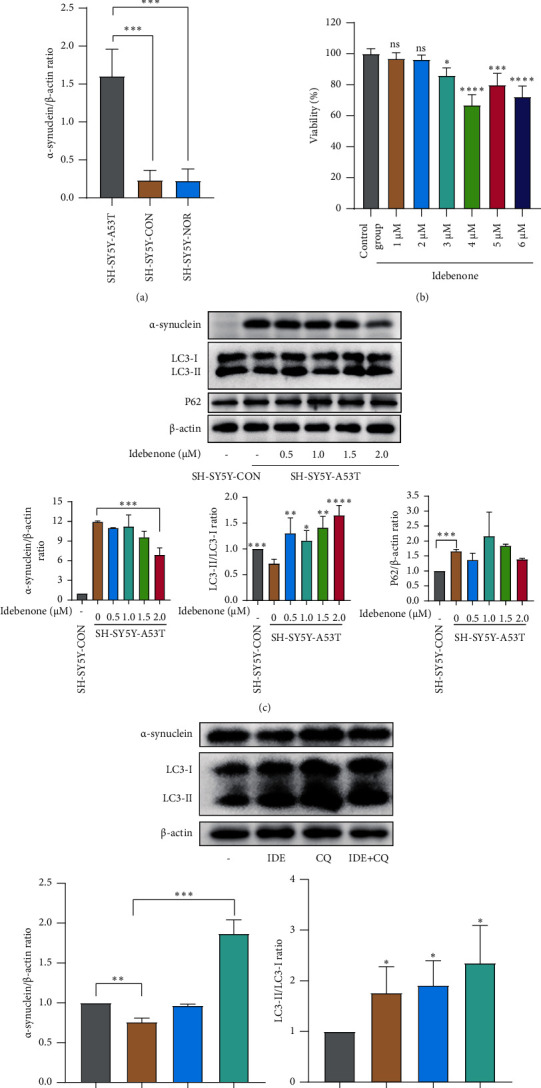
Effect of Idebenone on *α*-synuclein degradation and activation of autophagy in the SH-SY5Y-A53T PD cell line. (a) Western blot images of *α*-synuclein levels after lentiviral transfection; ^∗∗∗^*p* < 0.001. (b) Histogram of SH-SY5Y-A53T cell viability at different concentrations of Idebenone; ns, not significant, ^*∗*^*p* < 0.05, ^∗∗∗^*p* < 0.001, and ^∗∗∗∗^*p* < 0.0001. (c) Western blot images of *α*-synuclein accumulation, LC3-II, and p62 after Idebenone administration at concentrations ranging from 0 *μ*M to 2.0 *μ*M, and histograms of the relative protein expression of *α*-synuclein, LC3-II, and p62 in each group vs. “SH-SY5Y-A53T cells without Idebenone treatment,” ^*∗*^*p* < 0.05, ^∗∗^*p* < 0.01, ^∗∗∗^*p* < 0.001, and ^∗∗∗∗^*p* < 0.0001. (d) Western blot images of *α*-synuclein accumulation, LC3-II in each group, and histograms of the relative protein expression of *α*-synuclein and LC3-II in each group vs. “the control group without any treatment,” ^*∗*^*p* < 0.05. SH-SY5Y-A53T: SH-SY5Y cells overexpressing A53T mutant *α*-synuclein; SH-SY5Y-CON: SH-SY5Y cells transfected with a negative control plasmid; SH-SY5Y-NOR: SH-SY5Y cells without transfection; IDE: Idebenone.

First, we verified that the SH-SY5Y cell line overexpressed the A53T (SH-SY5Y-A53T) mutant strain using Western blot analysis (Figure 6(a)). Then, to explore the optimal dose of Idebenone administration without the risk of significant toxicity, SH-SY5Y-A53T cells were treated with Idebenone at different concentrations ranging from 1 *μ*M to 6 *μ*M for 18 h. As shown in Figure 6(b), cell viability began to decrease when the concentration reached 3 *μ*M. We then explored the dose-dependent effect of Idebenone on *α*-synuclein levels at concentrations below 3 *μ*M (0.5 *μ*M, 1.0 *μ*M, 1.5 *μ*M, and 2.0 *μ*M) and found that the inhibitory effect on the expression of *α*-synuclein was stable and highly significant at an Idebenone concentration of 2.0 *μ*M. To determine whether the clearance of *α*-synuclein was related to autophagy, the related indicators of LC3, p62 were detected. The results indicated that LC3-II increased at Idebenone concentrations ranging from 0.5 *μ*M to 2.0 *μ*M, while p62 did not change (Figure 6(c)).

To further confirm that *α*-synuclein was cleared by Idebenone-induced autophagy, 10 *μ*M CQ was first used to pretreat SH-SY5Y-A53T cells for 24 h, after which Idebenone was added to a final concentration of 2 *μ*M. CQ could inhibit the fusion of autophagosomes and lysosomes, thus leading to the accumulation of autophagosomes and interrupting autophagy flux. As shown in Figure 6(d), after pretreatment with both CQ and Idebenone, the level of *α*-synuclein increased significantly compared to pretreatment with Idebenone only, suggesting that CQ could eliminate the clearance effect of *α*-synuclein by Idebenone.

### 3.7. Idebenone May Activate Autophagy through Inhibiting AKT-mTOR Pathway

**Figure 7:**
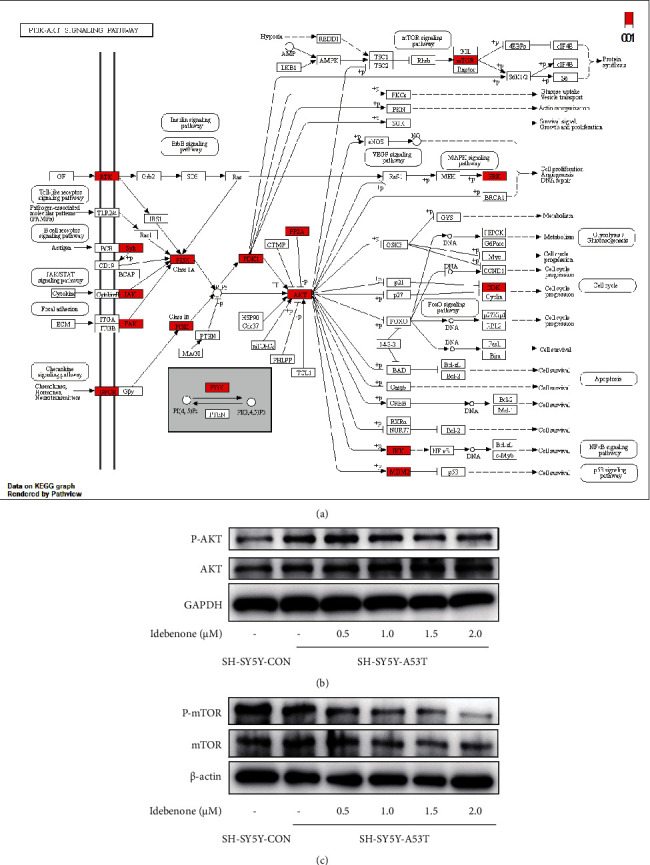
The effect of Idebenone on the AKT-mTOR pathway. (a) PI3K-AKT signaling pathway extracted from the KEGG analysis (ID: hsa04151). The potential targets and genes implicated in the pathway are shown in red. (b, c) Western blot images of the levels of p-AKT and AKT and and p-mTOR and mTOR, respectively.

Based on the previous pathway enrichment analysis and docking results, PI3K-AKT pathway was considered to be a potential mechanism for explaining the autophagy-enhancing effect of Idebenone. The PI3K-AKT pathway and its related genes are presented on Figure 7(a), which was extracted from the KEGG analysis. The western blot results showed the inhibiting effects of Idebenone on both AKT and mTOR (Figure 7(b)).

## 4. Discussion

Recently, it has been proposed that Idebenone exerts cytoprotective effects by activating fundamental pathways, rather than by functioning as a direct antioxidant agent, thus providing the possibility of discovering a new mode of action [6]. Previous studies have reported that Idebenone could improve Parkinsonian symptoms, but the underlying mechanism remains unclear. In this study, we explored the potential mechanism of Idebenone treatment by means of network pharmacology. Moreover, the activation of autophagy and promotion of *α*-synuclein degradation may contribute to the therapeutic effect of Idebenone against PD.

We obtained 87 PD-relevant targets through database prediction, among which APP, AKT1, PIK3CA, MAPK1, EGFR and AKT2 were regarded as hub genes in the PPI network analysis. After that, we conducted molecular docking and the results suggested that MAPK1 and AKT1 exhibit stable binding modes with Idebenone. MAPK, including the c-Jun N-terminal kinase (JNK), extracellular signal-regulated kinase 1 and 2 (ERK1/2), and p38, plays an important role in the neuroinflammation process, and an excessive inflammation response confers detrimental effects and contributes to neurodegeneration [35, 36]. Yan et al. demonstrated that Idebenone could suppress the phosphorylation of ERK, p38, and JNK, therefore inhibiting M1 microglial phenotype and enhanced M2 polarization [11]. Consistent with these findings, our GO clustering analysis also supported the regulation of MAP kinase activity as one of the important functions of Idebenone against PD. However, further studies are needed to investigate the precise anti-inflammatory effects of Idebenone in PD. AKT, also referred to as protein kinase B (PKB), has a serine/threonine-specific kinase domain and is involved in many psychological processes, including cell survival, metabolism, and protein synthesis [37]. A growing body of evidence has reported defective AKT kinase activity in PD [38]. Luo et al. revealed that AKT could directly bind to the antioxidative enzyme, quinone oxidoreductase (NQO1), which further phosphorylated NQO1 and promoted its polyubiquitination and degradation, decreasing the ability of cells to counteract oxidative stress in PD [39]. Tomilov et al. [23] suggested that Idebenone functions as an insulin sensitizer, preventing the binding of the p52Shc protein to the insulin receptor, promoting AKT phosphorylation, and therefore activating the insulin signaling pathway. In addition, AKT seems to be a meeting point of diabetes and PD. The results of the KEGG clustering analysis in this study indicated that the insulin signaling pathway may be a potential mechanism contributing to the function of Idebenone against PD. Indeed, insulin resistance is an increasingly relevant risk factor of PD [40], and administration of insulin has been reported to alleviate cognitive impairment in a 6-OHDA-induced PD model through reducing the levels of p-AKT and p-GSK3*β* [41].

To determine whether Idebenone exerts its protective effects in PD in vitro, we treated SH-SY5Y-A53T cells with Idebenone. Given that Tai et al. [42] reported that Idebenone could induce apoptosis of SH-SYTY cells, the dose of administered Idebenone had to be effective without the risk of toxicity. In this study, the level of *α*-synuclein decreased significantly at an Idebenone concentration of 2 *μ*M. Based on network pharmacology results, Idebenone can bind tightly with MAPK and AKT, which are both related to autophagy. Activating autophagy has been a therapeutic strategy to attenuate *α*-synuclein toxicity in PD pathogenesis. Thus, we postulated that autophagy may contribute to the clearance of *α*-synuclein. In this regard, we detected autophagy-relevant proteins and the results revealed that the levels of the autophagosome marker LC3-II increased after treatment, suggesting the activation of autophagy. To further verify whether autophagy was necessary for the therapeutic effect of Idebenone, we blocked autophagic flux using CQ and found that the clearance of *α*-synuclein was diminished.

Notably, one of the most characterized substrates of selective autophagy, p62 (also known as SQSTM1), did not change after drug treatment. On the one hand, autophagy is not the only way to degrade p62 [43, 44], while on the other hand, p62 takes part in numerous psychological processes, such as nutrient sensing, inflammation, apoptosis, and antioxidant response. As such, the level of p62 may be regulated by a number of other confounding factors [45, 46]. Therefore, p62 may be a less reliable marker in detecting autophagy.

In order to further understand the biological mechanisms of Idebenone therapy in PD, we referred to the results of the KEGG pathway enrichment analysis and postulated that the AKT/mTOR pathway may contribute to the activation of autophagy. Our experimental results suggested that Idebenone could suppress AKT phosphorylation (Ser473) and mTOR phosphorylation (Ser2448) in a dose-dependent manner. This appears to contradict the results of the study by Tomilov et al. [23], which suggested that Idebenone has an enhancing effect on AKT. In fact, in the study of Tomilov et al. [23], Idebenone upregulated p-AKT in the presence of insulin at concentrations of Idebenone below 5 *μ*M, while insulin independently enhanced p-AKT levels at Idebenone concentrations higher than 5 *μ*M. For reference, the concentration of Idebenone applied in our study ranged from 0.5 *μ*M to 2.0 *μ*M. To our knowledge, Idebenone may play a bilateral role on AKT activation depending on the drug concentration and pathological conditions. To some extent, Idebenone likely acts as a “regulator” to rescue cells from a pathological state.

There are several limitations in this study. Firstly, in the network pharmacology analysis, the potential targets from the database prediction are based on established knowledge, which restricts the power to identify novel target. Thus, we call for further exploration with the application of sequencing technology. Secondly, although we demonstrated that Idebenone can decrease *α*-synuclein levels in the SH-SY5Y-A53T cell line, whether administration of Idebenone can reach the optical concentration in the blood in the central nervous system in vivo and exert its autophagy-enhancing effects remains unclear. Lastly, more experiments are needed to verify other predicted outcomes, including the potential targets and pathways.

## 5. Conclusion

In summary, we applied network pharmacology to identify 87 related targets of Idebenone against PD. Further analysis revealed that MAPK1 and AKT1 could bind tightly with Idebenone. In addition, in vitro experiments showed that Idebenone may reduce the level of *α*-synuclein through the activation of autophagy. The underlying mechanism may relate to the suppression of the AKT/mTOR pathway. Our work provides new insights on the therapeutic mechanism of Idebenone treatment against PD.

## Figures and Tables

**Table 1:** The autodocking details of Idebenone binding to AKT1, AKT2, and MAPK1.

Drug	Targets	PDB ID	Binding energy (kcal·mol^−1^)
Idebenone	AKT1	4gv1	−5.23
	AKT2	3d0e	−4.67
	MAPK1	4n0s	−5.14

## Data Availability

The datasets supporting the conclusions of this study are included within this article.

## Supplementary Materials

Supplementary Materials Table S1: predicted targets of Idebenone. Table S2: targets associated with Parkinson's disease. Table S3: putative targets of Idebenone against PD. Table S4: the results of the GO and KEGG pathway enrichment. Table S5: the topological properties of potential targets in PPI network ().
