# Supplementary material for: Idebenone-Activating Autophagic Degradation of α-Synuclein via Inhibition of AKT-mTOR Pathway in a SH-SY5Y-A53T Model of Parkinson's Disease: A Network Pharmacological Approach
Source: Evid Based Complement Alternat Med. 2021 Sep 16;2021:8548380. doi: 10.1155/2021/8548380 (PMC8463184; doi:10.1155/2021/8548380)
Supplement: Supplementary Materials — Table S1: predicted targets of Idebenone. Table S2: targets associated with Parkinson's disease. Table S3: putative targets of Idebenone against PD. Table S4: the results of the GO and KEGG pathway enrichment. Table S5: the topological properties of potential targets in PPI network (). [file 8548380.f1.pdf]

Table. S1 Predicted targets of Idebenone

Targets predicted by Swiss Target Prediciton

**Target**	**Common name**	**Uniprot ID**	**ChEMBL ID**
Cytochrome P450 2D6	CYP2D6	P10635	CHEMBL289
Cytochrome P450 2C9	CYP2C9	P11712	CHEMBL3397
Cytochrome P450 3A4	CYP3A4	P08684	CHEMBL340
Cytochrome P450 2C19	CYP2C19	P33261	CHEMBL3622
Arachidonate 5-lipoxygenase	ALOX5	P09917	CHEMBL215
Macrophage-expressed gene 1 protein	MPEG1	Q2M385	CHEMBL341440
Phospholipase A2 group IIA	PLA2G2A	P14555	CHEMBL3474
Cytochrome P450 19A1	CYP19A1	P11511	CHEMBL1978
C-C chemokine receptor type 1	CCR1	P32246	CHEMBL2413
PI3-kinase p110-beta subunit	PIK3CB	P42338	CHEMBL3145
Inhibitor of apoptosis protein 3	XIAP	P98170	CHEMBL4198
PI3-kinase p110-delta subunit	PIK3CD	O00329	CHEMBL3130
PI3-kinase p110-gamma subunit	PIK3CG	P48736	CHEMBL3267
PI3-kinase p110-alpha subunit	PIK3CA	P42336	CHEMBL4005
MAP kinase p38 alpha	MAPK14	Q16539	CHEMBL260
Phosphodiesterase 2A	PDE2A	O00408	CHEMBL2652
Phosphodiesterase 4B	PDE4B	Q07343	CHEMBL275
Phosphodiesterase 10A (by homology)	PDE10A	Q9Y233	CHEMBL4409
Interleukin-6 receptor subunit beta	IL6ST	P40189	CHEMBL312473
Corticotropin releasing factor receptor 1	CRHR1	P34998	CHEMBL1800
Serine/threonine-protein kinase mTOR	MTOR	P42345	CHEMBL2842
p53-binding protein Mdm-2	MDM2	Q00987	CHEMBL5023
Ileal bile acid transporter	SLC10A2	Q12908	CHEMBL2778
Cyclin-dependent kinase 9	CDK9	P50750	CHEMBL3116
Vanilloid receptor	TRPV1	Q8NER1	CHEMBL4794
Dopamine D2 receptor (by homology)	DRD2	P14416	CHEMBL217
Cannabinoid receptor 2 (by homology)	CNR2	P34972	CHEMBL253
Neuropeptide Y receptor type 5	NPY5R	Q15761	CHEMBL4561
Thrombin	F2	P00734	CHEMBL204
c-Jun N-terminal kinase 1	MAPK8	P45983	CHEMBL2276
Beta amyloid A4 protein	APP	P05067	CHEMBL2487
Nerve growth factor receptor Trk-A	NTRK1	P04629	CHEMBL2815
Tyrosine-protein kinase JAK1	JAK1	P23458	CHEMBL2835
Tyrosine-protein kinase JAK2	JAK2	O60674	CHEMBL2971
Melatonin receptor 1A	MTNR1A	P48039	CHEMBL1945
Melatonin receptor 1B	MTNR1B	P49286	CHEMBL1946
Geranylgeranyl transferase type I	PGGT1B FNTA	P53609 P49	CHEMBL209516
c-Jun N-terminal kinase 2	MAPK9	P45984	CHEMBL4179
Sphingosine 1-phosphate receptor Edg-3	S1PR3	Q99500	CHEMBL3892
Sphingosine 1-phosphate receptor Edg-1	S1PR1	P21453	CHEMBL4333
Gamma-secretase	PSEN2 PSENEN	P49810 Q9N	CHEMBL209413
Smoothened homolog	SMO	Q99835	CHEMBL5971
Cyclin-dependent kinase 2	CDK2	P24941	CHEMBL301
MAP kinase ERK2	MAPK1	P28482	CHEMBL4040

Macrophage colony stimulating factor receptor	CSF1R	P07333	CHEMBL1844
Tyrosine-protein kinase SYK	SYK	P43405	CHEMBL2599
P-glycoprotein 1	ABCB1	P08183	CHEMBL4302
Prostanoid EP1 receptor	PTGER1	P34995	CHEMBL1811
Phosphodiesterase 3	PDE3A	Q14432	CHEMBL241
Adenosine A2a receptor	ADORA2A	P29274	CHEMBL251
Phosphodiesterase 3B	PDE3B	Q13370	CHEMBL290
HERG	KCNH2	Q12809	CHEMBL240
Vasopressin V2 receptor	AVPR2	P30518	CHEMBL1790
Vasopressin V1a receptor	AVPR1A	P37288	CHEMBL1889
Proto-oncogene tyrosine-protein kinase ROS	ROS1	P08922	CHEMBL5568
Geranylgeranyl transferase type I beta subunit	PGGT1B	P53609	CHEMBL4135
TGF-beta receptor type I	TGFBR1	P36897	CHEMBL4439
Lanosterol synthase	LSS	P48449	CHEMBL3593
Vitamin D receptor	VDR	P11473	CHEMBL1977
Muscarinic acetylcholine receptor M2	CHRM2	P08172	CHEMBL211
Muscarinic acetylcholine receptor M1	CHRM1	P11229	CHEMBL216
Epidermal growth factor receptor erbB1	EGFR	P00533	CHEMBL203
Cyclin-dependent kinase 2/cyclin A	CDK2 CCNA1 C(	P24941 P78	CHEMBL209412
Intercellular adhesion molecule (ICAM-1), Integrin	ITGAL ICAM1 IT	P20701 P05	CHEMBL209666
Subtilisin/kexin type 7	PCSK7	Q16549	CHEMBL2232
c-Jun N-terminal kinase 3	MAPK10	P53779	CHEMBL2637
DNA-dependent protein kinase	PRKDC	P78527	CHEMBL3142
Cyclin T1	CCNT1	O60563	CHEMBL2108
Thrombin and coagulation factor X	F10	P00742	CHEMBL244
Phosphodiesterase 7A	PDE7A	Q13946	CHEMBL3012
Monocarboxylate transporter 1	SLC16A1	P53985	CHEMBL4360
Inhibitor of nuclear factor kappa B kinase beta sub	IKBKB	O14920	CHEMBL1991
Serine/threonine-protein kinase PIM1	PIM1	P11309	CHEMBL2147
MAP kinase-activated protein kinase 2	MAPKAPK2	P49137	CHEMBL2208
Serine/threonine-protein kinase AKT2	AKT2	P31751	CHEMBL2431
Neurokinin 1 receptor	TACR1	P25103	CHEMBL249
3-phosphoinositide dependent protein kinase-1	PDPK1	O15530	CHEMBL2534
Serine/threonine-protein kinase PLK1	PLK1	P53350	CHEMBL3024
MAP/microtubule affinity-regulating kinase 2	MARK2	Q7KZI7	CHEMBL3831
MAP kinase p38 beta	MAPK11	Q15759	CHEMBL3961
Serine/threonine-protein kinase AKT	AKT1	P31749	CHEMBL4282
Serine/threonine-protein kinase MRCK-A	CDC42BPA	Q5VT25	CHEMBL4516
Serine/threonine-protein kinase Chk1	CHEK1	O14757	CHEMBL4630
Serine/threonine-protein kinase PLK3	PLK3	Q9H4B4	CHEMBL4897
Transient receptor potential cation channel subfami	TRPV4	Q9HBA0	CHEMBL3119
Interleukin-8 receptor A	CXCR1	P25024	CHEMBL4029
Serine/threonine protein phosphatase PP1-gamma c	PPP1CC	P36873	CHEMBL4438
Serine/threonine protein phosphatase 2A, catalytic	PPP2CA	P67775	CHEMBL4703
Serotonin 1a (5-HT1a) receptor	HTR1A	P08908	CHEMBL214
Focal adhesion kinase 1	PTK2	Q05397	CHEMBL2695
Renin	REN	P00797	CHEMBL286

Fatty acid synthase	FASN	P49327	CHEMBL4158
Orexin receptor 2	HCRTR2	O43614	CHEMBL4792
P2X purinoceptor 7	P2RX7	Q99572	CHEMBL4805
Orexin receptor 1	HCRTR1	O43613	CHEMBL5113
Proto-oncogene tyrosine-protein kinase MER	MERTK	Q12866	CHEMBL5331
Phosphodiesterase 5A	PDE5A	O76074	CHEMBL1827
Voltage-gated calcium channel alpha2/delta subuni	CACNA2D1	P54289	CHEMBL1919
Voltage-gated calcium channel alpha2/delta subuni	CACNA2D2	Q9NY47	CHEMBL3896
Tyrosine kinase non-receptor protein 2	TNK2	Q07912	CHEMBL4599

Target Class	Probability*	Known actives (3D/2D)
Cytochrome P450	1	2 / 1
Cytochrome P450	1	14 / 1
Cytochrome P450	1	13 / 1
Cytochrome P450	1	10 / 2
Oxidoreductase	0.298481093	98 / 43
Unclassified protein	0.18543728	0 / 15
Enzyme	0.120823672	17 / 1
Cytochrome P450	0.104671941	3 / 97
Family A G protein-coupled receptor	0.104671941	106 / 0
Enzyme	0.104671941	116 / 0
Other cytosolic protein	0.104671941	68 / 7
Enzyme	0.104671941	95 / 0
Enzyme	0.104671941	136 / 0
Enzyme	0.104671941	571 / 0
Kinase	0.104671941	338 / 0
Phosphodiesterase	0.104671941	54 / 0
Phosphodiesterase	0.104671941	130 / 0
Phosphodiesterase	0.104671941	344 / 0
Membrane receptor	0.104671941	2 / 0
Family B G protein-coupled receptor	0.104671941	98 / 0
Kinase	0.104671941	685 / 0
Other nuclear protein	0.104671941	191 / 0
Electrochemical transporter	0.104671941	36 / 0
Kinase	0.104671941	78 / 0
Voltage-gated ion channel	0.104671941	194 / 0
Family A G protein-coupled receptor	0.104671941	292 / 0
Family A G protein-coupled receptor	0.104671941	300 / 0
Family A G protein-coupled receptor	0.104671941	22 / 0
Protease	0.104671941	107 / 0
Kinase	0.104671941	186 / 0
Membrane receptor	0.104671941	34 / 0
Kinase	0.104671941	154 / 0
Kinase	0.104671941	127 / 0
Kinase	0.104671941	231 / 0
Family A G protein-coupled receptor	0.104671941	6 / 0
Family A G protein-coupled receptor	0.104671941	7 / 0
Enzyme	0.104671941	9 / 0
Kinase	0.104671941	89 / 0
Family A G protein-coupled receptor	0.104671941	64 / 0
Family A G protein-coupled receptor	0.104671941	82 / 0
Protease	0.104671941	83 / 0
Frizzled family G protein-coupled receptor	0.104671941	55 / 0
Kinase	0.104671941	108 / 0
Kinase	0.104671941	232 / 0

Kinase	0.104671941	104 / 0
Kinase	0.104671941	529 / 0
Primary active transporter	0.104671941	64 / 3
Family A G protein-coupled receptor	0.104671941	67 / 0
Phosphodiesterase	0.104671941	11 / 0
Family A G protein-coupled receptor	0.104671941	152 / 0
Phosphodiesterase	0.104671941	8 / 0
Voltage-gated ion channel	0.104671941	180 / 0
Family A G protein-coupled receptor	0.104671941	33 / 0
Family A G protein-coupled receptor	0.104671941	34 / 0
Kinase	0.104671941	23 / 0
Enzyme	0.104671941	1 / 0
Kinase	0.104671941	25 / 0
Enzyme	0.104671941	8 / 0
Nuclear receptor	0.104671941	93 / 0
Family A G protein-coupled receptor	0.104671941	32 / 0
Family A G protein-coupled receptor	0.104671941	60 / 0
Kinase	0.104671941	309 / 0
Other cytosolic protein	0.104671941	34 / 0
Membrane receptor	0.104671941	23 / 0
Protease	0.104671941	1 / 0
Kinase	0.104671941	109 / 0
Kinase	0.104671941	67 / 0
Other cytosolic protein	0.104671941	88 / 0
Protease	0.104671941	217 / 0
Phosphodiesterase	0.104671941	55 / 0
Electrochemical transporter	0.104671941	24 / 0
Kinase	0.104671941	53 / 0
Kinase	0.104671941	111 / 0
Kinase	0.104671941	40 / 0
Kinase	0.104671941	41 / 0
Family A G protein-coupled receptor	0.104671941	64 / 0
Kinase	0.104671941	69 / 0
Kinase	0.104671941	16 / 0
Kinase	0.104671941	3 / 0
Kinase	0.104671941	23 / 0
Kinase	0.104671941	107 / 0
Kinase	0.104671941	7 / 0
Kinase	0.104671941	126 / 0
Kinase	0.104671941	3 / 0
Voltage-gated ion channel	0.104671941	1 / 0
Family A G protein-coupled receptor	0.104671941	11 / 0
Phosphatase	0.104671941	1 / 0
Phosphatase	0.104671941	2 / 0
Family A G protein-coupled receptor	0.104671941	85 / 0
Kinase	0.104671941	18 / 0
Protease	0.104671941	591 / 0

Transferase	0.104671941	108 / 0
Family A G protein-coupled receptor	0.104671941	21 / 0
Ligand-gated ion channel	0.104671941	21 / 0
Family A G protein-coupled receptor	0.104671941	17 / 0
Kinase	0.104671941	61 / 0
Phosphodiesterase	0.104671941	133 / 0
Calcium channel auxiliary subunit alpha2delta far	0.104671941	2 / 0
Calcium channel auxiliary subunit alpha2delta far	0.104671941	2 / 0
Kinase	0.104671941	72 / 0

Table S2 Targets associtaetd with Parkinson's disease

Table S2-1 Targets obtained from DisGeNET

Score ⩾ 0.1

Gene	Gene_id	UniProt	ne_Full_Na	rotein_Cla	_diseases_	DSI_g	DPI_g	pLI	Score_gda
SNCA	6622	P37840	synuclein a	Transporte	449	0.427	0.885	0.8829	0.7
PARK7	11315	Q99497	Parkinsoni	Enzyme	161	0.535	0.808	0.75335	0.6
DDC	1644	P20711	dopa decarboxylase		164	0.535	0.769	3.75E-08	0.6
DRD2	1813	P14416	dopamine r	G-protein c	437	0.436	0.846	0.74709	0.6
ATP13A2	23400	Q9NQ11	ATPase cation transpo		160	0.566	0.577	8.96E-09	0.6
MAOB	4129	P27338	monoamine oxidase B		152	0.533	0.808	0.99903	0.6
PRKN	5071	O60260	parkin RBI	Enzyme	409	0.431	0.846	6.93E-07	0.6
PINK1	65018	Q9BXM7	PTEN indu	Kinase	209	0.516	0.769	1.87E-10	0.6
SLC18A2	6571	Q05940	solute carr	Transporte	150	0.554	0.769	0.075523	0.6
TH	7054	P07101	tyrosine hydroxylase		321	0.462	0.885	1.69E-06	0.6
DRD1	1812	P21728	dopamine r	G-protein c	181	0.529	0.808	0.9555	0.57
IGF1R	3480	P08069	insulin like	Kinase	556	0.399	0.885	0.96799	0.51
LRRK2	120892	Q5S007	leucine ricl	Kinase	231	0.51	0.808	2.58E-30	0.5
GAK	2580	O14976	cyclin G as	Kinase	13	0.805	0.231	0.005182	0.5
GBA	2629	P04062	glucosylceramidase be		319	0.5	0.808	1.44E-06	0.5
MAPT	4137	P10636	microtubule associated		469	0.446	0.923	0.006026	0.5
BST1	683	Q10588	bone marro	Enzyme	41	0.67	0.423	4.21E-11	0.5
HLA-DRA	3122	P01903	major histo	Immune re	92	0.581	0.769	0.51949	0.48
PARK16	100359403		Parkinson disease 16 (		5	0.861	0.077		0.4
DNM1L	10059	O00429	dynamin 1	Enzyme m	273	0.475	0.808	0.000568	0.4
PPARGC1	10891	Q9UBK2	PPARG co	Transcript	350	0.453	0.885	0.9973	0.4
CP	1356	P00450	ceruloplasr	Enzyme	283	0.466	0.846	4.2E-10	0.4
CYP2D6	1565	P10635	cytochrome P450 fam		432	0.437	0.885	1.08E-25	0.4
GDNF	2668	P39905	glial cell de	Signaling	409	0.434	0.885	0.2035	0.4
GFAP	2670	P14136	glial fibrillary acidic p		447	0.421	0.885	1.46E-06	0.4
TMEM23(	29058	Q96A57	transmembrane protei		15	0.769	0.115	0.000181	0.4
GSTM1	2944	P09488	glutathione S-transfera		627	0.38	0.923	0.002064	0.4
HFE	3077	Q30201	homeostatic iron regul		415	0.436	0.846	2.56E-08	0.4
HMOX1	3162	P09601	heme oxygenase 1		666	0.381	0.923	0.009682	0.4
HSPA9	3313	P38646	heat shock protein fan		198	0.502	0.885	0.96852	0.4
IL6	3569	P05231	interleukin 6		2367	0.248	0.962	0.31536	0.4
MAOA	4128	P21397	monoamine oxidase A		300	0.465	0.769	0.99928	0.4
MTHFR	4524	P42898	methylenetetrahydrofo		985	0.337	0.885	3.21E-10	0.4
NOS1	4842	P29475	nitric oxide synthase 1		521	0.408	0.885	1	0.4
ABCB1	5243	P08183	ATP bindi	Transporte	933	0.344	0.885	1.26E-05	0.4
VPS35	55737	Q96QK1	VPS35 ret	Transporte	66	0.656	0.5	0.96921	0.4
BDNF	627	P23560	brain deriv	Signaling	992	0.345	0.923	0.65626	0.4
SLC6A3	6531	Q01959	solute carr	Transporte	373	0.453	0.885	0.99758	0.4
SOD1	6647	P00441	superoxide	Enzyme	689	0.379	0.962	0.1773	0.4
SOD2	6648	P04179	superoxide	Enzyme	668	0.379	0.923	0.15499	0.4
TNF	7124	P01375	tumor necr	Signaling	2724	0.231	0.962	0.8033	0.4
GSTP1	2950	P09211	glutathione S-transfera		610	0.383	0.923	0.014155	0.38
DDIT4	54541	Q9NX09	DNA damage inducib		92	0.576	0.769	0.000683	0.38

CYP2E1	1571	P05181	cytochrome P450 fam		306	0.459	0.885	8.07E-09	0.37
MAP3K5	4217	Q99683	mitogen-ac	Kinase	170	0.513	0.808	0.51964	0.36
NGF	4803	P01138	nerve grow	Signaling	616	0.391	0.885	0.82026	0.36
NQO1	1728	P15559	NAD(P)H quinone del		368	0.434	0.885	1.19E-09	0.34
AIF1	199	P55008	allograft in	Calcium-b	166	0.521	0.808	1.88E-06	0.34
GSTA4	2941	O15217	glutathione S-transfera		40	0.678	0.462	0.010958	0.33
IGF2	3481	P01344	insulin like growth fac		604	0.39	0.885	0.044127	0.33
TRPM2	7226	O94759	transient re	Ion channe	133	0.546	0.731	8.96E-44	0.33
HLA-DRE	3127	Q30154	major histo	Immune re	62	0.608	0.731	3.64E-07	0.32
IGF2R	3482	P11717	insulin like	Receptor	166	0.518	0.731	1	0.32
INS	3630	P01308	insulin		405	0.445	0.923	0.30091	0.32
ENO2	2026	P09104	enolase 2	Enzyme	239	0.475	0.846	0.38396	0.31
FBP1	2203	P09467	fructose-bi	Enzyme	125	0.575	0.692	0.079124	0.31
FCER2	2208	P06734	Fc fragmen	Receptor	104	0.56	0.654	7.61E-12	0.31
GPX1	2876	P07203	glutathione	Enzyme	290	0.458	0.923	3.85E-05	0.31
HGF	3082	P14210	hepatocyte	Enzyme	671	0.374	0.885	0.99947	0.31
HSPA1A	3303	P0DMV8;	heat shock protein fan		229	0.486	0.769	0.030228	0.31
INSR	3643	P06213	insulin rece	Kinase	452	0.432	0.846	3.61E-05	0.31
MIR181C	406957		microRNA 181c		103	0.555	0.769		0.31
MAG	4099	P20916	myelin ass	Receptor	106	0.579	0.692	0.49228	0.31
MTA1	9112	Q13330	metastasis	Nucleic ac	118	0.547	0.692	1	0.31
BAG5	9529	Q9UL15	BAG cochaperone 5		11	0.769	0.346	1.81E-06	0.31
TCL1B	9623	O95988	T cell leukemia/lymph		100	0.561	0.5	0.010127	0.31
ADARB2	105	Q9NS39	adenosine	Enzyme	33	0.716	0.308	0.85603	0.3
COL19A1	1310	Q14993	collagen type XIX alp		4	0.89	0.154	4.84E-33	0.3
SLC2A14	144195	Q8TDB8	solute carr	Transporte	8	0.821	0.308	0.041173	0.3
EDN1	1906	P05305	endothelin	Signaling	679	0.38	0.846	0.45504	0.3
FGB	2244	P02675	fibrinogen	Signaling	95	0.584	0.692	0.56616	0.3
CNTNAP2	26047	Q9UHC6	contactin associated p		203	0.53	0.846	2.87E-09	0.3
GSK3B	2932	P49841	glycogen s	Kinase	393	0.43	0.846	0.95571	0.3
HBG1	3047	P69891	hemoglobin subunit ga		65	0.615	0.538	0.56611	0.3
HSPA4	3308	P34932	heat shock protein fan		550	0.394	0.923	0.99954	0.3
HSPA8	3312	P11142	heat shock protein fan		124	0.553	0.808	0.99863	0.3
IL1B	3553	P01584	interleukin 1 beta		1801	0.276	0.962	0.13005	0.3
DRAXIN	374946	Q8NBI3	dorsal inhibitory axon		1	1	0.038	1.89E-10	0.3
KCNJ4	3761	P48050	potassium	Ion channe	16	0.805	0.385	0.9649	0.3
MAP2	4133	P11137	microtubule associated		126	0.537	0.692	1	0.3
CEACAM	4680	P40199	CEA cell adhesion mo		110	0.561	0.731	1.19E-06	0.3
NR4A2	4929	P43354	nuclear rec	Nuclear re	166	0.529	0.846	0.99959	0.3
PITX3	5309	O75364	paired like homeodom		93	0.585	0.692	0.81276	0.3
NCAPG2	54892	Q86XI2	non-SMC condensin I		80	0.603	0.731	0.97764	0.3
SLC30A10	55532	Q6XR72	solute carr	Transporte	120	0.578	0.654	0.97952	0.3
NECTIN2	5819	Q92692	nectin cell adhesion m		106	0.575	0.769	0.96926	0.3
RPL6	6128	Q02878	ribosomal	Nucleic ac	27	0.7	0.385	0.99326	0.3
RPL23A	6147	P62750	ribosomal	Nucleic ac	11	0.78	0.308	0.90573	0.3
RPS8	6202	P62241	ribosomal	Nucleic ac	9	0.805	0.192	0.99003	0.3
TALDO1	6888	P37837	transaldolase 1		85	0.608	0.654	2.52E-06	0.3

TFAM	7019	Q00059	transcription factor A,		147	0.529	0.731	0.17259	0.3
RPL14	9045	P50914	ribosomal	Nucleic ac	22	0.722	0.308	0.89508	0.3
OPTN	10133	Q96CV9	optineurin		136	0.548	0.808	5.91E-18	0.26
SPR	6697	P35270	sepiapterin reductase		90	0.631	0.654	0.0434	0.26
TWNK	56652	Q96RR1	twinkle mt	Enzyme	245	0.516	0.692	0.003231	0.24
ATG7	10533	O95352	autophagy	Enzyme	179	0.505	0.769	7.38E-12	0.23
GRK5	2869	P34947	G protein-c	Kinase	86	0.588	0.769	0.67567	0.23
HTR1A	3350	P08908	5-hydroxyt	G-protein c	229	0.522	0.846	0.19956	0.23
PENK	5179	P01210	proenkepha	Signaling	124	0.56	0.808	0.000208	0.23
GDF5	8200	P43026	growth diff	Signaling	238	0.511	0.692	0.67002	0.23
ADH7	131	P40394	alcohol deh	Enzyme	42	0.656	0.462	1.51E-11	0.22
DLG4	1742	P78352	discs large	Receptor	91	0.595	0.654	0.99954	0.22
ABL1	25	P00519	ABL proto	Kinase	395	0.432	0.923	0.99998	0.22
PPP1R9B	84687	Q96SB3	protein phosphatase 1		42	0.678	0.423	0.99975	0.22
GRK6	2870	P43250	G protein-c	Kinase	51	0.67	0.654	0.000487	0.21
HSF1	3297	Q00613	heat shock	Transcript	185	0.507	0.769	0.74762	0.21
HSPD1	3329	P10809	heat shock protein fan		398	0.432	0.808	0.99257	0.21
LEP	3952	P41159	leptin		931	0.349	0.846	0.46491	0.21
ND3	4537	P03897	NADH dehydrogenase		113	0.604	0.654		0.21
PTN	5764	P21246	pleiotrophi	Signaling	164	0.515	0.808	0.007507	0.21
ARPC3	10094	O15145	actin relate	Cellular st	6	0.839	0.154	0.21578	0.2
ADCY5	111	O95622	adenylate cyclase 5		103	0.617	0.577	0.99928	0.2
COX10	1352	Q12887	cytochrome	Enzyme	81	0.623	0.654	0.08405	0.2
CCN2	1490	P29279	cellular co	Signaling	518	0.399	0.846	0.000502	0.2
GRK2	156	P25098	G protein-c	Kinase	159	0.532	0.654	0.99998	0.2
GRK3	157	P35626	G protein-c	Kinase	56	0.621	0.731	0.71059	0.2
DBN1	1627	Q16643	drebrin 1	Cellular st	54	0.633	0.538	0.99705	0.2
DLG1	1739	Q12959	discs large	Receptor	54	0.647	0.654	0.99393	0.2
GCH1	2643	P30793	GTP cyclo	Enzyme	254	0.511	0.846	0.90292	0.2
STK39	27347	Q9UEW8	serine/thre	Kinase	38	0.67	0.5	0.99916	0.2
APOE	348	P02649	apolipoprotein E		1049	0.338	0.962	0.001869	0.2
FASLG	356	P48023	Fas ligand	Signaling	398	0.43	0.885	0.18345	0.2
RRN3	54700	Q9NYV6	RRN3 hon	Transcript	26	0.711	0.346	1.62E-05	0.2
GJC2	57165	Q5T442	gap junctic	Cell-cell ju	115	0.593	0.692	0.010438	0.2
HCN3	57657	Q9P1Z3	hyperpolar	Ion channe	15	0.792	0.308	2.57E-05	0.2
RIT2	6014	Q99578	Ras like w	Enzyme m	46	0.647	0.538	0.000632	0.2
FEZ1	9638	Q99689	fasciculation and elon		41	0.666	0.5	0.75002	0.2
SLC41A1	254428	Q8IVJ1	solute carr	Transporte	13	0.769	0.385	0.65493	0.18
LAMP3	27074	Q9UQV4	lysosomal	Transporte	104	0.56	0.692	0.000267	0.18
MCCC1	56922	Q96RQ3	methylcrot	Enzyme	42	0.7	0.308	4.02E-12	0.17
CCDC62	84660	Q6P9F0	coiled-coil domain cor		15	0.805	0.308	4.39E-16	0.17
GPNMB	10457	Q14956	glycoprote	Signaling	103	0.566	0.692	2.88E-27	0.16
DGKQ	1609	P52824	diacylglyce	Kinase	17	0.736	0.423	1.76E-09	0.16
STH	246744	Q8IWL8	saitohin		27	0.705	0.385		0.16
VPS13C	54832	Q709C8	vacuolar protein sortin		71	0.653	0.346	7.65E-49	0.16
TMEM17:	84286	Q9BSA9	transmembrane protei		7	0.861	0.154	3.05E-09	0.16
HLA-DRE	3123	P01911	major histc	Immune re	1018	0.333	0.923	0.0011	0.15

CTSB	1508	P07858	cathepsin I	Enzyme	304	0.457	0.846	5.88E-11	0.14
SIPA1L2	57568	Q9P2F8	signal indu	Enzyme m	7	0.821	0.192	0.99215	0.14
SREBF1	6720	P36956	sterol regulatory elem		159	0.518	0.808	0.014708	0.14
NUCKS1	64710	Q9H1E3	nuclear casein kinase		36	0.691	0.5	0.96937	0.13
HIP1R	9026	O75146	huntingtin	Cellular st	29	0.7	0.423	1.46E-15	0.13
MAPT-AS	100128977		MAPT antisense RNA		34	0.711	0.269		0.12
BCKDK	10295	O14874	branched c	Kinase	12	0.769	0.154	5.66E-05	0.12
RAI1	10743	Q7Z5J4	retinoic aci	Transcript	205	0.545	0.731	1	0.12
FAM47E	100129583	Q6ZV65	family with sequence		1	1	0.038	0.001703	0.11
FAM47E-S	100631383	Q6ZV65	FAM47E-STBD1 rea		1	1	0.038	7.66E-12	0.11
LHFPL2	10184	Q6ZUX7	LHFPL tetraspan sub		8	0.931	0.154	0.056209	0.11
TCEANC2	127428	Q96MN5	transcriptio	Nucleic ac	1	1	0.038	2.76E-07	0.11
DLG2	1740	Q15700	discs large	Receptor	44	0.67	0.577	0.70796	0.11
INPP5F	22876	Q9Y2H2	inositol polyphosphate		9	0.805	0.231	2.36E-07	0.11
MMRN1	22915	Q13201	multimerin 1		272	0.465	0.846	2.07E-25	0.11
KANSL1	284058	Q7Z3B3	KAT8 regulatory NSI		140	0.592	0.731	0.99972	0.11
ITPKB	3707	P27987	inositol-tris	Kinase	17	0.805	0.192	0.99994	0.11
LMNA	4000	P02545	lamin A/C		824	0.384	0.885	0.9994	0.11
NSF	4905	P46459	N-ethylma	Enzyme	40	0.659	0.577	0.018338	0.11
PAM	5066	P19021	peptidylgly	Enzyme	155	0.542	0.692	6.93E-06	0.11
BIN3	55909	Q9NQY0	bridging integrator 3		5	0.931	0.115	5.82E-13	0.11
RREB1	6239	Q92766	ras responsive elemen		227	0.5	0.808	1	0.11
SH3GL2	6456	Q99962	SH3 domain containin		37	0.659	0.5	0.000119	0.11
TPM1	7168	P09493	tropomyos	Cellular st	117	0.56	0.615	0.001017	0.11
ITGA8	8516	P53708	integrin subunit alpha		46	0.641	0.577	6.08E-13	0.11
NAT2	10	P11245	N-acetyltra	Enzyme	311	0.451	0.885	3.27E-06	0.1
ZSCAN16	100129195		ZSCAN16 antisense I		14	0.792	0.269		0.1
CCNT2-A	100129961		CCNT2 antisense RN		12	0.931	0.115		0.1
MAPT-IT	100130148		MAPT intronic transc		2	0.931	0.115		0.1
BORCS7-	100528007		BORCS7-ASMT reac		20	0.805	0.154		0.1
CDH8	1006	P55286	cadherin 8		18	0.743	0.385	0.98806	0.1
FRY	10129	Q5TBA9	FRY microtubule binc		42	0.666	0.5	1	0.1
TNK2	10188	Q07912	tyrosine ki	Kinase	59	0.623	0.538	8.22E-12	0.1
DSCAS	101927698		DSC1/DSC2 antisens		3	0.931	0.115		0.1
TMC3-AS	101929655		TMC3 antisense RNA		3	1	0.038		0.1
CDK5	1020	Q00535	cyclin depe	Kinase	204	0.495	0.846	0.13892	0.1
MPHOSPI	10200	Q99547	M-phase phosphoprot		24	0.729	0.308	0.000411	0.1
LINC0222	102723839		long intergenic non-pr		2	0.931	0.077		0.1
LINC0221	104909134	P34998	LINC02210-CRHR1		123	0.57	0.692		0.1
LINC0247	105369734		long intergenic non-pr		4	0.861	0.154		0.1
LINC0245	105369738		long intergenic non-pr		2	0.931	0.115		0.1
AHSA1	10598	O95433	activator of HSP90 A		526	0.396	0.923	0.99896	0.1
LRRK2-D	107984474		LRRK2 divergent trai		6	0.861	0.154		0.1
LOC1079	107987479		cytochrome P450 2D6		76	0.597	0.769		0.1
CIT	11113	O14578	citron rho-	Kinase	127	0.555	0.692	0.9997	0.1
CHM	1121	P24386	CHM Rab	Enzyme	82	0.592	0.654	0.99899	0.1
SLC2A13	114134	Q96QE2	solute carr	Transporte	20	0.78	0.269	0.83307	0.1

NLRP3	114548	Q96P20	NLR family pyrin dor		805	0.361	0.962	0.000811	0.1
RAB39B	116442	Q96DA2	RAB39B, member RA		90	0.619	0.615	0.82529	0.1
AGAP1	116987	Q9UPQ3	ArfGAP with GTPase		13	0.839	0.154	0.99984	0.1
BORCS7	119032	Q96B45	BLOC-1 related comp		10	0.89	0.115	2.29E-07	0.1
KLHDC1	122773	Q8N7A1	kelch domain containi		1	1	0.038	4.48E-08	0.1
CNTN1	1272	Q12860	contactin 1		81	0.599	0.577	0.15131	0.1
COL5A2	1290	P05997	collagen type V alpha		139	0.568	0.731	1	0.1
COL13A1	1305	Q5TAT6	collagen type XIII alp		139	0.608	0.654	3.74E-14	0.1
COMT	1312	P21964	catechol-O	Enzyme	622	0.4	0.923	1.14E-06	0.1
SLCO6A1	133482	Q86UG4	solute carr	Transporte	449	0.412	0.885	2.88E-11	0.1
ADORA2	135	P29274	adenosine	G-protein c	213	0.526	0.769	0.5971	0.1
CRHR1	1394	P34998	corticotropin releasing		183	0.517	0.769	3.79E-05	0.1
CRK	1398	P46108	CRK proto-oncogene,		544	0.394	0.923	0.95936	0.1
CRP	1401	P02741	C-reactive protein		1483	0.299	0.962	0.003697	0.1
MAPK14	1432	Q16539	mitogen-ac	Kinase	626	0.379	0.923	0.37466	0.1
CSF2	1437	P04141	colony stin	Signaling	1028	0.33	0.962	0.83468	0.1
LINC0221	147081		long intergenic non-pr		10	0.861	0.154		0.1
PM20D1	148811	Q6GTS8	peptidase M20 domai		7	0.821	0.308	1.9E-18	0.1
CTSD	1509	P07339	cathepsin I	Enzyme	242	0.478	0.846	0.000917	0.1
CNKSR3	154043	Q6P9H4	CNKSR family memb		18	0.751	0.615	0.028166	0.1
CBLL2	158506	Q8N7E2	Cbl proto-	Enzyme	235	0.476	0.808		0.1
CYP17A1	1586	P05093	cytochrome P450 fam		326	0.462	0.769	0.000263	0.1
TMEM22	161145	Q8NBD8	transmembrane protei		5	0.931	0.115	0.13003	0.1
MDGA2	161357	Q7Z553	MAM domain contain		24	0.76	0.308	0.66457	0.1
BRINP1	1620	O60477	BMP/retinoic acid ind		64	0.615	0.615	0.99251	0.1
DBH	1621	P09172	dopamine beta-hydrox		202	0.511	0.846	3.34E-10	0.1
SPPL2C	162540	Q8IUH8	signal pept	Enzyme	14	0.76	0.192	0.000182	0.1
ACE	1636	P12821	angiotensir	Enzyme	1082	0.328	0.923	1.03E-37	0.1
SPTSSB	165679	Q8NFR3	serine palmitoyltransf		1	1	0.038	0.00087	0.1
PARK10	170534		Parkinson disease 10 (		3	0.89	0.038		0.1
ASXL1	171023	Q8IXJ9	ASXL transcriptional		296	0.482	0.808	1.25E-14	0.1
DRD3	1814	P35462	dopamine	G-protein c	199	0.529	0.692	0.002369	0.1
EEF1A2	1917	Q05639	eukaryotic	Enzyme	166	0.554	0.808	0.99558	0.1
EIF4G1	1981	Q04637	eukaryotic	Nucleic ac	139	0.553	0.808	1	0.1
C9orf72	203228	Q96LT7	C9orf72-SMCR8 con		258	0.496	0.769	1.06E-06	0.1
AKT1	207	P31749	AKT serin	Kinase	1250	0.311	0.962	0.9759	0.1
ESR1	2099	P03372	estrogen re	Nuclear re	1101	0.324	0.962	0.9992	0.1
ALDH1A1	216	P00352	aldehyde d	Enzyme	270	0.46	0.846	0.95448	0.1
ALDH2	217	P05091	aldehyde d	Enzyme	337	0.457	0.885	3.4E-10	0.1
SIRT2	22933	Q8IXJ6	sirtuin 2	Epigenetic	172	0.51	0.769	2.33E-08	0.1
TBC1D9	23158	Q6ZT07	TBC1 dor	Enzyme	491	0.399	0.846	2.8E-05	0.1
SYT11	23208	Q9BT88	synaptotag	Transporte	4	0.861	0.192	0.92676	0.1
MCF2L	23263	O15068	MCF.2 cel	Signaling	124	0.559	0.769	2.72E-07	0.1
FMR1	2332	Q06787	FMRP trai	Nucleic ac	346	0.473	0.769	0.64718	0.1
SYNM	23336	O15061	synemin		93	0.575	0.731	3.03E-24	0.1
SIRT1	23411	Q96EB6	sirtuin 1	Epigenetic	675	0.378	0.885	0.087326	0.1
TARDBP	23435	Q13148	TAR DNA binding pr		245	0.493	0.808	0.98542	0.1

SF3B1	23451	O75533	splicing fa	Nucleic ac	200	0.511	0.731	1	0.1
SMUG1	23583	Q53HV7	single-strand-selective		1034	0.322	0.923	0.000452	0.1
MTOR	2475	P42345	mechanisti	Kinase	960	0.343	0.885	1	0.1
FYN	2534	P06241	FYN proto	Kinase	116	0.556	0.731	0.99406	0.1
GABPA	2551	Q06546	GA bindin	Transcript	632	0.379	0.885	0.99812	0.1
FBXO7	25793	Q9Y3I1	F-box protein 7		76	0.65	0.769	5.79E-07	0.1
RNF19A	25897	Q9NV58	ring finger	Enzyme	523	0.397	0.923	0.025305	0.1
GAPDH	2597	P04406	glyceraldel	Enzyme	305	0.447	0.808	0.11388	0.1
GIGYF2	26058	Q6Y7W6	GRB10 interacting G		54	0.711	0.269	1	0.1
POLDIP2	26073	Q9Y2S7	DNA polymerase delt		530	0.396	0.923	0.41407	0.1
FGF20	26281	Q9NP95	fibroblast	Signaling	45	0.663	0.577	0.81379	0.1
GBAP1	2630		glucosylceramidase be		64	0.623	0.538		0.1
GCG	2641	P01275	glucagon		441	0.431	0.885	0.034814	0.1
HPGDS	27306	O60760	hematopoietic prostag		570	0.388	0.923	1.81E-06	0.1
GLP1R	2740	P43220	glucagon li	G-protein	288	0.471	0.846	0.29347	0.1
HTRA2	27429	O43464	HtrA serine peptidase		151	0.542	0.692	0.003576	0.1
GPR37	2861	O15354	G protein-	G-protein	23	0.716	0.385	0.000338	0.1
REM1	28954	O75628	RRAD and GEM like		177	0.531	0.885	5.34E-08	0.1
GRN	2896	P28799	granulin precursor		412	0.435	0.846	0.069666	0.1
GRIN2B	2904	Q13224	glutamate	Ion channe	249	0.51	0.692	1	0.1
CTNNA3	29119	Q9UI47	catenin alp	Cellular st	73	0.612	0.692	1.02E-12	0.1
GRM5	2915	P41594	glutamate	G-protein	189	0.525	0.846	0.99872	0.1
GSTT1	2952	P30711	glutathione S-transfer		541	0.393	0.923	0.000148	0.1
HTT	3064	P42858	huntingtin		188	0.548	0.654	1	0.1
RPA2P1	326628		replication protein A2		1	1	0.038		0.1
PRSS53	339105	Q2L4Q9	serine protease 53		2	1	0.038	9.35E-17	0.1
TMC3	342125	Q7Z5M5	transmembrane chann		3	0.931	0.077	3.89E-22	0.1
MCIDAS	345643	D6RGH6	multiciliate differentia		119	0.566	0.769	0.013264	0.1
IGF1	3479	P05019	insulin like growth fac		1206	0.318	0.885	0.2716	0.1
IGFALS	3483	P35858	insulin like	Receptor	203	0.511	0.846	5.44E-11	0.1
APP	351	P05067	amyloid be	Enzyme m	485	0.422	0.846	0.046544	0.1
IL1A	3552	P01583	interleukin 1 alpha		1002	0.333	0.962	0.00016	0.1
IL10	3586	P22301	interleukin 10		1679	0.281	0.923	0.005887	0.1
TMPRSS9	360200	Q7Z410	transmembrane serine		5	0.861	0.192	1.62E-17	0.1
ITIH1	3697	P19827	inter-alpha	Enzyme m	38	0.691	0.462	2.08E-31	0.1
GSTK1	373156	Q9Y2Q3	glutathione S-transfer		445	0.412	0.885	1.94E-06	0.1
TPTE2P6	374491		transmembrane phosp		4	0.931	0.038		0.1
LAMC2	3918	Q13753	laminin sul	Enzyme m	602	0.389	0.923	2.88E-12	0.1
LINC0112	400952		long intergenic non-pr		15	0.751	0.192		0.1
LRP2	4036	P98164	LDL receptor related		254	0.491	0.846	0.99999	0.1
LY6E	4061	Q16553	lymphocyte antigen 6		115	0.563	0.808	0.3211	0.1
ATXN3	4287	P54252	ataxin 3	Enzyme	207	0.521	0.808	0.10575	0.1
ACHE	43	P22303	acetylcholi	Enzyme	418	0.445	0.885	0.99827	0.1
MPZ	4359	P25189	myelin protein zero		249	0.503	0.846	0.27158	0.1
UNC13C	440279	Q8NB66	unc-13 homolog C		11	0.931	0.115	2.17E-15	0.1
TYW1B	441250	Q6NUM6	tRNA-yW synthesizin		3	1	0.038	5.43E-20	0.1
CDNF	441549	Q49AH0	cerebral dopamine neu		15	0.78	0.231	6.13E-07	0.1

DPY19L2l	442524		DPY19L2 pseudogene		2	1	0.038		0.1
MX2	4600	P20592	MX dynan	Enzyme m	14	0.751	0.231	6.48E-09	0.1
NEFL	4747	P07196	neurofilament light		247	0.488	0.769		0.1
NFE2L2	4780	Q16236	nuclear fac	Enzyme	823	0.357	0.885	0.003572	0.1
NOS2	4843	P35228	nitric oxide synthase 2		783	0.364	0.923	4.03E-15	0.1
NRTN	4902	Q99748	neurturin	Signaling	43	0.686	0.538	0.030223	0.1
PAFAH1E	5048	P43034	platelet activating fact		219	0.504	0.769	0.99995	0.1
PARK3	5072		Parkinson disease 3 (a		6	0.821	0.038		0.1
CHCHD2	51142	Q9Y6H1	coiled-coil-helix-coile		57	0.638	0.538	1.73E-07	0.1
IP6K2	51447	Q9UHH9	inositol he	Kinase	18	0.821	0.231	0.973	0.1
SYT17	51760	Q9BSW7	synaptotag	Transporte	5	1	0.038	1.1E-05	0.1
PIK3CA	5290	P42336	phosphatid	Kinase	1511	0.292	0.923	1	0.1
PIK3CB	5291	P42338	phosphatid	Kinase	1083	0.322	0.885	0.99964	0.1
PIK3CD	5293	O00329	phosphatid	Kinase	1119	0.319	0.885	0.99999	0.1
PIK3CG	5294	P48736	phosphatid	Kinase	1101	0.32	0.885	1.41E-06	0.1
DCUN1D1	54165	Q96GG9	defective in cullin ned		37	0.67	0.538	0.98526	0.1
TREM2	54209	Q9NZC2	triggering receptor ex		239	0.519	0.769	3.29E-09	0.1
POLG	5428	P54098	DNA poly	Enzyme	462	0.457	0.846	2.08E-09	0.1
PON1	5444	P27169	paraoxonase 1		496	0.409	0.885	9.81E-11	0.1
TET2	54790	Q6N021	tet methylcytosine dio		362	0.458	0.808	4.33E-54	0.1
CNNM2	54805	Q9H8M5	cyclin and CBS doma		91	0.641	0.346	0.99521	0.1
WBP1L	54838	Q9NX94	WW domain binding p		22	0.769	0.462	0.64011	0.1
PLPPR1	54886	Q8TBJ4	phospholip	Enzyme	28	0.78	0.077	0.56614	0.1
PREP	5550	P48147	prolyl endo	Enzyme	105	0.576	0.731	0.08025	0.1
PIP4P2	55529	Q8N4L2	phosphatidylinositol-4		1	1	0.038	0.000422	0.1
MAPK1	5594	P28482	mitogen-ac	Kinase	1059	0.33	0.923	0.99698	0.1
SLC50A1	55974	Q9BRV3	solute carr	Transporte	36	0.686	0.538	0.001926	0.1
PRNP	5621	F7VJQ1;P	prion protein		426	0.445	0.923	0.000632	0.1
PDSS2	57107	Q86YH6	decaprenyl diphosphat		83	0.626	0.577	4.37E-08	0.1
PTEN	5728	P60484	phosphatas	Enzyme	1349	0.305	0.923	0.25651	0.1
PTGS2	5743	P35354	prostaglan	Enzyme	1234	0.314	0.962	0.99597	0.1
BCHE	590	P06276	butyrylcholinesterase		392	0.447	0.923	1.06E-13	0.1
BCL2	596	P10415	BCL2 apo	Signaling	1456	0.291	0.885	0.55903	0.1
RET	5979	P07949	ret proto-o	Kinase	607	0.392	0.885	1	0.1
ATXN2	6311	Q99700	ataxin 2	Nucleic ac	341	0.482	0.808	0.85289	0.1
SCN2A	6326	Q99250	sodium vol	Ion channe	232	0.518	0.731	1	0.1
CCL2	6347	P13500	C-C motif	Signaling	1157	0.321	0.962	0.60786	0.1
DNAJC1	64215	Q96KC8	DnaJ heat shock prote		8	0.821	0.154	1.33E-08	0.1
CASC16	643714		cancer susceptibility 1		12	0.78	0.308		0.1
SNCA-AS	644248		SNCA antisense RNA		2	1	0.038		0.1
SMPD1	6609	P17405	sphingomy	Enzyme	247	0.499	0.885	3.89E-10	0.1
SNCB	6620	Q16143	synuclein b	Transporte	60	0.619	0.577	0.068062	0.1
SNCG	6623	O76070	synuclein g	Transporte	122	0.55	0.615	0.000848	0.1
THY1	7070	P04216	Thy-1 cell surface ant		197	0.496	0.808	0.049895	0.1
TLR4	7099	O00206	toll like receptor 4		1174	0.321	0.962	4.61E-09	0.1
TP53	7157	P04637	tumor prot	Transcript	2494	0.236	0.962	0.53235	0.1
TPO	7173	P07202	thyroid per	Enzyme	306	0.455	0.846	1.82E-21	0.1

UCHL1	7345	P09936	ubiquitin C	Enzyme	260	0.482	0.885	0.99095	0.1
VDR	7421	P11473	vitamin D	Nuclear re	852	0.352	0.885	1.68E-05	0.1
VEGFA	7422	P15692	vascular er	Signaling	1899	0.266	0.923	2.41E-05	0.1
WNT3	7473	P56703	Wnt family	Signaling	140	0.536	0.808	0.87515	0.1
YWHAE	7531	P62258	tyrosine 3-	Chaperone	176	0.53	0.692	0.98479	0.1
CA8	767	P35219	carbonic anhydrase 8		87	0.595	0.654	0.008252	0.1
ZNF165	7718	P49910	zinc finger protein 165		9	0.78	0.269	9.11E-09	0.1
ZP3	7784	P21754	zona pellucida glycopr		9	0.805	0.115	0.000285	0.1
MANF	7873	P55145	mesencephalic astrocy		62	0.621	0.654	0.001391	0.1
MUL1	79594	Q969V5	mitochondrial E3 ubic		241	0.473	0.808	1.91E-06	0.1
AIMP2	7965	Q13155	aminoacyl tRNA synth		555	0.393	0.923	0.008024	0.1
CCDC82	79780	Q8N4S0	coiled-coil domain cor		1	1	0.038	2.38E-07	0.1
RABEP2	79874	Q9H5N1	rabaptin, RAB GTPas		36	0.682	0.654	0.001139	0.1
KCNIP4	80333	Q6PIL6	potassium	Ion channe	31	0.716	0.385	0.82064	0.1
CAB39L	81617	Q9H9S4	calcium bi	Calcium-b	14	0.769	0.192	6.39E-05	0.1
BAP1	8314	Q92560	BRCA1 as	Enzyme	299	0.454	0.731	0.99443	0.1
CASP3	836	P42574	caspase 3	Enzyme	819	0.351	0.923	0.10017	0.1
PLA2G6	8398	O60733	phospholip	Enzyme m	350	0.476	0.846	2.48E-10	0.1
GPR65	8477	Q8IYL9	G protein-c	G-protein c	47	0.653	0.5	0.1114	0.1
LINGO1	84894	Q96FE5	leucine ricl	Receptor	44	0.682	0.385	0.99413	0.1
SLC45A3	85414	Q96JT2	solute carr	Transporte	19	0.78	0.192	0.005266	0.1
GBF1	8729	Q92538	golgi brefeldin A resis		14	0.839	0.269	4.75E-15	0.1
SQSTM1	8878	Q13501	sequestosome 1		470	0.428	0.885	0.00086	0.1
RAB29	8934	O14966	RAB29, member RAS		11	0.769	0.231	0.18152	0.1
NDUFAF2	91942	Q8N183	NADH:ubiquinone ox		89	0.641	0.577	2.08E-06	0.1
ADAMTS	92949	Q8N6G6	ADAMTS	Enzyme	40	0.682	0.654	3.12E-12	0.1
TPTE2	93492	Q6XPS3	transmemb	Enzyme	12	0.78	0.154	1.88E-12	0.1
GRAP2	9402	O75791	GRB2 related adaptor		538	0.394	0.923	0.000153	0.1
MAP4K4	9448	O95819	mitogen-ac	Kinase	80	0.588	0.692	1	0.1
SNCAIP	9627	Q9Y6H5	synuclein alpha intera		44	0.691	0.423	0.000328	0.1
ZNF646	9726	O15015	zinc finger	Transcripti	5	0.89	0.154	6.78E-06	0.1
TBC1D5	9779	Q92609	TBC1 dom	Enzyme	17	0.839	0.154	4.64E-18	0.1
CTIF	9811	O43310	cap bindin	Nucleic ac	10	0.821	0.231	0.99994	0.1
PLEKHM	9842	Q9Y4G2	pleckstrin homology a		41	0.691	0.577	0.008138	0.1

EL_gda	EI_gda	N_PMIDs	N_SNPs_gd	First_Ref	Last_Ref
	0.983	2577	53	1997	2020
	0.973	110	8	2003	2020
	1	28	0	1988	2019
	0.931	58	3	1995	2020
strong	0.946	56	4	2007	2019
	0.991	110	1	1993	2020
	0.974	304	20	1998	2020
	0.987	376	12	2002	2020
	0.966	29	1	1996	2020
	0.979	141	3	1989	2020
	1	10	0	1996	2020
	1	3	0	2009	2019
	0.983	987	35	2002	2020
	1	18	4	2009	2020
	0.989	264	15	2004	2020
	0.972	142	40	1999	2019
	0.92	25	8	2009	2019
	1	10	1	2010	2015
	0.963	27	0	2009	2019
	1	20	1	2010	2020
	0.857	21	3	2009	2019
	0.938	16	0	2004	2020
	0.879	58	0	1992	2019
	0.983	117	0	1993	2020
	1	16	0	1999	2020
	0.923	13	2	2016	2018
	0.75	20	0	1999	2019
	0.727	11	4	2002	2018
	0.905	21	2	1997	2020
	0.818	11	0	2006	2018
	1	23	1	2001	2019
	0.87	23	2	1994	2019
	1	21	4	2004	2019
	0.727	11	3	1997	2019
	0.9	20	4	2002	2018
	0.931	58	2	2011	2019
	0.906	96	6	1995	2020
	0.935	185	7	1994	2020
	0.971	35	3	1993	2020
	1	11	3	1997	2019
	0.98	50	0	1999	2020
	0.778	9	2	2003	2019
	1	8	0	2006	2018

0.875	8	2	1998	2013
0.833	6	0	2006	2020
1	6	0	2009	2018
1	4	0	2001	2019
1	5	0	2009	2019
1	4	0	2005	2018
1	3	0	2008	2017
1	3	0	2018	2019
1	2	0	2012	2017
1	3	0	2009	2016
1	3	0	2008	2020
1	2	0	2019	2020
1	2	0	2008	2010
1	2	0	1999	2015
1	2	0	2005	2009
1	1	0	2006	2006
1	2	0	2004	2005
1	2	0	1996	2009
1	1	0	2017	2017
1	2	0	2009	2017
1	1	0	2016	2016
1	1	0	2017	2017
1	1	0	2015	2015
1	1	0	2015	2015
1	1	0	2015	2015
1	1	0	2015	2015
1	1	0	2009	2009
1	1	0	2012	2012
1	1	0	2015	2015
1	23	3	2005	2019
1	1	0	2015	2015
1	15	0	2000	2019
0.923	13	1	2007	2020
0.875	32	2	2002	2020
1	1	0	2015	2015
1	1	0	2008	2008
1	1	0	2019	2019
1	1	0	2015	2015
0.973	73	2	1999	2019
0.893	28	4	2001	2020
1	1	0	2015	2015
1	1	0	2014	2014
1	1	0	2015	2015
1	1	0	2008	2008
1	1	0	2008	2008
1	1	0	2008	2008
1	1	0	2012	2012

0.9	10	1	2007	2020
1	1	0	2008	2008
1	7	1	2013	2020
1	7	2	2003	2016
1	4	0	2004	2015
1	3	0	2013	2019
1	4	2	2008	2015
1	4	0	2006	2020
1	4	0	1995	2007
1	4	0	2012	2017
1	3	0	2000	2020
0.667	3	0	2005	2019
1	2	0	2014	2017
1	3	0	2008	2018
1	2	0	2008	2010
1	3	0	2014	2018
1	2	0	2013	2020
1	2	0	2007	2019
1	2	0	2005	2011
1	2	0	2009	2018
1	1	0	2010	2010
1	1	0	2003	2003
	0	0		
1	1	0	2009	2009
1	1	0	2008	2008
1	1	0	2008	2008
1	1	0	2013	2013
1	1	0	2005	2005
1	33	3	1996	2019
1	11	2	2011	2017
0.922	90	1	1994	2020
1	1	0	2007	2007
	0	0		
1	1	0	2011	2011
1	1	0	2009	2009
0.938	16	2	2011	2020
1	1	0	2014	2014
1	10	5	2010	2017
1	9	3	2011	2019
1	13	7	2011	2017
1	12	2	2011	2017
1	8	2	2011	2019
1	9	2	2009	2020
1	7	3	2003	2015
1	7	5	2016	2018
1	12	5	2011	2020
1	6	2	2010	2019

1	5	1	2016	2020
1	6	1	2014	2018
0.75	4	1	2011	2019
1	5	2	2009	2017
1	4	1	2012	2015
1	6	32	2009	2019
1	4	1	2014	2019
1	2	2	2011	2016
1	6	3	2009	2017
1	6	3	2009	2017
1	1	1	2016	2016
1	2	1	2015	2015
1	4	2	2014	2018
1	3	1	2014	2018
1	2	1	2012	2012
1	5	11	2008	2014
1	2	1	2017	2020
1	2	1	2014	2019
0.833	6	4	2009	2012
1	2	1	2016	2017
1	2	1	2017	2018
1	2	1	2006	2019
1	4	3	2012	2019
1	1	1	2016	2016
1	3	2	2012	2018
1	13	1	1997	2016
1	1	1	2017	2017
1	4	2	2011	2017
1	1	1	2011	2011
1	1	2	2009	2009
1	1	1	2011	2011
1	1	1	2012	2012
	0	1		
1	1	1	2011	2011
1	1	1	2013	2013
1	16	0	2007	2020
1	26	1	2000	2019
1	1	1	2016	2016
1	8	32	2009	2016
1	2	4	2012	2014
1	1	1	2016	2016
1	10	0	2003	2019
1	4	6	2012	2017
0.917	12	0	1994	2017
1	29	1	1998	2019
0.892	37	1	2000	2020
1	5	6	2009	2017

1	22	2	2017	2020
0.8	10	1	2014	2018
1	1	1	2017	2017
1	1	1	2009	2009
1	1	1	2016	2016
1	1	1	2014	2014
1	1	1	2016	2016
1	1	1	2013	2013
0.953	85	6	1984	2020
0.909	11	0	1999	2019
1	17	2	2003	2019
1	3	20	2011	2014
1	10	0	2003	2019
1	10	0	2017	2020
1	11	0	2003	2019
0.97	33	0	2016	2020
1	8	6	2009	2016
1	2	1	2009	2012
0.917	12	1	2003	2020
1	1	1	2012	2012
1	54	0	2002	2019
1	1	1	2009	2009
1	2	1	2014	2017
1	1	1	2016	2016
1	1	1	2010	2010
0.917	12	7	2004	2019
1	4	7	2009	2014
0.667	12	0	1999	2019
1	1	1	2017	2017
0.917	12	0	2005	2020
0.957	23	0	2017	2020
0.917	24	1	2003	2020
0.974	39	0	2004	2020
0.783	23	5	2011	2019
0.812	16	1	2012	2019
0.95	20	4	2008	2020
0.909	11	3	1999	2019
1	11	1	2003	2019
1	11	4	2013	2019
1	18	4	2007	2020
0.9	10	0	2003	2017
0.917	12	0	2004	2019
0.979	47	1	2010	2019
0.929	14	0	2005	2020
1	12	0	2000	2019
0.812	16	2	2012	2020
0.947	19	2	2008	2020

0.957	23	0	2017	2020
1	18	0	2017	2020
1	21	0	2008	2020
1	13	0	2000	2019
0.977	43	0	2006	2020
0.955	22	5	2009	2019
1	11	0	2003	2019
1	10	4	2000	2020
0.8	25	9	2005	2017
1	10	0	2003	2019
0.833	24	3	2004	2019
1	26	1	2005	2019
1	18	0	2014	2019
1	15	0	1999	2019
1	16	0	2017	2019
0.969	32	4	2005	2018
1	11	0	2006	2019
1	12	0	2011	2019
0.917	12	3	2007	2018
1	13	3	2002	2019
1	1	1	2006	2006
1	17	0	2010	2020
0.583	12	0	1999	2019
1	15	0	2003	2019
1	1	1	2016	2016
1	1	1	2012	2012
1	1	1	2013	2013
1	14	0	2015	2019
0.818	11	1	2008	2019
1	10	1	2001	2020
1	14	5	2002	2018
0.636	11	0	2002	2019
0.882	17	0	2005	2019
1	2	1	2014	2017
1	1	1	2017	2017
0.909	11	0	1999	2019
1	1	1	2006	2006
0.968	31	0	2017	2020
1	1	1	2010	2010
0.979	47	0	2010	2019
0.833	12	0	2003	2018
0.917	12	0	2003	2019
1	17	0	2005	2019
1	26	0	2000	2019
1	1	1	2006	2006
1	1	1	2006	2006
1	18	1	2007	2020

1	1	1	2006	2006
1	1	1	2015	2015
1	12	0	2002	2019
0.983	59	6	2006	2020
1	11	2	1997	2018
0.964	28	0	2007	2020
0.957	23	0	2017	2020
0.917	12	0	1999	2013
0.857	21	3	2015	2019
1	1	1	2017	2017
1	2	1	2016	2017
1	26	0	2005	2020
1	26	0	2005	2020
1	26	1	2005	2020
1	26	0	2005	2020
1	1	1	2011	2011
0.8	20	3	2013	2020
0.692	13	3	2006	2019
0.955	22	2	1998	2018
0.957	23	0	2017	2020
1	1	2	2009	2009
1	1	1	2009	2009
1	1	1	2018	2018
1	11	0	2011	2020
1	1	1	2006	2006
1	14	0	2003	2019
1	1	1	2012	2012
1	13	2	2006	2020
1	18	0	2011	2019
0.963	27	2	2004	2019
1	10	0	2007	2018
1	16	2	2000	2019
1	17	0	1999	2019
1	10	0	2000	2020
0.833	18	0	2003	2018
1	1	1	2017	2017
0.818	11	0	2003	2019
1	1	1	2006	2006
1	1	2	2017	2017
1	1	10	2012	2012
1	14	5	2013	2019
0.966	89	2	1999	2020
0.959	74	1	1999	2020
1	13	0	2007	2019
1	14	4	2014	2019
1	13	2	2011	2020
1	21	0	2007	2019

0.948	77	4	1999	2020
0.955	22	8	2005	2020
0.818	11	3	2004	2018
1	4	4	2011	2014
0.957	23	0	2017	2020
1	1	1	2016	2016
1	1	1	2017	2017
1	1	1	2015	2015
1	14	0	2003	2019
1	56	2	2002	2019
1	13	1	2003	2019
1	1	1	2013	2013
1	1	1	2014	2014
1	1	1	2015	2015
1	1	1	2017	2017
1	21	0	1999	2019
1	16	1	2000	2020
1	16	3	2010	2019
1	1	1	2017	2017
0.824	17	2	2007	2015
1	1	1	2012	2012
1	1	2	2017	2017
1	11	0	2003	2020
0.929	14	3	2010	2020
1	2	2	2016	2017
1	1	1	2006	2006
1	1	1	2011	2011
1	11	0	2003	2019
1	1	1	2017	2017
1	42	4	2000	2019
1	1	1	2012	2012
1	1	1	2017	2017
1	1	1	2006	2006
1	4	1	2009	2012

Table S2 Targets associtaetd with Parkinson's disease
Table S2-2 Targets obtained from OMIN

Cytogenetic lo	Genomic coordinates	Gene/Locus
1p36		AD7CNTP
1p36		BCC1
1p36		CMM, MLM, DNS
1pter-p36.13		CTRCT8, CCV
1p36		DEL1p36, C1DELp36
1p36		IBD7
1p36		MS4
1p		PSORS7
1p36.33	1:1013496-1014539	ISG15, G1P2, IFI15, I
1p36.33	1:1020101-1056118	AGRN, CMS8
1p36.33	1:1211325-1216811	TNFRSF4, TXGP1L, (
1p36.33	1:1232236-1235040	B3GALT6, SEMDJL1
1p36.33	1:1232236-1235040	B3GALT6, SEMDJL1
1p36.33	1:1232236-1235040	B3GALT6, SEMDJL1
1p36.33	1:1280435-1292028	SCNN1D
1p36.33	1:1335277-1349417	DVL1, DRS2
1p36.33	1:1512142-1534686	ATAD3A, HAYOS, PI
1p36.33	1:1512142-1534686	ATAD3A, HAYOS, PI
1p36.33	1:1785284-1891116	GNB1, MRD42
1p36.33	1:1785284-1891116	GNB1, MRD42
1p36.33-p36.3	1:2228318-2310212	SKI, SGS
1p36.32	1:2403973-2413826	PEX10, NALD, PBD6
1p36.32	1:2403973-2413826	PEX10, NALD, PBD6
1p36.32	1:2556364-2565621	TNFRSF14, HVEM, T
1p36.32	1:3069202-3438620	PRDM16, MEL1, LVN
1p36.32	1:3069202-3438620	PRDM16, MEL1, LVN
1p36.32	1:3652515-3736200	TP73
1p36.32	1:3772760-3775981	SMIM1, VEL
1p36.31	1:5862809-5992424	NPHP4, SLSN4
1p36.31	1:5862809-5992424	NPHP4, SLSN4
1p36.31	1:6221192-6235963	ICMT
1p36.31	1:6424775-6461366	ESPN, USH1M
1p36.31	1:6424775-6461366	ESPN, USH1M
1p36.31	1:6424775-6461366	ESPN, USH1M
1p36.31	1:6460785-6466194	TNFRSF25, TNFRSF
1p36.31	1:6466091-6520060	PLEKHG5, KIAA072(
1p36.31	1:6466091-6520060	PLEKHG5, KIAA072(
1p36.2	1:7100000-15900000	SCZD12
1p36.23	1:7915870-7941606	TNFRSF9, ILA, CD13
1p36.23	1:7961653-7985504	DJ1, PARK7
1p36.23	1:8011724-8026308	MIG6, RALT
1p36.23	1:8860999-8878685	ENO1, PPH, MPB1
1p36.22	1:9151667-9151776	MIR34A, MIRN34A
1p36.22	1:9234766-9271336	H6PD, GDH, G6PDH,
1p36.22	1:9629888-9729113	PIK3CD, APDS, IMD
1p36.22	1:9942922-9996883	NMNAT1, NMNAT, I

1p36.22	1:10210569-10381602	KIF1B, CMT2A, CMT
1p36.22	1:10210569-10381602	KIF1B, CMT2A, CMT
1p36.22	1:10210569-10381602	KIF1B, CMT2A, CMT
1p36.22	1:10450030-10451997	CORT
1p36.22	1:11012653-11030527	TARDBP, TDP43, AL
1p36.22	1:11012653-11030527	TARDBP, TDP43, AL
1p36.22	1:11026522-11047238	MASP2
1p36.22	1:11106534-11273496	MTOR, FRAP1, SKS
1p36.22	1:11106534-11273496	MTOR, FRAP1, SKS
1p36.22	1:11273197-11299571	UBIAD1, TERE1, SC(
1p36.22	1:11785722-11806102	MTHFR
1p36.22	1:11785722-11806102	MTHFR
1p36.22	1:11785722-11806102	MTHFR
1p36.22	1:11785722-11806102	MTHFR
1p36.22	1:11785722-11806102	MTHFR
1p36.22	1:11806095-11843143	CLCN6
1p36.22	1:11845708-11847782	NPPA, PND, ANP, AT
1p36.22	1:11845708-11847782	NPPA, PND, ANP, AT
1p36.22	1:11857463-11858944	NPPB, BNP
1p36.22	1:11934716-11975536	PLOD1, LH1, LLH, E
1p36.22	1:11980180-12013514	MFN2, KIAA0214, CM
1p36.22	1:11980180-12013514	MFN2, KIAA0214, CM
1p36.22	1:11980180-12013514	MFN2, KIAA0214, CM
1p36.22	1:12063302-12144212	TNFRSF8, CD30, D1S
1p36.22	1:12166947-12209221	TNFRSF1B, TNFR2,
1p36.21	1:12500000-15900000	GBD2
1p36.21	1:15409887-15430338	EFHD2, SWS1
1p36.21	1:15438441-15449246	CTRC, CLCR
1p36.21	1:15456731-15472090	CELA2A, ELA2A, AC
1p36.21	1:15681505-15734768	PLEKHM2, SKIP, KIA
1p36.13-p34.3	1:15900000-39600000	ANIB3
1p36.13	1:16022035-16034049	CLCNKA
1p36.13	1:16043781-16057325	CLCNKB
1p36.13	1:16043781-16057325	CLCNKB
1p36.13	1:16124336-16156103	EPHA2, ECK, CTPP1,
1p36.13	1:16985957-17011971	ATP13A2, PARK9, KI
1p36.13	1:16985957-17011971	ATP13A2, PARK9, KI
1p36.13	1:17018721-17054031	SDHB, SDH2, SDHIP
1p36.13	1:17018721-17054031	SDHB, SDH2, SDHIP
1p36.13	1:17018721-17054031	SDHB, SDH2, SDHIP
1p36.13	1:17018721-17054031	SDHB, SDH2, SDHIP
1p36.13	1:17308194-17364003	PADI4, PADI5, PAD
1p36.13	1:17372195-17401698	PADI6, PREMBL2
1p36.13	1:18630845-18748865	PAX7, RMS2, MYOS(
1p36.13	1:18630845-18748865	PAX7, RMS2, MYOS(
1p36.13	1:18871429-18902798	ALDH4A1, ALDH4, P
1p36.13	1:19215659-19251523	EMC1, KIAA0090, CA

1p36.13	1:19975430-1998043[illegible]	PLA2G2A, PLA2B, PI[illegible]
1p36.13	1:20028348-2009191[illegible]	PLA2G5, FRFB
1p36.12	1:20633457-2065151[illegible]	PINK1, PARK6
1p36.12	1:20651776-2066136[illegible]	DDOST, OST, OST48
1p36.12	1:21217249-2134550[illegible]	ECE1
1p36.12	1:21217249-2134550[illegible]	ECE1
1p36.12	1:21508983-21578411	ALPL, HOPS, TNSAL
1p36.12	1:21508983-21578411	ALPL, HOPS, TNSAL
1p36.12	1:21508983-21578411	ALPL, HOPS, TNSAL
1p36.12	1:21508983-21578411	ALPL, HOPS, TNSAL
1p36.12	1:21822243-2193730[illegible]	HSPG2, PLC, SJS, SJ[illegible]
1p36.12	1:21822243-2193730[illegible]	HSPG2, PLC, SJS, SJ[illegible]
1p36.12	1:22052708-2210135[illegible]	CDC42, TKS
1p36.12	1:22117307-2214398[illegible]	WNT4, SERKAL
1p36.12	1:22117307-2214398[illegible]	WNT4, SERKAL
1p36.12	1:22636627-22639681	C1QA
1p36.12	1:22643632-2264810[illegible]	C1QC, C1QG
1p36.12	1:22653235-2266163[illegible]	C1QB
1p36.12	1:22710769-2292149[illegible]	EPHB2, EPHT3, DRT
1p36.12	1:22710769-2292149[illegible]	EPHB2, EPHT3, DRT
1p36.12	1:23019442-2308369[illegible]	KDM1A, LSD1, AOF[illegible]
1p36.12	1:23304687-2334431[illegible]	HNRPR, HNRNPR
1p36.12	1:23557925-2355950[illegible]	ID3
1p36.11	1:23691778-23696834	RPL11, DBA7
1p36.11	1:23795598-2380075[illegible]	GALE
1p36.11	1:23801876-2382545[illegible]	HMGCL
1p36.11	1:23845076-23868331	FUCA1
1p36.11	1:23870514-23913361	CNR2, CB2, CX5
1p36.11	1:24056040-24112134	MYOM3
1p36.11	1:24319332-24364481	GRHL3, SOM, TFCP[illegible]
1p36.11	1:24899510-2496515[illegible]	RUNX3, CBFA3, PEB
1p36.11	1:25272392-25330444	RHD
1p36.11	1:25360658-2543019[illegible]	RHCE, RHNA
1p36.11	1:25360658-2543019[illegible]	RHCE, RHNA
1p36.11	1:25543587-2559039[illegible]	LDLRAP1, ARH, FH[illegible]
1p36.11	1:25800192-2581822[illegible]	SELENON, SEPN1, S[illegible]
1p36.11	1:25800192-2581822[illegible]	SELENON, SEPN1, S[illegible]
1p36.11	1:25884178-2590687[illegible]	STMN1, LAP18, SM[illegible]
1p36.11	1:26317957-2632052[illegible]	CD52, CDW52
1p36.11	1:26432281-2647130[illegible]	DHDDS, HDS, RP59,
1p36.11	1:26432281-2647130[illegible]	DHDDS, HDS, RP59,
1p36.11	1:26432281-2647130[illegible]	DHDDS, HDS, RP59,
1p36.11	1:26787053-2680065[illegible]	PIGV, HPMRS1
1p36.11	1:26890487-2690046[illegible]	GPATCH3
1p36.11	1:26911488-2691410[illegible]	NR0B2, SHP
1p36.11	1:26949555-2696049[illegible]	KDF1, C1orf172, ECT
1p36.11	1:27098808-27155124	SLC9A1, NHE1, APN

1p36.11	1:27369109-27374851	FCN3, HAKA1
1p36.11	1:27392621-27395813	GPR3
1p35.3	1:27934954-27959151	SMPDL3B, ASML3B
1p35.3	1:28147165-28193935	PTAFR
1p35.2	1:30711276-30723584	MATN1, CRTM, CMI
1p35.2	1:30869465-30909734	SDC3, SYND3, SDCN
1p35.2	1:30931505-3106571ϵ	PUM1, KIAA0099, SC
1p35.2	1:32200594-3220538ϵ	CCDC28B, MGC1203
1p35.2-p35.1	1:32292082-32333627	HDAC1, RPD3L1
1p35.1	1:32679905-32703595	SYNC1
1p35.1	1:32775237-32818031	YARS1, YARS, CMTI
1p35.1	1:32862267-32872483	FNDC5, FRCP2
1p35.1	1:32885964-32894645	HPCA, DYT2
1p35.1	1:33007939-3303691C	AK2
1p35.1	1:33145398-33184773	TRIM62, DEAR1
1p34.3	1:34300000-3960000C	GBD3
1p34.3	1:34759739-3476232ϵ	GJB4, CX30.3, EKVP
1p34.3	1:34781213-34786363	GJB3, CX31, DFNA2I
1p34.3	1:34781213-34786363	GJB3, CX31, DFNA2I
1p34.3	1:34781213-34786363	GJB3, CX31, DFNA2I
1p34.3	1:34781213-34786363	GJB3, CX31, DFNA2I
1p34.3	1:34781213-34786363	GJB3, CX31, DFNA2I
1p34.3	1:34792957-3479574ϵ	GJA4, CX37
1p34.3	1:36095238-3612620ϵ	COL8A2, FECD1, PP(
1p34.3	1:36095238-3612620ϵ	COL8A2, FECD1, PP(
1p34.3	1:37474517-3748437ϵ	ZC3H12A, MCPIP, M
1p34.3	1:37534448-37554292	SNIP1, PMRED
1p34.3	1:37611349-37634905	RSPO1, FLJ40906
1p34.3	1:37611349-37634905	RSPO1, FLJ40906
1p34.3	1:38012715-3802481[illegible]	UTP11L, CGI94
1p34.3	1:39026294-39034635	NDUFS5
1p34.2	1:39838109-3988351C	TRIT1, IPT, COXPD3
1p34.2	1:40040064-40072648	CAP1
1p34.2	1:40071460-40097251	PPT1, CLN1
1p34.2	1:40258077-4029417[illegible]	ZMPSTE24, FACE1, [illegible]
1p34.2	1:40258077-4029417[illegible]	ZMPSTE24, FACE1, [illegible]
1p34.2	1:40300486-40317652	COL9A2, EDM2, STL
1p34.2	1:40300486-40317652	COL9A2, EDM2, STL
1p34.2	1:40783786-4084045ϵ	KCNQ4, DFNA2A
1p34.2	1:40979695-41012564	CTPS1, CTPS, IMD24
1p34.2	1:41478774-41484698	EDN2
1p34.2	1:42153409-4215581[illegible]	GUCA2B, UGN
1p34.2	1:42456340-4247338C	PPCS, CMD2C
1p34.2	1:42733092-42740253	CLDN19, HOMG5
1p34.2	1:42746334-42767083	P3H1, LEPRE1, GRO[illegible]
1p34.2	1:42817121-4284499C	ERMAP, SC, RD
1p34.2	1:42817121-4284499C	ERMAP, SC, RD

1p34.2	1:42925352-42958867	SLC2A1, GLUT1, HT[illegible]
1p34.2	1:42925352-42958867	SLC2A1, GLUT1, HT[illegible]
1p34.2	1:42925352-42958867	SLC2A1, GLUT1, HT[illegible]
1p34.2	1:42925352-42958867	SLC2A1, GLUT1, HT[illegible]
1p34.2	1:42925352-42958867	SLC2A1, GLUT1, HT[illegible]
1p34.2	1:43336874-43354465	MPL, TPOR, MPLV, [illegible]
1p34.2	1:43336874-43354465	MPL, TPOR, MPLV, [illegible]
1p34.2	1:43336874-43354465	MPL, TPOR, MPLV, [illegible]
1p34.2	1:43363396-43368073	ELOVL1, SSC1, IKSH[illegible]
1p34.2	1:43522237-43623671	PTPRF, LAR, BNAH2
1p34.1-p32	1:43700000-6080000[illegible]	PTOS1
1p34.1	1:44819843-44843252	PTCH2
1p34.1	1:44819843-44843252	PTCH2
1p34.1	1:44819843-44843252	PTCH2
1p34.1	1:44850521-44986721	EIF2B3
1p34.1	1:45012253-45015574	UROD
1p34.1	1:45012253-45015574	UROD
1p34.1	1:45329241-4534046[illegible]	MUTYH, MYH
1p34.1	1:45329241-4534046[illegible]	MUTYH, MYH
1p34.1	1:45500228-45513381	MMACHC
1p34.1	1:45511034-4552288[illegible]	PRDX1, PRXI, PAGA
1p34.1	1:46188680-46220304	POMGNT1, MEB, MI[illegible]
1p34.1	1:46188680-46220304	POMGNT1, MEB, MI[illegible]
1p34.1	1:46188680-46220304	POMGNT1, MEB, MI[illegible]
1p34.1	1:46188680-46220304	POMGNT1, MEB, MI[illegible]
1p34.1	1:46247687-4627847[illegible]	RAD54L, HR54, HRA[illegible]
1p34.1	1:46247687-4627847[illegible]	RAD54L, HR54, HRA[illegible]
1p34.1	1:46247687-4627847[illegible]	RAD54L, HR54, HRA[illegible]
1p33	1:46929176-46941475	CYP4A11
1p33	1:47137424-47149737	CYP4A22
1p33	1:47216289-4723238[illegible]	TAL1, TCL5, SCL
1p33	1:47333789-4737883[illegible]	CMPK1, CMPK, UM[illegible]
1p33	1:47416284-47418051	FOXE3, FKHL12, AS[illegible]
1p33	1:47416284-47418051	FOXE3, FKHL12, AS[illegible]
1p33	1:47416284-47418051	FOXE3, FKHL12, AS[illegible]
1p33-p32	1:50048054-50203771	ELAVL4, HUD, PNE[illegible]
1p32	1:50200000-6080000[illegible]	PARK10, AAOPD
1p32.3	1:50437027-5096026[illegible]	FAF1, HFAF1
1p32.3	1:51236272-5127344[illegible]	RNF11
1p32.3	1:53196823-5321419[illegible]	CPT2, IIAE4
1p32.3	1:53196823-5321419[illegible]	CPT2, IIAE4
1p32.3	1:53196823-5321419[illegible]	CPT2, IIAE4
1p32.3	1:53196823-5321419[illegible]	CPT2, IIAE4
1p32.3	1:53242363-5332806[illegible]	LRP8, APOER2, MCI
1p32.3	1:53894186-53911085	DIO1, TXDI1
1p32.3	1:54026680-54053572	TMEM59, DCF1
1p32.3	1:54132719-5415318[illegible]	CDCP2

1p32.3	1:54756897-54764522	PARS2, EIEE75
1p32.3	1:54849626-54887194	DHCR24, KIAA0018
1p32.3	1:54998932-55017171	BSND
1p32.3	1:54998932-55017171	BSND
1p32.3	1:55039547-55064852	PCSK9, NARC1, HCH
1p32.3	1:55039547-55064852	PCSK9, NARC1, HCH
1p32.3	1:55066358-55215373	USP24, KIAA1057
1p32.2	1:56854795-56918222	C8A
1p32.2	1:56929206-56974382	C8B
1p32.2	1:56994777-58250546	DAB1, SCA37
1p32.1	1:58575432-58577251	TACSTD2, TROP2, M
1p32.1	1:58780790-58784046	JUN
1p32.1	1:59814921-59876369	HOOK1, HK1
1p31-p21	1:60800000-10670000	AVSD1, AVCD
1p31.3	1:60800000-68500000	OPHLC, C1DUPp31.3
1p31	1:60800000-84400000	PAOD1
1p31.3-p31.1	1:60800000-84400000	POROK5, DSAP3
1p31.3	1:62597519-62606312	ANGPTL3, ANGPT5,
1p31.3	1:63322566-63325127	FOXD3, AIS1, VAMA
1p31.3	1:63593410-63660244	PGM1, GSD14, CDG1
1p31.3	1:65058433-65058507	MIR101-1, MIRN101-
1p31.3	1:65264693-65415870	DNAJC6, DJC6, KIAA
1p31.3	1:65264693-65415870	DNAJC6, DJC6, KIAA
1p31.3	1:65420651-65641558	LEPR, OBR, LEPRD
1p31.3	1:66999349-67054422	SLC35D1, UGTREL7,
1p31.3	1:67138638-67259978	IL23R, IBD17
1p31.3	1:67138638-67259978	IL23R, IBD17
1p31.3	1:67307350-67398723	IL12RB2
1p31.3	1:68428821-68450321	RPE65, RP20, LCA2
1p31.3	1:68428821-68450321	RPE65, RP20, LCA2
1p31.3	1:68428821-68450321	RPE65, RP20, LCA2
1p31.2	1:68500000-69300000	PBC3
1p31.1-p21.1	1:69300000-10670000	SPG29
1p31.1	1:70411217-70441948	CTH
1p31.1	1:70411217-70441948	CTH
1p31.1	1:70852357-71047815	PTGER3, EP3
1p31.1	1:74235386-74544427	TNNI3K, CCDD
1p31.1	1:75724346-75763678	ACADM, MCAD
1p31.1	1:77888514-77948642	NEXN, NELIN, CMD
1p31.1	1:77888514-77948642	NEXN, NELIN, CMD
1p22	1:84400000-94300000	UOX

Gene/Locus name	Gene/Locus MIM number
Alzheimer disease neuronal thread protein	607413
Basal cell carcinoma, susceptibility to, 1	605462
Cutaneous malignant melanoma/dysplastic nevus	155600
Cataract, congenital, Volkmann type	115665
Chromosome 1p36 deletion syndrome	607872
Inflammatory bowel disease 7	605225
Multiple sclerosis, susceptibility to, 4	612596
Psoriasis susceptibility 7	605606
ISG15 ubiquitin-like modifier	147571
Agrin	103320
Tumor necrosis factor receptor superfamily, member 4	600315
UDP-Gal:beta-Gal beta-1,3-galactosyltransferase polypeptide 6	615291
UDP-Gal:beta-Gal beta-1,3-galactosyltransferase polypeptide 6	615291
UDP-Gal:beta-Gal beta-1,3-galactosyltransferase polypeptide 6	615291
Sodium channel, voltage-gated, type I, delta polypeptide	601328
Dishevelled segment polarity protein 1	601365
ATPase family, AAA domain-containing, member 3A	612316
ATPase family, AAA domain-containing, member 3A	612316
Guanine nucleotide-binding protein, beta polypeptide-1	139380
Guanine nucleotide-binding protein, beta polypeptide-1	139380
SKI proto-oncogene	164780
Peroxisome biogenesis factor 10	602859
Peroxisome biogenesis factor 10	602859
Tumor necrosis factor receptor superfamily, member 14 (herpesvirus entry media	602746
PR domain-containing protein 16	605557
PR domain-containing protein 16	605557
p53-related protein	601990
Small integral membrane protein 1	615242
Nephrocystin 4	607215
Nephrocystin 4	607215
Isoprenylcysteine carboxylmethyltransferase	605851
Espin	606351
Espin	606351
Espin	606351
Tumor necrosis factor receptor superfamily, member 25	603366
Pleckstrin homology domain- and RhoGEF domain-containing protein G5	611101
Pleckstrin homology domain- and RhoGEF domain-containing protein G5	611101
Schizophrenia 12	608543
Tumor necrosis factor receptor superfamily, member 9 (interleukin-activated rece	602250
Oncogene DJ-1	602533
Mitogen-inducible gene 6	608069
Enolase-1, alpha	172430
Micro RNA 34A	611172
Hexose-6-phosphate dehydrogenase	138090
Phosphatidylinositol 3-kinase, catalytic, 110kD, delta	602839
Nicotinamide nucleotide adenylyltransferase 1	608700

Kinesin family member 1B	605995
Kinesin family member 1B	605995
Kinesin family member 1B	605995
Cortistatin	602784
TAR DNA-binding protein	605078
TAR DNA-binding protein	605078
Mannan-binding lectin serine protease 2	605102
Mechanistic target of rapamycin	601231
Mechanistic target of rapamycin	601231
UbiA prenyltransferase domain-containing protein 1	611632
Methylenetetrahydrofolate reductase	607093
Methylenetetrahydrofolate reductase	607093
Methylenetetrahydrofolate reductase	607093
Methylenetetrahydrofolate reductase	607093
Methylenetetrahydrofolate reductase	607093
Chloride channel 6	602726
Natriuretic peptide precursor A	108780
Natriuretic peptide precursor A	108780
Natriuretic peptide precursor B	600295
Procollagen-lysine, 2-oxoglutarate 5-dioxygenase (lysine hydroxylase)	153454
Mitofusin 2	608507
Mitofusin 2	608507
Mitofusin 2	608507
Tumor necrosis factor receptor superfamily, member 8 (CD30 antigen; Ki-1 antig	153243
Tumor necrosis factor receptor superfamily, member 1B	191191
Gallbladder disease 2	609918
EF-hand domain family, member D2	616450
Chymotrypsin	601405
Chymotrypsin-like elastase family, member 2A	609443
Pleckstrin homology domain-containing protein, family M, member 2	609613
Aneurysm, intracranial berry, 3	609122
Chloride channel, kidney, A	602024
Chloride channel, kidney, B	602023
Chloride channel, kidney, B	602023
Ephrin receptor EphA2	176946
ATPase, type 13A2	610513
ATPase, type 13A2	610513
Succinate dehydrogenase complex, subunit B, iron sulfur (Ip)	185470
Succinate dehydrogenase complex, subunit B, iron sulfur (Ip)	185470
Succinate dehydrogenase complex, subunit B, iron sulfur (Ip)	185470
Succinate dehydrogenase complex, subunit B, iron sulfur (Ip)	185470
Peptidylarginine deiminase, type IV	605347
Peptidylarginine deiminase, type VI	610363
Paired box homeotic gene-7	167410
Paired box homeotic gene-7	167410
Aldehyde dehydrogenase 4 family, member A1 (delta-1-pyrroline 5-carboxylate d	606811
Endoplasmic reticulum membrane protein complex, subunit 1	616846

Phospholipase A2, group IIA, platelets, synovial fluid	172411
Phospholipase A2, group V	601192
PTEN-induced putative kinase 1	608309
Dolichyl-diphosphooligosaccharide-protein glycosyltransferase	602202
Endothelin converting enzyme 1	600423
Endothelin converting enzyme 1	600423
Alkaline phosphatase, liver/bone/kidney	171760
Alkaline phosphatase, liver/bone/kidney	171760
Alkaline phosphatase, liver/bone/kidney	171760
Alkaline phosphatase, liver/bone/kidney	171760
Heparan sulfate proteoglycan of basement membrane (perlecan)	142461
Heparan sulfate proteoglycan of basement membrane (perlecan)	142461
Cell division cycle 42 (GTP-binding protein, 25kD)	116952
Wingless-type MMTV integration site family, member 4	603490
Wingless-type MMTV integration site family, member 4	603490
Complement component 1, q subcomponent, A chain	120550
Complement component 1, q subcomponent, C chain	120575
Complement component 1, q subcomponent, B chain	120570
eph tyrosine kinase 3 (ephrin receptor EphB2)	600997
eph tyrosine kinase 3 (ephrin receptor EphB2)	600997
Lysine-specific demethylase 1A	609132
Heterogeneous nuclear ribonucleoprotein R	607201
Inhibitor of DNA binding 3, dominant negative, helix-loop-helix protein	600277
Ribosomal protein L11	604175
UDP galactose-4-epimerase	606953
3-hydroxy-3-methylglutaryl-Coenzyme A lyase	613898
Fucosidase, alpha-L- 1, tissue	612280
Cannabinoid receptor 2	605051
Myomesin 3	616832
Grainyhead-like 3	608317
Runt-related transcription factor 3	600210
Rhesus system D polypeptide	111680
Rhesus system C and E polypeptides	111700
Rhesus system C and E polypeptides	111700
Low density lipoprotein receptor adaptor protein 1	605747
Selenoprotein N	606210
Selenoprotein N	606210
Stathmin	151442
CD52 molecule	114280
Dehydrodolichyl diphosphate synthase	608172
Dehydrodolichyl diphosphate synthase	608172
Dehydrodolichyl diphosphate synthase	608172
Phosphatidylinositol glycan, class V	610274
G-patch domain-containing protein 3	617486
Nuclear receptor subfamily 0, group B, member 2	604630
Keratinocyte differentiation factor 1	616758
Solute carrier family 9 (sodium/hydrogen exchanger), member 1 (antiporter, Na+/	107310

Ficolin 3	604973
G protein-coupled receptor-3	600241
Sphingolyelin phosphodiesterase, acid-like, 3B	617737
Platelet-activating factor receptor	173393
Matrilin 1, cartilage matrix protein	115437
Syndecan 3	186357
Pumilio RNA binding family member 1	607204
Coiled-coil domain-containing protein 28B	610162
Histone deacetylase-1	601241
Syncoilin 1	611750
Tyrosyl-tRNA synthetase 1	603623
Fibronectin type III domain-containing protein 5	611906
Hippocalcin, 23kD, Ca2+-binding protein	142622
Adenylate kinase-2, mitochondrial	103020
Tripartite motif-containing protein 62	616755
Gallbladder disease 3	609919
Gap junction protein, beta-4	605425
Gap junction protein, beta-3	603324
Gap junction protein, beta-3	603324
Gap junction protein, beta-3	603324
Gap junction protein, beta-3	603324
Gap junction protein, beta-3	603324
Gap junction protein, alpha-4, 37kD (connexin 37)	121012
Collagen VIII, alpha-2 polypeptide	120252
Collagen VIII, alpha-2 polypeptide	120252
Zinc finger CCCH domain-containing protein 12A	610562
SMAD nuclear interacting protein 1	608241
R-spondin 1	609595
R-spondin 1	609595
UTP11-like protein	609440
NADH-ubiquinone oxidoreductase subunit S5	603847
tRNA isopentenyltransferase 1	617840
Cyclase-associated actin cytoskeleton regulatory protein 1	617801
Palmitoyl-protein thioesterase 1	600722
Zinc metalloproteinase STE24	606480
Zinc metalloproteinase STE24	606480
Collagen IX, alpha-2 polypeptide	120260
Collagen IX, alpha-2 polypeptide	120260
Potassium voltage-gated channel, KQT-like subfamily, member 4	603537
Cytidine 5'-triphosphate synthetase 1	123860
Endothelin-2	131241
Guanylate cyclase activator 2B (uroguanylin)	601271
Phosphopantothenoylcysteine synthetase	609853
Claudin 19	610036
Prolyl 3-hydroxylase 1	610339
Erythroblast membrane-associated protein	609017
Erythroblast membrane-associated protein	609017

Solute carrier family 2 (facilitated glucose transporter), member 1	138140
Solute carrier family 2 (facilitated glucose transporter), member 1	138140
Solute carrier family 2 (facilitated glucose transporter), member 1	138140
Solute carrier family 2 (facilitated glucose transporter), member 1	138140
Solute carrier family 2 (facilitated glucose transporter), member 1	138140
MPL proto-oncogene (thrombopoietin receptor)	159530
MPL proto-oncogene (thrombopoietin receptor)	159530
MPL proto-oncogene (thrombopoietin receptor)	159530
Elongation of very long chain fatty acids-like 1	611813
Protein tyrosine phosphatase, receptor type, f polypeptide	179590
Ptosis, congenital 1, autosomal dominant	178300
Patched 2	603673
Patched 2	603673
Patched 2	603673
Eukaryotic translation initiation factor 2B, subunit 3	606273
Uroporphyrinogen decarboxylase	613521
Uroporphyrinogen decarboxylase	613521
mutY DNA glycosylase	604933
mutY DNA glycosylase	604933
Metabolism of cobalamin associated C	609831
Peroxiredoxin 1	176763
Protein 0-mannose beta-1,2-N-acetylglucosaminyltransferase	606822
Protein 0-mannose beta-1,2-N-acetylglucosaminyltransferase	606822
Protein 0-mannose beta-1,2-N-acetylglucosaminyltransferase	606822
Protein 0-mannose beta-1,2-N-acetylglucosaminyltransferase	606822
RAD54 like	603615
RAD54 like	603615
RAD54 like	603615
Cytochrome P450, subfamily IVA, polypeptide 11	601310
Cytochrome P450, family 4, subfamily A, polypeptide 22	615341
T-cell acute lymphocytic leukemia-1	187040
Cytidine monophosphate (UMP-CMP) kinase 1, cytosolic	191710
Forkhead box E3	601094
Forkhead box E3	601094
Forkhead box E3	601094
ELAV-like RNA binding protein 4 (Hu antigen D)	168360
Parkinson disease 10	606852
Fas-associated factor 1	604460
Ring finger protein 11	612598
Carnitine palmitoyltransferase II	600650
Carnitine palmitoyltransferase II	600650
Carnitine palmitoyltransferase II	600650
Carnitine palmitoyltransferase II	600650
Low density lipoprotein receptor-related protein 8 (Apolipoprotein E receptor 2)	602600
Deiodinase, iodothyronine, type I	147892
Transmembrane protein 59	617084
Cub domain-containing protein 2	612320

Prolyl-tRNA synthetase 2	612036
24-dehydrocholesterol reductase	606418
Barttin	606412
Barttin	606412
Proprotein convertase, subtilisin/kexin-type, 9	607786
Proprotein convertase, subtilisin/kexin-type, 9	607786
Ubiquitin-specific protease 24	610569
Complement component-8, alpha polypeptide	120950
Complement component-8, beta polypeptide	120960
DAB adaptor protein 1	603448
Tumor-associated calcium signal transducer 2	137290
Jun proto-oncogene, AP-1 transcription factor subunit	165160
Hook microtubule tethering protein 1	607820
Atrioventricular septal defect, susceptibility to, 1	606215
Omphalocele due to duplication of 1p31.3	164750
Peripheral arterial occlusive disease 1	606787
Porokeratosis 5, disseminated superficial actinic	612293
Angiopoietin-like 3	604774
Forkhead box D3	611539
Phosphoglucomutase-1	171900
Micro RNA 101-1	612511
DNAJ heat shock protein family (Hsp40) member C6	608375
DNAJ heat shock protein family (Hsp40) member C6	608375
Leptin receptor	601007
Solute carrier family 35 (UDP-glucuronic acid/UDP-N-acetylgalactosamine dual	610804
Interleukin 23 receptor	607562
Interleukin 23 receptor	607562
Interleukin 12 receptor, beta-2	601642
RPE65 retinoid isomerohydrolase	180069
RPE65 retinoid isomerohydrolase	180069
RPE65 retinoid isomerohydrolase	180069
Biliary cirrhosis, primary, 3	613008
Spastic paraplegia 29, autosomal dominant	609727
Cystathionine gamma-lyase	607657
Cystathionine gamma-lyase	607657
Prostaglandin E receptor 3, EP3 subtype	176806
TNNI3-interacting kinase	613932
Acyl-Coenzyme A dehydrogenase, C-4 to C-12 straight chain	607008
Nexilin F-actin binding protein	613121
Nexilin F-actin binding protein	613121
Urate oxidase, pseudogene	191540

Approved	Entrez Gene I	Ensembl Gene ID	Comments
BCC1	100307118		associated with rs7538
CMM	1243		some linkage studies n
CCV	7792		linked to Rh in Scottisl
			contiguous gene deletic
IBD7	57042		associated with rs6426
MS4	100271696		associated with rs1049
PSORS7	65245		
ISG15	9636	ENSG00000187608,ENST00000649529.1	
AGRN	375790	ENSG00000188157,ENST00000620552.4	
TNFRSF4	7293	ENSG00000186827,ENST00000379236.4	mutation identified in 1
B3GALT6	126792	ENSG00000176022,ENST00000379198.5	
B3GALT6	126792	ENSG00000176022,ENST00000379198.5	
B3GALT6	126792	ENSG00000176022,ENST00000379198.5	
SCNN1D	6339	ENSG00000162572,ENST00000379116.10	
DVL1	1855	ENSG00000107404,ENST00000378888.10	
ATAD3A	55210	ENSG00000197785,ENST00000378756.8	one family with AR inl
ATAD3A	55210	ENSG00000197785,ENST00000378756.8	one family with AR inl
GNB1	2782	ENSG00000078369,ENST00000615252.4	
GNB1	2782	ENSG00000078369,ENST00000615252.4	
SKI	6497	ENSG00000157933,ENST00000378536.5	formerly mapped to 1q
PEX10	5192	ENSG00000157911,ENST00000447513.6	
PEX10	5192	ENSG00000157911,ENST00000447513.6	
TNFRSF1	8764	ENSG00000157873,ENST00000426449.5	
PRDM16	63976	ENSG00000142611,ENST00000270722.10	
PRDM16	63976	ENSG00000142611,ENST00000270722.10	
TP73	7161	ENSG00000078900,ENST00000378288.8	imprinted
SMIM1	388588	ENSG00000235169,ENST00000444870.7	
NPHP4	261734	ENSG00000131697,ENST00000478423.6	
NPHP4	261734	ENSG00000131697,ENST00000478423.6	
ICMT	23463	ENSG00000116237,ENST00000343813.10	
ESPN	83715	ENSG00000187017,ENST00000645284.1	mutation identified in 1
ESPN	83715	ENSG00000187017,ENST00000645284.1	mutation identified in 1
ESPN	83715	ENSG00000187017,ENST00000645284.1	mutation identified in 1
TNFRSF2	8718	ENSG00000215788,ENST00000453341.1	
PLEKHG5	57449	ENSG00000171680,ENST00000400915.7	
PLEKHG5	57449	ENSG00000171680,ENST00000400915.7	
SCZD12	619488		max lod at D1S1612
TNFRSF9	3604	ENSG00000049249,ENST00000377507.7	
PARK7	11315	ENSG00000116288,ENST00000493678.5	
ERRFI1	54206	ENSG00000116285,ENST00000377482.10	
ENO1	2023	ENSG00000074800,ENST00000464920.2	
MIR34A	407040	ENSG00000284357,ENST00000385130.1	
H6PD	9563	ENSG00000049239,ENST00000602477.1	digenic triallelic mutati
PIK3CD	5293	ENSG00000171608,ENST00000377346.9	
NMNAT1	64802	ENSG00000173614,ENST00000403197.5	

KIF1B	23095	ENSG00000054523,ENST00000377086.5	mutation identified in 1
KIF1B	23095	ENSG00000054523,ENST00000377086.5	mutation identified in 1
KIF1B	23095	ENSG00000054523,ENST00000377086.5	mutation identified in 1
CORT	1325	ENSG00000241563,ENST00000377049.3	
TARDBP	23435	ENSG00000120948,ENST00000240185.7	pseudogenes on 2, 6, 8
TARDBP	23435	ENSG00000120948,ENST00000240185.7	pseudogenes on 2, 6, 8
MASP2	10747	ENSG00000009724,ENST00000400897.8	
MTOR	2475	ENSG00000198793,ENST00000361445.9	
MTOR	2475	ENSG00000198793,ENST00000361445.9	
UBIAD1	29914	ENSG00000120942,ENST00000376804.2	
MTHFR	4524	ENSG00000177000,ENST00000376583.7	
MTHFR	4524	ENSG00000177000,ENST00000376583.7	
MTHFR	4524	ENSG00000177000,ENST00000376583.7	
MTHFR	4524	ENSG00000177000,ENST00000376583.7	
MTHFR	4524	ENSG00000177000,ENST00000376583.7	
CLCN6	1185	ENSG00000011021,ENST00000312413.10	
NPPA	4878	ENSG00000175206,ENST00000376480.7	
NPPA	4878	ENSG00000175206,ENST00000376480.7	
NPPB	4879	ENSG00000120937,ENST00000376468.4	
PLOD1	5351	ENSG00000083444,ENST00000196061.5	
MFN2	9927	ENSG00000116688,ENST00000444836.5	
MFN2	9927	ENSG00000116688,ENST00000444836.5	
MFN2	9927	ENSG00000116688,ENST00000444836.5	
TNFRSF8	943	ENSG00000120949,ENST00000263932.7	
TNFRSF1	7133	ENSG00000028137,ENST00000376259.7	
GBD2	100048905		between D1S1597 and
EFHD2	79180	ENSG00000142634,ENST00000375980.9	
CTRC	11330	ENSG00000162438,ENST00000375949.5	
CELA2A	63036	ENSG00000142615,ENST00000359621.5	
PLEKHM2	23207	ENSG00000116786,ENST00000375799.8	
ANIB3	497657		
CLCNKA	1187	ENSG00000186510,ENST00000375692.5	11kb from CLCNKB;
CLCNKB	1188	ENSG00000184908,ENST00000375679.8	unequal crossingover v
CLCNKB	1188	ENSG00000184908,ENST00000375679.8	unequal crossingover v
EPHA2	1969	ENSG00000142627,ENST00000358432.8	
ATP13A2	23400	ENSG00000159363,ENST00000452699.5	mutation identified in 1
ATP13A2	23400	ENSG00000159363,ENST00000452699.5	mutation identified in 1
SDHB	6390	ENSG00000117118,ENST00000375499.8	1 of 2 polypeptides
SDHB	6390	ENSG00000117118,ENST00000375499.8	1 of 2 polypeptides
SDHB	6390	ENSG00000117118,ENST00000375499.8	1 of 2 polypeptides
SDHB	6390	ENSG00000117118,ENST00000375499.8	1 of 2 polypeptides
PADI4	23569	ENSG00000159339,ENST00000375448.4	
PADI6	353238	ENSG00000276747,ENST00000619609.1	
PAX7	5081	ENSG00000009709,ENST00000420770.7	fused with FKHR in rh
PAX7	5081	ENSG00000009709,ENST00000420770.7	fused with FKHR in rh
ALDH4A1	8659	ENSG00000159423,ENST00000538309.5	
EMC1	23065	ENSG00000127463,ENST00000375199.7	

PLA2G2A	5320	ENSG00000188257,ENST00000375111.7	germline mutation iden
PLA2G5	5322	ENSG00000127472,ENST00000375108.4	
PINK1	65018	ENSG00000158828,ENST00000321556.5	
DDOST	1650	ENSG00000244038,ENST00000602624.6	mutation (cmpd het) id
ECE1	1889	ENSG00000117298,ENST00000357071.8	mutation identified in 1
ECE1	1889	ENSG00000117298,ENST00000357071.8	mutation identified in 1
ALPL	249	ENSG00000162551,ENST00000374840.8	
ALPL	249	ENSG00000162551,ENST00000374840.8	
ALPL	249	ENSG00000162551,ENST00000374840.8	
ALPL	249	ENSG00000162551,ENST00000374840.8	
HSPG2	3339	ENSG00000142798,ENST00000374695.8	
HSPG2	3339	ENSG00000142798,ENST00000374695.8	
CDC42	998	ENSG00000070831,ENST00000344548.7	
WNT4	54361	ENSG00000162552,ENST00000290167.11	mutation identified in 1
WNT4	54361	ENSG00000162552,ENST00000290167.11	mutation identified in 1
C1QA	712	ENSG00000173372,ENST00000402322.1	
C1QC	714	ENSG00000159189,ENST00000374639.7	
C1QB	713	ENSG00000173369,ENST00000314933.6	
EPHB2	2048	ENSG00000133216,ENST00000400191.7	mutation identified in 1
EPHB2	2048	ENSG00000133216,ENST00000400191.7	mutation identified in 1
KDM1A	23028	ENSG00000004487,ENST00000400181.8	
HNRNPR	10236	ENSG00000125944,ENST00000374616.7	
ID3	3399	ENSG00000117318,ENST00000374561.6	
RPL11	6135	ENSG00000142676,ENST00000643754.2	
GALE	2582	ENSG00000117308,ENST00000374497.7	
HMGCL	3155	ENSG00000117305,ENST00000374490.8	
FUCA1	2517	ENSG00000179163,ENST00000374479.3	8cM distal to RH; pseu
CNR2	1269	ENSG00000188822,ENST00000374472.5	
MYOM3	127294	ENSG00000142661,ENST00000374434.4	
GRHL3	57822	ENSG00000158055,ENST00000356046.6	
RUNX3	864	ENSG00000020633,ENST00000399916.5	
RHD	6007	ENSG00000187010,ENST00000454452.6	
RHCE	6006	ENSG00000188672,ENST00000413854.5	?order: C-E-D
RHCE	6006	ENSG00000188672,ENST00000413854.5	?order: C-E-D
LDLRAP1	26119	ENSG00000157978,ENST00000374338.5	
SELENON	57190	ENSG00000162430,ENST00000361547.7	
SELENON	57190	ENSG00000162430,ENST00000361547.7	
STMN1	3925	ENSG00000117632,ENST00000426559.6	
CD52	1043	ENSG00000169442,ENST00000374213.3	
DHDDS	79947	ENSG00000117682,ENST00000525682.6	mutation identified in 1
DHDDS	79947	ENSG00000117682,ENST00000525682.6	mutation identified in 1
DHDDS	79947	ENSG00000117682,ENST00000525682.6	mutation identified in 1
PIGV	55650	ENSG00000060642,ENST00000674273.1	
GPATCH3	63906	ENSG00000198746,ENST00000361720.10	
NR0B2	8431	ENSG00000131910,ENST00000254227.4	
KDF1	126695	ENSG00000175707,ENST00000320567.6	mutation identified in 1
SLC9A1	6548	ENSG00000090020,ENST00000263980.8	mutation identified in 1

FCN3	8547	ENSG00000142748,ENST00000270879.8	
GPR3	2827	ENSG00000181773,ENST00000374024.4	
SMPDL3B	27293	ENSG00000130768,ENST00000373888.8	
PTAFR	5724	ENSG00000169403,ENST00000373857.8	
MATN1	4146	ENSG00000162510,ENST00000373765.5	
SDC3	9672	ENSG00000162512,ENST00000339394.7	
PUM1	9698	ENSG00000134644,ENST00000426105.6	
CCDC28B	79140	ENSG00000160050,ENST00000421922.6	
HDAC1	3065	ENSG00000116478,ENST00000373548.8	
SYNC	81493	ENSG00000162520,ENST00000373484.4	
YARS1	8565	ENSG00000134684,ENST00000373477.8	
FNDC5	252995	ENSG00000160097,ENST00000496770.1	
HPCA	3208	ENSG00000121905,ENST00000373467.4	
AK2	204	ENSG00000004455,ENST00000467905.5	
TRIM62	55223	ENSG00000116525,ENST00000543586.1	
GBD3	100048906		new D2S255
GJB4	127534	ENSG00000189433,ENST00000339480.3	
GJB3	2707	ENSG00000188910,ENST00000373362.3	same YAC as GJA4
GJB3	2707	ENSG00000188910,ENST00000373362.3	same YAC as GJA4
GJB3	2707	ENSG00000188910,ENST00000373362.3	same YAC as GJA4
GJB3	2707	ENSG00000188910,ENST00000373362.3	same YAC as GJA4
GJB3	2707	ENSG00000188910,ENST00000373362.3	same YAC as GJA4
GJA4	2701	ENSG00000187513,ENST00000342280.5	
COL8A2	1296	ENSG00000171812,ENST00000481785.1	
COL8A2	1296	ENSG00000171812,ENST00000481785.1	
ZC3H12A	80149	ENSG00000163874,ENST00000373087.7	
SNIP1	79753	ENSG00000163877,ENST00000296215.8	
RSPO1	284654	ENSG00000169218,ENST00000401068.1	
RSPO1	284654	ENSG00000169218,ENST00000401068.1	
UTP11	51118	ENSG00000183520,ENST00000373014.5	
NDUFS5	4725	ENSG00000168653,ENST00000372967.3	
TRIT1	54802	ENSG00000043514,ENST00000372818.5	
CAP1	10487	ENSG00000131236,ENST00000372797.7	
PPT1	5538	ENSG00000131238,ENST00000642050.1	
ZMPSTE2	10269	ENSG00000084073,ENST00000372759.4	
ZMPSTE2	10269	ENSG00000084073,ENST00000372759.4	
COL9A2	1298	ENSG00000049089,ENST00000372748.8	mutation identified in1
COL9A2	1298	ENSG00000049089,ENST00000372748.8	mutation identified in1
KCNQ4	9132	ENSG00000117013,ENST00000347132.10	
CTPS1	1503	ENSG00000171793,ENST00000649215.1	
EDN2	1907	ENSG00000127129,ENST00000372587.5	vasoactive intestinal co
GUCA2B	2981	ENSG00000044012,ENST00000372581.2	
PPCS	79717	ENSG00000127125,ENST00000372562.1	
CLDN19	149461	ENSG00000164007,ENST00000372539.3	
P3H1	64175	ENSG00000117385,ENST00000397054.7	
ERMAP	114625	ENSG00000164010,ENST00000372517.8	
ERMAP	114625	ENSG00000164010,ENST00000372517.8	

SLC2A1	6513	ENSG00000117394,ENST00000426263.9	probably in 1p33
SLC2A1	6513	ENSG00000117394,ENST00000426263.9	probably in 1p33
SLC2A1	6513	ENSG00000117394,ENST00000426263.9	probably in 1p33
SLC2A1	6513	ENSG00000117394,ENST00000426263.9	probably in 1p33
SLC2A1	6513	ENSG00000117394,ENST00000426263.9	probably in 1p33
MPL	4352	ENSG00000117400,ENST00000372470.9	
MPL	4352	ENSG00000117400,ENST00000372470.9	
MPL	4352	ENSG00000117400,ENST00000372470.9	
ELOVL1	64834	ENSG00000066322,ENST00000621943.4	
PTPRF	5792	ENSG00000142949,ENST00000359947.9	mutation identified in 1
PTOS1	5765		
PTCH2	8643	ENSG00000117425,ENST00000447098.6	
PTCH2	8643	ENSG00000117425,ENST00000447098.6	
PTCH2	8643	ENSG00000117425,ENST00000447098.6	
EIF2B3	8891	ENSG00000070785,ENST00000372183.7	
UROD	7389	ENSG00000126088,ENST00000246337.9	
UROD	7389	ENSG00000126088,ENST00000246337.9	
MUTYH	4595	ENSG00000132781,ENST00000372115.7	
MUTYH	4595	ENSG00000132781,ENST00000372115.7	
MMACHC	25974	ENSG00000132763,ENST00000616135.1	
PRDX1	5052	ENSG00000117450,ENST00000424390.1	pseudogene PAGB on
POMGNT	55624	ENSG00000085998,ENST00000371992.1	
POMGNT	55624	ENSG00000085998,ENST00000371992.1	
POMGNT	55624	ENSG00000085998,ENST00000371992.1	
POMGNT	55624	ENSG00000085998,ENST00000371992.1	
RAD54L	8438	ENSG00000085999,ENST00000442598.5	
RAD54L	8438	ENSG00000085999,ENST00000442598.5	
RAD54L	8438	ENSG00000085999,ENST00000442598.5	
CYP4A11	1579	ENSG00000187048,ENST00000310638.9	
CYP4A22	284541	ENSG00000162365,ENST00000371891.8	
TAL1	6886	ENSG00000162367,ENST00000371884.6	proximal to MYCL1
CMPK1	51727	ENSG00000162368,ENST00000450808.2	
FOXE3	2301	ENSG00000186790,ENST00000335071.4	
FOXE3	2301	ENSG00000186790,ENST00000335071.4	
FOXE3	2301	ENSG00000186790,ENST00000335071.4	
ELAVL4	1996	ENSG00000162374,ENST00000371824.6	
PARK10	170534		max lod at D1S2652; [illegible]
FAF1	11124	ENSG00000185104,ENST00000396153.7	
RNF11	26994	ENSG00000123091,ENST00000242719.4	
CPT2	1376	ENSG00000157184,ENST00000371486.4	formerly at 1p13
CPT2	1376	ENSG00000157184,ENST00000371486.4	formerly at 1p13
CPT2	1376	ENSG00000157184,ENST00000371486.4	formerly at 1p13
CPT2	1376	ENSG00000157184,ENST00000371486.4	formerly at 1p13
LRP8	7804	ENSG00000157193,ENST00000371454.6	
DIO1	1733	ENSG00000211452,ENST00000361921.7	
TMEM59	9528	ENSG00000116209,ENST00000371341.5	
CDCP2	200008	ENSG00000157211,ENST00000530059.2	?associated with PARK

PARS2	25973	ENSG00000162396,ENST00000371279.4	
DHCR24	1718	ENSG00000116133,ENST00000371269.9	
BSND	7809	ENSG00000162399,ENST00000651561.1	
BSND	7809	ENSG00000162399,ENST00000651561.1	
PCSK9	255738	ENSG00000169174,ENST00000302118.5	
PCSK9	255738	ENSG00000169174,ENST00000302118.5	
USP24	23358	ENSG00000162402,ENST00000294383.7	
C8A	731	ENSG00000157131,ENST00000361249.4	
C8B	732	ENSG00000021852,ENST00000371237.9	
DAB1	1600	ENSG00000173406,ENST00000371236.6	
TACSTD2	4070	ENSG00000184292,ENST00000371225.4	
JUN	3725	ENSG00000177606,ENST00000371222.4	
HOOK1	51361	ENSG00000134709,ENST00000371208.5	
AVSD1	7446		duplication of 710kb a
PAOD1	171513		
POROK5	100190982		between D1S438 and I
ANGPTL3	27329	ENSG00000132855,ENST00000371129.4	
FOXD3	27022	ENSG00000187140,ENST00000371116.4	
PGM1	5236	ENSG00000079739,ENST00000371083.4	distal to ACADM; for
MIR101-1	406893	ENSG00000199135,ENST00000362265.1	another locus, MIRN1
DNAJC6	9829	ENSG00000116675,ENST00000371069.5	
DNAJC6	9829	ENSG00000116675,ENST00000371069.5	
LEPR	3953	ENSG00000116678,ENST00000371060.7	
SLC35D1	23169	ENSG00000116704,ENST00000235345.6	
IL23R	149233	ENSG00000162594,ENST00000347310.10	
IL23R	149233	ENSG00000162594,ENST00000347310.10	
IL12RB2	3595	ENSG00000081985,ENST00000371000.5	
RPE65	6121	ENSG00000116745,ENST00000262340.6	
RPE65	6121	ENSG00000116745,ENST00000262340.6	
RPE65	6121	ENSG00000116745,ENST00000262340.6	
PBC3	100303717		associated with rs3790
SPG29	619379		max lod at D1S2865
CTH	1491	ENSG00000116761,ENST00000411986.6	previously assigned to
CTH	1491	ENSG00000116761,ENST00000411986.6	previously assigned to
PTGER3	5733	ENSG00000050628,ENST00000370931.7	
TNNI3K	51086	ENSG00000116783,ENST00000326637.8	
ACADM	34	ENSG00000117054,ENST00000370841.8	
NEXN	91624	ENSG00000162614,ENST00000330010.12	
NEXN	91624	ENSG00000162614,ENST00000330010.12	
UOX	391051		nonsense mutations res

Phenotype
{Basal cell carcinoma, susceptibility to, 1}
{Melanoma, cutaneous malignant, 1}
Cataract 8, multiple types
Chromosome 1p36 deletion syndrome
{Inflammatory bowel disease 7}
{Multiple sclerosis, susceptibility to, 4}
{Psoriasis susceptibility 7}
Immunodeficiency 38
Myasthenic syndrome, congenital, 8, with pre- and postsynaptic defects
?Immunodeficiency 16
Al-Gazali syndrome
Ehlers-Danlos syndrome, spondylodysplastic type, 2
Spondyloepimetaphyseal dysplasia with joint laxity, type 1, with or without fractures
Robinow syndrome, autosomal dominant 2
?Pontocerebellar hypoplasia, hypotonia, and respiratory insufficiency syndrome, neonatal lethal
Harel-Yoon syndrome
Leukemia, acute lymphoblastic, somatic
Mental retardation, autosomal dominant 42
Shprintzen-Goldberg syndrome
Peroxisome biogenesis disorder 6A (Zellweger)
Peroxisome biogenesis disorder 6B
Cardiomyopathy, dilated, 1LL
Left ventricular noncompaction 8
[Blood group, Vel system]
Nephronophthisis 4
Senior-Loken syndrome 4
?Usher syndrome, type 1M
Deafness, autosomal recessive 36
Deafness, neurosensory, without vestibular involvement, autosomal dominant
Charcot-Marie-Tooth disease, recessive intermediate C
Spinal muscular atrophy, distal, autosomal recessive, 4
{Schizophrenia 12}
Parkinson disease 7, autosomal recessive early-onset
Enolase deficiency
Cortisone reductase deficiency 1
Immunodeficiency 14
Leber congenital amaurosis 9

?Charcot-Marie-Tooth disease, type 2A1
{Neuroblastoma, susceptibility to, 1}
Pheochromocytoma

Amyotrophic lateral sclerosis 10, with or without FTD
Frontotemporal lobar degeneration, TARDBP-related
MASP2 deficiency
Focal cortical dysplasia, type II, somatic
Smith-Kingsmore syndrome
Corneal dystrophy, Schnyder type
{Neural tube defects, susceptibility to}
{Schizophrenia, susceptibility to}
{Thromboembolism, susceptibility to}
{Vascular disease, susceptibility to}
Homocystinuria due to MTHFR deficiency

Atrial fibrillation, familial, 6
Atrial standstill 2

Ehlers-Danlos syndrome, kyphoscoliotic type, 1
Charcot-Marie-Tooth disease, axonal, type 2A2A
Charcot-Marie-Tooth disease, axonal, type 2A2B
Hereditary motor and sensory neuropathy VIA

Gallbladder disease 2

{Pancreatitis, chronic, susceptibility to}
Abdominal obesity-metabolic syndrome 4

Aneurysm, intracranial berry, 3
Bartter syndrome, type 4b, digenic
Bartter syndrome, type 3
Bartter syndrome, type 4b, digenic
Cataract 6, multiple types
Kufor-Rakeb syndrome
Spastic paraplegia 78, autosomal recessive
Gastrointestinal stromal tumor
Paraganglioma and gastric stromal sarcoma
Paragangliomas 4
Pheochromocytoma
{Rheumatoid arthritis, susceptibility to}
Preimplantation embryonic lethality 2
Myopathy, congenital, progressive, with scoliosis
Rhabdomyosarcoma 2, alveolar
Hyperprolinemia, type II
Cerebellar atrophy, visual impairment, and psychomotor retardation

{?Colorectal cancer, susceptibility to}
[Fleck retina, familial benign]
Parkinson disease 6, early onset
?Congenital disorder of glycosylation, type Ir
?Hirschsprung disease, cardiac defects, and autonomic dysfunction
{Hypertension, essential, susceptibility to}
Hypophosphatasia, adult
Hypophosphatasia, childhood
Hypophosphatasia, infantile
Odontohypophosphatasia
Dyssegmental dysplasia, Silverman-Handmaker type
Schwartz-Jampel syndrome, type 1
Takenouchi-Kosaki syndrome
?SERKAL syndrome
Mullerian aplasia and hyperandrogenism
C1q deficiency
C1q deficiency
C1q deficiency
?Bleeding disorder, platelet-type, 22
{Prostate cancer/brain cancer susceptibility, somatic}
Cleft palate, psychomotor retardation, and distinctive facial features

Diamond-Blackfan anemia 7
Galactose epimerase deficiency
HMG-CoA lyase deficiency
Fucosidosis

Van der Woude syndrome 2

[Rh-negative blood type]
[Blood group, Rhesus]
Rh-null disease, amorph type
Hypercholesterolemia, familial, 4
Muscular dystrophy, rigid spine, 1
Myopathy, congenital, with fiber-type disproportion

?Congenital disorder of glycosylation, type 1bb
Developmental delay and seizures with or without movement abnormalities
Retinitis pigmentosa 59
Hyperphosphatasia with mental retardation syndrome 1

Obesity, mild, early-onset
?Ectodermal dysplasia 12, hypohidrotic/hair/tooth/nail type
?Lichtenstein-Knorr syndrome

Immunodeficiency due to ficolin 3 deficiency

{Obesity, association with}
Spinocerebellar ataxia 47
{Bardet-Biedl syndrome 1, modifier of}

Charcot-Marie-Tooth disease, dominant intermediate C

Dystonia 2, torsion, autosomal recessive
Reticular dysgenesis

Gallbladder disease 3
Erythrokeratodermia variabilis et progressiva 2
Deafness, autosomal dominant 2B
Deafness, autosomal dominant, with peripheral neuropathy
Deafness, autosomal recessive
Deafness, digenic, GJB2/GJB3
Erythrokeratodermia variabilis et progressiva 1

Corneal dystrophy, Fuchs endothelial, 1
Corneal dystrophy, posterior polymorphous 2

Psychomotor retardation, epilepsy, and craniofacial dysmorphism
Palmoplantar hyperkeratosis and true hermaphroditism
Palmoplantar hyperkeratosis with squamous cell carcinoma of skin and sex reversal

Combined oxidative phosphorylation deficiency 35

Ceroid lipofuscinosis, neuronal, 1
Mandibuloacral dysplasia with type B lipodystrophy
Restrictive dermopathy, lethal
?Stickler syndrome, type V
Epiphyseal dysplasia, multiple, 2
Deafness, autosomal dominant 2A
Immunodeficiency 24
ntractor (VIC) = mouse and rat equivalent

Cardiomyopathy, dilated, 2C
Hypomagnesemia 5, renal, with ocular involvement
Osteogenesis imperfecta, type VIII
[Blood group, Radin]
[Blood group, Scianna system]

{Epilepsy, idiopathic generalized, susceptibility to, 12}
Dystonia 9
GLUT1 deficiency syndrome 1, infantile onset, severe
GLUT1 deficiency syndrome 2, childhood onset
Stomatin-deficient cryohydrocytosis with neurologic defects
Myelofibrosis with myeloid metaplasia, somatic
Thrombocythemia 2
Thrombocytopenia, congenital amegakaryocytic
Ichthyotic keratoderma, spasticity, hypomyelination, and dysmorphic facies
?Breasts and/or nipples, aplasia or hypoplasia of, 2
Ptosis, hereditary congenital, 1
Basal cell carcinoma, somatic
Basal cell nevus syndrome
Medulloblastoma, somatic
Leukoencephalopathy with vanishing white matter
Porphyria cutanea tarda
Porphyria, hepatoerythropoietic
Adenomas, multiple colorectal
Gastric cancer, somatic
Methylmalonic aciduria and homocystinuria, cblC type
Methylmalonic aciduria and homocystinuria, cblC type, digenic
Muscular dystrophy-dystroglycanopathy (congenital with brain and eye anomalies), type A, 3
Muscular dystrophy-dystroglycanopathy (congenital with mental retardation), type B, 3
Muscular dystrophy-dystroglycanopathy (limb-girdle), type C, 3
Retinitis pigmentosa 76
{Breast cancer, invasive ductal}
Adenocarcinoma, colonic, somatic
Lymphoma, non-Hodgkin, somatic

Leukemia, T-cell acute lymphocytic, somatic

{Aortic aneurysm, familial thoracic 11, susceptibility to}
Anterior segment dysgenesis 2, multiple subtypes
Cataract 34, multiple types

{Parkinson disease 10}

{Encephalopathy, acute, infection-induced, 4, susceptibility to}
CPT II deficiency, infantile
CPT II deficiency, lethal neonatal
CPT II deficiency, myopathic, stress-induced
{Myocardial infarction, susceptibility to}

[illegible]10

Epileptic encephalopathy, early infantile, 75
Desmosterolosis
Bartter syndrome, type 4a
Sensorineural deafness with mild renal dysfunction
{Low density lipoprotein cholesterol level QTL 1}
Hypercholesterolemia, familial, 3

C8 deficiency, type I
C8 deficiency, type II
Spinocerebellar ataxia 37
Corneal dystrophy, gelatinous drop-like

{Atrioventricular septal defect, susceptibility to, 1}
Omphalocele due to duplication of 1p31.3
Peripheral arterial occlusive disease 1
Porokeratosis 5, disseminated superficial actinic
Hypobetalipoproteinemia, familial, 2
{Autoimmune disease, susceptibility to, 1}
Congenital disorder of glycosylation, type It
)1-2, on 9p24
Parkinson disease 19a, juvenile-onset
Parkinson disease 19b, early-onset
Obesity, morbid, due to leptin receptor deficiency
Schneckenbecken dysplasia
{Inflammatory bowel disease 17, protection against}
{Psoriasis, protection against}

Leber congenital amaurosis 2
Retinitis pigmentosa 20
Retinitis pigmentosa 87 with choroidal involvement
{Biliary cirrhosis, primary, 3}
Spastic paraplegia 29, autosomal dominant
Cystathioninuria
Homocysteine, total plasma, elevated

Cardiac conduction disease with or without dilated cardiomyopathy
Acyl-CoA dehydrogenase, medium chain, deficiency of
Cardiomyopathy, dilated, 1CC
Cardiomyopathy, hypertrophic, 20
[Urate oxidase deficiency]

Phenotype MIM number	Inheritance	Pheno map key	Mouse Gene (from MGI)
605462		2	
155600	Autosomal dominant	2	
115665	Autosomal dominant	2	
607872	Isolated cases	4	
605225		2	
612596		2	
605606		2	
616126	Autosomal recessive	3	Isg15
615120	Autosomal recessive	3	Agrn
615593	Autosomal recessive	3	Tnfrsf4
609465		3	B3galt6
615349	Autosomal recessive	3	B3galt6
271640	Autosomal recessive	3	B3galt6
616331	Autosomal dominant	3	Dvl1
618810	Autosomal recessive	3	Atad3a
617183	Autosomal dominant; Autoso	3	Atad3a
613065		3	Gnb1
616973	Autosomal dominant	3	Gnb1
182212	Autosomal dominant	3	Ski
614870	Autosomal recessive	3	Pex10
614871	Autosomal recessive	3	Pex10
		Tnfrsf14	
615373	Autosomal dominant	3	Prdm16
615373	Autosomal dominant	3	Prdm16
		Trp73	
615264	Autosomal recessive	3	Smim1
606966	Autosomal recessive	3	Nphp4
606996	Autosomal recessive	3	Nphp4
		Icmt	
618632	Autosomal recessive	3	Espn
609006	Autosomal recessive	3	Espn
609006	Autosomal recessive	3	Espn
		Tnfrsf25	
615376	Autosomal recessive	3	Plekhg5
611067	Autosomal recessive	3	Plekhg5
181500	Autosomal dominant	2	
		Tnfrsf9	
606324	Autosomal recessive	3	Park7
		Errfi1	
		1	Eno1b,Eno1
604931	Autosomal recessive	3	H6pd
615513	Autosomal dominant	3	Pik3cd
608553	Autosomal recessive	3	Nmnat1

118210	Autosomal dominant	3	Kif1b
256700	Autosomal dominant; Somati	3	Kif1b
171300	Autosomal dominant	3	Kif1b
		Cort	
612069	Autosomal dominant	3	Tardbp
612069	Autosomal dominant	3	Tardbp
613791	Autosomal recessive	3	Masp2
607341		3	Mtor
616638	Autosomal dominant	3	Mtor
121800	Autosomal dominant	3	Ubiad1
601634	Autosomal recessive	3	Mthfr
181500	Autosomal dominant	3	Mthfr
188050	Autosomal dominant	3	Mthfr
		3	Mthfr
236250	Autosomal recessive	3	Mthfr
		Clcn6	
612201	Autosomal dominant	3	Nppa
615745	Autosomal recessive	3	Nppa
225400	Autosomal recessive	3	Plod1
609260	Autosomal dominant	3	Mfn2
617087	Autosomal recessive	3	Mfn2
601152	Autosomal dominant	3	Mfn2
		Tnfrsf8	
		Tnfrsf1b	
609918		2	
		Efhd2	
167800	Autosomal dominant	3	Ctrc
618620	Autosomal dominant	3	Cela2a
		Plekhm2	
609122	Autosomal dominant	2	
613090	Digenic recessive	3	Clcnkb
607364	Autosomal recessive	3	Clcnka
613090	Digenic recessive	3	Clcnka
116600	Autosomal dominant	3	Epha2
606693	Autosomal recessive	3	Atp13a2
617225	Autosomal recessive	3	Atp13a2
606764	Autosomal dominant; Isolate	3	Sdhb
606864		3	Sdhb
115310	Autosomal dominant	3	Sdhb
171300	Autosomal dominant	3	Sdhb
180300		3	Padi4
617234	Autosomal recessive	3	Padi6
618578	Autosomal recessive	3	Pax7
268220	Somatic mutation	3	Pax7
239510	Autosomal recessive	3	Aldh4a1
616875	Autosomal recessive	3	Emc1

114500	Autosomal dominant; Somati	3	
228980	Autosomal recessive	3	Pla2g5
605909	Autosomal recessive	3	Pink1
614507	Autosomal recessive	3	Ddost
613870	Autosomal dominant	3	Ece1
145500	Multifactorial	3	Ece1
146300	Autosomal dominant; Autoso	3	Alpl
241510	Autosomal recessive	3	Alpl
241500	Autosomal recessive	3	Alpl
146300	Autosomal dominant; Autoso	3	Alpl
224410	Autosomal recessive	3	Hspg2
255800	Autosomal recessive	3	Hspg2
616737	Autosomal dominant	3	
611812	Autosomal recessive	3	Wnt4
158330	Autosomal dominant	3	Wnt4
613652	Autosomal recessive	3	C1qa
613652	Autosomal recessive	3	C1qc
613652	Autosomal recessive	3	C1qb
618462	Autosomal recessive	3	Ephb2
603688		3	Ephb2
616728	Autosomal dominant	3	Kdm1a
		Hnrnpr	
		Id3	
612562	Autosomal dominant	3	Rpl11
230350	Autosomal recessive	3	Gale
246450	Autosomal recessive	3	Hmgcl
230000	Autosomal recessive	3	Fuca1
		Cnr2	
		Myom3	
606713	Autosomal dominant	3	Grhl3
		Runx3	
		3	Rhd
		3	Rhd
617970		3	Rhd
603813	Autosomal recessive	3	Ldlrap1
602771	Autosomal recessive	3	Selenon
255310	Autosomal dominant; Autoso	3	Selenon
		Stmn1	
613861	Autosomal recessive	3	Dhdds
617836	Autosomal dominant	3	Dhdds
613861	Autosomal recessive	3	Dhdds
239300	Autosomal recessive	3	Pigv
		Gpatch3	
601665	Autosomal dominant; Autoso	3	Nr0b2
617337	Autosomal dominant	3	Kdf1
616291	Autosomal recessive	3	Slc9a1

613860	Autosomal recessive	3	
		Gpr3	
		Smpdl3b	
		Ptafr	
		Matn1	
601665	Autosomal dominant; Autoso	3	Sdc3
617931	Autosomal dominant	3	Pum1
209900	Autosomal recessive; Digenic	3	Ccdc28b
		Hdac1	
		Sync	
608323	Autosomal dominant	3	Yars
		Fndc5	
224500	Autosomal recessive	3	Hpca
267500	Autosomal recessive	3	Ak2
		Trim62	
609919		2	
617524	Autosomal dominant	3	Gjb4
612644	Autosomal dominant	3	Gjb3
		3	Gjb3
		3	Gjb3
220290	Autosomal recessive; Digenic	3	Gjb3
133200	Autosomal dominant; Autoso	3	Gjb3
		Gja4	
136800	Autosomal dominant	3	Col8a2
609140	Autosomal dominant	3	Col8a2
		Zc3h12a	
614501	Autosomal recessive	3	Snip1
610644	Autosomal recessive	3	Rspo1
610644	Autosomal recessive	3	Rspo1
		Utp11	
617873	Autosomal recessive	3	Trit1
		Cap1	
256730	Autosomal recessive	3	Ppt1
608612	Autosomal recessive	3	Zmpste24
275210	Autosomal recessive	3	Zmpste24
614284	Autosomal recessive	3	Col9a2
600204	Autosomal dominant	3	Col9a2
600101	Autosomal dominant	3	Kcnq4
615897	Autosomal recessive	3	Ctps
		Edn2	
		Guca2b	
618189	Autosomal recessive	3	Ppcs
248190	Autosomal recessive	3	Cldn19
610915	Autosomal recessive	3	P3h1
111620		3	Ermap
111750		3	Ermap

614847	Autosomal dominant	3	Slc2a1
601042	Autosomal dominant	3	Slc2a1
606777	Autosomal dominant; Autoso	3	Slc2a1
612126	Autosomal dominant	3	Slc2a1
608885	Autosomal dominant	3	Slc2a1
254450		3	Mpl
601977	Autosomal dominant; Somati	3	Mpl
604498	Autosomal recessive	3	Mpl
618527	Autosomal dominant	3	Elovl1
616001	Autosomal recessive	3	Ptprf
178300	Autosomal dominant	2	
605462		3	Ptch2
109400	Autosomal dominant	3	Ptch2
155255		3	Ptch2
603896	Autosomal recessive	3	Eif2b3
176100	Autosomal dominant; Autoso	3	Urod
176100	Autosomal dominant; Autoso	3	Urod
608456	Autosomal recessive	3	Mutyh
613659		3	Mutyh
277400	Autosomal recessive	3	Mmachc
277400	Autosomal recessive	3	Prdx1
253280	Autosomal recessive	3	Pomgnt1
613151	Autosomal recessive	3	Pomgnt1
613157	Autosomal recessive	3	Pomgnt1
617123	Autosomal recessive	3	Pomgnt1
114480	Autosomal dominant; Somati	3	Rad54l
		3	Rad54l
605027		3	Rad54l
		Cyp4a10,Cyp4a32,Cyp4a31	
		Cyp4a12a,Cyp4a12b	
613065		3	Tal1
		Cmpk1	
617349	Autosomal dominant	3	
610256	Autosomal recessive	3	
612968		3	
		Elavl4	
606852		2	
		Faf1	
		Rnf11	
614212	Autosomal dominant; Autoso	3	Cpt2
600649	Autosomal recessive	3	Cpt2
608836	Autosomal recessive	3	Cpt2
255110	Autosomal dominant; Autoso	3	Cpt2
608446		3	Lrp8
		Dio1	
		Tmem59	
		Cdcp2	

618437	Autosomal recessive	3	Pars2
602398	Autosomal recessive	3	Dhcr24
602522	Autosomal recessive	3	Bsnd
602522	Autosomal recessive	3	Bsnd
603776	Autosomal dominant	3	Pcsk9
603776	Autosomal dominant	3	Pcsk9
		Usp24	
613790	Autosomal recessive	3	C8a
613789	Autosomal recessive	3	C8b
615945	Autosomal dominant	3	Dab1
204870	Autosomal recessive	3	Tacstd2
		Jun	
		Hook1	
606215		2	
164750	Isolated cases	4	
606787		2	
612293		2	
605019	Autosomal recessive	3	Angptl3
607836	Autosomal dominant	3	Foxd3
614921	Autosomal recessive	3	Pgm1
615528	Autosomal recessive	3	Dnajc6
615528	Autosomal recessive	3	Dnajc6
614963	Autosomal recessive	3	Lepr
269250	Autosomal recessive	3	Slc35d1
612261		3	Il23r
605606		3	Il23r
		Il12rb2	
204100	Autosomal recessive	3	Rpe65
613794	Autosomal recessive	3	Rpe65
618697	Autosomal dominant	3	Rpe65
613008		2	
609727	Autosomal dominant	2	
219500	Autosomal recessive	3	Cth
		3	Cth
		Ptger3	
616117	Autosomal dominant	3	Tnni3k
201450	Autosomal recessive	3	Acadm
613122	Autosomal dominant	3	Nexn
613876	Autosomal dominant	3	Nexn
		1	

Table S2 Targets associtaetd with Parkinson's disease

Table S2-3 Targets obtained from GeneCards

Relavance score ⩾ 1

Gene Sym	Description	Category	Gifts
SNCA	Synuclein Alpha	Protein Coding	51
LRRK2	Leucine Rich Repeat Kinase 2	Protein Coding	51
PRKN	Parkin RBR E3 Ubiquitin Protein Ligase	Protein Coding	41
PARK7	Parkinsonism Associated Deglycase	Protein Coding	47
PINK1	PTEN Induced Kinase 1	Protein Coding	48
MAPT	Microtubule Associated Protein Tau	Protein Coding	52
GBA	Glucosylceramidase Beta	Protein Coding	49
ATP13A2	ATPase Cation Transporting 13A2	Protein Coding	43
APOE	Apolipoprotein E	Protein Coding	52
SLC6A3	Solute Carrier Family 6 Member 3	Protein Coding	51
APP	Amyloid Beta Precursor Protein	Protein Coding	52
UCHL1	Ubiquitin C-Terminal Hydrolase L1	Protein Coding	53
TH	Tyrosine Hydroxylase	Protein Coding	53
SNCAIP	Synuclein Alpha Interacting Protein	Protein Coding	44
VPS35	VPS35 Retromer Complex Component	Protein Coding	45
PSEN1	Presenilin 1	Protein Coding	53
PRNP	Prion Protein	Protein Coding	48
SYNJ1	Synaptojanin 1	Protein Coding	47
TNF	Tumor Necrosis Factor	Protein Coding	53
PLA2G6	Phospholipase A2 Group VI	Protein Coding	50
IL6	Interleukin 6	Protein Coding	51
BDNF	Brain Derived Neurotrophic Factor	Protein Coding	48
SOD1	Superoxide Dismutase 1	Protein Coding	54
HTRA2	HtrA Serine Peptidase 2	Protein Coding	48
FBXO7	F-Box Protein 7	Protein Coding	42
PRKAG2	Protein Kinase AMP-Activated Non-Catalytic Subunit (	Protein Coding	51
COMT	Catechol-O-Methyltransferase	Protein Coding	52
GIGYF2	GRB10 Interacting GYF Protein 2	Protein Coding	39
HTT	Huntingtin	Protein Coding	45
VCP	Valosin Containing Protein	Protein Coding	50
GDNF	Glial Cell Derived Neurotrophic Factor	Protein Coding	49
GCH1	GTP Cyclohydrolase 1	Protein Coding	47
DRD2	Dopamine Receptor D2	Protein Coding	52
SQSTM1	Sequestosome 1	Protein Coding	50
IL10	Interleukin 10	Protein Coding	49
NEFL	Neurofilament Light	Protein Coding	47
POLG	DNA Polymerase Gamma, Catalytic Subunit	Protein Coding	47
C9orf72	C9orf72-SMCR8 Complex Subunit	Protein Coding	42
ATXN2	Ataxin 2	Protein Coding	47
DNAJC6	DnaJ Heat Shock Protein Family (Hsp40) Member C6	Protein Coding	43
ATXN3	Ataxin 3	Protein Coding	45
GFAP	Glial Fibrillary Acidic Protein	Protein Coding	49
CAT	Catalase	Protein Coding	51

TARDBP	TAR DNA Binding Protein	Protein Coding	47
SLC18A2	Solute Carrier Family 18 Member A2	Protein Coding	48
AKT1	AKT Serine/Threonine Kinase 1	Protein Coding	56
MFN2	Mitofusin 2	Protein Coding	50
NR4A2	Nuclear Receptor Subfamily 4 Group A Member 2	Protein Coding	47
PSEN2	Presenilin 2	Protein Coding	51
ACE	Angiotensin I Converting Enzyme	Protein Coding	51
DNAJC13	DnaJ Heat Shock Protein Family (Hsp40) Member C13	Protein Coding	39
VPS13C	Vacuolar Protein Sorting 13 Homolog C	Protein Coding	36
TP53	Tumor Protein P53	Protein Coding	55
EIF4G1	Eukaryotic Translation Initiation Factor 4 Gamma 1	Protein Coding	47
CP	Ceruloplasmin	Protein Coding	49
INS	Insulin	Protein Coding	50
VEGFA	Vascular Endothelial Growth Factor A	Protein Coding	51
CHAT	Choline O-Acetyltransferase	Protein Coding	49
IFNG	Interferon Gamma	Protein Coding	50
NDUFS4	NADH:Ubiquinone Oxidoreductase Subunit S4	Protein Coding	43
SNCB	Synuclein Beta	Protein Coding	44
CYP2D6	Cytochrome P450 Family 2 Subfamily D Member 6	Protein Coding	50
IL1B	Interleukin 1 Beta	Protein Coding	50
NGF	Nerve Growth Factor	Protein Coding	52
SLC6A4	Solute Carrier Family 6 Member 4	Protein Coding	49
MT-ND1	Mitochondrially Encoded NADH:Ubiquinone Oxidoredu	Protein Coding	34
MT-ND5	Mitochondrially Encoded NADH:Ubiquinone Oxidoredu	Protein Coding	33
BCHE	Butyrylcholinesterase	Protein Coding	50
TTR	Transthyretin	Protein Coding	51
DYNC1H1	Dynein Cytoplasmic 1 Heavy Chain 1	Protein Coding	45
SOD2	Superoxide Dismutase 2	Protein Coding	52
CTSD	Cathepsin D	Protein Coding	54
MTOR	Mechanistic Target Of Rapamycin Kinase	Protein Coding	54
RAB39B	RAB39B, Member RAS Oncogene Family	Protein Coding	40
GAA	Glucosidase Alpha, Acid	Protein Coding	48
MAOA	Monoamine Oxidase A	Protein Coding	51
HLA-DRB	Major Histocompatibility Complex, Class II, DR Beta 1	Protein Coding	47
HMOX1	Heme Oxygenase 1	Protein Coding	54
TLR4	Toll Like Receptor 4	Protein Coding	52
ATP7B	ATPase Copper Transporting Beta	Protein Coding	48
DCTN1	Dynactin Subunit 1	Protein Coding	47
DRD3	Dopamine Receptor D3	Protein Coding	45
GRN	Granulin Precursor	Protein Coding	48
TBP	TATA-Box Binding Protein	Protein Coding	49
HTR2A	5-Hydroxytryptamine Receptor 2A	Protein Coding	49
MAOB	Monoamine Oxidase B	Protein Coding	44
CHCHD2	Coiled-Coil-Helix-Coiled-Coil-Helix Domain Containin	Protein Coding	39
DDC	Dopa Decarboxylase	Protein Coding	53
SORL1	Sortilin Related Receptor 1	Protein Coding	44
HCRT	Hypocretin Neuropeptide Precursor	Protein Coding	42

FUS	FUS RNA Binding Protein	Protein Coding	45
MIR132	MicroRNA 132	RNA Gene	22
CASP8	Caspase 8	Protein Coding	54
RET	Ret Proto-Oncogene	Protein Coding	55
TOR1A	Torsin Family 1 Member A	Protein Coding	45
IGF1	Insulin Like Growth Factor 1	Protein Coding	50
MIR21	MicroRNA 21	RNA Gene	24
PON1	Paraoxonase 1	Protein Coding	47
FGF20	Fibroblast Growth Factor 20	Protein Coding	40
DRD4	Dopamine Receptor D4	Protein Coding	47
LMNA	Lamin A/C	Protein Coding	49
RAB7A	RAB7A, Member RAS Oncogene Family	Protein Coding	49
CCL2	C-C Motif Chemokine Ligand 2	Protein Coding	50
GRIN2B	Glutamate Ionotropic Receptor NMDA Type Subunit 2	Protein Coding	51
BAX	BCL2 Associated X, Apoptosis Regulator	Protein Coding	50
COQ2	Coenzyme Q2, Polyprenyltransferase	Protein Coding	42
TLR2	Toll Like Receptor 2	Protein Coding	52
PRODH	Proline Dehydrogenase 1	Protein Coding	47
MPZ	Myelin Protein Zero	Protein Coding	45
CRP	C-Reactive Protein	Protein Coding	48
SERPINA	Serpin Family A Member 3	Protein Coding	45
ATP1A3	ATPase Na+/K+ Transporting Subunit Alpha 3	Protein Coding	48
TYR	Tyrosinase	Protein Coding	48
GLA	Galactosidase Alpha	Protein Coding	50
APOA1	Apolipoprotein A1	Protein Coding	50
UBE2L3	Ubiquitin Conjugating Enzyme E2 L3	Protein Coding	46
NOS3	Nitric Oxide Synthase 3	Protein Coding	52
NPC1	NPC Intracellular Cholesterol Transporter 1	Protein Coding	48
SYP	Synaptophysin	Protein Coding	45
PANK2	Pantothenate Kinase 2	Protein Coding	45
TAF1	TATA-Box Binding Protein Associated Factor 1	Protein Coding	46
NTRK2	Neurotrophic Receptor Tyrosine Kinase 2	Protein Coding	55
A2M	Alpha-2-Macroglobulin	Protein Coding	46
LAMP2	Lysosomal Associated Membrane Protein 2	Protein Coding	46
PODXL	Podocalyxin Like	Protein Coding	43
RPS27A	Ribosomal Protein S27a	Protein Coding	44
PDYN	Prodynorphin	Protein Coding	44
LDLR	Low Density Lipoprotein Receptor	Protein Coding	50
PTEN	Phosphatase And Tensin Homolog	Protein Coding	54
TFAM	Transcription Factor A, Mitochondrial	Protein Coding	45
PITX3	Paired Like Homeodomain 3	Protein Coding	41
SOX2	SRY-Box Transcription Factor 2	Protein Coding	48
ALB	Albumin	Protein Coding	51
HFE	Homeostatic Iron Regulator	Protein Coding	45
FTL	Ferritin Light Chain	Protein Coding	48
MPO	Myeloperoxidase	Protein Coding	51
DRD5	Dopamine Receptor D5	Protein Coding	47

CASP3	Caspase 3	Protein Coding	52
CHMP2B	Charged Multivesicular Body Protein 2B	Protein Coding	45
HLA-DQB	Major Histocompatibility Complex, Class II, DQ Beta 1	Protein Coding	45
IGF2R	Insulin Like Growth Factor 2 Receptor	Protein Coding	47
CACNA1A	Calcium Voltage-Gated Channel Subunit Alpha1 A	Protein Coding	50
FAS	Fas Cell Surface Death Receptor	Protein Coding	52
MT-ND6	Mitochondrially Encoded NADH:Ubiquinone Oxidored	Protein Coding	33
C19orf12	Chromosome 19 Open Reading Frame 12	Protein Coding	37
LOC10662	GBA Recombination Region	Biological Region	2
NOD2	Nucleotide Binding Oligomerization Domain Containing	Protein Coding	49
LEP	Leptin	Protein Coding	49
NRTN	Neurturin	Protein Coding	41
ATXN1	Ataxin 1	Protein Coding	45
FMR1	FMRP Translational Regulator 1	Protein Coding	46
ADH1C	Alcohol Dehydrogenase 1C (Class I), Gamma Polypepti	Protein Coding	43
MAPK10	Mitogen-Activated Protein Kinase 10	Protein Coding	52
NOS2	Nitric Oxide Synthase 2	Protein Coding	51
AR	Androgen Receptor	Protein Coding	54
TREM2	Triggering Receptor Expressed On Myeloid Cells 2	Protein Coding	44
STAT3	Signal Transducer And Activator Of Transcription 3	Protein Coding	54
BAP1	BRCA1 Associated Protein 1	Protein Coding	47
SLC11A2	Solute Carrier Family 11 Member 2	Protein Coding	48
TPP1	Tripeptidyl Peptidase 1	Protein Coding	44
RYR1	Ryanodine Receptor 1	Protein Coding	48
PINK1-AS	PINK1 Antisense RNA	RNA Gene	13
EGFR	Epidermal Growth Factor Receptor	Protein Coding	56
DYRK1A	Dual Specificity Tyrosine Phosphorylation Regulated K	Protein Coding	52
FGF8	Fibroblast Growth Factor 8	Protein Coding	49
ACHE	Acetylcholinesterase (Cartwright Blood Group)	Protein Coding	47
MIR34C	MicroRNA 34c	RNA Gene	21
WASHC5	WASH Complex Subunit 5	Protein Coding	33
CDC42	Cell Division Cycle 42	Protein Coding	53
ASPA	Aspartoacylase	Protein Coding	45
MIR22	MicroRNA 22	RNA Gene	20
H19	H19 Imprinted Maternally Expressed Transcript	RNA Gene	28
ATXN8OS	ATXN8 Opposite Strand LncRNA	RNA Gene	27
PARK16	Parkinson Disease 16 (Susceptibility)	Genetic Locus	5
GRM1	Glutamate Metabotropic Receptor 1	Protein Coding	52
MYH7	Myosin Heavy Chain 7	Protein Coding	48
CCR6	C-C Motif Chemokine Receptor 6	Protein Coding	46
PDGFRB	Platelet Derived Growth Factor Receptor Beta	Protein Coding	56
STUB1	STIP1 Homology And U-Box Containing Protein 1	Protein Coding	47
NTF4	Neurotrophin 4	Protein Coding	45
LMX1B	LIM Homeobox Transcription Factor 1 Beta	Protein Coding	45
PACRG	Parkin Coregulated	Protein Coding	39
SCN5A	Sodium Voltage-Gated Channel Alpha Subunit 5	Protein Coding	51
FA2H	Fatty Acid 2-Hydroxylase	Protein Coding	45

HSPB1	Heat Shock Protein Family B (Small) Member 1	Protein Coding	52
STX1B	Syntaxin 1B	Protein Coding	42
MTHFR	Methylenetetrahydrofolate Reductase	Protein Coding	48
ABCA1	ATP Binding Cassette Subfamily A Member 1	Protein Coding	49
DDIT3	DNA Damage Inducible Transcript 3	Protein Coding	47
SLC6A2	Solute Carrier Family 6 Member 2	Protein Coding	49
OGDH	Oxoglutarate Dehydrogenase	Protein Coding	46
SPG11	SPG11 Vesicle Trafficking Associated, Spatacsin	Protein Coding	40
ABCB1	ATP Binding Cassette Subfamily B Member 1	Protein Coding	52
CDKN2A	Cyclin Dependent Kinase Inhibitor 2A	Protein Coding	52
CHM	CHM Rab Escort Protein	Protein Coding	41
FOXO1	Forkhead Box O1	Protein Coding	50
HSPA9	Heat Shock Protein Family A (Hsp70) Member 9	Protein Coding	48
BMP2	Bone Morphogenetic Protein 2	Protein Coding	48
ERBB2	Erb-B2 Receptor Tyrosine Kinase 2	Protein Coding	56
PARK10	Parkinson Disease 10 (Susceptibility)	Genetic Locus	5
ESR1	Estrogen Receptor 1	Protein Coding	55
APOB	Apolipoprotein B	Protein Coding	45
CST3	Cystatin C	Protein Coding	45
SPR	Sepiapterin Reductase	Protein Coding	51
GLUD2	Glutamate Dehydrogenase 2	Protein Coding	41
CDK5	Cyclin Dependent Kinase 5	Protein Coding	54
TNNT2	Troponin T2, Cardiac Type	Protein Coding	49
MSX1	Msh Homeobox 1	Protein Coding	45
TGFB1	Transforming Growth Factor Beta 1	Protein Coding	54
MMP9	Matrix Metallopeptidase 9	Protein Coding	54
TSC2	TSC Complex Subunit 2	Protein Coding	51
PPARG	Peroxisome Proliferator Activated Receptor Gamma	Protein Coding	54
MCCC1	Methylcrotonoyl-CoA Carboxylase 1	Protein Coding	45
CXCL8	C-X-C Motif Chemokine Ligand 8	Protein Coding	43
TF	Transferrin	Protein Coding	51
TNFRSF1.	TNF Receptor Superfamily Member 1A	Protein Coding	51
PSENEN	Presenilin Enhancer, Gamma-Secretase Subunit	Protein Coding	45
CTNNB1	Catenin Beta 1	Protein Coding	54
MT-ND4	Mitochondrially Encoded NADH:Ubiquinone Oxidoredı	Protein Coding	33
RAC2	Rac Family Small GTPase 2	Protein Coding	52
MIRLET7	MicroRNA Let-7d	RNA Gene	22
CRYAB	Crystallin Alpha B	Protein Coding	47
HLA-DQA	Major Histocompatibility Complex, Class II, DQ Alpha	Protein Coding	44
NDUFV2	NADH:Ubiquinone Oxidoreductase Core Subunit V2	Protein Coding	46
HEXA	Hexosaminidase Subunit Alpha	Protein Coding	47
NFE2L2	Nuclear Factor, Erythroid 2 Like 2	Protein Coding	49
JAG1	Jagged Canonical Notch Ligand 1	Protein Coding	51
EPO	Erythropoietin	Protein Coding	43
REN	Renin	Protein Coding	50
KIF1B	Kinesin Family Member 1B	Protein Coding	45
DDOST	Dolichyl-Diphosphooligosaccharide--Protein Glycosyltr	Protein Coding	45

C1QBP	Complement C1q Binding Protein	Protein Coding	47
CYCS	Cytochrome C, Somatic	Protein Coding	50
MT-CO1	Mitochondrially Encoded Cytochrome C Oxidase I	Protein Coding	35
NAGLU	N-Acetyl-Alpha-Glucosaminidase	Protein Coding	44
HRAS	HRas Proto-Oncogene, GTPase	Protein Coding	53
DBH	Dopamine Beta-Hydroxylase	Protein Coding	51
WDR45	WD Repeat Domain 45	Protein Coding	41
APPL1	Adaptor Protein, Phosphotyrosine Interacting With PH I	Protein Coding	47
MT-ATP6	Mitochondrially Encoded ATP Synthase Membrane Sub	Protein Coding	33
RAB11A	RAB11A, Member RAS Oncogene Family	Protein Coding	48
CCL5	C-C Motif Chemokine Ligand 5	Protein Coding	45
KALRN	Kalirin RhoGEF Kinase	Protein Coding	43
FASLG	Fas Ligand	Protein Coding	49
TMEM23(	Transmembrane Protein 230	Protein Coding	37
PARK12	Parkinson Disease 12 (Susceptibility)	Genetic Locus	4
MAPK1	Mitogen-Activated Protein Kinase 1	Protein Coding	52
GJA1	Gap Junction Protein Alpha 1	Protein Coding	52
NTRK1	Neurotrophic Receptor Tyrosine Kinase 1	Protein Coding	50
TNNI3	Troponin I3, Cardiac Type	Protein Coding	50
SGCE	Sarcoglycan Epsilon	Protein Coding	43
AGL	Amylo-Alpha-1, 6-Glucosidase, 4-Alpha-Glucanotransf	Protein Coding	45
EGF	Epidermal Growth Factor	Protein Coding	52
SNCG	Synuclein Gamma	Protein Coding	44
CRYAA	Crystallin Alpha A	Protein Coding	45
MBP	Myelin Basic Protein	Protein Coding	45
PARK21	Parkinson Disease 21	Genetic Locus	2
PDGFB	Platelet Derived Growth Factor Subunit B	Protein Coding	51
GJB1	Gap Junction Protein Beta 1	Protein Coding	48
COASY	Coenzyme A Synthase	Protein Coding	46
CRPPA	CDP-L-Ribitol Pyrophosphorylase A	Protein Coding	31
TBK1	TANK Binding Kinase 1	Protein Coding	51
MIR433	MicroRNA 433	RNA Gene	21
SLC2A1	Solute Carrier Family 2 Member 1	Protein Coding	54
MIR133B	MicroRNA 133b	RNA Gene	21
THAP1	THAP Domain Containing 1	Protein Coding	40
NQO2	N-Ribosyldihydronicotinamide:Quinone Reductase 2	Protein Coding	47
G6PD	Glucose-6-Phosphate Dehydrogenase	Protein Coding	52
MYH6	Myosin Heavy Chain 6	Protein Coding	47
MYC	MYC Proto-Oncogene, BHLH Transcription Factor	Protein Coding	53
F2	Coagulation Factor II, Thrombin	Protein Coding	50
MT-CYB	Mitochondrially Encoded Cytochrome B	Protein Coding	32
JAK2	Janus Kinase 2	Protein Coding	55
PRKRA	Protein Activator Of Interferon Induced Protein Kinase	Protein Coding	43
MT-ND2	Mitochondrially Encoded NADH:Ubiquinone Oxidored	Protein Coding	33
ABCA7	ATP Binding Cassette Subfamily A Member 7	Protein Coding	44
MAPK14	Mitogen-Activated Protein Kinase 14	Protein Coding	53
PICALM	Phosphatidylinositol Binding Clathrin Assembly Protein	Protein Coding	44

TBX5	T-Box Transcription Factor 5	Protein Coding	47
PIK3CA	Phosphatidylinositol-4,5-Bisphosphate 3-Kinase Catalyt	Protein Coding	54
CD40	CD40 Molecule	Protein Coding	50
WT1	WT1 Transcription Factor	Protein Coding	50
TMEM106	Transmembrane Protein 106B	Protein Coding	39
CDH1	Cadherin 1	Protein Coding	52
APC	APC Regulator Of WNT Signaling Pathway	Protein Coding	50
SERPINC1	Serpin Family C Member 1	Protein Coding	49
BCL2	BCL2 Apoptosis Regulator	Protein Coding	53
WASHC4	WASH Complex Subunit 4	Protein Coding	31
NKX2-5	NK2 Homeobox 5	Protein Coding	46
SLC30A10	Solute Carrier Family 30 Member 10	Protein Coding	43
NCAPD2	Non-SMC Condensin I Complex Subunit D2	Protein Coding	41
FGFR1	Fibroblast Growth Factor Receptor 1	Protein Coding	56
CXCR4	C-X-C Motif Chemokine Receptor 4	Protein Coding	54
BACE1	Beta-Secretase 1	Protein Coding	48
GAPDH	Glyceraldehyde-3-Phosphate Dehydrogenase	Protein Coding	50
TWNK	Twinkle MtDNA Helicase	Protein Coding	34
DRD1	Dopamine Receptor D1	Protein Coding	45
CASP9	Caspase 9	Protein Coding	49
LPL	Lipoprotein Lipase	Protein Coding	51
MME	Membrane Metalloendopeptidase	Protein Coding	52
CD40LG	CD40 Ligand	Protein Coding	49
CFH	Complement Factor H	Protein Coding	47
FIG4	FIG4 Phosphoinositide 5-Phosphatase	Protein Coding	44
GARS1	Glycyl-TRNA Synthetase 1	Protein Coding	37
MECP2	Methyl-CpG Binding Protein 2	Protein Coding	47
NOS1	Nitric Oxide Synthase 1	Protein Coding	50
KCNQ1	Potassium Voltage-Gated Channel Subfamily Q Membe	Protein Coding	51
KIT	KIT Proto-Oncogene, Receptor Tyrosine Kinase	Protein Coding	54
ATXN7	Ataxin 7	Protein Coding	40
ESR2	Estrogen Receptor 2	Protein Coding	51
SMPD1	Sphingomyelin Phosphodiesterase 1	Protein Coding	49
SLC39A14	Solute Carrier Family 39 Member 14	Protein Coding	44
GYS1	Glycogen Synthase 1	Protein Coding	51
MYBPC3	Myosin Binding Protein C3	Protein Coding	48
RRAS	RAS Related	Protein Coding	44
SNX27	Sorting Nexin 27	Protein Coding	39
FN1	Fibronectin 1	Protein Coding	51
ADORA2A	Adenosine A2a Receptor	Protein Coding	46
NEFH	Neurofilament Heavy	Protein Coding	47
NOTCH1	Notch Receptor 1	Protein Coding	52
IL17A	Interleukin 17A	Protein Coding	44
MAPK8	Mitogen-Activated Protein Kinase 8	Protein Coding	51
JUP	Junction Plakoglobin	Protein Coding	48
MT-TL1	Mitochondrially Encoded TRNA-Leu (UUA/G) 1	RNA Gene	16
HSP90AA	Heat Shock Protein 90 Alpha Family Class A Member 1	Protein Coding	50

CXCL12	C-X-C Motif Chemokine Ligand 12	Protein Coding	47
CNR1	Cannabinoid Receptor 1	Protein Coding	48
PNKD	PNKD Metallo-Beta-Lactamase Domain Containing	Protein Coding	43
U2AF1	U2 Small Nuclear RNA Auxiliary Factor 1	Protein Coding	42
IGF2	Insulin Like Growth Factor 2	Protein Coding	49
SRGAP3	SLIT-ROBO Rho GTPase Activating Protein 3	Protein Coding	41
CHCHD10	Coiled-Coil-Helix-Coiled-Coil-Helix Domain Containin	Protein Coding	40
ATM	ATM Serine/Threonine Kinase	Protein Coding	55
KCNH2	Potassium Voltage-Gated Channel Subfamily H Membe	Protein Coding	51
DSC2	Desmocollin 2	Protein Coding	47
HNRNPA1	Heterogeneous Nuclear Ribonucleoprotein A1	Protein Coding	47
ERCC6	ERCC Excision Repair 6, Chromatin Remodeling Facto	Protein Coding	46
REST	RE1 Silencing Transcription Factor	Protein Coding	45
CHI3L1	Chitinase 3 Like 1	Protein Coding	45
NPC2	NPC Intracellular Cholesterol Transporter 2	Protein Coding	43
CACNA1C	Calcium Voltage-Gated Channel Subunit Alpha1 C	Protein Coding	50
VDR	Vitamin D Receptor	Protein Coding	53
IGHMBP2	Immunoglobulin Mu DNA Binding Protein 2	Protein Coding	42
HNRNPA2	Heterogeneous Nuclear Ribonucleoprotein A2/B1	Protein Coding	47
ADAM17	ADAM Metallopeptidase Domain 17	Protein Coding	52
VIM	Vimentin	Protein Coding	51
RAB40AL	RAB40A Like	Protein Coding	26
ERBB4	Erb-B2 Receptor Tyrosine Kinase 4	Protein Coding	56
SLC1A2	Solute Carrier Family 1 Member 2	Protein Coding	50
SNX3	Sorting Nexin 3	Protein Coding	44
SMN1	Survival Of Motor Neuron 1, Telomeric	Protein Coding	42
GSK3B	Glycogen Synthase Kinase 3 Beta	Protein Coding	51
HTR1A	5-Hydroxytryptamine Receptor 1A	Protein Coding	48
CBS	Cystathionine Beta-Synthase	Protein Coding	50
PTPN11	Protein Tyrosine Phosphatase Non-Receptor Type 11	Protein Coding	55
DCAF17	DDB1 And CUL4 Associated Factor 17	Protein Coding	37
GAD1	Glutamate Decarboxylase 1	Protein Coding	52
CCL3	C-C Motif Chemokine Ligand 3	Protein Coding	41
NAT2	N-Acetyltransferase 2	Protein Coding	44
NF1	Neurofibromin 1	Protein Coding	50
GSR	Glutathione-Disulfide Reductase	Protein Coding	50
MIR17	MicroRNA 17	RNA Gene	21
KLK6	Kallikrein Related Peptidase 6	Protein Coding	44
TGM2	Transglutaminase 2	Protein Coding	50
DSC3	Desmocollin 3	Protein Coding	43
PARP1	Poly(ADP-Ribose) Polymerase 1	Protein Coding	51
NCSTN	Nicastrin	Protein Coding	48
GPR37	G Protein-Coupled Receptor 37	Protein Coding	45
DMD	Dystrophin	Protein Coding	48
NEK1	NIMA Related Kinase 1	Protein Coding	44
NPPB	Natriuretic Peptide B	Protein Coding	45
ACTC1	Actin Alpha Cardiac Muscle 1	Protein Coding	43

IL1A	Interleukin 1 Alpha	Protein Coding	45
CLN3	CLN3 Lysosomal/Endosomal Transmembrane Protein,	Protein Coding	44
ATN1	Atrophin 1	Protein Coding	45
MT-ND3	Mitochondrially Encoded NADH:Ubiquinone Oxidored	Protein Coding	32
H2AC18	H2A Clustered Histone 18	Protein Coding	27
PTGS2	Prostaglandin-Endoperoxide Synthase 2	Protein Coding	50
RELN	Reelin	Protein Coding	44
HLA-DRA	Major Histocompatibility Complex, Class II, DR Alpha	Protein Coding	48
PYGM	Glycogen Phosphorylase, Muscle Associated	Protein Coding	47
OPTN	Optineurin	Protein Coding	45
CAV1	Caveolin 1	Protein Coding	50
PVALB	Parvalbumin	Protein Coding	41
ARSA	Arylsulfatase A	Protein Coding	48
HP	Haptoglobin	Protein Coding	45
CCND1	Cyclin D1	Protein Coding	53
CSF1R	Colony Stimulating Factor 1 Receptor	Protein Coding	53
NQO1	NAD(P)H Quinone Dehydrogenase 1	Protein Coding	51
JPH3	Junctophilin 3	Protein Coding	42
SYT11	Synaptotagmin 11	Protein Coding	43
DNM1L	Dynamin 1 Like	Protein Coding	48
CLU	Clusterin	Protein Coding	48
MDM2	MDM2 Proto-Oncogene	Protein Coding	54
NRG1	Neuregulin 1	Protein Coding	49
NTF3	Neurotrophin 3	Protein Coding	45
FXN	Frataxin	Protein Coding	46
MIRLET7	MicroRNA Let-7i	RNA Gene	20
SHH	Sonic Hedgehog Signaling Molecule	Protein Coding	50
SLC11A1	Solute Carrier Family 11 Member 1	Protein Coding	48
MT-CO2	Mitochondrially Encoded Cytochrome C Oxidase II	Protein Coding	35
SST	Somatostatin	Protein Coding	43
BSCL2	BSCL2 Lipid Droplet Biogenesis Associated, Seipin	Protein Coding	43
HGF	Hepatocyte Growth Factor	Protein Coding	53
BMP4	Bone Morphogenetic Protein 4	Protein Coding	50
PROM1	Prominin 1	Protein Coding	46
EPM2A	EPM2A Glucan Phosphatase, Laforin	Protein Coding	43
MT-TK	Mitochondrially Encoded TRNA-Lys (AAA/G)	RNA Gene	15
PPARGC1	PPARG Coactivator 1 Alpha	Protein Coding	48
SETX	Senataxin	Protein Coding	41
IGF1R	Insulin Like Growth Factor 1 Receptor	Protein Coding	56
CNTF	Ciliary Neurotrophic Factor	Protein Coding	43
HSPA8	Heat Shock Protein Family A (Hsp70) Member 8	Protein Coding	48
NDUFS3	NADH:Ubiquinone Oxidoreductase Core Subunit S3	Protein Coding	48
TLR5	Toll Like Receptor 5	Protein Coding	49
GRIN2A	Glutamate Ionotropic Receptor NMDA Type Subunit 2	Protein Coding	52
HLA-B	Major Histocompatibility Complex, Class I, B	Protein Coding	46
IL1RN	Interleukin 1 Receptor Antagonist	Protein Coding	50
MMP13	Matrix Metallopeptidase 13	Protein Coding	51

BRCA2	BRCA2 DNA Repair Associated	Protein Coding	50
MAPK3	Mitogen-Activated Protein Kinase 3	Protein Coding	51
IRS1	Insulin Receptor Substrate 1	Protein Coding	49
MIR146A	MicroRNA 146a	RNA Gene	24
TOMM40	Translocase Of Outer Mitochondrial Membrane 40	Protein Coding	41
PON2	Paraoxonase 2	Protein Coding	44
CAV3	Caveolin 3	Protein Coding	44
ATP7A	ATPase Copper Transporting Alpha	Protein Coding	47
LOX	Lysyl Oxidase	Protein Coding	46
DES	Desmin	Protein Coding	49
MSH2	MutS Homolog 2	Protein Coding	50
TPH1	Tryptophan Hydroxylase 1	Protein Coding	45
MAP2	Microtubule Associated Protein 2	Protein Coding	44
XDH	Xanthine Dehydrogenase	Protein Coding	47
MTR	5-Methyltetrahydrofolate-Homocysteine Methyltransfer:	Protein Coding	47
TERT	Telomerase Reverse Transcriptase	Protein Coding	53
SAG	S-Antigen Visual Arrestin	Protein Coding	46
CYBB	Cytochrome B-245 Beta Chain	Protein Coding	48
BRCA1	BRCA1 DNA Repair Associated	Protein Coding	52
DKK1	Dickkopf WNT Signaling Pathway Inhibitor 1	Protein Coding	47
S100B	S100 Calcium Binding Protein B	Protein Coding	47
SRC	SRC Proto-Oncogene, Non-Receptor Tyrosine Kinase	Protein Coding	53
SLC17A5	Solute Carrier Family 17 Member 5	Protein Coding	45
ABL1	ABL Proto-Oncogene 1, Non-Receptor Tyrosine Kinase	Protein Coding	54
TSC1	TSC Complex Subunit 1	Protein Coding	50
RBFOX3	RNA Binding Fox-1 Homolog 3	Protein Coding	36
IL2RB	Interleukin 2 Receptor Subunit Beta	Protein Coding	50
SLC26A4	Solute Carrier Family 26 Member 4	Protein Coding	44
TRPM7	Transient Receptor Potential Cation Channel Subfamily	Protein Coding	47
IREB2	Iron Responsive Element Binding Protein 2	Protein Coding	44
TMEM67	Transmembrane Protein 67	Protein Coding	38
MIR34A	MicroRNA 34a	RNA Gene	22
ALS2	Alsin Rho Guanine Nucleotide Exchange Factor ALS2	Protein Coding	45
SDHB	Succinate Dehydrogenase Complex Iron Sulfur Subunit	Protein Coding	49
STH	Saitohin	Protein Coding	31
CREBBP	CREB Binding Protein	Protein Coding	54
GDAP1	Ganglioside Induced Differentiation Associated Protein	Protein Coding	43
ATP2B3	ATPase Plasma Membrane Ca2+ Transporting 3	Protein Coding	47
SCARB2	Scavenger Receptor Class B Member 2	Protein Coding	45
RAB29	RAB29, Member RAS Oncogene Family	Protein Coding	34
GAK	Cyclin G Associated Kinase	Protein Coding	45
CYP1B1	Cytochrome P450 Family 1 Subfamily B Member 1	Protein Coding	50
SERPINE1	Serpin Family E Member 1	Protein Coding	52
GNAL	G Protein Subunit Alpha L	Protein Coding	47
NDUFV1	NADH:Ubiquinone Oxidoreductase Core Subunit V1	Protein Coding	47
CDKN1A	Cyclin Dependent Kinase Inhibitor 1A	Protein Coding	51
ECE1	Endothelin Converting Enzyme 1	Protein Coding	48

MAG	Myelin Associated Glycoprotein	Protein Coding	47
UBQLN2	Ubiquilin 2	Protein Coding	44
HSPA4	Heat Shock Protein Family A (Hsp70) Member 4	Protein Coding	43
NTS	Neurotensin	Protein Coding	41
MIR125A	MicroRNA 125a	RNA Gene	21
DNAJC5	DnaJ Heat Shock Protein Family (Hsp40) Member C5	Protein Coding	45
CSF3	Colony Stimulating Factor 3	Protein Coding	41
MAP2K1	Mitogen-Activated Protein Kinase Kinase 1	Protein Coding	55
EP300	E1A Binding Protein P300	Protein Coding	52
GLUD1	Glutamate Dehydrogenase 1	Protein Coding	51
DMPK	DM1 Protein Kinase	Protein Coding	49
CALB1	Calbindin 1	Protein Coding	43
ICAM1	Intercellular Adhesion Molecule 1	Protein Coding	52
KIF5A	Kinesin Family Member 5A	Protein Coding	45
PAX6	Paired Box 6	Protein Coding	49
TNNT1	Troponin T1, Slow Skeletal Type	Protein Coding	44
HAMP	Hepcidin Antimicrobial Peptide	Protein Coding	44
NHLRC1	NHL Repeat Containing E3 Ubiquitin Protein Ligase 1	Protein Coding	41
HTR3A	5-Hydroxytryptamine Receptor 3A	Protein Coding	47
CYC1	Cytochrome C1	Protein Coding	46
SLC25A4	Solute Carrier Family 25 Member 4	Protein Coding	48
HPCA	Hippocalcin	Protein Coding	42
PLAU	Plasminogen Activator, Urokinase	Protein Coding	53
CRH	Corticotropin Releasing Hormone	Protein Coding	45
GLI3	GLI Family Zinc Finger 3	Protein Coding	50
TAC1	Tachykinin Precursor 1	Protein Coding	44
TSPO	Translocator Protein	Protein Coding	44
VPS13A	Vacuolar Protein Sorting 13 Homolog A	Protein Coding	41
SCN9A	Sodium Voltage-Gated Channel Alpha Subunit 9	Protein Coding	48
GSN	Gelsolin	Protein Coding	50
GM2A	GM2 Ganglioside Activator	Protein Coding	44
DNMT1	DNA Methyltransferase 1	Protein Coding	52
PDGFRA	Platelet Derived Growth Factor Receptor Alpha	Protein Coding	56
LINGO1	Leucine Rich Repeat And Ig Domain Containing 1	Protein Coding	44
VPS26A	VPS26, Retromer Complex Component A	Protein Coding	41
EPHA3	EPH Receptor A3	Protein Coding	46
RB1	RB Transcriptional Corepressor 1	Protein Coding	51
MMP1	Matrix Metallopeptidase 1	Protein Coding	53
RNF19A	Ring Finger Protein 19A, RBR E3 Ubiquitin Protein Li	Protein Coding	40
ITPR1	Inositol 1,4,5-Trisphosphate Receptor Type 1	Protein Coding	50
NRAS	NRAS Proto-Oncogene, GTPase	Protein Coding	51
GPT	Glutamic--Pyruvic Transaminase	Protein Coding	42
IL4	Interleukin 4	Protein Coding	48
PABPN1	Poly(A) Binding Protein Nuclear 1	Protein Coding	45
SCN1A	Sodium Voltage-Gated Channel Alpha Subunit 1	Protein Coding	48
C4A	Complement C4A (Rodgers Blood Group)	Protein Coding	44
CTSB	Cathepsin B	Protein Coding	52

VHL	Von Hippel-Lindau Tumor Suppressor	Protein Coding	47
KCNJ2	Potassium Inwardly Rectifying Channel Subfamily J M	Protein Coding	49
SCN8A	Sodium Voltage-Gated Channel Alpha Subunit 8	Protein Coding	47
G6PC	Glucose-6-Phosphatase Catalytic Subunit	Protein Coding	44
GJA5	Gap Junction Protein Alpha 5	Protein Coding	47
SCN2A	Sodium Voltage-Gated Channel Alpha Subunit 2	Protein Coding	48
AIMP2	Aminoacyl TRNA Synthetase Complex Interacting Mul	Protein Coding	43
TBX20	T-Box Transcription Factor 20	Protein Coding	43
MIR9-1	MicroRNA 9-1	RNA Gene	21
PFKM	Phosphofructokinase, Muscle	Protein Coding	51
HSPG2	Heparan Sulfate Proteoglycan 2	Protein Coding	48
SMARCB	SWI/SNF Related, Matrix Associated, Actin Dependent	Protein Coding	47
SPG7	SPG7 Matrix AAA Peptidase Subunit, Paraplegin	Protein Coding	44
GSTP1	Glutathione S-Transferase Pi 1	Protein Coding	51
NDUFA1	NADH:Ubiquinone Oxidoreductase Subunit A1	Protein Coding	45
CAPN1	Calpain 1	Protein Coding	51
PSAP	Prosaposin	Protein Coding	47
SLC1A3	Solute Carrier Family 1 Member 3	Protein Coding	51
CFTR	CF Transmembrane Conductance Regulator	Protein Coding	52
PRDM10	PR/SET Domain 10	Protein Coding	35
ADNP	Activity Dependent Neuroprotector Homeobox	Protein Coding	43
HLA-DRB	Major Histocompatibility Complex, Class II, DR Beta	Protein Coding	40
SLC40A1	Solute Carrier Family 40 Member 1	Protein Coding	47
COX5A	Cytochrome C Oxidase Subunit 5A	Protein Coding	44
GDF6	Growth Differentiation Factor 6	Protein Coding	43
NDUFS2	NADH:Ubiquinone Oxidoreductase Core Subunit S2	Protein Coding	45
CDK4	Cyclin Dependent Kinase 4	Protein Coding	56
IL2	Interleukin 2	Protein Coding	47
CCR5	C-C Motif Chemokine Receptor 5 (Gene/Pseudogene)	Protein Coding	48
STXBP1	Syntaxin Binding Protein 1	Protein Coding	50
INSR	Insulin Receptor	Protein Coding	55
TUBA4A	Tubulin Alpha 4a	Protein Coding	47
ABCC8	ATP Binding Cassette Subfamily C Member 8	Protein Coding	47
FGF2	Fibroblast Growth Factor 2	Protein Coding	49
PARL	Presenilin Associated Rhomboid Like	Protein Coding	42
SPAST	Spastin	Protein Coding	41
TIA1	TIA1 Cytotoxic Granule Associated RNA Binding Prot	Protein Coding	44
GAL	Galanin And GMAP Prepropeptide	Protein Coding	45
PITX2	Paired Like Homeodomain 2	Protein Coding	47
SMN2	Survival Of Motor Neuron 2, Centromeric	Protein Coding	43
MIR24-1	MicroRNA 24-1	RNA Gene	20
LAMP1	Lysosomal Associated Membrane Protein 1	Protein Coding	44
GRM5	Glutamate Metabotropic Receptor 5	Protein Coding	48
NPY	Neuropeptide Y	Protein Coding	47
GNE	Glucosamine (UDP-N-Acetyl)-2-Epimerase/N-Acetylm	Protein Coding	45
NDUFAF2	NADH:Ubiquinone Oxidoreductase Complex Assembly	Protein Coding	41
SLC20A2	Solute Carrier Family 20 Member 2	Protein Coding	47

HTR2C	5-Hydroxytryptamine Receptor 2C	Protein Coding	48
CHKB	Choline Kinase Beta	Protein Coding	47
MT-CO3	Mitochondrially Encoded Cytochrome C Oxidase III	Protein Coding	32
MMP2	Matrix Metallopeptidase 2	Protein Coding	55
VAPB	VAMP Associated Protein B And C	Protein Coding	47
UBB	Ubiquitin B	Protein Coding	44
GFRA1	GDNF Family Receptor Alpha 1	Protein Coding	45
CPT2	Carnitine Palmitoyltransferase 2	Protein Coding	50
RAB5A	RAB5A, Member RAS Oncogene Family	Protein Coding	47
MSH6	MutS Homolog 6	Protein Coding	51
CETP	Cholesteryl Ester Transfer Protein	Protein Coding	48
CDKN2B	Cyclin Dependent Kinase Inhibitor 2B	Protein Coding	48
PNPLA2	Patatin Like Phospholipase Domain Containing 2	Protein Coding	45
CHD7	Chromodomain Helicase DNA Binding Protein 7	Protein Coding	46
HNF1A	HNF1 Homeobox A	Protein Coding	47
MSN	Moesin	Protein Coding	48
MMP3	Matrix Metallopeptidase 3	Protein Coding	52
ALOX5	Arachidonate 5-Lipoxygenase	Protein Coding	50
NTRK3	Neurotrophic Receptor Tyrosine Kinase 3	Protein Coding	52
RIT2	Ras Like Without CAAX 2	Protein Coding	40
PRDX1	Peroxiredoxin 1	Protein Coding	51
SDHA	Succinate Dehydrogenase Complex Flavoprotein Subun	Protein Coding	48
HSD17B1(	Hydroxysteroid 17-Beta Dehydrogenase 10	Protein Coding	47
SMARCA4	SWI/SNF Related, Matrix Associated, Actin Dependent	Protein Coding	52
CASQ2	Calsequestrin 2	Protein Coding	45
MEF2C	Myocyte Enhancer Factor 2C	Protein Coding	50
NDUFS1	NADH:Ubiquinone Oxidoreductase Core Subunit S1	Protein Coding	47
MIR144	MicroRNA 144	RNA Gene	17
LRP1	LDL Receptor Related Protein 1	Protein Coding	48
GRIA3	Glutamate Ionotropic Receptor AMPA Type Subunit 3	Protein Coding	51
GRIA1	Glutamate Ionotropic Receptor AMPA Type Subunit 1	Protein Coding	50
KANSL1	KAT8 Regulatory NSL Complex Subunit 1	Protein Coding	39
HOTAIR	HOX Transcript Antisense RNA	RNA Gene	25
PTCH1	Patched 1	Protein Coding	51
NDUFS7	NADH:Ubiquinone Oxidoreductase Core Subunit S7	Protein Coding	47
AVP	Arginine Vasopressin	Protein Coding	47
MIR20A	MicroRNA 20a	RNA Gene	19
GPNMB	Glycoprotein Nmb	Protein Coding	45
STK11	Serine/Threonine Kinase 11	Protein Coding	50
CHGA	Chromogranin A	Protein Coding	44
DNMT3B	DNA Methyltransferase 3 Beta	Protein Coding	51
CR1	Complement C3b/C4b Receptor 1 (Knops Blood Group	Protein Coding	45
SERPINA1	Serpin Family A Member 1	Protein Coding	51
LRP5	LDL Receptor Related Protein 5	Protein Coding	50
ACO1	Aconitase 1	Protein Coding	45
TFRC	Transferrin Receptor	Protein Coding	50
CHRNA4	Cholinergic Receptor Nicotinic Alpha 4 Subunit	Protein Coding	50

TIMP1	TIMP Metallopeptidase Inhibitor 1	Protein Coding	47
POMC	Proopiomelanocortin	Protein Coding	50
GAP43	Growth Associated Protein 43	Protein Coding	43
GSTO1	Glutathione S-Transferase Omega 1	Protein Coding	44
PARK3	Parkinson Disease 3 (Autosomal Dominant, Lewy Body	Genetic Locus	4
TTN	Titin	Protein Coding	48
NKX2-1	NK2 Homeobox 1	Protein Coding	47
BICD2	BICD Cargo Adaptor 2	Protein Coding	41
NDUFA13	NADH:Ubiquinone Oxidoreductase Subunit A13	Protein Coding	45
KCNE1	Potassium Voltage-Gated Channel Subfamily E Regulat	Protein Coding	45
AIF1	Allograft Inflammatory Factor 1	Protein Coding	41
LRRK1	Leucine Rich Repeat Kinase 1	Protein Coding	39
ERBB3	Erb-B2 Receptor Tyrosine Kinase 3	Protein Coding	55
CX3CR1	C-X3-C Motif Chemokine Receptor 1	Protein Coding	46
OGG1	8-Oxoguanine DNA Glycosylase	Protein Coding	48
TFEB	Transcription Factor EB	Protein Coding	43
SNAP25	Synaptosome Associated Protein 25	Protein Coding	51
MATR3	Matrin 3	Protein Coding	42
SPTLC1	Serine Palmitoyltransferase Long Chain Base Subunit 1	Protein Coding	48
KRAS	KRAS Proto-Oncogene, GTPase	Protein Coding	52
MIR212	MicroRNA 212	RNA Gene	20
MIR223	MicroRNA 223	RNA Gene	22
SIAH1	Siah E3 Ubiquitin Protein Ligase 1	Protein Coding	47
BRAF	B-Raf Proto-Oncogene, Serine/Threonine Kinase	Protein Coding	55
CD4	CD4 Molecule	Protein Coding	51
NDUFB3	NADH:Ubiquinone Oxidoreductase Subunit B3	Protein Coding	43
HPRT1	Hypoxanthine Phosphoribosyltransferase 1	Protein Coding	49
CLN6	CLN6 Transmembrane ER Protein	Protein Coding	38
SOX9	SRY-Box Transcription Factor 9	Protein Coding	48
AGTR1	Angiotensin II Receptor Type 1	Protein Coding	51
CNBP	CCHC-Type Zinc Finger Nucleic Acid Binding Protein	Protein Coding	43
GRIN1	Glutamate Ionotropic Receptor NMDA Type Subunit 1	Protein Coding	51
HIF1A	Hypoxia Inducible Factor 1 Subunit Alpha	Protein Coding	49
IDH1	Isocitrate Dehydrogenase (NADP(+)) 1	Protein Coding	54
SCN4A	Sodium Voltage-Gated Channel Alpha Subunit 4	Protein Coding	45
NDUFAF1	NADH:Ubiquinone Oxidoreductase Complex Assembly	Protein Coding	42
STK39	Serine/Threonine Kinase 39	Protein Coding	45
DCAF8	DDB1 And CUL4 Associated Factor 8	Protein Coding	39
MIR29B1	MicroRNA 29b-1	RNA Gene	22
CUL1	Cullin 1	Protein Coding	47
PLP1	Proteolipid Protein 1	Protein Coding	44
NDUFA6	NADH:Ubiquinone Oxidoreductase Subunit A6	Protein Coding	45
NDUFB11	NADH:Ubiquinone Oxidoreductase Subunit B11	Protein Coding	40
NDUFB9	NADH:Ubiquinone Oxidoreductase Subunit B9	Protein Coding	45
SDHD	Succinate Dehydrogenase Complex Subunit D	Protein Coding	45
CALCA	Calcitonin Related Polypeptide Alpha	Protein Coding	44
TYMP	Thymidine Phosphorylase	Protein Coding	48

DAPK1	Death Associated Protein Kinase 1	Protein Coding	50
HLA-A	Major Histocompatibility Complex, Class I, A	Protein Coding	48
NFKB1	Nuclear Factor Kappa B Subunit 1	Protein Coding	54
NDUFS8	NADH:Ubiquinone Oxidoreductase Core Subunit S8	Protein Coding	46
IL18	Interleukin 18	Protein Coding	45
ACTB	Actin Beta	Protein Coding	50
LRP2	LDL Receptor Related Protein 2	Protein Coding	47
B2M	Beta-2-Microglobulin	Protein Coding	50
PPP3CA	Protein Phosphatase 3 Catalytic Subunit Alpha	Protein Coding	54
IDUA	Alpha-L-Iduronidase	Protein Coding	43
CHRNA7	Cholinergic Receptor Nicotinic Alpha 7 Subunit	Protein Coding	47
PHOX2B	Paired Like Homeobox 2B	Protein Coding	44
NLRP3	NLR Family Pyrin Domain Containing 3	Protein Coding	48
MET	MET Proto-Oncogene, Receptor Tyrosine Kinase	Protein Coding	56
CASP1	Caspase 1	Protein Coding	51
FOXG1	Forkhead Box G1	Protein Coding	44
TK2	Thymidine Kinase 2	Protein Coding	43
ATCAY	ATCAY Kinesin Light Chain Interacting Caytaxin	Protein Coding	39
PRRT2	Proline Rich Transmembrane Protein 2	Protein Coding	40
ADAM10	ADAM Metallopeptidase Domain 10	Protein Coding	54
GSTM1	Glutathione S-Transferase Mu 1	Protein Coding	43
KMT2D	Lysine Methyltransferase 2D	Protein Coding	42
FTH1	Ferritin Heavy Chain 1	Protein Coding	51
ZFYVE26	Zinc Finger FYVE-Type Containing 26	Protein Coding	40
MYO7A	Myosin VIIA	Protein Coding	43
MTFMT	Mitochondrial Methionyl-TRNA Formyltransferase	Protein Coding	43
SLC41A1	Solute Carrier Family 41 Member 1	Protein Coding	39
SPP1	Secreted Phosphoprotein 1	Protein Coding	48
DLG4	Discs Large MAGUK Scaffold Protein 4	Protein Coding	47
RAD51	RAD51 Recombinase	Protein Coding	52
IL4R	Interleukin 4 Receptor	Protein Coding	49
JUN	Jun Proto-Oncogene, AP-1 Transcription Factor Subuni	Protein Coding	51
NDUFA9	NADH:Ubiquinone Oxidoreductase Subunit A9	Protein Coding	45
NNMT	Nicotinamide N-Methyltransferase	Protein Coding	46
DNAAF3	Dynein Axonemal Assembly Factor 3	Protein Coding	36
SORT1	Sortilin 1	Protein Coding	44
CCDC62	Coiled-Coil Domain Containing 62	Protein Coding	33
ADIPOQ	Adiponectin, C1Q And Collagen Domain Containing	Protein Coding	47
SUMO1	Small Ubiquitin Like Modifier 1	Protein Coding	48
ATP6AP2	ATPase H+ Transporting Accessory Protein 2	Protein Coding	45
KLF4	Kruppel Like Factor 4	Protein Coding	47
KCNE2	Potassium Voltage-Gated Channel Subfamily E Regulat	Protein Coding	41
MBL2	Mannose Binding Lectin 2	Protein Coding	48
TNFRSF1]	TNF Receptor Superfamily Member 1B	Protein Coding	49
COL4A1	Collagen Type IV Alpha 1 Chain	Protein Coding	49
ALDH1A1	Aldehyde Dehydrogenase 1 Family Member A1	Protein Coding	48
COL17A1	Collagen Type XVII Alpha 1 Chain	Protein Coding	45

NDUFB8	NADH:Ubiquinone Oxidoreductase Subunit B8	Protein Coding	43
CDKN3	Cyclin Dependent Kinase Inhibitor 3	Protein Coding	41
PRPH2	Peripherin 2	Protein Coding	40
SPG21	SPG21 Abhydrolase Domain Containing, Maspardin	Protein Coding	41
ANG	Angiogenin	Protein Coding	47
CNTNAP2	Contactin Associated Protein 2	Protein Coding	45
ATXN10	Ataxin 10	Protein Coding	43
SIGMAR1	Sigma Non-Opioid Intracellular Receptor 1	Protein Coding	47
UBE2E3	Ubiquitin Conjugating Enzyme E2 E3	Protein Coding	43
AURKA	Aurora Kinase A	Protein Coding	51
GGT1	Gamma-Glutamyltransferase 1	Protein Coding	48
CDKN1B	Cyclin Dependent Kinase Inhibitor 1B	Protein Coding	50
PAH	Phenylalanine Hydroxylase	Protein Coding	50
XK	X-Linked Kx Blood Group	Protein Coding	41
GSTO2	Glutathione S-Transferase Omega 2	Protein Coding	44
MALAT1	Metastasis Associated Lung Adenocarcinoma Transcrip	RNA Gene	25
XPR1	Xenotropic And Polytropic Retrovirus Receptor 1	Protein Coding	44
USH2A	Usherin	Protein Coding	39
ABCG2	ATP Binding Cassette Subfamily G Member 2 (Junior I	Protein Coding	52
DNAJB6	DnaJ Heat Shock Protein Family (Hsp40) Member B6	Protein Coding	43
WNT3	Wnt Family Member 3	Protein Coding	48
ADCYAP1	Adenylate Cyclase Activating Polypeptide 1	Protein Coding	42
HIP1R	Huntingtin Interacting Protein 1 Related	Protein Coding	41
TPI1	Triosephosphate Isomerase 1	Protein Coding	48
NDP	Norrin Cystine Knot Growth Factor NDP	Protein Coding	44
NDUFA10	NADH:Ubiquinone Oxidoreductase Subunit A10	Protein Coding	45
ARSG	Arylsulfatase G	Protein Coding	44
EIF4EBP1	Eukaryotic Translation Initiation Factor 4E Binding Prc	Protein Coding	48
ELANE	Elastase, Neutrophil Expressed	Protein Coding	48
HMGCR	3-Hydroxy-3-Methylglutaryl-CoA Reductase	Protein Coding	47
NEAT1	Nuclear Paraspeckle Assembly Transcript 1	RNA Gene	24
FLG	Filaggrin	Protein Coding	41
NDUFS6	NADH:Ubiquinone Oxidoreductase Subunit S6	Protein Coding	44
NBN	Nibrin	Protein Coding	49
AFG3L2	AFG3 Like Matrix AAA Peptidase Subunit 2	Protein Coding	45
PFN1	Profilin 1	Protein Coding	49
SLC25A13	Solute Carrier Family 25 Member 13	Protein Coding	47
NOTCH2	Notch Receptor 2	Protein Coding	51
UBE2D2	Ubiquitin Conjugating Enzyme E2 D2	Protein Coding	45
DGKQ	Diacylglycerol Kinase Theta	Protein Coding	42
RRM2B	Ribonucleotide Reductase Regulatory TP53 Inducible S	Protein Coding	50
MIR29A	MicroRNA 29a	RNA Gene	22
HSPD1	Heat Shock Protein Family D (Hsp60) Member 1	Protein Coding	49
PLD3	Phospholipase D Family Member 3	Protein Coding	42
FBN1	Fibrillin 1	Protein Coding	47
TBX3	T-Box Transcription Factor 3	Protein Coding	46
EPRS1	Glutamyl-Prolyl-TRNA Synthetase 1	Protein Coding	37

RD3	Retinal Degeneration 3, GUCY2D Regulator	Protein Coding	39
FKRP	Fukutin Related Protein	Protein Coding	41
EZH2	Enhancer Of Zeste 2 Polycomb Repressive Complex 2 S	Protein Coding	54
ALK	ALK Receptor Tyrosine Kinase	Protein Coding	52
IAPP	Islet Amyloid Polypeptide	Protein Coding	41
CACNA1C	Calcium Voltage-Gated Channel Subunit Alpha1 G	Protein Coding	49
CD36	CD36 Molecule	Protein Coding	50
TFR2	Transferrin Receptor 2	Protein Coding	45
MIR18A	MicroRNA 18a	RNA Gene	18
ABCB7	ATP Binding Cassette Subfamily B Member 7	Protein Coding	44
EIF4E	Eukaryotic Translation Initiation Factor 4E	Protein Coding	51
SLC5A7	Solute Carrier Family 5 Member 7	Protein Coding	44
KCNQ2	Potassium Voltage-Gated Channel Subfamily Q Membe	Protein Coding	48
TMEM126	Transmembrane Protein 126B	Protein Coding	39
NDUFA2	NADH:Ubiquinone Oxidoreductase Subunit A2	Protein Coding	43
POLG2	DNA Polymerase Gamma 2, Accessory Subunit	Protein Coding	45
FGF10	Fibroblast Growth Factor 10	Protein Coding	48
GLE1	GLE1 RNA Export Mediator	Protein Coding	41
ATP2A2	ATPase Sarcoplasmic/Endoplasmic Reticulum Ca2+ Tr	Protein Coding	52
OPRD1	Opioid Receptor Delta 1	Protein Coding	44
GLI1	GLI Family Zinc Finger 1	Protein Coding	48
NDUFA12	NADH:Ubiquinone Oxidoreductase Subunit A12	Protein Coding	45
SURF1	SURF1 Cytochrome C Oxidase Assembly Factor	Protein Coding	44
PLCB1	Phospholipase C Beta 1	Protein Coding	50
CAPN3	Calpain 3	Protein Coding	48
OTX2	Orthodenticle Homeobox 2	Protein Coding	46
SLC18A3	Solute Carrier Family 18 Member A3	Protein Coding	45
RAB10	RAB10, Member RAS Oncogene Family	Protein Coding	44
AGER	Advanced Glycosylation End-Product Specific Receptor	Protein Coding	45
BCS1L	BCS1 Homolog, Ubiquinol-Cytochrome C Reductase C	Protein Coding	44
PLA2G2A	Phospholipase A2 Group IIA	Protein Coding	47
SACS	Sacsin Molecular Chaperone	Protein Coding	37
UBE2N	Ubiquitin Conjugating Enzyme E2 N	Protein Coding	48
RYR2	Ryanodine Receptor 2	Protein Coding	48
THBD	Thrombomodulin	Protein Coding	45
UBE3A	Ubiquitin Protein Ligase E3A	Protein Coding	49
HRH3	Histamine Receptor H3	Protein Coding	43
ACMSD	Aminocarboxymuconate Semialdehyde Decarboxylase	Protein Coding	40
MECOM	MDS1 And EVI1 Complex Locus	Protein Coding	48
CDK6	Cyclin Dependent Kinase 6	Protein Coding	55
CHIT1	Chitinase 1	Protein Coding	45
TPPP3	Tubulin Polymerization Promoting Protein Family Mem	Protein Coding	38
ADRB2	Adrenoceptor Beta 2	Protein Coding	50
HK1	Hexokinase 1	Protein Coding	52
STAT1	Signal Transducer And Activator Of Transcription 1	Protein Coding	55
TSEN54	TRNA Splicing Endonuclease Subunit 54	Protein Coding	40
PDE10A	Phosphodiesterase 10A	Protein Coding	46

IL12B	Interleukin 12B	Protein Coding	45
DNM2	Dynamin 2	Protein Coding	51
MASP2	Mannan Binding Lectin Serine Peptidase 2	Protein Coding	45
CSMD1	CUB And Sushi Multiple Domains 1	Protein Coding	37
KARS1	Lysyl-TRNA Synthetase 1	Protein Coding	39
NEU1	Neuraminidase 1	Protein Coding	45
SIRT1	Sirtuin 1	Protein Coding	51
ZNF746	Zinc Finger Protein 746	Protein Coding	37
ENO2	Enolase 2	Protein Coding	49
ODC1	Ornithine Decarboxylase 1	Protein Coding	48
ISG15	ISG15 Ubiquitin Like Modifier	Protein Coding	48
TUBA1B	Tubulin Alpha 1b	Protein Coding	44
CAST	Calpastatin	Protein Coding	47
STBD1	Starch Binding Domain 1	Protein Coding	37
KIF1A	Kinesin Family Member 1A	Protein Coding	44
DHFR	Dihydrofolate Reductase	Protein Coding	51
OMP	Olfactory Marker Protein	Protein Coding	39
YWHAQ	Tyrosine 3-Monooxygenase/Tryptophan 5-Monooxygen	Protein Coding	48
WNT1	Wnt Family Member 1	Protein Coding	48
DDX41	DEAD-Box Helicase 41	Protein Coding	44
RAB7B	RAB7B, Member RAS Oncogene Family	Protein Coding	31
RNF11	Ring Finger Protein 11	Protein Coding	37
MYORG	Myogenesis Regulating Glycosidase (Putative)	Protein Coding	28
FIS1	Fission, Mitochondrial 1	Protein Coding	40
PPP2R2B	Protein Phosphatase 2 Regulatory Subunit Bbeta	Protein Coding	47
CCK	Cholecystokinin	Protein Coding	42
PNKP	Polynucleotide Kinase 3'-Phosphatase	Protein Coding	47
DGUOK	Deoxyguanosine Kinase	Protein Coding	45
APOL1	Apolipoprotein L1	Protein Coding	43
AGT	Angiotensinogen	Protein Coding	51
CYBA	Cytochrome B-245 Alpha Chain	Protein Coding	47
TGFBR2	Transforming Growth Factor Beta Receptor 2	Protein Coding	54
MIR15A	MicroRNA 15a	RNA Gene	16
DNM1	Dynamin 1	Protein Coding	52
NUDT1	Nudix Hydrolase 1	Protein Coding	43
TRAP1	TNF Receptor Associated Protein 1	Protein Coding	43
DNMT3A	DNA Methyltransferase 3 Alpha	Protein Coding	52
NDUFAF5	NADH:Ubiquinone Oxidoreductase Complex Assembly	Protein Coding	37
FOS	Fos Proto-Oncogene, AP-1 Transcription Factor Subuni	Protein Coding	52
SULT2B1	Sulfotransferase Family 2B Member 1	Protein Coding	47
PENK	Proenkephalin	Protein Coding	39
TP73	Tumor Protein P73	Protein Coding	47
LGI1	Leucine Rich Glioma Inactivated 1	Protein Coding	44
HBS1L	HBS1 Like Translational GTPase	Protein Coding	40
CALM1	Calmodulin 1	Protein Coding	45
MYL2	Myosin Light Chain 2	Protein Coding	50
C3	Complement C3	Protein Coding	49

TIMP3	TIMP Metallopeptidase Inhibitor 3	Protein Coding	47
GBA2	Glucosylceramidase Beta 2	Protein Coding	42
LINGO2	Leucine Rich Repeat And Ig Domain Containing 2	Protein Coding	36
MAPT-AS	MAPT Antisense RNA 1	RNA Gene	18
CSTB	Cystatin B	Protein Coding	47
ATL1	Atlastin GTPase 1	Protein Coding	42
DAO	D-Amino Acid Oxidase	Protein Coding	46
SERPINH	Serpin Family H Member 1	Protein Coding	47
AQP2	Aquaporin 2	Protein Coding	48
VPS29	VPS29 Retromer Complex Component	Protein Coding	42
SOS1	SOS Ras/Rac Guanine Nucleotide Exchange Factor 1	Protein Coding	49
HMGB1	High Mobility Group Box 1	Protein Coding	45
WNT5A	Wnt Family Member 5A	Protein Coding	51
PIK3C3	Phosphatidylinositol 3-Kinase Catalytic Subunit Type 3	Protein Coding	50
CLN5	CLN5 Intracellular Trafficking Protein	Protein Coding	42
PAX3	Paired Box 3	Protein Coding	48
CDKL5	Cyclin Dependent Kinase Like 5	Protein Coding	43
SEMA3A	Semaphorin 3A	Protein Coding	46
MIR210	MicroRNA 210	RNA Gene	21
FAF1	Fas Associated Factor 1	Protein Coding	45
GYG1	Glycogenin 1	Protein Coding	47
AVPR2	Arginine Vasopressin Receptor 2	Protein Coding	50
REEP1	Receptor Accessory Protein 1	Protein Coding	40
BIN1	Bridging Integrator 1	Protein Coding	48
USP24	Ubiquitin Specific Peptidase 24	Protein Coding	43
PGK1	Phosphoglycerate Kinase 1	Protein Coding	50
TREX1	Three Prime Repair Exonuclease 1	Protein Coding	44
UBE2E2	Ubiquitin Conjugating Enzyme E2 E2	Protein Coding	40
HBG2	Hemoglobin Subunit Gamma 2	Protein Coding	43
SPTLC2	Serine Palmitoyltransferase Long Chain Base Subunit 2	Protein Coding	49
MT-ATP8	Mitochondrially Encoded ATP Synthase Membrane Sul	Protein Coding	30
ISL1	ISL LIM Homeobox 1	Protein Coding	47
CRX	Cone-Rod Homeobox	Protein Coding	44
CLCN1	Chloride Voltage-Gated Channel 1	Protein Coding	45
GPX1	Glutathione Peroxidase 1	Protein Coding	49
HDAC4	Histone Deacetylase 4	Protein Coding	52
TCOF1	Treacle Ribosome Biogenesis Factor 1	Protein Coding	44
CTNNA3	Catenin Alpha 3	Protein Coding	40
TCAP	Titin-Cap	Protein Coding	43
MAP2K3	Mitogen-Activated Protein Kinase Kinase 3	Protein Coding	51
HNMT	Histamine N-Methyltransferase	Protein Coding	44
SEPTIN4	Septin 4	Protein Coding	31
ATP5F1A	ATP Synthase F1 Subunit Alpha	Protein Coding	37
PIK3C2A	Phosphatidylinositol-4-Phosphate 3-Kinase Catalytic Su	Protein Coding	47
KCNA1	Potassium Voltage-Gated Channel Subfamily A Membe	Protein Coding	47
CARD9	Caspase Recruitment Domain Family Member 9	Protein Coding	45
ATP2C1	ATPase Secretory Pathway Ca2+ Transporting 1	Protein Coding	46

CLN8	CLN8 Transmembrane ER And ERGIC Protein	Protein Coding	41
TGFB2	Transforming Growth Factor Beta 2	Protein Coding	51
PPARA	Peroxisome Proliferator Activated Receptor Alpha	Protein Coding	47
DSG1	Desmoglein 1	Protein Coding	45
BCR	BCR Activator Of RhoGEF And GTPase	Protein Coding	53
EEF1A1	Eukaryotic Translation Elongation Factor 1 Alpha 1	Protein Coding	44
TBX1	T-Box Transcription Factor 1	Protein Coding	44
ATG12	Autophagy Related 12	Protein Coding	43
LOC1095(	Chromosome 9 Open Reading Frame 72 Repeat Instabil	Biological Region	2
RIC3	RIC3 Acetylcholine Receptor Chaperone	Protein Coding	35
MT-ND4L	Mitochondrially Encoded NADH:Ubiquinone Oxidoredu	Protein Coding	29
SCN3A	Sodium Voltage-Gated Channel Alpha Subunit 3	Protein Coding	48
YAP1	Yes Associated Protein 1	Protein Coding	49
PAX5	Paired Box 5	Protein Coding	48
BTD	Biotinidase	Protein Coding	45
MYCN	MYCN Proto-Oncogene, BHLH Transcription Factor	Protein Coding	48
CD209	CD209 Molecule	Protein Coding	44
KCTD7	Potassium Channel Tetramerization Domain Containing	Protein Coding	39
P2RX7	Purinergic Receptor P2X 7	Protein Coding	47
CDCP2	CUB Domain Containing Protein 2	Protein Coding	31
RETREG1	Reticulophagy Regulator 1	Protein Coding	32
MIR148B	MicroRNA 148b	RNA Gene	20
PSPN	Persephin	Protein Coding	40
SMC1A	Structural Maintenance Of Chromosomes 1A	Protein Coding	47
UNC13A	Unc-13 Homolog A	Protein Coding	41
NPPA	Natriuretic Peptide A	Protein Coding	48
ALDH2	Aldehyde Dehydrogenase 2 Family Member	Protein Coding	52
SEPTIN5	Septin 5	Protein Coding	32
GABRA1	Gamma-Aminobutyric Acid Type A Receptor Subunit A	Protein Coding	48
GSTT1	Glutathione S-Transferase Theta 1	Protein Coding	35
FOXRED1	FAD Dependent Oxidoreductase Domain Containing 1	Protein Coding	41
VLDLR	Very Low Density Lipoprotein Receptor	Protein Coding	49
NAGA	Alpha-N-Acetylgalactosaminidase	Protein Coding	44
MCOLN1	Mucolipin 1	Protein Coding	45
NDUFA11	NADH:Ubiquinone Oxidoreductase Subunit A11	Protein Coding	37
MYL3	Myosin Light Chain 3	Protein Coding	46
MIR93	MicroRNA 93	RNA Gene	21
TET1	Tet Methylcytosine Dioxygenase 1	Protein Coding	39
TBC1D5	TBC1 Domain Family Member 5	Protein Coding	38
NDUFAF6	NADH:Ubiquinone Oxidoreductase Complex Assembly	Protein Coding	36
ATP13A3	ATPase 13A3	Protein Coding	39
SNHG1	Small Nucleolar RNA Host Gene 1	RNA Gene	21
AARS2	Alanyl-TRNA Synthetase 2, Mitochondrial	Protein Coding	43
APAF1	Apoptotic Peptidase Activating Factor 1	Protein Coding	48
AQP4	Aquaporin 4	Protein Coding	45
WRN	WRN RecQ Like Helicase	Protein Coding	47
DPP6	Dipeptidyl Peptidase Like 6	Protein Coding	43

ERCC8	ERCC Excision Repair 8, CSA Ubiquitin Ligase Comp	Protein Coding	41
IFT43	Intraflagellar Transport 43	Protein Coding	37
GLS	Glutaminase	Protein Coding	48
MGMT	O-6-Methylguanine-DNA Methyltransferase	Protein Coding	50
USP40	Ubiquitin Specific Peptidase 40	Protein Coding	36
EPG5	Ectopic P-Granules Autophagy Protein 5 Homolog	Protein Coding	37
BCKDHA	Branched Chain Keto Acid Dehydrogenase E1 Subunit	Protein Coding	45
FGFR2	Fibroblast Growth Factor Receptor 2	Protein Coding	55
NEDD8	NEDD8 Ubiquitin Like Modifier	Protein Coding	43
PDE8B	Phosphodiesterase 8B	Protein Coding	47
AGRN	Agrin	Protein Coding	45
EIF2AK3	Eukaryotic Translation Initiation Factor 2 Alpha Kinase	Protein Coding	50
TOMM20	Translocase Of Outer Mitochondrial Membrane 20	Protein Coding	41
EZR	Ezrin	Protein Coding	47
EEA1	Early Endosome Antigen 1	Protein Coding	45
MB	Myoglobin	Protein Coding	45
SPTAN1	Spectrin Alpha, Non-Erythrocytic 1	Protein Coding	49
GNB1	G Protein Subunit Beta 1	Protein Coding	47
HS1BP3	HCLS1 Binding Protein 3	Protein Coding	37
DICER1	Dicer 1, Ribonuclease III	Protein Coding	48
ARFGAP1	ADP Ribosylation Factor GTPase Activating Protein 1	Protein Coding	44
ALX4	ALX Homeobox 4	Protein Coding	40
ALMS1	ALMS1 Centrosome And Basal Body Associated Protei	Protein Coding	41
APH1A	Aph-1 Homolog A, Gamma-Secretase Subunit	Protein Coding	44
HSPA5	Heat Shock Protein Family A (Hsp70) Member 5	Protein Coding	48
MAP3K13	Mitogen-Activated Protein Kinase Kinase Kinase 13	Protein Coding	43
LTF	Lactotransferrin	Protein Coding	45
WWOX	WW Domain Containing Oxidoreductase	Protein Coding	48
COL1A1	Collagen Type I Alpha 1 Chain	Protein Coding	52
NDUFAF3	NADH:Ubiquinone Oxidoreductase Complex Assembly	Protein Coding	41
BCL2L1	BCL2 Like 1	Protein Coding	49
DBT	Dihydrolipoamide Branched Chain Transacylase E2	Protein Coding	43
CLEC7A	C-Type Lectin Domain Containing 7A	Protein Coding	45
PPT1	Palmitoyl-Protein Thioesterase 1	Protein Coding	47
NDUFAF4	NADH:Ubiquinone Oxidoreductase Complex Assembly	Protein Coding	43
PCSK1N	Proprotein Convertase Subtilisin/Kexin Type 1 Inhibito	Protein Coding	34
PIKFYVE	Phosphoinositide Kinase, FYVE-Type Zinc Finger Cont	Protein Coding	49
VDAC1	Voltage Dependent Anion Channel 1	Protein Coding	47
PLG	Plasminogen	Protein Coding	49
MIR6084	MicroRNA 6084	RNA Gene	11
MIR19A	MicroRNA 19a	RNA Gene	19
SIL1	SIL1 Nucleotide Exchange Factor	Protein Coding	41
MIR10B	MicroRNA 10b	RNA Gene	21
POU1F1	POU Class 1 Homeobox 1	Protein Coding	44
ATG5	Autophagy Related 5	Protein Coding	45
MARS1	Methionyl-TRNA Synthetase 1	Protein Coding	36
ARTN	Artemin	Protein Coding	41

PRL	Prolactin	Protein Coding	44
IFT74	Intraflagellar Transport 74	Protein Coding	37
TIMMDC1	Translocase Of Inner Mitochondrial Membrane Domain	Protein Coding	37
NGFR	Nerve Growth Factor Receptor	Protein Coding	47
ARL13B	ADP Ribosylation Factor Like GTPase 13B	Protein Coding	39
CREB1	CAMP Responsive Element Binding Protein 1	Protein Coding	50
HNRNPDL	Heterogeneous Nuclear Ribonucleoprotein D Like	Protein Coding	40
MFSD8	Major Facilitator Superfamily Domain Containing 8	Protein Coding	38
ARID1B	AT-Rich Interaction Domain 1B	Protein Coding	46
WAS	WASP Actin Nucleation Promoting Factor	Protein Coding	50
DNAH8	Dynein Axonemal Heavy Chain 8	Protein Coding	38
MIR182	MicroRNA 182	RNA Gene	20
HLA-DPB	Major Histocompatibility Complex, Class II, DP Beta 1	Protein Coding	45
GHRL	Ghrelin And Obestatin Prepropeptide	Protein Coding	44
CYP19A1	Cytochrome P450 Family 19 Subfamily A Member 1	Protein Coding	50
CLIP1	CAP-Gly Domain Containing Linker Protein 1	Protein Coding	45
DARS2	Aspartyl-TRNA Synthetase 2, Mitochondrial	Protein Coding	43
PGAM5	PGAM Family Member 5, Mitochondrial Serine/Threon	Protein Coding	35
CALB2	Calbindin 2	Protein Coding	42
PTRHD1	Peptidyl-TRNA Hydrolase Domain Containing 1	Protein Coding	35
PRKCSH	Protein Kinase C Substrate 80K-H	Protein Coding	45
CFAP410	Cilia And Flagella Associated Protein 410	Protein Coding	30
CCNF	Cyclin F	Protein Coding	41
PRSS1	Serine Protease 1	Protein Coding	47
GALC	Galactosylceramidase	Protein Coding	44
CTSC	Cathepsin C	Protein Coding	46
NUBPL	Nucleotide Binding Protein Like	Protein Coding	39
WDTC1	WD And Tetratricopeptide Repeats 1	Protein Coding	35
PTS	6-Pyruvoyltetrahydropterin Synthase	Protein Coding	48
CBSL	Cystathionine Beta-Synthase Like	Protein Coding	18
FLNA	Filamin A	Protein Coding	50
TGM5	Transglutaminase 5	Protein Coding	43
BCKDHB	Branched Chain Keto Acid Dehydrogenase E1 Subunit	Protein Coding	44
PEX5	Peroxisomal Biogenesis Factor 5	Protein Coding	43
GNPTAB	N-Acetylglucosamine-1-Phosphate Transferase Subunit	Protein Coding	42
GLUL	Glutamate-Ammonia Ligase	Protein Coding	51
TNFRSF1(	TNF Receptor Superfamily Member 10b	Protein Coding	52
PLA2G4A	Phospholipase A2 Group IVA	Protein Coding	50
MPV17	Mitochondrial Inner Membrane Protein MPV17	Protein Coding	43
TECPR2	Tectonin Beta-Propeller Repeat Containing 2	Protein Coding	36
ITM2B	Integral Membrane Protein 2B	Protein Coding	44
RBM8A	RNA Binding Motif Protein 8A	Protein Coding	41
ELP1	Elongator Complex Protein 1	Protein Coding	35
ADA	Adenosine Deaminase	Protein Coding	51
SAA1	Serum Amyloid A1	Protein Coding	43
MAP3K11	Mitogen-Activated Protein Kinase Kinase Kinase 11	Protein Coding	49
MAPK8IP	Mitogen-Activated Protein Kinase 8 Interacting Protein	Protein Coding	45

MIR106B	MicroRNA 106b	RNA Gene	22
MIR424	MicroRNA 424	RNA Gene	17
UBC	Ubiquitin C	Protein Coding	44
TBC1D24	TBC1 Domain Family Member 24	Protein Coding	39
ANXA5	Annexin A5	Protein Coding	48
CYP17A1	Cytochrome P450 Family 17 Subfamily A Member 1	Protein Coding	50
AXIN1	Axin 1	Protein Coding	48
STS	Steroid Sulfatase	Protein Coding	47
SORCS1	Sortilin Related VPS10 Domain Containing Receptor 1	Protein Coding	39
COL5A1	Collagen Type V Alpha 1 Chain	Protein Coding	45
NRGN	Neurogranin	Protein Coding	40
WASHC1	WASH Complex Subunit 1	Protein Coding	24
GABRB3	Gamma-Aminobutyric Acid Type A Receptor Subunit B	Protein Coding	50
TLR1	Toll Like Receptor 1	Protein Coding	49
EIF4A2	Eukaryotic Translation Initiation Factor 4A2	Protein Coding	47
MT-TS1	Mitochondrially Encoded TRNA-Ser (UCN) 1	RNA Gene	15
CDK1	Cyclin Dependent Kinase 1	Protein Coding	47
ARID1A	AT-Rich Interaction Domain 1A	Protein Coding	46
LIG4	DNA Ligase 4	Protein Coding	50
GLI2	GLI Family Zinc Finger 2	Protein Coding	49
ATG16L1	Autophagy Related 16 Like 1	Protein Coding	45
GABRG2	Gamma-Aminobutyric Acid Type A Receptor Subunit G	Protein Coding	48
ADAMTS2	ADAM Metallopeptidase With Thrombospondin Type 1	Protein Coding	44
ANO3	Anoctamin 3	Protein Coding	43
PRKCG	Protein Kinase C Gamma	Protein Coding	52
PC	Pyruvate Carboxylase	Protein Coding	48
SYN1	Synapsin I	Protein Coding	45
LMOD1	Leiomodin 1	Protein Coding	41
VIP	Vasoactive Intestinal Peptide	Protein Coding	46
ACTA2	Actin Alpha 2, Smooth Muscle	Protein Coding	49
PTH	Parathyroid Hormone	Protein Coding	48
GK	Glycerol Kinase	Protein Coding	48
CD44	CD44 Molecule (Indian Blood Group)	Protein Coding	49
SERPINI1	Serpin Family I Member 1	Protein Coding	47
MT-TQ	Mitochondrially Encoded TRNA-Gln (CAA/G)	RNA Gene	13
GLIS1	GLIS Family Zinc Finger 1	Protein Coding	34
TLR9	Toll Like Receptor 9	Protein Coding	46
SMAD3	SMAD Family Member 3	Protein Coding	50
HJV	Hemojuvelin BMP Co-Receptor	Protein Coding	37
SELE	Selectin E	Protein Coding	45
PML	Promyelocytic Leukemia	Protein Coding	47
PDHA1	Pyruvate Dehydrogenase E1 Subunit Alpha 1	Protein Coding	50
IL6R	Interleukin 6 Receptor	Protein Coding	50
COX6B1	Cytochrome C Oxidase Subunit 6B1	Protein Coding	43
HSPA1A	Heat Shock Protein Family A (Hsp70) Member 1A	Protein Coding	45
APTX	Aprataxin	Protein Coding	47
PRPH	Peripherin	Protein Coding	45

ARIH1	Ariadne RBR E3 Ubiquitin Protein Ligase 1	Protein Coding	40
PON3	Paraoxonase 3	Protein Coding	45
NCAM1	Neural Cell Adhesion Molecule 1	Protein Coding	47
OLR1	Oxidized Low Density Lipoprotein Receptor 1	Protein Coding	45
BRD4	Bromodomain Containing 4	Protein Coding	45
MFHAS1	Malignant Fibrous Histiocytoma Amplified Sequence 1	Protein Coding	36
ENG	Endoglin	Protein Coding	48
SCN1B	Sodium Voltage-Gated Channel Beta Subunit 1	Protein Coding	45
GH1	Growth Hormone 1	Protein Coding	46
TJP2	Tight Junction Protein 2	Protein Coding	47
ARFGAP3	ADP Ribosylation Factor GTPase Activating Protein 3	Protein Coding	41
CHRNB2	Cholinergic Receptor Nicotinic Beta 2 Subunit	Protein Coding	45
ARX	Aristaless Related Homeobox	Protein Coding	43
CYP27A1	Cytochrome P450 Family 27 Subfamily A Member 1	Protein Coding	48
SET	SET Nuclear Proto-Oncogene	Protein Coding	49
AHR	Aryl Hydrocarbon Receptor	Protein Coding	50
UBE3C	Ubiquitin Protein Ligase E3C	Protein Coding	41
TXN	Thioredoxin	Protein Coding	47
SPTBN2	Spectrin Beta, Non-Erythrocytic 2	Protein Coding	43
XRCC1	X-Ray Repair Cross Complementing 1	Protein Coding	45
SORCS2	Sortilin Related VPS10 Domain Containing Receptor 2	Protein Coding	37
APOC3	Apolipoprotein C3	Protein Coding	44
IFNA1	Interferon Alpha 1	Protein Coding	40
MIF	Macrophage Migration Inhibitory Factor	Protein Coding	51
SNX1	Sorting Nexin 1	Protein Coding	41
DOCK8	Dedicator Of Cytokinesis 8	Protein Coding	44
CRHR1	Corticotropin Releasing Hormone Receptor 1	Protein Coding	46
PLA2G7	Phospholipase A2 Group VII	Protein Coding	51
SLC33A1	Solute Carrier Family 33 Member 1	Protein Coding	44
HSPB8	Heat Shock Protein Family B (Small) Member 8	Protein Coding	46
PHYH	Phytanoyl-CoA 2-Hydroxylase	Protein Coding	45
MAP1B	Microtubule Associated Protein 1B	Protein Coding	44
PIN1	Peptidylprolyl Cis/Trans Isomerase, NIMA-Interacting	Protein Coding	47
NOP56	NOP56 Ribonucleoprotein	Protein Coding	43
ANKRD5(	Ankyrin Repeat Domain 50	Protein Coding	33
MLH1	MutL Homolog 1	Protein Coding	49
PTPRC	Protein Tyrosine Phosphatase Receptor Type C	Protein Coding	52
KNG1	Kininogen 1	Protein Coding	45
MT-TS2	Mitochondrially Encoded TRNA-Ser (AGU/C) 2	RNA Gene	14
IBA57	Iron-Sulfur Cluster Assembly Factor IBA57	Protein Coding	36
GCG	Glucagon	Protein Coding	42
OCRL	OCRL Inositol Polyphosphate-5-Phosphatase	Protein Coding	45
BGLAP	Bone Gamma-Carboxyglutamate Protein	Protein Coding	41
TPPP	Tubulin Polymerization Promoting Protein	Protein Coding	41
TCIRG1	T Cell Immune Regulator 1, ATPase H+ Transporting \	Protein Coding	45
ENTR1	Endosome Associated Trafficking Regulator 1	Protein Coding	29
HSPBP1	HSPA (Hsp70) Binding Protein 1	Protein Coding	37

ETFDH	Electron Transfer Flavoprotein Dehydrogenase	Protein Coding	45
LARS2	Leucyl-TRNA Synthetase 2, Mitochondrial	Protein Coding	46
SNX6	Sorting Nexin 6	Protein Coding	37
TTPA	Alpha Tocopherol Transfer Protein	Protein Coding	42
ACADS	Acyl-CoA Dehydrogenase Short Chain	Protein Coding	47
VCAM1	Vascular Cell Adhesion Molecule 1	Protein Coding	47
YARS2	Tyrosyl-TRNA Synthetase 2	Protein Coding	44
TPK1	Thiamin Pyrophosphokinase 1	Protein Coding	46
TTBK2	Tau Tubulin Kinase 2	Protein Coding	43
PTGS1	Prostaglandin-Endoperoxide Synthase 1	Protein Coding	48
MYD88	MYD88 Innate Immune Signal Transduction Adaptor	Protein Coding	51
ETFA	Electron Transfer Flavoprotein Subunit Alpha	Protein Coding	46
TRPC6	Transient Receptor Potential Cation Channel Subfamily	Protein Coding	50
PES1	Pescadillo Ribosomal Biogenesis Factor 1	Protein Coding	39
HM13	Histocompatibility Minor 13	Protein Coding	39
KEAP1	Kelch Like ECH Associated Protein 1	Protein Coding	48
ACO2	Aconitase 2	Protein Coding	48
AMPD1	Adenosine Monophosphate Deaminase 1	Protein Coding	46
CAMKK2	Calcium/Calmodulin Dependent Protein Kinase Kinase	Protein Coding	48
ALYREF	Aly/REF Export Factor	Protein Coding	38
EPHB2	EPH Receptor B2	Protein Coding	53
SI	Sucrase-Isomaltase	Protein Coding	44
UBQLN1	Ubiquilin 1	Protein Coding	44
PMPCA	Peptidase, Mitochondrial Processing Subunit Alpha	Protein Coding	44
SRGAP2	SLIT-ROBO Rho GTPase Activating Protein 2	Protein Coding	36
APEX1	Apurinic/Apyrimidinic Endodeoxyribonuclease 1	Protein Coding	47
SNX5	Sorting Nexin 5	Protein Coding	43
DLST	Dihydrolipoamide S-Succinyltransferase	Protein Coding	47
IFNB1	Interferon Beta 1	Protein Coding	43
NR2E3	Nuclear Receptor Subfamily 2 Group E Member 3	Protein Coding	41
ANTXR2	ANTXR Cell Adhesion Molecule 2	Protein Coding	48
SCO2	Synthesis Of Cytochrome C Oxidase 2	Protein Coding	46
FGF14	Fibroblast Growth Factor 14	Protein Coding	45
PEX3	Peroxisomal Biogenesis Factor 3	Protein Coding	43
GP1BA	Glycoprotein Ib Platelet Subunit Alpha	Protein Coding	47
PSMB8	Proteasome 20S Subunit Beta 8	Protein Coding	51
PRICKLE	Prickle Planar Cell Polarity Protein 1	Protein Coding	43
NPHP1	Nephrocystin 1	Protein Coding	44
MAP2K2	Mitogen-Activated Protein Kinase Kinase 2	Protein Coding	55
PJVK	Pejvakin	Protein Coding	28
EXOSC3	Exosome Component 3	Protein Coding	43
LINC0126	Long Intergenic Non-Protein Coding RNA 1262	RNA Gene	14
SCN11A	Sodium Voltage-Gated Channel Alpha Subunit 11	Protein Coding	43
UNG	Uracil DNA Glycosylase	Protein Coding	48
BMP6	Bone Morphogenetic Protein 6	Protein Coding	45
APOC1	Apolipoprotein C1	Protein Coding	41
MT-TH	Mitochondrially Encoded TRNA-His (CAU/C)	RNA Gene	14

PRDX5	Peroxiredoxin 5	Protein Coding	48
ERLIN2	ER Lipid Raft Associated 2	Protein Coding	41
GAD2	Glutamate Decarboxylase 2	Protein Coding	47
GNAQ	G Protein Subunit Alpha Q	Protein Coding	51
CLPB	Caseinolytic Mitochondrial Matrix Peptidase Chaperone	Protein Coding	44
MIR155	MicroRNA 155	RNA Gene	19
KCNC3	Potassium Voltage-Gated Channel Subfamily C Membe	Protein Coding	44
PLCB4	Phospholipase C Beta 4	Protein Coding	48
AXIN2	Axin 2	Protein Coding	49
VAC14	VAC14 Component Of PIKFYVE Complex	Protein Coding	43
HSPB2	Heat Shock Protein Family B (Small) Member 2	Protein Coding	42
POTEF	POTE Ankyrin Domain Family Member F	Protein Coding	29
PRKAG2-.	PRKAG2 Antisense RNA 1	RNA Gene	15
OGT	O-Linked N-Acetylglucosamine (GlcNAc) Transferase	Protein Coding	45
OPA1	OPA1 Mitochondrial Dynamin Like GTPase	Protein Coding	44
PLEKHG5	Pleckstrin Homology And RhoGEF Domain Containing	Protein Coding	41
LHX3	LIM Homeobox 3	Protein Coding	43
MT-TF	Mitochondrially Encoded TRNA-Phe (UUU/C)	RNA Gene	14
SNX2	Sorting Nexin 2	Protein Coding	40
COX10	Cytochrome C Oxidase Assembly Factor Heme A:Farne	Protein Coding	45
SPART	Spartin	Protein Coding	32
TALDO1	Transaldolase 1	Protein Coding	47
NOTCH3	Notch Receptor 3	Protein Coding	50
MSMB	Microseminoprotein Beta	Protein Coding	41
CFI	Complement Factor I	Protein Coding	48
FLT1	Fms Related Receptor Tyrosine Kinase 1	Protein Coding	52
NT5C1A	5'-Nucleotidase, Cytosolic IA	Protein Coding	40
KCNT1	Potassium Sodium-Activated Channel Subfamily T Mer	Protein Coding	44
DLD	Dihydrolipoamide Dehydrogenase	Protein Coding	51
P4HTM	Prolyl 4-Hydroxylase, Transmembrane	Protein Coding	37
FLI1	Fli-1 Proto-Oncogene, ETS Transcription Factor	Protein Coding	51
MSR1	Macrophage Scavenger Receptor 1	Protein Coding	47
TGM6	Transglutaminase 6	Protein Coding	39
BCOR	BCL6 Corepressor	Protein Coding	43
PLD1	Phospholipase D1	Protein Coding	50
ITGB2	Integrin Subunit Beta 2	Protein Coding	52
GFM1	G Elongation Factor Mitochondrial 1	Protein Coding	45
GDF15	Growth Differentiation Factor 15	Protein Coding	43
VPS26B	VPS26, Retromer Complex Component B	Protein Coding	36
ENO1	Enolase 1	Protein Coding	48
PCA3	Prostate Cancer Associated 3	RNA Gene	23
FRZB	Frizzled Related Protein	Protein Coding	43
C1R	Complement C1r	Protein Coding	48
BAG3	BAG Cochaperone 3	Protein Coding	45
MYOD1	Myogenic Differentiation 1	Protein Coding	47
CPOX	Coproporphyrinogen Oxidase	Protein Coding	44
ISCU	Iron-Sulfur Cluster Assembly Enzyme	Protein Coding	45

KIF7	Kinesin Family Member 7	Protein Coding	40
KDR	Kinase Insert Domain Receptor	Protein Coding	55
IKBKG	Inhibitor Of Nuclear Factor Kappa B Kinase Regulator	Protein Coding	50
MTRR	5-Methyltetrahydrofolate-Homocysteine Methyltransfer	Protein Coding	44
HPGDS	Hematopoietic Prostaglandin D Synthase	Protein Coding	43
CYP2E1	Cytochrome P450 Family 2 Subfamily E Member 1	Protein Coding	47
RPL18	Ribosomal Protein L18	Protein Coding	45
SLC25A22	Solute Carrier Family 25 Member 22	Protein Coding	42
BECN1	Beclin 1	Protein Coding	48
EIF4A1	Eukaryotic Translation Initiation Factor 4A1	Protein Coding	45
AP4S1	Adaptor Related Protein Complex 4 Subunit Sigma 1	Protein Coding	39
GNGT2	G Protein Subunit Gamma Transducin 2	Protein Coding	41
MOGS	Mannosyl-Oligosaccharide Glucosidase	Protein Coding	43
ATP1A2	ATPase Na+/K+ Transporting Subunit Alpha 2	Protein Coding	49
TWIST2	Twist Family BHLH Transcription Factor 2	Protein Coding	43
COQ8A	Coenzyme Q8A	Protein Coding	36
VPS11	VPS11 Core Subunit Of CORVET And HOPS Comple	Protein Coding	40
NES	Nestin	Protein Coding	41
IL7	Interleukin 7	Protein Coding	44
HADHA	Hydroxyacyl-CoA Dehydrogenase Trifunctional Multie	Protein Coding	47
HRH2	Histamine Receptor H2	Protein Coding	45
ARHGAP3	Rho GTPase Activating Protein 39	Protein Coding	36
NAIP	NLR Family Apoptosis Inhibitory Protein	Protein Coding	41
GIGYF1	GRB10 Interacting GYF Protein 1	Protein Coding	35
RAD51C	RAD51 Paralog C	Protein Coding	44
MYH11	Myosin Heavy Chain 11	Protein Coding	47
AP5Z1	Adaptor Related Protein Complex 5 Subunit Zeta 1	Protein Coding	37
CACNA1H	Calcium Voltage-Gated Channel Subunit Alpha1 H	Protein Coding	51
LPA	Lipoprotein(A)	Protein Coding	41
SUMO2	Small Ubiquitin Like Modifier 2	Protein Coding	41
LGALS3	Galectin 3	Protein Coding	46
GRAP2	GRB2 Related Adaptor Protein 2	Protein Coding	44
MIR324	MicroRNA 324	RNA Gene	19
CSNK1D	Casein Kinase 1 Delta	Protein Coding	51
TAF15	TATA-Box Binding Protein Associated Factor 15	Protein Coding	44
MT-TT	Mitochondrially Encoded TRNA-Thr (ACN)	RNA Gene	16
PANK1	Pantothenate Kinase 1	Protein Coding	43
LRP8	LDL Receptor Related Protein 8	Protein Coding	47
CHEK1	Checkpoint Kinase 1	Protein Coding	51
POGZ	Pogo Transposable Element Derived With ZNF Domain	Protein Coding	41
MAP3K9	Mitogen-Activated Protein Kinase Kinase Kinase 9	Protein Coding	44
PRKAA1	Protein Kinase AMP-Activated Catalytic Subunit Alpha	Protein Coding	49
EFNB1	Ephrin B1	Protein Coding	48
ALAD	Aminolevulinate Dehydratase	Protein Coding	47
CALR	Calreticulin	Protein Coding	52
IL1R1	Interleukin 1 Receptor Type 1	Protein Coding	47
ADRA2B	Adrenoceptor Alpha 2B	Protein Coding	47

NGEF	Neuronal Guanine Nucleotide Exchange Factor	Protein Coding	40
AP4B1	Adaptor Related Protein Complex 4 Subunit Beta 1	Protein Coding	43
LOC10866	Ataxin 3 Repeat Instability Region	Biological Region	2
CTSF	Cathepsin F	Protein Coding	47
AP4M1	Adaptor Related Protein Complex 4 Subunit Mu 1	Protein Coding	40
RAF1	Raf-1 Proto-Oncogene, Serine/Threonine Kinase	Protein Coding	56
CTSH	Cathepsin H	Protein Coding	48
MIR30B	MicroRNA 30b	RNA Gene	21
VSNL1	Visinin Like 1	Protein Coding	43
RNU1-1	RNA, U1 Small Nuclear 1	RNA Gene	16
CHMP4B	Charged Multivesicular Body Protein 4B	Protein Coding	42
SLC16A2	Solute Carrier Family 16 Member 2	Protein Coding	46
WASHC3	WASH Complex Subunit 3	Protein Coding	28
IDE	Insulin Degrading Enzyme	Protein Coding	50
SUFU	SUFU Negative Regulator Of Hedgehog Signaling	Protein Coding	44
ARHGEF9	Cdc42 Guanine Nucleotide Exchange Factor 9	Protein Coding	41
TAZ	Tafazzin	Protein Coding	45
LORICRIN	Loricrin Cornified Envelope Precursor Protein	Protein Coding	28
IFIH1	Interferon Induced With Helicase C Domain 1	Protein Coding	49
LINC0173	Long Intergenic Non-Protein Coding RNA 1734	RNA Gene	10
VCL	Vinculin	Protein Coding	49
PHOX2A	Paired Like Homeobox 2A	Protein Coding	43
NUP85	Nucleoporin 85	Protein Coding	41
SYT12	Synaptotagmin 12	Protein Coding	36
MCM2	Minichromosome Maintenance Complex Component 2	Protein Coding	48
PCSK9	Proprotein Convertase Subtilisin/Kexin Type 9	Protein Coding	52
MT-TW	Mitochondrially Encoded TRNA-Trp (UGA/G)	RNA Gene	13
RANBP17	RAN Binding Protein 17	Protein Coding	39
ANK2	Ankyrin 2	Protein Coding	43
SNX14	Sorting Nexin 14	Protein Coding	37
SLC22A5	Solute Carrier Family 22 Member 5	Protein Coding	48
RPL35	Ribosomal Protein L35	Protein Coding	46
FYN	FYN Proto-Oncogene, Src Family Tyrosine Kinase	Protein Coding	50
SYNE1	Spectrin Repeat Containing Nuclear Envelope Protein 1	Protein Coding	42
REM1	RRAD And GEM Like GTPase 1	Protein Coding	35
SKP1	S-Phase Kinase Associated Protein 1	Protein Coding	44
VMA21	Vacuolar ATPase Assembly Factor VMA21	Protein Coding	36
CYP2C19	Cytochrome P450 Family 2 Subfamily C Member 19	Protein Coding	48
COQ7	Coenzyme Q7, Hydroxylase	Protein Coding	43
LMNB1	Lamin B1	Protein Coding	48
KCNA2	Potassium Voltage-Gated Channel Subfamily A Membe	Protein Coding	47
NR1H2	Nuclear Receptor Subfamily 1 Group H Member 2	Protein Coding	49
ANXA11	Annexin A11	Protein Coding	45
PSMD4	Proteasome 26S Subunit, Non-ATPase 4	Protein Coding	45
CASP7	Caspase 7	Protein Coding	51
CDKN2B-	CDKN2B Antisense RNA 1	RNA Gene	22
UNC5C	Unc-5 Netrin Receptor C	Protein Coding	42

RBFOX1	RNA Binding Fox-1 Homolog 1	Protein Coding	39
RPS6KB1	Ribosomal Protein S6 Kinase B1	Protein Coding	51
MMP8	Matrix Metallopeptidase 8	Protein Coding	48
CSF1	Colony Stimulating Factor 1	Protein Coding	45
EPHA4	EPH Receptor A4	Protein Coding	50
TBX22	T-Box Transcription Factor 22	Protein Coding	40
CD14	CD14 Molecule	Protein Coding	46
PHF1	PHD Finger Protein 1	Protein Coding	40
XBP1	X-Box Binding Protein 1	Protein Coding	47
LOC11080	Solute Carrier Family 6 Member 4 Gene Promoter	Biological Region	2
CYP1A1	Cytochrome P450 Family 1 Subfamily A Member 1	Protein Coding	48
NFKBIA	NFKB Inhibitor Alpha	Protein Coding	51
KDM6A	Lysine Demethylase 6A	Protein Coding	47
CALM2	Calmodulin 2	Protein Coding	47
SCOC-AS	SCOC Antisense RNA 1	RNA Gene	14
SLC52A2	Solute Carrier Family 52 Member 2	Protein Coding	39
PPM1D	Protein Phosphatase, Mg2+/Mn2+ Dependent 1D	Protein Coding	49
VAMP2	Vesicle Associated Membrane Protein 2	Protein Coding	44
HELLS	Helicase, Lymphoid Specific	Protein Coding	45
DCC	DCC Netrin 1 Receptor	Protein Coding	47
RNU4ATA	RNA, U4atac Small Nuclear (U12-Dependent Splicing)	RNA Gene	20
MIR4422H	MIR4422 Host Gene	RNA Gene	11
UCH1LAS	Antisense UCHL1	RNA Gene	4
OPRM1	Opioid Receptor Mu 1	Protein Coding	50
CS	Citrate Synthase	Protein Coding	45
CNTN2	Contactin 2	Protein Coding	47
CAPN2	Calpain 2	Protein Coding	48
IFNGR1	Interferon Gamma Receptor 1	Protein Coding	51
KCNQ3	Potassium Voltage-Gated Channel Subfamily Q Membe	Protein Coding	47
DPY19L2I	DPY19L2 Pseudogene 2	Pseudogene	21
UCHL1-A:	UCHL1 Antisense RNA 1 (Head To Head)	RNA Gene	16
LOC10868	FRAXA Repeat Instability Region	Biological Region	2
HCCS	Holocytochrome C Synthase	Protein Coding	43
DNAJB1	DnaJ Heat Shock Protein Family (Hsp40) Member B1	Protein Coding	45
LMOD3	Leiomodin 3	Protein Coding	40
PRPF6	Pre-MRNA Processing Factor 6	Protein Coding	43
XIAP	X-Linked Inhibitor Of Apoptosis	Protein Coding	51
ACTA1	Actin Alpha 1, Skeletal Muscle	Protein Coding	48
GATD1	Glutamine Amidotransferase Like Class 1 Domain Cont	Protein Coding	26
MIR126	MicroRNA 126	RNA Gene	23
VPS13B	Vacuolar Protein Sorting 13 Homolog B	Protein Coding	40
GOSR2	Golgi SNAP Receptor Complex Member 2	Protein Coding	44
TNFAIP3	TNF Alpha Induced Protein 3	Protein Coding	49
PIK3CG	Phosphatidylinositol-4,5-Bisphosphate 3-Kinase Cataly	Protein Coding	49
PSMB9	Proteasome 20S Subunit Beta 9	Protein Coding	47
RELA	RELA Proto-Oncogene, NF-KB Subunit	Protein Coding	51
JRK	Jrk Helix-Turn-Helix Protein	Protein Coding	36

SUCLG1	Succinate-CoA Ligase GDP/ADP-Forming Subunit Alp	Protein Coding	45
CDK5R1	Cyclin Dependent Kinase 5 Regulatory Subunit 1	Protein Coding	47
ATAD1	ATPase Family AAA Domain Containing 1	Protein Coding	41
CYFIP2	Cytoplasmic FMR1 Interacting Protein 2	Protein Coding	44
TRAPPC2	Trafficking Protein Particle Complex 2 Like	Protein Coding	37
MDH2	Malate Dehydrogenase 2	Protein Coding	50
HDAC9	Histone Deacetylase 9	Protein Coding	48
WFS1	Wolframin ER Transmembrane Glycoprotein	Protein Coding	45
IL12A	Interleukin 12A	Protein Coding	45
TYROBP	Transmembrane Immune Signaling Adaptor TYROBP	Protein Coding	44
MGC3280	Uncharacterized LOC153163	RNA Gene	12
TAP1	Transporter 1, ATP Binding Cassette Subfamily B Men	Protein Coding	48
DNAJC12	DnaJ Heat Shock Protein Family (Hsp40) Member C12	Protein Coding	40
ACAN	Aggrecan	Protein Coding	47
IL15	Interleukin 15	Protein Coding	42
RHOA	Ras Homolog Family Member A	Protein Coding	48
MT-TV	Mitochondrially Encoded TRNA-Val (GUN)	RNA Gene	15
RTN2	Reticulon 2	Protein Coding	41
MIR659	MicroRNA 659	RNA Gene	18
TENM4	Teneurin Transmembrane Protein 4	Protein Coding	35
PAWR	Pro-Apoptotic WT1 Regulator	Protein Coding	41
HBA2	Hemoglobin Subunit Alpha 2	Protein Coding	43
CSRP3	Cysteine And Glycine Rich Protein 3	Protein Coding	42
AIFM1	Apoptosis Inducing Factor Mitochondria Associated 1	Protein Coding	51
TAF1L	TATA-Box Binding Protein Associated Factor 1 Like	Protein Coding	34
FLVCR1	FLVCR Heme Transporter 1	Protein Coding	41
BPTF	Bromodomain PHD Finger Transcription Factor	Protein Coding	41
GPHN	Gephyrin	Protein Coding	48
ALG11	ALG11 Alpha-1,2-Mannosyltransferase	Protein Coding	41
ALAS2	5'-Aminolevulinate Synthase 2	Protein Coding	46
PAX2	Paired Box 2	Protein Coding	48
DSP	Desmoplakin	Protein Coding	51
COL25A1	Collagen Type XXV Alpha 1 Chain	Protein Coding	40
DHCR24	24-Dehydrocholesterol Reductase	Protein Coding	45
HLA-G	Major Histocompatibility Complex, Class I, G	Protein Coding	46
TAP2	Transporter 2, ATP Binding Cassette Subfamily B Men	Protein Coding	45
CXCL10	C-X-C Motif Chemokine Ligand 10	Protein Coding	45
PRKAA2	Protein Kinase AMP-Activated Catalytic Subunit Alpha	Protein Coding	51
KCNJ11	Potassium Inwardly Rectifying Channel Subfamily J Me	Protein Coding	49
GATA5	GATA Binding Protein 5	Protein Coding	40
HLA-DPA	Major Histocompatibility Complex, Class II, DP Alpha	Protein Coding	42
HPGD	15-Hydroxyprostaglandin Dehydrogenase	Protein Coding	50
ZAP70	Zeta Chain Of T Cell Receptor Associated Protein Kina	Protein Coding	53
VSX2	Visual System Homeobox 2	Protein Coding	41
PSNP2	Supranuclear Palsy, Progressive, 2	Genetic Locus	3
PTPRQ	Protein Tyrosine Phosphatase Receptor Type Q	Protein Coding	35
SHBG	Sex Hormone Binding Globulin	Protein Coding	43

TRPV1	Transient Receptor Potential Cation Channel Subfamily	Protein Coding	47
CIZ1	CDKN1A Interacting Zinc Finger Protein 1	Protein Coding	38
TMEM70	Transmembrane Protein 70	Protein Coding	39
OXT	Oxytocin/Neurophysin I Prepropeptide	Protein Coding	41
CLCN2	Chloride Voltage-Gated Channel 2	Protein Coding	46
APOD	Apolipoprotein D	Protein Coding	44
MIR320A	MicroRNA 320a	RNA Gene	20
DOCK3	Dedicator Of Cytokinesis 3	Protein Coding	42
UBQLN4	Ubiquilin 4	Protein Coding	41
HNF4A	Hepatocyte Nuclear Factor 4 Alpha	Protein Coding	52
MIR128-1	MicroRNA 128-1	RNA Gene	20
POU5F1	POU Class 5 Homeobox 1	Protein Coding	48
PECAM1	Platelet And Endothelial Cell Adhesion Molecule 1	Protein Coding	41
HTRA1	HtrA Serine Peptidase 1	Protein Coding	44
FARS2	Phenylalanyl-TRNA Synthetase 2, Mitochondrial	Protein Coding	45
MYRF	Myelin Regulatory Factor	Protein Coding	35
SLC25A11	Solute Carrier Family 25 Member 11	Protein Coding	44
DDHD2	DDHD Domain Containing 2	Protein Coding	41
SLC6A20	Solute Carrier Family 6 Member 20	Protein Coding	41
TBX4	T-Box Transcription Factor 4	Protein Coding	42
INA	Internexin Neuronal Intermediate Filament Protein Alph	Protein Coding	41
UQCRFS1	Ubiquinol-Cytochrome C Reductase, Rieske Iron-Sulfur	Protein Coding	44
VTN	Vitronectin	Protein Coding	45
RTN4	Reticulon 4	Protein Coding	46
IARS2	Isoleucyl-TRNA Synthetase 2, Mitochondrial	Protein Coding	42
LOXL3	Lysyl Oxidase Like 3	Protein Coding	41
CIB1	Calcium And Integrin Binding 1	Protein Coding	42
GLB1	Galactosidase Beta 1	Protein Coding	48
BLVRB	Biliverdin Reductase B	Protein Coding	41
LPP	LIM Domain Containing Preferred Translocation Partne	Protein Coding	44
M6PR	Mannose-6-Phosphate Receptor, Cation Dependent	Protein Coding	44
TACR1	Tachykinin Receptor 1	Protein Coding	47
NIPA1	NIPA Magnesium Transporter 1	Protein Coding	39
LRP6	LDL Receptor Related Protein 6	Protein Coding	49
IGFBP3	Insulin Like Growth Factor Binding Protein 3	Protein Coding	47
PRKACA	Protein Kinase CAMP-Activated Catalytic Subunit Alpl	Protein Coding	52
ADCY10	Adenylate Cyclase 10	Protein Coding	45
GPD2	Glycerol-3-Phosphate Dehydrogenase 2	Protein Coding	47
PCDH19	Protocadherin 19	Protein Coding	43
CDK5RAF	CDK5 Regulatory Subunit Associated Protein 2	Protein Coding	43
ZFYVE27	Zinc Finger FYVE-Type Containing 27	Protein Coding	38
GAMT	Guanidinoacetate N-Methyltransferase	Protein Coding	47
TMEM24C	Transmembrane Protein 240	Protein Coding	33
PRPS1	Phosphoribosyl Pyrophosphate Synthetase 1	Protein Coding	45
ADCY5	Adenylate Cyclase 5	Protein Coding	49
SERPINF1	Serpin Family F Member 1	Protein Coding	46
RAC1	Rac Family Small GTPase 1	Protein Coding	51

VPS53	VPS53 Subunit Of GARP Complex	Protein Coding	41
CALM3	Calmodulin 3	Protein Coding	43
EDAR	Ectodysplasin A Receptor	Protein Coding	43
MT-TP	Mitochondrially Encoded TRNA-Pro (CCN)	RNA Gene	11
GFI1B	Growth Factor Independent 1B Transcriptional Repress	Protein Coding	41
SLC2A3	Solute Carrier Family 2 Member 3	Protein Coding	50
PSNP3	Supranuclear Palsy, Progressive, 3	Genetic Locus	3
NEFM	Neurofilament Medium	Protein Coding	44
SELENOT	Selenoprotein T	Protein Coding	29
AQP1	Aquaporin 1 (Colton Blood Group)	Protein Coding	47
CASP6	Caspase 6	Protein Coding	50
ETFB	Electron Transfer Flavoprotein Subunit Beta	Protein Coding	46
LIF	LIF Interleukin 6 Family Cytokine	Protein Coding	44
FOXO3	Forkhead Box O3	Protein Coding	45
MIR92A1	MicroRNA 92a-1	RNA Gene	20
CHEK2	Checkpoint Kinase 2	Protein Coding	55
MITF	Melanocyte Inducing Transcription Factor	Protein Coding	48
MT-RNR1	Mitochondrially Encoded 12S RRNA	RNA Gene	16
MOBP	Myelin Associated Oligodendrocyte Basic Protein	Protein Coding	37
TPMT	Thiopurine S-Methyltransferase	Protein Coding	49
SCN4B	Sodium Voltage-Gated Channel Beta Subunit 4	Protein Coding	44
MIAT	Myocardial Infarction Associated Transcript	RNA Gene	22
HNRNPH2	Heterogeneous Nuclear Ribonucleoprotein H2	Protein Coding	39
CRAT	Carnitine O-Acetyltransferase	Protein Coding	45
PSMC4	Proteasome 26S Subunit, ATPase 4	Protein Coding	41
MIR30D	MicroRNA 30d	RNA Gene	17
GANAB	Glucosidase II Alpha Subunit	Protein Coding	46
SOX18	SRY-Box Transcription Factor 18	Protein Coding	41
FGB	Fibrinogen Beta Chain	Protein Coding	47
ITPR3	Inositol 1,4,5-Trisphosphate Receptor Type 3	Protein Coding	47
APOH	Apolipoprotein H	Protein Coding	45
ATRX	ATRX Chromatin Remodeler	Protein Coding	47
RHO	Rhodopsin	Protein Coding	49
NALCN	Sodium Leak Channel, Non-Selective	Protein Coding	41
PIGQ	Phosphatidylinositol Glycan Anchor Biosynthesis Class	Protein Coding	43
SETD2	SET Domain Containing 2, Histone Lysine Methyltrans	Protein Coding	47
SOX11	SRY-Box Transcription Factor 11	Protein Coding	41
AKR1B1	Aldo-Keto Reductase Family 1 Member B	Protein Coding	49
SUGCT	Succinyl-CoA:Glutarate-CoA Transferase	Protein Coding	37
IDH2	Isocitrate Dehydrogenase (NADP(+)) 2	Protein Coding	53
COL3A1	Collagen Type III Alpha 1 Chain	Protein Coding	48
FLNC	Filamin C	Protein Coding	45
TPM1	Tropomyosin 1	Protein Coding	50
PCBD1	Pterin-4 Alpha-Carbinolamine Dehydratase 1	Protein Coding	47
PRND	Prion Like Protein Doppel	Protein Coding	39
FGF1	Fibroblast Growth Factor 1	Protein Coding	48
CASP2	Caspase 2	Protein Coding	50

PLEKHG4	Pleckstrin Homology And RhoGEF Domain Containing	Protein Coding	41
PDE6A	Phosphodiesterase 6A	Protein Coding	48
SAMD12	Sterile Alpha Motif Domain Containing 12	Protein Coding	38
ANXA2	Annexin A2	Protein Coding	49
MIR107	MicroRNA 107	RNA Gene	20
COQ4	Coenzyme Q4	Protein Coding	40
CR2	Complement C3d Receptor 2	Protein Coding	46
TPH2	Tryptophan Hydroxylase 2	Protein Coding	48
MICA	MHC Class I Polypeptide-Related Sequence A	Protein Coding	40
SCN2B	Sodium Voltage-Gated Channel Beta Subunit 2	Protein Coding	45
PNPO	Pyridoxamine 5'-Phosphate Oxidase	Protein Coding	46
CTC1	CST Telomere Replication Complex Component 1	Protein Coding	35
PRKCD	Protein Kinase C Delta	Protein Coding	55
TUBB	Tubulin Beta Class I	Protein Coding	51
MT-TL2	Mitochondrially Encoded TRNA-Leu (CUN) 2	RNA Gene	14
ZEB2	Zinc Finger E-Box Binding Homeobox 2	Protein Coding	50
CHGB	Chromogranin B	Protein Coding	41
MIR30A	MicroRNA 30a	RNA Gene	20
STAT6	Signal Transducer And Activator Of Transcription 6	Protein Coding	51
HDAC6	Histone Deacetylase 6	Protein Coding	53
EIF2AK2	Eukaryotic Translation Initiation Factor 2 Alpha Kinase	Protein Coding	47
GUSB	Glucuronidase Beta	Protein Coding	48
RUNX1	RUNX Family Transcription Factor 1	Protein Coding	50
PLEC	Plectin	Protein Coding	43
CTSK	Cathepsin K	Protein Coding	50
NDUFB10	NADH:Ubiquinone Oxidoreductase Subunit B10	Protein Coding	43
PTPA	Protein Phosphatase 2 Phosphatase Activator	Protein Coding	36
CDR1	Cerebellar Degeneration Related Protein 1	Protein Coding	32
KCND3	Potassium Voltage-Gated Channel Subfamily D Membe	Protein Coding	47
UBE2G2	Ubiquitin Conjugating Enzyme E2 G2	Protein Coding	45
PRKCB	Protein Kinase C Beta	Protein Coding	49
BTNL2	Butyrophilin Like 2	Protein Coding	39
MIR195	MicroRNA 195	RNA Gene	20
MIR199B	MicroRNA 199b	RNA Gene	20
PRKCA	Protein Kinase C Alpha	Protein Coding	52
GDI1	GDP Dissociation Inhibitor 1	Protein Coding	45
HSD3B7	Hydroxy-Delta-5-Steroid Dehydrogenase, 3 Beta- And S	Protein Coding	43
ATG7	Autophagy Related 7	Protein Coding	44
MMP14	Matrix Metallopeptidase 14	Protein Coding	52
ATP13A5	ATPase 13A5	Protein Coding	34
NPM1	Nucleophosmin 1	Protein Coding	50
NPTX2	Neuronal Pentraxin 2	Protein Coding	40
NPEPPS	Aminopeptidase Puromycin Sensitive	Protein Coding	43
NANOG	Nanog Homeobox	Protein Coding	41
SMARCA2	SWI/SNF Related, Matrix Associated, Actin Dependent	Protein Coding	51
TNFSF13I	TNF Superfamily Member 13b	Protein Coding	47
MIR376A1	MicroRNA 376a-1	RNA Gene	16

ARNT2	Aryl Hydrocarbon Receptor Nuclear Translocator 2	Protein Coding	45
LCN2	Lipocalin 2	Protein Coding	45
TEK	TEK Receptor Tyrosine Kinase	Protein Coding	52
TUBA8	Tubulin Alpha 8	Protein Coding	47
ATPAF2	ATP Synthase Mitochondrial F1 Complex Assembly Fa	Protein Coding	39
DDHD1	DDHD Domain Containing 1	Protein Coding	37
ORAI1	ORAI Calcium Release-Activated Calcium Modulator 1	Protein Coding	45
ITGA2B	Integrin Subunit Alpha 2b	Protein Coding	52
MC1R	Melanocortin 1 Receptor	Protein Coding	48
EPHX1	Epoxide Hydrolase 1	Protein Coding	48
GRIA2	Glutamate Ionotropic Receptor AMPA Type Subunit 2	Protein Coding	50
CCND2	Cyclin D2	Protein Coding	51
ALG2	ALG2 Alpha-1,3/1,6-Mannosyltransferase	Protein Coding	42
PDE4D	Phosphodiesterase 4D	Protein Coding	50
BTK	Bruton Tyrosine Kinase	Protein Coding	55
AOC3	Amine Oxidase Copper Containing 3	Protein Coding	45
ANO5	Anoctamin 5	Protein Coding	39
CYP1A2	Cytochrome P450 Family 1 Subfamily A Member 2	Protein Coding	47
MIR221	MicroRNA 221	RNA Gene	21
IFT88	Intraflagellar Transport 88	Protein Coding	41
STAT5B	Signal Transducer And Activator Of Transcription 5B	Protein Coding	50
SOD3	Superoxide Dismutase 3	Protein Coding	40
PURA	Purine Rich Element Binding Protein A	Protein Coding	44
SDHC	Succinate Dehydrogenase Complex Subunit C	Protein Coding	46
THBS1	Thrombospondin 1	Protein Coding	45
TBX2	T-Box Transcription Factor 2	Protein Coding	45
SLC25A24	Solute Carrier Family 25 Member 24	Protein Coding	44
S100A8	S100 Calcium Binding Protein A8	Protein Coding	44
PDSS2	Decaprenyl Diphosphate Synthase Subunit 2	Protein Coding	41
MIR197	MicroRNA 197	RNA Gene	20
LDHA	Lactate Dehydrogenase A	Protein Coding	53
MIR124-1	MicroRNA 124-1	RNA Gene	21
DDX3X	DEAD-Box Helicase 3 X-Linked	Protein Coding	49
GTPBP3	GTP Binding Protein 3, Mitochondrial	Protein Coding	41
ATP1A1	ATPase Na+/K+ Transporting Subunit Alpha 1	Protein Coding	51
MKKS	McKusick-Kaufman Syndrome	Protein Coding	40
BCL2L11	BCL2 Like 11	Protein Coding	47
CHRNA2	Cholinergic Receptor Nicotinic Alpha 2 Subunit	Protein Coding	47
SLC22A18	Solute Carrier Family 22 Member 18	Protein Coding	44
ZMYM3	Zinc Finger MYM-Type Containing 3	Protein Coding	36
PCNA	Proliferating Cell Nuclear Antigen	Protein Coding	52
CTSL	Cathepsin L	Protein Coding	48
UBTF	Upstream Binding Transcription Factor	Protein Coding	44
COQ9	Coenzyme Q9	Protein Coding	42
SCN3B	Sodium Voltage-Gated Channel Beta Subunit 3	Protein Coding	45
MT-TE	Mitochondrially Encoded TRNA-Glu (GAA/G)	RNA Gene	13
TIMP2	TIMP Metallopeptidase Inhibitor 2	Protein Coding	45

GSS	Glutathione Synthetase	Protein Coding	48
CCR2	C-C Motif Chemokine Receptor 2	Protein Coding	47
PTRH2	Peptidyl-TRNA Hydrolase 2	Protein Coding	45
PDSS1	Decaprenyl Diphosphate Synthase Subunit 1	Protein Coding	44
MYOCD	Myocardin	Protein Coding	41
CDKN2C	Cyclin Dependent Kinase Inhibitor 2C	Protein Coding	48
CHERP	Calcium Homeostasis Endoplasmic Reticulum Protein	Protein Coding	35
MOG	Myelin Oligodendrocyte Glycoprotein	Protein Coding	47
RAI1	Retinoic Acid Induced 1	Protein Coding	41
BAK1	BCL2 Antagonist/Killer 1	Protein Coding	46
TRAF6	TNF Receptor Associated Factor 6	Protein Coding	48
SLC6A1	Solute Carrier Family 6 Member 1	Protein Coding	49
GPX3	Glutathione Peroxidase 3	Protein Coding	46
WARS2	Tryptophanyl TRNA Synthetase 2, Mitochondrial	Protein Coding	45
CARS2	Cysteinyl-TRNA Synthetase 2, Mitochondrial	Protein Coding	41
MEGF10	Multiple EGF Like Domains 10	Protein Coding	41
TRIT1	TRNA Isopentenyltransferase 1	Protein Coding	43
PSMF1	Proteasome Inhibitor Subunit 1	Protein Coding	40
BAD	BCL2 Associated Agonist Of Cell Death	Protein Coding	48
QDPR	Quinoid Dihydropteridine Reductase	Protein Coding	50
SCYL1	SCY1 Like Pseudokinase 1	Protein Coding	43
UQCRQ	Ubiquinol-Cytochrome C Reductase Complex III Subur	Protein Coding	43
CDC6	Cell Division Cycle 6	Protein Coding	47
WNT2	Wnt Family Member 2	Protein Coding	45
TGFA	Transforming Growth Factor Alpha	Protein Coding	48
SPARC	Secreted Protein Acidic And Cysteine Rich	Protein Coding	52
COQ6	Coenzyme Q6, Monooxygenase	Protein Coding	43
IQSEC2	IQ Motif And Sec7 Domain ArfGEF 2	Protein Coding	39
ATF6	Activating Transcription Factor 6	Protein Coding	49
TNFSF10	TNF Superfamily Member 10	Protein Coding	48
INPP5B	Inositol Polyphosphate-5-Phosphatase B	Protein Coding	43
CYP2C9	Cytochrome P450 Family 2 Subfamily C Member 9	Protein Coding	50
HAVCR2	Hepatitis A Virus Cellular Receptor 2	Protein Coding	45
COL18A1	Collagen Type XVIII Alpha 1 Chain	Protein Coding	46
FAR1	Fatty Acyl-CoA Reductase 1	Protein Coding	44
SRF	Serum Response Factor	Protein Coding	43
PLCG1	Phospholipase C Gamma 1	Protein Coding	48
PKP2	Plakophilin 2	Protein Coding	46
TCF20	Transcription Factor 20	Protein Coding	39
TOP1	DNA Topoisomerase I	Protein Coding	48
GATA2	GATA Binding Protein 2	Protein Coding	49
CA2	Carbonic Anhydrase 2	Protein Coding	53
TRMU	TRNA 5-Methylaminomethyl-2-Thiouridylate Methyltra	Protein Coding	40
MUTYH	MutY DNA Glycosylase	Protein Coding	47
SYK	Spleen Associated Tyrosine Kinase	Protein Coding	51
AKT2	AKT Serine/Threonine Kinase 2	Protein Coding	56
CNR2	Cannabinoid Receptor 2	Protein Coding	47

ANO10	Anoctamin 10	Protein Coding	40
SFPQ	Splicing Factor Proline And Glutamine Rich	Protein Coding	44
EGR1	Early Growth Response 1	Protein Coding	45
NRXN1	Neurexin 1	Protein Coding	48
KDM4C	Lysine Demethylase 4C	Protein Coding	41
PRKG1	Protein Kinase CGMP-Dependent 1	Protein Coding	52
HDAC1	Histone Deacetylase 1	Protein Coding	51
COX15	Cytochrome C Oxidase Assembly Homolog COX15	Protein Coding	43
DNAJB2	DnaJ Heat Shock Protein Family (Hsp40) Member B2	Protein Coding	44
H1-2	H1.2 Linker Histone, Cluster Member	Protein Coding	34
MIR181C	MicroRNA 181c	RNA Gene	21
MAP3K5	Mitogen-Activated Protein Kinase Kinase Kinase 5	Protein Coding	49
MT-TI	Mitochondrially Encoded TRNA-Ile (AUU/C)	RNA Gene	14
GABRD	Gamma-Aminobutyric Acid Type A Receptor Subunit I	Protein Coding	47
DNMBP	Dynamin Binding Protein	Protein Coding	40
CACNB4	Calcium Voltage-Gated Channel Auxiliary Subunit Beta	Protein Coding	47
OCA2	OCA2 Melanosomal Transmembrane Protein	Protein Coding	43
KCNJ5	Potassium Inwardly Rectifying Channel Subfamily J Me	Protein Coding	48
FCGR3A	Fc Fragment Of IgG Receptor IIIa	Protein Coding	46
OSM	Oncostatin M	Protein Coding	44
ACAT1	Acetyl-CoA Acetyltransferase 1	Protein Coding	51
MGME1	Mitochondrial Genome Maintenance Exonuclease 1	Protein Coding	36
THAP3	THAP Domain Containing 3	Protein Coding	36
MIR214	MicroRNA 214	RNA Gene	20
SP1	Sp1 Transcription Factor	Protein Coding	45
GSK3A	Glycogen Synthase Kinase 3 Alpha	Protein Coding	50
ASXL1	ASXL Transcriptional Regulator 1	Protein Coding	44
ARSH	Arylsulfatase Family Member H	Protein Coding	35
KRT18	Keratin 18	Protein Coding	50
FABP3	Fatty Acid Binding Protein 3	Protein Coding	45
HSP90B1	Heat Shock Protein 90 Beta Family Member 1	Protein Coding	47
WDR81	WD Repeat Domain 81	Protein Coding	37
MRPL44	Mitochondrial Ribosomal Protein L44	Protein Coding	41
BLK	BLK Proto-Oncogene, Src Family Tyrosine Kinase	Protein Coding	52
CDH2	Cadherin 2	Protein Coding	51
PYY	Peptide YY	Protein Coding	43
MT3	Metallothionein 3	Protein Coding	40
YWHAE	Tyrosine 3-Monooxygenase/Tryptophan 5-Monooxygen	Protein Coding	51
PTCH2	Patched 2	Protein Coding	46
IL1RAPL2	Interleukin 1 Receptor Accessory Protein Like 2	Protein Coding	38
HMOX2	Heme Oxygenase 2	Protein Coding	49
TCN2	Transcobalamin 2	Protein Coding	45
RAB8A	RAB8A, Member RAS Oncogene Family	Protein Coding	41
PDE6H	Phosphodiesterase 6H	Protein Coding	42
BLOC1S1	Biogenesis Of Lysosomal Organelles Complex 1 Subun	Protein Coding	37
SLC4A1	Solute Carrier Family 4 Member 1 (Diego Blood Group	Protein Coding	48
MIR34B	MicroRNA 34b	RNA Gene	22

SLC25A6	Solute Carrier Family 25 Member 6	Protein Coding	44
DPYSL2	Dihydropyrimidinase Like 2	Protein Coding	47
KMT2C	Lysine Methyltransferase 2C	Protein Coding	41
ABCC9	ATP Binding Cassette Subfamily C Member 9	Protein Coding	46
SOCS3	Suppressor Of Cytokine Signaling 3	Protein Coding	45
BST1	Bone Marrow Stromal Cell Antigen 1	Protein Coding	43
HADHB	Hydroxyacyl-CoA Dehydrogenase Trifunctional Multie	Protein Coding	48
MYOT	Myotilin	Protein Coding	41
UQCC2	Ubiquinol-Cytochrome C Reductase Complex Assembl	Protein Coding	35
MBNL1	Muscleblind Like Splicing Regulator 1	Protein Coding	40
COPA	COPI Coat Complex Subunit Alpha	Protein Coding	42
PLAUR	Plasminogen Activator, Urokinase Receptor	Protein Coding	46
SREBF1	Sterol Regulatory Element Binding Transcription Facto	Protein Coding	45
POLR2A	RNA Polymerase II Subunit A	Protein Coding	45
DLEU2	Deleted In Lymphocytic Leukemia 2	RNA Gene	24
NUP62	Nucleoporin 62	Protein Coding	46
SLC12A5	Solute Carrier Family 12 Member 5	Protein Coding	48
GAN	Gigaxonin	Protein Coding	40
SYT1	Synaptotagmin 1	Protein Coding	47
SERPINE2	Serpin Family E Member 2	Protein Coding	43
PAFAH1B	Platelet Activating Factor Acetylhydrolase 1b Regulato	Protein Coding	47
ATP8A2	ATPase Phospholipid Transporting 8A2	Protein Coding	42
SAMHD1	SAM And HD Domain Containing Deoxynucleoside Tr	Protein Coding	44
ETM2	Essential Tremor 2	Genetic Locus	5
TRIM44	Tripartite Motif Containing 44	Protein Coding	39
RTN4IP1	Reticulon 4 Interacting Protein 1	Protein Coding	41
SDCCAG8	Serologically Defined Colon Cancer Antigen 8	Protein Coding	43
ATP6V0A	ATPase H+ Transporting V0 Subunit A2	Protein Coding	44
SMO	Smoothened, Frizzled Class Receptor	Protein Coding	50
MIR326	MicroRNA 326	RNA Gene	21
TBX19	T-Box Transcription Factor 19	Protein Coding	41
MIR30E	MicroRNA 30e	RNA Gene	21
CYP27B1	Cytochrome P450 Family 27 Subfamily B Member 1	Protein Coding	48
CCNE1	Cyclin E1	Protein Coding	48
ATP5PD	ATP Synthase Peripheral Stalk Subunit D	Protein Coding	33
GLT8D1	Glycosyltransferase 8 Domain Containing 1	Protein Coding	39
SETBP1	SET Binding Protein 1	Protein Coding	41
FCGR2B	Fc Fragment Of IgG Receptor IIb	Protein Coding	49
ABCD1	ATP Binding Cassette Subfamily D Member 1	Protein Coding	47
TUBA1A	Tubulin Alpha 1a	Protein Coding	50
TSFM	Ts Translation Elongation Factor, Mitochondrial	Protein Coding	43
FADD	Fas Associated Via Death Domain	Protein Coding	48
CCL26	C-C Motif Chemokine Ligand 26	Protein Coding	39
PSMD5	Proteasome 26S Subunit, Non-ATPase 5	Protein Coding	39
UQCRB	Ubiquinol-Cytochrome C Reductase Binding Protein	Protein Coding	43
MTHFD1	Methylenetetrahydrofolate Dehydrogenase, Cyclohydrol	Protein Coding	45
ETV6	ETS Variant Transcription Factor 6	Protein Coding	48

SOX4	SRY-Box Transcription Factor 4	Protein Coding	44
ATP5PO	ATP Synthase Peripheral Stalk Subunit OSCP	Protein Coding	33
AGTR2	Angiotensin II Receptor Type 2	Protein Coding	44
AKT3	AKT Serine/Threonine Kinase 3	Protein Coding	56
ACE2	Angiotensin I Converting Enzyme 2	Protein Coding	50
ACTG1	Actin Gamma 1	Protein Coding	51
IGFBP1	Insulin Like Growth Factor Binding Protein 1	Protein Coding	45
MIR328	MicroRNA 328	RNA Gene	19
DVL1	Dishevelled Segment Polarity Protein 1	Protein Coding	48
BIRC5	Baculoviral IAP Repeat Containing 5	Protein Coding	48
TOR1B	Torsin Family 1 Member B	Protein Coding	39
ITGA8	Integrin Subunit Alpha 8	Protein Coding	43
TUBG1	Tubulin Gamma 1	Protein Coding	49
PANK4	Pantothenate Kinase 4 (Inactive)	Protein Coding	41
SARS2	Seryl-TRNA Synthetase 2, Mitochondrial	Protein Coding	43
RPS6	Ribosomal Protein S6	Protein Coding	45
RPS3A	Ribosomal Protein S3A	Protein Coding	41
NSF	N-Ethylmaleimide Sensitive Factor, Vesicle Fusing ATI	Protein Coding	45
GABRB2	Gamma-Aminobutyric Acid Type A Receptor Subunit I	Protein Coding	48
TMPRSS6	Transmembrane Serine Protease 6	Protein Coding	45
ADH1B	Alcohol Dehydrogenase 1B (Class I), Beta Polypeptide	Protein Coding	44
COX4I1	Cytochrome C Oxidase Subunit 4I1	Protein Coding	45
ACTN4	Actinin Alpha 4	Protein Coding	47
CD80	CD80 Molecule	Protein Coding	43
CYP3A4	Cytochrome P450 Family 3 Subfamily A Member 4	Protein Coding	51
PIEZO1	Piezo Type Mechanosensitive Ion Channel Component 1	Protein Coding	39
GYS2	Glycogen Synthase 2	Protein Coding	45
MT-RNR2	Mitochondrially Encoded 16S RRNA	RNA Gene	20
ADAM22	ADAM Metallopeptidase Domain 22	Protein Coding	42
SMAD6	SMAD Family Member 6	Protein Coding	46
COL11A1	Collagen Type XI Alpha 1 Chain	Protein Coding	44
TPM2	Tropomyosin 2	Protein Coding	45
IRS2	Insulin Receptor Substrate 2	Protein Coding	45
KCNB1	Potassium Voltage-Gated Channel Subfamily B Membe	Protein Coding	48
DISC1	DISC1 Scaffold Protein	Protein Coding	44
DGCR8	DGCR8 Microprocessor Complex Subunit	Protein Coding	42
KIF5C	Kinesin Family Member 5C	Protein Coding	41
GRK2	G Protein-Coupled Receptor Kinase 2	Protein Coding	39
CAMP	Cathelicidin Antimicrobial Peptide	Protein Coding	43
KCNN3	Potassium Calcium-Activated Channel Subfamily N Me	Protein Coding	44
RPSA	Ribosomal Protein SA	Protein Coding	46
PADI4	Peptidyl Arginine Deiminase 4	Protein Coding	45
CHRM3	Cholinergic Receptor Muscarinic 3	Protein Coding	50
AD5	Alzheimer Disease 5	Genetic Locus	4
IKBKB	Inhibitor Of Nuclear Factor Kappa B Kinase Subunit B	Protein Coding	54
HAX1	HCLS1 Associated Protein X-1	Protein Coding	43
GLP1R	Glucagon Like Peptide 1 Receptor	Protein Coding	46

SCG2	Secretogranin II	Protein Coding	40
LRPAP1	LDL Receptor Related Protein Associated Protein 1	Protein Coding	44
APBB1	Amyloid Beta Precursor Protein Binding Family B Mem	Protein Coding	44
EEF1A2	Eukaryotic Translation Elongation Factor 1 Alpha 2	Protein Coding	47
DSG3	Desmoglein 3	Protein Coding	40
TUBB2A	Tubulin Beta 2A Class IIa	Protein Coding	46
IDH3A	Isocitrate Dehydrogenase (NAD(+)) 3 Catalytic Subuni	Protein Coding	45
MIR103A1	MicroRNA 103a-1	RNA Gene	18
SLPI	Secretory Leukocyte Peptidase Inhibitor	Protein Coding	41
MYOG	Myogenin	Protein Coding	41
FARP2	FERM, ARH/RhoGEF And Pleckstrin Domain Protein	Protein Coding	41
DCTN2	Dynactin Subunit 2	Protein Coding	42
TRIM21	Tripartite Motif Containing 21	Protein Coding	43
BBS1	Bardet-Biedl Syndrome 1	Protein Coding	39
ACADM	Acyl-CoA Dehydrogenase Medium Chain	Protein Coding	48
P3H3	Prolyl 3-Hydroxylase 3	Protein Coding	33
CD68	CD68 Molecule	Protein Coding	41
MYOC	Myocilin	Protein Coding	43
FLNB	Filamin B	Protein Coding	46
FBXW7	F-Box And WD Repeat Domain Containing 7	Protein Coding	45
MMP12	Matrix Metallopeptidase 12	Protein Coding	45
DEPDC5	DEP Domain Containing 5, GATOR1 Subcomplex Sub	Protein Coding	41
MIR27A	MicroRNA 27a	RNA Gene	22
EFHC1	EF-Hand Domain Containing 1	Protein Coding	41
ATP5F1C	ATP Synthase F1 Subunit Gamma	Protein Coding	33
DDIT4	DNA Damage Inducible Transcript 4	Protein Coding	45
ADRA1A	Adrenoceptor Alpha 1A	Protein Coding	48
PLK1	Polo Like Kinase 1	Protein Coding	50
CLTC	Clathrin Heavy Chain	Protein Coding	48
ATP5PF	ATP Synthase Peripheral Stalk Subunit F6	Protein Coding	32
MX1	MX Dynamin Like GTPase 1	Protein Coding	42
PREP	Prolyl Endopeptidase	Protein Coding	43
CXCL9	C-X-C Motif Chemokine Ligand 9	Protein Coding	40
LOC10866	TATA-Box Binding Protein Repeat Instability Region	Biological Region	2
MIR26B	MicroRNA 26b	RNA Gene	22
IFNGR2	Interferon Gamma Receptor 2	Protein Coding	44
RIPK1	Receptor Interacting Serine/Threonine Kinase 1	Protein Coding	50
THRB	Thyroid Hormone Receptor Beta	Protein Coding	51
RICTOR	RPTOR Independent Companion Of MTOR Complex 2	Protein Coding	44
AD6	Alzheimer Disease 6	Genetic Locus	3
PRKAR1B	Protein Kinase CAMP-Dependent Type I Regulatory Su	Protein Coding	48
BCL11A	BAF Chromatin Remodeling Complex Subunit BCL11A	Protein Coding	44
SNCA-AS	SNCA Antisense RNA 1	RNA Gene	12
TUBB4A	Tubulin Beta 4A Class IVa	Protein Coding	46
C1QA	Complement C1q A Chain	Protein Coding	46
PRDM8	PR/SET Domain 8	Protein Coding	37
CNTNAP1	Contactin Associated Protein 1	Protein Coding	44

TBX18	T-Box Transcription Factor 18	Protein Coding	42
PEX2	Peroxisomal Biogenesis Factor 2	Protein Coding	43
SEMA5A	Semaphorin 5A	Protein Coding	42
PRICKLE2	Prickle Planar Cell Polarity Protein 2	Protein Coding	39
ARG1	Arginase 1	Protein Coding	51
PCCB	Propionyl-CoA Carboxylase Subunit Beta	Protein Coding	47
FGG	Fibrinogen Gamma Chain	Protein Coding	50
GLRX	Glutaredoxin	Protein Coding	45
CPLX1	Complexin 1	Protein Coding	44
SLC2A9	Solute Carrier Family 2 Member 9	Protein Coding	47
VCAN	Versican	Protein Coding	48
CBL	Cbl Proto-Oncogene	Protein Coding	52
VDAC2	Voltage Dependent Anion Channel 2	Protein Coding	44
ADSL	Adenylosuccinate Lyase	Protein Coding	48
TJP1	Tight Junction Protein 1	Protein Coding	45
SRSF6	Serine And Arginine Rich Splicing Factor 6	Protein Coding	40
COL6A1	Collagen Type VI Alpha 1 Chain	Protein Coding	44
ALG13	ALG13 UDP-N-Acetylglucosaminyltransferase Subunit	Protein Coding	38
COX6A1	Cytochrome C Oxidase Subunit 6A1	Protein Coding	45
GNB4	G Protein Subunit Beta 4	Protein Coding	44
SLCO2A1	Solute Carrier Organic Anion Transporter Family Mem	Protein Coding	44
TNR	Tenascin R	Protein Coding	42
TACO1	Translational Activator Of Cytochrome C Oxidase I	Protein Coding	40
CTNND1	Catenin Delta 1	Protein Coding	47
MACROH	MacroH2A.1 Histone	Protein Coding	36
HMBS	Hydroxymethylbilane Synthase	Protein Coding	46
ITCH	Itchy E3 Ubiquitin Protein Ligase	Protein Coding	49
ATP13A1	ATPase 13A1	Protein Coding	40
EIF2S1	Eukaryotic Translation Initiation Factor 2 Subunit Alph	Protein Coding	46
ZEB1	Zinc Finger E-Box Binding Homeobox 1	Protein Coding	50
SULT1A3	Sulfotransferase Family 1A Member 3	Protein Coding	39
WNT3A	Wnt Family Member 3A	Protein Coding	49
TGIF1	TGFB Induced Factor Homeobox 1	Protein Coding	47
LGALS4	Galectin 4	Protein Coding	40
MRPS14	Mitochondrial Ribosomal Protein S14	Protein Coding	35
C12orf65	Chromosome 12 Open Reading Frame 65	Protein Coding	37
RLBP1	Retinaldehyde Binding Protein 1	Protein Coding	45
MIR181A1	MicroRNA 181a-1	RNA Gene	20
TRPS1	Transcriptional Repressor GATA Binding 1	Protein Coding	46
ENO3	Enolase 3	Protein Coding	50
AURKB	Aurora Kinase B	Protein Coding	50
H3C1	H3 Clustered Histone 1	Protein Coding	33
PTX3	Pentraxin 3	Protein Coding	43
GABARAI	GABA Type A Receptor Associated Protein Like 2	Protein Coding	45
CHUK	Component Of Inhibitor Of Nuclear Factor Kappa B Ki	Protein Coding	52
EXT1	Exostosin Glycosyltransferase 1	Protein Coding	48
MIR486-1	MicroRNA 486-1	RNA Gene	17

AD7	Alzheimer Disease 7	Genetic Locus	3
VIPR1	Vasoactive Intestinal Peptide Receptor 1	Protein Coding	47
GC	GC Vitamin D Binding Protein	Protein Coding	44
TBX6	T-Box Transcription Factor 6	Protein Coding	40
PRKAG3	Protein Kinase AMP-Activated Non-Catalytic Subunit (	Protein Coding	44
MIR141	MicroRNA 141	RNA Gene	22
PKLR	Pyruvate Kinase L/R	Protein Coding	49
VDAC3	Voltage Dependent Anion Channel 3	Protein Coding	43
TCERG1	Transcription Elongation Regulator 1	Protein Coding	40
PRDM16	PR/SET Domain 16	Protein Coding	46
NTN1	Netrin 1	Protein Coding	46
AD10	Alzheimer Disease-10	Genetic Locus	3
GRIN2C	Glutamate Ionotropic Receptor NMDA Type Subunit 2(	Protein Coding	45
SGK1	Serum/Glucocorticoid Regulated Kinase 1	Protein Coding	50
UCP2	Uncoupling Protein 2	Protein Coding	47
SIRT3	Sirtuin 3	Protein Coding	50
TPM3	Tropomyosin 3	Protein Coding	47
ERCC4	ERCC Excision Repair 4, Endonuclease Catalytic Subu	Protein Coding	47
LARS1	Leucyl-TRNA Synthetase 1	Protein Coding	37
DKK3	Dickkopf WNT Signaling Pathway Inhibitor 3	Protein Coding	42
NUP107	Nucleoporin 107	Protein Coding	43
SNAI2	Snail Family Transcriptional Repressor 2	Protein Coding	46
TTN-AS1	TTN Antisense RNA 1	RNA Gene	14
HSF1	Heat Shock Transcription Factor 1	Protein Coding	46
SLC9A7	Solute Carrier Family 9 Member A7	Protein Coding	39
ITGAL	Integrin Subunit Alpha L	Protein Coding	47
PRPF31	Pre-MRNA Processing Factor 31	Protein Coding	44
RYR3	Ryanodine Receptor 3	Protein Coding	43
HERC2	HECT And RLD Domain Containing E3 Ubiquitin Prot	Protein Coding	45
YME1L1	YME1 Like 1 ATPase	Protein Coding	43
MIR17HG	MiR-17-92a-1 Cluster Host Gene	RNA Gene	28
MSTN	Myostatin	Protein Coding	47
KIDINS22	Kinase D Interacting Substrate 220	Protein Coding	41
GRM2	Glutamate Metabotropic Receptor 2	Protein Coding	45
PTGDS	Prostaglandin D2 Synthase	Protein Coding	46
TSLP	Thymic Stromal Lymphopoietin	Protein Coding	40
CDC14A	Cell Division Cycle 14A	Protein Coding	44
IDO1	Indoleamine 2,3-Dioxygenase 1	Protein Coding	47
TGM1	Transglutaminase 1	Protein Coding	46
GDF5	Growth Differentiation Factor 5	Protein Coding	49
NEDD4	NEDD4 E3 Ubiquitin Protein Ligase	Protein Coding	47
XRCC3	X-Ray Repair Cross Complementing 3	Protein Coding	42
MEF2A	Myocyte Enhancer Factor 2A	Protein Coding	48
ALS7	Amyotrophic Lateral Sclerosis 7	Genetic Locus	4
FHIT	Fragile Histidine Triad Diadenosine Triphosphatase	Protein Coding	46
CCNB1	Cyclin B1	Protein Coding	48
KRT8	Keratin 8	Protein Coding	48

ALS3	Amyotrophic Lateral Sclerosis 3 (Autosomal Dominant)	Genetic Locus	4
CD163	CD163 Molecule	Protein Coding	44
AD11	Alzheimer Disease-11	Genetic Locus	2
AD8	Alzheimer Disease 8	Genetic Locus	2
MRE11	MRE11 Homolog, Double Strand Break Repair Nucleas	Protein Coding	43
CHRNA3	Cholinergic Receptor Nicotinic Alpha 3 Subunit	Protein Coding	45
FRMD4A	FERM Domain Containing 4A	Protein Coding	39
OR56A5	Olfactory Receptor Family 56 Subfamily A Member 5	Protein Coding	25
PRDM2	PR/SET Domain 2	Protein Coding	41
ACADVL	Acyl-CoA Dehydrogenase Very Long Chain	Protein Coding	47
TTC19	Tetratricopeptide Repeat Domain 19	Protein Coding	40
AD12	Alzheimer Disease 12	Genetic Locus	2
AD13	Alzheimer Disease-13	Genetic Locus	2
AD14	Alzheimer Disease 14	Genetic Locus	2
AD15	Alzheimer Disease-15	Genetic Locus	2
PLOD1	Procollagen-Lysine,2-Oxoglutarate 5-Dioxygenase 1	Protein Coding	42
UROD	Uroporphyrinogen Decarboxylase	Protein Coding	47
FKBP1A	FKBP Prolyl Isomerase 1A	Protein Coding	48
NOG	Noggin	Protein Coding	45
PSMA6	Proteasome 20S Subunit Alpha 6	Protein Coding	47
TPT1	Tumor Protein, Translationally-Controlled 1	Protein Coding	48
DBN1	Drebrin 1	Protein Coding	41
ACAD9	Acyl-CoA Dehydrogenase Family Member 9	Protein Coding	44
PSMB4	Proteasome 20S Subunit Beta 4	Protein Coding	45
DSTYK	Dual Serine/Threonine And Tyrosine Protein Kinase	Protein Coding	43
AD17	Alzheimer Disease 17	Genetic Locus	2
COL13A1	Collagen Type XIII Alpha 1 Chain	Protein Coding	42
CACNA1I	Calcium Voltage-Gated Channel Subunit Alpha1 D	Protein Coding	48
PHB	Prohibitin	Protein Coding	49
CD34	CD34 Molecule	Protein Coding	45
FBXO48	F-Box Protein 48	Protein Coding	29
LEPQTL1	Leptin, Serum Levels Of	Genetic Locus	4
FOLH1	Folate Hydrolase 1	Protein Coding	48
CD59	CD59 Molecule (CD59 Blood Group)	Protein Coding	48
NR1H3	Nuclear Receptor Subfamily 1 Group H Member 3	Protein Coding	48
PPP1R1B	Protein Phosphatase 1 Regulatory Inhibitor Subunit 1B	Protein Coding	44
STX6	Syntaxin 6	Protein Coding	42
AD16	Alzheimer Disease 16	Genetic Locus	2
EIF2B5	Eukaryotic Translation Initiation Factor 2B Subunit Eps	Protein Coding	44
UBR5	Ubiquitin Protein Ligase E3 Component N-Recognin 5	Protein Coding	43
GRID2	Glutamate Ionotropic Receptor Delta Type Subunit 2	Protein Coding	47
HAP1	Huntingtin Associated Protein 1	Protein Coding	40
MTO1	Mitochondrial TRNA Translation Optimization 1	Protein Coding	43
RAB27A	RAB27A, Member RAS Oncogene Family	Protein Coding	50
ALDOA	Aldolase, Fructose-Bisphosphate A	Protein Coding	50
ADM	Adrenomedullin	Protein Coding	45
PRKCZ	Protein Kinase C Zeta	Protein Coding	50

TXNRD2	Thioredoxin Reductase 2	Protein Coding	48
GRB2	Growth Factor Receptor Bound Protein 2	Protein Coding	51
BCKDK	Branched Chain Keto Acid Dehydrogenase Kinase	Protein Coding	47
UBA1	Ubiquitin Like Modifier Activating Enzyme 1	Protein Coding	48
GNA14	G Protein Subunit Alpha 14	Protein Coding	44
ALDH18A	Aldehyde Dehydrogenase 18 Family Member A1	Protein Coding	45
GJC3	Gap Junction Protein Gamma 3	Protein Coding	39
PLCG2	Phospholipase C Gamma 2	Protein Coding	52
INS-IGF2	INS-IGF2 Readthrough	Protein Coding	28
HADH	Hydroxyacyl-CoA Dehydrogenase	Protein Coding	48
HEPH	Hephaestin	Protein Coding	40
PTBP1	Polypyrimidine Tract Binding Protein 1	Protein Coding	43
LYN	LYN Proto-Oncogene, Src Family Tyrosine Kinase	Protein Coding	51
CFL1	Cofilin 1	Protein Coding	47
GRK1	G Protein-Coupled Receptor Kinase 1	Protein Coding	44
SSTR2	Somatostatin Receptor 2	Protein Coding	49
LIFR	LIF Receptor Subunit Alpha	Protein Coding	48
FH	Fumarate Hydratase	Protein Coding	47
GABRB1	Gamma-Aminobutyric Acid Type A Receptor Subunit I	Protein Coding	46
TYK2	Tyrosine Kinase 2	Protein Coding	54
MIR10A	MicroRNA 10a	RNA Gene	21
CD33	CD33 Molecule	Protein Coding	45
VPS13D	Vacuolar Protein Sorting 13 Homolog D	Protein Coding	35
PRPF8	Pre-MRNA Processing Factor 8	Protein Coding	42
PDE4A	Phosphodiesterase 4A	Protein Coding	45
GMPR	Guanosine Monophosphate Reductase	Protein Coding	44
TNFSF12	TNF Superfamily Member 12	Protein Coding	42
CAMK2G	Calcium/Calmodulin Dependent Protein Kinase II Gam	Protein Coding	48
EWSR1	EWS RNA Binding Protein 1	Protein Coding	44
PET117	PET117 Cytochrome C Oxidase Chaperone	Protein Coding	30
IL11	Interleukin 11	Protein Coding	43
LEF1	Lymphoid Enhancer Binding Factor 1	Protein Coding	48
BRAT1	BRCA1 Associated ATM Activator 1	Protein Coding	39
CHRM2	Cholinergic Receptor Muscarinic 2	Protein Coding	48
IRF8	Interferon Regulatory Factor 8	Protein Coding	47
ORC6	Origin Recognition Complex Subunit 6	Protein Coding	41
KNL1	Kinetochore Scaffold 1	Protein Coding	33
FZD4	Frizzled Class Receptor 4	Protein Coding	52
ECHS1	Enoyl-CoA Hydratase, Short Chain 1	Protein Coding	48
ADAMTSI	ADAMTS Like 1	Protein Coding	42
SLC2A2	Solute Carrier Family 2 Member 2	Protein Coding	50
NUP133	Nucleoporin 133	Protein Coding	41
GADD45A	Growth Arrest And DNA Damage Inducible Alpha	Protein Coding	46
LMX1A	LIM Homeobox Transcription Factor 1 Alpha	Protein Coding	41
RCAN1	Regulator Of Calcineurin 1	Protein Coding	44
HNRNPU	Heterogeneous Nuclear Ribonucleoprotein U	Protein Coding	44
FLCN	Folliculin	Protein Coding	42

CTTN	Cortactin	Protein Coding	44
DZIP1L	DAZ Interacting Zinc Finger Protein 1 Like	Protein Coding	37
ELAC2	ElaC Ribonuclease Z 2	Protein Coding	42
LRP12	LDL Receptor Related Protein 12	Protein Coding	40
SEPTIN9	Septin 9	Protein Coding	36
AGK	Acylglycerol Kinase	Protein Coding	43
KRT17	Keratin 17	Protein Coding	47
REG1A	Regenerating Family Member 1 Alpha	Protein Coding	43
HUWE1	HECT, UBA And WWE Domain Containing E3 Ubiqui	Protein Coding	45
WRAP53	WD Repeat Containing Antisense To TP53	Protein Coding	42
IL6ST	Interleukin 6 Signal Transducer	Protein Coding	47
ATP2A1	ATPase Sarcoplasmic/Endoplasmic Reticulum Ca2+ Tı	Protein Coding	49
IFNAR1	Interferon Alpha And Beta Receptor Subunit 1	Protein Coding	47
MIR574	MicroRNA 574	RNA Gene	19
TUBB3	Tubulin Beta 3 Class III	Protein Coding	51
HSD17B4	Hydroxysteroid 17-Beta Dehydrogenase 4	Protein Coding	48
MIR146B	MicroRNA 146b	RNA Gene	20
TNK2	Tyrosine Kinase Non Receptor 2	Protein Coding	48
MIR29C	MicroRNA 29c	RNA Gene	18
EPHX2	Epoxide Hydrolase 2	Protein Coding	49
SYNCRIP	Synaptotagmin Binding Cytoplasmic RNA Interacting F	Protein Coding	40
CA8	Carbonic Anhydrase 8	Protein Coding	47
CXCR2	C-X-C Motif Chemokine Receptor 2	Protein Coding	50
RASSF1	Ras Association Domain Family Member 1	Protein Coding	45
RAB38	RAB38, Member RAS Oncogene Family	Protein Coding	41
MIR127	MicroRNA 127	RNA Gene	21
FANCA	FA Complementation Group A	Protein Coding	50
PUF60	Poly(U) Binding Splicing Factor 60	Protein Coding	42
PDE5A	Phosphodiesterase 5A	Protein Coding	46
SLC1A1	Solute Carrier Family 1 Member 1	Protein Coding	49
MIR193A	MicroRNA 193a	RNA Gene	18
PCBP2	Poly(RC) Binding Protein 2	Protein Coding	41
DUSP1	Dual Specificity Phosphatase 1	Protein Coding	48
CNTN1	Contactin 1	Protein Coding	45
MYH14	Myosin Heavy Chain 14	Protein Coding	47
OLIG2	Oligodendrocyte Transcription Factor 2	Protein Coding	41
UTRN	Utrophin	Protein Coding	41
HHEX	Hematopoietically Expressed Homeobox	Protein Coding	44
DHPS	Deoxyhypusine Synthase	Protein Coding	43
MIR205	MicroRNA 205	RNA Gene	20
AGFG1	ArfGAP With FG Repeats 1	Protein Coding	40
LYRM4	LYR Motif Containing 4	Protein Coding	39
FBXL18	F-Box And Leucine Rich Repeat Protein 18	Protein Coding	36
ERN1	Endoplasmic Reticulum To Nucleus Signaling 1	Protein Coding	47
CHRM1	Cholinergic Receptor Muscarinic 1	Protein Coding	47
PRKAG1	Protein Kinase AMP-Activated Non-Catalytic Subunit (	Protein Coding	49
GRP	Gastrin Releasing Peptide	Protein Coding	42

KIF11	Kinesin Family Member 11	Protein Coding	49
CCDC40	Coiled-Coil Domain Containing 40	Protein Coding	38
MS4A6A	Membrane Spanning 4-Domains A6A	Protein Coding	39
CCN6	Cellular Communication Network Factor 6	Protein Coding	34
EPAS1	Endothelial PAS Domain Protein 1	Protein Coding	50
SOAT1	Sterol O-Acyltransferase 1	Protein Coding	45
ZMPSTE2	Zinc Metallopeptidase STE24	Protein Coding	44
PCCA	Propionyl-CoA Carboxylase Subunit Alpha	Protein Coding	47
RTN4R	Reticulon 4 Receptor	Protein Coding	44
IGLON5	IgLON Family Member 5	Protein Coding	33
HDAC2	Histone Deacetylase 2	Protein Coding	53
TRMT5	TRNA Methyltransferase 5	Protein Coding	40
TIAM1	TIAM Rac1 Associated GEF 1	Protein Coding	46
PCNT	Pericentrin	Protein Coding	42
RPS19	Ribosomal Protein S19	Protein Coding	49
KRIT1	KRIT1 Ankyrin Repeat Containing	Protein Coding	43
GPC1	Glypican 1	Protein Coding	45
MT-TN	Mitochondrially Encoded TRNA-Asn (AAU/C)	RNA Gene	13
RPS6KA3	Ribosomal Protein S6 Kinase A3	Protein Coding	53
ALOX5AP	Arachidonate 5-Lipoxygenase Activating Protein	Protein Coding	45
DDRGK1	DDRGK Domain Containing 1	Protein Coding	38
LACTB	Lactamase Beta	Protein Coding	37
LOXL1	Lysyl Oxidase Like 1	Protein Coding	44
LIMK1	LIM Domain Kinase 1	Protein Coding	51
USH1C	USH1 Protein Network Component Harmonin	Protein Coding	43
TKT	Transketolase	Protein Coding	49
RB1CC1	RB1 Inducible Coiled-Coil 1	Protein Coding	44
CSNK2A1	Casein Kinase 2 Alpha 1	Protein Coding	52
HTR6	5-Hydroxytryptamine Receptor 6	Protein Coding	45
UQCRC1	Ubiquinol-Cytochrome C Reductase Core Protein 1	Protein Coding	43
GRIK2	Glutamate Ionotropic Receptor Kainate Type Subunit 2	Protein Coding	48
CNNM2	Cyclin And CBS Domain Divalent Metal Cation Transp	Protein Coding	43
PPIF	Peptidylprolyl Isomerase F	Protein Coding	44
ITGA6	Integrin Subunit Alpha 6	Protein Coding	51
HLA-DQA	Major Histocompatibility Complex, Class II, DQ Alpha	Protein Coding	37
U2AF2	U2 Small Nuclear RNA Auxiliary Factor 2	Protein Coding	38
ASS1	Argininosuccinate Synthase 1	Protein Coding	50
LOC10946	Huntingtin Repeat Instability Region	Biological Region	2
SGCB	Sarcoglycan Beta	Protein Coding	41
SRSF7	Serine And Arginine Rich Splicing Factor 7	Protein Coding	41
MIR106A	MicroRNA 106a	RNA Gene	18
GMPPB	GDP-Mannose Pyrophosphorylase B	Protein Coding	44
CASK	Calcium/Calmodulin Dependent Serine Protein Kinase	Protein Coding	50
AMPH	Amphiphysin	Protein Coding	44
NODAL	Nodal Growth Differentiation Factor	Protein Coding	43
PRSS3	Serine Protease 3	Protein Coding	43
SFRP1	Secreted Frizzled Related Protein 1	Protein Coding	44

MPP2	Membrane Palmitoylated Protein 2	Protein Coding	40
SKIV2L	Ski2 Like RNA Helicase	Protein Coding	44
SNAI1	Snail Family Transcriptional Repressor 1	Protein Coding	45
PDK3	Pyruvate Dehydrogenase Kinase 3	Protein Coding	48
SLC25A1	Solute Carrier Family 25 Member 1	Protein Coding	47
UBE2V1	Ubiquitin Conjugating Enzyme E2 V1	Protein Coding	43
ACVR1	Activin A Receptor Type 1	Protein Coding	51
SLC2A4	Solute Carrier Family 2 Member 4	Protein Coding	47
PLEKHM1	Pleckstrin Homology And RUN Domain Containing M1	Protein Coding	42
HMGCL	3-Hydroxy-3-Methylglutaryl-CoA Lyase	Protein Coding	48
NRP1	Neuropilin 1	Protein Coding	48
TRAF3IP2	TRAF3 Interacting Protein 2	Protein Coding	45
DHCR7	7-Dehydrocholesterol Reductase	Protein Coding	47
RPL5	Ribosomal Protein L5	Protein Coding	48
GABARAI	GABA Type A Receptor Associated Protein Like 1	Protein Coding	44
PEBP1	Phosphatidylethanolamine Binding Protein 1	Protein Coding	46
AUP1	AUP1 Lipid Droplet Regulating VLDL Assembly Facto	Protein Coding	38
POSTN	Periostin	Protein Coding	45
POLR3A	RNA Polymerase III Subunit A	Protein Coding	45
ATP5PB	ATP Synthase Peripheral Stalk-Membrane Subunit B	Protein Coding	31
FOXM1	Forkhead Box M1	Protein Coding	45
HSPA1B	Heat Shock Protein Family A (Hsp70) Member 1B	Protein Coding	41
COL6A3	Collagen Type VI Alpha 3 Chain	Protein Coding	45
WNT4	Wnt Family Member 4	Protein Coding	48
TMEM175	Transmembrane Protein 175	Protein Coding	33
CCNE2	Cyclin E2	Protein Coding	43
FUCA1	Alpha-L-Fucosidase 1	Protein Coding	47
DCX	Doublecortin	Protein Coding	46
RBCK1	RANBP2-Type And C3HC4-Type Zinc Finger Contain	Protein Coding	43
NDUFA8	NADH:Ubiquinone Oxidoreductase Subunit A8	Protein Coding	42
RANBP2	RAN Binding Protein 2	Protein Coding	46
BMI1	BMI1 Proto-Oncogene, Polycomb Ring Finger	Protein Coding	45
MAPK12	Mitogen-Activated Protein Kinase 12	Protein Coding	49
TBCD	Tubulin Folding Cofactor D	Protein Coding	41
DNAL4	Dynein Axonemal Light Chain 4	Protein Coding	43
OPA3	Outer Mitochondrial Membrane Lipid Metabolism Regu	Protein Coding	40
F2R	Coagulation Factor II Thrombin Receptor	Protein Coding	48
SV2A	Synaptic Vesicle Glycoprotein 2A	Protein Coding	44
CACNA1E	Calcium Voltage-Gated Channel Subunit Alpha1 E	Protein Coding	46
MIR298	MicroRNA 298	RNA Gene	17
CCT3	Chaperonin Containing TCP1 Subunit 3	Protein Coding	43
CYP7B1	Cytochrome P450 Family 7 Subfamily B Member 1	Protein Coding	47
ATP5F1E	ATP Synthase F1 Subunit Epsilon	Protein Coding	32
SHC1	SHC Adaptor Protein 1	Protein Coding	47
MAP3K1	Mitogen-Activated Protein Kinase Kinase Kinase 1	Protein Coding	51
P4HB	Prolyl 4-Hydroxylase Subunit Beta	Protein Coding	51
BSND	Barttin CLCNK Type Accessory Subunit Beta	Protein Coding	41

AMBP	Alpha-1-Microglobulin/Bikunin Precursor	Protein Coding	43
TRPM4	Transient Receptor Potential Cation Channel Subfamily	Protein Coding	47
CHKA	Choline Kinase Alpha	Protein Coding	43
USF1	Upstream Transcription Factor 1	Protein Coding	44
FGGY	FGGY Carbohydrate Kinase Domain Containing	Protein Coding	38
IMPDH1	Inosine Monophosphate Dehydrogenase 1	Protein Coding	50
CFAP43	Cilia And Flagella Associated Protein 43	Protein Coding	31
POR	Cytochrome P450 Oxidoreductase	Protein Coding	50
HK2	Hexokinase 2	Protein Coding	49
KLHL7	Kelch Like Family Member 7	Protein Coding	41
MIR363	MicroRNA 363	RNA Gene	16
YWHAG	Tyrosine 3-Monooxygenase/Tryptophan 5-Monooxyger	Protein Coding	51
ABCD3	ATP Binding Cassette Subfamily D Member 3	Protein Coding	45
RPL26	Ribosomal Protein L26	Protein Coding	43
TACR3	Tachykinin Receptor 3	Protein Coding	48
MCL1	MCL1 Apoptosis Regulator, BCL2 Family Member	Protein Coding	49
PLTP	Phospholipid Transfer Protein	Protein Coding	44
MIR100	MicroRNA 100	RNA Gene	21
PAX7	Paired Box 7	Protein Coding	45
PIGO	Phosphatidylinositol Glycan Anchor Biosynthesis Class	Protein Coding	43
USP8	Ubiquitin Specific Peptidase 8	Protein Coding	49
ROCK1	Rho Associated Coiled-Coil Containing Protein Kinase	Protein Coding	51
CARS1	Cysteinyl-TRNA Synthetase 1	Protein Coding	33
EFTUD2	Elongation Factor Tu GTP Binding Domain Containing	Protein Coding	43
IRAK1	Interleukin 1 Receptor Associated Kinase 1	Protein Coding	51
PDPK1	3-Phosphoinositide Dependent Protein Kinase 1	Protein Coding	50
CCS	Copper Chaperone For Superoxide Dismutase	Protein Coding	44
USP9X	Ubiquitin Specific Peptidase 9 X-Linked	Protein Coding	48
RARS1	Arginyl-TRNA Synthetase 1	Protein Coding	36
PBX1	PBX Homeobox 1	Protein Coding	50
ADH5	Alcohol Dehydrogenase 5 (Class III), Chi Polypeptide	Protein Coding	47
HMGA2	High Mobility Group AT-Hook 2	Protein Coding	46
PAX4	Paired Box 4	Protein Coding	44
MT-TA	Mitochondrially Encoded TRNA-Ala (GCN)	RNA Gene	12
MTIF3	Mitochondrial Translational Initiation Factor 3	Protein Coding	36
RAB3A	RAB3A, Member RAS Oncogene Family	Protein Coding	45
ASCL1	Achaete-Scute Family BHLH Transcription Factor 1	Protein Coding	44
BIRC3	Baculoviral IAP Repeat Containing 3	Protein Coding	48
PPP2R1A	Protein Phosphatase 2 Scaffold Subunit Aalpha	Protein Coding	48
TNC	Tenascin C	Protein Coding	49
UQCRC2	Ubiquinol-Cytochrome C Reductase Core Protein 2	Protein Coding	48
PHGDH	Phosphoglycerate Dehydrogenase	Protein Coding	50
ANXA1	Annexin A1	Protein Coding	50
HTR1B	5-Hydroxytryptamine Receptor 1B	Protein Coding	45
COL5A2	Collagen Type V Alpha 2 Chain	Protein Coding	42
IGHM	Immunoglobulin Heavy Constant Mu	Protein Coding	32
MBTPS2	Membrane Bound Transcription Factor Peptidase, Site :	Protein Coding	44

FASTKD2	FAST Kinase Domains 2	Protein Coding	40
SRSF9	Serine And Arginine Rich Splicing Factor 9	Protein Coding	40
NONO	Non-POU Domain Containing Octamer Binding	Protein Coding	45
CHRNA1	Cholinergic Receptor Nicotinic Alpha 1 Subunit	Protein Coding	47
IRF3	Interferon Regulatory Factor 3	Protein Coding	48
PDGFA	Platelet Derived Growth Factor Subunit A	Protein Coding	45
NOD1	Nucleotide Binding Oligomerization Domain Containing	Protein Coding	46
WARS1	Tryptophanyl-TRNA Synthetase 1	Protein Coding	39
PIP	Prolactin Induced Protein	Protein Coding	38
HBG1	Hemoglobin Subunit Gamma 1	Protein Coding	43
AATF	Apoptosis Antagonizing Transcription Factor	Protein Coding	41
MAT1A	Methionine Adenosyltransferase 1A	Protein Coding	48
HIC1	HIC ZBTB Transcriptional Repressor 1	Protein Coding	41
MIR15B	MicroRNA 15b	RNA Gene	19
KCNMA1	Potassium Calcium-Activated Channel Subfamily M Al	Protein Coding	51
FANCG	FA Complementation Group G	Protein Coding	44
PPP3R1	Protein Phosphatase 3 Regulatory Subunit B, Alpha	Protein Coding	47
PEMT	Phosphatidylethanolamine N-Methyltransferase	Protein Coding	41
MCM7	Minichromosome Maintenance Complex Component 7	Protein Coding	46
DMBT1	Deleted In Malignant Brain Tumors 1	Protein Coding	41
NGLY1	N-Glycanase 1	Protein Coding	44
HINT1	Histidine Triad Nucleotide Binding Protein 1	Protein Coding	47
HAPLN1	Hyaluronan And Proteoglycan Link Protein 1	Protein Coding	43
PNP	Purine Nucleoside Phosphorylase	Protein Coding	49
MTNR1A	Melatonin Receptor 1A	Protein Coding	44
CXCL1	C-X-C Motif Chemokine Ligand 1	Protein Coding	44
PSMA4	Proteasome 20S Subunit Alpha 4	Protein Coding	45
LRRC37A	Leucine Rich Repeat Containing 37A	Protein Coding	32
SRM	Spermidine Synthase	Protein Coding	42
GPC4	Glypican 4	Protein Coding	47
NDE1	NudE Neurodevelopment Protein 1	Protein Coding	43
MIR185	MicroRNA 185	RNA Gene	22
AGA	Aspartylglucosaminidase	Protein Coding	44
FANCD2	FA Complementation Group D2	Protein Coding	48
ROCK2	Rho Associated Coiled-Coil Containing Protein Kinase 2	Protein Coding	49
STMN1	Stathmin 1	Protein Coding	45
SPRY2	Sprouty RTK Signaling Antagonist 2	Protein Coding	47
MLX	MAX Dimerization Protein MLX	Protein Coding	44
TMED10	Transmembrane P24 Trafficking Protein 10	Protein Coding	45
ITIH2	Inter-Alpha-Trypsin Inhibitor Heavy Chain 2	Protein Coding	40
PRKDC	Protein Kinase, DNA-Activated, Catalytic Subunit	Protein Coding	51
WDR45B	WD Repeat Domain 45B	Protein Coding	37
LRRC37A	Leucine Rich Repeat Containing 37 Member A3	Protein Coding	31
MIRLET7.	MicroRNA Let-7a-1	RNA Gene	22
ACOX1	Acyl-CoA Oxidase 1	Protein Coding	46
COLEC12	Collectin Subfamily Member 12	Protein Coding	40
VGF	VGF Nerve Growth Factor Inducible	Protein Coding	40

SEPTIN7	Septin 7	Protein Coding	32
IGFBP2	Insulin Like Growth Factor Binding Protein 2	Protein Coding	45
TRAF3IP1	TRAF3 Interacting Protein 1	Protein Coding	40
LTBP2	Latent Transforming Growth Factor Beta Binding Prote	Protein Coding	44
POMGNT:	Protein O-Linked Mannose N-Acetylglucosaminyltransf	Protein Coding	37
RPTOR	Regulatory Associated Protein Of MTOR Complex 1	Protein Coding	45
ATR	ATR Serine/Threonine Kinase	Protein Coding	53
PER1	Period Circadian Regulator 1	Protein Coding	43
MIR26A1	MicroRNA 26a-1	RNA Gene	21
NDUFB7	NADH:Ubiquinone Oxidoreductase Subunit B7	Protein Coding	40
FLOT1	Flotillin 1	Protein Coding	43
LCOR	Ligand Dependent Nuclear Receptor Corepressor	Protein Coding	39
ADH1A	Alcohol Dehydrogenase 1A (Class I), Alpha Polypeptid	Protein Coding	43
RBPJ	Recombination Signal Binding Protein For Immunoglob	Protein Coding	48
LOC11125	NOS1 1f And 1g Alternate Promoter Region	Biological Region	2
TIMM8A	Translocase Of Inner Mitochondrial Membrane 8A	Protein Coding	43
KCNJ10	Potassium Inwardly Rectifying Channel Subfamily J M	Protein Coding	46
EPHB4	EPH Receptor B4	Protein Coding	52
RHEB	Ras Homolog, MTORC1 Binding	Protein Coding	49
NIPBL	NIPBL Cohesin Loading Factor	Protein Coding	41
MIR19B1	MicroRNA 19b-1	RNA Gene	18
PGM1	Phosphoglucomutase 1	Protein Coding	49
RAP2B	RAP2B, Member Of RAS Oncogene Family	Protein Coding	40
GALK1	Galactokinase 1	Protein Coding	49
IL1R2	Interleukin 1 Receptor Type 2	Protein Coding	47
MIR196A2	MicroRNA 196a-2	RNA Gene	22
GPX2	Glutathione Peroxidase 2	Protein Coding	45
CALHM1	Calcium Homeostasis Modulator 1	Protein Coding	35
SRCAP	Snf2 Related CREBBP Activator Protein	Protein Coding	41
POLR2F	RNA Polymerase II Subunit F	Protein Coding	41
HSPA1L	Heat Shock Protein Family A (Hsp70) Member 1 Like	Protein Coding	44
SLC25A41	Solute Carrier Family 25 Member 41	Protein Coding	33
CLDN16	Claudin 16	Protein Coding	41
ACSS2	Acyl-CoA Synthetase Short Chain Family Member 2	Protein Coding	45
DAOA	D-Amino Acid Oxidase Activator	Protein Coding	31
ARR3	Arrestin 3	Protein Coding	43
CTNNA1	Catenin Alpha 1	Protein Coding	49
MBD5	Methyl-CpG Binding Domain Protein 5	Protein Coding	39
NDUFA4	NDUFA4 Mitochondrial Complex Associated	Protein Coding	43
MIR192	MicroRNA 192	RNA Gene	22
PDP1	Pyruvate Dehyrogenase Phosphatase Catalytic Subunit	Protein Coding	47
CD1A	CD1a Molecule	Protein Coding	43
HBEGF	Heparin Binding EGF Like Growth Factor	Protein Coding	44
LRP4	LDL Receptor Related Protein 4	Protein Coding	44
CDR2	Cerebellar Degeneration Related Protein 2	Protein Coding	39
BBS9	Bardet-Biedl Syndrome 9	Protein Coding	41
COQ8B	Coenzyme Q8B	Protein Coding	33

TMEM237	Transmembrane Protein 237	Protein Coding	38
SHOC2	SHOC2 Leucine Rich Repeat Scaffold Protein	Protein Coding	43
BACE2	Beta-Secretase 2	Protein Coding	41
TFG	Trafficking From ER To Golgi Regulator	Protein Coding	46
SCARB1	Scavenger Receptor Class B Member 1	Protein Coding	47
NDUFB6	NADH:Ubiquinone Oxidoreductase Subunit B6	Protein Coding	41
AHSA1	Activator Of HSP90 ATPase Activity 1	Protein Coding	40
ITGB1	Integrin Subunit Beta 1	Protein Coding	51
WNT7A	Wnt Family Member 7A	Protein Coding	51
ETS1	ETS Proto-Oncogene 1, Transcription Factor	Protein Coding	51
INPP5K	Inositol Polyphosphate-5-Phosphatase K	Protein Coding	44
CLOCK	Clock Circadian Regulator	Protein Coding	45
DNAH1	Dynein Axonemal Heavy Chain 1	Protein Coding	38
MRPS22	Mitochondrial Ribosomal Protein S22	Protein Coding	43
SMCR8	SMCR8-C9orf72 Complex Subunit	Protein Coding	33
VPS16	VPS16 Core Subunit Of CORVET And HOPS Comple	Protein Coding	35
EIF2B2	Eukaryotic Translation Initiation Factor 2B Subunit Be	Protein Coding	45
MAN2B1	Mannosidase Alpha Class 2B Member 1	Protein Coding	45
SNRNP200	Small Nuclear Ribonucleoprotein U5 Subunit 200	Protein Coding	43
NOX1	NADPH Oxidase 1	Protein Coding	43
AICDA	Activation Induced Cytidine Deaminase	Protein Coding	47
CHRNA5	Cholinergic Receptor Nicotinic Alpha 5 Subunit	Protein Coding	45
CEL	Carboxyl Ester Lipase	Protein Coding	47
DLAT	Dihydrolipoamide S-Acetyltransferase	Protein Coding	47
PSMA7	Proteasome 20S Subunit Alpha 7	Protein Coding	46
CELF1	CUGBP Elav-Like Family Member 1	Protein Coding	39
GLRX5	Glutaredoxin 5	Protein Coding	41
BMPR1B	Bone Morphogenetic Protein Receptor Type 1B	Protein Coding	52
NDUFS5	NADH:Ubiquinone Oxidoreductase Subunit S5	Protein Coding	42
NT5E	5'-Nucleotidase Ecto	Protein Coding	52
OPRL1	Opioid Related Nociceptin Receptor 1	Protein Coding	46
CA10	Carbonic Anhydrase 10	Protein Coding	40
MEF2D	Myocyte Enhancer Factor 2D	Protein Coding	45
H4-16	H4 Histone 16	Protein Coding	35
TUBB1	Tubulin Beta 1 Class VI	Protein Coding	48
COX6C	Cytochrome C Oxidase Subunit 6C	Protein Coding	40
NDRG2	NDRG Family Member 2	Protein Coding	40
LY6E	Lymphocyte Antigen 6 Family Member E	Protein Coding	40
CD24	CD24 Molecule	Protein Coding	32
ITGAX	Integrin Subunit Alpha X	Protein Coding	45
VAPA	VAMP Associated Protein A	Protein Coding	44
MIR375	MicroRNA 375	RNA Gene	20
FANCC	FA Complementation Group C	Protein Coding	48
HPSE	Heparanase	Protein Coding	46
PSMC5	Proteasome 26S Subunit, ATPase 5	Protein Coding	43
FGF9	Fibroblast Growth Factor 9	Protein Coding	45
PSMA1	Proteasome 20S Subunit Alpha 1	Protein Coding	45

PSMD3	Proteasome 26S Subunit, Non-ATPase 3	Protein Coding	43
CX3CL1	C-X3-C Motif Chemokine Ligand 1	Protein Coding	44
AKR1A1	Aldo-Keto Reductase Family 1 Member A1	Protein Coding	45
TMOD2	Tropomodulin 2	Protein Coding	39
SKAP2	Src Kinase Associated Phosphoprotein 2	Protein Coding	41
TREM1	Triggering Receptor Expressed On Myeloid Cells 1	Protein Coding	43
GPHA2	Glycoprotein Hormone Subunit Alpha 2	Protein Coding	36
CLPX	Caseinolytic Mitochondrial Matrix Peptidase Chaperone	Protein Coding	40
GLRB	Glycine Receptor Beta	Protein Coding	50
PQBP1	Polyglutamine Binding Protein 1	Protein Coding	41
VPS41	VPS41 Subunit Of HOPS Complex	Protein Coding	39
FGR	FGR Proto-Oncogene, Src Family Tyrosine Kinase	Protein Coding	50
APBA1	Amyloid Beta Precursor Protein Binding Family A Men	Protein Coding	39
PIK3R2	Phosphoinositide-3-Kinase Regulatory Subunit 2	Protein Coding	51
LRPPRC	Leucine Rich Pentatricopeptide Repeat Containing	Protein Coding	43
PRKD1	Protein Kinase D1	Protein Coding	51
IL1RL1	Interleukin 1 Receptor Like 1	Protein Coding	43
IL18RAP	Interleukin 18 Receptor Accessory Protein	Protein Coding	40
CAVIN1	Caveolae Associated Protein 1	Protein Coding	33
THBS4	Thrombospondin 4	Protein Coding	44
OAT	Ornithine Aminotransferase	Protein Coding	48
CYP24A1	Cytochrome P450 Family 24 Subfamily A Member 1	Protein Coding	48
LYST	Lysosomal Trafficking Regulator	Protein Coding	39
SIRT2	Sirtuin 2	Protein Coding	50
CDK2	Cyclin Dependent Kinase 2	Protein Coding	54
SLC39A1	Solute Carrier Family 39 Member 1	Protein Coding	39
PADI2	Peptidyl Arginine Deiminase 2	Protein Coding	43
MARK3	Microtubule Affinity Regulating Kinase 3	Protein Coding	50
CCKBR	Cholecystokinin B Receptor	Protein Coding	46
NAPB	NSF Attachment Protein Beta	Protein Coding	39
DVL3	Dishevelled Segment Polarity Protein 3	Protein Coding	47
MAP3K7	Mitogen-Activated Protein Kinase Kinase Kinase 7	Protein Coding	52
C1QB	Complement C1q B Chain	Protein Coding	45
PSMD2	Proteasome 26S Subunit, Non-ATPase 2	Protein Coding	44
GNA11	G Protein Subunit Alpha 11	Protein Coding	48
FASN	Fatty Acid Synthase	Protein Coding	51
RNASEH2	Ribonuclease H2 Subunit B	Protein Coding	37
TBL1X	Transducin Beta Like 1 X-Linked	Protein Coding	37
MIR148A	MicroRNA 148a	RNA Gene	19
BUB1	BUB1 Mitotic Checkpoint Serine/Threonine Kinase	Protein Coding	51
SON	SON DNA Binding Protein	Protein Coding	40
CYP51A1	Cytochrome P450 Family 51 Subfamily A Member 1	Protein Coding	45
MIRLET7(	MicroRNA Let-7g	RNA Gene	20
KLF6	Kruppel Like Factor 6	Protein Coding	45
FAM13A	Family With Sequence Similarity 13 Member A	Protein Coding	38
MYBPC1	Myosin Binding Protein C1	Protein Coding	45
NAA50	N-Alpha-Acetyltransferase 50, NatE Catalytic Subunit	Protein Coding	38

HNRNPC	Heterogeneous Nuclear Ribonucleoprotein C	Protein Coding	43
GNAO1	G Protein Subunit Alpha O1	Protein Coding	48
PIGW	Phosphatidylinositol Glycan Anchor Biosynthesis Class	Protein Coding	38
ADRB1	Adrenoceptor Beta 1	Protein Coding	49
DIABLO	Diablo IAP-Binding Mitochondrial Protein	Protein Coding	49
IGH	Immunoglobulin Heavy Locus	Protein Coding	16
SIX1	SIX Homeobox 1	Protein Coding	45
EEF2	Eukaryotic Translation Elongation Factor 2	Protein Coding	50
GNAT2	G Protein Subunit Alpha Transducin 2	Protein Coding	47
HRH1	Histamine Receptor H1	Protein Coding	47
VANGL1	VANGL Planar Cell Polarity Protein 1	Protein Coding	44
RNPC3	RNA Binding Region (RNP1, RRM) Containing 3	Protein Coding	36
GFI1	Growth Factor Independent 1 Transcriptional Represso	Protein Coding	43
AIMP1	Aminoacyl TRNA Synthetase Complex Interacting Mul	Protein Coding	44
DCDC2	Doublecortin Domain Containing 2	Protein Coding	40
SLC19A1	Solute Carrier Family 19 Member 1	Protein Coding	47
S100A6	S100 Calcium Binding Protein A6	Protein Coding	44
POLR3B	RNA Polymerase III Subunit B	Protein Coding	44
KDM6B	Lysine Demethylase 6B	Protein Coding	44
GJD3	Gap Junction Protein Delta 3	Protein Coding	34
MIR16-1	MicroRNA 16-1	RNA Gene	22
NPS	Neuropeptide S	Protein Coding	34
SFRP4	Secreted Frizzled Related Protein 4	Protein Coding	45
DCHS2	Dachsous Cadherin-Related 2	Protein Coding	33
SCP2	Sterol Carrier Protein 2	Protein Coding	47
GGCT	Gamma-Glutamylcyclotransferase	Protein Coding	40
DLL1	Delta Like Canonical Notch Ligand 1	Protein Coding	46
APRT	Adenine Phosphoribosyltransferase	Protein Coding	48
ANKS6	Ankyrin Repeat And Sterile Alpha Motif Domain Conta	Protein Coding	39
RPL10	Ribosomal Protein L10	Protein Coding	48
RPS26	Ribosomal Protein S26	Protein Coding	43
KCNH5	Potassium Voltage-Gated Channel Subfamily H Membe	Protein Coding	44
MMRN1	Multimerin 1	Protein Coding	40
PSMC1	Proteasome 26S Subunit, ATPase 1	Protein Coding	42
DERL1	Derlin 1	Protein Coding	37
AUTS2	Activator Of Transcription And Developmental Regulat	Protein Coding	40
POLD1	DNA Polymerase Delta 1, Catalytic Subunit	Protein Coding	47
ZNF750	Zinc Finger Protein 750	Protein Coding	37
SDC1	Syndecan 1	Protein Coding	45
MIP	Major Intrinsic Protein Of Lens Fiber	Protein Coding	43
GPRIN3	GPRIN Family Member 3	Protein Coding	32
PSMA5	Proteasome 20S Subunit Alpha 5	Protein Coding	44
UBD	Ubiquitin D	Protein Coding	40
GNPTG	N-Acetylglucosamine-1-Phosphate Transferase Subunit	Protein Coding	40
REPS1	RALBP1 Associated Eps Domain Containing 1	Protein Coding	40
NUP42	Nucleoporin 42	Protein Coding	29
FZD6	Frizzled Class Receptor 6	Protein Coding	48

DPM3	Dolichyl-Phosphate Mannosyltransferase Subunit 3, Re	Protein Coding	41
NR2F2	Nuclear Receptor Subfamily 2 Group F Member 2	Protein Coding	50
SYNJ2	Synaptojanin 2	Protein Coding	41
HIBCH	3-Hydroxyisobutyryl-CoA Hydrolase	Protein Coding	44
PSMB1	Proteasome 20S Subunit Beta 1	Protein Coding	44
TMEFF2	Transmembrane Protein With EGF Like And Two Folli	Protein Coding	40
CDH17	Cadherin 17	Protein Coding	43
LONP1	Lon Peptidase 1, Mitochondrial	Protein Coding	44
GSTA4	Glutathione S-Transferase Alpha 4	Protein Coding	43
NCKIPSD	NCK Interacting Protein With SH3 Domain	Protein Coding	37
PAK1	P21 (RAC1) Activated Kinase 1	Protein Coding	49
NOX4	NADPH Oxidase 4	Protein Coding	44
MAPRE3	Microtubule Associated Protein RP/EB Family Member	Protein Coding	40
ACTG2	Actin Gamma 2, Smooth Muscle	Protein Coding	47
SRSF4	Serine And Arginine Rich Splicing Factor 4	Protein Coding	40
FANCI	FA Complementation Group I	Protein Coding	43
DLG1	Discs Large MAGUK Scaffold Protein 1	Protein Coding	46
CLSTN1	Calsyntenin 1	Protein Coding	39
LGALS1	Galectin 1	Protein Coding	45
PTGIS	Prostaglandin I2 Synthase	Protein Coding	48
RPL7A	Ribosomal Protein L7a	Protein Coding	43
LAMA4	Laminin Subunit Alpha 4	Protein Coding	45
FDXR	Ferredoxin Reductase	Protein Coding	45
EPHA2	EPH Receptor A2	Protein Coding	54
CYP2A6	Cytochrome P450 Family 2 Subfamily A Member 6	Protein Coding	48
JCAD	Junctional Cadherin 5 Associated	Protein Coding	27
IL27	Interleukin 27	Protein Coding	40
FIP1L1	Factor Interacting With PAPOLA And CPSF1	Protein Coding	39
USP2	Ubiquitin Specific Peptidase 2	Protein Coding	44
ATF4	Activating Transcription Factor 4	Protein Coding	48
CHD1	Chromodomain Helicase DNA Binding Protein 1	Protein Coding	47
IGF2BP2	Insulin Like Growth Factor 2 MRNA Binding Protein 2	Protein Coding	44
FAM126A	Family With Sequence Similarity 126 Member A	Protein Coding	39
CAPN5	Calpain 5	Protein Coding	44
BSG	Basigin (Ok Blood Group)	Protein Coding	45
GPX4	Glutathione Peroxidase 4	Protein Coding	48
ROR2	Receptor Tyrosine Kinase Like Orphan Receptor 2	Protein Coding	50
MRAS	Muscle RAS Oncogene Homolog	Protein Coding	47
DNAJC19	DnaJ Heat Shock Protein Family (Hsp40) Member C19	Protein Coding	40
CENPJ	Centromere Protein J	Protein Coding	43
CPT1A	Carnitine Palmitoyltransferase 1A	Protein Coding	49
NEUROG2	Neurogenin 2	Protein Coding	40
MAPK11	Mitogen-Activated Protein Kinase 11	Protein Coding	50
GABBR2	Gamma-Aminobutyric Acid Type B Receptor Subunit 2	Protein Coding	48
SLC25A5	Solute Carrier Family 25 Member 5	Protein Coding	45
RPS24	Ribosomal Protein S24	Protein Coding	43
JAK1	Janus Kinase 1	Protein Coding	52

TTC37	Tetratricopeptide Repeat Domain 37	Protein Coding	40
RNF146	Ring Finger Protein 146	Protein Coding	37
TUFM	Tu Translation Elongation Factor, Mitochondrial	Protein Coding	45
ANK3	Ankyrin 3	Protein Coding	45
ATG13	Autophagy Related 13	Protein Coding	40
PSMC6	Proteasome 26S Subunit, ATPase 6	Protein Coding	42
NSUN2	NOP2/Sun RNA Methyltransferase 2	Protein Coding	43
HCFC1	Host Cell Factor C1	Protein Coding	48
MGAM	Maltase-Glucoamylase	Protein Coding	43
MGRN1	Mahogunin Ring Finger 1	Protein Coding	40
GSAP	Gamma-Secretase Activating Protein	Protein Coding	32
PSMB3	Proteasome 20S Subunit Beta 3	Protein Coding	42
GCDH	Glutaryl-CoA Dehydrogenase	Protein Coding	47
FDPS	Farnesyl Diphosphate Synthase	Protein Coding	46
PSMB5	Proteasome 20S Subunit Beta 5	Protein Coding	45
GNAI3	G Protein Subunit Alpha I3	Protein Coding	47
PKM	Pyruvate Kinase M1/2	Protein Coding	47
STX11	Syntaxin 11	Protein Coding	41
CUL2	Cullin 2	Protein Coding	43
DDR2	Discoidin Domain Receptor Tyrosine Kinase 2	Protein Coding	52
CXCL11	C-X-C Motif Chemokine Ligand 11	Protein Coding	41
ADRA2A	Adrenoceptor Alpha 2A	Protein Coding	48
BUB1B	BUB1 Mitotic Checkpoint Serine/Threonine Kinase B	Protein Coding	51
TGM3	Transglutaminase 3	Protein Coding	43
MAPK13	Mitogen-Activated Protein Kinase 13	Protein Coding	50
NDUFAF7	NADH:Ubiquinone Oxidoreductase Complex Assembly	Protein Coding	35
SMC3	Structural Maintenance Of Chromosomes 3	Protein Coding	47
FOXP2	Forkhead Box P2	Protein Coding	45
CCAT1	Colon Cancer Associated Transcript 1	RNA Gene	15
RPS19BP1	Ribosomal Protein S19 Binding Protein 1	Protein Coding	35
RBMS3	RNA Binding Motif Single Stranded Interacting Protein	Protein Coding	36
PCMT1	Protein-L-Isoaspartate (D-Aspartate) O-Methyltransfera	Protein Coding	41
GNPAT	Glyceronephosphate O-Acyltransferase	Protein Coding	46
NEDD4L	NEDD4 Like E3 Ubiquitin Protein Ligase	Protein Coding	48
XRCC5	X-Ray Repair Cross Complementing 5	Protein Coding	45
TAT	Tyrosine Aminotransferase	Protein Coding	45
CA4	Carbonic Anhydrase 4	Protein Coding	48
SPATA7	Spermatogenesis Associated 7	Protein Coding	38
MLST8	MTOR Associated Protein, LST8 Homolog	Protein Coding	44
PITRM1	Pitrilysin Metallopeptidase 1	Protein Coding	39
TRA2B	Transformer 2 Beta Homolog	Protein Coding	40
GORAB	Golgin, RAB6 Interacting	Protein Coding	39
FCER2	Fc Fragment Of IgE Receptor II	Protein Coding	47
MTMR14	Myotubularin Related Protein 14	Protein Coding	44
RPL11	Ribosomal Protein L11	Protein Coding	49
RREB1	Ras Responsive Element Binding Protein 1	Protein Coding	44
UFD1	Ubiquitin Recognition Factor In ER Associated Degrada	Protein Coding	36

NUP160	Nucleoporin 160	Protein Coding	40
RPS27	Ribosomal Protein S27	Protein Coding	43
SCT	Secretin	Protein Coding	36
CD1C	CD1c Molecule	Protein Coding	41
SEPTIN1	Septin 1	Protein Coding	31
COA7	Cytochrome C Oxidase Assembly Factor 7 (Putative)	Protein Coding	34
E2F1	E2F Transcription Factor 1	Protein Coding	45
FUT4	Fucosyltransferase 4	Protein Coding	38
CH25H	Cholesterol 25-Hydroxylase	Protein Coding	39
FKBP5	FKBP Prolyl Isomerase 5	Protein Coding	47
PHACTR1	Phosphatase And Actin Regulator 1	Protein Coding	38
MDH1	Malate Dehydrogenase 1	Protein Coding	45
ASPN	Asporin	Protein Coding	40
HIVEP2	HIVEP Zinc Finger 2	Protein Coding	38
AP1S1	Adaptor Related Protein Complex 1 Subunit Sigma 1	Protein Coding	40
PSMA2	Proteasome 20S Subunit Alpha 2	Protein Coding	44
COX7B	Cytochrome C Oxidase Subunit 7B	Protein Coding	41
COA3	Cytochrome C Oxidase Assembly Factor 3	Protein Coding	35
SOX2-OT	SOX2 Overlapping Transcript	RNA Gene	22
PSCA	Prostate Stem Cell Antigen	Protein Coding	40
CCT5	Chaperonin Containing TCP1 Subunit 5	Protein Coding	45
SRSF2	Serine And Arginine Rich Splicing Factor 2	Protein Coding	41
TIMM13	Translocase Of Inner Mitochondrial Membrane 13	Protein Coding	37
ARNTL	Aryl Hydrocarbon Receptor Nuclear Translocator Like	Protein Coding	44
STMN2	Stathmin 2	Protein Coding	40
NOTCH2N	Notch 2 N-Terminal Like C	Protein Coding	12
CNTFR	Ciliary Neurotrophic Factor Receptor	Protein Coding	45
SUPT4H1	SPT4 Homolog, DSIF Elongation Factor Subunit	Protein Coding	39
DLG3	Discs Large MAGUK Scaffold Protein 3	Protein Coding	43
KDM1A	Lysine Demethylase 1A	Protein Coding	48
APOC2	Apolipoprotein C2	Protein Coding	46
B4GALNT	Beta-1,4-N-Acetyl-Galactosaminyltransferase 2	Protein Coding	41
FBXL5	F-Box And Leucine Rich Repeat Protein 5	Protein Coding	36
SPTB	Spectrin Beta, Erythrocytic	Protein Coding	41
UGCG	UDP-Glucose Ceramide Glucosyltransferase	Protein Coding	43
GSTM3	Glutathione S-Transferase Mu 3	Protein Coding	47
FDFT1	Farnesyl-Diphosphate Farnesyltransferase 1	Protein Coding	46
OMD	Osteomodulin	Protein Coding	39
RPS20	Ribosomal Protein S20	Protein Coding	45
BLZF1	Basic Leucine Zipper Nuclear Factor 1	Protein Coding	40
LOC10902	Junctophilin 3 Repeat Instability Region	Biological Region	2
ALDH7A1	Aldehyde Dehydrogenase 7 Family Member A1	Protein Coding	48
CRLF1	Cytokine Receptor Like Factor 1	Protein Coding	42
CCKAR	Cholecystokinin A Receptor	Protein Coding	47
TRH	Thyrotropin Releasing Hormone	Protein Coding	43
HHIP	Hedgehog Interacting Protein	Protein Coding	43
NSD2	Nuclear Receptor Binding SET Domain Protein 2	Protein Coding	36

SLC22A12	Solute Carrier Family 22 Member 12	Protein Coding	45
STN1	STN1 Subunit Of CST Complex	Protein Coding	33
ACP1	Acid Phosphatase 1	Protein Coding	45
RTN3	Reticulon 3	Protein Coding	43
CISD2	CDGSH Iron Sulfur Domain 2	Protein Coding	43
TBC1D7	TBC1 Domain Family Member 7	Protein Coding	43
HFM1	Helicase For Meiosis 1	Protein Coding	40
KLK8	Kallikrein Related Peptidase 8	Protein Coding	43
PSMD1	Proteasome 26S Subunit, Non-ATPase 1	Protein Coding	41
TTBK1	Tau Tubulin Kinase 1	Protein Coding	36
HNRNPH1	Heterogeneous Nuclear Ribonucleoprotein H1	Protein Coding	39
MED13	Mediator Complex Subunit 13	Protein Coding	41
COX4I2	Cytochrome C Oxidase Subunit 4I2	Protein Coding	42
GCLC	Glutamate-Cysteine Ligase Catalytic Subunit	Protein Coding	45
CYBRD1	Cytochrome B Reductase 1	Protein Coding	41
MIR338	MicroRNA 338	RNA Gene	18
ACACA	Acetyl-CoA Carboxylase Alpha	Protein Coding	50
BANK1	B Cell Scaffold Protein With Ankyrin Repeats 1	Protein Coding	37
KISS1	KiSS-1 Metastasis Suppressor	Protein Coding	43
YWHAZ	Tyrosine 3-Monooxygenase/Tryptophan 5-Monooxygen	Protein Coding	50
NAE1	NEDD8 Activating Enzyme E1 Subunit 1	Protein Coding	43
ACTN1	Actinin Alpha 1	Protein Coding	50
ANKS1B	Ankyrin Repeat And Sterile Alpha Motif Domain Conta	Protein Coding	40
NDUFA5	NADH:Ubiquinone Oxidoreductase Subunit A5	Protein Coding	43
ITIH4	Inter-Alpha-Trypsin Inhibitor Heavy Chain 4	Protein Coding	44
FLAD1	Flavin Adenine Dinucleotide Synthetase 1	Protein Coding	42
CLEC4E	C-Type Lectin Domain Family 4 Member E	Protein Coding	39
PNMA2	PNMA Family Member 2	Protein Coding	37
CYP46A1	Cytochrome P450 Family 46 Subfamily A Member 1	Protein Coding	42
PPID	Peptidylprolyl Isomerase D	Protein Coding	45
AMT	Aminomethyltransferase	Protein Coding	47
ACTN2	Actinin Alpha 2	Protein Coding	48
PDXK	Pyridoxal Kinase	Protein Coding	48
PNMT	Phenylethanolamine N-Methyltransferase	Protein Coding	45
PSMA3	Proteasome 20S Subunit Alpha 3	Protein Coding	45
FANCM	FA Complementation Group M	Protein Coding	43
UBE2L6	Ubiquitin Conjugating Enzyme E2 L6	Protein Coding	41
ADORA1	Adenosine A1 Receptor	Protein Coding	48
TRIM28	Tripartite Motif Containing 28	Protein Coding	44
MIPEP	Mitochondrial Intermediate Peptidase	Protein Coding	43
FGF21	Fibroblast Growth Factor 21	Protein Coding	41
MAP2K7	Mitogen-Activated Protein Kinase Kinase 7	Protein Coding	47
ATP6V1A	ATPase H+ Transporting V1 Subunit A	Protein Coding	44
PPP1R3C	Protein Phosphatase 1 Regulatory Subunit 3C	Protein Coding	40
ANK1	Ankyrin 1	Protein Coding	44
FKBP10	FKBP Prolyl Isomerase 10	Protein Coding	41
MELTF	Melanotransferrin	Protein Coding	34

MIR183	MicroRNA 183	RNA Gene	19
CLEC6A	C-Type Lectin Domain Containing 6A	Protein Coding	38
F2RL1	F2R Like Trypsin Receptor 1	Protein Coding	47
SYN2	Synapsin II	Protein Coding	40
LBP	Lipopolysaccharide Binding Protein	Protein Coding	45
NDUFAB1	NADH:Ubiquinone Oxidoreductase Subunit AB1	Protein Coding	41
ABCB6	ATP Binding Cassette Subfamily B Member 6 (Langere	Protein Coding	48
RABAC1	Rab Acceptor 1	Protein Coding	37
PTK2	Protein Tyrosine Kinase 2	Protein Coding	49
NMT1	N-Myristoyltransferase 1	Protein Coding	44
AKAP9	A-Kinase Anchoring Protein 9	Protein Coding	43
BMP5	Bone Morphogenetic Protein 5	Protein Coding	43
STXBP2	Syntaxin Binding Protein 2	Protein Coding	46
VPS33B	VPS33B Late Endosome And Lysosome Associated	Protein Coding	42
MAPK9	Mitogen-Activated Protein Kinase 9	Protein Coding	50
SLC25A2(	Solute Carrier Family 25 Member 20	Protein Coding	47
SUCLA2	Succinate-CoA Ligase ADP-Forming Subunit Beta	Protein Coding	47
UBE2J2	Ubiquitin Conjugating Enzyme E2 J2	Protein Coding	42
SERAC1	Serine Active Site Containing 1	Protein Coding	37
PSMB2	Proteasome 20S Subunit Beta 2	Protein Coding	42
OPRK1	Opioid Receptor Kappa 1	Protein Coding	46
ATXN3L	Ataxin 3 Like	Protein Coding	35
HES1	Hes Family BHLH Transcription Factor 1	Protein Coding	45
BRD3	Bromodomain Containing 3	Protein Coding	43
NUS1	NUS1 Dehydrodolichyl Diphosphate Synthase Subunit	Protein Coding	40
LDHB	Lactate Dehydrogenase B	Protein Coding	48
CYB5A	Cytochrome B5 Type A	Protein Coding	45
RAN	RAN, Member RAS Oncogene Family	Protein Coding	46
PRKACG	Protein Kinase CAMP-Activated Catalytic Subunit Gan	Protein Coding	49
BDKRB2	Bradykinin Receptor B2	Protein Coding	45
CAD	Carbamoyl-Phosphate Synthetase 2, Aspartate Transcai	Protein Coding	51
NT5C2	5'-Nucleotidase, Cytosolic II	Protein Coding	47
TRAT1	T Cell Receptor Associated Transmembrane Adaptor 1	Protein Coding	38
UBE2D1	Ubiquitin Conjugating Enzyme E2 D1	Protein Coding	47
ACSL4	Acyl-CoA Synthetase Long Chain Family Member 4	Protein Coding	45
IBSP	Integrin Binding Sialoprotein	Protein Coding	39
COX5B	Cytochrome C Oxidase Subunit 5B	Protein Coding	42
RP2	RP2 Activator Of ARL3 GTPase	Protein Coding	43
MAP2K4	Mitogen-Activated Protein Kinase Kinase 4	Protein Coding	47
CKAP5	Cytoskeleton Associated Protein 5	Protein Coding	41
MIR224	MicroRNA 224	RNA Gene	18
SOX5	SRY-Box Transcription Factor 5	Protein Coding	48
MIR128-2	MicroRNA 128-2	RNA Gene	21
DLG2	Discs Large MAGUK Scaffold Protein 2	Protein Coding	42
FBL	Fibrillarin	Protein Coding	44
SPHK2	Sphingosine Kinase 2	Protein Coding	44
CSH1	Chorionic Somatomammotropin Hormone 1	Protein Coding	39

LRG1	Leucine Rich Alpha-2-Glycoprotein 1	Protein Coding	40
RMDN3	Regulator Of Microtubule Dynamics 3	Protein Coding	34
TBPL1	TATA-Box Binding Protein Like 1	Protein Coding	41
NUDT6	Nudix Hydrolase 6	Protein Coding	38
STX1A	Syntaxin 1A	Protein Coding	48
MIR137	MicroRNA 137	RNA Gene	18
RP9	RP9 Pre-MRNA Splicing Factor	Protein Coding	39
MARS2	Methionyl-TRNA Synthetase 2, Mitochondrial	Protein Coding	43
MIR92A2	MicroRNA 92a-2	RNA Gene	17
CUL4B	Cullin 4B	Protein Coding	45
POLI	DNA Polymerase Iota	Protein Coding	43
ATG9A	Autophagy Related 9A	Protein Coding	41
RSU1	Ras Suppressor Protein 1	Protein Coding	41
ELP3	Elongator Acetyltransferase Complex Subunit 3	Protein Coding	40
PIGG	Phosphatidylinositol Glycan Anchor Biosynthesis Class	Protein Coding	41
KAT8	Lysine Acetyltransferase 8	Protein Coding	41
GABARAI	GABA Type A Receptor-Associated Protein	Protein Coding	45
CD200R1	CD200 Receptor 1	Protein Coding	41
ACTR3C	Actin Related Protein 3C	Protein Coding	31
PER3	Period Circadian Regulator 3	Protein Coding	44
CACNA2I	Calcium Voltage-Gated Channel Auxiliary Subunit Alpl	Protein Coding	47
RIT1	Ras Like Without CAAX 1	Protein Coding	47
CABIN1	Calcineurin Binding Protein 1	Protein Coding	43
SREBF2	Sterol Regulatory Element Binding Transcription Factoi	Protein Coding	44
APLP2	Amyloid Beta Precursor Like Protein 2	Protein Coding	43
DVL2	Dishevelled Segment Polarity Protein 2	Protein Coding	47
NPPC	Natriuretic Peptide C	Protein Coding	41
TNFRSF1(	TNF Receptor Superfamily Member 10a	Protein Coding	47
CHROMR	Cholesterol Induced Regulator Of Metabolism RNA	RNA Gene	11
TOMM22	Translocase Of Outer Mitochondrial Membrane 22	Protein Coding	37
GRK5	G Protein-Coupled Receptor Kinase 5	Protein Coding	44
ATAD3A	ATPase Family AAA Domain Containing 3A	Protein Coding	39
AOC1	Amine Oxidase Copper Containing 1	Protein Coding	41
CDC37	Cell Division Cycle 37	Protein Coding	43
TRAF2	TNF Receptor Associated Factor 2	Protein Coding	46
ABCC1	ATP Binding Cassette Subfamily C Member 1	Protein Coding	48
SIN3A	SIN3 Transcription Regulator Family Member A	Protein Coding	47
PIK3R4	Phosphoinositide-3-Kinase Regulatory Subunit 4	Protein Coding	48
UBE2J1	Ubiquitin Conjugating Enzyme E2 J1	Protein Coding	41
CDH13	Cadherin 13	Protein Coding	43
NUCKS1	Nuclear Casein Kinase And Cyclin Dependent Kinase S	Protein Coding	37
GHRH	Growth Hormone Releasing Hormone	Protein Coding	41
CD1E	CD1e Molecule	Protein Coding	40
ALLC	Allantoicase	Protein Coding	36
MRPS34	Mitochondrial Ribosomal Protein S34	Protein Coding	38
NEUROG:	Neurogenin 3	Protein Coding	42
KIAA1191	KIAA1191	Protein Coding	35

CCSAP	Centriole, Cilia And Spindle Associated Protein	Protein Coding	31
WIPI2	WD Repeat Domain, Phosphoinositide Interacting 2	Protein Coding	42
TAC3	Tachykinin Precursor 3	Protein Coding	46
ROBO3	Roundabout Guidance Receptor 3	Protein Coding	43
CYP2B6	Cytochrome P450 Family 2 Subfamily B Member 6	Protein Coding	49
CCDC28B	Coiled-Coil Domain Containing 28B	Protein Coding	38
ARMC9	Armadillo Repeat Containing 9	Protein Coding	39
IGFALS	Insulin Like Growth Factor Binding Protein Acid Labile	Protein Coding	45
OAS1	2'-5'-Oligoadenylate Synthetase 1	Protein Coding	46
PGM3	Phosphoglucomutase 3	Protein Coding	44
WDR6	WD Repeat Domain 6	Protein Coding	36
TAB2	TGF-Beta Activated Kinase 1 (MAP3K7) Binding Prot	Protein Coding	48
HNRNPK	Heterogeneous Nuclear Ribonucleoprotein K	Protein Coding	47
ALOX12	Arachidonate 12-Lipoxygenase, 12S Type	Protein Coding	45
NUP93	Nucleoporin 93	Protein Coding	42
MICU1	Mitochondrial Calcium Uptake 1	Protein Coding	40
IPPK	Inositol-Pentakisphosphate 2-Kinase	Protein Coding	39
GEMIN8	Gem Nuclear Organelle Associated Protein 8	Protein Coding	37
SF3B6	Splicing Factor 3b Subunit 6	Protein Coding	36
STIL	STIL Centriolar Assembly Protein	Protein Coding	42
MICB	MHC Class I Polypeptide-Related Sequence B	Protein Coding	43
ATG14	Autophagy Related 14	Protein Coding	39
RDX	Radixin	Protein Coding	50
PTPN3	Protein Tyrosine Phosphatase Non-Receptor Type 3	Protein Coding	45
KLC1	Kinesin Light Chain 1	Protein Coding	42
OXA1L	OXA1L Mitochondrial Inner Membrane Protein	Protein Coding	40
SPINT2	Serine Peptidase Inhibitor, Kunitz Type 2	Protein Coding	45
RPS17	Ribosomal Protein S17	Protein Coding	43
RBMX	RNA Binding Motif Protein X-Linked	Protein Coding	42
CD207	CD207 Molecule	Protein Coding	40
MACF1	Microtubule Actin Crosslinking Factor 1	Protein Coding	41
TSHZ3	Teashirt Zinc Finger Homeobox 3	Protein Coding	39
CDCA7L	Cell Division Cycle Associated 7 Like	Protein Coding	40
NME1	NME/NM23 Nucleoside Diphosphate Kinase 1	Protein Coding	49
PRKACB	Protein Kinase CAMP-Activated Catalytic Subunit Beta	Protein Coding	49
CDC34	Cell Division Cycle 34	Protein Coding	48
MARK1	Microtubule Affinity Regulating Kinase 1	Protein Coding	43
NME8	NME/NM23 Family Member 8	Protein Coding	41
RPS15	Ribosomal Protein S15	Protein Coding	41
PCDH11X	Protocadherin 11 X-Linked	Protein Coding	36
ZIC1	Zic Family Member 1	Protein Coding	47
ATP5F1D	ATP Synthase F1 Subunit Delta	Protein Coding	35
RNF43	Ring Finger Protein 43	Protein Coding	39
CALML3	Calmodulin Like 3	Protein Coding	43
COX7A2	Cytochrome C Oxidase Subunit 7A2	Protein Coding	39
INHBA	Inhibin Subunit Beta A	Protein Coding	45
ALDOB	Aldolase, Fructose-Bisphosphate B	Protein Coding	45

RPL34	Ribosomal Protein L34	Protein Coding	42
POMK	Protein O-Mannose Kinase	Protein Coding	37
CNOT3	CCR4-NOT Transcription Complex Subunit 3	Protein Coding	43
APEH	Acylaminoacyl-Peptide Hydrolase	Protein Coding	41
SLC25A3	Solute Carrier Family 25 Member 3	Protein Coding	47
FBP1	Fructose-Bisphosphatase 1	Protein Coding	51
TFPI	Tissue Factor Pathway Inhibitor	Protein Coding	46
ETM3	Tremor, Hereditary Essential, 3	Genetic Locus	2
CFL2	Cofilin 2	Protein Coding	46
SORCS3	Sortilin Related VPS10 Domain Containing Receptor 3	Protein Coding	37
PCK1	Phosphoenolpyruvate Carboxykinase 1	Protein Coding	48
PRR12	Proline Rich 12	Protein Coding	33
NFIA	Nuclear Factor I A	Protein Coding	46
SGCG	Sarcoglycan Gamma	Protein Coding	44
DOCK7	Dedicator Of Cytokinesis 7	Protein Coding	42
TNKS	Tankyrase	Protein Coding	44
RSPO1	R-Spondin 1	Protein Coding	45
MCPH1	Microcephalin 1	Protein Coding	42
IFNL3	Interferon Lambda 3	Protein Coding	36
HCRTR2	Hypocretin Receptor 2	Protein Coding	44
HOOK1	Hook Microtubule Tethering Protein 1	Protein Coding	38
NR4A1	Nuclear Receptor Subfamily 4 Group A Member 1	Protein Coding	48
CEP55	Centrosomal Protein 55	Protein Coding	41
PSAT1	Phosphoserine Aminotransferase 1	Protein Coding	48
PMS1	PMS1 Homolog 1, Mismatch Repair System Componer	Protein Coding	41
TIMM50	Translocase Of Inner Mitochondrial Membrane 50	Protein Coding	39
JMJD1C	Jumonji Domain Containing 1C	Protein Coding	41
CYB5R3	Cytochrome B5 Reductase 3	Protein Coding	45
GRHL2	Grainyhead Like Transcription Factor 2	Protein Coding	41
CD1B	CD1b Molecule	Protein Coding	41
TCP1	T-Complex 1	Protein Coding	43
KCNH1	Potassium Voltage-Gated Channel Subfamily H Membe	Protein Coding	47
SNORD56	Small Nucleolar RNA, C/D Box 56B	RNA Gene	13
SNX10	Sorting Nexin 10	Protein Coding	40
SEMA3C	Semaphorin 3C	Protein Coding	44
CNGB1	Cyclic Nucleotide Gated Channel Subunit Beta 1	Protein Coding	43
NEBL	Nebulette	Protein Coding	39
ALOX15	Arachidonate 15-Lipoxygenase	Protein Coding	45
FLII	FLII Actin Remodeling Protein	Protein Coding	43
RRAS2	RAS Related 2	Protein Coding	47
CCDC88C	Coiled-Coil Domain Containing 88C	Protein Coding	41
MTNR1B	Melatonin Receptor 1B	Protein Coding	47
NAT1	N-Acetyltransferase 1	Protein Coding	46
RGS2	Regulator Of G Protein Signaling 2	Protein Coding	44
TUBB4B	Tubulin Beta 4B Class IVb	Protein Coding	45
LAS1L	LAS1 Like Ribosome Biogenesis Factor	Protein Coding	38
GALE	UDP-Galactose-4-Epimerase	Protein Coding	47

LAMP3	Lysosomal Associated Membrane Protein 3	Protein Coding	40
ERG	ETS Transcription Factor ERG	Protein Coding	47
NFU1	NFU1 Iron-Sulfur Cluster Scaffold	Protein Coding	43
MAK	Male Germ Cell Associated Kinase	Protein Coding	44
SUPT5H	SPT5 Homolog, DSIF Elongation Factor Subunit	Protein Coding	40
PPP1CB	Protein Phosphatase 1 Catalytic Subunit Beta	Protein Coding	47
PPARD	Peroxisome Proliferator Activated Receptor Delta	Protein Coding	48
EXOC3L2	Exocyst Complex Component 3 Like 2	Protein Coding	34
ZNF365	Zinc Finger Protein 365	Protein Coding	39
NMNAT1	Nicotinamide Nucleotide Adenylyltransferase 1	Protein Coding	48
PSMC3	Proteasome 26S Subunit, ATPase 3	Protein Coding	43
JAG2	Jagged Canonical Notch Ligand 2	Protein Coding	43
IL17C	Interleukin 17C	Protein Coding	39
PLN	Phospholamban	Protein Coding	45
DEPTOR	DEP Domain Containing MTOR Interacting Protein	Protein Coding	41
DBNL	Drebrin Like	Protein Coding	40
CCBE1	Collagen And Calcium Binding EGF Domains 1	Protein Coding	41
PSMC3IP	PSMC3 Interacting Protein	Protein Coding	40
PRDX2	Peroxiredoxin 2	Protein Coding	48
ADAM8	ADAM Metallopeptidase Domain 8	Protein Coding	44
NEK11	NIMA Related Kinase 11	Protein Coding	39
ADAM19	ADAM Metallopeptidase Domain 19	Protein Coding	43
MARCKS	Myristoylated Alanine Rich Protein Kinase C Substrate	Protein Coding	41
GEMIN2	Gem Nuclear Organelle Associated Protein 2	Protein Coding	39
SSBP1	Single Stranded DNA Binding Protein 1	Protein Coding	40
NCL	Nucleolin	Protein Coding	44
ESRRB	Estrogen Related Receptor Beta	Protein Coding	51
RPL21	Ribosomal Protein L21	Protein Coding	44
RSPO2	R-Spondin 2	Protein Coding	44
GABRR3	Gamma-Aminobutyric Acid Type A Receptor Subunit R	Protein Coding	37
SCO1	Synthesis Of Cytochrome C Oxidase 1	Protein Coding	48
SEC23B	SEC23 Homolog B, COPII Coat Complex Component	Protein Coding	43
SCFD1	Sec1 Family Domain Containing 1	Protein Coding	38
DAXX	Death Domain Associated Protein	Protein Coding	45
ALAS1	5'-Aminolevulinate Synthase 1	Protein Coding	45
DAB2IP	DAB2 Interacting Protein	Protein Coding	40
SIPA1L2	Signal Induced Proliferation Associated 1 Like 2	Protein Coding	39
MPI	Mannose Phosphate Isomerase	Protein Coding	45
ADPRH	ADP-Ribosylarginine Hydrolase	Protein Coding	38
SRY	Sex Determining Region Y	Protein Coding	37
AUH	AU RNA Binding Methylglutaconyl-CoA Hydratase	Protein Coding	44
TIMM23	Translocase Of Inner Mitochondrial Membrane 23	Protein Coding	32
CD38	CD38 Molecule	Protein Coding	46
ATF2	Activating Transcription Factor 2	Protein Coding	47
PDGFC	Platelet Derived Growth Factor C	Protein Coding	44
ABT1	Activator Of Basal Transcription 1	Protein Coding	35
BACE1-AS	BACE1 Antisense RNA	RNA Gene	14

IVD	Isovaleryl-CoA Dehydrogenase	Protein Coding	46
DCXR	Dicarbonyl And L-Xylulose Reductase	Protein Coding	44
BCYRN1	Brain Cytoplasmic RNA 1	RNA Gene	18
MPP5	Membrane Palmitoylated Protein 5	Protein Coding	40
DOCK6	Dedicator Of Cytokinesis 6	Protein Coding	41
MROH8	Maestro Heat Like Repeat Family Member 8	Protein Coding	30
HPS4	HPS4 Biogenesis Of Lysosomal Organelles Complex 3	Protein Coding	40
XRCC6	X-Ray Repair Cross Complementing 6	Protein Coding	47
PLB1	Phospholipase B1	Protein Coding	37
GZMM	Granzyme M	Protein Coding	40
FEN1	Flap Structure-Specific Endonuclease 1	Protein Coding	48
RPL7	Ribosomal Protein L7	Protein Coding	42
PPCS	Phosphopantothenoylcysteine Synthetase	Protein Coding	41
NCOR1	Nuclear Receptor Corepressor 1	Protein Coding	45
AMPD2	Adenosine Monophosphate Deaminase 2	Protein Coding	49
CSN1S1	Casein Alpha S1	Protein Coding	34
CILK1	Ciliogenesis Associated Kinase 1	Protein Coding	36
HSP90AB	Heat Shock Protein 90 Alpha Family Class B Member 1	Protein Coding	47
SLX4	SLX4 Structure-Specific Endonuclease Subunit	Protein Coding	39
PCGF2	Polycomb Group Ring Finger 2	Protein Coding	40
RPLP1	Ribosomal Protein Lateral Stalk Subunit P1	Protein Coding	39
P3H1	Prolyl 3-Hydroxylase 1	Protein Coding	40
TBCK	TBC1 Domain Containing Kinase	Protein Coding	41
CNTN4	Contactin 4	Protein Coding	41
CSNK2B	Casein Kinase 2 Beta	Protein Coding	49
MIR96	MicroRNA 96	RNA Gene	21
KCNJ8	Potassium Inwardly Rectifying Channel Subfamily J M	Protein Coding	44
PSMC2	Proteasome 26S Subunit, ATPase 2	Protein Coding	40
ESRRG	Estrogen Related Receptor Gamma	Protein Coding	46
PPP2CA	Protein Phosphatase 2 Catalytic Subunit Alpha	Protein Coding	50
TFCP2	Transcription Factor CP2	Protein Coding	41
MARK4	Microtubule Affinity Regulating Kinase 4	Protein Coding	44
CHD4	Chromodomain Helicase DNA Binding Protein 4	Protein Coding	46
MIR497	MicroRNA 497	RNA Gene	18
MIR20B	MicroRNA 20b	RNA Gene	16
ARPC1B	Actin Related Protein 2/3 Complex Subunit 1B	Protein Coding	44
LIPT1	Lipoyltransferase 1	Protein Coding	42
MYO6	Myosin VI	Protein Coding	47
CREB3L4	CAMP Responsive Element Binding Protein 3 Like 4	Protein Coding	38
UTS2R	Urotensin 2 Receptor	Protein Coding	43
WDR41	WD Repeat Domain 41	Protein Coding	37
ULK1	Unc-51 Like Autophagy Activating Kinase 1	Protein Coding	47
CLCN6	Chloride Voltage-Gated Channel 6	Protein Coding	43
CRTAP	Cartilage Associated Protein	Protein Coding	41
APBA2	Amyloid Beta Precursor Protein Binding Family A Men	Protein Coding	43
SLC25A1	Solute Carrier Family 25 Member 12	Protein Coding	47
RPLP0	Ribosomal Protein Lateral Stalk Subunit P0	Protein Coding	42

CDC27	Cell Division Cycle 27	Protein Coding	43
NAV1	Neuron Navigator 1	Protein Coding	38
TMEM163	Transmembrane Protein 163	Protein Coding	33
MAPRE2	Microtubule Associated Protein RP/EB Family Member	Protein Coding	42
MEOX2	Mesenchyme Homeobox 2	Protein Coding	41
SH3GL2	SH3 Domain Containing GRB2 Like 2, Endophilin A1	Protein Coding	43
GDF9	Growth Differentiation Factor 9	Protein Coding	40
INSL3	Insulin Like 3	Protein Coding	42
RPS2	Ribosomal Protein S2	Protein Coding	44
GJD2	Gap Junction Protein Delta 2	Protein Coding	43
ELAVL1	ELAV Like RNA Binding Protein 1	Protein Coding	43
WAPL	WAPL Cohesin Release Factor	Protein Coding	32
CUL3	Cullin 3	Protein Coding	48
CUX1	Cut Like Homeobox 1	Protein Coding	44
APLP1	Amyloid Beta Precursor Like Protein 1	Protein Coding	41
SULT1A1	Sulfotransferase Family 1A Member 1	Protein Coding	44
NPY4R	Neuropeptide Y Receptor Y4	Protein Coding	37
SLC25A17	Solute Carrier Family 25 Member 17	Protein Coding	40
PRNT	Prion Locus LncRNA, Testis Expressed	RNA Gene	26
SRP72	Signal Recognition Particle 72	Protein Coding	40
FPR2	Formyl Peptide Receptor 2	Protein Coding	47
FAU	FAU Ubiquitin Like And Ribosomal Protein S30 Fusion	Protein Coding	40
ODAPH	Odontogenesis Associated Phosphoprotein	Protein Coding	28
DPYSL5	Dihydropyrimidinase Like 5	Protein Coding	38
NUB1	Negative Regulator Of Ubiquitin Like Proteins 1	Protein Coding	39
BAZ2B	Bromodomain Adjacent To Zinc Finger Domain 2B	Protein Coding	40
URI1	URI1 Prefoldin Like Chaperone	Protein Coding	37
MSI1	Musashi RNA Binding Protein 1	Protein Coding	41
ISCA2	Iron-Sulfur Cluster Assembly 2	Protein Coding	40
RPS15A	Ribosomal Protein S15a	Protein Coding	43
MAZ	MYC Associated Zinc Finger Protein	Protein Coding	41
APH1B	Aph-1 Homolog B, Gamma-Secretase Subunit	Protein Coding	44
KIR2DL3	Killer Cell Immunoglobulin Like Receptor, Two Ig Don	Protein Coding	37
RBX1	Ring-Box 1	Protein Coding	44
ADCY1	Adenylate Cyclase 1	Protein Coding	49
SALL2	Spalt Like Transcription Factor 2	Protein Coding	43
VEZT	Vezatin, Adherens Junctions Transmembrane Protein	Protein Coding	35
TRPC3	Transient Receptor Potential Cation Channel Subfamily	Protein Coding	50
AP1S2	Adaptor Related Protein Complex 1 Subunit Sigma 2	Protein Coding	44
RPS14	Ribosomal Protein S14	Protein Coding	45
PGD	Phosphogluconate Dehydrogenase	Protein Coding	48
WASF1	WASP Family Member 1	Protein Coding	45
ALDH9A1	Aldehyde Dehydrogenase 9 Family Member A1	Protein Coding	42
ASPH	Aspartate Beta-Hydroxylase	Protein Coding	44
ATXN2L	Ataxin 2 Like	Protein Coding	38
BLMH	Bleomycin Hydrolase	Protein Coding	44
PYCR1	Pyrroline-5-Carboxylate Reductase 1	Protein Coding	48

TGOLN2	Trans-Golgi Network Protein 2	Protein Coding	38
KCNA5	Potassium Voltage-Gated Channel Subfamily A Membe	Protein Coding	47
APCS	Amyloid P Component, Serum	Protein Coding	42
SPRN	Shadow Of Prion Protein	Protein Coding	33
MIR218-1	MicroRNA 218-1	RNA Gene	18
PRSS2	Serine Protease 2	Protein Coding	40
NEDD9	Neural Precursor Cell Expressed, Developmentally Dow	Protein Coding	44
CAPRIN1	Cell Cycle Associated Protein 1	Protein Coding	37
UBE2G1	Ubiquitin Conjugating Enzyme E2 G1	Protein Coding	44
IL13RA2	Interleukin 13 Receptor Subunit Alpha 2	Protein Coding	41
PZP	PZP Alpha-2-Macroglobulin Like	Protein Coding	40
CARTPT	CART Prepropeptide	Protein Coding	44
TUBB6	Tubulin Beta 6 Class V	Protein Coding	43
GPR3	G Protein-Coupled Receptor 3	Protein Coding	39
GRIA4	Glutamate Ionotropic Receptor AMPA Type Subunit 4	Protein Coding	50
SACM1L	SAC1 Like Phosphatidylinositide Phosphatase	Protein Coding	40
WAC	WW Domain Containing Adaptor With Coiled-Coil	Protein Coding	40
CTH	Cystathionine Gamma-Lyase	Protein Coding	51
MSTO1	Misato Mitochondrial Distribution And Morphology Re	Protein Coding	40
MIR135A1	MicroRNA 135a-1	RNA Gene	21
KIFAP3	Kinesin Associated Protein 3	Protein Coding	42
S100A1	S100 Calcium Binding Protein A1	Protein Coding	43
PGA4	Pepsinogen A4	Protein Coding	30
HBD	Hemoglobin Subunit Delta	Protein Coding	40
TXNDC15	Thioredoxin Domain Containing 15	Protein Coding	37
KCNIP3	Potassium Voltage-Gated Channel Interacting Protein 3	Protein Coding	40
MIR219A1	MicroRNA 219a-1	RNA Gene	20
ABAT	4-Aminobutyrate Aminotransferase	Protein Coding	46
SPEG	Striated Muscle Enriched Protein Kinase	Protein Coding	42
TECRL	Trans-2,3-Enoyl-CoA Reductase Like	Protein Coding	36
RPS11	Ribosomal Protein S11	Protein Coding	41
CANX	Calnexin	Protein Coding	46
PAICS	Phosphoribosylaminoimidazole Carboxylase And Phosp	Protein Coding	43
IL26	Interleukin 26	Protein Coding	35
OTOG	Otogelin	Protein Coding	33
GPI	Glucose-6-Phosphate Isomerase	Protein Coding	48
ANKK1	Ankyrin Repeat And Kinase Domain Containing 1	Protein Coding	39
SMTNL2	Smoothelin Like 2	Protein Coding	33
PSMB6	Proteasome 20S Subunit Beta 6	Protein Coding	43
PLA2G1B	Phospholipase A2 Group IB	Protein Coding	48
EHHADH	Enoyl-CoA Hydratase And 3-Hydroxyacyl CoA Dehydr	Protein Coding	45
AREG	Amphiregulin	Protein Coding	45
MAGED2	MAGE Family Member D2	Protein Coding	40
SH3GL1	SH3 Domain Containing GRB2 Like 1, Endophilin A2	Protein Coding	43
TTC3	Tetratricopeptide Repeat Domain 3	Protein Coding	40
PPIG	Peptidylprolyl Isomerase G	Protein Coding	43
GRIP1	Glutamate Receptor Interacting Protein 1	Protein Coding	43

REV3L	REV3 Like, DNA Directed Polymerase Zeta Catalytic S	Protein Coding	45
IGF2BP3	Insulin Like Growth Factor 2 MRNA Binding Protein 3	Protein Coding	43
RAI14	Retinoic Acid Induced 14	Protein Coding	37
UBE2T	Ubiquitin Conjugating Enzyme E2 T	Protein Coding	44
EBP	EBP Cholestenol Delta-Isomerase	Protein Coding	44
ACY1	Aminoacylase 1	Protein Coding	47
ATOH1	Atonal BHLH Transcription Factor 1	Protein Coding	39
TOX3	TOX High Mobility Group Box Family Member 3	Protein Coding	38
ORMDL3	ORMDL Sphingolipid Biosynthesis Regulator 3	Protein Coding	40
GTF2E2	General Transcription Factor IIE Subunit 2	Protein Coding	44
C1orf210	Chromosome 1 Open Reading Frame 210	Protein Coding	31
SLC29A1	Solute Carrier Family 29 Member 1 (Augustine Blood (	Protein Coding	48
PLEKHB1	Pleckstrin Homology Domain Containing B1	Protein Coding	37
PRKAB1	Protein Kinase AMP-Activated Non-Catalytic Subunit I	Protein Coding	49
CD58	CD58 Molecule	Protein Coding	41
MTHFSD	Methenyltetrahydrofolate Synthetase Domain Containin	Protein Coding	36
LMLN	Leishmanolysin Like Peptidase	Protein Coding	35
L2HGDH	L-2-Hydroxyglutarate Dehydrogenase	Protein Coding	40
CDC20	Cell Division Cycle 20	Protein Coding	45
SLC30A6	Solute Carrier Family 30 Member 6	Protein Coding	39
IARS1	Isoleucyl-TRNA Synthetase 1	Protein Coding	35
INPP5D	Inositol Polyphosphate-5-Phosphatase D	Protein Coding	47
PNOC	Prepronociceptin	Protein Coding	38
TECR	Trans-2,3-Enoyl-CoA Reductase	Protein Coding	44
MCM5	Minichromosome Maintenance Complex Component 5	Protein Coding	46
TBL2	Transducin Beta Like 2	Protein Coding	40
SYNM	Synemin	Protein Coding	38
MFN1	Mitofusin 1	Protein Coding	43
DOP1B	DOP1 Leucine Zipper Like Protein B	Protein Coding	27
TFB1M	Transcription Factor B1, Mitochondrial	Protein Coding	43
APBB2	Amyloid Beta Precursor Protein Binding Family B Men	Protein Coding	40
QPCT	Glutaminyl-Peptide Cyclotransferase	Protein Coding	44
FDX2	Ferredoxin 2	Protein Coding	29
GLYAT	Glycine-N-Acyltransferase	Protein Coding	40
CCNA2	Cyclin A2	Protein Coding	46
PNPT1	Polyribonucleotide Nucleotidyltransferase 1	Protein Coding	43
TNFRSF2	TNF Receptor Superfamily Member 21	Protein Coding	47
GHSR	Growth Hormone Secretagogue Receptor	Protein Coding	48
KCNJ6	Potassium Inwardly Rectifying Channel Subfamily J Mc	Protein Coding	48
CBLL1	Cbl Proto-Oncogene Like 1	Protein Coding	38
HCRTR1	Hypocretin Receptor 1	Protein Coding	44
NACC1	Nucleus Accumbens Associated 1	Protein Coding	40
DROSHA	Drosha Ribonuclease III	Protein Coding	43
DNAAF2	Dynein Axonemal Assembly Factor 2	Protein Coding	38
TUBGCP6	Tubulin Gamma Complex Associated Protein 6	Protein Coding	40
MIR184	MicroRNA 184	RNA Gene	23
AKT1S1	AKT1 Substrate 1	Protein Coding	41

LINC0119	Long Intergenic Non-Protein Coding RNA 1194	RNA Gene	17
ADH6	Alcohol Dehydrogenase 6 (Class V)	Protein Coding	41
HLA-DMA	Major Histocompatibility Complex, Class II, DM Alpha	Protein Coding	41
RPL10AP7	Ribosomal Protein L10a Pseudogene 7	Pseudogene	6
BLOC1S6	Biogenesis Of Lysosomal Organelles Complex 1 Subun	Protein Coding	39
SNRNP70	Small Nuclear Ribonucleoprotein U1 Subunit 70	Protein Coding	39
PCK2	Phosphoenolpyruvate Carboxykinase 2, Mitochondrial	Protein Coding	48
GPC6	Glypican 6	Protein Coding	44
UVSSA	UV Stimulated Scaffold Protein A	Protein Coding	36
CYTL1	Cytokine Like 1	Protein Coding	35
PAX1	Paired Box 1	Protein Coding	43
RSPO4	R-Spondin 4	Protein Coding	38
MARCHF4	Membrane Associated Ring-CH-Type Finger 4	Protein Coding	27
DNASE1L	Deoxyribonuclease 1 Like 1	Protein Coding	39
MALRD1	MAM And LDL Receptor Class A Domain Containing	Protein Coding	28
CD47	CD47 Molecule	Protein Coding	45
PTGER2	Prostaglandin E Receptor 2	Protein Coding	49
ELF3	E74 Like ETS Transcription Factor 3	Protein Coding	40
PPY	Pancreatic Polypeptide	Protein Coding	41
RPS6KA1	Ribosomal Protein S6 Kinase A1	Protein Coding	52
USP25	Ubiquitin Specific Peptidase 25	Protein Coding	41
ABCA2	ATP Binding Cassette Subfamily A Member 2	Protein Coding	41
GYPE	Glycophorin E (MNS Blood Group)	Protein Coding	32
AMD1	Adenosylmethionine Decarboxylase 1	Protein Coding	45
SFMBT1	Scm Like With Four Mbt Domains 1	Protein Coding	37
NELL1	Neural EGFL Like 1	Protein Coding	40
AD9	Alzheimer Disease 9	Genetic Locus	3
SLC25A38	Solute Carrier Family 25 Member 38	Protein Coding	40
IL4I1	Interleukin 4 Induced 1	Protein Coding	38
ELOB	Elongin B	Protein Coding	34
ETHE1	ETHE1 Persulfide Dioxygenase	Protein Coding	43
TRIB3	Tribbles Pseudokinase 3	Protein Coding	45
HIKESHI	Heat Shock Protein Nuclear Import Factor Hikeshi	Protein Coding	33
RGS1	Regulator Of G Protein Signaling 1	Protein Coding	39
UCN	Urocortin	Protein Coding	39
TRAF3	TNF Receptor Associated Factor 3	Protein Coding	48
SYBU	Syntabulin	Protein Coding	36
COX8A	Cytochrome C Oxidase Subunit 8A	Protein Coding	41
CYP2C8	Cytochrome P450 Family 2 Subfamily C Member 8	Protein Coding	50
NEO1	Neogenin 1	Protein Coding	41
PSMB7	Proteasome 20S Subunit Beta 7	Protein Coding	47
LSS	Lanosterol Synthase	Protein Coding	46
LMAN1	Lectin, Mannose Binding 1	Protein Coding	45
RPL13	Ribosomal Protein L13	Protein Coding	42
GRM4	Glutamate Metabotropic Receptor 4	Protein Coding	46
RFX2	Regulatory Factor X2	Protein Coding	41
CDC25A	Cell Division Cycle 25A	Protein Coding	49

RPS18	Ribosomal Protein S18	Protein Coding	41
WIPI1	WD Repeat Domain, Phosphoinositide Interacting 1	Protein Coding	40
ICAM3	Intercellular Adhesion Molecule 3	Protein Coding	43
DHX30	DExH-Box Helicase 30	Protein Coding	40
LASP1	LIM And SH3 Protein 1	Protein Coding	44
COPB2	COPI Coat Complex Subunit Beta 2	Protein Coding	44
SLC25A26	Solute Carrier Family 25 Member 26	Protein Coding	39
SHMT1	Serine Hydroxymethyltransferase 1	Protein Coding	47
SERPINB1	Serpin Family B Member 1	Protein Coding	41
DIO2	Iodothyronine Deiodinase 2	Protein Coding	39
PTPRO	Protein Tyrosine Phosphatase Receptor Type O	Protein Coding	46
PTK2B	Protein Tyrosine Kinase 2 Beta	Protein Coding	50
PRIMPOL	Primase And DNA Directed Polymerase	Protein Coding	34
LGALS3B	Galectin 3 Binding Protein	Protein Coding	42
LAMC1	Laminin Subunit Gamma 1	Protein Coding	44
RORB	RAR Related Orphan Receptor B	Protein Coding	46
COMMD1	Copper Metabolism Domain Containing 1	Protein Coding	43
GCNT2	Glucosaminyl (N-Acetyl) Transferase 2 (I Blood Group	Protein Coding	45
SYN3	Synapsin III	Protein Coding	43
SREK1IP1	SREK1 Interacting Protein 1	Protein Coding	32
RCBTB1	RCC1 And BTB Domain Containing Protein 1	Protein Coding	41
FAM47E	Family With Sequence Similarity 47 Member E	Protein Coding	31
MT-TG	Mitochondrially Encoded TRNA-Gly (GGN)	RNA Gene	12
MHRT	Myosin Heavy Chain Associated RNA Transcript	RNA Gene	12
DNM3	Dynamin 3	Protein Coding	44
RPS16	Ribosomal Protein S16	Protein Coding	41
NAA15	N-Alpha-Acetyltransferase 15, NatA Auxiliary Subunit	Protein Coding	40
APBA3	Amyloid Beta Precursor Protein Binding Family A Mem	Protein Coding	40
CHRNB4	Cholinergic Receptor Nicotinic Beta 4 Subunit	Protein Coding	44
CFHR2	Complement Factor H Related 2	Protein Coding	37
VPS54	VPS54 Subunit Of GARP Complex	Protein Coding	37
MS4A2	Membrane Spanning 4-Domains A2	Protein Coding	43
KMT2B	Lysine Methyltransferase 2B	Protein Coding	40
FERMT3	Fermitin Family Member 3	Protein Coding	44
CREB3	CAMP Responsive Element Binding Protein 3	Protein Coding	39
CA9	Carbonic Anhydrase 9	Protein Coding	46
ACVR2B	Activin A Receptor Type 2B	Protein Coding	51
RBM20	RNA Binding Motif Protein 20	Protein Coding	37
HTR4	5-Hydroxytryptamine Receptor 4	Protein Coding	45
NOL3	Nucleolar Protein 3	Protein Coding	43
FTSJ1	FtsJ RNA 2'-O-Methyltransferase 1	Protein Coding	42
RNF31	Ring Finger Protein 31	Protein Coding	43
GEMIN7	Gem Nuclear Organelle Associated Protein 7	Protein Coding	36
TRAK1	Trafficking Kinesin Protein 1	Protein Coding	41
UHRF1	Ubiquitin Like With PHD And Ring Finger Domains 1	Protein Coding	41
SPPL2C	Signal Peptide Peptidase Like 2C	Protein Coding	32
FZD2	Frizzled Class Receptor 2	Protein Coding	48

ITGAV	Integrin Subunit Alpha V	Protein Coding	48
CCND3	Cyclin D3	Protein Coding	49
CEP250	Centrosomal Protein 250	Protein Coding	41
LOC10946	ATXN8 Opposite Strand (Non-Protein Coding) Repeat	Biological Region	2
CELSR2	Cadherin EGF LAG Seven-Pass G-Type Receptor 2	Protein Coding	41
VPS37A	VPS37A Subunit Of ESCRT-I	Protein Coding	42
MDK	Midkine	Protein Coding	45
PEG3	Paternally Expressed 3	Protein Coding	40
ATXN8	Ataxin 8	Protein Coding	16
PTN	Pleiotrophin	Protein Coding	44
CYS1	Cystin 1	Protein Coding	30
TRIM9	Tripartite Motif Containing 9	Protein Coding	40
SLC26A1	Solute Carrier Family 26 Member 1	Protein Coding	41
ROR1	Receptor Tyrosine Kinase Like Orphan Receptor 1	Protein Coding	47
YBX3	Y-Box Binding Protein 3	Protein Coding	40
NUMA1	Nuclear Mitotic Apparatus Protein 1	Protein Coding	45
MAPKAPI	MAPK Activated Protein Kinase 3	Protein Coding	51
ERCC6L2	ERCC Excision Repair 6 Like 2	Protein Coding	38
DAB1	DAB Adaptor Protein 1	Protein Coding	44
BCL2L13	BCL2 Like 13	Protein Coding	40
SNTA1	Syntrophin Alpha 1	Protein Coding	44
EPHA6	EPH Receptor A6	Protein Coding	43
BIRC2	Baculoviral IAP Repeat Containing 2	Protein Coding	47
AZIN2	Antizyme Inhibitor 2	Protein Coding	37
PPIA	Peptidylprolyl Isomerase A	Protein Coding	48
MSRA	Methionine Sulfoxide Reductase A	Protein Coding	43
HOMER1	Homer Scaffold Protein 1	Protein Coding	41
BOLA3	BolA Family Member 3	Protein Coding	39
CELSR1	Cadherin EGF LAG Seven-Pass G-Type Receptor 1	Protein Coding	43
UBA7	Ubiquitin Like Modifier Activating Enzyme 7	Protein Coding	43
RBM45	RNA Binding Motif Protein 45	Protein Coding	35
APBB3	Amyloid Beta Precursor Protein Binding Family B Men	Protein Coding	39
SH3KBP1	SH3 Domain Containing Kinase Binding Protein 1	Protein Coding	43
RHOT1	Ras Homolog Family Member T1	Protein Coding	43
MIR16-2	MicroRNA 16-2	RNA Gene	19
TBC1D20	TBC1 Domain Family Member 20	Protein Coding	39
ATP5F1B	ATP Synthase F1 Subunit Beta	Protein Coding	35
YY1AP1	YY1 Associated Protein 1	Protein Coding	39
PTGES	Prostaglandin E Synthase	Protein Coding	42
MT-TC	Mitochondrially Encoded TRNA-Cys (UGU/C)	RNA Gene	11
PPIB	Peptidylprolyl Isomerase B	Protein Coding	51
RALGPS2	Ral GEF With PH Domain And SH3 Binding Motif 2	Protein Coding	36
SYVN1	Synoviolin 1	Protein Coding	41
FMOD	Fibromodulin	Protein Coding	41
STAT2	Signal Transducer And Activator Of Transcription 2	Protein Coding	49
MIR24-2	MicroRNA 24-2	RNA Gene	19
CKB	Creatine Kinase B	Protein Coding	47

HIPK3	Homeodomain Interacting Protein Kinase 3	Protein Coding	44
NANS	N-Acetylneuraminate Synthase	Protein Coding	45
FBXO38	F-Box Protein 38	Protein Coding	39
DEGS2	Delta 4-Desaturase, Sphingolipid 2	Protein Coding	38
LYRM7	LYR Motif Containing 7	Protein Coding	38
STAP1	Signal Transducing Adaptor Family Member 1	Protein Coding	39
IFT81	Intraflagellar Transport 81	Protein Coding	40
H3-3A	H3.3 Histone A	Protein Coding	35
ARNT	Aryl Hydrocarbon Receptor Nuclear Translocator	Protein Coding	45
DDAH2	Dimethylarginine Dimethylaminohydrolase 2	Protein Coding	44
ATOX1	Antioxidant 1 Copper Chaperone	Protein Coding	41
C1orf112	Chromosome 1 Open Reading Frame 112	Protein Coding	33
FRRS1L	Ferric Chelate Reductase 1 Like	Protein Coding	35
RPS10	Ribosomal Protein S10	Protein Coding	45
BAMBI	BMP And Activin Membrane Bound Inhibitor	Protein Coding	44
UGT1A9	UDP Glucuronosyltransferase Family 1 Member A9	Protein Coding	44
KMO	Kynurenine 3-Monooxygenase	Protein Coding	45
KAT5	Lysine Acetyltransferase 5	Protein Coding	48
PDHB	Pyruvate Dehydrogenase E1 Subunit Beta	Protein Coding	47
SCD	Stearoyl-CoA Desaturase	Protein Coding	49
YWHAH	Tyrosine 3-Monooxygenase/Tryptophan 5-Monooxygen	Protein Coding	49
PRAME	Preferentially Expressed Antigen In Melanoma	Protein Coding	39
RACK1	Receptor For Activated C Kinase 1	Protein Coding	39
MRRF	Mitochondrial Ribosome Recycling Factor	Protein Coding	40
NR4A3	Nuclear Receptor Subfamily 4 Group A Member 3	Protein Coding	47
CDCA7	Cell Division Cycle Associated 7	Protein Coding	44
MTUS1	Microtubule Associated Scaffold Protein 1	Protein Coding	38
COA5	Cytochrome C Oxidase Assembly Factor 5	Protein Coding	37
FUT8	Fucosyltransferase 8	Protein Coding	44
PLS1	Plastin 1	Protein Coding	40
TTLL1	Tubulin Tyrosine Ligase Like 1	Protein Coding	37
SRRM4	Serine/Arginine Repetitive Matrix 4	Protein Coding	35
RIPPLY3	Ripply Transcriptional Repressor 3	Protein Coding	31
GNB5	G Protein Subunit Beta 5	Protein Coding	45
GPR161	G Protein-Coupled Receptor 161	Protein Coding	40
TM2D1	TM2 Domain Containing 1	Protein Coding	33
DUSP11	Dual Specificity Phosphatase 11	Protein Coding	35
NPAS2	Neuronal PAS Domain Protein 2	Protein Coding	42
RIPK2	Receptor Interacting Serine/Threonine Kinase 2	Protein Coding	48
FGF12	Fibroblast Growth Factor 12	Protein Coding	44
CAMK2N1	Calcium/Calmodulin Dependent Protein Kinase II Inhib	Protein Coding	34
MIR4697	MicroRNA 4697	RNA Gene	12
FCER1A	Fc Fragment Of IgE Receptor Ia	Protein Coding	44
RNF114	Ring Finger Protein 114	Protein Coding	41
SEC24C	SEC24 Homolog C, COPII Coat Complex Component	Protein Coding	45
RAB28	RAB28, Member RAS Oncogene Family	Protein Coding	41
CNP	2',3'-Cyclic Nucleotide 3' Phosphodiesterase	Protein Coding	43

NNT	Nicotinamide Nucleotide Transhydrogenase	Protein Coding	47
STX10	Syntaxin 10	Protein Coding	36
RPS3	Ribosomal Protein S3	Protein Coding	45
RPN1	Ribophorin I	Protein Coding	43
STAB1	Stabilin 1	Protein Coding	40
SERTAD3	SERTA Domain Containing 3	Protein Coding	31
MED31	Mediator Complex Subunit 31	Protein Coding	35
RCVRN	Recoverin	Protein Coding	40
ADRA2C	Adrenoceptor Alpha 2C	Protein Coding	47
MIR485	MicroRNA 485	RNA Gene	18
CNOT8	CCR4-NOT Transcription Complex Subunit 8	Protein Coding	41
COL19A1	Collagen Type XIX Alpha 1 Chain	Protein Coding	39
CCL13	C-C Motif Chemokine Ligand 13	Protein Coding	40
GLG1	Golgi Glycoprotein 1	Protein Coding	39
CAMK2A	Calcium/Calmodulin Dependent Protein Kinase II Alpha	Protein Coding	52
LPO	Lactoperoxidase	Protein Coding	40
L3MBTL3	L3MBTL Histone Methyl-Lysine Binding Protein 3	Protein Coding	37
GANC	Glucosidase Alpha, Neutral C	Protein Coding	38
CCDC115	Coiled-Coil Domain Containing 115	Protein Coding	37
SIRT6	Sirtuin 6	Protein Coding	47
COPE	COPI Coat Complex Subunit Epsilon	Protein Coding	41
LINC0177	Long Intergenic Non-Protein Coding RNA 1772	RNA Gene	12
YIF1A	Yip1 Interacting Factor Homolog A, Membrane Traffic	Protein Coding	36
PCBP4	Poly(RC) Binding Protein 4	Protein Coding	39
FRMPD4	FERM And PDZ Domain Containing 4	Protein Coding	39
CREM	CAMP Responsive Element Modulator	Protein Coding	43
SDC3	Syndecan 3	Protein Coding	43
PHF8	PHD Finger Protein 8	Protein Coding	42
SLC17A8	Solute Carrier Family 17 Member 8	Protein Coding	43
PITPNM2	Phosphatidylinositol Transfer Protein Membrane Associ	Protein Coding	36
PDHX	Pyruvate Dehydrogenase Complex Component X	Protein Coding	48
PLA2G3	Phospholipase A2 Group III	Protein Coding	39
CERS1	Ceramide Synthase 1	Protein Coding	43
PMVK	Phosphomevalonate Kinase	Protein Coding	47
SPG19	Spastic Paraplegia 19 (Autosomal Dominant)	Genetic Locus	5
TEC	Tec Protein Tyrosine Kinase	Protein Coding	47
FHL5	Four And A Half LIM Domains 5	Protein Coding	37
MOB2	MOB Kinase Activator 2	Protein Coding	35
RAB25	RAB25, Member RAS Oncogene Family	Protein Coding	40
CEP57	Centrosomal Protein 57	Protein Coding	42
FABP12	Fatty Acid Binding Protein 12	Protein Coding	31
LFNG	LFNG O-Fucosylpeptide 3-Beta-N-Acetylglucosaminyl	Protein Coding	48
RNF112	Ring Finger Protein 112	Protein Coding	35
KYNU	Kynureninase	Protein Coding	47
CFAP298	Cilia And Flagella Associated Protein 298	Protein Coding	32
HSPA2	Heat Shock Protein Family A (Hsp70) Member 2	Protein Coding	45
MED7	Mediator Complex Subunit 7	Protein Coding	37

SPG29	Spastic Paraplegia 29 (Autosomal Dominant)	Genetic Locus	5
GLO1	Glyoxalase I	Protein Coding	47
TARS1	Threonyl-TRNA Synthetase 1	Protein Coding	36
HAR1B	Highly Accelerated Region 1B	RNA Gene	18
AOX1	Aldehyde Oxidase 1	Protein Coding	45
CEACAM(	CEA Cell Adhesion Molecule 6	Protein Coding	41
CALML5	Calmodulin Like 5	Protein Coding	39
MEIS2	Meis Homeobox 2	Protein Coding	43
C14orf178	Chromosome 14 Open Reading Frame 178	RNA Gene	28
HAR1A	Highly Accelerated Region 1A	RNA Gene	21
SLC25A37	Solute Carrier Family 25 Member 37	Protein Coding	39
KHDRBS3	KH RNA Binding Domain Containing, Signal Transduc	Protein Coding	39
TIMM23B	Translocase Of Inner Mitochondrial Membrane 23 Hom	Protein Coding	25
SSB	Small RNA Binding Exonuclease Protection Factor La	Protein Coding	44
SNAP91	Synaptosome Associated Protein 91	Protein Coding	41
RPL15	Ribosomal Protein L15	Protein Coding	45
SFN	Stratifin	Protein Coding	48
ELAVL4	ELAV Like RNA Binding Protein 4	Protein Coding	41
IFNA17	Interferon Alpha 17	Protein Coding	35
RSPH3	Radial Spoke Head 3	Protein Coding	37
ATP5MG	ATP Synthase Membrane Subunit G	Protein Coding	31
DHX16	DEAH-Box Helicase 16	Protein Coding	41
MCTP2	Multiple C2 And Transmembrane Domain Containing 2	Protein Coding	40
BCAP31	B Cell Receptor Associated Protein 31	Protein Coding	42
CD99	CD99 Molecule (Xg Blood Group)	Protein Coding	41
ENSG00000202498		RNA Gene	8
SERPINB2	Serpin Family B Member 2	Protein Coding	45
APOBEC3	Apolipoprotein B MRNA Editing Enzyme Catalytic Sul	Protein Coding	43
CENPB	Centromere Protein B	Protein Coding	39
WEE1	WEE1 G2 Checkpoint Kinase	Protein Coding	48
PIK3R5	Phosphoinositide-3-Kinase Regulatory Subunit 5	Protein Coding	46
FIBP	FGF1 Intracellular Binding Protein	Protein Coding	43
VEGFD	Vascular Endothelial Growth Factor D	Protein Coding	36
GNAI1	G Protein Subunit Alpha I1	Protein Coding	47
RBP1	Retinol Binding Protein 1	Protein Coding	40
ECSIT	ECSIT Signaling Integrator	Protein Coding	40
INSM1	INSM Transcriptional Repressor 1	Protein Coding	37
CGB3	Chorionic Gonadotropin Subunit Beta 3	Protein Coding	32
PANK3	Pantothenate Kinase 3	Protein Coding	40
PPHLN1	Periphilin 1	Protein Coding	38
KCNJ3	Potassium Inwardly Rectifying Channel Subfamily J Me	Protein Coding	46
RNASE13	Ribonuclease A Family Member 13 (Inactive)	Protein Coding	31
NOS1AP	Nitric Oxide Synthase 1 Adaptor Protein	Protein Coding	39
RBM28	RNA Binding Motif Protein 28	Protein Coding	40
TADA2B	Transcriptional Adaptor 2B	Protein Coding	37
RRAGB	Ras Related GTP Binding B	Protein Coding	39
PRPF4	Pre-MRNA Processing Factor 4	Protein Coding	43

SRL	Sarcalumenin	Protein Coding	35
HAS2	Hyaluronan Synthase 2	Protein Coding	41
PLXNA1	Plexin A1	Protein Coding	43
MFGE8	Milk Fat Globule-EGF Factor 8 Protein	Protein Coding	45
UBA52	Ubiquitin A-52 Residue Ribosomal Protein Fusion Prod	Protein Coding	43
PM20D1	Peptidase M20 Domain Containing 1	Protein Coding	39
MIR191	MicroRNA 191	RNA Gene	21
AFF4	AF4/FMR2 Family Member 4	Protein Coding	43
H3-3B	H3.3 Histone B	Protein Coding	34
KLC2	Kinesin Light Chain 2	Protein Coding	42
PAK4	P21 (RAC1) Activated Kinase 4	Protein Coding	50
SNRPA	Small Nuclear Ribonucleoprotein Polypeptide A	Protein Coding	41
UBE2K	Ubiquitin Conjugating Enzyme E2 K	Protein Coding	43
PLS3	Plastin 3	Protein Coding	42
MIR133A1	MicroRNA 133a-1	RNA Gene	18
RPL10A	Ribosomal Protein L10a	Protein Coding	43
IGFBP4	Insulin Like Growth Factor Binding Protein 4	Protein Coding	44
CFAP300	Cilia And Flagella Associated Protein 300	Protein Coding	27
PYCR2	Pyrroline-5-Carboxylate Reductase 2	Protein Coding	45
RPL6	Ribosomal Protein L6	Protein Coding	40
PRSS3P2	PRSS3 Pseudogene 2	Pseudogene	17
KEL	Kell Metallo-Endopeptidase (Kell Blood Group)	Protein Coding	43
PLD2	Phospholipase D2	Protein Coding	49
SPG37	Spastic Paraplegia 37 (Autosomal Dominant)	Genetic Locus	4
ZNF483	Zinc Finger Protein 483	Protein Coding	36
LAP3	Leucine Aminopeptidase 3	Protein Coding	44
CSNK2A2	Casein Kinase 2 Alpha 2	Protein Coding	48
HNRNPM	Heterogeneous Nuclear Ribonucleoprotein M	Protein Coding	41
ZNF470	Zinc Finger Protein 470	Protein Coding	33
TOR1AIP2	Torsin 1A Interacting Protein 2	Protein Coding	35
ASAH2B	N-Acylsphingosine Amidohydrolase 2B	Protein Coding	28
SRSF1	Serine And Arginine Rich Splicing Factor 1	Protein Coding	43
TEF	TEF Transcription Factor, PAR BZIP Family Member	Protein Coding	40
RPS23	Ribosomal Protein S23	Protein Coding	41
DDB1	Damage Specific DNA Binding Protein 1	Protein Coding	43
ITGB6	Integrin Subunit Beta 6	Protein Coding	47
SELENOP	Selenoprotein P	Protein Coding	31
BNIP3	BCL2 Interacting Protein 3	Protein Coding	44
HLA-DMB	Major Histocompatibility Complex, Class II, DM Beta	Protein Coding	43
KIF3A	Kinesin Family Member 3A	Protein Coding	42
COPZ1	COPI Coat Complex Subunit Zeta 1	Protein Coding	41
XIST	X Inactive Specific Transcript	RNA Gene	24
RASA1	RAS P21 Protein Activator 1	Protein Coding	48
BID	BH3 Interacting Domain Death Agonist	Protein Coding	47
PIBF1	Progesterone Immunomodulatory Binding Factor 1	Protein Coding	41
RAB1B	RAB1B, Member RAS Oncogene Family	Protein Coding	43
FZD5	Frizzled Class Receptor 5	Protein Coding	48

SLC2A13	Solute Carrier Family 2 Member 13	Protein Coding	40
AASS	Aminoadipate-Semialdehyde Synthase	Protein Coding	44
ZNF569	Zinc Finger Protein 569	Protein Coding	35
PLOD3	Procollagen-Lysine,2-Oxoglutarate 5-Dioxygenase 3	Protein Coding	46
MTX1	Metaxin 1	Protein Coding	40
YWHAB	Tyrosine 3-Monooxygenase/Tryptophan 5-Monooxygen	Protein Coding	50
SPG41	Spastic Paraplegia 41 (Autosomal Dominant)	Genetic Locus	6
SPG38	Spastic Paraplegia 38 (Autosomal Dominant, Silver Syı	Genetic Locus	4
FAM47E-S	FAM47E-STBD1 Readthrough	Protein Coding	20
HLA-DOA	Major Histocompatibility Complex, Class II, DO Alpha	Protein Coding	42
PMEL	Premelanosome Protein	Protein Coding	39
SFXN4	Sideroflexin 4	Protein Coding	41
UBIAD1	UbiA Prenyltransferase Domain Containing 1	Protein Coding	42
MIR370	MicroRNA 370	RNA Gene	21
MSRB1	Methionine Sulfoxide Reductase B1	Protein Coding	41
PAK3	P21 (RAC1) Activated Kinase 3	Protein Coding	51
SLC1A4	Solute Carrier Family 1 Member 4	Protein Coding	45
RAB11B	RAB11B, Member RAS Oncogene Family	Protein Coding	43
MAT2A	Methionine Adenosyltransferase 2A	Protein Coding	48
MAGT1	Magnesium Transporter 1	Protein Coding	40
KLHL32	Kelch Like Family Member 32	Protein Coding	33
CARNMT	Carnosine N-Methyltransferase 1	Protein Coding	29
UBE2D3	Ubiquitin Conjugating Enzyme E2 D3	Protein Coding	45
ADORA3	Adenosine A3 Receptor	Protein Coding	47
FHL2	Four And A Half LIM Domains 2	Protein Coding	47
PRDX6	Peroxiredoxin 6	Protein Coding	48
MIR503	MicroRNA 503	RNA Gene	17
PLXNC1	Plexin C1	Protein Coding	40
TDP2	Tyrosyl-DNA Phosphodiesterase 2	Protein Coding	41
FOXA2	Forkhead Box A2	Protein Coding	45
GPD1L	Glycerol-3-Phosphate Dehydrogenase 1 Like	Protein Coding	44
NEK2	NIMA Related Kinase 2	Protein Coding	52
PRDM1	PR/SET Domain 1	Protein Coding	47
PIM1	Pim-1 Proto-Oncogene, Serine/Threonine Kinase	Protein Coding	52
IRS4	Insulin Receptor Substrate 4	Protein Coding	39
HCK	HCK Proto-Oncogene, Src Family Tyrosine Kinase	Protein Coding	51
SPHK1	Sphingosine Kinase 1	Protein Coding	48
NRIP1	Nuclear Receptor Interacting Protein 1	Protein Coding	44
CRADD	CASP2 And RIPK1 Domain Containing Adaptor With	Protein Coding	45
NTNG1	Netrin G1	Protein Coding	41
UNC80	Unc-80 Homolog, NALCN Channel Complex Subunit	Protein Coding	34
TUT1	Terminal Uridylyl Transferase 1, U6 SnRNA-Specific	Protein Coding	37
MIR409	MicroRNA 409	RNA Gene	19
TIMM17A	Translocase Of Inner Mitochondrial Membrane 17A	Protein Coding	41
ELAVL2	ELAV Like RNA Binding Protein 2	Protein Coding	43
RPL28	Ribosomal Protein L28	Protein Coding	41
TXNIP	Thioredoxin Interacting Protein	Protein Coding	39

HPR	Haptoglobin-Related Protein	Protein Coding	40
ANAPC2	Anaphase Promoting Complex Subunit 2	Protein Coding	42
MAPKAPI	MAPK Activated Protein Kinase 2	Protein Coding	50
B3GAT1	Beta-1,3-Glucuronyltransferase 1	Protein Coding	44
MANF	Mesencephalic Astrocyte Derived Neurotrophic Factor	Protein Coding	41
AP2M1	Adaptor Related Protein Complex 2 Subunit Mu 1	Protein Coding	45
CIC	Capicua Transcriptional Repressor	Protein Coding	43
GNAI2	G Protein Subunit Alpha I2	Protein Coding	48
CCNQ	Cyclin Q	Protein Coding	28
SNHG3	Small Nucleolar RNA Host Gene 3	RNA Gene	19
SLIT2	Slit Guidance Ligand 2	Protein Coding	47
CSNK1E	Casein Kinase 1 Epsilon	Protein Coding	49
FUZ	Fuzzy Planar Cell Polarity Protein	Protein Coding	39
RPS27L	Ribosomal Protein S27 Like	Protein Coding	39
CD200	CD200 Molecule	Protein Coding	41
STK32B	Serine/Threonine Kinase 32B	Protein Coding	35
UNC13B	Unc-13 Homolog B	Protein Coding	41
RAD51B	RAD51 Paralog B	Protein Coding	39
DNAJA1	DnaJ Heat Shock Protein Family (Hsp40) Member A1	Protein Coding	43
PAPSS2	3'-Phosphoadenosine 5'-Phosphosulfate Synthase 2	Protein Coding	45
LARP7	La Ribonucleoprotein 7, Transcriptional Regulator	Protein Coding	41
MT2A	Metallothionein 2A	Protein Coding	43
CXCL16	C-X-C Motif Chemokine Ligand 16	Protein Coding	41
GPT2	Glutamic--Pyruvic Transaminase 2	Protein Coding	47
SYNPO	Synaptopodin	Protein Coding	40
HNRNPF	Heterogeneous Nuclear Ribonucleoprotein F	Protein Coding	41
RPS13	Ribosomal Protein S13	Protein Coding	44
AK2	Adenylate Kinase 2	Protein Coding	50
MACROD	Mono-ADP Ribosylhydrolase 2	Protein Coding	36
CTPS1	CTP Synthase 1	Protein Coding	46
NDUFA3	NADH:Ubiquinone Oxidoreductase Subunit A3	Protein Coding	40
PIGY	Phosphatidylinositol Glycan Anchor Biosynthesis Class	Protein Coding	37
KCNA4	Potassium Voltage-Gated Channel Subfamily A Membe	Protein Coding	42
ADAMTS!	ADAM Metallopeptidase With Thrombospondin Type 1	Protein Coding	39
EOGT	EGF Domain Specific O-Linked N-Acetylglucosamine	Protein Coding	38
RBM10	RNA Binding Motif Protein 10	Protein Coding	41
PYROXD1	Pyridine Nucleotide-Disulphide Oxidoreductase Domair	Protein Coding	37
MRPS2	Mitochondrial Ribosomal Protein S2	Protein Coding	39
ACKR2	Atypical Chemokine Receptor 2	Protein Coding	39
KCNE5	Potassium Voltage-Gated Channel Subfamily E Regulat	Protein Coding	32
NEGR1	Neuronal Growth Regulator 1	Protein Coding	40
CPQ	Carboxypeptidase Q	Protein Coding	35
MAP2K6	Mitogen-Activated Protein Kinase Kinase 6	Protein Coding	48
PAEP	Progestagen Associated Endometrial Protein	Protein Coding	40
CLUAP1	Clusterin Associated Protein 1	Protein Coding	37
IGHG1	Immunoglobulin Heavy Constant Gamma 1 (G1m Mark	Protein Coding	35
STX3	Syntaxin 3	Protein Coding	43

RTL10	Retrotransposon Gag Like 10	Protein Coding	24
SRI	Sorcin	Protein Coding	44
DHX9	DExH-Box Helicase 9	Protein Coding	41
PARS2	Prolyl-TRNA Synthetase 2, Mitochondrial	Protein Coding	43
TACR2	Tachykinin Receptor 2	Protein Coding	44
LGMN	Legumain	Protein Coding	44
SLC22A2	Solute Carrier Family 22 Member 2	Protein Coding	45
LGALS2	Galectin 2	Protein Coding	42
RPL14	Ribosomal Protein L14	Protein Coding	40
OMA1	OMA1 Zinc Metallopeptidase	Protein Coding	38
FOXA1	Forkhead Box A1	Protein Coding	44
EEF1D	Eukaryotic Translation Elongation Factor 1 Delta	Protein Coding	43
GRK6	G Protein-Coupled Receptor Kinase 6	Protein Coding	47
ACSS1	Acyl-CoA Synthetase Short Chain Family Member 1	Protein Coding	40
PRDX3	Peroxiredoxin 3	Protein Coding	44
PSRC1	Proline And Serine Rich Coiled-Coil 1	Protein Coding	39
GNLY	Granulysin	Protein Coding	40
PBRM1	Polybromo 1	Protein Coding	45
KLK11	Kallikrein Related Peptidase 11	Protein Coding	42
FAAH	Fatty Acid Amide Hydrolase	Protein Coding	48
LINC0221	Long Intergenic Non-Protein Coding RNA 2210	RNA Gene	16
MCCC2	Methylcrotonoyl-CoA Carboxylase 2	Protein Coding	45
GSDMB	Gasdermin B	Protein Coding	37
MRPS7	Mitochondrial Ribosomal Protein S7	Protein Coding	40
SMOC2	SPARC Related Modular Calcium Binding 2	Protein Coding	43
MIR181A2	MicroRNA 181a-2	RNA Gene	20
CLP1	Cleavage Factor Polyribonucleotide Kinase Subunit 1	Protein Coding	41
GSTZ1	Glutathione S-Transferase Zeta 1	Protein Coding	43
SNORD97	Small Nucleolar RNA, C/D Box 97	RNA Gene	15
RFWD3	Ring Finger And WD Repeat Domain 3	Protein Coding	41
AGPS	Alkylglycerone Phosphate Synthase	Protein Coding	44
QKI	QKI, KH Domain Containing RNA Binding	Protein Coding	44
CXADR	CXADR Ig-Like Cell Adhesion Molecule	Protein Coding	43
HDAC3	Histone Deacetylase 3	Protein Coding	51
NDN	Necdin, MAGE Family Member	Protein Coding	41
FAM136A	Family With Sequence Similarity 136 Member A	Protein Coding	38
MPG	N-Methylpurine DNA Glycosylase	Protein Coding	43
ABR	ABR Activator Of RhoGEF And GTPase	Protein Coding	41
RPL30	Ribosomal Protein L30	Protein Coding	41
MTA1	Metastasis Associated 1	Protein Coding	44
IFI16	Interferon Gamma Inducible Protein 16	Protein Coding	44
NISCH	Nischarin	Protein Coding	41
RPL23	Ribosomal Protein L23	Protein Coding	40
CRKL	CRK Like Proto-Oncogene, Adaptor Protein	Protein Coding	48
TRAPPC1	Trafficking Protein Particle Complex 12	Protein Coding	37
CDK10	Cyclin Dependent Kinase 10	Protein Coding	44
ATAT1	Alpha Tubulin Acetyltransferase 1	Protein Coding	35

KHDRBS1	KH RNA Binding Domain Containing, Signal Transduc	Protein Coding	42
LIMS2	LIM Zinc Finger Domain Containing 2	Protein Coding	41
LOC11136	NOS2 5' Regulatory Region	Biological Region	2
LETM1	Leucine Zipper And EF-Hand Containing Transmembra	Protein Coding	43
MXI1	MAX Interactor 1, Dimerization Protein	Protein Coding	43
BAG1	BAG Cochaperone 1	Protein Coding	44
GABRA5	Gamma-Aminobutyric Acid Type A Receptor Subunit A	Protein Coding	47
GABRR2	Gamma-Aminobutyric Acid Type A Receptor Subunit F	Protein Coding	41
MXD3	MAX Dimerization Protein 3	Protein Coding	35
NEURL1	Neuralized E3 Ubiquitin Protein Ligase 1	Protein Coding	37
TUBGCP2	Tubulin Gamma Complex Associated Protein 2	Protein Coding	38
PGAM1	Phosphoglycerate Mutase 1	Protein Coding	44
TUBE1	Tubulin Epsilon 1	Protein Coding	38
SLC17A6	Solute Carrier Family 17 Member 6	Protein Coding	41
ZFAND2B	Zinc Finger AN1-Type Containing 2B	Protein Coding	36
GRM3	Glutamate Metabotropic Receptor 3	Protein Coding	47
CDC42SE	CDC42 Small Effector 1	Protein Coding	35
PRKCE	Protein Kinase C Epsilon	Protein Coding	51
SFXN2	Sideroflexin 2	Protein Coding	39
DAAM1	Dishevelled Associated Activator Of Morphogenesis 1	Protein Coding	42
MADD	MAP Kinase Activating Death Domain	Protein Coding	42
EMC1	ER Membrane Protein Complex Subunit 1	Protein Coding	35
PFKL	Phosphofructokinase, Liver Type	Protein Coding	45
LHX8	LIM Homeobox 8	Protein Coding	37
WASF2	WASP Family Member 2	Protein Coding	44
OTX1	Orthodenticle Homeobox 1	Protein Coding	43
GABBR1	Gamma-Aminobutyric Acid Type B Receptor Subunit 1	Protein Coding	48
RAB1A	RAB1A, Member RAS Oncogene Family	Protein Coding	42
RUVBL1	RuvB Like AAA ATPase 1	Protein Coding	45
MMP20	Matrix Metallopeptidase 20	Protein Coding	45
INPP5F	Inositol Polyphosphate-5-Phosphatase F	Protein Coding	39
KCTD11	Potassium Channel Tetramerization Domain Containing	Protein Coding	34
DKK4	Dickkopf WNT Signaling Pathway Inhibitor 4	Protein Coding	39
FGF16	Fibroblast Growth Factor 16	Protein Coding	40
IL1RL2	Interleukin 1 Receptor Like 2	Protein Coding	43
CLRN3	Clarin 3	Protein Coding	33
DECR1	2,4-Dienoyl-CoA Reductase 1	Protein Coding	42
MIR511	MicroRNA 511	RNA Gene	16
RCC1	Regulator Of Chromosome Condensation 1	Protein Coding	41
RPS9	Ribosomal Protein S9	Protein Coding	43
HERPUD1	Homocysteine Inducible ER Protein With Ubiquitin Like	Protein Coding	39
FOXI1	Forkhead Box I1	Protein Coding	39
RPS25	Ribosomal Protein S25	Protein Coding	39
MIR27B	MicroRNA 27b	RNA Gene	21
TRIM63	Tripartite Motif Containing 63	Protein Coding	41
LRP1-AS	LRP1 Antisense RNA	RNA Gene	12
DKK2	Dickkopf WNT Signaling Pathway Inhibitor 2	Protein Coding	45

MELK	Maternal Embryonic Leucine Zipper Kinase	Protein Coding	46
EN1	Engrailed Homeobox 1	Protein Coding	37
PSMD6	Proteasome 26S Subunit, Non-ATPase 6	Protein Coding	39
COL14A1	Collagen Type XIV Alpha 1 Chain	Protein Coding	43
PRMT1	Protein Arginine Methyltransferase 1	Protein Coding	50
PTPN12	Protein Tyrosine Phosphatase Non-Receptor Type 12	Protein Coding	46
TXN2	Thioredoxin 2	Protein Coding	47
HRH4	Histamine Receptor H4	Protein Coding	44
LINC0108	Long Intergenic Non-Protein Coding RNA 1080	RNA Gene	14
TOLLIP	Toll Interacting Protein	Protein Coding	45
CAMK1D	Calcium/Calmodulin Dependent Protein Kinase ID	Protein Coding	43
BDNF-AS	BDNF Antisense RNA	RNA Gene	20
EPM2AIP	EPM2A Interacting Protein 1	Protein Coding	36
CA12	Carbonic Anhydrase 12	Protein Coding	48
LIG3	DNA Ligase 3	Protein Coding	45
HGS	Hepatocyte Growth Factor-Regulated Tyrosine Kinase S	Protein Coding	45
PTPN6	Protein Tyrosine Phosphatase Non-Receptor Type 6	Protein Coding	51
GATAD2E	GATA Zinc Finger Domain Containing 2B	Protein Coding	43
EMG1	EMG1 N1-Specific Pseudouridine Methyltransferase	Protein Coding	41
HTR2B	5-Hydroxytryptamine Receptor 2B	Protein Coding	44
CNOT1	CCR4-NOT Transcription Complex Subunit 1	Protein Coding	40
BAG6	BAG Cochaperone 6	Protein Coding	37
THY1	Thy-1 Cell Surface Antigen	Protein Coding	45
MEOX1	Mesenchyme Homeobox 1	Protein Coding	43
PPT2	Palmitoyl-Protein Thioesterase 2	Protein Coding	39
CSNK1A1	Casein Kinase 1 Alpha 1	Protein Coding	53
CPT1C	Carnitine Palmitoyltransferase 1C	Protein Coding	43
HCP5	HLA Complex P5	RNA Gene	32
FKBP4	FKBP Prolyl Isomerase 4	Protein Coding	46
PEX11A	Peroxisomal Biogenesis Factor 11 Alpha	Protein Coding	37
NELFA	Negative Elongation Factor Complex Member A	Protein Coding	37
NDUFC2	NADH:Ubiquinone Oxidoreductase Subunit C2	Protein Coding	40
NECTIN2	Nectin Cell Adhesion Molecule 2	Protein Coding	38
SLC45A3	Solute Carrier Family 45 Member 3	Protein Coding	40
DDX21	DExD-Box Helicase 21	Protein Coding	39
CACNA1I	Calcium Voltage-Gated Channel Subunit Alpha1 I	Protein Coding	45
TLL1	Tolloid Like 1	Protein Coding	44
AFDN	Afadin, Adherens Junction Formation Factor	Protein Coding	35
FNDC5	Fibronectin Type III Domain Containing 5	Protein Coding	35
CNDP1	Carnosine Dipeptidase 1	Protein Coding	45
SLC7A1	Solute Carrier Family 7 Member 1	Protein Coding	45
HNRNPR	Heterogeneous Nuclear Ribonucleoprotein R	Protein Coding	40
EXOSC9	Exosome Component 9	Protein Coding	40
WNT11	Wnt Family Member 11	Protein Coding	45
SRSF3	Serine And Arginine Rich Splicing Factor 3	Protein Coding	41
NDUFB1	NADH:Ubiquinone Oxidoreductase Subunit B1	Protein Coding	39
DYT17	Dystonia 17	Genetic Locus	5

SNIP1	Smad Nuclear Interacting Protein 1	Protein Coding	40
NECAP1	NECAP Endocytosis Associated 1	Protein Coding	40
LINC0161	Long Intergenic Non-Protein Coding RNA 1616	RNA Gene	10
TCIM	Transcriptional And Immune Response Regulator	Protein Coding	26
ADD2	Adducin 2	Protein Coding	42
MIR30C1	MicroRNA 30c-1	RNA Gene	22
IGFBP6	Insulin Like Growth Factor Binding Protein 6	Protein Coding	44
LOC10866	Protein Phosphatase 2 Regulatory Subunit Bbeta Repea	Biological Region	2
MIR136	MicroRNA 136	RNA Gene	20
PHB2	Prohibitin 2	Protein Coding	42
ICAM2	Intercellular Adhesion Molecule 2	Protein Coding	47
MARK2	Microtubule Affinity Regulating Kinase 2	Protein Coding	44
BCAR1	BCAR1 Scaffold Protein, Cas Family Member	Protein Coding	45
HLA-DOB	Major Histocompatibility Complex, Class II, DO Beta	Protein Coding	42
SEPTIN2	Septin 2	Protein Coding	34
CTRL	Chymotrypsin Like	Protein Coding	41
LOC10902	Cystatin B Upstream Repeat Instability Region	Biological Region	2
HAAO	3-Hydroxyanthranilate 3,4-Dioxygenase	Protein Coding	44
TEFM	Transcription Elongation Factor, Mitochondrial	Protein Coding	35
CHP2	Calcineurin Like EF-Hand Protein 2	Protein Coding	35
ERGIC1	Endoplasmic Reticulum-Golgi Intermediate Compartme	Protein Coding	40
SPPL2B	Signal Peptide Peptidase Like 2B	Protein Coding	34
QARS1	Glutaminyl-TRNA Synthetase 1	Protein Coding	25
HMMR	Hyaluronan Mediated Motility Receptor	Protein Coding	44
ZDHHC8	Zinc Finger DHHC-Type Palmitoyltransferase 8	Protein Coding	40
RPS12	Ribosomal Protein S12	Protein Coding	43
AAK1	AP2 Associated Kinase 1	Protein Coding	43
PSMD12	Proteasome 26S Subunit, Non-ATPase 12	Protein Coding	43
HNRNPA3	Heterogeneous Nuclear Ribonucleoprotein A3	Protein Coding	38
GABPB2	GA Binding Protein Transcription Factor Subunit Beta	Protein Coding	37
SEMG1	Semenogelin 1	Protein Coding	37
CHP1	Calcineurin Like EF-Hand Protein 1	Protein Coding	39
FMO3	Flavin Containing Dimethylaniline Monoxygenase 3	Protein Coding	48
LALBA	Lactalbumin Alpha	Protein Coding	40
ELK1	ETS Transcription Factor ELK1	Protein Coding	45
SYT4	Synaptotagmin 4	Protein Coding	38
RABEP1	Rabaptin, RAB GTPase Binding Effector Protein 1	Protein Coding	40
SERBP1	SERPINE1 MRNA Binding Protein 1	Protein Coding	36
CLPP	Caseinolytic Mitochondrial Matrix Peptidase Proteolyti	Protein Coding	45
SEL1L	SEL1L Adaptor Subunit Of ERAD E3 Ubiquitin Ligas	Protein Coding	43
TMEM59	Transmembrane Protein 59	Protein Coding	40
RPL3	Ribosomal Protein L3	Protein Coding	43
AP5B1	Adaptor Related Protein Complex 5 Subunit Beta 1	Protein Coding	32
ATG2A	Autophagy Related 2A	Protein Coding	37
RAB8B	RAB8B, Member RAS Oncogene Family	Protein Coding	41
NEURL4	Neuralized E3 Ubiquitin Protein Ligase 4	Protein Coding	33
KLK1	Kallikrein 1	Protein Coding	45

PRR5	Proline Rich 5	Protein Coding	40
HMGCS2	3-Hydroxy-3-Methylglutaryl-CoA Synthase 2	Protein Coding	45
FYCO1	FYVE And Coiled-Coil Domain Autophagy Adaptor 1	Protein Coding	40
MIR494	MicroRNA 494	RNA Gene	17
LOC10192	Uncharacterized LOC101928174	RNA Gene	10
IDH3B	Isocitrate Dehydrogenase (NAD(+)) 3 Non-Catalytic Su	Protein Coding	46
PPA2	Inorganic Pyrophosphatase 2	Protein Coding	41
SRCIN1	SRC Kinase Signaling Inhibitor 1	Protein Coding	36
NDUFB5	NADH:Ubiquinone Oxidoreductase Subunit B5	Protein Coding	37
ARCN1	Archain 1	Protein Coding	41
MIR18B	MicroRNA 18b	RNA Gene	16
POLB	DNA Polymerase Beta	Protein Coding	48
POGK	Pogo Transposable Element Derived With KRAB Doma	Protein Coding	37
TPM4	Tropomyosin 4	Protein Coding	43
LUM	Lumican	Protein Coding	43
SFRP2	Secreted Frizzled Related Protein 2	Protein Coding	44
CCT4	Chaperonin Containing TCP1 Subunit 4	Protein Coding	41
RPL4	Ribosomal Protein L4	Protein Coding	43
LAG3	Lymphocyte Activating 3	Protein Coding	39
ATIC	5-Aminoimidazole-4-Carboxamide Ribonucleotide Forn	Protein Coding	47
ENPEP	Glutamyl Aminopeptidase	Protein Coding	44
SMAD1	SMAD Family Member 1	Protein Coding	45
MIR26A2	MicroRNA 26a-2	RNA Gene	21
ATP6V0A	ATPase H+ Transporting V0 Subunit A1	Protein Coding	43
LSM14A	LSM14A MRNA Processing Body Assembly Factor	Protein Coding	36
TRAIP	TRAF Interacting Protein	Protein Coding	41
SETD1A	SET Domain Containing 1A, Histone Lysine Methyltrai	Protein Coding	41
GCSH	Glycine Cleavage System Protein H	Protein Coding	44
GRIK3	Glutamate Ionotropic Receptor Kainate Type Subunit 3	Protein Coding	43
POLH	DNA Polymerase Eta	Protein Coding	48
SENP3	SUMO Specific Peptidase 3	Protein Coding	40
GABRG3	Gamma-Aminobutyric Acid Type A Receptor Subunit (	Protein Coding	42
KRT15	Keratin 15	Protein Coding	41
CNKSR3	CNKSR Family Member 3	Protein Coding	36
SELENOS	Selenoprotein S	Protein Coding	32
FZD3	Frizzled Class Receptor 3	Protein Coding	45
PDE4B	Phosphodiesterase 4B	Protein Coding	45
RAP1B	RAP1B, Member Of RAS Oncogene Family	Protein Coding	47
GOLGA2	Golgin A2	Protein Coding	42
CLEC5A	C-Type Lectin Domain Containing 5A	Protein Coding	35
ETFRF1	Electron Transfer Flavoprotein Regulatory Factor 1	Protein Coding	27
CEBPB	CCAAT Enhancer Binding Protein Beta	Protein Coding	45
CTF1	Cardiotrophin 1	Protein Coding	41
AP2A1	Adaptor Related Protein Complex 2 Subunit Alpha 1	Protein Coding	40
IQGAP1	IQ Motif Containing GTPase Activating Protein 1	Protein Coding	45
WIF1	WNT Inhibitory Factor 1	Protein Coding	46
EPHB1	EPH Receptor B1	Protein Coding	48

TEAD2	TEA Domain Transcription Factor 2	Protein Coding	40
ZNF804A	Zinc Finger Protein 804A	Protein Coding	35
MIR1224	MicroRNA 1224	RNA Gene	19
MS4A4A	Membrane Spanning 4-Domains A4A	Protein Coding	35
ATL3	Atlastin GTPase 3	Protein Coding	39
MAB21L2	Mab-21 Like 2	Protein Coding	40
RPL26L1	Ribosomal Protein L26 Like 1	Protein Coding	39
GYG2	Glycogenin 2	Protein Coding	41
RPLP2	Ribosomal Protein Lateral Stalk Subunit P2	Protein Coding	42
PAM16	Presequence Translocase Associated Motor 16	Protein Coding	37
RRAGC	Ras Related GTP Binding C	Protein Coding	41
GRHPR	Glyoxylate And Hydroxypyruvate Reductase	Protein Coding	46
KCNIP4	Potassium Voltage-Gated Channel Interacting Protein 4	Protein Coding	39
NDUFB4	NADH:Ubiquinone Oxidoreductase Subunit B4	Protein Coding	40
NDUFA7	NADH:Ubiquinone Oxidoreductase Subunit A7	Protein Coding	39
SHMT2	Serine Hydroxymethyltransferase 2	Protein Coding	45
IL34	Interleukin 34	Protein Coding	40
CDIP1	Cell Death Inducing P53 Target 1	Protein Coding	36
LRCH3	Leucine Rich Repeats And Calponin Homology Domain	Protein Coding	35
TNFRSF1	TNF Receptor Superfamily Member 14	Protein Coding	45
TUBG2	Tubulin Gamma 2	Protein Coding	38
RPL8	Ribosomal Protein L8	Protein Coding	42
MRPL3	Mitochondrial Ribosomal Protein L3	Protein Coding	42
TMOD1	Tropomodulin 1	Protein Coding	39
PABPC1	Poly(A) Binding Protein Cytoplasmic 1	Protein Coding	43
TRIM25	Tripartite Motif Containing 25	Protein Coding	46
STOML2	Stomatin Like 2	Protein Coding	39
ATP6V1B	ATPase H+ Transporting V1 Subunit B2	Protein Coding	47
OSR2	Odd-Skipped Related Transciption Factor 2	Protein Coding	39
ASRGL1	Asparaginase And Isoaspartyl Peptidase 1	Protein Coding	44
PCDH10	Protocadherin 10	Protein Coding	40
DALRD3	DALR Anticodon Binding Domain Containing 3	Protein Coding	35
MYCBP2	MYC Binding Protein 2	Protein Coding	39
FMR1-AS	FMR1 Antisense RNA 1	RNA Gene	17
PLEKHA1	Pleckstrin Homology Domain Containing A1	Protein Coding	41
HTR7	5-Hydroxytryptamine Receptor 7	Protein Coding	47
HHAT	Hedgehog Acyltransferase	Protein Coding	41
SLC50A1	Solute Carrier Family 50 Member 1	Protein Coding	36
SCG5	Secretogranin V	Protein Coding	39
BORCS7	BLOC-1 Related Complex Subunit 7	Protein Coding	26
SCN7A	Sodium Voltage-Gated Channel Alpha Subunit 7	Protein Coding	41
SLC25A2	Solute Carrier Family 25 Member 28	Protein Coding	39
CDC5L	Cell Division Cycle 5 Like	Protein Coding	40
SERPIND	Serpin Family D Member 1	Protein Coding	47
PRKCI	Protein Kinase C Iota	Protein Coding	50
SFXN1	Sideroflexin 1	Protein Coding	40
MAP4K4	Mitogen-Activated Protein Kinase Kinase Kinase Kinas	Protein Coding	48

PLCD1	Phospholipase C Delta 1	Protein Coding	48
GABRR1	Gamma-Aminobutyric Acid Type A Receptor Subunit R	Protein Coding	43
LHFPL2	LHFPL Tetraspan Subfamily Member 2	Protein Coding	35
PPP1R8	Protein Phosphatase 1 Regulatory Subunit 8	Protein Coding	40
TIAL1	TIA1 Cytotoxic Granule Associated RNA Binding Prote	Protein Coding	41
NTSR1	Neurotensin Receptor 1	Protein Coding	44
TNFSF14	TNF Superfamily Member 14	Protein Coding	43
HOMER3	Homer Scaffold Protein 3	Protein Coding	41
NFIC	Nuclear Factor I C	Protein Coding	40
TAF6	TATA-Box Binding Protein Associated Factor 6	Protein Coding	41
NCOR2	Nuclear Receptor Corepressor 2	Protein Coding	44
ATP6AP1	ATPase H+ Transporting Accessory Protein 1	Protein Coding	42
P4HA2	Prolyl 4-Hydroxylase Subunit Alpha 2	Protein Coding	47
MRPS18C	Mitochondrial Ribosomal Protein S18C	Protein Coding	38
PCBP1	Poly(RC) Binding Protein 1	Protein Coding	43
MIR19B2	MicroRNA 19b-2	RNA Gene	17
ASXL2	ASXL Transcriptional Regulator 2	Protein Coding	39
MIR196B	MicroRNA 196b	RNA Gene	20
TSPYL1	TSPY Like 1	Protein Coding	41
RCBTB2	RCC1 And BTB Domain Containing Protein 2	Protein Coding	38
STIP1	Stress Induced Phosphoprotein 1	Protein Coding	44
DDR1	Discoidin Domain Receptor Tyrosine Kinase 1	Protein Coding	48
DYTN	Dystrotelin	Protein Coding	31
KLRG1	Killer Cell Lectin Like Receptor G1	Protein Coding	38
RNASE2	Ribonuclease A Family Member 2	Protein Coding	39
PPP1R17	Protein Phosphatase 1 Regulatory Subunit 17	Protein Coding	37
UBE2A	Ubiquitin Conjugating Enzyme E2 A	Protein Coding	47
FRAXA	Fragile Site, Folic Acid Type, Rare, Fra(X)(Q27.3) A (I	Genetic Locus	8
GRIK1	Glutamate Ionotropic Receptor Kainate Type Subunit 1	Protein Coding	47
OBSCN	Obscurin, Cytoskeletal Calmodulin And Titin-Interactin	Protein Coding	41
COA6	Cytochrome C Oxidase Assembly Factor 6	Protein Coding	36
ZFYVE21	Zinc Finger FYVE-Type Containing 21	Protein Coding	36
SEC16A	SEC16 Homolog A, Endoplasmic Reticulum Export Fa	Protein Coding	38
AKAP13	A-Kinase Anchoring Protein 13	Protein Coding	45
UBE2E1	Ubiquitin Conjugating Enzyme E2 E1	Protein Coding	42
TRPC1	Transient Receptor Potential Cation Channel Subfamily	Protein Coding	43
CDNF	Cerebral Dopamine Neurotrophic Factor	Protein Coding	37
AHDC1	AT-Hook DNA Binding Motif Containing 1	Protein Coding	37
BRD2	Bromodomain Containing 2	Protein Coding	45
ORC4	Origin Recognition Complex Subunit 4	Protein Coding	41
RASGRP2	RAS Guanyl Releasing Protein 2	Protein Coding	47
GOT1	Glutamic-Oxaloacetic Transaminase 1	Protein Coding	48
CELF2	CUGBP Elav-Like Family Member 2	Protein Coding	41
DDX3Y	DEAD-Box Helicase 3 Y-Linked	Protein Coding	39
NDUFV2-,	NDUFV2 Antisense RNA 1	RNA Gene	12
GRM7	Glutamate Metabotropic Receptor 7	Protein Coding	45
CCT6A	Chaperonin Containing TCP1 Subunit 6A	Protein Coding	40

ADH4	Alcohol Dehydrogenase 4 (Class II), Pi Polypeptide	Protein Coding	44
PTGDR	Prostaglandin D2 Receptor	Protein Coding	48
EN2	Engrailed Homeobox 2	Protein Coding	40
ACP3	Acid Phosphatase 3	Protein Coding	36
CBR1	Carbonyl Reductase 1	Protein Coding	47
ABCF2	ATP Binding Cassette Subfamily F Member 2	Protein Coding	40
PDCL3	Phosducin Like 3	Protein Coding	36
OSTF1	Osteoclast Stimulating Factor 1	Protein Coding	41
PDIA2	Protein Disulfide Isomerase Family A Member 2	Protein Coding	40
FCGRT	Fc Fragment Of IgG Receptor And Transporter	Protein Coding	41
P2RY2	Purinergic Receptor P2Y2	Protein Coding	47
DDAH1	Dimethylarginine Dimethylaminohydrolase 1	Protein Coding	44
STX8	Syntaxin 8	Protein Coding	40
DDX17	DEAD-Box Helicase 17	Protein Coding	41
ZCWPW1	Zinc Finger CW-Type And PWWP Domain Containing	Protein Coding	33
CACNG2	Calcium Voltage-Gated Channel Auxiliary Subunit Gan	Protein Coding	45
MLLT3	MLLT3 Super Elongation Complex Subunit	Protein Coding	40
IMMT	Inner Membrane Mitochondrial Protein	Protein Coding	41
CARD16	Caspase Recruitment Domain Family Member 16	Protein Coding	39
RUVBL2	RuvB Like AAA ATPase 2	Protein Coding	45
MIR431	MicroRNA 431	RNA Gene	20
SLC22A3	Solute Carrier Family 22 Member 3	Protein Coding	44
ST13	ST13 Hsp70 Interacting Protein	Protein Coding	40
TMEM165	Transmembrane Protein 165	Protein Coding	39
CIRBP	Cold Inducible RNA Binding Protein	Protein Coding	39
GFPT2	Glutamine-Fructose-6-Phosphate Transaminase 2	Protein Coding	44
CYFIP1	Cytoplasmic FMR1 Interacting Protein 1	Protein Coding	42
ARRB2	Arrestin Beta 2	Protein Coding	45
GABRA6	Gamma-Aminobutyric Acid Type A Receptor Subunit A	Protein Coding	45
USP6	Ubiquitin Specific Peptidase 6	Protein Coding	40
ATP6V1E	ATPase H+ Transporting V1 Subunit E1	Protein Coding	44
FTMT	Ferritin Mitochondrial	Protein Coding	37
RBBP4	RB Binding Protein 4, Chromatin Remodeling Factor	Protein Coding	45
DHX37	DEAH-Box Helicase 37	Protein Coding	38
GSTA1	Glutathione S-Transferase Alpha 1	Protein Coding	43
MYO1D	Myosin ID	Protein Coding	41
SWI5	SWI5 Homologous Recombination Repair Protein	Protein Coding	29
PIR	Pirin	Protein Coding	40
RPL3L	Ribosomal Protein L3 Like	Protein Coding	40
RPS4Y1	Ribosomal Protein S4 Y-Linked 1	Protein Coding	35
ABI1	Abl Interactor 1	Protein Coding	44
SPG36	Spastic Paraplegia 36 (Autosomal Dominant)	Genetic Locus	4
ACTL7B	Actin Like 7B	Protein Coding	35
MAPK7	Mitogen-Activated Protein Kinase 7	Protein Coding	50
MGAT5	Alpha-1,6-Mannosylglycoprotein 6-Beta-N-Acetylgluco	Protein Coding	40
CRY1	Cryptochrome Circadian Regulator 1	Protein Coding	45
HYOU1	Hypoxia Up-Regulated 1	Protein Coding	44

RPH3AL	Rabphilin 3A Like (Without C2 Domains)	Protein Coding	39
TSBP1	Testis Expressed Basic Protein 1	Protein Coding	23
PSMD7	Proteasome 26S Subunit, Non-ATPase 7	Protein Coding	43
MIR135B	MicroRNA 135b	RNA Gene	20
ARNTL2	Aryl Hydrocarbon Receptor Nuclear Translocator Like	Protein Coding	40
MED1	Mediator Complex Subunit 1	Protein Coding	41
GABRE	Gamma-Aminobutyric Acid Type A Receptor Subunit I	Protein Coding	39
RAB3IP	RAB3A Interacting Protein	Protein Coding	38
GPR50	G Protein-Coupled Receptor 50	Protein Coding	43
AMOT	Angiomotin	Protein Coding	41
AP3B2	Adaptor Related Protein Complex 3 Subunit Beta 2	Protein Coding	44
BYSL	Bystin Like	Protein Coding	39
LGALS8	Galectin 8	Protein Coding	43
MIR186	MicroRNA 186	RNA Gene	19
TREML2	Triggering Receptor Expressed On Myeloid Cells Like 2	Protein Coding	40
MAP1LC3	Microtubule Associated Protein 1 Light Chain 3 Alpha	Protein Coding	45
PAK6	P21 (RAC1) Activated Kinase 6	Protein Coding	47
GNL3	G Protein Nucleolar 3	Protein Coding	41
HSD17B1	Hydroxysteroid 17-Beta Dehydrogenase 1	Protein Coding	45
TUBA3D	Tubulin Alpha 3d	Protein Coding	37
MIR590	MicroRNA 590	RNA Gene	19
JAKMIP1	Janus Kinase And Microtubule Interacting Protein 1	Protein Coding	37
ANKRD12	Ankyrin Repeat Domain 12	Protein Coding	36
CTBP2	C-Terminal Binding Protein 2	Protein Coding	45
ANXA6	Annexin A6	Protein Coding	44
NAXE	NAD(P)HX Epimerase	Protein Coding	36
MYO1C	Myosin IC	Protein Coding	44
PARP4	Poly(ADP-Ribose) Polymerase Family Member 4	Protein Coding	44
GCLM	Glutamate-Cysteine Ligase Modifier Subunit	Protein Coding	43
RPL36	Ribosomal Protein L36	Protein Coding	41
TOMM20I	Translocase Of Outer Mitochondrial Membrane 20 Like	Protein Coding	31
BRINP1	BMP/Retinoic Acid Inducible Neural Specific 1	Protein Coding	32
PTPRD	Protein Tyrosine Phosphatase Receptor Type D	Protein Coding	45
GPKOW	G-Patch Domain And KOW Motifs	Protein Coding	36
RBM39	RNA Binding Motif Protein 39	Protein Coding	40
RPL27A	Ribosomal Protein L27a	Protein Coding	42
GFRA2	GDNF Family Receptor Alpha 2	Protein Coding	45
EPS8L3	EPS8 Like 3	Protein Coding	40
SSR4	Signal Sequence Receptor Subunit 4	Protein Coding	43
KLK7	Kallikrein Related Peptidase 7	Protein Coding	41
LOC64236	Uncharacterized LOC642361	RNA Gene	12
PPP2R1B	Protein Phosphatase 2 Scaffold Subunit Abeta	Protein Coding	49
MIR873	MicroRNA 873	RNA Gene	17
HMGB2	High Mobility Group Box 2	Protein Coding	44
SEMA4D	Semaphorin 4D	Protein Coding	45
PHACTR2	Phosphatase And Actin Regulator 2	Protein Coding	35
NUMB	NUMB Endocytic Adaptor Protein	Protein Coding	48

CNDP2	Carnosine Dipeptidase 2	Protein Coding	44
PPP1R3B	Protein Phosphatase 1 Regulatory Subunit 3B	Protein Coding	38
VIPR2	Vasoactive Intestinal Peptide Receptor 2	Protein Coding	45
AP5M1	Adaptor Related Protein Complex 5 Subunit Mu 1	Protein Coding	33
LAMTOR:	Late Endosomal/Lysosomal Adaptor, MAPK And MTC	Protein Coding	41
MCAM	Melanoma Cell Adhesion Molecule	Protein Coding	41
ADAMTS:	ADAM Metallopeptidase With Thrombospondin Type 1	Protein Coding	36
ISM1	Isthmin 1	Protein Coding	34
P3H2	Prolyl 3-Hydroxylase 2	Protein Coding	37
ITGA5	Integrin Subunit Alpha 5	Protein Coding	50
PTCD3	Pentatricopeptide Repeat Domain 3	Protein Coding	36
NSDHL	NAD(P) Dependent Steroid Dehydrogenase-Like	Protein Coding	44
CLDN3	Claudin 3	Protein Coding	41
TMEM119	Transmembrane Protein 119	Protein Coding	34
FARSA	Phenylalanyl-TRNA Synthetase Subunit Alpha	Protein Coding	41
BNIP3L	BCL2 Interacting Protein 3 Like	Protein Coding	43
IL32	Interleukin 32	Protein Coding	41
NOVA1	NOVA Alternative Splicing Regulator 1	Protein Coding	40
DBP	D-Box Binding PAR BZIP Transcription Factor	Protein Coding	39
LOC10960	Transcription Factor 4 Repeat Instability Region	Biological Region	2
MAIP1	Matrix AAA Peptidase Interacting Protein 1	Protein Coding	27
MRPS27	Mitochondrial Ribosomal Protein S27	Protein Coding	37
OPLAH	5-Oxoprolinase, ATP-Hydrolysing	Protein Coding	41
FZD7	Frizzled Class Receptor 7	Protein Coding	45
SGCZ	Sarcoglycan Zeta	Protein Coding	36
ACTL6B	Actin Like 6B	Protein Coding	43
S1PR1	Sphingosine-1-Phosphate Receptor 1	Protein Coding	46
MEST	Mesoderm Specific Transcript	Protein Coding	40
CAPNS1	Calpain Small Subunit 1	Protein Coding	42
TBX15	T-Box Transcription Factor 15	Protein Coding	42
FSTL5	Follistatin Like 5	Protein Coding	36
DNAJA3	DnaJ Heat Shock Protein Family (Hsp40) Member A3	Protein Coding	41
GRIK4	Glutamate Ionotropic Receptor Kainate Type Subunit 4	Protein Coding	44
ADCY2	Adenylate Cyclase 2	Protein Coding	45
BUD23	BUD23 RRNA Methyltransferase And Ribosome Matu	Protein Coding	31
FABP7	Fatty Acid Binding Protein 7	Protein Coding	44
KTN1	Kinectin 1	Protein Coding	40
TFB2M	Transcription Factor B2, Mitochondrial	Protein Coding	39
MAPKAP1	MAPK Associated Protein 1	Protein Coding	45
RSPO3	R-Spondin 3	Protein Coding	38
CRK	CRK Proto-Oncogene, Adaptor Protein	Protein Coding	47
ALDH4A1	Aldehyde Dehydrogenase 4 Family Member A1	Protein Coding	45
BAG5	BAG Cochaperone 5	Protein Coding	40
PLXNA2	Plexin A2	Protein Coding	43
EIF3A	Eukaryotic Translation Initiation Factor 3 Subunit A	Protein Coding	42
UNC13C	Unc-13 Homolog C	Protein Coding	36
BARHL1	BarH Like Homeobox 1	Protein Coding	35

KIF13B	Kinesin Family Member 13B	Protein Coding	41
PCDH17	Protocadherin 17	Protein Coding	37
MNDA	Myeloid Cell Nuclear Differentiation Antigen	Protein Coding	42
LUC7L2	LUC7 Like 2, Pre-MRNA Splicing Factor	Protein Coding	37
ETV4	ETS Variant Transcription Factor 4	Protein Coding	45
ARRB1	Arrestin Beta 1	Protein Coding	45
ATF3	Activating Transcription Factor 3	Protein Coding	45
STUM	Stum, Mechanosensory Transduction Mediator Homolo	Protein Coding	26
MOB3B	MOB Kinase Activator 3B	Protein Coding	37
ANKMY1	Ankyrin Repeat And MYND Domain Containing 1	Protein Coding	36
GPAM	Glycerol-3-Phosphate Acyltransferase, Mitochondrial	Protein Coding	44
DGCR5	DiGeorge Syndrome Critical Region Gene 5	RNA Gene	22
PTPRZ1	Protein Tyrosine Phosphatase Receptor Type Z1	Protein Coding	43
MINPP1	Multiple Inositol-Polyphosphate Phosphatase 1	Protein Coding	46
MIR7-1	MicroRNA 7-1	RNA Gene	20
KLK15	Kallikrein Related Peptidase 15	Protein Coding	42
PPP5C	Protein Phosphatase 5 Catalytic Subunit	Protein Coding	46
TAS1R2	Taste 1 Receptor Member 2	Protein Coding	36
WASHC2(	WASH Complex Subunit 2C	Protein Coding	28
IGF2BP1	Insulin Like Growth Factor 2 MRNA Binding Protein 1	Protein Coding	41
GALNT12	Polypeptide N-Acetylgalactosaminyltransferase 12	Protein Coding	43
TYRO3	TYRO3 Protein Tyrosine Kinase	Protein Coding	49
LAD1	Ladinin 1	Protein Coding	37
TREML1	Triggering Receptor Expressed On Myeloid Cells Like [illegible]	Protein Coding	39
GBAP1	Glucosylceramidase Beta Pseudogene 1	Pseudogene	16
GRIK5	Glutamate Ionotropic Receptor Kainate Type Subunit 5	Protein Coding	44
IL13RA1	Interleukin 13 Receptor Subunit Alpha 1	Protein Coding	44
S100A10	S100 Calcium Binding Protein A10	Protein Coding	47
PRRG4	Proline Rich And Gla Domain 4	Protein Coding	34
CHRNB3	Cholinergic Receptor Nicotinic Beta 3 Subunit	Protein Coding	41
SLC8A3	Solute Carrier Family 8 Member A3	Protein Coding	44
RPL23A	Ribosomal Protein L23a	Protein Coding	41
DDX1	DEAD-Box Helicase 1	Protein Coding	43
CD93	CD93 Molecule	Protein Coding	41
UTP4	UTP4 Small Subunit Processome Component	Protein Coding	32
GRB10	Growth Factor Receptor Bound Protein 10	Protein Coding	44
AGO2	Argonaute RISC Catalytic Component 2	Protein Coding	41
RPL13A	Ribosomal Protein L13a	Protein Coding	42
COX6A2	Cytochrome C Oxidase Subunit 6A2	Protein Coding	39
B3GALNT	Beta-1,3-N-Acetylgalactosaminyltransferase 1 (Globosi	Protein Coding	42
DUSP19	Dual Specificity Phosphatase 19	Protein Coding	40
CXCL6	C-X-C Motif Chemokine Ligand 6	Protein Coding	41
MIR4274	MicroRNA 4274	RNA Gene	12
ALKBH3	AlkB Homolog 3, Alpha-Ketoglutaratedependent Dioxy	Protein Coding	39
CADPS2	Calcium Dependent Secretion Activator 2	Protein Coding	39
NTPCR	Nucleoside-Triphosphatase, Cancer-Related	Protein Coding	38
SLC17A7	Solute Carrier Family 17 Member 7	Protein Coding	43

HSPE1	Heat Shock Protein Family E (Hsp10) Member 1	Protein Coding	41
FAM3B	FAM3 Metabolism Regulating Signaling Molecule B	Protein Coding	40
MAL	Mal, T Cell Differentiation Protein	Protein Coding	38
NR1D1	Nuclear Receptor Subfamily 1 Group D Member 1	Protein Coding	48
DHRS2	Dehydrogenase/Reductase 2	Protein Coding	39
AK1	Adenylate Kinase 1	Protein Coding	49
AGAP1	ArfGAP With GTPase Domain, Ankyrin Repeat And Pl	Protein Coding	40
G3BP1	G3BP Stress Granule Assembly Factor 1	Protein Coding	41
CHORDC1	Cysteine And Histidine Rich Domain Containing 1	Protein Coding	36
ILF3	Interleukin Enhancer Binding Factor 3	Protein Coding	39
ACTL7A	Actin Like 7A	Protein Coding	36
FGF13	Fibroblast Growth Factor 13	Protein Coding	43
ACTL6A	Actin Like 6A	Protein Coding	40
NOA1	Nitric Oxide Associated 1	Protein Coding	35
AZU1	Azurocidin 1	Protein Coding	40
TMC3	Transmembrane Channel Like 3	Protein Coding	31
AGR2	Anterior Gradient 2, Protein Disulphide Isomerase Fami	Protein Coding	42
RAB11FIP	RAB11 Family Interacting Protein 2	Protein Coding	41
RAP1GAP	RAP1 GTPase Activating Protein	Protein Coding	40
RPN2	Ribophorin II	Protein Coding	43
LINGO4	Leucine Rich Repeat And Ig Domain Containing 4	Protein Coding	32
EMC3	ER Membrane Protein Complex Subunit 3	Protein Coding	32
SLIT3	Slit Guidance Ligand 3	Protein Coding	43
SLC25A27	Solute Carrier Family 25 Member 27	Protein Coding	39
JAGN1	Jagunal Homolog 1	Protein Coding	37
KPNA5	Karyopherin Subunit Alpha 5	Protein Coding	39
TMEM229	Transmembrane Protein 229B	Protein Coding	29
POU2F1	POU Class 2 Homeobox 1	Protein Coding	46
MRPL45	Mitochondrial Ribosomal Protein L45	Protein Coding	35
ACLY	ATP Citrate Lyase	Protein Coding	47
TBL3	Transducin Beta Like 3	Protein Coding	40
KPNA2	Karyopherin Subunit Alpha 2	Protein Coding	47
TK1	Thymidine Kinase 1	Protein Coding	48
H1-1	H1.1 Linker Histone, Cluster Member	Protein Coding	32
OXCT1	3-Oxoacid CoA-Transferase 1	Protein Coding	46
NUDT19	Nudix Hydrolase 19	Protein Coding	36
SESN2	Sestrin 2	Protein Coding	39
BSN	Bassoon Presynaptic Cytomatrix Protein	Protein Coding	36
QSER1	Glutamine And Serine Rich 1	Protein Coding	31
CPEB4	Cytoplasmic Polyadenylation Element Binding Protein 4	Protein Coding	37
ZP3	Zona Pellucida Glycoprotein 3	Protein Coding	43
SF3B3	Splicing Factor 3b Subunit 3	Protein Coding	40
TBC1D4	TBC1 Domain Family Member 4	Protein Coding	45
NDUFV3	NADH:Ubiquinone Oxidoreductase Subunit V3	Protein Coding	42
HTR1D	5-Hydroxytryptamine Receptor 1D	Protein Coding	47
EPB41L1	Erythrocyte Membrane Protein Band 4.1 Like 1	Protein Coding	44
CCT7	Chaperonin Containing TCP1 Subunit 7	Protein Coding	42

PROSER1	Proline And Serine Rich 1	Protein Coding	30
MRPS23	Mitochondrial Ribosomal Protein S23	Protein Coding	38
TTC39B	Tetratricopeptide Repeat Domain 39B	Protein Coding	37
WBP11	WW Domain Binding Protein 11	Protein Coding	37
PRDM15	PR/SET Domain 15	Protein Coding	33
DNAJC1	DnaJ Heat Shock Protein Family (Hsp40) Member C1	Protein Coding	38
MAGEA4	MAGE Family Member A4	Protein Coding	37
FOSB	FosB Proto-Oncogene, AP-1 Transcription Factor Subu	Protein Coding	43
PNPLA4	Patatin Like Phospholipase Domain Containing 4	Protein Coding	38
EDC3	Enhancer Of MRNA Decapping 3	Protein Coding	42
FBXO32	F-Box Protein 32	Protein Coding	41
PIGK	Phosphatidylinositol Glycan Anchor Biosynthesis Class	Protein Coding	42
TUBA1C	Tubulin Alpha 1c	Protein Coding	43
PSMD9	Proteasome 26S Subunit, Non-ATPase 9	Protein Coding	44
EIF3D	Eukaryotic Translation Initiation Factor 3 Subunit D	Protein Coding	40
MRPS9	Mitochondrial Ribosomal Protein S9	Protein Coding	40
SATB1	SATB Homeobox 1	Protein Coding	43
MYO1B	Myosin IB	Protein Coding	39
TRIM27	Tripartite Motif Containing 27	Protein Coding	42
CHML	CHM Like Rab Escort Protein	Protein Coding	39
FOSL2	FOS Like 2, AP-1 Transcription Factor Subunit	Protein Coding	42
RCSD1	RCSD Domain Containing 1	Protein Coding	36
GCN1	GCN1 Activator Of EIF2AK4	Protein Coding	29
ASTN2	Astrotactin 2	Protein Coding	37
PTPRT	Protein Tyrosine Phosphatase Receptor Type T	Protein Coding	43
SPTBN1	Spectrin Beta, Non-Erythrocytic 1	Protein Coding	45
GATD3A	Glutamine Amidotransferase Like Class 1 Domain Cont	Protein Coding	27
PICK1	Protein Interacting With PRKCA 1	Protein Coding	42
MCM3	Minichromosome Maintenance Complex Component 3	Protein Coding	47
TMPRSS5	Transmembrane Serine Protease 5	Protein Coding	39
NDFIP1	Nedd4 Family Interacting Protein 1	Protein Coding	38
FAF2	Fas Associated Factor Family Member 2	Protein Coding	39
DGCR6	DiGeorge Syndrome Critical Region Gene 6	Protein Coding	34
DDX5	DEAD-Box Helicase 5	Protein Coding	48
PRSS53	Serine Protease 53	Protein Coding	34
CPLX2	Complexin 2	Protein Coding	41
NDUFB2	NADH:Ubiquinone Oxidoreductase Subunit B2	Protein Coding	40
MYBBP1A	MYB Binding Protein 1a	Protein Coding	41
ZHX2	Zinc Fingers And Homeoboxes 2	Protein Coding	38
DPP7	Dipeptidyl Peptidase 7	Protein Coding	41
KIF12	Kinesin Family Member 12	Protein Coding	36
RAB6A	RAB6A, Member RAS Oncogene Family	Protein Coding	42
MIR6070	MicroRNA 6070	RNA Gene	10
ETV5	ETS Variant Transcription Factor 5	Protein Coding	40
TBX10	T-Box Transcription Factor 10	Protein Coding	37
KAT2A	Lysine Acetyltransferase 2A	Protein Coding	49
NVL	Nuclear VCP Like	Protein Coding	39

PPRC1	PPARG Related Coactivator 1	Protein Coding	39
PIK3R3	Phosphoinositide-3-Kinase Regulatory Subunit 3	Protein Coding	45
FARP1	FERM, ARH/RhoGEF And Pleckstrin Domain Protein	Protein Coding	39
USP7	Ubiquitin Specific Peptidase 7	Protein Coding	48
ADRA1B	Adrenoceptor Alpha 1B	Protein Coding	47
CDK7	Cyclin Dependent Kinase 7	Protein Coding	47
MUL1	Mitochondrial E3 Ubiquitin Protein Ligase 1	Protein Coding	39
PAK2	P21 (RAC1) Activated Kinase 2	Protein Coding	47
ALDH1B1	Aldehyde Dehydrogenase 1 Family Member B1	Protein Coding	46
CA3	Carbonic Anhydrase 3	Protein Coding	43
SEPTIN12	Septin 12	Protein Coding	31
MOV10	Mov10 RISC Complex RNA Helicase	Protein Coding	41
UGT1A	UDP Glucuronosyltransferase Family 1 Member A Con	Genetic Locus	10
UQCR10	Ubiquinol-Cytochrome C Reductase, Complex III Subu	Protein Coding	40
PMPCB	Peptidase, Mitochondrial Processing Subunit Beta	Protein Coding	43
LRRFIP1	LRR Binding FLII Interacting Protein 1	Protein Coding	38
NGDN	Neuroguidin	Protein Coding	34
POLRMT	RNA Polymerase Mitochondrial	Protein Coding	43
TMEM128	Transmembrane Protein 128	Protein Coding	33
CBX5	Chromobox 5	Protein Coding	45
PLXNA4	Plexin A4	Protein Coding	39
GABRQ	Gamma-Aminobutyric Acid Type A Receptor Subunit T	Protein Coding	38
NCKAP1	NCK Associated Protein 1	Protein Coding	40
SH3GL3	SH3 Domain Containing GRB2 Like 3, Endophilin A3	Protein Coding	43
RNF20	Ring Finger Protein 20	Protein Coding	40
TRIM39	Tripartite Motif Containing 39	Protein Coding	39
MIR7-3	MicroRNA 7-3	RNA Gene	18
PABPC4	Poly(A) Binding Protein Cytoplasmic 4	Protein Coding	43
FZD1	Frizzled Class Receptor 1	Protein Coding	45
ATP2B1	ATPase Plasma Membrane Ca2+ Transporting 1	Protein Coding	44
CHMP3	Charged Multivesicular Body Protein 3	Protein Coding	39
CRHR2	Corticotropin Releasing Hormone Receptor 2	Protein Coding	43
IPO9	Importin 9	Protein Coding	36
ID1	Inhibitor Of DNA Binding 1, HLH Protein	Protein Coding	44
ACADSB	Acyl-CoA Dehydrogenase Short/Branched Chain	Protein Coding	47
UGT1A10	UDP Glucuronosyltransferase Family 1 Member A10	Protein Coding	41
TOR2A	Torsin Family 2 Member A	Protein Coding	37
ARHGAP9	Rho GTPase Activating Protein 9	Protein Coding	43
NPTXR	Neuronal Pentraxin Receptor	Protein Coding	39
MYCNOS	MYCN Opposite Strand	RNA Gene	25
PDE3B	Phosphodiesterase 3B	Protein Coding	44
RXFP2	Relaxin Family Peptide Receptor 2	Protein Coding	44
KCNN1	Potassium Calcium-Activated Channel Subfamily N Me	Protein Coding	40
RAB5B	RAB5B, Member RAS Oncogene Family	Protein Coding	41
MCC	MCC Regulator Of WNT Signaling Pathway	Protein Coding	41
PTK7	Protein Tyrosine Kinase 7 (Inactive)	Protein Coding	44
FBXO2	F-Box Protein 2	Protein Coding	39

GSDMA	Gasdermin A	Protein Coding	37
GGA2	Golgi Associated, Gamma Adaptin Ear Containing, AR	Protein Coding	40
H3-4	H3.4 Histone	Protein Coding	33
ESRRA	Estrogen Related Receptor Alpha	Protein Coding	48
POLR2K	RNA Polymerase II Subunit K	Protein Coding	38
THAP7	THAP Domain Containing 7	Protein Coding	35
KRTCAP2	Keratinocyte Associated Protein 2	Protein Coding	34
ADRA1D	Adrenoceptor Alpha 1D	Protein Coding	47
SEPTIN6	Septin 6	Protein Coding	31
OARD1	O-Acyl-ADP-Ribose Deacylase 1	Protein Coding	35
COL4A6	Collagen Type IV Alpha 6 Chain	Protein Coding	44
PGRMC2	Progesterone Receptor Membrane Component 2	Protein Coding	40
DFFA	DNA Fragmentation Factor Subunit Alpha	Protein Coding	47
PTPRR	Protein Tyrosine Phosphatase Receptor Type R	Protein Coding	41
EFNA5	Ephrin A5	Protein Coding	45
PABPC3	Poly(A) Binding Protein Cytoplasmic 3	Protein Coding	39
DNAH14	Dynein Axonemal Heavy Chain 14	Protein Coding	32
SV2C	Synaptic Vesicle Glycoprotein 2C	Protein Coding	40
CSNK1A1	Casein Kinase 1 Alpha 1 Like	Protein Coding	37
NCS1	Neuronal Calcium Sensor 1	Protein Coding	43
MARCKSl	MARCKS Like 1	Protein Coding	39
NPW	Neuropeptide W	Protein Coding	36
STK4	Serine/Threonine Kinase 4	Protein Coding	50
ZFAT	Zinc Finger And AT-Hook Domain Containing	Protein Coding	36
SLC9A9	Solute Carrier Family 9 Member A9	Protein Coding	39
CELSR3	Cadherin EGF LAG Seven-Pass G-Type Receptor 3	Protein Coding	41
USP36	Ubiquitin Specific Peptidase 36	Protein Coding	39
RILP	Rab Interacting Lysosomal Protein	Protein Coding	37
PPP1CA	Protein Phosphatase 1 Catalytic Subunit Alpha	Protein Coding	50
SRR	Serine Racemase	Protein Coding	44
RAB3IL1	RAB3A Interacting Protein Like 1	Protein Coding	38
SNX13	Sorting Nexin 13	Protein Coding	37
ACKR3	Atypical Chemokine Receptor 3	Protein Coding	42
CFAP126	Cilia And Flagella Associated Protein 126	Protein Coding	28
CCDC82	Coiled-Coil Domain Containing 82	Protein Coding	31
MSRB3	Methionine Sulfoxide Reductase B3	Protein Coding	44
SRRM2	Serine/Arginine Repetitive Matrix 2	Protein Coding	35
H3C6	H3 Clustered Histone 6	Protein Coding	28
ACSL5	Acyl-CoA Synthetase Long Chain Family Member 5	Protein Coding	44
SOAT2	Sterol O-Acyltransferase 2	Protein Coding	45
THBS3	Thrombospondin 3	Protein Coding	42
KIF3B	Kinesin Family Member 3B	Protein Coding	40
PTTG1	PTTG1 Regulator Of Sister Chromatid Separation, Sec	Protein Coding	44
BAG2	BAG Cochaperone 2	Protein Coding	42
DCAF7	DDB1 And CUL4 Associated Factor 7	Protein Coding	36
CYP21A1I	Cytochrome P450 Family 21 Subfamily A Member 1, P	Pseudogene	17
DCLK1	Doublecortin Like Kinase 1	Protein Coding	43

HDGF	Heparin Binding Growth Factor	Protein Coding	42
CHRNA6	Cholinergic Receptor Nicotinic Alpha 6 Subunit	Protein Coding	43
IMPDH2	Inosine Monophosphate Dehydrogenase 2	Protein Coding	47
COPS5	COP9 Signalosome Subunit 5	Protein Coding	44
SULF1	Sulfatase 1	Protein Coding	41
ASPSCR1	ASPSCR1 Tether For SLC2A4, UBX Domain Containi	Protein Coding	43
TST	Thiosulfate Sulfurtransferase	Protein Coding	43
PTPN5	Protein Tyrosine Phosphatase Non-Receptor Type 5	Protein Coding	44
GPR65	G Protein-Coupled Receptor 65	Protein Coding	40
SPAG8	Sperm Associated Antigen 8	Protein Coding	32
AP1M1	Adaptor Related Protein Complex 1 Subunit Mu 1	Protein Coding	41
LMOD2	Leiomodin 2	Protein Coding	34
CIAPIN1	Cytokine Induced Apoptosis Inhibitor 1	Protein Coding	40
RNF144B	Ring Finger Protein 144B	Protein Coding	37
ZMYM5	Zinc Finger MYM-Type Containing 5	Protein Coding	35
SEC23A	Sec23 Homolog A, COPII Coat Complex Component	Protein Coding	44
BCL2A1	BCL2 Related Protein A1	Protein Coding	42
TAB1	TGF-Beta Activated Kinase 1 (MAP3K7) Binding Prot	Protein Coding	44
PIM2	Pim-2 Proto-Oncogene, Serine/Threonine Kinase	Protein Coding	45
KCNN2	Potassium Calcium-Activated Channel Subfamily N Me	Protein Coding	44
MRPS31	Mitochondrial Ribosomal Protein S31	Protein Coding	37
DMTN	Dematin Actin Binding Protein	Protein Coding	38
PCLAF	PCNA Clamp Associated Factor	Protein Coding	30
TOM1L2	Target Of Myb1 Like 2 Membrane Trafficking Protein	Protein Coding	39
TRADD	TNFRSF1A Associated Via Death Domain	Protein Coding	44
MYL1	Myosin Light Chain 1	Protein Coding	43
BAIAP2	BAR/IMD Domain Containing Adaptor Protein 2	Protein Coding	44
SLC30A3	Solute Carrier Family 30 Member 3	Protein Coding	39
NUDT16L	Nudix Hydrolase 16 Like 1	Protein Coding	37
KCTD6	Potassium Channel Tetramerization Domain Containing	Protein Coding	35
CYP17A1-	CYP17A1 Antisense RNA 1	RNA Gene	15
TRIM22	Tripartite Motif Containing 22	Protein Coding	40
KCNAB1	Potassium Voltage-Gated Channel Subfamily A Membe	Protein Coding	41
SH3GLB1	SH3 Domain Containing GRB2 Like, Endophilin B1	Protein Coding	41
PDIA3	Protein Disulfide Isomerase Family A Member 3	Protein Coding	47
EIF3L	Eukaryotic Translation Initiation Factor 3 Subunit L	Protein Coding	39
AP5S1	Adaptor Related Protein Complex 5 Subunit Sigma 1	Protein Coding	32
CXorf56	Chromosome X Open Reading Frame 56	Protein Coding	36
RAD23B	RAD23 Homolog B, Nucleotide Excision Repair Protei	Protein Coding	45
FBXO3	F-Box Protein 3	Protein Coding	39
SLC18A1	Solute Carrier Family 18 Member A1	Protein Coding	46
ANXA9	Annexin A9	Protein Coding	40
IL9R	Interleukin 9 Receptor	Protein Coding	36
CMTR2	Cap Methyltransferase 2	Protein Coding	33
GTF2H4	General Transcription Factor IIH Subunit 4	Protein Coding	41
CA1	Carbonic Anhydrase 1	Protein Coding	48
MPZL1	Myelin Protein Zero Like 1	Protein Coding	43

ALDH6A1	Aldehyde Dehydrogenase 6 Family Member A1	Protein Coding	47
CBR3	Carbonyl Reductase 3	Protein Coding	45
GPR55	G Protein-Coupled Receptor 55	Protein Coding	41
ARHGAP2	Rho GTPase Activating Protein 24	Protein Coding	40
MAD2L1	Mitotic Arrest Deficient 2 Like 1	Protein Coding	45
HLA-DQB	Major Histocompatibility Complex, Class II, DQ Beta 2	Protein Coding	37
VWA5A	Von Willebrand Factor A Domain Containing 5A	Protein Coding	36
CASP4	Caspase 4	Protein Coding	47
BCL7C	BAF Chromatin Remodeling Complex Subunit BCL7C	Protein Coding	37
PSMD14	Proteasome 26S Subunit, Non-ATPase 14	Protein Coding	43
CDK5RAF	CDK5 Regulatory Subunit Associated Protein 1	Protein Coding	38
S100G	S100 Calcium Binding Protein G	Protein Coding	36
KLK2	Kallikrein Related Peptidase 2	Protein Coding	43
H4C11	H4 Clustered Histone 11	Protein Coding	30
ITSN2	Intersectin 2	Protein Coding	40
NOP2	NOP2 Nucleolar Protein	Protein Coding	37
RFC4	Replication Factor C Subunit 4	Protein Coding	42
HSPA6	Heat Shock Protein Family A (Hsp70) Member 6	Protein Coding	45
JPH4	Junctophilin 4	Protein Coding	35
FERMT2	Fermitin Family Member 2	Protein Coding	39
CLEC3B	C-Type Lectin Domain Family 3 Member B	Protein Coding	41
ZFP3	ZFP3 Zinc Finger Protein	Protein Coding	35
RPS8	Ribosomal Protein S8	Protein Coding	40
TUBA3C	Tubulin Alpha 3c	Protein Coding	38
SIRT5	Sirtuin 5	Protein Coding	46
BST2	Bone Marrow Stromal Cell Antigen 2	Protein Coding	41
DLX1	Distal-Less Homeobox 1	Protein Coding	40
KIFC3	Kinesin Family Member C3	Protein Coding	39
RGMA	Repulsive Guidance Molecule BMP Co-Receptor A	Protein Coding	41
MBTPS1	Membrane Bound Transcription Factor Peptidase, Site 1	Protein Coding	44
MUCL3	Mucin Like 3	Protein Coding	27
KCNMB1	Potassium Calcium-Activated Channel Subfamily M Re	Protein Coding	43
MBD4	Methyl-CpG Binding Domain 4, DNA Glycosylase	Protein Coding	44
PSMD11	Proteasome 26S Subunit, Non-ATPase 11	Protein Coding	42
CFAP53	Cilia And Flagella Associated Protein 53	Protein Coding	35
EIF3E	Eukaryotic Translation Initiation Factor 3 Subunit E	Protein Coding	39
DFFB	DNA Fragmentation Factor Subunit Beta	Protein Coding	44
SEPTIN8	Septin 8	Protein Coding	31
HEYL	Hes Related Family BHLH Transcription Factor With Y	Protein Coding	40
BACH1	BTB Domain And CNC Homolog 1	Protein Coding	42
PSME3	Proteasome Activator Subunit 3	Protein Coding	43
EPS15	Epidermal Growth Factor Receptor Pathway Substrate	Protein Coding	47
ME3	Malic Enzyme 3	Protein Coding	41
ZNF148	Zinc Finger Protein 148	Protein Coding	41
MIR1294	MicroRNA 1294	RNA Gene	13
HS3ST1	Heparan Sulfate-Glucosamine 3-Sulfotransferase 1	Protein Coding	43
CCAR2	Cell Cycle And Apoptosis Regulator 2	Protein Coding	37

INTS7	Integrator Complex Subunit 7	Protein Coding	37
PGRMC1	Progesterone Receptor Membrane Component 1	Protein Coding	45
RAI2	Retinoic Acid Induced 2	Protein Coding	35
MIR548D1	MicroRNA 548d-1	RNA Gene	12
TDO2	Tryptophan 2,3-Dioxygenase	Protein Coding	47
ABCB8	ATP Binding Cassette Subfamily B Member 8	Protein Coding	44
RALA	RAS Like Proto-Oncogene A	Protein Coding	47
IER3	Immediate Early Response 3	Protein Coding	39
FRAXE	Fragile Site, Folic Acid Type, Rare, Fra(X)(Q28) E	Genetic Locus	6
ALCAM	Activated Leukocyte Cell Adhesion Molecule	Protein Coding	44
CASP14	Caspase 14	Protein Coding	46
IGSF6	Immunoglobulin Superfamily Member 6	Protein Coding	36
MT-TY	Mitochondrially Encoded TRNA-Tyr (UAU/C)	RNA Gene	11
OCIAD1	OCIA Domain Containing 1	Protein Coding	36
DYNLL1	Dynein Light Chain LC8-Type 1	Protein Coding	44
DAP3	Death Associated Protein 3	Protein Coding	40
IMPA1	Inositol Monophosphatase 1	Protein Coding	50
MRC2	Mannose Receptor C Type 2	Protein Coding	41
TBKBP1	TBK1 Binding Protein 1	Protein Coding	36
OLFM4	Olfactomedin 4	Protein Coding	39
MIR383	MicroRNA 383	RNA Gene	16
PYGB	Glycogen Phosphorylase B	Protein Coding	45
TRAPPC4	Trafficking Protein Particle Complex 4	Protein Coding	39
KCTD21	Potassium Channel Tetramerization Domain Containing	Protein Coding	32
PDLIM5	PDZ And LIM Domain 5	Protein Coding	40
FOXO4	Forkhead Box O4	Protein Coding	44
MT1F	Metallothionein 1F	Protein Coding	39
CYGB	Cytoglobin	Protein Coding	40
UCHL3	Ubiquitin C-Terminal Hydrolase L3	Protein Coding	44
MRPS30	Mitochondrial Ribosomal Protein S30	Protein Coding	36
INKA2	Inka Box Actin Regulator 2	Protein Coding	26
RHOU	Ras Homolog Family Member U	Protein Coding	38
RRAGA	Ras Related GTP Binding A	Protein Coding	39
ID2	Inhibitor Of DNA Binding 2	Protein Coding	45
LRP10	LDL Receptor Related Protein 10	Protein Coding	39
HCST	Hematopoietic Cell Signal Transducer	Protein Coding	40
PLK2	Polo Like Kinase 2	Protein Coding	44
FER	FER Tyrosine Kinase	Protein Coding	48
MIR5193	MicroRNA 5193	RNA Gene	14
JKAMP	JNK1/MAPK8 Associated Membrane Protein	Protein Coding	35
ORC3	Origin Recognition Complex Subunit 3	Protein Coding	38
GLRX2	Glutaredoxin 2	Protein Coding	39
MTPN	Myotrophin	Protein Coding	36
SCNM1	Sodium Channel Modifier 1	Protein Coding	33
TEAD4	TEA Domain Transcription Factor 4	Protein Coding	43
LACRT	Lacritin	Protein Coding	33
GRK3	G Protein-Coupled Receptor Kinase 3	Protein Coding	40

ATRNL1	Attractin Like 1	Protein Coding	36
SLC8A2	Solute Carrier Family 8 Member A2	Protein Coding	41
PDK2	Pyruvate Dehydrogenase Kinase 2	Protein Coding	44
MT-TR	Mitochondrially Encoded TRNA-Arg (CGN)	RNA Gene	12
RYK	Receptor Like Tyrosine Kinase	Protein Coding	43
LOC10866	Calcium Voltage-Gated Channel Subunit Alpha1 A Rep	Biological Region	2
RBM19	RNA Binding Motif Protein 19	Protein Coding	36
PUSL1	Pseudouridine Synthase Like 1	Protein Coding	33
CLCF1	Cardiotrophin Like Cytokine Factor 1	Protein Coding	42
TUBB7P	Tubulin Beta 7 Pseudogene	Pseudogene	18
WASL	WASP Like Actin Nucleation Promoting Factor	Protein Coding	43
SIX2	SIX Homeobox 2	Protein Coding	40
AS3MT	Arsenite Methyltransferase	Protein Coding	41
CD74	CD74 Molecule	Protein Coding	44
LIX1	Limb And CNS Expressed 1	Protein Coding	34
RPL36A	Ribosomal Protein L36a	Protein Coding	36
ZNF526	Zinc Finger Protein 526	Protein Coding	36
MIRLET7.	MicroRNA Let-7a-3	RNA Gene	20
OTUB1	OTU Deubiquitinase, Ubiquitin Aldehyde Binding 1	Protein Coding	40
ATP6V1E.	ATPase H+ Transporting V1 Subunit E2	Protein Coding	39
GPR156	G Protein-Coupled Receptor 156	Protein Coding	39
PRORP	Protein Only RNase P Catalytic Subunit	Protein Coding	28
FUBP1	Far Upstream Element Binding Protein 1	Protein Coding	40
IRF2	Interferon Regulatory Factor 2	Protein Coding	45
SIAH2	Siah E3 Ubiquitin Protein Ligase 2	Protein Coding	45
ETS2	ETS Proto-Oncogene 2, Transcription Factor	Protein Coding	43
PPP1R15A	Protein Phosphatase 1 Regulatory Subunit 15A	Protein Coding	41
RPL37A	Ribosomal Protein L37a	Protein Coding	40
SEPTIN14	Septin 14	Protein Coding	24
AP2A2	Adaptor Related Protein Complex 2 Subunit Alpha 2	Protein Coding	38
MT-TM	Mitochondrially Encoded TRNA-Met (AUA/G)	RNA Gene	12
COX7A2L	Cytochrome C Oxidase Subunit 7A2 Like	Protein Coding	41
CENPC	Centromere Protein C	Protein Coding	35
UBXN6	UBX Domain Protein 6	Protein Coding	39
KCTD4	Potassium Channel Tetramerization Domain Containing	Protein Coding	34
NPFF	Neuropeptide FF-Amide Peptide Precursor	Protein Coding	35
JUND	JunD Proto-Oncogene, AP-1 Transcription Factor Subu	Protein Coding	43
MRPL28	Mitochondrial Ribosomal Protein L28	Protein Coding	39
PDYN-AS	PDYN Antisense RNA 1	RNA Gene	12
RER1	Retention In Endoplasmic Reticulum Sorting Receptor 1	Protein Coding	36
FDX1	Ferredoxin 1	Protein Coding	41
PHLDA1	Pleckstrin Homology Like Domain Family A Member 1	Protein Coding	37
YRDC	YrdC N6-Threonylcarbamoyltransferase Domain Conta	Protein Coding	33
PMF1	Polyamine Modulated Factor 1	Protein Coding	38
PRRC2B	Proline Rich Coiled-Coil 2B	Protein Coding	35
LIMK2	LIM Domain Kinase 2	Protein Coding	49
STAU1	Staufen Double-Stranded RNA Binding Protein 1	Protein Coding	39

DNALI1	Dynein Axonemal Light Intermediate Chain 1	Protein Coding	39
RNF41	Ring Finger Protein 41	Protein Coding	41
DRAP1	DR1 Associated Protein 1	Protein Coding	39
RPS4Y2	Ribosomal Protein S4 Y-Linked 2	Protein Coding	30
MAP3K14	Mitogen-Activated Protein Kinase Kinase Kinase 14	Protein Coding	45
THTPA	Thiamine Triphosphatase	Protein Coding	37
CACYBP	Calcyclin Binding Protein	Protein Coding	41
RILPL1	Rab Interacting Lysosomal Protein Like 1	Protein Coding	36
MATK	Megakaryocyte-Associated Tyrosine Kinase	Protein Coding	47
RSL24D1	Ribosomal L24 Domain Containing 1	Protein Coding	39
DDX20	DEAD-Box Helicase 20	Protein Coding	43
TUSC7	Tumor Suppressor Candidate 7	RNA Gene	18
ANGPTL7	Angiopoietin Like 7	Protein Coding	40
SLC17A9	Solute Carrier Family 17 Member 9	Protein Coding	39
CHCHD6	Coiled-Coil-Helix-Coiled-Coil-Helix Domain Containin	Protein Coding	37
ZNRF3	Zinc And Ring Finger 3	Protein Coding	36
TYW1B	TRNA-YW Synthesizing Protein 1 Homolog B	Protein Coding	31
BBC3	BCL2 Binding Component 3	Protein Coding	43
UQCRH	Ubiquinol-Cytochrome C Reductase Hinge Protein	Protein Coding	40
GDI2	GDP Dissociation Inhibitor 2	Protein Coding	41
KCNS2	Potassium Voltage-Gated Channel Modifier Subfamily	Protein Coding	40
CCNG1	Cyclin G1	Protein Coding	43
PPP1CC	Protein Phosphatase 1 Catalytic Subunit Gamma	Protein Coding	46
SLC3A2	Solute Carrier Family 3 Member 2	Protein Coding	43
HOXB7	Homeobox B7	Protein Coding	41
ACSM3	Acyl-CoA Synthetase Medium Chain Family Member 3	Protein Coding	40
RHBDF1	Rhomboid 5 Homolog 1	Protein Coding	39
RFX4	Regulatory Factor X4	Protein Coding	38
ATP1B1	ATPase Na+/K+ Transporting Subunit Beta 1	Protein Coding	48
GNA15	G Protein Subunit Alpha 15	Protein Coding	41
PSMD8	Proteasome 26S Subunit, Non-ATPase 8	Protein Coding	41
SOGA3	SOGA Family Member 3	Protein Coding	33
RGS5	Regulator Of G Protein Signaling 5	Protein Coding	41
GPRC6A	G Protein-Coupled Receptor Class C Group 6 Member	Protein Coding	40
GTF3C6	General Transcription Factor IIIC Subunit 6	Protein Coding	34
KPNA1	Karyopherin Subunit Alpha 1	Protein Coding	43
NOC2L	NOC2 Like Nucleolar Associated Transcriptional Repr	Protein Coding	39
SRRT	Serrate, RNA Effector Molecule	Protein Coding	39
GOT2	Glutamic-Oxaloacetic Transaminase 2	Protein Coding	45
NLRP2	NLR Family Pyrin Domain Containing 2	Protein Coding	43
SECISBP2	SECIS Binding Protein 2	Protein Coding	44
LETMD1	LETM1 Domain Containing 1	Protein Coding	37
CAND1	Cullin Associated And Neddylation Dissociated 1	Protein Coding	38
XRN1	5'-3' Exoribonuclease 1	Protein Coding	36
COIL	Coilin	Protein Coding	41
MIR497H	Mir-497-195 Cluster Host Gene	RNA Gene	14
MIR4448	MicroRNA 4448	RNA Gene	13

BCL2L2	BCL2 Like 2	Protein Coding	45
PLEKHG2	Pleckstrin Homology And RhoGEF Domain Containing	Protein Coding	39
MED22	Mediator Complex Subunit 22	Protein Coding	38
SAMM50	SAMM50 Sorting And Assembly Machinery Componer	Protein Coding	38
MIR505	MicroRNA 505	RNA Gene	17
GNA12	G Protein Subunit Alpha 12	Protein Coding	43
MAPRE1	Microtubule Associated Protein RP/EB Family Member	Protein Coding	45
TXNRD1	Thioredoxin Reductase 1	Protein Coding	48
NDUFA4L	NDUFA4 Mitochondrial Complex Associated Like 2	Protein Coding	38
DDX10	DEAD-Box Helicase 10	Protein Coding	40
KRT6C	Keratin 6C	Protein Coding	39
AHCYL1	Adenosylhomocysteinase Like 1	Protein Coding	44
NDUFC1	NADH:Ubiquinone Oxidoreductase Subunit C1	Protein Coding	36
CHL1	Cell Adhesion Molecule L1 Like	Protein Coding	40
PRSS12	Serine Protease 12	Protein Coding	41
STYX	Serine/Threonine/Tyrosine Interacting Protein	Protein Coding	37
MICALL2	MICAL Like 2	Protein Coding	36
UBFD1	Ubiquitin Family Domain Containing 1	Protein Coding	35
TLE3	TLE Family Member 3, Transcriptional Corepressor	Protein Coding	45
SFRP5	Secreted Frizzled Related Protein 5	Protein Coding	40
PDZK1	PDZ Domain Containing 1	Protein Coding	41
GUCY1B1	Guanylate Cyclase 1 Soluble Subunit Beta 1	Protein Coding	35
FTSJ3	FtsJ RNA 2'-O-Methyltransferase 3	Protein Coding	38
ARHGAP2	Rho GTPase Activating Protein 27	Protein Coding	37
TOMM70	Translocase Of Outer Mitochondrial Membrane 70	Protein Coding	31
MCU	Mitochondrial Calcium Uniporter	Protein Coding	36
SART3	Spliceosome Associated Factor 3, U4/U6 Recycling Pro	Protein Coding	37
DYNLT1	Dynein Light Chain Tctex-Type 1	Protein Coding	39
WNT9B	Wnt Family Member 9B	Protein Coding	42
RUFY3	RUN And FYVE Domain Containing 3	Protein Coding	35
PAM	Peptidylglycine Alpha-Amidating Monooxygenase	Protein Coding	44
NBR1	NBR1 Autophagy Cargo Receptor	Protein Coding	40
CISD1	CDGSH Iron Sulfur Domain 1	Protein Coding	39
RPL12	Ribosomal Protein L12	Protein Coding	43
EIF3I	Eukaryotic Translation Initiation Factor 3 Subunit I	Protein Coding	40
ILF2	Interleukin Enhancer Binding Factor 2	Protein Coding	39
FAM111B	Family With Sequence Similarity 111 Member B	Protein Coding	37
AMOTL2	Angiomotin Like 2	Protein Coding	37
STOM	Stomatin	Protein Coding	40
SPSB2	SplA/Ryanodine Receptor Domain And SOCS Box Cor	Protein Coding	37
NBPF3	NBPF Member 3	Protein Coding	35
STK24	Serine/Threonine Kinase 24	Protein Coding	45
PCSK6	Proprotein Convertase Subtilisin/Kexin Type 6	Protein Coding	41
GET4	Guided Entry Of Tail-Anchored Proteins Factor 4	Protein Coding	35
FNDC3B	Fibronectin Type III Domain Containing 3B	Protein Coding	37
AGPAT1	1-Acylglycerol-3-Phosphate O-Acyltransferase 1	Protein Coding	42
CLASRP	CLK4 Associating Serine/Arginine Rich Protein	Protein Coding	33

MGLL	Monoglyceride Lipase	Protein Coding	46
MIR520D	MicroRNA 520d	RNA Gene	17
ADARB1	Adenosine Deaminase RNA Specific B1	Protein Coding	44
NOC3L	NOC3 Like DNA Replication Regulator	Protein Coding	35
LYAR	Ly1 Antibody Reactive	Protein Coding	39
TNFAIP8I	TNF Alpha Induced Protein 8 Like 2	Protein Coding	35
ATP5MC1	ATP Synthase Membrane Subunit C Locus 1	Protein Coding	31
KCNIP2	Potassium Voltage-Gated Channel Interacting Protein 2	Protein Coding	41
VPS4B	Vacuolar Protein Sorting 4 Homolog B	Protein Coding	43
MIR124-3	MicroRNA 124-3	RNA Gene	18
STAMBP	STAM Binding Protein	Protein Coding	47
PSME2	Proteasome Activator Subunit 2	Protein Coding	44
TSC22D3	TSC22 Domain Family Member 3	Protein Coding	41
SAGE1	Sarcoma Antigen 1	Protein Coding	31
ODF2L	Outer Dense Fiber Of Sperm Tails 2 Like	Protein Coding	36
RAD23A	RAD23 Homolog A, Nucleotide Excision Repair Protei	Protein Coding	44
STAC	SH3 And Cysteine Rich Domain	Protein Coding	39
MPZL3	Myelin Protein Zero Like 3	Protein Coding	37
HOMER2	Homer Scaffold Protein 2	Protein Coding	43
BSPH1	Binder Of Sperm Protein Homolog 1	Protein Coding	27
ZWINT	ZW10 Interacting Kinetochore Protein	Protein Coding	38
PKDCC	Protein Kinase Domain Containing, Cytoplasmic	Protein Coding	37
FAM120A	Family With Sequence Similarity 120A	Protein Coding	39
P2RY6	Pyrimidinergic Receptor P2Y6	Protein Coding	43
NLGN2	Neuroligin 2	Protein Coding	40
PFKFB3	6-Phosphofructo-2-Kinase/Fructose-2,6-Biphosphatase	Protein Coding	46
FBXO42	F-Box Protein 42	Protein Coding	34
SLC22A6	Solute Carrier Family 22 Member 6	Protein Coding	45
MCEE	Methylmalonyl-CoA Epimerase	Protein Coding	43
TMED2	Transmembrane P24 Trafficking Protein 2	Protein Coding	37
EFNA1	Ephrin A1	Protein Coding	44
RGS10	Regulator Of G Protein Signaling 10	Protein Coding	44
SLC22A1	Solute Carrier Family 22 Member 1	Protein Coding	44
PWP2	PWP2 Small Subunit Processome Component	Protein Coding	36
PPP6R3	Protein Phosphatase 6 Regulatory Subunit 3	Protein Coding	37
TMC3-AS	TMC3 Antisense RNA 1	RNA Gene	12
VASP	Vasodilator Stimulated Phosphoprotein	Protein Coding	44
BARX1	BARX Homeobox 1	Protein Coding	38
MTERF1	Mitochondrial Transcription Termination Factor 1	Protein Coding	36
WDR48	WD Repeat Domain 48	Protein Coding	39
REEP4	Receptor Accessory Protein 4	Protein Coding	37
EP400P1	EP400 Pseudogene 1	Pseudogene	19
UPF1	UPF1 RNA Helicase And ATPase	Protein Coding	41
EIF3B	Eukaryotic Translation Initiation Factor 3 Subunit B	Protein Coding	39
EPHB3	EPH Receptor B3	Protein Coding	46
NR2C2	Nuclear Receptor Subfamily 2 Group C Member 2	Protein Coding	44
HSPB6	Heat Shock Protein Family B (Small) Member 6	Protein Coding	40

IRGC	Immunity Related GTPase Cinema	Protein Coding	37
MGST1	Microsomal Glutathione S-Transferase 1	Protein Coding	44
NEIL1	Nei Like DNA Glycosylase 1	Protein Coding	40
NFKBIB	NFKB Inhibitor Beta	Protein Coding	42
H4C9	H4 Clustered Histone 9	Protein Coding	31
CAMKMT	Calmodulin-Lysine N-Methyltransferase	Protein Coding	37
PHF5GP	PHD Finger Protein 5G Pseudogene	Pseudogene	6
SNAP23	Synaptosome Associated Protein 23	Protein Coding	45
RPIA	Ribose 5-Phosphate Isomerase A	Protein Coding	44
RFC5	Replication Factor C Subunit 5	Protein Coding	41
CASP5	Caspase 5	Protein Coding	45
H1-0	H1.0 Linker Histone	Protein Coding	33
MRPL19	Mitochondrial Ribosomal Protein L19	Protein Coding	38
FABP5	Fatty Acid Binding Protein 5	Protein Coding	43
PMEPA1	Prostate Transmembrane Protein, Androgen Induced 1	Protein Coding	39
RING1	Ring Finger Protein 1	Protein Coding	41
PAK5	P21 (RAC1) Activated Kinase 5	Protein Coding	38
AP1G1	Adaptor Related Protein Complex 1 Subunit Gamma 1	Protein Coding	40
DDX54	DEAD-Box Helicase 54	Protein Coding	38
RPAP3	RNA Polymerase II Associated Protein 3	Protein Coding	34
SART1	Spliceosome Associated Factor 1, Recruiter Of U4/U6.U	Protein Coding	41
MRPL11	Mitochondrial Ribosomal Protein L11	Protein Coding	39
GRPR	Gastrin Releasing Peptide Receptor	Protein Coding	43
PRDX4	Peroxiredoxin 4	Protein Coding	44
UBE2M	Ubiquitin Conjugating Enzyme E2 M	Protein Coding	43
BCAT1	Branched Chain Amino Acid Transaminase 1	Protein Coding	48
CRYZ	Crystallin Zeta	Protein Coding	44
TFPT	TCF3 Fusion Partner	Protein Coding	36
ARHGEF7	Rho Guanine Nucleotide Exchange Factor 7	Protein Coding	43
FNIP1	Folliculin Interacting Protein 1	Protein Coding	37
CNOT7	CCR4-NOT Transcription Complex Subunit 7	Protein Coding	41
MIR7-2	MicroRNA 7-2	RNA Gene	17
KPNA4	Karyopherin Subunit Alpha 4	Protein Coding	43
TRIM31	Tripartite Motif Containing 31	Protein Coding	39
MRM2	Mitochondrial RRNA Methyltransferase 2	Protein Coding	32
CPT1B	Carnitine Palmitoyltransferase 1B	Protein Coding	45
ATP8B2	ATPase Phospholipid Transporting 8B2	Protein Coding	40
DNAJA2	DnaJ Heat Shock Protein Family (Hsp40) Member A2	Protein Coding	40
OXLD1	Oxidoreductase Like Domain Containing 1	Protein Coding	29
ZNF385B	Zinc Finger Protein 385B	Protein Coding	37
ACP2	Acid Phosphatase 2, Lysosomal	Protein Coding	44
RANGRF	RAN Guanine Nucleotide Release Factor	Protein Coding	37
DPH6	Diphthamine Biosynthesis 6	Protein Coding	35
BRF1	BRF1 RNA Polymerase III Transcription Initiation Fact	Protein Coding	42
SCARA5	Scavenger Receptor Class A Member 5	Protein Coding	40
GFRA4	GDNF Family Receptor Alpha 4	Protein Coding	34
GSTK1	Glutathione S-Transferase Kappa 1	Protein Coding	41

TCEANC2	Transcription Elongation Factor A N-Terminal And Ce	Protein Coding	33
TRIB2	Tribbles Pseudokinase 2	Protein Coding	39
GART	Phosphoribosylglycinamide Formyltransferase, Phospho	Protein Coding	43
NDEL1	NudE Neurodevelopment Protein 1 Like 1	Protein Coding	43
PSME1	Proteasome Activator Subunit 1	Protein Coding	42
LNX1	Ligand Of Numb-Protein X 1	Protein Coding	43
SGSM1	Small G Protein Signaling Modulator 1	Protein Coding	37
CLTA	Clathrin Light Chain A	Protein Coding	42
TREML4	Triggering Receptor Expressed On Myeloid Cells Like	Protein Coding	32
H3C14	H3 Clustered Histone 14	Protein Coding	29
KCNAB3	Potassium Voltage-Gated Channel Subfamily A Regula	Protein Coding	40
DDX6	DEAD-Box Helicase 6	Protein Coding	46
MPP3	Membrane Palmitoylated Protein 3	Protein Coding	39
ADAMTS	ADAM Metallopeptidase With Thrombospondin Type 1	Protein Coding	38
GADD45C	GADD45G Interacting Protein 1	Protein Coding	36
MX2	MX Dynamin Like GTPase 2	Protein Coding	40
SYT17	Synaptotagmin 17	Protein Coding	35
HBZ	Hemoglobin Subunit Zeta	Protein Coding	40
AGO1	Argonaute RISC Component 1	Protein Coding	38
DNAJC30	DnaJ Heat Shock Protein Family (Hsp40) Member C30	Protein Coding	35
AEBP2	AE Binding Protein 2	Protein Coding	39
DEFA6	Defensin Alpha 6	Protein Coding	37
XIRP2	Xin Actin Binding Repeat Containing 2	Protein Coding	36
GNAZ	G Protein Subunit Alpha Z	Protein Coding	44
ATP5MC2	ATP Synthase Membrane Subunit C Locus 2	Protein Coding	30
SLC6A15	Solute Carrier Family 6 Member 15	Protein Coding	41
HAGLROS	HAGLR Opposite Strand LncRNA	RNA Gene	14
GLS2	Glutaminase 2	Protein Coding	42
ATP13A4	ATPase 13A4	Protein Coding	36
TBC1D15	TBC1 Domain Family Member 15	Protein Coding	39
KIF5B	Kinesin Family Member 5B	Protein Coding	44
MIR129-1	MicroRNA 129-1	RNA Gene	18
NSL1	NSL1 Component Of MIS12 Kinetochore Complex	Protein Coding	37
SAR1A	Secretion Associated Ras Related GTPase 1A	Protein Coding	42
CAMK1	Calcium/Calmodulin Dependent Protein Kinase I	Protein Coding	44
SPRR2D	Small Proline Rich Protein 2D	Protein Coding	31
CDC7	Cell Division Cycle 7	Protein Coding	45
GUK1	Guanylate Kinase 1	Protein Coding	43
RAPGEF2	Rap Guanine Nucleotide Exchange Factor 2	Protein Coding	43
KBTBD11	Kelch Repeat And BTB Domain Containing 11	Protein Coding	34
TAF9B	TATA-Box Binding Protein Associated Factor 9b	Protein Coding	37
NMD3	NMD3 Ribosome Export Adaptor	Protein Coding	36
CDIPT	CDP-Diacylglycerol--Inositol 3-Phosphatidyltransferase	Protein Coding	42
HES6	Hes Family BHLH Transcription Factor 6	Protein Coding	40
ERC1	ELKS/RAB6-Interacting/CAST Family Member 1	Protein Coding	42
GLRX3	Glutaredoxin 3	Protein Coding	41
PHF7	PHD Finger Protein 7	Protein Coding	36

RPS23P3	Ribosomal Protein S23 Pseudogene 3	Pseudogene	6
HDLBP	High Density Lipoprotein Binding Protein	Protein Coding	40
MIDN	Midnolin	Protein Coding	35
SRP14	Signal Recognition Particle 14	Protein Coding	39
MCFD2	Multiple Coagulation Factor Deficiency 2, ER Cargo R	Protein Coding	44
SMG1	SMG1 Nonsense Mediated MRNA Decay Associated P	Protein Coding	42
MAGED1	MAGE Family Member D1	Protein Coding	44
MYL12A	Myosin Light Chain 12A	Protein Coding	40
RNF121	Ring Finger Protein 121	Protein Coding	34
CDO1	Cysteine Dioxygenase Type 1	Protein Coding	43
TUBGCP5	Tubulin Gamma Complex Associated Protein 5	Protein Coding	34
ZC3HC1	Zinc Finger C3HC-Type Containing 1	Protein Coding	39
RGS14	Regulator Of G Protein Signaling 14	Protein Coding	45
SP2	Sp2 Transcription Factor	Protein Coding	40
APOBEC3	Apolipoprotein B MRNA Editing Enzyme Catalytic Sul	Protein Coding	37
RBM33	RNA Binding Motif Protein 33	Protein Coding	33
EEF2K	Eukaryotic Elongation Factor 2 Kinase	Protein Coding	48
NPY5R	Neuropeptide Y Receptor Y5	Protein Coding	43
NOP58	NOP58 Ribonucleoprotein	Protein Coding	39
CCNT2	Cyclin T2	Protein Coding	40
APEX2	Apurinic/Apyrimidinic Endodeoxyribonuclease 2	Protein Coding	37
MRPS6	Mitochondrial Ribosomal Protein S6	Protein Coding	38
USP39	Ubiquitin Specific Peptidase 39	Protein Coding	39
YPEL5	Yippee Like 5	Protein Coding	37
CAPZB	Capping Actin Protein Of Muscle Z-Line Subunit Beta	Protein Coding	41
KLHDC4	Kelch Domain Containing 4	Protein Coding	37
DSTN	Destrin, Actin Depolymerizing Factor	Protein Coding	40
MRPS28	Mitochondrial Ribosomal Protein S28	Protein Coding	39
LY86	Lymphocyte Antigen 86	Protein Coding	39
PPP3CC	Protein Phosphatase 3 Catalytic Subunit Gamma	Protein Coding	44
UBE2S	Ubiquitin Conjugating Enzyme E2 S	Protein Coding	43
NAA25	N-Alpha-Acetyltransferase 25, NatB Auxiliary Subunit	Protein Coding	36
SOCS6	Suppressor Of Cytokine Signaling 6	Protein Coding	42
CSN3	Casein Kappa	Protein Coding	36
HGFAC	HGF Activator	Protein Coding	42
SIPA1L1	Signal Induced Proliferation Associated 1 Like 1	Protein Coding	39
EXD2	Exonuclease 3'-5' Domain Containing 2	Protein Coding	33
BHMT2	Betaine--Homocysteine S-Methyltransferase 2	Protein Coding	43
TC2N	Tandem C2 Domains, Nuclear	Protein Coding	35
PDCD2	Programmed Cell Death 2	Protein Coding	37
MARCHF:	Membrane Associated Ring-CH-Type Finger 3	Protein Coding	28
KPTN	Kaptin, Actin Binding Protein	Protein Coding	38
CKAP4	Cytoskeleton Associated Protein 4	Protein Coding	39
ASGR1	Asialoglycoprotein Receptor 1	Protein Coding	41
ZBTB8OS	Zinc Finger And BTB Domain Containing 8 Opposite S	Protein Coding	36
SLIT1	Slit Guidance Ligand 1	Protein Coding	43
SRSF10	Serine And Arginine Rich Splicing Factor 10	Protein Coding	37

ABCA5	ATP Binding Cassette Subfamily A Member 5	Protein Coding	44
IGSF5	Immunoglobulin Superfamily Member 5	Protein Coding	33
POLL	DNA Polymerase Lambda	Protein Coding	44
LRRC20	Leucine Rich Repeat Containing 20	Protein Coding	37
RMC1	Regulator Of MON1-CCZ1	Protein Coding	25
CCNA1	Cyclin A1	Protein Coding	44
MDM4	MDM4 Regulator Of P53	Protein Coding	44
DDX27	DEAD-Box Helicase 27	Protein Coding	37
TIMM44	Translocase Of Inner Mitochondrial Membrane 44	Protein Coding	39
PPP2CB	Protein Phosphatase 2 Catalytic Subunit Beta	Protein Coding	47
P2RX4	Purinergic Receptor P2X 4	Protein Coding	44
DHFRP2	Dihydrofolate Reductase Pseudogene 2	Pseudogene	12
ID3	Inhibitor Of DNA Binding 3, HLH Protein	Protein Coding	43
LDB1	LIM Domain Binding 1	Protein Coding	40
VRK3	VRK Serine/Threonine Kinase 3	Protein Coding	41
C17orf80	Chromosome 17 Open Reading Frame 80	Protein Coding	33
ZNF23	Zinc Finger Protein 23	Protein Coding	40
MRTO4	MRT4 Homolog, Ribosome Maturation Factor	Protein Coding	35
REEP3	Receptor Accessory Protein 3	Protein Coding	37
RAB35	RAB35, Member RAS Oncogene Family	Protein Coding	42
ACYP1	Acylphosphatase 1	Protein Coding	40
TBC1D22	TBC1 Domain Family Member 22A	Protein Coding	36
ULK4	Unc-51 Like Kinase 4	Protein Coding	41
CCDC60	Coiled-Coil Domain Containing 60	Protein Coding	33
DIO3	Iodothyronine Deiodinase 3	Protein Coding	41
SUPV3L1	Suv3 Like RNA Helicase	Protein Coding	39
HCAR2	Hydroxycarboxylic Acid Receptor 2	Protein Coding	39
MANEA	Mannosidase Endo-Alpha	Protein Coding	37
VASH2	Vasohibin 2	Protein Coding	36
UXT	Ubiquitously Expressed Prefoldin Like Chaperone	Protein Coding	36
UBE2I	Ubiquitin Conjugating Enzyme E2 I	Protein Coding	50
BTC	Betacellulin	Protein Coding	45
TNPO2	Transportin 2	Protein Coding	37
ATP5MC3	ATP Synthase Membrane Subunit C Locus 3	Protein Coding	31
CCDC190	Coiled-Coil Domain Containing 190	Protein Coding	23
GAS2	Growth Arrest Specific 2	Protein Coding	40
SEC16B	SEC16 Homolog B, Endoplasmic Reticulum Export Fa	Protein Coding	38
HIBADH	3-Hydroxyisobutyrate Dehydrogenase	Protein Coding	42
LIN28A	Lin-28 Homolog A	Protein Coding	42
ITIH3	Inter-Alpha-Trypsin Inhibitor Heavy Chain 3	Protein Coding	41
STARD3N	STARD3 N-Terminal Like	Protein Coding	37
UXS1	UDP-Glucuronate Decarboxylase 1	Protein Coding	41
DNAJC17	DnaJ Heat Shock Protein Family (Hsp40) Member C17	Protein Coding	37
AK8	Adenylate Kinase 8	Protein Coding	40
FKBP15	FKBP Prolyl Isomerase 15	Protein Coding	35
DIMT1	DIMT1 RRNA Methyltransferase And Ribosome Matu	Protein Coding	37
COTL1	Coactosin Like F-Actin Binding Protein 1	Protein Coding	41

HAGH	Hydroxyacylglutathione Hydrolase	Protein Coding	43
AKR1B10	Aldo-Keto Reductase Family 1 Member B10	Protein Coding	45
ARF6	ADP Ribosylation Factor 6	Protein Coding	46
TCEAL2	Transcription Elongation Factor A Like 2	Protein Coding	31
PPP2R2C	Protein Phosphatase 2 Regulatory Subunit Bgamma	Protein Coding	43
GRIN3A	Glutamate Ionotropic Receptor NMDA Type Subunit 3.	Protein Coding	38
TMEM12(	Transmembrane Protein 120A	Protein Coding	35
OSGEPL1	O-Sialoglycoprotein Endopeptidase Like 1	Protein Coding	36
TCP10	T-Complex 10	Pseudogene	32
TGIF2	TGFB Induced Factor Homeobox 2	Protein Coding	40
MTREX	Mtr4 Exosome RNA Helicase	Protein Coding	31
ZSWIM7	Zinc Finger SWIM-Type Containing 7	Protein Coding	32
TNFAIP8I	TNF Alpha Induced Protein 8 Like 1	Protein Coding	32
MAFK	MAF BZIP Transcription Factor K	Protein Coding	37
MTSS1	MTSS I-BAR Domain Containing 1	Protein Coding	39
SMURF1	SMAD Specific E3 Ubiquitin Protein Ligase 1	Protein Coding	47
TCEA1	Transcription Elongation Factor A1	Protein Coding	39
HSPH1	Heat Shock Protein Family H (Hsp110) Member 1	Protein Coding	44
RPL31	Ribosomal Protein L31	Protein Coding	43
VAC14-AS	VAC14 Antisense RNA 1	RNA Gene	14
WDR77	WD Repeat Domain 77	Protein Coding	40
CFAP20	Cilia And Flagella Associated Protein 20	Protein Coding	33
WNT9A	Wnt Family Member 9A	Protein Coding	42
LOC64338	TAR DNA Binding Protein Pseudogene	Pseudogene	7
RPS6KA5	Ribosomal Protein S6 Kinase A5	Protein Coding	47
PTMA	Prothymosin Alpha	Protein Coding	40
DHX15	DEAH-Box Helicase 15	Protein Coding	39
GTPBP1	GTP Binding Protein 1	Protein Coding	39
CPEB1	Cytoplasmic Polyadenylation Element Binding Protein 1	Protein Coding	41
ARHGAP1	Rho GTPase Activating Protein 15	Protein Coding	41
NEIL2	Nei Like DNA Glycosylase 2	Protein Coding	40
SESN1	Sestrin 1	Protein Coding	41
PXMP2	Peroxisomal Membrane Protein 2	Protein Coding	38
ENOPH1	Enolase-Phosphatase 1	Protein Coding	40
LOC10783	SIRT1 Promoter Region	Biological Region	2
MAP1LC3	Microtubule Associated Protein 1 Light Chain 3 Beta	Protein Coding	44
RPL17	Ribosomal Protein L17	Protein Coding	39
GLCE	Glucuronic Acid Epimerase	Protein Coding	41
MBD1	Methyl-CpG Binding Domain Protein 1	Protein Coding	41
TSBP1-AS	TSBP1 And BTNL2 Antisense RNA 1	RNA Gene	9
IMPAD1	Inositol Monophosphatase Domain Containing 1	Protein Coding	41
SLC7A11	Solute Carrier Family 7 Member 11	Protein Coding	45
GIMAP4	GTPase, IMAP Family Member 4	Protein Coding	40
RRP12	Ribosomal RNA Processing 12 Homolog	Protein Coding	36
PARVA	Parvin Alpha	Protein Coding	45
TARBP2	TARBP2 Subunit Of RISC Loading Complex	Protein Coding	43
PRR14	Proline Rich 14	Protein Coding	33

SDR39U1	Short Chain Dehydrogenase/Reductase Family 39U Me	Protein Coding	35
BDH1	3-Hydroxybutyrate Dehydrogenase 1	Protein Coding	44
NME2	NME/NM23 Nucleoside Diphosphate Kinase 2	Protein Coding	48
CNTNAP4	Contactin Associated Protein Family Member 4	Protein Coding	37
ATP1B2	ATPase Na+/K+ Transporting Subunit Beta 2	Protein Coding	42
MAPRE1P	MAPRE1 Pseudogene 1	Pseudogene	8
PSMD10	Proteasome 26S Subunit, Non-ATPase 10	Protein Coding	41
RPL26P19	Ribosomal Protein L26 Pseudogene 19	Pseudogene	9
YOD1	YOD1 Deubiquitinase	Protein Coding	38
USP15	Ubiquitin Specific Peptidase 15	Protein Coding	48
AKIRIN2	Akirin 2	Protein Coding	35
MED27	Mediator Complex Subunit 27	Protein Coding	37
HSPA14	Heat Shock Protein Family A (Hsp70) Member 14	Protein Coding	40
ZNF574	Zinc Finger Protein 574	Protein Coding	33
NUBP1	Nucleotide Binding Protein 1	Protein Coding	41
STK3	Serine/Threonine Kinase 3	Protein Coding	47
OSBPL8	Oxysterol Binding Protein Like 8	Protein Coding	39
CLIP3	CAP-Gly Domain Containing Linker Protein 3	Protein Coding	37
RABIF	RAB Interacting Factor	Protein Coding	39
ENDOG	Endonuclease G	Protein Coding	44
MTOR-AS	MTOR Antisense RNA 1	RNA Gene	14
TRMT61B	TRNA Methyltransferase 61B	Protein Coding	34
CLK2	CDC Like Kinase 2	Protein Coding	42
B3GALT2	Beta-1,3-Galactosyltransferase 2	Protein Coding	36
VTA1	Vesicle Trafficking 1	Protein Coding	39
ABHD11	Abhydrolase Domain Containing 11	Protein Coding	38
PDIA4	Protein Disulfide Isomerase Family A Member 4	Protein Coding	43
STK19	Serine/Threonine Kinase 19	Protein Coding	39
EIF3FP3	Eukaryotic Translation Initiation Factor 3 Subunit F Ps	Pseudogene	8
CALCOC(	Calcium Binding And Coiled-Coil Domain 2	Protein Coding	40
C11orf58	Chromosome 11 Open Reading Frame 58	Protein Coding	37
CSRP1	Cysteine And Glycine Rich Protein 1	Protein Coding	43
PPP1R9B	Protein Phosphatase 1 Regulatory Subunit 9B	Protein Coding	40
NMNAT2	Nicotinamide Nucleotide Adenylyltransferase 2	Protein Coding	43
UQCR11	Ubiquinol-Cytochrome C Reductase, Complex III Subu	Protein Coding	37
S100A16	S100 Calcium Binding Protein A16	Protein Coding	37
FERD3L	Fer3 Like BHLH Transcription Factor	Protein Coding	33
FBXW5	F-Box And WD Repeat Domain Containing 5	Protein Coding	37
GABRA4	Gamma-Aminobutyric Acid Type A Receptor Subunit A	Protein Coding	45
WDR5	WD Repeat Domain 5	Protein Coding	45
GABPA	GA Binding Protein Transcription Factor Subunit Alpha	Protein Coding	39
C1orf105	Chromosome 1 Open Reading Frame 105	Protein Coding	32
TOM1	Target Of Myb1 Membrane Trafficking Protein	Protein Coding	43
BCAT2	Branched Chain Amino Acid Transaminase 2	Protein Coding	45
IFNK	Interferon Kappa	Protein Coding	39
H1-5	H1.5 Linker Histone, Cluster Member	Protein Coding	33
MDGA2	MAM Domain Containing Glycosylphosphatidylinositol	Protein Coding	37

DGKD	Diacylglycerol Kinase Delta	Protein Coding	47
TENT5D	Terminal Nucleotidyltransferase 5D	Protein Coding	26
CKAP2	Cytoskeleton Associated Protein 2	Protein Coding	37
PDIA6	Protein Disulfide Isomerase Family A Member 6	Protein Coding	41
MRPL14	Mitochondrial Ribosomal Protein L14	Protein Coding	37
USP1	Ubiquitin Specific Peptidase 1	Protein Coding	45
GIMAP5	GTPase, IMAP Family Member 5	Protein Coding	38
MAGI1	Membrane Associated Guanylate Kinase, WW And PD	Protein Coding	41
ETV3	ETS Variant Transcription Factor 3	Protein Coding	36
CCDC59	Coiled-Coil Domain Containing 59	Protein Coding	35
RABGGT	Rab Geranylgeranyltransferase Subunit Alpha	Protein Coding	41
MRPL21	Mitochondrial Ribosomal Protein L21	Protein Coding	36
VTRNA2-	Vault RNA 2-1	RNA Gene	16
UBE2C	Ubiquitin Conjugating Enzyme E2 C	Protein Coding	47
RBM14	RNA Binding Motif Protein 14	Protein Coding	36
TCF19	Transcription Factor 19	Protein Coding	37
BRMS1	BRMS1 Transcriptional Repressor And Anoikis Regula	Protein Coding	39
PSMD13	Proteasome 26S Subunit, Non-ATPase 13	Protein Coding	41
ACOT8	Acyl-CoA Thioesterase 8	Protein Coding	40
TMEM252	Transmembrane Protein 252	Protein Coding	31
RIOX1	Ribosomal Oxygenase 1	Protein Coding	24
SPATA8	Spermatogenesis Associated 8	RNA Gene	33
S100A13	S100 Calcium Binding Protein A13	Protein Coding	39
ZNF202	Zinc Finger Protein 202	Protein Coding	39
CLIC1	Chloride Intracellular Channel 1	Protein Coding	43
MRM3	Mitochondrial RRNA Methyltransferase 3	Protein Coding	30
SND1	Staphylococcal Nuclease And Tudor Domain Containin	Protein Coding	43
TSHZ2	Teashirt Zinc Finger Homeobox 2	Protein Coding	40
PNRC2	Proline Rich Nuclear Receptor Coactivator 2	Protein Coding	36
KHSRP	KH-Type Splicing Regulatory Protein	Protein Coding	41
COX8C	Cytochrome C Oxidase Subunit 8C	Protein Coding	32
ACTR3	Actin Related Protein 3	Protein Coding	43
CAMK2D	Calcium/Calmodulin Dependent Protein Kinase II Delta	Protein Coding	50
DUSP3	Dual Specificity Phosphatase 3	Protein Coding	47
RPP25L	Ribonuclease P/MRP Subunit P25 Like	Protein Coding	34
LSM4	LSM4 Homolog, U6 Small Nuclear RNA And MRNA	Protein Coding	41
KCNK5	Potassium Two Pore Domain Channel Subfamily K Me	Protein Coding	39
GPSM3	G Protein Signaling Modulator 3	Protein Coding	34
UNC45A	Unc-45 Myosin Chaperone A	Protein Coding	36
ASIC2	Acid Sensing Ion Channel Subunit 2	Protein Coding	40
AADAT	Aminoadipate Aminotransferase	Protein Coding	41
GALNT2	Polypeptide N-Acetylgalactosaminyltransferase 2	Protein Coding	44
GIT1	GIT ArfGAP 1	Protein Coding	45
DNAJB8	DnaJ Heat Shock Protein Family (Hsp40) Member B8	Protein Coding	33
SNX33	Sorting Nexin 33	Protein Coding	34
KIAA1841	KIAA1841	Protein Coding	35
PPP2R5E	Protein Phosphatase 2 Regulatory Subunit B'Epsilon	Protein Coding	40

CA5A	Carbonic Anhydrase 5A	Protein Coding	44
PPP1R14A	Protein Phosphatase 1 Regulatory Inhibitor Subunit 14A	Protein Coding	42
ZC2HC1C	Zinc Finger C2HC-Type Containing 1C	Protein Coding	32
FAM107A	Family With Sequence Similarity 107 Member A	Protein Coding	37
HMG20A	High Mobility Group 20A	Protein Coding	38
PDE12	Phosphodiesterase 12	Protein Coding	36
LTK	Leukocyte Receptor Tyrosine Kinase	Protein Coding	43
ATP1B4	ATPase Na+/K+ Transporting Family Member Beta 4	Protein Coding	34
CXCL14	C-X-C Motif Chemokine Ligand 14	Protein Coding	40
IFI30	IFI30 Lysosomal Thiol Reductase	Protein Coding	40
SHC3	SHC Adaptor Protein 3	Protein Coding	42
AKAP8L	A-Kinase Anchoring Protein 8 Like	Protein Coding	36
HNRNPD	Heterogeneous Nuclear Ribonucleoprotein D	Protein Coding	44
TMX3	Thioredoxin Related Transmembrane Protein 3	Protein Coding	39
RPL10L	Ribosomal Protein L10 Like	Protein Coding	35
SOCS5	Suppressor Of Cytokine Signaling 5	Protein Coding	43
PTGES3	Prostaglandin E Synthase 3	Protein Coding	46
IGKV1-5	Immunoglobulin Kappa Variable 1-5	Protein Coding	20
SF3B2	Splicing Factor 3b Subunit 2	Protein Coding	39
MRPL27	Mitochondrial Ribosomal Protein L27	Protein Coding	35
NMUR1	Neuromedin U Receptor 1	Protein Coding	42
SIGIRR	Single Ig And TIR Domain Containing	Protein Coding	41
CDC42EP3	CDC42 Effector Protein 3	Protein Coding	40
BASP1	Brain Abundant Membrane Attached Signal Protein 1	Protein Coding	37
PLPP3	Phospholipid Phosphatase 3	Protein Coding	35
PTP4A1	Protein Tyrosine Phosphatase 4A1	Protein Coding	42
CDK14	Cyclin Dependent Kinase 14	Protein Coding	41
SLC27A1	Solute Carrier Family 27 Member 1	Protein Coding	43
HIVEP3	HIVEP Zinc Finger 3	Protein Coding	37
TGFB1I1	Transforming Growth Factor Beta 1 Induced Transcript	Protein Coding	42
NCALD	Neurocalcin Delta	Protein Coding	43
CEBPZ	CCAAT Enhancer Binding Protein Zeta	Protein Coding	37
SMARCD	SWI/SNF Related, Matrix Associated, Actin Dependent	Protein Coding	41
LATS1	Large Tumor Suppressor Kinase 1	Protein Coding	45
CKMT1B	Creatine Kinase, Mitochondrial 1B	Protein Coding	39
COX7C	Cytochrome C Oxidase Subunit 7C	Protein Coding	37
CCT8	Chaperonin Containing TCP1 Subunit 8	Protein Coding	40
INTS3	Integrator Complex Subunit 3	Protein Coding	39
MEIG1	Meiosis/Spermiogenesis Associated 1	Protein Coding	31
DNAJC9	DnaJ Heat Shock Protein Family (Hsp40) Member C9	Protein Coding	36
CBFA2T2	CBFA2/RUNX1 Partner Transcriptional Co-Repressor	Protein Coding	39
STARD7	StAR Related Lipid Transfer Domain Containing 7	Protein Coding	37
ARID3A	AT-Rich Interaction Domain 3A	Protein Coding	39
RPA2	Replication Protein A2	Protein Coding	46
FIGN	Fidgetin, Microtubule Severing Factor	Protein Coding	35
ACTR2	Actin Related Protein 2	Protein Coding	44
STK40	Serine/Threonine Kinase 40	Protein Coding	39

BMX	BMX Non-Receptor Tyrosine Kinase	Protein Coding	46
UBXN7	UBX Domain Protein 7	Protein Coding	34
TANK	TRAF Family Member Associated NFKB Activator	Protein Coding	43
SUSD3	Sushi Domain Containing 3	Protein Coding	35
RPL23AP2	Ribosomal Protein L23a Pseudogene 28	Pseudogene	7
MRAP2	Melanocortin 2 Receptor Accessory Protein 2	Protein Coding	35
RABGGTI	Rab Geranylgeranyltransferase Subunit Beta	Protein Coding	40
NNAT	Neuronatin	Protein Coding	33
DHX40	DEAH-Box Helicase 40	Protein Coding	37
ACSBG1	Acyl-CoA Synthetase Bubblegum Family Member 1	Protein Coding	41
PIAS2	Protein Inhibitor Of Activated STAT 2	Protein Coding	44
NPL	N-Acetylneuraminate Pyruvate Lyase	Protein Coding	40
PELP1	Proline, Glutamate And Leucine Rich Protein 1	Protein Coding	40
ABCF3	ATP Binding Cassette Subfamily F Member 3	Protein Coding	37
RIOX2	Ribosomal Oxygenase 2	Protein Coding	33
HMG20B	High Mobility Group 20B	Protein Coding	36
SUCLG2	Succinate-CoA Ligase GDP-Forming Subunit Beta	Protein Coding	41
ATF5	Activating Transcription Factor 5	Protein Coding	37
HYI	Hydroxypyruvate Isomerase (Putative)	Protein Coding	35
ATP2C2	ATPase Secretory Pathway Ca2+ Transporting 2	Protein Coding	40
IGBP1	Immunoglobulin Binding Protein 1	Protein Coding	43
OAS2	2'-5'-Oligoadenylate Synthetase 2	Protein Coding	40
RBM23	RNA Binding Motif Protein 23	Protein Coding	36
KIAA1586	KIAA1586	Protein Coding	34
SRP68	Signal Recognition Particle 68	Protein Coding	36
MIR133A2	MicroRNA 133a-2	RNA Gene	20
VAMP3	Vesicle Associated Membrane Protein 3	Protein Coding	42
EFS	Embryonal Fyn-Associated Substrate	Protein Coding	36
STX4	Syntaxin 4	Protein Coding	43
CAPNS2	Calpain Small Subunit 2	Protein Coding	37
RAB3D	RAB3D, Member RAS Oncogene Family	Protein Coding	40
WDR3	WD Repeat Domain 3	Protein Coding	38
WDR18	WD Repeat Domain 18	Protein Coding	35
TUBGCP3	Tubulin Gamma Complex Associated Protein 3	Protein Coding	39
PPP1R12A	Protein Phosphatase 1 Regulatory Subunit 12A	Protein Coding	42
RAB32	RAB32, Member RAS Oncogene Family	Protein Coding	39
FAM166B	Family With Sequence Similarity 166 Member B	Protein Coding	31
TMEM97	Transmembrane Protein 97	Protein Coding	36
ADAM15	ADAM Metallopeptidase Domain 15	Protein Coding	41
CYP20A1	Cytochrome P450 Family 20 Subfamily A Member 1	Protein Coding	39
FAAP24	FA Core Complex Associated Protein 24	Protein Coding	31
NRN1	Neuritin 1	Protein Coding	40
TEX29	Testis Expressed 29	Protein Coding	32
LRRC8E	Leucine Rich Repeat Containing 8 VRAC Subunit E	Protein Coding	36
PDCD7	Programmed Cell Death 7	Protein Coding	33
MOB3C	MOB Kinase Activator 3C	Protein Coding	33
ULK2	Unc-51 Like Autophagy Activating Kinase 2	Protein Coding	44

GADD45C	Growth Arrest And DNA Damage Inducible Gamma	Protein Coding	42
PILRA	Paired Immunoglobin Like Type 2 Receptor Alpha	Protein Coding	36
TRAF5	TNF Receptor Associated Factor 5	Protein Coding	44
ACAA1	Acetyl-CoA Acyltransferase 1	Protein Coding	44
MPRIP	Myosin Phosphatase Rho Interacting Protein	Protein Coding	37
MAB21L1	Mab-21 Like 1	Protein Coding	39
CDK18	Cyclin Dependent Kinase 18	Protein Coding	40
BAIAP2L1	BAR/IMD Domain Containing Adaptor Protein 2 Like	Protein Coding	39
OR1L6	Olfactory Receptor Family 1 Subfamily L Member 6	Protein Coding	31
POLDIP2	DNA Polymerase Delta Interacting Protein 2	Protein Coding	36
VPS37C	VPS37C Subunit Of ESCRT-I	Protein Coding	37
NCEH1	Neutral Cholesterol Ester Hydrolase 1	Protein Coding	40
NAALADI	N-Acetylated Alpha-Linked Acidic Dipeptidase Like 2	Protein Coding	36
MIR103A2	MicroRNA 103a-2	RNA Gene	20
TAOK1	TAO Kinase 1	Protein Coding	44
SSR2	Signal Sequence Receptor Subunit 2	Protein Coding	41
TAGLN2	Transgelin 2	Protein Coding	41
PAGR1	PAXIP1 Associated Glutamate Rich Protein 1	Protein Coding	35
NOL6	Nucleolar Protein 6	Protein Coding	36
SLC35B3	Solute Carrier Family 35 Member B3	Protein Coding	36
ZFAND5	Zinc Finger AN1-Type Containing 5	Protein Coding	40
NSFL1C	NSFL1 Cofactor	Protein Coding	40
TET3	Tet Methylcytosine Dioxygenase 3	Protein Coding	40
PCDH18	Protocadherin 18	Protein Coding	37
CPNE3	Copine 3	Protein Coding	38
ANP32E	Acidic Nuclear Phosphoprotein 32 Family Member E	Protein Coding	35
COX7A1	Cytochrome C Oxidase Subunit 7A1	Protein Coding	38
SLC1A5	Solute Carrier Family 1 Member 5	Protein Coding	44
LARP4	La Ribonucleoprotein 4	Protein Coding	35
MCM10	Minichromosome Maintenance 10 Replication Initiation	Protein Coding	39
GMDS	GDP-Mannose 4,6-Dehydratase	Protein Coding	45
FMNL1	Formin Like 1	Protein Coding	38
PYM1	PYM Homolog 1, Exon Junction Complex Associated F	Protein Coding	29
MYDGF	Myeloid Derived Growth Factor	Protein Coding	35
GSPT2	G1 To S Phase Transition 2	Protein Coding	39
SDF4	Stromal Cell Derived Factor 4	Protein Coding	38
POLDIP3	DNA Polymerase Delta Interacting Protein 3	Protein Coding	38
DLGAP1	DLG Associated Protein 1	Protein Coding	44
GTDC1	Glycosyltransferase Like Domain Containing 1	Protein Coding	37
MTURN	Maturin, Neural Progenitor Differentiation Regulator H	Protein Coding	31
MTERF4	Mitochondrial Transcription Termination Factor 4	Protein Coding	32
LPAR3	Lysophosphatidic Acid Receptor 3	Protein Coding	45
RPL9P21	Ribosomal Protein L9 Pseudogene 21	Pseudogene	8
RPL22	Ribosomal Protein L22	Protein Coding	44
BAG4	BAG Cochaperone 4	Protein Coding	40
ANXA4	Annexin A4	Protein Coding	45
EPS8L2	EPS8 Like 2	Protein Coding	40

ABCF1	ATP Binding Cassette Subfamily F Member 1	Protein Coding	40
ZBTB17	Zinc Finger And BTB Domain Containing 17	Protein Coding	42
H2BC3	H2B Clustered Histone 3	Protein Coding	29
GGTA1P	Glycoprotein Alpha-Galactosyltransferase 1, Pseudogen	Pseudogene	21
CNTROB	Centrobin, Centriole Duplication And Spindle Assembly	Protein Coding	37
PFAS	Phosphoribosylformylglycinamidine Synthase	Protein Coding	42
WDR24	WD Repeat Domain 24	Protein Coding	33
HIRIP3	HIRA Interacting Protein 3	Protein Coding	36
STK25	Serine/Threonine Kinase 25	Protein Coding	41
SCYL3	SCY1 Like Pseudokinase 3	Protein Coding	39
BNIP1	BCL2 Interacting Protein 1	Protein Coding	37
CCT2	Chaperonin Containing TCP1 Subunit 2	Protein Coding	42
SLC48A1	Solute Carrier Family 48 Member 1	Protein Coding	36
DMRT2	Doublesex And Mab-3 Related Transcription Factor 2	Protein Coding	36
SMDT1	Single-Pass Membrane Protein With Aspartate Rich Ta	Protein Coding	34
ARPC2	Actin Related Protein 2/3 Complex Subunit 2	Protein Coding	44
NOLC1	Nucleolar And Coiled-Body Phosphoprotein 1	Protein Coding	39
ERMP1	Endoplasmic Reticulum Metallopeptidase 1	Protein Coding	36
ZKSCAN8	Zinc Finger With KRAB And SCAN Domains 8	Protein Coding	34
FAM214B	Family With Sequence Similarity 214 Member B	Protein Coding	36
MYL9	Myosin Light Chain 9	Protein Coding	44
GMPS	Guanine Monophosphate Synthase	Protein Coding	45
E2F6	E2F Transcription Factor 6	Protein Coding	41
PHACTR4	Phosphatase And Actin Regulator 4	Protein Coding	33
EIF2D	Eukaryotic Translation Initiation Factor 2D	Protein Coding	35
PROB1	Proline Rich Basic Protein 1	Protein Coding	27
MRPS17	Mitochondrial Ribosomal Protein S17	Protein Coding	39
UBE2Z	Ubiquitin Conjugating Enzyme E2 Z	Protein Coding	42
AOAH	Acyloxyacyl Hydrolase	Protein Coding	39
NKAPD1	NKAP Domain Containing 1	Protein Coding	24
EIF6	Eukaryotic Translation Initiation Factor 6	Protein Coding	41
CYB561	Cytochrome B561	Protein Coding	42
CACNB1	Calcium Voltage-Gated Channel Auxiliary Subunit Beta	Protein Coding	43
RGS22	Regulator Of G Protein Signaling 22	Protein Coding	36
TFAP2D	Transcription Factor AP-2 Delta	Protein Coding	36
RCN2	Reticulocalbin 2	Protein Coding	40
CST2	Cystatin SA	Protein Coding	37
ZNF212	Zinc Finger Protein 212	Protein Coding	39
H2AZ2	H2A.Z Variant Histone 2	Protein Coding	28
TPCN2	Two Pore Segment Channel 2	Protein Coding	39
TYRL	Tyrosinase Like (Pseudogene)	Pseudogene	11
TAF11	TATA-Box Binding Protein Associated Factor 11	Protein Coding	40
AP1B1	Adaptor Related Protein Complex 1 Subunit Beta 1	Protein Coding	43
PPP1R21	Protein Phosphatase 1 Regulatory Subunit 21	Protein Coding	33
DPCD	Deleted In Primary Ciliary Dyskinesia Homolog (Mouse	Protein Coding	35
RWDD4	RWD Domain Containing 4	Protein Coding	35
SLC25A31	Solute Carrier Family 25 Member 31	Protein Coding	40

MRPL58	Mitochondrial Ribosomal Protein L58	Protein Coding	32
OIP5-AS1	OIP5 Antisense RNA 1	RNA Gene	18
XCR1	X-C Motif Chemokine Receptor 1	Protein Coding	39
LINC0140	Long Intergenic Non-Protein Coding RNA 1405	RNA Gene	15
SELENOV	Selenoprotein W	Protein Coding	31
MIR129-2	MicroRNA 129-2	RNA Gene	19
CCNL1	Cyclin L1	Protein Coding	40
IK	IK Cytokine	Protein Coding	39
RAB39A	RAB39A, Member RAS Oncogene Family	Protein Coding	33
RPL34P11	Ribosomal Protein L34 Pseudogene 11	Pseudogene	7
PCBD2	Pterin-4 Alpha-Carbinolamine Dehydratase 2	Protein Coding	38
SEC22B	SEC22 Homolog B, Vesicle Trafficking Protein (Gene/I	Protein Coding	36
CCDC186	Coiled-Coil Domain Containing 186	Protein Coding	31
ZMAT5	Zinc Finger Matrin-Type 5	Protein Coding	32
C22orf31	Chromosome 22 Open Reading Frame 31	Protein Coding	29
NACA	Nascent Polypeptide Associated Complex Subunit Alph	Protein Coding	39
FAM207A	Family With Sequence Similarity 207 Member A	Protein Coding	31
HLA-DRB	Major Histocompatibility Complex, Class II, DR Beta 6	Pseudogene	17
PRSS23	Serine Protease 23	Protein Coding	39
DIRC1	Disrupted In Renal Carcinoma 1	RNA Gene	27
RASSF8	Ras Association Domain Family Member 8	Protein Coding	37
HSD17B1	Hydroxysteroid 17-Beta Dehydrogenase 12	Protein Coding	41
FBF1	Fas Binding Factor 1	Protein Coding	34
ARL6IP5	ADP Ribosylation Factor Like GTPase 6 Interacting Pr	Protein Coding	38
H1-10	H1.10 Linker Histone	Protein Coding	28
KIFC1	Kinesin Family Member C1	Protein Coding	42
ATG10	Autophagy Related 10	Protein Coding	40
CLK3	CDC Like Kinase 3	Protein Coding	43
RPS21	Ribosomal Protein S21	Protein Coding	40
CA6	Carbonic Anhydrase 6	Protein Coding	43
RERGL	RERG Like	Protein Coding	32
PDS5B	PDS5 Cohesin Associated Factor B	Protein Coding	36
KAZN	Kazrin, Periplakin Interacting Protein	Protein Coding	34
ECI2	Enoyl-CoA Delta Isomerase 2	Protein Coding	40
SPDYA	Speedy/RINGO Cell Cycle Regulator Family Member A	Protein Coding	36
BCAR3	BCAR3 Adaptor Protein, NSP Family Member	Protein Coding	41
KIF4A	Kinesin Family Member 4A	Protein Coding	41
IFNA5	Interferon Alpha 5	Protein Coding	38
AQP9	Aquaporin 9	Protein Coding	44
TMEM139	Transmembrane Protein 139	Protein Coding	33
RAB21	RAB21, Member RAS Oncogene Family	Protein Coding	38
FAAP100	FA Core Complex Associated Protein 100	Protein Coding	31
LCP2	Lymphocyte Cytosolic Protein 2	Protein Coding	43
BLOC1S2	Biogenesis Of Lysosomal Organelles Complex 1 Subun	Protein Coding	36
PLIN3	Perilipin 3	Protein Coding	43
C15orf48	Chromosome 15 Open Reading Frame 48	Protein Coding	34
TRPT1	TRNA Phosphotransferase 1	Protein Coding	35

CDC14C	Cell Division Cycle 14C	Protein Coding	21
UBA2	Ubiquitin Like Modifier Activating Enzyme 2	Protein Coding	45
BMP2K	BMP2 Inducible Kinase	Protein Coding	40
KYAT1	Kynurenine Aminotransferase 1	Protein Coding	35
PTGER1	Prostaglandin E Receptor 1	Protein Coding	41
DRG2	Developmentally Regulated GTP Binding Protein 2	Protein Coding	39
MRPL36	Mitochondrial Ribosomal Protein L36	Protein Coding	36
CSRNP3	Cysteine And Serine Rich Nuclear Protein 3	Protein Coding	33
LY6G5B	Lymphocyte Antigen 6 Family Member G5B	Protein Coding	31
NECAB1	N-Terminal EF-Hand Calcium Binding Protein 1	Protein Coding	33
DHX58	DExH-Box Helicase 58	Protein Coding	41
ACAD11	Acyl-CoA Dehydrogenase Family Member 11	Protein Coding	36
H2BS1	H2B.S Histone 1	Protein Coding	21
MIER1	MIER1 Transcriptional Regulator	Protein Coding	35
PKNOX1	PBX/Knotted 1 Homeobox 1	Protein Coding	41
PDLIM2	PDZ And LIM Domain 2	Protein Coding	39
TBCA	Tubulin Folding Cofactor A	Protein Coding	38
RAB5C	RAB5C, Member RAS Oncogene Family	Protein Coding	42
NUAK1	NUAK Family Kinase 1	Protein Coding	44
NAAA	N-Acylethanolamine Acid Amidase	Protein Coding	41
ACOT2	Acyl-CoA Thioesterase 2	Protein Coding	40
NME3	NME/NM23 Nucleoside Diphosphate Kinase 3	Protein Coding	44
NUCB2	Nucleobindin 2	Protein Coding	40
TBCC	Tubulin Folding Cofactor C	Protein Coding	37
MAP1A	Microtubule Associated Protein 1A	Protein Coding	41
MRPL12	Mitochondrial Ribosomal Protein L12	Protein Coding	40
ITIH1	Inter-Alpha-Trypsin Inhibitor Heavy Chain 1	Protein Coding	40
OLFML2A	Olfactomedin Like 2A	Protein Coding	36
PAQR6	Progestin And AdipoQ Receptor Family Member 6	Protein Coding	33
IMPA2	Inositol Monophosphatase 2	Protein Coding	46
SERF1A	Small EDRK-Rich Factor 1A	Protein Coding	32
MIR124-2	MicroRNA 124-2	RNA Gene	19
LEPROT	Leptin Receptor Overlapping Transcript	Protein Coding	35
GAL3ST1	Galactose-3-O-Sulfotransferase 1	Protein Coding	37
RNF185	Ring Finger Protein 185	Protein Coding	37
RHOT2	Ras Homolog Family Member T2	Protein Coding	40
SRPK3	SRSF Protein Kinase 3	Protein Coding	40
C1orf115	Chromosome 1 Open Reading Frame 115	Protein Coding	28
ING3	Inhibitor Of Growth Family Member 3	Protein Coding	40
P2RX6	Purinergic Receptor P2X 6	Protein Coding	41
RPH3A	Rabphilin 3A	Protein Coding	41
RSRP1	Arginine And Serine Rich Protein 1	Protein Coding	28
ATP5IF1	ATP Synthase Inhibitory Factor Subunit 1	Protein Coding	31
NKD2	NKD Inhibitor Of WNT Signaling Pathway 2	Protein Coding	35
WDR75	WD Repeat Domain 75	Protein Coding	35
COX6B2	Cytochrome C Oxidase Subunit 6B2	Protein Coding	34
ABI3	ABI Family Member 3	Protein Coding	39

PRRC2C	Proline Rich Coiled-Coil 2C	Protein Coding	34
TPRG1	Tumor Protein P63 Regulated 1	Protein Coding	35
CPLX3	Complexin 3	Protein Coding	37
UTP25	UTP25 Small Subunit Processor Component	Protein Coding	26
MACROD	Mono-ADP Ribosylhydrolase 1	Protein Coding	36
RPS3P7	Ribosomal Protein S3 Pseudogene 7	Pseudogene	9
TIPRL	TOR Signaling Pathway Regulator	Protein Coding	35
CA5B	Carbonic Anhydrase 5B	Protein Coding	42
KANSL2	KAT8 Regulatory NSL Complex Subunit 2	Protein Coding	35
SEC13	SEC13 Homolog, Nuclear Pore And COPII Coat Comp	Protein Coding	43
ZNF783	Zinc Finger Family Member 783	Protein Coding	29
POLE3	DNA Polymerase Epsilon 3, Accessory Subunit	Protein Coding	43
MSRB2	Methionine Sulfoxide Reductase B2	Protein Coding	42
KIF2C	Kinesin Family Member 2C	Protein Coding	44
IFNA8	Interferon Alpha 8	Protein Coding	35
SRPK2	SRSF Protein Kinase 2	Protein Coding	46
EIF4A1P6	Eukaryotic Translation Initiation Factor 4A1 Pseudoger	Pseudogene	8
PRKAR2A	Protein Kinase CAMP-Dependent Type II Regulatory S	Protein Coding	48
RANBP9	RAN Binding Protein 9	Protein Coding	41
RPS2P34	Ribosomal Protein S2 Pseudogene 34	Pseudogene	6
MLLT11	MLLT11 Transcription Factor 7 Cofactor	Protein Coding	36
IGKV2D-2	Immunoglobulin Kappa Variable 2D-29	Protein Coding	14
ARL6IP6	ADP Ribosylation Factor Like GTPase 6 Interacting Pr	Protein Coding	36
OR7E28P	Olfactory Receptor Family 7 Subfamily E Member 28 F	Pseudogene	10
JADE2	Jade Family PHD Finger 2	Protein Coding	33
PSD	Pleckstrin And Sec7 Domain Containing	Protein Coding	41
E2F8	E2F Transcription Factor 8	Protein Coding	36
MCRIP2	MAPK Regulated Corepressor Interacting Protein 2	Protein Coding	25
DIAPH2	Diaphanous Related Formin 2	Protein Coding	44
EIF2S2	Eukaryotic Translation Initiation Factor 2 Subunit Beta	Protein Coding	42
ARHGEF1	Rho Guanine Nucleotide Exchange Factor 19	Protein Coding	39
QPRT	Quinolinate Phosphoribosyltransferase	Protein Coding	44
SENP1	SUMO Specific Peptidase 1	Protein Coding	44
MSTO2P	Misato Family Member 2, Pseudogene	Pseudogene	14
ERP29	Endoplasmic Reticulum Protein 29	Protein Coding	36
RPS3AP46	RPS3A Pseudogene 46	Pseudogene	9
SIK2	Salt Inducible Kinase 2	Protein Coding	45
CALU	Calumenin	Protein Coding	41
EPB41L2	Erythrocyte Membrane Protein Band 4.1 Like 2	Protein Coding	40
ZRSR2	Zinc Finger CCCH-Type, RNA Binding Motif And Seri	Protein Coding	36
BANF1P1	BANF1 Pseudogene 1	Pseudogene	9
KCTD14	Potassium Channel Tetramerization Domain Containing	Protein Coding	32
GLIPR2	GLI Pathogenesis Related 2	Protein Coding	38
SYPL2	Synaptophysin Like 2	Protein Coding	36
CEP112	Centrosomal Protein 112	Protein Coding	32
PTPRE	Protein Tyrosine Phosphatase Receptor Type E	Protein Coding	43
SPATA4	Spermatogenesis Associated 4	Protein Coding	35

IP6K2	Inositol Hexakisphosphate Kinase 2	Protein Coding	41
PYGO2	Pygopus Family PHD Finger 2	Protein Coding	39
TRDMT1	TRNA Aspartic Acid Methyltransferase 1	Protein Coding	41
LOC10028	Uncharacterized LOC100287944	RNA Gene	14
ILKAP	ILK Associated Serine/Threonine Phosphatase	Protein Coding	40
LRRC47	Leucine Rich Repeat Containing 47	Protein Coding	31
ACTBL2	Actin Beta Like 2	Protein Coding	34
MAP3K10	Mitogen-Activated Protein Kinase Kinase Kinase 10	Protein Coding	44
APOBEC4	Apolipoprotein B MRNA Editing Enzyme Catalytic Pol	Protein Coding	35
C2orf88	Chromosome 2 Open Reading Frame 88	Protein Coding	32
MED30	Mediator Complex Subunit 30	Protein Coding	37
STARD4	StAR Related Lipid Transfer Domain Containing 4	Protein Coding	36
CERK	Ceramide Kinase	Protein Coding	43
HPF1	Histone PARylation Factor 1	Protein Coding	25
ANKRD36	Ankyrin Repeat Domain 36B Pseudogene 1	Pseudogene	18
DYNLRB2	Dynein Light Chain Roadblock-Type 2	Protein Coding	40
DYNC1I1	Dynein Cytoplasmic 1 Intermediate Chain 1	Protein Coding	41
SEPTIN3	Septin 3	Protein Coding	31
RAB2A	RAB2A, Member RAS Oncogene Family	Protein Coding	43
SPNS1	Sphingolipid Transporter 1 (Putative)	Protein Coding	36
GLRA2	Glycine Receptor Alpha 2	Protein Coding	45
ZRANB2	Zinc Finger RANBP2-Type Containing 2	Protein Coding	36
PPIL2	Peptidylprolyl Isomerase Like 2	Protein Coding	42
SMYD3	SET And MYND Domain Containing 3	Protein Coding	43
SDCBP	Syndecan Binding Protein	Protein Coding	43
TRAK2	Trafficking Kinesin Protein 2	Protein Coding	40
FMNL2	Formin Like 2	Protein Coding	39
GBF1	Golgi Brefeldin A Resistant Guanine Nucleotide Exchar	Protein Coding	44
AK4	Adenylate Kinase 4	Protein Coding	41
SLC44A1	Solute Carrier Family 44 Member 1	Protein Coding	44
SORBS3	Sorbin And SH3 Domain Containing 3	Protein Coding	41
LIMA1	LIM Domain And Actin Binding 1	Protein Coding	40
ITGB1BP1	Integrin Subunit Beta 1 Binding Protein 1	Protein Coding	39
PLCB2	Phospholipase C Beta 2	Protein Coding	46
EFNA4	Ephrin A4	Protein Coding	43
PLRG1	Pleiotropic Regulator 1	Protein Coding	39
UPK3B	Uroplakin 3B	Protein Coding	28
GOLGA4	Golgin A4	Protein Coding	37
RRBP1	Ribosome Binding Protein 1	Protein Coding	40
EXOC3-A	EXOC3 Antisense RNA 1	RNA Gene	25
CFDP1	Craniofacial Development Protein 1	Protein Coding	37
CHRM4	Cholinergic Receptor Muscarinic 4	Protein Coding	46
NTM	Neurotrimin	Protein Coding	41
HSPB7	Heat Shock Protein Family B (Small) Member 7	Protein Coding	40
ZNF282	Zinc Finger Protein 282	Protein Coding	35
NUDC	Nuclear Distribution C, Dynein Complex Regulator	Protein Coding	43
TUBA3E	Tubulin Alpha 3e	Protein Coding	39

CASC16	Cancer Susceptibility 16	RNA Gene	15
GBP2	Guanylate Binding Protein 2	Protein Coding	37
ME2	Malic Enzyme 2	Protein Coding	45
P4HA1	Prolyl 4-Hydroxylase Subunit Alpha 1	Protein Coding	41
MARCHF:	Membrane Associated Ring-CH-Type Finger 5	Protein Coding	31
ODAM	Odontogenic, Ameloblast Associated	Protein Coding	36
SPAG6	Sperm Associated Antigen 6	Protein Coding	36
ANKRD13	Ankyrin Repeat Domain 13D	Protein Coding	36
C3orf67	Chromosome 3 Open Reading Frame 67	Protein Coding	32
CD2BP2	CD2 Cytoplasmic Tail Binding Protein 2	Protein Coding	38
BIN3	Bridging Integrator 3	Protein Coding	37
PGAP4	Post-GPI Attachment To Proteins GalNAc Transferase	Protein Coding	25
HMGN1	High Mobility Group Nucleosome Binding Domain 1	Protein Coding	42
COX7B2	Cytochrome C Oxidase Subunit 7B2	Protein Coding	32
ZC3HAV1	Zinc Finger CCCH-Type Containing, Antiviral 1	Protein Coding	37
ULK3	Unc-51 Like Kinase 3	Protein Coding	41
HINT2	Histidine Triad Nucleotide Binding Protein 2	Protein Coding	39
RPAP1	RNA Polymerase II Associated Protein 1	Protein Coding	35
KCNH7	Potassium Voltage-Gated Channel Subfamily H Membe	Protein Coding	42
SEC31A	SEC31 Homolog A, COPII Coat Complex Component	Protein Coding	43
UCHL5	Ubiquitin C-Terminal Hydrolase L5	Protein Coding	43
SYNJ2BP	Synaptojanin 2 Binding Protein	Protein Coding	39
EEF1B2	Eukaryotic Translation Elongation Factor 1 Beta 2	Protein Coding	44
SUB1	SUB1 Regulator Of Transcription	Protein Coding	39
LSM7	LSM7 Homolog, U6 Small Nuclear RNA And MRNA I	Protein Coding	37
POMZP3	POM121 And ZP3 Fusion	Protein Coding	33
C19orf33	Chromosome 19 Open Reading Frame 33	Protein Coding	31
MOB3A	MOB Kinase Activator 3A	Protein Coding	32
FOXK1	Forkhead Box K1	Protein Coding	36
CYCSP42	CYCS Pseudogene 42	Pseudogene	7
UBAC1	UBA Domain Containing 1	Protein Coding	37
TMCO6	Transmembrane And Coiled-Coil Domains 6	Protein Coding	33
EXOSC1	Exosome Component 1	Protein Coding	38
ERICH2	Glutamate Rich 2	Protein Coding	27
RAB12	RAB12, Member RAS Oncogene Family	Protein Coding	34
TNIP2	TNFAIP3 Interacting Protein 2	Protein Coding	39
BAALC-A	BAALC Antisense RNA 1	RNA Gene	14
CAB39L	Calcium Binding Protein 39 Like	Protein Coding	36
MRPS36P:	Mitochondrial Ribosomal Protein S36 Pseudogene 2	Pseudogene	8
CD300LF	CD300 Molecule Like Family Member F	Protein Coding	41
IFNA21	Interferon Alpha 21	Protein Coding	36
PRPF38A	Pre-MRNA Processing Factor 38A	Protein Coding	36
TRAPPC3	Trafficking Protein Particle Complex 3	Protein Coding	41
GTF2E1	General Transcription Factor IIE Subunit 1	Protein Coding	40
DPY19L2I	DPY19L2 Pseudogene 1	Pseudogene	18
CASZ1	Castor Zinc Finger 1	Protein Coding	37
ACYP2	Acylphosphatase 2	Protein Coding	41

SFR1	SWI5 Dependent Homologous Recombination Repair P	Protein Coding	32
C1orf127	Chromosome 1 Open Reading Frame 127	Protein Coding	35
DAD1	Defender Against Cell Death 1	Protein Coding	42
OLFM3	Olfactomedin 3	Protein Coding	37
MTHFD2	Methylenetetrahydrofolate Dehydrogenase (NADP+ De	Protein Coding	44
UBAP2L	Ubiquitin Associated Protein 2 Like	Protein Coding	35
AGAP6	ArfGAP With GTPase Domain, Ankyrin Repeat And Pl	Protein Coding	29
OR9G9	Olfactory Receptor Family 9 Subfamily G Member 9	Protein Coding	17
SNORD50	Small Nucleolar RNA, C/D Box 50B	RNA Gene	12
NPM1P10	Nucleophosmin 1 Pseudogene 10	Pseudogene	7
PXDNL	Peroxidasin Like	Protein Coding	36
RNF32	Ring Finger Protein 32	Protein Coding	36
STX5	Syntaxin 5	Protein Coding	41
DNAJB12	DnaJ Heat Shock Protein Family (Hsp40) Member B12	Protein Coding	37
DBNDD2	Dysbindin Domain Containing 2	Protein Coding	31
SLC7A6O	Solute Carrier Family 7 Member 6 Opposite Strand	Protein Coding	31
PGLYRP2	Peptidoglycan Recognition Protein 2	Protein Coding	37
NDUFC2-]	NDUFC2-KCTD14 Readthrough	Protein Coding	22
SRPK1	SRSF Protein Kinase 1	Protein Coding	47
NMT2	N-Myristoyltransferase 2	Protein Coding	41
CHRFAM'	CHRNA7 (Exons 5-10) And FAM7A (Exons A-E) Fus	Protein Coding	33
LDLRAD4	Low Density Lipoprotein Receptor Class A Domain Co	Protein Coding	34
FYTTD1	Forty-Two-Three Domain Containing 1	Protein Coding	35
C9orf78	Chromosome 9 Open Reading Frame 78	Protein Coding	34
ENSA	Endosulfine Alpha	Protein Coding	42
NPY1R	Neuropeptide Y Receptor Y1	Protein Coding	47
FAM114A	Family With Sequence Similarity 114 Member A2	Protein Coding	35
ACSL3	Acyl-CoA Synthetase Long Chain Family Member 3	Protein Coding	43
KHDC4	KH Domain Containing 4, Pre-MRNA Splicing Factor	Protein Coding	26
GHITM	Growth Hormone Inducible Transmembrane Protein	Protein Coding	37
ZYX	Zyxin	Protein Coding	47
PRPF19	Pre-MRNA Processing Factor 19	Protein Coding	39
MRPS18A	Mitochondrial Ribosomal Protein S18A	Protein Coding	38
SURF2	Surfeit 2	Protein Coding	37
GUSBP3	GUSB Pseudogene 3	Pseudogene	13
TBC1D3P	TBC1 Domain Family Member 3 Pseudogene 2	Pseudogene	13
FNDC3A	Fibronectin Type III Domain Containing 3A	Protein Coding	37
MSL3	MSL Complex Subunit 3	Protein Coding	37
VPS4A	Vacuolar Protein Sorting 4 Homolog A	Protein Coding	42
TRL-AAG	TRNA-Leu (Anticodon AAG) 2-3	RNA Gene	10
EDEM1	ER Degradation Enhancing Alpha-Mannosidase Like Pr	Protein Coding	43
HMGCS1	3-Hydroxy-3-Methylglutaryl-CoA Synthase 1	Protein Coding	44
PLCD4	Phospholipase C Delta 4	Protein Coding	44
VSTM2L	V-Set And Transmembrane Domain Containing 2 Like	Protein Coding	37
UBL4A	Ubiquitin Like 4A	Protein Coding	36
CMAS	Cytidine Monophosphate N-Acetylneuraminic Acid Syn	Protein Coding	39
PLAAT4	Phospholipase A And Acyltransferase 4	Protein Coding	29

SNAPIN	SNAP Associated Protein	Protein Coding	38
IPO5	Importin 5	Protein Coding	39
ARID3B	AT-Rich Interaction Domain 3B	Protein Coding	39
NIPSNAP1	Nipsnap Homolog 1	Protein Coding	40
HLA-DQB	HLA-DQB1 Antisense RNA 1	RNA Gene	12
ZDHHC8F	ZDHHC8 Pseudogene 1	Pseudogene	17
NASPP1	Nuclear Autoantigenic Sperm Protein Pseudogene 1	Pseudogene	8
RPS3P6	Ribosomal Protein S3 Pseudogene 6	Pseudogene	8
SAE1	SUMO1 Activating Enzyme Subunit 1	Protein Coding	47
LIN7A	Lin-7 Homolog A, Crumbs Cell Polarity Complex Com	Protein Coding	40
BBX	BBX High Mobility Group Box Domain Containing	Protein Coding	36
RASA3	RAS P21 Protein Activator 3	Protein Coding	40
APOL2	Apolipoprotein L2	Protein Coding	40
CELF4	CUGBP Elav-Like Family Member 4	Protein Coding	39
GAPDHS	Glyceraldehyde-3-Phosphate Dehydrogenase, Spermato	Protein Coding	43
FLOT2	Flotillin 2	Protein Coding	41
TMEM30A	Transmembrane Protein 30A	Protein Coding	38
TICRR	TOPBP1 Interacting Checkpoint And Replication Regul	Protein Coding	32
SH2D1B	SH2 Domain Containing 1B	Protein Coding	40
KDELR1	KDEL Endoplasmic Reticulum Protein Retention Recep	Protein Coding	41
VPS37B	VPS37B Subunit Of ESCRT-I	Protein Coding	35
KLF12	Kruppel Like Factor 12	Protein Coding	39
ZXDC	ZXD Family Zinc Finger C	Protein Coding	36
AK3	Adenylate Kinase 3	Protein Coding	43
SUN5	Sad1 And UNC84 Domain Containing 5	Protein Coding	37
CYMP	Chymosin, Pseudogene	Pseudogene	13
RALYL	RALY RNA Binding Protein Like	Protein Coding	36
EIF3G	Eukaryotic Translation Initiation Factor 3 Subunit G	Protein Coding	39
CARM1	Coactivator Associated Arginine Methyltransferase 1	Protein Coding	48
TMOD3	Tropomodulin 3	Protein Coding	40
EIF1AX	Eukaryotic Translation Initiation Factor 1A X-Linked	Protein Coding	42
GMCL2	Germ Cell-Less 2, Spermatogenesis Associated	Protein Coding	18
MRPL23	Mitochondrial Ribosomal Protein L23	Protein Coding	37
SGK3	Serum/Glucocorticoid Regulated Kinase Family Membe	Protein Coding	44
MTERF3	Mitochondrial Transcription Termination Factor 3	Protein Coding	34
ZNF620	Zinc Finger Protein 620	Protein Coding	32
SEPHS2	Selenophosphate Synthetase 2	Protein Coding	39
RALBP1	RalA Binding Protein 1	Protein Coding	45
AKAP1	A-Kinase Anchoring Protein 1	Protein Coding	41
CUL5	Cullin 5	Protein Coding	45
RAB9A	RAB9A, Member RAS Oncogene Family	Protein Coding	41
RRP15	Ribosomal RNA Processing 15 Homolog	Protein Coding	35
UGDH	UDP-Glucose 6-Dehydrogenase	Protein Coding	45
GORASP2	Golgi Reassembly Stacking Protein 2	Protein Coding	41
EIF4B	Eukaryotic Translation Initiation Factor 4B	Protein Coding	43
TCF25	Transcription Factor 25	Protein Coding	37
PKN1	Protein Kinase N1	Protein Coding	48

DPY19L2I	DPY19L2 Pseudogene 3	Pseudogene	13
PAICSP3	Phosphoribosylaminoimidazole Carboxylase, Phosphori	Pseudogene	8
CUEDC1	CUE Domain Containing 1	Protein Coding	36
LIMD1	LIM Domains Containing 1	Protein Coding	41
OSCP1	Organic Solute Carrier Partner 1	Protein Coding	35
MYOZ1	Myozenin 1	Protein Coding	37
MIS18BP1	MIS18 Binding Protein 1	Protein Coding	32
SLC32A1	Solute Carrier Family 32 Member 1	Protein Coding	43
CCDC14	Coiled-Coil Domain Containing 14	Protein Coding	36
PTGES2	Prostaglandin E Synthase 2	Protein Coding	44
RAB11FIP	RAB11 Family Interacting Protein 5	Protein Coding	40
PRPSAP2	Phosphoribosyl Pyrophosphate Synthetase Associated P	Protein Coding	35
UBR3	Ubiquitin Protein Ligase E3 Component N-Recognin 3	Protein Coding	37
PLSCR3	Phospholipid Scramblase 3	Protein Coding	39
FAM171A	Family With Sequence Similarity 171 Member A1	Protein Coding	34
NOL11	Nucleolar Protein 11	Protein Coding	34
ZKSCAN1	Zinc Finger With KRAB And SCAN Domains 1	Protein Coding	38
AKAP14	A-Kinase Anchoring Protein 14	Protein Coding	33
OR4X2	Olfactory Receptor Family 4 Subfamily X Member 2 ((	Protein Coding	32
SYT6	Synaptotagmin 6	Protein Coding	37
OGFR	Opioid Growth Factor Receptor	Protein Coding	39
EEF1G	Eukaryotic Translation Elongation Factor 1 Gamma	Protein Coding	42
EIF3F	Eukaryotic Translation Initiation Factor 3 Subunit F	Protein Coding	44
UQCRHL	Ubiquinol-Cytochrome C Reductase Hinge Protein Like	Protein Coding	22
RBIS	Ribosomal Biogenesis Factor	Protein Coding	25
EFHD2	EF-Hand Domain Family Member D2	Protein Coding	37
VSIG4	V-Set And Immunoglobulin Domain Containing 4	Protein Coding	37
RUSC1-AS	RUSC1 Antisense RNA 1	RNA Gene	27
ACSL1	Acyl-CoA Synthetase Long Chain Family Member 1	Protein Coding	46
CHURC1	Churchill Domain Containing 1	Protein Coding	33
MORF4L1	Mortality Factor 4 Like 1	Protein Coding	43
NUCB1	Nucleobindin 1	Protein Coding	40
SIRT7	Sirtuin 7	Protein Coding	43
DCTPP1	DCTP Pyrophosphatase 1	Protein Coding	37
SARM1	Sterile Alpha And TIR Motif Containing 1	Protein Coding	39
OGFOD2	2-Oxoglutarate And Iron Dependent Oxygenase Domair	Protein Coding	34
RNF10	Ring Finger Protein 10	Protein Coding	40
ANXA8	Annexin A8	Protein Coding	36
INO80	INO80 Complex ATPase Subunit	Protein Coding	37
CHCHD3	Coiled-Coil-Helix-Coiled-Coil-Helix Domain Containin	Protein Coding	37
ITPKB	Inositol-Trisphosphate 3-Kinase B	Protein Coding	44
GPR22	G Protein-Coupled Receptor 22	Protein Coding	36
SLAIN1	SLAIN Motif Family Member 1	Protein Coding	35
SS18L2	SS18 Like 2	Protein Coding	33
ABLIM1	Actin Binding LIM Protein 1	Protein Coding	41
LURAP1L	Leucine Rich Adaptor Protein 1 Like	Protein Coding	33
CCDC33	Coiled-Coil Domain Containing 33	Protein Coding	35

SLC35G1	Solute Carrier Family 35 Member G1	Protein Coding	32
WDR46	WD Repeat Domain 46	Protein Coding	37
CDKN2D	Cyclin Dependent Kinase Inhibitor 2D	Protein Coding	42
GSTM2	Glutathione S-Transferase Mu 2	Protein Coding	42
ARRDC4	Arrestin Domain Containing 4	Protein Coding	35
CRACR2E	Calcium Release Activated Channel Regulator 2B	Protein Coding	32
RBM6	RNA Binding Motif Protein 6	Protein Coding	37
CYB5B	Cytochrome B5 Type B	Protein Coding	43
STK26	Serine/Threonine Kinase 26	Protein Coding	40
RNF138	Ring Finger Protein 138	Protein Coding	41
EIF5	Eukaryotic Translation Initiation Factor 5	Protein Coding	43
C11orf16	Chromosome 11 Open Reading Frame 16	Protein Coding	33
BCAN	Brevican	Protein Coding	42
ADPRS	ADP-Ribosylserine Hydrolase	Protein Coding	31
PPIC	Peptidylprolyl Isomerase C	Protein Coding	41
TSSC4	Tumor Suppressing Subtransferable Candidate 4	Protein Coding	35
TERB2	Telomere Repeat Binding Bouquet Formation Protein 2	Protein Coding	23
OTUD7B	OTU Deubiquitinase 7B	Protein Coding	36
PPP2R5C	Protein Phosphatase 2 Regulatory Subunit B'Gamma	Protein Coding	44
ARMC1	Armadillo Repeat Containing 1	Protein Coding	35
EDIL3	EGF Like Repeats And Discoidin Domains 3	Protein Coding	42
ENKUR	Enkurin, TRPC Channel Interacting Protein	Protein Coding	36
ODF2	Outer Dense Fiber Of Sperm Tails 2	Protein Coding	40
RNF115	Ring Finger Protein 115	Protein Coding	36
TOX2	TOX High Mobility Group Box Family Member 2	Protein Coding	38
CACNG3	Calcium Voltage-Gated Channel Auxiliary Subunit Gan	Protein Coding	41
DYRK2	Dual Specificity Tyrosine Phosphorylation Regulated K	Protein Coding	45
H2BC13	H2B Clustered Histone 13	Protein Coding	28
SLC23A2	Solute Carrier Family 23 Member 2	Protein Coding	43
RPL37	Ribosomal Protein L37	Protein Coding	40
PGLYRP4	Peptidoglycan Recognition Protein 4	Protein Coding	37
SSBP3	Single Stranded DNA Binding Protein 3	Protein Coding	39
TTLL7	Tubulin Tyrosine Ligase Like 7	Protein Coding	33
USP30	Ubiquitin Specific Peptidase 30	Protein Coding	41
DCUN1D1	Defective In Cullin Neddylation 1 Domain Containing 1	Protein Coding	39
DNAJC14	DnaJ Heat Shock Protein Family (Hsp40) Member C14	Protein Coding	34
TNMD	Tenomodulin	Protein Coding	34
TUT7	Terminal Uridylyl Transferase 7	Protein Coding	29
TRPM2	Transient Receptor Potential Cation Channel Subfamily	Protein Coding	43
ATAD3B	ATPase Family AAA Domain Containing 3B	Protein Coding	37
FZR1	Fizzy And Cell Division Cycle 20 Related 1	Protein Coding	43
SPTSSB	Serine Palmitoyltransferase Small Subunit B	Protein Coding	33
CERS6	Ceramide Synthase 6	Protein Coding	39
GON4L	Gon-4 Like	Protein Coding	35
RANGAP1	Ran GTPase Activating Protein 1	Protein Coding	43
ASPHD1	Aspartate Beta-Hydroxylase Domain Containing 1	Protein Coding	32
PLSCR1	Phospholipid Scramblase 1	Protein Coding	44

SRXN1	Sulfiredoxin 1	Protein Coding	36
CACUL1	CDK2 Associated Cullin Domain 1	Protein Coding	33
BRWD1	Bromodomain And WD Repeat Domain Containing 1	Protein Coding	39
REEP5	Receptor Accessory Protein 5	Protein Coding	40
RCN1	Reticulocalbin 1	Protein Coding	40
RABL3	RAB, Member Of RAS Oncogene Family Like 3	Protein Coding	33
KLC4	Kinesin Light Chain 4	Protein Coding	38
GNA13	G Protein Subunit Alpha 13	Protein Coding	45
NAP1L1	Nucleosome Assembly Protein 1 Like 1	Protein Coding	40
DCST1-AS	DCST1 Antisense RNA 1	RNA Gene	13
IFNW1	Interferon Omega 1	Protein Coding	39
LINC0138	Long Intergenic Non-Protein Coding RNA 1387	RNA Gene	16
PPP1R14C	Protein Phosphatase 1 Regulatory Inhibitor Subunit 14C	Protein Coding	39
DDX47	DEAD-Box Helicase 47	Protein Coding	38
GSTA3	Glutathione S-Transferase Alpha 3	Protein Coding	45
TIPIN	TIMELESS Interacting Protein	Protein Coding	39
PDZRN4	PDZ Domain Containing Ring Finger 4	Protein Coding	36
ST3GAL6	ST3 Beta-Galactoside Alpha-2,3-Sialyltransferase 6	Protein Coding	42
SCAMP3	Secretory Carrier Membrane Protein 3	Protein Coding	39
CWF19L2	CWF19 Like Cell Cycle Control Factor 2	Protein Coding	32
ADRM1	Adhesion Regulating Molecule 1	Protein Coding	41
USP14	Ubiquitin Specific Peptidase 14	Protein Coding	47
ELOVL7	ELOVL Fatty Acid Elongase 7	Protein Coding	38
TCL1B	T Cell Leukemia/Lymphoma 1B	Protein Coding	36
C3orf62	Chromosome 3 Open Reading Frame 62	Protein Coding	30
METAP2	Methionyl Aminopeptidase 2	Protein Coding	44
SLCO2B1	Solute Carrier Organic Anion Transporter Family Mem	Protein Coding	45
PIAS4	Protein Inhibitor Of Activated STAT 4	Protein Coding	44
LINC0148	Long Intergenic Non-Protein Coding RNA 1483	RNA Gene	13
PPAN	Peter Pan Homolog	Protein Coding	34
UBE2B	Ubiquitin Conjugating Enzyme E2 B	Protein Coding	47
H2AC21	H2A Clustered Histone 21	Protein Coding	28
CREG1	Cellular Repressor Of E1A Stimulated Genes 1	Protein Coding	39
TRIM47	Tripartite Motif Containing 47	Protein Coding	35
DECR2	2,4-Dienoyl-CoA Reductase 2	Protein Coding	40
RAB26	RAB26, Member RAS Oncogene Family	Protein Coding	39
HTT-AS	HTT Antisense RNA	RNA Gene	16
PFDN2	Prefoldin Subunit 2	Protein Coding	37
SMC4	Structural Maintenance Of Chromosomes 4	Protein Coding	40
EIF3M	Eukaryotic Translation Initiation Factor 3 Subunit M	Protein Coding	37
ADI1	Acireductone Dioxygenase 1	Protein Coding	43
RPA4	Replication Protein A4	Protein Coding	36
NAA11	N-Alpha-Acetyltransferase 11, NatA Catalytic Subunit	Protein Coding	33
SLC14A2	Solute Carrier Family 14 Member 2	Protein Coding	41
PLPPR1	Phospholipid Phosphatase Related 1	Protein Coding	27
NPFFR1	Neuropeptide FF Receptor 1	Protein Coding	41
UBXN1	UBX Domain Protein 1	Protein Coding	36

GPR26	G Protein-Coupled Receptor 26	Protein Coding	37
RPL23AP5	Ribosomal Protein L23a Pseudogene 5	Pseudogene	9
RBBP6	RB Binding Protein 6, Ubiquitin Ligase	Protein Coding	40
AMBRA1	Autophagy And Beclin 1 Regulator 1	Protein Coding	40
HNRNPH3	Heterogeneous Nuclear Ribonucleoprotein H3	Protein Coding	40
ACTR1B	Actin Related Protein 1B	Protein Coding	39
ROPN1L	Rhophilin Associated Tail Protein 1 Like	Protein Coding	37
EME1	Essential Meiotic Structure-Specific Endonuclease 1	Protein Coding	41
ARFIP2	ADP Ribosylation Factor Interacting Protein 2	Protein Coding	40
CYB5R1	Cytochrome B5 Reductase 1	Protein Coding	43
CBX1	Chromobox 1	Protein Coding	40
FUNDC2	FUN14 Domain Containing 2	Protein Coding	35
UBXN10	UBX Domain Protein 10	Protein Coding	32
LAIR1	Leukocyte Associated Immunoglobulin Like Receptor 1	Protein Coding	41
SLC4A1A	Solute Carrier Family 4 Member 1 Adaptor Protein	Protein Coding	37
VAT1	Vesicle Amine Transport 1	Protein Coding	39
CLHC1	Clathrin Heavy Chain Linker Domain Containing 1	Protein Coding	31
ZNF2	Zinc Finger Protein 2	Protein Coding	36
GNB2	G Protein Subunit Beta 2	Protein Coding	43
GSTM5	Glutathione S-Transferase Mu 5	Protein Coding	42
ZNF764	Zinc Finger Protein 764	Protein Coding	35
SPATA2	Spermatogenesis Associated 2	Protein Coding	35
MIR548A	MicroRNA 548at	RNA Gene	12
LINC0069	Long Intergenic Non-Protein Coding RNA 693	RNA Gene	11
DBI	Diazepam Binding Inhibitor, Acyl-CoA Binding Protein	Protein Coding	47
CPXM1	Carboxypeptidase X, M14 Family Member 1	Protein Coding	37
TIGAR	TP53 Induced Glycolysis Regulatory Phosphatase	Protein Coding	39
UPF3A	UPF3A Regulator Of Nonsense Mediated MRNA Deca	Protein Coding	36
SF3A3	Splicing Factor 3a Subunit 3	Protein Coding	38
ORAI3	ORAI Calcium Release-Activated Calcium Modulator 3	Protein Coding	35
TXNL1	Thioredoxin Like 1	Protein Coding	40
PRB1	Proline Rich Protein BstNI Subfamily 1 (Gene/Pseudog	Protein Coding	35
ECHDC1	Ethylmalonyl-CoA Decarboxylase 1	Protein Coding	38
THEM4	Thioesterase Superfamily Member 4	Protein Coding	40
KCTD3	Potassium Channel Tetramerization Domain Containing	Protein Coding	35
C1D	C1D Nuclear Receptor Corepressor	Protein Coding	39
BLOC1S5	Biogenesis Of Lysosomal Organelles Complex 1 Subun	Protein Coding	35
GPANK1	G-Patch Domain And Ankyrin Repeats 1	Protein Coding	33
SEC61B	SEC61 Translocon Subunit Beta	Protein Coding	39
SLC2A14	Solute Carrier Family 2 Member 14	Protein Coding	35
MYOF	Myoferlin	Protein Coding	40
TDRKH	Tudor And KH Domain Containing	Protein Coding	39
PTPDC1	Protein Tyrosine Phosphatase Domain Containing 1	Protein Coding	33
FAM20B	FAM20B Glycosaminoglycan Xylosylkinase	Protein Coding	38
FBXO41	F-Box Protein 41	Protein Coding	32
CYB5RL	Cytochrome B5 Reductase Like	Protein Coding	31
DDX43	DEAD-Box Helicase 43	Protein Coding	37

C2orf49	Chromosome 2 Open Reading Frame 49	Protein Coding	32
FAM47B	Family With Sequence Similarity 47 Member B	Protein Coding	27
PTK6	Protein Tyrosine Kinase 6	Protein Coding	48
MIR1-1HC	MIR1-1HG Antisense RNA 1	RNA Gene	17
CYCSP38	CYCS Pseudogene 38	Pseudogene	10
TRMT2A	TRNA Methyltransferase 2 Homolog A	Protein Coding	39
STRAP	Serine/Threonine Kinase Receptor Associated Protein	Protein Coding	39
TPD52	Tumor Protein D52	Protein Coding	41
ABHD10	Abhydrolase Domain Containing 10	Protein Coding	37
ZCCHC8	Zinc Finger CCHC-Type Containing 8	Protein Coding	36
ATP6AP1l	ATPase H+ Transporting Accessory Protein 1 Like	Protein Coding	27
CDKL2	Cyclin Dependent Kinase Like 2	Protein Coding	39
SEPTIN11	Septin 11	Protein Coding	29
MRGBP	MRG Domain Binding Protein	Protein Coding	34
ACAD10	Acyl-CoA Dehydrogenase Family Member 10	Protein Coding	38
ITGB3BP	Integrin Subunit Beta 3 Binding Protein	Protein Coding	42
POU5F1P4	POU Class 5 Homeobox 1 Pseudogene 4	Pseudogene	16
HERC5	HECT And RLD Domain Containing E3 Ubiquitin Prot	Protein Coding	40
AGFG2	ArfGAP With FG Repeats 2	Protein Coding	35
MARCHF	Membrane Associated Ring-CH-Type Finger 10	Protein Coding	26
SH3BP5	SH3 Domain Binding Protein 5	Protein Coding	41
MRPL1	Mitochondrial Ribosomal Protein L1	Protein Coding	40
USP48	Ubiquitin Specific Peptidase 48	Protein Coding	40
POP7	POP7 Homolog, Ribonuclease P/MRP Subunit	Protein Coding	35
C2CD2	C2 Calcium Dependent Domain Containing 2	Protein Coding	35
WWP1	WW Domain Containing E3 Ubiquitin Protein Ligase 1	Protein Coding	43
TXNDC5	Thioredoxin Domain Containing 5	Protein Coding	40
RALY	RALY Heterogeneous Nuclear Ribonucleoprotein	Protein Coding	39
RCN3	Reticulocalbin 3	Protein Coding	35
NARF	Nuclear Prelamin A Recognition Factor	Protein Coding	38
EXOC3	Exocyst Complex Component 3	Protein Coding	38
TRUB1	TruB Pseudouridine Synthase Family Member 1	Protein Coding	38
SMAP1	Small ArfGAP 1	Protein Coding	37
GPRC5A	G Protein-Coupled Receptor Class C Group 5 Member	Protein Coding	42
ZBTB4	Zinc Finger And BTB Domain Containing 4	Protein Coding	35
ACAA2	Acetyl-CoA Acyltransferase 2	Protein Coding	45
ZNF688	Zinc Finger Protein 688	Protein Coding	33
OR14K1	Olfactory Receptor Family 14 Subfamily K Member 1	Protein Coding	21
TEKT4P2	Tektin 4 Pseudogene 2	Pseudogene	13
AVEN	Apoptosis And Caspase Activation Inhibitor	Protein Coding	37
TTK	TTK Protein Kinase	Protein Coding	47
MRPL10	Mitochondrial Ribosomal Protein L10	Protein Coding	37
TXLNGY	Taxilin Gamma Pseudogene, Y-Linked	Pseudogene	20
AGMAT	Agmatinase	Protein Coding	40
PALM	Paralemmin	Protein Coding	37
NOB1	NIN1 (RPN12) Binding Protein 1 Homolog	Protein Coding	39
DIDO1	Death Inducer-Obliterator 1	Protein Coding	38

FKBPL	FKBP Prolyl Isomerase Like	Protein Coding	37
IGSF9B	Immunoglobulin Superfamily Member 9B	Protein Coding	31
CDK3	Cyclin Dependent Kinase 3	Protein Coding	41
CBX3	Chromobox 3	Protein Coding	43
SRARP	Steroid Receptor Associated And Regulated Protein	Protein Coding	25
PELI1	Pellino E3 Ubiquitin Protein Ligase 1	Protein Coding	41
GCC1	GRIP And Coiled-Coil Domain Containing 1	Protein Coding	36
HYPK	Huntingtin Interacting Protein K	Protein Coding	33
SKA3	Spindle And Kinetochore Associated Complex Subunit :	Protein Coding	35
TTC27	Tetratricopeptide Repeat Domain 27	Protein Coding	33
SLC39A6	Solute Carrier Family 39 Member 6	Protein Coding	40
MED8	Mediator Complex Subunit 8	Protein Coding	36
STK16	Serine/Threonine Kinase 16	Protein Coding	40
RBM4B	RNA Binding Motif Protein 4B	Protein Coding	36
DNAJC10	DnaJ Heat Shock Protein Family (Hsp40) Member C10	Protein Coding	37
SQOR	Sulfide Quinone Oxidoreductase	Protein Coding	29
MTCP1	Mature T Cell Proliferation 1	Protein Coding	36
TWF1	Twinfilin Actin Binding Protein 1	Protein Coding	39
MTCO3P1	MT-CO3 Pseudogene 1	Pseudogene	8
TMCC2	Transmembrane And Coiled-Coil Domain Family 2	Protein Coding	36
PRRC1	Proline Rich Coiled-Coil 1	Protein Coding	33
TTC23	Tetratricopeptide Repeat Domain 23	Protein Coding	33
MRPL49	Mitochondrial Ribosomal Protein L49	Protein Coding	37
UBE2H	Ubiquitin Conjugating Enzyme E2 H	Protein Coding	45
LOC72839	Uncharacterized LOC728392	Protein Coding	16
P4HA3-AS	P4HA3 Antisense RNA 1	RNA Gene	10
SORD2P	Sorbitol Dehydrogenase 2, Pseudogene	Pseudogene	9
LOC10050	Uncharacterized LOC100506725	RNA Gene	8
LRRTM1	Leucine Rich Repeat Transmembrane Neuronal 1	Protein Coding	40
DBF4B	DBF4 Zinc Finger B	Protein Coding	34
SYNGR3	Synaptogyrin 3	Protein Coding	37
MIR190A	MicroRNA 190a	RNA Gene	20
PLXNA3	Plexin A3	Protein Coding	40
QPCTL	Glutaminyl-Peptide Cyclotransferase Like	Protein Coding	35
STOML1	Stomatin Like 1	Protein Coding	37
ATRAID	All-Trans Retinoic Acid Induced Differentiation Factor	Protein Coding	36
RAB3C	RAB3C, Member RAS Oncogene Family	Protein Coding	37
ZW10	Zw10 Kinetochore Protein	Protein Coding	39
ZNF184	Zinc Finger Protein 184	Protein Coding	38
CMPK1	Cytidine/Uridine Monophosphate Kinase 1	Protein Coding	44
KAT7	Lysine Acetyltransferase 7	Protein Coding	44
NELFB	Negative Elongation Factor Complex Member B	Protein Coding	36
RMND5A	Required For Meiotic Nuclear Division 5 Homolog A	Protein Coding	37
TIPARP	TCDD Inducible Poly(ADP-Ribose) Polymerase	Protein Coding	36
DNPEP	Aspartyl Aminopeptidase	Protein Coding	40
ELMOD1	ELMO Domain Containing 1	Protein Coding	36
RIOK1	RIO Kinase 1	Protein Coding	41

RPF2	Ribosome Production Factor 2 Homolog	Protein Coding	35
PTMS	Parathymosin	Protein Coding	38
GNL2	G Protein Nucleolar 2	Protein Coding	38
RBBP7	RB Binding Protein 7, Chromatin Remodeling Factor	Protein Coding	41
WASH3P	WASP Family Homolog 3, Pseudogene	Pseudogene	21
STARD10	StAR Related Lipid Transfer Domain Containing 10	Protein Coding	39
MXD1	MAX Dimerization Protein 1	Protein Coding	39
MAT2B	Methionine Adenosyltransferase 2B	Protein Coding	39
ITGB5	Integrin Subunit Beta 5	Protein Coding	47
KLHDC1	Kelch Domain Containing 1	Protein Coding	35
CCDC43	Coiled-Coil Domain Containing 43	Protein Coding	33
RSRC2	Arginine And Serine Rich Coiled-Coil 2	Protein Coding	33
ILRUN	Inflammation And Lipid Regulator With UBA-Like Anc	Protein Coding	27
SELENOI	Selenoprotein I	Protein Coding	31
TXNDC12	Thioredoxin Domain Containing 12	Protein Coding	40
WBP1L	WW Domain Binding Protein 1 Like	Protein Coding	31
PACSIN2	Protein Kinase C And Casein Kinase Substrate In Neur	Protein Coding	41
MDN1	Midasin AAA ATPase 1	Protein Coding	38
PCYT1B	Phosphate Cytidylyltransferase 1, Choline, Beta	Protein Coding	42
LSAMP	Limbic System Associated Membrane Protein	Protein Coding	39
GOSR1	Golgi SNAP Receptor Complex Member 1	Protein Coding	40
CDK11A	Cyclin Dependent Kinase 11A	Protein Coding	39
ZNF83	Zinc Finger Protein 83	Protein Coding	33
TP53TG3C	TP53 Target 3C	Protein Coding	22
LDLRAD4	LDLRAD4 Antisense RNA 1	RNA Gene	12
CFAP97D	CFAP97 Domain Containing 2	Protein Coding	11
LOC64332	Uncharacterized LOC643327	RNA Gene	9
LOC10099	Uncharacterized LOC100996842	Protein Coding	8
LOC10192	Uncharacterized LOC101927967	RNA Gene	8
KCNIP1	Potassium Voltage-Gated Channel Interacting Protein 1	Protein Coding	41
MTCH1	Mitochondrial Carrier 1	Protein Coding	39
YIPF3	Yip1 Domain Family Member 3	Protein Coding	36
DDX19B	DEAD-Box Helicase 19B	Protein Coding	37
CEP170	Centrosomal Protein 170	Protein Coding	40
OXSR1	Oxidative Stress Responsive Kinase 1	Protein Coding	43
CIART	Circadian Associated Repressor Of Transcription	Protein Coding	30
SLC38A2	Solute Carrier Family 38 Member 2	Protein Coding	43
DTWD2	DTW Domain Containing 2	Protein Coding	32
TMOD4	Tropomodulin 4	Protein Coding	39
MIR626	MicroRNA 626	RNA Gene	14
SESN3	Sestrin 3	Protein Coding	40
FLNC-AS	FLNC Antisense RNA 1	RNA Gene	11
MTA2	Metastasis Associated 1 Family Member 2	Protein Coding	41
DYNC1LI	Dynein Cytoplasmic 1 Light Intermediate Chain 2	Protein Coding	36
NOP16	NOP16 Nucleolar Protein	Protein Coding	33
BOD1	Biorientation Of Chromosomes In Cell Division 1	Protein Coding	36
CNPY2	Canopy FGF Signaling Regulator 2	Protein Coding	36

DMRTA1	DMRT Like Family A1	Protein Coding	36
ADAP2	ArfGAP With Dual PH Domains 2	Protein Coding	37
RAB14	RAB14, Member RAS Oncogene Family	Protein Coding	40
OSBPL11	Oxysterol Binding Protein Like 11	Protein Coding	36
RIOK2	RIO Kinase 2	Protein Coding	40
RHBDD1	Rhomboid Domain Containing 1	Protein Coding	36
CDK17	Cyclin Dependent Kinase 17	Protein Coding	40
NUP50	Nucleoporin 50	Protein Coding	40
SGTA	Small Glutamine Rich Tetratricopeptide Repeat Contair	Protein Coding	39
SNRNP40	Small Nuclear Ribonucleoprotein U5 Subunit 40	Protein Coding	37
KANSL1-/	KANSL1 Antisense RNA 1	RNA Gene	16
FBXL7	F-Box And Leucine Rich Repeat Protein 7	Protein Coding	36
THAP4	THAP Domain Containing 4	Protein Coding	34
ABCE1	ATP Binding Cassette Subfamily E Member 1	Protein Coding	40
ZNF22	Zinc Finger Protein 22	Protein Coding	36
NUAK2	NUAK Family Kinase 2	Protein Coding	40
ZNF165	Zinc Finger Protein 165	Protein Coding	36
SPC24	SPC24 Component Of NDC80 Kinetochore Complex	Protein Coding	35
OLFM1	Olfactomedin 1	Protein Coding	41
SNORD35	Small Nucleolar RNA, C/D Box 35A	RNA Gene	16
LOC10537	Uncharacterized LOC105372310	RNA Gene	6
CINP	Cyclin Dependent Kinase 2 Interacting Protein	Protein Coding	36
SPATA13	Spermatogenesis Associated 13	Protein Coding	38
AIFM3	Apoptosis Inducing Factor Mitochondria Associated 3	Protein Coding	39
SNURF	SNRPN Upstream Reading Frame	Protein Coding	33
KIRREL3-	KIRREL3 Antisense RNA 3	RNA Gene	20
ELMOD2	ELMO Domain Containing 2	Protein Coding	36
ARAP1	ArfGAP With RhoGAP Domain, Ankyrin Repeat And I	Protein Coding	41
EIF4G2	Eukaryotic Translation Initiation Factor 4 Gamma 2	Protein Coding	43
TRIM46	Tripartite Motif Containing 46	Protein Coding	35
MIR663AI	MIR663A Host Gene	RNA Gene	13
PREB	Prolactin Regulatory Element Binding	Protein Coding	41
TTLL6	Tubulin Tyrosine Ligase Like 6	Protein Coding	36
CLK1	CDC Like Kinase 1	Protein Coding	43
PCDHGC3	Protocadherin Gamma Subfamily C, 3	Protein Coding	37
lnc-HLA-DQA1-9		RNA Gene	4
lnc-HLA-DRB1-7		RNA Gene	4
DNAJB14	DnaJ Heat Shock Protein Family (Hsp40) Member B14	Protein Coding	36
COPB1	COPI Coat Complex Subunit Beta 1	Protein Coding	39
SLAIN2	SLAIN Motif Family Member 2	Protein Coding	35
RAP1GDS	Rap1 GTPase-GDP Dissociation Stimulator 1	Protein Coding	43
LZTS3	Leucine Zipper Tumor Suppressor Family Member 3	Protein Coding	32
MLEC	Malectin	Protein Coding	38
ZFP64	ZFP64 Zinc Finger Protein	Protein Coding	37
IGHA1	Immunoglobulin Heavy Constant Alpha 1	Protein Coding	29
CWC15	CWC15 Spliceosome Associated Protein Homolog	Protein Coding	33
CORO1C	Coronin 1C	Protein Coding	40

APLF	Aprataxin And PNKP Like Factor	Protein Coding	37
ZNF831	Zinc Finger Protein 831	Protein Coding	33
HNRNPL	Heterogeneous Nuclear Ribonucleoprotein L	Protein Coding	40
ALDOC	Aldolase, Fructose-Bisphosphate C	Protein Coding	41
HSP90AA4	Heat Shock Protein 90 Alpha Family Class A Member 4	Pseudogene	17
C15orf39	Chromosome 15 Open Reading Frame 39	Protein Coding	35
MOB1A	MOB Kinase Activator 1A	Protein Coding	41
RTF1	RTF1 Homolog, Paf1/RNA Polymerase II Complex Co	Protein Coding	37
PCGF3	Polycomb Group Ring Finger 3	Protein Coding	35
MESD	Mesoderm Development LRP Chaperone	Protein Coding	32
CENPI	Centromere Protein I	Protein Coding	35
HMCES	5-Hydroxymethylcytosine Binding, ES Cell Specific	Protein Coding	31
RASGRP3	RAS Guanyl Releasing Protein 3	Protein Coding	43
DDX18	DEAD-Box Helicase 18	Protein Coding	41
ELF2	E74 Like ETS Transcription Factor 2	Protein Coding	39
PIP5K1A	Phosphatidylinositol-4-Phosphate 5-Kinase Type 1 Alph	Protein Coding	45
HTR2A-A	HTR2A Antisense RNA 1	RNA Gene	14
RHBDL1	Rhomboid Like 1	Protein Coding	36
MZF1	Myeloid Zinc Finger 1	Protein Coding	36
INKA1	Inka Box Actin Regulator 1	Protein Coding	26
MRPL35	Mitochondrial Ribosomal Protein L35	Protein Coding	36
MPST	Mercaptopyruvate Sulfurtransferase	Protein Coding	44
CSTL1	Cystatin Like 1	Protein Coding	32
CLIC4	Chloride Intracellular Channel 4	Protein Coding	41
PDE1A	Phosphodiesterase 1A	Protein Coding	42
HP1BP3	Heterochromatin Protein 1 Binding Protein 3	Protein Coding	38
FMO1	Flavin Containing Dimethylaniline Monoxygenase 1	Protein Coding	44
LOC10272	Uncharacterized LOC102723370	RNA Gene	4
NUDT9	Nudix Hydrolase 9	Protein Coding	40
ZFYVE16	Zinc Finger FYVE-Type Containing 16	Protein Coding	40
AIDA	Axin Interactor, Dorsalization Associated	Protein Coding	34
UGGT2	UDP-Glucose Glycoprotein Glucosyltransferase 2	Protein Coding	37
POLR3D	RNA Polymerase III Subunit D	Protein Coding	37
USP10	Ubiquitin Specific Peptidase 10	Protein Coding	46
IPCEF1	Interaction Protein For Cytohesin Exchange Factors 1	Protein Coding	36
ERMN	Ermin	Protein Coding	36
RHBDD2	Rhomboid Domain Containing 2	Protein Coding	37
SHISA6	Shisa Family Member 6	Protein Coding	34
PPA1	Inorganic Pyrophosphatase 1	Protein Coding	42
TMEM72-	TMEM72 Antisense RNA 1	RNA Gene	16
DEDD	Death Effector Domain Containing	Protein Coding	36
GDPD3	Glycerophosphodiester Phosphodiesterase Domain Cont	Protein Coding	33
PRR16	Proline Rich 16	Protein Coding	34
HIC2	HIC ZBTB Transcriptional Repressor 2	Protein Coding	36
ANKRA2	Ankyrin Repeat Family A Member 2	Protein Coding	37
USP13	Ubiquitin Specific Peptidase 13	Protein Coding	44
ZNF286B	Zinc Finger Protein 286B (Pseudogene)	Pseudogene	26

ANP32B	Acidic Nuclear Phosphoprotein 32 Family Member B	Protein Coding	37
CHTOP	Chromatin Target Of PRMT1	Protein Coding	34
DOK1	Docking Protein 1	Protein Coding	44
CAMKV	CaM Kinase Like Vesicle Associated	Protein Coding	39
DUT	Deoxyuridine Triphosphatase	Protein Coding	44
MRPS18B	Mitochondrial Ribosomal Protein S18B	Protein Coding	37
POLR3C	RNA Polymerase III Subunit C	Protein Coding	37
FZD10	Frizzled Class Receptor 10	Protein Coding	44
MAGEH1	MAGE Family Member H1	Protein Coding	35
EMC8	ER Membrane Protein Complex Subunit 8	Protein Coding	33
ECH1	Enoyl-CoA Hydratase 1	Protein Coding	41
GOLGA5	Golgin A5	Protein Coding	39
POLD3	DNA Polymerase Delta 3, Accessory Subunit	Protein Coding	41
ZNF747	Zinc Finger Protein 747	Protein Coding	33
ZMAT4	Zinc Finger Matrin-Type 4	Protein Coding	34
NOL7	Nucleolar Protein 7	Protein Coding	35
VAT1L	Vesicle Amine Transport 1 Like	Protein Coding	36
CSNK1G1	Casein Kinase 1 Gamma 1	Protein Coding	44
USPL1	Ubiquitin Specific Peptidase Like 1	Protein Coding	33
ARL4C	ADP Ribosylation Factor Like GTPase 4C	Protein Coding	36
BIK	BCL2 Interacting Killer	Protein Coding	40
RASSF3	Ras Association Domain Family Member 3	Protein Coding	37
SFXN5	Sideroflexin 5	Protein Coding	39
WSB1	WD Repeat And SOCS Box Containing 1	Protein Coding	42
SLIRP	SRA Stem-Loop Interacting RNA Binding Protein	Protein Coding	35
ZMAT3	Zinc Finger Matrin-Type 3	Protein Coding	36
PEAK1	Pseudopodium Enriched Atypical Kinase 1	Protein Coding	35
PWP1	PWP1 Homolog, Endonuclein	Protein Coding	36
RPS6KA6	Ribosomal Protein S6 Kinase A6	Protein Coding	44
INO80C	INO80 Complex Subunit C	Protein Coding	35
ZCCHC17	Zinc Finger CCHC-Type Containing 17	Protein Coding	35
NKAP	NFKB Activating Protein	Protein Coding	35
ENSG00000237631		Pseudogene	2
ERP44	Endoplasmic Reticulum Protein 44	Protein Coding	41
SERF1B	Small EDRK-Rich Factor 1B	Protein Coding	31
CDC42BP	CDC42 Binding Protein Kinase Beta	Protein Coding	42
FGFBP1	Fibroblast Growth Factor Binding Protein 1	Protein Coding	40
MIR187	MicroRNA 187	RNA Gene	18
SENP2	SUMO Specific Peptidase 2	Protein Coding	41
RNR1	RNA, Ribosomal 45S Cluster 1	RNA Gene	10
MAP1S	Microtubule Associated Protein 1S	Protein Coding	36
NFE2L1	Nuclear Factor, Erythroid 2 Like 1	Protein Coding	44
INIP	INTS3 And NABP Interacting Protein	Protein Coding	34
MPC2	Mitochondrial Pyruvate Carrier 2	Protein Coding	39
SMG1P5	SMG1 Pseudogene 5	Pseudogene	12
TPCN1	Two Pore Segment Channel 1	Protein Coding	37
HSDL2	Hydroxysteroid Dehydrogenase Like 2	Protein Coding	35

PCYOX1	Prenylcysteine Oxidase 1	Protein Coding	39
CYB5D1	Cytochrome B5 Domain Containing 1	Protein Coding	32
SPCS3	Signal Peptidase Complex Subunit 3	Protein Coding	37
RNF34	Ring Finger Protein 34	Protein Coding	41
COX17	Cytochrome C Oxidase Copper Chaperone COX17	Protein Coding	40
ATP6V0C	ATPase H+ Transporting V0 Subunit C	Protein Coding	41
SPCS2	Signal Peptidase Complex Subunit 2	Protein Coding	36
APOO	Apolipoprotein O	Protein Coding	35
MTG1	Mitochondrial Ribosome Associated GTPase 1	Protein Coding	36
DMKN	Dermokine	Protein Coding	35
PIMREG	PICALM Interacting Mitotic Regulator	Protein Coding	28
TAF9	TATA-Box Binding Protein Associated Factor 9	Protein Coding	41
RPS12P5	Ribosomal Protein S12 Pseudogene 5	Pseudogene	6
MICU3	Mitochondrial Calcium Uptake Family Member 3	Protein Coding	31
EVI2A	Ecotropic Viral Integration Site 2A	Protein Coding	36
ENSG00000283563		Protein Coding	7
SPCS1	Signal Peptidase Complex Subunit 1	Protein Coding	37
MED24	Mediator Complex Subunit 24	Protein Coding	38
ZNF622	Zinc Finger Protein 622	Protein Coding	36
SH3RF2	SH3 Domain Containing Ring Finger 2	Protein Coding	38
ATP6V1G	ATPase H+ Transporting V1 Subunit G1	Protein Coding	39
ARHGDIB	Rho GDP Dissociation Inhibitor Beta	Protein Coding	43
TPRA1	Transmembrane Protein Adipocyte Associated 1	Protein Coding	35
SUMF2	Sulfatase Modifying Factor 2	Protein Coding	38
BET1L	Bet1 Golgi Vesicular Membrane Trafficking Protein Lik	Protein Coding	36
MIRLET7.	MicroRNA Let-7a-2	RNA Gene	21
OR2J3	Olfactory Receptor Family 2 Subfamily J Member 3	Protein Coding	34
H2BC1	H2B Clustered Histone 1	Protein Coding	28
MED9	Mediator Complex Subunit 9	Protein Coding	36
KCNK17	Potassium Two Pore Domain Channel Subfamily K Me	Protein Coding	37
MAP4	Microtubule Associated Protein 4	Protein Coding	42
WDR1	WD Repeat Domain 1	Protein Coding	39
SYNGR2	Synaptogyrin 2	Protein Coding	39
FAM3A	FAM3 Metabolism Regulating Signaling Molecule A	Protein Coding	36
PLAGL2	PLAG1 Like Zinc Finger 2	Protein Coding	37
PRKRIP1	PRKR Interacting Protein 1	Protein Coding	33
MOB1B	MOB Kinase Activator 1B	Protein Coding	39
LOC10928	TBX3 Promoter Region	Biological Region	2
LAMB4	Laminin Subunit Beta 4	Protein Coding	35
OSBPL5	Oxysterol Binding Protein Like 5	Protein Coding	41
L3HYPDH	Trans-L-3-Hydroxyproline Dehydratase	Protein Coding	32
NMUR2	Neuromedin U Receptor 2	Protein Coding	40
CIAO3	Cytosolic Iron-Sulfur Assembly Component 3	Protein Coding	29
GIPC1	GIPC PDZ Domain Containing Family Member 1	Protein Coding	41
LSM6	LSM6 Homolog, U6 Small Nuclear RNA And MRNA I	Protein Coding	37
SMG5	SMG5 Nonsense Mediated MRNA Decay Factor	Protein Coding	36
NCBP3	Nuclear Cap Binding Subunit 3	Protein Coding	27

TEX2	Testis Expressed 2	Protein Coding	33
NIT1	Nitrilase 1	Protein Coding	38
MTARC1	Mitochondrial Amidoxime Reducing Component 1	Protein Coding	28
RPS12P4	Ribosomal Protein S12 Pseudogene 4	Pseudogene	7
CASC4	Cancer Susceptibility 4	Protein Coding	36
CAPZA1	Capping Actin Protein Of Muscle Z-Line Subunit Alpha	Protein Coding	41
C1orf87	Chromosome 1 Open Reading Frame 87	Protein Coding	32
SYT10	Synaptotagmin 10	Protein Coding	33
OXER1	Oxoeicosanoid Receptor 1	Protein Coding	39
SYF2	SYF2 Pre-MRNA Splicing Factor	Protein Coding	36
HMBOX1	Homeobox Containing 1	Protein Coding	36
GPR78	G Protein-Coupled Receptor 78	Protein Coding	33
DUSP4	Dual Specificity Phosphatase 4	Protein Coding	44
MTARC2	Mitochondrial Amidoxime Reducing Component 2	Protein Coding	30
ATG3	Autophagy Related 3	Protein Coding	43
OTUB2	OTU Deubiquitinase, Ubiquitin Aldehyde Binding 2	Protein Coding	39
CAMKK1	Calcium/Calmodulin Dependent Protein Kinase Kinase	Protein Coding	46
ELK3	ETS Transcription Factor ELK3	Protein Coding	40
METTL16	Methyltransferase Like 16	Protein Coding	31
B3GALT4	Beta-1,3-Galactosyltransferase 4	Protein Coding	40
CHL1-AS1	CHL1 Antisense RNA 1	RNA Gene	13
MPV17L	MPV17 Mitochondrial Inner Membrane Protein Like	Protein Coding	33
CDCA3	Cell Division Cycle Associated 3	Protein Coding	39
ORM2	Orosomucoid 2	Protein Coding	39
CSNK1G2	Casein Kinase 1 Gamma 2	Protein Coding	47
FOXS1	Forkhead Box S1	Protein Coding	35
MRPL52	Mitochondrial Ribosomal Protein L52	Protein Coding	34
RPP25	Ribonuclease P And MRP Subunit P25	Protein Coding	35
UBE2D4	Ubiquitin Conjugating Enzyme E2 D4 (Putative)	Protein Coding	42
TRAM1	Translocation Associated Membrane Protein 1	Protein Coding	40
ZADH2	Zinc Binding Alcohol Dehydrogenase Domain Containi	Protein Coding	37
RNR2	RNA, Ribosomal 45S Cluster 2	RNA Gene	10
PLEKHN1	Pleckstrin Homology Domain Containing N1	Protein Coding	31
PAXX	PAXX Non-Homologous End Joining Factor	Protein Coding	27
SSH3	Slingshot Protein Phosphatase 3	Protein Coding	40
NIPSNAP2	Nipsnap Homolog 2	Protein Coding	31
ENC1	Ectodermal-Neural Cortex 1	Protein Coding	38
GRSF1	G-Rich RNA Sequence Binding Factor 1	Protein Coding	38
CYP1B1-A	CYP1B1 Antisense RNA 1	RNA Gene	21
LINC0258	Long Intergenic Non-Protein Coding RNA 2582	RNA Gene	11
ESYT1	Extended Synaptotagmin 1	Protein Coding	39
NENF	Neudesin Neurotrophic Factor	Protein Coding	40
TCP10L	T-Complex 10 Like	Protein Coding	36
STRIP2	Striatin Interacting Protein 2	Protein Coding	35
KCNH4	Potassium Voltage-Gated Channel Subfamily H Membe	Protein Coding	38
RAD1	RAD1 Checkpoint DNA Exonuclease	Protein Coding	40
TSPAN5	Tetraspanin 5	Protein Coding	39

CENPS	Centromere Protein S	Protein Coding	29
PLAAT5	Phospholipase A And Acyltransferase 5	Protein Coding	28
CIAO1	Cytosolic Iron-Sulfur Assembly Component 1	Protein Coding	41
CAP1	Cyclase Associated Actin Cytoskeleton Regulatory Prot	Protein Coding	41
HAUS8	HAUS Augmin Like Complex Subunit 8	Protein Coding	35
MED19	Mediator Complex Subunit 19	Protein Coding	34
MMGT1	Membrane Magnesium Transporter 1	Protein Coding	33
LENG1	Leukocyte Receptor Cluster Member 1	Protein Coding	32
RETSAT	Retinol Saturase	Protein Coding	40
DUS3L	Dihydrouridine Synthase 3 Like	Protein Coding	35
UBXN4	UBX Domain Protein 4	Protein Coding	36
TAF7L	TATA-Box Binding Protein Associated Factor 7 Like	Protein Coding	35
PPME1	Protein Phosphatase Methylesterase 1	Protein Coding	39
UBE2O	Ubiquitin Conjugating Enzyme E2 O	Protein Coding	41
TTC39A	Tetratricopeptide Repeat Domain 39A	Protein Coding	33
INTS12	Integrator Complex Subunit 12	Protein Coding	34
FAIM2	Fas Apoptotic Inhibitory Molecule 2	Protein Coding	37
DNTTIP2	Deoxynucleotidyltransferase Terminal Interacting Prote	Protein Coding	35
FCRLB	Fc Receptor Like B	Protein Coding	36
IKBIP	IKBKB Interacting Protein	Protein Coding	36
BPIFB3	BPI Fold Containing Family B Member 3	Protein Coding	33
TP53TG1	TP53 Target 1	RNA Gene	24
IBGC1	Idiopathic Basal Ganglia Calcification 1	Genetic Locus	4
SPICE1	Spindle And Centriole Associated Protein 1	Protein Coding	34
MYO1F	Myosin IF	Protein Coding	40
DHRSX	Dehydrogenase/Reductase X-Linked	Protein Coding	34
OSTC	Oligosaccharyltransferase Complex Non-Catalytic Subu	Protein Coding	35
PPFIBP2	PPFIA Binding Protein 2	Protein Coding	39
LINC0143	Long Intergenic Non-Protein Coding RNA 1432	RNA Gene	13
NRCAM	Neuronal Cell Adhesion Molecule	Protein Coding	43
NAPA	NSF Attachment Protein Alpha	Protein Coding	40
ASMTL	Acetylserotonin O-Methyltransferase Like	Protein Coding	36
LRRC1	Leucine Rich Repeat Containing 1	Protein Coding	37
LINC0047	Long Intergenic Non-Protein Coding RNA 476	RNA Gene	20
CUTA	CutA Divalent Cation Tolerance Homolog	Protein Coding	37
ATP5MF	ATP Synthase Membrane Subunit F	Protein Coding	29
MT1X	Metallothionein 1X	Protein Coding	41
NLK	Nemo Like Kinase	Protein Coding	44
GNRH2	Gonadotropin Releasing Hormone 2	Protein Coding	35
TRI-AAT4	TRNA-Ile (Anticodon AAT) 4-1	RNA Gene	9
TRS-AGA	TRNA-Ser (Anticodon AGA) 2-6	RNA Gene	9
TRT-AGT	TRNA-Thr (Anticodon AGT) 1-2	RNA Gene	9
OR9Q1	Olfactory Receptor Family 9 Subfamily Q Member 1	Protein Coding	33
RTKN	Rhotekin	Protein Coding	40
YKT6	YKT6 V-SNARE Homolog	Protein Coding	41
ECI1	Enoyl-CoA Delta Isomerase 1	Protein Coding	41
NSRP1	Nuclear Speckle Splicing Regulatory Protein 1	Protein Coding	34

PRXL2A	Peroxiredoxin Like 2A	Protein Coding	28
RALGDS	Ral Guanine Nucleotide Dissociation Stimulator	Protein Coding	43
ARHGEF4	Rho Guanine Nucleotide Exchange Factor 40	Protein Coding	35
ARL8B	ADP Ribosylation Factor Like GTPase 8B	Protein Coding	37
CCDC107	Coiled-Coil Domain Containing 107	Protein Coding	33
PNN	Pinin, Desmosome Associated Protein	Protein Coding	37
NUSAP1	Nucleolar And Spindle Associated Protein 1	Protein Coding	35
SOX7	SRY-Box Transcription Factor 7	Protein Coding	36
ALDH16A	Aldehyde Dehydrogenase 16 Family Member A1	Protein Coding	37
TMPRSS9	Transmembrane Serine Protease 9	Protein Coding	36
LIMCH1	LIM And Calponin Homology Domains 1	Protein Coding	38
FAM122A	Family With Sequence Similarity 122A	Protein Coding	32
BOLA1	BolA Family Member 1	Protein Coding	37
CSTF2	Cleavage Stimulation Factor Subunit 2	Protein Coding	40
RAD51AP	RAD51 Associated Protein 1	Protein Coding	36
CSAG1	Chondrosarcoma Associated Gene 1	Protein Coding	27
UBE2Q1-A	UBE2Q1 Antisense RNA 1	RNA Gene	11
LOC10192	Uncharacterized LOC101928120	Protein Coding	10
TPM1-AS	TPM1 Antisense RNA	RNA Gene	10
LINC0255	Long Intergenic Non-Protein Coding RNA 2555	RNA Gene	10
ENSG00000243295		RNA Gene	9
LINC0247	Long Intergenic Non-Protein Coding RNA 2471	RNA Gene	9
RNU6-116	RNA, U6 Small Nuclear 1169, Pseudogene	Pseudogene	8
ENSG00000258407		RNA Gene	8
CICP26	Capicua Transcriptional Repressor Pseudogene 26	Pseudogene	8
RNU7-97P	RNA, U7 Small Nuclear 97 Pseudogene	Pseudogene	6
ENSG00000271267		Pseudogene	5
ARHGEF5	Rho Guanine Nucleotide Exchange Factor 5	Protein Coding	39
AFAP1L2	Actin Filament Associated Protein 1 Like 2	Protein Coding	37
ERG28	Ergosterol Biosynthesis 28 Homolog	Protein Coding	28
PABPC1L	Poly(A) Binding Protein Cytoplasmic 1 Like	Protein Coding	34
TMED9	Transmembrane P24 Trafficking Protein 9	Protein Coding	36
LMAN2	Lectin, Mannose Binding 2	Protein Coding	39
MON1A	MON1 Homolog A, Secretory Trafficking Associated	Protein Coding	37
ZFC3H1	Zinc Finger C3H1-Type Containing	Protein Coding	35
IPO4	Importin 4	Protein Coding	36
ZDHHC23	Zinc Finger DHHC-Type Palmitoyltransferase 23	Protein Coding	35
AGR3	Anterior Gradient 3, Protein Disulphide Isomerase Fami	Protein Coding	39
SKAP1	Src Kinase Associated Phosphoprotein 1	Protein Coding	41
PAQR3	Progestin And AdipoQ Receptor Family Member 3	Protein Coding	35
PARP2	Poly(ADP-Ribose) Polymerase 2	Protein Coding	47
PGLS	6-Phosphogluconolactonase	Protein Coding	40
EAF1	ELL Associated Factor 1	Protein Coding	36
TAX1BP1	Tax1 Binding Protein 1	Protein Coding	42
FKBP8	FKBP Prolyl Isomerase 8	Protein Coding	44
DIXDC1	DIX Domain Containing 1	Protein Coding	35
DMAC1	Distal Membrane Arm Assembly Complex 1	Protein Coding	24

ARRDC1	Arrestin Domain Containing 1	Protein Coding	35
PAAF1	Proteasomal ATPase Associated Factor 1	Protein Coding	35
ARIH2	Ariadne RBR E3 Ubiquitin Protein Ligase 2	Protein Coding	39
PUS7L	Pseudouridine Synthase 7 Like	Protein Coding	35
RGL1	Ral Guanine Nucleotide Dissociation Stimulator Like 1	Protein Coding	40
ASB6	Ankyrin Repeat And SOCS Box Containing 6	Protein Coding	36
CCSER2	Coiled-Coil Serine Rich Protein 2	Protein Coding	31
RIMS4	Regulating Synaptic Membrane Exocytosis 4	Protein Coding	35
CENPQ	Centromere Protein Q	Protein Coding	37
NTMT1	N-Terminal Xaa-Pro-Lys N-Methyltransferase 1	Protein Coding	39
PLA1A	Phospholipase A1 Member A	Protein Coding	41
ATP6V0D	ATPase H+ Transporting V0 Subunit D1	Protein Coding	43
BZW2	Basic Leucine Zipper And W2 Domains 2	Protein Coding	37
ERGIC2	ERGIC And Golgi 2	Protein Coding	37
PTPN23	Protein Tyrosine Phosphatase Non-Receptor Type 23	Protein Coding	39
SYPL1	Synaptophysin Like 1	Protein Coding	38
MT1G	Metallothionein 1G	Protein Coding	35
GDA	Guanine Deaminase	Protein Coding	44
COPS8	COP9 Signalosome Subunit 8	Protein Coding	37
CBX4	Chromobox 4	Protein Coding	44
HDDC3	HD Domain Containing 3	Protein Coding	35
SYNE3	Spectrin Repeat Containing Nuclear Envelope Family M	Protein Coding	36
TSGA13	Testis Specific 13	Protein Coding	27
TMX1	Thioredoxin Related Transmembrane Protein 1	Protein Coding	39
DPP3	Dipeptidyl Peptidase 3	Protein Coding	40
MRPL24	Mitochondrial Ribosomal Protein L24	Protein Coding	39
EIF4E2	Eukaryotic Translation Initiation Factor 4E Family Mer	Protein Coding	40
BEGAIN	Brain Enriched Guanylate Kinase Associated	Protein Coding	36
ORMDL1	ORMDL Sphingolipid Biosynthesis Regulator 1	Protein Coding	35
VRTN	Vertebrae Development Associated	Protein Coding	30
ARL4D	ADP Ribosylation Factor Like GTPase 4D	Protein Coding	36
C6orf118	Chromosome 6 Open Reading Frame 118	Protein Coding	31
OCIAD2	OCIA Domain Containing 2	Protein Coding	37
ANKZF1	Ankyrin Repeat And Zinc Finger Peptidyl TRNA Hydro	Protein Coding	36
LRRC8D	Leucine Rich Repeat Containing 8 VRAC Subunit D	Protein Coding	36
TOMM7	Translocase Of Outer Mitochondrial Membrane 7	Protein Coding	37
SPX	Spexin Hormone	Protein Coding	29
LRRC37A	Leucine Rich Repeat Containing 37 Member A2	Protein Coding	30
CCNT2-A:	CCNT2 Antisense RNA 1	RNA Gene	15
PABPN1L	PABPN1 Like, Cytoplasmic	Protein Coding	30
LINC0222	Long Intergenic Non-Protein Coding RNA 2224	RNA Gene	9
MRPL20	Mitochondrial Ribosomal Protein L20	Protein Coding	37
CDK16	Cyclin Dependent Kinase 16	Protein Coding	42
IGSF11	Immunoglobulin Superfamily Member 11	Protein Coding	37
NXT2	Nuclear Transport Factor 2 Like Export Factor 2	Protein Coding	36
ECHDC2	Enoyl-CoA Hydratase Domain Containing 2	Protein Coding	36
ETNPPL	Ethanolamine-Phosphate Phospho-Lyase	Protein Coding	36

DDX42	DEAD-Box Helicase 42	Protein Coding	40
RAB13	RAB13, Member RAS Oncogene Family	Protein Coding	40
LINC0221	LINC02210-CRHR1 Readthrough	Protein Coding	14
RCC2	Regulator Of Chromosome Condensation 2	Protein Coding	40
DLGAP5	DLG Associated Protein 5	Protein Coding	39
DSN1	DSN1 Component Of MIS12 Kinetochore Complex	Protein Coding	37
C8orf86	Chromosome 8 Open Reading Frame 86	Protein Coding	28
TIGD1	Tigger Transposable Element Derived 1	Protein Coding	32
DCTN6	Dynactin Subunit 6	Protein Coding	36
SPATA46	Spermatogenesis Associated 46	Protein Coding	26
CAAP1	Caspase Activity And Apoptosis Inhibitor 1	Protein Coding	31
LDLRAD1	Low Density Lipoprotein Receptor Class A Domain Co	Protein Coding	29
MYL10	Myosin Light Chain 10	Protein Coding	36
ZSCAN21	Zinc Finger And SCAN Domain Containing 21	Protein Coding	36
LYPLA2	Lysophospholipase 2	Protein Coding	40
SCG3	Secretogranin III	Protein Coding	41
ARPC4	Actin Related Protein 2/3 Complex Subunit 4	Protein Coding	38
DNAJC11	DnaJ Heat Shock Protein Family (Hsp40) Member C11	Protein Coding	36
TSNAXIP	Translin Associated Factor X Interacting Protein 1	Protein Coding	33
C12orf40	Chromosome 12 Open Reading Frame 40	Protein Coding	30
RCOR3	REST Corepressor 3	Protein Coding	35
MND1	Meiotic Nuclear Divisions 1	Protein Coding	37
PFDN4	Prefoldin Subunit 4	Protein Coding	39
MIR188	MicroRNA 188	RNA Gene	17
MTCH2	Mitochondrial Carrier 2	Protein Coding	41
AKNAD1	AKNA Domain Containing 1	Protein Coding	33
PNMA8A	PNMA Family Member 8A	Protein Coding	25
PDZD2	PDZ Domain Containing 2	Protein Coding	39
YTHDF2	YTH N6-Methyladenosine RNA Binding Protein 2	Protein Coding	39
FAM200B	Family With Sequence Similarity 200 Member B	Protein Coding	28
DUX3	Double Homeobox 3	Protein Coding	17
BZW1	Basic Leucine Zipper And W2 Domains 1	Protein Coding	36
TOB1	Transducer Of ERBB2, 1	Protein Coding	41
ABLIM3	Actin Binding LIM Protein Family Member 3	Protein Coding	37
TMX2	Thioredoxin Related Transmembrane Protein 2	Protein Coding	37
HAUS6	HAUS Augmin Like Complex Subunit 6	Protein Coding	36
KPRP	Keratinocyte Proline Rich Protein	Protein Coding	31
LACTB2	Lactamase Beta 2	Protein Coding	37
GPN2	GPN-Loop GTPase 2	Protein Coding	33
RABGEF1	RAB Guanine Nucleotide Exchange Factor 1	Protein Coding	44
GPATCH1	G-Patch Domain Containing 1	Protein Coding	33
HOXC6	Homeobox C6	Protein Coding	39
PLEKHO2	Pleckstrin Homology Domain Containing O2	Protein Coding	36
DNAJC8	DnaJ Heat Shock Protein Family (Hsp40) Member C8	Protein Coding	36
CHID1	Chitinase Domain Containing 1	Protein Coding	36
TSKS	Testis Specific Serine Kinase Substrate	Protein Coding	34
TSG1	Tumor Suppressor TSG1	RNA Gene	13

FGFBP2	Fibroblast Growth Factor Binding Protein 2	Protein Coding	35
DEF6	DEF6 Guanine Nucleotide Exchange Factor	Protein Coding	39
CORO1B	Coronin 1B	Protein Coding	37
LINC0005	Long Intergenic Non-Protein Coding RNA 51	RNA Gene	17
LINC0130	Long Intergenic Non-Protein Coding RNA 1307	RNA Gene	13
CDKL3	Cyclin Dependent Kinase Like 3	Protein Coding	40
ENY2	ENY2 Transcription And Export Complex 2 Subunit	Protein Coding	35
POLA2	DNA Polymerase Alpha 2, Accessory Subunit	Protein Coding	41
PLXNB2	Plexin B2	Protein Coding	40
PARVG	Parvin Gamma	Protein Coding	36
C1orf174	Chromosome 1 Open Reading Frame 174	Protein Coding	32
PATL1	PAT1 Homolog 1, Processing Body MRNA Decay Fact	Protein Coding	36
DCTN3	Dynactin Subunit 3	Protein Coding	37
MT1B	Metallothionein 1B	Protein Coding	36
CDC42EP	CDC42 Effector Protein 4	Protein Coding	37
TMEM183	Transmembrane Protein 183A	Protein Coding	32
TRIM69	Tripartite Motif Containing 69	Protein Coding	36
GIMAP8	GTPase, IMAP Family Member 8	Protein Coding	35
PTP4A2	Protein Tyrosine Phosphatase 4A2	Protein Coding	43
FOXO3B	Forkhead Box O3B	Protein Coding	17
SNORA48	Small Nucleolar RNA, H/ACA Box 48	RNA Gene	16
SNORD10	Small Nucleolar RNA, C/D Box 10	RNA Gene	14
RPARP-A	RPARP Antisense RNA 1	RNA Gene	13
ASH1L-A	ASH1L Antisense RNA 1	RNA Gene	13
KLHL7-D	KLHL7 Divergent Transcript	RNA Gene	12
HSD17B1	Hydroxysteroid 17-Beta Dehydrogenase 1 Pseudogene 1	Pseudogene	9
ENSG00000225342		RNA Gene	9
ENSG00000233223		Uncategorized	8
ENSG00000255046		RNA Gene	8
ENSG00000226816		RNA Gene	8
ENSG00000265749		RNA Gene	8
RNU6-100	RNA, U6 Small Nuclear 1000, Pseudogene	Pseudogene	7
HMGN2P1	High Mobility Group Nucleosomal Binding Domain 2 P	Pseudogene	6
ENSG00000220506		Pseudogene	6
ENSG00000226134		RNA Gene	6
ENSG00000272356		RNA Gene	6
piR-38051-158		RNA Gene	5
RF00994-1086		RNA Gene	5
lnc-IGF2BP3-1		RNA Gene	5
lnc-CDC42BPA-5		RNA Gene	5
lnc-LTK-1		RNA Gene	5
ENSG00000265380		RNA Gene	5
lnc-UTP23-10		RNA Gene	4
LOC10537	Uncharacterized LOC105373117	RNA Gene	3
ENSG00000248188		Pseudogene	2
FSIP1	Fibrous Sheath Interacting Protein 1	Protein Coding	34
GNG5	G Protein Subunit Gamma 5	Protein Coding	42

LONRF3	LON Peptidase N-Terminal Domain And Ring Finger 3	Protein Coding	35
POGLUT3	Protein O-Glucosyltransferase 3	Protein Coding	27
STK17B	Serine/Threonine Kinase 17b	Protein Coding	44
USE1	Unconventional SNARE In The ER 1	Protein Coding	37
ELK4	ETS Transcription Factor ELK4	Protein Coding	37
TSPAN14	Tetraspanin 14	Protein Coding	36
TCEA2	Transcription Elongation Factor A2	Protein Coding	37
ANKS4B	Ankyrin Repeat And Sterile Alpha Motif Domain Conta	Protein Coding	32
CCNB3	Cyclin B3	Protein Coding	37
USP6NL	USP6 N-Terminal Like	Protein Coding	40
LRRC46	Leucine Rich Repeat Containing 46	Protein Coding	33
MBD3	Methyl-CpG Binding Domain Protein 3	Protein Coding	43
BPHL	Biphenyl Hydrolase Like	Protein Coding	41
USP12	Ubiquitin Specific Peptidase 12	Protein Coding	37
CUL9	Cullin 9	Protein Coding	39
CCZ1	CCZ1 Homolog, Vacuolar Protein Trafficking And Bio	Protein Coding	32
SMURF2	SMAD Specific E3 Ubiquitin Protein Ligase 2	Protein Coding	45
DIRAS1	DIRAS Family GTPase 1	Protein Coding	37
FAM184A	Family With Sequence Similarity 184 Member A	Protein Coding	36
PDIA5	Protein Disulfide Isomerase Family A Member 5	Protein Coding	40
RHBDL2	Rhomboid Like 2	Protein Coding	38
SUDS3	SDS3 Homolog, SIN3A Corepressor Complex Compon	Protein Coding	35
MLLT6	MLLT6, PHD Finger Containing	Protein Coding	35
SCEL	Sciellin	Protein Coding	36
ZCCHC12	Zinc Finger CCHC-Type Containing 12	Protein Coding	34
ENKD1	Enkurin Domain Containing 1	Protein Coding	33
ARHGAP1	Rho GTPase Activating Protein 17	Protein Coding	41
MIR4306	MicroRNA 4306	RNA Gene	13
SELENON	Selenoprotein M	Protein Coding	28
MYL5	Myosin Light Chain 5	Protein Coding	37
ZC3H15	Zinc Finger CCCH-Type Containing 15	Protein Coding	32
SLC35B1	Solute Carrier Family 35 Member B1	Protein Coding	39
SIVA1	SIVA1 Apoptosis Inducing Factor	Protein Coding	37
MT1H	Metallothionein 1H	Protein Coding	37
IFRD2	Interferon Related Developmental Regulator 2	Protein Coding	37
GRIPAP1	GRIP1 Associated Protein 1	Protein Coding	36
CALCOC(	Calcium Binding And Coiled-Coil Domain 1	Protein Coding	37
HCFC1R1	Host Cell Factor C1 Regulator 1	Protein Coding	31
FAHD1	Fumarylacetoacetate Hydrolase Domain Containing 1	Protein Coding	39
MTRES1	Mitochondrial Transcription Rescue Factor 1	Protein Coding	24
UTP23	UTP23 Small Subunit Processome Component	Protein Coding	35
PARD6B	Par-6 Family Cell Polarity Regulator Beta	Protein Coding	39
CA14	Carbonic Anhydrase 14	Protein Coding	45
ENSG00000288520		Protein Coding	7
TBC1D10(	TBC1 Domain Family Member 10C	Protein Coding	37
CAPSL	Calcyphosine Like	Protein Coding	35
L3MBTL4	L3MBTL Histone Methyl-Lysine Binding Protein 4	Protein Coding	32

CLIC3	Chloride Intracellular Channel 3	Protein Coding	40
FRMD8	FERM Domain Containing 8	Protein Coding	34
C10orf88	Chromosome 10 Open Reading Frame 88	Protein Coding	34
TAFA4	TAFA Chemokine Like Family Member 4	Protein Coding	28
MZT2A	Mitotic Spindle Organizing Protein 2A	Protein Coding	33
TIMM17B	Translocase Of Inner Mitochondrial Membrane 17B	Protein Coding	38
NME6	NME/NM23 Nucleoside Diphosphate Kinase 6	Protein Coding	40
TSPAN13	Tetraspanin 13	Protein Coding	35
RILPL2	Rab Interacting Lysosomal Protein Like 2	Protein Coding	35
PHPT1	Phosphohistidine Phosphatase 1	Protein Coding	38
SCAANT1	SCA7/ATXN7 Antisense RNA 1	RNA Gene	12
KLK9	Kallikrein Related Peptidase 9	Protein Coding	33
RBM42	RNA Binding Motif Protein 42	Protein Coding	33
PDE1B	Phosphodiesterase 1B	Protein Coding	44
JTB	Jumping Translocation Breakpoint	Protein Coding	37
CHAC2	ChaC Cation Transport Regulator Homolog 2	Protein Coding	35
LARP6	La Ribonucleoprotein 6, Translational Regulator	Protein Coding	35
EIF2A	Eukaryotic Translation Initiation Factor 2A	Protein Coding	40
HSPA7	Heat Shock Protein Family A (Hsp70) Member 7	Pseudogene	22
C19orf57	Chromosome 19 Open Reading Frame 57	Protein Coding	33
CLIP4	CAP-Gly Domain Containing Linker Protein Family Me	Protein Coding	36
MVB12A	Multivesicular Body Subunit 12A	Protein Coding	31
DTWD1	DTW Domain Containing 1	Protein Coding	33
AGAP2	ArfGAP With GTPase Domain, Ankyrin Repeat And Pl	Protein Coding	43
SPZ1	Spermatogenic Leucine Zipper 1	Protein Coding	34
TP53TG5	TP53 Target 5	Protein Coding	34
TMEM53	Transmembrane Protein 53	Protein Coding	33
CHRAC1	Chromatin Accessibility Complex Subunit 1	Protein Coding	38
TRIM41	Tripartite Motif Containing 41	Protein Coding	36
NUFIP1	Nuclear FMR1 Interacting Protein 1	Protein Coding	35
MRPS24	Mitochondrial Ribosomal Protein S24	Protein Coding	37
C5orf15	Chromosome 5 Open Reading Frame 15	Protein Coding	33
TCP11	T-Complex 11	Protein Coding	37
C8orf58	Chromosome 8 Open Reading Frame 58	Protein Coding	28
LINC0032	Long Intergenic Non-Protein Coding RNA 324	RNA Gene	20
LINC0150	Long Intergenic Non-Protein Coding RNA 1500	RNA Gene	12
MKNK1	MAPK Interacting Serine/Threonine Kinase 1	Protein Coding	48
MRPS15	Mitochondrial Ribosomal Protein S15	Protein Coding	36
PARP3	Poly(ADP-Ribose) Polymerase Family Member 3	Protein Coding	41
COPS7B	COP9 Signalosome Subunit 7B	Protein Coding	37
NLN	Neurolysin	Protein Coding	40
GIMAP6	GTPase, IMAP Family Member 6	Protein Coding	37
MAST2	Microtubule Associated Serine/Threonine Kinase 2	Protein Coding	41
NECAB3	N-Terminal EF-Hand Calcium Binding Protein 3	Protein Coding	33
DSCR9	Down Syndrome Critical Region 9	RNA Gene	27
ACSM6	Acyl-CoA Synthetase Medium Chain Family Member 6	Protein Coding	31
APOOL	Apolipoprotein O Like	Protein Coding	35

CRYBG1	Crystallin Beta-Gamma Domain Containing 1	Protein Coding	28
CXXC4	CXXC Finger Protein 4	Protein Coding	39
SCCPDH	Saccharopine Dehydrogenase (Putative)	Protein Coding	36
RGS3	Regulator Of G Protein Signaling 3	Protein Coding	40
ZNF740	Zinc Finger Protein 740	Protein Coding	33
TAOK3	TAO Kinase 3	Protein Coding	41
TMEM168	Transmembrane Protein 168	Protein Coding	34
BTF3	Basic Transcription Factor 3	Protein Coding	41
METTL2B	Methyltransferase Like 2B	Protein Coding	33
AGPAT3	1-Acylglycerol-3-Phosphate O-Acyltransferase 3	Protein Coding	40
GUCD1	Guanylyl Cyclase Domain Containing 1	Protein Coding	33
NPIPB13	Nuclear Pore Complex Interacting Protein Family, Men	Protein Coding	16
ENSG00000255439		Protein Coding	9
CCNB1IP1	Cyclin B1 Interacting Protein 1	Protein Coding	38
CHCHD4	Coiled-Coil-Helix-Coiled-Coil-Helix Domain Containin	Protein Coding	36
NFYA	Nuclear Transcription Factor Y Subunit Alpha	Protein Coding	40
FN3KRP	Fructosamine 3 Kinase Related Protein	Protein Coding	39
EXOSC7	Exosome Component 7	Protein Coding	37
TTYH3	Tweety Family Member 3	Protein Coding	35
SETD3	SET Domain Containing 3, Actin Histidine Methyltrans	Protein Coding	37
TMEM267	Transmembrane Protein 267	Protein Coding	24
LRRC59	Leucine Rich Repeat Containing 59	Protein Coding	36
TMUB1	Transmembrane And Ubiquitin Like Domain Containing	Protein Coding	35
STX7	Syntaxin 7	Protein Coding	41
TCP11L1	T-Complex 11 Like 1	Protein Coding	32
RAB3B	RAB3B, Member RAS Oncogene Family	Protein Coding	37
ACOT9	Acyl-CoA Thioesterase 9	Protein Coding	38
RAB40B	RAB40B, Member RAS Oncogene Family	Protein Coding	37
SPATS2	Spermatogenesis Associated Serine Rich 2	Protein Coding	35
NXF2	Nuclear RNA Export Factor 2	Protein Coding	33
FRA6E	Fragile Site, Aphidicolin Type, Common, Fra(6)(Q26)	Uncategorized	5
TAAR1	Trace Amine Associated Receptor 1	Protein Coding	37
LINC0100	Long Intergenic Non-Protein Coding RNA 1006	RNA Gene	21
STRBP	Spermatid Perinuclear RNA Binding Protein	Protein Coding	36
UBE2R2	Ubiquitin Conjugating Enzyme E2 R2	Protein Coding	44
SSR1	Signal Sequence Receptor Subunit 1	Protein Coding	41
PLK3	Polo Like Kinase 3	Protein Coding	44
PRPF4B	Pre-MRNA Processing Factor 4B	Protein Coding	41
ARHGAP1	Rho GTPase Activating Protein 19	Protein Coding	36
ZFYVE1	Zinc Finger FYVE-Type Containing 1	Protein Coding	37
CLEC19A	C-Type Lectin Domain Containing 19A	Protein Coding	25
LINC0252	Long Intergenic Non-Protein Coding RNA 2527	RNA Gene	10
TRIM17	Tripartite Motif Containing 17	Protein Coding	38
CARF	Calcium Responsive Transcription Factor	Protein Coding	34
PIP4K2C	Phosphatidylinositol-5-Phosphate 4-Kinase Type 2 Gan	Protein Coding	39
XAF1	XIAP Associated Factor 1	Protein Coding	41
PCIF1	PDX1 C-Terminal Inhibiting Factor 1	Protein Coding	35

PCBP3	Poly(RC) Binding Protein 3	Protein Coding	36
GLB1L	Galactosidase Beta 1 Like	Protein Coding	36
PCDHGA1	Protocadherin Gamma Subfamily A, 12	Protein Coding	34
HNRNPCI	Heterogeneous Nuclear Ribonucleoprotein C Like 1	Protein Coding	30
FOXJ2	Forkhead Box J2	Protein Coding	36
FCHO1	FCH And Mu Domain Containing Endocytic Adaptor 1	Protein Coding	35
THAP11	THAP Domain Containing 11	Protein Coding	37
CHAF1B	Chromatin Assembly Factor 1 Subunit B	Protein Coding	39
ATPAF1	ATP Synthase Mitochondrial F1 Complex Assembly Fa	Protein Coding	38
ASCL3	Achaete-Scute Family BHLH Transcription Factor 3	Protein Coding	33
C16orf70	Chromosome 16 Open Reading Frame 70	Protein Coding	35
HIPK1	Homeodomain Interacting Protein Kinase 1	Protein Coding	41
IDH3G	Isocitrate Dehydrogenase (NAD(+)) 3 Non-Catalytic Su	Protein Coding	43
GCAT	Glycine C-Acetyltransferase	Protein Coding	43
PRDM16-I	PRDM16 Divergent Transcript	RNA Gene	17
MYL6	Myosin Light Chain 6	Protein Coding	42
ANKRD39	Ankyrin Repeat Domain 39	Protein Coding	33
TEPSIN	TEPSIN Adaptor Related Protein Complex 4 Accessory	Protein Coding	25
RSPH10B2	Radial Spoke Head 10 Homolog B2	Protein Coding	27
MRPL48	Mitochondrial Ribosomal Protein L48	Protein Coding	36
MGST3	Microsomal Glutathione S-Transferase 3	Protein Coding	44
SBNO1	Strawberry Notch Homolog 1	Protein Coding	36
ARGLU1	Arginine And Glutamate Rich 1	Protein Coding	36
RTL8A	Retrotransposon Gag Like 8A	Protein Coding	22
NT5DC3	5'-Nucleotidase Domain Containing 3	Protein Coding	34
AKAP11	A-Kinase Anchoring Protein 11	Protein Coding	38
C3orf20	Chromosome 3 Open Reading Frame 20	Protein Coding	33
RPUSD2	RNA Pseudouridine Synthase Domain Containing 2	Protein Coding	32
RASL12	RAS Like Family 12	Protein Coding	37
GRAMD4	GRAM Domain Containing 4	Protein Coding	36
PCDHA7	Protocadherin Alpha 7	Protein Coding	33
SAP30BP	SAP30 Binding Protein	Protein Coding	38
KIAA0930	KIAA0930	Protein Coding	35
PRR15	Proline Rich 15	Protein Coding	33
METTL8	Methyltransferase Like 8	Protein Coding	32
SCAF4	SR-Related CTD Associated Factor 4	Protein Coding	36
TRIAP1	TP53 Regulated Inhibitor Of Apoptosis 1	Protein Coding	36
CATIP	Ciliogenesis Associated TTC17 Interacting Protein	Protein Coding	30
PLA2G4C	Phospholipase A2 Group IVC	Protein Coding	44
NASP	Nuclear Autoantigenic Sperm Protein	Protein Coding	36
BHLHB9	Basic Helix-Loop-Helix Family Member B9	Protein Coding	34
GFRA3	GDNF Family Receptor Alpha 3	Protein Coding	42
ZNF646	Zinc Finger Protein 646	Protein Coding	35
ZNF396	Zinc Finger Protein 396	Protein Coding	31
SSC4D	Scavenger Receptor Cysteine Rich Family Member Wit	Protein Coding	28
LINC0047	Long Intergenic Non-Protein Coding RNA 479	RNA Gene	22
LINC0011	Long Intergenic Non-Protein Coding RNA 112	RNA Gene	16

ZSCAN16-	ZSCAN16 Antisense RNA 1	RNA Gene	13
GSTM2P1	Glutathione S-Transferase Mu 2 Pseudogene 1	Pseudogene	11
LINC0206	Long Intergenic Non-Protein Coding RNA 2067	RNA Gene	11
MIR4308	MicroRNA 4308	RNA Gene	10
RPL13P12	Ribosomal Protein L13 Pseudogene 12	Pseudogene	10
LINC0101	Long Intergenic Non-Protein Coding RNA 1012	RNA Gene	10
SIRLNT	SIRT1 Regulating LncRNA Tumor Promoter	RNA Gene	9
OR5AZ1P	Olfactory Receptor Family 5 Subfamily AZ Member 1 I	Pseudogene	9
ENSG00000237356		RNA Gene	9
LINC0245	Long Intergenic Non-Protein Coding RNA 2451	RNA Gene	9
ENSG00000234181		RNA Gene	8
ENSG00000253939		RNA Gene	8
ENSG00000254143		RNA Gene	8
ENSG00000243276		RNA Gene	8
PABPC1P	Poly(A) Binding Protein Cytoplasmic 1 Pseudogene 11	Pseudogene	8
ENSG00000250509		RNA Gene	8
GPR89P	G Protein-Coupled Receptor 89 Pseudogene	Pseudogene	8
ENSG00000257545		Uncategorized	8
TRD-GTC	TRNA-Asp (Anticodon GTC) 2-11	RNA Gene	8
ENSG00000260911		RNA Gene	8
TRP-CGG	TRNA-Pro (Anticodon CGG) 1-3	RNA Gene	8
ENSG00000260267		RNA Gene	8
ENSG00000260304		RNA Gene	8
TRW-CCA	TRNA-Trp (Anticodon CCA) 1-1	RNA Gene	8
TRG-TCC	TRNA-Gly (Anticodon TCC) 3-1	RNA Gene	8
ENSG00000259495		RNA Gene	8
ENSG00000236545		RNA Gene	8
ENSG00000271725		RNA Gene	8
ENSG00000267583		RNA Gene	8
PSMC1P7	Proteasome 26S Subunit, ATPase 1 Pseudogene 7	Pseudogene	8
ENSG00000251095		RNA Gene	7
ENSG00000253496		RNA Gene	7
ENSG00000255495		RNA Gene	7
EEF1GP4	Eukaryotic Translation Elongation Factor 1 Gamma Ps	Pseudogene	7
RNU1-138	RNA, U1 Small Nuclear 138, Pseudogene	Pseudogene	7
ENSG00000231064		RNA Gene	7
OR5BD1P	Olfactory Receptor Family 5 Subfamily BD Member 1	Pseudogene	7
ENSG00000223523		RNA Gene	7
ENSG00000201944		RNA Gene	7
ENSG00000258784		RNA Gene	7
LINC0233	Long Intergenic Non-Protein Coding RNA 2331	RNA Gene	7
LOC10798	Uncharacterized LOC107984875	RNA Gene	7
ENSG00000259675		RNA Gene	7
ENSG00000266929		RNA Gene	7
ENSG00000269947		RNA Gene	7
ENSG00000254153		RNA Gene	6
ENSG00000234580		RNA Gene	6

ENSG00000255020		Uncategorized	6
ENSG00000250075		Uncategorized	6
PSMA6P4	Proteasome Subunit Alpha 6 Pseudogene 4	Pseudogene	6
HNRNPCI	Heterogeneous Nuclear Ribonucleoprotein C Pseudogen	Pseudogene	6
COX6CP4	Cytochrome C Oxidase Subunit 6C Pseudogene 4	Pseudogene	6
RN7SL81	RNA, 7SL, Cytoplasmic 813, Pseudogene	Pseudogene	6
RPA2P1	Replication Protein A2 Pseudogene 1	Pseudogene	6
LOC10042	GABA Type A Receptor-Associated Protein Pseudogen	Pseudogene	6
ENSG00000228318		RNA Gene	6
FRG1CP	FSHD Region Gene 1 Family Member C, Pseudogene	Pseudogene	6
AKR1B1P	Aldo-Keto Reductase Family 1 Member B1 Pseudogene	Pseudogene	6
ENSG00000285521		RNA Gene	6
LOC3399	HCG1813818	RNA Gene	6
lnc-CHD9-4		RNA Gene	6
ENSG00000287401		RNA Gene	6
ENSG00000258853		RNA Gene	6
ENSG00000257435		RNA Gene	6
LINC0170	Long Intergenic Non-Protein Coding RNA 1709	RNA Gene	6
ENSG00000262372		RNA Gene	6
ENSG00000269899		RNA Gene	6
ENSG00000253505		RNA Gene	6
lnc-WNT3-1		RNA Gene	5
ENSG00000227809		Uncategorized	5
lnc-MFSD4A-1		RNA Gene	5
RAD1P1	RAD1 Pseudogene 1	Pseudogene	5
lnc-LMNB1-1		RNA Gene	5
lnc-CERS6-1		RNA Gene	5
CYP17A1-AS1-001		RNA Gene	5
lnc-WDHD1-3		RNA Gene	5
lnc-FAM3B-3		RNA Gene	5
HSALNG0007472		RNA Gene	5
piR-42491-008		RNA Gene	5
lnc-IPCEF1-6		RNA Gene	5
RPL9P22	Ribosomal Protein L9 Pseudogene 22	Pseudogene	5
NONHSAG022015.2		RNA Gene	5
NONHSAG045793.2		RNA Gene	5
NONHSAG038251.2		RNA Gene	5
NONHSAG010911.2		RNA Gene	5
HSALNG0081495		RNA Gene	5
HSALNG0081496		RNA Gene	5
lnc-KANSL1-4		RNA Gene	5
lnc-CTC1-2		RNA Gene	5
MG604298		RNA Gene	5
RF00017-4854		RNA Gene	5
lnc-RFX8-3		RNA Gene	5
lnc-BNC2-5		RNA Gene	5
lnc-C2CD2-3		RNA Gene	5

lnc-PRSS53-1	RNA Gene	5
lnc-BTNL2-2	RNA Gene	5
RF00017-6393	RNA Gene	5
RF00017-6395	RNA Gene	5
AB372576	RNA Gene	5
lnc-RAB29-1	RNA Gene	5
lnc-NMD3-2	RNA Gene	5
L13705-001	RNA Gene	5
ENSG00000236654	Pseudogene	5
ENSG00000273133	RNA Gene	5
ENSG00000273920	RNA Gene	5
lnc-ELOVL3-1	RNA Gene	4
piR-50444-343	RNA Gene	4
piR-36037-004	RNA Gene	4
piR-45438	RNA Gene	4
piR-37170-008	RNA Gene	4
piR-37106	RNA Gene	4
ENSG00000253790	Pseudogene	4
piR-38351-289	RNA Gene	4
ENSG00000234981	Pseudogene	4
lnc-DEFB136-3	RNA Gene	4
piR-34930	RNA Gene	4
lnc-ESD-2	RNA Gene	4
piR-59109	RNA Gene	4
lnc-EGR3-4	RNA Gene	4
piR-51036	RNA Gene	4
DQ866751	RNA Gene	4
piR-43401	RNA Gene	4
lnc-TOM1L2-7	RNA Gene	4
piR-59412-001	RNA Gene	4
piR-43106-068	RNA Gene	4
piR-43134	RNA Gene	4
piR-54316	RNA Gene	4
RF00994-103	RNA Gene	4
piR-35674-050	RNA Gene	4
lnc-FDFT1-1	RNA Gene	4
lnc-WISP3-8	RNA Gene	4
ENSG00000214846	Pseudogene	4
HSALNG0064740	RNA Gene	4
NONHSAG040481.2	RNA Gene	4
piR-36441-001	RNA Gene	4
piR-60985-091	RNA Gene	4
ENSG00000205485	Pseudogene	4
NONHSAG017121.2	RNA Gene	4
piR-37213	RNA Gene	4
lnc-CPLX1-12	RNA Gene	4
piR-50308-469	RNA Gene	4

piR-57845-019		RNA Gene	4
piR-57801-025		RNA Gene	4
RF00017-7528		RNA Gene	4
ENSG00000286553		RNA Gene	4
piR-32246		RNA Gene	4
piR-32250		RNA Gene	4
lnc-ARL3-2		RNA Gene	4
lnc-CCAR2-5		RNA Gene	4
lnc-BST1-1		RNA Gene	4
lnc-RPS27L-8		RNA Gene	4
piR-57133-264		RNA Gene	4
piR-57133-539		RNA Gene	4
AB330774-009		RNA Gene	4
RF00017-362		RNA Gene	4
piR-32606		RNA Gene	4
piR-48950-167		RNA Gene	4
lnc-C12orf40-2		RNA Gene	4
LOC39061	Ribosomal Protein L7 Like 1 Pseudogene	Pseudogene	4
lnc-NOS1AP-2		RNA Gene	4
piR-31199-012		RNA Gene	4
L13713-165		RNA Gene	4
ENSG00000275512		RNA Gene	4
ENSG00000260674		Pseudogene	4
RF00017-2418		RNA Gene	4
piR-42219		RNA Gene	4
hsa-miR-5095-131		RNA Gene	3
RF02541-018		RNA Gene	3
LOC1053(	Uncharacterized LOC105369180	RNA Gene	3
lnc-CCDC73-1		RNA Gene	3
piR-61157-011		RNA Gene	3
ENSG00000213153		Pseudogene	3
piR-50357-077		RNA Gene	3
MG828668-125		RNA Gene	3
ENSG00000287962		RNA Gene	3
piR-55809-038		RNA Gene	3
ENSG00000278153		RNA Gene	3
LOC10537	Uncharacterized LOC105371265	RNA Gene	3
piR-61945-390		RNA Gene	3
LOC10537	Uncharacterized LOC105371702	RNA Gene	3
LOC10537	Uncharacterized LOC105374341	RNA Gene	2
piR-37895-114		RNA Gene	2
LOC10537	Uncharacterized LOC105378347	RNA Gene	2
ENSG00000223368		Pseudogene	2
piR-32214-089		RNA Gene	2
RF00017-4334		RNA Gene	2
ENSG00000259208		Pseudogene	2
DAP	Death Associated Protein	Protein Coding	39

HLA-V	Major Histocompatibility Complex, Class I, V (Pseudog	Pseudogene	12
PRELID3A	PRELI Domain Containing 3A	Protein Coding	28
METTL25	Methyltransferase Like 25	Protein Coding	29
RPL41	Ribosomal Protein L41	Protein Coding	29
RAB15	RAB15, Member RAS Oncogene Family	Protein Coding	36
MXRA7	Matrix Remodeling Associated 7	Protein Coding	33
POTEE	POTE Ankyrin Domain Family Member E	Protein Coding	30
ATP5ME	ATP Synthase Membrane Subunit E	Protein Coding	30
ANXA10	Annexin A10	Protein Coding	39
ACP6	Acid Phosphatase 6, Lysophosphatidic	Protein Coding	41
ING2	Inhibitor Of Growth Family Member 2	Protein Coding	37
ACBD4	Acyl-CoA Binding Domain Containing 4	Protein Coding	37
C1orf131	Chromosome 1 Open Reading Frame 131	Protein Coding	31
TASP1	Taspase 1	Protein Coding	36
NAA80	N-Alpha-Acetyltransferase 80, NatH Catalytic Subunit	Protein Coding	27
DHRS4	Dehydrogenase/Reductase 4	Protein Coding	45
PDZD9	PDZ Domain Containing 9	Protein Coding	31
MRGPRE	MAS Related GPR Family Member E	Protein Coding	30
SERP1	Stress Associated Endoplasmic Reticulum Protein 1	Protein Coding	33
SLC26A4-	SLC26A4 Antisense RNA 1	RNA Gene	14
PLBD1	Phospholipase B Domain Containing 1	Protein Coding	36
TCEANC	Transcription Elongation Factor A N-Terminal And Ce	Protein Coding	29
DRAM1	DNA Damage Regulated Autophagy Modulator 1	Protein Coding	33
TMEM186	Transmembrane Protein 186	Protein Coding	33
UBE2V2	Ubiquitin Conjugating Enzyme E2 V2	Protein Coding	43
AVPI1	Arginine Vasopressin Induced 1	Protein Coding	36
MRPL34	Mitochondrial Ribosomal Protein L34	Protein Coding	35
H2AC1	H2A Clustered Histone 1	Protein Coding	29
GMFB	Glia Maturation Factor Beta	Protein Coding	41
TRIM40	Tripartite Motif Containing 40	Protein Coding	33
CCIN	Calicin	Protein Coding	35
TRMT1L	TRNA Methyltransferase 1 Like	Protein Coding	32
ADPGK	ADP Dependent Glucokinase	Protein Coding	43
FAM131C	Family With Sequence Similarity 131 Member C	Protein Coding	31
NMRK2	Nicotinamide Riboside Kinase 2	Protein Coding	38
TRIR	Telomerase RNA Component Interacting RNase	Protein Coding	25
DNAJC4	DnaJ Heat Shock Protein Family (Hsp40) Member C4	Protein Coding	32
KIAA0825	KIAA0825	Protein Coding	34
IGSF10	Immunoglobulin Superfamily Member 10	Protein Coding	37
PTBP3	Polypyrimidine Tract Binding Protein 3	Protein Coding	36
EXOSC4	Exosome Component 4	Protein Coding	37
CAVIN4	Caveolae Associated Protein 4	Protein Coding	27
ERGIC3	ERGIC And Golgi 3	Protein Coding	37
ARPP19	CAMP Regulated Phosphoprotein 19	Protein Coding	37
CAPZA2	Capping Actin Protein Of Muscle Z-Line Subunit Alpha	Protein Coding	41
LMCD1	LIM And Cysteine Rich Domains 1	Protein Coding	40
TRIM16	Tripartite Motif Containing 16	Protein Coding	34

METTL21	Methyltransferase Like 21A	Protein Coding	37
LYRM2	LYR Motif Containing 2	Protein Coding	35
FAHD2A	Fumarylacetoacetate Hydrolase Domain Containing 2A	Protein Coding	35
SYCP2L	Synaptonemal Complex Protein 2 Like	Protein Coding	33
PLA2G4D	Phospholipase A2 Group IVD	Protein Coding	40
SH3BGRL	SH3 Domain Binding Glutamate Rich Protein Like 3	Protein Coding	37
EIF3K	Eukaryotic Translation Initiation Factor 3 Subunit K	Protein Coding	39
ZSCAN5A	Zinc Finger And SCAN Domain Containing 5A	Protein Coding	33
ODF4	Outer Dense Fiber Of Sperm Tails 4	Protein Coding	31
EMC7	ER Membrane Protein Complex Subunit 7	Protein Coding	31
EMC10	ER Membrane Protein Complex Subunit 10	Protein Coding	32
RETREG2	Reticulophagy Regulator Family Member 2	Protein Coding	24
MIR545	MicroRNA 545	RNA Gene	14
NECAP2	NECAP Endocytosis Associated 2	Protein Coding	36
PELI3	Pellino E3 Ubiquitin Protein Ligase Family Member 3	Protein Coding	36
BTG2	BTG Anti-Proliferation Factor 2	Protein Coding	39
SEC11C	SEC11 Homolog C, Signal Peptidase Complex Subunit	Protein Coding	36
CRIP2	Cysteine Rich Protein 2	Protein Coding	37
AGO4	Argonaute RISC Component 4	Protein Coding	36
YPEL3	Yippee Like 3	Protein Coding	36
SNRNP35	Small Nuclear Ribonucleoprotein U11/U12 Subunit 35	Protein Coding	32
PASD1	PAS Domain Containing Repressor 1	Protein Coding	31
ISOC2	Isochorismatase Domain Containing 2	Protein Coding	35
KRTAP10	Keratin Associated Protein 10-7	Protein Coding	26
ARMCX3	Armadillo Repeat Containing X-Linked 3	Protein Coding	35
TAF1B	TATA-Box Binding Protein Associated Factor, RNA P	Protein Coding	35
GRPEL1	GrpE Like 1, Mitochondrial	Protein Coding	39
ACBD6	Acyl-CoA Binding Domain Containing 6	Protein Coding	37
DDX55	DEAD-Box Helicase 55	Protein Coding	37
ALDH8A1	Aldehyde Dehydrogenase 8 Family Member A1	Protein Coding	37
PHYHD1	Phytanoyl-CoA Dioxygenase Domain Containing 1	Protein Coding	32
EEPD1	Endonuclease/Exonuclease/Phosphatase Family Domain	Protein Coding	34
TDRD12	Tudor Domain Containing 12	Protein Coding	28
MIS18A	MIS18 Kinetochore Protein A	Protein Coding	33
EEF1A1P1	Eukaryotic Translation Elongation Factor 1 Alpha 1 Ps	Pseudogene	8
LANCL2	LanC Like 2	Protein Coding	36
FAM32A	Family With Sequence Similarity 32 Member A	Protein Coding	35
PPP6R2	Protein Phosphatase 6 Regulatory Subunit 2	Protein Coding	35
ZSCAN16	Zinc Finger And SCAN Domain Containing 16	Protein Coding	33
PSME3IP1	Proteasome Activator Subunit 3 Interacting Protein 1	Protein Coding	28
SEC24A	SEC24 Homolog A, COPII Coat Complex Component	Protein Coding	39
MRPL53	Mitochondrial Ribosomal Protein L53	Protein Coding	33
PEF1	Penta-EF-Hand Domain Containing 1	Protein Coding	39
CLEC2B	C-Type Lectin Domain Family 2 Member B	Protein Coding	38
USP47	Ubiquitin Specific Peptidase 47	Protein Coding	39
RASL10B	RAS Like Family 10 Member B	Protein Coding	34
CYB561D	Cytochrome B561 Family Member D2	Protein Coding	34

HMGCLL	3-Hydroxymethyl-3-Methylglutaryl-CoA Lyase Like 1	Protein Coding	38
IQCH	IQ Motif Containing H	Protein Coding	33
BEND5	BEN Domain Containing 5	Protein Coding	34
ARMC7	Armadillo Repeat Containing 7	Protein Coding	32
MT4	Metallothionein 4	Protein Coding	33
VBP1	VHL Binding Protein 1	Protein Coding	39
FBXW8	F-Box And WD Repeat Domain Containing 8	Protein Coding	36
ESF1	ESF1 Nucleolar Pre-RRNA Processing Protein Homolo	Protein Coding	33
PPFIBP1	PPFIA Binding Protein 1	Protein Coding	41
COA4	Cytochrome C Oxidase Assembly Factor 4 Homolog	Protein Coding	34
STK33	Serine/Threonine Kinase 33	Protein Coding	40
NKIRAS1	NFKB Inhibitor Interacting Ras Like 1	Protein Coding	36
CDC42EP	CDC42 Effector Protein 2	Protein Coding	37
BUD31	BUD31 Homolog	Protein Coding	39
RWDD2B	RWD Domain Containing 2B	Protein Coding	36
TBC1D9B	TBC1 Domain Family Member 9B	Protein Coding	33
LMNTD1	Lamin Tail Domain Containing 1	Protein Coding	31
CNN2	Calponin 2	Protein Coding	40
AFG3L1P	AFG3 Like Matrix AAA Peptidase Subunit 1, Pseudoge	Pseudogene	18
ZNF720	Zinc Finger Protein 720	Protein Coding	30
HIF3A	Hypoxia Inducible Factor 3 Subunit Alpha	Protein Coding	40
WWC3	WWC Family Member 3	Protein Coding	34
PTPRB	Protein Tyrosine Phosphatase Receptor Type B	Protein Coding	45
CENPT	Centromere Protein T	Protein Coding	37
EPB41L5	Erythrocyte Membrane Protein Band 4.1 Like 5	Protein Coding	37
DHRS7B	Dehydrogenase/Reductase 7B	Protein Coding	35
SASH3	SAM And SH3 Domain Containing 3	Protein Coding	35
DLK2	Delta Like Non-Canonical Notch Ligand 2	Protein Coding	34
RNF183	Ring Finger Protein 183	Protein Coding	33
NOL12	Nucleolar Protein 12	Protein Coding	32
CCDC15	Coiled-Coil Domain Containing 15	Protein Coding	32
ALKBH7	AlkB Homolog 7	Protein Coding	30
ARPC5	Actin Related Protein 2/3 Complex Subunit 5	Protein Coding	41
C1RL	Complement C1r Subcomponent Like	Protein Coding	38
CCZ1B	CCZ1 Homolog B, Vacuolar Protein Trafficking And B	Protein Coding	30
PAIP2	Poly(A) Binding Protein Interacting Protein 2	Protein Coding	39
FAM50A	Family With Sequence Similarity 50 Member A	Protein Coding	35
LLPH	LLP Homolog, Long-Term Synaptic Facilitation Factor	Protein Coding	32
ZNF839	Zinc Finger Protein 839	Protein Coding	35
ACOT13	Acyl-CoA Thioesterase 13	Protein Coding	39
SRSF8	Serine And Arginine Rich Splicing Factor 8	Protein Coding	30
GCHFR	GTP Cyclohydrolase I Feedback Regulator	Protein Coding	39
ADAT2	Adenosine Deaminase TRNA Specific 2	Protein Coding	34
PHF20L1	PHD Finger Protein 20 Like 1	Protein Coding	34
C9orf43	Chromosome 9 Open Reading Frame 43	Protein Coding	33
WDR89	WD Repeat Domain 89	Protein Coding	33
LRRC42	Leucine Rich Repeat Containing 42	Protein Coding	31

ZC2HC1A	Zinc Finger C2HC-Type Containing 1A	Protein Coding	31
ZBED3	Zinc Finger BED-Type Containing 3	Protein Coding	32
CEP162	Centrosomal Protein 162	Protein Coding	32
NPTN	Neuroplastin	Protein Coding	40
MON1B	MON1 Homolog B, Secretory Trafficking Associated	Protein Coding	36
TSSK6	Testis Specific Serine Kinase 6	Protein Coding	37
CSNK1G3	Casein Kinase 1 Gamma 3	Protein Coding	42
TTC1	Tetratricopeptide Repeat Domain 1	Protein Coding	37
EIF1	Eukaryotic Translation Initiation Factor 1	Protein Coding	38
MED11	Mediator Complex Subunit 11	Protein Coding	31
PCMTD2	Protein-L-Isoaspartate (D-Aspartate) O-Methyltransfera	Protein Coding	33
PLEKHJ1	Pleckstrin Homology Domain Containing J1	Protein Coding	36
TCP11L2	T-Complex 11 Like 2	Protein Coding	35
SNX21	Sorting Nexin Family Member 21	Protein Coding	33
HINT3	Histidine Triad Nucleotide Binding Protein 3	Protein Coding	33
C3orf33	Chromosome 3 Open Reading Frame 33	Protein Coding	32
TMA16	Translation Machinery Associated 16 Homolog	Protein Coding	31
SPANXN3	SPANX Family Member N3	Protein Coding	27
RTL8C	Retrotransposon Gag Like 8C	Protein Coding	24
HEBP1	Heme Binding Protein 1	Protein Coding	36
PFKFB1	6-Phosphofructo-2-Kinase/Fructose-2,6-Biphosphatase	Protein Coding	44
RBPJL	Recombination Signal Binding Protein For Immunoglob	Protein Coding	37
ACOT1	Acyl-CoA Thioesterase 1	Protein Coding	38
TRIM52	Tripartite Motif Containing 52	Protein Coding	30
AKAP8	A-Kinase Anchoring Protein 8	Protein Coding	41
NIPSNAP:	Nipsnap Homolog 3A	Protein Coding	36
C8orf33	Chromosome 8 Open Reading Frame 33	Protein Coding	32
LARP1B	La Ribonucleoprotein 1B	Protein Coding	31
TBC1D17	TBC1 Domain Family Member 17	Protein Coding	36
PAFAH2	Platelet Activating Factor Acetylhydrolase 2	Protein Coding	39
LINC0110	Long Intergenic Non-Protein Coding RNA 1104	RNA Gene	15
CCDC106	Coiled-Coil Domain Containing 106	Protein Coding	34
COMMD2	COMM Domain Containing 2	Protein Coding	33
MIR595	MicroRNA 595	RNA Gene	16
AAMDC	Adipogenesis Associated Mth938 Domain Containing	Protein Coding	33
OSGIN1	Oxidative Stress Induced Growth Inhibitor 1	Protein Coding	38
SDF2	Stromal Cell Derived Factor 2	Protein Coding	39
IFNA16	Interferon Alpha 16	Protein Coding	32
BDH2	3-Hydroxybutyrate Dehydrogenase 2	Protein Coding	39
PRSS33	Serine Protease 33	Protein Coding	36
IMP4	IMP U3 Small Nucleolar Ribonucleoprotein 4	Protein Coding	35
C11orf54	Chromosome 11 Open Reading Frame 54	Protein Coding	33
KCTD18	Potassium Channel Tetramerization Domain Containing	Protein Coding	32
FAM90A1	Family With Sequence Similarity 90 Member A1	Protein Coding	32
NUP62CL	Nucleoporin 62 C-Terminal Like	Protein Coding	31
PROSER2	Proline And Serine Rich 2	Protein Coding	30
ARMCX2	Armadillo Repeat Containing X-Linked 2	Protein Coding	36

PFKFB2	6-Phosphofructo-2-Kinase/Fructose-2,6-Biphosphatase	Protein Coding	42
RBM7	RNA Binding Motif Protein 7	Protein Coding	37
OXSM	3-Oxoacyl-ACP Synthase, Mitochondrial	Protein Coding	38
HIGD2A	HIG1 Hypoxia Inducible Domain Family Member 2A	Protein Coding	35
GTPBP8	GTP Binding Protein 8 (Putative)	Protein Coding	34
C18orf21	Chromosome 18 Open Reading Frame 21	Protein Coding	30
SPINK6	Serine Peptidase Inhibitor Kazal Type 6	Protein Coding	33
ATP5MD	ATP Synthase Membrane Subunit DAPIT	Protein Coding	29
ZDHHC7	Zinc Finger DHHC-Type Palmitoyltransferase 7	Protein Coding	38
SLC41A3	Solute Carrier Family 41 Member 3	Protein Coding	36
ZSCAN18	Zinc Finger And SCAN Domain Containing 18	Protein Coding	35
DUSP21	Dual Specificity Phosphatase 21	Protein Coding	32
KIAA1143	KIAA1143	Protein Coding	31
C4orf46	Chromosome 4 Open Reading Frame 46	Protein Coding	31
DUSP12	Dual Specificity Phosphatase 12	Protein Coding	37
EMC4	ER Membrane Protein Complex Subunit 4	Protein Coding	32
TSSK2	Testis Specific Serine Kinase 2	Protein Coding	36
PARP10	Poly(ADP-Ribose) Polymerase Family Member 10	Protein Coding	37
JMJD8	Jumonji Domain Containing 8	Protein Coding	32
PPP1R32	Protein Phosphatase 1 Regulatory Subunit 32	Protein Coding	31
ZSCAN1	Zinc Finger And SCAN Domain Containing 1	Protein Coding	31
MAPK15	Mitogen-Activated Protein Kinase 15	Protein Coding	41
ZNF277	Zinc Finger Protein 277	Protein Coding	37
FILIP1	Filamin A Interacting Protein 1	Protein Coding	35
DSEL	Dermatan Sulfate Epimerase Like	Protein Coding	33
MTRF1	Mitochondrial Translation Release Factor 1	Protein Coding	37
USP42	Ubiquitin Specific Peptidase 42	Protein Coding	37
PCP4	Purkinje Cell Protein 4	Protein Coding	36
NIM1K	NIM1 Serine/Threonine Protein Kinase	Protein Coding	33
RAB2B	RAB2B, Member RAS Oncogene Family	Protein Coding	36
FAM160B	Family With Sequence Similarity 160 Member B2	Protein Coding	33
TMEM25	Transmembrane Protein 255A	Protein Coding	32
ZC3HAV1	Zinc Finger CCCH-Type Containing, Antiviral 1 Like	Protein Coding	32
NXPE3	Neurexophilin And PC-Esterase Domain Family Memb	Protein Coding	30
GAGE1	G Antigen 1	Protein Coding	30
C3orf36	Chromosome 3 Putative Open Reading Frame 36	RNA Gene	29
DLGAP1-	DLGAP1 Antisense RNA 2	RNA Gene	16
C1orf53	Chromosome 1 Open Reading Frame 53	Protein Coding	29
FUBP3	Far Upstream Element Binding Protein 3	Protein Coding	37
C7orf50	Chromosome 7 Open Reading Frame 50	Protein Coding	33
RFTN1	Raftlin, Lipid Raft Linker 1	Protein Coding	36
RAB5IF	RAB5 Interacting Factor	Protein Coding	25
ZNF75A	Zinc Finger Protein 75a	Protein Coding	36
KRT36	Keratin 36	Protein Coding	35
C7orf31	Chromosome 7 Open Reading Frame 31	Protein Coding	33
MYL12B	Myosin Light Chain 12B	Protein Coding	39
LYPLAL1	Lysophospholipase Like 1	Protein Coding	37

JHY	Junctional Cadherin Complex Regulator	Protein Coding	27
TMX4	Thioredoxin Related Transmembrane Protein 4	Protein Coding	37
RHBDD3	Rhomboid Domain Containing 3	Protein Coding	33
IQCD	IQ Motif Containing D	Protein Coding	32
UBL4B	Ubiquitin Like 4B	Protein Coding	31
FKBP9	FKBP Prolyl Isomerase 9	Protein Coding	36
MPPED1	Metallophosphoesterase Domain Containing 1	Protein Coding	34
HACD3	3-Hydroxyacyl-CoA Dehydratase 3	Protein Coding	35
PDZD4	PDZ Domain Containing 4	Protein Coding	35
DAPK2	Death Associated Protein Kinase 2	Protein Coding	43
ARHGEF1	Rho Guanine Nucleotide Exchange Factor 16	Protein Coding	39
SPIC	Spi-C Transcription Factor	Protein Coding	36
CD300LB	CD300 Molecule Like Family Member B	Protein Coding	39
RXFP3	Relaxin Family Peptide Receptor 3	Protein Coding	43
GLMP	Glycosylated Lysosomal Membrane Protein	Protein Coding	31
SPATA20	Spermatogenesis Associated 20	Protein Coding	34
KLHL1	Kelch Like Family Member 1	Protein Coding	39
FAM162A	Family With Sequence Similarity 162 Member A	Protein Coding	33
ZNF343	Zinc Finger Protein 343	Protein Coding	36
AMIGO2	Adhesion Molecule With Ig Like Domain 2	Protein Coding	39
ZNF410	Zinc Finger Protein 410	Protein Coding	35
CACNG4	Calcium Voltage-Gated Channel Auxiliary Subunit Gan	Protein Coding	41
DENND4I	DENN Domain Containing 4B	Protein Coding	32
RALGPS1	Ral GEF With PH Domain And SH3 Binding Motif 1	Protein Coding	37
LRRC4	Leucine Rich Repeat Containing 4	Protein Coding	39
LYPLA1	Lysophospholipase 1	Protein Coding	41
RHBDL3	Rhomboid Like 3	Protein Coding	36
DCUN1D2	Defective In Cullin Neddylation 1 Domain Containing 2	Protein Coding	35
MYRF-AS	MYRF Antisense RNA 1	RNA Gene	13
TXLNG	Taxilin Gamma	Protein Coding	33
QTRT1	Queuine TRNA-Ribosyltransferase Catalytic Subunit 1	Protein Coding	39
H2BC14	H2B Clustered Histone 14	Protein Coding	28
C1orf35	Chromosome 1 Open Reading Frame 35	Protein Coding	33
ADAD2	Adenosine Deaminase Domain Containing 2	Protein Coding	35
FMNL3	Formin Like 3	Protein Coding	37
SCAMP2	Secretory Carrier Membrane Protein 2	Protein Coding	38
INPP4B	Inositol Polyphosphate-4-Phosphatase Type II B	Protein Coding	40
HSD17B7	Hydroxysteroid 17-Beta Dehydrogenase 7	Protein Coding	43
HSP90AB	Heat Shock Protein 90 Alpha Family Class B Member 2	Pseudogene	18
GPR17	G Protein-Coupled Receptor 17	Protein Coding	41
UBL3	Ubiquitin Like 3	Protein Coding	37
STRN3	Striatin 3	Protein Coding	39
C14orf93	Chromosome 14 Open Reading Frame 93	Protein Coding	32
CMTM2	CKLF Like MARVEL Transmembrane Domain Contai	Protein Coding	36
SLC25A14	Solute Carrier Family 25 Member 14	Protein Coding	41
ARL5A	ADP Ribosylation Factor Like GTPase 5A	Protein Coding	36
CCDC144	CCDC144A N-Terminal Pseudogene	Pseudogene	31

HSPA13	Heat Shock Protein Family A (Hsp70) Member 13	Protein Coding	37
THUMPD2	THUMP Domain Containing 2	Protein Coding	33
LETM2	Leucine Zipper And EF-Hand Containing Transmembra	Protein Coding	35
MEX3B	Mex-3 RNA Binding Family Member B	Protein Coding	38
NIPSNAP3	Nipsnap Homolog 3B	Protein Coding	35
NMRK1	Nicotinamide Riboside Kinase 1	Protein Coding	38
KLF7	Kruppel Like Factor 7	Protein Coding	41
TOMM5	Translocase Of Outer Mitochondrial Membrane 5	Protein Coding	34
STOML3	Stomatin Like 3	Protein Coding	36
PDILT	Protein Disulfide Isomerase Like, Testis Expressed	Protein Coding	31
WDR5B	WD Repeat Domain 5B	Protein Coding	33
HELB	DNA Helicase B	Protein Coding	35
ART4	ADP-Ribosyltransferase 4 (Dombrock Blood Group)	Protein Coding	40
FAM53A	Family With Sequence Similarity 53 Member A	Protein Coding	33
FAM126B	Family With Sequence Similarity 126 Member B	Protein Coding	34
UGGT1	UDP-Glucose Glycoprotein Glucosyltransferase 1	Protein Coding	39
CFAP58	Cilia And Flagella Associated Protein 58	Protein Coding	30
RUSC1	RUN And SH3 Domain Containing 1	Protein Coding	36
VLDLR-A	VLDLR Antisense RNA 1	RNA Gene	14
RAPGEFL	Rap Guanine Nucleotide Exchange Factor Like 1	Protein Coding	34
TMA7	Translation Machinery Associated 7 Homolog	Protein Coding	29
EMC2	ER Membrane Protein Complex Subunit 2	Protein Coding	33
C11orf45	Chromosome 11 Open Reading Frame 45	Protein Coding	29
EML3	EMAP Like 3	Protein Coding	35
TMEM205	Transmembrane Protein 205	Protein Coding	32
SLC29A4	Solute Carrier Family 29 Member 4	Protein Coding	43
SLC8B1	Solute Carrier Family 8 Member B1	Protein Coding	39
REXO2	RNA Exonuclease 2	Protein Coding	38
EPB41L4A	Erythrocyte Membrane Protein Band 4.1 Like 4A	Protein Coding	35
GPR12	G Protein-Coupled Receptor 12	Protein Coding	39
FAM133B	Family With Sequence Similarity 133 Member B	Protein Coding	31
SP140L	SP140 Nuclear Body Protein Like	Protein Coding	35
CC2D1B	Coiled-Coil And C2 Domain Containing 1B	Protein Coding	32
IFI6	Interferon Alpha Inducible Protein 6	Protein Coding	38
DMAC2	Distal Membrane Arm Assembly Complex 2	Protein Coding	26
KIAA0513	KIAA0513	Protein Coding	35
BEND2	BEN Domain Containing 2	Protein Coding	31
TPPP2	Tubulin Polymerization Promoting Protein Family Mem	Protein Coding	35
KLF8	Kruppel Like Factor 8	Protein Coding	38
STON2	Stonin 2	Protein Coding	35
RAB43	RAB43, Member RAS Oncogene Family	Protein Coding	35
TBC1D21	TBC1 Domain Family Member 21	Protein Coding	33
PLAAT1	Phospholipase A And Acyltransferase 1	Protein Coding	26
ZNF219	Zinc Finger Protein 219	Protein Coding	36
TMEM14C	Transmembrane Protein 14C	Protein Coding	37
FAM234A	Family With Sequence Similarity 234 Member A	Protein Coding	27
PLEKHF2	Pleckstrin Homology And FYVE Domain Containing 2	Protein Coding	37

MIR384	MicroRNA 384	RNA Gene	10
LINC0066	Long Intergenic Non-Protein Coding RNA 663	RNA Gene	18
FAM168B	Family With Sequence Similarity 168 Member B	Protein Coding	33
OCEL1	Occludin/ELL Domain Containing 1	Protein Coding	33
USP5	Ubiquitin Specific Peptidase 5	Protein Coding	42
FKBP2	FKBP Prolyl Isomerase 2	Protein Coding	41
ANAPC11	Anaphase Promoting Complex Subunit 11	Protein Coding	41
PTPN7	Protein Tyrosine Phosphatase Non-Receptor Type 7	Protein Coding	43
TMED7	Transmembrane P24 Trafficking Protein 7	Protein Coding	33
MTERF2	Mitochondrial Transcription Termination Factor 2	Protein Coding	28
LSMEM1	Leucine Rich Single-Pass Membrane Protein 1	Protein Coding	31
MFSD13A	Major Facilitator Superfamily Domain Containing 13A	Protein Coding	27
ERH	ERH MRNA Splicing And Mitosis Factor	Protein Coding	36
UPP1	Uridine Phosphorylase 1	Protein Coding	41
RGS18	Regulator Of G Protein Signaling 18	Protein Coding	38
AMY2B	Amylase Alpha 2B	Protein Coding	40
C22orf46	Chromosome 22 Open Reading Frame 46	Pseudogene	27
SCAND2P	SCAN Domain Containing 2 Pseudogene	Pseudogene	25
ATP1B3	ATPase Na+/K+ Transporting Subunit Beta 3	Protein Coding	43
KCNMB2	Potassium Calcium-Activated Channel Subfamily M Re	Protein Coding	36
FAM151A	Family With Sequence Similarity 151 Member A	Protein Coding	34
C10orf90	Chromosome 10 Open Reading Frame 90	Protein Coding	32
CMTM3	CKLF Like MARVEL Transmembrane Domain Contai	Protein Coding	36
REXO1	RNA Exonuclease 1 Homolog	Protein Coding	36
PCNP	PEST Proteolytic Signal Containing Nuclear Protein	Protein Coding	35
EBF4	EBF Family Member 4	Protein Coding	35
SRSF11	Serine And Arginine Rich Splicing Factor 11	Protein Coding	38
CLDN20	Claudin 20	Protein Coding	33
PDAP1	PDGFA Associated Protein 1	Protein Coding	36
FAM8A1	Family With Sequence Similarity 8 Member A1	Protein Coding	33
GCNA	Germ Cell Nuclear Acidic Peptidase	Protein Coding	25
DOCK10	Dedicator Of Cytokinesis 10	Protein Coding	39
LAMTOR	Late Endosomal/Lysosomal Adaptor, MAPK And MTC	Protein Coding	35
TPTEP2-C	TPTEP2-CSNK1E Readthrough	Protein Coding	14
LOC1111	Enhancer In Exon 7 Of CSF1R	Biological Region	2
PUDP	Pseudouridine 5'-Phosphatase	Protein Coding	33
PTTG2	Pituitary Tumor-Transforming 2	Protein Coding	31
FMO6P	Flavin Containing Dimethylaniline Monoxygenase 6, Ps	Pseudogene	24
GLOD4	Glyoxalase Domain Containing 4	Protein Coding	38
BAALC-A	BAALC Antisense RNA 2	RNA Gene	18
PARP15	Poly(ADP-Ribose) Polymerase Family Member 15	Protein Coding	36
UBE2DNL	Ubiquitin Conjugating Enzyme E2 D N-Terminal Like (	Pseudogene	18
ERDA1	Expanded Repeat Domain, CAG/CTG 1	Genetic Locus	7
TENM3-A	TENM3 Antisense RNA 1	RNA Gene	11
GPR61	G Protein-Coupled Receptor 61	Protein Coding	38
ERVFRD-	Endogenous Retrovirus Group FRD Member 1, Envelo	Protein Coding	32
UBL5	Ubiquitin Like 5	Protein Coding	37

HDHD3	Haloacid Dehalogenase Like Hydrolase Domain Contai	Protein Coding	36
FBXW9	F-Box And WD Repeat Domain Containing 9	Protein Coding	32
IL6-AS1	IL6 Antisense RNA 1	RNA Gene	10
CDK2AP2	Cyclin Dependent Kinase 2 Associated Protein 2	Protein Coding	34
RNASE11	Ribonuclease A Family Member 11 (Inactive)	Protein Coding	31
TMEM10	Transmembrane Protein 109	Protein Coding	33
EEF1A1P	Eukaryotic Translation Elongation Factor 1 Alpha 1 Ps	Pseudogene	15
KYAT3	Kynurenine Aminotransferase 3	Protein Coding	32
PCDHB10	Protocadherin Beta 10	Protein Coding	33
RESP18	Regulated Endocrine Specific Protein 18	Protein Coding	32
ZRANB1	Zinc Finger RANBP2-Type Containing 1	Protein Coding	36
TPD52L3	TPD52 Like 3	Protein Coding	33
C6orf201	Chromosome 6 Open Reading Frame 201	Protein Coding	31
ACSF2	Acyl-CoA Synthetase Family Member 2	Protein Coding	39
C1orf198	Chromosome 1 Open Reading Frame 198	Protein Coding	32
EVA1B	Eva-1 Homolog B	Protein Coding	30
C5orf51	Chromosome 5 Open Reading Frame 51	Protein Coding	31
GPR21	G Protein-Coupled Receptor 21	Protein Coding	36
CA7	Carbonic Anhydrase 7	Protein Coding	40
SGK2	Serum/Glucocorticoid Regulated Kinase 2	Protein Coding	41
HMGB4	High Mobility Group Box 4	Protein Coding	33
TARP	TCR Gamma Alternate Reading Frame Protein	Protein Coding	21
OSTCP1	Oligosaccharyltransferase Complex Subunit Pseudogen	Pseudogene	16
DUSP18	Dual Specificity Phosphatase 18	Protein Coding	37
OR2W1	Olfactory Receptor Family 2 Subfamily W Member 1	Protein Coding	34
C11orf71	Chromosome 11 Open Reading Frame 71	Protein Coding	29
R3HDML	R3H Domain Containing Like	Protein Coding	32
CALM1P1	Calmodulin 1 Pseudogene 1	Pseudogene	7
STK35	Serine/Threonine Kinase 35	Protein Coding	37
IGKV2-30	Immunoglobulin Kappa Variable 2-30	Protein Coding	14
MIR153-1	MicroRNA 153-1	RNA Gene	17
SPRR4	Small Proline Rich Protein 4	Protein Coding	27
CFAP299	Cilia And Flagella Associated Protein 299	Protein Coding	22
DGLUCY	D-Glutamate Cyclase	Protein Coding	27
GLIPR1L2	GLIPR1 Like 2	Protein Coding	32
OR13C4	Olfactory Receptor Family 13 Subfamily C Member 4	Protein Coding	29
GPR37L1	G Protein-Coupled Receptor 37 Like 1	Protein Coding	41
MIR4487	MicroRNA 4487	RNA Gene	13
FAAH2	Fatty Acid Amide Hydrolase 2	Protein Coding	37
RITA1	RBPJ Interacting And Tubulin Associated 1	Protein Coding	31
C11orf53	Chromosome 11 Open Reading Frame 53	Protein Coding	31
CCDC149	Coiled-Coil Domain Containing 149	Protein Coding	28
POTEKP	POTE Ankyrin Domain Family Member K, Pseudogene	Pseudogene	22

GC Id	Relevance sco	GeneCards Link
GC04M089724	226.81	https://www.genecards.org/cgi-bin/carddisp.pl?gene=SNCA
GC12P040196	216.41	https://www.genecards.org/cgi-bin/carddisp.pl?gene=LRRK2
GC06M161348	202.25	https://www.genecards.org/cgi-bin/carddisp.pl?gene=PRKN
GC01P007968	171.14	https://www.genecards.org/cgi-bin/carddisp.pl?gene=PARK7
GC01P020634	162.94	https://www.genecards.org/cgi-bin/carddisp.pl?gene=PINK1
GC17P045894	150.56	https://www.genecards.org/cgi-bin/carddisp.pl?gene=MAPT
GC01M155234	148.44	https://www.genecards.org/cgi-bin/carddisp.pl?gene=GBA
GC01M016985	145.66	https://www.genecards.org/cgi-bin/carddisp.pl?gene=ATP13A2
GC19P044906	132.91	https://www.genecards.org/cgi-bin/carddisp.pl?gene=APOE
GC05M001392	124.66	https://www.genecards.org/cgi-bin/carddisp.pl?gene=SLC6A3
GC21M025880	119.46	https://www.genecards.org/cgi-bin/carddisp.pl?gene=APP
GC04P041256	116.72	https://www.genecards.org/cgi-bin/carddisp.pl?gene=UCHL1
GC11M002163	113.85	https://www.genecards.org/cgi-bin/carddisp.pl?gene=TH
GC05P122311	111.02	https://www.genecards.org/cgi-bin/carddisp.pl?gene=SNCAIP
GC16M046678	107.07	https://www.genecards.org/cgi-bin/carddisp.pl?gene=VPS35
GC14P073136	106.91	https://www.genecards.org/cgi-bin/carddisp.pl?gene=PSEN1
GC20P004686	104.49	https://www.genecards.org/cgi-bin/carddisp.pl?gene=PRNP
GC21M032628	102.52	https://www.genecards.org/cgi-bin/carddisp.pl?gene=SYNJ1
GC06P033397	101.57	https://www.genecards.org/cgi-bin/carddisp.pl?gene=TNF
GC22M045345	101.03	https://www.genecards.org/cgi-bin/carddisp.pl?gene=PLA2G6
GC07P022765	99.81	https://www.genecards.org/cgi-bin/carddisp.pl?gene=IL6
GC11M027654	98.9	https://www.genecards.org/cgi-bin/carddisp.pl?gene=BDNF
GC21P031659	95.5	https://www.genecards.org/cgi-bin/carddisp.pl?gene=SOD1
GC02P074529	95.45	https://www.genecards.org/cgi-bin/carddisp.pl?gene=HTRA2
GC22P032474	93.43	https://www.genecards.org/cgi-bin/carddisp.pl?gene=FBXO7
GC07M151556	92.55	https://www.genecards.org/cgi-bin/carddisp.pl?gene=PRKAG2
GC22P019941	91.39	https://www.genecards.org/cgi-bin/carddisp.pl?gene=COMT
GC02P232698	89.24	https://www.genecards.org/cgi-bin/carddisp.pl?gene=GIGYF2
GC04P003041	88.25	https://www.genecards.org/cgi-bin/carddisp.pl?gene=HTT
GC09M035056	88.11	https://www.genecards.org/cgi-bin/carddisp.pl?gene=VCP
GC05M037812	85.45	https://www.genecards.org/cgi-bin/carddisp.pl?gene=GDNF
GC14M054842	85.07	https://www.genecards.org/cgi-bin/carddisp.pl?gene=GCH1
GC11M113409	84.69	https://www.genecards.org/cgi-bin/carddisp.pl?gene=DRD2
GC05P179806	83.57	https://www.genecards.org/cgi-bin/carddisp.pl?gene=SQSTM1
GC01M206767	83.4	https://www.genecards.org/cgi-bin/carddisp.pl?gene=IL10
GC08M024950	83.02	https://www.genecards.org/cgi-bin/carddisp.pl?gene=NEFL
GC15M089316	82.96	https://www.genecards.org/cgi-bin/carddisp.pl?gene=POLG
GC09M027539	81.93	https://www.genecards.org/cgi-bin/carddisp.pl?gene=C9orf72
GC12M111443	81.63	https://www.genecards.org/cgi-bin/carddisp.pl?gene=ATXN2
GC01P065248	80.71	https://www.genecards.org/cgi-bin/carddisp.pl?gene=DNAJC6
GC14M093534	79.96	https://www.genecards.org/cgi-bin/carddisp.pl?gene=ATXN3
GC17M044905	79.68	https://www.genecards.org/cgi-bin/carddisp.pl?gene=GFAP
GC11P034460	78.56	https://www.genecards.org/cgi-bin/carddisp.pl?gene=CAT

GC01P011013	77.4	https://www.genecards.org/cgi-bin/carddisp.pl?gene=TARDBP
GC10P117241	77.33	https://www.genecards.org/cgi-bin/carddisp.pl?gene=SLC18A2
GC14M104769	77.31	https://www.genecards.org/cgi-bin/carddisp.pl?gene=AKT1
GC01P011980	77.16	https://www.genecards.org/cgi-bin/carddisp.pl?gene=MFN2
GC02M156324	76.33	https://www.genecards.org/cgi-bin/carddisp.pl?gene=NR4A2
GC01P226870	75.48	https://www.genecards.org/cgi-bin/carddisp.pl?gene=PSEN2
GC17P063477	75.3	https://www.genecards.org/cgi-bin/carddisp.pl?gene=ACE
GC03P132417	75.26	https://www.genecards.org/cgi-bin/carddisp.pl?gene=DNAJC13
GC15M061852	74.75	https://www.genecards.org/cgi-bin/carddisp.pl?gene=VPS13C
GC17M007661	74.26	https://www.genecards.org/cgi-bin/carddisp.pl?gene=TP53
GC03P184314	73.64	https://www.genecards.org/cgi-bin/carddisp.pl?gene=EIF4G1
GC03M149162	73.4	https://www.genecards.org/cgi-bin/carddisp.pl?gene=CP
GC11M002159	72.8	https://www.genecards.org/cgi-bin/carddisp.pl?gene=INS
GC06P043770	72.38	https://www.genecards.org/cgi-bin/carddisp.pl?gene=VEGFA
GC10P049609	71.78	https://www.genecards.org/cgi-bin/carddisp.pl?gene=CHAT
GC12M068064	71.23	https://www.genecards.org/cgi-bin/carddisp.pl?gene=IFNG
GC05P053560	71.18	https://www.genecards.org/cgi-bin/carddisp.pl?gene=NDUFS4
GC05M176620	71.16	https://www.genecards.org/cgi-bin/carddisp.pl?gene=SNCB
GC22M044576	70.83	https://www.genecards.org/cgi-bin/carddisp.pl?gene=CYP2D6
GC02M112829	70.74	https://www.genecards.org/cgi-bin/carddisp.pl?gene=IL1B
GC01M115285	70.29	https://www.genecards.org/cgi-bin/carddisp.pl?gene=NGF
GC17M030194	70.17	https://www.genecards.org/cgi-bin/carddisp.pl?gene=SLC6A4
GCMTP003309	70.16	https://www.genecards.org/cgi-bin/carddisp.pl?gene=MT-ND1
GCMTP012339	70.07	https://www.genecards.org/cgi-bin/carddisp.pl?gene=MT-ND5
GC03M165772	69.77	https://www.genecards.org/cgi-bin/carddisp.pl?gene=BCHE
GC18P031557	69.31	https://www.genecards.org/cgi-bin/carddisp.pl?gene=TTR
GC14P104308	69.2	https://www.genecards.org/cgi-bin/carddisp.pl?gene=DYNC1H1
GC06M159669	69.16	https://www.genecards.org/cgi-bin/carddisp.pl?gene=SOD2
GC11M001752	68.89	https://www.genecards.org/cgi-bin/carddisp.pl?gene=CTSD
GC01M011106	68.85	https://www.genecards.org/cgi-bin/carddisp.pl?gene=MTOR
GC0XM155259	67.05	https://www.genecards.org/cgi-bin/carddisp.pl?gene=RAB39B
GC17P080101	67.03	https://www.genecards.org/cgi-bin/carddisp.pl?gene=GAA
GC0XP043654	66.91	https://www.genecards.org/cgi-bin/carddisp.pl?gene=MAOA
GC06M032578	66.88	https://www.genecards.org/cgi-bin/carddisp.pl?gene=HLA-DRB1
GC22P035380	66.87	https://www.genecards.org/cgi-bin/carddisp.pl?gene=HMOX1
GC09P117704	66.62	https://www.genecards.org/cgi-bin/carddisp.pl?gene=TLR4
GC13M051905	66.09	https://www.genecards.org/cgi-bin/carddisp.pl?gene=ATP7B
GC02M074361	65.81	https://www.genecards.org/cgi-bin/carddisp.pl?gene=DCTN1
GC03M114128	65.69	https://www.genecards.org/cgi-bin/carddisp.pl?gene=DRD3
GC17P044345	65.49	https://www.genecards.org/cgi-bin/carddisp.pl?gene=GRN
GC06P170554	65.46	https://www.genecards.org/cgi-bin/carddisp.pl?gene=TBP
GC13M046831	64.08	https://www.genecards.org/cgi-bin/carddisp.pl?gene=HTR2A
GC0XM043766	63.86	https://www.genecards.org/cgi-bin/carddisp.pl?gene=MAOB
GC07M056101	63.74	https://www.genecards.org/cgi-bin/carddisp.pl?gene=CHCHD2
GC07M050458	63.67	https://www.genecards.org/cgi-bin/carddisp.pl?gene=DDC
GC11P121452	63.61	https://www.genecards.org/cgi-bin/carddisp.pl?gene=SORL1
GC17M042185	63.2	https://www.genecards.org/cgi-bin/carddisp.pl?gene=HCRT

GC16P031418	63.09	https://www.genecards.org/cgi-bin/carddisp.pl?gene=FUS
GC17M002049	62.81	https://www.genecards.org/cgi-bin/carddisp.pl?gene=MIR132
GC02P201233	62.51	https://www.genecards.org/cgi-bin/carddisp.pl?gene=CASP8
GC10P043081	62.26	https://www.genecards.org/cgi-bin/carddisp.pl?gene=RET
GC09M129812	61.71	https://www.genecards.org/cgi-bin/carddisp.pl?gene=TOR1A
GC12M102395	61.19	https://www.genecards.org/cgi-bin/carddisp.pl?gene=IGF1
GC17P059841	60.56	https://www.genecards.org/cgi-bin/carddisp.pl?gene=MIR21
GC07M095297	60.49	https://www.genecards.org/cgi-bin/carddisp.pl?gene=PON1
GC08M016992	60.45	https://www.genecards.org/cgi-bin/carddisp.pl?gene=FGF20
GC11P000669	60.09	https://www.genecards.org/cgi-bin/carddisp.pl?gene=DRD4
GC01P156082	59.6	https://www.genecards.org/cgi-bin/carddisp.pl?gene=LMNA
GC03P128737	59.53	https://www.genecards.org/cgi-bin/carddisp.pl?gene=RAB7A
GC17P034255	58.66	https://www.genecards.org/cgi-bin/carddisp.pl?gene=CCL2
GC12M013437	58.62	https://www.genecards.org/cgi-bin/carddisp.pl?gene=GRIN2B
GC19P048954	58.26	https://www.genecards.org/cgi-bin/carddisp.pl?gene=BAX
GC04M083138	58.12	https://www.genecards.org/cgi-bin/carddisp.pl?gene=COQ2
GC04P153684	57.5	https://www.genecards.org/cgi-bin/carddisp.pl?gene=TLR2
GC22M018912	57.5	https://www.genecards.org/cgi-bin/carddisp.pl?gene=PRODH
GC01M161304	57.01	https://www.genecards.org/cgi-bin/carddisp.pl?gene=MPZ
GC01M159715	56.96	https://www.genecards.org/cgi-bin/carddisp.pl?gene=CRP
GC14P094612	56.88	https://www.genecards.org/cgi-bin/carddisp.pl?gene=SERPINA3
GC19M041966	56.7	https://www.genecards.org/cgi-bin/carddisp.pl?gene=ATP1A3
GC11P089177	56.62	https://www.genecards.org/cgi-bin/carddisp.pl?gene=TYR
GC0XM101397	56.59	https://www.genecards.org/cgi-bin/carddisp.pl?gene=GLA
GC11M116835	56.52	https://www.genecards.org/cgi-bin/carddisp.pl?gene=APOA1
GC22P021549	55.67	https://www.genecards.org/cgi-bin/carddisp.pl?gene=UBE2L3
GC07P150990	55.64	https://www.genecards.org/cgi-bin/carddisp.pl?gene=NOS3
GC18M023506	55.6	https://www.genecards.org/cgi-bin/carddisp.pl?gene=NPC1
GC0XM049187	55.54	https://www.genecards.org/cgi-bin/carddisp.pl?gene=SYP
GC20P003887	55.52	https://www.genecards.org/cgi-bin/carddisp.pl?gene=PANK2
GC0XP071366	55.37	https://www.genecards.org/cgi-bin/carddisp.pl?gene=TAF1
GC09P084668	55.2	https://www.genecards.org/cgi-bin/carddisp.pl?gene=NTRK2
GC12M009067	55.17	https://www.genecards.org/cgi-bin/carddisp.pl?gene=A2M
GC0XM120426	54.92	https://www.genecards.org/cgi-bin/carddisp.pl?gene=LAMP2
GC07M131500	54.76	https://www.genecards.org/cgi-bin/carddisp.pl?gene=PODXL
GC02P055231	54.64	https://www.genecards.org/cgi-bin/carddisp.pl?gene=RPS27A
GC20M001978	54.53	https://www.genecards.org/cgi-bin/carddisp.pl?gene=PDYN
GC19P011061	54.44	https://www.genecards.org/cgi-bin/carddisp.pl?gene=LDLR
GC10P087863	54.44	https://www.genecards.org/cgi-bin/carddisp.pl?gene=PTEN
GC10P058385	54.41	https://www.genecards.org/cgi-bin/carddisp.pl?gene=TFAM
GC10M102230	53.84	https://www.genecards.org/cgi-bin/carddisp.pl?gene=PITX3
GC03P181711	53.84	https://www.genecards.org/cgi-bin/carddisp.pl?gene=SOX2
GC04P073397	53.34	https://www.genecards.org/cgi-bin/carddisp.pl?gene=ALB
GC06P026087	53.01	https://www.genecards.org/cgi-bin/carddisp.pl?gene=HFE
GC19P048965	52.86	https://www.genecards.org/cgi-bin/carddisp.pl?gene=FTL
GC17M058269	52.7	https://www.genecards.org/cgi-bin/carddisp.pl?gene=MPO
GC04P009783	52.69	https://www.genecards.org/cgi-bin/carddisp.pl?gene=DRD5

GC04M184627	52.22	https://www.genecards.org/cgi-bin/carddisp.pl?gene=CASP3
GC03P087277	52.18	https://www.genecards.org/cgi-bin/carddisp.pl?gene=CHMP2B
GC06M032660	52.16	https://www.genecards.org/cgi-bin/carddisp.pl?gene=HLA-DQB1
GC06P159969	52.02	https://www.genecards.org/cgi-bin/carddisp.pl?gene=IGF2R
GC19M013206	51.87	https://www.genecards.org/cgi-bin/carddisp.pl?gene=CACNA1A
GC10P088969	51.69	https://www.genecards.org/cgi-bin/carddisp.pl?gene=FAS
GCMTM014151	51.63	https://www.genecards.org/cgi-bin/carddisp.pl?gene=MT-ND6
GC19M029699	51.52	https://www.genecards.org/cgi-bin/carddisp.pl?gene=C19orf12
GC01U905776	51.32	https://www.genecards.org/cgi-bin/carddisp.pl?gene=LOC106627981
GC16P050693	51.15	https://www.genecards.org/cgi-bin/carddisp.pl?gene=NOD2
GC07P128241	51.08	https://www.genecards.org/cgi-bin/carddisp.pl?gene=LEP
GC19P005805	50.53	https://www.genecards.org/cgi-bin/carddisp.pl?gene=NRTN
GC06M016299	50.53	https://www.genecards.org/cgi-bin/carddisp.pl?gene=ATXN1
GC0XP147912	50.48	https://www.genecards.org/cgi-bin/carddisp.pl?gene=FMR1
GC04M099336	50.47	https://www.genecards.org/cgi-bin/carddisp.pl?gene=ADH1C
GC04M085990	50.16	https://www.genecards.org/cgi-bin/carddisp.pl?gene=MAPK10
GC17M027756	50.13	https://www.genecards.org/cgi-bin/carddisp.pl?gene=NOS2
GC0XP067544	50.01	https://www.genecards.org/cgi-bin/carddisp.pl?gene=AR
GC06M041359	49.88	https://www.genecards.org/cgi-bin/carddisp.pl?gene=TREM2
GC17M042313	49.87	https://www.genecards.org/cgi-bin/carddisp.pl?gene=STAT3
GC03M052401	49.71	https://www.genecards.org/cgi-bin/carddisp.pl?gene=BAP1
GC12M050979	49.68	https://www.genecards.org/cgi-bin/carddisp.pl?gene=SLC11A2
GC11M006614	49.67	https://www.genecards.org/cgi-bin/carddisp.pl?gene=TPP1
GC19P038434	49.59	https://www.genecards.org/cgi-bin/carddisp.pl?gene=RYR1
GC01M020642	49.59	https://www.genecards.org/cgi-bin/carddisp.pl?gene=PINK1-AS
GC07P055019	49.53	https://www.genecards.org/cgi-bin/carddisp.pl?gene=EGFR
GC21P037365	49.52	https://www.genecards.org/cgi-bin/carddisp.pl?gene=DYRK1A
GC10M101770	49.51	https://www.genecards.org/cgi-bin/carddisp.pl?gene=FGF8
GC07M100889	49.45	https://www.genecards.org/cgi-bin/carddisp.pl?gene=ACHE
GC11P111579	49.42	https://www.genecards.org/cgi-bin/carddisp.pl?gene=MIR34C
GC08M127923	49.38	https://www.genecards.org/cgi-bin/carddisp.pl?gene=WASHC5
GC01P022043	49.31	https://www.genecards.org/cgi-bin/carddisp.pl?gene=CDC42
GC17P003472	49.28	https://www.genecards.org/cgi-bin/carddisp.pl?gene=ASPA
GC17M001713	49.24	https://www.genecards.org/cgi-bin/carddisp.pl?gene=MIR22
GC11M001995	49.17	https://www.genecards.org/cgi-bin/carddisp.pl?gene=H19
GC13P070107	49.02	https://www.genecards.org/cgi-bin/carddisp.pl?gene=ATXN8OS
GC01U902505	48.98	https://www.genecards.org/cgi-bin/carddisp.pl?gene=PARK16
GC06P145973	48.95	https://www.genecards.org/cgi-bin/carddisp.pl?gene=GRM1
GC14M023412	48.85	https://www.genecards.org/cgi-bin/carddisp.pl?gene=MYH7
GC06P167111	48.82	https://www.genecards.org/cgi-bin/carddisp.pl?gene=CCR6
GC05M150113	48.56	https://www.genecards.org/cgi-bin/carddisp.pl?gene=PDGFRB
GC16P001253	48.43	https://www.genecards.org/cgi-bin/carddisp.pl?gene=STUB1
GC19M049061	48.36	https://www.genecards.org/cgi-bin/carddisp.pl?gene=NTF4
GC09P126614	48.28	https://www.genecards.org/cgi-bin/carddisp.pl?gene=LMX1B
GC06P162727	47.98	https://www.genecards.org/cgi-bin/carddisp.pl?gene=PACRG
GC03M038549	47.87	https://www.genecards.org/cgi-bin/carddisp.pl?gene=SCN5A
GC16M074746	47.83	https://www.genecards.org/cgi-bin/carddisp.pl?gene=FA2H

GC07P076302	47.63	https://www.genecards.org/cgi-bin/carddisp.pl?gene=HSPB1
GC16M030989	47.55	https://www.genecards.org/cgi-bin/carddisp.pl?gene=STX1B
GC01M011785	47.45	https://www.genecards.org/cgi-bin/carddisp.pl?gene=MTHFR
GC09M104781	47.03	https://www.genecards.org/cgi-bin/carddisp.pl?gene=ABCA1
GC12M057516	46.98	https://www.genecards.org/cgi-bin/carddisp.pl?gene=DDIT3
GC16P055656	46.78	https://www.genecards.org/cgi-bin/carddisp.pl?gene=SLC6A2
GC07P044606	46.54	https://www.genecards.org/cgi-bin/carddisp.pl?gene=OGDH
GC15M044562	46.42	https://www.genecards.org/cgi-bin/carddisp.pl?gene=SPG11
GC07M087504	46.12	https://www.genecards.org/cgi-bin/carddisp.pl?gene=ABCB1
GC09M021967	46.11	https://www.genecards.org/cgi-bin/carddisp.pl?gene=CDKN2A
GC0XM085861	46.07	https://www.genecards.org/cgi-bin/carddisp.pl?gene=CHM
GC13M040555	45.9	https://www.genecards.org/cgi-bin/carddisp.pl?gene=FOXO1
GC05M138554	45.86	https://www.genecards.org/cgi-bin/carddisp.pl?gene=HSPA9
GC20P006696	45.77	https://www.genecards.org/cgi-bin/carddisp.pl?gene=BMP2
GC17P039687	45.67	https://www.genecards.org/cgi-bin/carddisp.pl?gene=ERBB2
GC01U900034	45.65	https://www.genecards.org/cgi-bin/carddisp.pl?gene=PARK10
GC06P151656	45.5	https://www.genecards.org/cgi-bin/carddisp.pl?gene=ESR1
GC02M020956	45.49	https://www.genecards.org/cgi-bin/carddisp.pl?gene=APOB
GC20M023608	45.47	https://www.genecards.org/cgi-bin/carddisp.pl?gene=CST3
GC02P072850	45.36	https://www.genecards.org/cgi-bin/carddisp.pl?gene=SPR
GC0XP121047	45.33	https://www.genecards.org/cgi-bin/carddisp.pl?gene=GLUD2
GC07M151053	45.16	https://www.genecards.org/cgi-bin/carddisp.pl?gene=CDK5
GC01M201359	45.03	https://www.genecards.org/cgi-bin/carddisp.pl?gene=TNNT2
GC04P004861	44.81	https://www.genecards.org/cgi-bin/carddisp.pl?gene=MSX1
GC19M041301	44.71	https://www.genecards.org/cgi-bin/carddisp.pl?gene=TGFB1
GC20P046008	44.62	https://www.genecards.org/cgi-bin/carddisp.pl?gene=MMP9
GC16P002385	44.62	https://www.genecards.org/cgi-bin/carddisp.pl?gene=TSC2
GC03P012287	44.41	https://www.genecards.org/cgi-bin/carddisp.pl?gene=PPARG
GC03M183015	44.4	https://www.genecards.org/cgi-bin/carddisp.pl?gene=MCCC1
GC04P073740	44.33	https://www.genecards.org/cgi-bin/carddisp.pl?gene=CXCL8
GC03P133666	44.27	https://www.genecards.org/cgi-bin/carddisp.pl?gene=TF
GC12M006328	44.27	https://www.genecards.org/cgi-bin/carddisp.pl?gene=TNFRSF1A
GC19P037726	43.82	https://www.genecards.org/cgi-bin/carddisp.pl?gene=PSENEN
GC03P041236	43.75	https://www.genecards.org/cgi-bin/carddisp.pl?gene=CTNNB1
GCMTP010762	43.66	https://www.genecards.org/cgi-bin/carddisp.pl?gene=MT-ND4
GC22M037227	43.64	https://www.genecards.org/cgi-bin/carddisp.pl?gene=RAC2
GC09P094178	43.57	https://www.genecards.org/cgi-bin/carddisp.pl?gene=MIRLET7D
GC11M111908	43.53	https://www.genecards.org/cgi-bin/carddisp.pl?gene=CRYAB
GC06P033442	43.45	https://www.genecards.org/cgi-bin/carddisp.pl?gene=HLA-DQA1
GC18P009092	43.35	https://www.genecards.org/cgi-bin/carddisp.pl?gene=NDUFV2
GC15M072340	43.33	https://www.genecards.org/cgi-bin/carddisp.pl?gene=HEXA
GC02M177227	43.25	https://www.genecards.org/cgi-bin/carddisp.pl?gene=NFE2L2
GC20M010637	43.24	https://www.genecards.org/cgi-bin/carddisp.pl?gene=JAG1
GC07P100720	43.23	https://www.genecards.org/cgi-bin/carddisp.pl?gene=EPO
GC01M204154	43.19	https://www.genecards.org/cgi-bin/carddisp.pl?gene=REN
GC01P010210	42.74	https://www.genecards.org/cgi-bin/carddisp.pl?gene=KIF1B
GC01M020651	42.64	https://www.genecards.org/cgi-bin/carddisp.pl?gene=DDOST

GC17M005432	42.46	https://www.genecards.org/cgi-bin/carddisp.pl?gene=C1QBP
GC07M025118	42.45	https://www.genecards.org/cgi-bin/carddisp.pl?gene=CYCS
GCMTP005906	42.34	https://www.genecards.org/cgi-bin/carddisp.pl?gene=MT-CO1
GC17P042535	42.17	https://www.genecards.org/cgi-bin/carddisp.pl?gene=NAGLU
GC11M000522	42.16	https://www.genecards.org/cgi-bin/carddisp.pl?gene=HRAS
GC09P133636	42.12	https://www.genecards.org/cgi-bin/carddisp.pl?gene=DBH
GC0XM049074	42.07	https://www.genecards.org/cgi-bin/carddisp.pl?gene=WDR45
GC03P057227	42.03	https://www.genecards.org/cgi-bin/carddisp.pl?gene=APPL1
GCMTP008531	41.9	https://www.genecards.org/cgi-bin/carddisp.pl?gene=MT-ATP6
GC15P071832	41.89	https://www.genecards.org/cgi-bin/carddisp.pl?gene=RAB11A
GC17M035871	41.88	https://www.genecards.org/cgi-bin/carddisp.pl?gene=CCL5
GC03P124033	41.84	https://www.genecards.org/cgi-bin/carddisp.pl?gene=KALRN
GC01P172628	41.74	https://www.genecards.org/cgi-bin/carddisp.pl?gene=FASLG
GC20M005069	41.43	https://www.genecards.org/cgi-bin/carddisp.pl?gene=TMEM230
GC0XU900750	41.4	https://www.genecards.org/cgi-bin/carddisp.pl?gene=PARK12
GC22M021754	41.37	https://www.genecards.org/cgi-bin/carddisp.pl?gene=MAPK1
GC06P121436	41.29	https://www.genecards.org/cgi-bin/carddisp.pl?gene=GJA1
GC01P156786	41.24	https://www.genecards.org/cgi-bin/carddisp.pl?gene=NTRK1
GC19M055151	41.18	https://www.genecards.org/cgi-bin/carddisp.pl?gene=TNNI3
GC07M094585	41.12	https://www.genecards.org/cgi-bin/carddisp.pl?gene=SGCE
GC01P099850	41.09	https://www.genecards.org/cgi-bin/carddisp.pl?gene=AGL
GC04P109912	41.04	https://www.genecards.org/cgi-bin/carddisp.pl?gene=EGF
GC10P086957	40.91	https://www.genecards.org/cgi-bin/carddisp.pl?gene=SNCG
GC21P043169	40.86	https://www.genecards.org/cgi-bin/carddisp.pl?gene=CRYAA
GC18M076978	40.85	https://www.genecards.org/cgi-bin/carddisp.pl?gene=MBP
GC03U903043	40.82	https://www.genecards.org/cgi-bin/carddisp.pl?gene=PARK21
GC22M044780	40.66	https://www.genecards.org/cgi-bin/carddisp.pl?gene=PDGFB
GC0XP071215	40.42	https://www.genecards.org/cgi-bin/carddisp.pl?gene=GJB1
GC17P042561	40.34	https://www.genecards.org/cgi-bin/carddisp.pl?gene=COASY
GC07M015917	40.33	https://www.genecards.org/cgi-bin/carddisp.pl?gene=CRPPA
GC12P064451	40.31	https://www.genecards.org/cgi-bin/carddisp.pl?gene=TBK1
GC14P104250	40.29	https://www.genecards.org/cgi-bin/carddisp.pl?gene=MIR433
GC01M042925	40.28	https://www.genecards.org/cgi-bin/carddisp.pl?gene=SLC2A1
GC06P052148	40.2	https://www.genecards.org/cgi-bin/carddisp.pl?gene=MIR133B
GC08M042836	40.15	https://www.genecards.org/cgi-bin/carddisp.pl?gene=THAP1
GC06P003004	39.97	https://www.genecards.org/cgi-bin/carddisp.pl?gene=NQO2
GC0XM154531	39.82	https://www.genecards.org/cgi-bin/carddisp.pl?gene=G6PD
GC14M023380	39.78	https://www.genecards.org/cgi-bin/carddisp.pl?gene=MYH6
GC08P127735	39.74	https://www.genecards.org/cgi-bin/carddisp.pl?gene=MYC
GC11P046720	39.71	https://www.genecards.org/cgi-bin/carddisp.pl?gene=F2
GCMTP014749	39.66	https://www.genecards.org/cgi-bin/carddisp.pl?gene=MT-CYB
GC09P004985	39.54	https://www.genecards.org/cgi-bin/carddisp.pl?gene=JAK2
GC02M178431	39.51	https://www.genecards.org/cgi-bin/carddisp.pl?gene=PRKRA
GCMTP004472	39.51	https://www.genecards.org/cgi-bin/carddisp.pl?gene=MT-ND2
GC19P001040	39.4	https://www.genecards.org/cgi-bin/carddisp.pl?gene=ABCA7
GC06P046047	39.34	https://www.genecards.org/cgi-bin/carddisp.pl?gene=MAPK14
GC11M085957	39.27	https://www.genecards.org/cgi-bin/carddisp.pl?gene=PICALM

GC12M114353	39.25	https://www.genecards.org/cgi-bin/carddisp.pl?gene=TBX5
GC03P179148	39.05	https://www.genecards.org/cgi-bin/carddisp.pl?gene=PIK3CA
GC20P046118	39.02	https://www.genecards.org/cgi-bin/carddisp.pl?gene=CD40
GC11M032365	38.92	https://www.genecards.org/cgi-bin/carddisp.pl?gene=WT1
GC07P012217	38.78	https://www.genecards.org/cgi-bin/carddisp.pl?gene=TMEM106B
GC16P068737	38.77	https://www.genecards.org/cgi-bin/carddisp.pl?gene=CDH1
GC05P112707	38.76	https://www.genecards.org/cgi-bin/carddisp.pl?gene=APC
GC01M174131	38.74	https://www.genecards.org/cgi-bin/carddisp.pl?gene=SERPINC1
GC18M063123	38.67	https://www.genecards.org/cgi-bin/carddisp.pl?gene=BCL2
GC12P105108	38.66	https://www.genecards.org/cgi-bin/carddisp.pl?gene=WASHC4
GC05M173232	38.53	https://www.genecards.org/cgi-bin/carddisp.pl?gene=NKX2-5
GC01M219685	38.41	https://www.genecards.org/cgi-bin/carddisp.pl?gene=SLC30A10
GC12P006493	38.41	https://www.genecards.org/cgi-bin/carddisp.pl?gene=NCAPD2
GC08M038400	38.4	https://www.genecards.org/cgi-bin/carddisp.pl?gene=FGFR1
GC02M136114	38.28	https://www.genecards.org/cgi-bin/carddisp.pl?gene=CXCR4
GC11M117285	38.26	https://www.genecards.org/cgi-bin/carddisp.pl?gene=BACE1
GC12P006630	38.16	https://www.genecards.org/cgi-bin/carddisp.pl?gene=GAPDH
GC10P100994	38.16	https://www.genecards.org/cgi-bin/carddisp.pl?gene=TWNK
GC05M175440	37.96	https://www.genecards.org/cgi-bin/carddisp.pl?gene=DRD1
GC01M015491	37.86	https://www.genecards.org/cgi-bin/carddisp.pl?gene=CASP9
GC08P019901	37.85	https://www.genecards.org/cgi-bin/carddisp.pl?gene=LPL
GC03P155024	37.76	https://www.genecards.org/cgi-bin/carddisp.pl?gene=MME
GC0XP136649	37.76	https://www.genecards.org/cgi-bin/carddisp.pl?gene=CD40LG
GC01P196621	37.67	https://www.genecards.org/cgi-bin/carddisp.pl?gene=CFH
GC06P109691	37.57	https://www.genecards.org/cgi-bin/carddisp.pl?gene=FIG4
GC07P030595	37.54	https://www.genecards.org/cgi-bin/carddisp.pl?gene=GARS1
GC0XM154021	37.54	https://www.genecards.org/cgi-bin/carddisp.pl?gene=MECP2
GC12M117208	37.53	https://www.genecards.org/cgi-bin/carddisp.pl?gene=NOS1
GC11P002444	37.45	https://www.genecards.org/cgi-bin/carddisp.pl?gene=KCNQ1
GC04P054657	37.31	https://www.genecards.org/cgi-bin/carddisp.pl?gene=KIT
GC03P063864	37.17	https://www.genecards.org/cgi-bin/carddisp.pl?gene=ATXN7
GC14M064084	37.17	https://www.genecards.org/cgi-bin/carddisp.pl?gene=ESR2
GC11P006390	37.15	https://www.genecards.org/cgi-bin/carddisp.pl?gene=SMPD1
GC08P022367	36.95	https://www.genecards.org/cgi-bin/carddisp.pl?gene=SLC39A14
GC19M048970	36.92	https://www.genecards.org/cgi-bin/carddisp.pl?gene=GYS1
GC11M059693	36.73	https://www.genecards.org/cgi-bin/carddisp.pl?gene=MYBPC3
GC19M049635	36.68	https://www.genecards.org/cgi-bin/carddisp.pl?gene=RRAS
GC01P151611	36.68	https://www.genecards.org/cgi-bin/carddisp.pl?gene=SNX27
GC02M215360	36.67	https://www.genecards.org/cgi-bin/carddisp.pl?gene=FN1
GC22P024417	36.66	https://www.genecards.org/cgi-bin/carddisp.pl?gene=ADORA2A
GC22P029480	36.64	https://www.genecards.org/cgi-bin/carddisp.pl?gene=NEFH
GC09M136582	36.51	https://www.genecards.org/cgi-bin/carddisp.pl?gene=NOTCH1
GC06P052186	36.49	https://www.genecards.org/cgi-bin/carddisp.pl?gene=IL17A
GC10P048306	36.45	https://www.genecards.org/cgi-bin/carddisp.pl?gene=MAPK8
GC17M041754	36.37	https://www.genecards.org/cgi-bin/carddisp.pl?gene=JUP
GCMTP003232	36.21	https://www.genecards.org/cgi-bin/carddisp.pl?gene=MT-TL1
GC14M102080	36.17	https://www.genecards.org/cgi-bin/carddisp.pl?gene=HSP90AA1

GC10M044370	36.1	https://www.genecards.org/cgi-bin/carddisp.pl?gene=CXCL12
GC06M088139	36.09	https://www.genecards.org/cgi-bin/carddisp.pl?gene=CNR1
GC02P218270	36.08	https://www.genecards.org/cgi-bin/carddisp.pl?gene=PNKD
GC21M043092	36.08	https://www.genecards.org/cgi-bin/carddisp.pl?gene=U2AF1
GC11M002130	36.05	https://www.genecards.org/cgi-bin/carddisp.pl?gene=IGF2
GC03M008980	35.97	https://www.genecards.org/cgi-bin/carddisp.pl?gene=SRGAP3
GC22M023765	35.96	https://www.genecards.org/cgi-bin/carddisp.pl?gene=CHCHD10
GC11P108222	35.93	https://www.genecards.org/cgi-bin/carddisp.pl?gene=ATM
GC07M150944	35.93	https://www.genecards.org/cgi-bin/carddisp.pl?gene=KCNH2
GC18M031058	35.88	https://www.genecards.org/cgi-bin/carddisp.pl?gene=DSC2
GC12P054280	35.84	https://www.genecards.org/cgi-bin/carddisp.pl?gene=HNRNPA1
GC10M049454	35.75	https://www.genecards.org/cgi-bin/carddisp.pl?gene=ERCC6
GC04P056907	35.64	https://www.genecards.org/cgi-bin/carddisp.pl?gene=REST
GC01M203148	35.59	https://www.genecards.org/cgi-bin/carddisp.pl?gene=CHI3L1
GC14M074476	35.54	https://www.genecards.org/cgi-bin/carddisp.pl?gene=NPC2
GC12P001970	35.53	https://www.genecards.org/cgi-bin/carddisp.pl?gene=CACNA1C
GC12M047841	35.52	https://www.genecards.org/cgi-bin/carddisp.pl?gene=VDR
GC11P068921	35.48	https://www.genecards.org/cgi-bin/carddisp.pl?gene=IGHMBP2
GC07M026174	35.41	https://www.genecards.org/cgi-bin/carddisp.pl?gene=HNRNPA2B1
GC02M009488	35.39	https://www.genecards.org/cgi-bin/carddisp.pl?gene=ADAM17
GC10P017227	35.28	https://www.genecards.org/cgi-bin/carddisp.pl?gene=VIM
GC0XP102937	35.27	https://www.genecards.org/cgi-bin/carddisp.pl?gene=RAB40AL
GC02M211375	35.23	https://www.genecards.org/cgi-bin/carddisp.pl?gene=ERBB4
GC11M035272	35.22	https://www.genecards.org/cgi-bin/carddisp.pl?gene=SLC1A2
GC06M108211	35.2	https://www.genecards.org/cgi-bin/carddisp.pl?gene=SNX3
GC05P070924	35.1	https://www.genecards.org/cgi-bin/carddisp.pl?gene=SMN1
GC03M119821	34.96	https://www.genecards.org/cgi-bin/carddisp.pl?gene=GSK3B
GC05M063960	34.87	https://www.genecards.org/cgi-bin/carddisp.pl?gene=HTR1A
GC21M043053	34.78	https://www.genecards.org/cgi-bin/carddisp.pl?gene=CBS
GC12P112418	34.77	https://www.genecards.org/cgi-bin/carddisp.pl?gene=PTPN11
GC02P171434	34.68	https://www.genecards.org/cgi-bin/carddisp.pl?gene=DCAF17
GC02P170813	34.58	https://www.genecards.org/cgi-bin/carddisp.pl?gene=GAD1
GC17M036088	34.57	https://www.genecards.org/cgi-bin/carddisp.pl?gene=CCL3
GC08P018391	34.47	https://www.genecards.org/cgi-bin/carddisp.pl?gene=NAT2
GC17P031007	34.46	https://www.genecards.org/cgi-bin/carddisp.pl?gene=NF1
GC08M030678	34.38	https://www.genecards.org/cgi-bin/carddisp.pl?gene=GSR
GC13P091350	34.3	https://www.genecards.org/cgi-bin/carddisp.pl?gene=MIR17
GC19M050958	34.26	https://www.genecards.org/cgi-bin/carddisp.pl?gene=KLK6
GC20M038127	34.24	https://www.genecards.org/cgi-bin/carddisp.pl?gene=TGM2
GC18M030990	34.23	https://www.genecards.org/cgi-bin/carddisp.pl?gene=DSC3
GC01M226360	34.21	https://www.genecards.org/cgi-bin/carddisp.pl?gene=PARP1
GC01P160343	34.15	https://www.genecards.org/cgi-bin/carddisp.pl?gene=NCSTN
GC07M124745	34.01	https://www.genecards.org/cgi-bin/carddisp.pl?gene=GPR37
GC0XM031047	33.99	https://www.genecards.org/cgi-bin/carddisp.pl?gene=DMD
GC04M169393	33.92	https://www.genecards.org/cgi-bin/carddisp.pl?gene=NEK1
GC01M011858	33.92	https://www.genecards.org/cgi-bin/carddisp.pl?gene=NPPB
GC15M034788	33.88	https://www.genecards.org/cgi-bin/carddisp.pl?gene=ACTC1

GC02M112773	33.82	https://www.genecards.org/cgi-bin/carddisp.pl?gene=IL1A
GC16M028466	33.59	https://www.genecards.org/cgi-bin/carddisp.pl?gene=CLN3
GC12P007874	33.56	https://www.genecards.org/cgi-bin/carddisp.pl?gene=ATN1
GCMTP010061	33.5	https://www.genecards.org/cgi-bin/carddisp.pl?gene=MT-ND3
GC01M149914	33.44	https://www.genecards.org/cgi-bin/carddisp.pl?gene=H2AC18
GC01M186640	33.42	https://www.genecards.org/cgi-bin/carddisp.pl?gene=PTGS2
GC07M103471	33.4	https://www.genecards.org/cgi-bin/carddisp.pl?gene=RELN
GC06P032439	33.4	https://www.genecards.org/cgi-bin/carddisp.pl?gene=HLA-DRA
GC11M064746	33.32	https://www.genecards.org/cgi-bin/carddisp.pl?gene=PYGM
GC10P013141	33.31	https://www.genecards.org/cgi-bin/carddisp.pl?gene=OPTN
GC07P116524	33.28	https://www.genecards.org/cgi-bin/carddisp.pl?gene=CAV1
GC22M036800	33.27	https://www.genecards.org/cgi-bin/carddisp.pl?gene=PVALB
GC22M050622	33.22	https://www.genecards.org/cgi-bin/carddisp.pl?gene=ARSA
GC16P072089	33.22	https://www.genecards.org/cgi-bin/carddisp.pl?gene=HP
GC11P069641	33.18	https://www.genecards.org/cgi-bin/carddisp.pl?gene=CCND1
GC05M150053	33.16	https://www.genecards.org/cgi-bin/carddisp.pl?gene=CSF1R
GC16M069706	33.15	https://www.genecards.org/cgi-bin/carddisp.pl?gene=NQO1
GC16P087601	33.09	https://www.genecards.org/cgi-bin/carddisp.pl?gene=JPH3
GC01P155829	33.03	https://www.genecards.org/cgi-bin/carddisp.pl?gene=SYT11
GC12P032679	33.01	https://www.genecards.org/cgi-bin/carddisp.pl?gene=DNM1L
GC08M027596	32.99	https://www.genecards.org/cgi-bin/carddisp.pl?gene=CLU
GC12P068808	32.99	https://www.genecards.org/cgi-bin/carddisp.pl?gene=MDM2
GC08P031639	32.97	https://www.genecards.org/cgi-bin/carddisp.pl?gene=NRG1
GC12P005432	32.95	https://www.genecards.org/cgi-bin/carddisp.pl?gene=NTF3
GC09P069035	32.89	https://www.genecards.org/cgi-bin/carddisp.pl?gene=FXN
GC12P062606	32.88	https://www.genecards.org/cgi-bin/carddisp.pl?gene=MIRLET7I
GC07M155799	32.84	https://www.genecards.org/cgi-bin/carddisp.pl?gene=SHH
GC02P218382	32.84	https://www.genecards.org/cgi-bin/carddisp.pl?gene=SLC11A1
GCMTP007587	32.83	https://www.genecards.org/cgi-bin/carddisp.pl?gene=MT-CO2
GC03M187668	32.83	https://www.genecards.org/cgi-bin/carddisp.pl?gene=SST
GC11M063080	32.8	https://www.genecards.org/cgi-bin/carddisp.pl?gene=BSCL2
GC07M081699	32.79	https://www.genecards.org/cgi-bin/carddisp.pl?gene=HGF
GC14M053949	32.7	https://www.genecards.org/cgi-bin/carddisp.pl?gene=BMP4
GC04M015965	32.65	https://www.genecards.org/cgi-bin/carddisp.pl?gene=PROM1
GC06M145382	32.61	https://www.genecards.org/cgi-bin/carddisp.pl?gene=EPM2A
GCMTP008297	32.54	https://www.genecards.org/cgi-bin/carddisp.pl?gene=MT-TK
GC04M023755	32.44	https://www.genecards.org/cgi-bin/carddisp.pl?gene=PPARGC1A
GC09M132261	32.4	https://www.genecards.org/cgi-bin/carddisp.pl?gene=SETX
GC15P098648	32.38	https://www.genecards.org/cgi-bin/carddisp.pl?gene=IGF1R
GC11P058622	32.32	https://www.genecards.org/cgi-bin/carddisp.pl?gene=CNTF
GC11M123057	32.31	https://www.genecards.org/cgi-bin/carddisp.pl?gene=HSPA8
GC11P047567	32.31	https://www.genecards.org/cgi-bin/carddisp.pl?gene=NDUFS3
GC01M223109	32.27	https://www.genecards.org/cgi-bin/carddisp.pl?gene=TLR5
GC16M009753	32.13	https://www.genecards.org/cgi-bin/carddisp.pl?gene=GRIN2A
GC06M031289	32.12	https://www.genecards.org/cgi-bin/carddisp.pl?gene=HLA-B
GC02P114906	32.12	https://www.genecards.org/cgi-bin/carddisp.pl?gene=IL1RN
GC11M102942	32.1	https://www.genecards.org/cgi-bin/carddisp.pl?gene=MMP13

GC13P032315	32.07	https://www.genecards.org/cgi-bin/carddisp.pl?gene=BRCA2
GC16M030117	32.05	https://www.genecards.org/cgi-bin/carddisp.pl?gene=MAPK3
GC02M226731	32.05	https://www.genecards.org/cgi-bin/carddisp.pl?gene=IRS1
GC05P160485	32.03	https://www.genecards.org/cgi-bin/carddisp.pl?gene=MIR146A
GC19P044890	32.03	https://www.genecards.org/cgi-bin/carddisp.pl?gene=TOMM40
GC07M095404	32.02	https://www.genecards.org/cgi-bin/carddisp.pl?gene=PON2
GC03P008733	31.98	https://www.genecards.org/cgi-bin/carddisp.pl?gene=CAV3
GC0XP077924	31.88	https://www.genecards.org/cgi-bin/carddisp.pl?gene=ATP7A
GC05M122063	31.85	https://www.genecards.org/cgi-bin/carddisp.pl?gene=LOX
GC02P219418	31.85	https://www.genecards.org/cgi-bin/carddisp.pl?gene=DES
GC02P047402	31.79	https://www.genecards.org/cgi-bin/carddisp.pl?gene=MSH2
GC11M018040	31.72	https://www.genecards.org/cgi-bin/carddisp.pl?gene=TPH1
GC02P209424	31.72	https://www.genecards.org/cgi-bin/carddisp.pl?gene=MAP2
GC02M031294	31.69	https://www.genecards.org/cgi-bin/carddisp.pl?gene=XDH
GC01P236795	31.66	https://www.genecards.org/cgi-bin/carddisp.pl?gene=MTR
GC05M001253	31.65	https://www.genecards.org/cgi-bin/carddisp.pl?gene=TERT
GC02P233309	31.61	https://www.genecards.org/cgi-bin/carddisp.pl?gene=SAG
GC0XP037780	31.58	https://www.genecards.org/cgi-bin/carddisp.pl?gene=CYBB
GC17M043044	31.57	https://www.genecards.org/cgi-bin/carddisp.pl?gene=BRCA1
GC10P052314	31.55	https://www.genecards.org/cgi-bin/carddisp.pl?gene=DKK1
GC21M047166	31.47	https://www.genecards.org/cgi-bin/carddisp.pl?gene=S100B
GC20P037344	31.32	https://www.genecards.org/cgi-bin/carddisp.pl?gene=SRC
GC06M073593	31.32	https://www.genecards.org/cgi-bin/carddisp.pl?gene=SLC17A5
GC09P130713	31.3	https://www.genecards.org/cgi-bin/carddisp.pl?gene=ABL1
GC09M132891	31.29	https://www.genecards.org/cgi-bin/carddisp.pl?gene=TSC1
GC17M079089	31.26	https://www.genecards.org/cgi-bin/carddisp.pl?gene=RBFOX3
GC22M037125	31.21	https://www.genecards.org/cgi-bin/carddisp.pl?gene=IL2RB
GC07P107660	31.2	https://www.genecards.org/cgi-bin/carddisp.pl?gene=SLC26A4
GC15M050552	31.15	https://www.genecards.org/cgi-bin/carddisp.pl?gene=TRPM7
GC15P078437	31.11	https://www.genecards.org/cgi-bin/carddisp.pl?gene=IREB2
GC08P093754	31.1	https://www.genecards.org/cgi-bin/carddisp.pl?gene=TMEM67
GC01M009151	31.02	https://www.genecards.org/cgi-bin/carddisp.pl?gene=MIR34A
GC02M201701	30.98	https://www.genecards.org/cgi-bin/carddisp.pl?gene=ALS2
GC01M017020	30.92	https://www.genecards.org/cgi-bin/carddisp.pl?gene=SDHB
GC17P045999	30.89	https://www.genecards.org/cgi-bin/carddisp.pl?gene=STH
GC16M003726	30.89	https://www.genecards.org/cgi-bin/carddisp.pl?gene=CREBBP
GC08P074315	30.86	https://www.genecards.org/cgi-bin/carddisp.pl?gene=GDAP1
GC0XP153517	30.79	https://www.genecards.org/cgi-bin/carddisp.pl?gene=ATP2B3
GC04M076158	30.79	https://www.genecards.org/cgi-bin/carddisp.pl?gene=SCARB2
GC01M205768	30.72	https://www.genecards.org/cgi-bin/carddisp.pl?gene=RAB29
GC04M000849	30.72	https://www.genecards.org/cgi-bin/carddisp.pl?gene=GAK
GC02M038034	30.67	https://www.genecards.org/cgi-bin/carddisp.pl?gene=CYP1B1
GC07P101127	30.67	https://www.genecards.org/cgi-bin/carddisp.pl?gene=SERPINE1
GC18P011689	30.66	https://www.genecards.org/cgi-bin/carddisp.pl?gene=GNAL
GC11P067623	30.52	https://www.genecards.org/cgi-bin/carddisp.pl?gene=NDUFV1
GC06P046057	30.45	https://www.genecards.org/cgi-bin/carddisp.pl?gene=CDKN1A
GC01M021217	30.45	https://www.genecards.org/cgi-bin/carddisp.pl?gene=ECE1

GC19P035292	30.43	https://www.genecards.org/cgi-bin/carddisp.pl?gene=MAG
GC0XP056563	30.42	https://www.genecards.org/cgi-bin/carddisp.pl?gene=UBQLN2
GC05P133051	30.42	https://www.genecards.org/cgi-bin/carddisp.pl?gene=HSPA4
GC12P085876	30.4	https://www.genecards.org/cgi-bin/carddisp.pl?gene=NTS
GC19P051723	30.38	https://www.genecards.org/cgi-bin/carddisp.pl?gene=MIR125A
GC20P063895	30.37	https://www.genecards.org/cgi-bin/carddisp.pl?gene=DNAJC5
GC17P040015	30.24	https://www.genecards.org/cgi-bin/carddisp.pl?gene=CSF3
GC15P066386	30.22	https://www.genecards.org/cgi-bin/carddisp.pl?gene=MAP2K1
GC22P041091	30.09	https://www.genecards.org/cgi-bin/carddisp.pl?gene=EP300
GC10M087050	30.06	https://www.genecards.org/cgi-bin/carddisp.pl?gene=GLUD1
GC19M045769	30.06	https://www.genecards.org/cgi-bin/carddisp.pl?gene=DMPK
GC08M090058	30.05	https://www.genecards.org/cgi-bin/carddisp.pl?gene=CALB1
GC19P010270	30.04	https://www.genecards.org/cgi-bin/carddisp.pl?gene=ICAM1
GC12P057549	30	https://www.genecards.org/cgi-bin/carddisp.pl?gene=KIF5A
GC11M031784	29.97	https://www.genecards.org/cgi-bin/carddisp.pl?gene=PAX6
GC19M055132	29.89	https://www.genecards.org/cgi-bin/carddisp.pl?gene=TNNT1
GC19P037707	29.89	https://www.genecards.org/cgi-bin/carddisp.pl?gene=HAMP
GC06M018065	29.87	https://www.genecards.org/cgi-bin/carddisp.pl?gene=NHLRC1
GC11P113974	29.81	https://www.genecards.org/cgi-bin/carddisp.pl?gene=HTR3A
GC08P144095	29.8	https://www.genecards.org/cgi-bin/carddisp.pl?gene=CYC1
GC04P185143	29.79	https://www.genecards.org/cgi-bin/carddisp.pl?gene=SLC25A4
GC01P032885	29.78	https://www.genecards.org/cgi-bin/carddisp.pl?gene=HPCA
GC10P073909	29.75	https://www.genecards.org/cgi-bin/carddisp.pl?gene=PLAU
GC08M066176	29.74	https://www.genecards.org/cgi-bin/carddisp.pl?gene=CRH
GC07M041960	29.74	https://www.genecards.org/cgi-bin/carddisp.pl?gene=GLI3
GC07P097731	29.71	https://www.genecards.org/cgi-bin/carddisp.pl?gene=TAC1
GC22P043151	29.67	https://www.genecards.org/cgi-bin/carddisp.pl?gene=TSPO
GC09P077177	29.65	https://www.genecards.org/cgi-bin/carddisp.pl?gene=VPS13A
GC02M166195	29.62	https://www.genecards.org/cgi-bin/carddisp.pl?gene=SCN9A
GC09P121207	29.61	https://www.genecards.org/cgi-bin/carddisp.pl?gene=GSN
GC05P151229	29.53	https://www.genecards.org/cgi-bin/carddisp.pl?gene=GM2A
GC19M010133	29.49	https://www.genecards.org/cgi-bin/carddisp.pl?gene=DNMT1
GC04P054229	29.4	https://www.genecards.org/cgi-bin/carddisp.pl?gene=PDGFRA
GC15M077613	29.39	https://www.genecards.org/cgi-bin/carddisp.pl?gene=LINGO1
GC10P069123	29.37	https://www.genecards.org/cgi-bin/carddisp.pl?gene=VPS26A
GC03P089077	29.36	https://www.genecards.org/cgi-bin/carddisp.pl?gene=EPHA3
GC13P048303	29.32	https://www.genecards.org/cgi-bin/carddisp.pl?gene=RB1
GC11M102810	29.32	https://www.genecards.org/cgi-bin/carddisp.pl?gene=MMP1
GC08M100257	29.31	https://www.genecards.org/cgi-bin/carddisp.pl?gene=RNF19A
GC03P004486	29.29	https://www.genecards.org/cgi-bin/carddisp.pl?gene=ITPR1
GC01M114704	29.26	https://www.genecards.org/cgi-bin/carddisp.pl?gene=NRAS
GC08P144502	29.16	https://www.genecards.org/cgi-bin/carddisp.pl?gene=GPT
GC05P132673	29.14	https://www.genecards.org/cgi-bin/carddisp.pl?gene=IL4
GC14P025088	29.12	https://www.genecards.org/cgi-bin/carddisp.pl?gene=PABPN1
GC02M165989	29.12	https://www.genecards.org/cgi-bin/carddisp.pl?gene=SCN1A
GC06P033433	29.09	https://www.genecards.org/cgi-bin/carddisp.pl?gene=C4A
GC08M011842	29.04	https://www.genecards.org/cgi-bin/carddisp.pl?gene=CTSB

GC03P010205	29.01	https://www.genecards.org/cgi-bin/carddisp.pl?gene=VHL
GC17P070168	29	https://www.genecards.org/cgi-bin/carddisp.pl?gene=KCNJ2
GC12P051590	29	https://www.genecards.org/cgi-bin/carddisp.pl?gene=SCN8A
GC17P042900	28.94	https://www.genecards.org/cgi-bin/carddisp.pl?gene=G6PC
GC01M147756	28.89	https://www.genecards.org/cgi-bin/carddisp.pl?gene=GJA5
GC02P165130	28.84	https://www.genecards.org/cgi-bin/carddisp.pl?gene=SCN2A
GC07P006016	28.78	https://www.genecards.org/cgi-bin/carddisp.pl?gene=AIMP2
GC07M035237	28.77	https://www.genecards.org/cgi-bin/carddisp.pl?gene=TBX20
GC01M156420	28.76	https://www.genecards.org/cgi-bin/carddisp.pl?gene=MIR9-1
GC12P048105	28.72	https://www.genecards.org/cgi-bin/carddisp.pl?gene=PFKM
GC01M021822	28.7	https://www.genecards.org/cgi-bin/carddisp.pl?gene=HSPG2
GC22P023786	28.66	https://www.genecards.org/cgi-bin/carddisp.pl?gene=SMARCB1
GC16P089492	28.66	https://www.genecards.org/cgi-bin/carddisp.pl?gene=SPG7
GC11P067583	28.64	https://www.genecards.org/cgi-bin/carddisp.pl?gene=GSTP1
GC0XP119871	28.61	https://www.genecards.org/cgi-bin/carddisp.pl?gene=NDUFA1
GC11P065198	28.6	https://www.genecards.org/cgi-bin/carddisp.pl?gene=CAPN1
GC10M071816	28.53	https://www.genecards.org/cgi-bin/carddisp.pl?gene=PSAP
GC05P036606	28.52	https://www.genecards.org/cgi-bin/carddisp.pl?gene=SLC1A3
GC07P117465	28.51	https://www.genecards.org/cgi-bin/carddisp.pl?gene=CFTR
GC11M129899	28.5	https://www.genecards.org/cgi-bin/carddisp.pl?gene=PRDM10
GC20M050888	28.44	https://www.genecards.org/cgi-bin/carddisp.pl?gene=ADNP
GC06M032530	28.43	https://www.genecards.org/cgi-bin/carddisp.pl?gene=HLA-DRB5
GC02M189560	28.43	https://www.genecards.org/cgi-bin/carddisp.pl?gene=SLC40A1
GC15M074919	28.4	https://www.genecards.org/cgi-bin/carddisp.pl?gene=COX5A
GC08M096142	28.37	https://www.genecards.org/cgi-bin/carddisp.pl?gene=GDF6
GC01P161197	28.25	https://www.genecards.org/cgi-bin/carddisp.pl?gene=NDUFS2
GC12M057743	28.23	https://www.genecards.org/cgi-bin/carddisp.pl?gene=CDK4
GC04M122451	28.2	https://www.genecards.org/cgi-bin/carddisp.pl?gene=IL2
GC03P046384	28.19	https://www.genecards.org/cgi-bin/carddisp.pl?gene=CCR5
GC09P127581	28.13	https://www.genecards.org/cgi-bin/carddisp.pl?gene=STXBP1
GC19M007112	28.06	https://www.genecards.org/cgi-bin/carddisp.pl?gene=INSR
GC02M219249	28	https://www.genecards.org/cgi-bin/carddisp.pl?gene=TUBA4A
GC11M017392	27.97	https://www.genecards.org/cgi-bin/carddisp.pl?gene=ABCC8
GC04P122826	27.84	https://www.genecards.org/cgi-bin/carddisp.pl?gene=FGF2
GC03M183825	27.83	https://www.genecards.org/cgi-bin/carddisp.pl?gene=PARL
GC02P032063	27.76	https://www.genecards.org/cgi-bin/carddisp.pl?gene=SPAST
GC02M070209	27.76	https://www.genecards.org/cgi-bin/carddisp.pl?gene=TIA1
GC11P068701	27.75	https://www.genecards.org/cgi-bin/carddisp.pl?gene=GAL
GC04M110617	27.74	https://www.genecards.org/cgi-bin/carddisp.pl?gene=PITX2
GC05P070049	27.71	https://www.genecards.org/cgi-bin/carddisp.pl?gene=SMN2
GC09P095086	27.71	https://www.genecards.org/cgi-bin/carddisp.pl?gene=MIR24-1
GC13P113297	27.67	https://www.genecards.org/cgi-bin/carddisp.pl?gene=LAMP1
GC11M088504	27.67	https://www.genecards.org/cgi-bin/carddisp.pl?gene=GRM5
GC07P024290	27.61	https://www.genecards.org/cgi-bin/carddisp.pl?gene=NPY
GC09M036214	27.61	https://www.genecards.org/cgi-bin/carddisp.pl?gene=GNE
GC05P060945	27.59	https://www.genecards.org/cgi-bin/carddisp.pl?gene=NDUFAF2
GC08M042416	27.55	https://www.genecards.org/cgi-bin/carddisp.pl?gene=SLC20A2

GC0XP114584	27.54	https://www.genecards.org/cgi-bin/carddisp.pl?gene=HTR2C
GC22M050578	27.53	https://www.genecards.org/cgi-bin/carddisp.pl?gene=CHKB
GCMTP009209	27.5	https://www.genecards.org/cgi-bin/carddisp.pl?gene=MT-CO3
GC16P055390	27.48	https://www.genecards.org/cgi-bin/carddisp.pl?gene=MMP2
GC20P058389	27.44	https://www.genecards.org/cgi-bin/carddisp.pl?gene=VAPB
GC17P016380	27.41	https://www.genecards.org/cgi-bin/carddisp.pl?gene=UBB
GC10M116056	27.32	https://www.genecards.org/cgi-bin/carddisp.pl?gene=GFRA1
GC01P053196	27.3	https://www.genecards.org/cgi-bin/carddisp.pl?gene=CPT2
GC03P019963	27.29	https://www.genecards.org/cgi-bin/carddisp.pl?gene=RAB5A
GC02P047695	27.27	https://www.genecards.org/cgi-bin/carddisp.pl?gene=MSH6
GC16P056961	27.27	https://www.genecards.org/cgi-bin/carddisp.pl?gene=CETP
GC09M022002	27.25	https://www.genecards.org/cgi-bin/carddisp.pl?gene=CDKN2B
GC11P000884	27.2	https://www.genecards.org/cgi-bin/carddisp.pl?gene=PNPLA2
GC08P060678	27.2	https://www.genecards.org/cgi-bin/carddisp.pl?gene=CHD7
GC12P120978	27.18	https://www.genecards.org/cgi-bin/carddisp.pl?gene=HNF1A
GC0XP065588	27.17	https://www.genecards.org/cgi-bin/carddisp.pl?gene=MSN
GC11M102835	27.12	https://www.genecards.org/cgi-bin/carddisp.pl?gene=MMP3
GC10P045374	27.12	https://www.genecards.org/cgi-bin/carddisp.pl?gene=ALOX5
GC15M087859	27.11	https://www.genecards.org/cgi-bin/carddisp.pl?gene=NTRK3
GC18M042743	27.07	https://www.genecards.org/cgi-bin/carddisp.pl?gene=RIT2
GC01M045511	27.06	https://www.genecards.org/cgi-bin/carddisp.pl?gene=PRDX1
GC05P000208	27.06	https://www.genecards.org/cgi-bin/carddisp.pl?gene=SDHA
GC0XM053431	27.05	https://www.genecards.org/cgi-bin/carddisp.pl?gene=HSD17B10
GC19P010932	27.04	https://www.genecards.org/cgi-bin/carddisp.pl?gene=SMARCA4
GC01M115700	26.94	https://www.genecards.org/cgi-bin/carddisp.pl?gene=CASQ2
GC05M088718	26.92	https://www.genecards.org/cgi-bin/carddisp.pl?gene=MEF2C
GC02M206114	26.86	https://www.genecards.org/cgi-bin/carddisp.pl?gene=NDUFS1
GC17M029703	26.83	https://www.genecards.org/cgi-bin/carddisp.pl?gene=MIR144
GC12P057128	26.82	https://www.genecards.org/cgi-bin/carddisp.pl?gene=LRP1
GC0XP123184	26.74	https://www.genecards.org/cgi-bin/carddisp.pl?gene=GRIA3
GC05P153467	26.73	https://www.genecards.org/cgi-bin/carddisp.pl?gene=GRIA1
GC17M046031	26.69	https://www.genecards.org/cgi-bin/carddisp.pl?gene=KANSL1
GC12M053962	26.66	https://www.genecards.org/cgi-bin/carddisp.pl?gene=HOTAIR
GC09M095442	26.66	https://www.genecards.org/cgi-bin/carddisp.pl?gene=PTCH1
GC19P001470	26.64	https://www.genecards.org/cgi-bin/carddisp.pl?gene=NDUFS7
GC20M003082	26.64	https://www.genecards.org/cgi-bin/carddisp.pl?gene=AVP
GC13P091427	26.63	https://www.genecards.org/cgi-bin/carddisp.pl?gene=MIR20A
GC07P023238	26.6	https://www.genecards.org/cgi-bin/carddisp.pl?gene=GPNMB
GC19P001177	26.58	https://www.genecards.org/cgi-bin/carddisp.pl?gene=STK11
GC14P092923	26.56	https://www.genecards.org/cgi-bin/carddisp.pl?gene=CHGA
GC20P032762	26.56	https://www.genecards.org/cgi-bin/carddisp.pl?gene=DNMT3B
GC01P207496	26.52	https://www.genecards.org/cgi-bin/carddisp.pl?gene=CR1
GC14M094376	26.49	https://www.genecards.org/cgi-bin/carddisp.pl?gene=SERPINA1
GC11P068298	26.43	https://www.genecards.org/cgi-bin/carddisp.pl?gene=LRP5
GC09P032374	26.39	https://www.genecards.org/cgi-bin/carddisp.pl?gene=ACO1
GC03M196027	26.38	https://www.genecards.org/cgi-bin/carddisp.pl?gene=TFRC
GC20M063343	26.35	https://www.genecards.org/cgi-bin/carddisp.pl?gene=CHRNA4

GC0XP047583	26.35	https://www.genecards.org/cgi-bin/carddisp.pl?gene=TIMP1
GC02M025160	26.35	https://www.genecards.org/cgi-bin/carddisp.pl?gene=POMC
GC03P115623	26.34	https://www.genecards.org/cgi-bin/carddisp.pl?gene=GAP43
GC10P104235	26.34	https://www.genecards.org/cgi-bin/carddisp.pl?gene=GSTO1
GC02U990073	26.29	https://www.genecards.org/cgi-bin/carddisp.pl?gene=PARK3
GC02M178525	26.29	https://www.genecards.org/cgi-bin/carddisp.pl?gene=TTN
GC14M036516	26.28	https://www.genecards.org/cgi-bin/carddisp.pl?gene=NKX2-1
GC09M092711	26.28	https://www.genecards.org/cgi-bin/carddisp.pl?gene=BICD2
GC19P019515	26.27	https://www.genecards.org/cgi-bin/carddisp.pl?gene=NDUFA13
GC21M034446	26.24	https://www.genecards.org/cgi-bin/carddisp.pl?gene=KCNE1
GC06P033396	26.13	https://www.genecards.org/cgi-bin/carddisp.pl?gene=AIF1
GC15P100919	26.12	https://www.genecards.org/cgi-bin/carddisp.pl?gene=LRRK1
GC12P056086	26.07	https://www.genecards.org/cgi-bin/carddisp.pl?gene=ERBB3
GC03M039279	26.03	https://www.genecards.org/cgi-bin/carddisp.pl?gene=CX3CR1
GC03P009751	26.02	https://www.genecards.org/cgi-bin/carddisp.pl?gene=OGG1
GC06M041835	25.95	https://www.genecards.org/cgi-bin/carddisp.pl?gene=TFEB
GC20P010218	25.94	https://www.genecards.org/cgi-bin/carddisp.pl?gene=SNAP25
GC05P139274	25.92	https://www.genecards.org/cgi-bin/carddisp.pl?gene=MATR3
GC09M092031	25.92	https://www.genecards.org/cgi-bin/carddisp.pl?gene=SPTLC1
GC12M025204	25.92	https://www.genecards.org/cgi-bin/carddisp.pl?gene=KRAS
GC17M002050	25.91	https://www.genecards.org/cgi-bin/carddisp.pl?gene=MIR212
GC0XP066018	25.89	https://www.genecards.org/cgi-bin/carddisp.pl?gene=MIR223
GC16M048357	25.88	https://www.genecards.org/cgi-bin/carddisp.pl?gene=SIAH1
GC07M140719	25.87	https://www.genecards.org/cgi-bin/carddisp.pl?gene=BRAF
GC12P006786	25.87	https://www.genecards.org/cgi-bin/carddisp.pl?gene=CD4
GC02P201071	25.83	https://www.genecards.org/cgi-bin/carddisp.pl?gene=NDUFB3
GC0XP134460	25.81	https://www.genecards.org/cgi-bin/carddisp.pl?gene=HPRT1
GC15M068206	25.79	https://www.genecards.org/cgi-bin/carddisp.pl?gene=CLN6
GC17P072121	25.78	https://www.genecards.org/cgi-bin/carddisp.pl?gene=SOX9
GC03P148697	25.76	https://www.genecards.org/cgi-bin/carddisp.pl?gene=AGTR1
GC03M129167	25.75	https://www.genecards.org/cgi-bin/carddisp.pl?gene=CNBP
GC09P137138	25.71	https://www.genecards.org/cgi-bin/carddisp.pl?gene=GRIN1
GC14P061695	25.7	https://www.genecards.org/cgi-bin/carddisp.pl?gene=HIF1A
GC02M208236	25.7	https://www.genecards.org/cgi-bin/carddisp.pl?gene=IDH1
GC17M063938	25.64	https://www.genecards.org/cgi-bin/carddisp.pl?gene=SCN4A
GC15M041387	25.63	https://www.genecards.org/cgi-bin/carddisp.pl?gene=NDUFAF1
GC02M167954	25.62	https://www.genecards.org/cgi-bin/carddisp.pl?gene=STK39
GC01M160215	25.61	https://www.genecards.org/cgi-bin/carddisp.pl?gene=DCAF8
GC07M130877	25.6	https://www.genecards.org/cgi-bin/carddisp.pl?gene=MIR29B1
GC07P148697	25.6	https://www.genecards.org/cgi-bin/carddisp.pl?gene=CUL1
GC0XP103773	25.58	https://www.genecards.org/cgi-bin/carddisp.pl?gene=PLP1
GC22M042085	25.55	https://www.genecards.org/cgi-bin/carddisp.pl?gene=NDUFA6
GC0XM047142	25.54	https://www.genecards.org/cgi-bin/carddisp.pl?gene=NDUFB11
GC08P124539	25.52	https://www.genecards.org/cgi-bin/carddisp.pl?gene=NDUFB9
GC11P112087	25.5	https://www.genecards.org/cgi-bin/carddisp.pl?gene=SDHD
GC11M014945	25.5	https://www.genecards.org/cgi-bin/carddisp.pl?gene=CALCA
GC22M050525	25.5	https://www.genecards.org/cgi-bin/carddisp.pl?gene=TYMP

GC09P087497	25.49	https://www.genecards.org/cgi-bin/carddisp.pl?gene=DAPK1
GC06P033211	25.45	https://www.genecards.org/cgi-bin/carddisp.pl?gene=HLA-A
GC04P102501	25.43	https://www.genecards.org/cgi-bin/carddisp.pl?gene=NFKB1
GC11P068030	25.38	https://www.genecards.org/cgi-bin/carddisp.pl?gene=NDUFS8
GC11M112143	25.38	https://www.genecards.org/cgi-bin/carddisp.pl?gene=IL18
GC07M005527	25.34	https://www.genecards.org/cgi-bin/carddisp.pl?gene=ACTB
GC02M169127	25.33	https://www.genecards.org/cgi-bin/carddisp.pl?gene=LRP2
GC15P044711	25.32	https://www.genecards.org/cgi-bin/carddisp.pl?gene=B2M
GC04M101024	25.32	https://www.genecards.org/cgi-bin/carddisp.pl?gene=PPP3CA
GC04P000986	25.3	https://www.genecards.org/cgi-bin/carddisp.pl?gene=IDUA
GC15P031923	25.28	https://www.genecards.org/cgi-bin/carddisp.pl?gene=CHRNA7
GC04M041746	25.27	https://www.genecards.org/cgi-bin/carddisp.pl?gene=PHOX2B
GC01P247415	25.21	https://www.genecards.org/cgi-bin/carddisp.pl?gene=NLRP3
GC07P116672	25.2	https://www.genecards.org/cgi-bin/carddisp.pl?gene=MET
GC11M105025	25.15	https://www.genecards.org/cgi-bin/carddisp.pl?gene=CASP1
GC14P028766	25.12	https://www.genecards.org/cgi-bin/carddisp.pl?gene=FOXG1
GC16M066508	25.11	https://www.genecards.org/cgi-bin/carddisp.pl?gene=TK2
GC19P003880	25.08	https://www.genecards.org/cgi-bin/carddisp.pl?gene=ATCAY
GC16P029811	25.07	https://www.genecards.org/cgi-bin/carddisp.pl?gene=PRRT2
GC15M058588	25.06	https://www.genecards.org/cgi-bin/carddisp.pl?gene=ADAM10
GC01P109687	25.04	https://www.genecards.org/cgi-bin/carddisp.pl?gene=GSTM1
GC12M049018	25.04	https://www.genecards.org/cgi-bin/carddisp.pl?gene=KMT2D
GC11M061959	25.01	https://www.genecards.org/cgi-bin/carddisp.pl?gene=FTH1
GC14M067727	24.99	https://www.genecards.org/cgi-bin/carddisp.pl?gene=ZFYVE26
GC11P077128	24.99	https://www.genecards.org/cgi-bin/carddisp.pl?gene=MYO7A
GC15M065001	24.96	https://www.genecards.org/cgi-bin/carddisp.pl?gene=MTFMT
GC01M205789	24.96	https://www.genecards.org/cgi-bin/carddisp.pl?gene=SLC41A1
GC04P087975	24.95	https://www.genecards.org/cgi-bin/carddisp.pl?gene=SPP1
GC17M007189	24.92	https://www.genecards.org/cgi-bin/carddisp.pl?gene=DLG4
GC15P040694	24.91	https://www.genecards.org/cgi-bin/carddisp.pl?gene=RAD51
GC16P027325	24.89	https://www.genecards.org/cgi-bin/carddisp.pl?gene=IL4R
GC01M058780	24.89	https://www.genecards.org/cgi-bin/carddisp.pl?gene=JUN
GC12P004649	24.88	https://www.genecards.org/cgi-bin/carddisp.pl?gene=NDUFA9
GC11P114257	24.87	https://www.genecards.org/cgi-bin/carddisp.pl?gene=NNMT
GC19M055158	24.86	https://www.genecards.org/cgi-bin/carddisp.pl?gene=DNAAF3
GC01M109310	24.84	https://www.genecards.org/cgi-bin/carddisp.pl?gene=SORT1
GC12P122774	24.83	https://www.genecards.org/cgi-bin/carddisp.pl?gene=CCDC62
GC03P186842	24.79	https://www.genecards.org/cgi-bin/carddisp.pl?gene=ADIPOQ
GC02M202206	24.79	https://www.genecards.org/cgi-bin/carddisp.pl?gene=SUMO1
GC0XP040582	24.77	https://www.genecards.org/cgi-bin/carddisp.pl?gene=ATP6AP2
GC09M107484	24.75	https://www.genecards.org/cgi-bin/carddisp.pl?gene=KLF4
GC21P034364	24.72	https://www.genecards.org/cgi-bin/carddisp.pl?gene=KCNE2
GC10M052760	24.7	https://www.genecards.org/cgi-bin/carddisp.pl?gene=MBL2
GC01P012167	24.7	https://www.genecards.org/cgi-bin/carddisp.pl?gene=TNFRSF1B
GC13M110148	24.69	https://www.genecards.org/cgi-bin/carddisp.pl?gene=COL4A1
GC09M072900	24.68	https://www.genecards.org/cgi-bin/carddisp.pl?gene=ALDH1A1
GC10M104031	24.63	https://www.genecards.org/cgi-bin/carddisp.pl?gene=COL17A1

GC10M100523	24.62	https://www.genecards.org/cgi-bin/carddisp.pl?gene=NDUFB8
GC14P054398	24.61	https://www.genecards.org/cgi-bin/carddisp.pl?gene=CDKN3
GC06M042977	24.61	https://www.genecards.org/cgi-bin/carddisp.pl?gene=PRPH2
GC15M064963	24.58	https://www.genecards.org/cgi-bin/carddisp.pl?gene=SPG21
GC14P020810	24.58	https://www.genecards.org/cgi-bin/carddisp.pl?gene=ANG
GC07P146116	24.57	https://www.genecards.org/cgi-bin/carddisp.pl?gene=CNTNAP2
GC22P045673	24.54	https://www.genecards.org/cgi-bin/carddisp.pl?gene=ATXN10
GC09M034634	24.49	https://www.genecards.org/cgi-bin/carddisp.pl?gene=SIGMAR1
GC02P180967	24.43	https://www.genecards.org/cgi-bin/carddisp.pl?gene=UBE2E3
GC20M056370	24.43	https://www.genecards.org/cgi-bin/carddisp.pl?gene=AURKA
GC22P024583	24.42	https://www.genecards.org/cgi-bin/carddisp.pl?gene=GGT1
GC12P012716	24.41	https://www.genecards.org/cgi-bin/carddisp.pl?gene=CDKN1B
GC12M102836	24.38	https://www.genecards.org/cgi-bin/carddisp.pl?gene=PAH
GC0XP037685	24.38	https://www.genecards.org/cgi-bin/carddisp.pl?gene=XK
GC10P104268	24.37	https://www.genecards.org/cgi-bin/carddisp.pl?gene=GSTO2
GC11P065621	24.37	https://www.genecards.org/cgi-bin/carddisp.pl?gene=MALAT1
GC01P180632	24.31	https://www.genecards.org/cgi-bin/carddisp.pl?gene=XPR1
GC01M215622	24.29	https://www.genecards.org/cgi-bin/carddisp.pl?gene=USH2A
GC04M088090	24.28	https://www.genecards.org/cgi-bin/carddisp.pl?gene=ABCG2
GC07P157335	24.27	https://www.genecards.org/cgi-bin/carddisp.pl?gene=DNAJB6
GC17M046762	24.24	https://www.genecards.org/cgi-bin/carddisp.pl?gene=WNT3
GC18P000895	24.23	https://www.genecards.org/cgi-bin/carddisp.pl?gene=ADCYAP1
GC12P122834	24.22	https://www.genecards.org/cgi-bin/carddisp.pl?gene=HIP1R
GC12P006867	24.16	https://www.genecards.org/cgi-bin/carddisp.pl?gene=TPI1
GC0XM043948	24.16	https://www.genecards.org/cgi-bin/carddisp.pl?gene=NDP
GC02M239893	24.11	https://www.genecards.org/cgi-bin/carddisp.pl?gene=NDUFA10
GC17P068326	24.11	https://www.genecards.org/cgi-bin/carddisp.pl?gene=ARSG
GC08P038007	24.09	https://www.genecards.org/cgi-bin/carddisp.pl?gene=EIF4EBP1
GC19P000854	24.07	https://www.genecards.org/cgi-bin/carddisp.pl?gene=ELANE
GC05P075336	24.05	https://www.genecards.org/cgi-bin/carddisp.pl?gene=HMGCR
GC11P065418	24.03	https://www.genecards.org/cgi-bin/carddisp.pl?gene=NEAT1
GC01M152274	23.99	https://www.genecards.org/cgi-bin/carddisp.pl?gene=FLG
GC05P001801	23.99	https://www.genecards.org/cgi-bin/carddisp.pl?gene=NDUFS6
GC08M089933	23.95	https://www.genecards.org/cgi-bin/carddisp.pl?gene=NBN
GC18M012328	23.95	https://www.genecards.org/cgi-bin/carddisp.pl?gene=AFG3L2
GC17M004945	23.93	https://www.genecards.org/cgi-bin/carddisp.pl?gene=PFN1
GC07M096120	23.93	https://www.genecards.org/cgi-bin/carddisp.pl?gene=SLC25A13
GC01M119911	23.93	https://www.genecards.org/cgi-bin/carddisp.pl?gene=NOTCH2
GC05P139526	23.93	https://www.genecards.org/cgi-bin/carddisp.pl?gene=UBE2D2
GC04M000942	23.89	https://www.genecards.org/cgi-bin/carddisp.pl?gene=DGKQ
GC08M102204	23.89	https://www.genecards.org/cgi-bin/carddisp.pl?gene=RRM2B
GC07M130876	23.84	https://www.genecards.org/cgi-bin/carddisp.pl?gene=MIR29A
GC02M197486	23.84	https://www.genecards.org/cgi-bin/carddisp.pl?gene=HSPD1
GC19P040348	23.83	https://www.genecards.org/cgi-bin/carddisp.pl?gene=PLD3
GC15M048408	23.8	https://www.genecards.org/cgi-bin/carddisp.pl?gene=FBN1
GC12M114670	23.8	https://www.genecards.org/cgi-bin/carddisp.pl?gene=TBX3
GC01M219969	23.79	https://www.genecards.org/cgi-bin/carddisp.pl?gene=EPRS1

GC01M211476	23.78	https://www.genecards.org/cgi-bin/carddisp.pl?gene=RD3
GC19P046746	23.77	https://www.genecards.org/cgi-bin/carddisp.pl?gene=FKRP
GC07M148807	23.75	https://www.genecards.org/cgi-bin/carddisp.pl?gene=EZH2
GC02M029156	23.75	https://www.genecards.org/cgi-bin/carddisp.pl?gene=ALK
GC12P021354	23.73	https://www.genecards.org/cgi-bin/carddisp.pl?gene=IAPP
GC17P050561	23.72	https://www.genecards.org/cgi-bin/carddisp.pl?gene=CACNA1G
GC07P080369	23.72	https://www.genecards.org/cgi-bin/carddisp.pl?gene=CD36
GC07M100620	23.68	https://www.genecards.org/cgi-bin/carddisp.pl?gene=TFR2
GC13P091422	23.6	https://www.genecards.org/cgi-bin/carddisp.pl?gene=MIR18A
GC0XM075053	23.57	https://www.genecards.org/cgi-bin/carddisp.pl?gene=ABCB7
GC04M098871	23.56	https://www.genecards.org/cgi-bin/carddisp.pl?gene=EIF4E
GC02P107969	23.55	https://www.genecards.org/cgi-bin/carddisp.pl?gene=SLC5A7
GC20M063400	23.54	https://www.genecards.org/cgi-bin/carddisp.pl?gene=KCNQ2
GC11P085628	23.54	https://www.genecards.org/cgi-bin/carddisp.pl?gene=TMEM126B
GC05M140640	23.51	https://www.genecards.org/cgi-bin/carddisp.pl?gene=NDUFA2
GC17M064477	23.5	https://www.genecards.org/cgi-bin/carddisp.pl?gene=POLG2
GC05M044340	23.49	https://www.genecards.org/cgi-bin/carddisp.pl?gene=FGF10
GC09P128504	23.48	https://www.genecards.org/cgi-bin/carddisp.pl?gene=GLE1
GC12P110280	23.47	https://www.genecards.org/cgi-bin/carddisp.pl?gene=ATP2A2
GC01P028812	23.47	https://www.genecards.org/cgi-bin/carddisp.pl?gene=OPRD1
GC12P057460	23.45	https://www.genecards.org/cgi-bin/carddisp.pl?gene=GLI1
GC12M094898	23.4	https://www.genecards.org/cgi-bin/carddisp.pl?gene=NDUFA12
GC09M133351	23.37	https://www.genecards.org/cgi-bin/carddisp.pl?gene=SURF1
GC20P008061	23.33	https://www.genecards.org/cgi-bin/carddisp.pl?gene=PLCB1
GC15P042359	23.33	https://www.genecards.org/cgi-bin/carddisp.pl?gene=CAPN3
GC14M056799	23.32	https://www.genecards.org/cgi-bin/carddisp.pl?gene=OTX2
GC10P049610	23.31	https://www.genecards.org/cgi-bin/carddisp.pl?gene=SLC18A3
GC02P026033	23.27	https://www.genecards.org/cgi-bin/carddisp.pl?gene=RAB10
GC06M032180	23.25	https://www.genecards.org/cgi-bin/carddisp.pl?gene=AGER
GC02P218658	23.24	https://www.genecards.org/cgi-bin/carddisp.pl?gene=BCS1L
GC01M019975	23.22	https://www.genecards.org/cgi-bin/carddisp.pl?gene=PLA2G2A
GC13M023328	23.2	https://www.genecards.org/cgi-bin/carddisp.pl?gene=SACS
GC12M093406	23.2	https://www.genecards.org/cgi-bin/carddisp.pl?gene=UBE2N
GC01P237042	23.19	https://www.genecards.org/cgi-bin/carddisp.pl?gene=RYR2
GC20M023026	23.18	https://www.genecards.org/cgi-bin/carddisp.pl?gene=THBD
GC15M025333	23.16	https://www.genecards.org/cgi-bin/carddisp.pl?gene=UBE3A
GC20M062214	23.15	https://www.genecards.org/cgi-bin/carddisp.pl?gene=HRH3
GC02P134838	23.14	https://www.genecards.org/cgi-bin/carddisp.pl?gene=ACMSD
GC03M169083	23.12	https://www.genecards.org/cgi-bin/carddisp.pl?gene=MECOM
GC07M092604	23.09	https://www.genecards.org/cgi-bin/carddisp.pl?gene=CDK6
GC01M203181	23.08	https://www.genecards.org/cgi-bin/carddisp.pl?gene=CHIT1
GC16M067389	23.05	https://www.genecards.org/cgi-bin/carddisp.pl?gene=TPPP3
GC05P148825	23.02	https://www.genecards.org/cgi-bin/carddisp.pl?gene=ADRB2
GC10P069269	23.01	https://www.genecards.org/cgi-bin/carddisp.pl?gene=HK1
GC02M190964	22.98	https://www.genecards.org/cgi-bin/carddisp.pl?gene=STAT1
GC17P075515	22.96	https://www.genecards.org/cgi-bin/carddisp.pl?gene=TSEN54
GC06M165327	22.85	https://www.genecards.org/cgi-bin/carddisp.pl?gene=PDE10A

GC05M159314	22.85	https://www.genecards.org/cgi-bin/carddisp.pl?gene=IL12B
GC19P010718	22.84	https://www.genecards.org/cgi-bin/carddisp.pl?gene=DNM2
GC01M011026	22.84	https://www.genecards.org/cgi-bin/carddisp.pl?gene=MASP2
GC08M002953	22.84	https://www.genecards.org/cgi-bin/carddisp.pl?gene=CSMD1
GC16M075628	22.83	https://www.genecards.org/cgi-bin/carddisp.pl?gene=KARS1
GC06M031857	22.83	https://www.genecards.org/cgi-bin/carddisp.pl?gene=NEU1
GC10P067884	22.82	https://www.genecards.org/cgi-bin/carddisp.pl?gene=SIRT1
GC07M149472	22.79	https://www.genecards.org/cgi-bin/carddisp.pl?gene=ZNF746
GC12P006913	22.78	https://www.genecards.org/cgi-bin/carddisp.pl?gene=ENO2
GC02M010432	22.75	https://www.genecards.org/cgi-bin/carddisp.pl?gene=ODC1
GC01P001001	22.74	https://www.genecards.org/cgi-bin/carddisp.pl?gene=ISG15
GC12M049127	22.73	https://www.genecards.org/cgi-bin/carddisp.pl?gene=TUBA1B
GC05P096525	22.72	https://www.genecards.org/cgi-bin/carddisp.pl?gene=CAST
GC04P076306	22.72	https://www.genecards.org/cgi-bin/carddisp.pl?gene=STBD1
GC02M240713	22.71	https://www.genecards.org/cgi-bin/carddisp.pl?gene=KIF1A
GC05M080626	22.71	https://www.genecards.org/cgi-bin/carddisp.pl?gene=DHFR
GC11P077102	22.69	https://www.genecards.org/cgi-bin/carddisp.pl?gene=OMP
GC02M009583	22.68	https://www.genecards.org/cgi-bin/carddisp.pl?gene=YWHAQ
GC12P049041	22.67	https://www.genecards.org/cgi-bin/carddisp.pl?gene=WNT1
GC05M177511	22.66	https://www.genecards.org/cgi-bin/carddisp.pl?gene=DDX41
GC01M205976	22.65	https://www.genecards.org/cgi-bin/carddisp.pl?gene=RAB7B
GC01P051236	22.65	https://www.genecards.org/cgi-bin/carddisp.pl?gene=RNF11
GC09M034366	22.64	https://www.genecards.org/cgi-bin/carddisp.pl?gene=MYORG
GC07M101239	22.62	https://www.genecards.org/cgi-bin/carddisp.pl?gene=FIS1
GC05M146582	22.6	https://www.genecards.org/cgi-bin/carddisp.pl?gene=PPP2R2B
GC03M042274	22.58	https://www.genecards.org/cgi-bin/carddisp.pl?gene=CCK
GC19M049861	22.56	https://www.genecards.org/cgi-bin/carddisp.pl?gene=PNKP
GC02P073926	22.53	https://www.genecards.org/cgi-bin/carddisp.pl?gene=DGUOK
GC22P036253	22.52	https://www.genecards.org/cgi-bin/carddisp.pl?gene=APOL1
GC01M230702	22.51	https://www.genecards.org/cgi-bin/carddisp.pl?gene=AGT
GC16M088643	22.49	https://www.genecards.org/cgi-bin/carddisp.pl?gene=CYBA
GC03P030623	22.48	https://www.genecards.org/cgi-bin/carddisp.pl?gene=TGFBR2
GC13M050049	22.46	https://www.genecards.org/cgi-bin/carddisp.pl?gene=MIR15A
GC09P128191	22.44	https://www.genecards.org/cgi-bin/carddisp.pl?gene=DNM1
GC07P002242	22.41	https://www.genecards.org/cgi-bin/carddisp.pl?gene=NUDT1
GC16M003652	22.4	https://www.genecards.org/cgi-bin/carddisp.pl?gene=TRAP1
GC02M025228	22.34	https://www.genecards.org/cgi-bin/carddisp.pl?gene=DNMT3A
GC20P013786	22.32	https://www.genecards.org/cgi-bin/carddisp.pl?gene=NDUFAF5
GC14P075278	22.31	https://www.genecards.org/cgi-bin/carddisp.pl?gene=FOS
GC19P048552	22.31	https://www.genecards.org/cgi-bin/carddisp.pl?gene=SULT2B1
GC08M056436	22.24	https://www.genecards.org/cgi-bin/carddisp.pl?gene=PENK
GC01P003652	22.24	https://www.genecards.org/cgi-bin/carddisp.pl?gene=TP73
GC10P093757	22.23	https://www.genecards.org/cgi-bin/carddisp.pl?gene=LGI1
GC06M134960	22.21	https://www.genecards.org/cgi-bin/carddisp.pl?gene=HBS1L
GC14P090396	22.19	https://www.genecards.org/cgi-bin/carddisp.pl?gene=CALM1
GC12M110910	22.18	https://www.genecards.org/cgi-bin/carddisp.pl?gene=MYL2
GC19M006677	22.16	https://www.genecards.org/cgi-bin/carddisp.pl?gene=C3

GC22P032800	22.15	https://www.genecards.org/cgi-bin/carddisp.pl?gene=TIMP3
GC09M035726	22.15	https://www.genecards.org/cgi-bin/carddisp.pl?gene=GBA2
GC09M027940	22.14	https://www.genecards.org/cgi-bin/carddisp.pl?gene=LINGO2
GC17M045799	22.12	https://www.genecards.org/cgi-bin/carddisp.pl?gene=MAPT-AS1
GC21M043772	22.07	https://www.genecards.org/cgi-bin/carddisp.pl?gene=CSTB
GC14P050532	22.06	https://www.genecards.org/cgi-bin/carddisp.pl?gene=ATL1
GC12P108859	22.04	https://www.genecards.org/cgi-bin/carddisp.pl?gene=DAO
GC11P075562	22.04	https://www.genecards.org/cgi-bin/carddisp.pl?gene=SERPINH1
GC12P049950	22.02	https://www.genecards.org/cgi-bin/carddisp.pl?gene=AQP2
GC12M110491	22.01	https://www.genecards.org/cgi-bin/carddisp.pl?gene=VPS29
GC02M038981	22.01	https://www.genecards.org/cgi-bin/carddisp.pl?gene=SOS1
GC13M030456	22	https://www.genecards.org/cgi-bin/carddisp.pl?gene=HMGB1
GC03M055465	21.96	https://www.genecards.org/cgi-bin/carddisp.pl?gene=WNT5A
GC18P041955	21.96	https://www.genecards.org/cgi-bin/carddisp.pl?gene=PIK3C3
GC13P076990	21.93	https://www.genecards.org/cgi-bin/carddisp.pl?gene=CLN5
GC02M222199	21.93	https://www.genecards.org/cgi-bin/carddisp.pl?gene=PAX3
GC0XP018425	21.93	https://www.genecards.org/cgi-bin/carddisp.pl?gene=CDKL5
GC07M083955	21.93	https://www.genecards.org/cgi-bin/carddisp.pl?gene=SEMA3A
GC11M000622	21.88	https://www.genecards.org/cgi-bin/carddisp.pl?gene=MIR210
GC01M050439	21.87	https://www.genecards.org/cgi-bin/carddisp.pl?gene=FAF1
GC03P148991	21.86	https://www.genecards.org/cgi-bin/carddisp.pl?gene=GYG1
GC0XP153902	21.86	https://www.genecards.org/cgi-bin/carddisp.pl?gene=AVPR2
GC02M086213	21.85	https://www.genecards.org/cgi-bin/carddisp.pl?gene=REEP1
GC02M127048	21.84	https://www.genecards.org/cgi-bin/carddisp.pl?gene=BIN1
GC01M055066	21.84	https://www.genecards.org/cgi-bin/carddisp.pl?gene=USP24
GC0XP077925	21.84	https://www.genecards.org/cgi-bin/carddisp.pl?gene=PGK1
GC03P048466	21.81	https://www.genecards.org/cgi-bin/carddisp.pl?gene=TREX1
GC03P023221	21.78	https://www.genecards.org/cgi-bin/carddisp.pl?gene=UBE2E2
GC11M005349	21.77	https://www.genecards.org/cgi-bin/carddisp.pl?gene=HBG2
GC14M077505	21.76	https://www.genecards.org/cgi-bin/carddisp.pl?gene=SPTLC2
GCMTP008368	21.75	https://www.genecards.org/cgi-bin/carddisp.pl?gene=MT-ATP8
GC05P051383	21.73	https://www.genecards.org/cgi-bin/carddisp.pl?gene=ISL1
GC19P047819	21.72	https://www.genecards.org/cgi-bin/carddisp.pl?gene=CRX
GC07P143316	21.71	https://www.genecards.org/cgi-bin/carddisp.pl?gene=CLCN1
GC03M049370	21.69	https://www.genecards.org/cgi-bin/carddisp.pl?gene=GPX1
GC02M239048	21.68	https://www.genecards.org/cgi-bin/carddisp.pl?gene=HDAC4
GC05P150358	21.64	https://www.genecards.org/cgi-bin/carddisp.pl?gene=TCOF1
GC10M065912	21.59	https://www.genecards.org/cgi-bin/carddisp.pl?gene=CTNNA3
GC17P039664	21.57	https://www.genecards.org/cgi-bin/carddisp.pl?gene=TCAP
GC17P026156	21.56	https://www.genecards.org/cgi-bin/carddisp.pl?gene=MAP2K3
GC02P137964	21.54	https://www.genecards.org/cgi-bin/carddisp.pl?gene=HNMT
GC17M058522	21.52	https://www.genecards.org/cgi-bin/carddisp.pl?gene=SEPTIN4
GC18M046081	21.51	https://www.genecards.org/cgi-bin/carddisp.pl?gene=ATP5F1A
GC11M017165	21.47	https://www.genecards.org/cgi-bin/carddisp.pl?gene=PIK3C2A
GC12P006584	21.46	https://www.genecards.org/cgi-bin/carddisp.pl?gene=KCNA1
GC09M136361	21.44	https://www.genecards.org/cgi-bin/carddisp.pl?gene=CARD9
GC03P130850	21.43	https://www.genecards.org/cgi-bin/carddisp.pl?gene=ATP2C1

GC08P001755	21.43	https://www.genecards.org/cgi-bin/carddisp.pl?gene=CLN8
GC01P218345	21.42	https://www.genecards.org/cgi-bin/carddisp.pl?gene=TGFB2
GC22P046150	21.41	https://www.genecards.org/cgi-bin/carddisp.pl?gene=PPARA
GC18P031318	21.41	https://www.genecards.org/cgi-bin/carddisp.pl?gene=DSG1
GC22P023179	21.39	https://www.genecards.org/cgi-bin/carddisp.pl?gene=BCR
GC06M073515	21.39	https://www.genecards.org/cgi-bin/carddisp.pl?gene=EEF1A1
GC22P019747	21.39	https://www.genecards.org/cgi-bin/carddisp.pl?gene=TBX1
GC05M115828	21.39	https://www.genecards.org/cgi-bin/carddisp.pl?gene=ATG12
GC09U902947	21.39	https://www.genecards.org/cgi-bin/carddisp.pl?gene=LOC109504728
GC11M008092	21.38	https://www.genecards.org/cgi-bin/carddisp.pl?gene=RIC3
GCMTP010472	21.37	https://www.genecards.org/cgi-bin/carddisp.pl?gene=MT-ND4L
GC02M165087	21.37	https://www.genecards.org/cgi-bin/carddisp.pl?gene=SCN3A
GC11P102110	21.33	https://www.genecards.org/cgi-bin/carddisp.pl?gene=YAP1
GC09M036828	21.31	https://www.genecards.org/cgi-bin/carddisp.pl?gene=PAX5
GC03P015621	21.3	https://www.genecards.org/cgi-bin/carddisp.pl?gene=BTD
GC02P015950	21.28	https://www.genecards.org/cgi-bin/carddisp.pl?gene=MYCN
GC19M007739	21.24	https://www.genecards.org/cgi-bin/carddisp.pl?gene=CD209
GC07P066628	21.23	https://www.genecards.org/cgi-bin/carddisp.pl?gene=KCTD7
GC12P122630	21.22	https://www.genecards.org/cgi-bin/carddisp.pl?gene=P2RX7
GC01M054133	21.21	https://www.genecards.org/cgi-bin/carddisp.pl?gene=CDCP2
GC05M016472	21.19	https://www.genecards.org/cgi-bin/carddisp.pl?gene=RETREG1
GC12P054337	21.19	https://www.genecards.org/cgi-bin/carddisp.pl?gene=MIR148B
GC19M006375	21.18	https://www.genecards.org/cgi-bin/carddisp.pl?gene=PSPN
GC0XM053374	21.17	https://www.genecards.org/cgi-bin/carddisp.pl?gene=SMC1A
GC19M017602	21.16	https://www.genecards.org/cgi-bin/carddisp.pl?gene=UNC13A
GC01M011846	21.16	https://www.genecards.org/cgi-bin/carddisp.pl?gene=NPPA
GC12P111766	21.14	https://www.genecards.org/cgi-bin/carddisp.pl?gene=ALDH2
GC22P019714	21.12	https://www.genecards.org/cgi-bin/carddisp.pl?gene=SEPTIN5
GC05P161847	21.1	https://www.genecards.org/cgi-bin/carddisp.pl?gene=GABRA1
GC22Mi00270	21.07	https://www.genecards.org/cgi-bin/carddisp.pl?gene=GSTT1
GC11P126269	21.04	https://www.genecards.org/cgi-bin/carddisp.pl?gene=FOXRED1
GC09P002611	21.03	https://www.genecards.org/cgi-bin/carddisp.pl?gene=VLDLR
GC22M042058	21.02	https://www.genecards.org/cgi-bin/carddisp.pl?gene=NAGA
GC19P007523	21.02	https://www.genecards.org/cgi-bin/carddisp.pl?gene=MCOLN1
GC19M005891	21.02	https://www.genecards.org/cgi-bin/carddisp.pl?gene=NDUFA11
GC03M046836	21	https://www.genecards.org/cgi-bin/carddisp.pl?gene=MYL3
GC07M100246	21	https://www.genecards.org/cgi-bin/carddisp.pl?gene=MIR93
GC10P068560	20.99	https://www.genecards.org/cgi-bin/carddisp.pl?gene=TET1
GC03M017157	20.99	https://www.genecards.org/cgi-bin/carddisp.pl?gene=TBC1D5
GC08P094895	20.97	https://www.genecards.org/cgi-bin/carddisp.pl?gene=NDUFAF6
GC03M194402	20.96	https://www.genecards.org/cgi-bin/carddisp.pl?gene=ATP13A3
GC11M063085	20.95	https://www.genecards.org/cgi-bin/carddisp.pl?gene=SNHG1
GC06M044297	20.92	https://www.genecards.org/cgi-bin/carddisp.pl?gene=AARS2
GC12P098645	20.91	https://www.genecards.org/cgi-bin/carddisp.pl?gene=APAF1
GC18M026852	20.91	https://www.genecards.org/cgi-bin/carddisp.pl?gene=AQP4
GC08P031033	20.91	https://www.genecards.org/cgi-bin/carddisp.pl?gene=WRN
GC07P153887	20.9	https://www.genecards.org/cgi-bin/carddisp.pl?gene=DPP6

GC05M060873	20.89	https://www.genecards.org/cgi-bin/carddisp.pl?gene=ERCC8
GC14P075902	20.87	https://www.genecards.org/cgi-bin/carddisp.pl?gene=IFT43
GC02P190880	20.86	https://www.genecards.org/cgi-bin/carddisp.pl?gene=GLS
GC10P129467	20.86	https://www.genecards.org/cgi-bin/carddisp.pl?gene=MGMT
GC02M233475	20.83	https://www.genecards.org/cgi-bin/carddisp.pl?gene=USP40
GC18M045800	20.83	https://www.genecards.org/cgi-bin/carddisp.pl?gene=EPG5
GC19P041398	20.82	https://www.genecards.org/cgi-bin/carddisp.pl?gene=BCKDHA
GC10M121478	20.82	https://www.genecards.org/cgi-bin/carddisp.pl?gene=FGFR2
GC14M024216	20.78	https://www.genecards.org/cgi-bin/carddisp.pl?gene=NEDD8
GC05P077180	20.76	https://www.genecards.org/cgi-bin/carddisp.pl?gene=PDE8B
GC01P001020	20.74	https://www.genecards.org/cgi-bin/carddisp.pl?gene=AGRN
GC02M088637	20.74	https://www.genecards.org/cgi-bin/carddisp.pl?gene=EIF2AK3
GC01M235109	20.74	https://www.genecards.org/cgi-bin/carddisp.pl?gene=TOMM20
GC06M158765	20.73	https://www.genecards.org/cgi-bin/carddisp.pl?gene=EZR
GC12M092770	20.73	https://www.genecards.org/cgi-bin/carddisp.pl?gene=EEA1
GC22M035606	20.72	https://www.genecards.org/cgi-bin/carddisp.pl?gene=MB
GC09P128552	20.7	https://www.genecards.org/cgi-bin/carddisp.pl?gene=SPTAN1
GC01M001785	20.68	https://www.genecards.org/cgi-bin/carddisp.pl?gene=GNB1
GC02M020560	20.67	https://www.genecards.org/cgi-bin/carddisp.pl?gene=HS1BP3
GC14M095086	20.66	https://www.genecards.org/cgi-bin/carddisp.pl?gene=DICER1
GC20P063272	20.65	https://www.genecards.org/cgi-bin/carddisp.pl?gene=ARFGAP1
GC11M044238	20.6	https://www.genecards.org/cgi-bin/carddisp.pl?gene=ALX4
GC02P073385	20.58	https://www.genecards.org/cgi-bin/carddisp.pl?gene=ALMS1
GC01M150265	20.55	https://www.genecards.org/cgi-bin/carddisp.pl?gene=APH1A
GC09M125234	20.54	https://www.genecards.org/cgi-bin/carddisp.pl?gene=HSPA5
GC03P185282	20.54	https://www.genecards.org/cgi-bin/carddisp.pl?gene=MAP3K13
GC03M046435	20.53	https://www.genecards.org/cgi-bin/carddisp.pl?gene=LTF
GC16P078099	20.53	https://www.genecards.org/cgi-bin/carddisp.pl?gene=WWOX
GC17M050183	20.52	https://www.genecards.org/cgi-bin/carddisp.pl?gene=COL1A1
GC03P049020	20.52	https://www.genecards.org/cgi-bin/carddisp.pl?gene=NDUFAF3
GC20M031664	20.51	https://www.genecards.org/cgi-bin/carddisp.pl?gene=BCL2L1
GC01M100186	20.5	https://www.genecards.org/cgi-bin/carddisp.pl?gene=DBT
GC12M013368	20.49	https://www.genecards.org/cgi-bin/carddisp.pl?gene=CLEC7A
GC01M040072	20.47	https://www.genecards.org/cgi-bin/carddisp.pl?gene=PPT1
GC06M096889	20.46	https://www.genecards.org/cgi-bin/carddisp.pl?gene=NDUFAF4
GC0XM048831	20.45	https://www.genecards.org/cgi-bin/carddisp.pl?gene=PCSK1N
GC02P208266	20.44	https://www.genecards.org/cgi-bin/carddisp.pl?gene=PIKFYVE
GC05M133975	20.44	https://www.genecards.org/cgi-bin/carddisp.pl?gene=VDAC1
GC06P160702	20.44	https://www.genecards.org/cgi-bin/carddisp.pl?gene=PLG
GC01P020633	20.4	https://www.genecards.org/cgi-bin/carddisp.pl?gene=MIR6084
GC13P091426	20.39	https://www.genecards.org/cgi-bin/carddisp.pl?gene=MIR19A
GC05M138957	20.38	https://www.genecards.org/cgi-bin/carddisp.pl?gene=SIL1
GC02P176150	20.38	https://www.genecards.org/cgi-bin/carddisp.pl?gene=MIR10B
GC03M087259	20.36	https://www.genecards.org/cgi-bin/carddisp.pl?gene=POU1F1
GC06M106045	20.35	https://www.genecards.org/cgi-bin/carddisp.pl?gene=ATG5
GC12P057476	20.35	https://www.genecards.org/cgi-bin/carddisp.pl?gene=MARS1
GC01P043933	20.35	https://www.genecards.org/cgi-bin/carddisp.pl?gene=ARTN

GC06M022230	20.33	https://www.genecards.org/cgi-bin/carddisp.pl?gene=PRL
GC09P026947	20.32	https://www.genecards.org/cgi-bin/carddisp.pl?gene=IFT74
GC03P119498	20.32	https://www.genecards.org/cgi-bin/carddisp.pl?gene=TIMMDC1
GC17P049495	20.31	https://www.genecards.org/cgi-bin/carddisp.pl?gene=NGFR
GC03P093980	20.31	https://www.genecards.org/cgi-bin/carddisp.pl?gene=ARL13B
GC02P207529	20.31	https://www.genecards.org/cgi-bin/carddisp.pl?gene=CREB1
GC04M082422	20.3	https://www.genecards.org/cgi-bin/carddisp.pl?gene=HNRNPDL
GC04M127917	20.3	https://www.genecards.org/cgi-bin/carddisp.pl?gene=MFSD8
GC06P156777	20.29	https://www.genecards.org/cgi-bin/carddisp.pl?gene=ARID1B
GC0XP048676	20.29	https://www.genecards.org/cgi-bin/carddisp.pl?gene=WAS
GC06P046078	20.28	https://www.genecards.org/cgi-bin/carddisp.pl?gene=DNAH8
GC07M129770	20.22	https://www.genecards.org/cgi-bin/carddisp.pl?gene=MIR182
GC06P033447	20.2	https://www.genecards.org/cgi-bin/carddisp.pl?gene=HLA-DPB1
GC03M010285	20.2	https://www.genecards.org/cgi-bin/carddisp.pl?gene=GHRL
GC15M051208	20.19	https://www.genecards.org/cgi-bin/carddisp.pl?gene=CYP19A1
GC12M122271	20.17	https://www.genecards.org/cgi-bin/carddisp.pl?gene=CLIP1
GC01P173824	20.17	https://www.genecards.org/cgi-bin/carddisp.pl?gene=DARS2
GC12P132710	20.16	https://www.genecards.org/cgi-bin/carddisp.pl?gene=PGAM5
GC16P071392	20.15	https://www.genecards.org/cgi-bin/carddisp.pl?gene=CALB2
GC02M024790	20.15	https://www.genecards.org/cgi-bin/carddisp.pl?gene=PTRHD1
GC19P011435	20.14	https://www.genecards.org/cgi-bin/carddisp.pl?gene=PRKCSH
GC21M044330	20.13	https://www.genecards.org/cgi-bin/carddisp.pl?gene=CFAP410
GC16P002429	20.13	https://www.genecards.org/cgi-bin/carddisp.pl?gene=CCNF
GC07P144706	20.09	https://www.genecards.org/cgi-bin/carddisp.pl?gene=PRSS1
GC14M087837	20.06	https://www.genecards.org/cgi-bin/carddisp.pl?gene=GALC
GC11M088211	20.06	https://www.genecards.org/cgi-bin/carddisp.pl?gene=CTSC
GC14P031489	20.04	https://www.genecards.org/cgi-bin/carddisp.pl?gene=NUBPL
GC01P027245	20.03	https://www.genecards.org/cgi-bin/carddisp.pl?gene=WDTC1
GC11P112226	20.03	https://www.genecards.org/cgi-bin/carddisp.pl?gene=PTS
GC21M006445	20.03	https://www.genecards.org/cgi-bin/carddisp.pl?gene=CBSL
GC0XM154348	20.01	https://www.genecards.org/cgi-bin/carddisp.pl?gene=FLNA
GC15M043234	20	https://www.genecards.org/cgi-bin/carddisp.pl?gene=TGM5
GC06P080106	20	https://www.genecards.org/cgi-bin/carddisp.pl?gene=BCKDHB
GC12P007889	19.99	https://www.genecards.org/cgi-bin/carddisp.pl?gene=PEX5
GC12M101745	19.97	https://www.genecards.org/cgi-bin/carddisp.pl?gene=GNPTAB
GC01M182350	19.95	https://www.genecards.org/cgi-bin/carddisp.pl?gene=GLUL
GC08M023006	19.92	https://www.genecards.org/cgi-bin/carddisp.pl?gene=TNFRSF10B
GC01P186798	19.91	https://www.genecards.org/cgi-bin/carddisp.pl?gene=PLA2G4A
GC02M027309	19.82	https://www.genecards.org/cgi-bin/carddisp.pl?gene=MPV17
GC14P102362	19.78	https://www.genecards.org/cgi-bin/carddisp.pl?gene=TECPR2
GC13P048233	19.78	https://www.genecards.org/cgi-bin/carddisp.pl?gene=ITM2B
GC01M145921	19.75	https://www.genecards.org/cgi-bin/carddisp.pl?gene=RBM8A
GC09M108868	19.75	https://www.genecards.org/cgi-bin/carddisp.pl?gene=ELP1
GC20M044620	19.74	https://www.genecards.org/cgi-bin/carddisp.pl?gene=ADA
GC11P018267	19.72	https://www.genecards.org/cgi-bin/carddisp.pl?gene=SAA1
GC11M065598	19.72	https://www.genecards.org/cgi-bin/carddisp.pl?gene=MAP3K11
GC11P045961	19.71	https://www.genecards.org/cgi-bin/carddisp.pl?gene=MAPK8IP1

GC07M100247	19.71	https://www.genecards.org/cgi-bin/carddisp.pl?gene=MIR106B
GC0XM134625	19.7	https://www.genecards.org/cgi-bin/carddisp.pl?gene=MIR424
GC12M124911	19.69	https://www.genecards.org/cgi-bin/carddisp.pl?gene=UBC
GC16P002475	19.69	https://www.genecards.org/cgi-bin/carddisp.pl?gene=TBC1D24
GC04M121667	19.68	https://www.genecards.org/cgi-bin/carddisp.pl?gene=ANXA5
GC10M102830	19.67	https://www.genecards.org/cgi-bin/carddisp.pl?gene=CYP17A1
GC16M000287	19.64	https://www.genecards.org/cgi-bin/carddisp.pl?gene=AXIN1
GC0XP007146	19.63	https://www.genecards.org/cgi-bin/carddisp.pl?gene=STS
GC10M106573	19.62	https://www.genecards.org/cgi-bin/carddisp.pl?gene=SORCS1
GC09P134641	19.61	https://www.genecards.org/cgi-bin/carddisp.pl?gene=COL5A1
GC11P124739	19.59	https://www.genecards.org/cgi-bin/carddisp.pl?gene=NRGN
GC09M000016	19.59	https://www.genecards.org/cgi-bin/carddisp.pl?gene=WASHC1
GC15M026543	19.58	https://www.genecards.org/cgi-bin/carddisp.pl?gene=GABRB3
GC04M038797	19.58	https://www.genecards.org/cgi-bin/carddisp.pl?gene=TLR1
GC03P186783	19.57	https://www.genecards.org/cgi-bin/carddisp.pl?gene=EIF4A2
GCMTM007447	19.54	https://www.genecards.org/cgi-bin/carddisp.pl?gene=MT-TS1
GC10P060772	19.51	https://www.genecards.org/cgi-bin/carddisp.pl?gene=CDK1
GC01P026706	19.51	https://www.genecards.org/cgi-bin/carddisp.pl?gene=ARID1A
GC13M108207	19.49	https://www.genecards.org/cgi-bin/carddisp.pl?gene=LIG4
GC02P120735	19.46	https://www.genecards.org/cgi-bin/carddisp.pl?gene=GLI2
GC02P233215	19.46	https://www.genecards.org/cgi-bin/carddisp.pl?gene=ATG16L1
GC05P162000	19.45	https://www.genecards.org/cgi-bin/carddisp.pl?gene=GABRG2
GC05M179110	19.44	https://www.genecards.org/cgi-bin/carddisp.pl?gene=ADAMTS2
GC11P026188	19.41	https://www.genecards.org/cgi-bin/carddisp.pl?gene=ANO3
GC19P053879	19.41	https://www.genecards.org/cgi-bin/carddisp.pl?gene=PRKCG
GC11M066848	19.39	https://www.genecards.org/cgi-bin/carddisp.pl?gene=PC
GC0XM047571	19.38	https://www.genecards.org/cgi-bin/carddisp.pl?gene=SYN1
GC01M201896	19.38	https://www.genecards.org/cgi-bin/carddisp.pl?gene=LMOD1
GC06P152750	19.37	https://www.genecards.org/cgi-bin/carddisp.pl?gene=VIP
GC10M088935	19.36	https://www.genecards.org/cgi-bin/carddisp.pl?gene=ACTA2
GC11M013492	19.35	https://www.genecards.org/cgi-bin/carddisp.pl?gene=PTH
GC0XP030671	19.29	https://www.genecards.org/cgi-bin/carddisp.pl?gene=GK
GC11P035139	19.26	https://www.genecards.org/cgi-bin/carddisp.pl?gene=CD44
GC03P167735	19.23	https://www.genecards.org/cgi-bin/carddisp.pl?gene=SERPINI1
GCMTM004331	19.22	https://www.genecards.org/cgi-bin/carddisp.pl?gene=MT-TQ
GC01M053507	19.22	https://www.genecards.org/cgi-bin/carddisp.pl?gene=GLIS1
GC03M052222	19.21	https://www.genecards.org/cgi-bin/carddisp.pl?gene=TLR9
GC15P067063	19.2	https://www.genecards.org/cgi-bin/carddisp.pl?gene=SMAD3
GC01M146018	19.17	https://www.genecards.org/cgi-bin/carddisp.pl?gene=HJV
GC01M169722	19.16	https://www.genecards.org/cgi-bin/carddisp.pl?gene=SELE
GC15P073994	19.16	https://www.genecards.org/cgi-bin/carddisp.pl?gene=PML
GC0XP019343	19.14	https://www.genecards.org/cgi-bin/carddisp.pl?gene=PDHA1
GC01P154405	19.14	https://www.genecards.org/cgi-bin/carddisp.pl?gene=IL6R
GC19P037718	19.11	https://www.genecards.org/cgi-bin/carddisp.pl?gene=COX6B1
GC06P033429	19.1	https://www.genecards.org/cgi-bin/carddisp.pl?gene=HSPA1A
GC09M032886	19.1	https://www.genecards.org/cgi-bin/carddisp.pl?gene=APTX
GC12P049293	19.09	https://www.genecards.org/cgi-bin/carddisp.pl?gene=PRPH

GC15P072474	19.08	https://www.genecards.org/cgi-bin/carddisp.pl?gene=ARIH1
GC07M095359	19.06	https://www.genecards.org/cgi-bin/carddisp.pl?gene=PON3
GC11P112961	19.05	https://www.genecards.org/cgi-bin/carddisp.pl?gene=NCAM1
GC12M013371	19.03	https://www.genecards.org/cgi-bin/carddisp.pl?gene=OLR1
GC19M015236	19.03	https://www.genecards.org/cgi-bin/carddisp.pl?gene=BRD4
GC08M008783	19.01	https://www.genecards.org/cgi-bin/carddisp.pl?gene=MFHAS1
GC09M127815	19	https://www.genecards.org/cgi-bin/carddisp.pl?gene=ENG
GC19P035030	18.98	https://www.genecards.org/cgi-bin/carddisp.pl?gene=SCN1B
GC17M063917	18.98	https://www.genecards.org/cgi-bin/carddisp.pl?gene=GH1
GC09P069121	18.97	https://www.genecards.org/cgi-bin/carddisp.pl?gene=TJP2
GC22M042796	18.97	https://www.genecards.org/cgi-bin/carddisp.pl?gene=ARFGAP3
GC01P154540	18.95	https://www.genecards.org/cgi-bin/carddisp.pl?gene=CHRNB2
GC0XM025021	18.94	https://www.genecards.org/cgi-bin/carddisp.pl?gene=ARX
GC02P218781	18.92	https://www.genecards.org/cgi-bin/carddisp.pl?gene=CYP27A1
GC09P128684	18.92	https://www.genecards.org/cgi-bin/carddisp.pl?gene=SET
GC07P016916	18.91	https://www.genecards.org/cgi-bin/carddisp.pl?gene=AHR
GC07P157138	18.91	https://www.genecards.org/cgi-bin/carddisp.pl?gene=UBE3C
GC09M110243	18.9	https://www.genecards.org/cgi-bin/carddisp.pl?gene=TXN
GC11M066684	18.89	https://www.genecards.org/cgi-bin/carddisp.pl?gene=SPTBN2
GC19M043543	18.89	https://www.genecards.org/cgi-bin/carddisp.pl?gene=XRCC1
GC04P007196	18.88	https://www.genecards.org/cgi-bin/carddisp.pl?gene=SORCS2
GC11P116829	18.87	https://www.genecards.org/cgi-bin/carddisp.pl?gene=APOC3
GC09P021478	18.85	https://www.genecards.org/cgi-bin/carddisp.pl?gene=IFNA1
GC22P023894	18.85	https://www.genecards.org/cgi-bin/carddisp.pl?gene=MIF
GC15P064094	18.85	https://www.genecards.org/cgi-bin/carddisp.pl?gene=SNX1
GC09P000214	18.84	https://www.genecards.org/cgi-bin/carddisp.pl?gene=DOCK8
GC17P045784	18.84	https://www.genecards.org/cgi-bin/carddisp.pl?gene=CRHR1
GC06M046704	18.82	https://www.genecards.org/cgi-bin/carddisp.pl?gene=PLA2G7
GC03M155821	18.82	https://www.genecards.org/cgi-bin/carddisp.pl?gene=SLC33A1
GC12P119178	18.8	https://www.genecards.org/cgi-bin/carddisp.pl?gene=HSPB8
GC10M013277	18.8	https://www.genecards.org/cgi-bin/carddisp.pl?gene=PHYH
GC05P072107	18.79	https://www.genecards.org/cgi-bin/carddisp.pl?gene=MAP1B
GC19P009835	18.79	https://www.genecards.org/cgi-bin/carddisp.pl?gene=PIN1
GC20P002960	18.78	https://www.genecards.org/cgi-bin/carddisp.pl?gene=NOP56
GC04M124664	18.77	https://www.genecards.org/cgi-bin/carddisp.pl?gene=ANKRD50
GC03P036993	18.75	https://www.genecards.org/cgi-bin/carddisp.pl?gene=MLH1
GC01P198607	18.75	https://www.genecards.org/cgi-bin/carddisp.pl?gene=PTPRC
GC03P186717	18.74	https://www.genecards.org/cgi-bin/carddisp.pl?gene=KNG1
GCMTP012215	18.73	https://www.genecards.org/cgi-bin/carddisp.pl?gene=MT-TS2
GC01P228165	18.72	https://www.genecards.org/cgi-bin/carddisp.pl?gene=IBA57
GC02M162142	18.71	https://www.genecards.org/cgi-bin/carddisp.pl?gene=GCG
GC0XP129539	18.71	https://www.genecards.org/cgi-bin/carddisp.pl?gene=OCRL
GC01P156242	18.71	https://www.genecards.org/cgi-bin/carddisp.pl?gene=BGLAP
GC05M000659	18.71	https://www.genecards.org/cgi-bin/carddisp.pl?gene=TPPP
GC11P068038	18.7	https://www.genecards.org/cgi-bin/carddisp.pl?gene=TCIRG1
GC09M136403	18.7	https://www.genecards.org/cgi-bin/carddisp.pl?gene=ENTR1
GC19M055262	18.7	https://www.genecards.org/cgi-bin/carddisp.pl?gene=HSPBP1

GC04P158672	18.67	https://www.genecards.org/cgi-bin/carddisp.pl?gene=ETFDH
GC03P045405	18.66	https://www.genecards.org/cgi-bin/carddisp.pl?gene=LARS2
GC14M034561	18.64	https://www.genecards.org/cgi-bin/carddisp.pl?gene=SNX6
GC08M063048	18.62	https://www.genecards.org/cgi-bin/carddisp.pl?gene=TTPA
GC12P120822	18.62	https://www.genecards.org/cgi-bin/carddisp.pl?gene=ACADS
GC01P100719	18.62	https://www.genecards.org/cgi-bin/carddisp.pl?gene=VCAM1
GC12M032725	18.61	https://www.genecards.org/cgi-bin/carddisp.pl?gene=YARS2
GC07M144451	18.6	https://www.genecards.org/cgi-bin/carddisp.pl?gene=TPK1
GC15M042738	18.6	https://www.genecards.org/cgi-bin/carddisp.pl?gene=TTBK2
GC09P122370	18.59	https://www.genecards.org/cgi-bin/carddisp.pl?gene=PTGS1
GC03P038179	18.57	https://www.genecards.org/cgi-bin/carddisp.pl?gene=MYD88
GC15M076215	18.56	https://www.genecards.org/cgi-bin/carddisp.pl?gene=ETFA
GC11M101356	18.56	https://www.genecards.org/cgi-bin/carddisp.pl?gene=TRPC6
GC22M030576	18.55	https://www.genecards.org/cgi-bin/carddisp.pl?gene=PES1
GC20P031514	18.54	https://www.genecards.org/cgi-bin/carddisp.pl?gene=HM13
GC19M010486	18.53	https://www.genecards.org/cgi-bin/carddisp.pl?gene=KEAP1
GC22P041469	18.53	https://www.genecards.org/cgi-bin/carddisp.pl?gene=ACO2
GC01M114673	18.53	https://www.genecards.org/cgi-bin/carddisp.pl?gene=AMPD1
GC12M121276	18.5	https://www.genecards.org/cgi-bin/carddisp.pl?gene=CAMKK2
GC17M081887	18.49	https://www.genecards.org/cgi-bin/carddisp.pl?gene=ALYREF
GC01P022710	18.49	https://www.genecards.org/cgi-bin/carddisp.pl?gene=EPHB2
GC03M164978	18.48	https://www.genecards.org/cgi-bin/carddisp.pl?gene=SI
GC09M083659	18.48	https://www.genecards.org/cgi-bin/carddisp.pl?gene=UBQLN1
GC09P136410	18.46	https://www.genecards.org/cgi-bin/carddisp.pl?gene=PMPCA
GC01P206203	18.45	https://www.genecards.org/cgi-bin/carddisp.pl?gene=SRGAP2
GC14P020455	18.44	https://www.genecards.org/cgi-bin/carddisp.pl?gene=APEX1
GC20M017970	18.43	https://www.genecards.org/cgi-bin/carddisp.pl?gene=SNX5
GC14P074881	18.41	https://www.genecards.org/cgi-bin/carddisp.pl?gene=DLST
GC09M021077	18.35	https://www.genecards.org/cgi-bin/carddisp.pl?gene=IFNB1
GC15P071792	18.33	https://www.genecards.org/cgi-bin/carddisp.pl?gene=NR2E3
GC04M079901	18.33	https://www.genecards.org/cgi-bin/carddisp.pl?gene=ANTXR2
GC22M050523	18.31	https://www.genecards.org/cgi-bin/carddisp.pl?gene=SCO2
GC13M101710	18.31	https://www.genecards.org/cgi-bin/carddisp.pl?gene=FGF14
GC06P143450	18.28	https://www.genecards.org/cgi-bin/carddisp.pl?gene=PEX3
GC17P004932	18.27	https://www.genecards.org/cgi-bin/carddisp.pl?gene=GP1BA
GC06M032840	18.25	https://www.genecards.org/cgi-bin/carddisp.pl?gene=PSMB8
GC12M042456	18.22	https://www.genecards.org/cgi-bin/carddisp.pl?gene=PRICKLE1
GC02M110122	18.22	https://www.genecards.org/cgi-bin/carddisp.pl?gene=NPHP1
GC19M004090	18.2	https://www.genecards.org/cgi-bin/carddisp.pl?gene=MAP2K2
GC02P178450	18.2	https://www.genecards.org/cgi-bin/carddisp.pl?gene=PJVK
GC09M037772	18.2	https://www.genecards.org/cgi-bin/carddisp.pl?gene=EXOSC3
GC04P189661	18.2	https://www.genecards.org/cgi-bin/carddisp.pl?gene=LINC01262
GC03M038862	18.19	https://www.genecards.org/cgi-bin/carddisp.pl?gene=SCN11A
GC12P109097	18.16	https://www.genecards.org/cgi-bin/carddisp.pl?gene=UNG
GC06P007726	18.14	https://www.genecards.org/cgi-bin/carddisp.pl?gene=BMP6
GC19P044914	18.13	https://www.genecards.org/cgi-bin/carddisp.pl?gene=APOC1
GCMTP012140	18.13	https://www.genecards.org/cgi-bin/carddisp.pl?gene=MT-TH

GC11P064317	18.13	https://www.genecards.org/cgi-bin/carddisp.pl?gene=PRDX5
GC08P037736	18.11	https://www.genecards.org/cgi-bin/carddisp.pl?gene=ERLIN2
GC10P026216	18.09	https://www.genecards.org/cgi-bin/carddisp.pl?gene=GAD2
GC09M077716	18.07	https://www.genecards.org/cgi-bin/carddisp.pl?gene=GNAQ
GC11M072292	18.05	https://www.genecards.org/cgi-bin/carddisp.pl?gene=CLPB
GC21P025573	18.05	https://www.genecards.org/cgi-bin/carddisp.pl?gene=MIR155
GC19M050314	18.03	https://www.genecards.org/cgi-bin/carddisp.pl?gene=KCNC3
GC20P009024	18.02	https://www.genecards.org/cgi-bin/carddisp.pl?gene=PLCB4
GC17M065528	18.01	https://www.genecards.org/cgi-bin/carddisp.pl?gene=AXIN2
GC16M070688	18.01	https://www.genecards.org/cgi-bin/carddisp.pl?gene=VAC14
GC11P111913	18	https://www.genecards.org/cgi-bin/carddisp.pl?gene=HSPB2
GC02M130073	17.99	https://www.genecards.org/cgi-bin/carddisp.pl?gene=POTEF
GC07P151877	17.97	https://www.genecards.org/cgi-bin/carddisp.pl?gene=PRKAG2-AS1
GC0XP071534	17.96	https://www.genecards.org/cgi-bin/carddisp.pl?gene=OGT
GC03P193594	17.94	https://www.genecards.org/cgi-bin/carddisp.pl?gene=OPA1
GC01M006466	17.9	https://www.genecards.org/cgi-bin/carddisp.pl?gene=PLEKHG5
GC09M136196	17.89	https://www.genecards.org/cgi-bin/carddisp.pl?gene=LHX3
GCMTP000580	17.89	https://www.genecards.org/cgi-bin/carddisp.pl?gene=MT-TF
GC05P122774	17.88	https://www.genecards.org/cgi-bin/carddisp.pl?gene=SNX2
GC17P014069	17.88	https://www.genecards.org/cgi-bin/carddisp.pl?gene=COX10
GC13M036302	17.87	https://www.genecards.org/cgi-bin/carddisp.pl?gene=SPART
GC11P000749	17.84	https://www.genecards.org/cgi-bin/carddisp.pl?gene=TALDO1
GC19M015159	17.83	https://www.genecards.org/cgi-bin/carddisp.pl?gene=NOTCH3
GC10M046033	17.82	https://www.genecards.org/cgi-bin/carddisp.pl?gene=MSMB
GC04M109740	17.78	https://www.genecards.org/cgi-bin/carddisp.pl?gene=CFI
GC13M028300	17.76	https://www.genecards.org/cgi-bin/carddisp.pl?gene=FLT1
GC01M039659	17.76	https://www.genecards.org/cgi-bin/carddisp.pl?gene=NT5C1A
GC09P135702	17.75	https://www.genecards.org/cgi-bin/carddisp.pl?gene=KCNT1
GC07P107890	17.72	https://www.genecards.org/cgi-bin/carddisp.pl?gene=DLD
GC03P049150	17.71	https://www.genecards.org/cgi-bin/carddisp.pl?gene=P4HTM
GC11P128686	17.71	https://www.genecards.org/cgi-bin/carddisp.pl?gene=FLI1
GC08M016107	17.67	https://www.genecards.org/cgi-bin/carddisp.pl?gene=MSR1
GC20P002380	17.66	https://www.genecards.org/cgi-bin/carddisp.pl?gene=TGM6
GC0XM040049	17.66	https://www.genecards.org/cgi-bin/carddisp.pl?gene=BCOR
GC03M171600	17.63	https://www.genecards.org/cgi-bin/carddisp.pl?gene=PLD1
GC21M044885	17.63	https://www.genecards.org/cgi-bin/carddisp.pl?gene=ITGB2
GC03P158644	17.63	https://www.genecards.org/cgi-bin/carddisp.pl?gene=GFM1
GC19P022252	17.63	https://www.genecards.org/cgi-bin/carddisp.pl?gene=GDF15
GC11P134224	17.62	https://www.genecards.org/cgi-bin/carddisp.pl?gene=VPS26B
GC01M008861	17.62	https://www.genecards.org/cgi-bin/carddisp.pl?gene=ENO1
GC09P076691	17.6	https://www.genecards.org/cgi-bin/carddisp.pl?gene=PCA3
GC02M182833	17.6	https://www.genecards.org/cgi-bin/carddisp.pl?gene=FRZB
GC12M007110	17.59	https://www.genecards.org/cgi-bin/carddisp.pl?gene=C1R
GC10P119651	17.58	https://www.genecards.org/cgi-bin/carddisp.pl?gene=BAG3
GC11P017741	17.58	https://www.genecards.org/cgi-bin/carddisp.pl?gene=MYOD1
GC03M098576	17.57	https://www.genecards.org/cgi-bin/carddisp.pl?gene=CPOX
GC12P108561	17.57	https://www.genecards.org/cgi-bin/carddisp.pl?gene=ISCU

GC15M089608	17.55	https://www.genecards.org/cgi-bin/carddisp.pl?gene=KIF7
GC04M055078	17.53	https://www.genecards.org/cgi-bin/carddisp.pl?gene=KDR
GC0XP154541	17.53	https://www.genecards.org/cgi-bin/carddisp.pl?gene=IKBKG
GC05P007851	17.53	https://www.genecards.org/cgi-bin/carddisp.pl?gene=MTRR
GC04M094298	17.49	https://www.genecards.org/cgi-bin/carddisp.pl?gene=HPGDS
GC10P133520	17.47	https://www.genecards.org/cgi-bin/carddisp.pl?gene=CYP2E1
GC19M048615	17.45	https://www.genecards.org/cgi-bin/carddisp.pl?gene=RPL18
GC11M000791	17.45	https://www.genecards.org/cgi-bin/carddisp.pl?gene=SLC25A22
GC17M042810	17.42	https://www.genecards.org/cgi-bin/carddisp.pl?gene=BECN1
GC17P007572	17.41	https://www.genecards.org/cgi-bin/carddisp.pl?gene=EIF4A1
GC14P031025	17.41	https://www.genecards.org/cgi-bin/carddisp.pl?gene=AP4S1
GC17M049202	17.41	https://www.genecards.org/cgi-bin/carddisp.pl?gene=GNGT2
GC02M074461	17.4	https://www.genecards.org/cgi-bin/carddisp.pl?gene=MOGS
GC01P160085	17.39	https://www.genecards.org/cgi-bin/carddisp.pl?gene=ATP1A2
GC02P238848	17.39	https://www.genecards.org/cgi-bin/carddisp.pl?gene=TWIST2
GC01P226899	17.38	https://www.genecards.org/cgi-bin/carddisp.pl?gene=COQ8A
GC11P119067	17.37	https://www.genecards.org/cgi-bin/carddisp.pl?gene=VPS11
GC01M156668	17.37	https://www.genecards.org/cgi-bin/carddisp.pl?gene=NES
GC08M078689	17.36	https://www.genecards.org/cgi-bin/carddisp.pl?gene=IL7
GC02M026190	17.35	https://www.genecards.org/cgi-bin/carddisp.pl?gene=HADHA
GC05P175659	17.33	https://www.genecards.org/cgi-bin/carddisp.pl?gene=HRH2
GC08M144529	17.32	https://www.genecards.org/cgi-bin/carddisp.pl?gene=ARHGAP39
GC05M070968	17.32	https://www.genecards.org/cgi-bin/carddisp.pl?gene=NAIP
GC07M100679	17.32	https://www.genecards.org/cgi-bin/carddisp.pl?gene=GIGYF1
GC17P058692	17.32	https://www.genecards.org/cgi-bin/carddisp.pl?gene=RAD51C
GC16M015704	17.31	https://www.genecards.org/cgi-bin/carddisp.pl?gene=MYH11
GC07P004775	17.31	https://www.genecards.org/cgi-bin/carddisp.pl?gene=AP5Z1
GC16P001153	17.3	https://www.genecards.org/cgi-bin/carddisp.pl?gene=CACNA1H
GC06M160531	17.28	https://www.genecards.org/cgi-bin/carddisp.pl?gene=LPA
GC17M075165	17.28	https://www.genecards.org/cgi-bin/carddisp.pl?gene=SUMO2
GC14P055124	17.27	https://www.genecards.org/cgi-bin/carddisp.pl?gene=LGALS3
GC22P039901	17.25	https://www.genecards.org/cgi-bin/carddisp.pl?gene=GRAP2
GC17M007223	17.24	https://www.genecards.org/cgi-bin/carddisp.pl?gene=MIR324
GC17M082239	17.23	https://www.genecards.org/cgi-bin/carddisp.pl?gene=CSNK1D
GC17P035713	17.23	https://www.genecards.org/cgi-bin/carddisp.pl?gene=TAF15
GCMTP015890	17.23	https://www.genecards.org/cgi-bin/carddisp.pl?gene=MT-TT
GC10M089579	17.21	https://www.genecards.org/cgi-bin/carddisp.pl?gene=PANK1
GC01M053243	17.2	https://www.genecards.org/cgi-bin/carddisp.pl?gene=LRP8
GC11P125625	17.2	https://www.genecards.org/cgi-bin/carddisp.pl?gene=CHEK1
GC01M151375	17.18	https://www.genecards.org/cgi-bin/carddisp.pl?gene=POGZ
GC14M070722	17.16	https://www.genecards.org/cgi-bin/carddisp.pl?gene=MAP3K9
GC05M040759	17.15	https://www.genecards.org/cgi-bin/carddisp.pl?gene=PRKAA1
GC0XP068828	17.13	https://www.genecards.org/cgi-bin/carddisp.pl?gene=EFNB1
GC09M113386	17.13	https://www.genecards.org/cgi-bin/carddisp.pl?gene=ALAD
GC19P012938	17.11	https://www.genecards.org/cgi-bin/carddisp.pl?gene=CALR
GC02P102136	17.09	https://www.genecards.org/cgi-bin/carddisp.pl?gene=IL1R1
GC02M096112	17.08	https://www.genecards.org/cgi-bin/carddisp.pl?gene=ADRA2B

GC02M232878	17.07	https://www.genecards.org/cgi-bin/carddisp.pl?gene=NGEF
GC01M113894	17.07	https://www.genecards.org/cgi-bin/carddisp.pl?gene=AP4B1
GC14U902087	17.05	https://www.genecards.org/cgi-bin/carddisp.pl?gene=LOC108663987
GC11M066612	17.04	https://www.genecards.org/cgi-bin/carddisp.pl?gene=CTSF
GC07P100101	17.03	https://www.genecards.org/cgi-bin/carddisp.pl?gene=AP4M1
GC03M012583	17	https://www.genecards.org/cgi-bin/carddisp.pl?gene=RAF1
GC15M078925	17	https://www.genecards.org/cgi-bin/carddisp.pl?gene=CTSH
GC08M134800	17	https://www.genecards.org/cgi-bin/carddisp.pl?gene=MIR30B
GC02P017539	16.99	https://www.genecards.org/cgi-bin/carddisp.pl?gene=VSNL1
GC01M016514	16.99	https://www.genecards.org/cgi-bin/carddisp.pl?gene=RNU1-1
GC20P033812	16.98	https://www.genecards.org/cgi-bin/carddisp.pl?gene=CHMP4B
GC0XP074423	16.97	https://www.genecards.org/cgi-bin/carddisp.pl?gene=SLC16A2
GC12M102013	16.97	https://www.genecards.org/cgi-bin/carddisp.pl?gene=WASHC3
GC10M092451	16.95	https://www.genecards.org/cgi-bin/carddisp.pl?gene=IDE
GC10P102503	16.94	https://www.genecards.org/cgi-bin/carddisp.pl?gene=SUFU
GC0XM063634	16.93	https://www.genecards.org/cgi-bin/carddisp.pl?gene=ARHGEF9
GC0XP154411	16.93	https://www.genecards.org/cgi-bin/carddisp.pl?gene=TAZ
GC01P153262	16.92	https://www.genecards.org/cgi-bin/carddisp.pl?gene=LORICRIN
GC02M162267	16.9	https://www.genecards.org/cgi-bin/carddisp.pl?gene=IFIH1
GC20M039214	16.87	https://www.genecards.org/cgi-bin/carddisp.pl?gene=LINC01734
GC10P073995	16.86	https://www.genecards.org/cgi-bin/carddisp.pl?gene=VCL
GC11M072239	16.86	https://www.genecards.org/cgi-bin/carddisp.pl?gene=PHOX2A
GC17P075205	16.86	https://www.genecards.org/cgi-bin/carddisp.pl?gene=NUP85
GC11P067024	16.84	https://www.genecards.org/cgi-bin/carddisp.pl?gene=SYT12
GC03P127598	16.84	https://www.genecards.org/cgi-bin/carddisp.pl?gene=MCM2
GC01P055039	16.84	https://www.genecards.org/cgi-bin/carddisp.pl?gene=PCSK9
GCMTP005514	16.83	https://www.genecards.org/cgi-bin/carddisp.pl?gene=MT-TW
GC05P170861	16.83	https://www.genecards.org/cgi-bin/carddisp.pl?gene=RANBP17
GC04P112818	16.83	https://www.genecards.org/cgi-bin/carddisp.pl?gene=ANK2
GC06M085505	16.83	https://www.genecards.org/cgi-bin/carddisp.pl?gene=SNX14
GC05P132369	16.81	https://www.genecards.org/cgi-bin/carddisp.pl?gene=SLC22A5
GC09M124857	16.81	https://www.genecards.org/cgi-bin/carddisp.pl?gene=RPL35
GC06M111660	16.8	https://www.genecards.org/cgi-bin/carddisp.pl?gene=FYN
GC06M152121	16.78	https://www.genecards.org/cgi-bin/carddisp.pl?gene=SYNE1
GC20P031475	16.78	https://www.genecards.org/cgi-bin/carddisp.pl?gene=REM1
GC05M134148	16.77	https://www.genecards.org/cgi-bin/carddisp.pl?gene=SKP1
GC0XP151396	16.76	https://www.genecards.org/cgi-bin/carddisp.pl?gene=VMA21
GC10P094762	16.76	https://www.genecards.org/cgi-bin/carddisp.pl?gene=CYP2C19
GC16P019067	16.74	https://www.genecards.org/cgi-bin/carddisp.pl?gene=COQ7
GC05P126776	16.72	https://www.genecards.org/cgi-bin/carddisp.pl?gene=LMNB1
GC01M110519	16.71	https://www.genecards.org/cgi-bin/carddisp.pl?gene=KCNA2
GC19P050329	16.7	https://www.genecards.org/cgi-bin/carddisp.pl?gene=NR1H2
GC10M080150	16.7	https://www.genecards.org/cgi-bin/carddisp.pl?gene=ANXA11
GC01P151227	16.69	https://www.genecards.org/cgi-bin/carddisp.pl?gene=PSMD4
GC10P113679	16.69	https://www.genecards.org/cgi-bin/carddisp.pl?gene=CASP7
GC09P021994	16.68	https://www.genecards.org/cgi-bin/carddisp.pl?gene=CDKN2B-AS1
GC04M095162	16.68	https://www.genecards.org/cgi-bin/carddisp.pl?gene=UNC5C

GC16P005240	16.67	https://www.genecards.org/cgi-bin/carddisp.pl?gene=RBFOX1
GC17P059893	16.67	https://www.genecards.org/cgi-bin/carddisp.pl?gene=RPS6KB1
GC11M102617	16.66	https://www.genecards.org/cgi-bin/carddisp.pl?gene=MMP8
GC01P109911	16.64	https://www.genecards.org/cgi-bin/carddisp.pl?gene=CSF1
GC02M221418	16.64	https://www.genecards.org/cgi-bin/carddisp.pl?gene=EPHA4
GC0XP080014	16.62	https://www.genecards.org/cgi-bin/carddisp.pl?gene=TBX22
GC05M140631	16.6	https://www.genecards.org/cgi-bin/carddisp.pl?gene=CD14
GC06P033598	16.59	https://www.genecards.org/cgi-bin/carddisp.pl?gene=PHF1
GC22M028794	16.58	https://www.genecards.org/cgi-bin/carddisp.pl?gene=XBP1
GC17U902739	16.57	https://www.genecards.org/cgi-bin/carddisp.pl?gene=LOC110806262
GC15M074719	16.55	https://www.genecards.org/cgi-bin/carddisp.pl?gene=CYP1A1
GC14M035401	16.55	https://www.genecards.org/cgi-bin/carddisp.pl?gene=NFKBIA
GC0XP044873	16.55	https://www.genecards.org/cgi-bin/carddisp.pl?gene=KDM6A
GC02M047124	16.53	https://www.genecards.org/cgi-bin/carddisp.pl?gene=CALM2
GC04M140283	16.52	https://www.genecards.org/cgi-bin/carddisp.pl?gene=SCOC-AS1
GC08P144354	16.5	https://www.genecards.org/cgi-bin/carddisp.pl?gene=SLC52A2
GC17P060600	16.48	https://www.genecards.org/cgi-bin/carddisp.pl?gene=PPM1D
GC17M008762	16.47	https://www.genecards.org/cgi-bin/carddisp.pl?gene=VAMP2
GC10P094501	16.45	https://www.genecards.org/cgi-bin/carddisp.pl?gene=HELLS
GC18P052340	16.4	https://www.genecards.org/cgi-bin/carddisp.pl?gene=DCC
GC02P121531	16.4	https://www.genecards.org/cgi-bin/carddisp.pl?gene=RNU4ATAC
GC01P055220	16.39	https://www.genecards.org/cgi-bin/carddisp.pl?gene=MIR4422HG
GC04U901561	16.39	https://www.genecards.org/cgi-bin/carddisp.pl?gene=UCH1LAS
GC06P154075	16.39	https://www.genecards.org/cgi-bin/carddisp.pl?gene=OPRM1
GC12M056271	16.38	https://www.genecards.org/cgi-bin/carddisp.pl?gene=CS
GC01P205043	16.38	https://www.genecards.org/cgi-bin/carddisp.pl?gene=CNTN2
GC01P223701	16.37	https://www.genecards.org/cgi-bin/carddisp.pl?gene=CAPN2
GC06M137197	16.37	https://www.genecards.org/cgi-bin/carddisp.pl?gene=IFNGR1
GC08M132120	16.35	https://www.genecards.org/cgi-bin/carddisp.pl?gene=KCNQ3
GC07M103175	16.35	https://www.genecards.org/cgi-bin/carddisp.pl?gene=DPY19L2P2
GC04M041223	16.35	https://www.genecards.org/cgi-bin/carddisp.pl?gene=UCHL1-AS1
GC0XU902349	16.34	https://www.genecards.org/cgi-bin/carddisp.pl?gene=LOC108684022
GC0XP011111	16.33	https://www.genecards.org/cgi-bin/carddisp.pl?gene=HCCS
GC19M014514	16.33	https://www.genecards.org/cgi-bin/carddisp.pl?gene=DNAJB1
GC03M069156	16.32	https://www.genecards.org/cgi-bin/carddisp.pl?gene=LMOD3
GC20P063981	16.28	https://www.genecards.org/cgi-bin/carddisp.pl?gene=PRPF6
GC0XP123859	16.27	https://www.genecards.org/cgi-bin/carddisp.pl?gene=XIAP
GC01M229431	16.27	https://www.genecards.org/cgi-bin/carddisp.pl?gene=ACTA1
GC11M000767	16.26	https://www.genecards.org/cgi-bin/carddisp.pl?gene=GATD1
GC09P136670	16.25	https://www.genecards.org/cgi-bin/carddisp.pl?gene=MIR126
GC08P099011	16.25	https://www.genecards.org/cgi-bin/carddisp.pl?gene=VPS13B
GC17P046924	16.24	https://www.genecards.org/cgi-bin/carddisp.pl?gene=GOSR2
GC06P137866	16.24	https://www.genecards.org/cgi-bin/carddisp.pl?gene=TNFAIP3
GC07P106865	16.23	https://www.genecards.org/cgi-bin/carddisp.pl?gene=PIK3CG
GC06P033443	16.23	https://www.genecards.org/cgi-bin/carddisp.pl?gene=PSMB9
GC11M065653	16.21	https://www.genecards.org/cgi-bin/carddisp.pl?gene=RELA
GC08M142657	16.2	https://www.genecards.org/cgi-bin/carddisp.pl?gene=JRK

GC02M084423	16.16	https://www.genecards.org/cgi-bin/carddisp.pl?gene=SUCLG1
GC17P032486	16.16	https://www.genecards.org/cgi-bin/carddisp.pl?gene=CDK5R1
GC10M087751	16.14	https://www.genecards.org/cgi-bin/carddisp.pl?gene=ATAD1
GC05P157244	16.13	https://www.genecards.org/cgi-bin/carddisp.pl?gene=CYFIP2
GC16P088856	16.12	https://www.genecards.org/cgi-bin/carddisp.pl?gene=TRAPPC2L
GC07P076048	16.11	https://www.genecards.org/cgi-bin/carddisp.pl?gene=MDH2
GC07P018086	16.09	https://www.genecards.org/cgi-bin/carddisp.pl?gene=HDAC9
GC04P006271	16.07	https://www.genecards.org/cgi-bin/carddisp.pl?gene=WFS1
GC03P159988	16.06	https://www.genecards.org/cgi-bin/carddisp.pl?gene=IL12A
GC19M035904	16.06	https://www.genecards.org/cgi-bin/carddisp.pl?gene=TYROBP
GC05M122436	16.05	https://www.genecards.org/cgi-bin/carddisp.pl?gene=MGC32805
GC06M032866	16.04	https://www.genecards.org/cgi-bin/carddisp.pl?gene=TAP1
GC10M067796	16.04	https://www.genecards.org/cgi-bin/carddisp.pl?gene=DNAJC12
GC15P088814	16.03	https://www.genecards.org/cgi-bin/carddisp.pl?gene=ACAN
GC04P141636	16.02	https://www.genecards.org/cgi-bin/carddisp.pl?gene=IL15
GC03M049359	16.01	https://www.genecards.org/cgi-bin/carddisp.pl?gene=RHOA
GCMTP001605	15.99	https://www.genecards.org/cgi-bin/carddisp.pl?gene=MT-TV
GC19M045485	15.98	https://www.genecards.org/cgi-bin/carddisp.pl?gene=RTN2
GC22M044777	15.96	https://www.genecards.org/cgi-bin/carddisp.pl?gene=MIR659
GC11M078652	15.96	https://www.genecards.org/cgi-bin/carddisp.pl?gene=TENM4
GC12M079574	15.95	https://www.genecards.org/cgi-bin/carddisp.pl?gene=PAWR
GC16P001213	15.94	https://www.genecards.org/cgi-bin/carddisp.pl?gene=HBA2
GC11M019160	15.93	https://www.genecards.org/cgi-bin/carddisp.pl?gene=CSRP3
GC0XM130129	15.87	https://www.genecards.org/cgi-bin/carddisp.pl?gene=AIFM1
GC09M032619	15.85	https://www.genecards.org/cgi-bin/carddisp.pl?gene=TAF1L
GC01P212858	15.82	https://www.genecards.org/cgi-bin/carddisp.pl?gene=FLVCR1
GC17P067825	15.82	https://www.genecards.org/cgi-bin/carddisp.pl?gene=BPTF
GC14P066507	15.78	https://www.genecards.org/cgi-bin/carddisp.pl?gene=GPHN
GC13P052012	15.77	https://www.genecards.org/cgi-bin/carddisp.pl?gene=ALG11
GC0XM055009	15.75	https://www.genecards.org/cgi-bin/carddisp.pl?gene=ALAS2
GC10P100735	15.75	https://www.genecards.org/cgi-bin/carddisp.pl?gene=PAX2
GC06P007541	15.74	https://www.genecards.org/cgi-bin/carddisp.pl?gene=DSP
GC04M108810	15.68	https://www.genecards.org/cgi-bin/carddisp.pl?gene=COL25A1
GC01M054849	15.67	https://www.genecards.org/cgi-bin/carddisp.pl?gene=DHCR24
GC06P033174	15.66	https://www.genecards.org/cgi-bin/carddisp.pl?gene=HLA-G
GC06M032821	15.62	https://www.genecards.org/cgi-bin/carddisp.pl?gene=TAP2
GC04M076021	15.6	https://www.genecards.org/cgi-bin/carddisp.pl?gene=CXCL10
GC01P056645	15.59	https://www.genecards.org/cgi-bin/carddisp.pl?gene=PRKAA2
GC11M017364	15.59	https://www.genecards.org/cgi-bin/carddisp.pl?gene=KCNJ11
GC20M062464	15.58	https://www.genecards.org/cgi-bin/carddisp.pl?gene=GATA5
GC06M033064	15.58	https://www.genecards.org/cgi-bin/carddisp.pl?gene=HLA-DPA1
GC04M174490	15.58	https://www.genecards.org/cgi-bin/carddisp.pl?gene=HPGD
GC02P097696	15.58	https://www.genecards.org/cgi-bin/carddisp.pl?gene=ZAP70
GC14P074239	15.58	https://www.genecards.org/cgi-bin/carddisp.pl?gene=VSX2
GC01U901209	15.53	https://www.genecards.org/cgi-bin/carddisp.pl?gene=PSNP2
GC12P080402	15.53	https://www.genecards.org/cgi-bin/carddisp.pl?gene=PTPRQ
GC17P007613	15.53	https://www.genecards.org/cgi-bin/carddisp.pl?gene=SHBG

GC17M003565	15.53	https://www.genecards.org/cgi-bin/carddisp.pl?gene=TRPV1
GC09M128166	15.51	https://www.genecards.org/cgi-bin/carddisp.pl?gene=CIZ1
GC08P073972	15.51	https://www.genecards.org/cgi-bin/carddisp.pl?gene=TMEM70
GC20P003068	15.49	https://www.genecards.org/cgi-bin/carddisp.pl?gene=OXT
GC03M184346	15.48	https://www.genecards.org/cgi-bin/carddisp.pl?gene=CLCN2
GC03M195568	15.48	https://www.genecards.org/cgi-bin/carddisp.pl?gene=APOD
GC08M022245	15.47	https://www.genecards.org/cgi-bin/carddisp.pl?gene=MIR320A
GC03P050675	15.47	https://www.genecards.org/cgi-bin/carddisp.pl?gene=DOCK3
GC01M156033	15.47	https://www.genecards.org/cgi-bin/carddisp.pl?gene=UBQLN4
GC20P044355	15.46	https://www.genecards.org/cgi-bin/carddisp.pl?gene=HNF4A
GC02P135665	15.44	https://www.genecards.org/cgi-bin/carddisp.pl?gene=MIR128-1
GC06M031177	15.44	https://www.genecards.org/cgi-bin/carddisp.pl?gene=POU5F1
GC17M064319	15.43	https://www.genecards.org/cgi-bin/carddisp.pl?gene=PECAM1
GC10P122461	15.42	https://www.genecards.org/cgi-bin/carddisp.pl?gene=HTRA1
GC06P005261	15.41	https://www.genecards.org/cgi-bin/carddisp.pl?gene=FARS2
GC11P061753	15.41	https://www.genecards.org/cgi-bin/carddisp.pl?gene=MYRF
GC17M004937	15.41	https://www.genecards.org/cgi-bin/carddisp.pl?gene=SLC25A11
GC08P038208	15.4	https://www.genecards.org/cgi-bin/carddisp.pl?gene=DDHD2
GC03M045755	15.39	https://www.genecards.org/cgi-bin/carddisp.pl?gene=SLC6A20
GC17P061451	15.38	https://www.genecards.org/cgi-bin/carddisp.pl?gene=TBX4
GC10P103277	15.38	https://www.genecards.org/cgi-bin/carddisp.pl?gene=INA
GC19M029205	15.38	https://www.genecards.org/cgi-bin/carddisp.pl?gene=UQCRFS1
GC17M029696	15.36	https://www.genecards.org/cgi-bin/carddisp.pl?gene=VTN
GC02M054934	15.32	https://www.genecards.org/cgi-bin/carddisp.pl?gene=RTN4
GC01P220094	15.31	https://www.genecards.org/cgi-bin/carddisp.pl?gene=IARS2
GC02M074532	15.31	https://www.genecards.org/cgi-bin/carddisp.pl?gene=LOXL3
GC15M090229	15.31	https://www.genecards.org/cgi-bin/carddisp.pl?gene=CIB1
GC03M033013	15.3	https://www.genecards.org/cgi-bin/carddisp.pl?gene=GLB1
GC19M040447	15.27	https://www.genecards.org/cgi-bin/carddisp.pl?gene=BLVRB
GC03P188153	15.24	https://www.genecards.org/cgi-bin/carddisp.pl?gene=LPP
GC12M008951	15.23	https://www.genecards.org/cgi-bin/carddisp.pl?gene=M6PR
GC02M075010	15.23	https://www.genecards.org/cgi-bin/carddisp.pl?gene=TACR1
GC15P022773	15.22	https://www.genecards.org/cgi-bin/carddisp.pl?gene=NIPA1
GC12M013402	15.22	https://www.genecards.org/cgi-bin/carddisp.pl?gene=LRP6
GC07M045912	15.21	https://www.genecards.org/cgi-bin/carddisp.pl?gene=IGFBP3
GC19M014092	15.2	https://www.genecards.org/cgi-bin/carddisp.pl?gene=PRKACA
GC01M167809	15.2	https://www.genecards.org/cgi-bin/carddisp.pl?gene=ADCY10
GC02P156435	15.18	https://www.genecards.org/cgi-bin/carddisp.pl?gene=GPD2
GC0XM100291	15.18	https://www.genecards.org/cgi-bin/carddisp.pl?gene=PCDH19
GC09M120388	15.16	https://www.genecards.org/cgi-bin/carddisp.pl?gene=CDK5RAP2
GC10P097739	15.16	https://www.genecards.org/cgi-bin/carddisp.pl?gene=ZFYVE27
GC19M001397	15.14	https://www.genecards.org/cgi-bin/carddisp.pl?gene=GAMT
GC01M001538	15.13	https://www.genecards.org/cgi-bin/carddisp.pl?gene=TMEM240
GC0XP107628	15.12	https://www.genecards.org/cgi-bin/carddisp.pl?gene=PRPS1
GC03M123282	15.1	https://www.genecards.org/cgi-bin/carddisp.pl?gene=ADCY5
GC17P001761	15.1	https://www.genecards.org/cgi-bin/carddisp.pl?gene=SERPINF1
GC07P006380	15.09	https://www.genecards.org/cgi-bin/carddisp.pl?gene=RAC1

GC17M000508	15.09	https://www.genecards.org/cgi-bin/carddisp.pl?gene=VPS53
GC19P046601	15.09	https://www.genecards.org/cgi-bin/carddisp.pl?gene=CALM3
GC02M108894	15.08	https://www.genecards.org/cgi-bin/carddisp.pl?gene=EDAR
GCMTM015957	15.08	https://www.genecards.org/cgi-bin/carddisp.pl?gene=MT-TP
GC09P132945	15.07	https://www.genecards.org/cgi-bin/carddisp.pl?gene=GFI1B
GC12M007919	15.06	https://www.genecards.org/cgi-bin/carddisp.pl?gene=SLC2A3
GC11U900957	15.05	https://www.genecards.org/cgi-bin/carddisp.pl?gene=PSNP3
GC08P024913	15.01	https://www.genecards.org/cgi-bin/carddisp.pl?gene=NEFM
GC03P150603	15	https://www.genecards.org/cgi-bin/carddisp.pl?gene=SELENOT
GC07P030911	15	https://www.genecards.org/cgi-bin/carddisp.pl?gene=AQP1
GC04M109688	14.97	https://www.genecards.org/cgi-bin/carddisp.pl?gene=CASP6
GC19M051345	14.95	https://www.genecards.org/cgi-bin/carddisp.pl?gene=ETFB
GC22M030240	14.94	https://www.genecards.org/cgi-bin/carddisp.pl?gene=LIF
GC06P108559	14.94	https://www.genecards.org/cgi-bin/carddisp.pl?gene=FOXO3
GC13P091420	14.93	https://www.genecards.org/cgi-bin/carddisp.pl?gene=MIR92A1
GC22M028687	14.89	https://www.genecards.org/cgi-bin/carddisp.pl?gene=CHEK2
GC03P069788	14.89	https://www.genecards.org/cgi-bin/carddisp.pl?gene=MITF
GCMTP000642	14.89	https://www.genecards.org/cgi-bin/carddisp.pl?gene=MT-RNR1
GC03P039515	14.88	https://www.genecards.org/cgi-bin/carddisp.pl?gene=MOBP
GC06M018128	14.88	https://www.genecards.org/cgi-bin/carddisp.pl?gene=TPMT
GC11M118134	14.87	https://www.genecards.org/cgi-bin/carddisp.pl?gene=SCN4B
GC22P026646	14.87	https://www.genecards.org/cgi-bin/carddisp.pl?gene=MIAT
GC0XP101408	14.86	https://www.genecards.org/cgi-bin/carddisp.pl?gene=HNRNPH2
GC09M129094	14.85	https://www.genecards.org/cgi-bin/carddisp.pl?gene=CRAT
GC19P039972	14.82	https://www.genecards.org/cgi-bin/carddisp.pl?gene=PSMC4
GC08M134804	14.82	https://www.genecards.org/cgi-bin/carddisp.pl?gene=MIR30D
GC11M063073	14.8	https://www.genecards.org/cgi-bin/carddisp.pl?gene=GANAB
GC20M064047	14.79	https://www.genecards.org/cgi-bin/carddisp.pl?gene=SOX18
GC04P154564	14.79	https://www.genecards.org/cgi-bin/carddisp.pl?gene=FGB
GC06P033620	14.78	https://www.genecards.org/cgi-bin/carddisp.pl?gene=ITPR3
GC17M066212	14.76	https://www.genecards.org/cgi-bin/carddisp.pl?gene=APOH
GC0XM077504	14.74	https://www.genecards.org/cgi-bin/carddisp.pl?gene=ATRX
GC03P130392	14.73	https://www.genecards.org/cgi-bin/carddisp.pl?gene=RHO
GC13M101053	14.73	https://www.genecards.org/cgi-bin/carddisp.pl?gene=NALCN
GC16P001238	14.71	https://www.genecards.org/cgi-bin/carddisp.pl?gene=PIGQ
GC03M047033	14.68	https://www.genecards.org/cgi-bin/carddisp.pl?gene=SETD2
GC02P005704	14.66	https://www.genecards.org/cgi-bin/carddisp.pl?gene=SOX11
GC07M134442	14.65	https://www.genecards.org/cgi-bin/carddisp.pl?gene=AKR1B1
GC07P040134	14.64	https://www.genecards.org/cgi-bin/carddisp.pl?gene=SUGCT
GC15M090083	14.63	https://www.genecards.org/cgi-bin/carddisp.pl?gene=IDH2
GC02P188974	14.61	https://www.genecards.org/cgi-bin/carddisp.pl?gene=COL3A1
GC07P128830	14.61	https://www.genecards.org/cgi-bin/carddisp.pl?gene=FLNC
GC15P072846	14.61	https://www.genecards.org/cgi-bin/carddisp.pl?gene=TPM1
GC10M070882	14.6	https://www.genecards.org/cgi-bin/carddisp.pl?gene=PCBD1
GC20P004702	14.57	https://www.genecards.org/cgi-bin/carddisp.pl?gene=PRND
GC05M142555	14.56	https://www.genecards.org/cgi-bin/carddisp.pl?gene=FGF1
GC07P144746	14.55	https://www.genecards.org/cgi-bin/carddisp.pl?gene=CASP2

GC16P067311	14.53	https://www.genecards.org/cgi-bin/carddisp.pl?gene=PLEKHG4
GC05M149857	14.53	https://www.genecards.org/cgi-bin/carddisp.pl?gene=PDE6A
GC08M118131	14.53	https://www.genecards.org/cgi-bin/carddisp.pl?gene=SAMD12
GC15M060347	14.52	https://www.genecards.org/cgi-bin/carddisp.pl?gene=ANXA2
GC10M089601	14.5	https://www.genecards.org/cgi-bin/carddisp.pl?gene=MIR107
GC09P128322	14.5	https://www.genecards.org/cgi-bin/carddisp.pl?gene=COQ4
GC01P207454	14.49	https://www.genecards.org/cgi-bin/carddisp.pl?gene=CR2
GC12P071938	14.45	https://www.genecards.org/cgi-bin/carddisp.pl?gene=TPH2
GC06P031399	14.44	https://www.genecards.org/cgi-bin/carddisp.pl?gene=MICA
GC11M118163	14.44	https://www.genecards.org/cgi-bin/carddisp.pl?gene=SCN2B
GC17P047941	14.44	https://www.genecards.org/cgi-bin/carddisp.pl?gene=PNPO
GC17M008779	14.44	https://www.genecards.org/cgi-bin/carddisp.pl?gene=CTC1
GC03P053156	14.42	https://www.genecards.org/cgi-bin/carddisp.pl?gene=PRKCD
GC06P030720	14.4	https://www.genecards.org/cgi-bin/carddisp.pl?gene=TUBB
GCMTP012268	14.39	https://www.genecards.org/cgi-bin/carddisp.pl?gene=MT-TL2
GC02M144384	14.39	https://www.genecards.org/cgi-bin/carddisp.pl?gene=ZEB2
GC20P005911	14.34	https://www.genecards.org/cgi-bin/carddisp.pl?gene=CHGB
GC06M071403	14.34	https://www.genecards.org/cgi-bin/carddisp.pl?gene=MIR30A
GC12M057095	14.29	https://www.genecards.org/cgi-bin/carddisp.pl?gene=STAT6
GC0XP048801	14.29	https://www.genecards.org/cgi-bin/carddisp.pl?gene=HDAC6
GC02M037099	14.29	https://www.genecards.org/cgi-bin/carddisp.pl?gene=EIF2AK2
GC07M065960	14.26	https://www.genecards.org/cgi-bin/carddisp.pl?gene=GUSB
GC21M034787	14.24	https://www.genecards.org/cgi-bin/carddisp.pl?gene=RUNX1
GC08M143916	14.24	https://www.genecards.org/cgi-bin/carddisp.pl?gene=PLEC
GC01M150796	14.23	https://www.genecards.org/cgi-bin/carddisp.pl?gene=CTSK
GC16P002379	14.23	https://www.genecards.org/cgi-bin/carddisp.pl?gene=NDUFB10
GC09P129111	14.23	https://www.genecards.org/cgi-bin/carddisp.pl?gene=PTPA
GC0XM140782	14.22	https://www.genecards.org/cgi-bin/carddisp.pl?gene=CDR1
GC01M111770	14.19	https://www.genecards.org/cgi-bin/carddisp.pl?gene=KCND3
GC21M044768	14.19	https://www.genecards.org/cgi-bin/carddisp.pl?gene=UBE2G2
GC16P023872	14.18	https://www.genecards.org/cgi-bin/carddisp.pl?gene=PRKCB
GC06M032393	14.17	https://www.genecards.org/cgi-bin/carddisp.pl?gene=BTNL2
GC17M007018	14.15	https://www.genecards.org/cgi-bin/carddisp.pl?gene=MIR195
GC09M128244	14.15	https://www.genecards.org/cgi-bin/carddisp.pl?gene=MIR199B
GC17P066302	14.14	https://www.genecards.org/cgi-bin/carddisp.pl?gene=PRKCA
GC0XP154436	14.14	https://www.genecards.org/cgi-bin/carddisp.pl?gene=GDI1
GC16P030985	14.12	https://www.genecards.org/cgi-bin/carddisp.pl?gene=HSD3B7
GC03P011273	14.1	https://www.genecards.org/cgi-bin/carddisp.pl?gene=ATG7
GC14P025005	14.1	https://www.genecards.org/cgi-bin/carddisp.pl?gene=MMP14
GC03M193274	14.1	https://www.genecards.org/cgi-bin/carddisp.pl?gene=ATP13A5
GC05P171387	14.1	https://www.genecards.org/cgi-bin/carddisp.pl?gene=NPM1
GC07P098620	14.09	https://www.genecards.org/cgi-bin/carddisp.pl?gene=NPTX2
GC17P047522	14.07	https://www.genecards.org/cgi-bin/carddisp.pl?gene=NPEPPS
GC12P007787	14.06	https://www.genecards.org/cgi-bin/carddisp.pl?gene=NANOG
GC09P001980	14.06	https://www.genecards.org/cgi-bin/carddisp.pl?gene=SMARCA2
GC13P108251	14.05	https://www.genecards.org/cgi-bin/carddisp.pl?gene=TNFSF13B
GC14P104448	14.05	https://www.genecards.org/cgi-bin/carddisp.pl?gene=MIR376A1

GC15P080404	14.05	https://www.genecards.org/cgi-bin/carddisp.pl?gene=ARNT2
GC09P128149	14.01	https://www.genecards.org/cgi-bin/carddisp.pl?gene=LCN2
GC09P027109	13.98	https://www.genecards.org/cgi-bin/carddisp.pl?gene=TEK
GC22P018110	13.97	https://www.genecards.org/cgi-bin/carddisp.pl?gene=TUBA8
GC17M017977	13.97	https://www.genecards.org/cgi-bin/carddisp.pl?gene=ATPAF2
GC14M053036	13.96	https://www.genecards.org/cgi-bin/carddisp.pl?gene=DDHD1
GC12P122635	13.96	https://www.genecards.org/cgi-bin/carddisp.pl?gene=ORAI1
GC17M044380	13.93	https://www.genecards.org/cgi-bin/carddisp.pl?gene=ITGA2B
GC16P089912	13.92	https://www.genecards.org/cgi-bin/carddisp.pl?gene=MC1R
GC01P225810	13.92	https://www.genecards.org/cgi-bin/carddisp.pl?gene=EPHX1
GC04P157204	13.92	https://www.genecards.org/cgi-bin/carddisp.pl?gene=GRIA2
GC12P006576	13.92	https://www.genecards.org/cgi-bin/carddisp.pl?gene=CCND2
GC09M099216	13.91	https://www.genecards.org/cgi-bin/carddisp.pl?gene=ALG2
GC05M058969	13.91	https://www.genecards.org/cgi-bin/carddisp.pl?gene=PDE4D
GC0XM101349	13.86	https://www.genecards.org/cgi-bin/carddisp.pl?gene=BTK
GC17P042851	13.86	https://www.genecards.org/cgi-bin/carddisp.pl?gene=AOC3
GC11P021799	13.84	https://www.genecards.org/cgi-bin/carddisp.pl?gene=ANO5
GC15P074748	13.84	https://www.genecards.org/cgi-bin/carddisp.pl?gene=CYP1A2
GC0XM045746	13.83	https://www.genecards.org/cgi-bin/carddisp.pl?gene=MIR221
GC13P020566	13.83	https://www.genecards.org/cgi-bin/carddisp.pl?gene=IFT88
GC17M042199	13.83	https://www.genecards.org/cgi-bin/carddisp.pl?gene=STAT5B
GC04P024798	13.83	https://www.genecards.org/cgi-bin/carddisp.pl?gene=SOD3
GC05P140076	13.82	https://www.genecards.org/cgi-bin/carddisp.pl?gene=PURA
GC01P161314	13.82	https://www.genecards.org/cgi-bin/carddisp.pl?gene=SDHC
GC15P039581	13.82	https://www.genecards.org/cgi-bin/carddisp.pl?gene=THBS1
GC17P061399	13.81	https://www.genecards.org/cgi-bin/carddisp.pl?gene=TBX2
GC01M108134	13.79	https://www.genecards.org/cgi-bin/carddisp.pl?gene=SLC25A24
GC01M153391	13.79	https://www.genecards.org/cgi-bin/carddisp.pl?gene=S100A8
GC06M107152	13.78	https://www.genecards.org/cgi-bin/carddisp.pl?gene=PDSS2
GC01P109549	13.77	https://www.genecards.org/cgi-bin/carddisp.pl?gene=MIR197
GC11P018394	13.76	https://www.genecards.org/cgi-bin/carddisp.pl?gene=LDHA
GC08M009903	13.76	https://www.genecards.org/cgi-bin/carddisp.pl?gene=MIR124-1
GC0XP041333	13.75	https://www.genecards.org/cgi-bin/carddisp.pl?gene=DDX3X
GC19P022230	13.75	https://www.genecards.org/cgi-bin/carddisp.pl?gene=GTPBP3
GC01P116372	13.74	https://www.genecards.org/cgi-bin/carddisp.pl?gene=ATP1A1
GC20M010403	13.73	https://www.genecards.org/cgi-bin/carddisp.pl?gene=MKKS
GC02P111119	13.72	https://www.genecards.org/cgi-bin/carddisp.pl?gene=BCL2L11
GC08M027459	13.7	https://www.genecards.org/cgi-bin/carddisp.pl?gene=CHRNA2
GC11P002899	13.7	https://www.genecards.org/cgi-bin/carddisp.pl?gene=SLC22A18
GC0XM071239	13.68	https://www.genecards.org/cgi-bin/carddisp.pl?gene=ZMYM3
GC20M005114	13.66	https://www.genecards.org/cgi-bin/carddisp.pl?gene=PCNA
GC09P087725	13.66	https://www.genecards.org/cgi-bin/carddisp.pl?gene=CTSL
GC17M044205	13.65	https://www.genecards.org/cgi-bin/carddisp.pl?gene=UBTF
GC16P057447	13.65	https://www.genecards.org/cgi-bin/carddisp.pl?gene=COQ9
GC11M123629	13.64	https://www.genecards.org/cgi-bin/carddisp.pl?gene=SCN3B
GCMTM014676	13.63	https://www.genecards.org/cgi-bin/carddisp.pl?gene=MT-TE
GC17M078852	13.62	https://www.genecards.org/cgi-bin/carddisp.pl?gene=TIMP2

GC20M034928	13.59	https://www.genecards.org/cgi-bin/carddisp.pl?gene=GSS
GC03P046356	13.57	https://www.genecards.org/cgi-bin/carddisp.pl?gene=CCR2
GC17M059674	13.56	https://www.genecards.org/cgi-bin/carddisp.pl?gene=PTRH2
GC10P026697	13.56	https://www.genecards.org/cgi-bin/carddisp.pl?gene=PDSS1
GC17P012665	13.54	https://www.genecards.org/cgi-bin/carddisp.pl?gene=MYOCD
GC01P050960	13.54	https://www.genecards.org/cgi-bin/carddisp.pl?gene=CDKN2C
GC19M016489	13.5	https://www.genecards.org/cgi-bin/carddisp.pl?gene=CHERP
GC06P033153	13.5	https://www.genecards.org/cgi-bin/carddisp.pl?gene=MOG
GC17P017682	13.5	https://www.genecards.org/cgi-bin/carddisp.pl?gene=RAI1
GC06M033572	13.47	https://www.genecards.org/cgi-bin/carddisp.pl?gene=BAK1
GC11M036467	13.46	https://www.genecards.org/cgi-bin/carddisp.pl?gene=TRAF6
GC03P011009	13.46	https://www.genecards.org/cgi-bin/carddisp.pl?gene=SLC6A1
GC05P150997	13.45	https://www.genecards.org/cgi-bin/carddisp.pl?gene=GPX3
GC01M119031	13.42	https://www.genecards.org/cgi-bin/carddisp.pl?gene=WARS2
GC13M110641	13.4	https://www.genecards.org/cgi-bin/carddisp.pl?gene=CARS2
GC05P127228	13.39	https://www.genecards.org/cgi-bin/carddisp.pl?gene=MEGF10
GC01M039842	13.38	https://www.genecards.org/cgi-bin/carddisp.pl?gene=TRIT1
GC20P001113	13.37	https://www.genecards.org/cgi-bin/carddisp.pl?gene=PSMF1
GC11M064273	13.37	https://www.genecards.org/cgi-bin/carddisp.pl?gene=BAD
GC04M017460	13.35	https://www.genecards.org/cgi-bin/carddisp.pl?gene=QDPR
GC11P065525	13.35	https://www.genecards.org/cgi-bin/carddisp.pl?gene=SCYL1
GC05P132866	13.35	https://www.genecards.org/cgi-bin/carddisp.pl?gene=UQCRQ
GC17P040287	13.34	https://www.genecards.org/cgi-bin/carddisp.pl?gene=CDC6
GC07M117276	13.31	https://www.genecards.org/cgi-bin/carddisp.pl?gene=WNT2
GC02M070447	13.28	https://www.genecards.org/cgi-bin/carddisp.pl?gene=TGFA
GC05M151639	13.27	https://www.genecards.org/cgi-bin/carddisp.pl?gene=SPARC
GC14P073949	13.26	https://www.genecards.org/cgi-bin/carddisp.pl?gene=COQ6
GC0XM053225	13.25	https://www.genecards.org/cgi-bin/carddisp.pl?gene=IQSEC2
GC01P161766	13.25	https://www.genecards.org/cgi-bin/carddisp.pl?gene=ATF6
GC03M172505	13.23	https://www.genecards.org/cgi-bin/carddisp.pl?gene=TNFSF10
GC01M037860	13.21	https://www.genecards.org/cgi-bin/carddisp.pl?gene=INPP5B
GC10P094938	13.21	https://www.genecards.org/cgi-bin/carddisp.pl?gene=CYP2C9
GC05M157063	13.18	https://www.genecards.org/cgi-bin/carddisp.pl?gene=HAVCR2
GC21P045405	13.17	https://www.genecards.org/cgi-bin/carddisp.pl?gene=COL18A1
GC11P013668	13.17	https://www.genecards.org/cgi-bin/carddisp.pl?gene=FAR1
GC06P043171	13.17	https://www.genecards.org/cgi-bin/carddisp.pl?gene=SRF
GC20P041136	13.16	https://www.genecards.org/cgi-bin/carddisp.pl?gene=PLCG1
GC12M032790	13.14	https://www.genecards.org/cgi-bin/carddisp.pl?gene=PKP2
GC22M042160	13.14	https://www.genecards.org/cgi-bin/carddisp.pl?gene=TCF20
GC20P041028	13.13	https://www.genecards.org/cgi-bin/carddisp.pl?gene=TOP1
GC03M128479	13.12	https://www.genecards.org/cgi-bin/carddisp.pl?gene=GATA2
GC08P085463	13.12	https://www.genecards.org/cgi-bin/carddisp.pl?gene=CA2
GC22P046330	13.11	https://www.genecards.org/cgi-bin/carddisp.pl?gene=TRMU
GC01M045329	13.11	https://www.genecards.org/cgi-bin/carddisp.pl?gene=MUTYH
GC09P091113	13.11	https://www.genecards.org/cgi-bin/carddisp.pl?gene=SYK
GC19M040230	13.07	https://www.genecards.org/cgi-bin/carddisp.pl?gene=AKT2
GC01M023870	13.05	https://www.genecards.org/cgi-bin/carddisp.pl?gene=CNR2

GC03M043396	13.05	https://www.genecards.org/cgi-bin/carddisp.pl?gene=ANO10
GC01M035176	13.04	https://www.genecards.org/cgi-bin/carddisp.pl?gene=SFPQ
GC05P138465	13.04	https://www.genecards.org/cgi-bin/carddisp.pl?gene=EGR1
GC02M049918	13.04	https://www.genecards.org/cgi-bin/carddisp.pl?gene=NRXN1
GC09P006720	13.03	https://www.genecards.org/cgi-bin/carddisp.pl?gene=KDM4C
GC10P050991	13.03	https://www.genecards.org/cgi-bin/carddisp.pl?gene=PRKG1
GC01P032292	13.03	https://www.genecards.org/cgi-bin/carddisp.pl?gene=HDAC1
GC10M099696	13.02	https://www.genecards.org/cgi-bin/carddisp.pl?gene=COX15
GC02P219279	12.97	https://www.genecards.org/cgi-bin/carddisp.pl?gene=DNAJB2
GC06M026056	12.96	https://www.genecards.org/cgi-bin/carddisp.pl?gene=H1-2
GC19P013876	12.93	https://www.genecards.org/cgi-bin/carddisp.pl?gene=MIR181C
GC06M136557	12.93	https://www.genecards.org/cgi-bin/carddisp.pl?gene=MAP3K5
GCMTP004265	12.92	https://www.genecards.org/cgi-bin/carddisp.pl?gene=MT-TI
GC01P002019	12.91	https://www.genecards.org/cgi-bin/carddisp.pl?gene=GABRD
GC10M099875	12.9	https://www.genecards.org/cgi-bin/carddisp.pl?gene=DNMBP
GC02M151832	12.9	https://www.genecards.org/cgi-bin/carddisp.pl?gene=CACNB4
GC15M027754	12.89	https://www.genecards.org/cgi-bin/carddisp.pl?gene=OCA2
GC11P128891	12.88	https://www.genecards.org/cgi-bin/carddisp.pl?gene=KCNJ5
GC01M161541	12.88	https://www.genecards.org/cgi-bin/carddisp.pl?gene=FCGR3A
GC22M030262	12.85	https://www.genecards.org/cgi-bin/carddisp.pl?gene=OSM
GC11P108121	12.85	https://www.genecards.org/cgi-bin/carddisp.pl?gene=ACAT1
GC20P017952	12.85	https://www.genecards.org/cgi-bin/carddisp.pl?gene=MGME1
GC01P006728	12.84	https://www.genecards.org/cgi-bin/carddisp.pl?gene=THAP3
GC01M172229	12.82	https://www.genecards.org/cgi-bin/carddisp.pl?gene=MIR214
GC12P053380	12.81	https://www.genecards.org/cgi-bin/carddisp.pl?gene=SP1
GC19M042230	12.79	https://www.genecards.org/cgi-bin/carddisp.pl?gene=GSK3A
GC20P032359	12.79	https://www.genecards.org/cgi-bin/carddisp.pl?gene=ASXL1
GC0XP003006	12.79	https://www.genecards.org/cgi-bin/carddisp.pl?gene=ARSH
GC12P052948	12.77	https://www.genecards.org/cgi-bin/carddisp.pl?gene=KRT18
GC01M031365	12.77	https://www.genecards.org/cgi-bin/carddisp.pl?gene=FABP3
GC12P103930	12.75	https://www.genecards.org/cgi-bin/carddisp.pl?gene=HSP90B1
GC17P001716	12.74	https://www.genecards.org/cgi-bin/carddisp.pl?gene=WDR81
GC02P223957	12.74	https://www.genecards.org/cgi-bin/carddisp.pl?gene=MRPL44
GC08P011486	12.73	https://www.genecards.org/cgi-bin/carddisp.pl?gene=BLK
GC18M027950	12.73	https://www.genecards.org/cgi-bin/carddisp.pl?gene=CDH2
GC17M043952	12.72	https://www.genecards.org/cgi-bin/carddisp.pl?gene=PYY
GC16P056589	12.7	https://www.genecards.org/cgi-bin/carddisp.pl?gene=MT3
GC17M001346	12.7	https://www.genecards.org/cgi-bin/carddisp.pl?gene=YWHAE
GC01M044819	12.69	https://www.genecards.org/cgi-bin/carddisp.pl?gene=PTCH2
GC0XP104566	12.69	https://www.genecards.org/cgi-bin/carddisp.pl?gene=IL1RAPL2
GC16P004474	12.68	https://www.genecards.org/cgi-bin/carddisp.pl?gene=HMOX2
GC22P030606	12.68	https://www.genecards.org/cgi-bin/carddisp.pl?gene=TCN2
GC19P016111	12.68	https://www.genecards.org/cgi-bin/carddisp.pl?gene=RAB8A
GC12P014983	12.66	https://www.genecards.org/cgi-bin/carddisp.pl?gene=PDE6H
GC12P055718	12.66	https://www.genecards.org/cgi-bin/carddisp.pl?gene=BLOC1S1
GC17M044264	12.66	https://www.genecards.org/cgi-bin/carddisp.pl?gene=SLC4A1
GC11P111578	12.65	https://www.genecards.org/cgi-bin/carddisp.pl?gene=MIR34B

GC0XM001386	12.64	https://www.genecards.org/cgi-bin/carddisp.pl?gene=SLC25A6
GC08P026514	12.64	https://www.genecards.org/cgi-bin/carddisp.pl?gene=DPYSL2
GC07M152134	12.63	https://www.genecards.org/cgi-bin/carddisp.pl?gene=KMT2C
GC12M021797	12.62	https://www.genecards.org/cgi-bin/carddisp.pl?gene=ABCC9
GC17M078356	12.62	https://www.genecards.org/cgi-bin/carddisp.pl?gene=SOCS3
GC04P015704	12.61	https://www.genecards.org/cgi-bin/carddisp.pl?gene=BST1
GC02P026243	12.58	https://www.genecards.org/cgi-bin/carddisp.pl?gene=HADHB
GC05P137867	12.57	https://www.genecards.org/cgi-bin/carddisp.pl?gene=MYOT
GC06M033694	12.57	https://www.genecards.org/cgi-bin/carddisp.pl?gene=UQCC2
GC03P152243	12.56	https://www.genecards.org/cgi-bin/carddisp.pl?gene=MBNL1
GC01M160288	12.56	https://www.genecards.org/cgi-bin/carddisp.pl?gene=COPA
GC19M043646	12.56	https://www.genecards.org/cgi-bin/carddisp.pl?gene=PLAUR
GC17M017810	12.55	https://www.genecards.org/cgi-bin/carddisp.pl?gene=SREBF1
GC17P007934	12.54	https://www.genecards.org/cgi-bin/carddisp.pl?gene=POLR2A
GC13M049982	12.54	https://www.genecards.org/cgi-bin/carddisp.pl?gene=DLEU2
GC19M049906	12.53	https://www.genecards.org/cgi-bin/carddisp.pl?gene=NUP62
GC20P046021	12.53	https://www.genecards.org/cgi-bin/carddisp.pl?gene=SLC12A5
GC16P081318	12.49	https://www.genecards.org/cgi-bin/carddisp.pl?gene=GAN
GC12P078863	12.48	https://www.genecards.org/cgi-bin/carddisp.pl?gene=SYT1
GC02M223975	12.48	https://www.genecards.org/cgi-bin/carddisp.pl?gene=SERPINE2
GC17P002593	12.48	https://www.genecards.org/cgi-bin/carddisp.pl?gene=PAFAH1B1
GC13P025373	12.47	https://www.genecards.org/cgi-bin/carddisp.pl?gene=ATP8A2
GC20M036890	12.45	https://www.genecards.org/cgi-bin/carddisp.pl?gene=SAMHD1
GC02U990025	12.45	https://www.genecards.org/cgi-bin/carddisp.pl?gene=ETM2
GC11P035684	12.44	https://www.genecards.org/cgi-bin/carddisp.pl?gene=TRIM44
GC06M106571	12.43	https://www.genecards.org/cgi-bin/carddisp.pl?gene=RTN4IP1
GC01P243255	12.43	https://www.genecards.org/cgi-bin/carddisp.pl?gene=SDCCAG8
GC12P123712	12.43	https://www.genecards.org/cgi-bin/carddisp.pl?gene=ATP6V0A2
GC07P129303	12.4	https://www.genecards.org/cgi-bin/carddisp.pl?gene=SMO
GC11M075335	12.4	https://www.genecards.org/cgi-bin/carddisp.pl?gene=MIR326
GC01P168280	12.38	https://www.genecards.org/cgi-bin/carddisp.pl?gene=TBX19
GC01P040754	12.34	https://www.genecards.org/cgi-bin/carddisp.pl?gene=MIR30E
GC12M057757	12.34	https://www.genecards.org/cgi-bin/carddisp.pl?gene=CYP27B1
GC19P029811	12.33	https://www.genecards.org/cgi-bin/carddisp.pl?gene=CCNE1
GC17M075039	12.33	https://www.genecards.org/cgi-bin/carddisp.pl?gene=ATP5PD
GC03M052704	12.33	https://www.genecards.org/cgi-bin/carddisp.pl?gene=GLT8D1
GC18P044680	12.33	https://www.genecards.org/cgi-bin/carddisp.pl?gene=SETBP1
GC01P161663	12.29	https://www.genecards.org/cgi-bin/carddisp.pl?gene=FCGR2B
GC0XP153724	12.28	https://www.genecards.org/cgi-bin/carddisp.pl?gene=ABCD1
GC12M049184	12.27	https://www.genecards.org/cgi-bin/carddisp.pl?gene=TUBA1A
GC12P057778	12.27	https://www.genecards.org/cgi-bin/carddisp.pl?gene=TSFM
GC11P070203	12.27	https://www.genecards.org/cgi-bin/carddisp.pl?gene=FADD
GC07M075769	12.27	https://www.genecards.org/cgi-bin/carddisp.pl?gene=CCL26
GC09M120815	12.25	https://www.genecards.org/cgi-bin/carddisp.pl?gene=PSMD5
GC08M096225	12.25	https://www.genecards.org/cgi-bin/carddisp.pl?gene=UQCRB
GC14P064388	12.24	https://www.genecards.org/cgi-bin/carddisp.pl?gene=MTHFD1
GC12P011649	12.24	https://www.genecards.org/cgi-bin/carddisp.pl?gene=ETV6

GC06P021593	12.23	https://www.genecards.org/cgi-bin/carddisp.pl?gene=SOX4
GC21M033904	12.22	https://www.genecards.org/cgi-bin/carddisp.pl?gene=ATP5PO
GC0XP116170	12.21	https://www.genecards.org/cgi-bin/carddisp.pl?gene=AGTR2
GC01M243488	12.19	https://www.genecards.org/cgi-bin/carddisp.pl?gene=AKT3
GC0XM015494	12.19	https://www.genecards.org/cgi-bin/carddisp.pl?gene=ACE2
GC17M081509	12.18	https://www.genecards.org/cgi-bin/carddisp.pl?gene=ACTG1
GC07P046473	12.17	https://www.genecards.org/cgi-bin/carddisp.pl?gene=IGFBP1
GC16M067203	12.17	https://www.genecards.org/cgi-bin/carddisp.pl?gene=MIR328
GC01M001335	12.15	https://www.genecards.org/cgi-bin/carddisp.pl?gene=DVL1
GC17P078214	12.14	https://www.genecards.org/cgi-bin/carddisp.pl?gene=BIRC5
GC09P129803	12.12	https://www.genecards.org/cgi-bin/carddisp.pl?gene=TOR1B
GC10M015513	12.12	https://www.genecards.org/cgi-bin/carddisp.pl?gene=ITGA8
GC17P042609	12.11	https://www.genecards.org/cgi-bin/carddisp.pl?gene=TUBG1
GC01M002508	12.11	https://www.genecards.org/cgi-bin/carddisp.pl?gene=PANK4
GC19M041439	12.11	https://www.genecards.org/cgi-bin/carddisp.pl?gene=SARS2
GC09M019366	12.1	https://www.genecards.org/cgi-bin/carddisp.pl?gene=RPS6
GC04P151099	12.1	https://www.genecards.org/cgi-bin/carddisp.pl?gene=RPS3A
GC17P046590	12.09	https://www.genecards.org/cgi-bin/carddisp.pl?gene=NSF
GC05M161288	12.07	https://www.genecards.org/cgi-bin/carddisp.pl?gene=GABRB2
GC22M037066	12.07	https://www.genecards.org/cgi-bin/carddisp.pl?gene=TMPRSS6
GC04M099304	12.06	https://www.genecards.org/cgi-bin/carddisp.pl?gene=ADH1B
GC16P085798	12.05	https://www.genecards.org/cgi-bin/carddisp.pl?gene=COX4I1
GC19P038647	12.05	https://www.genecards.org/cgi-bin/carddisp.pl?gene=ACTN4
GC03M119524	12.05	https://www.genecards.org/cgi-bin/carddisp.pl?gene=CD80
GC07M099759	12.05	https://www.genecards.org/cgi-bin/carddisp.pl?gene=CYP3A4
GC16M088715	12.04	https://www.genecards.org/cgi-bin/carddisp.pl?gene=PIEZO1
GC12M021531	12.01	https://www.genecards.org/cgi-bin/carddisp.pl?gene=GYS2
GCMTP001674	12.01	https://www.genecards.org/cgi-bin/carddisp.pl?gene=MT-RNR2
GC07P087934	12	https://www.genecards.org/cgi-bin/carddisp.pl?gene=ADAM22
GC15P066702	12	https://www.genecards.org/cgi-bin/carddisp.pl?gene=SMAD6
GC01M102876	11.98	https://www.genecards.org/cgi-bin/carddisp.pl?gene=COL11A1
GC09M035672	11.98	https://www.genecards.org/cgi-bin/carddisp.pl?gene=TPM2
GC13M109752	11.98	https://www.genecards.org/cgi-bin/carddisp.pl?gene=IRS2
GC20M049293	11.96	https://www.genecards.org/cgi-bin/carddisp.pl?gene=KCNB1
GC01P231626	11.95	https://www.genecards.org/cgi-bin/carddisp.pl?gene=DISC1
GC22P020080	11.95	https://www.genecards.org/cgi-bin/carddisp.pl?gene=DGCR8
GC02P148875	11.95	https://www.genecards.org/cgi-bin/carddisp.pl?gene=KIF5C
GC11P067266	11.95	https://www.genecards.org/cgi-bin/carddisp.pl?gene=GRK2
GC03P048250	11.94	https://www.genecards.org/cgi-bin/carddisp.pl?gene=CAMP
GC01M154697	11.94	https://www.genecards.org/cgi-bin/carddisp.pl?gene=KCNN3
GC03P039406	11.93	https://www.genecards.org/cgi-bin/carddisp.pl?gene=RPSA
GC01P017308	11.92	https://www.genecards.org/cgi-bin/carddisp.pl?gene=PADI4
GC01P239386	11.92	https://www.genecards.org/cgi-bin/carddisp.pl?gene=CHRM3
GC12U990107	11.92	https://www.genecards.org/cgi-bin/carddisp.pl?gene=AD5
GC08P042271	11.91	https://www.genecards.org/cgi-bin/carddisp.pl?gene=IKBKB
GC01P154273	11.89	https://www.genecards.org/cgi-bin/carddisp.pl?gene=HAX1
GC06P039048	11.89	https://www.genecards.org/cgi-bin/carddisp.pl?gene=GLP1R

GC02M223596	11.88	https://www.genecards.org/cgi-bin/carddisp.pl?gene=SCG2
GC04M003508	11.87	https://www.genecards.org/cgi-bin/carddisp.pl?gene=LRPAP1
GC11M006396	11.87	https://www.genecards.org/cgi-bin/carddisp.pl?gene=APBB1
GC20M063488	11.86	https://www.genecards.org/cgi-bin/carddisp.pl?gene=EEF1A2
GC18P031447	11.86	https://www.genecards.org/cgi-bin/carddisp.pl?gene=DSG3
GC06M003153	11.86	https://www.genecards.org/cgi-bin/carddisp.pl?gene=TUBB2A
GC15P078131	11.86	https://www.genecards.org/cgi-bin/carddisp.pl?gene=IDH3A
GC05M168560	11.85	https://www.genecards.org/cgi-bin/carddisp.pl?gene=MIR103A1
GC20M045252	11.85	https://www.genecards.org/cgi-bin/carddisp.pl?gene=SLPI
GC01M203083	11.84	https://www.genecards.org/cgi-bin/carddisp.pl?gene=MYOG
GC02P241357	11.84	https://www.genecards.org/cgi-bin/carddisp.pl?gene=FARP2
GC12M057530	11.83	https://www.genecards.org/cgi-bin/carddisp.pl?gene=DCTN2
GC11M004384	11.83	https://www.genecards.org/cgi-bin/carddisp.pl?gene=TRIM21
GC11P066499	11.83	https://www.genecards.org/cgi-bin/carddisp.pl?gene=BBS1
GC01P075724	11.83	https://www.genecards.org/cgi-bin/carddisp.pl?gene=ACADM
GC12P007827	11.81	https://www.genecards.org/cgi-bin/carddisp.pl?gene=P3H3
GC17P007579	11.8	https://www.genecards.org/cgi-bin/carddisp.pl?gene=CD68
GC01M171604	11.8	https://www.genecards.org/cgi-bin/carddisp.pl?gene=MYOC
GC03P058008	11.79	https://www.genecards.org/cgi-bin/carddisp.pl?gene=FLNB
GC04M152321	11.79	https://www.genecards.org/cgi-bin/carddisp.pl?gene=FBXW7
GC11M102862	11.79	https://www.genecards.org/cgi-bin/carddisp.pl?gene=MMP12
GC22P031753	11.78	https://www.genecards.org/cgi-bin/carddisp.pl?gene=DEPDC5
GC19M013987	11.78	https://www.genecards.org/cgi-bin/carddisp.pl?gene=MIR27A
GC06P052362	11.78	https://www.genecards.org/cgi-bin/carddisp.pl?gene=EFHC1
GC10P007789	11.77	https://www.genecards.org/cgi-bin/carddisp.pl?gene=ATP5F1C
GC10P072273	11.77	https://www.genecards.org/cgi-bin/carddisp.pl?gene=DDIT4
GC08M026747	11.76	https://www.genecards.org/cgi-bin/carddisp.pl?gene=ADRA1A
GC16P023869	11.76	https://www.genecards.org/cgi-bin/carddisp.pl?gene=PLK1
GC17P059619	11.75	https://www.genecards.org/cgi-bin/carddisp.pl?gene=CLTC
GC21M025717	11.74	https://www.genecards.org/cgi-bin/carddisp.pl?gene=ATP5PF
GC21P041420	11.73	https://www.genecards.org/cgi-bin/carddisp.pl?gene=MX1
GC06M105277	11.73	https://www.genecards.org/cgi-bin/carddisp.pl?gene=PREP
GC04M076001	11.73	https://www.genecards.org/cgi-bin/carddisp.pl?gene=CXCL9
GC06U903633	11.71	https://www.genecards.org/cgi-bin/carddisp.pl?gene=LOC108663996
GC02P218402	11.71	https://www.genecards.org/cgi-bin/carddisp.pl?gene=MIR26B
GC21P033402	11.7	https://www.genecards.org/cgi-bin/carddisp.pl?gene=IFNGR2
GC06P003064	11.7	https://www.genecards.org/cgi-bin/carddisp.pl?gene=RIPK1
GC03M024117	11.7	https://www.genecards.org/cgi-bin/carddisp.pl?gene=THRB
GC05M038939	11.7	https://www.genecards.org/cgi-bin/carddisp.pl?gene=RICTOR
GC10U990101	11.69	https://www.genecards.org/cgi-bin/carddisp.pl?gene=AD6
GC07M000549	11.69	https://www.genecards.org/cgi-bin/carddisp.pl?gene=PRKAR1B
GC02M060451	11.67	https://www.genecards.org/cgi-bin/carddisp.pl?gene=BCL11A
GC04P089838	11.65	https://www.genecards.org/cgi-bin/carddisp.pl?gene=SNCA-AS1
GC19M006496	11.63	https://www.genecards.org/cgi-bin/carddisp.pl?gene=TUBB4A
GC01P022636	11.63	https://www.genecards.org/cgi-bin/carddisp.pl?gene=C1QA
GC04P080183	11.62	https://www.genecards.org/cgi-bin/carddisp.pl?gene=PRDM8
GC17P042680	11.62	https://www.genecards.org/cgi-bin/carddisp.pl?gene=CNTNAP1

GC06M084687	11.6	https://www.genecards.org/cgi-bin/carddisp.pl?gene=TBX18
GC08M076980	11.59	https://www.genecards.org/cgi-bin/carddisp.pl?gene=PEX2
GC05M009036	11.58	https://www.genecards.org/cgi-bin/carddisp.pl?gene=SEMA5A
GC03M064079	11.57	https://www.genecards.org/cgi-bin/carddisp.pl?gene=PRICKLE2
GC06P131473	11.57	https://www.genecards.org/cgi-bin/carddisp.pl?gene=ARG1
GC03P136250	11.56	https://www.genecards.org/cgi-bin/carddisp.pl?gene=PCCB
GC04M154604	11.54	https://www.genecards.org/cgi-bin/carddisp.pl?gene=FGG
GC05M095752	11.54	https://www.genecards.org/cgi-bin/carddisp.pl?gene=GLRX
GC04M000784	11.53	https://www.genecards.org/cgi-bin/carddisp.pl?gene=CPLX1
GC04M009772	11.5	https://www.genecards.org/cgi-bin/carddisp.pl?gene=SLC2A9
GC05P083471	11.48	https://www.genecards.org/cgi-bin/carddisp.pl?gene=VCAN
GC11P119206	11.47	https://www.genecards.org/cgi-bin/carddisp.pl?gene=CBL
GC10P075210	11.47	https://www.genecards.org/cgi-bin/carddisp.pl?gene=VDAC2
GC22P040346	11.46	https://www.genecards.org/cgi-bin/carddisp.pl?gene=ADSL
GC15M029699	11.45	https://www.genecards.org/cgi-bin/carddisp.pl?gene=TJP1
GC20P043457	11.43	https://www.genecards.org/cgi-bin/carddisp.pl?gene=SRSF6
GC21P045981	11.43	https://www.genecards.org/cgi-bin/carddisp.pl?gene=COL6A1
GC0XP111665	11.41	https://www.genecards.org/cgi-bin/carddisp.pl?gene=ALG13
GC12P120439	11.41	https://www.genecards.org/cgi-bin/carddisp.pl?gene=COX6A1
GC03M179397	11.4	https://www.genecards.org/cgi-bin/carddisp.pl?gene=GNB4
GC03M133932	11.39	https://www.genecards.org/cgi-bin/carddisp.pl?gene=SLCO2A1
GC01M175291	11.39	https://www.genecards.org/cgi-bin/carddisp.pl?gene=TNR
GC17P063600	11.38	https://www.genecards.org/cgi-bin/carddisp.pl?gene=TACO1
GC11P057777	11.38	https://www.genecards.org/cgi-bin/carddisp.pl?gene=CTNND1
GC05M135334	11.37	https://www.genecards.org/cgi-bin/carddisp.pl?gene=MACROH2A1
GC11P119084	11.37	https://www.genecards.org/cgi-bin/carddisp.pl?gene=HMBS
GC20P034363	11.37	https://www.genecards.org/cgi-bin/carddisp.pl?gene=ITCH
GC19M019645	11.36	https://www.genecards.org/cgi-bin/carddisp.pl?gene=ATP13A1
GC14P067359	11.36	https://www.genecards.org/cgi-bin/carddisp.pl?gene=EIF2S1
GC10P031318	11.35	https://www.genecards.org/cgi-bin/carddisp.pl?gene=ZEB1
GC16P030199	11.35	https://www.genecards.org/cgi-bin/carddisp.pl?gene=SULT1A3
GC01P228072	11.35	https://www.genecards.org/cgi-bin/carddisp.pl?gene=WNT3A
GC18P003411	11.35	https://www.genecards.org/cgi-bin/carddisp.pl?gene=TGIF1
GC19M041436	11.34	https://www.genecards.org/cgi-bin/carddisp.pl?gene=LGALS4
GC01M175010	11.34	https://www.genecards.org/cgi-bin/carddisp.pl?gene=MRPS14
GC12P123232	11.33	https://www.genecards.org/cgi-bin/carddisp.pl?gene=C12orf65
GC15M089209	11.33	https://www.genecards.org/cgi-bin/carddisp.pl?gene=RLBP1
GC01M198860	11.33	https://www.genecards.org/cgi-bin/carddisp.pl?gene=MIR181A1
GC08M115408	11.32	https://www.genecards.org/cgi-bin/carddisp.pl?gene=TRPS1
GC17P004948	11.3	https://www.genecards.org/cgi-bin/carddisp.pl?gene=ENO3
GC17M008770	11.3	https://www.genecards.org/cgi-bin/carddisp.pl?gene=AURKB
GC06P026032	11.29	https://www.genecards.org/cgi-bin/carddisp.pl?gene=H3C1
GC03P157436	11.29	https://www.genecards.org/cgi-bin/carddisp.pl?gene=PTX3
GC16P075566	11.26	https://www.genecards.org/cgi-bin/carddisp.pl?gene=GABARAPL2
GC10M100188	11.24	https://www.genecards.org/cgi-bin/carddisp.pl?gene=CHUK
GC08M117798	11.23	https://www.genecards.org/cgi-bin/carddisp.pl?gene=EXT1
GC08M041660	11.23	https://www.genecards.org/cgi-bin/carddisp.pl?gene=MIR486-1

GC10U900571	11.22	https://www.genecards.org/cgi-bin/carddisp.pl?gene=AD7
GC03P042490	11.21	https://www.genecards.org/cgi-bin/carddisp.pl?gene=VIPR1
GC04M071741	11.21	https://www.genecards.org/cgi-bin/carddisp.pl?gene=GC
GC16M030085	11.2	https://www.genecards.org/cgi-bin/carddisp.pl?gene=TBX6
GC02M218823	11.19	https://www.genecards.org/cgi-bin/carddisp.pl?gene=PRKAG3
GC12P007879	11.19	https://www.genecards.org/cgi-bin/carddisp.pl?gene=MIR141
GC01M155289	11.17	https://www.genecards.org/cgi-bin/carddisp.pl?gene=PKLR
GC08P042368	11.16	https://www.genecards.org/cgi-bin/carddisp.pl?gene=VDAC3
GC05P146447	11.16	https://www.genecards.org/cgi-bin/carddisp.pl?gene=TCERG1
GC01P003068	11.15	https://www.genecards.org/cgi-bin/carddisp.pl?gene=PRDM16
GC17P009021	11.14	https://www.genecards.org/cgi-bin/carddisp.pl?gene=NTN1
GC07U901794	11.14	https://www.genecards.org/cgi-bin/carddisp.pl?gene=AD10
GC17M074842	11.14	https://www.genecards.org/cgi-bin/carddisp.pl?gene=GRIN2C
GC06M134169	11.13	https://www.genecards.org/cgi-bin/carddisp.pl?gene=SGK1
GC11M073974	11.13	https://www.genecards.org/cgi-bin/carddisp.pl?gene=UCP2
GC11M000215	11.13	https://www.genecards.org/cgi-bin/carddisp.pl?gene=SIRT3
GC01M154127	11.1	https://www.genecards.org/cgi-bin/carddisp.pl?gene=TPM3
GC16P013920	11.1	https://www.genecards.org/cgi-bin/carddisp.pl?gene=ERCC4
GC05M146114	11.08	https://www.genecards.org/cgi-bin/carddisp.pl?gene=LARS1
GC11M011962	11.08	https://www.genecards.org/cgi-bin/carddisp.pl?gene=DKK3
GC12P068686	11.07	https://www.genecards.org/cgi-bin/carddisp.pl?gene=NUP107
GC08M048854	11.06	https://www.genecards.org/cgi-bin/carddisp.pl?gene=SNAI2
GC02P178521	11.05	https://www.genecards.org/cgi-bin/carddisp.pl?gene=TTN-AS1
GC08P144291	11.04	https://www.genecards.org/cgi-bin/carddisp.pl?gene=HSF1
GC0XM046599	11.04	https://www.genecards.org/cgi-bin/carddisp.pl?gene=SLC9A7
GC16P030472	11.03	https://www.genecards.org/cgi-bin/carddisp.pl?gene=ITGAL
GC19P054165	11.03	https://www.genecards.org/cgi-bin/carddisp.pl?gene=PRPF31
GC15P033310	11.03	https://www.genecards.org/cgi-bin/carddisp.pl?gene=RYR3
GC15M028111	11.02	https://www.genecards.org/cgi-bin/carddisp.pl?gene=HERC2
GC10M027110	11.02	https://www.genecards.org/cgi-bin/carddisp.pl?gene=YME1L1
GC13P091347	11.01	https://www.genecards.org/cgi-bin/carddisp.pl?gene=MIR17HG
GC02M190055	11	https://www.genecards.org/cgi-bin/carddisp.pl?gene=MSTN
GC02M008715	10.99	https://www.genecards.org/cgi-bin/carddisp.pl?gene=KIDINS220
GC03P051707	10.98	https://www.genecards.org/cgi-bin/carddisp.pl?gene=GRM2
GC09P136977	10.98	https://www.genecards.org/cgi-bin/carddisp.pl?gene=PTGDS
GC05P111070	10.98	https://www.genecards.org/cgi-bin/carddisp.pl?gene=TSLP
GC01P100351	10.98	https://www.genecards.org/cgi-bin/carddisp.pl?gene=CDC14A
GC08P039891	10.98	https://www.genecards.org/cgi-bin/carddisp.pl?gene=IDO1
GC14M024249	10.97	https://www.genecards.org/cgi-bin/carddisp.pl?gene=TGM1
GC20M035433	10.96	https://www.genecards.org/cgi-bin/carddisp.pl?gene=GDF5
GC15M055826	10.96	https://www.genecards.org/cgi-bin/carddisp.pl?gene=NEDD4
GC14M103697	10.95	https://www.genecards.org/cgi-bin/carddisp.pl?gene=XRCC3
GC15P099565	10.95	https://www.genecards.org/cgi-bin/carddisp.pl?gene=MEF2A
GC20U900157	10.93	https://www.genecards.org/cgi-bin/carddisp.pl?gene=ALS7
GC03M059747	10.93	https://www.genecards.org/cgi-bin/carddisp.pl?gene=FHIT
GC05P069167	10.92	https://www.genecards.org/cgi-bin/carddisp.pl?gene=CCNB1
GC12M052897	10.92	https://www.genecards.org/cgi-bin/carddisp.pl?gene=KRT8

GC18U990005	10.89	https://www.genecards.org/cgi-bin/carddisp.pl?gene=ALS3
GC12M007471	10.88	https://www.genecards.org/cgi-bin/carddisp.pl?gene=CD163
GC09U901247	10.88	https://www.genecards.org/cgi-bin/carddisp.pl?gene=AD11
GC20U900057	10.88	https://www.genecards.org/cgi-bin/carddisp.pl?gene=AD8
GC11M094416	10.87	https://www.genecards.org/cgi-bin/carddisp.pl?gene=MRE11
GC15M078594	10.87	https://www.genecards.org/cgi-bin/carddisp.pl?gene=CHRNA3
GC10M013643	10.86	https://www.genecards.org/cgi-bin/carddisp.pl?gene=FRMD4A
GC11M005967	10.86	https://www.genecards.org/cgi-bin/carddisp.pl?gene=OR56A5
GC01P013700	10.85	https://www.genecards.org/cgi-bin/carddisp.pl?gene=PRDM2
GC17P007219	10.85	https://www.genecards.org/cgi-bin/carddisp.pl?gene=ACADVL
GC17P015999	10.84	https://www.genecards.org/cgi-bin/carddisp.pl?gene=TTC19
GC08U901173	10.83	https://www.genecards.org/cgi-bin/carddisp.pl?gene=AD12
GC01U902342	10.83	https://www.genecards.org/cgi-bin/carddisp.pl?gene=AD13
GC01U902204	10.83	https://www.genecards.org/cgi-bin/carddisp.pl?gene=AD14
GC03U901230	10.83	https://www.genecards.org/cgi-bin/carddisp.pl?gene=AD15
GC01P011934	10.83	https://www.genecards.org/cgi-bin/carddisp.pl?gene=PLOD1
GC01P045012	10.83	https://www.genecards.org/cgi-bin/carddisp.pl?gene=UROD
GC20M001369	10.82	https://www.genecards.org/cgi-bin/carddisp.pl?gene=FKBP1A
GC17P056593	10.82	https://www.genecards.org/cgi-bin/carddisp.pl?gene=NOG
GC14P035278	10.82	https://www.genecards.org/cgi-bin/carddisp.pl?gene=PSMA6
GC13M045333	10.81	https://www.genecards.org/cgi-bin/carddisp.pl?gene=TPT1
GC05M177456	10.81	https://www.genecards.org/cgi-bin/carddisp.pl?gene=DBN1
GC03P130385	10.8	https://www.genecards.org/cgi-bin/carddisp.pl?gene=ACAD9
GC01P151372	10.8	https://www.genecards.org/cgi-bin/carddisp.pl?gene=PSMB4
GC01M205111	10.8	https://www.genecards.org/cgi-bin/carddisp.pl?gene=DSTYK
GC06U902426	10.79	https://www.genecards.org/cgi-bin/carddisp.pl?gene=AD17
GC10P069801	10.79	https://www.genecards.org/cgi-bin/carddisp.pl?gene=COL13A1
GC03P053328	10.78	https://www.genecards.org/cgi-bin/carddisp.pl?gene=CACNA1D
GC17M049404	10.78	https://www.genecards.org/cgi-bin/carddisp.pl?gene=PHB
GC01M207880	10.77	https://www.genecards.org/cgi-bin/carddisp.pl?gene=CD34
GC02M068460	10.77	https://www.genecards.org/cgi-bin/carddisp.pl?gene=FBXO48
GC02U903086	10.77	https://www.genecards.org/cgi-bin/carddisp.pl?gene=LEPQTL1
GC11M059742	10.76	https://www.genecards.org/cgi-bin/carddisp.pl?gene=FOLH1
GC11M033704	10.76	https://www.genecards.org/cgi-bin/carddisp.pl?gene=CD59
GC11P047248	10.76	https://www.genecards.org/cgi-bin/carddisp.pl?gene=NR1H3
GC17P039626	10.75	https://www.genecards.org/cgi-bin/carddisp.pl?gene=PPP1R1B
GC01M181291	10.75	https://www.genecards.org/cgi-bin/carddisp.pl?gene=STX6
GC0XU901247	10.75	https://www.genecards.org/cgi-bin/carddisp.pl?gene=AD16
GC03P184135	10.75	https://www.genecards.org/cgi-bin/carddisp.pl?gene=EIF2B5
GC08M102252	10.72	https://www.genecards.org/cgi-bin/carddisp.pl?gene=UBR5
GC04P092304	10.7	https://www.genecards.org/cgi-bin/carddisp.pl?gene=GRID2
GC17M041717	10.7	https://www.genecards.org/cgi-bin/carddisp.pl?gene=HAP1
GC06P073461	10.7	https://www.genecards.org/cgi-bin/carddisp.pl?gene=MTO1
GC15M055202	10.7	https://www.genecards.org/cgi-bin/carddisp.pl?gene=RAB27A
GC16P030082	10.67	https://www.genecards.org/cgi-bin/carddisp.pl?gene=ALDOA
GC11P010304	10.66	https://www.genecards.org/cgi-bin/carddisp.pl?gene=ADM
GC01P002050	10.66	https://www.genecards.org/cgi-bin/carddisp.pl?gene=PRKCZ

GC22M019863	10.65	https://www.genecards.org/cgi-bin/carddisp.pl?gene=TXNRD2
GC17M075318	10.64	https://www.genecards.org/cgi-bin/carddisp.pl?gene=GRB2
GC16P031414	10.63	https://www.genecards.org/cgi-bin/carddisp.pl?gene=BCKDK
GC0XP047190	10.63	https://www.genecards.org/cgi-bin/carddisp.pl?gene=UBA1
GC09M077423	10.62	https://www.genecards.org/cgi-bin/carddisp.pl?gene=GNA14
GC10M095605	10.61	https://www.genecards.org/cgi-bin/carddisp.pl?gene=ALDH18A1
GC07M099923	10.6	https://www.genecards.org/cgi-bin/carddisp.pl?gene=GJC3
GC16P081773	10.6	https://www.genecards.org/cgi-bin/carddisp.pl?gene=PLCG2
GC11M002177	10.58	https://www.genecards.org/cgi-bin/carddisp.pl?gene=INS-IGF2
GC04P107989	10.58	https://www.genecards.org/cgi-bin/carddisp.pl?gene=HADH
GC0XP066162	10.58	https://www.genecards.org/cgi-bin/carddisp.pl?gene=HEPH
GC19P000797	10.57	https://www.genecards.org/cgi-bin/carddisp.pl?gene=PTBP1
GC08P055879	10.57	https://www.genecards.org/cgi-bin/carddisp.pl?gene=LYN
GC11M065823	10.56	https://www.genecards.org/cgi-bin/carddisp.pl?gene=CFL1
GC13P113667	10.55	https://www.genecards.org/cgi-bin/carddisp.pl?gene=GRK1
GC17P073165	10.55	https://www.genecards.org/cgi-bin/carddisp.pl?gene=SSTR2
GC05M038475	10.54	https://www.genecards.org/cgi-bin/carddisp.pl?gene=LIFR
GC01M241499	10.54	https://www.genecards.org/cgi-bin/carddisp.pl?gene=FH
GC04P046949	10.53	https://www.genecards.org/cgi-bin/carddisp.pl?gene=GABRB1
GC19M010350	10.52	https://www.genecards.org/cgi-bin/carddisp.pl?gene=TYK2
GC17M048579	10.52	https://www.genecards.org/cgi-bin/carddisp.pl?gene=MIR10A
GC19P051217	10.52	https://www.genecards.org/cgi-bin/carddisp.pl?gene=CD33
GC01P012231	10.51	https://www.genecards.org/cgi-bin/carddisp.pl?gene=VPS13D
GC17M001650	10.5	https://www.genecards.org/cgi-bin/carddisp.pl?gene=PRPF8
GC19P010416	10.5	https://www.genecards.org/cgi-bin/carddisp.pl?gene=PDE4A
GC06P016238	10.5	https://www.genecards.org/cgi-bin/carddisp.pl?gene=GMPR
GC17P007935	10.5	https://www.genecards.org/cgi-bin/carddisp.pl?gene=TNFSF12
GC10M073812	10.49	https://www.genecards.org/cgi-bin/carddisp.pl?gene=CAMK2G
GC22P029269	10.48	https://www.genecards.org/cgi-bin/carddisp.pl?gene=EWSR1
GC20P018119	10.46	https://www.genecards.org/cgi-bin/carddisp.pl?gene=PET117
GC19M055364	10.45	https://www.genecards.org/cgi-bin/carddisp.pl?gene=IL11
GC04M108047	10.45	https://www.genecards.org/cgi-bin/carddisp.pl?gene=LEF1
GC07M002568	10.45	https://www.genecards.org/cgi-bin/carddisp.pl?gene=BRAT1
GC07P136868	10.45	https://www.genecards.org/cgi-bin/carddisp.pl?gene=CHRM2
GC16P085898	10.44	https://www.genecards.org/cgi-bin/carddisp.pl?gene=IRF8
GC16P046689	10.44	https://www.genecards.org/cgi-bin/carddisp.pl?gene=ORC6
GC15P040595	10.43	https://www.genecards.org/cgi-bin/carddisp.pl?gene=KNL1
GC11M086945	10.43	https://www.genecards.org/cgi-bin/carddisp.pl?gene=FZD4
GC10M133362	10.42	https://www.genecards.org/cgi-bin/carddisp.pl?gene=ECHS1
GC09P017906	10.42	https://www.genecards.org/cgi-bin/carddisp.pl?gene=ADAMTSL1
GC03M171024	10.42	https://www.genecards.org/cgi-bin/carddisp.pl?gene=SLC2A2
GC01M229441	10.41	https://www.genecards.org/cgi-bin/carddisp.pl?gene=NUP133
GC01P067685	10.41	https://www.genecards.org/cgi-bin/carddisp.pl?gene=GADD45A
GC01M165171	10.41	https://www.genecards.org/cgi-bin/carddisp.pl?gene=LMX1A
GC21M034513	10.4	https://www.genecards.org/cgi-bin/carddisp.pl?gene=RCAN1
GC01M244850	10.4	https://www.genecards.org/cgi-bin/carddisp.pl?gene=HNRNPU
GC17M017212	10.4	https://www.genecards.org/cgi-bin/carddisp.pl?gene=FLCN

GC11P070398	10.39	https://www.genecards.org/cgi-bin/carddisp.pl?gene=CTTN
GC03M138061	10.37	https://www.genecards.org/cgi-bin/carddisp.pl?gene=DZIP1L
GC17M012991	10.37	https://www.genecards.org/cgi-bin/carddisp.pl?gene=ELAC2
GC08M104489	10.36	https://www.genecards.org/cgi-bin/carddisp.pl?gene=LRP12
GC17P077282	10.34	https://www.genecards.org/cgi-bin/carddisp.pl?gene=SEPTIN9
GC07P141551	10.34	https://www.genecards.org/cgi-bin/carddisp.pl?gene=AGK
GC17M041619	10.34	https://www.genecards.org/cgi-bin/carddisp.pl?gene=KRT17
GC02P079120	10.34	https://www.genecards.org/cgi-bin/carddisp.pl?gene=REG1A
GC0XM053532	10.33	https://www.genecards.org/cgi-bin/carddisp.pl?gene=HUWE1
GC17P007950	10.32	https://www.genecards.org/cgi-bin/carddisp.pl?gene=WRAP53
GC05M055935	10.31	https://www.genecards.org/cgi-bin/carddisp.pl?gene=IL6ST
GC16P028891	10.31	https://www.genecards.org/cgi-bin/carddisp.pl?gene=ATP2A1
GC21P033324	10.31	https://www.genecards.org/cgi-bin/carddisp.pl?gene=IFNAR1
GC04P038872	10.3	https://www.genecards.org/cgi-bin/carddisp.pl?gene=MIR574
GC16P089919	10.29	https://www.genecards.org/cgi-bin/carddisp.pl?gene=TUBB3
GC05P119452	10.28	https://www.genecards.org/cgi-bin/carddisp.pl?gene=HSD17B4
GC10P102436	10.28	https://www.genecards.org/cgi-bin/carddisp.pl?gene=MIR146B
GC03M195863	10.28	https://www.genecards.org/cgi-bin/carddisp.pl?gene=TNK2
GC01M207802	10.28	https://www.genecards.org/cgi-bin/carddisp.pl?gene=MIR29C
GC08P027490	10.28	https://www.genecards.org/cgi-bin/carddisp.pl?gene=EPHX2
GC06M085607	10.27	https://www.genecards.org/cgi-bin/carddisp.pl?gene=SYNCRIP
GC08M060187	10.26	https://www.genecards.org/cgi-bin/carddisp.pl?gene=CA8
GC02P218125	10.25	https://www.genecards.org/cgi-bin/carddisp.pl?gene=CXCR2
GC03M050329	10.24	https://www.genecards.org/cgi-bin/carddisp.pl?gene=RASSF1
GC11M088113	10.24	https://www.genecards.org/cgi-bin/carddisp.pl?gene=RAB38
GC14P104252	10.23	https://www.genecards.org/cgi-bin/carddisp.pl?gene=MIR127
GC16M089744	10.23	https://www.genecards.org/cgi-bin/carddisp.pl?gene=FANCA
GC08M143816	10.23	https://www.genecards.org/cgi-bin/carddisp.pl?gene=PUF60
GC04M119494	10.21	https://www.genecards.org/cgi-bin/carddisp.pl?gene=PDE5A
GC09P004490	10.2	https://www.genecards.org/cgi-bin/carddisp.pl?gene=SLC1A1
GC17P031559	10.19	https://www.genecards.org/cgi-bin/carddisp.pl?gene=MIR193A
GC12P053452	10.19	https://www.genecards.org/cgi-bin/carddisp.pl?gene=PCBP2
GC05M172768	10.19	https://www.genecards.org/cgi-bin/carddisp.pl?gene=DUSP1
GC12P040692	10.18	https://www.genecards.org/cgi-bin/carddisp.pl?gene=CNTN1
GC19P050192	10.18	https://www.genecards.org/cgi-bin/carddisp.pl?gene=MYH14
GC21P033025	10.18	https://www.genecards.org/cgi-bin/carddisp.pl?gene=OLIG2
GC06P144285	10.17	https://www.genecards.org/cgi-bin/carddisp.pl?gene=UTRN
GC10P092689	10.16	https://www.genecards.org/cgi-bin/carddisp.pl?gene=HHEX
GC19M012676	10.15	https://www.genecards.org/cgi-bin/carddisp.pl?gene=DHPS
GC01P209432	10.13	https://www.genecards.org/cgi-bin/carddisp.pl?gene=MIR205
GC02P227473	10.12	https://www.genecards.org/cgi-bin/carddisp.pl?gene=AGFG1
GC06M005032	10.12	https://www.genecards.org/cgi-bin/carddisp.pl?gene=LYRM4
GC07M005431	10.11	https://www.genecards.org/cgi-bin/carddisp.pl?gene=FBXL18
GC17M064039	10.1	https://www.genecards.org/cgi-bin/carddisp.pl?gene=ERN1
GC11M063096	10.08	https://www.genecards.org/cgi-bin/carddisp.pl?gene=CHRM1
GC12M049002	10.08	https://www.genecards.org/cgi-bin/carddisp.pl?gene=PRKAG1
GC18P059220	10.08	https://www.genecards.org/cgi-bin/carddisp.pl?gene=GRP

GC10P092593	10.08	https://www.genecards.org/cgi-bin/carddisp.pl?gene=KIF11
GC17P080037	10.06	https://www.genecards.org/cgi-bin/carddisp.pl?gene=CCDC40
GC11M060366	10.05	https://www.genecards.org/cgi-bin/carddisp.pl?gene=MS4A6A
GC06P112053	10.05	https://www.genecards.org/cgi-bin/carddisp.pl?gene=CCN6
GC02P046293	10.04	https://www.genecards.org/cgi-bin/carddisp.pl?gene=EPAS1
GC01P179262	10.04	https://www.genecards.org/cgi-bin/carddisp.pl?gene=SOAT1
GC01P040258	10.04	https://www.genecards.org/cgi-bin/carddisp.pl?gene=ZMPSTE24
GC13P100089	10.04	https://www.genecards.org/cgi-bin/carddisp.pl?gene=PCCA
GC22M020234	10.04	https://www.genecards.org/cgi-bin/carddisp.pl?gene=RTN4R
GC19P051311	10.03	https://www.genecards.org/cgi-bin/carddisp.pl?gene=IGLON5
GC06M113933	10.03	https://www.genecards.org/cgi-bin/carddisp.pl?gene=HDAC2
GC14M060971	10.03	https://www.genecards.org/cgi-bin/carddisp.pl?gene=TRMT5
GC21M031118	10.03	https://www.genecards.org/cgi-bin/carddisp.pl?gene=TIAM1
GC21P046324	9.99	https://www.genecards.org/cgi-bin/carddisp.pl?gene=PCNT
GC19P041859	9.98	https://www.genecards.org/cgi-bin/carddisp.pl?gene=RPS19
GC07M092198	9.98	https://www.genecards.org/cgi-bin/carddisp.pl?gene=KRIT1
GC02P240435	9.98	https://www.genecards.org/cgi-bin/carddisp.pl?gene=GPC1
GCMTM005659	9.97	https://www.genecards.org/cgi-bin/carddisp.pl?gene=MT-TN
GC0XM020149	9.96	https://www.genecards.org/cgi-bin/carddisp.pl?gene=RPS6KA3
GC13P030713	9.96	https://www.genecards.org/cgi-bin/carddisp.pl?gene=ALOX5AP
GC20M003191	9.95	https://www.genecards.org/cgi-bin/carddisp.pl?gene=DDRGK1
GC15P071910	9.94	https://www.genecards.org/cgi-bin/carddisp.pl?gene=LACTB
GC15P073925	9.93	https://www.genecards.org/cgi-bin/carddisp.pl?gene=LOXL1
GC07P074082	9.93	https://www.genecards.org/cgi-bin/carddisp.pl?gene=LIMK1
GC11M017494	9.93	https://www.genecards.org/cgi-bin/carddisp.pl?gene=USH1C
GC03M053224	9.92	https://www.genecards.org/cgi-bin/carddisp.pl?gene=TKT
GC08M052622	9.91	https://www.genecards.org/cgi-bin/carddisp.pl?gene=RB1CC1
GC20M000472	9.91	https://www.genecards.org/cgi-bin/carddisp.pl?gene=CSNK2A1
GC01P019666	9.9	https://www.genecards.org/cgi-bin/carddisp.pl?gene=HTR6
GC03M048598	9.9	https://www.genecards.org/cgi-bin/carddisp.pl?gene=UQCRC1
GC06P101181	9.89	https://www.genecards.org/cgi-bin/carddisp.pl?gene=GRIK2
GC10P102918	9.88	https://www.genecards.org/cgi-bin/carddisp.pl?gene=CNNM2
GC10P083194	9.87	https://www.genecards.org/cgi-bin/carddisp.pl?gene=PPIF
GC02P172427	9.87	https://www.genecards.org/cgi-bin/carddisp.pl?gene=ITGA6
GC06P032741	9.86	https://www.genecards.org/cgi-bin/carddisp.pl?gene=HLA-DQA2
GC19P055654	9.86	https://www.genecards.org/cgi-bin/carddisp.pl?gene=U2AF2
GC09P130444	9.85	https://www.genecards.org/cgi-bin/carddisp.pl?gene=ASS1
GC04U902740	9.85	https://www.genecards.org/cgi-bin/carddisp.pl?gene=LOC109461479
GC04M052019	9.84	https://www.genecards.org/cgi-bin/carddisp.pl?gene=SGCB
GC02M038709	9.84	https://www.genecards.org/cgi-bin/carddisp.pl?gene=SRSF7
GC0XM134249	9.84	https://www.genecards.org/cgi-bin/carddisp.pl?gene=MIR106A
GC03M049716	9.84	https://www.genecards.org/cgi-bin/carddisp.pl?gene=GMPPB
GC0XM041514	9.84	https://www.genecards.org/cgi-bin/carddisp.pl?gene=CASK
GC07M038638	9.83	https://www.genecards.org/cgi-bin/carddisp.pl?gene=AMPH
GC10M070431	9.83	https://www.genecards.org/cgi-bin/carddisp.pl?gene=NODAL
GC09P033750	9.82	https://www.genecards.org/cgi-bin/carddisp.pl?gene=PRSS3
GC08M041238	9.82	https://www.genecards.org/cgi-bin/carddisp.pl?gene=SFRP1

GC17M043875	9.82	https://www.genecards.org/cgi-bin/carddisp.pl?gene=MPP2
GC06P031958	9.8	https://www.genecards.org/cgi-bin/carddisp.pl?gene=SKIV2L
GC20P049982	9.8	https://www.genecards.org/cgi-bin/carddisp.pl?gene=SNAI1
GC0XP024465	9.79	https://www.genecards.org/cgi-bin/carddisp.pl?gene=PDK3
GC22M019176	9.79	https://www.genecards.org/cgi-bin/carddisp.pl?gene=SLC25A1
GC20M050082	9.79	https://www.genecards.org/cgi-bin/carddisp.pl?gene=UBE2V1
GC02M157736	9.79	https://www.genecards.org/cgi-bin/carddisp.pl?gene=ACVR1
GC17P007282	9.79	https://www.genecards.org/cgi-bin/carddisp.pl?gene=SLC2A4
GC17M045435	9.77	https://www.genecards.org/cgi-bin/carddisp.pl?gene=PLEKHM1
GC01M023801	9.77	https://www.genecards.org/cgi-bin/carddisp.pl?gene=HMGCL
GC10M033177	9.76	https://www.genecards.org/cgi-bin/carddisp.pl?gene=NRP1
GC06M111555	9.76	https://www.genecards.org/cgi-bin/carddisp.pl?gene=TRAF3IP2
GC11M071428	9.76	https://www.genecards.org/cgi-bin/carddisp.pl?gene=DHCR7
GC01P092832	9.75	https://www.genecards.org/cgi-bin/carddisp.pl?gene=RPL5
GC12P010212	9.75	https://www.genecards.org/cgi-bin/carddisp.pl?gene=GABARAPL1
GC12P118135	9.75	https://www.genecards.org/cgi-bin/carddisp.pl?gene=PEBP1
GC02M074526	9.74	https://www.genecards.org/cgi-bin/carddisp.pl?gene=AUP1
GC13M037562	9.74	https://www.genecards.org/cgi-bin/carddisp.pl?gene=POSTN
GC10M077969	9.73	https://www.genecards.org/cgi-bin/carddisp.pl?gene=POLR3A
GC01P111449	9.73	https://www.genecards.org/cgi-bin/carddisp.pl?gene=ATP5PB
GC12M002857	9.72	https://www.genecards.org/cgi-bin/carddisp.pl?gene=FOXM1
GC06P033427	9.72	https://www.genecards.org/cgi-bin/carddisp.pl?gene=HSPA1B
GC02M237324	9.71	https://www.genecards.org/cgi-bin/carddisp.pl?gene=COL6A3
GC01M022190	9.71	https://www.genecards.org/cgi-bin/carddisp.pl?gene=WNT4
GC04P000932	9.7	https://www.genecards.org/cgi-bin/carddisp.pl?gene=TMEM175
GC08M094879	9.7	https://www.genecards.org/cgi-bin/carddisp.pl?gene=CCNE2
GC01M023845	9.69	https://www.genecards.org/cgi-bin/carddisp.pl?gene=FUCA1
GC0XM111293	9.69	https://www.genecards.org/cgi-bin/carddisp.pl?gene=DCX
GC20P000407	9.68	https://www.genecards.org/cgi-bin/carddisp.pl?gene=RBCK1
GC09M122132	9.68	https://www.genecards.org/cgi-bin/carddisp.pl?gene=NDUFA8
GC02P108719	9.67	https://www.genecards.org/cgi-bin/carddisp.pl?gene=RANBP2
GC10P022326	9.67	https://www.genecards.org/cgi-bin/carddisp.pl?gene=BMI1
GC22M050246	9.67	https://www.genecards.org/cgi-bin/carddisp.pl?gene=MAPK12
GC17P082752	9.66	https://www.genecards.org/cgi-bin/carddisp.pl?gene=TBCD
GC22M038778	9.66	https://www.genecards.org/cgi-bin/carddisp.pl?gene=DNAL4
GC19M045527	9.66	https://www.genecards.org/cgi-bin/carddisp.pl?gene=OPA3
GC05P076716	9.65	https://www.genecards.org/cgi-bin/carddisp.pl?gene=F2R
GC01M149903	9.65	https://www.genecards.org/cgi-bin/carddisp.pl?gene=SV2A
GC01P181317	9.63	https://www.genecards.org/cgi-bin/carddisp.pl?gene=CACNA1E
GC20M058818	9.63	https://www.genecards.org/cgi-bin/carddisp.pl?gene=MIR298
GC01M156308	9.63	https://www.genecards.org/cgi-bin/carddisp.pl?gene=CCT3
GC08M064587	9.62	https://www.genecards.org/cgi-bin/carddisp.pl?gene=CYP7B1
GC20M059026	9.61	https://www.genecards.org/cgi-bin/carddisp.pl?gene=ATP5F1E
GC01M154962	9.58	https://www.genecards.org/cgi-bin/carddisp.pl?gene=SHC1
GC05P056815	9.56	https://www.genecards.org/cgi-bin/carddisp.pl?gene=MAP3K1
GC17M081843	9.56	https://www.genecards.org/cgi-bin/carddisp.pl?gene=P4HB
GC01P054998	9.56	https://www.genecards.org/cgi-bin/carddisp.pl?gene=BSND

GC09M114060	9.55	https://www.genecards.org/cgi-bin/carddisp.pl?gene=AMBP
GC19P049157	9.55	https://www.genecards.org/cgi-bin/carddisp.pl?gene=TRPM4
GC11M068052	9.54	https://www.genecards.org/cgi-bin/carddisp.pl?gene=CHKA
GC01M161039	9.54	https://www.genecards.org/cgi-bin/carddisp.pl?gene=USF1
GC01P059296	9.54	https://www.genecards.org/cgi-bin/carddisp.pl?gene=FGGY
GC07M128392	9.54	https://www.genecards.org/cgi-bin/carddisp.pl?gene=IMPDH1
GC10M104129	9.53	https://www.genecards.org/cgi-bin/carddisp.pl?gene=CFAP43
GC07P075899	9.53	https://www.genecards.org/cgi-bin/carddisp.pl?gene=POR
GC02P074833	9.51	https://www.genecards.org/cgi-bin/carddisp.pl?gene=HK2
GC07P023105	9.48	https://www.genecards.org/cgi-bin/carddisp.pl?gene=KLHL7
GC0XM134214	9.47	https://www.genecards.org/cgi-bin/carddisp.pl?gene=MIR363
GC07M076327	9.46	https://www.genecards.org/cgi-bin/carddisp.pl?gene=YWHAG
GC01P094418	9.46	https://www.genecards.org/cgi-bin/carddisp.pl?gene=ABCD3
GC17M008377	9.46	https://www.genecards.org/cgi-bin/carddisp.pl?gene=RPL26
GC04M103586	9.46	https://www.genecards.org/cgi-bin/carddisp.pl?gene=TACR3
GC01M150673	9.46	https://www.genecards.org/cgi-bin/carddisp.pl?gene=MCL1
GC20M045898	9.46	https://www.genecards.org/cgi-bin/carddisp.pl?gene=PLTP
GC11M122152	9.46	https://www.genecards.org/cgi-bin/carddisp.pl?gene=MIR100
GC01P018631	9.45	https://www.genecards.org/cgi-bin/carddisp.pl?gene=PAX7
GC09M035088	9.45	https://www.genecards.org/cgi-bin/carddisp.pl?gene=PIGO
GC15P050424	9.44	https://www.genecards.org/cgi-bin/carddisp.pl?gene=USP8
GC18M020946	9.44	https://www.genecards.org/cgi-bin/carddisp.pl?gene=ROCK1
GC11M003000	9.43	https://www.genecards.org/cgi-bin/carddisp.pl?gene=CARS1
GC17M044852	9.43	https://www.genecards.org/cgi-bin/carddisp.pl?gene=EFTUD2
GC0XM154010	9.43	https://www.genecards.org/cgi-bin/carddisp.pl?gene=IRAK1
GC16P002537	9.43	https://www.genecards.org/cgi-bin/carddisp.pl?gene=PDPK1
GC11P066593	9.43	https://www.genecards.org/cgi-bin/carddisp.pl?gene=CCS
GC0XP041085	9.42	https://www.genecards.org/cgi-bin/carddisp.pl?gene=USP9X
GC05P168487	9.42	https://www.genecards.org/cgi-bin/carddisp.pl?gene=RARS1
GC01P164524	9.42	https://www.genecards.org/cgi-bin/carddisp.pl?gene=PBX1
GC04M099070	9.41	https://www.genecards.org/cgi-bin/carddisp.pl?gene=ADH5
GC12P065824	9.4	https://www.genecards.org/cgi-bin/carddisp.pl?gene=HMGA2
GC07M127610	9.39	https://www.genecards.org/cgi-bin/carddisp.pl?gene=PAX4
GCMTM005589	9.39	https://www.genecards.org/cgi-bin/carddisp.pl?gene=MT-TA
GC13M027436	9.39	https://www.genecards.org/cgi-bin/carddisp.pl?gene=MTIF3
GC19M018196	9.38	https://www.genecards.org/cgi-bin/carddisp.pl?gene=RAB3A
GC12P102957	9.38	https://www.genecards.org/cgi-bin/carddisp.pl?gene=ASCL1
GC11P102317	9.38	https://www.genecards.org/cgi-bin/carddisp.pl?gene=BIRC3
GC19P052189	9.37	https://www.genecards.org/cgi-bin/carddisp.pl?gene=PPP2R1A
GC09M115019	9.37	https://www.genecards.org/cgi-bin/carddisp.pl?gene=TNC
GC16P021963	9.36	https://www.genecards.org/cgi-bin/carddisp.pl?gene=UQCRC2
GC01P119660	9.34	https://www.genecards.org/cgi-bin/carddisp.pl?gene=PHGDH
GC09P073151	9.34	https://www.genecards.org/cgi-bin/carddisp.pl?gene=ANXA1
GC06M077481	9.34	https://www.genecards.org/cgi-bin/carddisp.pl?gene=HTR1B
GC02M189031	9.34	https://www.genecards.org/cgi-bin/carddisp.pl?gene=COL5A2
GC14M105854	9.34	https://www.genecards.org/cgi-bin/carddisp.pl?gene=IGHM
GC0XP021839	9.33	https://www.genecards.org/cgi-bin/carddisp.pl?gene=MBTPS2

GC02P206766	9.33	https://www.genecards.org/cgi-bin/carddisp.pl?gene=FASTKD2
GC12M120461	9.33	https://www.genecards.org/cgi-bin/carddisp.pl?gene=SRSF9
GC0XP071283	9.33	https://www.genecards.org/cgi-bin/carddisp.pl?gene=NONO
GC02M174747	9.32	https://www.genecards.org/cgi-bin/carddisp.pl?gene=CHRNA1
GC19M049659	9.31	https://www.genecards.org/cgi-bin/carddisp.pl?gene=IRF3
GC07M000497	9.31	https://www.genecards.org/cgi-bin/carddisp.pl?gene=PDGFA
GC07M030424	9.31	https://www.genecards.org/cgi-bin/carddisp.pl?gene=NOD1
GC14M100334	9.3	https://www.genecards.org/cgi-bin/carddisp.pl?gene=WARS1
GC07P143132	9.29	https://www.genecards.org/cgi-bin/carddisp.pl?gene=PIP
GC11M005340	9.29	https://www.genecards.org/cgi-bin/carddisp.pl?gene=HBG1
GC17P036948	9.28	https://www.genecards.org/cgi-bin/carddisp.pl?gene=AATF
GC10M080271	9.27	https://www.genecards.org/cgi-bin/carddisp.pl?gene=MAT1A
GC17P002054	9.26	https://www.genecards.org/cgi-bin/carddisp.pl?gene=HIC1
GC03P160404	9.26	https://www.genecards.org/cgi-bin/carddisp.pl?gene=MIR15B
GC10M076869	9.25	https://www.genecards.org/cgi-bin/carddisp.pl?gene=KCNMA1
GC09M035073	9.25	https://www.genecards.org/cgi-bin/carddisp.pl?gene=FANCG
GC02M068143	9.25	https://www.genecards.org/cgi-bin/carddisp.pl?gene=PPP3R1
GC17M017506	9.24	https://www.genecards.org/cgi-bin/carddisp.pl?gene=PEMT
GC07M100092	9.23	https://www.genecards.org/cgi-bin/carddisp.pl?gene=MCM7
GC10P122560	9.22	https://www.genecards.org/cgi-bin/carddisp.pl?gene=DMBT1
GC03M025718	9.22	https://www.genecards.org/cgi-bin/carddisp.pl?gene=NGLY1
GC05M131159	9.22	https://www.genecards.org/cgi-bin/carddisp.pl?gene=HINT1
GC05M083637	9.22	https://www.genecards.org/cgi-bin/carddisp.pl?gene=HAPLN1
GC14P020468	9.21	https://www.genecards.org/cgi-bin/carddisp.pl?gene=PNP
GC04M186533	9.21	https://www.genecards.org/cgi-bin/carddisp.pl?gene=MTNR1A
GC04P073869	9.21	https://www.genecards.org/cgi-bin/carddisp.pl?gene=CXCL1
GC15P078540	9.2	https://www.genecards.org/cgi-bin/carddisp.pl?gene=PSMA4
GC17P046239	9.2	https://www.genecards.org/cgi-bin/carddisp.pl?gene=LRRC37A
GC01M011054	9.2	https://www.genecards.org/cgi-bin/carddisp.pl?gene=SRM
GC0XM133300	9.19	https://www.genecards.org/cgi-bin/carddisp.pl?gene=GPC4
GC16P015663	9.18	https://www.genecards.org/cgi-bin/carddisp.pl?gene=NDE1
GC22P020034	9.18	https://www.genecards.org/cgi-bin/carddisp.pl?gene=MIR185
GC04M177430	9.17	https://www.genecards.org/cgi-bin/carddisp.pl?gene=AGA
GC03P010026	9.17	https://www.genecards.org/cgi-bin/carddisp.pl?gene=FANCD2
GC02M011181	9.17	https://www.genecards.org/cgi-bin/carddisp.pl?gene=ROCK2
GC01M025884	9.17	https://www.genecards.org/cgi-bin/carddisp.pl?gene=STMN1
GC13M080335	9.16	https://www.genecards.org/cgi-bin/carddisp.pl?gene=SPRY2
GC17P042567	9.16	https://www.genecards.org/cgi-bin/carddisp.pl?gene=MLX
GC14M075132	9.16	https://www.genecards.org/cgi-bin/carddisp.pl?gene=TMED10
GC10P007703	9.15	https://www.genecards.org/cgi-bin/carddisp.pl?gene=ITIH2
GC08M047773	9.14	https://www.genecards.org/cgi-bin/carddisp.pl?gene=PRKDC
GC17M082614	9.14	https://www.genecards.org/cgi-bin/carddisp.pl?gene=WDR45B
GC17M064854	9.14	https://www.genecards.org/cgi-bin/carddisp.pl?gene=LRRC37A3
GC09P094175	9.13	https://www.genecards.org/cgi-bin/carddisp.pl?gene=MIRLET7A1
GC17M075941	9.13	https://www.genecards.org/cgi-bin/carddisp.pl?gene=ACOX1
GC18M000309	9.13	https://www.genecards.org/cgi-bin/carddisp.pl?gene=COLEC12
GC07M101162	9.12	https://www.genecards.org/cgi-bin/carddisp.pl?gene=VGF

GC07P035828	9.12	https://www.genecards.org/cgi-bin/carddisp.pl?gene=SEPTIN7
GC02P216632	9.12	https://www.genecards.org/cgi-bin/carddisp.pl?gene=IGFBP2
GC02P238320	9.11	https://www.genecards.org/cgi-bin/carddisp.pl?gene=TRAF3IP1
GC14M074498	9.11	https://www.genecards.org/cgi-bin/carddisp.pl?gene=LTBP2
GC03M043121	9.11	https://www.genecards.org/cgi-bin/carddisp.pl?gene=POMGNT2
GC17P080544	9.1	https://www.genecards.org/cgi-bin/carddisp.pl?gene=RPTOR
GC03M142449	9.1	https://www.genecards.org/cgi-bin/carddisp.pl?gene=ATR
GC17M008761	9.1	https://www.genecards.org/cgi-bin/carddisp.pl?gene=PER1
GC03P037969	9.1	https://www.genecards.org/cgi-bin/carddisp.pl?gene=MIR26A1
GC19M014566	9.09	https://www.genecards.org/cgi-bin/carddisp.pl?gene=NDUFB7
GC06M030808	9.09	https://www.genecards.org/cgi-bin/carddisp.pl?gene=FLOT1
GC10P096832	9.09	https://www.genecards.org/cgi-bin/carddisp.pl?gene=LCOR
GC04M099276	9.09	https://www.genecards.org/cgi-bin/carddisp.pl?gene=ADH1A
GC04P026165	9.09	https://www.genecards.org/cgi-bin/carddisp.pl?gene=RBPJ
GC12U902742	9.08	https://www.genecards.org/cgi-bin/carddisp.pl?gene=LOC111258525
GC0XM101345	9.08	https://www.genecards.org/cgi-bin/carddisp.pl?gene=TIMM8A
GC01M159998	9.08	https://www.genecards.org/cgi-bin/carddisp.pl?gene=KCNJ10
GC07M100803	9.08	https://www.genecards.org/cgi-bin/carddisp.pl?gene=EPHB4
GC07M151466	9.07	https://www.genecards.org/cgi-bin/carddisp.pl?gene=RHEB
GC05P036876	9.07	https://www.genecards.org/cgi-bin/carddisp.pl?gene=NIPBL
GC13P091428	9.07	https://www.genecards.org/cgi-bin/carddisp.pl?gene=MIR19B1
GC01P063593	9.07	https://www.genecards.org/cgi-bin/carddisp.pl?gene=PGM1
GC03P153162	9.06	https://www.genecards.org/cgi-bin/carddisp.pl?gene=RAP2B
GC17M075751	9.06	https://www.genecards.org/cgi-bin/carddisp.pl?gene=GALK1
GC02P101991	9.05	https://www.genecards.org/cgi-bin/carddisp.pl?gene=IL1R2
GC12P054167	9.05	https://www.genecards.org/cgi-bin/carddisp.pl?gene=MIR196A2
GC14M064939	9.05	https://www.genecards.org/cgi-bin/carddisp.pl?gene=GPX2
GC10M103454	9.05	https://www.genecards.org/cgi-bin/carddisp.pl?gene=CALHM1
GC16P030700	9.05	https://www.genecards.org/cgi-bin/carddisp.pl?gene=SRCAP
GC22P037952	9.05	https://www.genecards.org/cgi-bin/carddisp.pl?gene=POLR2F
GC06M031809	9.05	https://www.genecards.org/cgi-bin/carddisp.pl?gene=HSPA1L
GC19M006426	9.04	https://www.genecards.org/cgi-bin/carddisp.pl?gene=SLC25A41
GC03P190322	9.04	https://www.genecards.org/cgi-bin/carddisp.pl?gene=CLDN16
GC20P034873	9.03	https://www.genecards.org/cgi-bin/carddisp.pl?gene=ACSS2
GC13P105465	9.03	https://www.genecards.org/cgi-bin/carddisp.pl?gene=DAOA
GC0XP070269	9.03	https://www.genecards.org/cgi-bin/carddisp.pl?gene=ARR3
GC05P138613	9.03	https://www.genecards.org/cgi-bin/carddisp.pl?gene=CTNNA1
GC02P148021	9.01	https://www.genecards.org/cgi-bin/carddisp.pl?gene=MBD5
GC07M010938	9	https://www.genecards.org/cgi-bin/carddisp.pl?gene=NDUFA4
GC11M064891	9	https://www.genecards.org/cgi-bin/carddisp.pl?gene=MIR192
GC08P093857	9	https://www.genecards.org/cgi-bin/carddisp.pl?gene=PDP1
GC01P158255	9	https://www.genecards.org/cgi-bin/carddisp.pl?gene=CD1A
GC05M140332	8.99	https://www.genecards.org/cgi-bin/carddisp.pl?gene=HBEGF
GC11M059680	8.99	https://www.genecards.org/cgi-bin/carddisp.pl?gene=LRP4
GC16M022357	8.99	https://www.genecards.org/cgi-bin/carddisp.pl?gene=CDR2
GC07P033112	8.99	https://www.genecards.org/cgi-bin/carddisp.pl?gene=BBS9
GC19M041470	8.99	https://www.genecards.org/cgi-bin/carddisp.pl?gene=COQ8B

GC02M201620	8.99	https://www.genecards.org/cgi-bin/carddisp.pl?gene=TMEM237
GC10P110919	8.99	https://www.genecards.org/cgi-bin/carddisp.pl?gene=SHOC2
GC21P041168	8.99	https://www.genecards.org/cgi-bin/carddisp.pl?gene=BACE2
GC03P100709	8.98	https://www.genecards.org/cgi-bin/carddisp.pl?gene=TFG
GC12M124776	8.97	https://www.genecards.org/cgi-bin/carddisp.pl?gene=SCARB1
GC09M032553	8.97	https://www.genecards.org/cgi-bin/carddisp.pl?gene=NDUFB6
GC14P077457	8.94	https://www.genecards.org/cgi-bin/carddisp.pl?gene=AHSA1
GC10M032900	8.94	https://www.genecards.org/cgi-bin/carddisp.pl?gene=ITGB1
GC03M015408	8.93	https://www.genecards.org/cgi-bin/carddisp.pl?gene=WNT7A
GC11M128458	8.92	https://www.genecards.org/cgi-bin/carddisp.pl?gene=ETS1
GC17M001494	8.92	https://www.genecards.org/cgi-bin/carddisp.pl?gene=INPP5K
GC04M055427	8.9	https://www.genecards.org/cgi-bin/carddisp.pl?gene=CLOCK
GC03P052350	8.9	https://www.genecards.org/cgi-bin/carddisp.pl?gene=DNAH1
GC03P139005	8.89	https://www.genecards.org/cgi-bin/carddisp.pl?gene=MRPS22
GC17P018720	8.89	https://www.genecards.org/cgi-bin/carddisp.pl?gene=SMCR8
GC20P002840	8.89	https://www.genecards.org/cgi-bin/carddisp.pl?gene=VPS16
GC14P075002	8.89	https://www.genecards.org/cgi-bin/carddisp.pl?gene=EIF2B2
GC19M012663	8.88	https://www.genecards.org/cgi-bin/carddisp.pl?gene=MAN2B1
GC02M096275	8.88	https://www.genecards.org/cgi-bin/carddisp.pl?gene=SNRNP200
GC0XM100843	8.87	https://www.genecards.org/cgi-bin/carddisp.pl?gene=NOX1
GC12M008602	8.87	https://www.genecards.org/cgi-bin/carddisp.pl?gene=AICDA
GC15P078565	8.86	https://www.genecards.org/cgi-bin/carddisp.pl?gene=CHRNA5
GC09P133061	8.86	https://www.genecards.org/cgi-bin/carddisp.pl?gene=CEL
GC11P112024	8.86	https://www.genecards.org/cgi-bin/carddisp.pl?gene=DLAT
GC20M062136	8.86	https://www.genecards.org/cgi-bin/carddisp.pl?gene=PSMA7
GC11M059698	8.85	https://www.genecards.org/cgi-bin/carddisp.pl?gene=CELF1
GC14P095533	8.85	https://www.genecards.org/cgi-bin/carddisp.pl?gene=GLRX5
GC04P094757	8.85	https://www.genecards.org/cgi-bin/carddisp.pl?gene=BMPR1B
GC01P039026	8.84	https://www.genecards.org/cgi-bin/carddisp.pl?gene=NDUFS5
GC06P085449	8.83	https://www.genecards.org/cgi-bin/carddisp.pl?gene=NT5E
GC20P064080	8.83	https://www.genecards.org/cgi-bin/carddisp.pl?gene=OPRL1
GC17M051630	8.83	https://www.genecards.org/cgi-bin/carddisp.pl?gene=CA10
GC01M156463	8.83	https://www.genecards.org/cgi-bin/carddisp.pl?gene=MEF2D
GC12M014768	8.83	https://www.genecards.org/cgi-bin/carddisp.pl?gene=H4-16
GC20P059020	8.83	https://www.genecards.org/cgi-bin/carddisp.pl?gene=TUBB1
GC08M099899	8.82	https://www.genecards.org/cgi-bin/carddisp.pl?gene=COX6C
GC14M021016	8.82	https://www.genecards.org/cgi-bin/carddisp.pl?gene=NDRG2
GC08P143017	8.81	https://www.genecards.org/cgi-bin/carddisp.pl?gene=LY6E
GC06M106969	8.81	https://www.genecards.org/cgi-bin/carddisp.pl?gene=CD24
GC16P031432	8.8	https://www.genecards.org/cgi-bin/carddisp.pl?gene=ITGAX
GC18P009904	8.8	https://www.genecards.org/cgi-bin/carddisp.pl?gene=VAPA
GC02M219001	8.79	https://www.genecards.org/cgi-bin/carddisp.pl?gene=MIR375
GC09M095099	8.79	https://www.genecards.org/cgi-bin/carddisp.pl?gene=FANCC
GC04M083292	8.78	https://www.genecards.org/cgi-bin/carddisp.pl?gene=HPSE
GC17P063827	8.78	https://www.genecards.org/cgi-bin/carddisp.pl?gene=PSMC5
GC13P021671	8.78	https://www.genecards.org/cgi-bin/carddisp.pl?gene=FGF9
GC11M014505	8.77	https://www.genecards.org/cgi-bin/carddisp.pl?gene=PSMA1

GC17P039980	8.77	https://www.genecards.org/cgi-bin/carddisp.pl?gene=PSMD3
GC16P057372	8.77	https://www.genecards.org/cgi-bin/carddisp.pl?gene=CX3CL1
GC01P045550	8.76	https://www.genecards.org/cgi-bin/carddisp.pl?gene=AKR1A1
GC15P051751	8.74	https://www.genecards.org/cgi-bin/carddisp.pl?gene=TMOD2
GC07M026706	8.74	https://www.genecards.org/cgi-bin/carddisp.pl?gene=SKAP2
GC06M041267	8.74	https://www.genecards.org/cgi-bin/carddisp.pl?gene=TREM1
GC11M065009	8.74	https://www.genecards.org/cgi-bin/carddisp.pl?gene=GPHA2
GC15M065148	8.73	https://www.genecards.org/cgi-bin/carddisp.pl?gene=CLPX
GC04P157076	8.73	https://www.genecards.org/cgi-bin/carddisp.pl?gene=GLRB
GC0XP048897	8.73	https://www.genecards.org/cgi-bin/carddisp.pl?gene=PQBP1
GC07M038730	8.73	https://www.genecards.org/cgi-bin/carddisp.pl?gene=VPS41
GC01M027622	8.73	https://www.genecards.org/cgi-bin/carddisp.pl?gene=FGR
GC09M069427	8.72	https://www.genecards.org/cgi-bin/carddisp.pl?gene=APBA1
GC19P018153	8.72	https://www.genecards.org/cgi-bin/carddisp.pl?gene=PIK3R2
GC02M043850	8.71	https://www.genecards.org/cgi-bin/carddisp.pl?gene=LRPPRC
GC14M029576	8.71	https://www.genecards.org/cgi-bin/carddisp.pl?gene=PRKD1
GC02P102294	8.71	https://www.genecards.org/cgi-bin/carddisp.pl?gene=IL1RL1
GC02P102418	8.71	https://www.genecards.org/cgi-bin/carddisp.pl?gene=IL18RAP
GC17M042404	8.71	https://www.genecards.org/cgi-bin/carddisp.pl?gene=CAVIN1
GC05P079991	8.7	https://www.genecards.org/cgi-bin/carddisp.pl?gene=THBS4
GC10M124397	8.69	https://www.genecards.org/cgi-bin/carddisp.pl?gene=OAT
GC20M054153	8.68	https://www.genecards.org/cgi-bin/carddisp.pl?gene=CYP24A1
GC01M235661	8.68	https://www.genecards.org/cgi-bin/carddisp.pl?gene=LYST
GC19M038878	8.68	https://www.genecards.org/cgi-bin/carddisp.pl?gene=SIRT2
GC12P055966	8.67	https://www.genecards.org/cgi-bin/carddisp.pl?gene=CDK2
GC01M153960	8.66	https://www.genecards.org/cgi-bin/carddisp.pl?gene=SLC39A1
GC01M017066	8.66	https://www.genecards.org/cgi-bin/carddisp.pl?gene=PADI2
GC14P103385	8.66	https://www.genecards.org/cgi-bin/carddisp.pl?gene=MARK3
GC11P006259	8.66	https://www.genecards.org/cgi-bin/carddisp.pl?gene=CCKBR
GC20M023374	8.66	https://www.genecards.org/cgi-bin/carddisp.pl?gene=NAPB
GC03P184155	8.65	https://www.genecards.org/cgi-bin/carddisp.pl?gene=DVL3
GC06M090513	8.64	https://www.genecards.org/cgi-bin/carddisp.pl?gene=MAP3K7
GC01P022652	8.64	https://www.genecards.org/cgi-bin/carddisp.pl?gene=C1QB
GC03P184298	8.64	https://www.genecards.org/cgi-bin/carddisp.pl?gene=PSMD2
GC19P003094	8.64	https://www.genecards.org/cgi-bin/carddisp.pl?gene=GNA11
GC17M082078	8.63	https://www.genecards.org/cgi-bin/carddisp.pl?gene=FASN
GC13P050909	8.63	https://www.genecards.org/cgi-bin/carddisp.pl?gene=RNASEH2B
GC0XP009463	8.63	https://www.genecards.org/cgi-bin/carddisp.pl?gene=TBL1X
GC07M025993	8.62	https://www.genecards.org/cgi-bin/carddisp.pl?gene=MIR148A
GC02M110637	8.61	https://www.genecards.org/cgi-bin/carddisp.pl?gene=BUB1
GC21P033542	8.61	https://www.genecards.org/cgi-bin/carddisp.pl?gene=SON
GC07M092112	8.61	https://www.genecards.org/cgi-bin/carddisp.pl?gene=CYP51A1
GC03M052268	8.61	https://www.genecards.org/cgi-bin/carddisp.pl?gene=MIRLET7G
GC10M003779	8.6	https://www.genecards.org/cgi-bin/carddisp.pl?gene=KLF6
GC04M088725	8.59	https://www.genecards.org/cgi-bin/carddisp.pl?gene=FAM13A
GC12P101568	8.59	https://www.genecards.org/cgi-bin/carddisp.pl?gene=MYBPC1
GC03M113716	8.59	https://www.genecards.org/cgi-bin/carddisp.pl?gene=NAA50

GC14M021210	8.59	https://www.genecards.org/cgi-bin/carddisp.pl?gene=HNRNPC
GC16P056231	8.59	https://www.genecards.org/cgi-bin/carddisp.pl?gene=GNAO1
GC17P036534	8.59	https://www.genecards.org/cgi-bin/carddisp.pl?gene=PIGW
GC10P114044	8.58	https://www.genecards.org/cgi-bin/carddisp.pl?gene=ADRB1
GC12M122208	8.57	https://www.genecards.org/cgi-bin/carddisp.pl?gene=DIABLO
GC14M105824	8.57	https://www.genecards.org/cgi-bin/carddisp.pl?gene=IGH
GC14M060643	8.56	https://www.genecards.org/cgi-bin/carddisp.pl?gene=SIX1
GC19M003976	8.56	https://www.genecards.org/cgi-bin/carddisp.pl?gene=EEF2
GC01M109603	8.55	https://www.genecards.org/cgi-bin/carddisp.pl?gene=GNAT2
GC03P011113	8.55	https://www.genecards.org/cgi-bin/carddisp.pl?gene=HRH1
GC01P115641	8.54	https://www.genecards.org/cgi-bin/carddisp.pl?gene=VANGL1
GC01P103525	8.54	https://www.genecards.org/cgi-bin/carddisp.pl?gene=RNPC3
GC01M092474	8.54	https://www.genecards.org/cgi-bin/carddisp.pl?gene=GFI1
GC04P106315	8.54	https://www.genecards.org/cgi-bin/carddisp.pl?gene=AIMP1
GC06M024171	8.53	https://www.genecards.org/cgi-bin/carddisp.pl?gene=DCDC2
GC21M045493	8.53	https://www.genecards.org/cgi-bin/carddisp.pl?gene=SLC19A1
GC01M153535	8.53	https://www.genecards.org/cgi-bin/carddisp.pl?gene=S100A6
GC12P106357	8.52	https://www.genecards.org/cgi-bin/carddisp.pl?gene=POLR3B
GC17P007834	8.52	https://www.genecards.org/cgi-bin/carddisp.pl?gene=KDM6B
GC17M040360	8.52	https://www.genecards.org/cgi-bin/carddisp.pl?gene=GJD3
GC13M050048	8.51	https://www.genecards.org/cgi-bin/carddisp.pl?gene=MIR16-1
GC10P127549	8.51	https://www.genecards.org/cgi-bin/carddisp.pl?gene=NPS
GC07M037912	8.51	https://www.genecards.org/cgi-bin/carddisp.pl?gene=SFRP4
GC04M154232	8.5	https://www.genecards.org/cgi-bin/carddisp.pl?gene=DCHS2
GC01P052927	8.5	https://www.genecards.org/cgi-bin/carddisp.pl?gene=SCP2
GC07M030496	8.49	https://www.genecards.org/cgi-bin/carddisp.pl?gene=GGCT
GC06M170282	8.49	https://www.genecards.org/cgi-bin/carddisp.pl?gene=DLL1
GC16M088810	8.49	https://www.genecards.org/cgi-bin/carddisp.pl?gene=APRT
GC09M098731	8.48	https://www.genecards.org/cgi-bin/carddisp.pl?gene=ANKS6
GC0XP154389	8.48	https://www.genecards.org/cgi-bin/carddisp.pl?gene=RPL10
GC12P056043	8.47	https://www.genecards.org/cgi-bin/carddisp.pl?gene=RPS26
GC14M062699	8.47	https://www.genecards.org/cgi-bin/carddisp.pl?gene=KCNH5
GC04P089879	8.47	https://www.genecards.org/cgi-bin/carddisp.pl?gene=MMRN1
GC14P090256	8.46	https://www.genecards.org/cgi-bin/carddisp.pl?gene=PSMC1
GC08M123013	8.46	https://www.genecards.org/cgi-bin/carddisp.pl?gene=DERL1
GC07P069598	8.46	https://www.genecards.org/cgi-bin/carddisp.pl?gene=AUTS2
GC19P050385	8.46	https://www.genecards.org/cgi-bin/carddisp.pl?gene=POLD1
GC17M082829	8.45	https://www.genecards.org/cgi-bin/carddisp.pl?gene=ZNF750
GC02M020200	8.45	https://www.genecards.org/cgi-bin/carddisp.pl?gene=SDC1
GC12M056449	8.44	https://www.genecards.org/cgi-bin/carddisp.pl?gene=MIP
GC04M089236	8.44	https://www.genecards.org/cgi-bin/carddisp.pl?gene=GPRIN3
GC01M109399	8.44	https://www.genecards.org/cgi-bin/carddisp.pl?gene=PSMA5
GC06M029556	8.44	https://www.genecards.org/cgi-bin/carddisp.pl?gene=UBD
GC16P001351	8.43	https://www.genecards.org/cgi-bin/carddisp.pl?gene=GNPTG
GC06M138903	8.43	https://www.genecards.org/cgi-bin/carddisp.pl?gene=REPS1
GC07P023182	8.42	https://www.genecards.org/cgi-bin/carddisp.pl?gene=NUP42
GC08P103298	8.42	https://www.genecards.org/cgi-bin/carddisp.pl?gene=FZD6

GC01M155112	8.42	https://www.genecards.org/cgi-bin/carddisp.pl?gene=DPM3
GC15P096325	8.41	https://www.genecards.org/cgi-bin/carddisp.pl?gene=NR2F2
GC06P157981	8.41	https://www.genecards.org/cgi-bin/carddisp.pl?gene=SYNJ2
GC02M190189	8.41	https://www.genecards.org/cgi-bin/carddisp.pl?gene=HIBCH
GC06M170535	8.41	https://www.genecards.org/cgi-bin/carddisp.pl?gene=PSMB1
GC02M191950	8.4	https://www.genecards.org/cgi-bin/carddisp.pl?gene=TMEFF2
GC08M094127	8.4	https://www.genecards.org/cgi-bin/carddisp.pl?gene=CDH17
GC19M005691	8.39	https://www.genecards.org/cgi-bin/carddisp.pl?gene=LONP1
GC06M052977	8.39	https://www.genecards.org/cgi-bin/carddisp.pl?gene=GSTA4
GC03M048676	8.39	https://www.genecards.org/cgi-bin/carddisp.pl?gene=NCKIPSD
GC11M077321	8.39	https://www.genecards.org/cgi-bin/carddisp.pl?gene=PAK1
GC11M089324	8.38	https://www.genecards.org/cgi-bin/carddisp.pl?gene=NOX4
GC02P026935	8.38	https://www.genecards.org/cgi-bin/carddisp.pl?gene=MAPRE3
GC02P073892	8.38	https://www.genecards.org/cgi-bin/carddisp.pl?gene=ACTG2
GC01M029147	8.37	https://www.genecards.org/cgi-bin/carddisp.pl?gene=SRSF4
GC15P089243	8.36	https://www.genecards.org/cgi-bin/carddisp.pl?gene=FANCI
GC03M197042	8.36	https://www.genecards.org/cgi-bin/carddisp.pl?gene=DLG1
GC01M009729	8.36	https://www.genecards.org/cgi-bin/carddisp.pl?gene=CLSTN1
GC22P037675	8.35	https://www.genecards.org/cgi-bin/carddisp.pl?gene=LGALS1
GC20M049503	8.35	https://www.genecards.org/cgi-bin/carddisp.pl?gene=PTGIS
GC09P133348	8.34	https://www.genecards.org/cgi-bin/carddisp.pl?gene=RPL7A
GC06M112107	8.34	https://www.genecards.org/cgi-bin/carddisp.pl?gene=LAMA4
GC17M074862	8.34	https://www.genecards.org/cgi-bin/carddisp.pl?gene=FDXR
GC01M016124	8.33	https://www.genecards.org/cgi-bin/carddisp.pl?gene=EPHA2
GC19M040843	8.32	https://www.genecards.org/cgi-bin/carddisp.pl?gene=CYP2A6
GC10M030012	8.31	https://www.genecards.org/cgi-bin/carddisp.pl?gene=JCAD
GC16M028511	8.31	https://www.genecards.org/cgi-bin/carddisp.pl?gene=IL27
GC04P053383	8.31	https://www.genecards.org/cgi-bin/carddisp.pl?gene=FIP1L1
GC11M119355	8.3	https://www.genecards.org/cgi-bin/carddisp.pl?gene=USP2
GC22P039519	8.3	https://www.genecards.org/cgi-bin/carddisp.pl?gene=ATF4
GC05M098853	8.3	https://www.genecards.org/cgi-bin/carddisp.pl?gene=CHD1
GC03M185643	8.3	https://www.genecards.org/cgi-bin/carddisp.pl?gene=IGF2BP2
GC07M022889	8.3	https://www.genecards.org/cgi-bin/carddisp.pl?gene=FAM126A
GC11P077066	8.3	https://www.genecards.org/cgi-bin/carddisp.pl?gene=CAPN5
GC19P000571	8.29	https://www.genecards.org/cgi-bin/carddisp.pl?gene=BSG
GC19P001103	8.29	https://www.genecards.org/cgi-bin/carddisp.pl?gene=GPX4
GC09M091564	8.29	https://www.genecards.org/cgi-bin/carddisp.pl?gene=ROR2
GC03P138347	8.29	https://www.genecards.org/cgi-bin/carddisp.pl?gene=MRAS
GC03M180983	8.29	https://www.genecards.org/cgi-bin/carddisp.pl?gene=DNAJC19
GC13M024882	8.29	https://www.genecards.org/cgi-bin/carddisp.pl?gene=CENPJ
GC11M068754	8.28	https://www.genecards.org/cgi-bin/carddisp.pl?gene=CPT1A
GC04M112513	8.28	https://www.genecards.org/cgi-bin/carddisp.pl?gene=NEUROG2
GC22M050263	8.28	https://www.genecards.org/cgi-bin/carddisp.pl?gene=MAPK11
GC09M098288	8.28	https://www.genecards.org/cgi-bin/carddisp.pl?gene=GABBR2
GC0XP119468	8.28	https://www.genecards.org/cgi-bin/carddisp.pl?gene=SLC25A5
GC10P078033	8.28	https://www.genecards.org/cgi-bin/carddisp.pl?gene=RPS24
GC01M064833	8.27	https://www.genecards.org/cgi-bin/carddisp.pl?gene=JAK1

GC05M095463	8.27	https://www.genecards.org/cgi-bin/carddisp.pl?gene=TTC37
GC06P127266	8.27	https://www.genecards.org/cgi-bin/carddisp.pl?gene=RNF146
GC16M028853	8.26	https://www.genecards.org/cgi-bin/carddisp.pl?gene=TUFM
GC10M060026	8.26	https://www.genecards.org/cgi-bin/carddisp.pl?gene=ANK3
GC11P046621	8.26	https://www.genecards.org/cgi-bin/carddisp.pl?gene=ATG13
GC14P052707	8.25	https://www.genecards.org/cgi-bin/carddisp.pl?gene=PSMC6
GC05M006599	8.25	https://www.genecards.org/cgi-bin/carddisp.pl?gene=NSUN2
GC0XM153947	8.24	https://www.genecards.org/cgi-bin/carddisp.pl?gene=HCFC1
GC07P144806	8.24	https://www.genecards.org/cgi-bin/carddisp.pl?gene=MGAM
GC16P004641	8.24	https://www.genecards.org/cgi-bin/carddisp.pl?gene=MGRN1
GC07M077310	8.24	https://www.genecards.org/cgi-bin/carddisp.pl?gene=GSAP
GC17P038752	8.23	https://www.genecards.org/cgi-bin/carddisp.pl?gene=PSMB3
GC19P012891	8.23	https://www.genecards.org/cgi-bin/carddisp.pl?gene=GCDH
GC01P155308	8.22	https://www.genecards.org/cgi-bin/carddisp.pl?gene=FDPS
GC14M023016	8.22	https://www.genecards.org/cgi-bin/carddisp.pl?gene=PSMB5
GC01P109548	8.22	https://www.genecards.org/cgi-bin/carddisp.pl?gene=GNAI3
GC15M072199	8.21	https://www.genecards.org/cgi-bin/carddisp.pl?gene=PKM
GC06P144150	8.21	https://www.genecards.org/cgi-bin/carddisp.pl?gene=STX11
GC10M035046	8.21	https://www.genecards.org/cgi-bin/carddisp.pl?gene=CUL2
GC01P162631	8.21	https://www.genecards.org/cgi-bin/carddisp.pl?gene=DDR2
GC04M076033	8.21	https://www.genecards.org/cgi-bin/carddisp.pl?gene=CXCL11
GC10P111077	8.21	https://www.genecards.org/cgi-bin/carddisp.pl?gene=ADRA2A
GC15P040161	8.2	https://www.genecards.org/cgi-bin/carddisp.pl?gene=BUB1B
GC20P002276	8.2	https://www.genecards.org/cgi-bin/carddisp.pl?gene=TGM3
GC06P046048	8.2	https://www.genecards.org/cgi-bin/carddisp.pl?gene=MAPK13
GC02P037231	8.2	https://www.genecards.org/cgi-bin/carddisp.pl?gene=NDUFAF7
GC10P110567	8.2	https://www.genecards.org/cgi-bin/carddisp.pl?gene=SMC3
GC07P114086	8.2	https://www.genecards.org/cgi-bin/carddisp.pl?gene=FOXP2
GC08M127207	8.19	https://www.genecards.org/cgi-bin/carddisp.pl?gene=CCAT1
GC22M044559	8.19	https://www.genecards.org/cgi-bin/carddisp.pl?gene=RPS19BP1
GC03P028575	8.19	https://www.genecards.org/cgi-bin/carddisp.pl?gene=RBMS3
GC06P149749	8.18	https://www.genecards.org/cgi-bin/carddisp.pl?gene=PCMT1
GC01P231241	8.18	https://www.genecards.org/cgi-bin/carddisp.pl?gene=GNPAT
GC18P058044	8.18	https://www.genecards.org/cgi-bin/carddisp.pl?gene=NEDD4L
GC02P216107	8.18	https://www.genecards.org/cgi-bin/carddisp.pl?gene=XRCC5
GC16M071565	8.18	https://www.genecards.org/cgi-bin/carddisp.pl?gene=TAT
GC17P060149	8.17	https://www.genecards.org/cgi-bin/carddisp.pl?gene=CA4
GC14P088384	8.17	https://www.genecards.org/cgi-bin/carddisp.pl?gene=SPATA7
GC16P002204	8.17	https://www.genecards.org/cgi-bin/carddisp.pl?gene=MLST8
GC10M003138	8.17	https://www.genecards.org/cgi-bin/carddisp.pl?gene=PITRM1
GC03M185914	8.16	https://www.genecards.org/cgi-bin/carddisp.pl?gene=TRA2B
GC01P170501	8.16	https://www.genecards.org/cgi-bin/carddisp.pl?gene=GORAB
GC19M007689	8.15	https://www.genecards.org/cgi-bin/carddisp.pl?gene=FCER2
GC03P009649	8.15	https://www.genecards.org/cgi-bin/carddisp.pl?gene=MTMR14
GC01P023691	8.15	https://www.genecards.org/cgi-bin/carddisp.pl?gene=RPL11
GC06P007107	8.15	https://www.genecards.org/cgi-bin/carddisp.pl?gene=RREB1
GC22M019450	8.15	https://www.genecards.org/cgi-bin/carddisp.pl?gene=UFD1

GC11M059719	8.15	https://www.genecards.org/cgi-bin/carddisp.pl?gene=NUP160
GC01P153991	8.14	https://www.genecards.org/cgi-bin/carddisp.pl?gene=RPS27
GC11M000628	8.14	https://www.genecards.org/cgi-bin/carddisp.pl?gene=SCT
GC01P158289	8.13	https://www.genecards.org/cgi-bin/carddisp.pl?gene=CD1C
GC16M030378	8.13	https://www.genecards.org/cgi-bin/carddisp.pl?gene=SEPTIN1
GC01M052684	8.13	https://www.genecards.org/cgi-bin/carddisp.pl?gene=COA7
GC20M033675	8.12	https://www.genecards.org/cgi-bin/carddisp.pl?gene=E2F1
GC11P094544	8.11	https://www.genecards.org/cgi-bin/carddisp.pl?gene=FUT4
GC10M089205	8.1	https://www.genecards.org/cgi-bin/carddisp.pl?gene=CH25H
GC06M041289	8.09	https://www.genecards.org/cgi-bin/carddisp.pl?gene=FKBP5
GC06P012717	8.09	https://www.genecards.org/cgi-bin/carddisp.pl?gene=PHACTR1
GC02P063557	8.09	https://www.genecards.org/cgi-bin/carddisp.pl?gene=MDH1
GC09M092458	8.08	https://www.genecards.org/cgi-bin/carddisp.pl?gene=ASPN
GC06M142751	8.07	https://www.genecards.org/cgi-bin/carddisp.pl?gene=HIVEP2
GC07P101154	8.07	https://www.genecards.org/cgi-bin/carddisp.pl?gene=AP1S1
GC07M042916	8.06	https://www.genecards.org/cgi-bin/carddisp.pl?gene=PSMA2
GC0XP077899	8.06	https://www.genecards.org/cgi-bin/carddisp.pl?gene=COX7B
GC17M042795	8.05	https://www.genecards.org/cgi-bin/carddisp.pl?gene=COA3
GC03P180989	8.04	https://www.genecards.org/cgi-bin/carddisp.pl?gene=SOX2-OT
GC08P142670	8.04	https://www.genecards.org/cgi-bin/carddisp.pl?gene=PSCA
GC05P010236	8.04	https://www.genecards.org/cgi-bin/carddisp.pl?gene=CCT5
GC17M076734	8.04	https://www.genecards.org/cgi-bin/carddisp.pl?gene=SRSF2
GC19M002425	8.04	https://www.genecards.org/cgi-bin/carddisp.pl?gene=TIMM13
GC11P013276	8.04	https://www.genecards.org/cgi-bin/carddisp.pl?gene=ARNTL
GC08P079610	8.04	https://www.genecards.org/cgi-bin/carddisp.pl?gene=STMN2
GC01P149390	8.03	https://www.genecards.org/cgi-bin/carddisp.pl?gene=NOTCH2NLC
GC09M034551	8.03	https://www.genecards.org/cgi-bin/carddisp.pl?gene=CNTFR
GC17M058345	8.02	https://www.genecards.org/cgi-bin/carddisp.pl?gene=SUPT4H1
GC0XP070444	8.02	https://www.genecards.org/cgi-bin/carddisp.pl?gene=DLG3
GC01P023019	8.02	https://www.genecards.org/cgi-bin/carddisp.pl?gene=KDM1A
GC19P044945	8	https://www.genecards.org/cgi-bin/carddisp.pl?gene=APOC2
GC17P049132	7.99	https://www.genecards.org/cgi-bin/carddisp.pl?gene=B4GALNT2
GC04M015606	7.99	https://www.genecards.org/cgi-bin/carddisp.pl?gene=FBXL5
GC14M064746	7.99	https://www.genecards.org/cgi-bin/carddisp.pl?gene=SPTB
GC09P111896	7.98	https://www.genecards.org/cgi-bin/carddisp.pl?gene=UGCG
GC01M109733	7.97	https://www.genecards.org/cgi-bin/carddisp.pl?gene=GSTM3
GC08P011795	7.97	https://www.genecards.org/cgi-bin/carddisp.pl?gene=FDFT1
GC09M092414	7.97	https://www.genecards.org/cgi-bin/carddisp.pl?gene=OMD
GC08M056067	7.96	https://www.genecards.org/cgi-bin/carddisp.pl?gene=RPS20
GC01P169367	7.96	https://www.genecards.org/cgi-bin/carddisp.pl?gene=BLZF1
GC16U902451	7.95	https://www.genecards.org/cgi-bin/carddisp.pl?gene=LOC109029536
GC05M126541	7.95	https://www.genecards.org/cgi-bin/carddisp.pl?gene=ALDH7A1
GC19M018568	7.95	https://www.genecards.org/cgi-bin/carddisp.pl?gene=CRLF1
GC04M026483	7.95	https://www.genecards.org/cgi-bin/carddisp.pl?gene=CCKAR
GC03P129974	7.94	https://www.genecards.org/cgi-bin/carddisp.pl?gene=TRH
GC04P144645	7.94	https://www.genecards.org/cgi-bin/carddisp.pl?gene=HHIP
GC04P001872	7.93	https://www.genecards.org/cgi-bin/carddisp.pl?gene=NSD2

GC11P064609	7.93	https://www.genecards.org/cgi-bin/carddisp.pl?gene=SLC22A12
GC10M103872	7.93	https://www.genecards.org/cgi-bin/carddisp.pl?gene=STN1
GC02P000254	7.93	https://www.genecards.org/cgi-bin/carddisp.pl?gene=ACP1
GC11P063700	7.93	https://www.genecards.org/cgi-bin/carddisp.pl?gene=RTN3
GC04P102868	7.92	https://www.genecards.org/cgi-bin/carddisp.pl?gene=CISD2
GC06M013266	7.91	https://www.genecards.org/cgi-bin/carddisp.pl?gene=TBC1D7
GC01M091260	7.91	https://www.genecards.org/cgi-bin/carddisp.pl?gene=HFM1
GC19M050996	7.91	https://www.genecards.org/cgi-bin/carddisp.pl?gene=KLK8
GC02P231056	7.91	https://www.genecards.org/cgi-bin/carddisp.pl?gene=PSMD1
GC06P043243	7.9	https://www.genecards.org/cgi-bin/carddisp.pl?gene=TTBK1
GC05M179614	7.89	https://www.genecards.org/cgi-bin/carddisp.pl?gene=HNRNPH1
GC17M061942	7.89	https://www.genecards.org/cgi-bin/carddisp.pl?gene=MED13
GC20P031637	7.89	https://www.genecards.org/cgi-bin/carddisp.pl?gene=COX4I2
GC06M053497	7.89	https://www.genecards.org/cgi-bin/carddisp.pl?gene=GCLC
GC02P171522	7.88	https://www.genecards.org/cgi-bin/carddisp.pl?gene=CYBRD1
GC17M081126	7.88	https://www.genecards.org/cgi-bin/carddisp.pl?gene=MIR338
GC17M037084	7.88	https://www.genecards.org/cgi-bin/carddisp.pl?gene=ACACA
GC04P101411	7.88	https://www.genecards.org/cgi-bin/carddisp.pl?gene=BANK1
GC01M204190	7.88	https://www.genecards.org/cgi-bin/carddisp.pl?gene=KISS1
GC08M100917	7.88	https://www.genecards.org/cgi-bin/carddisp.pl?gene=YWHAZ
GC16M066803	7.88	https://www.genecards.org/cgi-bin/carddisp.pl?gene=NAE1
GC14M068874	7.87	https://www.genecards.org/cgi-bin/carddisp.pl?gene=ACTN1
GC12M098726	7.86	https://www.genecards.org/cgi-bin/carddisp.pl?gene=ANKS1B
GC07M123536	7.86	https://www.genecards.org/cgi-bin/carddisp.pl?gene=NDUFA5
GC03M052812	7.86	https://www.genecards.org/cgi-bin/carddisp.pl?gene=ITIH4
GC01P154983	7.86	https://www.genecards.org/cgi-bin/carddisp.pl?gene=FLAD1
GC12M008535	7.86	https://www.genecards.org/cgi-bin/carddisp.pl?gene=CLEC4E
GC08M026504	7.85	https://www.genecards.org/cgi-bin/carddisp.pl?gene=PNMA2
GC14P099684	7.85	https://www.genecards.org/cgi-bin/carddisp.pl?gene=CYP46A1
GC04M158709	7.84	https://www.genecards.org/cgi-bin/carddisp.pl?gene=PPID
GC03M049417	7.83	https://www.genecards.org/cgi-bin/carddisp.pl?gene=AMT
GC01P236686	7.83	https://www.genecards.org/cgi-bin/carddisp.pl?gene=ACTN2
GC21P043719	7.83	https://www.genecards.org/cgi-bin/carddisp.pl?gene=PDXK
GC17P039667	7.83	https://www.genecards.org/cgi-bin/carddisp.pl?gene=PNMT
GC14P058244	7.82	https://www.genecards.org/cgi-bin/carddisp.pl?gene=PSMA3
GC14P045135	7.82	https://www.genecards.org/cgi-bin/carddisp.pl?gene=FANCM
GC11M059789	7.82	https://www.genecards.org/cgi-bin/carddisp.pl?gene=UBE2L6
GC01P203090	7.81	https://www.genecards.org/cgi-bin/carddisp.pl?gene=ADORA1
GC19P058544	7.81	https://www.genecards.org/cgi-bin/carddisp.pl?gene=TRIM28
GC13M023730	7.81	https://www.genecards.org/cgi-bin/carddisp.pl?gene=MIPEP
GC19P048764	7.8	https://www.genecards.org/cgi-bin/carddisp.pl?gene=FGF21
GC19P007903	7.8	https://www.genecards.org/cgi-bin/carddisp.pl?gene=MAP2K7
GC03P113747	7.79	https://www.genecards.org/cgi-bin/carddisp.pl?gene=ATP6V1A
GC10M091628	7.79	https://www.genecards.org/cgi-bin/carddisp.pl?gene=PPP1R3C
GC08M041653	7.78	https://www.genecards.org/cgi-bin/carddisp.pl?gene=ANK1
GC17P041812	7.78	https://www.genecards.org/cgi-bin/carddisp.pl?gene=FKBP10
GC03M196987	7.77	https://www.genecards.org/cgi-bin/carddisp.pl?gene=MELTF

GC07M129788	7.77	https://www.genecards.org/cgi-bin/carddisp.pl?gene=MIR183
GC12P008455	7.77	https://www.genecards.org/cgi-bin/carddisp.pl?gene=CLEC6A
GC05P076818	7.77	https://www.genecards.org/cgi-bin/carddisp.pl?gene=F2RL1
GC03P012020	7.76	https://www.genecards.org/cgi-bin/carddisp.pl?gene=SYN2
GC20P038346	7.76	https://www.genecards.org/cgi-bin/carddisp.pl?gene=LBP
GC16M023582	7.76	https://www.genecards.org/cgi-bin/carddisp.pl?gene=NDUFAB1
GC02M219209	7.76	https://www.genecards.org/cgi-bin/carddisp.pl?gene=ABCB6
GC19M041956	7.76	https://www.genecards.org/cgi-bin/carddisp.pl?gene=RABAC1
GC08M140657	7.75	https://www.genecards.org/cgi-bin/carddisp.pl?gene=PTK2
GC17P045052	7.74	https://www.genecards.org/cgi-bin/carddisp.pl?gene=NMT1
GC07P091940	7.74	https://www.genecards.org/cgi-bin/carddisp.pl?gene=AKAP9
GC06M055728	7.74	https://www.genecards.org/cgi-bin/carddisp.pl?gene=BMP5
GC19P007637	7.74	https://www.genecards.org/cgi-bin/carddisp.pl?gene=STXBP2
GC15M090998	7.73	https://www.genecards.org/cgi-bin/carddisp.pl?gene=VPS33B
GC05M180234	7.73	https://www.genecards.org/cgi-bin/carddisp.pl?gene=MAPK9
GC03M048869	7.73	https://www.genecards.org/cgi-bin/carddisp.pl?gene=SLC25A20
GC13M047745	7.73	https://www.genecards.org/cgi-bin/carddisp.pl?gene=SUCLA2
GC01M001254	7.72	https://www.genecards.org/cgi-bin/carddisp.pl?gene=UBE2J2
GC06M158109	7.71	https://www.genecards.org/cgi-bin/carddisp.pl?gene=SERAC1
GC01M035599	7.71	https://www.genecards.org/cgi-bin/carddisp.pl?gene=PSMB2
GC08M053227	7.71	https://www.genecards.org/cgi-bin/carddisp.pl?gene=OPRK1
GC0XM013336	7.71	https://www.genecards.org/cgi-bin/carddisp.pl?gene=ATXN3L
GC03P194136	7.7	https://www.genecards.org/cgi-bin/carddisp.pl?gene=HES1
GC09M134030	7.7	https://www.genecards.org/cgi-bin/carddisp.pl?gene=BRD3
GC06P117675	7.7	https://www.genecards.org/cgi-bin/carddisp.pl?gene=NUS1
GC12M021635	7.69	https://www.genecards.org/cgi-bin/carddisp.pl?gene=LDHB
GC18M074250	7.69	https://www.genecards.org/cgi-bin/carddisp.pl?gene=CYB5A
GC12P130871	7.69	https://www.genecards.org/cgi-bin/carddisp.pl?gene=RAN
GC09M069013	7.68	https://www.genecards.org/cgi-bin/carddisp.pl?gene=PRKACG
GC14P096205	7.68	https://www.genecards.org/cgi-bin/carddisp.pl?gene=BDKRB2
GC02P027217	7.68	https://www.genecards.org/cgi-bin/carddisp.pl?gene=CAD
GC10M103088	7.68	https://www.genecards.org/cgi-bin/carddisp.pl?gene=NT5C2
GC03P108823	7.68	https://www.genecards.org/cgi-bin/carddisp.pl?gene=TRAT1
GC10P058334	7.67	https://www.genecards.org/cgi-bin/carddisp.pl?gene=UBE2D1
GC0XM109624	7.66	https://www.genecards.org/cgi-bin/carddisp.pl?gene=ACSL4
GC04P087799	7.66	https://www.genecards.org/cgi-bin/carddisp.pl?gene=IBSP
GC02P097628	7.66	https://www.genecards.org/cgi-bin/carddisp.pl?gene=COX5B
GC0XP046836	7.66	https://www.genecards.org/cgi-bin/carddisp.pl?gene=RP2
GC17P012020	7.66	https://www.genecards.org/cgi-bin/carddisp.pl?gene=MAP2K4
GC11M059679	7.66	https://www.genecards.org/cgi-bin/carddisp.pl?gene=CKAP5
GC0XM151958	7.65	https://www.genecards.org/cgi-bin/carddisp.pl?gene=MIR224
GC12M023529	7.65	https://www.genecards.org/cgi-bin/carddisp.pl?gene=SOX5
GC03P035751	7.64	https://www.genecards.org/cgi-bin/carddisp.pl?gene=MIR128-2
GC11M083455	7.64	https://www.genecards.org/cgi-bin/carddisp.pl?gene=DLG2
GC19M039834	7.64	https://www.genecards.org/cgi-bin/carddisp.pl?gene=FBL
GC19P048619	7.63	https://www.genecards.org/cgi-bin/carddisp.pl?gene=SPHK2
GC17M063894	7.63	https://www.genecards.org/cgi-bin/carddisp.pl?gene=CSH1

GC19M004538	7.63	https://www.genecards.org/cgi-bin/carddisp.pl?gene=LRG1
GC15M040735	7.63	https://www.genecards.org/cgi-bin/carddisp.pl?gene=RMDN3
GC06P133897	7.63	https://www.genecards.org/cgi-bin/carddisp.pl?gene=TBPL1
GC04M122888	7.62	https://www.genecards.org/cgi-bin/carddisp.pl?gene=NUDT6
GC07M073700	7.62	https://www.genecards.org/cgi-bin/carddisp.pl?gene=STX1A
GC01M098046	7.62	https://www.genecards.org/cgi-bin/carddisp.pl?gene=MIR137
GC07M033094	7.62	https://www.genecards.org/cgi-bin/carddisp.pl?gene=RP9
GC02P197705	7.62	https://www.genecards.org/cgi-bin/carddisp.pl?gene=MARS2
GC0XM134215	7.62	https://www.genecards.org/cgi-bin/carddisp.pl?gene=MIR92A2
GC0XM120524	7.62	https://www.genecards.org/cgi-bin/carddisp.pl?gene=CUL4B
GC18P054274	7.61	https://www.genecards.org/cgi-bin/carddisp.pl?gene=POLI
GC02M219219	7.61	https://www.genecards.org/cgi-bin/carddisp.pl?gene=ATG9A
GC10M016672	7.6	https://www.genecards.org/cgi-bin/carddisp.pl?gene=RSU1
GC08P028089	7.6	https://www.genecards.org/cgi-bin/carddisp.pl?gene=ELP3
GC04P000486	7.6	https://www.genecards.org/cgi-bin/carddisp.pl?gene=PIGG
GC16P031411	7.6	https://www.genecards.org/cgi-bin/carddisp.pl?gene=KAT8
GC17M007240	7.6	https://www.genecards.org/cgi-bin/carddisp.pl?gene=GABARAP
GC03M112921	7.6	https://www.genecards.org/cgi-bin/carddisp.pl?gene=CD200R1
GC07M149887	7.6	https://www.genecards.org/cgi-bin/carddisp.pl?gene=ACTR3C
GC01P007785	7.59	https://www.genecards.org/cgi-bin/carddisp.pl?gene=PER3
GC07M081946	7.58	https://www.genecards.org/cgi-bin/carddisp.pl?gene=CACNA2D1
GC01M155897	7.58	https://www.genecards.org/cgi-bin/carddisp.pl?gene=RIT1
GC22P024011	7.58	https://www.genecards.org/cgi-bin/carddisp.pl?gene=CABIN1
GC22P041833	7.57	https://www.genecards.org/cgi-bin/carddisp.pl?gene=SREBF2
GC11P130069	7.56	https://www.genecards.org/cgi-bin/carddisp.pl?gene=APLP2
GC17M007225	7.56	https://www.genecards.org/cgi-bin/carddisp.pl?gene=DVL2
GC02M231921	7.56	https://www.genecards.org/cgi-bin/carddisp.pl?gene=NPPC
GC08M023190	7.56	https://www.genecards.org/cgi-bin/carddisp.pl?gene=TNFRSF10A
GC02P178416	7.56	https://www.genecards.org/cgi-bin/carddisp.pl?gene=CHROMR
GC22P038681	7.56	https://www.genecards.org/cgi-bin/carddisp.pl?gene=TOMM22
GC10P119207	7.55	https://www.genecards.org/cgi-bin/carddisp.pl?gene=GRK5
GC01P001514	7.55	https://www.genecards.org/cgi-bin/carddisp.pl?gene=ATAD3A
GC07P150824	7.55	https://www.genecards.org/cgi-bin/carddisp.pl?gene=AOC1
GC19M010391	7.54	https://www.genecards.org/cgi-bin/carddisp.pl?gene=CDC37
GC09P136881	7.53	https://www.genecards.org/cgi-bin/carddisp.pl?gene=TRAF2
GC16P015949	7.53	https://www.genecards.org/cgi-bin/carddisp.pl?gene=ABCC1
GC15M075369	7.52	https://www.genecards.org/cgi-bin/carddisp.pl?gene=SIN3A
GC03M130678	7.52	https://www.genecards.org/cgi-bin/carddisp.pl?gene=PIK3R4
GC06M089326	7.52	https://www.genecards.org/cgi-bin/carddisp.pl?gene=UBE2J1
GC16P082626	7.51	https://www.genecards.org/cgi-bin/carddisp.pl?gene=CDH13
GC01M205681	7.51	https://www.genecards.org/cgi-bin/carddisp.pl?gene=NUCKS1
GC20M037251	7.51	https://www.genecards.org/cgi-bin/carddisp.pl?gene=GHRH
GC01P158354	7.5	https://www.genecards.org/cgi-bin/carddisp.pl?gene=CD1E
GC02P003658	7.5	https://www.genecards.org/cgi-bin/carddisp.pl?gene=ALLC
GC16M001771	7.5	https://www.genecards.org/cgi-bin/carddisp.pl?gene=MRPS34
GC10M069571	7.5	https://www.genecards.org/cgi-bin/carddisp.pl?gene=NEUROG3
GC05M176346	7.49	https://www.genecards.org/cgi-bin/carddisp.pl?gene=KIAA1191

GC01M229322	7.49	https://www.genecards.org/cgi-bin/carddisp.pl?gene=CCSAP
GC07P005190	7.49	https://www.genecards.org/cgi-bin/carddisp.pl?gene=WIPI2
GC12M057009	7.49	https://www.genecards.org/cgi-bin/carddisp.pl?gene=TAC3
GC11P124865	7.49	https://www.genecards.org/cgi-bin/carddisp.pl?gene=ROBO3
GC19P040991	7.48	https://www.genecards.org/cgi-bin/carddisp.pl?gene=CYP2B6
GC01P032200	7.47	https://www.genecards.org/cgi-bin/carddisp.pl?gene=CCDC28B
GC02P231198	7.47	https://www.genecards.org/cgi-bin/carddisp.pl?gene=ARMC9
GC16M001790	7.46	https://www.genecards.org/cgi-bin/carddisp.pl?gene=IGFALS
GC12P112906	7.46	https://www.genecards.org/cgi-bin/carddisp.pl?gene=OAS1
GC06M083161	7.46	https://www.genecards.org/cgi-bin/carddisp.pl?gene=PGM3
GC03P049007	7.46	https://www.genecards.org/cgi-bin/carddisp.pl?gene=WDR6
GC06P149218	7.46	https://www.genecards.org/cgi-bin/carddisp.pl?gene=TAB2
GC09M083992	7.45	https://www.genecards.org/cgi-bin/carddisp.pl?gene=HNRNPK
GC17P006995	7.45	https://www.genecards.org/cgi-bin/carddisp.pl?gene=ALOX12
GC16P056731	7.44	https://www.genecards.org/cgi-bin/carddisp.pl?gene=NUP93
GC10M072367	7.44	https://www.genecards.org/cgi-bin/carddisp.pl?gene=MICU1
GC09M092613	7.44	https://www.genecards.org/cgi-bin/carddisp.pl?gene=IPPK
GC0XM014002	7.44	https://www.genecards.org/cgi-bin/carddisp.pl?gene=GEMIN8
GC02M024067	7.44	https://www.genecards.org/cgi-bin/carddisp.pl?gene=SF3B6
GC01M047250	7.44	https://www.genecards.org/cgi-bin/carddisp.pl?gene=STIL
GC06P033390	7.43	https://www.genecards.org/cgi-bin/carddisp.pl?gene=MICB
GC14M055366	7.43	https://www.genecards.org/cgi-bin/carddisp.pl?gene=ATG14
GC11M109864	7.42	https://www.genecards.org/cgi-bin/carddisp.pl?gene=RDX
GC09M109375	7.42	https://www.genecards.org/cgi-bin/carddisp.pl?gene=PTPN3
GC14P104321	7.42	https://www.genecards.org/cgi-bin/carddisp.pl?gene=KLC1
GC14P022766	7.42	https://www.genecards.org/cgi-bin/carddisp.pl?gene=OXA1L
GC19P038244	7.42	https://www.genecards.org/cgi-bin/carddisp.pl?gene=SPINT2
GC15M082536	7.41	https://www.genecards.org/cgi-bin/carddisp.pl?gene=RPS17
GC0XM136848	7.41	https://www.genecards.org/cgi-bin/carddisp.pl?gene=RBMX
GC02M070830	7.41	https://www.genecards.org/cgi-bin/carddisp.pl?gene=CD207
GC01P039082	7.41	https://www.genecards.org/cgi-bin/carddisp.pl?gene=MACF1
GC19M031150	7.39	https://www.genecards.org/cgi-bin/carddisp.pl?gene=TSHZ3
GC07M021900	7.39	https://www.genecards.org/cgi-bin/carddisp.pl?gene=CDCA7L
GC17P051154	7.39	https://www.genecards.org/cgi-bin/carddisp.pl?gene=NME1
GC01P084078	7.39	https://www.genecards.org/cgi-bin/carddisp.pl?gene=PRKACB
GC19P000532	7.39	https://www.genecards.org/cgi-bin/carddisp.pl?gene=CDC34
GC01P220528	7.37	https://www.genecards.org/cgi-bin/carddisp.pl?gene=MARK1
GC07P037889	7.37	https://www.genecards.org/cgi-bin/carddisp.pl?gene=NME8
GC19P001438	7.37	https://www.genecards.org/cgi-bin/carddisp.pl?gene=RPS15
GC0XP091779	7.36	https://www.genecards.org/cgi-bin/carddisp.pl?gene=PCDH11X
GC03P147393	7.36	https://www.genecards.org/cgi-bin/carddisp.pl?gene=ZIC1
GC19P001242	7.36	https://www.genecards.org/cgi-bin/carddisp.pl?gene=ATP5F1D
GC17M058352	7.36	https://www.genecards.org/cgi-bin/carddisp.pl?gene=RNF43
GC10P005556	7.35	https://www.genecards.org/cgi-bin/carddisp.pl?gene=CALML3
GC06M075237	7.34	https://www.genecards.org/cgi-bin/carddisp.pl?gene=COX7A2
GC07M041668	7.34	https://www.genecards.org/cgi-bin/carddisp.pl?gene=INHBA
GC09M101420	7.34	https://www.genecards.org/cgi-bin/carddisp.pl?gene=ALDOB

GC04P108620	7.33	https://www.genecards.org/cgi-bin/carddisp.pl?gene=RPL34
GC08P043093	7.33	https://www.genecards.org/cgi-bin/carddisp.pl?gene=POMK
GC19P054141	7.33	https://www.genecards.org/cgi-bin/carddisp.pl?gene=CNOT3
GC03P049673	7.33	https://www.genecards.org/cgi-bin/carddisp.pl?gene=APEH
GC12P098593	7.33	https://www.genecards.org/cgi-bin/carddisp.pl?gene=SLC25A3
GC09M094603	7.33	https://www.genecards.org/cgi-bin/carddisp.pl?gene=FBP1
GC02M187464	7.32	https://www.genecards.org/cgi-bin/carddisp.pl?gene=TFPI
GC06U902316	7.32	https://www.genecards.org/cgi-bin/carddisp.pl?gene=ETM3
GC14M034706	7.32	https://www.genecards.org/cgi-bin/carddisp.pl?gene=CFL2
GC10P104642	7.32	https://www.genecards.org/cgi-bin/carddisp.pl?gene=SORCS3
GC20P057561	7.31	https://www.genecards.org/cgi-bin/carddisp.pl?gene=PCK1
GC19P049591	7.31	https://www.genecards.org/cgi-bin/carddisp.pl?gene=PRR12
GC01P060865	7.31	https://www.genecards.org/cgi-bin/carddisp.pl?gene=NFIA
GC13P023160	7.31	https://www.genecards.org/cgi-bin/carddisp.pl?gene=SGCG
GC01M062454	7.31	https://www.genecards.org/cgi-bin/carddisp.pl?gene=DOCK7
GC08P009555	7.3	https://www.genecards.org/cgi-bin/carddisp.pl?gene=TNKS
GC01M037612	7.3	https://www.genecards.org/cgi-bin/carddisp.pl?gene=RSPO1
GC08P006406	7.3	https://www.genecards.org/cgi-bin/carddisp.pl?gene=MCPH1
GC19M039243	7.3	https://www.genecards.org/cgi-bin/carddisp.pl?gene=IFNL3
GC06P055106	7.3	https://www.genecards.org/cgi-bin/carddisp.pl?gene=HCRTR2
GC01P059814	7.29	https://www.genecards.org/cgi-bin/carddisp.pl?gene=HOOK1
GC12P052022	7.29	https://www.genecards.org/cgi-bin/carddisp.pl?gene=NR4A1
GC10P093496	7.29	https://www.genecards.org/cgi-bin/carddisp.pl?gene=CEP55
GC09P078297	7.28	https://www.genecards.org/cgi-bin/carddisp.pl?gene=PSAT1
GC02P189784	7.28	https://www.genecards.org/cgi-bin/carddisp.pl?gene=PMS1
GC19P039480	7.27	https://www.genecards.org/cgi-bin/carddisp.pl?gene=TIMM50
GC10M063167	7.27	https://www.genecards.org/cgi-bin/carddisp.pl?gene=JMJD1C
GC22M042617	7.27	https://www.genecards.org/cgi-bin/carddisp.pl?gene=CYB5R3
GC08P101492	7.27	https://www.genecards.org/cgi-bin/carddisp.pl?gene=GRHL2
GC01M158297	7.26	https://www.genecards.org/cgi-bin/carddisp.pl?gene=CD1B
GC06M159778	7.26	https://www.genecards.org/cgi-bin/carddisp.pl?gene=TCP1
GC01M210678	7.25	https://www.genecards.org/cgi-bin/carddisp.pl?gene=KCNH1
GC14P071398	7.25	https://www.genecards.org/cgi-bin/carddisp.pl?gene=SNORD56B
GC07P026291	7.25	https://www.genecards.org/cgi-bin/carddisp.pl?gene=SNX10
GC07M080742	7.25	https://www.genecards.org/cgi-bin/carddisp.pl?gene=SEMA3C
GC16M057884	7.24	https://www.genecards.org/cgi-bin/carddisp.pl?gene=CNGB1
GC10M020779	7.24	https://www.genecards.org/cgi-bin/carddisp.pl?gene=NEBL
GC17M004630	7.23	https://www.genecards.org/cgi-bin/carddisp.pl?gene=ALOX15
GC17M018244	7.22	https://www.genecards.org/cgi-bin/carddisp.pl?gene=FLII
GC11M014299	7.22	https://www.genecards.org/cgi-bin/carddisp.pl?gene=RRAS2
GC14M091271	7.22	https://www.genecards.org/cgi-bin/carddisp.pl?gene=CCDC88C
GC11P092969	7.22	https://www.genecards.org/cgi-bin/carddisp.pl?gene=MTNR1B
GC08P018180	7.22	https://www.genecards.org/cgi-bin/carddisp.pl?gene=NAT1
GC01P192809	7.21	https://www.genecards.org/cgi-bin/carddisp.pl?gene=RGS2
GC09P137241	7.21	https://www.genecards.org/cgi-bin/carddisp.pl?gene=TUBB4B
GC0XM065512	7.21	https://www.genecards.org/cgi-bin/carddisp.pl?gene=LAS1L
GC01M023795	7.21	https://www.genecards.org/cgi-bin/carddisp.pl?gene=GALE

GC03M183122	7.2	https://www.genecards.org/cgi-bin/carddisp.pl?gene=LAMP3
GC21M038367	7.2	https://www.genecards.org/cgi-bin/carddisp.pl?gene=ERG
GC02M069395	7.2	https://www.genecards.org/cgi-bin/carddisp.pl?gene=NFU1
GC06M010762	7.2	https://www.genecards.org/cgi-bin/carddisp.pl?gene=MAK
GC19P039436	7.19	https://www.genecards.org/cgi-bin/carddisp.pl?gene=SUPT5H
GC02P028752	7.18	https://www.genecards.org/cgi-bin/carddisp.pl?gene=PPP1CB
GC06P046031	7.17	https://www.genecards.org/cgi-bin/carddisp.pl?gene=PPARD
GC19M045212	7.17	https://www.genecards.org/cgi-bin/carddisp.pl?gene=EXOC3L2
GC10P062374	7.17	https://www.genecards.org/cgi-bin/carddisp.pl?gene=ZNF365
GC01P009944	7.16	https://www.genecards.org/cgi-bin/carddisp.pl?gene=NMNAT1
GC11M059696	7.16	https://www.genecards.org/cgi-bin/carddisp.pl?gene=PSMC3
GC14M105140	7.16	https://www.genecards.org/cgi-bin/carddisp.pl?gene=JAG2
GC16P088638	7.16	https://www.genecards.org/cgi-bin/carddisp.pl?gene=IL17C
GC06P118548	7.16	https://www.genecards.org/cgi-bin/carddisp.pl?gene=PLN
GC08P119873	7.15	https://www.genecards.org/cgi-bin/carddisp.pl?gene=DEPTOR
GC07P044044	7.15	https://www.genecards.org/cgi-bin/carddisp.pl?gene=DBNL
GC18M059430	7.15	https://www.genecards.org/cgi-bin/carddisp.pl?gene=CCBE1
GC17M042572	7.15	https://www.genecards.org/cgi-bin/carddisp.pl?gene=PSMC3IP
GC19M012796	7.14	https://www.genecards.org/cgi-bin/carddisp.pl?gene=PRDX2
GC10M133262	7.14	https://www.genecards.org/cgi-bin/carddisp.pl?gene=ADAM8
GC03P131026	7.14	https://www.genecards.org/cgi-bin/carddisp.pl?gene=NEK11
GC05M157395	7.14	https://www.genecards.org/cgi-bin/carddisp.pl?gene=ADAM19
GC06P113857	7.13	https://www.genecards.org/cgi-bin/carddisp.pl?gene=MARCKS
GC14P039114	7.13	https://www.genecards.org/cgi-bin/carddisp.pl?gene=GEMIN2
GC07P144876	7.12	https://www.genecards.org/cgi-bin/carddisp.pl?gene=SSBP1
GC02M231453	7.11	https://www.genecards.org/cgi-bin/carddisp.pl?gene=NCL
GC14P076310	7.11	https://www.genecards.org/cgi-bin/carddisp.pl?gene=ESRRB
GC13P027251	7.11	https://www.genecards.org/cgi-bin/carddisp.pl?gene=RPL21
GC08M107899	7.11	https://www.genecards.org/cgi-bin/carddisp.pl?gene=RSPO2
GC03M097986	7.11	https://www.genecards.org/cgi-bin/carddisp.pl?gene=GABRR3
GC17M010672	7.11	https://www.genecards.org/cgi-bin/carddisp.pl?gene=SCO1
GC20P018507	7.09	https://www.genecards.org/cgi-bin/carddisp.pl?gene=SEC23B
GC14P030622	7.09	https://www.genecards.org/cgi-bin/carddisp.pl?gene=SCFD1
GC06M033318	7.09	https://www.genecards.org/cgi-bin/carddisp.pl?gene=DAXX
GC03P052198	7.08	https://www.genecards.org/cgi-bin/carddisp.pl?gene=ALAS1
GC09P121566	7.08	https://www.genecards.org/cgi-bin/carddisp.pl?gene=DAB2IP
GC01M232397	7.08	https://www.genecards.org/cgi-bin/carddisp.pl?gene=SIPA1L2
GC15P074890	7.07	https://www.genecards.org/cgi-bin/carddisp.pl?gene=MPI
GC03P119579	7.07	https://www.genecards.org/cgi-bin/carddisp.pl?gene=ADPRH
GC0YM002698	7.07	https://www.genecards.org/cgi-bin/carddisp.pl?gene=SRY
GC09M091213	7.06	https://www.genecards.org/cgi-bin/carddisp.pl?gene=AUH
GC10P045972	7.06	https://www.genecards.org/cgi-bin/carddisp.pl?gene=TIMM23
GC04P015779	7.06	https://www.genecards.org/cgi-bin/carddisp.pl?gene=CD38
GC02M175072	7.06	https://www.genecards.org/cgi-bin/carddisp.pl?gene=ATF2
GC04M156760	7.06	https://www.genecards.org/cgi-bin/carddisp.pl?gene=PDGFC
GC06P028049	7.05	https://www.genecards.org/cgi-bin/carddisp.pl?gene=ABT1
GC11P117300	7.05	https://www.genecards.org/cgi-bin/carddisp.pl?gene=BACE1-AS

GC15P040405	7.05	https://www.genecards.org/cgi-bin/carddisp.pl?gene=IVD
GC17M082036	7.05	https://www.genecards.org/cgi-bin/carddisp.pl?gene=DCXR
GC02P047331	7.05	https://www.genecards.org/cgi-bin/carddisp.pl?gene=BCYRN1
GC14P067241	7.05	https://www.genecards.org/cgi-bin/carddisp.pl?gene=MPP5
GC19M011172	7.05	https://www.genecards.org/cgi-bin/carddisp.pl?gene=DOCK6
GC20M037101	7.04	https://www.genecards.org/cgi-bin/carddisp.pl?gene=MROH8
GC22M026443	7.04	https://www.genecards.org/cgi-bin/carddisp.pl?gene=HPS4
GC22P041622	7.04	https://www.genecards.org/cgi-bin/carddisp.pl?gene=XRCC6
GC02P028460	7.03	https://www.genecards.org/cgi-bin/carddisp.pl?gene=PLB1
GC19P000544	7.03	https://www.genecards.org/cgi-bin/carddisp.pl?gene=GZMM
GC11P061811	7.03	https://www.genecards.org/cgi-bin/carddisp.pl?gene=FEN1
GC08M073290	7.03	https://www.genecards.org/cgi-bin/carddisp.pl?gene=RPL7
GC01P042456	7.03	https://www.genecards.org/cgi-bin/carddisp.pl?gene=PPCS
GC17M016029	7.03	https://www.genecards.org/cgi-bin/carddisp.pl?gene=NCOR1
GC01P109616	7.03	https://www.genecards.org/cgi-bin/carddisp.pl?gene=AMPD2
GC04P069932	7.02	https://www.genecards.org/cgi-bin/carddisp.pl?gene=CSN1S1
GC06M053002	7.02	https://www.genecards.org/cgi-bin/carddisp.pl?gene=CILK1
GC06P044246	7.02	https://www.genecards.org/cgi-bin/carddisp.pl?gene=HSP90AB1
GC16M003581	7.02	https://www.genecards.org/cgi-bin/carddisp.pl?gene=SLX4
GC17M038733	7.02	https://www.genecards.org/cgi-bin/carddisp.pl?gene=PCGF2
GC15P071842	7.01	https://www.genecards.org/cgi-bin/carddisp.pl?gene=RPLP1
GC01M042746	7	https://www.genecards.org/cgi-bin/carddisp.pl?gene=P3H1
GC04M106041	7	https://www.genecards.org/cgi-bin/carddisp.pl?gene=TBCK
GC03P002117	7	https://www.genecards.org/cgi-bin/carddisp.pl?gene=CNTN4
GC06P033402	7	https://www.genecards.org/cgi-bin/carddisp.pl?gene=CSNK2B
GC07M129774	6.99	https://www.genecards.org/cgi-bin/carddisp.pl?gene=MIR96
GC12M021764	6.99	https://www.genecards.org/cgi-bin/carddisp.pl?gene=KCNJ8
GC07P103344	6.99	https://www.genecards.org/cgi-bin/carddisp.pl?gene=PSMC2
GC01M216503	6.98	https://www.genecards.org/cgi-bin/carddisp.pl?gene=ESRRG
GC05M134194	6.97	https://www.genecards.org/cgi-bin/carddisp.pl?gene=PPP2CA
GC12M051093	6.97	https://www.genecards.org/cgi-bin/carddisp.pl?gene=TFCP2
GC19P045079	6.97	https://www.genecards.org/cgi-bin/carddisp.pl?gene=MARK4
GC12M006570	6.97	https://www.genecards.org/cgi-bin/carddisp.pl?gene=CHD4
GC17M007022	6.96	https://www.genecards.org/cgi-bin/carddisp.pl?gene=MIR497
GC0XM134245	6.95	https://www.genecards.org/cgi-bin/carddisp.pl?gene=MIR20B
GC07P099374	6.94	https://www.genecards.org/cgi-bin/carddisp.pl?gene=ARPC1B
GC02P099158	6.94	https://www.genecards.org/cgi-bin/carddisp.pl?gene=LIPT1
GC06P075749	6.94	https://www.genecards.org/cgi-bin/carddisp.pl?gene=MYO6
GC01P153967	6.94	https://www.genecards.org/cgi-bin/carddisp.pl?gene=CREB3L4
GC17P082374	6.93	https://www.genecards.org/cgi-bin/carddisp.pl?gene=UTS2R
GC05M077425	6.93	https://www.genecards.org/cgi-bin/carddisp.pl?gene=WDR41
GC12P131894	6.93	https://www.genecards.org/cgi-bin/carddisp.pl?gene=ULK1
GC01P011806	6.93	https://www.genecards.org/cgi-bin/carddisp.pl?gene=CLCN6
GC03P033113	6.93	https://www.genecards.org/cgi-bin/carddisp.pl?gene=CRTAP
GC15P028888	6.93	https://www.genecards.org/cgi-bin/carddisp.pl?gene=APBA2
GC02M171783	6.92	https://www.genecards.org/cgi-bin/carddisp.pl?gene=SLC25A12
GC12M120196	6.92	https://www.genecards.org/cgi-bin/carddisp.pl?gene=RPLP0

GC17M047117	6.92	https://www.genecards.org/cgi-bin/carddisp.pl?gene=CDC27
GC01P201540	6.92	https://www.genecards.org/cgi-bin/carddisp.pl?gene=NAV1
GC02M134455	6.92	https://www.genecards.org/cgi-bin/carddisp.pl?gene=TMEM163
GC18P034976	6.91	https://www.genecards.org/cgi-bin/carddisp.pl?gene=MAPRE2
GC07M015617	6.91	https://www.genecards.org/cgi-bin/carddisp.pl?gene=MEOX2
GC09P017569	6.91	https://www.genecards.org/cgi-bin/carddisp.pl?gene=SH3GL2
GC05M132861	6.91	https://www.genecards.org/cgi-bin/carddisp.pl?gene=GDF9
GC19M017816	6.91	https://www.genecards.org/cgi-bin/carddisp.pl?gene=INSL3
GC16M002160	6.91	https://www.genecards.org/cgi-bin/carddisp.pl?gene=RPS2
GC15M034751	6.9	https://www.genecards.org/cgi-bin/carddisp.pl?gene=GJD2
GC19M007958	6.9	https://www.genecards.org/cgi-bin/carddisp.pl?gene=ELAVL1
GC10M086436	6.9	https://www.genecards.org/cgi-bin/carddisp.pl?gene=WAPL
GC02M224470	6.89	https://www.genecards.org/cgi-bin/carddisp.pl?gene=CUL3
GC07P101815	6.89	https://www.genecards.org/cgi-bin/carddisp.pl?gene=CUX1
GC19P037732	6.89	https://www.genecards.org/cgi-bin/carddisp.pl?gene=APLP1
GC16M028606	6.89	https://www.genecards.org/cgi-bin/carddisp.pl?gene=SULT1A1
GC10M046461	6.88	https://www.genecards.org/cgi-bin/carddisp.pl?gene=NPY4R
GC22M044563	6.88	https://www.genecards.org/cgi-bin/carddisp.pl?gene=SLC25A17
GC20M004731	6.88	https://www.genecards.org/cgi-bin/carddisp.pl?gene=PRNT
GC04P056466	6.88	https://www.genecards.org/cgi-bin/carddisp.pl?gene=SRP72
GC19P051752	6.88	https://www.genecards.org/cgi-bin/carddisp.pl?gene=FPR2
GC11M065120	6.88	https://www.genecards.org/cgi-bin/carddisp.pl?gene=FAU
GC04P075555	6.88	https://www.genecards.org/cgi-bin/carddisp.pl?gene=ODAPH
GC02P026847	6.88	https://www.genecards.org/cgi-bin/carddisp.pl?gene=DPYSL5
GC07P151341	6.87	https://www.genecards.org/cgi-bin/carddisp.pl?gene=NUB1
GC02M159318	6.87	https://www.genecards.org/cgi-bin/carddisp.pl?gene=BAZ2B
GC19P029923	6.87	https://www.genecards.org/cgi-bin/carddisp.pl?gene=URI1
GC12M120341	6.87	https://www.genecards.org/cgi-bin/carddisp.pl?gene=MSI1
GC14P074493	6.87	https://www.genecards.org/cgi-bin/carddisp.pl?gene=ISCA2
GC16M018781	6.86	https://www.genecards.org/cgi-bin/carddisp.pl?gene=RPS15A
GC16P029806	6.86	https://www.genecards.org/cgi-bin/carddisp.pl?gene=MAZ
GC15P071873	6.85	https://www.genecards.org/cgi-bin/carddisp.pl?gene=APH1B
GC19P055318	6.85	https://www.genecards.org/cgi-bin/carddisp.pl?gene=KIR2DL3
GC22P040951	6.85	https://www.genecards.org/cgi-bin/carddisp.pl?gene=RBX1
GC07P045580	6.84	https://www.genecards.org/cgi-bin/carddisp.pl?gene=ADCY1
GC14M021521	6.84	https://www.genecards.org/cgi-bin/carddisp.pl?gene=SALL2
GC12P095217	6.84	https://www.genecards.org/cgi-bin/carddisp.pl?gene=VEZT
GC04M121879	6.83	https://www.genecards.org/cgi-bin/carddisp.pl?gene=TRPC3
GC0XM015825	6.83	https://www.genecards.org/cgi-bin/carddisp.pl?gene=AP1S2
GC05M150443	6.83	https://www.genecards.org/cgi-bin/carddisp.pl?gene=RPS14
GC01P010398	6.83	https://www.genecards.org/cgi-bin/carddisp.pl?gene=PGD
GC06M110099	6.82	https://www.genecards.org/cgi-bin/carddisp.pl?gene=WASF1
GC01M165665	6.82	https://www.genecards.org/cgi-bin/carddisp.pl?gene=ALDH9A1
GC08M061500	6.82	https://www.genecards.org/cgi-bin/carddisp.pl?gene=ASPH
GC16P028883	6.82	https://www.genecards.org/cgi-bin/carddisp.pl?gene=ATXN2L
GC17M030248	6.81	https://www.genecards.org/cgi-bin/carddisp.pl?gene=BLMH
GC17M081932	6.81	https://www.genecards.org/cgi-bin/carddisp.pl?gene=PYCR1

GC02M085318	6.81	https://www.genecards.org/cgi-bin/carddisp.pl?gene=TGOLN2
GC12P005043	6.8	https://www.genecards.org/cgi-bin/carddisp.pl?gene=KCNA5
GC01P159587	6.79	https://www.genecards.org/cgi-bin/carddisp.pl?gene=APCS
GC10M133420	6.79	https://www.genecards.org/cgi-bin/carddisp.pl?gene=SPRN
GC04P020668	6.79	https://www.genecards.org/cgi-bin/carddisp.pl?gene=MIR218-1
GC07P144731	6.79	https://www.genecards.org/cgi-bin/carddisp.pl?gene=PRSS2
GC06M011183	6.78	https://www.genecards.org/cgi-bin/carddisp.pl?gene=NEDD9
GC11P034051	6.78	https://www.genecards.org/cgi-bin/carddisp.pl?gene=CAPRIN1
GC17M004270	6.78	https://www.genecards.org/cgi-bin/carddisp.pl?gene=UBE2G1
GC0XM115003	6.77	https://www.genecards.org/cgi-bin/carddisp.pl?gene=IL13RA2
GC12M009148	6.77	https://www.genecards.org/cgi-bin/carddisp.pl?gene=PZP
GC05P071719	6.76	https://www.genecards.org/cgi-bin/carddisp.pl?gene=CARTPT
GC18P012307	6.76	https://www.genecards.org/cgi-bin/carddisp.pl?gene=TUBB6
GC01P027393	6.75	https://www.genecards.org/cgi-bin/carddisp.pl?gene=GPR3
GC11P105609	6.75	https://www.genecards.org/cgi-bin/carddisp.pl?gene=GRIA4
GC03P045730	6.75	https://www.genecards.org/cgi-bin/carddisp.pl?gene=SACM1L
GC10P028557	6.75	https://www.genecards.org/cgi-bin/carddisp.pl?gene=WAC
GC01P070411	6.74	https://www.genecards.org/cgi-bin/carddisp.pl?gene=CTH
GC01P155610	6.74	https://www.genecards.org/cgi-bin/carddisp.pl?gene=MSTO1
GC03M052296	6.74	https://www.genecards.org/cgi-bin/carddisp.pl?gene=MIR135A1
GC01M169921	6.74	https://www.genecards.org/cgi-bin/carddisp.pl?gene=KIFAP3
GC01P153627	6.73	https://www.genecards.org/cgi-bin/carddisp.pl?gene=S100A1
GC11P061222	6.73	https://www.genecards.org/cgi-bin/carddisp.pl?gene=PGA4
GC11M005232	6.73	https://www.genecards.org/cgi-bin/carddisp.pl?gene=HBD
GC05P134873	6.72	https://www.genecards.org/cgi-bin/carddisp.pl?gene=TXNDC15
GC02P095326	6.72	https://www.genecards.org/cgi-bin/carddisp.pl?gene=KCNIP3
GC06P033207	6.72	https://www.genecards.org/cgi-bin/carddisp.pl?gene=MIR219A1
GC16P008674	6.72	https://www.genecards.org/cgi-bin/carddisp.pl?gene=ABAT
GC02P219434	6.71	https://www.genecards.org/cgi-bin/carddisp.pl?gene=SPEG
GC04M064275	6.71	https://www.genecards.org/cgi-bin/carddisp.pl?gene=TECRL
GC19P049496	6.7	https://www.genecards.org/cgi-bin/carddisp.pl?gene=RPS11
GC05P179678	6.69	https://www.genecards.org/cgi-bin/carddisp.pl?gene=CANX
GC04P056435	6.69	https://www.genecards.org/cgi-bin/carddisp.pl?gene=PAICS
GC12M068201	6.69	https://www.genecards.org/cgi-bin/carddisp.pl?gene=IL26
GC11P017530	6.69	https://www.genecards.org/cgi-bin/carddisp.pl?gene=OTOG
GC19P034359	6.69	https://www.genecards.org/cgi-bin/carddisp.pl?gene=GPI
GC11P113387	6.69	https://www.genecards.org/cgi-bin/carddisp.pl?gene=ANKK1
GC17P004583	6.69	https://www.genecards.org/cgi-bin/carddisp.pl?gene=SMTNL2
GC17P004796	6.68	https://www.genecards.org/cgi-bin/carddisp.pl?gene=PSMB6
GC12M120322	6.68	https://www.genecards.org/cgi-bin/carddisp.pl?gene=PLA2G1B
GC03M185190	6.68	https://www.genecards.org/cgi-bin/carddisp.pl?gene=EHHADH
GC04P074445	6.67	https://www.genecards.org/cgi-bin/carddisp.pl?gene=AREG
GC0XP054807	6.67	https://www.genecards.org/cgi-bin/carddisp.pl?gene=MAGED2
GC19M004360	6.67	https://www.genecards.org/cgi-bin/carddisp.pl?gene=SH3GL1
GC21P037073	6.66	https://www.genecards.org/cgi-bin/carddisp.pl?gene=TTC3
GC02P169584	6.66	https://www.genecards.org/cgi-bin/carddisp.pl?gene=PPIG
GC12M066347	6.66	https://www.genecards.org/cgi-bin/carddisp.pl?gene=GRIP1

GC06M111299	6.66	https://www.genecards.org/cgi-bin/carddisp.pl?gene=REV3L
GC07M023316	6.65	https://www.genecards.org/cgi-bin/carddisp.pl?gene=IGF2BP3
GC05P034656	6.65	https://www.genecards.org/cgi-bin/carddisp.pl?gene=RAI14
GC01M202300	6.64	https://www.genecards.org/cgi-bin/carddisp.pl?gene=UBE2T
GC0XP048521	6.64	https://www.genecards.org/cgi-bin/carddisp.pl?gene=EBP
GC03P051983	6.63	https://www.genecards.org/cgi-bin/carddisp.pl?gene=ACY1
GC04P093828	6.63	https://www.genecards.org/cgi-bin/carddisp.pl?gene=ATOH1
GC16M052471	6.63	https://www.genecards.org/cgi-bin/carddisp.pl?gene=TOX3
GC17M039921	6.62	https://www.genecards.org/cgi-bin/carddisp.pl?gene=ORMDL3
GC08M030578	6.62	https://www.genecards.org/cgi-bin/carddisp.pl?gene=GTF2E2
GC01M043281	6.61	https://www.genecards.org/cgi-bin/carddisp.pl?gene=C1orf210
GC06P044219	6.6	https://www.genecards.org/cgi-bin/carddisp.pl?gene=SLC29A1
GC11P073647	6.6	https://www.genecards.org/cgi-bin/carddisp.pl?gene=PLEKHB1
GC12P119632	6.6	https://www.genecards.org/cgi-bin/carddisp.pl?gene=PRKAB1
GC01M116514	6.6	https://www.genecards.org/cgi-bin/carddisp.pl?gene=CD58
GC16M086530	6.6	https://www.genecards.org/cgi-bin/carddisp.pl?gene=MTHFSD
GC03P197961	6.59	https://www.genecards.org/cgi-bin/carddisp.pl?gene=LMLN
GC14M050237	6.59	https://www.genecards.org/cgi-bin/carddisp.pl?gene=L2HGDH
GC01P043358	6.59	https://www.genecards.org/cgi-bin/carddisp.pl?gene=CDC20
GC02P032166	6.59	https://www.genecards.org/cgi-bin/carddisp.pl?gene=SLC30A6
GC09M092211	6.59	https://www.genecards.org/cgi-bin/carddisp.pl?gene=IARS1
GC02P233059	6.58	https://www.genecards.org/cgi-bin/carddisp.pl?gene=INPP5D
GC08P028316	6.58	https://www.genecards.org/cgi-bin/carddisp.pl?gene=PNOC
GC19P014504	6.58	https://www.genecards.org/cgi-bin/carddisp.pl?gene=TECR
GC22P035400	6.58	https://www.genecards.org/cgi-bin/carddisp.pl?gene=MCM5
GC07M073568	6.57	https://www.genecards.org/cgi-bin/carddisp.pl?gene=TBL2
GC15P099098	6.57	https://www.genecards.org/cgi-bin/carddisp.pl?gene=SYNM
GC03P179347	6.57	https://www.genecards.org/cgi-bin/carddisp.pl?gene=MFN1
GC21P036157	6.57	https://www.genecards.org/cgi-bin/carddisp.pl?gene=DOP1B
GC06M155247	6.56	https://www.genecards.org/cgi-bin/carddisp.pl?gene=TFB1M
GC04M040812	6.56	https://www.genecards.org/cgi-bin/carddisp.pl?gene=APBB2
GC02P037344	6.56	https://www.genecards.org/cgi-bin/carddisp.pl?gene=QPCT
GC19M010311	6.56	https://www.genecards.org/cgi-bin/carddisp.pl?gene=FDX2
GC11M059800	6.56	https://www.genecards.org/cgi-bin/carddisp.pl?gene=GLYAT
GC04M121816	6.55	https://www.genecards.org/cgi-bin/carddisp.pl?gene=CCNA2
GC02M055634	6.55	https://www.genecards.org/cgi-bin/carddisp.pl?gene=PNPT1
GC06M047231	6.54	https://www.genecards.org/cgi-bin/carddisp.pl?gene=TNFRSF21
GC03M172443	6.54	https://www.genecards.org/cgi-bin/carddisp.pl?gene=GHSR
GC21M037607	6.54	https://www.genecards.org/cgi-bin/carddisp.pl?gene=KCNJ6
GC07P107743	6.53	https://www.genecards.org/cgi-bin/carddisp.pl?gene=CBLL1
GC01P031587	6.53	https://www.genecards.org/cgi-bin/carddisp.pl?gene=HCRTR1
GC19P013117	6.52	https://www.genecards.org/cgi-bin/carddisp.pl?gene=NACC1
GC05M031401	6.51	https://www.genecards.org/cgi-bin/carddisp.pl?gene=DROSHA
GC14M049625	6.51	https://www.genecards.org/cgi-bin/carddisp.pl?gene=DNAAF2
GC22M050217	6.51	https://www.genecards.org/cgi-bin/carddisp.pl?gene=TUBGCP6
GC15P079209	6.51	https://www.genecards.org/cgi-bin/carddisp.pl?gene=MIR184
GC19M049869	6.49	https://www.genecards.org/cgi-bin/carddisp.pl?gene=AKT1S1

GC05P012578	6.49	https://www.genecards.org/cgi-bin/carddisp.pl?gene=LINC01194
GC04M099202	6.49	https://www.genecards.org/cgi-bin/carddisp.pl?gene=ADH6
GC06M032950	6.49	https://www.genecards.org/cgi-bin/carddisp.pl?gene=HLA-DMA
GC04M015733	6.49	https://www.genecards.org/cgi-bin/carddisp.pl?gene=RPL10AP7
GC15P045588	6.49	https://www.genecards.org/cgi-bin/carddisp.pl?gene=BLOC1S6
GC19P049085	6.48	https://www.genecards.org/cgi-bin/carddisp.pl?gene=SNRNP70
GC14P024094	6.48	https://www.genecards.org/cgi-bin/carddisp.pl?gene=PCK2
GC13P093226	6.48	https://www.genecards.org/cgi-bin/carddisp.pl?gene=GPC6
GC04P001341	6.47	https://www.genecards.org/cgi-bin/carddisp.pl?gene=UVSSA
GC04M005016	6.47	https://www.genecards.org/cgi-bin/carddisp.pl?gene=CYTL1
GC20P021705	6.47	https://www.genecards.org/cgi-bin/carddisp.pl?gene=PAX1
GC20M000958	6.47	https://www.genecards.org/cgi-bin/carddisp.pl?gene=RSPO4
GC02M216260	6.46	https://www.genecards.org/cgi-bin/carddisp.pl?gene=MARCHF4
GC0XM154401	6.46	https://www.genecards.org/cgi-bin/carddisp.pl?gene=DNASE1L1
GC10P019048	6.45	https://www.genecards.org/cgi-bin/carddisp.pl?gene=MALRD1
GC03M108043	6.45	https://www.genecards.org/cgi-bin/carddisp.pl?gene=CD47
GC14P052314	6.44	https://www.genecards.org/cgi-bin/carddisp.pl?gene=PTGER2
GC01P202007	6.44	https://www.genecards.org/cgi-bin/carddisp.pl?gene=ELF3
GC17M043940	6.44	https://www.genecards.org/cgi-bin/carddisp.pl?gene=PPY
GC01P026540	6.44	https://www.genecards.org/cgi-bin/carddisp.pl?gene=RPS6KA1
GC21P015730	6.44	https://www.genecards.org/cgi-bin/carddisp.pl?gene=USP25
GC09M137007	6.44	https://www.genecards.org/cgi-bin/carddisp.pl?gene=ABCA2
GC04M143870	6.43	https://www.genecards.org/cgi-bin/carddisp.pl?gene=GYPE
GC06P110814	6.43	https://www.genecards.org/cgi-bin/carddisp.pl?gene=AMD1
GC03M052913	6.43	https://www.genecards.org/cgi-bin/carddisp.pl?gene=SFMBT1
GC11P020647	6.42	https://www.genecards.org/cgi-bin/carddisp.pl?gene=NELL1
GC00U936676	6.42	https://www.genecards.org/cgi-bin/carddisp.pl?gene=AD9
GC03P039389	6.42	https://www.genecards.org/cgi-bin/carddisp.pl?gene=SLC25A38
GC19M049890	6.42	https://www.genecards.org/cgi-bin/carddisp.pl?gene=IL4I1
GC16M002772	6.42	https://www.genecards.org/cgi-bin/carddisp.pl?gene=ELOB
GC19M043506	6.41	https://www.genecards.org/cgi-bin/carddisp.pl?gene=ETHE1
GC20P000361	6.41	https://www.genecards.org/cgi-bin/carddisp.pl?gene=TRIB3
GC11P086303	6.41	https://www.genecards.org/cgi-bin/carddisp.pl?gene=HIKESHI
GC01P192544	6.41	https://www.genecards.org/cgi-bin/carddisp.pl?gene=RGS1
GC02M027308	6.41	https://www.genecards.org/cgi-bin/carddisp.pl?gene=UCN
GC14P104312	6.4	https://www.genecards.org/cgi-bin/carddisp.pl?gene=TRAF3
GC08M109573	6.4	https://www.genecards.org/cgi-bin/carddisp.pl?gene=SYBU
GC11P063977	6.4	https://www.genecards.org/cgi-bin/carddisp.pl?gene=COX8A
GC10M095038	6.4	https://www.genecards.org/cgi-bin/carddisp.pl?gene=CYP2C8
GC15P073051	6.4	https://www.genecards.org/cgi-bin/carddisp.pl?gene=NEO1
GC09M124353	6.39	https://www.genecards.org/cgi-bin/carddisp.pl?gene=PSMB7
GC21M047163	6.39	https://www.genecards.org/cgi-bin/carddisp.pl?gene=LSS
GC18M059327	6.39	https://www.genecards.org/cgi-bin/carddisp.pl?gene=LMAN1
GC16P089623	6.39	https://www.genecards.org/cgi-bin/carddisp.pl?gene=RPL13
GC06M041265	6.39	https://www.genecards.org/cgi-bin/carddisp.pl?gene=GRM4
GC19M005993	6.39	https://www.genecards.org/cgi-bin/carddisp.pl?gene=RFX2
GC03M048173	6.38	https://www.genecards.org/cgi-bin/carddisp.pl?gene=CDC25A

GC06P033521	6.38	https://www.genecards.org/cgi-bin/carddisp.pl?gene=RPS18
GC17M068420	6.37	https://www.genecards.org/cgi-bin/carddisp.pl?gene=WIPI1
GC19M010335	6.37	https://www.genecards.org/cgi-bin/carddisp.pl?gene=ICAM3
GC03P047802	6.36	https://www.genecards.org/cgi-bin/carddisp.pl?gene=DHX30
GC17P038869	6.36	https://www.genecards.org/cgi-bin/carddisp.pl?gene=LASP1
GC03M139355	6.36	https://www.genecards.org/cgi-bin/carddisp.pl?gene=COPB2
GC03P066120	6.35	https://www.genecards.org/cgi-bin/carddisp.pl?gene=SLC25A26
GC17M018365	6.35	https://www.genecards.org/cgi-bin/carddisp.pl?gene=SHMT1
GC06M002833	6.35	https://www.genecards.org/cgi-bin/carddisp.pl?gene=SERPINB1
GC14M080197	6.35	https://www.genecards.org/cgi-bin/carddisp.pl?gene=DIO2
GC12P015366	6.35	https://www.genecards.org/cgi-bin/carddisp.pl?gene=PTPRO
GC08P027311	6.35	https://www.genecards.org/cgi-bin/carddisp.pl?gene=PTK2B
GC04P184649	6.35	https://www.genecards.org/cgi-bin/carddisp.pl?gene=PRIMPOL
GC17M078971	6.34	https://www.genecards.org/cgi-bin/carddisp.pl?gene=LGALS3BP
GC01P182992	6.34	https://www.genecards.org/cgi-bin/carddisp.pl?gene=LAMC1
GC09P074497	6.33	https://www.genecards.org/cgi-bin/carddisp.pl?gene=RORB
GC02P061888	6.33	https://www.genecards.org/cgi-bin/carddisp.pl?gene=COMMD1
GC06P010492	6.33	https://www.genecards.org/cgi-bin/carddisp.pl?gene=GCNT2
GC22M032512	6.32	https://www.genecards.org/cgi-bin/carddisp.pl?gene=SYN3
GC05M064718	6.32	https://www.genecards.org/cgi-bin/carddisp.pl?gene=SREK1IP1
GC13M049531	6.32	https://www.genecards.org/cgi-bin/carddisp.pl?gene=RCBTB1
GC04P076214	6.32	https://www.genecards.org/cgi-bin/carddisp.pl?gene=FAM47E
GCMTP009993	6.32	https://www.genecards.org/cgi-bin/carddisp.pl?gene=MT-TG
GC14P025075	6.32	https://www.genecards.org/cgi-bin/carddisp.pl?gene=MHRT
GC01P171810	6.31	https://www.genecards.org/cgi-bin/carddisp.pl?gene=DNM3
GC19M039433	6.31	https://www.genecards.org/cgi-bin/carddisp.pl?gene=RPS16
GC04P139301	6.31	https://www.genecards.org/cgi-bin/carddisp.pl?gene=NAA15
GC19M003750	6.31	https://www.genecards.org/cgi-bin/carddisp.pl?gene=APBA3
GC15M078624	6.31	https://www.genecards.org/cgi-bin/carddisp.pl?gene=CHRNB4
GC01P196943	6.3	https://www.genecards.org/cgi-bin/carddisp.pl?gene=CFHR2
GC02M063892	6.3	https://www.genecards.org/cgi-bin/carddisp.pl?gene=VPS54
GC11P060088	6.3	https://www.genecards.org/cgi-bin/carddisp.pl?gene=MS4A2
GC19P037722	6.3	https://www.genecards.org/cgi-bin/carddisp.pl?gene=KMT2B
GC11P064206	6.3	https://www.genecards.org/cgi-bin/carddisp.pl?gene=FERMT3
GC09P035722	6.3	https://www.genecards.org/cgi-bin/carddisp.pl?gene=CREB3
GC09P035673	6.3	https://www.genecards.org/cgi-bin/carddisp.pl?gene=CA9
GC03P038453	6.3	https://www.genecards.org/cgi-bin/carddisp.pl?gene=ACVR2B
GC10P110644	6.3	https://www.genecards.org/cgi-bin/carddisp.pl?gene=RBM20
GC05M148451	6.3	https://www.genecards.org/cgi-bin/carddisp.pl?gene=HTR4
GC16P067173	6.28	https://www.genecards.org/cgi-bin/carddisp.pl?gene=NOL3
GC0XP048476	6.28	https://www.genecards.org/cgi-bin/carddisp.pl?gene=FTSJ1
GC14P024146	6.28	https://www.genecards.org/cgi-bin/carddisp.pl?gene=RNF31
GC19P045075	6.28	https://www.genecards.org/cgi-bin/carddisp.pl?gene=GEMIN7
GC03P042016	6.28	https://www.genecards.org/cgi-bin/carddisp.pl?gene=TRAK1
GC19P004910	6.28	https://www.genecards.org/cgi-bin/carddisp.pl?gene=UHRF1
GC17P045844	6.27	https://www.genecards.org/cgi-bin/carddisp.pl?gene=SPPL2C
GC17P044557	6.27	https://www.genecards.org/cgi-bin/carddisp.pl?gene=FZD2

GC02P186589	6.27	https://www.genecards.org/cgi-bin/carddisp.pl?gene=ITGAV
GC06M041934	6.27	https://www.genecards.org/cgi-bin/carddisp.pl?gene=CCND3
GC20P035455	6.27	https://www.genecards.org/cgi-bin/carddisp.pl?gene=CEP250
GC13U901530	6.27	https://www.genecards.org/cgi-bin/carddisp.pl?gene=LOC109461478
GC01P109250	6.26	https://www.genecards.org/cgi-bin/carddisp.pl?gene=CELSR2
GC08P017246	6.26	https://www.genecards.org/cgi-bin/carddisp.pl?gene=VPS37A
GC11P046402	6.26	https://www.genecards.org/cgi-bin/carddisp.pl?gene=MDK
GC19M056810	6.26	https://www.genecards.org/cgi-bin/carddisp.pl?gene=PEG3
GC13U900338	6.26	https://www.genecards.org/cgi-bin/carddisp.pl?gene=ATXN8
GC07M137227	6.25	https://www.genecards.org/cgi-bin/carddisp.pl?gene=PTN
GC02M010056	6.25	https://www.genecards.org/cgi-bin/carddisp.pl?gene=CYS1
GC14M050975	6.25	https://www.genecards.org/cgi-bin/carddisp.pl?gene=TRIM9
GC04M000979	6.25	https://www.genecards.org/cgi-bin/carddisp.pl?gene=SLC26A1
GC01P063774	6.24	https://www.genecards.org/cgi-bin/carddisp.pl?gene=ROR1
GC12M013382	6.24	https://www.genecards.org/cgi-bin/carddisp.pl?gene=YBX3
GC11M072002	6.24	https://www.genecards.org/cgi-bin/carddisp.pl?gene=NUMA1
GC03P050648	6.24	https://www.genecards.org/cgi-bin/carddisp.pl?gene=MAPKAPK3
GC09P095871	6.24	https://www.genecards.org/cgi-bin/carddisp.pl?gene=ERCC6L2
GC01M056994	6.24	https://www.genecards.org/cgi-bin/carddisp.pl?gene=DAB1
GC22P017628	6.24	https://www.genecards.org/cgi-bin/carddisp.pl?gene=BCL2L13
GC20M033407	6.24	https://www.genecards.org/cgi-bin/carddisp.pl?gene=SNTA1
GC03P096814	6.24	https://www.genecards.org/cgi-bin/carddisp.pl?gene=EPHA6
GC11P102347	6.24	https://www.genecards.org/cgi-bin/carddisp.pl?gene=BIRC2
GC01P033081	6.24	https://www.genecards.org/cgi-bin/carddisp.pl?gene=AZIN2
GC07P044808	6.24	https://www.genecards.org/cgi-bin/carddisp.pl?gene=PPIA
GC08P010054	6.23	https://www.genecards.org/cgi-bin/carddisp.pl?gene=MSRA
GC05M079372	6.23	https://www.genecards.org/cgi-bin/carddisp.pl?gene=HOMER1
GC02M074136	6.23	https://www.genecards.org/cgi-bin/carddisp.pl?gene=BOLA3
GC22M046360	6.22	https://www.genecards.org/cgi-bin/carddisp.pl?gene=CELSR1
GC03M049805	6.22	https://www.genecards.org/cgi-bin/carddisp.pl?gene=UBA7
GC02P178112	6.21	https://www.genecards.org/cgi-bin/carddisp.pl?gene=RBM45
GC05M140558	6.21	https://www.genecards.org/cgi-bin/carddisp.pl?gene=APBB3
GC0XM019552	6.2	https://www.genecards.org/cgi-bin/carddisp.pl?gene=SH3KBP1
GC17P032142	6.2	https://www.genecards.org/cgi-bin/carddisp.pl?gene=RHOT1
GC03P160415	6.2	https://www.genecards.org/cgi-bin/carddisp.pl?gene=MIR16-2
GC20M000435	6.2	https://www.genecards.org/cgi-bin/carddisp.pl?gene=TBC1D20
GC12M056639	6.2	https://www.genecards.org/cgi-bin/carddisp.pl?gene=ATP5F1B
GC01M155659	6.2	https://www.genecards.org/cgi-bin/carddisp.pl?gene=YY1AP1
GC09M129738	6.2	https://www.genecards.org/cgi-bin/carddisp.pl?gene=PTGES
GCMTM005763	6.2	https://www.genecards.org/cgi-bin/carddisp.pl?gene=MT-TC
GC15M064155	6.19	https://www.genecards.org/cgi-bin/carddisp.pl?gene=PPIB
GC01P178725	6.19	https://www.genecards.org/cgi-bin/carddisp.pl?gene=RALGPS2
GC11M065122	6.19	https://www.genecards.org/cgi-bin/carddisp.pl?gene=SYVN1
GC01M203340	6.18	https://www.genecards.org/cgi-bin/carddisp.pl?gene=FMOD
GC12M056341	6.18	https://www.genecards.org/cgi-bin/carddisp.pl?gene=STAT2
GC19M013986	6.18	https://www.genecards.org/cgi-bin/carddisp.pl?gene=MIR24-2
GC14M103519	6.17	https://www.genecards.org/cgi-bin/carddisp.pl?gene=CKB

GC11P033278	6.17	https://www.genecards.org/cgi-bin/carddisp.pl?gene=HIPK3
GC09P098056	6.17	https://www.genecards.org/cgi-bin/carddisp.pl?gene=NANS
GC05P148383	6.17	https://www.genecards.org/cgi-bin/carddisp.pl?gene=FBXO38
GC14M100143	6.17	https://www.genecards.org/cgi-bin/carddisp.pl?gene=DEGS2
GC05P131170	6.17	https://www.genecards.org/cgi-bin/carddisp.pl?gene=LYRM7
GC04P067558	6.16	https://www.genecards.org/cgi-bin/carddisp.pl?gene=STAP1
GC12P110124	6.16	https://www.genecards.org/cgi-bin/carddisp.pl?gene=IFT81
GC01P226062	6.16	https://www.genecards.org/cgi-bin/carddisp.pl?gene=H3-3A
GC01M150809	6.16	https://www.genecards.org/cgi-bin/carddisp.pl?gene=ARNT
GC06M031727	6.15	https://www.genecards.org/cgi-bin/carddisp.pl?gene=DDAH2
GC05M151743	6.15	https://www.genecards.org/cgi-bin/carddisp.pl?gene=ATOX1
GC01P169662	6.14	https://www.genecards.org/cgi-bin/carddisp.pl?gene=C1orf112
GC09M109130	6.14	https://www.genecards.org/cgi-bin/carddisp.pl?gene=FRRS1L
GC06M041274	6.14	https://www.genecards.org/cgi-bin/carddisp.pl?gene=RPS10
GC10P028685	6.14	https://www.genecards.org/cgi-bin/carddisp.pl?gene=BAMBI
GC02P233671	6.14	https://www.genecards.org/cgi-bin/carddisp.pl?gene=UGT1A9
GC01P241532	6.13	https://www.genecards.org/cgi-bin/carddisp.pl?gene=KMO
GC11P065711	6.12	https://www.genecards.org/cgi-bin/carddisp.pl?gene=KAT5
GC03M058428	6.12	https://www.genecards.org/cgi-bin/carddisp.pl?gene=PDHB
GC10P100347	6.12	https://www.genecards.org/cgi-bin/carddisp.pl?gene=SCD
GC22P031944	6.12	https://www.genecards.org/cgi-bin/carddisp.pl?gene=YWHAH
GC22M022547	6.12	https://www.genecards.org/cgi-bin/carddisp.pl?gene=PRAME
GC05M181286	6.12	https://www.genecards.org/cgi-bin/carddisp.pl?gene=RACK1
GC09P122264	6.12	https://www.genecards.org/cgi-bin/carddisp.pl?gene=MRRF
GC09P099821	6.11	https://www.genecards.org/cgi-bin/carddisp.pl?gene=NR4A3
GC02P173354	6.11	https://www.genecards.org/cgi-bin/carddisp.pl?gene=CDCA7
GC08M017643	6.11	https://www.genecards.org/cgi-bin/carddisp.pl?gene=MTUS1
GC02M098599	6.11	https://www.genecards.org/cgi-bin/carddisp.pl?gene=COA5
GC14P065411	6.11	https://www.genecards.org/cgi-bin/carddisp.pl?gene=FUT8
GC03P142596	6.11	https://www.genecards.org/cgi-bin/carddisp.pl?gene=PLS1
GC22M043039	6.11	https://www.genecards.org/cgi-bin/carddisp.pl?gene=TTLL1
GC12P118981	6.11	https://www.genecards.org/cgi-bin/carddisp.pl?gene=SRRM4
GC21P037006	6.11	https://www.genecards.org/cgi-bin/carddisp.pl?gene=RIPPLY3
GC15M059780	6.1	https://www.genecards.org/cgi-bin/carddisp.pl?gene=GNB5
GC01M168080	6.1	https://www.genecards.org/cgi-bin/carddisp.pl?gene=GPR161
GC01M061681	6.1	https://www.genecards.org/cgi-bin/carddisp.pl?gene=TM2D1
GC02M073762	6.1	https://www.genecards.org/cgi-bin/carddisp.pl?gene=DUSP11
GC02P100803	6.1	https://www.genecards.org/cgi-bin/carddisp.pl?gene=NPAS2
GC08P089758	6.09	https://www.genecards.org/cgi-bin/carddisp.pl?gene=RIPK2
GC03M192139	6.09	https://www.genecards.org/cgi-bin/carddisp.pl?gene=FGF12
GC03M184259	6.09	https://www.genecards.org/cgi-bin/carddisp.pl?gene=CAMK2N2
GC11M133898	6.09	https://www.genecards.org/cgi-bin/carddisp.pl?gene=MIR4697
GC01P159259	6.08	https://www.genecards.org/cgi-bin/carddisp.pl?gene=FCER1A
GC20P049936	6.08	https://www.genecards.org/cgi-bin/carddisp.pl?gene=RNF114
GC10P073744	6.08	https://www.genecards.org/cgi-bin/carddisp.pl?gene=SEC24C
GC04M013296	6.08	https://www.genecards.org/cgi-bin/carddisp.pl?gene=RAB28
GC17P041966	6.07	https://www.genecards.org/cgi-bin/carddisp.pl?gene=CNP

GC05P043638	6.07	https://www.genecards.org/cgi-bin/carddisp.pl?gene=NNT
GC19M013144	6.07	https://www.genecards.org/cgi-bin/carddisp.pl?gene=STX10
GC11P075719	6.07	https://www.genecards.org/cgi-bin/carddisp.pl?gene=RPS3
GC03M128619	6.06	https://www.genecards.org/cgi-bin/carddisp.pl?gene=RPN1
GC03P052495	6.06	https://www.genecards.org/cgi-bin/carddisp.pl?gene=STAB1
GC19M041461	6.06	https://www.genecards.org/cgi-bin/carddisp.pl?gene=SERTAD3
GC17M006643	6.06	https://www.genecards.org/cgi-bin/carddisp.pl?gene=MED31
GC17M009896	6.05	https://www.genecards.org/cgi-bin/carddisp.pl?gene=RCVRN
GC04P003766	6.05	https://www.genecards.org/cgi-bin/carddisp.pl?gene=ADRA2C
GC14P104467	6.05	https://www.genecards.org/cgi-bin/carddisp.pl?gene=MIR485
GC05P154833	6.05	https://www.genecards.org/cgi-bin/carddisp.pl?gene=CNOT8
GC06P069866	6.05	https://www.genecards.org/cgi-bin/carddisp.pl?gene=COL19A1
GC17P034356	6.05	https://www.genecards.org/cgi-bin/carddisp.pl?gene=CCL13
GC16M074448	6.05	https://www.genecards.org/cgi-bin/carddisp.pl?gene=GLG1
GC05M150219	6.04	https://www.genecards.org/cgi-bin/carddisp.pl?gene=CAMK2A
GC17P058218	6.03	https://www.genecards.org/cgi-bin/carddisp.pl?gene=LPO
GC06P129978	6.03	https://www.genecards.org/cgi-bin/carddisp.pl?gene=L3MBTL3
GC15P042273	6.03	https://www.genecards.org/cgi-bin/carddisp.pl?gene=GANC
GC02M130344	6.02	https://www.genecards.org/cgi-bin/carddisp.pl?gene=CCDC115
GC19M004174	6.02	https://www.genecards.org/cgi-bin/carddisp.pl?gene=SIRT6
GC19M018899	6.02	https://www.genecards.org/cgi-bin/carddisp.pl?gene=COPE
GC01P016472	6.02	https://www.genecards.org/cgi-bin/carddisp.pl?gene=LINC01772
GC11M066286	6.02	https://www.genecards.org/cgi-bin/carddisp.pl?gene=YIF1A
GC03M051957	6.02	https://www.genecards.org/cgi-bin/carddisp.pl?gene=PCBP4
GC0XP011822	6.02	https://www.genecards.org/cgi-bin/carddisp.pl?gene=FRMPD4
GC10P035126	6	https://www.genecards.org/cgi-bin/carddisp.pl?gene=CREM
GC01M030869	6	https://www.genecards.org/cgi-bin/carddisp.pl?gene=SDC3
GC0XM053936	6	https://www.genecards.org/cgi-bin/carddisp.pl?gene=PHF8
GC12P100357	6	https://www.genecards.org/cgi-bin/carddisp.pl?gene=SLC17A8
GC12M122983	5.99	https://www.genecards.org/cgi-bin/carddisp.pl?gene=PITPNM2
GC11P034894	5.99	https://www.genecards.org/cgi-bin/carddisp.pl?gene=PDHX
GC22M031135	5.99	https://www.genecards.org/cgi-bin/carddisp.pl?gene=PLA2G3
GC19M018868	5.99	https://www.genecards.org/cgi-bin/carddisp.pl?gene=CERS1
GC01M154924	5.98	https://www.genecards.org/cgi-bin/carddisp.pl?gene=PMVK
GC09U900019	5.98	https://www.genecards.org/cgi-bin/carddisp.pl?gene=SPG19
GC04M048137	5.98	https://www.genecards.org/cgi-bin/carddisp.pl?gene=TEC
GC06P096562	5.98	https://www.genecards.org/cgi-bin/carddisp.pl?gene=FHL5
GC11M001470	5.98	https://www.genecards.org/cgi-bin/carddisp.pl?gene=MOB2
GC01P156061	5.97	https://www.genecards.org/cgi-bin/carddisp.pl?gene=RAB25
GC11P095789	5.97	https://www.genecards.org/cgi-bin/carddisp.pl?gene=CEP57
GC08M081524	5.97	https://www.genecards.org/cgi-bin/carddisp.pl?gene=FABP12
GC07P002512	5.97	https://www.genecards.org/cgi-bin/carddisp.pl?gene=LFNG
GC17P019415	5.97	https://www.genecards.org/cgi-bin/carddisp.pl?gene=RNF112
GC02P142748	5.97	https://www.genecards.org/cgi-bin/carddisp.pl?gene=KYNU
GC21M032593	5.97	https://www.genecards.org/cgi-bin/carddisp.pl?gene=CFAP298
GC14P064535	5.96	https://www.genecards.org/cgi-bin/carddisp.pl?gene=HSPA2
GC05M157137	5.96	https://www.genecards.org/cgi-bin/carddisp.pl?gene=MED7

GC01U901207	5.96	https://www.genecards.org/cgi-bin/carddisp.pl?gene=SPG29
GC06M041312	5.96	https://www.genecards.org/cgi-bin/carddisp.pl?gene=GLO1
GC05P033441	5.96	https://www.genecards.org/cgi-bin/carddisp.pl?gene=TARS1
GC20M063095	5.95	https://www.genecards.org/cgi-bin/carddisp.pl?gene=HAR1B
GC02P200585	5.94	https://www.genecards.org/cgi-bin/carddisp.pl?gene=AOX1
GC19P041750	5.94	https://www.genecards.org/cgi-bin/carddisp.pl?gene=CEACAM6
GC10M005498	5.94	https://www.genecards.org/cgi-bin/carddisp.pl?gene=CALML5
GC15M036889	5.94	https://www.genecards.org/cgi-bin/carddisp.pl?gene=MEIS2
GC14P077760	5.94	https://www.genecards.org/cgi-bin/carddisp.pl?gene=C14orf178
GC20P063101	5.94	https://www.genecards.org/cgi-bin/carddisp.pl?gene=HAR1A
GC08P023528	5.93	https://www.genecards.org/cgi-bin/carddisp.pl?gene=SLC25A37
GC08P135457	5.93	https://www.genecards.org/cgi-bin/carddisp.pl?gene=KHDRBS3
GC10P049942	5.93	https://www.genecards.org/cgi-bin/carddisp.pl?gene=TIMM23B
GC02P169791	5.93	https://www.genecards.org/cgi-bin/carddisp.pl?gene=SSB
GC06M083553	5.92	https://www.genecards.org/cgi-bin/carddisp.pl?gene=SNAP91
GC03P023960	5.92	https://www.genecards.org/cgi-bin/carddisp.pl?gene=RPL15
GC01P026873	5.92	https://www.genecards.org/cgi-bin/carddisp.pl?gene=SFN
GC01P050025	5.91	https://www.genecards.org/cgi-bin/carddisp.pl?gene=ELAVL4
GC09M021227	5.91	https://www.genecards.org/cgi-bin/carddisp.pl?gene=IFNA17
GC06M158972	5.91	https://www.genecards.org/cgi-bin/carddisp.pl?gene=RSPH3
GC11P118403	5.9	https://www.genecards.org/cgi-bin/carddisp.pl?gene=ATP5MG
GC06M030653	5.9	https://www.genecards.org/cgi-bin/carddisp.pl?gene=DHX16
GC15P097429	5.9	https://www.genecards.org/cgi-bin/carddisp.pl?gene=MCTP2
GC0XM153701	5.9	https://www.genecards.org/cgi-bin/carddisp.pl?gene=BCAP31
GC0XP002691	5.9	https://www.genecards.org/cgi-bin/carddisp.pl?gene=CD99
GC01M215630	5.89	https://www.genecards.org/cgi-bin/carddisp.pl?gene=ENSG00000202498
GC18P063871	5.89	https://www.genecards.org/cgi-bin/carddisp.pl?gene=SERPINB2
GC22P039078	5.88	https://www.genecards.org/cgi-bin/carddisp.pl?gene=APOBEC3G
GC20M003783	5.88	https://www.genecards.org/cgi-bin/carddisp.pl?gene=CENPB
GC11P009573	5.88	https://www.genecards.org/cgi-bin/carddisp.pl?gene=WEE1
GC17M008878	5.86	https://www.genecards.org/cgi-bin/carddisp.pl?gene=PIK3R5
GC11M065901	5.86	https://www.genecards.org/cgi-bin/carddisp.pl?gene=FIBP
GC0XM015345	5.86	https://www.genecards.org/cgi-bin/carddisp.pl?gene=VEGFD
GC07P079769	5.86	https://www.genecards.org/cgi-bin/carddisp.pl?gene=GNAI1
GC03M139517	5.86	https://www.genecards.org/cgi-bin/carddisp.pl?gene=RBP1
GC19M011505	5.86	https://www.genecards.org/cgi-bin/carddisp.pl?gene=ECSIT
GC20P020368	5.85	https://www.genecards.org/cgi-bin/carddisp.pl?gene=INSM1
GC19M049024	5.85	https://www.genecards.org/cgi-bin/carddisp.pl?gene=CGB3
GC05M168549	5.83	https://www.genecards.org/cgi-bin/carddisp.pl?gene=PANK3
GC12P042238	5.83	https://www.genecards.org/cgi-bin/carddisp.pl?gene=PPHLN1
GC02P154698	5.83	https://www.genecards.org/cgi-bin/carddisp.pl?gene=KCNJ3
GC14M021032	5.83	https://www.genecards.org/cgi-bin/carddisp.pl?gene=RNASE13
GC01P162069	5.83	https://www.genecards.org/cgi-bin/carddisp.pl?gene=NOS1AP
GC07M128298	5.83	https://www.genecards.org/cgi-bin/carddisp.pl?gene=RBM28
GC04P007043	5.82	https://www.genecards.org/cgi-bin/carddisp.pl?gene=TADA2B
GC0XP055717	5.82	https://www.genecards.org/cgi-bin/carddisp.pl?gene=RRAGB
GC09P113275	5.82	https://www.genecards.org/cgi-bin/carddisp.pl?gene=PRPF4

GC16M004189	5.81	https://www.genecards.org/cgi-bin/carddisp.pl?gene=SRL
GC08M121594	5.8	https://www.genecards.org/cgi-bin/carddisp.pl?gene=HAS2
GC03P126988	5.8	https://www.genecards.org/cgi-bin/carddisp.pl?gene=PLXNA1
GC15M088898	5.8	https://www.genecards.org/cgi-bin/carddisp.pl?gene=MFGE8
GC19P018563	5.79	https://www.genecards.org/cgi-bin/carddisp.pl?gene=UBA52
GC01M205829	5.79	https://www.genecards.org/cgi-bin/carddisp.pl?gene=PM20D1
GC03M049202	5.79	https://www.genecards.org/cgi-bin/carddisp.pl?gene=MIR191
GC05M132875	5.79	https://www.genecards.org/cgi-bin/carddisp.pl?gene=AFF4
GC17M075780	5.79	https://www.genecards.org/cgi-bin/carddisp.pl?gene=H3-3B
GC11P066257	5.78	https://www.genecards.org/cgi-bin/carddisp.pl?gene=KLC2
GC19P039125	5.78	https://www.genecards.org/cgi-bin/carddisp.pl?gene=PAK4
GC19P040750	5.78	https://www.genecards.org/cgi-bin/carddisp.pl?gene=SNRPA
GC04P039700	5.78	https://www.genecards.org/cgi-bin/carddisp.pl?gene=UBE2K
GC0XP115560	5.78	https://www.genecards.org/cgi-bin/carddisp.pl?gene=PLS3
GC18M021826	5.77	https://www.genecards.org/cgi-bin/carddisp.pl?gene=MIR133A1
GC06P046035	5.77	https://www.genecards.org/cgi-bin/carddisp.pl?gene=RPL10A
GC17P040443	5.77	https://www.genecards.org/cgi-bin/carddisp.pl?gene=IGFBP4
GC11P102048	5.77	https://www.genecards.org/cgi-bin/carddisp.pl?gene=CFAP300
GC01M225919	5.77	https://www.genecards.org/cgi-bin/carddisp.pl?gene=PYCR2
GC12M112320	5.76	https://www.genecards.org/cgi-bin/carddisp.pl?gene=RPL6
GC07Pi00773	5.76	https://www.genecards.org/cgi-bin/carddisp.pl?gene=PRSS3P2
GC07M142942	5.75	https://www.genecards.org/cgi-bin/carddisp.pl?gene=KEL
GC17P004808	5.75	https://www.genecards.org/cgi-bin/carddisp.pl?gene=PLD2
GC08U900825	5.75	https://www.genecards.org/cgi-bin/carddisp.pl?gene=SPG37
GC09P111525	5.75	https://www.genecards.org/cgi-bin/carddisp.pl?gene=ZNF483
GC04P017578	5.75	https://www.genecards.org/cgi-bin/carddisp.pl?gene=LAP3
GC16M058157	5.74	https://www.genecards.org/cgi-bin/carddisp.pl?gene=CSNK2A2
GC19P008444	5.74	https://www.genecards.org/cgi-bin/carddisp.pl?gene=HNRNPM
GC19P056568	5.74	https://www.genecards.org/cgi-bin/carddisp.pl?gene=ZNF470
GC01M181282	5.74	https://www.genecards.org/cgi-bin/carddisp.pl?gene=TOR1AIP2
GC10P050739	5.74	https://www.genecards.org/cgi-bin/carddisp.pl?gene=ASAH2B
GC17M058000	5.74	https://www.genecards.org/cgi-bin/carddisp.pl?gene=SRSF1
GC22P041367	5.74	https://www.genecards.org/cgi-bin/carddisp.pl?gene=TEF
GC05M082273	5.74	https://www.genecards.org/cgi-bin/carddisp.pl?gene=RPS23
GC11M061301	5.74	https://www.genecards.org/cgi-bin/carddisp.pl?gene=DDB1
GC02M160099	5.74	https://www.genecards.org/cgi-bin/carddisp.pl?gene=ITGB6
GC05M042800	5.73	https://www.genecards.org/cgi-bin/carddisp.pl?gene=SELENOP
GC10M131966	5.73	https://www.genecards.org/cgi-bin/carddisp.pl?gene=BNIP3
GC06M032934	5.73	https://www.genecards.org/cgi-bin/carddisp.pl?gene=HLA-DMB
GC05M132689	5.73	https://www.genecards.org/cgi-bin/carddisp.pl?gene=KIF3A
GC12P054301	5.72	https://www.genecards.org/cgi-bin/carddisp.pl?gene=COPZ1
GC0XM073820	5.72	https://www.genecards.org/cgi-bin/carddisp.pl?gene=XIST
GC05P087267	5.72	https://www.genecards.org/cgi-bin/carddisp.pl?gene=RASA1
GC22M017734	5.72	https://www.genecards.org/cgi-bin/carddisp.pl?gene=BID
GC13P072782	5.72	https://www.genecards.org/cgi-bin/carddisp.pl?gene=PIBF1
GC11P066270	5.72	https://www.genecards.org/cgi-bin/carddisp.pl?gene=RAB1B
GC02M207762	5.71	https://www.genecards.org/cgi-bin/carddisp.pl?gene=FZD5

GC12M039755	5.71	https://www.genecards.org/cgi-bin/carddisp.pl?gene=SLC2A13
GC07M122073	5.71	https://www.genecards.org/cgi-bin/carddisp.pl?gene=AASS
GC19M037410	5.71	https://www.genecards.org/cgi-bin/carddisp.pl?gene=ZNF569
GC07M101205	5.71	https://www.genecards.org/cgi-bin/carddisp.pl?gene=PLOD3
GC01P155208	5.71	https://www.genecards.org/cgi-bin/carddisp.pl?gene=MTX1
GC20P044885	5.71	https://www.genecards.org/cgi-bin/carddisp.pl?gene=YWHAB
GC11U901446	5.71	https://www.genecards.org/cgi-bin/carddisp.pl?gene=SPG41
GC04U900828	5.71	https://www.genecards.org/cgi-bin/carddisp.pl?gene=SPG38
GC04P076252	5.71	https://www.genecards.org/cgi-bin/carddisp.pl?gene=FAM47E-STBD1
GC06M033004	5.7	https://www.genecards.org/cgi-bin/carddisp.pl?gene=HLA-DOA
GC12M055954	5.7	https://www.genecards.org/cgi-bin/carddisp.pl?gene=PMEL
GC10M119140	5.69	https://www.genecards.org/cgi-bin/carddisp.pl?gene=SFXN4
GC01P011273	5.69	https://www.genecards.org/cgi-bin/carddisp.pl?gene=UBIAD1
GC14P104253	5.68	https://www.genecards.org/cgi-bin/carddisp.pl?gene=MIR370
GC16M001939	5.68	https://www.genecards.org/cgi-bin/carddisp.pl?gene=MSRB1
GC0XP110944	5.68	https://www.genecards.org/cgi-bin/carddisp.pl?gene=PAK3
GC02P064988	5.68	https://www.genecards.org/cgi-bin/carddisp.pl?gene=SLC1A4
GC19P008393	5.67	https://www.genecards.org/cgi-bin/carddisp.pl?gene=RAB11B
GC02P085538	5.67	https://www.genecards.org/cgi-bin/carddisp.pl?gene=MAT2A
GC0XM077826	5.67	https://www.genecards.org/cgi-bin/carddisp.pl?gene=MAGT1
GC06P096924	5.67	https://www.genecards.org/cgi-bin/carddisp.pl?gene=KLHL32
GC09M074982	5.67	https://www.genecards.org/cgi-bin/carddisp.pl?gene=CARNMT1
GC04M102794	5.67	https://www.genecards.org/cgi-bin/carddisp.pl?gene=UBE2D3
GC01M111499	5.67	https://www.genecards.org/cgi-bin/carddisp.pl?gene=ADORA3
GC02M105343	5.67	https://www.genecards.org/cgi-bin/carddisp.pl?gene=FHL2
GC01P173477	5.66	https://www.genecards.org/cgi-bin/carddisp.pl?gene=PRDX6
GC0XM134624	5.66	https://www.genecards.org/cgi-bin/carddisp.pl?gene=MIR503
GC12P094150	5.66	https://www.genecards.org/cgi-bin/carddisp.pl?gene=PLXNC1
GC06M024651	5.65	https://www.genecards.org/cgi-bin/carddisp.pl?gene=TDP2
GC20M022581	5.65	https://www.genecards.org/cgi-bin/carddisp.pl?gene=FOXA2
GC03P032123	5.65	https://www.genecards.org/cgi-bin/carddisp.pl?gene=GPD1L
GC01M211658	5.64	https://www.genecards.org/cgi-bin/carddisp.pl?gene=NEK2
GC06P105993	5.64	https://www.genecards.org/cgi-bin/carddisp.pl?gene=PRDM1
GC06P046065	5.64	https://www.genecards.org/cgi-bin/carddisp.pl?gene=PIM1
GC0XM108720	5.63	https://www.genecards.org/cgi-bin/carddisp.pl?gene=IRS4
GC20P032052	5.62	https://www.genecards.org/cgi-bin/carddisp.pl?gene=HCK
GC17P076376	5.62	https://www.genecards.org/cgi-bin/carddisp.pl?gene=SPHK1
GC21M014961	5.61	https://www.genecards.org/cgi-bin/carddisp.pl?gene=NRIP1
GC12P093677	5.61	https://www.genecards.org/cgi-bin/carddisp.pl?gene=CRADD
GC01P107140	5.61	https://www.genecards.org/cgi-bin/carddisp.pl?gene=NTNG1
GC02P209771	5.61	https://www.genecards.org/cgi-bin/carddisp.pl?gene=UNC80
GC11M062576	5.61	https://www.genecards.org/cgi-bin/carddisp.pl?gene=TUT1
GC14P104460	5.6	https://www.genecards.org/cgi-bin/carddisp.pl?gene=MIR409
GC01P201955	5.6	https://www.genecards.org/cgi-bin/carddisp.pl?gene=TIMM17A
GC09M023690	5.6	https://www.genecards.org/cgi-bin/carddisp.pl?gene=ELAVL2
GC19P055388	5.59	https://www.genecards.org/cgi-bin/carddisp.pl?gene=RPL28
GC01M145992	5.59	https://www.genecards.org/cgi-bin/carddisp.pl?gene=TXNIP

GC16P072097	5.59	https://www.genecards.org/cgi-bin/carddisp.pl?gene=HPR
GC09M137174	5.58	https://www.genecards.org/cgi-bin/carddisp.pl?gene=ANAPC2
GC01P206684	5.58	https://www.genecards.org/cgi-bin/carddisp.pl?gene=MAPKAPK2
GC11M134378	5.58	https://www.genecards.org/cgi-bin/carddisp.pl?gene=B3GAT1
GC03P051385	5.58	https://www.genecards.org/cgi-bin/carddisp.pl?gene=MANF
GC03P184174	5.58	https://www.genecards.org/cgi-bin/carddisp.pl?gene=AP2M1
GC19P042268	5.57	https://www.genecards.org/cgi-bin/carddisp.pl?gene=CIC
GC03P050226	5.57	https://www.genecards.org/cgi-bin/carddisp.pl?gene=GNAI2
GC0XM153588	5.57	https://www.genecards.org/cgi-bin/carddisp.pl?gene=CCNQ
GC01P028506	5.56	https://www.genecards.org/cgi-bin/carddisp.pl?gene=SNHG3
GC04P020287	5.56	https://www.genecards.org/cgi-bin/carddisp.pl?gene=SLIT2
GC22M044617	5.56	https://www.genecards.org/cgi-bin/carddisp.pl?gene=CSNK1E
GC19M049806	5.55	https://www.genecards.org/cgi-bin/carddisp.pl?gene=FUZ
GC15M064544	5.55	https://www.genecards.org/cgi-bin/carddisp.pl?gene=RPS27L
GC03P112332	5.55	https://www.genecards.org/cgi-bin/carddisp.pl?gene=CD200
GC04P005053	5.55	https://www.genecards.org/cgi-bin/carddisp.pl?gene=STK32B
GC09P035161	5.54	https://www.genecards.org/cgi-bin/carddisp.pl?gene=UNC13B
GC14P067819	5.53	https://www.genecards.org/cgi-bin/carddisp.pl?gene=RAD51B
GC09P033025	5.53	https://www.genecards.org/cgi-bin/carddisp.pl?gene=DNAJA1
GC10P087659	5.53	https://www.genecards.org/cgi-bin/carddisp.pl?gene=PAPSS2
GC04P112636	5.53	https://www.genecards.org/cgi-bin/carddisp.pl?gene=LARP7
GC16P056626	5.52	https://www.genecards.org/cgi-bin/carddisp.pl?gene=MT2A
GC17M004733	5.52	https://www.genecards.org/cgi-bin/carddisp.pl?gene=CXCL16
GC16P046885	5.52	https://www.genecards.org/cgi-bin/carddisp.pl?gene=GPT2
GC05P150601	5.52	https://www.genecards.org/cgi-bin/carddisp.pl?gene=SYNPO
GC10M043385	5.51	https://www.genecards.org/cgi-bin/carddisp.pl?gene=HNRNPF
GC11M017173	5.51	https://www.genecards.org/cgi-bin/carddisp.pl?gene=RPS13
GC01M033007	5.5	https://www.genecards.org/cgi-bin/carddisp.pl?gene=AK2
GC20P013925	5.5	https://www.genecards.org/cgi-bin/carddisp.pl?gene=MACROD2
GC01P040979	5.5	https://www.genecards.org/cgi-bin/carddisp.pl?gene=CTPS1
GC19P054102	5.49	https://www.genecards.org/cgi-bin/carddisp.pl?gene=NDUFA3
GC04M088520	5.49	https://www.genecards.org/cgi-bin/carddisp.pl?gene=PIGY
GC11M030009	5.49	https://www.genecards.org/cgi-bin/carddisp.pl?gene=KCNA4
GC03M064501	5.48	https://www.genecards.org/cgi-bin/carddisp.pl?gene=ADAMTS9
GC03M068975	5.48	https://www.genecards.org/cgi-bin/carddisp.pl?gene=EOGT
GC0XP047154	5.48	https://www.genecards.org/cgi-bin/carddisp.pl?gene=RBM10
GC12P021437	5.48	https://www.genecards.org/cgi-bin/carddisp.pl?gene=PYROXD1
GC09P135499	5.48	https://www.genecards.org/cgi-bin/carddisp.pl?gene=MRPS2
GC03P042804	5.48	https://www.genecards.org/cgi-bin/carddisp.pl?gene=ACKR2
GC0XM109623	5.47	https://www.genecards.org/cgi-bin/carddisp.pl?gene=KCNE5
GC01M071395	5.47	https://www.genecards.org/cgi-bin/carddisp.pl?gene=NEGR1
GC08P096645	5.47	https://www.genecards.org/cgi-bin/carddisp.pl?gene=CPQ
GC17P069414	5.46	https://www.genecards.org/cgi-bin/carddisp.pl?gene=MAP2K6
GC09P135561	5.46	https://www.genecards.org/cgi-bin/carddisp.pl?gene=PAEP
GC16P003500	5.46	https://www.genecards.org/cgi-bin/carddisp.pl?gene=CLUAP1
GC14M105736	5.46	https://www.genecards.org/cgi-bin/carddisp.pl?gene=IGHG1
GC11P059713	5.46	https://www.genecards.org/cgi-bin/carddisp.pl?gene=STX3

GC22M019846	5.46	https://www.genecards.org/cgi-bin/carddisp.pl?gene=RTL10
GC07M088205	5.46	https://www.genecards.org/cgi-bin/carddisp.pl?gene=SRI
GC01P182839	5.45	https://www.genecards.org/cgi-bin/carddisp.pl?gene=DHX9
GC01M054756	5.45	https://www.genecards.org/cgi-bin/carddisp.pl?gene=PARS2
GC10M069403	5.45	https://www.genecards.org/cgi-bin/carddisp.pl?gene=TACR2
GC14M092703	5.45	https://www.genecards.org/cgi-bin/carddisp.pl?gene=LGMN
GC06M160173	5.45	https://www.genecards.org/cgi-bin/carddisp.pl?gene=SLC22A2
GC22M037570	5.45	https://www.genecards.org/cgi-bin/carddisp.pl?gene=LGALS2
GC03P040458	5.44	https://www.genecards.org/cgi-bin/carddisp.pl?gene=RPL14
GC01M058415	5.44	https://www.genecards.org/cgi-bin/carddisp.pl?gene=OMA1
GC14M037589	5.44	https://www.genecards.org/cgi-bin/carddisp.pl?gene=FOXA1
GC08M143579	5.43	https://www.genecards.org/cgi-bin/carddisp.pl?gene=EEF1D
GC05P177403	5.43	https://www.genecards.org/cgi-bin/carddisp.pl?gene=GRK6
GC20M024986	5.43	https://www.genecards.org/cgi-bin/carddisp.pl?gene=ACSS1
GC10M119167	5.43	https://www.genecards.org/cgi-bin/carddisp.pl?gene=PRDX3
GC01M109279	5.43	https://www.genecards.org/cgi-bin/carddisp.pl?gene=PSRC1
GC02P085685	5.43	https://www.genecards.org/cgi-bin/carddisp.pl?gene=GNLY
GC03M052545	5.43	https://www.genecards.org/cgi-bin/carddisp.pl?gene=PBRM1
GC19M051023	5.43	https://www.genecards.org/cgi-bin/carddisp.pl?gene=KLK11
GC01P046394	5.43	https://www.genecards.org/cgi-bin/carddisp.pl?gene=FAAH
GC17P045755	5.42	https://www.genecards.org/cgi-bin/carddisp.pl?gene=LINC02210
GC05P071587	5.42	https://www.genecards.org/cgi-bin/carddisp.pl?gene=MCCC2
GC17M039904	5.42	https://www.genecards.org/cgi-bin/carddisp.pl?gene=GSDMB
GC17P075262	5.42	https://www.genecards.org/cgi-bin/carddisp.pl?gene=MRPS7
GC06P168441	5.42	https://www.genecards.org/cgi-bin/carddisp.pl?gene=SMOC2
GC09P124692	5.42	https://www.genecards.org/cgi-bin/carddisp.pl?gene=MIR181A2
GC11P057648	5.42	https://www.genecards.org/cgi-bin/carddisp.pl?gene=CLP1
GC14P077320	5.42	https://www.genecards.org/cgi-bin/carddisp.pl?gene=GSTZ1
GC11M010853	5.41	https://www.genecards.org/cgi-bin/carddisp.pl?gene=SNORD97
GC16M074621	5.41	https://www.genecards.org/cgi-bin/carddisp.pl?gene=RFWD3
GC02P177392	5.41	https://www.genecards.org/cgi-bin/carddisp.pl?gene=AGPS
GC06P163414	5.41	https://www.genecards.org/cgi-bin/carddisp.pl?gene=QKI
GC21P017512	5.41	https://www.genecards.org/cgi-bin/carddisp.pl?gene=CXADR
GC05M141583	5.41	https://www.genecards.org/cgi-bin/carddisp.pl?gene=HDAC3
GC15M023686	5.39	https://www.genecards.org/cgi-bin/carddisp.pl?gene=NDN
GC02M070296	5.39	https://www.genecards.org/cgi-bin/carddisp.pl?gene=FAM136A
GC16P001209	5.39	https://www.genecards.org/cgi-bin/carddisp.pl?gene=MPG
GC17M001003	5.39	https://www.genecards.org/cgi-bin/carddisp.pl?gene=ABR
GC08M098024	5.39	https://www.genecards.org/cgi-bin/carddisp.pl?gene=RPL30
GC14P105419	5.38	https://www.genecards.org/cgi-bin/carddisp.pl?gene=MTA1
GC01P158969	5.38	https://www.genecards.org/cgi-bin/carddisp.pl?gene=IFI16
GC03P052455	5.38	https://www.genecards.org/cgi-bin/carddisp.pl?gene=NISCH
GC17M038847	5.38	https://www.genecards.org/cgi-bin/carddisp.pl?gene=RPL23
GC22P020917	5.37	https://www.genecards.org/cgi-bin/carddisp.pl?gene=CRKL
GC02P003383	5.37	https://www.genecards.org/cgi-bin/carddisp.pl?gene=TRAPPC12
GC16P089680	5.37	https://www.genecards.org/cgi-bin/carddisp.pl?gene=CDK10
GC06P030626	5.37	https://www.genecards.org/cgi-bin/carddisp.pl?gene=ATAT1

GC01P031981	5.36	https://www.genecards.org/cgi-bin/carddisp.pl?gene=KHDRBS1
GC02M127638	5.36	https://www.genecards.org/cgi-bin/carddisp.pl?gene=LIMS2
GC17U902748	5.36	https://www.genecards.org/cgi-bin/carddisp.pl?gene=LOC111365141
GC04M001781	5.36	https://www.genecards.org/cgi-bin/carddisp.pl?gene=LETM1
GC10P110208	5.36	https://www.genecards.org/cgi-bin/carddisp.pl?gene=MXI1
GC09M033245	5.35	https://www.genecards.org/cgi-bin/carddisp.pl?gene=BAG1
GC15P026866	5.35	https://www.genecards.org/cgi-bin/carddisp.pl?gene=GABRA5
GC06M089257	5.35	https://www.genecards.org/cgi-bin/carddisp.pl?gene=GABRR2
GC05M177387	5.35	https://www.genecards.org/cgi-bin/carddisp.pl?gene=MXD3
GC10P103493	5.35	https://www.genecards.org/cgi-bin/carddisp.pl?gene=NEURL1
GC10M133278	5.35	https://www.genecards.org/cgi-bin/carddisp.pl?gene=TUBGCP2
GC10P097426	5.34	https://www.genecards.org/cgi-bin/carddisp.pl?gene=PGAM1
GC06M112070	5.33	https://www.genecards.org/cgi-bin/carddisp.pl?gene=TUBE1
GC11P022359	5.33	https://www.genecards.org/cgi-bin/carddisp.pl?gene=SLC17A6
GC02P219195	5.33	https://www.genecards.org/cgi-bin/carddisp.pl?gene=ZFAND2B
GC07P086643	5.33	https://www.genecards.org/cgi-bin/carddisp.pl?gene=GRM3
GC01M151050	5.33	https://www.genecards.org/cgi-bin/carddisp.pl?gene=CDC42SE1
GC02P045651	5.33	https://www.genecards.org/cgi-bin/carddisp.pl?gene=PRKCE
GC10P102714	5.33	https://www.genecards.org/cgi-bin/carddisp.pl?gene=SFXN2
GC14P059188	5.32	https://www.genecards.org/cgi-bin/carddisp.pl?gene=DAAM1
GC11P047290	5.32	https://www.genecards.org/cgi-bin/carddisp.pl?gene=MADD
GC01M019215	5.31	https://www.genecards.org/cgi-bin/carddisp.pl?gene=EMC1
GC21P044300	5.3	https://www.genecards.org/cgi-bin/carddisp.pl?gene=PFKL
GC01P075128	5.3	https://www.genecards.org/cgi-bin/carddisp.pl?gene=LHX8
GC01M027404	5.3	https://www.genecards.org/cgi-bin/carddisp.pl?gene=WASF2
GC02P063050	5.29	https://www.genecards.org/cgi-bin/carddisp.pl?gene=OTX1
GC06M029555	5.29	https://www.genecards.org/cgi-bin/carddisp.pl?gene=GABBR1
GC02M065048	5.29	https://www.genecards.org/cgi-bin/carddisp.pl?gene=RAB1A
GC03M128064	5.28	https://www.genecards.org/cgi-bin/carddisp.pl?gene=RUVBL1
GC11M102576	5.28	https://www.genecards.org/cgi-bin/carddisp.pl?gene=MMP20
GC10P119704	5.26	https://www.genecards.org/cgi-bin/carddisp.pl?gene=INPP5F
GC17P007351	5.26	https://www.genecards.org/cgi-bin/carddisp.pl?gene=KCTD11
GC08M042373	5.26	https://www.genecards.org/cgi-bin/carddisp.pl?gene=DKK4
GC0XP077447	5.26	https://www.genecards.org/cgi-bin/carddisp.pl?gene=FGF16
GC02P102186	5.26	https://www.genecards.org/cgi-bin/carddisp.pl?gene=IL1RL2
GC10M127877	5.26	https://www.genecards.org/cgi-bin/carddisp.pl?gene=CLRN3
GC08P090001	5.26	https://www.genecards.org/cgi-bin/carddisp.pl?gene=DECR1
GC10P017845	5.25	https://www.genecards.org/cgi-bin/carddisp.pl?gene=MIR511
GC01P028505	5.25	https://www.genecards.org/cgi-bin/carddisp.pl?gene=RCC1
GC19P054603	5.25	https://www.genecards.org/cgi-bin/carddisp.pl?gene=RPS9
GC16P056931	5.25	https://www.genecards.org/cgi-bin/carddisp.pl?gene=HERPUD1
GC05P170105	5.25	https://www.genecards.org/cgi-bin/carddisp.pl?gene=FOXI1
GC11M119015	5.24	https://www.genecards.org/cgi-bin/carddisp.pl?gene=RPS25
GC09P095098	5.24	https://www.genecards.org/cgi-bin/carddisp.pl?gene=MIR27B
GC01M026062	5.23	https://www.genecards.org/cgi-bin/carddisp.pl?gene=TRIM63
GC12M057145	5.23	https://www.genecards.org/cgi-bin/carddisp.pl?gene=LRP1-AS
GC04M106921	5.22	https://www.genecards.org/cgi-bin/carddisp.pl?gene=DKK2

GC09P036572	5.22	https://www.genecards.org/cgi-bin/carddisp.pl?gene=MELK
GC02M118842	5.22	https://www.genecards.org/cgi-bin/carddisp.pl?gene=EN1
GC03M063973	5.22	https://www.genecards.org/cgi-bin/carddisp.pl?gene=PSMD6
GC08P120060	5.21	https://www.genecards.org/cgi-bin/carddisp.pl?gene=COL14A1
GC19P049675	5.21	https://www.genecards.org/cgi-bin/carddisp.pl?gene=PRMT1
GC07P077537	5.21	https://www.genecards.org/cgi-bin/carddisp.pl?gene=PTPN12
GC22M036467	5.2	https://www.genecards.org/cgi-bin/carddisp.pl?gene=TXN2
GC18P024460	5.2	https://www.genecards.org/cgi-bin/carddisp.pl?gene=HRH4
GC13P079873	5.2	https://www.genecards.org/cgi-bin/carddisp.pl?gene=LINC01080
GC11M001274	5.2	https://www.genecards.org/cgi-bin/carddisp.pl?gene=TOLLIP
GC10P012349	5.2	https://www.genecards.org/cgi-bin/carddisp.pl?gene=CAMK1D
GC11P027513	5.2	https://www.genecards.org/cgi-bin/carddisp.pl?gene=BDNF-AS
GC03M036986	5.19	https://www.genecards.org/cgi-bin/carddisp.pl?gene=EPM2AIP1
GC15M063321	5.19	https://www.genecards.org/cgi-bin/carddisp.pl?gene=CA12
GC17P034980	5.19	https://www.genecards.org/cgi-bin/carddisp.pl?gene=LIG3
GC17P081683	5.19	https://www.genecards.org/cgi-bin/carddisp.pl?gene=HGS
GC12P007875	5.19	https://www.genecards.org/cgi-bin/carddisp.pl?gene=PTPN6
GC01M153805	5.19	https://www.genecards.org/cgi-bin/carddisp.pl?gene=GATAD2B
GC12P006970	5.18	https://www.genecards.org/cgi-bin/carddisp.pl?gene=EMG1
GC02M231108	5.18	https://www.genecards.org/cgi-bin/carddisp.pl?gene=HTR2B
GC16M058519	5.18	https://www.genecards.org/cgi-bin/carddisp.pl?gene=CNOT1
GC06M031639	5.18	https://www.genecards.org/cgi-bin/carddisp.pl?gene=BAG6
GC11M119417	5.18	https://www.genecards.org/cgi-bin/carddisp.pl?gene=THY1
GC17M043640	5.18	https://www.genecards.org/cgi-bin/carddisp.pl?gene=MEOX1
GC06P032153	5.18	https://www.genecards.org/cgi-bin/carddisp.pl?gene=PPT2
GC05M149492	5.17	https://www.genecards.org/cgi-bin/carddisp.pl?gene=CSNK1A1
GC19P049690	5.17	https://www.genecards.org/cgi-bin/carddisp.pl?gene=CPT1C
GC06P031400	5.17	https://www.genecards.org/cgi-bin/carddisp.pl?gene=HCP5
GC12P002795	5.16	https://www.genecards.org/cgi-bin/carddisp.pl?gene=FKBP4
GC15M089677	5.16	https://www.genecards.org/cgi-bin/carddisp.pl?gene=PEX11A
GC04M001985	5.16	https://www.genecards.org/cgi-bin/carddisp.pl?gene=NELFA
GC11M078068	5.16	https://www.genecards.org/cgi-bin/carddisp.pl?gene=NDUFC2
GC19P044849	5.16	https://www.genecards.org/cgi-bin/carddisp.pl?gene=NECTIN2
GC01M205626	5.16	https://www.genecards.org/cgi-bin/carddisp.pl?gene=SLC45A3
GC10P068956	5.16	https://www.genecards.org/cgi-bin/carddisp.pl?gene=DDX21
GC22P039570	5.16	https://www.genecards.org/cgi-bin/carddisp.pl?gene=CACNA1I
GC04P165873	5.15	https://www.genecards.org/cgi-bin/carddisp.pl?gene=TLL1
GC06P167827	5.15	https://www.genecards.org/cgi-bin/carddisp.pl?gene=AFDN
GC01M032864	5.15	https://www.genecards.org/cgi-bin/carddisp.pl?gene=FNDC5
GC18P074534	5.15	https://www.genecards.org/cgi-bin/carddisp.pl?gene=CNDP1
GC13M029509	5.14	https://www.genecards.org/cgi-bin/carddisp.pl?gene=SLC7A1
GC01M023303	5.14	https://www.genecards.org/cgi-bin/carddisp.pl?gene=HNRNPR
GC04P121801	5.14	https://www.genecards.org/cgi-bin/carddisp.pl?gene=EXOSC9
GC11M076186	5.13	https://www.genecards.org/cgi-bin/carddisp.pl?gene=WNT11
GC06P046053	5.13	https://www.genecards.org/cgi-bin/carddisp.pl?gene=SRSF3
GC14M092116	5.13	https://www.genecards.org/cgi-bin/carddisp.pl?gene=NDUFB1
GC20U900417	5.13	https://www.genecards.org/cgi-bin/carddisp.pl?gene=DYT17

GC01M037534	5.13	https://www.genecards.org/cgi-bin/carddisp.pl?gene=SNIP1
GC12P008099	5.12	https://www.genecards.org/cgi-bin/carddisp.pl?gene=NECAP1
GC11M029980	5.11	https://www.genecards.org/cgi-bin/carddisp.pl?gene=LINC01616
GC08P040153	5.11	https://www.genecards.org/cgi-bin/carddisp.pl?gene=TCIM
GC02M070626	5.11	https://www.genecards.org/cgi-bin/carddisp.pl?gene=ADD2
GC01P040757	5.11	https://www.genecards.org/cgi-bin/carddisp.pl?gene=MIR30C1
GC12P053097	5.11	https://www.genecards.org/cgi-bin/carddisp.pl?gene=IGFBP6
GC05U902798	5.11	https://www.genecards.org/cgi-bin/carddisp.pl?gene=LOC108660405
GC14P104255	5.11	https://www.genecards.org/cgi-bin/carddisp.pl?gene=MIR136
GC12M006965	5.1	https://www.genecards.org/cgi-bin/carddisp.pl?gene=PHB2
GC17M064002	5.1	https://www.genecards.org/cgi-bin/carddisp.pl?gene=ICAM2
GC11P063838	5.09	https://www.genecards.org/cgi-bin/carddisp.pl?gene=MARK2
GC16M075228	5.09	https://www.genecards.org/cgi-bin/carddisp.pl?gene=BCAR1
GC06M032814	5.09	https://www.genecards.org/cgi-bin/carddisp.pl?gene=HLA-DOB
GC02P241316	5.09	https://www.genecards.org/cgi-bin/carddisp.pl?gene=SEPTIN2
GC16M067927	5.09	https://www.genecards.org/cgi-bin/carddisp.pl?gene=CTRL
GC21U900882	5.08	https://www.genecards.org/cgi-bin/carddisp.pl?gene=LOC109029533
GC02M042767	5.08	https://www.genecards.org/cgi-bin/carddisp.pl?gene=HAAO
GC17M030897	5.08	https://www.genecards.org/cgi-bin/carddisp.pl?gene=TEFM
GC16P023871	5.07	https://www.genecards.org/cgi-bin/carddisp.pl?gene=CHP2
GC05P172834	5.07	https://www.genecards.org/cgi-bin/carddisp.pl?gene=ERGIC1
GC19P002328	5.07	https://www.genecards.org/cgi-bin/carddisp.pl?gene=SPPL2B
GC03M049223	5.07	https://www.genecards.org/cgi-bin/carddisp.pl?gene=QARS1
GC05P163480	5.07	https://www.genecards.org/cgi-bin/carddisp.pl?gene=HMMR
GC22P020129	5.06	https://www.genecards.org/cgi-bin/carddisp.pl?gene=ZDHHC8
GC06P132814	5.05	https://www.genecards.org/cgi-bin/carddisp.pl?gene=RPS12
GC02M069459	5.05	https://www.genecards.org/cgi-bin/carddisp.pl?gene=AAK1
GC17M067337	5.05	https://www.genecards.org/cgi-bin/carddisp.pl?gene=PSMD12
GC02P177212	5.05	https://www.genecards.org/cgi-bin/carddisp.pl?gene=HNRNPA3
GC01P151070	5.04	https://www.genecards.org/cgi-bin/carddisp.pl?gene=GABPB2
GC20P045206	5.04	https://www.genecards.org/cgi-bin/carddisp.pl?gene=SEMG1
GC15P041230	5.04	https://www.genecards.org/cgi-bin/carddisp.pl?gene=CHP1
GC01P171090	5.04	https://www.genecards.org/cgi-bin/carddisp.pl?gene=FMO3
GC12M048567	5.04	https://www.genecards.org/cgi-bin/carddisp.pl?gene=LALBA
GC0XM047635	5.03	https://www.genecards.org/cgi-bin/carddisp.pl?gene=ELK1
GC18M043267	5.03	https://www.genecards.org/cgi-bin/carddisp.pl?gene=SYT4
GC17P005282	5.02	https://www.genecards.org/cgi-bin/carddisp.pl?gene=RABEP1
GC01M067407	5.02	https://www.genecards.org/cgi-bin/carddisp.pl?gene=SERBP1
GC19P006381	5.02	https://www.genecards.org/cgi-bin/carddisp.pl?gene=CLPP
GC14M081471	5.02	https://www.genecards.org/cgi-bin/carddisp.pl?gene=SEL1L
GC01M054031	5.01	https://www.genecards.org/cgi-bin/carddisp.pl?gene=TMEM59
GC22M044553	5.01	https://www.genecards.org/cgi-bin/carddisp.pl?gene=RPL3
GC11M065773	5.01	https://www.genecards.org/cgi-bin/carddisp.pl?gene=AP5B1
GC11M065004	5.01	https://www.genecards.org/cgi-bin/carddisp.pl?gene=ATG2A
GC15P072824	5.01	https://www.genecards.org/cgi-bin/carddisp.pl?gene=RAB8B
GC17M007315	5.01	https://www.genecards.org/cgi-bin/carddisp.pl?gene=NEURL4
GC19M050819	5	https://www.genecards.org/cgi-bin/carddisp.pl?gene=KLK1

GC22P044668	5	https://www.genecards.org/cgi-bin/carddisp.pl?gene=PRR5
GC01M119747	5	https://www.genecards.org/cgi-bin/carddisp.pl?gene=HMGCS2
GC03M045917	4.99	https://www.genecards.org/cgi-bin/carddisp.pl?gene=FYCO1
GC14P104471	4.99	https://www.genecards.org/cgi-bin/carddisp.pl?gene=MIR494
GC15P034755	4.99	https://www.genecards.org/cgi-bin/carddisp.pl?gene=LOC101928174
GC20M002658	4.99	https://www.genecards.org/cgi-bin/carddisp.pl?gene=IDH3B
GC04M105369	4.99	https://www.genecards.org/cgi-bin/carddisp.pl?gene=PPA2
GC17M038530	4.99	https://www.genecards.org/cgi-bin/carddisp.pl?gene=SRCIN1
GC03P179604	4.99	https://www.genecards.org/cgi-bin/carddisp.pl?gene=NDUFB5
GC11P118572	4.99	https://www.genecards.org/cgi-bin/carddisp.pl?gene=ARCN1
GC0XM134246	4.98	https://www.genecards.org/cgi-bin/carddisp.pl?gene=MIR18B
GC08P042338	4.98	https://www.genecards.org/cgi-bin/carddisp.pl?gene=POLB
GC01P166809	4.98	https://www.genecards.org/cgi-bin/carddisp.pl?gene=POGK
GC19P022200	4.98	https://www.genecards.org/cgi-bin/carddisp.pl?gene=TPM4
GC12M091102	4.97	https://www.genecards.org/cgi-bin/carddisp.pl?gene=LUM
GC04M153780	4.97	https://www.genecards.org/cgi-bin/carddisp.pl?gene=SFRP2
GC02M061868	4.97	https://www.genecards.org/cgi-bin/carddisp.pl?gene=CCT4
GC15M066498	4.96	https://www.genecards.org/cgi-bin/carddisp.pl?gene=RPL4
GC12P006857	4.96	https://www.genecards.org/cgi-bin/carddisp.pl?gene=LAG3
GC02P215311	4.96	https://www.genecards.org/cgi-bin/carddisp.pl?gene=ATIC
GC04P110365	4.96	https://www.genecards.org/cgi-bin/carddisp.pl?gene=ENPEP
GC04P145481	4.95	https://www.genecards.org/cgi-bin/carddisp.pl?gene=SMAD1
GC12M057824	4.95	https://www.genecards.org/cgi-bin/carddisp.pl?gene=MIR26A2
GC17P042458	4.95	https://www.genecards.org/cgi-bin/carddisp.pl?gene=ATP6V0A1
GC19P034172	4.94	https://www.genecards.org/cgi-bin/carddisp.pl?gene=LSM14A
GC03M049906	4.94	https://www.genecards.org/cgi-bin/carddisp.pl?gene=TRAIP
GC16P030948	4.94	https://www.genecards.org/cgi-bin/carddisp.pl?gene=SETD1A
GC16M081081	4.94	https://www.genecards.org/cgi-bin/carddisp.pl?gene=GCSH
GC01M036795	4.94	https://www.genecards.org/cgi-bin/carddisp.pl?gene=GRIK3
GC06P043576	4.94	https://www.genecards.org/cgi-bin/carddisp.pl?gene=POLH
GC17P007937	4.94	https://www.genecards.org/cgi-bin/carddisp.pl?gene=SENP3
GC15P026971	4.94	https://www.genecards.org/cgi-bin/carddisp.pl?gene=GABRG3
GC17M041513	4.93	https://www.genecards.org/cgi-bin/carddisp.pl?gene=KRT15
GC06M154387	4.93	https://www.genecards.org/cgi-bin/carddisp.pl?gene=CNKSR3
GC15M102816	4.93	https://www.genecards.org/cgi-bin/carddisp.pl?gene=SELENOS
GC08P028494	4.92	https://www.genecards.org/cgi-bin/carddisp.pl?gene=FZD3
GC01P065792	4.92	https://www.genecards.org/cgi-bin/carddisp.pl?gene=PDE4B
GC12P068610	4.91	https://www.genecards.org/cgi-bin/carddisp.pl?gene=RAP1B
GC09M128255	4.9	https://www.genecards.org/cgi-bin/carddisp.pl?gene=GOLGA2
GC07M141927	4.9	https://www.genecards.org/cgi-bin/carddisp.pl?gene=CLEC5A
GC12P025196	4.9	https://www.genecards.org/cgi-bin/carddisp.pl?gene=ETFRF1
GC20P050190	4.9	https://www.genecards.org/cgi-bin/carddisp.pl?gene=CEBPB
GC16P030910	4.9	https://www.genecards.org/cgi-bin/carddisp.pl?gene=CTF1
GC19P049766	4.9	https://www.genecards.org/cgi-bin/carddisp.pl?gene=AP2A1
GC15P090388	4.9	https://www.genecards.org/cgi-bin/carddisp.pl?gene=IQGAP1
GC12M065050	4.89	https://www.genecards.org/cgi-bin/carddisp.pl?gene=WIF1
GC03P134598	4.89	https://www.genecards.org/cgi-bin/carddisp.pl?gene=EPHB1

GC19M049341	4.89	https://www.genecards.org/cgi-bin/carddisp.pl?gene=TEAD2
GC02P184598	4.89	https://www.genecards.org/cgi-bin/carddisp.pl?gene=ZNF804A
GC03P184241	4.88	https://www.genecards.org/cgi-bin/carddisp.pl?gene=MIR1224
GC11P060185	4.88	https://www.genecards.org/cgi-bin/carddisp.pl?gene=MS4A4A
GC11M063624	4.88	https://www.genecards.org/cgi-bin/carddisp.pl?gene=ATL3
GC04P150581	4.88	https://www.genecards.org/cgi-bin/carddisp.pl?gene=MAB21L2
GC05P172958	4.88	https://www.genecards.org/cgi-bin/carddisp.pl?gene=RPL26L1
GC0XP002828	4.88	https://www.genecards.org/cgi-bin/carddisp.pl?gene=GYG2
GC11P000875	4.88	https://www.genecards.org/cgi-bin/carddisp.pl?gene=RPLP2
GC16M004332	4.88	https://www.genecards.org/cgi-bin/carddisp.pl?gene=PAM16
GC01M038843	4.87	https://www.genecards.org/cgi-bin/carddisp.pl?gene=RRAGC
GC09P037412	4.86	https://www.genecards.org/cgi-bin/carddisp.pl?gene=GRHPR
GC04M020728	4.86	https://www.genecards.org/cgi-bin/carddisp.pl?gene=KCNIP4
GC03P120596	4.86	https://www.genecards.org/cgi-bin/carddisp.pl?gene=NDUFB4
GC19M008308	4.86	https://www.genecards.org/cgi-bin/carddisp.pl?gene=NDUFA7
GC12P057229	4.85	https://www.genecards.org/cgi-bin/carddisp.pl?gene=SHMT2
GC16P070613	4.85	https://www.genecards.org/cgi-bin/carddisp.pl?gene=IL34
GC16M004510	4.85	https://www.genecards.org/cgi-bin/carddisp.pl?gene=CDIP1
GC03P197791	4.85	https://www.genecards.org/cgi-bin/carddisp.pl?gene=LRCH3
GC01P002555	4.84	https://www.genecards.org/cgi-bin/carddisp.pl?gene=TNFRSF14
GC17P042659	4.84	https://www.genecards.org/cgi-bin/carddisp.pl?gene=TUBG2
GC08M144790	4.84	https://www.genecards.org/cgi-bin/carddisp.pl?gene=RPL8
GC03M131462	4.84	https://www.genecards.org/cgi-bin/carddisp.pl?gene=MRPL3
GC09P097501	4.83	https://www.genecards.org/cgi-bin/carddisp.pl?gene=TMOD1
GC08M100685	4.83	https://www.genecards.org/cgi-bin/carddisp.pl?gene=PABPC1
GC17M056836	4.83	https://www.genecards.org/cgi-bin/carddisp.pl?gene=TRIM25
GC09M035099	4.83	https://www.genecards.org/cgi-bin/carddisp.pl?gene=STOML2
GC08P020197	4.83	https://www.genecards.org/cgi-bin/carddisp.pl?gene=ATP6V1B2
GC08P098938	4.83	https://www.genecards.org/cgi-bin/carddisp.pl?gene=OSR2
GC11P062337	4.83	https://www.genecards.org/cgi-bin/carddisp.pl?gene=ASRGL1
GC04P133149	4.82	https://www.genecards.org/cgi-bin/carddisp.pl?gene=PCDH10
GC03M049015	4.82	https://www.genecards.org/cgi-bin/carddisp.pl?gene=DALRD3
GC13M077044	4.82	https://www.genecards.org/cgi-bin/carddisp.pl?gene=MYCBP2
GC0XM147910	4.82	https://www.genecards.org/cgi-bin/carddisp.pl?gene=FMR1-AS1
GC10P122374	4.82	https://www.genecards.org/cgi-bin/carddisp.pl?gene=PLEKHA1
GC10M090740	4.82	https://www.genecards.org/cgi-bin/carddisp.pl?gene=HTR7
GC01P210328	4.81	https://www.genecards.org/cgi-bin/carddisp.pl?gene=HHAT
GC01P155135	4.81	https://www.genecards.org/cgi-bin/carddisp.pl?gene=SLC50A1
GC15P032641	4.81	https://www.genecards.org/cgi-bin/carddisp.pl?gene=SCG5
GC10P102857	4.81	https://www.genecards.org/cgi-bin/carddisp.pl?gene=BORCS7
GC02M166403	4.81	https://www.genecards.org/cgi-bin/carddisp.pl?gene=SCN7A
GC10M099610	4.8	https://www.genecards.org/cgi-bin/carddisp.pl?gene=SLC25A28
GC06P044387	4.8	https://www.genecards.org/cgi-bin/carddisp.pl?gene=CDC5L
GC22P020810	4.79	https://www.genecards.org/cgi-bin/carddisp.pl?gene=SERPIND1
GC03P170222	4.79	https://www.genecards.org/cgi-bin/carddisp.pl?gene=PRKCI
GC05P175477	4.79	https://www.genecards.org/cgi-bin/carddisp.pl?gene=SFXN1
GC02P101773	4.79	https://www.genecards.org/cgi-bin/carddisp.pl?gene=MAP4K4

GC03M038008	4.79	https://www.genecards.org/cgi-bin/carddisp.pl?gene=PLCD1
GC06M089177	4.79	https://www.genecards.org/cgi-bin/carddisp.pl?gene=GABRR1
GC05M078485	4.79	https://www.genecards.org/cgi-bin/carddisp.pl?gene=LHFPL2
GC01P027830	4.78	https://www.genecards.org/cgi-bin/carddisp.pl?gene=PPP1R8
GC10M119571	4.78	https://www.genecards.org/cgi-bin/carddisp.pl?gene=TIAL1
GC20P062708	4.78	https://www.genecards.org/cgi-bin/carddisp.pl?gene=NTSR1
GC19M006663	4.78	https://www.genecards.org/cgi-bin/carddisp.pl?gene=TNFSF14
GC19M018933	4.78	https://www.genecards.org/cgi-bin/carddisp.pl?gene=HOMER3
GC19P003314	4.78	https://www.genecards.org/cgi-bin/carddisp.pl?gene=NFIC
GC07M100107	4.77	https://www.genecards.org/cgi-bin/carddisp.pl?gene=TAF6
GC12M124324	4.77	https://www.genecards.org/cgi-bin/carddisp.pl?gene=NCOR2
GC0XP154428	4.77	https://www.genecards.org/cgi-bin/carddisp.pl?gene=ATP6AP1
GC05M132191	4.77	https://www.genecards.org/cgi-bin/carddisp.pl?gene=P4HA2
GC04P083455	4.76	https://www.genecards.org/cgi-bin/carddisp.pl?gene=MRPS18C
GC02P070052	4.76	https://www.genecards.org/cgi-bin/carddisp.pl?gene=PCBP1
GC0XM134216	4.76	https://www.genecards.org/cgi-bin/carddisp.pl?gene=MIR19B2
GC02M025733	4.76	https://www.genecards.org/cgi-bin/carddisp.pl?gene=ASXL2
GC07M027224	4.76	https://www.genecards.org/cgi-bin/carddisp.pl?gene=MIR196B
GC06M116274	4.75	https://www.genecards.org/cgi-bin/carddisp.pl?gene=TSPYL1
GC13M048488	4.75	https://www.genecards.org/cgi-bin/carddisp.pl?gene=RCBTB2
GC11P064203	4.75	https://www.genecards.org/cgi-bin/carddisp.pl?gene=STIP1
GC06P033371	4.75	https://www.genecards.org/cgi-bin/carddisp.pl?gene=DDR1
GC02M206651	4.75	https://www.genecards.org/cgi-bin/carddisp.pl?gene=DYTN
GC12P008950	4.74	https://www.genecards.org/cgi-bin/carddisp.pl?gene=KLRG1
GC14P021144	4.73	https://www.genecards.org/cgi-bin/carddisp.pl?gene=RNASE2
GC07P031726	4.73	https://www.genecards.org/cgi-bin/carddisp.pl?gene=PPP1R17
GC0XP119574	4.73	https://www.genecards.org/cgi-bin/carddisp.pl?gene=UBE2A
GC0XU990057	4.73	https://www.genecards.org/cgi-bin/carddisp.pl?gene=FRAXA
GC21M029536	4.73	https://www.genecards.org/cgi-bin/carddisp.pl?gene=GRIK1
GC01P228208	4.73	https://www.genecards.org/cgi-bin/carddisp.pl?gene=OBSCN
GC01P234374	4.72	https://www.genecards.org/cgi-bin/carddisp.pl?gene=COA6
GC14P103715	4.72	https://www.genecards.org/cgi-bin/carddisp.pl?gene=ZFYVE21
GC09M136440	4.72	https://www.genecards.org/cgi-bin/carddisp.pl?gene=SEC16A
GC15P085381	4.71	https://www.genecards.org/cgi-bin/carddisp.pl?gene=AKAP13
GC03P023805	4.71	https://www.genecards.org/cgi-bin/carddisp.pl?gene=UBE2E1
GC03P142724	4.71	https://www.genecards.org/cgi-bin/carddisp.pl?gene=TRPC1
GC10M014771	4.7	https://www.genecards.org/cgi-bin/carddisp.pl?gene=CDNF
GC01M027546	4.7	https://www.genecards.org/cgi-bin/carddisp.pl?gene=AHDC1
GC06P033446	4.69	https://www.genecards.org/cgi-bin/carddisp.pl?gene=BRD2
GC02M147930	4.69	https://www.genecards.org/cgi-bin/carddisp.pl?gene=ORC4
GC11M064726	4.69	https://www.genecards.org/cgi-bin/carddisp.pl?gene=RASGRP2
GC10M099396	4.69	https://www.genecards.org/cgi-bin/carddisp.pl?gene=GOT1
GC10P010796	4.69	https://www.genecards.org/cgi-bin/carddisp.pl?gene=CELF2
GC0YP012963	4.68	https://www.genecards.org/cgi-bin/carddisp.pl?gene=DDX3Y
GC18M009073	4.68	https://www.genecards.org/cgi-bin/carddisp.pl?gene=NDUFV2-AS1
GC03P006770	4.68	https://www.genecards.org/cgi-bin/carddisp.pl?gene=GRM7
GC07P056051	4.68	https://www.genecards.org/cgi-bin/carddisp.pl?gene=CCT6A

GC04M099123	4.68	https://www.genecards.org/cgi-bin/carddisp.pl?gene=ADH4
GC14P052267	4.68	https://www.genecards.org/cgi-bin/carddisp.pl?gene=PTGDR
GC07P155459	4.68	https://www.genecards.org/cgi-bin/carddisp.pl?gene=EN2
GC03P132319	4.67	https://www.genecards.org/cgi-bin/carddisp.pl?gene=ACP3
GC21P036069	4.66	https://www.genecards.org/cgi-bin/carddisp.pl?gene=CBR1
GC07M151207	4.66	https://www.genecards.org/cgi-bin/carddisp.pl?gene=ABCF2
GC02P100562	4.66	https://www.genecards.org/cgi-bin/carddisp.pl?gene=PDCL3
GC09P075088	4.66	https://www.genecards.org/cgi-bin/carddisp.pl?gene=OSTF1
GC16P001224	4.66	https://www.genecards.org/cgi-bin/carddisp.pl?gene=PDIA2
GC19P049506	4.65	https://www.genecards.org/cgi-bin/carddisp.pl?gene=FCGRT
GC11P073217	4.65	https://www.genecards.org/cgi-bin/carddisp.pl?gene=P2RY2
GC01M085318	4.64	https://www.genecards.org/cgi-bin/carddisp.pl?gene=DDAH1
GC17M009250	4.64	https://www.genecards.org/cgi-bin/carddisp.pl?gene=STX8
GC22M038483	4.64	https://www.genecards.org/cgi-bin/carddisp.pl?gene=DDX17
GC07M100403	4.64	https://www.genecards.org/cgi-bin/carddisp.pl?gene=ZCWPW1
GC22M036561	4.64	https://www.genecards.org/cgi-bin/carddisp.pl?gene=CACNG2
GC09M020334	4.63	https://www.genecards.org/cgi-bin/carddisp.pl?gene=MLLT3
GC02M086144	4.63	https://www.genecards.org/cgi-bin/carddisp.pl?gene=IMMT
GC11M105041	4.63	https://www.genecards.org/cgi-bin/carddisp.pl?gene=CARD16
GC19P048993	4.63	https://www.genecards.org/cgi-bin/carddisp.pl?gene=RUVBL2
GC14P104251	4.62	https://www.genecards.org/cgi-bin/carddisp.pl?gene=MIR431
GC06P160348	4.62	https://www.genecards.org/cgi-bin/carddisp.pl?gene=SLC22A3
GC22M044564	4.62	https://www.genecards.org/cgi-bin/carddisp.pl?gene=ST13
GC04P055395	4.61	https://www.genecards.org/cgi-bin/carddisp.pl?gene=TMEM165
GC19P001269	4.61	https://www.genecards.org/cgi-bin/carddisp.pl?gene=CIRBP
GC05M180300	4.61	https://www.genecards.org/cgi-bin/carddisp.pl?gene=GFPT2
GC15M022867	4.6	https://www.genecards.org/cgi-bin/carddisp.pl?gene=CYFIP1
GC17P004711	4.6	https://www.genecards.org/cgi-bin/carddisp.pl?gene=ARRB2
GC05P161547	4.6	https://www.genecards.org/cgi-bin/carddisp.pl?gene=GABRA6
GC17P005116	4.6	https://www.genecards.org/cgi-bin/carddisp.pl?gene=USP6
GC22M017592	4.6	https://www.genecards.org/cgi-bin/carddisp.pl?gene=ATP6V1E1
GC05P121851	4.6	https://www.genecards.org/cgi-bin/carddisp.pl?gene=FTMT
GC01P032651	4.6	https://www.genecards.org/cgi-bin/carddisp.pl?gene=RBBP4
GC12M124946	4.6	https://www.genecards.org/cgi-bin/carddisp.pl?gene=DHX37
GC06M052791	4.6	https://www.genecards.org/cgi-bin/carddisp.pl?gene=GSTA1
GC17M032492	4.6	https://www.genecards.org/cgi-bin/carddisp.pl?gene=MYO1D
GC09P128275	4.59	https://www.genecards.org/cgi-bin/carddisp.pl?gene=SWI5
GC0XM015402	4.59	https://www.genecards.org/cgi-bin/carddisp.pl?gene=PIR
GC16M001943	4.59	https://www.genecards.org/cgi-bin/carddisp.pl?gene=RPL3L
GC0YP002841	4.59	https://www.genecards.org/cgi-bin/carddisp.pl?gene=RPS4Y1
GC10M026746	4.59	https://www.genecards.org/cgi-bin/carddisp.pl?gene=ABI1
GC12U900751	4.59	https://www.genecards.org/cgi-bin/carddisp.pl?gene=SPG36
GC09M108854	4.59	https://www.genecards.org/cgi-bin/carddisp.pl?gene=ACTL7B
GC17P019379	4.59	https://www.genecards.org/cgi-bin/carddisp.pl?gene=MAPK7
GC02P134119	4.59	https://www.genecards.org/cgi-bin/carddisp.pl?gene=MGAT5
GC12M106991	4.59	https://www.genecards.org/cgi-bin/carddisp.pl?gene=CRY1
GC11M119045	4.58	https://www.genecards.org/cgi-bin/carddisp.pl?gene=HYOU1

GC17M000212	4.58	https://www.genecards.org/cgi-bin/carddisp.pl?gene=RPH3AL
GC06M032288	4.58	https://www.genecards.org/cgi-bin/carddisp.pl?gene=TSBP1
GC16P074296	4.58	https://www.genecards.org/cgi-bin/carddisp.pl?gene=PSMD7
GC01M205448	4.58	https://www.genecards.org/cgi-bin/carddisp.pl?gene=MIR135B
GC12P027332	4.58	https://www.genecards.org/cgi-bin/carddisp.pl?gene=ARNTL2
GC17M039404	4.58	https://www.genecards.org/cgi-bin/carddisp.pl?gene=MED1
GC0XM151955	4.58	https://www.genecards.org/cgi-bin/carddisp.pl?gene=GABRE
GC12P069738	4.58	https://www.genecards.org/cgi-bin/carddisp.pl?gene=RAB3IP
GC0XP151176	4.58	https://www.genecards.org/cgi-bin/carddisp.pl?gene=GPR50
GC0XM112774	4.57	https://www.genecards.org/cgi-bin/carddisp.pl?gene=AMOT
GC15M082735	4.57	https://www.genecards.org/cgi-bin/carddisp.pl?gene=AP3B2
GC06P046104	4.57	https://www.genecards.org/cgi-bin/carddisp.pl?gene=BYSL
GC01P236518	4.56	https://www.genecards.org/cgi-bin/carddisp.pl?gene=LGALS8
GC01M071067	4.56	https://www.genecards.org/cgi-bin/carddisp.pl?gene=MIR186
GC06M041363	4.56	https://www.genecards.org/cgi-bin/carddisp.pl?gene=TREML2
GC20P034546	4.56	https://www.genecards.org/cgi-bin/carddisp.pl?gene=MAP1LC3A
GC15P040217	4.56	https://www.genecards.org/cgi-bin/carddisp.pl?gene=PAK6
GC03P052681	4.55	https://www.genecards.org/cgi-bin/carddisp.pl?gene=GNL3
GC17P042548	4.55	https://www.genecards.org/cgi-bin/carddisp.pl?gene=HSD17B1
GC02P132466	4.55	https://www.genecards.org/cgi-bin/carddisp.pl?gene=TUBA3D
GC07P074191	4.55	https://www.genecards.org/cgi-bin/carddisp.pl?gene=MIR590
GC04M006026	4.54	https://www.genecards.org/cgi-bin/carddisp.pl?gene=JAKMIP1
GC18P009136	4.54	https://www.genecards.org/cgi-bin/carddisp.pl?gene=ANKRD12
GC10M124984	4.53	https://www.genecards.org/cgi-bin/carddisp.pl?gene=CTBP2
GC05M151077	4.53	https://www.genecards.org/cgi-bin/carddisp.pl?gene=ANXA6
GC01P156591	4.53	https://www.genecards.org/cgi-bin/carddisp.pl?gene=NAXE
GC17M001464	4.53	https://www.genecards.org/cgi-bin/carddisp.pl?gene=MYO1C
GC13M024420	4.53	https://www.genecards.org/cgi-bin/carddisp.pl?gene=PARP4
GC01M093885	4.52	https://www.genecards.org/cgi-bin/carddisp.pl?gene=GCLM
GC19P005674	4.52	https://www.genecards.org/cgi-bin/carddisp.pl?gene=RPL36
GC14P058395	4.52	https://www.genecards.org/cgi-bin/carddisp.pl?gene=TOMM20L
GC09M119153	4.52	https://www.genecards.org/cgi-bin/carddisp.pl?gene=BRINP1
GC09M008307	4.52	https://www.genecards.org/cgi-bin/carddisp.pl?gene=PTPRD
GC0XM049113	4.51	https://www.genecards.org/cgi-bin/carddisp.pl?gene=GPKOW
GC20M035703	4.51	https://www.genecards.org/cgi-bin/carddisp.pl?gene=RBM39
GC11P008682	4.51	https://www.genecards.org/cgi-bin/carddisp.pl?gene=RPL27A
GC08M021690	4.51	https://www.genecards.org/cgi-bin/carddisp.pl?gene=GFRA2
GC01M109751	4.51	https://www.genecards.org/cgi-bin/carddisp.pl?gene=EPS8L3
GC0XP153793	4.51	https://www.genecards.org/cgi-bin/carddisp.pl?gene=SSR4
GC19M050977	4.51	https://www.genecards.org/cgi-bin/carddisp.pl?gene=KLK7
GC10P083265	4.5	https://www.genecards.org/cgi-bin/carddisp.pl?gene=LOC642361
GC11M111695	4.5	https://www.genecards.org/cgi-bin/carddisp.pl?gene=PPP2R1B
GC09M028880	4.5	https://www.genecards.org/cgi-bin/carddisp.pl?gene=MIR873
GC04M173331	4.5	https://www.genecards.org/cgi-bin/carddisp.pl?gene=HMGB2
GC09M089360	4.5	https://www.genecards.org/cgi-bin/carddisp.pl?gene=SEMA4D
GC06P143536	4.5	https://www.genecards.org/cgi-bin/carddisp.pl?gene=PHACTR2
GC14M073275	4.49	https://www.genecards.org/cgi-bin/carddisp.pl?gene=NUMB

GC18P074495	4.49	https://www.genecards.org/cgi-bin/carddisp.pl?gene=CNDP2
GC08M009136	4.49	https://www.genecards.org/cgi-bin/carddisp.pl?gene=PPP1R3B
GC07M159028	4.49	https://www.genecards.org/cgi-bin/carddisp.pl?gene=VIPR2
GC14P057268	4.49	https://www.genecards.org/cgi-bin/carddisp.pl?gene=AP5M1
GC01P156054	4.49	https://www.genecards.org/cgi-bin/carddisp.pl?gene=LAMTOR2
GC11M119308	4.48	https://www.genecards.org/cgi-bin/carddisp.pl?gene=MCAM
GC12M043353	4.48	https://www.genecards.org/cgi-bin/carddisp.pl?gene=ADAMTS20
GC20P013221	4.48	https://www.genecards.org/cgi-bin/carddisp.pl?gene=ISM1
GC03M189956	4.47	https://www.genecards.org/cgi-bin/carddisp.pl?gene=P3H2
GC12M054396	4.47	https://www.genecards.org/cgi-bin/carddisp.pl?gene=ITGA5
GC02P086106	4.47	https://www.genecards.org/cgi-bin/carddisp.pl?gene=PTCD3
GC0XP152830	4.47	https://www.genecards.org/cgi-bin/carddisp.pl?gene=NSDHL
GC07M073768	4.47	https://www.genecards.org/cgi-bin/carddisp.pl?gene=CLDN3
GC12M108589	4.47	https://www.genecards.org/cgi-bin/carddisp.pl?gene=TMEM119
GC19M012922	4.46	https://www.genecards.org/cgi-bin/carddisp.pl?gene=FARSA
GC08P026296	4.46	https://www.genecards.org/cgi-bin/carddisp.pl?gene=BNIP3L
GC16P004058	4.46	https://www.genecards.org/cgi-bin/carddisp.pl?gene=IL32
GC14M026443	4.46	https://www.genecards.org/cgi-bin/carddisp.pl?gene=NOVA1
GC19M048630	4.45	https://www.genecards.org/cgi-bin/carddisp.pl?gene=DBP
GC18U901253	4.45	https://www.genecards.org/cgi-bin/carddisp.pl?gene=LOC109609705
GC02P199956	4.45	https://www.genecards.org/cgi-bin/carddisp.pl?gene=MAIP1
GC05M072219	4.45	https://www.genecards.org/cgi-bin/carddisp.pl?gene=MRPS27
GC08M144051	4.45	https://www.genecards.org/cgi-bin/carddisp.pl?gene=OPLAH
GC02P202034	4.44	https://www.genecards.org/cgi-bin/carddisp.pl?gene=FZD7
GC08M014089	4.44	https://www.genecards.org/cgi-bin/carddisp.pl?gene=SGCZ
GC07M100643	4.44	https://www.genecards.org/cgi-bin/carddisp.pl?gene=ACTL6B
GC01P101236	4.43	https://www.genecards.org/cgi-bin/carddisp.pl?gene=S1PR1
GC07P130486	4.43	https://www.genecards.org/cgi-bin/carddisp.pl?gene=MEST
GC19P037745	4.43	https://www.genecards.org/cgi-bin/carddisp.pl?gene=CAPNS1
GC01M118883	4.43	https://www.genecards.org/cgi-bin/carddisp.pl?gene=TBX15
GC04M161383	4.42	https://www.genecards.org/cgi-bin/carddisp.pl?gene=FSTL5
GC16P004425	4.42	https://www.genecards.org/cgi-bin/carddisp.pl?gene=DNAJA3
GC11P120480	4.42	https://www.genecards.org/cgi-bin/carddisp.pl?gene=GRIK4
GC05P007396	4.42	https://www.genecards.org/cgi-bin/carddisp.pl?gene=ADCY2
GC07P073684	4.42	https://www.genecards.org/cgi-bin/carddisp.pl?gene=BUD23
GC06P122779	4.42	https://www.genecards.org/cgi-bin/carddisp.pl?gene=FABP7
GC14P055559	4.42	https://www.genecards.org/cgi-bin/carddisp.pl?gene=KTN1
GC01M246540	4.42	https://www.genecards.org/cgi-bin/carddisp.pl?gene=TFB2M
GC09M125437	4.42	https://www.genecards.org/cgi-bin/carddisp.pl?gene=MAPKAP1
GC06P127118	4.42	https://www.genecards.org/cgi-bin/carddisp.pl?gene=RSPO3
GC17M001420	4.42	https://www.genecards.org/cgi-bin/carddisp.pl?gene=CRK
GC01M018871	4.42	https://www.genecards.org/cgi-bin/carddisp.pl?gene=ALDH4A1
GC14M103556	4.42	https://www.genecards.org/cgi-bin/carddisp.pl?gene=BAG5
GC01M208023	4.42	https://www.genecards.org/cgi-bin/carddisp.pl?gene=PLXNA2
GC10M119034	4.42	https://www.genecards.org/cgi-bin/carddisp.pl?gene=EIF3A
GC15P053978	4.41	https://www.genecards.org/cgi-bin/carddisp.pl?gene=UNC13C
GC09P132582	4.41	https://www.genecards.org/cgi-bin/carddisp.pl?gene=BARHL1

GC08M029067	4.41	https://www.genecards.org/cgi-bin/carddisp.pl?gene=KIF13B
GC13P057630	4.41	https://www.genecards.org/cgi-bin/carddisp.pl?gene=PCDH17
GC01P158801	4.41	https://www.genecards.org/cgi-bin/carddisp.pl?gene=MNDA
GC07P139344	4.41	https://www.genecards.org/cgi-bin/carddisp.pl?gene=LUC7L2
GC17M043527	4.4	https://www.genecards.org/cgi-bin/carddisp.pl?gene=ETV4
GC11M075261	4.4	https://www.genecards.org/cgi-bin/carddisp.pl?gene=ARRB1
GC01P212565	4.4	https://www.genecards.org/cgi-bin/carddisp.pl?gene=ATF3
GC01P226550	4.4	https://www.genecards.org/cgi-bin/carddisp.pl?gene=STUM
GC09M027316	4.4	https://www.genecards.org/cgi-bin/carddisp.pl?gene=MOB3B
GC02M240479	4.39	https://www.genecards.org/cgi-bin/carddisp.pl?gene=ANKMY1
GC10M112148	4.39	https://www.genecards.org/cgi-bin/carddisp.pl?gene=GPAM
GC22P019451	4.39	https://www.genecards.org/cgi-bin/carddisp.pl?gene=DGCR5
GC07P121873	4.39	https://www.genecards.org/cgi-bin/carddisp.pl?gene=PTPRZ1
GC10P087504	4.39	https://www.genecards.org/cgi-bin/carddisp.pl?gene=MINPP1
GC09M083993	4.38	https://www.genecards.org/cgi-bin/carddisp.pl?gene=MIR7-1
GC19M050825	4.38	https://www.genecards.org/cgi-bin/carddisp.pl?gene=KLK15
GC19P046346	4.38	https://www.genecards.org/cgi-bin/carddisp.pl?gene=PPP5C
GC01M018839	4.37	https://www.genecards.org/cgi-bin/carddisp.pl?gene=TAS1R2
GC10P045728	4.37	https://www.genecards.org/cgi-bin/carddisp.pl?gene=WASHC2C
GC17P048997	4.37	https://www.genecards.org/cgi-bin/carddisp.pl?gene=IGF2BP1
GC09P098807	4.37	https://www.genecards.org/cgi-bin/carddisp.pl?gene=GALNT12
GC15P041557	4.37	https://www.genecards.org/cgi-bin/carddisp.pl?gene=TYRO3
GC01M201373	4.36	https://www.genecards.org/cgi-bin/carddisp.pl?gene=LAD1
GC06M041358	4.36	https://www.genecards.org/cgi-bin/carddisp.pl?gene=TREML1
GC01M155213	4.36	https://www.genecards.org/cgi-bin/carddisp.pl?gene=GBAP1
GC19M041998	4.36	https://www.genecards.org/cgi-bin/carddisp.pl?gene=GRIK5
GC0XP118727	4.36	https://www.genecards.org/cgi-bin/carddisp.pl?gene=IL13RA1
GC01M151955	4.35	https://www.genecards.org/cgi-bin/carddisp.pl?gene=S100A10
GC11P032851	4.35	https://www.genecards.org/cgi-bin/carddisp.pl?gene=PRRG4
GC08P042697	4.35	https://www.genecards.org/cgi-bin/carddisp.pl?gene=CHRNB3
GC14M070044	4.35	https://www.genecards.org/cgi-bin/carddisp.pl?gene=SLC8A3
GC17P028719	4.35	https://www.genecards.org/cgi-bin/carddisp.pl?gene=RPL23A
GC02P015591	4.35	https://www.genecards.org/cgi-bin/carddisp.pl?gene=DDX1
GC20M023079	4.35	https://www.genecards.org/cgi-bin/carddisp.pl?gene=CD93
GC16P069132	4.34	https://www.genecards.org/cgi-bin/carddisp.pl?gene=UTP4
GC07M050590	4.34	https://www.genecards.org/cgi-bin/carddisp.pl?gene=GRB10
GC08M140522	4.34	https://www.genecards.org/cgi-bin/carddisp.pl?gene=AGO2
GC19P049487	4.34	https://www.genecards.org/cgi-bin/carddisp.pl?gene=RPL13A
GC16M031439	4.34	https://www.genecards.org/cgi-bin/carddisp.pl?gene=COX6A2
GC03M161083	4.34	https://www.genecards.org/cgi-bin/carddisp.pl?gene=B3GALNT1
GC02P183078	4.34	https://www.genecards.org/cgi-bin/carddisp.pl?gene=DUSP19
GC04P073837	4.34	https://www.genecards.org/cgi-bin/carddisp.pl?gene=CXCL6
GC04P007461	4.34	https://www.genecards.org/cgi-bin/carddisp.pl?gene=MIR4274
GC11P043902	4.34	https://www.genecards.org/cgi-bin/carddisp.pl?gene=ALKBH3
GC07M122318	4.33	https://www.genecards.org/cgi-bin/carddisp.pl?gene=CADPS2
GC01P232950	4.33	https://www.genecards.org/cgi-bin/carddisp.pl?gene=NTPCR
GC19M049429	4.33	https://www.genecards.org/cgi-bin/carddisp.pl?gene=SLC17A7

GC02P197501	4.33	https://www.genecards.org/cgi-bin/carddisp.pl?gene=HSPE1
GC21P041304	4.33	https://www.genecards.org/cgi-bin/carddisp.pl?gene=FAM3B
GC02P095025	4.33	https://www.genecards.org/cgi-bin/carddisp.pl?gene=MAL
GC17M040092	4.33	https://www.genecards.org/cgi-bin/carddisp.pl?gene=NR1D1
GC14P025017	4.32	https://www.genecards.org/cgi-bin/carddisp.pl?gene=DHRS2
GC09M127866	4.32	https://www.genecards.org/cgi-bin/carddisp.pl?gene=AK1
GC02P235494	4.32	https://www.genecards.org/cgi-bin/carddisp.pl?gene=AGAP1
GC05P151771	4.32	https://www.genecards.org/cgi-bin/carddisp.pl?gene=G3BP1
GC11M090200	4.31	https://www.genecards.org/cgi-bin/carddisp.pl?gene=CHORDC1
GC19P010625	4.31	https://www.genecards.org/cgi-bin/carddisp.pl?gene=ILF3
GC09P108862	4.31	https://www.genecards.org/cgi-bin/carddisp.pl?gene=ACTL7A
GC0XM138615	4.31	https://www.genecards.org/cgi-bin/carddisp.pl?gene=FGF13
GC03P179562	4.31	https://www.genecards.org/cgi-bin/carddisp.pl?gene=ACTL6A
GC04M056963	4.31	https://www.genecards.org/cgi-bin/carddisp.pl?gene=NOA1
GC19P000825	4.31	https://www.genecards.org/cgi-bin/carddisp.pl?gene=AZU1
GC15M081331	4.3	https://www.genecards.org/cgi-bin/carddisp.pl?gene=TMC3
GC07M016818	4.3	https://www.genecards.org/cgi-bin/carddisp.pl?gene=AGR2
GC10M118004	4.3	https://www.genecards.org/cgi-bin/carddisp.pl?gene=RAB11FIP2
GC01M021596	4.3	https://www.genecards.org/cgi-bin/carddisp.pl?gene=RAP1GAP
GC20P037178	4.3	https://www.genecards.org/cgi-bin/carddisp.pl?gene=RPN2
GC01M151802	4.3	https://www.genecards.org/cgi-bin/carddisp.pl?gene=LINGO4
GC03M009965	4.29	https://www.genecards.org/cgi-bin/carddisp.pl?gene=EMC3
GC05M168661	4.29	https://www.genecards.org/cgi-bin/carddisp.pl?gene=SLIT3
GC06P046652	4.29	https://www.genecards.org/cgi-bin/carddisp.pl?gene=SLC25A27
GC03P009890	4.29	https://www.genecards.org/cgi-bin/carddisp.pl?gene=JAGN1
GC06P116681	4.29	https://www.genecards.org/cgi-bin/carddisp.pl?gene=KPNA5
GC14M067447	4.28	https://www.genecards.org/cgi-bin/carddisp.pl?gene=TMEM229B
GC01P167190	4.28	https://www.genecards.org/cgi-bin/carddisp.pl?gene=POU2F1
GC17P038297	4.28	https://www.genecards.org/cgi-bin/carddisp.pl?gene=MRPL45
GC17M041866	4.28	https://www.genecards.org/cgi-bin/carddisp.pl?gene=ACLY
GC16P002374	4.28	https://www.genecards.org/cgi-bin/carddisp.pl?gene=TBL3
GC17P068035	4.28	https://www.genecards.org/cgi-bin/carddisp.pl?gene=KPNA2
GC17M078175	4.28	https://www.genecards.org/cgi-bin/carddisp.pl?gene=TK1
GC06M026018	4.28	https://www.genecards.org/cgi-bin/carddisp.pl?gene=H1-1
GC05M041732	4.28	https://www.genecards.org/cgi-bin/carddisp.pl?gene=OXCT1
GC19P032876	4.28	https://www.genecards.org/cgi-bin/carddisp.pl?gene=NUDT19
GC01P028270	4.28	https://www.genecards.org/cgi-bin/carddisp.pl?gene=SESN2
GC03P049554	4.27	https://www.genecards.org/cgi-bin/carddisp.pl?gene=BSN
GC11P032892	4.27	https://www.genecards.org/cgi-bin/carddisp.pl?gene=QSER1
GC05P173888	4.27	https://www.genecards.org/cgi-bin/carddisp.pl?gene=CPEB4
GC07P076398	4.27	https://www.genecards.org/cgi-bin/carddisp.pl?gene=ZP3
GC16P070523	4.26	https://www.genecards.org/cgi-bin/carddisp.pl?gene=SF3B3
GC13M075284	4.26	https://www.genecards.org/cgi-bin/carddisp.pl?gene=TBC1D4
GC21P042879	4.25	https://www.genecards.org/cgi-bin/carddisp.pl?gene=NDUFV3
GC01M023191	4.25	https://www.genecards.org/cgi-bin/carddisp.pl?gene=HTR1D
GC20P036064	4.25	https://www.genecards.org/cgi-bin/carddisp.pl?gene=EPB41L1
GC02P073233	4.25	https://www.genecards.org/cgi-bin/carddisp.pl?gene=CCT7

GC13M039009	4.25	https://www.genecards.org/cgi-bin/carddisp.pl?gene=PROSER1
GC17M057834	4.25	https://www.genecards.org/cgi-bin/carddisp.pl?gene=MRPS23
GC09M015170	4.24	https://www.genecards.org/cgi-bin/carddisp.pl?gene=TTC39B
GC12M014784	4.24	https://www.genecards.org/cgi-bin/carddisp.pl?gene=WBP11
GC21M041798	4.24	https://www.genecards.org/cgi-bin/carddisp.pl?gene=PRDM15
GC10M021762	4.23	https://www.genecards.org/cgi-bin/carddisp.pl?gene=DNAJC1
GC0XP151912	4.23	https://www.genecards.org/cgi-bin/carddisp.pl?gene=MAGEA4
GC19P045467	4.23	https://www.genecards.org/cgi-bin/carddisp.pl?gene=FOSB
GC0XM007898	4.22	https://www.genecards.org/cgi-bin/carddisp.pl?gene=PNPLA4
GC15M074631	4.22	https://www.genecards.org/cgi-bin/carddisp.pl?gene=EDC3
GC08M123498	4.22	https://www.genecards.org/cgi-bin/carddisp.pl?gene=FBXO32
GC01M077088	4.22	https://www.genecards.org/cgi-bin/carddisp.pl?gene=PIGK
GC12P049188	4.22	https://www.genecards.org/cgi-bin/carddisp.pl?gene=TUBA1C
GC12P122644	4.22	https://www.genecards.org/cgi-bin/carddisp.pl?gene=PSMD9
GC22M036510	4.22	https://www.genecards.org/cgi-bin/carddisp.pl?gene=EIF3D
GC02P105020	4.21	https://www.genecards.org/cgi-bin/carddisp.pl?gene=MRPS9
GC03M018364	4.21	https://www.genecards.org/cgi-bin/carddisp.pl?gene=SATB1
GC02P191246	4.21	https://www.genecards.org/cgi-bin/carddisp.pl?gene=MYO1B
GC06M028903	4.21	https://www.genecards.org/cgi-bin/carddisp.pl?gene=TRIM27
GC01M241628	4.21	https://www.genecards.org/cgi-bin/carddisp.pl?gene=CHML
GC02P028392	4.21	https://www.genecards.org/cgi-bin/carddisp.pl?gene=FOSL2
GC01P167599	4.2	https://www.genecards.org/cgi-bin/carddisp.pl?gene=RCSD1
GC12M120128	4.2	https://www.genecards.org/cgi-bin/carddisp.pl?gene=GCN1
GC09M116425	4.2	https://www.genecards.org/cgi-bin/carddisp.pl?gene=ASTN2
GC20M042072	4.2	https://www.genecards.org/cgi-bin/carddisp.pl?gene=PTPRT
GC02P054456	4.2	https://www.genecards.org/cgi-bin/carddisp.pl?gene=SPTBN1
GC21P044134	4.19	https://www.genecards.org/cgi-bin/carddisp.pl?gene=GATD3A
GC22P038056	4.19	https://www.genecards.org/cgi-bin/carddisp.pl?gene=PICK1
GC06M052264	4.19	https://www.genecards.org/cgi-bin/carddisp.pl?gene=MCM3
GC11M113687	4.19	https://www.genecards.org/cgi-bin/carddisp.pl?gene=TMPRSS5
GC05P142108	4.19	https://www.genecards.org/cgi-bin/carddisp.pl?gene=NDFIP1
GC05P176447	4.19	https://www.genecards.org/cgi-bin/carddisp.pl?gene=FAF2
GC22P018906	4.19	https://www.genecards.org/cgi-bin/carddisp.pl?gene=DGCR6
GC17M064498	4.18	https://www.genecards.org/cgi-bin/carddisp.pl?gene=DDX5
GC16M031085	4.18	https://www.genecards.org/cgi-bin/carddisp.pl?gene=PRSS53
GC05P175797	4.18	https://www.genecards.org/cgi-bin/carddisp.pl?gene=CPLX2
GC07P140690	4.18	https://www.genecards.org/cgi-bin/carddisp.pl?gene=NDUFB2
GC17M004538	4.18	https://www.genecards.org/cgi-bin/carddisp.pl?gene=MYBBP1A
GC08P122781	4.18	https://www.genecards.org/cgi-bin/carddisp.pl?gene=ZHX2
GC09M137111	4.18	https://www.genecards.org/cgi-bin/carddisp.pl?gene=DPP7
GC09M114086	4.18	https://www.genecards.org/cgi-bin/carddisp.pl?gene=KIF12
GC11M073676	4.18	https://www.genecards.org/cgi-bin/carddisp.pl?gene=RAB6A
GC21M043609	4.17	https://www.genecards.org/cgi-bin/carddisp.pl?gene=MIR6070
GC03M186046	4.17	https://www.genecards.org/cgi-bin/carddisp.pl?gene=ETV5
GC11M067631	4.17	https://www.genecards.org/cgi-bin/carddisp.pl?gene=TBX10
GC17M042113	4.17	https://www.genecards.org/cgi-bin/carddisp.pl?gene=KAT2A
GC01M224227	4.17	https://www.genecards.org/cgi-bin/carddisp.pl?gene=NVL

GC10P102132	4.17	https://www.genecards.org/cgi-bin/carddisp.pl?gene=PPRC1
GC01M046041	4.17	https://www.genecards.org/cgi-bin/carddisp.pl?gene=PIK3R3
GC13P098142	4.17	https://www.genecards.org/cgi-bin/carddisp.pl?gene=FARP1
GC16M008892	4.16	https://www.genecards.org/cgi-bin/carddisp.pl?gene=USP7
GC05P159867	4.15	https://www.genecards.org/cgi-bin/carddisp.pl?gene=ADRA1B
GC05P069242	4.15	https://www.genecards.org/cgi-bin/carddisp.pl?gene=CDK7
GC01M020499	4.15	https://www.genecards.org/cgi-bin/carddisp.pl?gene=MUL1
GC03P196739	4.15	https://www.genecards.org/cgi-bin/carddisp.pl?gene=PAK2
GC09P038392	4.15	https://www.genecards.org/cgi-bin/carddisp.pl?gene=ALDH1B1
GC08P085373	4.15	https://www.genecards.org/cgi-bin/carddisp.pl?gene=CA3
GC16M004791	4.14	https://www.genecards.org/cgi-bin/carddisp.pl?gene=SEPTIN12
GC01P112673	4.14	https://www.genecards.org/cgi-bin/carddisp.pl?gene=MOV10
GC02P233586	4.14	https://www.genecards.org/cgi-bin/carddisp.pl?gene=UGT1A
GC22P029767	4.14	https://www.genecards.org/cgi-bin/carddisp.pl?gene=UQCR10
GC07P103297	4.13	https://www.genecards.org/cgi-bin/carddisp.pl?gene=PMPCB
GC02P237627	4.13	https://www.genecards.org/cgi-bin/carddisp.pl?gene=LRRFIP1
GC14P023469	4.13	https://www.genecards.org/cgi-bin/carddisp.pl?gene=NGDN
GC19M000617	4.13	https://www.genecards.org/cgi-bin/carddisp.pl?gene=POLRMT
GC04M004237	4.13	https://www.genecards.org/cgi-bin/carddisp.pl?gene=TMEM128
GC12M054230	4.12	https://www.genecards.org/cgi-bin/carddisp.pl?gene=CBX5
GC07M132123	4.12	https://www.genecards.org/cgi-bin/carddisp.pl?gene=PLXNA4
GC0XP152638	4.12	https://www.genecards.org/cgi-bin/carddisp.pl?gene=GABRQ
GC02M182909	4.12	https://www.genecards.org/cgi-bin/carddisp.pl?gene=NCKAP1
GC15P083447	4.12	https://www.genecards.org/cgi-bin/carddisp.pl?gene=SH3GL3
GC09P101533	4.12	https://www.genecards.org/cgi-bin/carddisp.pl?gene=RNF20
GC06P033230	4.11	https://www.genecards.org/cgi-bin/carddisp.pl?gene=TRIM39
GC19P004770	4.11	https://www.genecards.org/cgi-bin/carddisp.pl?gene=MIR7-3
GC01M039560	4.11	https://www.genecards.org/cgi-bin/carddisp.pl?gene=PABPC4
GC07P091264	4.11	https://www.genecards.org/cgi-bin/carddisp.pl?gene=FZD1
GC12M089588	4.11	https://www.genecards.org/cgi-bin/carddisp.pl?gene=ATP2B1
GC02M086505	4.1	https://www.genecards.org/cgi-bin/carddisp.pl?gene=CHMP3
GC07M030651	4.1	https://www.genecards.org/cgi-bin/carddisp.pl?gene=CRHR2
GC01P201829	4.1	https://www.genecards.org/cgi-bin/carddisp.pl?gene=IPO9
GC20P031605	4.1	https://www.genecards.org/cgi-bin/carddisp.pl?gene=ID1
GC10P123008	4.1	https://www.genecards.org/cgi-bin/carddisp.pl?gene=ACADSB
GC02P233636	4.1	https://www.genecards.org/cgi-bin/carddisp.pl?gene=UGT1A10
GC09M127732	4.09	https://www.genecards.org/cgi-bin/carddisp.pl?gene=TOR2A
GC12M057472	4.09	https://www.genecards.org/cgi-bin/carddisp.pl?gene=ARHGAP9
GC22M038818	4.09	https://www.genecards.org/cgi-bin/carddisp.pl?gene=NPTXR
GC02M015918	4.08	https://www.genecards.org/cgi-bin/carddisp.pl?gene=MYCNOS
GC11P014643	4.08	https://www.genecards.org/cgi-bin/carddisp.pl?gene=PDE3B
GC13P031739	4.08	https://www.genecards.org/cgi-bin/carddisp.pl?gene=RXFP2
GC19P022242	4.08	https://www.genecards.org/cgi-bin/carddisp.pl?gene=KCNN1
GC12P055973	4.08	https://www.genecards.org/cgi-bin/carddisp.pl?gene=RAB5B
GC05M113022	4.08	https://www.genecards.org/cgi-bin/carddisp.pl?gene=MCC
GC06P043076	4.08	https://www.genecards.org/cgi-bin/carddisp.pl?gene=PTK7
GC01M011637	4.08	https://www.genecards.org/cgi-bin/carddisp.pl?gene=FBXO2

GC17P039962	4.08	https://www.genecards.org/cgi-bin/carddisp.pl?gene=GSDMA
GC16M023464	4.08	https://www.genecards.org/cgi-bin/carddisp.pl?gene=GGA2
GC01M228427	4.07	https://www.genecards.org/cgi-bin/carddisp.pl?gene=H3-4
GC11P064305	4.07	https://www.genecards.org/cgi-bin/carddisp.pl?gene=ESRRA
GC08P100150	4.06	https://www.genecards.org/cgi-bin/carddisp.pl?gene=POLR2K
GC22M020999	4.06	https://www.genecards.org/cgi-bin/carddisp.pl?gene=THAP7
GC01M155141	4.06	https://www.genecards.org/cgi-bin/carddisp.pl?gene=KRTCAP2
GC20M004220	4.06	https://www.genecards.org/cgi-bin/carddisp.pl?gene=ADRA1D
GC0XM119616	4.06	https://www.genecards.org/cgi-bin/carddisp.pl?gene=SEPTIN6
GC06M041354	4.05	https://www.genecards.org/cgi-bin/carddisp.pl?gene=OARD1
GC0XM108155	4.05	https://www.genecards.org/cgi-bin/carddisp.pl?gene=COL4A6
GC04M128269	4.05	https://www.genecards.org/cgi-bin/carddisp.pl?gene=PGRMC2
GC01M010456	4.05	https://www.genecards.org/cgi-bin/carddisp.pl?gene=DFFA
GC12M070638	4.05	https://www.genecards.org/cgi-bin/carddisp.pl?gene=PTPRR
GC05M107376	4.05	https://www.genecards.org/cgi-bin/carddisp.pl?gene=EFNA5
GC13P025095	4.05	https://www.genecards.org/cgi-bin/carddisp.pl?gene=PABPC3
GC01P224896	4.04	https://www.genecards.org/cgi-bin/carddisp.pl?gene=DNAH14
GC05P076083	4.04	https://www.genecards.org/cgi-bin/carddisp.pl?gene=SV2C
GC13M037103	4.04	https://www.genecards.org/cgi-bin/carddisp.pl?gene=CSNK1A1L
GC09P130172	4.04	https://www.genecards.org/cgi-bin/carddisp.pl?gene=NCS1
GC01M032334	4.04	https://www.genecards.org/cgi-bin/carddisp.pl?gene=MARCKSL1
GC16P002382	4.04	https://www.genecards.org/cgi-bin/carddisp.pl?gene=NPW
GC20P044966	4.04	https://www.genecards.org/cgi-bin/carddisp.pl?gene=STK4
GC08M134477	4.04	https://www.genecards.org/cgi-bin/carddisp.pl?gene=ZFAT
GC03M143265	4.04	https://www.genecards.org/cgi-bin/carddisp.pl?gene=SLC9A9
GC03M048641	4.04	https://www.genecards.org/cgi-bin/carddisp.pl?gene=CELSR3
GC17M078787	4.04	https://www.genecards.org/cgi-bin/carddisp.pl?gene=USP36
GC17M001646	4.03	https://www.genecards.org/cgi-bin/carddisp.pl?gene=RILP
GC11M067415	4.03	https://www.genecards.org/cgi-bin/carddisp.pl?gene=PPP1CA
GC17P002303	4.03	https://www.genecards.org/cgi-bin/carddisp.pl?gene=SRR
GC11M061897	4.03	https://www.genecards.org/cgi-bin/carddisp.pl?gene=RAB3IL1
GC07M017798	4.03	https://www.genecards.org/cgi-bin/carddisp.pl?gene=SNX13
GC02P236537	4.03	https://www.genecards.org/cgi-bin/carddisp.pl?gene=ACKR3
GC01M161364	4.03	https://www.genecards.org/cgi-bin/carddisp.pl?gene=CFAP126
GC11M096352	4.03	https://www.genecards.org/cgi-bin/carddisp.pl?gene=CCDC82
GC12P065279	4.02	https://www.genecards.org/cgi-bin/carddisp.pl?gene=MSRB3
GC16P004051	4.02	https://www.genecards.org/cgi-bin/carddisp.pl?gene=SRRM2
GC06P028387	4.02	https://www.genecards.org/cgi-bin/carddisp.pl?gene=H3C6
GC10P112374	4.02	https://www.genecards.org/cgi-bin/carddisp.pl?gene=ACSL5
GC12P053103	4.02	https://www.genecards.org/cgi-bin/carddisp.pl?gene=SOAT2
GC01M155195	4.02	https://www.genecards.org/cgi-bin/carddisp.pl?gene=THBS3
GC20P032277	4.02	https://www.genecards.org/cgi-bin/carddisp.pl?gene=KIF3B
GC05P160422	4.01	https://www.genecards.org/cgi-bin/carddisp.pl?gene=PTTG1
GC06P057145	4.01	https://www.genecards.org/cgi-bin/carddisp.pl?gene=BAG2
GC17P063550	4.01	https://www.genecards.org/cgi-bin/carddisp.pl?gene=DCAF7
GC06P032005	4.01	https://www.genecards.org/cgi-bin/carddisp.pl?gene=CYP21A1P
GC13M035768	4.01	https://www.genecards.org/cgi-bin/carddisp.pl?gene=DCLK1

GC01M156749	4.01	https://www.genecards.org/cgi-bin/carddisp.pl?gene=HDGF
GC08M042752	4	https://www.genecards.org/cgi-bin/carddisp.pl?gene=CHRNA6
GC03M049204	4	https://www.genecards.org/cgi-bin/carddisp.pl?gene=IMPDH2
GC08M067043	4	https://www.genecards.org/cgi-bin/carddisp.pl?gene=COPS5
GC08P069466	4	https://www.genecards.org/cgi-bin/carddisp.pl?gene=SULF1
GC17P081976	3.99	https://www.genecards.org/cgi-bin/carddisp.pl?gene=ASPSCR1
GC22M037010	3.98	https://www.genecards.org/cgi-bin/carddisp.pl?gene=TST
GC11M018727	3.98	https://www.genecards.org/cgi-bin/carddisp.pl?gene=PTPN5
GC14P088005	3.98	https://www.genecards.org/cgi-bin/carddisp.pl?gene=GPR65
GC09M035797	3.98	https://www.genecards.org/cgi-bin/carddisp.pl?gene=SPAG8
GC19P022205	3.98	https://www.genecards.org/cgi-bin/carddisp.pl?gene=AP1M1
GC07P123655	3.97	https://www.genecards.org/cgi-bin/carddisp.pl?gene=LMOD2
GC16M057428	3.97	https://www.genecards.org/cgi-bin/carddisp.pl?gene=CIAPIN1
GC06P018277	3.97	https://www.genecards.org/cgi-bin/carddisp.pl?gene=RNF144B
GC13M019823	3.97	https://www.genecards.org/cgi-bin/carddisp.pl?gene=ZMYM5
GC14M039031	3.96	https://www.genecards.org/cgi-bin/carddisp.pl?gene=SEC23A
GC15M079961	3.96	https://www.genecards.org/cgi-bin/carddisp.pl?gene=BCL2A1
GC22P039401	3.96	https://www.genecards.org/cgi-bin/carddisp.pl?gene=TAB1
GC0XM048913	3.96	https://www.genecards.org/cgi-bin/carddisp.pl?gene=PIM2
GC05P114058	3.96	https://www.genecards.org/cgi-bin/carddisp.pl?gene=KCNN2
GC13M040729	3.95	https://www.genecards.org/cgi-bin/carddisp.pl?gene=MRPS31
GC08P022048	3.95	https://www.genecards.org/cgi-bin/carddisp.pl?gene=DMTN
GC15M064549	3.95	https://www.genecards.org/cgi-bin/carddisp.pl?gene=PCLAF
GC17M017843	3.95	https://www.genecards.org/cgi-bin/carddisp.pl?gene=TOM1L2
GC16M067154	3.94	https://www.genecards.org/cgi-bin/carddisp.pl?gene=TRADD
GC02M210290	3.94	https://www.genecards.org/cgi-bin/carddisp.pl?gene=MYL1
GC17P081035	3.94	https://www.genecards.org/cgi-bin/carddisp.pl?gene=BAIAP2
GC02M027254	3.94	https://www.genecards.org/cgi-bin/carddisp.pl?gene=SLC30A3
GC16P004693	3.94	https://www.genecards.org/cgi-bin/carddisp.pl?gene=NUDT16L1
GC03P058452	3.94	https://www.genecards.org/cgi-bin/carddisp.pl?gene=KCTD6
GC10P102747	3.94	https://www.genecards.org/cgi-bin/carddisp.pl?gene=CYP17A1-AS1
GC11P005689	3.94	https://www.genecards.org/cgi-bin/carddisp.pl?gene=TRIM22
GC03P156037	3.94	https://www.genecards.org/cgi-bin/carddisp.pl?gene=KCNAB1
GC01P086704	3.94	https://www.genecards.org/cgi-bin/carddisp.pl?gene=SH3GLB1
GC15P043746	3.93	https://www.genecards.org/cgi-bin/carddisp.pl?gene=PDIA3
GC22P037848	3.93	https://www.genecards.org/cgi-bin/carddisp.pl?gene=EIF3L
GC20P003820	3.93	https://www.genecards.org/cgi-bin/carddisp.pl?gene=AP5S1
GC0XM119538	3.92	https://www.genecards.org/cgi-bin/carddisp.pl?gene=CXorf56
GC09P107283	3.92	https://www.genecards.org/cgi-bin/carddisp.pl?gene=RAD23B
GC11M033740	3.92	https://www.genecards.org/cgi-bin/carddisp.pl?gene=FBXO3
GC08M020144	3.92	https://www.genecards.org/cgi-bin/carddisp.pl?gene=SLC18A1
GC01P150982	3.92	https://www.genecards.org/cgi-bin/carddisp.pl?gene=ANXA9
GC0XP155997	3.92	https://www.genecards.org/cgi-bin/carddisp.pl?gene=IL9R
GC16M071315	3.92	https://www.genecards.org/cgi-bin/carddisp.pl?gene=CMTR2
GC06P033372	3.92	https://www.genecards.org/cgi-bin/carddisp.pl?gene=GTF2H4
GC08M085327	3.92	https://www.genecards.org/cgi-bin/carddisp.pl?gene=CA1
GC01P167721	3.92	https://www.genecards.org/cgi-bin/carddisp.pl?gene=MPZL1

GC14M074059	3.92	https://www.genecards.org/cgi-bin/carddisp.pl?gene=ALDH6A1
GC21P036134	3.92	https://www.genecards.org/cgi-bin/carddisp.pl?gene=CBR3
GC02M230907	3.92	https://www.genecards.org/cgi-bin/carddisp.pl?gene=GPR55
GC04P085147	3.92	https://www.genecards.org/cgi-bin/carddisp.pl?gene=ARHGAP24
GC04M120055	3.91	https://www.genecards.org/cgi-bin/carddisp.pl?gene=MAD2L1
GC06M032756	3.91	https://www.genecards.org/cgi-bin/carddisp.pl?gene=HLA-DQB2
GC11P124115	3.91	https://www.genecards.org/cgi-bin/carddisp.pl?gene=VWA5A
GC11M104942	3.91	https://www.genecards.org/cgi-bin/carddisp.pl?gene=CASP4
GC16M030849	3.91	https://www.genecards.org/cgi-bin/carddisp.pl?gene=BCL7C
GC02P161308	3.91	https://www.genecards.org/cgi-bin/carddisp.pl?gene=PSMD14
GC20M033358	3.91	https://www.genecards.org/cgi-bin/carddisp.pl?gene=CDK5RAP1
GC0XP016668	3.91	https://www.genecards.org/cgi-bin/carddisp.pl?gene=S100G
GC19P050861	3.91	https://www.genecards.org/cgi-bin/carddisp.pl?gene=KLK2
GC06P028137	3.91	https://www.genecards.org/cgi-bin/carddisp.pl?gene=H4C11
GC02M024203	3.91	https://www.genecards.org/cgi-bin/carddisp.pl?gene=ITSN2
GC12M006556	3.91	https://www.genecards.org/cgi-bin/carddisp.pl?gene=NOP2
GC03M186789	3.9	https://www.genecards.org/cgi-bin/carddisp.pl?gene=RFC4
GC01P161524	3.9	https://www.genecards.org/cgi-bin/carddisp.pl?gene=HSPA6
GC14M023568	3.9	https://www.genecards.org/cgi-bin/carddisp.pl?gene=JPH4
GC14M052857	3.9	https://www.genecards.org/cgi-bin/carddisp.pl?gene=FERMT2
GC03P045293	3.9	https://www.genecards.org/cgi-bin/carddisp.pl?gene=CLEC3B
GC17P005078	3.9	https://www.genecards.org/cgi-bin/carddisp.pl?gene=ZFP3
GC01P044775	3.89	https://www.genecards.org/cgi-bin/carddisp.pl?gene=RPS8
GC13M019173	3.89	https://www.genecards.org/cgi-bin/carddisp.pl?gene=TUBA3C
GC06P013574	3.89	https://www.genecards.org/cgi-bin/carddisp.pl?gene=SIRT5
GC19M017403	3.89	https://www.genecards.org/cgi-bin/carddisp.pl?gene=BST2
GC02P172084	3.89	https://www.genecards.org/cgi-bin/carddisp.pl?gene=DLX1
GC16M057758	3.88	https://www.genecards.org/cgi-bin/carddisp.pl?gene=KIFC3
GC15M093035	3.88	https://www.genecards.org/cgi-bin/carddisp.pl?gene=RGMA
GC16M084053	3.88	https://www.genecards.org/cgi-bin/carddisp.pl?gene=MBTPS1
GC06P033628	3.88	https://www.genecards.org/cgi-bin/carddisp.pl?gene=MUCL3
GC05M170374	3.88	https://www.genecards.org/cgi-bin/carddisp.pl?gene=KCNMB1
GC03M129430	3.88	https://www.genecards.org/cgi-bin/carddisp.pl?gene=MBD4
GC17P032444	3.88	https://www.genecards.org/cgi-bin/carddisp.pl?gene=PSMD11
GC18M050227	3.87	https://www.genecards.org/cgi-bin/carddisp.pl?gene=CFAP53
GC08M108201	3.87	https://www.genecards.org/cgi-bin/carddisp.pl?gene=EIF3E
GC01P003797	3.87	https://www.genecards.org/cgi-bin/carddisp.pl?gene=DFFB
GC05M132751	3.87	https://www.genecards.org/cgi-bin/carddisp.pl?gene=SEPTIN8
GC01M039623	3.87	https://www.genecards.org/cgi-bin/carddisp.pl?gene=HEYL
GC21P029194	3.87	https://www.genecards.org/cgi-bin/carddisp.pl?gene=BACH1
GC17P042824	3.86	https://www.genecards.org/cgi-bin/carddisp.pl?gene=PSME3
GC01M051354	3.86	https://www.genecards.org/cgi-bin/carddisp.pl?gene=EPS15
GC11M086441	3.86	https://www.genecards.org/cgi-bin/carddisp.pl?gene=ME3
GC03M125225	3.86	https://www.genecards.org/cgi-bin/carddisp.pl?gene=ZNF148
GC05P154347	3.86	https://www.genecards.org/cgi-bin/carddisp.pl?gene=MIR1294
GC04M011394	3.85	https://www.genecards.org/cgi-bin/carddisp.pl?gene=HS3ST1
GC08P022604	3.85	https://www.genecards.org/cgi-bin/carddisp.pl?gene=CCAR2

GC01M211940	3.85	https://www.genecards.org/cgi-bin/carddisp.pl?gene=INTS7
GC0XP119236	3.85	https://www.genecards.org/cgi-bin/carddisp.pl?gene=PGRMC1
GC0XM017818	3.85	https://www.genecards.org/cgi-bin/carddisp.pl?gene=RAI2
GC08M123348	3.85	https://www.genecards.org/cgi-bin/carddisp.pl?gene=MIR548D1
GC04P155854	3.85	https://www.genecards.org/cgi-bin/carddisp.pl?gene=TDO2
GC07P151028	3.84	https://www.genecards.org/cgi-bin/carddisp.pl?gene=ABCB8
GC07P039622	3.84	https://www.genecards.org/cgi-bin/carddisp.pl?gene=RALA
GC06M030743	3.84	https://www.genecards.org/cgi-bin/carddisp.pl?gene=IER3
GC0XU990061	3.84	https://www.genecards.org/cgi-bin/carddisp.pl?gene=FRAXE
GC03P105366	3.84	https://www.genecards.org/cgi-bin/carddisp.pl?gene=ALCAM
GC19P015049	3.83	https://www.genecards.org/cgi-bin/carddisp.pl?gene=CASP14
GC16M021639	3.83	https://www.genecards.org/cgi-bin/carddisp.pl?gene=IGSF6
GCMTM005828	3.83	https://www.genecards.org/cgi-bin/carddisp.pl?gene=MT-TY
GC04P048805	3.83	https://www.genecards.org/cgi-bin/carddisp.pl?gene=OCIAD1
GC12P120469	3.83	https://www.genecards.org/cgi-bin/carddisp.pl?gene=DYNLL1
GC01P155657	3.83	https://www.genecards.org/cgi-bin/carddisp.pl?gene=DAP3
GC08M081656	3.82	https://www.genecards.org/cgi-bin/carddisp.pl?gene=IMPA1
GC17P062627	3.82	https://www.genecards.org/cgi-bin/carddisp.pl?gene=MRC2
GC17P047694	3.82	https://www.genecards.org/cgi-bin/carddisp.pl?gene=TBKBP1
GC13P053028	3.82	https://www.genecards.org/cgi-bin/carddisp.pl?gene=OLFM4
GC08M014853	3.82	https://www.genecards.org/cgi-bin/carddisp.pl?gene=MIR383
GC20P025248	3.82	https://www.genecards.org/cgi-bin/carddisp.pl?gene=PYGB
GC11P119018	3.82	https://www.genecards.org/cgi-bin/carddisp.pl?gene=TRAPPC4
GC11M078171	3.82	https://www.genecards.org/cgi-bin/carddisp.pl?gene=KCTD21
GC04P094451	3.81	https://www.genecards.org/cgi-bin/carddisp.pl?gene=PDLIM5
GC0XP071096	3.81	https://www.genecards.org/cgi-bin/carddisp.pl?gene=FOXO4
GC16P056657	3.81	https://www.genecards.org/cgi-bin/carddisp.pl?gene=MT1F
GC17M076527	3.81	https://www.genecards.org/cgi-bin/carddisp.pl?gene=CYGB
GC13P075550	3.8	https://www.genecards.org/cgi-bin/carddisp.pl?gene=UCHL3
GC05P044808	3.8	https://www.genecards.org/cgi-bin/carddisp.pl?gene=MRPS30
GC01M111722	3.8	https://www.genecards.org/cgi-bin/carddisp.pl?gene=INKA2
GC01P228644	3.8	https://www.genecards.org/cgi-bin/carddisp.pl?gene=RHOU
GC09P019049	3.8	https://www.genecards.org/cgi-bin/carddisp.pl?gene=RRAGA
GC02P008772	3.8	https://www.genecards.org/cgi-bin/carddisp.pl?gene=ID2
GC14P022871	3.8	https://www.genecards.org/cgi-bin/carddisp.pl?gene=LRP10
GC19P037735	3.8	https://www.genecards.org/cgi-bin/carddisp.pl?gene=HCST
GC05M058453	3.79	https://www.genecards.org/cgi-bin/carddisp.pl?gene=PLK2
GC05P108747	3.79	https://www.genecards.org/cgi-bin/carddisp.pl?gene=FER
GC03M049806	3.79	https://www.genecards.org/cgi-bin/carddisp.pl?gene=MIR5193
GC14P059484	3.79	https://www.genecards.org/cgi-bin/carddisp.pl?gene=JKAMP
GC06P087590	3.79	https://www.genecards.org/cgi-bin/carddisp.pl?gene=ORC3
GC01M193065	3.79	https://www.genecards.org/cgi-bin/carddisp.pl?gene=GLRX2
GC07M135926	3.79	https://www.genecards.org/cgi-bin/carddisp.pl?gene=MTPN
GC01P151138	3.78	https://www.genecards.org/cgi-bin/carddisp.pl?gene=SCNM1
GC12P002959	3.78	https://www.genecards.org/cgi-bin/carddisp.pl?gene=TEAD4
GC12M054640	3.78	https://www.genecards.org/cgi-bin/carddisp.pl?gene=LACRT
GC22P025517	3.78	https://www.genecards.org/cgi-bin/carddisp.pl?gene=GRK3

GC10P115093	3.78	https://www.genecards.org/cgi-bin/carddisp.pl?gene=ATRNL1
GC19M047428	3.78	https://www.genecards.org/cgi-bin/carddisp.pl?gene=SLC8A2
GC17P050095	3.78	https://www.genecards.org/cgi-bin/carddisp.pl?gene=PDK2
GCMTP010407	3.77	https://www.genecards.org/cgi-bin/carddisp.pl?gene=MT-TR
GC03M134065	3.77	https://www.genecards.org/cgi-bin/carddisp.pl?gene=RYK
GC19U902317	3.77	https://www.genecards.org/cgi-bin/carddisp.pl?gene=LOC108663985
GC12M113816	3.77	https://www.genecards.org/cgi-bin/carddisp.pl?gene=RBM19
GC01P001308	3.77	https://www.genecards.org/cgi-bin/carddisp.pl?gene=PUSL1
GC11M067364	3.76	https://www.genecards.org/cgi-bin/carddisp.pl?gene=CLCF1
GC04M189982	3.76	https://www.genecards.org/cgi-bin/carddisp.pl?gene=TUBB7P
GC07M123681	3.76	https://www.genecards.org/cgi-bin/carddisp.pl?gene=WASL
GC02M045005	3.76	https://www.genecards.org/cgi-bin/carddisp.pl?gene=SIX2
GC10P102869	3.76	https://www.genecards.org/cgi-bin/carddisp.pl?gene=AS3MT
GC05M150378	3.76	https://www.genecards.org/cgi-bin/carddisp.pl?gene=CD74
GC05M097091	3.76	https://www.genecards.org/cgi-bin/carddisp.pl?gene=LIX1
GC0XP101392	3.75	https://www.genecards.org/cgi-bin/carddisp.pl?gene=RPL36A
GC19P042220	3.75	https://www.genecards.org/cgi-bin/carddisp.pl?gene=ZNF526
GC22P046112	3.75	https://www.genecards.org/cgi-bin/carddisp.pl?gene=MIRLET7A3
GC11P063985	3.75	https://www.genecards.org/cgi-bin/carddisp.pl?gene=OTUB1
GC02M046489	3.75	https://www.genecards.org/cgi-bin/carddisp.pl?gene=ATP6V1E2
GC03M120164	3.75	https://www.genecards.org/cgi-bin/carddisp.pl?gene=GPR156
GC14P035123	3.75	https://www.genecards.org/cgi-bin/carddisp.pl?gene=PRORP
GC01M077944	3.75	https://www.genecards.org/cgi-bin/carddisp.pl?gene=FUBP1
GC04M184387	3.75	https://www.genecards.org/cgi-bin/carddisp.pl?gene=IRF2
GC03M150741	3.75	https://www.genecards.org/cgi-bin/carddisp.pl?gene=SIAH2
GC21P038805	3.75	https://www.genecards.org/cgi-bin/carddisp.pl?gene=ETS2
GC19P048872	3.75	https://www.genecards.org/cgi-bin/carddisp.pl?gene=PPP1R15A
GC02P216498	3.75	https://www.genecards.org/cgi-bin/carddisp.pl?gene=RPL37A
GC07M055795	3.75	https://www.genecards.org/cgi-bin/carddisp.pl?gene=SEPTIN14
GC11P000915	3.74	https://www.genecards.org/cgi-bin/carddisp.pl?gene=AP2A2
GCMTP004404	3.74	https://www.genecards.org/cgi-bin/carddisp.pl?gene=MT-TM
GC02M042312	3.74	https://www.genecards.org/cgi-bin/carddisp.pl?gene=COX7A2L
GC04M067471	3.74	https://www.genecards.org/cgi-bin/carddisp.pl?gene=CENPC
GC19M004444	3.74	https://www.genecards.org/cgi-bin/carddisp.pl?gene=UBXN6
GC13M045192	3.73	https://www.genecards.org/cgi-bin/carddisp.pl?gene=KCTD4
GC12M053516	3.73	https://www.genecards.org/cgi-bin/carddisp.pl?gene=NPFF
GC19M018279	3.73	https://www.genecards.org/cgi-bin/carddisp.pl?gene=JUND
GC16M000357	3.73	https://www.genecards.org/cgi-bin/carddisp.pl?gene=MRPL28
GC20P001948	3.73	https://www.genecards.org/cgi-bin/carddisp.pl?gene=PDYN-AS1
GC01P002391	3.73	https://www.genecards.org/cgi-bin/carddisp.pl?gene=RER1
GC11P110429	3.73	https://www.genecards.org/cgi-bin/carddisp.pl?gene=FDX1
GC12M076025	3.73	https://www.genecards.org/cgi-bin/carddisp.pl?gene=PHLDA1
GC01M037802	3.72	https://www.genecards.org/cgi-bin/carddisp.pl?gene=YRDC
GC01P156212	3.72	https://www.genecards.org/cgi-bin/carddisp.pl?gene=PMF1
GC09P131373	3.72	https://www.genecards.org/cgi-bin/carddisp.pl?gene=PRRC2B
GC22P031212	3.72	https://www.genecards.org/cgi-bin/carddisp.pl?gene=LIMK2
GC20M049113	3.72	https://www.genecards.org/cgi-bin/carddisp.pl?gene=STAU1

GC01P037563	3.72	https://www.genecards.org/cgi-bin/carddisp.pl?gene=DNALI1
GC12M056202	3.72	https://www.genecards.org/cgi-bin/carddisp.pl?gene=RNF41
GC11P065925	3.72	https://www.genecards.org/cgi-bin/carddisp.pl?gene=DRAP1
GC0YP020756	3.72	https://www.genecards.org/cgi-bin/carddisp.pl?gene=RPS4Y2
GC17M045263	3.72	https://www.genecards.org/cgi-bin/carddisp.pl?gene=MAP3K14
GC14P025014	3.72	https://www.genecards.org/cgi-bin/carddisp.pl?gene=THTPA
GC01P174968	3.72	https://www.genecards.org/cgi-bin/carddisp.pl?gene=CACYBP
GC12M123470	3.71	https://www.genecards.org/cgi-bin/carddisp.pl?gene=RILPL1
GC19M003777	3.71	https://www.genecards.org/cgi-bin/carddisp.pl?gene=MATK
GC15M055180	3.71	https://www.genecards.org/cgi-bin/carddisp.pl?gene=RSL24D1
GC01P111755	3.71	https://www.genecards.org/cgi-bin/carddisp.pl?gene=DDX20
GC03P116709	3.71	https://www.genecards.org/cgi-bin/carddisp.pl?gene=TUSC7
GC01P011189	3.7	https://www.genecards.org/cgi-bin/carddisp.pl?gene=ANGPTL7
GC20P062952	3.7	https://www.genecards.org/cgi-bin/carddisp.pl?gene=SLC17A9
GC03P126704	3.7	https://www.genecards.org/cgi-bin/carddisp.pl?gene=CHCHD6
GC22P028803	3.7	https://www.genecards.org/cgi-bin/carddisp.pl?gene=ZNRF3
GC07M072559	3.69	https://www.genecards.org/cgi-bin/carddisp.pl?gene=TYW1B
GC19M047220	3.69	https://www.genecards.org/cgi-bin/carddisp.pl?gene=BBC3
GC01P046303	3.69	https://www.genecards.org/cgi-bin/carddisp.pl?gene=UQCRH
GC10M005765	3.69	https://www.genecards.org/cgi-bin/carddisp.pl?gene=GDI2
GC08P098427	3.69	https://www.genecards.org/cgi-bin/carddisp.pl?gene=KCNS2
GC05P163457	3.69	https://www.genecards.org/cgi-bin/carddisp.pl?gene=CCNG1
GC12M110709	3.68	https://www.genecards.org/cgi-bin/carddisp.pl?gene=PPP1CC
GC11P062856	3.68	https://www.genecards.org/cgi-bin/carddisp.pl?gene=SLC3A2
GC17M048607	3.68	https://www.genecards.org/cgi-bin/carddisp.pl?gene=HOXB7
GC16P020610	3.68	https://www.genecards.org/cgi-bin/carddisp.pl?gene=ACSM3
GC16M000058	3.68	https://www.genecards.org/cgi-bin/carddisp.pl?gene=RHBDF1
GC12P106583	3.68	https://www.genecards.org/cgi-bin/carddisp.pl?gene=RFX4
GC01P169105	3.68	https://www.genecards.org/cgi-bin/carddisp.pl?gene=ATP1B1
GC19P003149	3.67	https://www.genecards.org/cgi-bin/carddisp.pl?gene=GNA15
GC19P038374	3.67	https://www.genecards.org/cgi-bin/carddisp.pl?gene=PSMD8
GC06M127438	3.67	https://www.genecards.org/cgi-bin/carddisp.pl?gene=SOGA3
GC01M163111	3.67	https://www.genecards.org/cgi-bin/carddisp.pl?gene=RGS5
GC06M116793	3.67	https://www.genecards.org/cgi-bin/carddisp.pl?gene=GPRC6A
GC06P110958	3.66	https://www.genecards.org/cgi-bin/carddisp.pl?gene=GTF3C6
GC03M122421	3.66	https://www.genecards.org/cgi-bin/carddisp.pl?gene=KPNA1
GC01M000952	3.66	https://www.genecards.org/cgi-bin/carddisp.pl?gene=NOC2L
GC07P100875	3.66	https://www.genecards.org/cgi-bin/carddisp.pl?gene=SRRT
GC16M058707	3.66	https://www.genecards.org/cgi-bin/carddisp.pl?gene=GOT2
GC19P054953	3.66	https://www.genecards.org/cgi-bin/carddisp.pl?gene=NLRP2
GC09P089318	3.66	https://www.genecards.org/cgi-bin/carddisp.pl?gene=SECISBP2
GC12P051047	3.65	https://www.genecards.org/cgi-bin/carddisp.pl?gene=LETMD1
GC12P067270	3.65	https://www.genecards.org/cgi-bin/carddisp.pl?gene=CAND1
GC03M142306	3.65	https://www.genecards.org/cgi-bin/carddisp.pl?gene=XRN1
GC17M056938	3.65	https://www.genecards.org/cgi-bin/carddisp.pl?gene=COIL
GC17M007015	3.65	https://www.genecards.org/cgi-bin/carddisp.pl?gene=MIR497HG
GC03P183886	3.65	https://www.genecards.org/cgi-bin/carddisp.pl?gene=MIR4448

GC14P025033	3.65	https://www.genecards.org/cgi-bin/carddisp.pl?gene=BCL2L2
GC19P039412	3.65	https://www.genecards.org/cgi-bin/carddisp.pl?gene=PLEKHG2
GC09M133338	3.65	https://www.genecards.org/cgi-bin/carddisp.pl?gene=MED22
GC22P043955	3.65	https://www.genecards.org/cgi-bin/carddisp.pl?gene=SAMM50
GC0XM139924	3.65	https://www.genecards.org/cgi-bin/carddisp.pl?gene=MIR505
GC07M002728	3.64	https://www.genecards.org/cgi-bin/carddisp.pl?gene=GNA12
GC20P032819	3.64	https://www.genecards.org/cgi-bin/carddisp.pl?gene=MAPRE1
GC12P104215	3.64	https://www.genecards.org/cgi-bin/carddisp.pl?gene=TXNRD1
GC12M057234	3.64	https://www.genecards.org/cgi-bin/carddisp.pl?gene=NDUFA4L2
GC11P108569	3.64	https://www.genecards.org/cgi-bin/carddisp.pl?gene=DDX10
GC12M052468	3.64	https://www.genecards.org/cgi-bin/carddisp.pl?gene=KRT6C
GC01P109984	3.64	https://www.genecards.org/cgi-bin/carddisp.pl?gene=AHCYL1
GC04M139267	3.64	https://www.genecards.org/cgi-bin/carddisp.pl?gene=NDUFC1
GC03P000213	3.63	https://www.genecards.org/cgi-bin/carddisp.pl?gene=CHL1
GC04M118280	3.63	https://www.genecards.org/cgi-bin/carddisp.pl?gene=PRSS12
GC14P052730	3.63	https://www.genecards.org/cgi-bin/carddisp.pl?gene=STYX
GC07M001441	3.63	https://www.genecards.org/cgi-bin/carddisp.pl?gene=MICALL2
GC16P023557	3.63	https://www.genecards.org/cgi-bin/carddisp.pl?gene=UBFD1
GC15M070047	3.62	https://www.genecards.org/cgi-bin/carddisp.pl?gene=TLE3
GC10M097766	3.62	https://www.genecards.org/cgi-bin/carddisp.pl?gene=SFRP5
GC01M145670	3.62	https://www.genecards.org/cgi-bin/carddisp.pl?gene=PDZK1
GC04P155759	3.62	https://www.genecards.org/cgi-bin/carddisp.pl?gene=GUCY1B1
GC17M063819	3.62	https://www.genecards.org/cgi-bin/carddisp.pl?gene=FTSJ3
GC17M045393	3.62	https://www.genecards.org/cgi-bin/carddisp.pl?gene=ARHGAP27
GC03M100364	3.62	https://www.genecards.org/cgi-bin/carddisp.pl?gene=TOMM70
GC10P072692	3.62	https://www.genecards.org/cgi-bin/carddisp.pl?gene=MCU
GC12M108522	3.62	https://www.genecards.org/cgi-bin/carddisp.pl?gene=SART3
GC06M158636	3.61	https://www.genecards.org/cgi-bin/carddisp.pl?gene=DYNLT1
GC17P046833	3.61	https://www.genecards.org/cgi-bin/carddisp.pl?gene=WNT9B
GC04P070704	3.61	https://www.genecards.org/cgi-bin/carddisp.pl?gene=RUFY3
GC05P102753	3.61	https://www.genecards.org/cgi-bin/carddisp.pl?gene=PAM
GC17P043170	3.61	https://www.genecards.org/cgi-bin/carddisp.pl?gene=NBR1
GC10P058269	3.6	https://www.genecards.org/cgi-bin/carddisp.pl?gene=CISD1
GC09M127447	3.6	https://www.genecards.org/cgi-bin/carddisp.pl?gene=RPL12
GC01P032221	3.6	https://www.genecards.org/cgi-bin/carddisp.pl?gene=EIF3I
GC01M153661	3.6	https://www.genecards.org/cgi-bin/carddisp.pl?gene=ILF2
GC11P059107	3.6	https://www.genecards.org/cgi-bin/carddisp.pl?gene=FAM111B
GC03M134355	3.6	https://www.genecards.org/cgi-bin/carddisp.pl?gene=AMOTL2
GC09M121338	3.6	https://www.genecards.org/cgi-bin/carddisp.pl?gene=STOM
GC12M006851	3.59	https://www.genecards.org/cgi-bin/carddisp.pl?gene=SPSB2
GC01P021440	3.59	https://www.genecards.org/cgi-bin/carddisp.pl?gene=NBPF3
GC13M098445	3.59	https://www.genecards.org/cgi-bin/carddisp.pl?gene=STK24
GC15M101297	3.59	https://www.genecards.org/cgi-bin/carddisp.pl?gene=PCSK6
GC07P000876	3.58	https://www.genecards.org/cgi-bin/carddisp.pl?gene=GET4
GC03P172039	3.58	https://www.genecards.org/cgi-bin/carddisp.pl?gene=FNDC3B
GC06M032168	3.58	https://www.genecards.org/cgi-bin/carddisp.pl?gene=AGPAT1
GC19P045039	3.57	https://www.genecards.org/cgi-bin/carddisp.pl?gene=CLASRP

GC03M127689	3.57	https://www.genecards.org/cgi-bin/carddisp.pl?gene=MGLL
GC19P053720	3.57	https://www.genecards.org/cgi-bin/carddisp.pl?gene=MIR520D
GC21P045073	3.57	https://www.genecards.org/cgi-bin/carddisp.pl?gene=ADARB1
GC10M094333	3.57	https://www.genecards.org/cgi-bin/carddisp.pl?gene=NOC3L
GC04M004269	3.57	https://www.genecards.org/cgi-bin/carddisp.pl?gene=LYAR
GC01P151129	3.57	https://www.genecards.org/cgi-bin/carddisp.pl?gene=TNFAIP8L2
GC17P048893	3.57	https://www.genecards.org/cgi-bin/carddisp.pl?gene=ATP5MC1
GC10M101825	3.57	https://www.genecards.org/cgi-bin/carddisp.pl?gene=KCNIP2
GC18M063389	3.56	https://www.genecards.org/cgi-bin/carddisp.pl?gene=VPS4B
GC20P063180	3.56	https://www.genecards.org/cgi-bin/carddisp.pl?gene=MIR124-3
GC02P073828	3.56	https://www.genecards.org/cgi-bin/carddisp.pl?gene=STAMBP
GC14M024143	3.56	https://www.genecards.org/cgi-bin/carddisp.pl?gene=PSME2
GC0XM107713	3.55	https://www.genecards.org/cgi-bin/carddisp.pl?gene=TSC22D3
GC0XP135889	3.55	https://www.genecards.org/cgi-bin/carddisp.pl?gene=SAGE1
GC01M086346	3.55	https://www.genecards.org/cgi-bin/carddisp.pl?gene=ODF2L
GC19P012946	3.55	https://www.genecards.org/cgi-bin/carddisp.pl?gene=RAD23A
GC03P036421	3.55	https://www.genecards.org/cgi-bin/carddisp.pl?gene=STAC
GC11M118226	3.55	https://www.genecards.org/cgi-bin/carddisp.pl?gene=MPZL3
GC15M082838	3.55	https://www.genecards.org/cgi-bin/carddisp.pl?gene=HOMER2
GC19M047968	3.55	https://www.genecards.org/cgi-bin/carddisp.pl?gene=BSPH1
GC10M056357	3.55	https://www.genecards.org/cgi-bin/carddisp.pl?gene=ZWINT
GC02P042049	3.55	https://www.genecards.org/cgi-bin/carddisp.pl?gene=PKDCC
GC09P093523	3.55	https://www.genecards.org/cgi-bin/carddisp.pl?gene=FAM120A
GC11P073264	3.55	https://www.genecards.org/cgi-bin/carddisp.pl?gene=P2RY6
GC17P007405	3.54	https://www.genecards.org/cgi-bin/carddisp.pl?gene=NLGN2
GC10P006144	3.54	https://www.genecards.org/cgi-bin/carddisp.pl?gene=PFKFB3
GC01M016248	3.54	https://www.genecards.org/cgi-bin/carddisp.pl?gene=FBXO42
GC11M063101	3.54	https://www.genecards.org/cgi-bin/carddisp.pl?gene=SLC22A6
GC02M071110	3.54	https://www.genecards.org/cgi-bin/carddisp.pl?gene=MCEE
GC12P123584	3.54	https://www.genecards.org/cgi-bin/carddisp.pl?gene=TMED2
GC01P155127	3.54	https://www.genecards.org/cgi-bin/carddisp.pl?gene=EFNA1
GC10M119499	3.54	https://www.genecards.org/cgi-bin/carddisp.pl?gene=RGS10
GC06P160121	3.53	https://www.genecards.org/cgi-bin/carddisp.pl?gene=SLC22A1
GC21P044107	3.53	https://www.genecards.org/cgi-bin/carddisp.pl?gene=PWP2
GC11P068460	3.53	https://www.genecards.org/cgi-bin/carddisp.pl?gene=PPP6R3
GC15P081324	3.53	https://www.genecards.org/cgi-bin/carddisp.pl?gene=TMC3-AS1
GC19P045507	3.53	https://www.genecards.org/cgi-bin/carddisp.pl?gene=VASP
GC09M093951	3.53	https://www.genecards.org/cgi-bin/carddisp.pl?gene=BARX1
GC07M091692	3.53	https://www.genecards.org/cgi-bin/carddisp.pl?gene=MTERF1
GC03P039093	3.52	https://www.genecards.org/cgi-bin/carddisp.pl?gene=WDR48
GC08M022138	3.52	https://www.genecards.org/cgi-bin/carddisp.pl?gene=REEP4
GC12P132085	3.52	https://www.genecards.org/cgi-bin/carddisp.pl?gene=EP400P1
GC19P018831	3.52	https://www.genecards.org/cgi-bin/carddisp.pl?gene=UPF1
GC07P002354	3.51	https://www.genecards.org/cgi-bin/carddisp.pl?gene=EIF3B
GC03P184561	3.51	https://www.genecards.org/cgi-bin/carddisp.pl?gene=EPHB3
GC03P014947	3.51	https://www.genecards.org/cgi-bin/carddisp.pl?gene=NR2C2
GC19M041410	3.51	https://www.genecards.org/cgi-bin/carddisp.pl?gene=HSPB6

GC19P043716	3.51	https://www.genecards.org/cgi-bin/carddisp.pl?gene=IRGC
GC12P016347	3.51	https://www.genecards.org/cgi-bin/carddisp.pl?gene=MGST1
GC15P075346	3.51	https://www.genecards.org/cgi-bin/carddisp.pl?gene=NEIL1
GC19P038899	3.51	https://www.genecards.org/cgi-bin/carddisp.pl?gene=NFKBIB
GC06P028391	3.51	https://www.genecards.org/cgi-bin/carddisp.pl?gene=H4C9
GC02P044361	3.51	https://www.genecards.org/cgi-bin/carddisp.pl?gene=CAMKMT
GC02M168231	3.51	https://www.genecards.org/cgi-bin/carddisp.pl?gene=PHF5GP
GC15P042491	3.51	https://www.genecards.org/cgi-bin/carddisp.pl?gene=SNAP23
GC02P088691	3.5	https://www.genecards.org/cgi-bin/carddisp.pl?gene=RPIA
GC12P118013	3.5	https://www.genecards.org/cgi-bin/carddisp.pl?gene=RFC5
GC11M104995	3.5	https://www.genecards.org/cgi-bin/carddisp.pl?gene=CASP5
GC22P037822	3.5	https://www.genecards.org/cgi-bin/carddisp.pl?gene=H1-0
GC02P075646	3.5	https://www.genecards.org/cgi-bin/carddisp.pl?gene=MRPL19
GC08P081282	3.5	https://www.genecards.org/cgi-bin/carddisp.pl?gene=FABP5
GC20M057648	3.5	https://www.genecards.org/cgi-bin/carddisp.pl?gene=PMEPA1
GC06P033208	3.49	https://www.genecards.org/cgi-bin/carddisp.pl?gene=RING1
GC20M009538	3.49	https://www.genecards.org/cgi-bin/carddisp.pl?gene=PAK5
GC16M071729	3.49	https://www.genecards.org/cgi-bin/carddisp.pl?gene=AP1G1
GC12M113157	3.49	https://www.genecards.org/cgi-bin/carddisp.pl?gene=DDX54
GC12M047661	3.49	https://www.genecards.org/cgi-bin/carddisp.pl?gene=RPAP3
GC11P065979	3.49	https://www.genecards.org/cgi-bin/carddisp.pl?gene=SART1
GC11M066436	3.49	https://www.genecards.org/cgi-bin/carddisp.pl?gene=MRPL11
GC0XP016141	3.48	https://www.genecards.org/cgi-bin/carddisp.pl?gene=GRPR
GC0XP023665	3.48	https://www.genecards.org/cgi-bin/carddisp.pl?gene=PRDX4
GC19M058555	3.48	https://www.genecards.org/cgi-bin/carddisp.pl?gene=UBE2M
GC12M024732	3.48	https://www.genecards.org/cgi-bin/carddisp.pl?gene=BCAT1
GC01M074705	3.48	https://www.genecards.org/cgi-bin/carddisp.pl?gene=CRYZ
GC19M054107	3.47	https://www.genecards.org/cgi-bin/carddisp.pl?gene=TFPT
GC13P111114	3.47	https://www.genecards.org/cgi-bin/carddisp.pl?gene=ARHGEF7
GC05M131641	3.47	https://www.genecards.org/cgi-bin/carddisp.pl?gene=FNIP1
GC08M017224	3.47	https://www.genecards.org/cgi-bin/carddisp.pl?gene=CNOT7
GC15P088611	3.47	https://www.genecards.org/cgi-bin/carddisp.pl?gene=MIR7-2
GC03M160494	3.47	https://www.genecards.org/cgi-bin/carddisp.pl?gene=KPNA4
GC06M030652	3.47	https://www.genecards.org/cgi-bin/carddisp.pl?gene=TRIM31
GC07M002234	3.47	https://www.genecards.org/cgi-bin/carddisp.pl?gene=MRM2
GC22M050569	3.47	https://www.genecards.org/cgi-bin/carddisp.pl?gene=CPT1B
GC01P154325	3.47	https://www.genecards.org/cgi-bin/carddisp.pl?gene=ATP8B2
GC16M046955	3.47	https://www.genecards.org/cgi-bin/carddisp.pl?gene=DNAJA2
GC17M081665	3.47	https://www.genecards.org/cgi-bin/carddisp.pl?gene=OXLD1
GC02M179441	3.47	https://www.genecards.org/cgi-bin/carddisp.pl?gene=ZNF385B
GC11M059690	3.47	https://www.genecards.org/cgi-bin/carddisp.pl?gene=ACP2
GC17P008288	3.46	https://www.genecards.org/cgi-bin/carddisp.pl?gene=RANGRF
GC15M035217	3.46	https://www.genecards.org/cgi-bin/carddisp.pl?gene=DPH6
GC14M105212	3.46	https://www.genecards.org/cgi-bin/carddisp.pl?gene=BRF1
GC08M027869	3.46	https://www.genecards.org/cgi-bin/carddisp.pl?gene=SCARA5
GC20M003587	3.46	https://www.genecards.org/cgi-bin/carddisp.pl?gene=GFRA4
GC07P144742	3.45	https://www.genecards.org/cgi-bin/carddisp.pl?gene=GSTK1

GC01P054053	3.45	https://www.genecards.org/cgi-bin/carddisp.pl?gene=TCEANC2
GC02P012706	3.45	https://www.genecards.org/cgi-bin/carddisp.pl?gene=TRIB2
GC21M033503	3.45	https://www.genecards.org/cgi-bin/carddisp.pl?gene=GART
GC17P008424	3.45	https://www.genecards.org/cgi-bin/carddisp.pl?gene=NDEL1
GC14P024136	3.45	https://www.genecards.org/cgi-bin/carddisp.pl?gene=PSME1
GC04M053459	3.45	https://www.genecards.org/cgi-bin/carddisp.pl?gene=LNX1
GC22P024806	3.45	https://www.genecards.org/cgi-bin/carddisp.pl?gene=SGSM1
GC09P036180	3.44	https://www.genecards.org/cgi-bin/carddisp.pl?gene=CLTA
GC06P041228	3.44	https://www.genecards.org/cgi-bin/carddisp.pl?gene=TREML4
GC01M149917	3.44	https://www.genecards.org/cgi-bin/carddisp.pl?gene=H3C14
GC17M007922	3.43	https://www.genecards.org/cgi-bin/carddisp.pl?gene=KCNAB3
GC11M118748	3.43	https://www.genecards.org/cgi-bin/carddisp.pl?gene=DDX6
GC17M043800	3.43	https://www.genecards.org/cgi-bin/carddisp.pl?gene=MPP3
GC05P005140	3.43	https://www.genecards.org/cgi-bin/carddisp.pl?gene=ADAMTS16
GC19M012953	3.43	https://www.genecards.org/cgi-bin/carddisp.pl?gene=GADD45GIP1
GC21P041361	3.43	https://www.genecards.org/cgi-bin/carddisp.pl?gene=MX2
GC16P019179	3.43	https://www.genecards.org/cgi-bin/carddisp.pl?gene=SYT17
GC16P000142	3.43	https://www.genecards.org/cgi-bin/carddisp.pl?gene=HBZ
GC01P035869	3.43	https://www.genecards.org/cgi-bin/carddisp.pl?gene=AGO1
GC07M073680	3.43	https://www.genecards.org/cgi-bin/carddisp.pl?gene=DNAJC30
GC12P019404	3.43	https://www.genecards.org/cgi-bin/carddisp.pl?gene=AEBP2
GC08M006924	3.43	https://www.genecards.org/cgi-bin/carddisp.pl?gene=DEFA6
GC02P166888	3.43	https://www.genecards.org/cgi-bin/carddisp.pl?gene=XIRP2
GC22P023070	3.42	https://www.genecards.org/cgi-bin/carddisp.pl?gene=GNAZ
GC12M053662	3.42	https://www.genecards.org/cgi-bin/carddisp.pl?gene=ATP5MC2
GC12M084859	3.42	https://www.genecards.org/cgi-bin/carddisp.pl?gene=SLC6A15
GC02P176177	3.42	https://www.genecards.org/cgi-bin/carddisp.pl?gene=HAGLROS
GC12M056470	3.42	https://www.genecards.org/cgi-bin/carddisp.pl?gene=GLS2
GC03M193401	3.41	https://www.genecards.org/cgi-bin/carddisp.pl?gene=ATP13A4
GC12P071839	3.41	https://www.genecards.org/cgi-bin/carddisp.pl?gene=TBC1D15
GC10M032035	3.41	https://www.genecards.org/cgi-bin/carddisp.pl?gene=KIF5B
GC07P128207	3.41	https://www.genecards.org/cgi-bin/carddisp.pl?gene=MIR129-1
GC01M212726	3.41	https://www.genecards.org/cgi-bin/carddisp.pl?gene=NSL1
GC10M070147	3.41	https://www.genecards.org/cgi-bin/carddisp.pl?gene=SAR1A
GC03M009774	3.41	https://www.genecards.org/cgi-bin/carddisp.pl?gene=CAMK1
GC01M153039	3.41	https://www.genecards.org/cgi-bin/carddisp.pl?gene=SPRR2D
GC01P091500	3.4	https://www.genecards.org/cgi-bin/carddisp.pl?gene=CDC7
GC01P228139	3.4	https://www.genecards.org/cgi-bin/carddisp.pl?gene=GUK1
GC04P159106	3.4	https://www.genecards.org/cgi-bin/carddisp.pl?gene=RAPGEF2
GC08P001973	3.4	https://www.genecards.org/cgi-bin/carddisp.pl?gene=KBTBD11
GC0XM078129	3.4	https://www.genecards.org/cgi-bin/carddisp.pl?gene=TAF9B
GC03P161104	3.4	https://www.genecards.org/cgi-bin/carddisp.pl?gene=NMD3
GC16M029870	3.4	https://www.genecards.org/cgi-bin/carddisp.pl?gene=CDIPT
GC02M238238	3.4	https://www.genecards.org/cgi-bin/carddisp.pl?gene=HES6
GC12P000972	3.4	https://www.genecards.org/cgi-bin/carddisp.pl?gene=ERC1
GC10P130136	3.4	https://www.genecards.org/cgi-bin/carddisp.pl?gene=GLRX3
GC03P052411	3.39	https://www.genecards.org/cgi-bin/carddisp.pl?gene=PHF7

GC04P066431	3.39	https://www.genecards.org/cgi-bin/carddisp.pl?gene=RPS23P3
GC02M241227	3.39	https://www.genecards.org/cgi-bin/carddisp.pl?gene=HDLBP
GC19P001248	3.39	https://www.genecards.org/cgi-bin/carddisp.pl?gene=MIDN
GC15M040035	3.39	https://www.genecards.org/cgi-bin/carddisp.pl?gene=SRP14
GC02M046901	3.39	https://www.genecards.org/cgi-bin/carddisp.pl?gene=MCFD2
GC16M018805	3.39	https://www.genecards.org/cgi-bin/carddisp.pl?gene=SMG1
GC0XP051803	3.39	https://www.genecards.org/cgi-bin/carddisp.pl?gene=MAGED1
GC18P003238	3.38	https://www.genecards.org/cgi-bin/carddisp.pl?gene=MYL12A
GC11P071928	3.38	https://www.genecards.org/cgi-bin/carddisp.pl?gene=RNF121
GC05M115804	3.38	https://www.genecards.org/cgi-bin/carddisp.pl?gene=CDO1
GC15M022983	3.38	https://www.genecards.org/cgi-bin/carddisp.pl?gene=TUBGCP5
GC07M130026	3.38	https://www.genecards.org/cgi-bin/carddisp.pl?gene=ZC3HC1
GC05P177357	3.37	https://www.genecards.org/cgi-bin/carddisp.pl?gene=RGS14
GC17P047896	3.37	https://www.genecards.org/cgi-bin/carddisp.pl?gene=SP2
GC22P039014	3.37	https://www.genecards.org/cgi-bin/carddisp.pl?gene=APOBEC3C
GC07P155644	3.36	https://www.genecards.org/cgi-bin/carddisp.pl?gene=RBM33
GC16P022217	3.36	https://www.genecards.org/cgi-bin/carddisp.pl?gene=EEF2K
GC04P163343	3.36	https://www.genecards.org/cgi-bin/carddisp.pl?gene=NPY5R
GC02P202265	3.36	https://www.genecards.org/cgi-bin/carddisp.pl?gene=NOP58
GC02P134918	3.36	https://www.genecards.org/cgi-bin/carddisp.pl?gene=CCNT2
GC0XP055000	3.35	https://www.genecards.org/cgi-bin/carddisp.pl?gene=APEX2
GC21P034108	3.35	https://www.genecards.org/cgi-bin/carddisp.pl?gene=MRPS6
GC02P085635	3.35	https://www.genecards.org/cgi-bin/carddisp.pl?gene=USP39
GC02P030108	3.35	https://www.genecards.org/cgi-bin/carddisp.pl?gene=YPEL5
GC01M019339	3.35	https://www.genecards.org/cgi-bin/carddisp.pl?gene=CAPZB
GC16M087696	3.35	https://www.genecards.org/cgi-bin/carddisp.pl?gene=KLHDC4
GC20P017550	3.35	https://www.genecards.org/cgi-bin/carddisp.pl?gene=DSTN
GC08M079919	3.35	https://www.genecards.org/cgi-bin/carddisp.pl?gene=MRPS28
GC06P006588	3.35	https://www.genecards.org/cgi-bin/carddisp.pl?gene=LY86
GC08P022440	3.35	https://www.genecards.org/cgi-bin/carddisp.pl?gene=PPP3CC
GC19M055399	3.34	https://www.genecards.org/cgi-bin/carddisp.pl?gene=UBE2S
GC12M112026	3.34	https://www.genecards.org/cgi-bin/carddisp.pl?gene=NAA25
GC18P070288	3.34	https://www.genecards.org/cgi-bin/carddisp.pl?gene=SOCS6
GC04P070242	3.34	https://www.genecards.org/cgi-bin/carddisp.pl?gene=CSN3
GC04P003443	3.34	https://www.genecards.org/cgi-bin/carddisp.pl?gene=HGFAC
GC14P071320	3.34	https://www.genecards.org/cgi-bin/carddisp.pl?gene=SIPA1L1
GC14P069191	3.34	https://www.genecards.org/cgi-bin/carddisp.pl?gene=EXD2
GC05P079071	3.34	https://www.genecards.org/cgi-bin/carddisp.pl?gene=BHMT2
GC14M093533	3.34	https://www.genecards.org/cgi-bin/carddisp.pl?gene=TC2N
GC06M170590	3.34	https://www.genecards.org/cgi-bin/carddisp.pl?gene=PDCD2
GC05M126869	3.33	https://www.genecards.org/cgi-bin/carddisp.pl?gene=MARCHF3
GC19M047475	3.33	https://www.genecards.org/cgi-bin/carddisp.pl?gene=KPTN
GC12M106237	3.33	https://www.genecards.org/cgi-bin/carddisp.pl?gene=CKAP4
GC17M007173	3.33	https://www.genecards.org/cgi-bin/carddisp.pl?gene=ASGR1
GC01M032600	3.33	https://www.genecards.org/cgi-bin/carddisp.pl?gene=ZBTB8OS
GC10M096998	3.33	https://www.genecards.org/cgi-bin/carddisp.pl?gene=SLIT1
GC01M023964	3.33	https://www.genecards.org/cgi-bin/carddisp.pl?gene=SRSF10

GC17M069244	3.32	https://www.genecards.org/cgi-bin/carddisp.pl?gene=ABCA5
GC21P039728	3.32	https://www.genecards.org/cgi-bin/carddisp.pl?gene=IGSF5
GC10M101578	3.32	https://www.genecards.org/cgi-bin/carddisp.pl?gene=POLL
GC10M070298	3.32	https://www.genecards.org/cgi-bin/carddisp.pl?gene=LRRC20
GC18P023504	3.32	https://www.genecards.org/cgi-bin/carddisp.pl?gene=RMC1
GC13P036431	3.32	https://www.genecards.org/cgi-bin/carddisp.pl?gene=CCNA1
GC01P204516	3.32	https://www.genecards.org/cgi-bin/carddisp.pl?gene=MDM4
GC20P049219	3.32	https://www.genecards.org/cgi-bin/carddisp.pl?gene=DDX27
GC19M007926	3.32	https://www.genecards.org/cgi-bin/carddisp.pl?gene=TIMM44
GC08M030762	3.32	https://www.genecards.org/cgi-bin/carddisp.pl?gene=PPP2CB
GC12P122632	3.32	https://www.genecards.org/cgi-bin/carddisp.pl?gene=P2RX4
GC06M031452	3.32	https://www.genecards.org/cgi-bin/carddisp.pl?gene=DHFRP2
GC01M023557	3.31	https://www.genecards.org/cgi-bin/carddisp.pl?gene=ID3
GC10M102106	3.31	https://www.genecards.org/cgi-bin/carddisp.pl?gene=LDB1
GC19M049976	3.31	https://www.genecards.org/cgi-bin/carddisp.pl?gene=VRK3
GC17P073232	3.31	https://www.genecards.org/cgi-bin/carddisp.pl?gene=C17orf80
GC16M071468	3.31	https://www.genecards.org/cgi-bin/carddisp.pl?gene=ZNF23
GC01P019251	3.31	https://www.genecards.org/cgi-bin/carddisp.pl?gene=MRTO4
GC10P063521	3.31	https://www.genecards.org/cgi-bin/carddisp.pl?gene=REEP3
GC12M120096	3.31	https://www.genecards.org/cgi-bin/carddisp.pl?gene=RAB35
GC14M075053	3.31	https://www.genecards.org/cgi-bin/carddisp.pl?gene=ACYP1
GC22P046762	3.31	https://www.genecards.org/cgi-bin/carddisp.pl?gene=TBC1D22A
GC03M041239	3.3	https://www.genecards.org/cgi-bin/carddisp.pl?gene=ULK4
GC12P119334	3.3	https://www.genecards.org/cgi-bin/carddisp.pl?gene=CCDC60
GC14P104335	3.3	https://www.genecards.org/cgi-bin/carddisp.pl?gene=DIO3
GC10P069182	3.3	https://www.genecards.org/cgi-bin/carddisp.pl?gene=SUPV3L1
GC12M122701	3.3	https://www.genecards.org/cgi-bin/carddisp.pl?gene=HCAR2
GC06P095577	3.3	https://www.genecards.org/cgi-bin/carddisp.pl?gene=MANEA
GC01P212935	3.3	https://www.genecards.org/cgi-bin/carddisp.pl?gene=VASH2
GC0XM047651	3.3	https://www.genecards.org/cgi-bin/carddisp.pl?gene=UXT
GC16P001312	3.3	https://www.genecards.org/cgi-bin/carddisp.pl?gene=UBE2I
GC04M074744	3.3	https://www.genecards.org/cgi-bin/carddisp.pl?gene=BTC
GC19M012699	3.3	https://www.genecards.org/cgi-bin/carddisp.pl?gene=TNPO2
GC02M175180	3.29	https://www.genecards.org/cgi-bin/carddisp.pl?gene=ATP5MC3
GC01M162824	3.29	https://www.genecards.org/cgi-bin/carddisp.pl?gene=CCDC190
GC11P022626	3.29	https://www.genecards.org/cgi-bin/carddisp.pl?gene=GAS2
GC01M177923	3.29	https://www.genecards.org/cgi-bin/carddisp.pl?gene=SEC16B
GC07M027525	3.29	https://www.genecards.org/cgi-bin/carddisp.pl?gene=HIBADH
GC01P026410	3.28	https://www.genecards.org/cgi-bin/carddisp.pl?gene=LIN28A
GC03P052794	3.28	https://www.genecards.org/cgi-bin/carddisp.pl?gene=ITIH3
GC07P038218	3.28	https://www.genecards.org/cgi-bin/carddisp.pl?gene=STARD3NL
GC02M106094	3.28	https://www.genecards.org/cgi-bin/carddisp.pl?gene=UXS1
GC15M040765	3.28	https://www.genecards.org/cgi-bin/carddisp.pl?gene=DNAJC17
GC09M132725	3.28	https://www.genecards.org/cgi-bin/carddisp.pl?gene=AK8
GC09M113161	3.28	https://www.genecards.org/cgi-bin/carddisp.pl?gene=FKBP15
GC05M062387	3.28	https://www.genecards.org/cgi-bin/carddisp.pl?gene=DIMT1
GC16M084566	3.27	https://www.genecards.org/cgi-bin/carddisp.pl?gene=COTL1

GC16M001795	3.27	https://www.genecards.org/cgi-bin/carddisp.pl?gene=HAGH
GC07P134527	3.27	https://www.genecards.org/cgi-bin/carddisp.pl?gene=AKR1B10
GC14P049895	3.27	https://www.genecards.org/cgi-bin/carddisp.pl?gene=ARF6
GC0XP102125	3.27	https://www.genecards.org/cgi-bin/carddisp.pl?gene=TCEAL2
GC04M006322	3.26	https://www.genecards.org/cgi-bin/carddisp.pl?gene=PPP2R2C
GC09M101569	3.26	https://www.genecards.org/cgi-bin/carddisp.pl?gene=GRIN3A
GC07M075986	3.26	https://www.genecards.org/cgi-bin/carddisp.pl?gene=TMEM120A
GC02M189746	3.26	https://www.genecards.org/cgi-bin/carddisp.pl?gene=OSGEPL1
GC06M167358	3.26	https://www.genecards.org/cgi-bin/carddisp.pl?gene=TCP10
GC20P036573	3.26	https://www.genecards.org/cgi-bin/carddisp.pl?gene=TGIF2
GC05P055308	3.26	https://www.genecards.org/cgi-bin/carddisp.pl?gene=MTREX
GC17M015977	3.25	https://www.genecards.org/cgi-bin/carddisp.pl?gene=ZSWIM7
GC19P004639	3.25	https://www.genecards.org/cgi-bin/carddisp.pl?gene=TNFAIP8L1
GC07P001531	3.25	https://www.genecards.org/cgi-bin/carddisp.pl?gene=MAFK
GC08M124550	3.25	https://www.genecards.org/cgi-bin/carddisp.pl?gene=MTSS1
GC07M099027	3.25	https://www.genecards.org/cgi-bin/carddisp.pl?gene=SMURF1
GC08M053966	3.25	https://www.genecards.org/cgi-bin/carddisp.pl?gene=TCEA1
GC13M031134	3.25	https://www.genecards.org/cgi-bin/carddisp.pl?gene=HSPH1
GC02P100985	3.25	https://www.genecards.org/cgi-bin/carddisp.pl?gene=RPL31
GC16P070755	3.25	https://www.genecards.org/cgi-bin/carddisp.pl?gene=VAC14-AS1
GC01M111439	3.25	https://www.genecards.org/cgi-bin/carddisp.pl?gene=WDR77
GC16M058113	3.24	https://www.genecards.org/cgi-bin/carddisp.pl?gene=CFAP20
GC01M227920	3.24	https://www.genecards.org/cgi-bin/carddisp.pl?gene=WNT9A
GC02P238231	3.24	https://www.genecards.org/cgi-bin/carddisp.pl?gene=LOC643387
GC14M090847	3.24	https://www.genecards.org/cgi-bin/carddisp.pl?gene=RPS6KA5
GC02P231707	3.24	https://www.genecards.org/cgi-bin/carddisp.pl?gene=PTMA
GC04M024519	3.24	https://www.genecards.org/cgi-bin/carddisp.pl?gene=DHX15
GC22P038705	3.24	https://www.genecards.org/cgi-bin/carddisp.pl?gene=GTPBP1
GC15M082543	3.24	https://www.genecards.org/cgi-bin/carddisp.pl?gene=CPEB1
GC02P143070	3.24	https://www.genecards.org/cgi-bin/carddisp.pl?gene=ARHGAP15
GC08P011769	3.24	https://www.genecards.org/cgi-bin/carddisp.pl?gene=NEIL2
GC06M108986	3.23	https://www.genecards.org/cgi-bin/carddisp.pl?gene=SESN1
GC12P132687	3.23	https://www.genecards.org/cgi-bin/carddisp.pl?gene=PXMP2
GC04P082430	3.23	https://www.genecards.org/cgi-bin/carddisp.pl?gene=ENOPH1
GC10U902689	3.23	https://www.genecards.org/cgi-bin/carddisp.pl?gene=LOC107832851
GC16P087384	3.23	https://www.genecards.org/cgi-bin/carddisp.pl?gene=MAP1LC3B
GC18M049488	3.23	https://www.genecards.org/cgi-bin/carddisp.pl?gene=RPL17
GC15P071840	3.23	https://www.genecards.org/cgi-bin/carddisp.pl?gene=GLCE
GC18M050266	3.23	https://www.genecards.org/cgi-bin/carddisp.pl?gene=MBD1
GC06P046007	3.22	https://www.genecards.org/cgi-bin/carddisp.pl?gene=TSBP1-AS1
GC08M056957	3.22	https://www.genecards.org/cgi-bin/carddisp.pl?gene=IMPAD1
GC04M138164	3.22	https://www.genecards.org/cgi-bin/carddisp.pl?gene=SLC7A11
GC07P150568	3.22	https://www.genecards.org/cgi-bin/carddisp.pl?gene=GIMAP4
GC10M097356	3.21	https://www.genecards.org/cgi-bin/carddisp.pl?gene=RRP12
GC11P012398	3.21	https://www.genecards.org/cgi-bin/carddisp.pl?gene=PARVA
GC12P053499	3.21	https://www.genecards.org/cgi-bin/carddisp.pl?gene=TARBP2
GC16P030651	3.21	https://www.genecards.org/cgi-bin/carddisp.pl?gene=PRR14

GC14M024439	3.21	https://www.genecards.org/cgi-bin/carddisp.pl?gene=SDR39U1
GC03M197521	3.2	https://www.genecards.org/cgi-bin/carddisp.pl?gene=BDH1
GC17P051165	3.2	https://www.genecards.org/cgi-bin/carddisp.pl?gene=NME2
GC16P076278	3.2	https://www.genecards.org/cgi-bin/carddisp.pl?gene=CNTNAP4
GC17P007948	3.2	https://www.genecards.org/cgi-bin/carddisp.pl?gene=ATP1B2
GC08M135623	3.2	https://www.genecards.org/cgi-bin/carddisp.pl?gene=MAPRE1P1
GC0XM108084	3.2	https://www.genecards.org/cgi-bin/carddisp.pl?gene=PSMD10
GC05M056504	3.2	https://www.genecards.org/cgi-bin/carddisp.pl?gene=RPL26P19
GC01M207044	3.19	https://www.genecards.org/cgi-bin/carddisp.pl?gene=YOD1
GC12P062260	3.19	https://www.genecards.org/cgi-bin/carddisp.pl?gene=USP15
GC06M087674	3.19	https://www.genecards.org/cgi-bin/carddisp.pl?gene=AKIRIN2
GC09M131860	3.19	https://www.genecards.org/cgi-bin/carddisp.pl?gene=MED27
GC10P014790	3.19	https://www.genecards.org/cgi-bin/carddisp.pl?gene=HSPA14
GC19P042068	3.19	https://www.genecards.org/cgi-bin/carddisp.pl?gene=ZNF574
GC16P010743	3.18	https://www.genecards.org/cgi-bin/carddisp.pl?gene=NUBP1
GC08M098372	3.18	https://www.genecards.org/cgi-bin/carddisp.pl?gene=STK3
GC12M076354	3.18	https://www.genecards.org/cgi-bin/carddisp.pl?gene=OSBPL8
GC19M041413	3.18	https://www.genecards.org/cgi-bin/carddisp.pl?gene=CLIP3
GC01M202878	3.18	https://www.genecards.org/cgi-bin/carddisp.pl?gene=RABIF
GC09P128818	3.17	https://www.genecards.org/cgi-bin/carddisp.pl?gene=ENDOG
GC01P011143	3.17	https://www.genecards.org/cgi-bin/carddisp.pl?gene=MTOR-AS1
GC02M028814	3.17	https://www.genecards.org/cgi-bin/carddisp.pl?gene=TRMT61B
GC01M155262	3.17	https://www.genecards.org/cgi-bin/carddisp.pl?gene=CLK2
GC01M193147	3.17	https://www.genecards.org/cgi-bin/carddisp.pl?gene=B3GALT2
GC06P142147	3.17	https://www.genecards.org/cgi-bin/carddisp.pl?gene=VTA1
GC07M073736	3.17	https://www.genecards.org/cgi-bin/carddisp.pl?gene=ABHD11
GC07M149003	3.17	https://www.genecards.org/cgi-bin/carddisp.pl?gene=PDIA4
GC06P031971	3.16	https://www.genecards.org/cgi-bin/carddisp.pl?gene=STK19
GC02P058261	3.16	https://www.genecards.org/cgi-bin/carddisp.pl?gene=EIF3FP3
GC17P048830	3.15	https://www.genecards.org/cgi-bin/carddisp.pl?gene=CALCOCO2
GC11P016613	3.15	https://www.genecards.org/cgi-bin/carddisp.pl?gene=C11orf58
GC01M201484	3.15	https://www.genecards.org/cgi-bin/carddisp.pl?gene=CSRP1
GC17M050133	3.15	https://www.genecards.org/cgi-bin/carddisp.pl?gene=PPP1R9B
GC01M183248	3.15	https://www.genecards.org/cgi-bin/carddisp.pl?gene=NMNAT2
GC19M001597	3.15	https://www.genecards.org/cgi-bin/carddisp.pl?gene=UQCR11
GC01M153606	3.15	https://www.genecards.org/cgi-bin/carddisp.pl?gene=S100A16
GC07M019184	3.15	https://www.genecards.org/cgi-bin/carddisp.pl?gene=FERD3L
GC09M136941	3.14	https://www.genecards.org/cgi-bin/carddisp.pl?gene=FBXW5
GC04M046836	3.14	https://www.genecards.org/cgi-bin/carddisp.pl?gene=GABRA4
GC09P134135	3.14	https://www.genecards.org/cgi-bin/carddisp.pl?gene=WDR5
GC21P025734	3.14	https://www.genecards.org/cgi-bin/carddisp.pl?gene=GABPA
GC01P172389	3.14	https://www.genecards.org/cgi-bin/carddisp.pl?gene=C1orf105
GC22P035299	3.14	https://www.genecards.org/cgi-bin/carddisp.pl?gene=TOM1
GC19M048795	3.14	https://www.genecards.org/cgi-bin/carddisp.pl?gene=BCAT2
GC09P027514	3.14	https://www.genecards.org/cgi-bin/carddisp.pl?gene=IFNK
GC06M028286	3.14	https://www.genecards.org/cgi-bin/carddisp.pl?gene=H1-5
GC14M046839	3.13	https://www.genecards.org/cgi-bin/carddisp.pl?gene=MDGA2

GC02P233356	3.13	https://www.genecards.org/cgi-bin/carddisp.pl?gene=DGKD
GC0XP080336	3.13	https://www.genecards.org/cgi-bin/carddisp.pl?gene=TENT5D
GC13P052455	3.13	https://www.genecards.org/cgi-bin/carddisp.pl?gene=CKAP2
GC02M010774	3.13	https://www.genecards.org/cgi-bin/carddisp.pl?gene=PDIA6
GC06M044189	3.13	https://www.genecards.org/cgi-bin/carddisp.pl?gene=MRPL14
GC01P062436	3.13	https://www.genecards.org/cgi-bin/carddisp.pl?gene=USP1
GC07P150722	3.12	https://www.genecards.org/cgi-bin/carddisp.pl?gene=GIMAP5
GC03M065330	3.12	https://www.genecards.org/cgi-bin/carddisp.pl?gene=MAGI1
GC01M157121	3.12	https://www.genecards.org/cgi-bin/carddisp.pl?gene=ETV3
GC12M082223	3.12	https://www.genecards.org/cgi-bin/carddisp.pl?gene=CCDC59
GC14M024265	3.12	https://www.genecards.org/cgi-bin/carddisp.pl?gene=RABGGTA
GC11M068908	3.11	https://www.genecards.org/cgi-bin/carddisp.pl?gene=MRPL21
GC05M136081	3.11	https://www.genecards.org/cgi-bin/carddisp.pl?gene=VTRNA2-1
GC20P045812	3.11	https://www.genecards.org/cgi-bin/carddisp.pl?gene=UBE2C
GC11P066632	3.11	https://www.genecards.org/cgi-bin/carddisp.pl?gene=RBM14
GC06P033377	3.11	https://www.genecards.org/cgi-bin/carddisp.pl?gene=TCF19
GC11M066346	3.11	https://www.genecards.org/cgi-bin/carddisp.pl?gene=BRMS1
GC11P000236	3.11	https://www.genecards.org/cgi-bin/carddisp.pl?gene=PSMD13
GC20M045841	3.11	https://www.genecards.org/cgi-bin/carddisp.pl?gene=ACOT8
GC09M068536	3.1	https://www.genecards.org/cgi-bin/carddisp.pl?gene=TMEM252
GC14P073492	3.1	https://www.genecards.org/cgi-bin/carddisp.pl?gene=RIOX1
GC15P096783	3.1	https://www.genecards.org/cgi-bin/carddisp.pl?gene=SPATA8
GC01M153618	3.1	https://www.genecards.org/cgi-bin/carddisp.pl?gene=S100A13
GC11M123724	3.1	https://www.genecards.org/cgi-bin/carddisp.pl?gene=ZNF202
GC06M032375	3.1	https://www.genecards.org/cgi-bin/carddisp.pl?gene=CLIC1
GC17P000783	3.1	https://www.genecards.org/cgi-bin/carddisp.pl?gene=MRM3
GC07P127652	3.1	https://www.genecards.org/cgi-bin/carddisp.pl?gene=SND1
GC20P052972	3.1	https://www.genecards.org/cgi-bin/carddisp.pl?gene=TSHZ2
GC01P023960	3.1	https://www.genecards.org/cgi-bin/carddisp.pl?gene=PNRC2
GC19M006413	3.09	https://www.genecards.org/cgi-bin/carddisp.pl?gene=KHSRP
GC14P093348	3.09	https://www.genecards.org/cgi-bin/carddisp.pl?gene=COX8C
GC02P113889	3.09	https://www.genecards.org/cgi-bin/carddisp.pl?gene=ACTR3
GC04M113452	3.09	https://www.genecards.org/cgi-bin/carddisp.pl?gene=CAMK2D
GC17M043766	3.09	https://www.genecards.org/cgi-bin/carddisp.pl?gene=DUSP3
GC09M034610	3.09	https://www.genecards.org/cgi-bin/carddisp.pl?gene=RPP25L
GC19M018306	3.09	https://www.genecards.org/cgi-bin/carddisp.pl?gene=LSM4
GC06M041323	3.09	https://www.genecards.org/cgi-bin/carddisp.pl?gene=KCNK5
GC06M032406	3.09	https://www.genecards.org/cgi-bin/carddisp.pl?gene=GPSM3
GC15P090933	3.09	https://www.genecards.org/cgi-bin/carddisp.pl?gene=UNC45A
GC17M033013	3.08	https://www.genecards.org/cgi-bin/carddisp.pl?gene=ASIC2
GC04M170060	3.08	https://www.genecards.org/cgi-bin/carddisp.pl?gene=AADAT
GC01P230057	3.08	https://www.genecards.org/cgi-bin/carddisp.pl?gene=GALNT2
GC17M029573	3.08	https://www.genecards.org/cgi-bin/carddisp.pl?gene=GIT1
GC03M128462	3.08	https://www.genecards.org/cgi-bin/carddisp.pl?gene=DNAJB8
GC15P075648	3.08	https://www.genecards.org/cgi-bin/carddisp.pl?gene=SNX33
GC02P061033	3.08	https://www.genecards.org/cgi-bin/carddisp.pl?gene=KIAA1841
GC14M063371	3.08	https://www.genecards.org/cgi-bin/carddisp.pl?gene=PPP2R5E

GC16M087882	3.07	https://www.genecards.org/cgi-bin/carddisp.pl?gene=CA5A
GC19M038251	3.07	https://www.genecards.org/cgi-bin/carddisp.pl?gene=PPP1R14A
GC14P075064	3.07	https://www.genecards.org/cgi-bin/carddisp.pl?gene=ZC2HC1C
GC03M058568	3.07	https://www.genecards.org/cgi-bin/carddisp.pl?gene=FAM107A
GC15P077420	3.07	https://www.genecards.org/cgi-bin/carddisp.pl?gene=HMG20A
GC03P057556	3.07	https://www.genecards.org/cgi-bin/carddisp.pl?gene=PDE12
GC15M041503	3.07	https://www.genecards.org/cgi-bin/carddisp.pl?gene=LTK
GC0XP120362	3.07	https://www.genecards.org/cgi-bin/carddisp.pl?gene=ATP1B4
GC05M135617	3.06	https://www.genecards.org/cgi-bin/carddisp.pl?gene=CXCL14
GC19P018173	3.06	https://www.genecards.org/cgi-bin/carddisp.pl?gene=IFI30
GC09M089005	3.06	https://www.genecards.org/cgi-bin/carddisp.pl?gene=SHC3
GC19M015380	3.06	https://www.genecards.org/cgi-bin/carddisp.pl?gene=AKAP8L
GC04M082352	3.06	https://www.genecards.org/cgi-bin/carddisp.pl?gene=HNRNPD
GC18M068673	3.05	https://www.genecards.org/cgi-bin/carddisp.pl?gene=TMX3
GC14M046651	3.05	https://www.genecards.org/cgi-bin/carddisp.pl?gene=RPL10L
GC02P046698	3.05	https://www.genecards.org/cgi-bin/carddisp.pl?gene=SOCS5
GC12M056667	3.05	https://www.genecards.org/cgi-bin/carddisp.pl?gene=PTGES3
GC02M089103	3.05	https://www.genecards.org/cgi-bin/carddisp.pl?gene=IGKV1-5
GC11P066050	3.05	https://www.genecards.org/cgi-bin/carddisp.pl?gene=SF3B2
GC17M050367	3.04	https://www.genecards.org/cgi-bin/carddisp.pl?gene=MRPL27
GC02M231558	3.04	https://www.genecards.org/cgi-bin/carddisp.pl?gene=NMUR1
GC11M000456	3.04	https://www.genecards.org/cgi-bin/carddisp.pl?gene=SIGIRR
GC02M037610	3.04	https://www.genecards.org/cgi-bin/carddisp.pl?gene=CDC42EP3
GC05P017065	3.04	https://www.genecards.org/cgi-bin/carddisp.pl?gene=BASP1
GC01M056495	3.04	https://www.genecards.org/cgi-bin/carddisp.pl?gene=PLPP3
GC06P063521	3.04	https://www.genecards.org/cgi-bin/carddisp.pl?gene=PTP4A1
GC07P090470	3.03	https://www.genecards.org/cgi-bin/carddisp.pl?gene=CDK14
GC19P022232	3.03	https://www.genecards.org/cgi-bin/carddisp.pl?gene=SLC27A1
GC01M041506	3.03	https://www.genecards.org/cgi-bin/carddisp.pl?gene=HIVEP3
GC16P031479	3.03	https://www.genecards.org/cgi-bin/carddisp.pl?gene=TGFB1I1
GC08M101704	3.03	https://www.genecards.org/cgi-bin/carddisp.pl?gene=NCALD
GC02M037201	3.02	https://www.genecards.org/cgi-bin/carddisp.pl?gene=CEBPZ
GC12P050085	3.02	https://www.genecards.org/cgi-bin/carddisp.pl?gene=SMARCD1
GC06M149658	3.02	https://www.genecards.org/cgi-bin/carddisp.pl?gene=LATS1
GC15P043593	3.02	https://www.genecards.org/cgi-bin/carddisp.pl?gene=CKMT1B
GC05P086617	3.01	https://www.genecards.org/cgi-bin/carddisp.pl?gene=COX7C
GC21M029055	3.01	https://www.genecards.org/cgi-bin/carddisp.pl?gene=CCT8
GC01P153728	3.01	https://www.genecards.org/cgi-bin/carddisp.pl?gene=INTS3
GC10P014954	3.01	https://www.genecards.org/cgi-bin/carddisp.pl?gene=MEIG1
GC10M073183	3.01	https://www.genecards.org/cgi-bin/carddisp.pl?gene=DNAJC9
GC20P033490	3.01	https://www.genecards.org/cgi-bin/carddisp.pl?gene=CBFA2T2
GC02M096184	3	https://www.genecards.org/cgi-bin/carddisp.pl?gene=STARD7
GC19P000926	3	https://www.genecards.org/cgi-bin/carddisp.pl?gene=ARID3A
GC01M027902	3	https://www.genecards.org/cgi-bin/carddisp.pl?gene=RPA2
GC02M163593	3	https://www.genecards.org/cgi-bin/carddisp.pl?gene=FIGN
GC02P065227	3	https://www.genecards.org/cgi-bin/carddisp.pl?gene=ACTR2
GC01M036339	3	https://www.genecards.org/cgi-bin/carddisp.pl?gene=STK40

GC0XP015392	3	https://www.genecards.org/cgi-bin/carddisp.pl?gene=BMX
GC03M196347	3	https://www.genecards.org/cgi-bin/carddisp.pl?gene=UBXN7
GC02P161136	3	https://www.genecards.org/cgi-bin/carddisp.pl?gene=TANK
GC09P093058	3	https://www.genecards.org/cgi-bin/carddisp.pl?gene=SUSD3
GC02M222044	2.99	https://www.genecards.org/cgi-bin/carddisp.pl?gene=RPL23AP28
GC06P084024	2.99	https://www.genecards.org/cgi-bin/carddisp.pl?gene=MRAP2
GC01P075786	2.99	https://www.genecards.org/cgi-bin/carddisp.pl?gene=RABGGTB
GC20P037521	2.99	https://www.genecards.org/cgi-bin/carddisp.pl?gene=NNAT
GC17P059565	2.99	https://www.genecards.org/cgi-bin/carddisp.pl?gene=DHX40
GC15M078167	2.99	https://www.genecards.org/cgi-bin/carddisp.pl?gene=ACSBG1
GC18M046808	2.98	https://www.genecards.org/cgi-bin/carddisp.pl?gene=PIAS2
GC01P182758	2.98	https://www.genecards.org/cgi-bin/carddisp.pl?gene=NPL
GC17M004669	2.98	https://www.genecards.org/cgi-bin/carddisp.pl?gene=PELP1
GC03P184186	2.98	https://www.genecards.org/cgi-bin/carddisp.pl?gene=ABCF3
GC03M097942	2.98	https://www.genecards.org/cgi-bin/carddisp.pl?gene=RIOX2
GC19P003572	2.98	https://www.genecards.org/cgi-bin/carddisp.pl?gene=HMG20B
GC03M067358	2.98	https://www.genecards.org/cgi-bin/carddisp.pl?gene=SUCLG2
GC19P049931	2.98	https://www.genecards.org/cgi-bin/carddisp.pl?gene=ATF5
GC01M043450	2.97	https://www.genecards.org/cgi-bin/carddisp.pl?gene=HYI
GC16P084368	2.97	https://www.genecards.org/cgi-bin/carddisp.pl?gene=ATP2C2
GC0XP070133	2.97	https://www.genecards.org/cgi-bin/carddisp.pl?gene=IGBP1
GC12P112978	2.97	https://www.genecards.org/cgi-bin/carddisp.pl?gene=OAS2
GC14M022902	2.97	https://www.genecards.org/cgi-bin/carddisp.pl?gene=RBM23
GC06P057046	2.97	https://www.genecards.org/cgi-bin/carddisp.pl?gene=KIAA1586
GC17M076038	2.97	https://www.genecards.org/cgi-bin/carddisp.pl?gene=SRP68
GC20P062877	2.97	https://www.genecards.org/cgi-bin/carddisp.pl?gene=MIR133A2
GC01P007765	2.97	https://www.genecards.org/cgi-bin/carddisp.pl?gene=VAMP3
GC14M023356	2.97	https://www.genecards.org/cgi-bin/carddisp.pl?gene=EFS
GC16P031033	2.96	https://www.genecards.org/cgi-bin/carddisp.pl?gene=STX4
GC16P055566	2.96	https://www.genecards.org/cgi-bin/carddisp.pl?gene=CAPNS2
GC19M011322	2.96	https://www.genecards.org/cgi-bin/carddisp.pl?gene=RAB3D
GC01P117929	2.96	https://www.genecards.org/cgi-bin/carddisp.pl?gene=WDR3
GC19P000983	2.96	https://www.genecards.org/cgi-bin/carddisp.pl?gene=WDR18
GC13M112485	2.96	https://www.genecards.org/cgi-bin/carddisp.pl?gene=TUBGCP3
GC12M079773	2.96	https://www.genecards.org/cgi-bin/carddisp.pl?gene=PPP1R12A
GC06P146543	2.96	https://www.genecards.org/cgi-bin/carddisp.pl?gene=RAB32
GC09M035554	2.95	https://www.genecards.org/cgi-bin/carddisp.pl?gene=FAM166B
GC17P028319	2.95	https://www.genecards.org/cgi-bin/carddisp.pl?gene=TMEM97
GC01P155023	2.95	https://www.genecards.org/cgi-bin/carddisp.pl?gene=ADAM15
GC02P203238	2.95	https://www.genecards.org/cgi-bin/carddisp.pl?gene=CYP20A1
GC19P032974	2.95	https://www.genecards.org/cgi-bin/carddisp.pl?gene=FAAP24
GC06M005997	2.94	https://www.genecards.org/cgi-bin/carddisp.pl?gene=NRN1
GC13P111316	2.94	https://www.genecards.org/cgi-bin/carddisp.pl?gene=TEX29
GC19P007888	2.94	https://www.genecards.org/cgi-bin/carddisp.pl?gene=LRRC8E
GC15M065117	2.94	https://www.genecards.org/cgi-bin/carddisp.pl?gene=PDCD7
GC01M046607	2.94	https://www.genecards.org/cgi-bin/carddisp.pl?gene=MOB3C
GC17M019841	2.94	https://www.genecards.org/cgi-bin/carddisp.pl?gene=ULK2

GC09P089605	2.94	https://www.genecards.org/cgi-bin/carddisp.pl?gene=GADD45G
GC07P100367	2.94	https://www.genecards.org/cgi-bin/carddisp.pl?gene=PILRA
GC01P211326	2.94	https://www.genecards.org/cgi-bin/carddisp.pl?gene=TRAF5
GC03M038103	2.94	https://www.genecards.org/cgi-bin/carddisp.pl?gene=ACAA1
GC17P017042	2.94	https://www.genecards.org/cgi-bin/carddisp.pl?gene=MPRIP
GC13M035473	2.94	https://www.genecards.org/cgi-bin/carddisp.pl?gene=MAB21L1
GC01P205504	2.94	https://www.genecards.org/cgi-bin/carddisp.pl?gene=CDK18
GC07M098292	2.94	https://www.genecards.org/cgi-bin/carddisp.pl?gene=BAIAP2L1
GC09P122750	2.93	https://www.genecards.org/cgi-bin/carddisp.pl?gene=OR1L6
GC17M028346	2.93	https://www.genecards.org/cgi-bin/carddisp.pl?gene=POLDIP2
GC11M061130	2.93	https://www.genecards.org/cgi-bin/carddisp.pl?gene=VPS37C
GC03M172630	2.93	https://www.genecards.org/cgi-bin/carddisp.pl?gene=NCEH1
GC03P174438	2.93	https://www.genecards.org/cgi-bin/carddisp.pl?gene=NAALADL2
GC20P003917	2.93	https://www.genecards.org/cgi-bin/carddisp.pl?gene=MIR103A2
GC17P029391	2.93	https://www.genecards.org/cgi-bin/carddisp.pl?gene=TAOK1
GC01M156009	2.93	https://www.genecards.org/cgi-bin/carddisp.pl?gene=SSR2
GC01M159918	2.92	https://www.genecards.org/cgi-bin/carddisp.pl?gene=TAGLN2
GC16P030050	2.92	https://www.genecards.org/cgi-bin/carddisp.pl?gene=PAGR1
GC09M033451	2.92	https://www.genecards.org/cgi-bin/carddisp.pl?gene=NOL6
GC06M008413	2.92	https://www.genecards.org/cgi-bin/carddisp.pl?gene=SLC35B3
GC09M072351	2.92	https://www.genecards.org/cgi-bin/carddisp.pl?gene=ZFAND5
GC20M001442	2.92	https://www.genecards.org/cgi-bin/carddisp.pl?gene=NSFL1C
GC02P073986	2.92	https://www.genecards.org/cgi-bin/carddisp.pl?gene=TET3
GC04M137518	2.92	https://www.genecards.org/cgi-bin/carddisp.pl?gene=PCDH18
GC08P086514	2.92	https://www.genecards.org/cgi-bin/carddisp.pl?gene=CPNE3
GC01M150190	2.92	https://www.genecards.org/cgi-bin/carddisp.pl?gene=ANP32E
GC19M041507	2.92	https://www.genecards.org/cgi-bin/carddisp.pl?gene=COX7A1
GC19M046760	2.92	https://www.genecards.org/cgi-bin/carddisp.pl?gene=SLC1A5
GC12P050392	2.92	https://www.genecards.org/cgi-bin/carddisp.pl?gene=LARP4
GC10P013161	2.92	https://www.genecards.org/cgi-bin/carddisp.pl?gene=MCM10
GC06M001624	2.91	https://www.genecards.org/cgi-bin/carddisp.pl?gene=GMDS
GC17P045222	2.91	https://www.genecards.org/cgi-bin/carddisp.pl?gene=FMNL1
GC12M055902	2.91	https://www.genecards.org/cgi-bin/carddisp.pl?gene=PYM1
GC19M004641	2.91	https://www.genecards.org/cgi-bin/carddisp.pl?gene=MYDGF
GC0XP051743	2.91	https://www.genecards.org/cgi-bin/carddisp.pl?gene=GSPT2
GC01M001216	2.91	https://www.genecards.org/cgi-bin/carddisp.pl?gene=SDF4
GC22M042583	2.91	https://www.genecards.org/cgi-bin/carddisp.pl?gene=POLDIP3
GC18M003488	2.91	https://www.genecards.org/cgi-bin/carddisp.pl?gene=DLGAP1
GC02M143938	2.91	https://www.genecards.org/cgi-bin/carddisp.pl?gene=GTDC1
GC07P030134	2.91	https://www.genecards.org/cgi-bin/carddisp.pl?gene=MTURN
GC02M241072	2.9	https://www.genecards.org/cgi-bin/carddisp.pl?gene=MTERF4
GC01M084811	2.9	https://www.genecards.org/cgi-bin/carddisp.pl?gene=LPAR3
GC10M044414	2.9	https://www.genecards.org/cgi-bin/carddisp.pl?gene=RPL9P21
GC01M006179	2.9	https://www.genecards.org/cgi-bin/carddisp.pl?gene=RPL22
GC08P038176	2.9	https://www.genecards.org/cgi-bin/carddisp.pl?gene=BAG4
GC02P069644	2.9	https://www.genecards.org/cgi-bin/carddisp.pl?gene=ANXA4
GC11P000696	2.9	https://www.genecards.org/cgi-bin/carddisp.pl?gene=EPS8L2

GC06P030571	2.9	https://www.genecards.org/cgi-bin/carddisp.pl?gene=ABCF1
GC01M015943	2.9	https://www.genecards.org/cgi-bin/carddisp.pl?gene=ZBTB17
GC06M026044	2.9	https://www.genecards.org/cgi-bin/carddisp.pl?gene=H2BC3
GC09M121444	2.89	https://www.genecards.org/cgi-bin/carddisp.pl?gene=GGTA1P
GC17P007932	2.89	https://www.genecards.org/cgi-bin/carddisp.pl?gene=CNTROB
GC17P008247	2.89	https://www.genecards.org/cgi-bin/carddisp.pl?gene=PFAS
GC16M001068	2.89	https://www.genecards.org/cgi-bin/carddisp.pl?gene=WDR24
GC16M029992	2.89	https://www.genecards.org/cgi-bin/carddisp.pl?gene=HIRIP3
GC02M241492	2.88	https://www.genecards.org/cgi-bin/carddisp.pl?gene=STK25
GC01M169821	2.88	https://www.genecards.org/cgi-bin/carddisp.pl?gene=SCYL3
GC05P173144	2.88	https://www.genecards.org/cgi-bin/carddisp.pl?gene=BNIP1
GC12P069585	2.88	https://www.genecards.org/cgi-bin/carddisp.pl?gene=CCT2
GC12P047753	2.88	https://www.genecards.org/cgi-bin/carddisp.pl?gene=SLC48A1
GC09P001003	2.88	https://www.genecards.org/cgi-bin/carddisp.pl?gene=DMRT2
GC22P042079	2.88	https://www.genecards.org/cgi-bin/carddisp.pl?gene=SMDT1
GC02P218217	2.88	https://www.genecards.org/cgi-bin/carddisp.pl?gene=ARPC2
GC10P102152	2.88	https://www.genecards.org/cgi-bin/carddisp.pl?gene=NOLC1
GC09M005756	2.88	https://www.genecards.org/cgi-bin/carddisp.pl?gene=ERMP1
GC06P028438	2.88	https://www.genecards.org/cgi-bin/carddisp.pl?gene=ZKSCAN8
GC09M035104	2.87	https://www.genecards.org/cgi-bin/carddisp.pl?gene=FAM214B
GC20P036541	2.87	https://www.genecards.org/cgi-bin/carddisp.pl?gene=MYL9
GC03P155870	2.87	https://www.genecards.org/cgi-bin/carddisp.pl?gene=GMPS
GC02M011434	2.87	https://www.genecards.org/cgi-bin/carddisp.pl?gene=E2F6
GC01P028369	2.87	https://www.genecards.org/cgi-bin/carddisp.pl?gene=PHACTR4
GC01M206571	2.87	https://www.genecards.org/cgi-bin/carddisp.pl?gene=EIF2D
GC05M139390	2.87	https://www.genecards.org/cgi-bin/carddisp.pl?gene=PROB1
GC07P055957	2.87	https://www.genecards.org/cgi-bin/carddisp.pl?gene=MRPS17
GC17P048908	2.87	https://www.genecards.org/cgi-bin/carddisp.pl?gene=UBE2Z
GC07M036519	2.87	https://www.genecards.org/cgi-bin/carddisp.pl?gene=AOAH
GC11P112075	2.87	https://www.genecards.org/cgi-bin/carddisp.pl?gene=NKAPD1
GC20M035278	2.87	https://www.genecards.org/cgi-bin/carddisp.pl?gene=EIF6
GC17M063432	2.86	https://www.genecards.org/cgi-bin/carddisp.pl?gene=CYB561
GC17M039173	2.86	https://www.genecards.org/cgi-bin/carddisp.pl?gene=CACNB1
GC08M099960	2.86	https://www.genecards.org/cgi-bin/carddisp.pl?gene=RGS22
GC06P050713	2.86	https://www.genecards.org/cgi-bin/carddisp.pl?gene=TFAP2D
GC15P076931	2.86	https://www.genecards.org/cgi-bin/carddisp.pl?gene=RCN2
GC20M023805	2.86	https://www.genecards.org/cgi-bin/carddisp.pl?gene=CST2
GC07P149239	2.86	https://www.genecards.org/cgi-bin/carddisp.pl?gene=ZNF212
GC07M044829	2.86	https://www.genecards.org/cgi-bin/carddisp.pl?gene=H2AZ2
GC11P069066	2.86	https://www.genecards.org/cgi-bin/carddisp.pl?gene=TPCN2
GC11M059744	2.86	https://www.genecards.org/cgi-bin/carddisp.pl?gene=TYRL
GC06M041281	2.86	https://www.genecards.org/cgi-bin/carddisp.pl?gene=TAF11
GC22M029327	2.86	https://www.genecards.org/cgi-bin/carddisp.pl?gene=AP1B1
GC02P048440	2.86	https://www.genecards.org/cgi-bin/carddisp.pl?gene=PPP1R21
GC10P101571	2.86	https://www.genecards.org/cgi-bin/carddisp.pl?gene=DPCD
GC04M183639	2.86	https://www.genecards.org/cgi-bin/carddisp.pl?gene=RWDD4
GC04P127730	2.86	https://www.genecards.org/cgi-bin/carddisp.pl?gene=SLC25A31

GC17P075013	2.85	https://www.genecards.org/cgi-bin/carddisp.pl?gene=MRPL58
GC15P041283	2.85	https://www.genecards.org/cgi-bin/carddisp.pl?gene=OIP5-AS1
GC03M046037	2.85	https://www.genecards.org/cgi-bin/carddisp.pl?gene=XCR1
GC12P110936	2.85	https://www.genecards.org/cgi-bin/carddisp.pl?gene=LINC01405
GC19P047781	2.85	https://www.genecards.org/cgi-bin/carddisp.pl?gene=SELENOW
GC11P043633	2.85	https://www.genecards.org/cgi-bin/carddisp.pl?gene=MIR129-2
GC03M157146	2.85	https://www.genecards.org/cgi-bin/carddisp.pl?gene=CCNL1
GC05P142825	2.85	https://www.genecards.org/cgi-bin/carddisp.pl?gene=IK
GC11P107928	2.85	https://www.genecards.org/cgi-bin/carddisp.pl?gene=RAB39A
GC03P028490	2.85	https://www.genecards.org/cgi-bin/carddisp.pl?gene=RPL34P11
GC05P134904	2.85	https://www.genecards.org/cgi-bin/carddisp.pl?gene=PCBD2
GC01M120150	2.85	https://www.genecards.org/cgi-bin/carddisp.pl?gene=SEC22B
GC10M114120	2.85	https://www.genecards.org/cgi-bin/carddisp.pl?gene=CCDC186
GC22M029730	2.85	https://www.genecards.org/cgi-bin/carddisp.pl?gene=ZMAT5
GC22M029058	2.85	https://www.genecards.org/cgi-bin/carddisp.pl?gene=C22orf31
GC12M056712	2.85	https://www.genecards.org/cgi-bin/carddisp.pl?gene=NACA
GC21P044940	2.85	https://www.genecards.org/cgi-bin/carddisp.pl?gene=FAM207A
GC06M032555	2.85	https://www.genecards.org/cgi-bin/carddisp.pl?gene=HLA-DRB6
GC11P086791	2.84	https://www.genecards.org/cgi-bin/carddisp.pl?gene=PRSS23
GC02M188733	2.84	https://www.genecards.org/cgi-bin/carddisp.pl?gene=DIRC1
GC12P025959	2.84	https://www.genecards.org/cgi-bin/carddisp.pl?gene=RASSF8
GC11P043702	2.84	https://www.genecards.org/cgi-bin/carddisp.pl?gene=HSD17B12
GC17M075909	2.84	https://www.genecards.org/cgi-bin/carddisp.pl?gene=FBF1
GC03P069084	2.84	https://www.genecards.org/cgi-bin/carddisp.pl?gene=ARL6IP5
GC03M129315	2.84	https://www.genecards.org/cgi-bin/carddisp.pl?gene=H1-10
GC06P033391	2.84	https://www.genecards.org/cgi-bin/carddisp.pl?gene=KIFC1
GC05P081972	2.84	https://www.genecards.org/cgi-bin/carddisp.pl?gene=ATG10
GC15P074598	2.84	https://www.genecards.org/cgi-bin/carddisp.pl?gene=CLK3
GC20P062387	2.84	https://www.genecards.org/cgi-bin/carddisp.pl?gene=RPS21
GC01P008945	2.83	https://www.genecards.org/cgi-bin/carddisp.pl?gene=CA6
GC12M018080	2.83	https://www.genecards.org/cgi-bin/carddisp.pl?gene=RERGL
GC13P032586	2.83	https://www.genecards.org/cgi-bin/carddisp.pl?gene=PDS5B
GC01P013893	2.83	https://www.genecards.org/cgi-bin/carddisp.pl?gene=KAZN
GC06M004115	2.83	https://www.genecards.org/cgi-bin/carddisp.pl?gene=ECI2
GC02P028782	2.83	https://www.genecards.org/cgi-bin/carddisp.pl?gene=SPDYA
GC01M093561	2.83	https://www.genecards.org/cgi-bin/carddisp.pl?gene=BCAR3
GC0XP070290	2.83	https://www.genecards.org/cgi-bin/carddisp.pl?gene=KIF4A
GC09M021304	2.83	https://www.genecards.org/cgi-bin/carddisp.pl?gene=IFNA5
GC15P058138	2.83	https://www.genecards.org/cgi-bin/carddisp.pl?gene=AQP9
GC07P144745	2.82	https://www.genecards.org/cgi-bin/carddisp.pl?gene=TMEM139
GC12P071754	2.82	https://www.genecards.org/cgi-bin/carddisp.pl?gene=RAB21
GC17M081540	2.82	https://www.genecards.org/cgi-bin/carddisp.pl?gene=FAAP100
GC05M170246	2.82	https://www.genecards.org/cgi-bin/carddisp.pl?gene=LCP2
GC10M100273	2.82	https://www.genecards.org/cgi-bin/carddisp.pl?gene=BLOC1S2
GC19M004839	2.82	https://www.genecards.org/cgi-bin/carddisp.pl?gene=PLIN3
GC15P045430	2.81	https://www.genecards.org/cgi-bin/carddisp.pl?gene=C15orf48
GC11M064223	2.81	https://www.genecards.org/cgi-bin/carddisp.pl?gene=TRPT1

GC07P048919	2.81	https://www.genecards.org/cgi-bin/carddisp.pl?gene=CDC14C
GC19P034428	2.81	https://www.genecards.org/cgi-bin/carddisp.pl?gene=UBA2
GC04P078776	2.81	https://www.genecards.org/cgi-bin/carddisp.pl?gene=BMP2K
GC09M128833	2.81	https://www.genecards.org/cgi-bin/carddisp.pl?gene=KYAT1
GC19M014444	2.81	https://www.genecards.org/cgi-bin/carddisp.pl?gene=PTGER1
GC17P018088	2.81	https://www.genecards.org/cgi-bin/carddisp.pl?gene=DRG2
GC05M001851	2.81	https://www.genecards.org/cgi-bin/carddisp.pl?gene=MRPL36
GC02P165469	2.81	https://www.genecards.org/cgi-bin/carddisp.pl?gene=CSRNP3
GC06P033407	2.8	https://www.genecards.org/cgi-bin/carddisp.pl?gene=LY6G5B
GC08P090791	2.8	https://www.genecards.org/cgi-bin/carddisp.pl?gene=NECAB1
GC17M042101	2.8	https://www.genecards.org/cgi-bin/carddisp.pl?gene=DHX58
GC03M132559	2.8	https://www.genecards.org/cgi-bin/carddisp.pl?gene=ACAD11
GC21P043567	2.8	https://www.genecards.org/cgi-bin/carddisp.pl?gene=H2BS1
GC01P066924	2.8	https://www.genecards.org/cgi-bin/carddisp.pl?gene=MIER1
GC21P042974	2.8	https://www.genecards.org/cgi-bin/carddisp.pl?gene=PKNOX1
GC08P022578	2.8	https://www.genecards.org/cgi-bin/carddisp.pl?gene=PDLIM2
GC05M077691	2.79	https://www.genecards.org/cgi-bin/carddisp.pl?gene=TBCA
GC17M042124	2.79	https://www.genecards.org/cgi-bin/carddisp.pl?gene=RAB5C
GC12M106063	2.79	https://www.genecards.org/cgi-bin/carddisp.pl?gene=NUAK1
GC04M075910	2.79	https://www.genecards.org/cgi-bin/carddisp.pl?gene=NAAA
GC14P073567	2.79	https://www.genecards.org/cgi-bin/carddisp.pl?gene=ACOT2
GC16M001770	2.79	https://www.genecards.org/cgi-bin/carddisp.pl?gene=NME3
GC11P017218	2.79	https://www.genecards.org/cgi-bin/carddisp.pl?gene=NUCB2
GC06M042744	2.79	https://www.genecards.org/cgi-bin/carddisp.pl?gene=TBCC
GC15P043516	2.79	https://www.genecards.org/cgi-bin/carddisp.pl?gene=MAP1A
GC17P081704	2.79	https://www.genecards.org/cgi-bin/carddisp.pl?gene=MRPL12
GC03P052777	2.79	https://www.genecards.org/cgi-bin/carddisp.pl?gene=ITIH1
GC09P124777	2.78	https://www.genecards.org/cgi-bin/carddisp.pl?gene=OLFML2A
GC01M156243	2.78	https://www.genecards.org/cgi-bin/carddisp.pl?gene=PAQR6
GC18P011981	2.78	https://www.genecards.org/cgi-bin/carddisp.pl?gene=IMPA2
GC05P070900	2.78	https://www.genecards.org/cgi-bin/carddisp.pl?gene=SERF1A
GC08P064379	2.78	https://www.genecards.org/cgi-bin/carddisp.pl?gene=MIR124-2
GC01P065420	2.78	https://www.genecards.org/cgi-bin/carddisp.pl?gene=LEPROT
GC22M030554	2.78	https://www.genecards.org/cgi-bin/carddisp.pl?gene=GAL3ST1
GC22P031160	2.78	https://www.genecards.org/cgi-bin/carddisp.pl?gene=RNF185
GC16P001248	2.78	https://www.genecards.org/cgi-bin/carddisp.pl?gene=RHOT2
GC0XP153776	2.78	https://www.genecards.org/cgi-bin/carddisp.pl?gene=SRPK3
GC01P220689	2.78	https://www.genecards.org/cgi-bin/carddisp.pl?gene=C1orf115
GC07P120950	2.78	https://www.genecards.org/cgi-bin/carddisp.pl?gene=ING3
GC22P021015	2.77	https://www.genecards.org/cgi-bin/carddisp.pl?gene=P2RX6
GC12P112570	2.77	https://www.genecards.org/cgi-bin/carddisp.pl?gene=RPH3A
GC01M025242	2.77	https://www.genecards.org/cgi-bin/carddisp.pl?gene=RSRP1
GC01P028237	2.77	https://www.genecards.org/cgi-bin/carddisp.pl?gene=ATP5IF1
GC05P000993	2.77	https://www.genecards.org/cgi-bin/carddisp.pl?gene=NKD2
GC02P189441	2.77	https://www.genecards.org/cgi-bin/carddisp.pl?gene=WDR75
GC19M055350	2.77	https://www.genecards.org/cgi-bin/carddisp.pl?gene=COX6B2
GC17P049210	2.77	https://www.genecards.org/cgi-bin/carddisp.pl?gene=ABI3

GC01P171456	2.77	https://www.genecards.org/cgi-bin/carddisp.pl?gene=PRRC2C
GC03P188948	2.77	https://www.genecards.org/cgi-bin/carddisp.pl?gene=TPRG1
GC15P074826	2.77	https://www.genecards.org/cgi-bin/carddisp.pl?gene=CPLX3
GC01P209828	2.76	https://www.genecards.org/cgi-bin/carddisp.pl?gene=UTP25
GC11M063998	2.76	https://www.genecards.org/cgi-bin/carddisp.pl?gene=MACROD1
GC16P078826	2.76	https://www.genecards.org/cgi-bin/carddisp.pl?gene=RPS3P7
GC01P168148	2.76	https://www.genecards.org/cgi-bin/carddisp.pl?gene=TIPRL
GC0XP015688	2.76	https://www.genecards.org/cgi-bin/carddisp.pl?gene=CA5B
GC12M048653	2.76	https://www.genecards.org/cgi-bin/carddisp.pl?gene=KANSL2
GC03M010293	2.76	https://www.genecards.org/cgi-bin/carddisp.pl?gene=SEC13
GC07P149262	2.76	https://www.genecards.org/cgi-bin/carddisp.pl?gene=ZNF783
GC09M113407	2.76	https://www.genecards.org/cgi-bin/carddisp.pl?gene=POLE3
GC10P023053	2.76	https://www.genecards.org/cgi-bin/carddisp.pl?gene=MSRB2
GC01P044739	2.76	https://www.genecards.org/cgi-bin/carddisp.pl?gene=KIF2C
GC09P021409	2.75	https://www.genecards.org/cgi-bin/carddisp.pl?gene=IFNA8
GC07M105110	2.75	https://www.genecards.org/cgi-bin/carddisp.pl?gene=SRPK2
GC13M060636	2.75	https://www.genecards.org/cgi-bin/carddisp.pl?gene=EIF4A1P6
GC03M048744	2.75	https://www.genecards.org/cgi-bin/carddisp.pl?gene=PRKAR2A
GC06M013621	2.75	https://www.genecards.org/cgi-bin/carddisp.pl?gene=RANBP9
GC09P082130	2.75	https://www.genecards.org/cgi-bin/carddisp.pl?gene=RPS2P34
GC01P151057	2.75	https://www.genecards.org/cgi-bin/carddisp.pl?gene=MLLT11
GC02P090420	2.75	https://www.genecards.org/cgi-bin/carddisp.pl?gene=IGKV2D-29
GC02P152717	2.75	https://www.genecards.org/cgi-bin/carddisp.pl?gene=ARL6IP6
GC02P158862	2.75	https://www.genecards.org/cgi-bin/carddisp.pl?gene=OR7E28P
GC05P134524	2.74	https://www.genecards.org/cgi-bin/carddisp.pl?gene=JADE2
GC10M102403	2.74	https://www.genecards.org/cgi-bin/carddisp.pl?gene=PSD
GC11M019203	2.74	https://www.genecards.org/cgi-bin/carddisp.pl?gene=E2F8
GC16P000636	2.74	https://www.genecards.org/cgi-bin/carddisp.pl?gene=MCRIP2
GC0XP096684	2.74	https://www.genecards.org/cgi-bin/carddisp.pl?gene=DIAPH2
GC20M034088	2.74	https://www.genecards.org/cgi-bin/carddisp.pl?gene=EIF2S2
GC01M016197	2.74	https://www.genecards.org/cgi-bin/carddisp.pl?gene=ARHGEF19
GC16P029664	2.74	https://www.genecards.org/cgi-bin/carddisp.pl?gene=QPRT
GC12M048042	2.74	https://www.genecards.org/cgi-bin/carddisp.pl?gene=SENP1
GC01P155745	2.74	https://www.genecards.org/cgi-bin/carddisp.pl?gene=MSTO2P
GC12P112013	2.73	https://www.genecards.org/cgi-bin/carddisp.pl?gene=ERP29
GC14P053612	2.73	https://www.genecards.org/cgi-bin/carddisp.pl?gene=RPS3AP46
GC11P111605	2.73	https://www.genecards.org/cgi-bin/carddisp.pl?gene=SIK2
GC07P128739	2.73	https://www.genecards.org/cgi-bin/carddisp.pl?gene=CALU
GC06M130820	2.73	https://www.genecards.org/cgi-bin/carddisp.pl?gene=EPB41L2
GC0XP015790	2.73	https://www.genecards.org/cgi-bin/carddisp.pl?gene=ZRSR2
GC14P068936	2.72	https://www.genecards.org/cgi-bin/carddisp.pl?gene=BANF1P1
GC11M078016	2.72	https://www.genecards.org/cgi-bin/carddisp.pl?gene=KCTD14
GC09P036127	2.72	https://www.genecards.org/cgi-bin/carddisp.pl?gene=GLIPR2
GC01P109466	2.72	https://www.genecards.org/cgi-bin/carddisp.pl?gene=SYPL2
GC17M065635	2.71	https://www.genecards.org/cgi-bin/carddisp.pl?gene=CEP112
GC10P127907	2.71	https://www.genecards.org/cgi-bin/carddisp.pl?gene=PTPRE
GC04M176184	2.71	https://www.genecards.org/cgi-bin/carddisp.pl?gene=SPATA4

GC03M048688	2.71	https://www.genecards.org/cgi-bin/carddisp.pl?gene=IP6K2
GC01M154957	2.71	https://www.genecards.org/cgi-bin/carddisp.pl?gene=PYGO2
GC10M017098	2.71	https://www.genecards.org/cgi-bin/carddisp.pl?gene=TRDMT1
GC12M106496	2.71	https://www.genecards.org/cgi-bin/carddisp.pl?gene=LOC100287944
GC02M238170	2.7	https://www.genecards.org/cgi-bin/carddisp.pl?gene=ILKAP
GC01M003778	2.7	https://www.genecards.org/cgi-bin/carddisp.pl?gene=LRRC47
GC05M057480	2.7	https://www.genecards.org/cgi-bin/carddisp.pl?gene=ACTBL2
GC19P040191	2.7	https://www.genecards.org/cgi-bin/carddisp.pl?gene=MAP3K10
GC01M183646	2.7	https://www.genecards.org/cgi-bin/carddisp.pl?gene=APOBEC4
GC02P189879	2.7	https://www.genecards.org/cgi-bin/carddisp.pl?gene=C2orf88
GC08P117521	2.7	https://www.genecards.org/cgi-bin/carddisp.pl?gene=MED30
GC05M111496	2.7	https://www.genecards.org/cgi-bin/carddisp.pl?gene=STARD4
GC22M046684	2.7	https://www.genecards.org/cgi-bin/carddisp.pl?gene=CERK
GC04M169730	2.7	https://www.genecards.org/cgi-bin/carddisp.pl?gene=HPF1
GC01M168245	2.7	https://www.genecards.org/cgi-bin/carddisp.pl?gene=ANKRD36BP1
GC16P080540	2.7	https://www.genecards.org/cgi-bin/carddisp.pl?gene=DYNLRB2
GC07P095772	2.7	https://www.genecards.org/cgi-bin/carddisp.pl?gene=DYNC1I1
GC22P042080	2.7	https://www.genecards.org/cgi-bin/carddisp.pl?gene=SEPTIN3
GC08P060516	2.69	https://www.genecards.org/cgi-bin/carddisp.pl?gene=RAB2A
GC16P028974	2.69	https://www.genecards.org/cgi-bin/carddisp.pl?gene=SPNS1
GC0XP014457	2.69	https://www.genecards.org/cgi-bin/carddisp.pl?gene=GLRA2
GC01M071063	2.69	https://www.genecards.org/cgi-bin/carddisp.pl?gene=ZRANB2
GC22P024152	2.69	https://www.genecards.org/cgi-bin/carddisp.pl?gene=PPIL2
GC01M245749	2.69	https://www.genecards.org/cgi-bin/carddisp.pl?gene=SMYD3
GC08P058539	2.69	https://www.genecards.org/cgi-bin/carddisp.pl?gene=SDCBP
GC02M201377	2.69	https://www.genecards.org/cgi-bin/carddisp.pl?gene=TRAK2
GC02P152335	2.69	https://www.genecards.org/cgi-bin/carddisp.pl?gene=FMNL2
GC10P102245	2.69	https://www.genecards.org/cgi-bin/carddisp.pl?gene=GBF1
GC01P065147	2.69	https://www.genecards.org/cgi-bin/carddisp.pl?gene=AK4
GC09P105244	2.69	https://www.genecards.org/cgi-bin/carddisp.pl?gene=SLC44A1
GC08P022544	2.69	https://www.genecards.org/cgi-bin/carddisp.pl?gene=SORBS3
GC12M050175	2.69	https://www.genecards.org/cgi-bin/carddisp.pl?gene=LIMA1
GC02M009391	2.69	https://www.genecards.org/cgi-bin/carddisp.pl?gene=ITGB1BP1
GC15M040278	2.68	https://www.genecards.org/cgi-bin/carddisp.pl?gene=PLCB2
GC01P155063	2.68	https://www.genecards.org/cgi-bin/carddisp.pl?gene=EFNA4
GC04M154534	2.68	https://www.genecards.org/cgi-bin/carddisp.pl?gene=PLRG1
GC07P076510	2.68	https://www.genecards.org/cgi-bin/carddisp.pl?gene=UPK3B
GC03P037243	2.68	https://www.genecards.org/cgi-bin/carddisp.pl?gene=GOLGA4
GC20M017613	2.68	https://www.genecards.org/cgi-bin/carddisp.pl?gene=RRBP1
GC05M000443	2.68	https://www.genecards.org/cgi-bin/carddisp.pl?gene=EXOC3-AS1
GC16M075294	2.67	https://www.genecards.org/cgi-bin/carddisp.pl?gene=CFDP1
GC11M059669	2.67	https://www.genecards.org/cgi-bin/carddisp.pl?gene=CHRM4
GC11P131370	2.67	https://www.genecards.org/cgi-bin/carddisp.pl?gene=NTM
GC01M016014	2.67	https://www.genecards.org/cgi-bin/carddisp.pl?gene=HSPB7
GC07P149195	2.67	https://www.genecards.org/cgi-bin/carddisp.pl?gene=ZNF282
GC01P026932	2.67	https://www.genecards.org/cgi-bin/carddisp.pl?gene=NUDC
GC02M130191	2.67	https://www.genecards.org/cgi-bin/carddisp.pl?gene=TUBA3E

GC16M052587	2.67	https://www.genecards.org/cgi-bin/carddisp.pl?gene=CASC16
GC01M089106	2.66	https://www.genecards.org/cgi-bin/carddisp.pl?gene=GBP2
GC18P050879	2.66	https://www.genecards.org/cgi-bin/carddisp.pl?gene=ME2
GC10M073007	2.66	https://www.genecards.org/cgi-bin/carddisp.pl?gene=P4HA1
GC10P092292	2.66	https://www.genecards.org/cgi-bin/carddisp.pl?gene=MARCHF5
GC04P070195	2.66	https://www.genecards.org/cgi-bin/carddisp.pl?gene=ODAM
GC10P022345	2.66	https://www.genecards.org/cgi-bin/carddisp.pl?gene=SPAG6
GC11P067288	2.66	https://www.genecards.org/cgi-bin/carddisp.pl?gene=ANKRD13D
GC03M058718	2.65	https://www.genecards.org/cgi-bin/carddisp.pl?gene=C3orf67
GC16M030350	2.65	https://www.genecards.org/cgi-bin/carddisp.pl?gene=CD2BP2
GC08M022620	2.65	https://www.genecards.org/cgi-bin/carddisp.pl?gene=BIN3
GC09M101476	2.65	https://www.genecards.org/cgi-bin/carddisp.pl?gene=PGAP4
GC21M039342	2.65	https://www.genecards.org/cgi-bin/carddisp.pl?gene=HMGN1
GC04M046734	2.65	https://www.genecards.org/cgi-bin/carddisp.pl?gene=COX7B2
GC07M139044	2.65	https://www.genecards.org/cgi-bin/carddisp.pl?gene=ZC3HAV1
GC15M074836	2.65	https://www.genecards.org/cgi-bin/carddisp.pl?gene=ULK3
GC09M035812	2.65	https://www.genecards.org/cgi-bin/carddisp.pl?gene=HINT2
GC15M041517	2.65	https://www.genecards.org/cgi-bin/carddisp.pl?gene=RPAP1
GC02M162371	2.65	https://www.genecards.org/cgi-bin/carddisp.pl?gene=KCNH7
GC04M082818	2.64	https://www.genecards.org/cgi-bin/carddisp.pl?gene=SEC31A
GC01M193012	2.64	https://www.genecards.org/cgi-bin/carddisp.pl?gene=UCHL5
GC14M070366	2.64	https://www.genecards.org/cgi-bin/carddisp.pl?gene=SYNJ2BP
GC02P206159	2.64	https://www.genecards.org/cgi-bin/carddisp.pl?gene=EEF1B2
GC05P032533	2.64	https://www.genecards.org/cgi-bin/carddisp.pl?gene=SUB1
GC19M002321	2.64	https://www.genecards.org/cgi-bin/carddisp.pl?gene=LSM7
GC07M076609	2.64	https://www.genecards.org/cgi-bin/carddisp.pl?gene=POMZP3
GC19P038304	2.64	https://www.genecards.org/cgi-bin/carddisp.pl?gene=C19orf33
GC19M002072	2.64	https://www.genecards.org/cgi-bin/carddisp.pl?gene=MOB3A
GC07P004644	2.64	https://www.genecards.org/cgi-bin/carddisp.pl?gene=FOXK1
GC21M015490	2.63	https://www.genecards.org/cgi-bin/carddisp.pl?gene=CYCSP42
GC09M135932	2.63	https://www.genecards.org/cgi-bin/carddisp.pl?gene=UBAC1
GC05P143005	2.63	https://www.genecards.org/cgi-bin/carddisp.pl?gene=TMCO6
GC10M097435	2.63	https://www.genecards.org/cgi-bin/carddisp.pl?gene=EXOSC1
GC02P170772	2.63	https://www.genecards.org/cgi-bin/carddisp.pl?gene=ERICH2
GC18P008600	2.63	https://www.genecards.org/cgi-bin/carddisp.pl?gene=RAB12
GC04M002741	2.63	https://www.genecards.org/cgi-bin/carddisp.pl?gene=TNIP2
GC08M103156	2.63	https://www.genecards.org/cgi-bin/carddisp.pl?gene=BAALC-AS1
GC13M049308	2.63	https://www.genecards.org/cgi-bin/carddisp.pl?gene=CAB39L
GC04P186900	2.63	https://www.genecards.org/cgi-bin/carddisp.pl?gene=MRPS36P2
GC17M074694	2.63	https://www.genecards.org/cgi-bin/carddisp.pl?gene=CD300LF
GC09M021165	2.63	https://www.genecards.org/cgi-bin/carddisp.pl?gene=IFNA21
GC01P052404	2.63	https://www.genecards.org/cgi-bin/carddisp.pl?gene=PRPF38A
GC01M036136	2.63	https://www.genecards.org/cgi-bin/carddisp.pl?gene=TRAPPC3
GC03P120743	2.62	https://www.genecards.org/cgi-bin/carddisp.pl?gene=GTF2E1
GC07M035095	2.62	https://www.genecards.org/cgi-bin/carddisp.pl?gene=DPY19L2P1
GC01M010642	2.62	https://www.genecards.org/cgi-bin/carddisp.pl?gene=CASZ1
GC02P053970	2.62	https://www.genecards.org/cgi-bin/carddisp.pl?gene=ACYP2

GC10P104120	2.62	https://www.genecards.org/cgi-bin/carddisp.pl?gene=SFR1
GC01M010946	2.62	https://www.genecards.org/cgi-bin/carddisp.pl?gene=C1orf127
GC14M022565	2.61	https://www.genecards.org/cgi-bin/carddisp.pl?gene=DAD1
GC01M101802	2.61	https://www.genecards.org/cgi-bin/carddisp.pl?gene=OLFM3
GC02P074198	2.61	https://www.genecards.org/cgi-bin/carddisp.pl?gene=MTHFD2
GC01P154219	2.61	https://www.genecards.org/cgi-bin/carddisp.pl?gene=UBAP2L
GC10P049973	2.61	https://www.genecards.org/cgi-bin/carddisp.pl?gene=AGAP6
GC11Pi00018	2.61	https://www.genecards.org/cgi-bin/carddisp.pl?gene=OR9G9
GC06M085687	2.61	https://www.genecards.org/cgi-bin/carddisp.pl?gene=SNORD50B
GC06M104024	2.61	https://www.genecards.org/cgi-bin/carddisp.pl?gene=NPM1P10
GC08M051319	2.61	https://www.genecards.org/cgi-bin/carddisp.pl?gene=PXDNL
GC07P156640	2.61	https://www.genecards.org/cgi-bin/carddisp.pl?gene=RNF32
GC11M062806	2.61	https://www.genecards.org/cgi-bin/carddisp.pl?gene=STX5
GC10M072332	2.61	https://www.genecards.org/cgi-bin/carddisp.pl?gene=DNAJB12
GC20P045405	2.61	https://www.genecards.org/cgi-bin/carddisp.pl?gene=DBNDD2
GC16M068284	2.6	https://www.genecards.org/cgi-bin/carddisp.pl?gene=SLC7A6OS
GC19M015468	2.6	https://www.genecards.org/cgi-bin/carddisp.pl?gene=PGLYRP2
GC11M078015	2.6	https://www.genecards.org/cgi-bin/carddisp.pl?gene=NDUFC2-KCTD14
GC06M041295	2.6	https://www.genecards.org/cgi-bin/carddisp.pl?gene=SRPK1
GC10M015144	2.6	https://www.genecards.org/cgi-bin/carddisp.pl?gene=NMT2
GC15M030360	2.6	https://www.genecards.org/cgi-bin/carddisp.pl?gene=CHRFAM7A
GC18P013217	2.6	https://www.genecards.org/cgi-bin/carddisp.pl?gene=LDLRAD4
GC03P197737	2.59	https://www.genecards.org/cgi-bin/carddisp.pl?gene=FYTTD1
GC09M129827	2.59	https://www.genecards.org/cgi-bin/carddisp.pl?gene=C9orf78
GC01M150669	2.59	https://www.genecards.org/cgi-bin/carddisp.pl?gene=ENSA
GC04M163323	2.59	https://www.genecards.org/cgi-bin/carddisp.pl?gene=NPY1R
GC05M153990	2.59	https://www.genecards.org/cgi-bin/carddisp.pl?gene=FAM114A2
GC02P222860	2.59	https://www.genecards.org/cgi-bin/carddisp.pl?gene=ACSL3
GC01M155913	2.59	https://www.genecards.org/cgi-bin/carddisp.pl?gene=KHDC4
GC10P084139	2.59	https://www.genecards.org/cgi-bin/carddisp.pl?gene=GHITM
GC07P144749	2.59	https://www.genecards.org/cgi-bin/carddisp.pl?gene=ZYX
GC11M060890	2.59	https://www.genecards.org/cgi-bin/carddisp.pl?gene=PRPF19
GC06M043671	2.59	https://www.genecards.org/cgi-bin/carddisp.pl?gene=MRPS18A
GC09P133474	2.59	https://www.genecards.org/cgi-bin/carddisp.pl?gene=SURF2
GC05M069639	2.59	https://www.genecards.org/cgi-bin/carddisp.pl?gene=GUSBP3
GC17M062264	2.59	https://www.genecards.org/cgi-bin/carddisp.pl?gene=TBC1D3P2
GC13P048975	2.59	https://www.genecards.org/cgi-bin/carddisp.pl?gene=FNDC3A
GC0XP011758	2.58	https://www.genecards.org/cgi-bin/carddisp.pl?gene=MSL3
GC16P069311	2.58	https://www.genecards.org/cgi-bin/carddisp.pl?gene=VPS4A
GC14P020612	2.58	https://www.genecards.org/cgi-bin/carddisp.pl?gene=TRL-AAG2-3
GC03P005187	2.58	https://www.genecards.org/cgi-bin/carddisp.pl?gene=EDEM1
GC05M043288	2.58	https://www.genecards.org/cgi-bin/carddisp.pl?gene=HMGCS1
GC02P218608	2.58	https://www.genecards.org/cgi-bin/carddisp.pl?gene=PLCD4
GC20P037903	2.58	https://www.genecards.org/cgi-bin/carddisp.pl?gene=VSTM2L
GC0XM154483	2.58	https://www.genecards.org/cgi-bin/carddisp.pl?gene=UBL4A
GC12P022046	2.58	https://www.genecards.org/cgi-bin/carddisp.pl?gene=CMAS
GC11P063538	2.58	https://www.genecards.org/cgi-bin/carddisp.pl?gene=PLAAT4

GC01P153660	2.58	https://www.genecards.org/cgi-bin/carddisp.pl?gene=SNAPIN
GC13P097953	2.58	https://www.genecards.org/cgi-bin/carddisp.pl?gene=IPO5
GC15P074541	2.57	https://www.genecards.org/cgi-bin/carddisp.pl?gene=ARID3B
GC22M029554	2.57	https://www.genecards.org/cgi-bin/carddisp.pl?gene=NIPSNAP1
GC06P032659	2.57	https://www.genecards.org/cgi-bin/carddisp.pl?gene=HLA-DQB1-AS1
GC22M023390	2.57	https://www.genecards.org/cgi-bin/carddisp.pl?gene=ZDHHC8P1
GC08M060936	2.57	https://www.genecards.org/cgi-bin/carddisp.pl?gene=NASPP1
GC12M062021	2.57	https://www.genecards.org/cgi-bin/carddisp.pl?gene=RPS3P6
GC19P047114	2.57	https://www.genecards.org/cgi-bin/carddisp.pl?gene=SAE1
GC12M080792	2.57	https://www.genecards.org/cgi-bin/carddisp.pl?gene=LIN7A
GC03P107522	2.57	https://www.genecards.org/cgi-bin/carddisp.pl?gene=BBX
GC13M113977	2.57	https://www.genecards.org/cgi-bin/carddisp.pl?gene=RASA3
GC22M036226	2.57	https://www.genecards.org/cgi-bin/carddisp.pl?gene=APOL2
GC18M037190	2.57	https://www.genecards.org/cgi-bin/carddisp.pl?gene=CELF4
GC19P037712	2.56	https://www.genecards.org/cgi-bin/carddisp.pl?gene=GAPDHS
GC17M029702	2.56	https://www.genecards.org/cgi-bin/carddisp.pl?gene=FLOT2
GC06M075252	2.56	https://www.genecards.org/cgi-bin/carddisp.pl?gene=TMEM30A
GC15P089575	2.56	https://www.genecards.org/cgi-bin/carddisp.pl?gene=TICRR
GC01M162395	2.56	https://www.genecards.org/cgi-bin/carddisp.pl?gene=SH2D1B
GC19M048382	2.56	https://www.genecards.org/cgi-bin/carddisp.pl?gene=KDELR1
GC12M122865	2.56	https://www.genecards.org/cgi-bin/carddisp.pl?gene=VPS37B
GC13M073686	2.56	https://www.genecards.org/cgi-bin/carddisp.pl?gene=KLF12
GC03M126437	2.55	https://www.genecards.org/cgi-bin/carddisp.pl?gene=ZXDC
GC09M004703	2.55	https://www.genecards.org/cgi-bin/carddisp.pl?gene=AK3
GC20M032983	2.55	https://www.genecards.org/cgi-bin/carddisp.pl?gene=SUN5
GC01P110480	2.55	https://www.genecards.org/cgi-bin/carddisp.pl?gene=CYMP
GC08P084182	2.55	https://www.genecards.org/cgi-bin/carddisp.pl?gene=RALYL
GC19M010115	2.55	https://www.genecards.org/cgi-bin/carddisp.pl?gene=EIF3G
GC19P010871	2.55	https://www.genecards.org/cgi-bin/carddisp.pl?gene=CARM1
GC15P051829	2.55	https://www.genecards.org/cgi-bin/carddisp.pl?gene=TMOD3
GC0XM020124	2.55	https://www.genecards.org/cgi-bin/carddisp.pl?gene=EIF1AX
GC05M178185	2.54	https://www.genecards.org/cgi-bin/carddisp.pl?gene=GMCL2
GC11P001948	2.54	https://www.genecards.org/cgi-bin/carddisp.pl?gene=MRPL23
GC08P066671	2.54	https://www.genecards.org/cgi-bin/carddisp.pl?gene=SGK3
GC08M096240	2.54	https://www.genecards.org/cgi-bin/carddisp.pl?gene=MTERF3
GC03P040477	2.54	https://www.genecards.org/cgi-bin/carddisp.pl?gene=ZNF620
GC16M030463	2.54	https://www.genecards.org/cgi-bin/carddisp.pl?gene=SEPHS2
GC18P009465	2.54	https://www.genecards.org/cgi-bin/carddisp.pl?gene=RALBP1
GC17P057085	2.54	https://www.genecards.org/cgi-bin/carddisp.pl?gene=AKAP1
GC11P108008	2.54	https://www.genecards.org/cgi-bin/carddisp.pl?gene=CUL5
GC0XP013707	2.54	https://www.genecards.org/cgi-bin/carddisp.pl?gene=RAB9A
GC01P218285	2.54	https://www.genecards.org/cgi-bin/carddisp.pl?gene=RRP15
GC04M039502	2.53	https://www.genecards.org/cgi-bin/carddisp.pl?gene=UGDH
GC02P170928	2.53	https://www.genecards.org/cgi-bin/carddisp.pl?gene=GORASP2
GC12P053006	2.53	https://www.genecards.org/cgi-bin/carddisp.pl?gene=EIF4B
GC16P089873	2.53	https://www.genecards.org/cgi-bin/carddisp.pl?gene=TCF25
GC19P014433	2.53	https://www.genecards.org/cgi-bin/carddisp.pl?gene=PKN1

GC07P029650	2.53	https://www.genecards.org/cgi-bin/carddisp.pl?gene=DPY19L2P3
GC06P073326	2.53	https://www.genecards.org/cgi-bin/carddisp.pl?gene=PAICSP3
GC17M057861	2.53	https://www.genecards.org/cgi-bin/carddisp.pl?gene=CUEDC1
GC03P045555	2.53	https://www.genecards.org/cgi-bin/carddisp.pl?gene=LIMD1
GC01M036415	2.53	https://www.genecards.org/cgi-bin/carddisp.pl?gene=OSCP1
GC10M073631	2.53	https://www.genecards.org/cgi-bin/carddisp.pl?gene=MYOZ1
GC14M045203	2.53	https://www.genecards.org/cgi-bin/carddisp.pl?gene=MIS18BP1
GC20P038724	2.53	https://www.genecards.org/cgi-bin/carddisp.pl?gene=SLC32A1
GC03M123897	2.53	https://www.genecards.org/cgi-bin/carddisp.pl?gene=CCDC14
GC09M128120	2.52	https://www.genecards.org/cgi-bin/carddisp.pl?gene=PTGES2
GC02M073148	2.52	https://www.genecards.org/cgi-bin/carddisp.pl?gene=RAB11FIP5
GC17P018845	2.51	https://www.genecards.org/cgi-bin/carddisp.pl?gene=PRPSAP2
GC02P169827	2.51	https://www.genecards.org/cgi-bin/carddisp.pl?gene=UBR3
GC17M007389	2.51	https://www.genecards.org/cgi-bin/carddisp.pl?gene=PLSCR3
GC10M015254	2.51	https://www.genecards.org/cgi-bin/carddisp.pl?gene=FAM171A1
GC17P067717	2.51	https://www.genecards.org/cgi-bin/carddisp.pl?gene=NOL11
GC07P100015	2.51	https://www.genecards.org/cgi-bin/carddisp.pl?gene=ZKSCAN1
GC0XP119895	2.51	https://www.genecards.org/cgi-bin/carddisp.pl?gene=AKAP14
GC11P048266	2.51	https://www.genecards.org/cgi-bin/carddisp.pl?gene=OR4X2
GC01M114089	2.51	https://www.genecards.org/cgi-bin/carddisp.pl?gene=SYT6
GC20P062804	2.51	https://www.genecards.org/cgi-bin/carddisp.pl?gene=OGFR
GC11M062560	2.5	https://www.genecards.org/cgi-bin/carddisp.pl?gene=EEF1G
GC11P007966	2.5	https://www.genecards.org/cgi-bin/carddisp.pl?gene=EIF3F
GC01M015808	2.5	https://www.genecards.org/cgi-bin/carddisp.pl?gene=UQCRHL
GC08M085215	2.5	https://www.genecards.org/cgi-bin/carddisp.pl?gene=RBIS
GC01P015409	2.5	https://www.genecards.org/cgi-bin/carddisp.pl?gene=EFHD2
GC0XM066021	2.5	https://www.genecards.org/cgi-bin/carddisp.pl?gene=VSIG4
GC01M155317	2.5	https://www.genecards.org/cgi-bin/carddisp.pl?gene=RUSC1-AS1
GC04M184755	2.5	https://www.genecards.org/cgi-bin/carddisp.pl?gene=ACSL1
GC14P064915	2.5	https://www.genecards.org/cgi-bin/carddisp.pl?gene=CHURC1
GC15P078810	2.5	https://www.genecards.org/cgi-bin/carddisp.pl?gene=MORF4L1
GC19P048900	2.5	https://www.genecards.org/cgi-bin/carddisp.pl?gene=NUCB1
GC17M081911	2.5	https://www.genecards.org/cgi-bin/carddisp.pl?gene=SIRT7
GC16M030435	2.49	https://www.genecards.org/cgi-bin/carddisp.pl?gene=DCTPP1
GC17P028364	2.49	https://www.genecards.org/cgi-bin/carddisp.pl?gene=SARM1
GC12P122974	2.49	https://www.genecards.org/cgi-bin/carddisp.pl?gene=OGFOD2
GC12P120569	2.49	https://www.genecards.org/cgi-bin/carddisp.pl?gene=RNF10
GC10M047460	2.49	https://www.genecards.org/cgi-bin/carddisp.pl?gene=ANXA8
GC15M040979	2.49	https://www.genecards.org/cgi-bin/carddisp.pl?gene=INO80
GC07M132784	2.49	https://www.genecards.org/cgi-bin/carddisp.pl?gene=CHCHD3
GC01M226631	2.49	https://www.genecards.org/cgi-bin/carddisp.pl?gene=ITPKB
GC07P107470	2.49	https://www.genecards.org/cgi-bin/carddisp.pl?gene=GPR22
GC13P077697	2.49	https://www.genecards.org/cgi-bin/carddisp.pl?gene=SLAIN1
GC03P042581	2.49	https://www.genecards.org/cgi-bin/carddisp.pl?gene=SS18L2
GC10M114437	2.49	https://www.genecards.org/cgi-bin/carddisp.pl?gene=ABLIM1
GC09P012775	2.48	https://www.genecards.org/cgi-bin/carddisp.pl?gene=LURAP1L
GC15P074217	2.48	https://www.genecards.org/cgi-bin/carddisp.pl?gene=CCDC33

GC10P093893	2.48	https://www.genecards.org/cgi-bin/carddisp.pl?gene=SLC35G1
GC06M033279	2.48	https://www.genecards.org/cgi-bin/carddisp.pl?gene=WDR46
GC19M010566	2.48	https://www.genecards.org/cgi-bin/carddisp.pl?gene=CDKN2D
GC01P109668	2.48	https://www.genecards.org/cgi-bin/carddisp.pl?gene=GSTM2
GC15P097960	2.48	https://www.genecards.org/cgi-bin/carddisp.pl?gene=ARRDC4
GC11P000880	2.48	https://www.genecards.org/cgi-bin/carddisp.pl?gene=CRACR2B
GC03P049940	2.48	https://www.genecards.org/cgi-bin/carddisp.pl?gene=RBM6
GC16P069424	2.47	https://www.genecards.org/cgi-bin/carddisp.pl?gene=CYB5B
GC0XP132023	2.47	https://www.genecards.org/cgi-bin/carddisp.pl?gene=STK26
GC18P032091	2.47	https://www.genecards.org/cgi-bin/carddisp.pl?gene=RNF138
GC14P103333	2.47	https://www.genecards.org/cgi-bin/carddisp.pl?gene=EIF5
GC11M008920	2.47	https://www.genecards.org/cgi-bin/carddisp.pl?gene=C11orf16
GC01P156641	2.47	https://www.genecards.org/cgi-bin/carddisp.pl?gene=BCAN
GC01P036089	2.47	https://www.genecards.org/cgi-bin/carddisp.pl?gene=ADPRS
GC05M123023	2.47	https://www.genecards.org/cgi-bin/carddisp.pl?gene=PPIC
GC11P002400	2.47	https://www.genecards.org/cgi-bin/carddisp.pl?gene=TSSC4
GC15P044957	2.46	https://www.genecards.org/cgi-bin/carddisp.pl?gene=TERB2
GC01M149937	2.46	https://www.genecards.org/cgi-bin/carddisp.pl?gene=OTUD7B
GC14P104547	2.46	https://www.genecards.org/cgi-bin/carddisp.pl?gene=PPP2R5C
GC08M065602	2.46	https://www.genecards.org/cgi-bin/carddisp.pl?gene=ARMC1
GC05M083940	2.46	https://www.genecards.org/cgi-bin/carddisp.pl?gene=EDIL3
GC10M024982	2.46	https://www.genecards.org/cgi-bin/carddisp.pl?gene=ENKUR
GC09P128455	2.46	https://www.genecards.org/cgi-bin/carddisp.pl?gene=ODF2
GC01M145738	2.46	https://www.genecards.org/cgi-bin/carddisp.pl?gene=RNF115
GC20P043914	2.46	https://www.genecards.org/cgi-bin/carddisp.pl?gene=TOX2
GC16P024267	2.45	https://www.genecards.org/cgi-bin/carddisp.pl?gene=CACNG3
GC12P067558	2.45	https://www.genecards.org/cgi-bin/carddisp.pl?gene=DYRK2
GC06M027810	2.45	https://www.genecards.org/cgi-bin/carddisp.pl?gene=H2BC13
GC20M004852	2.45	https://www.genecards.org/cgi-bin/carddisp.pl?gene=SLC23A2
GC05M040825	2.45	https://www.genecards.org/cgi-bin/carddisp.pl?gene=RPL37
GC01M153327	2.45	https://www.genecards.org/cgi-bin/carddisp.pl?gene=PGLYRP4
GC01M054225	2.45	https://www.genecards.org/cgi-bin/carddisp.pl?gene=SSBP3
GC01M083865	2.45	https://www.genecards.org/cgi-bin/carddisp.pl?gene=TTLL7
GC12P109027	2.44	https://www.genecards.org/cgi-bin/carddisp.pl?gene=USP30
GC03M182938	2.44	https://www.genecards.org/cgi-bin/carddisp.pl?gene=DCUN1D1
GC12M055820	2.44	https://www.genecards.org/cgi-bin/carddisp.pl?gene=DNAJC14
GC0XP100584	2.44	https://www.genecards.org/cgi-bin/carddisp.pl?gene=TNMD
GC09M086288	2.44	https://www.genecards.org/cgi-bin/carddisp.pl?gene=TUT7
GC21P044350	2.44	https://www.genecards.org/cgi-bin/carddisp.pl?gene=TRPM2
GC01P001472	2.44	https://www.genecards.org/cgi-bin/carddisp.pl?gene=ATAD3B
GC19P003506	2.44	https://www.genecards.org/cgi-bin/carddisp.pl?gene=FZR1
GC03M161344	2.44	https://www.genecards.org/cgi-bin/carddisp.pl?gene=SPTSSB
GC02P168455	2.43	https://www.genecards.org/cgi-bin/carddisp.pl?gene=CERS6
GC01M155754	2.43	https://www.genecards.org/cgi-bin/carddisp.pl?gene=GON4L
GC22M041244	2.43	https://www.genecards.org/cgi-bin/carddisp.pl?gene=RANGAP1
GC16P030058	2.43	https://www.genecards.org/cgi-bin/carddisp.pl?gene=ASPHD1
GC03M146515	2.43	https://www.genecards.org/cgi-bin/carddisp.pl?gene=PLSCR1

GC20M000647	2.43	https://www.genecards.org/cgi-bin/carddisp.pl?gene=SRXN1
GC10M118674	2.43	https://www.genecards.org/cgi-bin/carddisp.pl?gene=CACUL1
GC21M039184	2.43	https://www.genecards.org/cgi-bin/carddisp.pl?gene=BRWD1
GC05M112876	2.43	https://www.genecards.org/cgi-bin/carddisp.pl?gene=REEP5
GC11P032090	2.43	https://www.genecards.org/cgi-bin/carddisp.pl?gene=RCN1
GC03M120686	2.43	https://www.genecards.org/cgi-bin/carddisp.pl?gene=RABL3
GC06P043040	2.43	https://www.genecards.org/cgi-bin/carddisp.pl?gene=KLC4
GC17M065009	2.42	https://www.genecards.org/cgi-bin/carddisp.pl?gene=GNA13
GC12M076036	2.42	https://www.genecards.org/cgi-bin/carddisp.pl?gene=NAP1L1
GC01M155046	2.42	https://www.genecards.org/cgi-bin/carddisp.pl?gene=DCST1-AS1
GC09M021103	2.42	https://www.genecards.org/cgi-bin/carddisp.pl?gene=IFNW1
GC18P006512	2.42	https://www.genecards.org/cgi-bin/carddisp.pl?gene=LINC01387
GC06P150143	2.42	https://www.genecards.org/cgi-bin/carddisp.pl?gene=PPP1R14C
GC12P012814	2.41	https://www.genecards.org/cgi-bin/carddisp.pl?gene=DDX47
GC06M052896	2.41	https://www.genecards.org/cgi-bin/carddisp.pl?gene=GSTA3
GC15M066336	2.41	https://www.genecards.org/cgi-bin/carddisp.pl?gene=TIPIN
GC12P041188	2.41	https://www.genecards.org/cgi-bin/carddisp.pl?gene=PDZRN4
GC03P098732	2.41	https://www.genecards.org/cgi-bin/carddisp.pl?gene=ST3GAL6
GC01M155255	2.41	https://www.genecards.org/cgi-bin/carddisp.pl?gene=SCAMP3
GC11M107326	2.41	https://www.genecards.org/cgi-bin/carddisp.pl?gene=CWF19L2
GC20P062302	2.41	https://www.genecards.org/cgi-bin/carddisp.pl?gene=ADRM1
GC18P000158	2.4	https://www.genecards.org/cgi-bin/carddisp.pl?gene=USP14
GC05M060751	2.4	https://www.genecards.org/cgi-bin/carddisp.pl?gene=ELOVL7
GC14P095686	2.4	https://www.genecards.org/cgi-bin/carddisp.pl?gene=TCL1B
GC03M049268	2.4	https://www.genecards.org/cgi-bin/carddisp.pl?gene=C3orf62
GC12P095473	2.4	https://www.genecards.org/cgi-bin/carddisp.pl?gene=METAP2
GC11P075718	2.4	https://www.genecards.org/cgi-bin/carddisp.pl?gene=SLCO2B1
GC19P004007	2.4	https://www.genecards.org/cgi-bin/carddisp.pl?gene=PIAS4
GC17P069577	2.4	https://www.genecards.org/cgi-bin/carddisp.pl?gene=LINC01483
GC19P010107	2.39	https://www.genecards.org/cgi-bin/carddisp.pl?gene=PPAN
GC05P134371	2.39	https://www.genecards.org/cgi-bin/carddisp.pl?gene=UBE2B
GC01M149936	2.39	https://www.genecards.org/cgi-bin/carddisp.pl?gene=H2AC21
GC01M167498	2.39	https://www.genecards.org/cgi-bin/carddisp.pl?gene=CREG1
GC17M075874	2.39	https://www.genecards.org/cgi-bin/carddisp.pl?gene=TRIM47
GC16P001229	2.39	https://www.genecards.org/cgi-bin/carddisp.pl?gene=DECR2
GC16P002391	2.39	https://www.genecards.org/cgi-bin/carddisp.pl?gene=RAB26
GC04M003048	2.39	https://www.genecards.org/cgi-bin/carddisp.pl?gene=HTT-AS
GC01M161100	2.39	https://www.genecards.org/cgi-bin/carddisp.pl?gene=PFDN2
GC03P160399	2.38	https://www.genecards.org/cgi-bin/carddisp.pl?gene=SMC4
GC11P032564	2.38	https://www.genecards.org/cgi-bin/carddisp.pl?gene=EIF3M
GC02M003501	2.38	https://www.genecards.org/cgi-bin/carddisp.pl?gene=ADI1
GC0XP096883	2.38	https://www.genecards.org/cgi-bin/carddisp.pl?gene=RPA4
GC04M079225	2.38	https://www.genecards.org/cgi-bin/carddisp.pl?gene=NAA11
GC18P045212	2.38	https://www.genecards.org/cgi-bin/carddisp.pl?gene=SLC14A2
GC09P101029	2.38	https://www.genecards.org/cgi-bin/carddisp.pl?gene=PLPPR1
GC10M070247	2.38	https://www.genecards.org/cgi-bin/carddisp.pl?gene=NPFFR1
GC11M063078	2.37	https://www.genecards.org/cgi-bin/carddisp.pl?gene=UBXN1

GC10P123666	2.37	https://www.genecards.org/cgi-bin/carddisp.pl?gene=GPR26
GC16P000386	2.37	https://www.genecards.org/cgi-bin/carddisp.pl?gene=RPL23AP5
GC16P024537	2.37	https://www.genecards.org/cgi-bin/carddisp.pl?gene=RBBP6
GC11M059670	2.37	https://www.genecards.org/cgi-bin/carddisp.pl?gene=AMBRA1
GC10P068331	2.37	https://www.genecards.org/cgi-bin/carddisp.pl?gene=HNRNPH3
GC02M097638	2.37	https://www.genecards.org/cgi-bin/carddisp.pl?gene=ACTR1B
GC05P010441	2.37	https://www.genecards.org/cgi-bin/carddisp.pl?gene=ROPN1L
GC17P050373	2.37	https://www.genecards.org/cgi-bin/carddisp.pl?gene=EME1
GC11M006476	2.37	https://www.genecards.org/cgi-bin/carddisp.pl?gene=ARFIP2
GC01M202964	2.37	https://www.genecards.org/cgi-bin/carddisp.pl?gene=CYB5R1
GC17M048070	2.37	https://www.genecards.org/cgi-bin/carddisp.pl?gene=CBX1
GC0XP155025	2.37	https://www.genecards.org/cgi-bin/carddisp.pl?gene=FUNDC2
GC01P020186	2.36	https://www.genecards.org/cgi-bin/carddisp.pl?gene=UBXN10
GC19M054351	2.36	https://www.genecards.org/cgi-bin/carddisp.pl?gene=LAIR1
GC02P027663	2.36	https://www.genecards.org/cgi-bin/carddisp.pl?gene=SLC4A1AP
GC17M043014	2.36	https://www.genecards.org/cgi-bin/carddisp.pl?gene=VAT1
GC02M055172	2.36	https://www.genecards.org/cgi-bin/carddisp.pl?gene=CLHC1
GC02P095165	2.36	https://www.genecards.org/cgi-bin/carddisp.pl?gene=ZNF2
GC07P100673	2.36	https://www.genecards.org/cgi-bin/carddisp.pl?gene=GNB2
GC01P109711	2.35	https://www.genecards.org/cgi-bin/carddisp.pl?gene=GSTM5
GC16M030553	2.35	https://www.genecards.org/cgi-bin/carddisp.pl?gene=ZNF764
GC20M049903	2.35	https://www.genecards.org/cgi-bin/carddisp.pl?gene=SPATA2
GC17P042494	2.35	https://www.genecards.org/cgi-bin/carddisp.pl?gene=MIR548AT
GC03P028574	2.35	https://www.genecards.org/cgi-bin/carddisp.pl?gene=LINC00693
GC02P119366	2.35	https://www.genecards.org/cgi-bin/carddisp.pl?gene=DBI
GC20M002794	2.35	https://www.genecards.org/cgi-bin/carddisp.pl?gene=CPXM1
GC12P006580	2.35	https://www.genecards.org/cgi-bin/carddisp.pl?gene=TIGAR
GC13P114281	2.35	https://www.genecards.org/cgi-bin/carddisp.pl?gene=UPF3A
GC01M037956	2.35	https://www.genecards.org/cgi-bin/carddisp.pl?gene=SF3A3
GC16P030949	2.35	https://www.genecards.org/cgi-bin/carddisp.pl?gene=ORAI3
GC18M056597	2.34	https://www.genecards.org/cgi-bin/carddisp.pl?gene=TXNL1
GC12M013399	2.34	https://www.genecards.org/cgi-bin/carddisp.pl?gene=PRB1
GC06M127288	2.34	https://www.genecards.org/cgi-bin/carddisp.pl?gene=ECHDC1
GC01M151870	2.34	https://www.genecards.org/cgi-bin/carddisp.pl?gene=THEM4
GC01P215567	2.33	https://www.genecards.org/cgi-bin/carddisp.pl?gene=KCTD3
GC02M068041	2.33	https://www.genecards.org/cgi-bin/carddisp.pl?gene=C1D
GC06M008014	2.33	https://www.genecards.org/cgi-bin/carddisp.pl?gene=BLOC1S5
GC06M032372	2.33	https://www.genecards.org/cgi-bin/carddisp.pl?gene=GPANK1
GC09P099222	2.33	https://www.genecards.org/cgi-bin/carddisp.pl?gene=SEC61B
GC12M007812	2.33	https://www.genecards.org/cgi-bin/carddisp.pl?gene=SLC2A14
GC10M093306	2.33	https://www.genecards.org/cgi-bin/carddisp.pl?gene=MYOF
GC01M151803	2.33	https://www.genecards.org/cgi-bin/carddisp.pl?gene=TDRKH
GC09P094030	2.33	https://www.genecards.org/cgi-bin/carddisp.pl?gene=PTPDC1
GC01P179025	2.33	https://www.genecards.org/cgi-bin/carddisp.pl?gene=FAM20B
GC02M073254	2.33	https://www.genecards.org/cgi-bin/carddisp.pl?gene=FBXO41
GC01M054172	2.33	https://www.genecards.org/cgi-bin/carddisp.pl?gene=CYB5RL
GC06P073394	2.33	https://www.genecards.org/cgi-bin/carddisp.pl?gene=DDX43

GC02P105337	2.33	https://www.genecards.org/cgi-bin/carddisp.pl?gene=C2orf49
GC0XP034870	2.33	https://www.genecards.org/cgi-bin/carddisp.pl?gene=FAM47B
GC20M063528	2.33	https://www.genecards.org/cgi-bin/carddisp.pl?gene=PTK6
GC20M062544	2.32	https://www.genecards.org/cgi-bin/carddisp.pl?gene=MIR1-1HG-AS1
GC15P060558	2.32	https://www.genecards.org/cgi-bin/carddisp.pl?gene=CYCSP38
GC22M020099	2.32	https://www.genecards.org/cgi-bin/carddisp.pl?gene=TRMT2A
GC12P015882	2.32	https://www.genecards.org/cgi-bin/carddisp.pl?gene=STRAP
GC08M080034	2.32	https://www.genecards.org/cgi-bin/carddisp.pl?gene=TPD52
GC03P111978	2.32	https://www.genecards.org/cgi-bin/carddisp.pl?gene=ABHD10
GC12M122472	2.32	https://www.genecards.org/cgi-bin/carddisp.pl?gene=ZCCHC8
GC05P082279	2.32	https://www.genecards.org/cgi-bin/carddisp.pl?gene=ATP6AP1L
GC04M075576	2.31	https://www.genecards.org/cgi-bin/carddisp.pl?gene=CDKL2
GC04P076950	2.31	https://www.genecards.org/cgi-bin/carddisp.pl?gene=SEPTIN11
GC20P062881	2.31	https://www.genecards.org/cgi-bin/carddisp.pl?gene=MRGBP
GC12P111686	2.31	https://www.genecards.org/cgi-bin/carddisp.pl?gene=ACAD10
GC01M063440	2.31	https://www.genecards.org/cgi-bin/carddisp.pl?gene=ITGB3BP
GC01P155445	2.31	https://www.genecards.org/cgi-bin/carddisp.pl?gene=POU5F1P4
GC04P088457	2.31	https://www.genecards.org/cgi-bin/carddisp.pl?gene=HERC5
GC07P100539	2.31	https://www.genecards.org/cgi-bin/carddisp.pl?gene=AGFG2
GC17M062704	2.31	https://www.genecards.org/cgi-bin/carddisp.pl?gene=MARCHF10
GC03M015442	2.31	https://www.genecards.org/cgi-bin/carddisp.pl?gene=SH3BP5
GC04P077862	2.31	https://www.genecards.org/cgi-bin/carddisp.pl?gene=MRPL1
GC01M021678	2.3	https://www.genecards.org/cgi-bin/carddisp.pl?gene=USP48
GC07P100706	2.3	https://www.genecards.org/cgi-bin/carddisp.pl?gene=POP7
GC21M041885	2.3	https://www.genecards.org/cgi-bin/carddisp.pl?gene=C2CD2
GC08P086342	2.3	https://www.genecards.org/cgi-bin/carddisp.pl?gene=WWP1
GC06M007893	2.3	https://www.genecards.org/cgi-bin/carddisp.pl?gene=TXNDC5
GC20P033993	2.3	https://www.genecards.org/cgi-bin/carddisp.pl?gene=RALY
GC19P049527	2.3	https://www.genecards.org/cgi-bin/carddisp.pl?gene=RCN3
GC17P082458	2.3	https://www.genecards.org/cgi-bin/carddisp.pl?gene=NARF
GC05P000443	2.3	https://www.genecards.org/cgi-bin/carddisp.pl?gene=EXOC3
GC10P114938	2.3	https://www.genecards.org/cgi-bin/carddisp.pl?gene=TRUB1
GC06P070667	2.3	https://www.genecards.org/cgi-bin/carddisp.pl?gene=SMAP1
GC12P012890	2.29	https://www.genecards.org/cgi-bin/carddisp.pl?gene=GPRC5A
GC17M007459	2.29	https://www.genecards.org/cgi-bin/carddisp.pl?gene=ZBTB4
GC18M049782	2.29	https://www.genecards.org/cgi-bin/carddisp.pl?gene=ACAA2
GC16M030569	2.29	https://www.genecards.org/cgi-bin/carddisp.pl?gene=ZNF688
GC01P247738	2.29	https://www.genecards.org/cgi-bin/carddisp.pl?gene=OR14K1
GC21M009068	2.29	https://www.genecards.org/cgi-bin/carddisp.pl?gene=TEKT4P2
GC15M033866	2.29	https://www.genecards.org/cgi-bin/carddisp.pl?gene=AVEN
GC06P080003	2.29	https://www.genecards.org/cgi-bin/carddisp.pl?gene=TTK
GC17M047824	2.29	https://www.genecards.org/cgi-bin/carddisp.pl?gene=MRPL10
GC0YP019567	2.28	https://www.genecards.org/cgi-bin/carddisp.pl?gene=TXLNGY
GC01M015571	2.28	https://www.genecards.org/cgi-bin/carddisp.pl?gene=AGMAT
GC19P000708	2.28	https://www.genecards.org/cgi-bin/carddisp.pl?gene=PALM
GC16M069747	2.28	https://www.genecards.org/cgi-bin/carddisp.pl?gene=NOB1
GC20M062877	2.28	https://www.genecards.org/cgi-bin/carddisp.pl?gene=DIDO1

GC06M032401	2.28	https://www.genecards.org/cgi-bin/carddisp.pl?gene=FKBPL
GC11M133897	2.28	https://www.genecards.org/cgi-bin/carddisp.pl?gene=IGSF9B
GC17P076006	2.28	https://www.genecards.org/cgi-bin/carddisp.pl?gene=CDK3
GC07P026201	2.28	https://www.genecards.org/cgi-bin/carddisp.pl?gene=CBX3
GC01P016005	2.28	https://www.genecards.org/cgi-bin/carddisp.pl?gene=SRARP
GC02M064092	2.28	https://www.genecards.org/cgi-bin/carddisp.pl?gene=PELI1
GC07M127580	2.28	https://www.genecards.org/cgi-bin/carddisp.pl?gene=GCC1
GC15P043796	2.28	https://www.genecards.org/cgi-bin/carddisp.pl?gene=HYPK
GC13M021153	2.28	https://www.genecards.org/cgi-bin/carddisp.pl?gene=SKA3
GC02P032628	2.28	https://www.genecards.org/cgi-bin/carddisp.pl?gene=TTC27
GC18M036108	2.28	https://www.genecards.org/cgi-bin/carddisp.pl?gene=SLC39A6
GC01M043383	2.28	https://www.genecards.org/cgi-bin/carddisp.pl?gene=MED8
GC02P219248	2.27	https://www.genecards.org/cgi-bin/carddisp.pl?gene=STK16
GC11M066664	2.27	https://www.genecards.org/cgi-bin/carddisp.pl?gene=RBM4B
GC02P182716	2.27	https://www.genecards.org/cgi-bin/carddisp.pl?gene=DNAJC10
GC15P045632	2.27	https://www.genecards.org/cgi-bin/carddisp.pl?gene=SQOR
GC0XM155062	2.27	https://www.genecards.org/cgi-bin/carddisp.pl?gene=MTCP1
GC12M043793	2.27	https://www.genecards.org/cgi-bin/carddisp.pl?gene=TWF1
GC06M032706	2.27	https://www.genecards.org/cgi-bin/carddisp.pl?gene=MTCO3P1
GC01P205197	2.27	https://www.genecards.org/cgi-bin/carddisp.pl?gene=TMCC2
GC05P127517	2.27	https://www.genecards.org/cgi-bin/carddisp.pl?gene=PRRC1
GC15M099136	2.26	https://www.genecards.org/cgi-bin/carddisp.pl?gene=TTC23
GC11P065123	2.26	https://www.genecards.org/cgi-bin/carddisp.pl?gene=MRPL49
GC07M129830	2.26	https://www.genecards.org/cgi-bin/carddisp.pl?gene=UBE2H
GC17M005501	2.26	https://www.genecards.org/cgi-bin/carddisp.pl?gene=LOC728392
GC11P074312	2.26	https://www.genecards.org/cgi-bin/carddisp.pl?gene=P4HA3-AS1
GC15M044825	2.26	https://www.genecards.org/cgi-bin/carddisp.pl?gene=SORD2P
GC07P035826	2.26	https://www.genecards.org/cgi-bin/carddisp.pl?gene=LOC100506725
GC02M080264	2.26	https://www.genecards.org/cgi-bin/carddisp.pl?gene=LRRTM1
GC17P044708	2.26	https://www.genecards.org/cgi-bin/carddisp.pl?gene=DBF4B
GC16P001989	2.26	https://www.genecards.org/cgi-bin/carddisp.pl?gene=SYNGR3
GC15P071994	2.26	https://www.genecards.org/cgi-bin/carddisp.pl?gene=MIR190A
GC0XP154458	2.25	https://www.genecards.org/cgi-bin/carddisp.pl?gene=PLXNA3
GC19P045692	2.25	https://www.genecards.org/cgi-bin/carddisp.pl?gene=QPCTL
GC15M073978	2.25	https://www.genecards.org/cgi-bin/carddisp.pl?gene=STOML1
GC02P027212	2.25	https://www.genecards.org/cgi-bin/carddisp.pl?gene=ATRAID
GC05P058582	2.25	https://www.genecards.org/cgi-bin/carddisp.pl?gene=RAB3C
GC11M113733	2.25	https://www.genecards.org/cgi-bin/carddisp.pl?gene=ZW10
GC06M027450	2.25	https://www.genecards.org/cgi-bin/carddisp.pl?gene=ZNF184
GC01P047333	2.25	https://www.genecards.org/cgi-bin/carddisp.pl?gene=CMPK1
GC17P049788	2.25	https://www.genecards.org/cgi-bin/carddisp.pl?gene=KAT7
GC09P137255	2.25	https://www.genecards.org/cgi-bin/carddisp.pl?gene=NELFB
GC02P086721	2.25	https://www.genecards.org/cgi-bin/carddisp.pl?gene=RMND5A
GC03P156673	2.25	https://www.genecards.org/cgi-bin/carddisp.pl?gene=TIPARP
GC02M219373	2.24	https://www.genecards.org/cgi-bin/carddisp.pl?gene=DNPEP
GC11P107592	2.24	https://www.genecards.org/cgi-bin/carddisp.pl?gene=ELMOD1
GC06P007389	2.24	https://www.genecards.org/cgi-bin/carddisp.pl?gene=RIOK1

GC06P110982	2.24	https://www.genecards.org/cgi-bin/carddisp.pl?gene=RPF2
GC12P006765	2.24	https://www.genecards.org/cgi-bin/carddisp.pl?gene=PTMS
GC01M037566	2.24	https://www.genecards.org/cgi-bin/carddisp.pl?gene=GNL2
GC0XM016839	2.24	https://www.genecards.org/cgi-bin/carddisp.pl?gene=RBBP7
GC15P101960	2.24	https://www.genecards.org/cgi-bin/carddisp.pl?gene=WASH3P
GC11M072759	2.24	https://www.genecards.org/cgi-bin/carddisp.pl?gene=STARD10
GC02P069897	2.23	https://www.genecards.org/cgi-bin/carddisp.pl?gene=MXD1
GC05P163523	2.23	https://www.genecards.org/cgi-bin/carddisp.pl?gene=MAT2B
GC03M124761	2.23	https://www.genecards.org/cgi-bin/carddisp.pl?gene=ITGB5
GC14P049693	2.23	https://www.genecards.org/cgi-bin/carddisp.pl?gene=KLHDC1
GC17M044674	2.23	https://www.genecards.org/cgi-bin/carddisp.pl?gene=CCDC43
GC12M122503	2.23	https://www.genecards.org/cgi-bin/carddisp.pl?gene=RSRC2
GC06M041406	2.23	https://www.genecards.org/cgi-bin/carddisp.pl?gene=ILRUN
GC02P026310	2.23	https://www.genecards.org/cgi-bin/carddisp.pl?gene=SELENOI
GC01M052020	2.23	https://www.genecards.org/cgi-bin/carddisp.pl?gene=TXNDC12
GC10P102743	2.23	https://www.genecards.org/cgi-bin/carddisp.pl?gene=WBP1L
GC22M042835	2.23	https://www.genecards.org/cgi-bin/carddisp.pl?gene=PACSIN2
GC06M089642	2.23	https://www.genecards.org/cgi-bin/carddisp.pl?gene=MDN1
GC0XM024576	2.23	https://www.genecards.org/cgi-bin/carddisp.pl?gene=PCYT1B
GC03M115802	2.23	https://www.genecards.org/cgi-bin/carddisp.pl?gene=LSAMP
GC17P030477	2.23	https://www.genecards.org/cgi-bin/carddisp.pl?gene=GOSR1
GC01M001702	2.23	https://www.genecards.org/cgi-bin/carddisp.pl?gene=CDK11A
GC19M052594	2.22	https://www.genecards.org/cgi-bin/carddisp.pl?gene=ZNF83
GC16P033205	2.22	https://www.genecards.org/cgi-bin/carddisp.pl?gene=TP53TG3C
GC18M016788	2.22	https://www.genecards.org/cgi-bin/carddisp.pl?gene=LDLRAD4-AS1
GC13P114154	2.22	https://www.genecards.org/cgi-bin/carddisp.pl?gene=CFAP97D2
GC06P003458	2.22	https://www.genecards.org/cgi-bin/carddisp.pl?gene=LOC643327
GC17M007583	2.22	https://www.genecards.org/cgi-bin/carddisp.pl?gene=LOC100996842
GC02M077743	2.22	https://www.genecards.org/cgi-bin/carddisp.pl?gene=LOC101927967
GC05P170353	2.22	https://www.genecards.org/cgi-bin/carddisp.pl?gene=KCNIP1
GC06M041305	2.22	https://www.genecards.org/cgi-bin/carddisp.pl?gene=MTCH1
GC06M043479	2.22	https://www.genecards.org/cgi-bin/carddisp.pl?gene=YIPF3
GC16P070289	2.22	https://www.genecards.org/cgi-bin/carddisp.pl?gene=DDX19B
GC01M243124	2.22	https://www.genecards.org/cgi-bin/carddisp.pl?gene=CEP170
GC03P038183	2.22	https://www.genecards.org/cgi-bin/carddisp.pl?gene=OXSR1
GC01P150282	2.22	https://www.genecards.org/cgi-bin/carddisp.pl?gene=CIART
GC12M046358	2.22	https://www.genecards.org/cgi-bin/carddisp.pl?gene=SLC38A2
GC05M118836	2.21	https://www.genecards.org/cgi-bin/carddisp.pl?gene=DTWD2
GC01M151169	2.21	https://www.genecards.org/cgi-bin/carddisp.pl?gene=TMOD4
GC15P041691	2.21	https://www.genecards.org/cgi-bin/carddisp.pl?gene=MIR626
GC11M095166	2.21	https://www.genecards.org/cgi-bin/carddisp.pl?gene=SESN3
GC07M128851	2.21	https://www.genecards.org/cgi-bin/carddisp.pl?gene=FLNC-AS1
GC11M062594	2.21	https://www.genecards.org/cgi-bin/carddisp.pl?gene=MTA2
GC16M066754	2.21	https://www.genecards.org/cgi-bin/carddisp.pl?gene=DYNC1LI2
GC05M176383	2.21	https://www.genecards.org/cgi-bin/carddisp.pl?gene=NOP16
GC05M173607	2.21	https://www.genecards.org/cgi-bin/carddisp.pl?gene=BOD1
GC12M056309	2.21	https://www.genecards.org/cgi-bin/carddisp.pl?gene=CNPY2

GC09P022436	2.21	https://www.genecards.org/cgi-bin/carddisp.pl?gene=DMRTA1
GC17P030906	2.21	https://www.genecards.org/cgi-bin/carddisp.pl?gene=ADAP2
GC09M121178	2.21	https://www.genecards.org/cgi-bin/carddisp.pl?gene=RAB14
GC03M125529	2.2	https://www.genecards.org/cgi-bin/carddisp.pl?gene=OSBPL11
GC05M097160	2.2	https://www.genecards.org/cgi-bin/carddisp.pl?gene=RIOK2
GC02P226835	2.2	https://www.genecards.org/cgi-bin/carddisp.pl?gene=RHBDD1
GC12M096278	2.2	https://www.genecards.org/cgi-bin/carddisp.pl?gene=CDK17
GC22P045163	2.2	https://www.genecards.org/cgi-bin/carddisp.pl?gene=NUP50
GC19M002754	2.2	https://www.genecards.org/cgi-bin/carddisp.pl?gene=SGTA
GC01M031259	2.2	https://www.genecards.org/cgi-bin/carddisp.pl?gene=SNRNP40
GC17P046193	2.2	https://www.genecards.org/cgi-bin/carddisp.pl?gene=KANSL1-AS1
GC05P015553	2.2	https://www.genecards.org/cgi-bin/carddisp.pl?gene=FBXL7
GC02M241584	2.2	https://www.genecards.org/cgi-bin/carddisp.pl?gene=THAP4
GC04P145097	2.2	https://www.genecards.org/cgi-bin/carddisp.pl?gene=ABCE1
GC10P045000	2.2	https://www.genecards.org/cgi-bin/carddisp.pl?gene=ZNF22
GC01M205302	2.2	https://www.genecards.org/cgi-bin/carddisp.pl?gene=NUAK2
GC06P028080	2.19	https://www.genecards.org/cgi-bin/carddisp.pl?gene=ZNF165
GC19M011131	2.19	https://www.genecards.org/cgi-bin/carddisp.pl?gene=SPC24
GC09P135075	2.19	https://www.genecards.org/cgi-bin/carddisp.pl?gene=OLFM1
GC19P049606	2.19	https://www.genecards.org/cgi-bin/carddisp.pl?gene=SNORD35A
GC19M023866	2.19	https://www.genecards.org/cgi-bin/carddisp.pl?gene=LOC105372310
GC14M102342	2.19	https://www.genecards.org/cgi-bin/carddisp.pl?gene=CINP
GC13P024010	2.19	https://www.genecards.org/cgi-bin/carddisp.pl?gene=SPATA13
GC22P020965	2.19	https://www.genecards.org/cgi-bin/carddisp.pl?gene=AIFM3
GC15P024954	2.19	https://www.genecards.org/cgi-bin/carddisp.pl?gene=SNURF
GC11P127002	2.19	https://www.genecards.org/cgi-bin/carddisp.pl?gene=KIRREL3-AS3
GC04P140524	2.19	https://www.genecards.org/cgi-bin/carddisp.pl?gene=ELMOD2
GC11M072686	2.18	https://www.genecards.org/cgi-bin/carddisp.pl?gene=ARAP1
GC11M010856	2.18	https://www.genecards.org/cgi-bin/carddisp.pl?gene=EIF4G2
GC01P155173	2.18	https://www.genecards.org/cgi-bin/carddisp.pl?gene=TRIM46
GC20M026161	2.18	https://www.genecards.org/cgi-bin/carddisp.pl?gene=MIR663AHG
GC02M027130	2.18	https://www.genecards.org/cgi-bin/carddisp.pl?gene=PREB
GC17M048757	2.18	https://www.genecards.org/cgi-bin/carddisp.pl?gene=TTLL6
GC02M200853	2.18	https://www.genecards.org/cgi-bin/carddisp.pl?gene=CLK1
GC05P141475	2.18	https://www.genecards.org/cgi-bin/carddisp.pl?gene=PCDHGC3
GC06P033574	2.18	https://www.genecards.org/cgi-bin/carddisp.pl?gene=lnc-HLA-DQA1-9
GC06M032609	2.18	https://www.genecards.org/cgi-bin/carddisp.pl?gene=lnc-HLA-DRB1-7
GC04M099896	2.17	https://www.genecards.org/cgi-bin/carddisp.pl?gene=DNAJB14
GC11M014436	2.17	https://www.genecards.org/cgi-bin/carddisp.pl?gene=COPB1
GC04P048343	2.17	https://www.genecards.org/cgi-bin/carddisp.pl?gene=SLAIN2
GC04P098261	2.17	https://www.genecards.org/cgi-bin/carddisp.pl?gene=RAP1GDS1
GC20M003162	2.17	https://www.genecards.org/cgi-bin/carddisp.pl?gene=LZTS3
GC12P120687	2.17	https://www.genecards.org/cgi-bin/carddisp.pl?gene=MLEC
GC20M052051	2.17	https://www.genecards.org/cgi-bin/carddisp.pl?gene=ZFP64
GC14M105830	2.17	https://www.genecards.org/cgi-bin/carddisp.pl?gene=IGHA1
GC11M094962	2.17	https://www.genecards.org/cgi-bin/carddisp.pl?gene=CWC15
GC12M108645	2.17	https://www.genecards.org/cgi-bin/carddisp.pl?gene=CORO1C

GC02P068431	2.17	https://www.genecards.org/cgi-bin/carddisp.pl?gene=APLF
GC20P059146	2.17	https://www.genecards.org/cgi-bin/carddisp.pl?gene=ZNF831
GC19M038836	2.17	https://www.genecards.org/cgi-bin/carddisp.pl?gene=HNRNPL
GC17M029698	2.16	https://www.genecards.org/cgi-bin/carddisp.pl?gene=ALDOC
GC04P189472	2.16	https://www.genecards.org/cgi-bin/carddisp.pl?gene=HSP90AA4P
GC15P075195	2.16	https://www.genecards.org/cgi-bin/carddisp.pl?gene=C15orf39
GC02M074152	2.16	https://www.genecards.org/cgi-bin/carddisp.pl?gene=MOB1A
GC15P041410	2.16	https://www.genecards.org/cgi-bin/carddisp.pl?gene=RTF1
GC04P000691	2.16	https://www.genecards.org/cgi-bin/carddisp.pl?gene=PCGF3
GC15M080955	2.16	https://www.genecards.org/cgi-bin/carddisp.pl?gene=MESD
GC0XP101098	2.16	https://www.genecards.org/cgi-bin/carddisp.pl?gene=CENPI
GC03P129278	2.16	https://www.genecards.org/cgi-bin/carddisp.pl?gene=HMCES
GC02P033436	2.16	https://www.genecards.org/cgi-bin/carddisp.pl?gene=RASGRP3
GC02P117875	2.16	https://www.genecards.org/cgi-bin/carddisp.pl?gene=DDX18
GC04M139028	2.16	https://www.genecards.org/cgi-bin/carddisp.pl?gene=ELF2
GC01P151170	2.16	https://www.genecards.org/cgi-bin/carddisp.pl?gene=PIP5K1A
GC13P046852	2.15	https://www.genecards.org/cgi-bin/carddisp.pl?gene=HTR2A-AS1
GC16P001245	2.15	https://www.genecards.org/cgi-bin/carddisp.pl?gene=RHBDL1
GC19M058577	2.15	https://www.genecards.org/cgi-bin/carddisp.pl?gene=MZF1
GC03P049803	2.15	https://www.genecards.org/cgi-bin/carddisp.pl?gene=INKA1
GC02P086199	2.15	https://www.genecards.org/cgi-bin/carddisp.pl?gene=MRPL35
GC22P037019	2.15	https://www.genecards.org/cgi-bin/carddisp.pl?gene=MPST
GC20P023439	2.15	https://www.genecards.org/cgi-bin/carddisp.pl?gene=CSTL1
GC01P024745	2.15	https://www.genecards.org/cgi-bin/carddisp.pl?gene=CLIC4
GC02M182140	2.15	https://www.genecards.org/cgi-bin/carddisp.pl?gene=PDE1A
GC01M020742	2.15	https://www.genecards.org/cgi-bin/carddisp.pl?gene=HP1BP3
GC01P171248	2.15	https://www.genecards.org/cgi-bin/carddisp.pl?gene=FMO1
GC11P021753	2.15	https://www.genecards.org/cgi-bin/carddisp.pl?gene=LOC102723370
GC04P087422	2.15	https://www.genecards.org/cgi-bin/carddisp.pl?gene=NUDT9
GC05P080407	2.15	https://www.genecards.org/cgi-bin/carddisp.pl?gene=ZFYVE16
GC01M222730	2.15	https://www.genecards.org/cgi-bin/carddisp.pl?gene=AIDA
GC13M095801	2.14	https://www.genecards.org/cgi-bin/carddisp.pl?gene=UGGT2
GC08P022258	2.14	https://www.genecards.org/cgi-bin/carddisp.pl?gene=POLR3D
GC16P084734	2.14	https://www.genecards.org/cgi-bin/carddisp.pl?gene=USP10
GC06M154154	2.14	https://www.genecards.org/cgi-bin/carddisp.pl?gene=IPCEF1
GC02M157318	2.14	https://www.genecards.org/cgi-bin/carddisp.pl?gene=ERMN
GC07P075842	2.14	https://www.genecards.org/cgi-bin/carddisp.pl?gene=RHBDD2
GC17P011241	2.14	https://www.genecards.org/cgi-bin/carddisp.pl?gene=SHISA6
GC10M070202	2.14	https://www.genecards.org/cgi-bin/carddisp.pl?gene=PPA1
GC10M044811	2.14	https://www.genecards.org/cgi-bin/carddisp.pl?gene=TMEM72-AS1
GC01M161120	2.14	https://www.genecards.org/cgi-bin/carddisp.pl?gene=DEDD
GC16M030105	2.14	https://www.genecards.org/cgi-bin/carddisp.pl?gene=GDPD3
GC05P120464	2.14	https://www.genecards.org/cgi-bin/carddisp.pl?gene=PRR16
GC22P023724	2.14	https://www.genecards.org/cgi-bin/carddisp.pl?gene=HIC2
GC05M073552	2.14	https://www.genecards.org/cgi-bin/carddisp.pl?gene=ANKRA2
GC03P179652	2.14	https://www.genecards.org/cgi-bin/carddisp.pl?gene=USP13
GC17M018658	2.14	https://www.genecards.org/cgi-bin/carddisp.pl?gene=ZNF286B

GC09P097983	2.13	https://www.genecards.org/cgi-bin/carddisp.pl?gene=ANP32B
GC01P153633	2.13	https://www.genecards.org/cgi-bin/carddisp.pl?gene=CHTOP
GC02P074549	2.13	https://www.genecards.org/cgi-bin/carddisp.pl?gene=DOK1
GC03M049936	2.13	https://www.genecards.org/cgi-bin/carddisp.pl?gene=CAMKV
GC15P048331	2.13	https://www.genecards.org/cgi-bin/carddisp.pl?gene=DUT
GC06P030617	2.13	https://www.genecards.org/cgi-bin/carddisp.pl?gene=MRPS18B
GC01P145824	2.13	https://www.genecards.org/cgi-bin/carddisp.pl?gene=POLR3C
GC12P130162	2.13	https://www.genecards.org/cgi-bin/carddisp.pl?gene=FZD10
GC0XP055452	2.13	https://www.genecards.org/cgi-bin/carddisp.pl?gene=MAGEH1
GC16M085771	2.13	https://www.genecards.org/cgi-bin/carddisp.pl?gene=EMC8
GC19M038815	2.13	https://www.genecards.org/cgi-bin/carddisp.pl?gene=ECH1
GC14P092794	2.13	https://www.genecards.org/cgi-bin/carddisp.pl?gene=GOLGA5
GC11P074526	2.12	https://www.genecards.org/cgi-bin/carddisp.pl?gene=POLD3
GC16M030530	2.12	https://www.genecards.org/cgi-bin/carddisp.pl?gene=ZNF747
GC08M040530	2.12	https://www.genecards.org/cgi-bin/carddisp.pl?gene=ZMAT4
GC06P013615	2.12	https://www.genecards.org/cgi-bin/carddisp.pl?gene=NOL7
GC16P077788	2.12	https://www.genecards.org/cgi-bin/carddisp.pl?gene=VAT1L
GC15M064165	2.12	https://www.genecards.org/cgi-bin/carddisp.pl?gene=CSNK1G1
GC13P030617	2.11	https://www.genecards.org/cgi-bin/carddisp.pl?gene=USPL1
GC02M234493	2.11	https://www.genecards.org/cgi-bin/carddisp.pl?gene=ARL4C
GC22P043110	2.11	https://www.genecards.org/cgi-bin/carddisp.pl?gene=BIK
GC12P064507	2.11	https://www.genecards.org/cgi-bin/carddisp.pl?gene=RASSF3
GC02M072943	2.11	https://www.genecards.org/cgi-bin/carddisp.pl?gene=SFXN5
GC17P027294	2.11	https://www.genecards.org/cgi-bin/carddisp.pl?gene=WSB1
GC14P077708	2.11	https://www.genecards.org/cgi-bin/carddisp.pl?gene=SLIRP
GC03M179017	2.11	https://www.genecards.org/cgi-bin/carddisp.pl?gene=ZMAT3
GC15M077100	2.11	https://www.genecards.org/cgi-bin/carddisp.pl?gene=PEAK1
GC12P107685	2.11	https://www.genecards.org/cgi-bin/carddisp.pl?gene=PWP1
GC0XM084058	2.11	https://www.genecards.org/cgi-bin/carddisp.pl?gene=RPS6KA6
GC18M035452	2.11	https://www.genecards.org/cgi-bin/carddisp.pl?gene=INO80C
GC01P031298	2.11	https://www.genecards.org/cgi-bin/carddisp.pl?gene=ZCCHC17
GC0XM119943	2.11	https://www.genecards.org/cgi-bin/carddisp.pl?gene=NKAP
GC09P095919	2.1	https://www.genecards.org/cgi-bin/carddisp.pl?gene=ENSG00000237631
GC09M099979	2.1	https://www.genecards.org/cgi-bin/carddisp.pl?gene=ERP44
GC05P070025	2.1	https://www.genecards.org/cgi-bin/carddisp.pl?gene=SERF1B
GC14M102932	2.1	https://www.genecards.org/cgi-bin/carddisp.pl?gene=CDC42BPB
GC04M015937	2.1	https://www.genecards.org/cgi-bin/carddisp.pl?gene=FGFBP1
GC18M035904	2.1	https://www.genecards.org/cgi-bin/carddisp.pl?gene=MIR187
GC03P185582	2.1	https://www.genecards.org/cgi-bin/carddisp.pl?gene=SENP2
GC13U990028	2.1	https://www.genecards.org/cgi-bin/carddisp.pl?gene=RNR1
GC19P022236	2.1	https://www.genecards.org/cgi-bin/carddisp.pl?gene=MAP1S
GC17P048049	2.1	https://www.genecards.org/cgi-bin/carddisp.pl?gene=NFE2L1
GC09M112683	2.1	https://www.genecards.org/cgi-bin/carddisp.pl?gene=INIP
GC01M167916	2.1	https://www.genecards.org/cgi-bin/carddisp.pl?gene=MPC2
GC16M030267	2.1	https://www.genecards.org/cgi-bin/carddisp.pl?gene=SMG1P5
GC12P113221	2.1	https://www.genecards.org/cgi-bin/carddisp.pl?gene=TPCN1
GC09P112379	2.1	https://www.genecards.org/cgi-bin/carddisp.pl?gene=HSDL2

GC02P070257	2.1	https://www.genecards.org/cgi-bin/carddisp.pl?gene=PCYOX1
GC17P007857	2.1	https://www.genecards.org/cgi-bin/carddisp.pl?gene=CYB5D1
GC04P176319	2.09	https://www.genecards.org/cgi-bin/carddisp.pl?gene=SPCS3
GC12P121400	2.09	https://www.genecards.org/cgi-bin/carddisp.pl?gene=RNF34
GC03M119654	2.09	https://www.genecards.org/cgi-bin/carddisp.pl?gene=COX17
GC16P002513	2.09	https://www.genecards.org/cgi-bin/carddisp.pl?gene=ATP6V0C
GC11P075716	2.09	https://www.genecards.org/cgi-bin/carddisp.pl?gene=SPCS2
GC0XM023834	2.09	https://www.genecards.org/cgi-bin/carddisp.pl?gene=APOO
GC10P133394	2.08	https://www.genecards.org/cgi-bin/carddisp.pl?gene=MTG1
GC19M041509	2.08	https://www.genecards.org/cgi-bin/carddisp.pl?gene=DMKN
GC17P006444	2.08	https://www.genecards.org/cgi-bin/carddisp.pl?gene=PIMREG
GC05M069364	2.08	https://www.genecards.org/cgi-bin/carddisp.pl?gene=TAF9
GC03P029393	2.08	https://www.genecards.org/cgi-bin/carddisp.pl?gene=RPS12P5
GC08P017027	2.08	https://www.genecards.org/cgi-bin/carddisp.pl?gene=MICU3
GC17M031316	2.08	https://www.genecards.org/cgi-bin/carddisp.pl?gene=EVI2A
GC03P028349	2.07	https://www.genecards.org/cgi-bin/carddisp.pl?gene=ENSG00000283563
GC03P052807	2.07	https://www.genecards.org/cgi-bin/carddisp.pl?gene=SPCS1
GC17M040019	2.07	https://www.genecards.org/cgi-bin/carddisp.pl?gene=MED24
GC05M016451	2.07	https://www.genecards.org/cgi-bin/carddisp.pl?gene=ZNF622
GC05P145936	2.07	https://www.genecards.org/cgi-bin/carddisp.pl?gene=SH3RF2
GC09P114665	2.07	https://www.genecards.org/cgi-bin/carddisp.pl?gene=ATP6V1G1
GC12M014942	2.07	https://www.genecards.org/cgi-bin/carddisp.pl?gene=ARHGDIB
GC03M127573	2.07	https://www.genecards.org/cgi-bin/carddisp.pl?gene=TPRA1
GC07P056140	2.06	https://www.genecards.org/cgi-bin/carddisp.pl?gene=SUMF2
GC11M000371	2.06	https://www.genecards.org/cgi-bin/carddisp.pl?gene=BET1L
GC11M122146	2.06	https://www.genecards.org/cgi-bin/carddisp.pl?gene=MIRLET7A2
GC06P029112	2.06	https://www.genecards.org/cgi-bin/carddisp.pl?gene=OR2J3
GC06P025845	2.06	https://www.genecards.org/cgi-bin/carddisp.pl?gene=H2BC1
GC17P017476	2.06	https://www.genecards.org/cgi-bin/carddisp.pl?gene=MED9
GC06M041326	2.06	https://www.genecards.org/cgi-bin/carddisp.pl?gene=KCNK17
GC03M047850	2.06	https://www.genecards.org/cgi-bin/carddisp.pl?gene=MAP4
GC04M010075	2.06	https://www.genecards.org/cgi-bin/carddisp.pl?gene=WDR1
GC17P078168	2.06	https://www.genecards.org/cgi-bin/carddisp.pl?gene=SYNGR2
GC0XM154506	2.05	https://www.genecards.org/cgi-bin/carddisp.pl?gene=FAM3A
GC20M032192	2.05	https://www.genecards.org/cgi-bin/carddisp.pl?gene=PLAGL2
GC07P102384	2.05	https://www.genecards.org/cgi-bin/carddisp.pl?gene=PRKRIP1
GC04P070902	2.05	https://www.genecards.org/cgi-bin/carddisp.pl?gene=MOB1B
GC12U902713	2.05	https://www.genecards.org/cgi-bin/carddisp.pl?gene=LOC109286556
GC07M108023	2.05	https://www.genecards.org/cgi-bin/carddisp.pl?gene=LAMB4
GC11M003088	2.05	https://www.genecards.org/cgi-bin/carddisp.pl?gene=OSBPL5
GC14M059458	2.05	https://www.genecards.org/cgi-bin/carddisp.pl?gene=L3HYPDH
GC05M152391	2.05	https://www.genecards.org/cgi-bin/carddisp.pl?gene=NMUR2
GC16M001093	2.05	https://www.genecards.org/cgi-bin/carddisp.pl?gene=CIAO3
GC19M014450	2.05	https://www.genecards.org/cgi-bin/carddisp.pl?gene=GIPC1
GC04P146177	2.05	https://www.genecards.org/cgi-bin/carddisp.pl?gene=LSM6
GC01M156258	2.05	https://www.genecards.org/cgi-bin/carddisp.pl?gene=SMG5
GC17M003804	2.05	https://www.genecards.org/cgi-bin/carddisp.pl?gene=NCBP3

GC17M064147	2.04	https://www.genecards.org/cgi-bin/carddisp.pl?gene=TEX2
GC01P161118	2.04	https://www.genecards.org/cgi-bin/carddisp.pl?gene=NIT1
GC01P220788	2.04	https://www.genecards.org/cgi-bin/carddisp.pl?gene=MTARC1
GC02M041850	2.04	https://www.genecards.org/cgi-bin/carddisp.pl?gene=RPS12P4
GC15P044288	2.04	https://www.genecards.org/cgi-bin/carddisp.pl?gene=CASC4
GC01P112619	2.04	https://www.genecards.org/cgi-bin/carddisp.pl?gene=CAPZA1
GC01M059987	2.04	https://www.genecards.org/cgi-bin/carddisp.pl?gene=C1orf87
GC12M033374	2.04	https://www.genecards.org/cgi-bin/carddisp.pl?gene=SYT10
GC02M042762	2.04	https://www.genecards.org/cgi-bin/carddisp.pl?gene=OXER1
GC01M025222	2.04	https://www.genecards.org/cgi-bin/carddisp.pl?gene=SYF2
GC08P028890	2.04	https://www.genecards.org/cgi-bin/carddisp.pl?gene=HMBOX1
GC04P008560	2.04	https://www.genecards.org/cgi-bin/carddisp.pl?gene=GPR78
GC08M029334	2.04	https://www.genecards.org/cgi-bin/carddisp.pl?gene=DUSP4
GC01P220748	2.04	https://www.genecards.org/cgi-bin/carddisp.pl?gene=MTARC2
GC03M112532	2.04	https://www.genecards.org/cgi-bin/carddisp.pl?gene=ATG3
GC14P094023	2.03	https://www.genecards.org/cgi-bin/carddisp.pl?gene=OTUB2
GC17M003860	2.03	https://www.genecards.org/cgi-bin/carddisp.pl?gene=CAMKK1
GC12P096194	2.03	https://www.genecards.org/cgi-bin/carddisp.pl?gene=ELK3
GC17M002405	2.03	https://www.genecards.org/cgi-bin/carddisp.pl?gene=METTL16
GC06P033277	2.03	https://www.genecards.org/cgi-bin/carddisp.pl?gene=B3GALT4
GC03M000363	2.03	https://www.genecards.org/cgi-bin/carddisp.pl?gene=CHL1-AS1
GC16P015398	2.03	https://www.genecards.org/cgi-bin/carddisp.pl?gene=MPV17L
GC12M006844	2.03	https://www.genecards.org/cgi-bin/carddisp.pl?gene=CDCA3
GC09P114329	2.03	https://www.genecards.org/cgi-bin/carddisp.pl?gene=ORM2
GC19P001941	2.03	https://www.genecards.org/cgi-bin/carddisp.pl?gene=CSNK1G2
GC20M031844	2.03	https://www.genecards.org/cgi-bin/carddisp.pl?gene=FOXS1
GC14P022829	2.03	https://www.genecards.org/cgi-bin/carddisp.pl?gene=MRPL52
GC15M074954	2.03	https://www.genecards.org/cgi-bin/carddisp.pl?gene=RPP25
GC07P043926	2.02	https://www.genecards.org/cgi-bin/carddisp.pl?gene=UBE2D4
GC08M070573	2.02	https://www.genecards.org/cgi-bin/carddisp.pl?gene=TRAM1
GC18M075195	2.02	https://www.genecards.org/cgi-bin/carddisp.pl?gene=ZADH2
GC14U990054	2.02	https://www.genecards.org/cgi-bin/carddisp.pl?gene=RNR2
GC01P000966	2.02	https://www.genecards.org/cgi-bin/carddisp.pl?gene=PLEKHN1
GC09P136993	2.02	https://www.genecards.org/cgi-bin/carddisp.pl?gene=PAXX
GC11P067303	2.02	https://www.genecards.org/cgi-bin/carddisp.pl?gene=SSH3
GC07P055956	2.02	https://www.genecards.org/cgi-bin/carddisp.pl?gene=NIPSNAP2
GC05M074627	2.02	https://www.genecards.org/cgi-bin/carddisp.pl?gene=ENC1
GC04M070815	2.02	https://www.genecards.org/cgi-bin/carddisp.pl?gene=GRSF1
GC02P038075	2.02	https://www.genecards.org/cgi-bin/carddisp.pl?gene=CYP1B1-AS1
GC18P073325	2.02	https://www.genecards.org/cgi-bin/carddisp.pl?gene=LINC02582
GC12P056162	2.02	https://www.genecards.org/cgi-bin/carddisp.pl?gene=ESYT1
GC01P212432	2.02	https://www.genecards.org/cgi-bin/carddisp.pl?gene=NENF
GC21M032574	2.02	https://www.genecards.org/cgi-bin/carddisp.pl?gene=TCP10L
GC07P129433	2.01	https://www.genecards.org/cgi-bin/carddisp.pl?gene=STRIP2
GC17M042160	2.01	https://www.genecards.org/cgi-bin/carddisp.pl?gene=KCNH4
GC05M034905	2.01	https://www.genecards.org/cgi-bin/carddisp.pl?gene=RAD1
GC04M098470	2.01	https://www.genecards.org/cgi-bin/carddisp.pl?gene=TSPAN5

GC01P010433	2.01	https://www.genecards.org/cgi-bin/carddisp.pl?gene=CENPS
GC11M063462	2.01	https://www.genecards.org/cgi-bin/carddisp.pl?gene=PLAAT5
GC02P096295	2.01	https://www.genecards.org/cgi-bin/carddisp.pl?gene=CIAO1
GC01P040041	2.01	https://www.genecards.org/cgi-bin/carddisp.pl?gene=CAP1
GC19M017049	2.01	https://www.genecards.org/cgi-bin/carddisp.pl?gene=HAUS8
GC11M057703	2.01	https://www.genecards.org/cgi-bin/carddisp.pl?gene=MED19
GC0XM135962	2.01	https://www.genecards.org/cgi-bin/carddisp.pl?gene=MMGT1
GC19M054155	2.01	https://www.genecards.org/cgi-bin/carddisp.pl?gene=LENG1
GC02M085344	2	https://www.genecards.org/cgi-bin/carddisp.pl?gene=RETSAT
GC19M005786	2	https://www.genecards.org/cgi-bin/carddisp.pl?gene=DUS3L
GC02P135741	2	https://www.genecards.org/cgi-bin/carddisp.pl?gene=UBXN4
GC0XM101268	2	https://www.genecards.org/cgi-bin/carddisp.pl?gene=TAF7L
GC11P074170	2	https://www.genecards.org/cgi-bin/carddisp.pl?gene=PPME1
GC17M076389	2	https://www.genecards.org/cgi-bin/carddisp.pl?gene=UBE2O
GC01M051287	2	https://www.genecards.org/cgi-bin/carddisp.pl?gene=TTC39A
GC04M105682	2	https://www.genecards.org/cgi-bin/carddisp.pl?gene=INTS12
GC12M049866	2	https://www.genecards.org/cgi-bin/carddisp.pl?gene=FAIM2
GC01M093866	2	https://www.genecards.org/cgi-bin/carddisp.pl?gene=DNTTIP2
GC01P161721	2	https://www.genecards.org/cgi-bin/carddisp.pl?gene=FCRLB
GC12M098613	2	https://www.genecards.org/cgi-bin/carddisp.pl?gene=IKBIP
GC20P033059	2	https://www.genecards.org/cgi-bin/carddisp.pl?gene=BPIFB3
GC07M087325	1.99	https://www.genecards.org/cgi-bin/carddisp.pl?gene=TP53TG1
GC14U900456	1.99	https://www.genecards.org/cgi-bin/carddisp.pl?gene=IBGC1
GC03M113442	1.99	https://www.genecards.org/cgi-bin/carddisp.pl?gene=SPICE1
GC19M008520	1.99	https://www.genecards.org/cgi-bin/carddisp.pl?gene=MYO1F
GC0XM002219	1.99	https://www.genecards.org/cgi-bin/carddisp.pl?gene=DHRSX
GC04P108650	1.99	https://www.genecards.org/cgi-bin/carddisp.pl?gene=OSTC
GC11P007491	1.99	https://www.genecards.org/cgi-bin/carddisp.pl?gene=PPFIBP2
GC20P022054	1.99	https://www.genecards.org/cgi-bin/carddisp.pl?gene=LINC01432
GC07M108147	1.99	https://www.genecards.org/cgi-bin/carddisp.pl?gene=NRCAM
GC19M047489	1.99	https://www.genecards.org/cgi-bin/carddisp.pl?gene=NAPA
GC0XM001403	1.99	https://www.genecards.org/cgi-bin/carddisp.pl?gene=ASMTL
GC06P053798	1.98	https://www.genecards.org/cgi-bin/carddisp.pl?gene=LRRC1
GC09M095759	1.98	https://www.genecards.org/cgi-bin/carddisp.pl?gene=LINC00476
GC06M033417	1.98	https://www.genecards.org/cgi-bin/carddisp.pl?gene=CUTA
GC07M099449	1.98	https://www.genecards.org/cgi-bin/carddisp.pl?gene=ATP5MF
GC16P056698	1.98	https://www.genecards.org/cgi-bin/carddisp.pl?gene=MT1X
GC17P028042	1.98	https://www.genecards.org/cgi-bin/carddisp.pl?gene=NLK
GC20P003049	1.98	https://www.genecards.org/cgi-bin/carddisp.pl?gene=GNRH2
GC17M008778	1.98	https://www.genecards.org/cgi-bin/carddisp.pl?gene=TRI-AAT4-1
GC17M008777	1.98	https://www.genecards.org/cgi-bin/carddisp.pl?gene=TRS-AGA2-6
GC17M008774	1.98	https://www.genecards.org/cgi-bin/carddisp.pl?gene=TRT-AGT1-2
GC11P058023	1.98	https://www.genecards.org/cgi-bin/carddisp.pl?gene=OR9Q1
GC02M074454	1.98	https://www.genecards.org/cgi-bin/carddisp.pl?gene=RTKN
GC07P044200	1.98	https://www.genecards.org/cgi-bin/carddisp.pl?gene=YKT6
GC16M002239	1.98	https://www.genecards.org/cgi-bin/carddisp.pl?gene=ECI1
GC17P030115	1.97	https://www.genecards.org/cgi-bin/carddisp.pl?gene=NSRP1

GC10P083648	1.97	https://www.genecards.org/cgi-bin/carddisp.pl?gene=PRXL2A
GC09M133097	1.97	https://www.genecards.org/cgi-bin/carddisp.pl?gene=RALGDS
GC14P021070	1.97	https://www.genecards.org/cgi-bin/carddisp.pl?gene=ARHGEF40
GC03P005122	1.97	https://www.genecards.org/cgi-bin/carddisp.pl?gene=ARL8B
GC09P035658	1.97	https://www.genecards.org/cgi-bin/carddisp.pl?gene=CCDC107
GC14P039175	1.97	https://www.genecards.org/cgi-bin/carddisp.pl?gene=PNN
GC15P041342	1.97	https://www.genecards.org/cgi-bin/carddisp.pl?gene=NUSAP1
GC08M010723	1.97	https://www.genecards.org/cgi-bin/carddisp.pl?gene=SOX7
GC19P049453	1.96	https://www.genecards.org/cgi-bin/carddisp.pl?gene=ALDH16A1
GC19P002360	1.96	https://www.genecards.org/cgi-bin/carddisp.pl?gene=TMPRSS9
GC04P041362	1.96	https://www.genecards.org/cgi-bin/carddisp.pl?gene=LIMCH1
GC09P068780	1.96	https://www.genecards.org/cgi-bin/carddisp.pl?gene=FAM122A
GC01P149904	1.96	https://www.genecards.org/cgi-bin/carddisp.pl?gene=BOLA1
GC0XP100820	1.96	https://www.genecards.org/cgi-bin/carddisp.pl?gene=CSTF2
GC12P006581	1.96	https://www.genecards.org/cgi-bin/carddisp.pl?gene=RAD51AP1
GC0XM152727	1.96	https://www.genecards.org/cgi-bin/carddisp.pl?gene=CSAG1
GC01P154527	1.96	https://www.genecards.org/cgi-bin/carddisp.pl?gene=UBE2Q1-AS1
GC01P154963	1.96	https://www.genecards.org/cgi-bin/carddisp.pl?gene=LOC101928120
GC15M064813	1.96	https://www.genecards.org/cgi-bin/carddisp.pl?gene=TPM1-AS
GC12M040141	1.96	https://www.genecards.org/cgi-bin/carddisp.pl?gene=LINC02555
GC03P059065	1.96	https://www.genecards.org/cgi-bin/carddisp.pl?gene=ENSG00000243295
GC12P040158	1.96	https://www.genecards.org/cgi-bin/carddisp.pl?gene=LINC02471
GC15M041448	1.96	https://www.genecards.org/cgi-bin/carddisp.pl?gene=RNU6-1169P
GC14M088010	1.96	https://www.genecards.org/cgi-bin/carddisp.pl?gene=ENSG00000258407
GC01M227975	1.96	https://www.genecards.org/cgi-bin/carddisp.pl?gene=CICP26
GC17M042440	1.96	https://www.genecards.org/cgi-bin/carddisp.pl?gene=RNU7-97P
GC01M155537	1.96	https://www.genecards.org/cgi-bin/carddisp.pl?gene=ENSG00000271267
GC07P144355	1.96	https://www.genecards.org/cgi-bin/carddisp.pl?gene=ARHGEF5
GC10M114281	1.96	https://www.genecards.org/cgi-bin/carddisp.pl?gene=AFAP1L2
GC14M075650	1.96	https://www.genecards.org/cgi-bin/carddisp.pl?gene=ERG28
GC20P044910	1.96	https://www.genecards.org/cgi-bin/carddisp.pl?gene=PABPC1L
GC05P177594	1.96	https://www.genecards.org/cgi-bin/carddisp.pl?gene=TMED9
GC05M177388	1.95	https://www.genecards.org/cgi-bin/carddisp.pl?gene=LMAN2
GC03M049925	1.95	https://www.genecards.org/cgi-bin/carddisp.pl?gene=MON1A
GC12M071609	1.95	https://www.genecards.org/cgi-bin/carddisp.pl?gene=ZFC3H1
GC14M024181	1.95	https://www.genecards.org/cgi-bin/carddisp.pl?gene=IPO4
GC03P113947	1.95	https://www.genecards.org/cgi-bin/carddisp.pl?gene=ZDHHC23
GC07M016854	1.95	https://www.genecards.org/cgi-bin/carddisp.pl?gene=AGR3
GC17M048133	1.95	https://www.genecards.org/cgi-bin/carddisp.pl?gene=SKAP1
GC04M078887	1.95	https://www.genecards.org/cgi-bin/carddisp.pl?gene=PAQR3
GC14P020343	1.95	https://www.genecards.org/cgi-bin/carddisp.pl?gene=PARP2
GC19P022233	1.95	https://www.genecards.org/cgi-bin/carddisp.pl?gene=PGLS
GC03P015427	1.95	https://www.genecards.org/cgi-bin/carddisp.pl?gene=EAF1
GC07P027739	1.94	https://www.genecards.org/cgi-bin/carddisp.pl?gene=TAX1BP1
GC19M018503	1.94	https://www.genecards.org/cgi-bin/carddisp.pl?gene=FKBP8
GC11P111927	1.94	https://www.genecards.org/cgi-bin/carddisp.pl?gene=DIXDC1
GC09M007798	1.94	https://www.genecards.org/cgi-bin/carddisp.pl?gene=DMAC1

GC09P137605	1.94	https://www.genecards.org/cgi-bin/carddisp.pl?gene=ARRDC1
GC11P073876	1.94	https://www.genecards.org/cgi-bin/carddisp.pl?gene=PAAF1
GC03P048918	1.94	https://www.genecards.org/cgi-bin/carddisp.pl?gene=ARIH2
GC12M043728	1.94	https://www.genecards.org/cgi-bin/carddisp.pl?gene=PUS7L
GC01P183636	1.94	https://www.genecards.org/cgi-bin/carddisp.pl?gene=RGL1
GC09M129639	1.94	https://www.genecards.org/cgi-bin/carddisp.pl?gene=ASB6
GC10P084328	1.94	https://www.genecards.org/cgi-bin/carddisp.pl?gene=CCSER2
GC20M044751	1.94	https://www.genecards.org/cgi-bin/carddisp.pl?gene=RIMS4
GC06P049431	1.94	https://www.genecards.org/cgi-bin/carddisp.pl?gene=CENPQ
GC09P129608	1.93	https://www.genecards.org/cgi-bin/carddisp.pl?gene=NTMT1
GC03P119597	1.93	https://www.genecards.org/cgi-bin/carddisp.pl?gene=PLA1A
GC16M067438	1.93	https://www.genecards.org/cgi-bin/carddisp.pl?gene=ATP6V0D1
GC07P016646	1.93	https://www.genecards.org/cgi-bin/carddisp.pl?gene=BZW2
GC12M029337	1.93	https://www.genecards.org/cgi-bin/carddisp.pl?gene=ERGIC2
GC03P047397	1.93	https://www.genecards.org/cgi-bin/carddisp.pl?gene=PTPN23
GC07M106090	1.92	https://www.genecards.org/cgi-bin/carddisp.pl?gene=SYPL1
GC16M056666	1.92	https://www.genecards.org/cgi-bin/carddisp.pl?gene=MT1G
GC09P072114	1.92	https://www.genecards.org/cgi-bin/carddisp.pl?gene=GDA
GC02P237085	1.92	https://www.genecards.org/cgi-bin/carddisp.pl?gene=COPS8
GC17M079833	1.92	https://www.genecards.org/cgi-bin/carddisp.pl?gene=CBX4
GC15M090929	1.92	https://www.genecards.org/cgi-bin/carddisp.pl?gene=HDDC3
GC14M095408	1.92	https://www.genecards.org/cgi-bin/carddisp.pl?gene=SYNE3
GC07M130668	1.92	https://www.genecards.org/cgi-bin/carddisp.pl?gene=TSGA13
GC14P051240	1.92	https://www.genecards.org/cgi-bin/carddisp.pl?gene=TMX1
GC11P066497	1.92	https://www.genecards.org/cgi-bin/carddisp.pl?gene=DPP3
GC01M156737	1.92	https://www.genecards.org/cgi-bin/carddisp.pl?gene=MRPL24
GC02P232550	1.91	https://www.genecards.org/cgi-bin/carddisp.pl?gene=EIF4E2
GC14M100539	1.91	https://www.genecards.org/cgi-bin/carddisp.pl?gene=BEGAIN
GC02M189770	1.91	https://www.genecards.org/cgi-bin/carddisp.pl?gene=ORMDL1
GC14P074303	1.91	https://www.genecards.org/cgi-bin/carddisp.pl?gene=VRTN
GC17P043398	1.91	https://www.genecards.org/cgi-bin/carddisp.pl?gene=ARL4D
GC06M165264	1.91	https://www.genecards.org/cgi-bin/carddisp.pl?gene=C6orf118
GC04M048887	1.91	https://www.genecards.org/cgi-bin/carddisp.pl?gene=OCIAD2
GC02P219229	1.91	https://www.genecards.org/cgi-bin/carddisp.pl?gene=ANKZF1
GC01P089821	1.91	https://www.genecards.org/cgi-bin/carddisp.pl?gene=LRRC8D
GC07M022853	1.91	https://www.genecards.org/cgi-bin/carddisp.pl?gene=TOMM7
GC12P021526	1.91	https://www.genecards.org/cgi-bin/carddisp.pl?gene=SPX
GC17P046511	1.91	https://www.genecards.org/cgi-bin/carddisp.pl?gene=LRRC37A2
GC02M134735	1.9	https://www.genecards.org/cgi-bin/carddisp.pl?gene=CCNT2-AS1
GC16M088863	1.9	https://www.genecards.org/cgi-bin/carddisp.pl?gene=PABPN1L
GC05M044499	1.9	https://www.genecards.org/cgi-bin/carddisp.pl?gene=LINC02224
GC01M001401	1.9	https://www.genecards.org/cgi-bin/carddisp.pl?gene=MRPL20
GC0XP047217	1.9	https://www.genecards.org/cgi-bin/carddisp.pl?gene=CDK16
GC03M118900	1.9	https://www.genecards.org/cgi-bin/carddisp.pl?gene=IGSF11
GC0XP109535	1.9	https://www.genecards.org/cgi-bin/carddisp.pl?gene=NXT2
GC01M052895	1.89	https://www.genecards.org/cgi-bin/carddisp.pl?gene=ECHDC2
GC04M108742	1.89	https://www.genecards.org/cgi-bin/carddisp.pl?gene=ETNPPL

GC17P063773	1.89	https://www.genecards.org/cgi-bin/carddisp.pl?gene=DDX42
GC01M153981	1.89	https://www.genecards.org/cgi-bin/carddisp.pl?gene=RAB13
GC17P045757	1.89	https://www.genecards.org/cgi-bin/carddisp.pl?gene=LINC02210-CRHR1
GC01M017406	1.89	https://www.genecards.org/cgi-bin/carddisp.pl?gene=RCC2
GC14M055148	1.89	https://www.genecards.org/cgi-bin/carddisp.pl?gene=DLGAP5
GC20M036751	1.89	https://www.genecards.org/cgi-bin/carddisp.pl?gene=DSN1
GC08M038504	1.89	https://www.genecards.org/cgi-bin/carddisp.pl?gene=C8orf86
GC02M232547	1.89	https://www.genecards.org/cgi-bin/carddisp.pl?gene=TIGD1
GC08P030133	1.89	https://www.genecards.org/cgi-bin/carddisp.pl?gene=DCTN6
GC01M162373	1.89	https://www.genecards.org/cgi-bin/carddisp.pl?gene=SPATA46
GC09M026840	1.88	https://www.genecards.org/cgi-bin/carddisp.pl?gene=CAAP1
GC01M054009	1.88	https://www.genecards.org/cgi-bin/carddisp.pl?gene=LDLRAD1
GC07M101613	1.88	https://www.genecards.org/cgi-bin/carddisp.pl?gene=MYL10
GC07P100049	1.88	https://www.genecards.org/cgi-bin/carddisp.pl?gene=ZSCAN21
GC01P023790	1.88	https://www.genecards.org/cgi-bin/carddisp.pl?gene=LYPLA2
GC15P051681	1.88	https://www.genecards.org/cgi-bin/carddisp.pl?gene=SCG3
GC03P009792	1.88	https://www.genecards.org/cgi-bin/carddisp.pl?gene=ARPC4
GC01M006634	1.87	https://www.genecards.org/cgi-bin/carddisp.pl?gene=DNAJC11
GC16P067806	1.87	https://www.genecards.org/cgi-bin/carddisp.pl?gene=TSNAXIP1
GC12P039626	1.87	https://www.genecards.org/cgi-bin/carddisp.pl?gene=C12orf40
GC01P211258	1.87	https://www.genecards.org/cgi-bin/carddisp.pl?gene=RCOR3
GC04P153344	1.87	https://www.genecards.org/cgi-bin/carddisp.pl?gene=MND1
GC20P054207	1.87	https://www.genecards.org/cgi-bin/carddisp.pl?gene=PFDN4
GC0XP050003	1.86	https://www.genecards.org/cgi-bin/carddisp.pl?gene=MIR188
GC11M059711	1.86	https://www.genecards.org/cgi-bin/carddisp.pl?gene=MTCH2
GC01M108816	1.86	https://www.genecards.org/cgi-bin/carddisp.pl?gene=AKNAD1
GC19M046468	1.86	https://www.genecards.org/cgi-bin/carddisp.pl?gene=PNMA8A
GC05P031639	1.86	https://www.genecards.org/cgi-bin/carddisp.pl?gene=PDZD2
GC01P028751	1.86	https://www.genecards.org/cgi-bin/carddisp.pl?gene=YTHDF2
GC04P015683	1.86	https://www.genecards.org/cgi-bin/carddisp.pl?gene=FAM200B
GC00U990582	1.86	https://www.genecards.org/cgi-bin/carddisp.pl?gene=DUX3
GC02P200810	1.86	https://www.genecards.org/cgi-bin/carddisp.pl?gene=BZW1
GC17M050862	1.86	https://www.genecards.org/cgi-bin/carddisp.pl?gene=TOB1
GC05P149141	1.86	https://www.genecards.org/cgi-bin/carddisp.pl?gene=ABLIM3
GC11P057713	1.86	https://www.genecards.org/cgi-bin/carddisp.pl?gene=TMX2
GC09M019047	1.86	https://www.genecards.org/cgi-bin/carddisp.pl?gene=HAUS6
GC01P152760	1.86	https://www.genecards.org/cgi-bin/carddisp.pl?gene=KPRP
GC08M070635	1.86	https://www.genecards.org/cgi-bin/carddisp.pl?gene=LACTB2
GC01M026876	1.86	https://www.genecards.org/cgi-bin/carddisp.pl?gene=GPN2
GC07P066682	1.86	https://www.genecards.org/cgi-bin/carddisp.pl?gene=RABGEF1
GC19P033080	1.85	https://www.genecards.org/cgi-bin/carddisp.pl?gene=GPATCH1
GC12P053990	1.85	https://www.genecards.org/cgi-bin/carddisp.pl?gene=HOXC6
GC15P072823	1.85	https://www.genecards.org/cgi-bin/carddisp.pl?gene=PLEKHO2
GC01M028199	1.85	https://www.genecards.org/cgi-bin/carddisp.pl?gene=DNAJC8
GC11M000859	1.85	https://www.genecards.org/cgi-bin/carddisp.pl?gene=CHID1
GC19M049739	1.85	https://www.genecards.org/cgi-bin/carddisp.pl?gene=TSKS
GC06P093707	1.84	https://www.genecards.org/cgi-bin/carddisp.pl?gene=TSG1

GC04M015961	1.84	https://www.genecards.org/cgi-bin/carddisp.pl?gene=FGFBP2
GC06P046029	1.84	https://www.genecards.org/cgi-bin/carddisp.pl?gene=DEF6
GC11M067435	1.84	https://www.genecards.org/cgi-bin/carddisp.pl?gene=CORO1B
GC08P142198	1.84	https://www.genecards.org/cgi-bin/carddisp.pl?gene=LINC00051
GC01P101323	1.84	https://www.genecards.org/cgi-bin/carddisp.pl?gene=LINC01307
GC05M134242	1.84	https://www.genecards.org/cgi-bin/carddisp.pl?gene=CDKL3
GC08P109334	1.84	https://www.genecards.org/cgi-bin/carddisp.pl?gene=ENY2
GC11P065279	1.84	https://www.genecards.org/cgi-bin/carddisp.pl?gene=POLA2
GC22M050274	1.83	https://www.genecards.org/cgi-bin/carddisp.pl?gene=PLXNB2
GC22P044172	1.83	https://www.genecards.org/cgi-bin/carddisp.pl?gene=PARVG
GC01M003889	1.83	https://www.genecards.org/cgi-bin/carddisp.pl?gene=C1orf174
GC11M059636	1.83	https://www.genecards.org/cgi-bin/carddisp.pl?gene=PATL1
GC09M034614	1.83	https://www.genecards.org/cgi-bin/carddisp.pl?gene=DCTN3
GC16P056652	1.83	https://www.genecards.org/cgi-bin/carddisp.pl?gene=MT1B
GC17M073283	1.83	https://www.genecards.org/cgi-bin/carddisp.pl?gene=CDC42EP4
GC01P203007	1.83	https://www.genecards.org/cgi-bin/carddisp.pl?gene=TMEM183A
GC15P044729	1.83	https://www.genecards.org/cgi-bin/carddisp.pl?gene=TRIM69
GC07P150450	1.83	https://www.genecards.org/cgi-bin/carddisp.pl?gene=GIMAP8
GC01M031907	1.83	https://www.genecards.org/cgi-bin/carddisp.pl?gene=PTP4A2
GC17M018665	1.83	https://www.genecards.org/cgi-bin/carddisp.pl?gene=FOXO3B
GC17P007945	1.83	https://www.genecards.org/cgi-bin/carddisp.pl?gene=SNORA48
GC17P007941	1.83	https://www.genecards.org/cgi-bin/carddisp.pl?gene=SNORD10
GC10P102438	1.83	https://www.genecards.org/cgi-bin/carddisp.pl?gene=RPARP-AS1
GC01P155561	1.83	https://www.genecards.org/cgi-bin/carddisp.pl?gene=ASH1L-AS1
GC07M023101	1.83	https://www.genecards.org/cgi-bin/carddisp.pl?gene=KLHL7-DT
GC17P042545	1.83	https://www.genecards.org/cgi-bin/carddisp.pl?gene=HSD17B1P1
GC12M040186	1.83	https://www.genecards.org/cgi-bin/carddisp.pl?gene=ENSG00000225342
GC17M007584	1.83	https://www.genecards.org/cgi-bin/carddisp.pl?gene=ENSG00000233223
GC08M011857	1.83	https://www.genecards.org/cgi-bin/carddisp.pl?gene=ENSG00000255046
GC07P023206	1.83	https://www.genecards.org/cgi-bin/carddisp.pl?gene=ENSG00000226816
GC17P008365	1.83	https://www.genecards.org/cgi-bin/carddisp.pl?gene=ENSG00000265749
GC04M076356	1.83	https://www.genecards.org/cgi-bin/carddisp.pl?gene=RNU6-1000P
GC01P155148	1.83	https://www.genecards.org/cgi-bin/carddisp.pl?gene=HMGN2P18
GC06M111494	1.83	https://www.genecards.org/cgi-bin/carddisp.pl?gene=ENSG00000220506
GC13M097675	1.83	https://www.genecards.org/cgi-bin/carddisp.pl?gene=ENSG00000226134
GC06P111309	1.83	https://www.genecards.org/cgi-bin/carddisp.pl?gene=ENSG00000272356
GC02M101844	1.83	https://www.genecards.org/cgi-bin/carddisp.pl?gene=piR-38051-158
GC08M115643	1.83	https://www.genecards.org/cgi-bin/carddisp.pl?gene=RF00994-1086
GC07M023006	1.83	https://www.genecards.org/cgi-bin/carddisp.pl?gene=lnc-IGF2BP3-1
GC01M226732	1.83	https://www.genecards.org/cgi-bin/carddisp.pl?gene=lnc-CDC42BPA-5
GC15M041495	1.83	https://www.genecards.org/cgi-bin/carddisp.pl?gene=lnc-LTK-1
GC18M073711	1.83	https://www.genecards.org/cgi-bin/carddisp.pl?gene=ENSG00000265380
GC08P115576	1.83	https://www.genecards.org/cgi-bin/carddisp.pl?gene=lnc-UTP23-10
GC01M226691	1.83	https://www.genecards.org/cgi-bin/carddisp.pl?gene=LOC105373117
GC04P015743	1.83	https://www.genecards.org/cgi-bin/carddisp.pl?gene=ENSG00000248188
GC15M039594	1.83	https://www.genecards.org/cgi-bin/carddisp.pl?gene=FSIP1
GC01M084498	1.82	https://www.genecards.org/cgi-bin/carddisp.pl?gene=GNG5

GC0XP118974	1.82	https://www.genecards.org/cgi-bin/carddisp.pl?gene=LONRF3
GC11M108473	1.82	https://www.genecards.org/cgi-bin/carddisp.pl?gene=POGLUT3
GC02M196133	1.82	https://www.genecards.org/cgi-bin/carddisp.pl?gene=STK17B
GC19P022223	1.82	https://www.genecards.org/cgi-bin/carddisp.pl?gene=USE1
GC01M205577	1.82	https://www.genecards.org/cgi-bin/carddisp.pl?gene=ELK4
GC10P083203	1.82	https://www.genecards.org/cgi-bin/carddisp.pl?gene=TSPAN14
GC20P064257	1.82	https://www.genecards.org/cgi-bin/carddisp.pl?gene=TCEA2
GC16P021244	1.82	https://www.genecards.org/cgi-bin/carddisp.pl?gene=ANKS4B
GC0XP050202	1.82	https://www.genecards.org/cgi-bin/carddisp.pl?gene=CCNB3
GC10M011453	1.82	https://www.genecards.org/cgi-bin/carddisp.pl?gene=USP6NL
GC17P047831	1.81	https://www.genecards.org/cgi-bin/carddisp.pl?gene=LRRC46
GC19M001690	1.81	https://www.genecards.org/cgi-bin/carddisp.pl?gene=MBD3
GC06P003118	1.81	https://www.genecards.org/cgi-bin/carddisp.pl?gene=BPHL
GC13M027066	1.81	https://www.genecards.org/cgi-bin/carddisp.pl?gene=USP12
GC06P043182	1.81	https://www.genecards.org/cgi-bin/carddisp.pl?gene=CUL9
GC07P005898	1.81	https://www.genecards.org/cgi-bin/carddisp.pl?gene=CCZ1
GC17M064542	1.81	https://www.genecards.org/cgi-bin/carddisp.pl?gene=SMURF2
GC19M002714	1.81	https://www.genecards.org/cgi-bin/carddisp.pl?gene=DIRAS1
GC06M118959	1.81	https://www.genecards.org/cgi-bin/carddisp.pl?gene=FAM184A
GC03P123067	1.81	https://www.genecards.org/cgi-bin/carddisp.pl?gene=PDIA5
GC01M038885	1.81	https://www.genecards.org/cgi-bin/carddisp.pl?gene=RHBDL2
GC12P118376	1.81	https://www.genecards.org/cgi-bin/carddisp.pl?gene=SUDS3
GC17P038705	1.81	https://www.genecards.org/cgi-bin/carddisp.pl?gene=MLLT6
GC13P077535	1.81	https://www.genecards.org/cgi-bin/carddisp.pl?gene=SCEL
GC0XP118823	1.81	https://www.genecards.org/cgi-bin/carddisp.pl?gene=ZCCHC12
GC16M067662	1.81	https://www.genecards.org/cgi-bin/carddisp.pl?gene=ENKD1
GC16M024931	1.8	https://www.genecards.org/cgi-bin/carddisp.pl?gene=ARHGAP17
GC13P099643	1.8	https://www.genecards.org/cgi-bin/carddisp.pl?gene=MIR4306
GC22M031105	1.8	https://www.genecards.org/cgi-bin/carddisp.pl?gene=SELENOM
GC04P000673	1.8	https://www.genecards.org/cgi-bin/carddisp.pl?gene=MYL5
GC02P186486	1.8	https://www.genecards.org/cgi-bin/carddisp.pl?gene=ZC3H15
GC17M049700	1.8	https://www.genecards.org/cgi-bin/carddisp.pl?gene=SLC35B1
GC14P104753	1.8	https://www.genecards.org/cgi-bin/carddisp.pl?gene=SIVA1
GC16P056670	1.8	https://www.genecards.org/cgi-bin/carddisp.pl?gene=MT1H
GC03M050287	1.8	https://www.genecards.org/cgi-bin/carddisp.pl?gene=IFRD2
GC0XM048973	1.8	https://www.genecards.org/cgi-bin/carddisp.pl?gene=GRIPAP1
GC12M053709	1.8	https://www.genecards.org/cgi-bin/carddisp.pl?gene=CALCOCO1
GC16M003022	1.79	https://www.genecards.org/cgi-bin/carddisp.pl?gene=HCFC1R1
GC16P001826	1.79	https://www.genecards.org/cgi-bin/carddisp.pl?gene=FAHD1
GC06P107029	1.79	https://www.genecards.org/cgi-bin/carddisp.pl?gene=MTRES1
GC08P116767	1.79	https://www.genecards.org/cgi-bin/carddisp.pl?gene=UTP23
GC20P050731	1.79	https://www.genecards.org/cgi-bin/carddisp.pl?gene=PARD6B
GC01P150258	1.79	https://www.genecards.org/cgi-bin/carddisp.pl?gene=CA14
GC06M154160	1.79	https://www.genecards.org/cgi-bin/carddisp.pl?gene=ENSG00000288520
GC11P067403	1.79	https://www.genecards.org/cgi-bin/carddisp.pl?gene=TBC1D10C
GC05M035905	1.79	https://www.genecards.org/cgi-bin/carddisp.pl?gene=CAPSL
GC18M005954	1.79	https://www.genecards.org/cgi-bin/carddisp.pl?gene=L3MBTL4

GC09M136994	1.79	https://www.genecards.org/cgi-bin/carddisp.pl?gene=CLIC3
GC11P065386	1.78	https://www.genecards.org/cgi-bin/carddisp.pl?gene=FRMD8
GC10M122930	1.78	https://www.genecards.org/cgi-bin/carddisp.pl?gene=C10orf88
GC03M068714	1.78	https://www.genecards.org/cgi-bin/carddisp.pl?gene=TAFA4
GC02M131464	1.78	https://www.genecards.org/cgi-bin/carddisp.pl?gene=MZT2A
GC0XM048893	1.78	https://www.genecards.org/cgi-bin/carddisp.pl?gene=TIMM17B
GC03M048292	1.78	https://www.genecards.org/cgi-bin/carddisp.pl?gene=NME6
GC07P016759	1.78	https://www.genecards.org/cgi-bin/carddisp.pl?gene=TSPAN13
GC12M123410	1.78	https://www.genecards.org/cgi-bin/carddisp.pl?gene=RILPL2
GC09P136848	1.78	https://www.genecards.org/cgi-bin/carddisp.pl?gene=PHPT1
GC03M063911	1.78	https://www.genecards.org/cgi-bin/carddisp.pl?gene=SCAANT1
GC19M051002	1.78	https://www.genecards.org/cgi-bin/carddisp.pl?gene=KLK9
GC19P037715	1.77	https://www.genecards.org/cgi-bin/carddisp.pl?gene=RBM42
GC12P054549	1.77	https://www.genecards.org/cgi-bin/carddisp.pl?gene=PDE1B
GC01M153974	1.77	https://www.genecards.org/cgi-bin/carddisp.pl?gene=JTB
GC02P053767	1.77	https://www.genecards.org/cgi-bin/carddisp.pl?gene=CHAC2
GC15M070829	1.77	https://www.genecards.org/cgi-bin/carddisp.pl?gene=LARP6
GC03P150546	1.77	https://www.genecards.org/cgi-bin/carddisp.pl?gene=EIF2A
GC01P161606	1.76	https://www.genecards.org/cgi-bin/carddisp.pl?gene=HSPA7
GC19M013989	1.76	https://www.genecards.org/cgi-bin/carddisp.pl?gene=C19orf57
GC02P029097	1.76	https://www.genecards.org/cgi-bin/carddisp.pl?gene=CLIP4
GC19P022494	1.76	https://www.genecards.org/cgi-bin/carddisp.pl?gene=MVB12A
GC15P049620	1.75	https://www.genecards.org/cgi-bin/carddisp.pl?gene=DTWD1
GC12M057723	1.75	https://www.genecards.org/cgi-bin/carddisp.pl?gene=AGAP2
GC05P080319	1.75	https://www.genecards.org/cgi-bin/carddisp.pl?gene=SPZ1
GC20M045372	1.75	https://www.genecards.org/cgi-bin/carddisp.pl?gene=TP53TG5
GC01M044635	1.75	https://www.genecards.org/cgi-bin/carddisp.pl?gene=TMEM53
GC08P140511	1.75	https://www.genecards.org/cgi-bin/carddisp.pl?gene=CHRAC1
GC05P181222	1.75	https://www.genecards.org/cgi-bin/carddisp.pl?gene=TRIM41
GC13M044939	1.75	https://www.genecards.org/cgi-bin/carddisp.pl?gene=NUFIP1
GC07M043868	1.75	https://www.genecards.org/cgi-bin/carddisp.pl?gene=MRPS24
GC05M133955	1.75	https://www.genecards.org/cgi-bin/carddisp.pl?gene=C5orf15
GC06M041282	1.74	https://www.genecards.org/cgi-bin/carddisp.pl?gene=TCP11
GC08P022599	1.74	https://www.genecards.org/cgi-bin/carddisp.pl?gene=C8orf58
GC17M008769	1.74	https://www.genecards.org/cgi-bin/carddisp.pl?gene=LINC00324
GC14P058650	1.74	https://www.genecards.org/cgi-bin/carddisp.pl?gene=LINC01500
GC01M046557	1.74	https://www.genecards.org/cgi-bin/carddisp.pl?gene=MKNK1
GC01M036455	1.74	https://www.genecards.org/cgi-bin/carddisp.pl?gene=MRPS15
GC03P051951	1.74	https://www.genecards.org/cgi-bin/carddisp.pl?gene=PARP3
GC02P231781	1.74	https://www.genecards.org/cgi-bin/carddisp.pl?gene=COPS7B
GC05P065722	1.74	https://www.genecards.org/cgi-bin/carddisp.pl?gene=NLN
GC07M150625	1.74	https://www.genecards.org/cgi-bin/carddisp.pl?gene=GIMAP6
GC01P045786	1.74	https://www.genecards.org/cgi-bin/carddisp.pl?gene=MAST2
GC20M033659	1.74	https://www.genecards.org/cgi-bin/carddisp.pl?gene=NECAB3
GC21P037208	1.74	https://www.genecards.org/cgi-bin/carddisp.pl?gene=DSCR9
GC10P095194	1.74	https://www.genecards.org/cgi-bin/carddisp.pl?gene=ACSM6
GC0XP085003	1.74	https://www.genecards.org/cgi-bin/carddisp.pl?gene=APOOL

GC06P106361	1.74	https://www.genecards.org/cgi-bin/carddisp.pl?gene=CRYBG1
GC04M104468	1.74	https://www.genecards.org/cgi-bin/carddisp.pl?gene=CXXC4
GC01P246724	1.73	https://www.genecards.org/cgi-bin/carddisp.pl?gene=SCCPDH
GC09P114642	1.73	https://www.genecards.org/cgi-bin/carddisp.pl?gene=RGS3
GC12P053180	1.73	https://www.genecards.org/cgi-bin/carddisp.pl?gene=ZNF740
GC12M118149	1.73	https://www.genecards.org/cgi-bin/carddisp.pl?gene=TAOK3
GC07M112762	1.73	https://www.genecards.org/cgi-bin/carddisp.pl?gene=TMEM168
GC05P073498	1.73	https://www.genecards.org/cgi-bin/carddisp.pl?gene=BTF3
GC07P129284	1.73	https://www.genecards.org/cgi-bin/carddisp.pl?gene=METTL2B
GC21P043865	1.73	https://www.genecards.org/cgi-bin/carddisp.pl?gene=AGPAT3
GC22M027482	1.73	https://www.genecards.org/cgi-bin/carddisp.pl?gene=GUCD1
GC16M030222	1.73	https://www.genecards.org/cgi-bin/carddisp.pl?gene=NPIPB13
GC16M031086	1.73	https://www.genecards.org/cgi-bin/carddisp.pl?gene=ENSG00000255439
GC14M020311	1.73	https://www.genecards.org/cgi-bin/carddisp.pl?gene=CCNB1IP1
GC03M015412	1.72	https://www.genecards.org/cgi-bin/carddisp.pl?gene=CHCHD4
GC06P046092	1.72	https://www.genecards.org/cgi-bin/carddisp.pl?gene=NFYA
GC17P082716	1.72	https://www.genecards.org/cgi-bin/carddisp.pl?gene=FN3KRP
GC03P045294	1.72	https://www.genecards.org/cgi-bin/carddisp.pl?gene=EXOSC7
GC07P002638	1.72	https://www.genecards.org/cgi-bin/carddisp.pl?gene=TTYH3
GC14M099397	1.71	https://www.genecards.org/cgi-bin/carddisp.pl?gene=SETD3
GC05M043446	1.71	https://www.genecards.org/cgi-bin/carddisp.pl?gene=TMEM267
GC17M050375	1.71	https://www.genecards.org/cgi-bin/carddisp.pl?gene=LRRC59
GC07M151081	1.71	https://www.genecards.org/cgi-bin/carddisp.pl?gene=TMUB1
GC06M132404	1.71	https://www.genecards.org/cgi-bin/carddisp.pl?gene=STX7
GC11P033018	1.71	https://www.genecards.org/cgi-bin/carddisp.pl?gene=TCP11L1
GC01M051907	1.71	https://www.genecards.org/cgi-bin/carddisp.pl?gene=RAB3B
GC0XM023629	1.71	https://www.genecards.org/cgi-bin/carddisp.pl?gene=ACOT9
GC17M082654	1.7	https://www.genecards.org/cgi-bin/carddisp.pl?gene=RAB40B
GC12P049366	1.7	https://www.genecards.org/cgi-bin/carddisp.pl?gene=SPATS2
GC0XP102247	1.7	https://www.genecards.org/cgi-bin/carddisp.pl?gene=NXF2
GC06U990032	1.7	https://www.genecards.org/cgi-bin/carddisp.pl?gene=FRA6E
GC06M132644	1.7	https://www.genecards.org/cgi-bin/carddisp.pl?gene=TAAR1
GC07M156388	1.7	https://www.genecards.org/cgi-bin/carddisp.pl?gene=LINC01006
GC09M123110	1.7	https://www.genecards.org/cgi-bin/carddisp.pl?gene=STRBP
GC09P033817	1.7	https://www.genecards.org/cgi-bin/carddisp.pl?gene=UBE2R2
GC06M007268	1.7	https://www.genecards.org/cgi-bin/carddisp.pl?gene=SSR1
GC01P044799	1.7	https://www.genecards.org/cgi-bin/carddisp.pl?gene=PLK3
GC06P004021	1.7	https://www.genecards.org/cgi-bin/carddisp.pl?gene=PRPF4B
GC10M097222	1.7	https://www.genecards.org/cgi-bin/carddisp.pl?gene=ARHGAP19
GC14M072969	1.7	https://www.genecards.org/cgi-bin/carddisp.pl?gene=ZFYVE1
GC16P019297	1.69	https://www.genecards.org/cgi-bin/carddisp.pl?gene=CLEC19A
GC06M111903	1.69	https://www.genecards.org/cgi-bin/carddisp.pl?gene=LINC02527
GC01M228407	1.69	https://www.genecards.org/cgi-bin/carddisp.pl?gene=TRIM17
GC02P202912	1.69	https://www.genecards.org/cgi-bin/carddisp.pl?gene=CARF
GC12P057591	1.69	https://www.genecards.org/cgi-bin/carddisp.pl?gene=PIP4K2C
GC17P006757	1.69	https://www.genecards.org/cgi-bin/carddisp.pl?gene=XAF1
GC20P045934	1.69	https://www.genecards.org/cgi-bin/carddisp.pl?gene=PCIF1

GC21P045643	1.69	https://www.genecards.org/cgi-bin/carddisp.pl?gene=PCBP3
GC02M219237	1.69	https://www.genecards.org/cgi-bin/carddisp.pl?gene=GLB1L
GC05P142876	1.69	https://www.genecards.org/cgi-bin/carddisp.pl?gene=PCDHGA12
GC01M012848	1.69	https://www.genecards.org/cgi-bin/carddisp.pl?gene=HNRNPCL1
GC12P008032	1.69	https://www.genecards.org/cgi-bin/carddisp.pl?gene=FOXJ2
GC19P022237	1.69	https://www.genecards.org/cgi-bin/carddisp.pl?gene=FCHO1
GC16P067844	1.69	https://www.genecards.org/cgi-bin/carddisp.pl?gene=THAP11
GC21P036385	1.69	https://www.genecards.org/cgi-bin/carddisp.pl?gene=CHAF1B
GC01M046632	1.68	https://www.genecards.org/cgi-bin/carddisp.pl?gene=ATPAF1
GC11M008937	1.68	https://www.genecards.org/cgi-bin/carddisp.pl?gene=ASCL3
GC16P067109	1.68	https://www.genecards.org/cgi-bin/carddisp.pl?gene=C16orf70
GC01P113929	1.68	https://www.genecards.org/cgi-bin/carddisp.pl?gene=HIPK1
GC0XM153785	1.68	https://www.genecards.org/cgi-bin/carddisp.pl?gene=IDH3G
GC22P037807	1.68	https://www.genecards.org/cgi-bin/carddisp.pl?gene=GCAT
GC01M003059	1.68	https://www.genecards.org/cgi-bin/carddisp.pl?gene=PRDM16-DT
GC12P056158	1.68	https://www.genecards.org/cgi-bin/carddisp.pl?gene=MYL6
GC02M096836	1.67	https://www.genecards.org/cgi-bin/carddisp.pl?gene=ANKRD39
GC17M081229	1.67	https://www.genecards.org/cgi-bin/carddisp.pl?gene=TEPSIN
GC07P006793	1.67	https://www.genecards.org/cgi-bin/carddisp.pl?gene=RSPH10B2
GC11P073787	1.67	https://www.genecards.org/cgi-bin/carddisp.pl?gene=MRPL48
GC01P165600	1.67	https://www.genecards.org/cgi-bin/carddisp.pl?gene=MGST3
GC12M123289	1.67	https://www.genecards.org/cgi-bin/carddisp.pl?gene=SBNO1
GC13M106541	1.67	https://www.genecards.org/cgi-bin/carddisp.pl?gene=ARGLU1
GC0XM135055	1.66	https://www.genecards.org/cgi-bin/carddisp.pl?gene=RTL8A
GC12M103770	1.66	https://www.genecards.org/cgi-bin/carddisp.pl?gene=NT5DC3
GC13P042272	1.66	https://www.genecards.org/cgi-bin/carddisp.pl?gene=AKAP11
GC03P014674	1.66	https://www.genecards.org/cgi-bin/carddisp.pl?gene=C3orf20
GC15P040569	1.66	https://www.genecards.org/cgi-bin/carddisp.pl?gene=RPUSD2
GC15M065045	1.66	https://www.genecards.org/cgi-bin/carddisp.pl?gene=RASL12
GC22P046576	1.66	https://www.genecards.org/cgi-bin/carddisp.pl?gene=GRAMD4
GC05P142832	1.66	https://www.genecards.org/cgi-bin/carddisp.pl?gene=PCDHA7
GC17P075667	1.66	https://www.genecards.org/cgi-bin/carddisp.pl?gene=SAP30BP
GC22M045190	1.66	https://www.genecards.org/cgi-bin/carddisp.pl?gene=KIAA0930
GC07P029563	1.66	https://www.genecards.org/cgi-bin/carddisp.pl?gene=PRR15
GC02M171317	1.66	https://www.genecards.org/cgi-bin/carddisp.pl?gene=METTL8
GC21M031671	1.66	https://www.genecards.org/cgi-bin/carddisp.pl?gene=SCAF4
GC12M120443	1.66	https://www.genecards.org/cgi-bin/carddisp.pl?gene=TRIAP1
GC02P218356	1.65	https://www.genecards.org/cgi-bin/carddisp.pl?gene=CATIP
GC19M048047	1.65	https://www.genecards.org/cgi-bin/carddisp.pl?gene=PLA2G4C
GC01P045583	1.65	https://www.genecards.org/cgi-bin/carddisp.pl?gene=NASP
GC0XP102720	1.65	https://www.genecards.org/cgi-bin/carddisp.pl?gene=BHLHB9
GC05M138263	1.65	https://www.genecards.org/cgi-bin/carddisp.pl?gene=GFRA3
GC16P031408	1.65	https://www.genecards.org/cgi-bin/carddisp.pl?gene=ZNF646
GC18M035366	1.65	https://www.genecards.org/cgi-bin/carddisp.pl?gene=ZNF396
GC07M076390	1.65	https://www.genecards.org/cgi-bin/carddisp.pl?gene=SSC4D
GC21M041711	1.65	https://www.genecards.org/cgi-bin/carddisp.pl?gene=LINC00479
GC21P041716	1.65	https://www.genecards.org/cgi-bin/carddisp.pl?gene=LINC00112

GC06M028282	1.65	https://www.genecards.org/cgi-bin/carddisp.pl?gene=ZSCAN16-AS1
GC06M111046	1.65	https://www.genecards.org/cgi-bin/carddisp.pl?gene=GSTM2P1
GC03P161428	1.65	https://www.genecards.org/cgi-bin/carddisp.pl?gene=LINC02067
GC14M054878	1.65	https://www.genecards.org/cgi-bin/carddisp.pl?gene=MIR4308
GC17M017383	1.65	https://www.genecards.org/cgi-bin/carddisp.pl?gene=RPL13P12
GC06P028100	1.65	https://www.genecards.org/cgi-bin/carddisp.pl?gene=LINC01012
GC08M040300	1.65	https://www.genecards.org/cgi-bin/carddisp.pl?gene=SIRLNT
GC11M057917	1.65	https://www.genecards.org/cgi-bin/carddisp.pl?gene=OR5AZ1P
GC14P053169	1.65	https://www.genecards.org/cgi-bin/carddisp.pl?gene=ENSG00000237356
GC12P042648	1.65	https://www.genecards.org/cgi-bin/carddisp.pl?gene=LINC02451
GC09P035096	1.65	https://www.genecards.org/cgi-bin/carddisp.pl?gene=ENSG00000234181
GC08M039903	1.65	https://www.genecards.org/cgi-bin/carddisp.pl?gene=ENSG00000253939
GC08P040161	1.65	https://www.genecards.org/cgi-bin/carddisp.pl?gene=ENSG00000254143
GC03M118004	1.65	https://www.genecards.org/cgi-bin/carddisp.pl?gene=ENSG00000243276
GC09M017551	1.65	https://www.genecards.org/cgi-bin/carddisp.pl?gene=PABPC1P11
GC05P180418	1.65	https://www.genecards.org/cgi-bin/carddisp.pl?gene=ENSG00000250509
GC06M027736	1.65	https://www.genecards.org/cgi-bin/carddisp.pl?gene=GPR89P
GC12M106497	1.65	https://www.genecards.org/cgi-bin/carddisp.pl?gene=ENSG00000257545
GC17M008776	1.65	https://www.genecards.org/cgi-bin/carddisp.pl?gene=TRD-GTC2-11
GC16P031054	1.65	https://www.genecards.org/cgi-bin/carddisp.pl?gene=ENSG00000260911
GC17M008773	1.65	https://www.genecards.org/cgi-bin/carddisp.pl?gene=TRP-CGG1-3
GC16M031456	1.65	https://www.genecards.org/cgi-bin/carddisp.pl?gene=ENSG00000260267
GC16M031193	1.65	https://www.genecards.org/cgi-bin/carddisp.pl?gene=ENSG00000260304
GC17M008775	1.65	https://www.genecards.org/cgi-bin/carddisp.pl?gene=TRW-CCA1-1
GC17P008221	1.65	https://www.genecards.org/cgi-bin/carddisp.pl?gene=TRG-TCC3-1
GC15M080345	1.65	https://www.genecards.org/cgi-bin/carddisp.pl?gene=ENSG00000259495
GC21P041870	1.65	https://www.genecards.org/cgi-bin/carddisp.pl?gene=ENSG00000236545
GC15M081303	1.65	https://www.genecards.org/cgi-bin/carddisp.pl?gene=ENSG00000271725
GC18M035443	1.65	https://www.genecards.org/cgi-bin/carddisp.pl?gene=ENSG00000267583
GC03M161328	1.65	https://www.genecards.org/cgi-bin/carddisp.pl?gene=PSMC1P7
GC04P089410	1.65	https://www.genecards.org/cgi-bin/carddisp.pl?gene=ENSG00000251095
GC08M016598	1.65	https://www.genecards.org/cgi-bin/carddisp.pl?gene=ENSG00000253496
GC08P012195	1.65	https://www.genecards.org/cgi-bin/carddisp.pl?gene=ENSG00000255495
GC03M161324	1.65	https://www.genecards.org/cgi-bin/carddisp.pl?gene=EEF1GP4
GC04P113420	1.65	https://www.genecards.org/cgi-bin/carddisp.pl?gene=RNU1-138P
GC01P155219	1.65	https://www.genecards.org/cgi-bin/carddisp.pl?gene=ENSG00000231064
GC11M059791	1.65	https://www.genecards.org/cgi-bin/carddisp.pl?gene=OR5BD1P
GC02M188598	1.65	https://www.genecards.org/cgi-bin/carddisp.pl?gene=ENSG00000223523
GC01M205731	1.65	https://www.genecards.org/cgi-bin/carddisp.pl?gene=ENSG00000201944
GC14P055748	1.65	https://www.genecards.org/cgi-bin/carddisp.pl?gene=ENSG00000258784
GC14M053686	1.65	https://www.genecards.org/cgi-bin/carddisp.pl?gene=LINC02331
GC16P030534	1.65	https://www.genecards.org/cgi-bin/carddisp.pl?gene=LOC107984875
GC15M061639	1.65	https://www.genecards.org/cgi-bin/carddisp.pl?gene=ENSG00000259675
GC17P042536	1.65	https://www.genecards.org/cgi-bin/carddisp.pl?gene=ENSG00000266929
GC17P008277	1.65	https://www.genecards.org/cgi-bin/carddisp.pl?gene=ENSG00000269947
GC08M008456	1.65	https://www.genecards.org/cgi-bin/carddisp.pl?gene=ENSG00000254153
GC10M044591	1.65	https://www.genecards.org/cgi-bin/carddisp.pl?gene=ENSG00000234580

GC08M011347	1.65	https://www.genecards.org/cgi-bin/carddisp.pl?gene=ENSG00000255020
GC04M067418	1.65	https://www.genecards.org/cgi-bin/carddisp.pl?gene=ENSG00000250075
GC13P097475	1.65	https://www.genecards.org/cgi-bin/carddisp.pl?gene=PSMA6P4
GC15P079236	1.65	https://www.genecards.org/cgi-bin/carddisp.pl?gene=HNRNPCP3
GC15P036571	1.65	https://www.genecards.org/cgi-bin/carddisp.pl?gene=COX6CP4
GC02P168451	1.65	https://www.genecards.org/cgi-bin/carddisp.pl?gene=RN7SL813P
GC14P046996	1.65	https://www.genecards.org/cgi-bin/carddisp.pl?gene=RPA2P1
GC01P205625	1.65	https://www.genecards.org/cgi-bin/carddisp.pl?gene=LOC100420878
GC21M041441	1.65	https://www.genecards.org/cgi-bin/carddisp.pl?gene=ENSG00000228318
GC20M028558	1.65	https://www.genecards.org/cgi-bin/carddisp.pl?gene=FRG1CP
GC14M059135	1.65	https://www.genecards.org/cgi-bin/carddisp.pl?gene=AKR1B1P5
GC01P205776	1.65	https://www.genecards.org/cgi-bin/carddisp.pl?gene=ENSG00000285521
GC03M059598	1.65	https://www.genecards.org/cgi-bin/carddisp.pl?gene=LOC339902
GC16P052547	1.65	https://www.genecards.org/cgi-bin/carddisp.pl?gene=lnc-CHD9-4
GC04M076240	1.65	https://www.genecards.org/cgi-bin/carddisp.pl?gene=ENSG00000287401
GC15M027684	1.65	https://www.genecards.org/cgi-bin/carddisp.pl?gene=ENSG00000258853
GC12P032988	1.65	https://www.genecards.org/cgi-bin/carddisp.pl?gene=ENSG00000257435
GC01P101640	1.65	https://www.genecards.org/cgi-bin/carddisp.pl?gene=LINC01709
GC17P046035	1.65	https://www.genecards.org/cgi-bin/carddisp.pl?gene=ENSG00000262372
GC08M011846	1.65	https://www.genecards.org/cgi-bin/carddisp.pl?gene=ENSG00000269899
GC08M008414	1.65	https://www.genecards.org/cgi-bin/carddisp.pl?gene=ENSG00000253505
GC17M046773	1.65	https://www.genecards.org/cgi-bin/carddisp.pl?gene=lnc-WNT3-1
GC09P074372	1.65	https://www.genecards.org/cgi-bin/carddisp.pl?gene=ENSG00000227809
GC01P205716	1.65	https://www.genecards.org/cgi-bin/carddisp.pl?gene=lnc-MFSD4A-1
GC10P119621	1.65	https://www.genecards.org/cgi-bin/carddisp.pl?gene=RAD1P1
GC05P126854	1.65	https://www.genecards.org/cgi-bin/carddisp.pl?gene=lnc-LMNB1-1
GC02P168248	1.65	https://www.genecards.org/cgi-bin/carddisp.pl?gene=lnc-CERS6-1
GC10P102830	1.65	https://www.genecards.org/cgi-bin/carddisp.pl?gene=CYP17A1-AS1-001
GC14M054843	1.65	https://www.genecards.org/cgi-bin/carddisp.pl?gene=lnc-WDHD1-3
GC21P041362	1.65	https://www.genecards.org/cgi-bin/carddisp.pl?gene=lnc-FAM3B-3
GC01P155137	1.65	https://www.genecards.org/cgi-bin/carddisp.pl?gene=HSALNG0007472
GC01P155124	1.65	https://www.genecards.org/cgi-bin/carddisp.pl?gene=piR-42491-008
GC06M154397	1.65	https://www.genecards.org/cgi-bin/carddisp.pl?gene=lnc-IPCEF1-6
GC11P084639	1.65	https://www.genecards.org/cgi-bin/carddisp.pl?gene=RPL9P22
GC17P045991	1.65	https://www.genecards.org/cgi-bin/carddisp.pl?gene=NONHSAG022015.2
GC06P033688	1.65	https://www.genecards.org/cgi-bin/carddisp.pl?gene=NONHSAG045793.2
GC04P076253	1.65	https://www.genecards.org/cgi-bin/carddisp.pl?gene=NONHSAG038251.2
GC12P040225	1.65	https://www.genecards.org/cgi-bin/carddisp.pl?gene=NONHSAG010911.2
GC10P127873	1.65	https://www.genecards.org/cgi-bin/carddisp.pl?gene=HSALNG0081495
GC10P127884	1.65	https://www.genecards.org/cgi-bin/carddisp.pl?gene=HSALNG0081496
GC17M045988	1.65	https://www.genecards.org/cgi-bin/carddisp.pl?gene=lnc-KANSL1-4
GC17M008804	1.65	https://www.genecards.org/cgi-bin/carddisp.pl?gene=lnc-CTC1-2
GC01P155132	1.65	https://www.genecards.org/cgi-bin/carddisp.pl?gene=MG604298
GC05M126632	1.65	https://www.genecards.org/cgi-bin/carddisp.pl?gene=RF00017-4854
GC02M101710	1.65	https://www.genecards.org/cgi-bin/carddisp.pl?gene=lnc-RFX8-3
GC09M017590	1.65	https://www.genecards.org/cgi-bin/carddisp.pl?gene=lnc-BNC2-5
GC21M041828	1.65	https://www.genecards.org/cgi-bin/carddisp.pl?gene=lnc-C2CD2-3

GC16M031084	1.65	https://www.genecards.org/cgi-bin/carddisp.pl?gene=lnc-PRSS53-1
GC06M032435	1.65	https://www.genecards.org/cgi-bin/carddisp.pl?gene=lnc-BTNL2-2
GC07M076336	1.65	https://www.genecards.org/cgi-bin/carddisp.pl?gene=RF00017-6393
GC07P076388	1.65	https://www.genecards.org/cgi-bin/carddisp.pl?gene=RF00017-6395
GC01P155128	1.65	https://www.genecards.org/cgi-bin/carddisp.pl?gene=AB372576
GC01M205795	1.65	https://www.genecards.org/cgi-bin/carddisp.pl?gene=lnc-RAB29-1
GC03P161263	1.65	https://www.genecards.org/cgi-bin/carddisp.pl?gene=lnc-NMD3-2
GC10M102182	1.65	https://www.genecards.org/cgi-bin/carddisp.pl?gene=L13705-001
GC07P023480	1.65	https://www.genecards.org/cgi-bin/carddisp.pl?gene=ENSG00000236654
GC04M015565	1.65	https://www.genecards.org/cgi-bin/carddisp.pl?gene=ENSG00000273133
GC15P081335	1.65	https://www.genecards.org/cgi-bin/carddisp.pl?gene=ENSG00000273920
GC10P102213	1.65	https://www.genecards.org/cgi-bin/carddisp.pl?gene=lnc-ELOVL3-1
GC04P067608	1.65	https://www.genecards.org/cgi-bin/carddisp.pl?gene=piR-50444-343
GC17M008822	1.65	https://www.genecards.org/cgi-bin/carddisp.pl?gene=piR-36037-004
GC08P011850	1.65	https://www.genecards.org/cgi-bin/carddisp.pl?gene=piR-45438
GC01P205777	1.65	https://www.genecards.org/cgi-bin/carddisp.pl?gene=piR-37170-008
GC16P031452	1.65	https://www.genecards.org/cgi-bin/carddisp.pl?gene=piR-37106
GC08M039998	1.65	https://www.genecards.org/cgi-bin/carddisp.pl?gene=ENSG00000253790
GC21M041392	1.65	https://www.genecards.org/cgi-bin/carddisp.pl?gene=piR-38351-289
GC01P206529	1.65	https://www.genecards.org/cgi-bin/carddisp.pl?gene=ENSG00000234981
GC08M011877	1.65	https://www.genecards.org/cgi-bin/carddisp.pl?gene=lnc-DEFB136-3
GC04M000963	1.65	https://www.genecards.org/cgi-bin/carddisp.pl?gene=piR-34930
GC13M046892	1.65	https://www.genecards.org/cgi-bin/carddisp.pl?gene=lnc-ESD-2
GC08M011881	1.65	https://www.genecards.org/cgi-bin/carddisp.pl?gene=piR-59109
GC08M022636	1.65	https://www.genecards.org/cgi-bin/carddisp.pl?gene=lnc-EGR3-4
GC04M000879	1.65	https://www.genecards.org/cgi-bin/carddisp.pl?gene=piR-51036
GC01M156069	1.65	https://www.genecards.org/cgi-bin/carddisp.pl?gene=DQ866751
GC08P011849	1.65	https://www.genecards.org/cgi-bin/carddisp.pl?gene=piR-43401
GC17M017811	1.65	https://www.genecards.org/cgi-bin/carddisp.pl?gene=lnc-TOM1L2-7
GC01M232578	1.65	https://www.genecards.org/cgi-bin/carddisp.pl?gene=piR-59412-001
GC14M059168	1.65	https://www.genecards.org/cgi-bin/carddisp.pl?gene=piR-43106-068
GC08M011880	1.65	https://www.genecards.org/cgi-bin/carddisp.pl?gene=piR-43134
GC08P011843	1.65	https://www.genecards.org/cgi-bin/carddisp.pl?gene=piR-54316
GC01P232500	1.65	https://www.genecards.org/cgi-bin/carddisp.pl?gene=RF00994-103
GC10M069806	1.65	https://www.genecards.org/cgi-bin/carddisp.pl?gene=piR-35674-050
GC08P011844	1.65	https://www.genecards.org/cgi-bin/carddisp.pl?gene=lnc-FDFT1-1
GC06P111972	1.65	https://www.genecards.org/cgi-bin/carddisp.pl?gene=lnc-WISP3-8
GC04M015732	1.65	https://www.genecards.org/cgi-bin/carddisp.pl?gene=ENSG00000214846
GC08M040212	1.65	https://www.genecards.org/cgi-bin/carddisp.pl?gene=HSALNG0064740
GC05P060946	1.65	https://www.genecards.org/cgi-bin/carddisp.pl?gene=NONHSAG040481.2
GC17M008823	1.65	https://www.genecards.org/cgi-bin/carddisp.pl?gene=piR-36441-001
GC05P061032	1.65	https://www.genecards.org/cgi-bin/carddisp.pl?gene=piR-60985-091
GC07P076550	1.65	https://www.genecards.org/cgi-bin/carddisp.pl?gene=ENSG00000205485
GC15P071995	1.65	https://www.genecards.org/cgi-bin/carddisp.pl?gene=NONHSAG017121.2
GC17M008825	1.65	https://www.genecards.org/cgi-bin/carddisp.pl?gene=piR-37213
GC04M000882	1.65	https://www.genecards.org/cgi-bin/carddisp.pl?gene=lnc-CPLX1-12
GC04P000991	1.65	https://www.genecards.org/cgi-bin/carddisp.pl?gene=piR-50308-469

GC09M035186	1.65	https://www.genecards.org/cgi-bin/carddisp.pl?gene=piR-57845-019
GC08M040176	1.65	https://www.genecards.org/cgi-bin/carddisp.pl?gene=piR-57801-025
GC09M035272	1.65	https://www.genecards.org/cgi-bin/carddisp.pl?gene=RF00017-7528
GC06P154477	1.65	https://www.genecards.org/cgi-bin/carddisp.pl?gene=ENSG00000286553
GC08P011842	1.65	https://www.genecards.org/cgi-bin/carddisp.pl?gene=piR-32246
GC17P017806	1.65	https://www.genecards.org/cgi-bin/carddisp.pl?gene=piR-32250
GC10M102831	1.65	https://www.genecards.org/cgi-bin/carddisp.pl?gene=lnc-ARL3-2
GC08P022669	1.65	https://www.genecards.org/cgi-bin/carddisp.pl?gene=lnc-CCAR2-5
GC04P015745	1.65	https://www.genecards.org/cgi-bin/carddisp.pl?gene=lnc-BST1-1
GC15M064603	1.65	https://www.genecards.org/cgi-bin/carddisp.pl?gene=lnc-RPS27L-8
GC17M046770	1.65	https://www.genecards.org/cgi-bin/carddisp.pl?gene=piR-57133-264
GC07M023269	1.65	https://www.genecards.org/cgi-bin/carddisp.pl?gene=piR-57133-539
GC17M008787	1.65	https://www.genecards.org/cgi-bin/carddisp.pl?gene=AB330774-009
GC01M156071	1.65	https://www.genecards.org/cgi-bin/carddisp.pl?gene=RF00017-362
GC08P011848	1.65	https://www.genecards.org/cgi-bin/carddisp.pl?gene=piR-32606
GC05P126845	1.65	https://www.genecards.org/cgi-bin/carddisp.pl?gene=piR-48950-167
GC12P040050	1.65	https://www.genecards.org/cgi-bin/carddisp.pl?gene=lnc-C12orf40-2
GC15M079668	1.65	https://www.genecards.org/cgi-bin/carddisp.pl?gene=LOC390618
GC01P161959	1.65	https://www.genecards.org/cgi-bin/carddisp.pl?gene=lnc-NOS1AP-2
GC01M161943	1.65	https://www.genecards.org/cgi-bin/carddisp.pl?gene=piR-31199-012
GC03M161368	1.65	https://www.genecards.org/cgi-bin/carddisp.pl?gene=L13713-165
GC18M035403	1.65	https://www.genecards.org/cgi-bin/carddisp.pl?gene=ENSG00000275512
GC15P079743	1.65	https://www.genecards.org/cgi-bin/carddisp.pl?gene=ENSG00000260674
GC17P046793	1.65	https://www.genecards.org/cgi-bin/carddisp.pl?gene=RF00017-2418
GC08M011854	1.65	https://www.genecards.org/cgi-bin/carddisp.pl?gene=piR-42219
GC14M054883	1.65	https://www.genecards.org/cgi-bin/carddisp.pl?gene=hsa-miR-5095-131
GC04P089732	1.65	https://www.genecards.org/cgi-bin/carddisp.pl?gene=RF02541-018
GC08M022656	1.65	https://www.genecards.org/cgi-bin/carddisp.pl?gene=LOC105369180
GC11M032853	1.65	https://www.genecards.org/cgi-bin/carddisp.pl?gene=lnc-CCDC73-1
GC12P040000	1.65	https://www.genecards.org/cgi-bin/carddisp.pl?gene=piR-61157-011
GC11M133865	1.65	https://www.genecards.org/cgi-bin/carddisp.pl?gene=ENSG00000213153
GC02P168260	1.65	https://www.genecards.org/cgi-bin/carddisp.pl?gene=piR-50357-077
GC09M101108	1.65	https://www.genecards.org/cgi-bin/carddisp.pl?gene=MG828668-125
GC11M022010	1.65	https://www.genecards.org/cgi-bin/carddisp.pl?gene=ENSG00000287962
GC09P101132	1.65	https://www.genecards.org/cgi-bin/carddisp.pl?gene=piR-55809-038
GC20M013368	1.65	https://www.genecards.org/cgi-bin/carddisp.pl?gene=ENSG00000278153
GC16P052581	1.65	https://www.genecards.org/cgi-bin/carddisp.pl?gene=LOC105371265
GC04P089722	1.65	https://www.genecards.org/cgi-bin/carddisp.pl?gene=piR-61945-390
GC01P205775	1.65	https://www.genecards.org/cgi-bin/carddisp.pl?gene=LOC105371702
GC04P000872	1.65	https://www.genecards.org/cgi-bin/carddisp.pl?gene=LOC105374341
GC04M076285	1.65	https://www.genecards.org/cgi-bin/carddisp.pl?gene=piR-37895-114
GC10M069808	1.65	https://www.genecards.org/cgi-bin/carddisp.pl?gene=LOC105378347
GC09M073871	1.65	https://www.genecards.org/cgi-bin/carddisp.pl?gene=ENSG00000223368
GC11M032879	1.65	https://www.genecards.org/cgi-bin/carddisp.pl?gene=piR-32214-089
GC04M076276	1.65	https://www.genecards.org/cgi-bin/carddisp.pl?gene=RF00017-4334
GC15M079669	1.65	https://www.genecards.org/cgi-bin/carddisp.pl?gene=ENSG00000259208
GC05M010679	1.65	https://www.genecards.org/cgi-bin/carddisp.pl?gene=DAP

GC06P033163	1.65	https://www.genecards.org/cgi-bin/carddisp.pl?gene=HLA-V
GC18P013069	1.64	https://www.genecards.org/cgi-bin/carddisp.pl?gene=PRELID3A
GC12P082358	1.64	https://www.genecards.org/cgi-bin/carddisp.pl?gene=METTL25
GC12P056117	1.64	https://www.genecards.org/cgi-bin/carddisp.pl?gene=RPL41
GC14M064945	1.64	https://www.genecards.org/cgi-bin/carddisp.pl?gene=RAB15
GC17M076672	1.64	https://www.genecards.org/cgi-bin/carddisp.pl?gene=MXRA7
GC02P132458	1.64	https://www.genecards.org/cgi-bin/carddisp.pl?gene=POTEE
GC04M000673	1.64	https://www.genecards.org/cgi-bin/carddisp.pl?gene=ATP5ME
GC04P168081	1.63	https://www.genecards.org/cgi-bin/carddisp.pl?gene=ANXA10
GC01M147630	1.63	https://www.genecards.org/cgi-bin/carddisp.pl?gene=ACP6
GC04P183504	1.63	https://www.genecards.org/cgi-bin/carddisp.pl?gene=ING2
GC17P045132	1.63	https://www.genecards.org/cgi-bin/carddisp.pl?gene=ACBD4
GC01M231223	1.63	https://www.genecards.org/cgi-bin/carddisp.pl?gene=C1orf131
GC20M013389	1.63	https://www.genecards.org/cgi-bin/carddisp.pl?gene=TASP1
GC03M050310	1.63	https://www.genecards.org/cgi-bin/carddisp.pl?gene=NAA80
GC14P023953	1.63	https://www.genecards.org/cgi-bin/carddisp.pl?gene=DHRS4
GC16M021957	1.63	https://www.genecards.org/cgi-bin/carddisp.pl?gene=PDZD9
GC11M003248	1.63	https://www.genecards.org/cgi-bin/carddisp.pl?gene=MRGPRE
GC03M150541	1.63	https://www.genecards.org/cgi-bin/carddisp.pl?gene=SERP1
GC07M107653	1.62	https://www.genecards.org/cgi-bin/carddisp.pl?gene=SLC26A4-AS1
GC12M014503	1.62	https://www.genecards.org/cgi-bin/carddisp.pl?gene=PLBD1
GC0XP013652	1.62	https://www.genecards.org/cgi-bin/carddisp.pl?gene=TCEANC
GC12P101877	1.62	https://www.genecards.org/cgi-bin/carddisp.pl?gene=DRAM1
GC16M008780	1.62	https://www.genecards.org/cgi-bin/carddisp.pl?gene=TMEM186
GC08P047998	1.62	https://www.genecards.org/cgi-bin/carddisp.pl?gene=UBE2V2
GC10M097677	1.62	https://www.genecards.org/cgi-bin/carddisp.pl?gene=AVPI1
GC19P022228	1.62	https://www.genecards.org/cgi-bin/carddisp.pl?gene=MRPL34
GC06M025827	1.62	https://www.genecards.org/cgi-bin/carddisp.pl?gene=H2AC1
GC14M054474	1.62	https://www.genecards.org/cgi-bin/carddisp.pl?gene=GMFB
GC06P033226	1.62	https://www.genecards.org/cgi-bin/carddisp.pl?gene=TRIM40
GC09P036159	1.62	https://www.genecards.org/cgi-bin/carddisp.pl?gene=CCIN
GC01M185088	1.61	https://www.genecards.org/cgi-bin/carddisp.pl?gene=TRMT1L
GC15M072751	1.61	https://www.genecards.org/cgi-bin/carddisp.pl?gene=ADPGK
GC01M016059	1.61	https://www.genecards.org/cgi-bin/carddisp.pl?gene=FAM131C
GC19P003933	1.61	https://www.genecards.org/cgi-bin/carddisp.pl?gene=NMRK2
GC19M012865	1.61	https://www.genecards.org/cgi-bin/carddisp.pl?gene=TRIR
GC11P064230	1.61	https://www.genecards.org/cgi-bin/carddisp.pl?gene=DNAJC4
GC05M094184	1.61	https://www.genecards.org/cgi-bin/carddisp.pl?gene=KIAA0825
GC03M151425	1.61	https://www.genecards.org/cgi-bin/carddisp.pl?gene=IGSF10
GC09M112217	1.61	https://www.genecards.org/cgi-bin/carddisp.pl?gene=PTBP3
GC08P144079	1.61	https://www.genecards.org/cgi-bin/carddisp.pl?gene=EXOSC4
GC09P100576	1.61	https://www.genecards.org/cgi-bin/carddisp.pl?gene=CAVIN4
GC20P035542	1.61	https://www.genecards.org/cgi-bin/carddisp.pl?gene=ERGIC3
GC15M052547	1.61	https://www.genecards.org/cgi-bin/carddisp.pl?gene=ARPP19
GC07P116811	1.6	https://www.genecards.org/cgi-bin/carddisp.pl?gene=CAPZA2
GC03P008518	1.6	https://www.genecards.org/cgi-bin/carddisp.pl?gene=LMCD1
GC17M015627	1.6	https://www.genecards.org/cgi-bin/carddisp.pl?gene=TRIM16

GC02M207580	1.6	https://www.genecards.org/cgi-bin/carddisp.pl?gene=METTL21A
GC06M089568	1.6	https://www.genecards.org/cgi-bin/carddisp.pl?gene=LYRM2
GC02P095405	1.6	https://www.genecards.org/cgi-bin/carddisp.pl?gene=FAHD2A
GC06P010887	1.6	https://www.genecards.org/cgi-bin/carddisp.pl?gene=SYCP2L
GC15M042067	1.6	https://www.genecards.org/cgi-bin/carddisp.pl?gene=PLA2G4D
GC01P026289	1.6	https://www.genecards.org/cgi-bin/carddisp.pl?gene=SH3BGRL3
GC19P038619	1.6	https://www.genecards.org/cgi-bin/carddisp.pl?gene=EIF3K
GC19M056221	1.59	https://www.genecards.org/cgi-bin/carddisp.pl?gene=ZSCAN5A
GC17P008339	1.59	https://www.genecards.org/cgi-bin/carddisp.pl?gene=ODF4
GC15M034084	1.59	https://www.genecards.org/cgi-bin/carddisp.pl?gene=EMC7
GC19P050476	1.59	https://www.genecards.org/cgi-bin/carddisp.pl?gene=EMC10
GC02P219177	1.59	https://www.genecards.org/cgi-bin/carddisp.pl?gene=RETREG2
GC0XM074287	1.59	https://www.genecards.org/cgi-bin/carddisp.pl?gene=MIR545
GC01P016441	1.58	https://www.genecards.org/cgi-bin/carddisp.pl?gene=NECAP2
GC11P066466	1.58	https://www.genecards.org/cgi-bin/carddisp.pl?gene=PELI3
GC01P203305	1.58	https://www.genecards.org/cgi-bin/carddisp.pl?gene=BTG2
GC18P059139	1.58	https://www.genecards.org/cgi-bin/carddisp.pl?gene=SEC11C
GC14P105472	1.58	https://www.genecards.org/cgi-bin/carddisp.pl?gene=CRIP2
GC01P035807	1.58	https://www.genecards.org/cgi-bin/carddisp.pl?gene=AGO4
GC16M030093	1.58	https://www.genecards.org/cgi-bin/carddisp.pl?gene=YPEL3
GC12P123457	1.58	https://www.genecards.org/cgi-bin/carddisp.pl?gene=SNRNP35
GC0XP151563	1.58	https://www.genecards.org/cgi-bin/carddisp.pl?gene=PASD1
GC19M055452	1.58	https://www.genecards.org/cgi-bin/carddisp.pl?gene=ISOC2
GC21P044600	1.57	https://www.genecards.org/cgi-bin/carddisp.pl?gene=KRTAP10-7
GC0XP101622	1.57	https://www.genecards.org/cgi-bin/carddisp.pl?gene=ARMCX3
GC02P009834	1.57	https://www.genecards.org/cgi-bin/carddisp.pl?gene=TAF1B
GC04M007060	1.57	https://www.genecards.org/cgi-bin/carddisp.pl?gene=GRPEL1
GC01M181289	1.57	https://www.genecards.org/cgi-bin/carddisp.pl?gene=ACBD6
GC12P123602	1.57	https://www.genecards.org/cgi-bin/carddisp.pl?gene=DDX55
GC06M134917	1.57	https://www.genecards.org/cgi-bin/carddisp.pl?gene=ALDH8A1
GC09P128920	1.57	https://www.genecards.org/cgi-bin/carddisp.pl?gene=PHYHD1
GC07P036153	1.57	https://www.genecards.org/cgi-bin/carddisp.pl?gene=EEPD1
GC19P032719	1.56	https://www.genecards.org/cgi-bin/carddisp.pl?gene=TDRD12
GC21M032268	1.56	https://www.genecards.org/cgi-bin/carddisp.pl?gene=MIS18A
GC01P194159	1.56	https://www.genecards.org/cgi-bin/carddisp.pl?gene=EEF1A1P14
GC07P055365	1.56	https://www.genecards.org/cgi-bin/carddisp.pl?gene=LANCL2
GC19P022202	1.56	https://www.genecards.org/cgi-bin/carddisp.pl?gene=FAM32A
GC22P050343	1.55	https://www.genecards.org/cgi-bin/carddisp.pl?gene=PPP6R2
GC06P028433	1.55	https://www.genecards.org/cgi-bin/carddisp.pl?gene=ZSCAN16
GC16M057155	1.55	https://www.genecards.org/cgi-bin/carddisp.pl?gene=PSME3IP1
GC05P134649	1.55	https://www.genecards.org/cgi-bin/carddisp.pl?gene=SEC24A
GC02M074471	1.55	https://www.genecards.org/cgi-bin/carddisp.pl?gene=MRPL53
GC01M031630	1.55	https://www.genecards.org/cgi-bin/carddisp.pl?gene=PEF1
GC12M013365	1.55	https://www.genecards.org/cgi-bin/carddisp.pl?gene=CLEC2B
GC11P011819	1.54	https://www.genecards.org/cgi-bin/carddisp.pl?gene=USP47
GC17P035731	1.54	https://www.genecards.org/cgi-bin/carddisp.pl?gene=RASL10B
GC03P050350	1.54	https://www.genecards.org/cgi-bin/carddisp.pl?gene=CYB561D2

GC06M055407	1.54	https://www.genecards.org/cgi-bin/carddisp.pl?gene=HMGCLL1
GC15P067254	1.54	https://www.genecards.org/cgi-bin/carddisp.pl?gene=IQCH
GC01M048729	1.54	https://www.genecards.org/cgi-bin/carddisp.pl?gene=BEND5
GC17P075109	1.54	https://www.genecards.org/cgi-bin/carddisp.pl?gene=ARMC7
GC16P056565	1.53	https://www.genecards.org/cgi-bin/carddisp.pl?gene=MT4
GC0XP155197	1.53	https://www.genecards.org/cgi-bin/carddisp.pl?gene=VBP1
GC12P116910	1.53	https://www.genecards.org/cgi-bin/carddisp.pl?gene=FBXW8
GC20M013714	1.53	https://www.genecards.org/cgi-bin/carddisp.pl?gene=ESF1
GC12P027523	1.53	https://www.genecards.org/cgi-bin/carddisp.pl?gene=PPFIBP1
GC11M073872	1.53	https://www.genecards.org/cgi-bin/carddisp.pl?gene=COA4
GC11M008391	1.53	https://www.genecards.org/cgi-bin/carddisp.pl?gene=STK33
GC03M023891	1.53	https://www.genecards.org/cgi-bin/carddisp.pl?gene=NKIRAS1
GC11P065332	1.52	https://www.genecards.org/cgi-bin/carddisp.pl?gene=CDC42EP2
GC07P099408	1.52	https://www.genecards.org/cgi-bin/carddisp.pl?gene=BUD31
GC21M029004	1.52	https://www.genecards.org/cgi-bin/carddisp.pl?gene=RWDD2B
GC05M179862	1.52	https://www.genecards.org/cgi-bin/carddisp.pl?gene=TBC1D9B
GC12M025443	1.52	https://www.genecards.org/cgi-bin/carddisp.pl?gene=LMNTD1
GC19P001026	1.51	https://www.genecards.org/cgi-bin/carddisp.pl?gene=CNN2
GC16P089973	1.51	https://www.genecards.org/cgi-bin/carddisp.pl?gene=AFG3L1P
GC16P031716	1.51	https://www.genecards.org/cgi-bin/carddisp.pl?gene=ZNF720
GC19P046297	1.51	https://www.genecards.org/cgi-bin/carddisp.pl?gene=HIF3A
GC0XP010015	1.51	https://www.genecards.org/cgi-bin/carddisp.pl?gene=WWC3
GC12M070516	1.51	https://www.genecards.org/cgi-bin/carddisp.pl?gene=PTPRB
GC16M067828	1.51	https://www.genecards.org/cgi-bin/carddisp.pl?gene=CENPT
GC02P120013	1.51	https://www.genecards.org/cgi-bin/carddisp.pl?gene=EPB41L5
GC17P026155	1.51	https://www.genecards.org/cgi-bin/carddisp.pl?gene=DHRS7B
GC0XP129781	1.51	https://www.genecards.org/cgi-bin/carddisp.pl?gene=SASH3
GC06M043450	1.51	https://www.genecards.org/cgi-bin/carddisp.pl?gene=DLK2
GC09M113297	1.51	https://www.genecards.org/cgi-bin/carddisp.pl?gene=RNF183
GC22P037682	1.51	https://www.genecards.org/cgi-bin/carddisp.pl?gene=NOL12
GC11P124954	1.51	https://www.genecards.org/cgi-bin/carddisp.pl?gene=CCDC15
GC19P006372	1.51	https://www.genecards.org/cgi-bin/carddisp.pl?gene=ALKBH7
GC01M183621	1.51	https://www.genecards.org/cgi-bin/carddisp.pl?gene=ARPC5
GC12M007114	1.51	https://www.genecards.org/cgi-bin/carddisp.pl?gene=C1RL
GC07M006794	1.51	https://www.genecards.org/cgi-bin/carddisp.pl?gene=CCZ1B
GC05P139353	1.51	https://www.genecards.org/cgi-bin/carddisp.pl?gene=PAIP2
GC0XP154445	1.51	https://www.genecards.org/cgi-bin/carddisp.pl?gene=FAM50A
GC12M066116	1.51	https://www.genecards.org/cgi-bin/carddisp.pl?gene=LLPH
GC14P104310	1.5	https://www.genecards.org/cgi-bin/carddisp.pl?gene=ZNF839
GC06P024667	1.5	https://www.genecards.org/cgi-bin/carddisp.pl?gene=ACOT13
GC11P095066	1.5	https://www.genecards.org/cgi-bin/carddisp.pl?gene=SRSF8
GC15P040766	1.5	https://www.genecards.org/cgi-bin/carddisp.pl?gene=GCHFR
GC06M143422	1.5	https://www.genecards.org/cgi-bin/carddisp.pl?gene=ADAT2
GC08P132775	1.5	https://www.genecards.org/cgi-bin/carddisp.pl?gene=PHF20L1
GC09P113410	1.5	https://www.genecards.org/cgi-bin/carddisp.pl?gene=C9orf43
GC14M063597	1.5	https://www.genecards.org/cgi-bin/carddisp.pl?gene=WDR89
GC01P053946	1.5	https://www.genecards.org/cgi-bin/carddisp.pl?gene=LRRC42

GC08P078666	1.5	https://www.genecards.org/cgi-bin/carddisp.pl?gene=ZC2HC1A
GC05M077072	1.5	https://www.genecards.org/cgi-bin/carddisp.pl?gene=ZBED3
GC06M084124	1.5	https://www.genecards.org/cgi-bin/carddisp.pl?gene=CEP162
GC15M073560	1.49	https://www.genecards.org/cgi-bin/carddisp.pl?gene=NPTN
GC16P077190	1.49	https://www.genecards.org/cgi-bin/carddisp.pl?gene=MON1B
GC19M019514	1.49	https://www.genecards.org/cgi-bin/carddisp.pl?gene=TSSK6
GC05P123512	1.49	https://www.genecards.org/cgi-bin/carddisp.pl?gene=CSNK1G3
GC05P160009	1.49	https://www.genecards.org/cgi-bin/carddisp.pl?gene=TTC1
GC17P041688	1.49	https://www.genecards.org/cgi-bin/carddisp.pl?gene=EIF1
GC17P004731	1.49	https://www.genecards.org/cgi-bin/carddisp.pl?gene=MED11
GC20P064255	1.48	https://www.genecards.org/cgi-bin/carddisp.pl?gene=PCMTD2
GC19M002233	1.48	https://www.genecards.org/cgi-bin/carddisp.pl?gene=PLEKHJ1
GC12P106301	1.48	https://www.genecards.org/cgi-bin/carddisp.pl?gene=TCP11L2
GC20P045827	1.48	https://www.genecards.org/cgi-bin/carddisp.pl?gene=SNX21
GC06P125956	1.48	https://www.genecards.org/cgi-bin/carddisp.pl?gene=HINT3
GC03M155764	1.48	https://www.genecards.org/cgi-bin/carddisp.pl?gene=C3orf33
GC04P163494	1.48	https://www.genecards.org/cgi-bin/carddisp.pl?gene=TMA16
GC0XM143508	1.48	https://www.genecards.org/cgi-bin/carddisp.pl?gene=SPANXN3
GC0XP135033	1.48	https://www.genecards.org/cgi-bin/carddisp.pl?gene=RTL8C
GC12M012974	1.48	https://www.genecards.org/cgi-bin/carddisp.pl?gene=HEBP1
GC0XM054932	1.48	https://www.genecards.org/cgi-bin/carddisp.pl?gene=PFKFB1
GC20P045306	1.48	https://www.genecards.org/cgi-bin/carddisp.pl?gene=RBPJL
GC14P073493	1.47	https://www.genecards.org/cgi-bin/carddisp.pl?gene=ACOT1
GC05M181254	1.47	https://www.genecards.org/cgi-bin/carddisp.pl?gene=TRIM52
GC19M015354	1.47	https://www.genecards.org/cgi-bin/carddisp.pl?gene=AKAP8
GC09P104747	1.47	https://www.genecards.org/cgi-bin/carddisp.pl?gene=NIPSNAP3A
GC08P145052	1.47	https://www.genecards.org/cgi-bin/carddisp.pl?gene=C8orf33
GC04P128061	1.47	https://www.genecards.org/cgi-bin/carddisp.pl?gene=LARP1B
GC19P049877	1.47	https://www.genecards.org/cgi-bin/carddisp.pl?gene=TBC1D17
GC01M025971	1.47	https://www.genecards.org/cgi-bin/carddisp.pl?gene=PAFAH2
GC02P100208	1.47	https://www.genecards.org/cgi-bin/carddisp.pl?gene=LINC01104
GC19P055641	1.47	https://www.genecards.org/cgi-bin/carddisp.pl?gene=CCDC106
GC03M149738	1.47	https://www.genecards.org/cgi-bin/carddisp.pl?gene=COMMD2
GC07M158532	1.47	https://www.genecards.org/cgi-bin/carddisp.pl?gene=MIR595
GC11P077822	1.47	https://www.genecards.org/cgi-bin/carddisp.pl?gene=AAMDC
GC16P083982	1.46	https://www.genecards.org/cgi-bin/carddisp.pl?gene=OSGIN1
GC17M029699	1.46	https://www.genecards.org/cgi-bin/carddisp.pl?gene=SDF2
GC09M021216	1.46	https://www.genecards.org/cgi-bin/carddisp.pl?gene=IFNA16
GC04M103077	1.46	https://www.genecards.org/cgi-bin/carddisp.pl?gene=BDH2
GC16M002783	1.46	https://www.genecards.org/cgi-bin/carddisp.pl?gene=PRSS33
GC02P130342	1.46	https://www.genecards.org/cgi-bin/carddisp.pl?gene=IMP4
GC11P093741	1.46	https://www.genecards.org/cgi-bin/carddisp.pl?gene=C11orf54
GC02M200488	1.46	https://www.genecards.org/cgi-bin/carddisp.pl?gene=KCTD18
GC12M008221	1.46	https://www.genecards.org/cgi-bin/carddisp.pl?gene=FAM90A1
GC0XM107123	1.46	https://www.genecards.org/cgi-bin/carddisp.pl?gene=NUP62CL
GC10P011865	1.46	https://www.genecards.org/cgi-bin/carddisp.pl?gene=PROSER2
GC0XM101658	1.46	https://www.genecards.org/cgi-bin/carddisp.pl?gene=ARMCX2

GC01P207034	1.46	https://www.genecards.org/cgi-bin/carddisp.pl?gene=PFKFB2
GC11P114401	1.46	https://www.genecards.org/cgi-bin/carddisp.pl?gene=RBM7
GC03P025782	1.45	https://www.genecards.org/cgi-bin/carddisp.pl?gene=OXSM
GC05P176388	1.45	https://www.genecards.org/cgi-bin/carddisp.pl?gene=HIGD2A
GC03P112990	1.45	https://www.genecards.org/cgi-bin/carddisp.pl?gene=GTPBP8
GC18P035973	1.45	https://www.genecards.org/cgi-bin/carddisp.pl?gene=C18orf21
GC05P148202	1.45	https://www.genecards.org/cgi-bin/carddisp.pl?gene=SPINK6
GC10M103390	1.45	https://www.genecards.org/cgi-bin/carddisp.pl?gene=ATP5MD
GC16M084975	1.45	https://www.genecards.org/cgi-bin/carddisp.pl?gene=ZDHHC7
GC03M126006	1.45	https://www.genecards.org/cgi-bin/carddisp.pl?gene=SLC41A3
GC19M058090	1.45	https://www.genecards.org/cgi-bin/carddisp.pl?gene=ZSCAN18
GC0XP044844	1.45	https://www.genecards.org/cgi-bin/carddisp.pl?gene=DUSP21
GC03M044737	1.45	https://www.genecards.org/cgi-bin/carddisp.pl?gene=KIAA1143
GC04M158666	1.45	https://www.genecards.org/cgi-bin/carddisp.pl?gene=C4orf46
GC01P161749	1.44	https://www.genecards.org/cgi-bin/carddisp.pl?gene=DUSP12
GC15P034224	1.44	https://www.genecards.org/cgi-bin/carddisp.pl?gene=EMC4
GC22P019130	1.44	https://www.genecards.org/cgi-bin/carddisp.pl?gene=TSSK2
GC08M143977	1.44	https://www.genecards.org/cgi-bin/carddisp.pl?gene=PARP10
GC16M000681	1.43	https://www.genecards.org/cgi-bin/carddisp.pl?gene=JMJD8
GC11P061481	1.43	https://www.genecards.org/cgi-bin/carddisp.pl?gene=PPP1R32
GC19P058034	1.43	https://www.genecards.org/cgi-bin/carddisp.pl?gene=ZSCAN1
GC08P143716	1.43	https://www.genecards.org/cgi-bin/carddisp.pl?gene=MAPK15
GC07P112206	1.43	https://www.genecards.org/cgi-bin/carddisp.pl?gene=ZNF277
GC06M075291	1.43	https://www.genecards.org/cgi-bin/carddisp.pl?gene=FILIP1
GC18M067506	1.43	https://www.genecards.org/cgi-bin/carddisp.pl?gene=DSEL
GC13M041216	1.43	https://www.genecards.org/cgi-bin/carddisp.pl?gene=MTRF1
GC07P006078	1.42	https://www.genecards.org/cgi-bin/carddisp.pl?gene=USP42
GC21P039867	1.42	https://www.genecards.org/cgi-bin/carddisp.pl?gene=PCP4
GC05P043255	1.42	https://www.genecards.org/cgi-bin/carddisp.pl?gene=NIM1K
GC14M021459	1.42	https://www.genecards.org/cgi-bin/carddisp.pl?gene=RAB2B
GC08P022089	1.42	https://www.genecards.org/cgi-bin/carddisp.pl?gene=FAM160B2
GC0XM120258	1.42	https://www.genecards.org/cgi-bin/carddisp.pl?gene=TMEM255A
GC07M139025	1.42	https://www.genecards.org/cgi-bin/carddisp.pl?gene=ZC3HAV1L
GC03P101779	1.42	https://www.genecards.org/cgi-bin/carddisp.pl?gene=NXPE3
GC0XP049590	1.42	https://www.genecards.org/cgi-bin/carddisp.pl?gene=GAGE1
GC03M133928	1.42	https://www.genecards.org/cgi-bin/carddisp.pl?gene=C3orf36
GC18P003603	1.42	https://www.genecards.org/cgi-bin/carddisp.pl?gene=DLGAP1-AS2
GC01P197871	1.42	https://www.genecards.org/cgi-bin/carddisp.pl?gene=C1orf53
GC09P130580	1.42	https://www.genecards.org/cgi-bin/carddisp.pl?gene=FUBP3
GC07M000996	1.41	https://www.genecards.org/cgi-bin/carddisp.pl?gene=C7orf50
GC03M016357	1.41	https://www.genecards.org/cgi-bin/carddisp.pl?gene=RFTN1
GC20P036606	1.41	https://www.genecards.org/cgi-bin/carddisp.pl?gene=RAB5IF
GC16P004079	1.4	https://www.genecards.org/cgi-bin/carddisp.pl?gene=ZNF75A
GC17M041486	1.4	https://www.genecards.org/cgi-bin/carddisp.pl?gene=KRT36
GC07M025125	1.4	https://www.genecards.org/cgi-bin/carddisp.pl?gene=C7orf31
GC18P003261	1.4	https://www.genecards.org/cgi-bin/carddisp.pl?gene=MYL12B
GC01P219173	1.4	https://www.genecards.org/cgi-bin/carddisp.pl?gene=LYPLAL1

GC11P122881	1.4	https://www.genecards.org/cgi-bin/carddisp.pl?gene=JHY
GC20M007977	1.4	https://www.genecards.org/cgi-bin/carddisp.pl?gene=TMX4
GC22M029259	1.4	https://www.genecards.org/cgi-bin/carddisp.pl?gene=RHBDD3
GC12M113195	1.4	https://www.genecards.org/cgi-bin/carddisp.pl?gene=IQCD
GC01P110124	1.39	https://www.genecards.org/cgi-bin/carddisp.pl?gene=UBL4B
GC07P032957	1.39	https://www.genecards.org/cgi-bin/carddisp.pl?gene=FKBP9
GC22P043411	1.39	https://www.genecards.org/cgi-bin/carddisp.pl?gene=MPPED1
GC15P065530	1.39	https://www.genecards.org/cgi-bin/carddisp.pl?gene=HACD3
GC0XM153802	1.39	https://www.genecards.org/cgi-bin/carddisp.pl?gene=PDZD4
GC15M063907	1.38	https://www.genecards.org/cgi-bin/carddisp.pl?gene=DAPK2
GC01P003454	1.38	https://www.genecards.org/cgi-bin/carddisp.pl?gene=ARHGEF16
GC12P101474	1.38	https://www.genecards.org/cgi-bin/carddisp.pl?gene=SPIC
GC17M074520	1.38	https://www.genecards.org/cgi-bin/carddisp.pl?gene=CD300LB
GC05P033937	1.37	https://www.genecards.org/cgi-bin/carddisp.pl?gene=RXFP3
GC01M156290	1.37	https://www.genecards.org/cgi-bin/carddisp.pl?gene=GLMP
GC17P050543	1.37	https://www.genecards.org/cgi-bin/carddisp.pl?gene=SPATA20
GC13M069700	1.37	https://www.genecards.org/cgi-bin/carddisp.pl?gene=KLHL1
GC03P122384	1.37	https://www.genecards.org/cgi-bin/carddisp.pl?gene=FAM162A
GC20M002481	1.36	https://www.genecards.org/cgi-bin/carddisp.pl?gene=ZNF343
GC12M047077	1.36	https://www.genecards.org/cgi-bin/carddisp.pl?gene=AMIGO2
GC14P073886	1.35	https://www.genecards.org/cgi-bin/carddisp.pl?gene=ZNF410
GC17P066964	1.35	https://www.genecards.org/cgi-bin/carddisp.pl?gene=CACNG4
GC01M153901	1.35	https://www.genecards.org/cgi-bin/carddisp.pl?gene=DENND4B
GC09P126914	1.35	https://www.genecards.org/cgi-bin/carddisp.pl?gene=RALGPS1
GC07M128027	1.35	https://www.genecards.org/cgi-bin/carddisp.pl?gene=LRRC4
GC08M054046	1.34	https://www.genecards.org/cgi-bin/carddisp.pl?gene=LYPLA1
GC17P032266	1.34	https://www.genecards.org/cgi-bin/carddisp.pl?gene=RHBDL3
GC13M113455	1.34	https://www.genecards.org/cgi-bin/carddisp.pl?gene=DCUN1D2
GC11M061746	1.34	https://www.genecards.org/cgi-bin/carddisp.pl?gene=MYRF-AS1
GC0XP016787	1.34	https://www.genecards.org/cgi-bin/carddisp.pl?gene=TXLNG
GC19P010701	1.34	https://www.genecards.org/cgi-bin/carddisp.pl?gene=QTRT1
GC06P028120	1.33	https://www.genecards.org/cgi-bin/carddisp.pl?gene=H2BC14
GC01M228100	1.33	https://www.genecards.org/cgi-bin/carddisp.pl?gene=C1orf35
GC16P084191	1.32	https://www.genecards.org/cgi-bin/carddisp.pl?gene=ADAD2
GC12M049636	1.32	https://www.genecards.org/cgi-bin/carddisp.pl?gene=FMNL3
GC15M074843	1.32	https://www.genecards.org/cgi-bin/carddisp.pl?gene=SCAMP2
GC04M142023	1.32	https://www.genecards.org/cgi-bin/carddisp.pl?gene=INPP4B
GC01P162790	1.31	https://www.genecards.org/cgi-bin/carddisp.pl?gene=HSD17B7
GC04P013351	1.31	https://www.genecards.org/cgi-bin/carddisp.pl?gene=HSP90AB2P
GC02P127645	1.31	https://www.genecards.org/cgi-bin/carddisp.pl?gene=GPR17
GC13M029764	1.31	https://www.genecards.org/cgi-bin/carddisp.pl?gene=UBL3
GC14M030893	1.31	https://www.genecards.org/cgi-bin/carddisp.pl?gene=STRN3
GC14M022985	1.31	https://www.genecards.org/cgi-bin/carddisp.pl?gene=C14orf93
GC16P066579	1.31	https://www.genecards.org/cgi-bin/carddisp.pl?gene=CMTM2
GC0XP130339	1.31	https://www.genecards.org/cgi-bin/carddisp.pl?gene=SLC25A14
GC02M151789	1.3	https://www.genecards.org/cgi-bin/carddisp.pl?gene=ARL5A
GC17M020836	1.3	https://www.genecards.org/cgi-bin/carddisp.pl?gene=CCDC144NL

GC21M014372	1.3	https://www.genecards.org/cgi-bin/carddisp.pl?gene=HSPA13
GC02M039702	1.3	https://www.genecards.org/cgi-bin/carddisp.pl?gene=THUMPD2
GC08P038386	1.3	https://www.genecards.org/cgi-bin/carddisp.pl?gene=LETM2
GC15M082041	1.29	https://www.genecards.org/cgi-bin/carddisp.pl?gene=MEX3B
GC09P104763	1.29	https://www.genecards.org/cgi-bin/carddisp.pl?gene=NIPSNAP3B
GC09M075061	1.29	https://www.genecards.org/cgi-bin/carddisp.pl?gene=NMRK1
GC02M207074	1.29	https://www.genecards.org/cgi-bin/carddisp.pl?gene=KLF7
GC09M037582	1.29	https://www.genecards.org/cgi-bin/carddisp.pl?gene=TOMM5
GC13M038965	1.29	https://www.genecards.org/cgi-bin/carddisp.pl?gene=STOML3
GC16M020370	1.29	https://www.genecards.org/cgi-bin/carddisp.pl?gene=PDILT
GC03M122411	1.28	https://www.genecards.org/cgi-bin/carddisp.pl?gene=WDR5B
GC12P066302	1.28	https://www.genecards.org/cgi-bin/carddisp.pl?gene=HELB
GC12M014825	1.28	https://www.genecards.org/cgi-bin/carddisp.pl?gene=ART4
GC04M001610	1.28	https://www.genecards.org/cgi-bin/carddisp.pl?gene=FAM53A
GC02M200973	1.28	https://www.genecards.org/cgi-bin/carddisp.pl?gene=FAM126B
GC02P128091	1.28	https://www.genecards.org/cgi-bin/carddisp.pl?gene=UGGT1
GC10P104354	1.28	https://www.genecards.org/cgi-bin/carddisp.pl?gene=CFAP58
GC01P155320	1.28	https://www.genecards.org/cgi-bin/carddisp.pl?gene=RUSC1
GC09M002411	1.27	https://www.genecards.org/cgi-bin/carddisp.pl?gene=VLDLR-AS1
GC17P040177	1.27	https://www.genecards.org/cgi-bin/carddisp.pl?gene=RAPGEFL1
GC03P048440	1.27	https://www.genecards.org/cgi-bin/carddisp.pl?gene=TMA7
GC08P108443	1.26	https://www.genecards.org/cgi-bin/carddisp.pl?gene=EMC2
GC11M128899	1.26	https://www.genecards.org/cgi-bin/carddisp.pl?gene=C11orf45
GC11M062602	1.26	https://www.genecards.org/cgi-bin/carddisp.pl?gene=EML3
GC19M011342	1.26	https://www.genecards.org/cgi-bin/carddisp.pl?gene=TMEM205
GC07P005289	1.26	https://www.genecards.org/cgi-bin/carddisp.pl?gene=SLC29A4
GC12M113298	1.26	https://www.genecards.org/cgi-bin/carddisp.pl?gene=SLC8B1
GC11P114439	1.26	https://www.genecards.org/cgi-bin/carddisp.pl?gene=REXO2
GC05M112142	1.25	https://www.genecards.org/cgi-bin/carddisp.pl?gene=EPB41L4A
GC13M026755	1.25	https://www.genecards.org/cgi-bin/carddisp.pl?gene=GPR12
GC07M092560	1.25	https://www.genecards.org/cgi-bin/carddisp.pl?gene=FAM133B
GC02P230327	1.25	https://www.genecards.org/cgi-bin/carddisp.pl?gene=SP140L
GC01M052346	1.24	https://www.genecards.org/cgi-bin/carddisp.pl?gene=CC2D1B
GC01M027666	1.24	https://www.genecards.org/cgi-bin/carddisp.pl?gene=IFI6
GC19M041541	1.24	https://www.genecards.org/cgi-bin/carddisp.pl?gene=DMAC2
GC16P085027	1.23	https://www.genecards.org/cgi-bin/carddisp.pl?gene=KIAA0513
GC0XM018162	1.23	https://www.genecards.org/cgi-bin/carddisp.pl?gene=BEND2
GC14P021212	1.23	https://www.genecards.org/cgi-bin/carddisp.pl?gene=TPPP2
GC0XP056275	1.23	https://www.genecards.org/cgi-bin/carddisp.pl?gene=KLF8
GC14M081260	1.22	https://www.genecards.org/cgi-bin/carddisp.pl?gene=STON2
GC03M129088	1.21	https://www.genecards.org/cgi-bin/carddisp.pl?gene=RAB43
GC15P073873	1.21	https://www.genecards.org/cgi-bin/carddisp.pl?gene=TBC1D21
GC03P193244	1.21	https://www.genecards.org/cgi-bin/carddisp.pl?gene=PLAAT1
GC14M021090	1.21	https://www.genecards.org/cgi-bin/carddisp.pl?gene=ZNF219
GC06P010723	1.21	https://www.genecards.org/cgi-bin/carddisp.pl?gene=TMEM14C
GC16P001226	1.21	https://www.genecards.org/cgi-bin/carddisp.pl?gene=FAM234A
GC08P095133	1.2	https://www.genecards.org/cgi-bin/carddisp.pl?gene=PLEKHF2

GC0XM076919	1.2	https://www.genecards.org/cgi-bin/carddisp.pl?gene=MIR384
GC19M023687	1.2	https://www.genecards.org/cgi-bin/carddisp.pl?gene=LINC00663
GC02M131047	1.2	https://www.genecards.org/cgi-bin/carddisp.pl?gene=FAM168B
GC19P022225	1.2	https://www.genecards.org/cgi-bin/carddisp.pl?gene=OCEL1
GC12P007850	1.2	https://www.genecards.org/cgi-bin/carddisp.pl?gene=USP5
GC11P064240	1.19	https://www.genecards.org/cgi-bin/carddisp.pl?gene=FKBP2
GC17P081890	1.19	https://www.genecards.org/cgi-bin/carddisp.pl?gene=ANAPC11
GC01M202147	1.19	https://www.genecards.org/cgi-bin/carddisp.pl?gene=PTPN7
GC05M115613	1.19	https://www.genecards.org/cgi-bin/carddisp.pl?gene=TMED7
GC12M106977	1.19	https://www.genecards.org/cgi-bin/carddisp.pl?gene=MTERF2
GC07P112480	1.19	https://www.genecards.org/cgi-bin/carddisp.pl?gene=LSMEM1
GC10P102462	1.19	https://www.genecards.org/cgi-bin/carddisp.pl?gene=MFSD13A
GC14M069380	1.18	https://www.genecards.org/cgi-bin/carddisp.pl?gene=ERH
GC07P048088	1.18	https://www.genecards.org/cgi-bin/carddisp.pl?gene=UPP1
GC01P192158	1.18	https://www.genecards.org/cgi-bin/carddisp.pl?gene=RGS18
GC01P103554	1.18	https://www.genecards.org/cgi-bin/carddisp.pl?gene=AMY2B
GC22P041688	1.18	https://www.genecards.org/cgi-bin/carddisp.pl?gene=C22orf46
GC15P084631	1.17	https://www.genecards.org/cgi-bin/carddisp.pl?gene=SCAND2P
GC03P141876	1.17	https://www.genecards.org/cgi-bin/carddisp.pl?gene=ATP1B3
GC03P178272	1.17	https://www.genecards.org/cgi-bin/carddisp.pl?gene=KCNMB2
GC01M054610	1.17	https://www.genecards.org/cgi-bin/carddisp.pl?gene=FAM151A
GC10M126424	1.17	https://www.genecards.org/cgi-bin/carddisp.pl?gene=C10orf90
GC16P066609	1.17	https://www.genecards.org/cgi-bin/carddisp.pl?gene=CMTM3
GC19M001815	1.16	https://www.genecards.org/cgi-bin/carddisp.pl?gene=REXO1
GC03P101574	1.15	https://www.genecards.org/cgi-bin/carddisp.pl?gene=PCNP
GC20P002968	1.15	https://www.genecards.org/cgi-bin/carddisp.pl?gene=EBF4
GC01P070206	1.15	https://www.genecards.org/cgi-bin/carddisp.pl?gene=SRSF11
GC06P155264	1.14	https://www.genecards.org/cgi-bin/carddisp.pl?gene=CLDN20
GC07M099392	1.14	https://www.genecards.org/cgi-bin/carddisp.pl?gene=PDAP1
GC06P017600	1.14	https://www.genecards.org/cgi-bin/carddisp.pl?gene=FAM8A1
GC0XP071578	1.13	https://www.genecards.org/cgi-bin/carddisp.pl?gene=GCNA
GC02M224765	1.13	https://www.genecards.org/cgi-bin/carddisp.pl?gene=DOCK10
GC11M072085	1.13	https://www.genecards.org/cgi-bin/carddisp.pl?gene=LAMTOR1
GC22M045383	1.13	https://www.genecards.org/cgi-bin/carddisp.pl?gene=TPTEP2-CSNK1E
GC05U902908	1.13	https://www.genecards.org/cgi-bin/carddisp.pl?gene=LOC111188154
GC0XM006668	1.12	https://www.genecards.org/cgi-bin/carddisp.pl?gene=PUDP
GC04P037964	1.11	https://www.genecards.org/cgi-bin/carddisp.pl?gene=PTTG2
GC01P171106	1.11	https://www.genecards.org/cgi-bin/carddisp.pl?gene=FMO6P
GC17M000757	1.11	https://www.genecards.org/cgi-bin/carddisp.pl?gene=GLOD4
GC08M103132	1.11	https://www.genecards.org/cgi-bin/carddisp.pl?gene=BAALC-AS2
GC03P122577	1.11	https://www.genecards.org/cgi-bin/carddisp.pl?gene=PARP15
GC0XP084934	1.11	https://www.genecards.org/cgi-bin/carddisp.pl?gene=UBE2DNL
GC17U990141	1.1	https://www.genecards.org/cgi-bin/carddisp.pl?gene=ERDA1
GC04M181875	1.1	https://www.genecards.org/cgi-bin/carddisp.pl?gene=TENM3-AS1
GC01P109539	1.1	https://www.genecards.org/cgi-bin/carddisp.pl?gene=GPR61
GC06M011103	1.1	https://www.genecards.org/cgi-bin/carddisp.pl?gene=ERVFRD-1
GC19P009827	1.1	https://www.genecards.org/cgi-bin/carddisp.pl?gene=UBL5

GC09M113373	1.09	https://www.genecards.org/cgi-bin/carddisp.pl?gene=HDHD3
GC19M012688	1.09	https://www.genecards.org/cgi-bin/carddisp.pl?gene=FBXW9
GC07M022728	1.09	https://www.genecards.org/cgi-bin/carddisp.pl?gene=IL6-AS1
GC11M067506	1.09	https://www.genecards.org/cgi-bin/carddisp.pl?gene=CDK2AP2
GC14M020582	1.09	https://www.genecards.org/cgi-bin/carddisp.pl?gene=RNASE11
GC11P060913	1.08	https://www.genecards.org/cgi-bin/carddisp.pl?gene=TMEM109
GC09P133019	1.08	https://www.genecards.org/cgi-bin/carddisp.pl?gene=EEF1A1P5
GC01M088936	1.08	https://www.genecards.org/cgi-bin/carddisp.pl?gene=KYAT3
GC05P142849	1.08	https://www.genecards.org/cgi-bin/carddisp.pl?gene=PCDHB10
GC02M219327	1.08	https://www.genecards.org/cgi-bin/carddisp.pl?gene=RESP18
GC10P124918	1.08	https://www.genecards.org/cgi-bin/carddisp.pl?gene=ZRANB1
GC09P006319	1.07	https://www.genecards.org/cgi-bin/carddisp.pl?gene=TPD52L3
GC06P004079	1.07	https://www.genecards.org/cgi-bin/carddisp.pl?gene=C6orf201
GC17P050426	1.06	https://www.genecards.org/cgi-bin/carddisp.pl?gene=ACSF2
GC01M230837	1.06	https://www.genecards.org/cgi-bin/carddisp.pl?gene=C1orf198
GC01M036322	1.06	https://www.genecards.org/cgi-bin/carddisp.pl?gene=EVA1B
GC05P041932	1.06	https://www.genecards.org/cgi-bin/carddisp.pl?gene=C5orf51
GC09P123033	1.06	https://www.genecards.org/cgi-bin/carddisp.pl?gene=GPR21
GC16P066844	1.06	https://www.genecards.org/cgi-bin/carddisp.pl?gene=CA7
GC20P043558	1.06	https://www.genecards.org/cgi-bin/carddisp.pl?gene=SGK2
GC01P033860	1.06	https://www.genecards.org/cgi-bin/carddisp.pl?gene=HMGB4
GC07M038260	1.05	https://www.genecards.org/cgi-bin/carddisp.pl?gene=TARP
GC06M158841	1.05	https://www.genecards.org/cgi-bin/carddisp.pl?gene=OSTCP1
GC22M030652	1.05	https://www.genecards.org/cgi-bin/carddisp.pl?gene=DUSP18
GC06M029056	1.04	https://www.genecards.org/cgi-bin/carddisp.pl?gene=OR2W1
GC11M114391	1.04	https://www.genecards.org/cgi-bin/carddisp.pl?gene=C11orf71
GC20P044336	1.03	https://www.genecards.org/cgi-bin/carddisp.pl?gene=R3HDML
GC0XP095499	1.03	https://www.genecards.org/cgi-bin/carddisp.pl?gene=CALM1P1
GC20P002082	1.03	https://www.genecards.org/cgi-bin/carddisp.pl?gene=STK35
GC02M089769	1.03	https://www.genecards.org/cgi-bin/carddisp.pl?gene=IGKV2-30
GC02M219294	1.03	https://www.genecards.org/cgi-bin/carddisp.pl?gene=MIR153-1
GC01P152970	1.02	https://www.genecards.org/cgi-bin/carddisp.pl?gene=SPRR4
GC04P080336	1.02	https://www.genecards.org/cgi-bin/carddisp.pl?gene=CFAP299
GC14P091061	1.02	https://www.genecards.org/cgi-bin/carddisp.pl?gene=DGLUCY
GC12P075391	1.02	https://www.genecards.org/cgi-bin/carddisp.pl?gene=GLIPR1L2
GC09M104526	1.02	https://www.genecards.org/cgi-bin/carddisp.pl?gene=OR13C4
GC01P202122	1.01	https://www.genecards.org/cgi-bin/carddisp.pl?gene=GPR37L1
GC11P047424	1.01	https://www.genecards.org/cgi-bin/carddisp.pl?gene=MIR4487
GC0XP057121	1.01	https://www.genecards.org/cgi-bin/carddisp.pl?gene=FAAH2
GC12P113185	1.01	https://www.genecards.org/cgi-bin/carddisp.pl?gene=RITA1
GC11P111256	1.01	https://www.genecards.org/cgi-bin/carddisp.pl?gene=C11orf53
GC04M024807	1.01	https://www.genecards.org/cgi-bin/carddisp.pl?gene=CCDC149
GC02P131591	1	https://www.genecards.org/cgi-bin/carddisp.pl?gene=POTEKP

Table S3 Putative targets of Ideb

Gene symbol	Gene ID
NTRK1	4914
MAPKAPK2	9261
CYP2D6	1565
HCRTR2	3062
PIK3CB	5291
TACR1	6869
MAPK10	5602
CHRM1	1128
PTGER1	5731
CYP19A1	1588
HCRTR1	3061
JAK2	3717
XIAP	331
NCSTN	23385
ITGB2	3689
CCNA2	890
TRPV1	7442
HTR1A	3350
CRHR1	1394
MDM2	4193
AKT2	208
EGFR	1956
PDE5A	8654
PDE10A	10846
CDK2	1017
PPP2CA	5515
MAPK11	5600
ITGAL	3683
CCNA1	8900
CHEK1	1111
MAPK9	5601
ABCB1	5243
MAPK14	1432
TNK2	10188
MTNR1A	4543
LSS	4047
VDR	7421
NPY5R	4889
CNR2	1269
PIK3CG	5294
S1PR1	1901
PIK3CA	5290
SYK	6850
APH1A	51107
IL6ST	3572
JAK1	3716
AKT1	207

PSEN1	5663
CSF1R	1436
CYP3A4	1576
APP	351
CACNA2D1	781
MTNR1B	4544
CYP2C19	1557
SMO	6608
PTK2	5747
F2	2147
CYP2C9	1559
PRKDC	5591
PIK3CD	5293
PSEN2	5664
MAPK1	5594
IKBKB	3551
MARK2	2011
MAPK8	5599
PDE4B	5142
APH1B	83464
REN	5972
P2RX7	5027
PDE3B	5140
ALOX5	240
FASN	2194
PLK3	1263
PLK1	5347
PLA2G2A	5320
ADORA2A	135
KCNH2	3757
PIM1	5292
PPP1CC	5501
MTOR	2475
DRD2	1813
PDPK1	5170
PSENEN	55851
AVPR2	554
ICAM1	3383
CHRM2	1129
CDC42BPA	8476

enone agianst PD

Gene Full Name
neurotrophic receptor tyrosine kinase 1
MAPK activated protein kinase 2
cytochrome P450 family 2 subfamily D member 6
hypocretin receptor 2
phosphatidylinositol-4,5-bisphosphate 3-kinase catalytic subunit beta
tachykinin receptor 1
mitogen-activated protein kinase 10
cholinergic receptor muscarinic 1
prostaglandin E receptor 1
cytochrome P450 family 19 subfamily A member 1
hypocretin receptor 1
Janus kinase 2
X-linked inhibitor of apoptosis
nicastrin
integrin subunit beta 2
cyclin A2
transient receptor potential cation channel subfamily V member 1
5-hydroxytryptamine receptor 1A
corticotropin releasing hormone receptor 1
MDM2 proto-oncogene
AKT serine/threonine kinase 2
epidermal growth factor receptor
phosphodiesterase 5A
phosphodiesterase 10A
cyclin dependent kinase 2
protein phosphatase 2 catalytic subunit alpha
mitogen-activated protein kinase 11
integrin subunit alpha L
cyclin A1
checkpoint kinase 1
mitogen-activated protein kinase 9
ATP binding cassette subfamily B member 1
mitogen-activated protein kinase 14
tyrosine kinase non receptor 2
melatonin receptor 1A
lanosterol synthase
vitamin D receptor
neuropeptide Y receptor Y5
cannabinoid receptor 2
phosphatidylinositol-4,5-bisphosphate 3-kinase catalytic subunit gamma
sphingosine-1-phosphate receptor 1
phosphatidylinositol-4,5-bisphosphate 3-kinase catalytic subunit alpha
spleen associated tyrosine kinase
aph-1 homolog A, gamma-secretase subunit
interleukin 6 cytokine family signal transducer
Janus kinase 1
AKT serine/threonine kinase 1

presenilin 1
colony stimulating factor 1 receptor
cytochrome P450 family 3 subfamily A member 4
amyloid beta precursor protein
calcium voltage-gated channel auxiliary subunit alpha2delta 1
melatonin receptor 1B
cytochrome P450 family 2 subfamily C member 19
smoothened, frizzled class receptor
protein tyrosine kinase 2
coagulation factor II, thrombin
cytochrome P450 family 2 subfamily C member 9
protein kinase, DNA-activated, catalytic subunit
phosphatidylinositol-4,5-bisphosphate 3-kinase catalytic subunit delta
presenilin 2
mitogen-activated protein kinase 1
inhibitor of nuclear factor kappa B kinase subunit beta
microtubule affinity regulating kinase 2
mitogen-activated protein kinase 8
phosphodiesterase 4B
aph-1 homolog B, gamma-secretase subunit
renin
purinergic receptor P2X 7
phosphodiesterase 3B
arachidonate 5-lipoxygenase
fatty acid synthase
polo like kinase 3
polo like kinase 1
phospholipase A2 group IIA
adenosine A2a receptor
potassium voltage-gated channel subfamily H member 2
Pim-1 proto-oncogene, serine/threonine kinase
protein phosphatase 1 catalytic subunit gamma
mechanistic target of rapamycin kinase
dopamine receptor D2
3-phosphoinositide dependent protein kinase 1
presenilin enhancer, gamma-secretase subunit
arginine vasopressin receptor 2
intercellular adhesion molecule 1
cholinergic receptor muscarinic 2
CDC42 binding protein kinase alpha

Table. S4 The result of the GO and KEGG pathway enrichment

Table. S4-1 The result of biological process in GO analysis

ID	Description
GO:0018105	peptidyl-serine phosphorylation
GO:0018209	peptidyl-serine modification
GO:0032147	activation of protein kinase activity
GO:0071902	positive regulation of protein serine/threonine kinase activity
GO:0006874	cellular calcium ion homeostasis
GO:0055074	calcium ion homeostasis
GO:0072503	cellular divalent inorganic cation homeostasis
GO:0035690	cellular response to drug
GO:0051222	positive regulation of protein transport
GO:0007220	Notch receptor processing
GO:1904951	positive regulation of establishment of protein localization
GO:0042063	gliogenesis
GO:0007623	circadian rhythm
GO:0043406	positive regulation of MAP kinase activity
GO:0009314	response to radiation
GO:1903829	positive regulation of cellular protein localization
GO:0043405	regulation of MAP kinase activity
GO:2000379	positive regulation of reactive oxygen species metabolic process
GO:0048015	phosphatidylinositol-mediated signaling
GO:0048017	inositol lipid-mediated signaling
GO:0046777	protein autophosphorylation
GO:0034765	regulation of ion transmembrane transport
GO:0048511	rhythmic process
GO:0014065	phosphatidylinositol 3-kinase signaling
GO:1903522	regulation of blood circulation
GO:0051480	regulation of cytosolic calcium ion concentration
GO:0018108	peptidyl-tyrosine phosphorylation
GO:0042752	regulation of circadian rhythm
GO:0018212	peptidyl-tyrosine modification
GO:0051090	regulation of DNA-binding transcription factor activity
GO:0031293	membrane protein intracellular domain proteolysis
GO:0061900	glial cell activation
GO:1901653	cellular response to peptide
GO:0014066	regulation of phosphatidylinositol 3-kinase signaling
GO:0018107	peptidyl-threonine phosphorylation
GO:0042110	T cell activation
GO:0010001	glial cell differentiation
GO:0044070	regulation of anion transport
GO:0018210	peptidyl-threonine modification
GO:0032388	positive regulation of intracellular transport
GO:0070997	neuron death
GO:0009410	response to xenobiotic stimulus
GO:0007631	feeding behavior
GO:0050900	leukocyte migration
GO:0033002	muscle cell proliferation

GO:0048732	gland development
GO:0032386	regulation of intracellular transport
GO:0071868	cellular response to monoamine stimulus
GO:0071870	cellular response to catecholamine stimulus
GO:0050435	amyloid-beta metabolic process
GO:0150076	neuroinflammatory response
GO:0007187	G protein-coupled receptor signaling pathway, coupled to cyclic nucleotide second messe
GO:0050730	regulation of peptidyl-tyrosine phosphorylation
GO:0071867	response to monoamine
GO:0071869	response to catecholamine
GO:0010959	regulation of metal ion transport
GO:0048708	astrocyte differentiation
GO:0048013	ephrin receptor signaling pathway
GO:0014068	positive regulation of phosphatidylinositol 3-kinase signaling
GO:0060047	heart contraction
GO:0051403	stress-activated MAPK cascade
GO:0090316	positive regulation of intracellular protein transport
GO:0032890	regulation of organic acid transport
GO:0033619	membrane protein proteolysis
GO:0003015	heart process
GO:0006816	calcium ion transport
GO:0043434	response to peptide hormone
GO:0050804	modulation of chemical synaptic transmission
GO:0099177	regulation of trans-synaptic signaling
GO:0019932	second-messenger-mediated signaling
GO:0007200	phospholipase C-activating G protein-coupled receptor signaling pathway
GO:0030879	mammary gland development
GO:0007219	Notch signaling pathway
GO:0009416	response to light stimulus
GO:2000377	regulation of reactive oxygen species metabolic process
GO:0031098	stress-activated protein kinase signaling cascade
GO:0051924	regulation of calcium ion transport
GO:0007204	positive regulation of cytosolic calcium ion concentration
GO:0042535	positive regulation of tumor necrosis factor biosynthetic process
GO:0007568	aging
GO:0034764	positive regulation of transmembrane transport
GO:0035265	organ growth
GO:0050795	regulation of behavior
GO:0006509	membrane protein ectodomain proteolysis
GO:0032496	response to lipopolysaccharide
GO:0061180	mammary gland epithelium development
GO:0070838	divalent metal ion transport
GO:0045429	positive regulation of nitric oxide biosynthetic process
GO:0043271	negative regulation of ion transport
GO:0043279	response to alkaloid
GO:0072511	divalent inorganic cation transport
GO:1900182	positive regulation of protein localization to nucleus

GO:0042987	amyloid precursor protein catabolic process
GO:1904407	positive regulation of nitric oxide metabolic process
GO:0046620	regulation of organ growth
GO:0071496	cellular response to external stimulus
GO:0043270	positive regulation of ion transport
GO:0002237	response to molecule of bacterial origin
GO:0016485	protein processing
GO:0021782	glial cell development
GO:0048638	regulation of developmental growth
GO:0007188	adenylate cyclase-modulating G protein-coupled receptor signaling pathway
GO:0072593	reactive oxygen species metabolic process
GO:0051047	positive regulation of secretion
GO:0001505	regulation of neurotransmitter levels
GO:0038095	Fc-epsilon receptor signaling pathway
GO:0042391	regulation of membrane potential
GO:0010038	response to metal ion
GO:1903959	regulation of anion transmembrane transport
GO:0097366	response to bronchodilator
GO:0048143	astrocyte activation
GO:0050727	regulation of inflammatory response
GO:0051402	neuron apoptotic process
GO:0038093	Fc receptor signaling pathway
GO:1901214	regulation of neuron death
GO:0001819	positive regulation of cytokine production
GO:0033157	regulation of intracellular protein transport
GO:0070371	ERK1 and ERK2 cascade
GO:1903351	cellular response to dopamine
GO:1903428	positive regulation of reactive oxygen species biosynthetic process
GO:0008016	regulation of heart contraction
GO:0070588	calcium ion transmembrane transport
GO:0003012	muscle system process
GO:1903350	response to dopamine
GO:0071375	cellular response to peptide hormone stimulus
GO:2000060	positive regulation of ubiquitin-dependent protein catabolic process
GO:0038127	ERBB signaling pathway
GO:0022407	regulation of cell-cell adhesion
GO:0045576	mast cell activation
GO:1903532	positive regulation of secretion by cell
GO:0036092	phosphatidylinositol-3-phosphate biosynthetic process
GO:0048661	positive regulation of smooth muscle cell proliferation
GO:0042982	amyloid precursor protein metabolic process
GO:0046622	positive regulation of organ growth
GO:0034205	amyloid-beta formation
GO:0042533	tumor necrosis factor biosynthetic process
GO:0042534	regulation of tumor necrosis factor biosynthetic process
GO:0043491	protein kinase B signaling
GO:0045927	positive regulation of growth

GO:0034766	negative regulation of ion transmembrane transport
GO:0007599	hemostasis
GO:0050817	coagulation
GO:1904062	regulation of cation transmembrane transport
GO:1901215	negative regulation of neuron death
GO:0000187	activation of MAPK activity
GO:1903169	regulation of calcium ion transmembrane transport
GO:0009612	response to mechanical stimulus
GO:0045428	regulation of nitric oxide biosynthetic process
GO:0070374	positive regulation of ERK1 and ERK2 cascade
GO:0071241	cellular response to inorganic substance
GO:0033627	cell adhesion mediated by integrin
GO:0071276	cellular response to cadmium ion
GO:0051604	protein maturation
GO:0045862	positive regulation of proteolysis
GO:0031331	positive regulation of cellular catabolic process
GO:0006936	muscle contraction
GO:0030595	leukocyte chemotaxis
GO:0045124	regulation of bone resorption
GO:0072401	signal transduction involved in DNA integrity checkpoint
GO:0072422	signal transduction involved in DNA damage checkpoint
GO:1900180	regulation of protein localization to nucleus
GO:0034614	cellular response to reactive oxygen species
GO:0050806	positive regulation of synaptic transmission
GO:0006979	response to oxidative stress
GO:0050890	cognition
GO:0048660	regulation of smooth muscle cell proliferation
GO:0072395	signal transduction involved in cell cycle checkpoint
GO:0070372	regulation of ERK1 and ERK2 cascade
GO:0048659	smooth muscle cell proliferation
GO:0000302	response to reactive oxygen species
GO:0007173	epidermal growth factor receptor signaling pathway
GO:1903052	positive regulation of proteolysis involved in cellular protein catabolic process
GO:0006809	nitric oxide biosynthetic process
GO:0031669	cellular response to nutrient levels
GO:0030258	lipid modification
GO:0009895	negative regulation of catabolic process
GO:0007409	axonogenesis
GO:0046717	acid secretion
GO:0014002	astrocyte development
GO:0032368	regulation of lipid transport
GO:0051896	regulation of protein kinase B signaling
GO:0032436	positive regulation of proteasomal ubiquitin-dependent protein catabolic process
GO:0048872	homeostasis of number of cells
GO:0034763	negative regulation of transmembrane transport
GO:0046209	nitric oxide metabolic process
GO:0048639	positive regulation of developmental growth

GO:0048512	circadian behavior
GO:0042749	regulation of circadian sleep/wake cycle
GO:0007622	rhythmic behavior
GO:0043303	mast cell degranulation
GO:2001057	reactive nitrogen species metabolic process
GO:0002279	mast cell activation involved in immune response
GO:0046850	regulation of bone remodeling
GO:0042770	signal transduction in response to DNA damage
GO:0060291	long-term synaptic potentiation
GO:0007611	learning or memory
GO:0002448	mast cell mediated immunity
GO:1990090	cellular response to nerve growth factor stimulus
GO:0046942	carboxylic acid transport
GO:0031667	response to nutrient levels
GO:0015849	organic acid transport
GO:0034103	regulation of tissue remodeling
GO:0034504	protein localization to nucleus
GO:0022410	circadian sleep/wake cycle process
GO:0007596	blood coagulation
GO:0007193	adenylate cyclase-inhibiting G protein-coupled receptor signaling pathway
GO:0007159	leukocyte cell-cell adhesion
GO:0060048	cardiac muscle contraction
GO:0060078	regulation of postsynaptic membrane potential
GO:1903364	positive regulation of cellular protein catabolic process
GO:1990089	response to nerve growth factor
GO:0042738	exogenous drug catabolic process
GO:0031668	cellular response to extracellular stimulus
GO:0050714	positive regulation of protein secretion
GO:0009896	positive regulation of catabolic process
GO:0050852	T cell receptor signaling pathway
GO:0035296	regulation of tube diameter
GO:0097746	regulation of blood vessel diameter
GO:0000186	activation of MAPKK activity
GO:0051952	regulation of amine transport
GO:0035150	regulation of tube size
GO:0000077	DNA damage checkpoint
GO:0050994	regulation of lipid catabolic process
GO:0062197	cellular response to chemical stress
GO:0050673	epithelial cell proliferation
GO:0007411	axon guidance
GO:0043524	negative regulation of neuron apoptotic process
GO:0042220	response to cocaine
GO:0048010	vascular endothelial growth factor receptor signaling pathway
GO:0097485	neuron projection guidance
GO:0015908	fatty acid transport
GO:0042745	circadian sleep/wake cycle
GO:2000058	regulation of ubiquitin-dependent protein catabolic process

GO:2000191	regulation of fatty acid transport
GO:0015837	amine transport
GO:0060079	excitatory postsynaptic potential
GO:0030168	platelet activation
GO:0042133	neurotransmitter metabolic process
GO:1903426	regulation of reactive oxygen species biosynthetic process
GO:0002793	positive regulation of peptide secretion
GO:1903792	negative regulation of anion transport
GO:0034767	positive regulation of ion transmembrane transport
GO:1905952	regulation of lipid localization
GO:0031570	DNA integrity checkpoint
GO:0043467	regulation of generation of precursor metabolites and energy
GO:0019233	sensory perception of pain
GO:0046822	regulation of nucleocytoplasmic transport
GO:1901800	positive regulation of proteasomal protein catabolic process
GO:0051098	regulation of binding
GO:0045822	negative regulation of heart contraction
GO:0055017	cardiac muscle tissue growth
GO:1903305	regulation of regulated secretory pathway
GO:0042136	neurotransmitter biosynthetic process
GO:0045453	bone resorption
GO:0046824	positive regulation of nucleocytoplasmic transport
GO:0006869	lipid transport
GO:0042759	long-chain fatty acid biosynthetic process
GO:0048265	response to pain
GO:0046686	response to cadmium ion
GO:0002526	acute inflammatory response
GO:0099565	chemical synaptic transmission, postsynaptic
GO:0034599	cellular response to oxidative stress
GO:0006631	fatty acid metabolic process
GO:0031349	positive regulation of defense response
GO:0060326	cell chemotaxis
GO:1903037	regulation of leukocyte cell-cell adhesion
GO:0051955	regulation of amino acid transport
GO:1901568	fatty acid derivative metabolic process
GO:0072678	T cell migration
GO:0072676	lymphocyte migration
GO:0060419	heart growth
GO:0015800	acidic amino acid transport
GO:0001975	response to amphetamine
GO:0008210	estrogen metabolic process
GO:0032228	regulation of synaptic transmission, GABAergic
GO:0045907	positive regulation of vasoconstriction
GO:0042108	positive regulation of cytokine biosynthetic process
GO:0006690	icosanoid metabolic process
GO:0048871	multicellular organismal homeostasis
GO:0070106	interleukin-27-mediated signaling pathway

GO:0070757	interleukin-35-mediated signaling pathway
GO:0001503	ossification
GO:0050851	antigen receptor-mediated signaling pathway
GO:0015909	long-chain fatty acid transport
GO:0003018	vascular process in circulatory system
GO:0051897	positive regulation of protein kinase B signaling
GO:0060401	cytosolic calcium ion transport
GO:0043276	anoikis
GO:0015711	organic anion transport
GO:0051966	regulation of synaptic transmission, glutamatergic
GO:0006941	striated muscle contraction
GO:0048771	tissue remodeling
GO:0006721	terpenoid metabolic process
GO:0098739	import across plasma membrane
GO:0051954	positive regulation of amine transport
GO:0071312	cellular response to alkaloid
GO:1901385	regulation of voltage-gated calcium channel activity
GO:0019216	regulation of lipid metabolic process
GO:0071466	cellular response to xenobiotic stimulus
GO:1901796	regulation of signal transduction by p53 class mediator
GO:0051881	regulation of mitochondrial membrane potential
GO:0032434	regulation of proteasomal ubiquitin-dependent protein catabolic process
GO:1903409	reactive oxygen species biosynthetic process
GO:1905477	positive regulation of protein localization to membrane
GO:0033555	multicellular organismal response to stress
GO:0010876	lipid localization
GO:0071621	granulocyte chemotaxis
GO:0031330	negative regulation of cellular catabolic process
GO:0008202	steroid metabolic process
GO:0071214	cellular response to abiotic stimulus
GO:0104004	cellular response to environmental stimulus
GO:0005977	glycogen metabolic process
GO:0031670	cellular response to nutrient
GO:0032418	lysosome localization
GO:0038083	peptidyl-tyrosine autophosphorylation
GO:0042307	positive regulation of protein import into nucleus
GO:0006073	cellular glucan metabolic process
GO:0044042	glucan metabolic process
GO:0048167	regulation of synaptic plasticity
GO:1903960	negative regulation of anion transmembrane transport
GO:0071248	cellular response to metal ion
GO:0042310	vasoconstriction
GO:0003007	heart morphogenesis
GO:0090257	regulation of muscle system process
GO:0035270	endocrine system development
GO:0016572	histone phosphorylation
GO:0051590	positive regulation of neurotransmitter transport

GO:1903523	negative regulation of blood circulation
GO:0051091	positive regulation of DNA-binding transcription factor activity
GO:0046883	regulation of hormone secretion
GO:0001933	negative regulation of protein phosphorylation
GO:0098657	import into cell
GO:0055021	regulation of cardiac muscle tissue growth
GO:0072347	response to anesthetic
GO:0048608	reproductive structure development
GO:0050731	positive regulation of peptidyl-tyrosine phosphorylation
GO:1904591	positive regulation of protein import
GO:0045913	positive regulation of carbohydrate metabolic process
GO:0061458	reproductive system development
GO:0009214	cyclic nucleotide catabolic process
GO:0048266	behavioral response to pain
GO:0014855	striated muscle cell proliferation
GO:0140353	lipid export from cell
GO:0032868	response to insulin
GO:0006970	response to osmotic stress
GO:0010613	positive regulation of cardiac muscle hypertrophy
GO:0033003	regulation of mast cell activation
GO:0001666	response to hypoxia
GO:0060420	regulation of heart growth
GO:0097756	negative regulation of blood vessel diameter
GO:0002573	myeloid leukocyte differentiation
GO:0031396	regulation of protein ubiquitination
GO:0006720	isoprenoid metabolic process
GO:0050715	positive regulation of cytokine secretion
GO:0051588	regulation of neurotransmitter transport
GO:0014742	positive regulation of muscle hypertrophy
GO:1904646	cellular response to amyloid-beta
GO:0097553	calcium ion transmembrane import into cytosol
GO:0001776	leukocyte homeostasis
GO:0032760	positive regulation of tumor necrosis factor production
GO:0097530	granulocyte migration
GO:0014047	glutamate secretion
GO:0001780	neutrophil homeostasis
GO:0014048	regulation of glutamate secretion
GO:1990000	amyloid fibril formation
GO:0006112	energy reserve metabolic process
GO:0006909	phagocytosis
GO:0006606	protein import into nucleus
GO:0043523	regulation of neuron apoptotic process
GO:0097529	myeloid leukocyte migration
GO:0036293	response to decreased oxygen levels
GO:1903557	positive regulation of tumor necrosis factor superfamily cytokine production
GO:0032309	icosanoid secretion
GO:0051932	synaptic transmission, GABAergic

GO:1900271	regulation of long-term synaptic potentiation
GO:0019935	cyclic-nucleotide-mediated signaling
GO:0014075	response to amine
GO:0033628	regulation of cell adhesion mediated by integrin
GO:0043300	regulation of leukocyte degranulation
GO:0071354	cellular response to interleukin-6
GO:0015844	monoamine transport
GO:0046849	bone remodeling
GO:0007254	JNK cascade
GO:0045732	positive regulation of protein catabolic process
GO:1903050	regulation of proteolysis involved in cellular protein catabolic process
GO:0045187	regulation of circadian sleep/wake cycle, sleep
GO:0060644	mammary gland epithelial cell differentiation
GO:0042326	negative regulation of phosphorylation
GO:0071356	cellular response to tumor necrosis factor
GO:0002262	myeloid cell homeostasis
GO:0000075	cell cycle checkpoint
GO:0070849	response to epidermal growth factor
GO:0050708	regulation of protein secretion
GO:0017157	regulation of exocytosis
GO:0043393	regulation of protein binding
GO:0002429	immune response-activating cell surface receptor signaling pathway
GO:0002757	immune response-activating signal transduction
GO:0014074	response to purine-containing compound
GO:1903039	positive regulation of leukocyte cell-cell adhesion
GO:0008360	regulation of cell shape
GO:0001774	microglial cell activation
GO:0002269	leukocyte activation involved in inflammatory response
GO:0008542	visual learning
GO:0055023	positive regulation of cardiac muscle tissue growth
GO:0007584	response to nutrient
GO:0035249	synaptic transmission, glutamatergic
GO:0044843	cell cycle G1/S phase transition
GO:0031998	regulation of fatty acid beta-oxidation
GO:0051957	positive regulation of amino acid transport
GO:0001890	placenta development
GO:0071715	icosanoid transport
GO:1901571	fatty acid derivative transport
GO:0050729	positive regulation of inflammatory response
GO:0042116	macrophage activation
GO:0006835	dicarboxylic acid transport
GO:0014009	glial cell proliferation
GO:0046854	phosphatidylinositol phosphorylation
GO:0070741	response to interleukin-6
GO:1903793	positive regulation of anion transport
GO:0045088	regulation of innate immune response
GO:0032930	positive regulation of superoxide anion generation

GO:0070989	oxidative demethylation
GO:0070482	response to oxygen levels
GO:0046879	hormone secretion
GO:0060402	calcium ion transport into cytosol
GO:0060421	positive regulation of heart growth
GO:1903320	regulation of protein modification by small protein conjugation or removal
GO:0006914	autophagy
GO:0061919	process utilizing autophagic mechanism
GO:0034612	response to tumor necrosis factor
GO:0055024	regulation of cardiac muscle tissue development
GO:0050802	circadian sleep/wake cycle, sleep
GO:0007632	visual behavior
GO:0002027	regulation of heart rate
GO:0002791	regulation of peptide secretion
GO:0015718	monocarboxylic acid transport
GO:0051170	import into nucleus
GO:0000079	regulation of cyclin-dependent protein serine/threonine kinase activity
GO:0009914	hormone transport
GO:1904645	response to amyloid-beta
GO:0071216	cellular response to biotic stimulus
GO:0044264	cellular polysaccharide metabolic process
GO:0032891	negative regulation of organic acid transport
GO:0035813	regulation of renal sodium excretion
GO:1900273	positive regulation of long-term synaptic potentiation
GO:0042306	regulation of protein import into nucleus
GO:0030593	neutrophil chemotaxis
GO:1904029	regulation of cyclin-dependent protein kinase activity
GO:0051100	negative regulation of binding
GO:0032928	regulation of superoxide anion generation
GO:0035809	regulation of urine volume
GO:0002886	regulation of myeloid leukocyte mediated immunity
GO:0060043	regulation of cardiac muscle cell proliferation
GO:0072431	signal transduction involved in mitotic G1 DNA damage checkpoint
GO:1902400	intracellular signal transduction involved in G1 DNA damage checkpoint
GO:0019229	regulation of vasoconstriction
GO:1904589	regulation of protein import
GO:1903362	regulation of cellular protein catabolic process
GO:0001676	long-chain fatty acid metabolic process
GO:0035812	renal sodium excretion
GO:0045821	positive regulation of glycolytic process
GO:0050995	negative regulation of lipid catabolic process
GO:0016042	lipid catabolic process
GO:0046488	phosphatidylinositol metabolic process
GO:0072413	signal transduction involved in mitotic cell cycle checkpoint
GO:1902402	signal transduction involved in mitotic DNA damage checkpoint
GO:1902403	signal transduction involved in mitotic DNA integrity checkpoint
GO:1904019	epithelial cell apoptotic process

GO:0062012	regulation of small molecule metabolic process
GO:0043302	positive regulation of leukocyte degranulation
GO:0044062	regulation of excretion
GO:0045123	cellular extravasation
GO:0022409	positive regulation of cell-cell adhesion
GO:0005976	polysaccharide metabolic process
GO:0042035	regulation of cytokine biosynthetic process
GO:2001257	regulation of cation channel activity
GO:0006913	nucleocytoplasmic transport
GO:0055025	positive regulation of cardiac muscle tissue development
GO:0051953	negative regulation of amine transport
GO:0007569	cell aging
GO:0051169	nuclear transport
GO:0032412	regulation of ion transmembrane transporter activity
GO:0007588	excretion
GO:0031571	mitotic G1 DNA damage checkpoint
GO:0044819	mitotic G1/S transition checkpoint
GO:0061136	regulation of proteasomal protein catabolic process
GO:0019933	cAMP-mediated signaling
GO:0042098	T cell proliferation
GO:2000045	regulation of G1/S transition of mitotic cell cycle
GO:0015850	organic hydroxy compound transport
GO:0044783	G1 DNA damage checkpoint
GO:0046834	lipid phosphorylation
GO:0060135	maternal process involved in female pregnancy
GO:1990266	neutrophil migration
GO:0032768	regulation of monooxygenase activity
GO:1905475	regulation of protein localization to membrane
GO:0044839	cell cycle G2/M phase transition
GO:0003014	renal system process
GO:0016079	synaptic vesicle exocytosis
GO:0072331	signal transduction by p53 class mediator
GO:0040014	regulation of multicellular organism growth
GO:0022898	regulation of transmembrane transporter activity
GO:0035902	response to immobilization stress
GO:0006836	neurotransmitter transport
GO:0060038	cardiac muscle cell proliferation
GO:0042089	cytokine biosynthetic process
GO:0017038	protein import
GO:0014013	regulation of gliogenesis
GO:0042107	cytokine metabolic process
GO:0051209	release of sequestered calcium ion into cytosol
GO:0048520	positive regulation of behavior
GO:2000108	positive regulation of leukocyte apoptotic process
GO:0002697	regulation of immune effector process
GO:0051283	negative regulation of sequestering of calcium ion
GO:0000082	G1/S transition of mitotic cell cycle

GO:0007626	locomotory behavior
GO:0005979	regulation of glycogen biosynthetic process
GO:0010800	positive regulation of peptidyl-threonine phosphorylation
GO:0010962	regulation of glucan biosynthetic process
GO:0070102	interleukin-6-mediated signaling pathway
GO:0051282	regulation of sequestering of calcium ion
GO:0006109	regulation of carbohydrate metabolic process
GO:0032409	regulation of transporter activity
GO:0002532	production of molecular mediator involved in inflammatory response
GO:0031647	regulation of protein stability
GO:0046320	regulation of fatty acid oxidation
GO:1902806	regulation of cell cycle G1/S phase transition
GO:0051208	sequestering of calcium ion
GO:0030278	regulation of ossification
GO:1903524	positive regulation of blood circulation
GO:0002703	regulation of leukocyte mediated immunity
GO:0071222	cellular response to lipopolysaccharide
GO:1901987	regulation of cell cycle phase transition
GO:0002675	positive regulation of acute inflammatory response
GO:0006691	leukotriene metabolic process
GO:0009154	purine ribonucleotide catabolic process
GO:0030431	sleep
GO:0043304	regulation of mast cell degranulation
GO:0050482	arachidonic acid secretion
GO:1903963	arachidonate transport
GO:0030900	forebrain development
GO:0042176	regulation of protein catabolic process
GO:0046683	response to organophosphorus
GO:0002064	epithelial cell development
GO:0071456	cellular response to hypoxia
GO:0010611	regulation of cardiac muscle hypertrophy
GO:0009261	ribonucleotide catabolic process
GO:0033006	regulation of mast cell activation involved in immune response
GO:0051968	positive regulation of synaptic transmission, glutamatergic
GO:0055094	response to lipoprotein particle
GO:0071295	cellular response to vitamin
GO:0050707	regulation of cytokine secretion
GO:0046425	regulation of receptor signaling pathway via JAK-STAT
GO:0045787	positive regulation of cell cycle
GO:0071219	cellular response to molecule of bacterial origin
GO:0099003	vesicle-mediated transport in synapse
GO:0002888	positive regulation of myeloid leukocyte mediated immunity
GO:0014072	response to isoquinoline alkaloid
GO:0016242	negative regulation of macroautophagy
GO:0042755	eating behavior
GO:0043278	response to morphine
GO:2001025	positive regulation of response to drug

GO:0002433	immune response-regulating cell surface receptor signaling pathway involved in phagocy
GO:0038096	Fc-gamma receptor signaling pathway involved in phagocytosis
GO:0008306	associative learning
GO:0014743	regulation of muscle hypertrophy
GO:0070661	leukocyte proliferation
GO:0090068	positive regulation of cell cycle process
GO:0050867	positive regulation of cell activation
GO:0043281	regulation of cysteine-type endopeptidase activity involved in apoptotic process
GO:0071260	cellular response to mechanical stimulus
GO:0006865	amino acid transport
GO:0009411	response to UV
GO:0062013	positive regulation of small molecule metabolic process
GO:0032869	cellular response to insulin stimulus
GO:0002446	neutrophil mediated immunity
GO:0032148	activation of protein kinase B activity
GO:0032892	positive regulation of organic acid transport
GO:0071402	cellular response to lipoprotein particle stimulus
GO:0098664	G protein-coupled serotonin receptor signaling pathway
GO:0036294	cellular response to decreased oxygen levels
GO:0038094	Fc-gamma receptor signaling pathway
GO:0002831	regulation of response to biotic stimulus
GO:0002699	positive regulation of immune effector process
GO:0051651	maintenance of location in cell
GO:0051656	establishment of organelle localization
GO:0001893	maternal placenta development
GO:0032735	positive regulation of interleukin-12 production
GO:0060251	regulation of glial cell proliferation
GO:0070873	regulation of glycogen metabolic process
GO:0098801	regulation of renal system process
GO:0045785	positive regulation of cell adhesion
GO:0002431	Fc receptor mediated stimulatory signaling pathway
GO:0035264	multicellular organism growth
GO:0045834	positive regulation of lipid metabolic process
GO:1904892	regulation of receptor signaling pathway via STAT
GO:2001233	regulation of apoptotic signaling pathway
GO:0042509	regulation of tyrosine phosphorylation of STAT protein
GO:0042554	superoxide anion generation
GO:0090322	regulation of superoxide metabolic process
GO:0010507	negative regulation of autophagy
GO:0043154	negative regulation of cysteine-type endopeptidase activity involved in apoptotic process
GO:0050863	regulation of T cell activation
GO:0051092	positive regulation of NF-kappaB transcription factor activity
GO:0032885	regulation of polysaccharide biosynthetic process
GO:0098926	postsynaptic signal transduction
GO:0007260	tyrosine phosphorylation of STAT protein
GO:0042058	regulation of epidermal growth factor receptor signaling pathway
GO:0001894	tissue homeostasis

GO:0030099	myeloid cell differentiation
GO:1990778	protein localization to cell periphery
GO:0016202	regulation of striated muscle tissue development
GO:0045844	positive regulation of striated muscle tissue development
GO:0048636	positive regulation of muscle organ development
GO:0046326	positive regulation of glucose import
GO:0060045	positive regulation of cardiac muscle cell proliferation
GO:0060416	response to growth hormone
GO:1901863	positive regulation of muscle tissue development
GO:1904035	regulation of epithelial cell apoptotic process
GO:0055067	monovalent inorganic cation homeostasis
GO:0042445	hormone metabolic process
GO:0048738	cardiac muscle tissue development
GO:0071453	cellular response to oxygen levels
GO:1901861	regulation of muscle tissue development
GO:0007210	serotonin receptor signaling pathway
GO:0042417	dopamine metabolic process
GO:0046427	positive regulation of receptor signaling pathway via JAK-STAT
GO:1901019	regulation of calcium ion transmembrane transporter activity
GO:0006469	negative regulation of protein kinase activity
GO:0048634	regulation of muscle organ development
GO:0010506	regulation of autophagy
GO:0009187	cyclic nucleotide metabolic process
GO:0051899	membrane depolarization
GO:1901992	positive regulation of mitotic cell cycle phase transition
GO:1904063	negative regulation of cation transmembrane transport
GO:0031348	negative regulation of defense response
GO:2000116	regulation of cysteine-type endopeptidase activity
GO:0007259	receptor signaling pathway via JAK-STAT
GO:0050663	cytokine secretion
GO:1904894	positive regulation of receptor signaling pathway via STAT
GO:2000117	negative regulation of cysteine-type endopeptidase activity
GO:0032680	regulation of tumor necrosis factor production
GO:0061028	establishment of endothelial barrier
GO:0003073	regulation of systemic arterial blood pressure
GO:0033273	response to vitamin
GO:1901184	regulation of ERBB signaling pathway
GO:0001820	serotonin secretion
GO:0002291	T cell activation via T cell receptor contact with antigen bound to MHC molecule on anti
GO:0010749	regulation of nitric oxide mediated signal transduction
GO:0010918	positive regulation of mitochondrial membrane potential
GO:0014049	positive regulation of glutamate secretion
GO:0042756	drinking behavior
GO:0045792	negative regulation of cell size
GO:0045988	negative regulation of striated muscle contraction
GO:0048149	behavioral response to ethanol
GO:0072610	interleukin-12 secretion

GO:0090557	establishment of endothelial intestinal barrier
GO:1904526	regulation of microtubule binding
GO:1990535	neuron projection maintenance
GO:0002683	negative regulation of immune system process
GO:0009266	response to temperature stimulus
GO:0032640	tumor necrosis factor production
GO:1903555	regulation of tumor necrosis factor superfamily cytokine production
GO:0060249	anatomical structure homeostasis
GO:0045930	negative regulation of mitotic cell cycle
GO:0006633	fatty acid biosynthetic process
GO:0000086	G2/M transition of mitotic cell cycle
GO:0007093	mitotic cell cycle checkpoint
GO:0031279	regulation of cyclase activity
GO:0032881	regulation of polysaccharide metabolic process
GO:0034198	cellular response to amino acid starvation
GO:0051602	response to electrical stimulus
GO:0071364	cellular response to epidermal growth factor stimulus
GO:0021537	telencephalon development
GO:1901990	regulation of mitotic cell cycle phase transition
GO:0044773	mitotic DNA damage checkpoint
GO:0006195	purine nucleotide catabolic process
GO:0007212	dopamine receptor signaling pathway
GO:0010828	positive regulation of glucose transmembrane transport
GO:0034105	positive regulation of tissue remodeling
GO:0071706	tumor necrosis factor superfamily cytokine production
GO:0035747	natural killer cell chemotaxis
GO:0070673	response to interleukin-18
GO:1902563	regulation of neutrophil activation
GO:0050728	negative regulation of inflammatory response
GO:0097696	receptor signaling pathway via STAT
GO:0019395	fatty acid oxidation
GO:1901570	fatty acid derivative biosynthetic process
GO:0007269	neurotransmitter secretion
GO:0099643	signal release from synapse
GO:0031018	endocrine pancreas development
GO:0048255	mRNA stabilization
GO:0048538	thymus development
GO:0052547	regulation of peptidase activity
GO:0006937	regulation of muscle contraction
GO:0022600	digestive system process
GO:0051052	regulation of DNA metabolic process
GO:0034440	lipid oxidation
GO:0048259	regulation of receptor-mediated endocytosis
GO:0033673	negative regulation of kinase activity
GO:0002673	regulation of acute inflammatory response
GO:0005978	glycogen biosynthetic process
GO:0009250	glucan biosynthetic process

GO:0031648	protein destabilization
GO:0045981	positive regulation of nucleotide metabolic process
GO:1900544	positive regulation of purine nucleotide metabolic process
GO:1904036	negative regulation of epithelial cell apoptotic process
GO:1990928	response to amino acid starvation
GO:0010799	regulation of peptidyl-threonine phosphorylation
GO:1903580	positive regulation of ATP metabolic process
GO:0007229	integrin-mediated signaling pathway
GO:0010623	programmed cell death involved in cell development
GO:0038110	interleukin-2-mediated signaling pathway
GO:0042368	vitamin D biosynthetic process
GO:0051481	negative regulation of cytosolic calcium ion concentration
GO:0009408	response to heat
GO:0045861	negative regulation of proteolysis
GO:0007218	neuropeptide signaling pathway
GO:0071887	leukocyte apoptotic process
GO:0043949	regulation of cAMP-mediated signaling
GO:0033138	positive regulation of peptidyl-serine phosphorylation
GO:0002286	T cell activation involved in immune response
GO:0003300	cardiac muscle hypertrophy
GO:0044774	mitotic DNA integrity checkpoint
GO:1901989	positive regulation of cell cycle phase transition
GO:0022408	negative regulation of cell-cell adhesion
GO:0035094	response to nicotine
GO:1901988	negative regulation of cell cycle phase transition
GO:0043502	regulation of muscle adaptation
GO:0051341	regulation of oxidoreductase activity
GO:0002679	respiratory burst involved in defense response
GO:0007494	midgut development
GO:0050722	regulation of interleukin-1 beta biosynthetic process
GO:0071352	cellular response to interleukin-2
GO:0072672	neutrophil extravasation
GO:0098703	calcium ion import across plasma membrane
GO:0006275	regulation of DNA replication
GO:0071156	regulation of cell cycle arrest
GO:0043489	RNA stabilization
GO:1900087	positive regulation of G1/S transition of mitotic cell cycle
GO:0050769	positive regulation of neurogenesis
GO:0021953	central nervous system neuron differentiation
GO:0014897	striated muscle hypertrophy
GO:0046651	lymphocyte proliferation
GO:0019369	arachidonic acid metabolic process
GO:0048146	positive regulation of fibroblast proliferation
GO:0050999	regulation of nitric-oxide synthase activity
GO:0072523	purine-containing compound catabolic process
GO:2001258	negative regulation of cation channel activity
GO:0006939	smooth muscle contraction

GO:0033559	unsaturated fatty acid metabolic process
GO:0032943	mononuclear cell proliferation
GO:0014896	muscle hypertrophy
GO:0031529	ruffle organization
GO:0032731	positive regulation of interleukin-1 beta production
GO:0055078	sodium ion homeostasis
GO:1902373	negative regulation of mRNA catabolic process
GO:0010752	regulation of cGMP-mediated signaling
GO:0035810	positive regulation of urine volume
GO:0035815	positive regulation of renal sodium excretion
GO:0048308	organelle inheritance
GO:0048313	Golgi inheritance
GO:0050720	interleukin-1 beta biosynthetic process
GO:0060009	Sertoli cell development
GO:0070669	response to interleukin-2
GO:0072425	signal transduction involved in G2 DNA damage checkpoint
GO:1901386	negative regulation of voltage-gated calcium channel activity
GO:1902656	calcium ion import into cytosol
GO:0043312	neutrophil degranulation
GO:0050678	regulation of epithelial cell proliferation
GO:0022029	telencephalon cell migration
GO:1903307	positive regulation of regulated secretory pathway
GO:0002283	neutrophil activation involved in immune response
GO:0002696	positive regulation of leukocyte activation
GO:0045446	endothelial cell differentiation
GO:0051054	positive regulation of DNA metabolic process
GO:0007565	female pregnancy
GO:0043112	receptor metabolic process
GO:0006584	catecholamine metabolic process
GO:0009712	catechol-containing compound metabolic process
GO:0010524	positive regulation of calcium ion transport into cytosol
GO:0032655	regulation of interleukin-12 production
GO:0043392	negative regulation of DNA binding
GO:1904375	regulation of protein localization to cell periphery
GO:0099504	synaptic vesicle cycle
GO:0006661	phosphatidylinositol biosynthetic process
GO:0046456	icosanoid biosynthetic process
GO:0098900	regulation of action potential
GO:0051348	negative regulation of transferase activity
GO:0009415	response to water
GO:0033008	positive regulation of mast cell activation involved in immune response
GO:0033604	negative regulation of catecholamine secretion
GO:0042362	fat-soluble vitamin biosynthetic process
GO:0043306	positive regulation of mast cell degranulation
GO:0044539	long-chain fatty acid import into cell
GO:0045360	regulation of interleukin-1 biosynthetic process
GO:0045410	positive regulation of interleukin-6 biosynthetic process

GO:0045838	positive regulation of membrane potential
GO:0050961	detection of temperature stimulus involved in sensory perception
GO:0050965	detection of temperature stimulus involved in sensory perception of pain
GO:0060134	prepulse inhibition
GO:0140354	lipid import into cell
GO:1901841	regulation of high voltage-gated calcium channel activity
GO:0007613	memory
GO:0031398	positive regulation of protein ubiquitination
GO:0043666	regulation of phosphoprotein phosphatase activity
GO:0015980	energy derivation by oxidation of organic compounds
GO:0002685	regulation of leukocyte migration
GO:0006694	steroid biosynthetic process
GO:0042119	neutrophil activation
GO:0006977	DNA damage response, signal transduction by p53 class mediator resulting in cell cycle a
GO:0021885	forebrain cell migration
GO:0031295	T cell costimulation
GO:0032615	interleukin-12 production
GO:1903078	positive regulation of protein localization to plasma membrane
GO:0002221	pattern recognition receptor signaling pathway
GO:0014706	striated muscle tissue development
GO:0007162	negative regulation of cell adhesion
GO:0046890	regulation of lipid biosynthetic process
GO:0031294	lymphocyte costimulation
GO:0072089	stem cell proliferation
GO:0006206	pyrimidine nucleobase metabolic process
GO:0006837	serotonin transport
GO:0042222	interleukin-1 biosynthetic process
GO:0043217	myelin maintenance
GO:0055119	relaxation of cardiac muscle
GO:0071732	cellular response to nitric oxide
GO:0090335	regulation of brown fat cell differentiation
GO:2000369	regulation of clathrin-dependent endocytosis
GO:2000846	regulation of corticosteroid hormone secretion
GO:0031663	lipopolysaccharide-mediated signaling pathway
GO:0032722	positive regulation of chemokine production
GO:0050870	positive regulation of T cell activation
GO:0009150	purine ribonucleotide metabolic process
GO:0051928	positive regulation of calcium ion transport
GO:0001885	endothelial cell development
GO:0032732	positive regulation of interleukin-1 production
GO:0070527	platelet aggregation
GO:0097755	positive regulation of blood vessel diameter
GO:1902369	negative regulation of RNA catabolic process
GO:0006006	glucose metabolic process
GO:0051101	regulation of DNA binding
GO:0046324	regulation of glucose import
GO:1902808	positive regulation of cell cycle G1/S phase transition

GO:0050679	positive regulation of epithelial cell proliferation
GO:0006805	xenobiotic metabolic process
GO:2000134	negative regulation of G1/S transition of mitotic cell cycle
GO:0007635	chemosensory behavior
GO:0010744	positive regulation of macrophage derived foam cell differentiation
GO:0035930	corticosteroid hormone secretion
GO:0042753	positive regulation of circadian rhythm
GO:0045725	positive regulation of glycogen biosynthetic process
GO:0046068	cGMP metabolic process
GO:0048521	negative regulation of behavior
GO:0051709	regulation of killing of cells of other organism
GO:0055070	copper ion homeostasis
GO:1904996	positive regulation of leukocyte adhesion to vascular endothelial cell
GO:2000811	negative regulation of anoikis
GO:0046887	positive regulation of hormone secretion
GO:0060537	muscle tissue development
GO:0007517	muscle organ development
GO:0031640	killing of cells of other organism
GO:0050433	regulation of catecholamine secretion
GO:0034754	cellular hormone metabolic process
GO:0007252	I-kappaB phosphorylation
GO:0045780	positive regulation of bone resorption
GO:0046852	positive regulation of bone remodeling
GO:0070875	positive regulation of glycogen metabolic process
GO:0071318	cellular response to ATP
GO:1902001	fatty acid transmembrane transport
GO:1904377	positive regulation of protein localization to cell periphery
GO:0009259	ribonucleotide metabolic process
GO:0043500	muscle adaptation
GO:1902807	negative regulation of cell cycle G1/S phase transition
GO:0050432	catecholamine secretion
GO:0003158	endothelium development
GO:0042552	myelination
GO:0043161	proteasome-mediated ubiquitin-dependent protein catabolic process
GO:0046474	glycerophospholipid biosynthetic process
GO:0009166	nucleotide catabolic process
GO:0001508	action potential
GO:0007213	G protein-coupled acetylcholine receptor signaling pathway
GO:0016048	detection of temperature stimulus
GO:0032303	regulation of icosanoid secretion
GO:0060252	positive regulation of glial cell proliferation
GO:0060716	labyrinthine layer blood vessel development
GO:0071731	response to nitric oxide
GO:1902170	cellular response to reactive nitrogen species
GO:2000831	regulation of steroid hormone secretion
GO:0019722	calcium-mediated signaling
GO:0050807	regulation of synapse organization

GO:0051235	maintenance of location
GO:0007272	ensheathment of neurons
GO:0008366	axon ensheathment
GO:0051961	negative regulation of nervous system development
GO:0051926	negative regulation of calcium ion transport
GO:0019693	ribose phosphate metabolic process
GO:1903322	positive regulation of protein modification by small protein conjugation or removal
GO:0052548	regulation of endopeptidase activity
GO:0002705	positive regulation of leukocyte mediated immunity
GO:0006650	glycerophospholipid metabolic process
GO:0044706	multi-multicellular organism process
GO:0034605	cellular response to heat
GO:0003323	type B pancreatic cell development
GO:0019370	leukotriene biosynthetic process
GO:0019373	epoxygenase P450 pathway
GO:0033005	positive regulation of mast cell activation
GO:0042053	regulation of dopamine metabolic process
GO:0042069	regulation of catecholamine metabolic process
GO:0048148	behavioral response to cocaine
GO:0051580	regulation of neurotransmitter uptake
GO:0060008	Sertoli cell differentiation
GO:0006470	protein dephosphorylation
GO:0006644	phospholipid metabolic process
GO:0035924	cellular response to vascular endothelial growth factor stimulus
GO:0046323	glucose import
GO:0097191	extrinsic apoptotic signaling pathway
GO:0006163	purine nucleotide metabolic process
GO:0001649	osteoblast differentiation
GO:0007160	cell-matrix adhesion
GO:0007189	adenylate cyclase-activating G protein-coupled receptor signaling pathway
GO:0007586	digestion
GO:0033135	regulation of peptidyl-serine phosphorylation
GO:0035304	regulation of protein dephosphorylation
GO:0042737	drug catabolic process
GO:0050803	regulation of synapse structure or activity
GO:0046677	response to antibiotic
GO:0002438	acute inflammatory response to antigenic stimulus
GO:0003283	atrial septum development
GO:0032516	positive regulation of phosphoprotein phosphatase activity
GO:0050951	sensory perception of temperature stimulus
GO:2000269	regulation of fibroblast apoptotic process
GO:0070988	demethylation
GO:0072091	regulation of stem cell proliferation
GO:0002218	activation of innate immune response
GO:0033692	cellular polysaccharide biosynthetic process
GO:0042531	positive regulation of tyrosine phosphorylation of STAT protein
GO:1901292	nucleoside phosphate catabolic process

GO:1904427	positive regulation of calcium ion transmembrane transport
GO:0030534	adult behavior
GO:1904064	positive regulation of cation transmembrane transport
GO:0006635	fatty acid beta-oxidation
GO:0050805	negative regulation of synaptic transmission
GO:0002052	positive regulation of neuroblast proliferation
GO:0006925	inflammatory cell apoptotic process
GO:0007617	mating behavior
GO:0031281	positive regulation of cyclase activity
GO:0032469	endoplasmic reticulum calcium ion homeostasis
GO:0035929	steroid hormone secretion
GO:0042359	vitamin D metabolic process
GO:0046058	cAMP metabolic process
GO:0070935	3'-UTR-mediated mRNA stabilization
GO:0071404	cellular response to low-density lipoprotein particle stimulus
GO:0097305	response to alcohol
GO:0007612	learning
GO:0010256	endomembrane system organization
GO:0006801	superoxide metabolic process
GO:0045685	regulation of glial cell differentiation
GO:0002224	toll-like receptor signaling pathway
GO:0010212	response to ionizing radiation
GO:0014015	positive regulation of gliogenesis
GO:0086003	cardiac muscle cell contraction
GO:0007050	cell cycle arrest
GO:0032872	regulation of stress-activated MAPK cascade
GO:0072330	monocarboxylic acid biosynthetic process
GO:0062149	detection of stimulus involved in sensory perception of pain
GO:0071305	cellular response to vitamin D
GO:0051048	negative regulation of secretion
GO:0021536	diencephalon development
GO:0070302	regulation of stress-activated protein kinase signaling cascade
GO:0001101	response to acid chemical
GO:0010721	negative regulation of cell development
GO:0002092	positive regulation of receptor internalization
GO:0009110	vitamin biosynthetic process
GO:0010288	response to lead ion
GO:0021895	cerebral cortex neuron differentiation
GO:0021904	dorsal/ventral neural tube patterning
GO:0044346	fibroblast apoptotic process
GO:0046697	decidualization
GO:0060396	growth hormone receptor signaling pathway
GO:2000209	regulation of anoikis
GO:0032675	regulation of interleukin-6 production
GO:0006110	regulation of glycolytic process
GO:0031397	negative regulation of protein ubiquitination
GO:0048678	response to axon injury

GO:0051937	catecholamine transport
GO:0019318	hexose metabolic process
GO:0000271	polysaccharide biosynthetic process
GO:0010827	regulation of glucose transmembrane transport
GO:0031016	pancreas development
GO:0032413	negative regulation of ion transmembrane transporter activity
GO:2000300	regulation of synaptic vesicle exocytosis
GO:0001667	ameboidal-type cell migration
GO:0051346	negative regulation of hydrolase activity
GO:0072521	purine-containing compound metabolic process
GO:1900034	regulation of cellular response to heat
GO:1903312	negative regulation of mRNA metabolic process
GO:0002068	glandular epithelial cell development
GO:0003309	type B pancreatic cell differentiation
GO:0043951	negative regulation of cAMP-mediated signaling
GO:0060330	regulation of response to interferon-gamma
GO:0060334	regulation of interferon-gamma-mediated signaling pathway
GO:0071378	cellular response to growth hormone stimulus
GO:0010631	epithelial cell migration
GO:1901991	negative regulation of mitotic cell cycle phase transition
GO:0070509	calcium ion import
GO:0031589	cell-substrate adhesion
GO:0001764	neuron migration
GO:0010951	negative regulation of endopeptidase activity
GO:0030072	peptide hormone secretion
GO:0046394	carboxylic acid biosynthetic process
GO:0090132	epithelium migration
GO:0045017	glycerolipid biosynthetic process
GO:0016053	organic acid biosynthetic process
GO:0034109	homotypic cell-cell adhesion
GO:0110110	positive regulation of animal organ morphogenesis
GO:1902930	regulation of alcohol biosynthetic process
GO:0002360	T cell lineage commitment
GO:0010758	regulation of macrophage chemotaxis
GO:0022011	myelination in peripheral nervous system
GO:0032292	peripheral nervous system axon ensheathment
GO:0035640	exploration behavior
GO:0048169	regulation of long-term neuronal synaptic plasticity
GO:0032642	regulation of chemokine production
GO:0055117	regulation of cardiac muscle contraction
GO:0071158	positive regulation of cell cycle arrest
GO:0010948	negative regulation of cell cycle process
GO:0090130	tissue migration
GO:0032635	interleukin-6 production
GO:1902652	secondary alcohol metabolic process
GO:0010498	proteasomal protein catabolic process
GO:0048145	regulation of fibroblast proliferation

GO:2000106	regulation of leukocyte apoptotic process
GO:0001964	startle response
GO:0006706	steroid catabolic process
GO:0007176	regulation of epidermal growth factor-activated receptor activity
GO:0035883	enteroendocrine cell differentiation
GO:0045932	negative regulation of muscle contraction
GO:0050820	positive regulation of coagulation
GO:0060259	regulation of feeding behavior
GO:0060307	regulation of ventricular cardiac muscle cell membrane repolarization
GO:0030111	regulation of Wnt signaling pathway
GO:0043087	regulation of GTPase activity
GO:0048144	fibroblast proliferation
GO:0050818	regulation of coagulation
GO:0050886	endocrine process
GO:0045931	positive regulation of mitotic cell cycle
GO:0002028	regulation of sodium ion transport
GO:0008654	phospholipid biosynthetic process
GO:0072659	protein localization to plasma membrane
GO:0022604	regulation of cell morphogenesis
GO:0051249	regulation of lymphocyte activation
GO:0006972	hyperosmotic response
GO:0007205	protein kinase C-activating G protein-coupled receptor signaling pathway
GO:0008209	androgen metabolic process
GO:0010818	T cell chemotaxis
GO:0034698	response to gonadotropin
GO:0045408	regulation of interleukin-6 biosynthetic process
GO:0045745	positive regulation of G protein-coupled receptor signaling pathway
GO:0095500	acetylcholine receptor signaling pathway
GO:1904994	regulation of leukocyte adhesion to vascular endothelial cell
GO:0034637	cellular carbohydrate biosynthetic process
GO:0042446	hormone biosynthetic process
GO:0016125	sterol metabolic process
GO:0010466	negative regulation of peptidase activity
GO:0006066	alcohol metabolic process
GO:0001892	embryonic placenta development
GO:0043470	regulation of carbohydrate catabolic process
GO:0001906	cell killing
GO:0007263	nitric oxide mediated signal transduction
GO:0014044	Schwann cell development
GO:0033032	regulation of myeloid cell apoptotic process
GO:0042226	interleukin-6 biosynthetic process
GO:0030307	positive regulation of cell growth
GO:0045921	positive regulation of exocytosis
GO:0061097	regulation of protein tyrosine kinase activity
GO:0098659	inorganic cation import across plasma membrane
GO:0099587	inorganic ion import across plasma membrane
GO:1903321	negative regulation of protein modification by small protein conjugation or removal

GO:1901617	organic hydroxy compound biosynthetic process
GO:0032410	negative regulation of transporter activity
GO:0032465	regulation of cytokinesis
GO:0032602	chemokine production
GO:0061053	somite development
GO:0016241	regulation of macroautophagy
GO:0032651	regulation of interleukin-1 beta production
GO:0043255	regulation of carbohydrate biosynthetic process
GO:0010743	regulation of macrophage derived foam cell differentiation
GO:0019098	reproductive behavior
GO:0048873	homeostasis of number of cells within a tissue
GO:0071549	cellular response to dexamethasone stimulus
GO:0090314	positive regulation of protein targeting to membrane
GO:1901889	negative regulation of cell junction assembly
GO:1905144	response to acetylcholine
GO:1905145	cellular response to acetylcholine
GO:0010975	regulation of neuron projection development
GO:0001659	temperature homeostasis
GO:0060333	interferon-gamma-mediated signaling pathway
GO:0006260	DNA replication
GO:0098742	cell-cell adhesion via plasma-membrane adhesion molecules
GO:0010921	regulation of phosphatase activity
GO:0046165	alcohol biosynthetic process
GO:0045833	negative regulation of lipid metabolic process
GO:0043542	endothelial cell migration
GO:0001963	synaptic transmission, dopaminergic
GO:0010543	regulation of platelet activation
GO:0031572	G2 DNA damage checkpoint
GO:0032770	positive regulation of monooxygenase activity
GO:0033198	response to ATP
GO:0040018	positive regulation of multicellular organism growth
GO:0045737	positive regulation of cyclin-dependent protein serine/threonine kinase activity
GO:0048710	regulation of astrocyte differentiation
GO:0086011	membrane repolarization during action potential
GO:0090075	relaxation of muscle
GO:0099625	ventricular cardiac muscle cell membrane repolarization
GO:0032092	positive regulation of protein binding
GO:1901655	cellular response to ketone
GO:0001959	regulation of cytokine-mediated signaling pathway
GO:0007416	synapse assembly
GO:0043488	regulation of mRNA stability
GO:0007616	long-term memory
GO:0035590	purinergic nucleotide receptor signaling pathway
GO:0038128	ERBB2 signaling pathway
GO:0060674	placenta blood vessel development
GO:0099623	regulation of cardiac muscle cell membrane repolarization
GO:0051099	positive regulation of binding

GO:2001235	positive regulation of apoptotic signaling pathway
GO:0060485	mesenchyme development
GO:0050810	regulation of steroid biosynthetic process
GO:1903076	regulation of protein localization to plasma membrane
GO:0002285	lymphocyte activation involved in immune response
GO:0019217	regulation of fatty acid metabolic process
GO:0032755	positive regulation of interleukin-6 production
GO:0090277	positive regulation of peptide hormone secretion
GO:0044262	cellular carbohydrate metabolic process
GO:0010039	response to iron ion
GO:0010955	negative regulation of protein processing
GO:0019934	cGMP-mediated signaling
GO:0033028	myeloid cell apoptotic process
GO:0048854	brain morphogenesis
GO:0060603	mammary gland duct morphogenesis
GO:1901380	negative regulation of potassium ion transmembrane transport
GO:1902692	regulation of neuroblast proliferation
GO:1903318	negative regulation of protein maturation
GO:2000352	negative regulation of endothelial cell apoptotic process
GO:0008217	regulation of blood pressure
GO:0006942	regulation of striated muscle contraction
GO:0030316	osteoclast differentiation
GO:0051591	response to cAMP
GO:0043487	regulation of RNA stability
GO:0031341	regulation of cell killing
GO:0010922	positive regulation of phosphatase activity
GO:0032102	negative regulation of response to external stimulus
GO:0046328	regulation of JNK cascade
GO:0005996	monosaccharide metabolic process
GO:0003230	cardiac atrium development
GO:0015695	organic cation transport
GO:1904031	positive regulation of cyclin-dependent protein kinase activity
GO:1905332	positive regulation of morphogenesis of an epithelium
GO:0098656	anion transmembrane transport
GO:0031532	actin cytoskeleton reorganization
GO:0032611	interleukin-1 beta production
GO:0050808	synapse organization
GO:0060759	regulation of response to cytokine stimulus
GO:0010522	regulation of calcium ion transport into cytosol
GO:0021549	cerebellum development
GO:0034329	cell junction assembly
GO:0009112	nucleobase metabolic process
GO:0010742	macrophage derived foam cell differentiation
GO:0030224	monocyte differentiation
GO:0045740	positive regulation of DNA replication
GO:0071392	cellular response to estradiol stimulus
GO:0090077	foam cell differentiation

GO:0090218	positive regulation of lipid kinase activity
GO:0090313	regulation of protein targeting to membrane
GO:1901020	negative regulation of calcium ion transmembrane transporter activity
GO:1903131	mononuclear cell differentiation
GO:0001935	endothelial cell proliferation
GO:0071478	cellular response to radiation
GO:0016236	macroautophagy
GO:0050768	negative regulation of neurogenesis
GO:0018958	phenol-containing compound metabolic process
GO:0032652	regulation of interleukin-1 production
GO:1901888	regulation of cell junction assembly
GO:0046928	regulation of neurotransmitter secretion
GO:0046486	glycerolipid metabolic process
GO:0014037	Schwann cell differentiation
GO:0030212	hyaluronan metabolic process
GO:0033280	response to vitamin D
GO:0034405	response to fluid shear stress
GO:0043029	T cell homeostasis
GO:0045730	respiratory burst
GO:0086010	membrane depolarization during action potential
GO:0007009	plasma membrane organization
GO:0008593	regulation of Notch signaling pathway
GO:0034404	nucleobase-containing small molecule biosynthetic process
GO:0043266	regulation of potassium ion transport
GO:0021510	spinal cord development
GO:0051963	regulation of synapse assembly
GO:2001023	regulation of response to drug
GO:0007190	activation of adenylate cyclase activity
GO:0010667	negative regulation of cardiac muscle cell apoptotic process
GO:0010831	positive regulation of myotube differentiation
GO:0010907	positive regulation of glucose metabolic process
GO:0032094	response to food
GO:0046676	negative regulation of insulin secretion
GO:0048246	macrophage chemotaxis
GO:0086091	regulation of heart rate by cardiac conduction
GO:0010952	positive regulation of peptidase activity
GO:0001909	leukocyte mediated cytotoxicity
GO:0030330	DNA damage response, signal transduction by p53 class mediator
GO:0050905	neuromuscular process
GO:0050866	negative regulation of cell activation
GO:0061013	regulation of mRNA catabolic process
GO:0009062	fatty acid catabolic process
GO:1904659	glucose transmembrane transport
GO:0060306	regulation of membrane repolarization
GO:1905521	regulation of macrophage migration
GO:2000648	positive regulation of stem cell proliferation
GO:2000826	regulation of heart morphogenesis

GO:0021761	limbic system development
GO:0051149	positive regulation of muscle cell differentiation
GO:0016101	diterpenoid metabolic process
GO:0001990	regulation of systemic arterial blood pressure by hormone
GO:0007618	mating
GO:0019835	cytolysis
GO:0021532	neural tube patterning
GO:0021795	cerebral cortex cell migration
GO:0042596	fear response
GO:0044058	regulation of digestive system process
GO:0046621	negative regulation of organ growth
GO:0051281	positive regulation of release of sequestered calcium ion into cytosol
GO:0071548	response to dexamethasone
GO:0072583	clathrin-dependent endocytosis
GO:0022037	metencephalon development
GO:0031623	receptor internalization
GO:0035637	multicellular organismal signaling
GO:1902905	positive regulation of supramolecular fiber organization
GO:0001938	positive regulation of endothelial cell proliferation
GO:0008645	hexose transmembrane transport
GO:0010906	regulation of glucose metabolic process
GO:0014902	myotube differentiation
GO:0098693	regulation of synaptic vesicle cycle
GO:0030100	regulation of endocytosis
GO:0010664	negative regulation of striated muscle cell apoptotic process
GO:0042572	retinol metabolic process
GO:0043267	negative regulation of potassium ion transport
GO:0050873	brown fat cell differentiation
GO:0006898	receptor-mediated endocytosis
GO:0002223	stimulatory C-type lectin receptor signaling pathway
GO:0006096	glycolytic process
GO:0021987	cerebral cortex development
GO:0043200	response to amino acid
GO:0070555	response to interleukin-1
GO:0006757	ATP generation from ADP
GO:0015749	monosaccharide transmembrane transport
GO:0090276	regulation of peptide hormone secretion
GO:0031641	regulation of myelination
GO:0046688	response to copper ion
GO:0099622	cardiac muscle cell membrane repolarization
GO:0035303	regulation of dephosphorylation
GO:0032612	interleukin-1 production
GO:1900542	regulation of purine nucleotide metabolic process
GO:0002220	innate immune response activating cell surface receptor signaling pathway
GO:0034219	carbohydrate transmembrane transport
GO:0006775	fat-soluble vitamin metabolic process
GO:0008631	intrinsic apoptotic signaling pathway in response to oxidative stress

GO:0045332	phospholipid translocation
GO:0045687	positive regulation of glial cell differentiation
GO:0045746	negative regulation of Notch signaling pathway
GO:1903170	negative regulation of calcium ion transmembrane transport
GO:1903531	negative regulation of secretion by cell
GO:0002758	innate immune response-activating signal transduction
GO:0006140	regulation of nucleotide metabolic process
GO:1902749	regulation of cell cycle G2/M phase transition
GO:0045089	positive regulation of innate immune response
GO:2001020	regulation of response to DNA damage stimulus
GO:0030225	macrophage differentiation
GO:0034204	lipid translocation
GO:0035307	positive regulation of protein dephosphorylation
GO:0044060	regulation of endocrine process
GO:0060999	positive regulation of dendritic spine development
GO:0061756	leukocyte adhesion to vascular endothelial cell
GO:0007265	Ras protein signal transduction
GO:0022612	gland morphogenesis
GO:0090278	negative regulation of peptide hormone secretion
GO:0008406	gonad development
GO:0044242	cellular lipid catabolic process
GO:0070252	actin-mediated cell contraction
GO:1903578	regulation of ATP metabolic process
GO:0006282	regulation of DNA repair
GO:0046031	ADP metabolic process
GO:0001504	neurotransmitter uptake
GO:0002067	glandular epithelial cell differentiation
GO:0002437	inflammatory response to antigenic stimulus
GO:0007157	heterophilic cell-cell adhesion via plasma membrane cell adhesion molecules
GO:0032369	negative regulation of lipid transport
GO:0045776	negative regulation of blood pressure
GO:0060443	mammary gland morphogenesis
GO:0060711	labyrinthine layer development
GO:1900015	regulation of cytokine production involved in inflammatory response
GO:0090150	establishment of protein localization to membrane
GO:0048762	mesenchymal cell differentiation
GO:0051251	positive regulation of lymphocyte activation

GeneRatio	BgRatio	pvalue	p.adjust	qvalue
18/87	299/18670	2.05E-15	6.64E-12	3.63E-12
18/87	322/18670	7.45E-15	1.21E-11	6.60E-12
18/87	333/18670	1.33E-14	1.44E-11	7.88E-12
17/87	334/18670	2.09E-13	1.69E-10	9.27E-11
19/87	458/18670	2.64E-13	1.71E-10	9.35E-11
19/87	471/18670	4.33E-13	2.34E-10	1.28E-10
19/87	493/18670	9.70E-13	4.49E-10	2.46E-10
18/87	433/18670	1.18E-12	4.76E-10	2.61E-10
18/87	440/18670	1.54E-12	5.54E-10	3.03E-10
6/87	10/18670	1.78E-12	5.76E-10	3.15E-10
18/87	456/18670	2.80E-12	8.25E-10	4.51E-10
15/87	290/18670	4.97E-12	1.34E-09	7.35E-10
13/87	208/18670	1.39E-11	3.32E-09	1.82E-09
14/87	258/18670	1.43E-11	3.32E-09	1.82E-09
17/87	448/18670	2.27E-11	4.88E-09	2.67E-09
15/87	324/18670	2.41E-11	4.88E-09	2.67E-09
15/87	337/18670	4.20E-11	7.67E-09	4.20E-09
10/87	102/18670	4.26E-11	7.67E-09	4.20E-09
12/87	181/18670	4.50E-11	7.68E-09	4.20E-09
12/87	184/18670	5.46E-11	8.84E-09	4.84E-09
13/87	235/18670	6.41E-11	9.90E-09	5.42E-09
17/87	483/18670	7.35E-11	1.08E-08	5.93E-09
14/87	295/18670	8.55E-11	1.18E-08	6.43E-09
11/87	148/18670	8.92E-11	1.18E-08	6.43E-09
14/87	297/18670	9.35E-11	1.18E-08	6.43E-09
15/87	357/18670	9.43E-11	1.18E-08	6.43E-09
15/87	363/18670	1.19E-10	1.43E-08	7.82E-09
10/87	114/18670	1.30E-10	1.46E-08	7.98E-09
15/87	366/18670	1.34E-10	1.46E-08	7.98E-09
16/87	432/18670	1.35E-10	1.46E-08	7.98E-09
6/87	18/18670	1.53E-10	1.59E-08	8.73E-09
8/87	58/18670	2.54E-10	2.57E-08	1.41E-08
15/87	385/18670	2.70E-10	2.65E-08	1.45E-08
10/87	124/18670	3.01E-10	2.86E-08	1.57E-08
10/87	126/18670	3.52E-10	3.26E-08	1.78E-08
16/87	464/18670	3.84E-10	3.41E-08	1.87E-08
12/87	218/18670	3.90E-10	3.41E-08	1.87E-08
9/87	95/18670	5.75E-10	4.90E-08	2.68E-08
10/87	134/18670	6.47E-10	5.38E-08	2.94E-08
12/87	229/18670	6.86E-10	5.55E-08	3.04E-08
14/87	348/18670	7.46E-10	5.89E-08	3.23E-08
13/87	292/18670	9.34E-10	7.21E-08	3.94E-08
9/87	101/18670	9.99E-10	7.53E-08	4.12E-08
16/87	499/18670	1.10E-09	8.04E-08	4.40E-08
12/87	239/18670	1.12E-09	8.04E-08	4.40E-08

15/87	434/18670	1.40E-09	9.89E-08	5.41E-08
14/87	370/18670	1.65E-09	1.14E-07	6.22E-08
9/87	109/18670	1.98E-09	1.31E-07	7.16E-08
9/87	109/18670	1.98E-09	1.31E-07	7.16E-08
7/87	47/18670	2.02E-09	1.31E-07	7.16E-08
8/87	75/18670	2.09E-09	1.33E-07	7.29E-08
12/87	256/18670	2.44E-09	1.49E-07	8.15E-08
12/87	256/18670	2.44E-09	1.49E-07	8.15E-08
9/87	113/18670	2.73E-09	1.61E-07	8.80E-08
9/87	113/18670	2.73E-09	1.61E-07	8.80E-08
14/87	394/18670	3.69E-09	2.14E-07	1.17E-07
8/87	81/18670	3.90E-09	2.22E-07	1.21E-07
8/87	86/18670	6.32E-09	3.53E-07	1.93E-07
8/87	87/18670	6.93E-09	3.81E-07	2.08E-07
12/87	282/18670	7.23E-09	3.91E-07	2.14E-07
12/87	286/18670	8.46E-09	4.50E-07	2.46E-07
10/87	176/18670	9.17E-09	4.79E-07	2.62E-07
7/87	59/18670	1.05E-08	5.30E-07	2.90E-07
7/87	59/18670	1.05E-08	5.30E-07	2.90E-07
12/87	292/18670	1.07E-08	5.32E-07	2.91E-07
14/87	434/18670	1.26E-08	6.18E-07	3.38E-07
14/87	436/18670	1.33E-08	6.36E-07	3.48E-07
14/87	436/18670	1.33E-08	6.36E-07	3.48E-07
14/87	437/18670	1.37E-08	6.45E-07	3.53E-07
14/87	439/18670	1.45E-08	6.73E-07	3.69E-07
8/87	100/18670	2.10E-08	9.60E-07	5.25E-07
9/87	143/18670	2.18E-08	9.79E-07	5.36E-07
10/87	193/18670	2.21E-08	9.83E-07	5.38E-07
12/87	314/18670	2.39E-08	1.05E-06	5.72E-07
10/87	195/18670	2.44E-08	1.05E-06	5.77E-07
12/87	315/18670	2.47E-08	1.05E-06	5.77E-07
11/87	254/18670	2.68E-08	1.13E-06	6.17E-07
12/87	319/18670	2.84E-08	1.18E-06	6.44E-07
5/87	20/18670	2.87E-08	1.18E-06	6.44E-07
12/87	321/18670	3.04E-08	1.23E-06	6.75E-07
10/87	204/18670	3.75E-08	1.48E-06	8.10E-07
10/87	204/18670	3.75E-08	1.48E-06	8.10E-07
7/87	71/18670	3.90E-08	1.52E-06	8.32E-07
6/87	42/18670	3.94E-08	1.52E-06	8.32E-07
12/87	330/18670	4.12E-08	1.57E-06	8.61E-07
7/87	72/18670	4.30E-08	1.61E-06	8.82E-07
14/87	479/18670	4.33E-08	1.61E-06	8.82E-07
6/87	43/18670	4.56E-08	1.68E-06	9.20E-07
9/87	157/18670	4.90E-08	1.78E-06	9.76E-07
8/87	112/18670	5.14E-08	1.82E-06	9.93E-07
14/87	486/18670	5.18E-08	1.82E-06	9.93E-07
7/87	74/18670	5.22E-08	1.82E-06	9.93E-07

6/87	44/18670	5.26E-08	1.82E-06	9.93E-07
6/87	44/18670	5.26E-08	1.82E-06	9.93E-07
8/87	113/18670	5.51E-08	1.87E-06	1.02E-06
12/87	339/18670	5.54E-08	1.87E-06	1.02E-06
11/87	275/18670	6.03E-08	2.02E-06	1.10E-06
12/87	343/18670	6.29E-08	2.08E-06	1.14E-06
10/87	217/18670	6.71E-08	2.19E-06	1.20E-06
8/87	116/18670	6.76E-08	2.19E-06	1.20E-06
12/87	347/18670	7.14E-08	2.29E-06	1.25E-06
10/87	221/18670	7.96E-08	2.53E-06	1.38E-06
11/87	284/18670	8.36E-08	2.63E-06	1.44E-06
13/87	428/18670	8.84E-08	2.76E-06	1.51E-06
12/87	355/18670	9.15E-08	2.82E-06	1.55E-06
9/87	169/18670	9.23E-08	2.82E-06	1.55E-06
13/87	434/18670	1.04E-07	3.15E-06	1.72E-06
12/87	362/18670	1.13E-07	3.39E-06	1.86E-06
5/87	26/18670	1.19E-07	3.54E-06	1.94E-06
6/87	51/18670	1.31E-07	3.86E-06	2.11E-06
5/87	27/18670	1.46E-07	4.25E-06	2.33E-06
12/87	374/18670	1.61E-07	4.66E-06	2.55E-06
10/87	239/18670	1.65E-07	4.74E-06	2.60E-06
10/87	241/18670	1.79E-07	5.08E-06	2.78E-06
11/87	313/18670	2.23E-07	6.26E-06	3.43E-06
13/87	464/18670	2.24E-07	6.26E-06	3.43E-06
10/87	250/18670	2.51E-07	6.94E-06	3.80E-06
11/87	317/18670	2.53E-07	6.94E-06	3.80E-06
7/87	93/18670	2.56E-07	6.96E-06	3.81E-06
6/87	57/18670	2.58E-07	6.96E-06	3.81E-06
10/87	251/18670	2.60E-07	6.97E-06	3.82E-06
11/87	319/18670	2.69E-07	7.15E-06	3.91E-06
13/87	472/18670	2.72E-07	7.18E-06	3.93E-06
7/87	94/18670	2.75E-07	7.20E-06	3.94E-06
11/87	321/18670	2.86E-07	7.43E-06	4.06E-06
7/87	95/18670	2.96E-07	7.62E-06	4.17E-06
8/87	142/18670	3.24E-07	8.26E-06	4.52E-06
12/87	402/18670	3.49E-07	8.83E-06	4.83E-06
6/87	60/18670	3.52E-07	8.84E-06	4.84E-06
12/87	403/18670	3.58E-07	8.93E-06	4.89E-06
4/87	14/18670	4.25E-07	1.05E-05	5.75E-06
7/87	101/18670	4.51E-07	1.11E-05	6.05E-06
6/87	63/18670	4.72E-07	1.14E-05	6.25E-06
6/87	63/18670	4.72E-07	1.14E-05	6.25E-06
5/87	34/18670	4.89E-07	1.16E-05	6.33E-06
5/87	34/18670	4.89E-07	1.16E-05	6.33E-06
5/87	34/18670	4.89E-07	1.16E-05	6.33E-06
10/87	269/18670	4.92E-07	1.16E-05	6.33E-06
10/87	270/18670	5.09E-07	1.19E-05	6.50E-06

7/87	103/18670	5.15E-07	1.19E-05	6.53E-06
11/87	341/18670	5.22E-07	1.20E-05	6.56E-06
11/87	342/18670	5.37E-07	1.21E-05	6.61E-06
11/87	342/18670	5.37E-07	1.21E-05	6.61E-06
9/87	208/18670	5.38E-07	1.21E-05	6.61E-06
8/87	152/18670	5.44E-07	1.21E-05	6.61E-06
8/87	152/18670	5.44E-07	1.21E-05	6.61E-06
9/87	210/18670	5.83E-07	1.29E-05	7.04E-06
6/87	66/18670	6.24E-07	1.37E-05	7.48E-06
9/87	215/18670	7.10E-07	1.53E-05	8.40E-06
9/87	215/18670	7.10E-07	1.53E-05	8.40E-06
6/87	68/18670	7.47E-07	1.60E-05	8.77E-06
5/87	37/18670	7.58E-07	1.62E-05	8.85E-06
10/87	285/18670	8.34E-07	1.77E-05	9.66E-06
11/87	358/18670	8.42E-07	1.77E-05	9.70E-06
11/87	362/18670	9.39E-07	1.96E-05	1.07E-05
11/87	363/18670	9.65E-07	2.00E-05	1.10E-05
9/87	224/18670	1.00E-06	2.06E-05	1.13E-05
5/87	40/18670	1.13E-06	2.31E-05	1.26E-05
6/87	73/18670	1.14E-06	2.31E-05	1.26E-05
6/87	73/18670	1.14E-06	2.31E-05	1.26E-05
7/87	116/18670	1.15E-06	2.31E-05	1.26E-05
8/87	168/18670	1.16E-06	2.31E-05	1.26E-05
8/87	168/18670	1.16E-06	2.31E-05	1.26E-05
12/87	451/18670	1.17E-06	2.31E-05	1.26E-05
10/87	296/18670	1.18E-06	2.31E-05	1.26E-05
8/87	169/18670	1.22E-06	2.37E-05	1.30E-05
6/87	74/18670	1.24E-06	2.40E-05	1.31E-05
10/87	300/18670	1.33E-06	2.55E-05	1.39E-05
8/87	171/18670	1.33E-06	2.55E-05	1.39E-05
9/87	232/18670	1.34E-06	2.55E-05	1.40E-05
7/87	119/18670	1.37E-06	2.60E-05	1.42E-05
7/87	120/18670	1.45E-06	2.73E-05	1.50E-05
6/87	77/18670	1.56E-06	2.93E-05	1.60E-05
9/87	237/18670	1.60E-06	2.97E-05	1.63E-05
9/87	238/18670	1.65E-06	3.06E-05	1.67E-05
10/87	308/18670	1.68E-06	3.10E-05	1.69E-05
12/87	468/18670	1.73E-06	3.16E-05	1.73E-05
7/87	124/18670	1.81E-06	3.29E-05	1.80E-05
5/87	44/18670	1.84E-06	3.33E-05	1.82E-05
7/87	125/18670	1.91E-06	3.44E-05	1.88E-05
9/87	244/18670	2.03E-06	3.63E-05	1.99E-05
6/87	81/18670	2.11E-06	3.76E-05	2.06E-05
9/87	246/18670	2.17E-06	3.84E-05	2.10E-05
7/87	128/18670	2.24E-06	3.94E-05	2.16E-05
6/87	82/18670	2.27E-06	3.97E-05	2.17E-05
8/87	184/18670	2.30E-06	4.00E-05	2.19E-05

5/87	46/18670	2.31E-06	4.00E-05	2.19E-05
4/87	21/18670	2.48E-06	4.27E-05	2.34E-05
5/87	47/18670	2.57E-06	4.39E-05	2.40E-05
5/87	47/18670	2.57E-06	4.39E-05	2.40E-05
6/87	85/18670	2.80E-06	4.75E-05	2.60E-05
5/87	48/18670	2.86E-06	4.80E-05	2.63E-05
5/87	48/18670	2.86E-06	4.80E-05	2.63E-05
7/87	133/18670	2.89E-06	4.83E-05	2.64E-05
6/87	86/18670	3.00E-06	4.97E-05	2.72E-05
9/87	256/18670	3.01E-06	4.97E-05	2.72E-05
5/87	49/18670	3.17E-06	5.20E-05	2.84E-05
5/87	49/18670	3.17E-06	5.20E-05	2.84E-05
10/87	331/18670	3.20E-06	5.21E-05	2.85E-05
12/87	499/18670	3.35E-06	5.43E-05	2.97E-05
10/87	333/18670	3.38E-06	5.44E-05	2.98E-05
6/87	88/18670	3.43E-06	5.51E-05	3.01E-05
9/87	262/18670	3.63E-06	5.77E-05	3.16E-05
4/87	23/18670	3.64E-06	5.77E-05	3.16E-05
10/87	336/18670	3.66E-06	5.77E-05	3.16E-05
6/87	89/18670	3.67E-06	5.77E-05	3.16E-05
10/87	337/18670	3.75E-06	5.88E-05	3.22E-05
7/87	140/18670	4.06E-06	6.27E-05	3.43E-05
7/87	140/18670	4.06E-06	6.27E-05	3.43E-05
7/87	140/18670	4.06E-06	6.27E-05	3.43E-05
5/87	52/18670	4.28E-06	6.57E-05	3.60E-05
4/87	24/18670	4.35E-06	6.62E-05	3.62E-05
9/87	268/18670	4.37E-06	6.62E-05	3.62E-05
9/87	268/18670	4.37E-06	6.62E-05	3.62E-05
11/87	425/18670	4.43E-06	6.67E-05	3.65E-05
8/87	202/18670	4.60E-06	6.90E-05	3.77E-05
7/87	143/18670	4.68E-06	6.95E-05	3.81E-05
7/87	143/18670	4.68E-06	6.95E-05	3.81E-05
5/87	53/18670	4.71E-06	6.97E-05	3.81E-05
6/87	93/18670	4.74E-06	6.98E-05	3.82E-05
7/87	144/18670	4.90E-06	7.18E-05	3.93E-05
7/87	145/18670	5.13E-06	7.49E-05	4.10E-05
5/87	54/18670	5.17E-06	7.51E-05	4.11E-05
10/87	350/18670	5.24E-06	7.59E-05	4.15E-05
11/87	434/18670	5.40E-06	7.78E-05	4.26E-05
9/87	276/18670	5.55E-06	7.95E-05	4.35E-05
7/87	147/18670	5.61E-06	8.01E-05	4.39E-05
5/87	55/18670	5.67E-06	8.05E-05	4.40E-05
6/87	96/18670	5.70E-06	8.05E-05	4.40E-05
9/87	277/18670	5.71E-06	8.05E-05	4.40E-05
6/87	97/18670	6.05E-06	8.49E-05	4.65E-05
4/87	26/18670	6.08E-06	8.49E-05	4.65E-05
7/87	149/18670	6.14E-06	8.53E-05	4.67E-05

4/87	27/18670	7.11E-06	9.85E-05	5.39E-05
6/87	100/18670	7.22E-06	9.91E-05	5.43E-05
6/87	100/18670	7.22E-06	9.91E-05	5.43E-05
7/87	153/18670	7.30E-06	9.94E-05	5.44E-05
7/87	153/18670	7.30E-06	9.94E-05	5.44E-05
6/87	101/18670	7.65E-06	0.0001037	5.67E-05
9/87	288/18670	7.82E-06	0.0001056	5.78E-05
4/87	28/18670	8.27E-06	0.0001106	6.05E-05
7/87	156/18670	8.30E-06	0.0001106	6.05E-05
7/87	156/18670	8.30E-06	0.0001106	6.05E-05
7/87	157/18670	8.65E-06	0.0001144	6.26E-05
7/87	157/18670	8.65E-06	0.0001144	6.26E-05
6/87	104/18670	9.05E-06	0.0001183	6.48E-05
6/87	104/18670	9.05E-06	0.0001183	6.48E-05
6/87	104/18670	9.05E-06	0.0001183	6.48E-05
10/87	373/18670	9.16E-06	0.0001192	6.52E-05
4/87	29/18670	9.56E-06	0.0001235	6.76E-05
6/87	105/18670	9.57E-06	0.0001235	6.76E-05
7/87	160/18670	9.79E-06	0.0001259	6.89E-05
6/87	106/18670	1.01E-05	0.0001294	7.08E-05
5/87	62/18670	1.03E-05	0.0001305	7.14E-05
5/87	62/18670	1.03E-05	0.0001305	7.14E-05
10/87	380/18670	1.08E-05	0.0001363	7.46E-05
4/87	30/18670	1.10E-05	0.000138	7.55E-05
4/87	30/18670	1.10E-05	0.000138	7.55E-05
5/87	63/18670	1.11E-05	0.0001391	7.61E-05
6/87	108/18670	1.12E-05	0.0001397	7.64E-05
6/87	108/18670	1.12E-05	0.0001397	7.64E-05
9/87	302/18670	1.14E-05	0.0001415	7.74E-05
10/87	383/18670	1.15E-05	0.0001421	7.77E-05
10/87	384/18670	1.18E-05	0.0001448	7.92E-05
9/87	304/18670	1.21E-05	0.0001469	8.04E-05
9/87	304/18670	1.21E-05	0.0001469	8.04E-05
4/87	31/18670	1.26E-05	0.0001526	8.35E-05
7/87	167/18670	1.29E-05	0.0001562	8.55E-05
5/87	65/18670	1.30E-05	0.0001562	8.55E-05
6/87	111/18670	1.32E-05	0.000158	8.65E-05
6/87	112/18670	1.39E-05	0.0001657	9.07E-05
5/87	66/18670	1.40E-05	0.0001666	9.12E-05
4/87	32/18670	1.43E-05	0.0001681	9.20E-05
4/87	32/18670	1.43E-05	0.0001681	9.20E-05
4/87	32/18670	1.43E-05	0.0001681	9.20E-05
4/87	32/18670	1.43E-05	0.0001681	9.20E-05
5/87	67/18670	1.51E-05	0.0001761	9.64E-05
6/87	114/18670	1.53E-05	0.0001787	9.78E-05
11/87	486/18670	1.56E-05	0.000181	9.91E-05
3/87	11/18670	1.57E-05	0.000181	9.91E-05

3/87	11/18670	1.57E-05	0.000181	9.91E-05
10/87	398/18670	1.61E-05	0.0001847	0.0001011
9/87	316/18670	1.64E-05	0.0001878	0.0001028
5/87	69/18670	1.74E-05	0.0001984	0.0001086
7/87	175/18670	1.75E-05	0.0001994	0.0001091
7/87	176/18670	1.82E-05	0.0002054	0.0001124
7/87	176/18670	1.82E-05	0.0002054	0.0001124
4/87	34/18670	1.83E-05	0.0002063	0.0001129
11/87	495/18670	1.85E-05	0.0002078	0.0001137
5/87	70/18670	1.87E-05	0.0002085	0.0001141
7/87	177/18670	1.89E-05	0.0002101	0.000115
7/87	179/18670	2.03E-05	0.0002251	0.0001232
6/87	120/18670	2.05E-05	0.0002251	0.0001232
6/87	120/18670	2.05E-05	0.0002251	0.0001232
4/87	35/18670	2.06E-05	0.0002251	0.0001232
4/87	35/18670	2.06E-05	0.0002251	0.0001232
4/87	35/18670	2.06E-05	0.0002251	0.0001232
10/87	410/18670	2.07E-05	0.0002255	0.0001234
7/87	180/18670	2.10E-05	0.0002272	0.0001243
7/87	180/18670	2.10E-05	0.0002272	0.0001243
5/87	72/18670	2.14E-05	0.0002306	0.0001262
6/87	122/18670	2.26E-05	0.0002405	0.0001316
6/87	122/18670	2.26E-05	0.0002405	0.0001316
6/87	122/18670	2.26E-05	0.0002405	0.0001316
5/87	73/18670	2.29E-05	0.0002434	0.0001332
10/87	415/18670	2.30E-05	0.0002436	0.0001333
6/87	123/18670	2.36E-05	0.0002464	0.0001348
8/87	253/18670	2.36E-05	0.0002464	0.0001348
9/87	331/18670	2.36E-05	0.0002464	0.0001348
9/87	331/18670	2.36E-05	0.0002464	0.0001348
9/87	331/18670	2.36E-05	0.0002464	0.0001348
5/87	74/18670	2.45E-05	0.0002526	0.0001382
5/87	74/18670	2.45E-05	0.0002526	0.0001382
5/87	74/18670	2.45E-05	0.0002526	0.0001382
4/87	37/18670	2.58E-05	0.0002649	0.000145
4/87	37/18670	2.58E-05	0.0002649	0.000145
5/87	75/18670	2.61E-05	0.0002663	0.0001457
5/87	75/18670	2.61E-05	0.0002663	0.0001457
7/87	187/18670	2.69E-05	0.000273	0.0001494
3/87	13/18670	2.70E-05	0.0002737	0.0001498
7/87	188/18670	2.78E-05	0.0002795	0.000153
5/87	76/18670	2.79E-05	0.0002795	0.000153
8/87	259/18670	2.79E-05	0.0002795	0.000153
8/87	259/18670	2.79E-05	0.0002795	0.000153
6/87	127/18670	2.83E-05	0.0002824	0.0001546
4/87	38/18670	2.88E-05	0.0002842	0.0001556
4/87	38/18670	2.88E-05	0.0002842	0.0001556

4/87	38/18670	2.88E-05	0.0002842	0.0001556
8/87	261/18670	2.95E-05	0.0002908	0.0001592
8/87	262/18670	3.03E-05	0.0002979	0.0001631
10/87	429/18670	3.05E-05	0.0002987	0.0001635
7/87	191/18670	3.08E-05	0.0003004	0.0001644
5/87	78/18670	3.16E-05	0.0003067	0.0001679
5/87	78/18670	3.16E-05	0.0003067	0.0001679
10/87	431/18670	3.17E-05	0.0003069	0.000168
7/87	192/18670	3.18E-05	0.0003069	0.000168
4/87	39/18670	3.19E-05	0.0003072	0.0001681
5/87	79/18670	3.36E-05	0.0003218	0.0001761
10/87	434/18670	3.37E-05	0.0003218	0.0001761
3/87	14/18670	3.43E-05	0.0003258	0.0001783
3/87	14/18670	3.43E-05	0.0003258	0.0001783
5/87	81/18670	3.79E-05	0.0003596	0.0001968
4/87	41/18670	3.90E-05	0.000369	0.0002019
8/87	272/18670	3.96E-05	0.0003729	0.0002041
5/87	83/18670	4.27E-05	0.000401	0.0002194
4/87	42/18670	4.30E-05	0.0004017	0.0002198
4/87	42/18670	4.30E-05	0.0004017	0.0002198
9/87	359/18670	4.46E-05	0.000415	0.0002271
5/87	84/18670	4.52E-05	0.0004187	0.0002292
5/87	84/18670	4.52E-05	0.0004187	0.0002292
7/87	204/18670	4.68E-05	0.0004289	0.0002347
7/87	204/18670	4.68E-05	0.0004289	0.0002347
6/87	139/18670	4.71E-05	0.0004289	0.0002347
6/87	139/18670	4.71E-05	0.0004289	0.0002347
6/87	139/18670	4.71E-05	0.0004289	0.0002347
4/87	43/18670	4.72E-05	0.0004289	0.0002347
4/87	43/18670	4.72E-05	0.0004289	0.0002347
6/87	140/18670	4.90E-05	0.0004434	0.0002426
5/87	86/18670	5.06E-05	0.0004559	0.0002495
5/87	86/18670	5.06E-05	0.0004559	0.0002495
6/87	141/18670	5.10E-05	0.0004575	0.0002504
4/87	44/18670	5.18E-05	0.0004637	0.0002538
3/87	16/18670	5.24E-05	0.0004651	0.0002546
3/87	16/18670	5.24E-05	0.0004651	0.0002546
3/87	16/18670	5.24E-05	0.0004651	0.0002546
5/87	87/18670	5.35E-05	0.0004741	0.0002594
9/87	369/18670	5.51E-05	0.0004855	0.0002657
6/87	143/18670	5.51E-05	0.0004855	0.0002657
7/87	210/18670	5.62E-05	0.0004895	0.0002679
7/87	210/18670	5.62E-05	0.0004895	0.0002679
9/87	370/18670	5.63E-05	0.0004895	0.0002679
5/87	88/18670	5.66E-05	0.0004895	0.0002679
4/87	45/18670	5.66E-05	0.0004895	0.0002679
4/87	45/18670	5.66E-05	0.0004895	0.0002679

4/87	45/18670	5.66E-05	0.0004895	0.0002679
7/87	212/18670	5.97E-05	0.0005146	0.0002816
4/87	46/18670	6.18E-05	0.0005272	0.0002885
4/87	46/18670	6.18E-05	0.0005272	0.0002885
4/87	46/18670	6.18E-05	0.0005272	0.0002885
4/87	46/18670	6.18E-05	0.0005272	0.0002885
5/87	90/18670	6.30E-05	0.0005296	0.0002899
5/87	90/18670	6.30E-05	0.0005296	0.0002899
7/87	214/18670	6.33E-05	0.0005296	0.0002899
7/87	214/18670	6.33E-05	0.0005296	0.0002899
7/87	214/18670	6.33E-05	0.0005296	0.0002899
3/87	17/18670	6.34E-05	0.0005296	0.0002899
3/87	17/18670	6.34E-05	0.0005296	0.0002899
10/87	468/18670	6.34E-05	0.0005296	0.0002899
8/87	291/18670	6.37E-05	0.0005305	0.0002903
6/87	147/18670	6.43E-05	0.0005342	0.0002924
7/87	216/18670	6.72E-05	0.0005566	0.0003046
4/87	47/18670	6.73E-05	0.0005566	0.0003046
10/87	472/18670	6.81E-05	0.0005613	0.0003072
7/87	217/18670	6.91E-05	0.0005643	0.0003088
7/87	217/18670	6.91E-05	0.0005643	0.0003088
10/87	473/18670	6.93E-05	0.0005643	0.0003088
10/87	473/18670	6.93E-05	0.0005643	0.0003088
6/87	149/18670	6.93E-05	0.0005643	0.0003088
7/87	218/18670	7.12E-05	0.0005781	0.0003164
6/87	150/18670	7.19E-05	0.0005827	0.0003189
4/87	48/18670	7.32E-05	0.0005861	0.0003208
4/87	48/18670	7.32E-05	0.0005861	0.0003208
4/87	48/18670	7.32E-05	0.0005861	0.0003208
4/87	48/18670	7.32E-05	0.0005861	0.0003208
7/87	219/18670	7.32E-05	0.0005861	0.0003208
5/87	93/18670	7.37E-05	0.0005881	0.0003218
8/87	298/18670	7.52E-05	0.0005987	0.0003276
3/87	18/18670	7.58E-05	0.0006008	0.0003288
3/87	18/18670	7.58E-05	0.0006008	0.0003288
6/87	152/18670	7.74E-05	0.0006118	0.0003348
4/87	49/18670	7.94E-05	0.0006247	0.0003419
4/87	49/18670	7.94E-05	0.0006247	0.0003419
6/87	153/18670	8.03E-05	0.0006298	0.0003447
5/87	95/18670	8.15E-05	0.0006383	0.0003493
5/87	96/18670	8.57E-05	0.0006653	0.0003641
4/87	50/18670	8.60E-05	0.0006653	0.0003641
4/87	50/18670	8.60E-05	0.0006653	0.0003641
4/87	50/18670	8.60E-05	0.0006653	0.0003641
4/87	50/18670	8.60E-05	0.0006653	0.0003641
8/87	305/18670	8.84E-05	0.0006819	0.0003732
3/87	19/18670	8.97E-05	0.0006891	0.0003771

3/87	19/18670	8.97E-05	0.0006891	0.0003771
9/87	394/18670	9.10E-05	0.0006973	0.0003816
8/87	308/18670	9.46E-05	0.0007229	0.0003956
6/87	158/18670	9.59E-05	0.000731	0.0004
4/87	52/18670	0.000100393	0.0007638	0.000418
7/87	231/18670	0.000102248	0.0007747	0.000424
10/87	496/18670	0.000102547	0.0007747	0.000424
10/87	496/18670	0.000102547	0.0007747	0.000424
8/87	312/18670	0.000103418	0.0007795	0.0004266
5/87	100/18670	0.000104027	0.0007823	0.0004281
3/87	20/18670	0.00010521	0.0007893	0.000432
4/87	53/18670	0.000108204	0.0008099	0.0004432
5/87	101/18670	0.000109047	0.0008143	0.0004457
10/87	500/18670	0.000109536	0.0008161	0.0004466
6/87	163/18670	0.000113772	0.0008438	0.0004618
6/87	163/18670	0.000113772	0.0008438	0.0004618
5/87	102/18670	0.000114251	0.0008454	0.0004627
8/87	317/18670	0.000115421	0.0008521	0.0004663
4/87	54/18670	0.000116447	0.0008577	0.0004694
7/87	236/18670	0.000116811	0.0008585	0.0004698
5/87	103/18670	0.000119643	0.0008773	0.0004801
3/87	21/18670	0.000122332	0.000891	0.0004876
3/87	21/18670	0.000122332	0.000891	0.0004876
3/87	21/18670	0.000122332	0.000891	0.0004876
4/87	55/18670	0.000125136	0.000908	0.0004969
5/87	104/18670	0.000125228	0.000908	0.0004969
5/87	106/18670	0.000136997	0.0009911	0.0005424
6/87	169/18670	0.000138684	0.0010011	0.0005478
3/87	22/18670	0.000141171	0.0010145	0.0005552
3/87	22/18670	0.000141171	0.0010145	0.0005552
4/87	57/18670	0.000143909	0.0010251	0.000561
4/87	57/18670	0.000143909	0.0010251	0.000561
4/87	57/18670	0.000143909	0.0010251	0.000561
4/87	57/18670	0.000143909	0.0010251	0.000561
4/87	58/18670	0.000154022	0.0010923	0.0005978
4/87	58/18670	0.000154022	0.0010923	0.0005978
7/87	247/18670	0.00015483	0.0010956	0.0005996
5/87	109/18670	0.000156217	0.001103	0.0006037
3/87	23/18670	0.0001618	0.0011329	0.00062
3/87	23/18670	0.0001618	0.0011329	0.00062
3/87	23/18670	0.0001618	0.0011329	0.00062
8/87	333/18670	0.00016184	0.0011329	0.00062
6/87	174/18670	0.000162589	0.0011357	0.0006215
4/87	59/18670	0.00016464	0.0011426	0.0006253
4/87	59/18670	0.00016464	0.0011426	0.0006253
4/87	59/18670	0.00016464	0.0011426	0.0006253
5/87	111/18670	0.000170131	0.0011782	0.0006448

9/87	429/18670	0.00017283	0.0011943	0.0006536
3/87	24/18670	0.000184293	0.0012681	0.000694
3/87	24/18670	0.000184293	0.0012681	0.000694
4/87	61/18670	0.000187446	0.0012871	0.0007044
7/87	255/18670	0.000188339	0.0012905	0.0007062
5/87	114/18670	0.000192755	0.0013152	0.0007198
5/87	114/18670	0.000192755	0.0013152	0.0007198
6/87	180/18670	0.000195459	0.0013308	0.0007283
8/87	343/18670	0.000197981	0.0013452	0.0007362
4/87	62/18670	0.000199665	0.0013538	0.0007409
3/87	25/18670	0.00020872	0.0014116	0.0007725
5/87	116/18670	0.000209065	0.0014116	0.0007725
8/87	346/18670	0.000210033	0.0014152	0.0007745
7/87	260/18670	0.000212105	0.0014197	0.0007769
4/87	63/18670	0.000212448	0.0014197	0.0007769
4/87	63/18670	0.000212448	0.0014197	0.0007769
4/87	63/18670	0.000212448	0.0014197	0.0007769
6/87	183/18670	0.000213751	0.0014254	0.0007801
6/87	184/18670	0.00022014	0.001459	0.0007985
6/87	184/18670	0.00022014	0.001459	0.0007985
6/87	184/18670	0.00022014	0.001459	0.0007985
7/87	262/18670	0.000222264	0.0014701	0.0008045
4/87	64/18670	0.00022581	0.0014845	0.0008124
4/87	64/18670	0.00022581	0.0014845	0.0008124
4/87	64/18670	0.00022581	0.0014845	0.0008124
5/87	118/18670	0.000226407	0.0014854	0.0008129
4/87	65/18670	0.000239767	0.0015696	0.000859
6/87	187/18670	0.000240213	0.0015696	0.000859
7/87	266/18670	0.000243763	0.0015896	0.0008699
5/87	120/18670	0.000244824	0.0015901	0.0008702
5/87	120/18670	0.000244824	0.0015901	0.0008702
7/87	267/18670	0.000249392	0.0016166	0.0008847
4/87	66/18670	0.000254334	0.0016453	0.0009004
7/87	268/18670	0.000255125	0.0016471	0.0009014
3/87	27/18670	0.000263656	0.0016988	0.0009297
7/87	270/18670	0.00026691	0.0017164	0.0009393
4/87	67/18670	0.000269526	0.0017298	0.0009466
5/87	123/18670	0.000274559	0.0017586	0.0009624
6/87	192/18670	0.000276849	0.0017698	0.0009685
5/87	124/18670	0.000285056	0.0018115	0.0009914
5/87	124/18670	0.000285056	0.0018115	0.0009914
5/87	124/18670	0.000285056	0.0018115	0.0009914
3/87	28/18670	0.000294302	0.001863	0.0010195
3/87	28/18670	0.000294302	0.001863	0.0010195
9/87	462/18670	0.000299097	0.0018896	0.0010341
5/87	126/18670	0.000306961	0.0019355	0.0010592
7/87	279/18670	0.000325493	0.0020391	0.0011159

6/87	198/18670	0.000326462	0.0020391	0.0011159
3/87	29/18670	0.000327158	0.0020391	0.0011159
3/87	29/18670	0.000327158	0.0020391	0.0011159
3/87	29/18670	0.000327158	0.0020391	0.0011159
3/87	29/18670	0.000327158	0.0020391	0.0011159
5/87	128/18670	0.000330119	0.0020536	0.0011239
6/87	199/18670	0.000335368	0.0020822	0.0011395
7/87	283/18670	0.000354645	0.0021977	0.0012027
4/87	72/18670	0.000355421	0.0021983	0.0012031
7/87	284/18670	0.000362251	0.0022323	0.0012216
3/87	30/18670	0.000362288	0.0022323	0.0012216
6/87	202/18670	0.000363234	0.0022339	0.0012225
5/87	131/18670	0.00036731	0.0022546	0.0012339
6/87	203/18670	0.000372915	0.0022847	0.0012503
4/87	73/18670	0.000374698	0.0022913	0.001254
6/87	205/18670	0.000392883	0.0023935	0.0013099
6/87	205/18670	0.000392883	0.0023935	0.0013099
9/87	480/18670	0.00039535	0.002398	0.0013124
3/87	31/18670	0.000399757	0.002398	0.0013124
3/87	31/18670	0.000399757	0.002398	0.0013124
3/87	31/18670	0.000399757	0.002398	0.0013124
3/87	31/18670	0.000399757	0.002398	0.0013124
3/87	31/18670	0.000399757	0.002398	0.0013124
3/87	31/18670	0.000399757	0.002398	0.0013124
3/87	31/18670	0.000399757	0.002398	0.0013124
8/87	381/18670	0.000401028	0.002398	0.0013124
8/87	381/18670	0.000401028	0.002398	0.0013124
5/87	134/18670	0.000407588	0.0024328	0.0013314
6/87	207/18670	0.000413678	0.0024601	0.0013463
6/87	207/18670	0.000413678	0.0024601	0.0013463
4/87	75/18670	0.000415476	0.0024662	0.0013497
3/87	32/18670	0.000439631	0.0025859	0.0014152
3/87	32/18670	0.000439631	0.0025859	0.0014152
3/87	32/18670	0.000439631	0.0025859	0.0014152
3/87	32/18670	0.000439631	0.0025859	0.0014152
3/87	32/18670	0.000439631	0.0025859	0.0014152
6/87	210/18670	0.000446473	0.0026214	0.0014346
5/87	137/18670	0.000451116	0.0026439	0.0014469
8/87	389/18670	0.000460227	0.0026924	0.0014735
6/87	212/18670	0.000469441	0.0027414	0.0015003
6/87	213/18670	0.000481265	0.002757	0.0015088
3/87	33/18670	0.00048197	0.002757	0.0015088
3/87	33/18670	0.00048197	0.002757	0.0015088
3/87	33/18670	0.00048197	0.002757	0.0015088
3/87	33/18670	0.00048197	0.002757	0.0015088
3/87	33/18670	0.00048197	0.002757	0.0015088
3/87	33/18670	0.00048197	0.002757	0.0015088

5/87	139/18670	0.000482023	0.002757	0.0015088
5/87	139/18670	0.000482023	0.002757	0.0015088
4/87	78/18670	0.000482449	0.002757	0.0015088
4/87	78/18670	0.000482449	0.002757	0.0015088
7/87	298/18670	0.000483178	0.002757	0.0015088
7/87	298/18670	0.000483178	0.002757	0.0015088
8/87	394/18670	0.000500705	0.002852	0.0015608
6/87	215/18670	0.000505611	0.0028743	0.001573
4/87	79/18670	0.000506386	0.0028743	0.001573
5/87	141/18670	0.000514499	0.002905	0.0015898
5/87	141/18670	0.000514499	0.002905	0.0015898
5/87	141/18670	0.000514499	0.002905	0.0015898
6/87	216/18670	0.000518138	0.0029205	0.0015983
9/87	499/18670	0.000523389	0.0029439	0.0016111
3/87	34/18670	0.000526838	0.0029439	0.0016111
3/87	34/18670	0.000526838	0.0029439	0.0016111
3/87	34/18670	0.000526838	0.0029439	0.0016111
3/87	34/18670	0.000526838	0.0029439	0.0016111
6/87	217/18670	0.000530905	0.0029589	0.0016193
5/87	142/18670	0.000531341	0.0029589	0.0016193
8/87	400/18670	0.000553049	0.0030745	0.0016826
6/87	219/18670	0.000557175	0.0030868	0.0016893
6/87	219/18670	0.000557175	0.0030868	0.0016893
8/87	401/18670	0.00056219	0.0031093	0.0017016
3/87	35/18670	0.000574296	0.0031494	0.0017236
3/87	35/18670	0.000574296	0.0031494	0.0017236
3/87	35/18670	0.000574296	0.0031494	0.0017236
3/87	35/18670	0.000574296	0.0031494	0.0017236
3/87	35/18670	0.000574296	0.0031494	0.0017236
8/87	403/18670	0.000580838	0.0031799	0.0017402
5/87	145/18670	0.000584364	0.0031938	0.0017479
5/87	146/18670	0.000602891	0.0032785	0.0017942
5/87	146/18670	0.000602891	0.0032785	0.0017942
5/87	146/18670	0.000602891	0.0032785	0.0017942
8/87	406/18670	0.000609749	0.0033095	0.0018112
4/87	83/18670	0.000610639	0.0033095	0.0018112
3/87	36/18670	0.000624403	0.0033728	0.0018458
3/87	36/18670	0.000624403	0.0033728	0.0018458
4/87	84/18670	0.00063891	0.0034397	0.0018824
4/87	84/18670	0.00063891	0.0034397	0.0018824
7/87	314/18670	0.000658763	0.0035407	0.0019377
5/87	149/18670	0.000661125	0.0035475	0.0019414
3/87	37/18670	0.000677218	0.0036219	0.0019821
3/87	37/18670	0.000677218	0.0036219	0.0019821
4/87	86/18670	0.000698216	0.0037219	0.0020369
4/87	86/18670	0.000698216	0.0037219	0.0020369
6/87	229/18670	0.000704064	0.0037469	0.0020506

8/87	416/18670	0.000714651	0.003797	0.002078
7/87	319/18670	0.000722953	0.0038314	0.0020968
5/87	152/18670	0.000723477	0.0038314	0.0020968
4/87	87/18670	0.000729286	0.0038493	0.0021066
4/87	87/18670	0.000729286	0.0038493	0.0021066
3/87	38/18670	0.0007328	0.0038493	0.0021066
3/87	38/18670	0.0007328	0.0038493	0.0021066
3/87	38/18670	0.0007328	0.0038493	0.0021066
4/87	88/18670	0.00076132	0.0039862	0.0021815
4/87	88/18670	0.00076132	0.0039862	0.0021815
5/87	154/18670	0.000767423	0.0040116	0.0021954
6/87	233/18670	0.000770573	0.0040152	0.0021974
6/87	233/18670	0.000770573	0.0040152	0.0021974
6/87	234/18670	0.000787935	0.0040963	0.0022418
5/87	155/18670	0.000790129	0.0040963	0.0022418
3/87	39/18670	0.000791205	0.0040963	0.0022418
3/87	39/18670	0.000791205	0.0040963	0.0022418
4/87	89/18670	0.000794338	0.0040994	0.0022435
4/87	89/18670	0.000794338	0.0040994	0.0022435
6/87	235/18670	0.000805599	0.0041509	0.0022717
5/87	156/18670	0.000813334	0.0041841	0.0022898
7/87	328/18670	0.000850934	0.0043706	0.0023919
3/87	40/18670	0.000852489	0.0043717	0.0023925
4/87	91/18670	0.000863387	0.0044067	0.0024116
4/87	91/18670	0.000863387	0.0044067	0.0024116
4/87	91/18670	0.000863387	0.0044067	0.0024116
6/87	239/18670	0.000879337	0.004474	0.0024485
6/87	239/18670	0.000879337	0.004474	0.0024485
5/87	159/18670	0.000886006	0.0045009	0.0024632
6/87	240/18670	0.00089856	0.0045478	0.0024888
4/87	92/18670	0.000899452	0.0045478	0.0024888
4/87	92/18670	0.000899452	0.0045478	0.0024888
5/87	160/18670	0.000911273	0.0046004	0.0025176
3/87	41/18670	0.000916708	0.0046206	0.0025287
4/87	93/18670	0.000936566	0.0046367	0.0025375
4/87	93/18670	0.000936566	0.0046367	0.0025375
4/87	93/18670	0.000936566	0.0046367	0.0025375
2/87	10/18670	0.00094279	0.0046367	0.0025375
2/87	10/18670	0.00094279	0.0046367	0.0025375
2/87	10/18670	0.00094279	0.0046367	0.0025375
2/87	10/18670	0.00094279	0.0046367	0.0025375
2/87	10/18670	0.00094279	0.0046367	0.0025375
2/87	10/18670	0.00094279	0.0046367	0.0025375
2/87	10/18670	0.00094279	0.0046367	0.0025375
2/87	10/18670	0.00094279	0.0046367	0.0025375
2/87	10/18670	0.00094279	0.0046367	0.0025375
2/87	10/18670	0.00094279	0.0046367	0.0025375

2/87	10/18670	0.00094279	0.0046367	0.0025375
2/87	10/18670	0.00094279	0.0046367	0.0025375
2/87	10/18670	0.00094279	0.0046367	0.0025375
8/87	435/18670	0.000953991	0.0046847	0.0025638
6/87	243/18670	0.00095818	0.0046981	0.0025711
5/87	163/18670	0.000990301	0.004841	0.0026493
5/87	163/18670	0.000990301	0.004841	0.0026493
8/87	439/18670	0.001011759	0.0049384	0.0027026
7/87	338/18670	0.001013494	0.0049395	0.0027032
5/87	164/18670	0.001017743	0.0049527	0.0027104
6/87	247/18670	0.001042363	0.0050649	0.0027718
5/87	165/18670	0.001045746	0.0050737	0.0027767
3/87	43/18670	0.001054166	0.0050766	0.0027782
3/87	43/18670	0.001054166	0.0050766	0.0027782
3/87	43/18670	0.001054166	0.0050766	0.0027782
3/87	43/18670	0.001054166	0.0050766	0.0027782
3/87	43/18670	0.001054166	0.0050766	0.0027782
6/87	249/18670	0.001086532	0.0052235	0.0028586
8/87	444/18670	0.001087884	0.0052235	0.0028586
4/87	97/18670	0.001095846	0.0052539	0.0028753
3/87	44/18670	0.00112751	0.0053739	0.002941
3/87	44/18670	0.00112751	0.0053739	0.002941
3/87	44/18670	0.00112751	0.0053739	0.002941
3/87	44/18670	0.00112751	0.0053739	0.002941
5/87	168/18670	0.001133195	0.0053931	0.0029514
2/87	11/18670	0.001148811	0.0054434	0.002979
2/87	11/18670	0.001148811	0.0054434	0.002979
2/87	11/18670	0.001148811	0.0054434	0.002979
5/87	169/18670	0.001163516	0.005497	0.0030083
5/87	169/18670	0.001163516	0.005497	0.0030083
4/87	99/18670	0.001182214	0.0055691	0.0030478
4/87	99/18670	0.001182214	0.0055691	0.0030478
5/87	170/18670	0.001194433	0.0056104	0.0030704
5/87	170/18670	0.001194433	0.0056104	0.0030704
3/87	45/18670	0.001204001	0.0056308	0.0030816
3/87	45/18670	0.001204001	0.0056308	0.0030816
3/87	45/18670	0.001204001	0.0056308	0.0030816
8/87	452/18670	0.001219173	0.0056936	0.0031159
5/87	171/18670	0.001225956	0.0057143	0.0031272
4/87	100/18670	0.001227138	0.0057143	0.0031272
7/87	351/18670	0.001260387	0.0058607	0.0032074
4/87	101/18670	0.001273245	0.0058763	0.0032159
4/87	101/18670	0.001273245	0.0058763	0.0032159
6/87	257/18670	0.001277766	0.0058763	0.0032159
3/87	46/18670	0.001283688	0.0058763	0.0032159
3/87	46/18670	0.001283688	0.0058763	0.0032159
3/87	46/18670	0.001283688	0.0058763	0.0032159

3/87	46/18670	0.001283688	0.0058763	0.0032159
3/87	46/18670	0.001283688	0.0058763	0.0032159
3/87	46/18670	0.001283688	0.0058763	0.0032159
3/87	46/18670	0.001283688	0.0058763	0.0032159
3/87	46/18670	0.001283688	0.0058763	0.0032159
3/87	47/18670	0.001366622	0.00623	0.0034094
3/87	47/18670	0.001366622	0.00623	0.0034094
4/87	103/18670	0.001369073	0.00623	0.0034094
2/87	12/18670	0.001374401	0.00623	0.0034094
2/87	12/18670	0.001374401	0.00623	0.0034094
2/87	12/18670	0.001374401	0.00623	0.0034094
2/87	12/18670	0.001374401	0.00623	0.0034094
5/87	176/18670	0.001392894	0.006305	0.0034505
7/87	358/18670	0.001411601	0.0063808	0.003492
4/87	104/18670	0.001418826	0.0063956	0.0035001
4/87	104/18670	0.001418826	0.0063956	0.0035001
3/87	48/18670	0.001452851	0.0065398	0.003579
4/87	105/18670	0.001469829	0.0066071	0.0036158
4/87	106/18670	0.001522097	0.0068043	0.0037238
4/87	106/18670	0.001522097	0.0068043	0.0037238
4/87	106/18670	0.001522097	0.0068043	0.0037238
4/87	106/18670	0.001522097	0.0068043	0.0037238
5/87	180/18670	0.001538078	0.0068663	0.0037577
3/87	49/18670	0.001542423	0.0068762	0.0037631
6/87	267/18670	0.001551904	0.006909	0.003781
4/87	107/18670	0.001575647	0.0069954	0.0038284
4/87	107/18670	0.001575647	0.0069954	0.0038284
2/87	13/18670	0.001619379	0.007131	0.0039025
2/87	13/18670	0.001619379	0.007131	0.0039025
2/87	13/18670	0.001619379	0.007131	0.0039025
2/87	13/18670	0.001619379	0.007131	0.0039025
2/87	13/18670	0.001619379	0.007131	0.0039025
2/87	13/18670	0.001619379	0.007131	0.0039025
4/87	108/18670	0.001630495	0.0071605	0.0039187
4/87	108/18670	0.001630495	0.0071605	0.0039187
3/87	50/18670	0.001635386	0.0071625	0.0039198
3/87	50/18670	0.001635386	0.0071625	0.0039198
8/87	474/18670	0.001646519	0.0072016	0.0039412
5/87	183/18670	0.001654064	0.0072248	0.0039539
4/87	109/18670	0.001686658	0.0073573	0.0040264
6/87	272/18670	0.001704745	0.0074262	0.0040641
3/87	51/18670	0.001731785	0.0074936	0.004101
3/87	51/18670	0.001731785	0.0074936	0.004101
3/87	51/18670	0.001731785	0.0074936	0.004101
3/87	51/18670	0.001731785	0.0074936	0.004101
3/87	51/18670	0.001731785	0.0074936	0.004101
4/87	110/18670	0.001744152	0.007527	0.0041193

4/87	110/18670	0.001744152	0.007527	0.0041193
6/87	274/18670	0.001768987	0.0076241	0.0041724
4/87	111/18670	0.001802994	0.0077603	0.0042469
3/87	52/18670	0.001831666	0.007842	0.0042917
3/87	52/18670	0.001831666	0.007842	0.0042917
3/87	52/18670	0.001831666	0.007842	0.0042917
3/87	52/18670	0.001831666	0.007842	0.0042917
2/87	14/18670	0.001883562	0.0079487	0.0043501
2/87	14/18670	0.001883562	0.0079487	0.0043501
2/87	14/18670	0.001883562	0.0079487	0.0043501
2/87	14/18670	0.001883562	0.0079487	0.0043501
2/87	14/18670	0.001883562	0.0079487	0.0043501
2/87	14/18670	0.001883562	0.0079487	0.0043501
2/87	14/18670	0.001883562	0.0079487	0.0043501
2/87	14/18670	0.001883562	0.0079487	0.0043501
2/87	14/18670	0.001883562	0.0079487	0.0043501
2/87	14/18670	0.001883562	0.0079487	0.0043501
2/87	14/18670	0.001883562	0.0079487	0.0043501
8/87	485/18670	0.001900763	0.0080109	0.0043841
7/87	378/18670	0.001922907	0.0080937	0.0044294
3/87	53/18670	0.001935074	0.0081238	0.0044459
3/87	53/18670	0.001935074	0.0081238	0.0044459
8/87	488/18670	0.001975231	0.0082817	0.0045323
7/87	380/18670	0.001981037	0.0082953	0.0045397
4/87	114/18670	0.001987769	0.0083127	0.0045493
5/87	191/18670	0.001994754	0.0083312	0.0045594
5/87	192/18670	0.002040687	0.0084525	0.0046257
5/87	192/18670	0.002040687	0.0084525	0.0046257
3/87	54/18670	0.002042052	0.0084525	0.0046257
3/87	54/18670	0.002042052	0.0084525	0.0046257
3/87	54/18670	0.002042052	0.0084525	0.0046257
3/87	54/18670	0.002042052	0.0084525	0.0046257
3/87	54/18670	0.002042052	0.0084525	0.0046257
4/87	115/18670	0.002052165	0.0084835	0.0046427
5/87	193/18670	0.00208739	0.0086181	0.0047164
4/87	116/18670	0.00211799	0.0087333	0.0047794
3/87	55/18670	0.002152643	0.0087453	0.004786
3/87	55/18670	0.002152643	0.0087453	0.004786
6/87	285/18670	0.00215584	0.0087453	0.004786
2/87	15/18670	0.00216677	0.0087453	0.004786
2/87	15/18670	0.00216677	0.0087453	0.004786
2/87	15/18670	0.00216677	0.0087453	0.004786
2/87	15/18670	0.00216677	0.0087453	0.004786
2/87	15/18670	0.00216677	0.0087453	0.004786
2/87	15/18670	0.00216677	0.0087453	0.004786
2/87	15/18670	0.00216677	0.0087453	0.004786
2/87	15/18670	0.00216677	0.0087453	0.004786

2/87	15/18670	0.00216677	0.0087453	0.004786
2/87	15/18670	0.00216677	0.0087453	0.004786
2/87	15/18670	0.00216677	0.0087453	0.004786
2/87	15/18670	0.00216677	0.0087453	0.004786
2/87	15/18670	0.00216677	0.0087453	0.004786
2/87	15/18670	0.00216677	0.0087453	0.004786
4/87	117/18670	0.002185261	0.0087871	0.0048089
4/87	117/18670	0.002185261	0.0087871	0.0048089
4/87	117/18670	0.002185261	0.0087871	0.0048089
6/87	286/18670	0.002193939	0.0088111	0.004822
5/87	196/18670	0.002232188	0.0089425	0.0048939
5/87	196/18670	0.002232188	0.0089425	0.0048939
8/87	498/18670	0.00224027	0.0089638	0.0049056
3/87	56/18670	0.00226689	0.0090147	0.0049334
3/87	56/18670	0.00226689	0.0090147	0.0049334
3/87	56/18670	0.00226689	0.0090147	0.0049334
3/87	56/18670	0.00226689	0.0090147	0.0049334
3/87	56/18670	0.00226689	0.0090147	0.0049334
5/87	197/18670	0.002282043	0.0090639	0.0049603
7/87	390/18670	0.002292462	0.0090941	0.0049769
6/87	289/18670	0.002311298	0.0091576	0.0050116
5/87	198/18670	0.002332706	0.0092311	0.0050519
3/87	57/18670	0.002384833	0.0094259	0.0051585
4/87	120/18670	0.002395904	0.0094581	0.0051761
2/87	16/18670	0.002468826	0.0096403	0.0052758
2/87	16/18670	0.002468826	0.0096403	0.0052758
2/87	16/18670	0.002468826	0.0096403	0.0052758
2/87	16/18670	0.002468826	0.0096403	0.0052758
2/87	16/18670	0.002468826	0.0096403	0.0052758
2/87	16/18670	0.002468826	0.0096403	0.0052758
2/87	16/18670	0.002468826	0.0096403	0.0052758
2/87	16/18670	0.002468826	0.0096403	0.0052758
2/87	16/18670	0.002468826	0.0096403	0.0052758
3/87	58/18670	0.002506514	0.009764	0.0053435
3/87	58/18670	0.002506514	0.009764	0.0053435
5/87	202/18670	0.002543585	0.0098965	0.005416
7/87	399/18670	0.002603971	0.0101193	0.0055379
4/87	123/18670	0.002620133	0.010155	0.0055575
3/87	59/18670	0.002631971	0.010155	0.0055575
3/87	59/18670	0.002631971	0.010155	0.0055575
3/87	59/18670	0.002631971	0.010155	0.0055575
3/87	59/18670	0.002631971	0.010155	0.0055575
3/87	59/18670	0.002631971	0.010155	0.0055575
5/87	204/18670	0.002654068	0.0102281	0.0055975
4/87	124/18670	0.002697969	0.0103849	0.0056833
3/87	60/18670	0.002761245	0.0105372	0.0057666
3/87	60/18670	0.002761245	0.0105372	0.0057666

5/87	206/18670	0.002767996	0.0105372	0.0057666
4/87	125/18670	0.002777377	0.0105372	0.0057666
4/87	125/18670	0.002777377	0.0105372	0.0057666
2/87	17/18670	0.00278955	0.0105372	0.0057666
2/87	17/18670	0.00278955	0.0105372	0.0057666
2/87	17/18670	0.00278955	0.0105372	0.0057666
2/87	17/18670	0.00278955	0.0105372	0.0057666
2/87	17/18670	0.00278955	0.0105372	0.0057666
2/87	17/18670	0.00278955	0.0105372	0.0057666
2/87	17/18670	0.00278955	0.0105372	0.0057666
2/87	17/18670	0.00278955	0.0105372	0.0057666
2/87	17/18670	0.00278955	0.0105372	0.0057666
2/87	17/18670	0.00278955	0.0105372	0.0057666
2/87	17/18670	0.00278955	0.0105372	0.0057666
4/87	127/18670	0.002940976	0.0110963	0.0060726
7/87	408/18670	0.002947164	0.0111067	0.0060783
7/87	410/18670	0.003027939	0.0113844	0.0062303
3/87	62/18670	0.003031392	0.0113844	0.0062303
3/87	62/18670	0.003031392	0.0113844	0.0062303
4/87	129/18670	0.003111054	0.0116556	0.0063787
2/87	18/18670	0.003128766	0.0116556	0.0063787
2/87	18/18670	0.003128766	0.0116556	0.0063787
2/87	18/18670	0.003128766	0.0116556	0.0063787
2/87	18/18670	0.003128766	0.0116556	0.0063787
2/87	18/18670	0.003128766	0.0116556	0.0063787
2/87	18/18670	0.003128766	0.0116556	0.0063787
3/87	63/18670	0.003172339	0.0118043	0.0064601
7/87	414/18670	0.003194581	0.0118734	0.0064979
4/87	130/18670	0.003198561	0.0118746	0.0064986
4/87	131/18670	0.003287734	0.0121917	0.0066721
3/87	64/18670	0.00331725	0.0122871	0.0067243
4/87	132/18670	0.003378589	0.0124858	0.006833
4/87	132/18670	0.003378589	0.0124858	0.006833
7/87	419/18670	0.003412671	0.0125973	0.0068941
5/87	217/18670	0.003458937	0.0127099	0.0069557
3/87	65/18670	0.00346616	0.0127099	0.0069557
4/87	133/18670	0.00347114	0.0127099	0.0069557
2/87	19/18670	0.0034863	0.0127099	0.0069557
2/87	19/18670	0.0034863	0.0127099	0.0069557
2/87	19/18670	0.0034863	0.0127099	0.0069557
2/87	19/18670	0.0034863	0.0127099	0.0069557
2/87	19/18670	0.0034863	0.0127099	0.0069557
2/87	19/18670	0.0034863	0.0127099	0.0069557
2/87	19/18670	0.0034863	0.0127099	0.0069557
2/87	19/18670	0.0034863	0.0127099	0.0069557
5/87	218/18670	0.003527364	0.0128307	0.0070218
5/87	218/18670	0.003527364	0.0128307	0.0070218

6/87	315/18670	0.003537179	0.012852	0.0070335
4/87	134/18670	0.003565403	0.0129256	0.0070737
4/87	134/18670	0.003565403	0.0129256	0.0070737
6/87	316/18670	0.003592404	0.0130089	0.0071193
3/87	66/18670	0.003619105	0.013091	0.0071642
7/87	424/18670	0.003641977	0.013159	0.0072015
4/87	135/18670	0.003661393	0.0132144	0.0072318
7/87	425/18670	0.003689217	0.0133001	0.0072786
4/87	136/18670	0.003759125	0.0135321	0.0074057
6/87	319/18670	0.00376194	0.0135321	0.0074057
5/87	222/18670	0.003810845	0.0136928	0.0074936
4/87	137/18670	0.003858614	0.0137244	0.0075109
2/87	20/18670	0.003861977	0.0137244	0.0075109
2/87	20/18670	0.003861977	0.0137244	0.0075109
2/87	20/18670	0.003861977	0.0137244	0.0075109
2/87	20/18670	0.003861977	0.0137244	0.0075109
2/87	20/18670	0.003861977	0.0137244	0.0075109
2/87	20/18670	0.003861977	0.0137244	0.0075109
2/87	20/18670	0.003861977	0.0137244	0.0075109
2/87	20/18670	0.003861977	0.0137244	0.0075109
2/87	20/18670	0.003861977	0.0137244	0.0075109
6/87	321/18670	0.003878225	0.0137671	0.0075342
7/87	430/18670	0.00393247	0.0139307	0.0076238
3/87	68/18670	0.003937228	0.0139307	0.0076238
3/87	68/18670	0.003937228	0.0139307	0.0076238
5/87	224/18670	0.003958554	0.0139909	0.0076567
7/87	432/18670	0.004033119	0.0142108	0.0077771
5/87	225/18670	0.004033928	0.0142108	0.0077771
5/87	225/18670	0.004033928	0.0142108	0.0077771
4/87	139/18670	0.004062923	0.014251	0.0077991
4/87	139/18670	0.004062923	0.014251	0.0077991
4/87	139/18670	0.004062923	0.014251	0.0077991
4/87	139/18670	0.004062923	0.014251	0.0077991
4/87	140/18670	0.004167771	0.014603	0.0079917
5/87	227/18670	0.004187754	0.0146571	0.0080213
6/87	327/18670	0.004243139	0.0147988	0.0080989
2/87	21/18670	0.004255624	0.0147988	0.0080989
2/87	21/18670	0.004255624	0.0147988	0.0080989
2/87	21/18670	0.004255624	0.0147988	0.0080989
2/87	21/18670	0.004255624	0.0147988	0.0080989
2/87	21/18670	0.004255624	0.0147988	0.0080989
3/87	70/18670	0.004271881	0.0148235	0.0081124
3/87	70/18670	0.004271881	0.0148235	0.0081124
4/87	142/18670	0.004382932	0.0151926	0.0083144
3/87	71/18670	0.004445485	0.0153438	0.0083971
3/87	71/18670	0.004445485	0.0153438	0.0083971
3/87	71/18670	0.004445485	0.0153438	0.0083971

3/87	71/18670	0.004445485	0.0153438	0.0083971
4/87	144/18670	0.004605473	0.0158622	0.0086808
4/87	144/18670	0.004605473	0.0158622	0.0086808
3/87	72/18670	0.004623313	0.015872	0.0086862
3/87	72/18670	0.004623313	0.015872	0.0086862
2/87	22/18670	0.004667069	0.015872	0.0086862
2/87	22/18670	0.004667069	0.015872	0.0086862
2/87	22/18670	0.004667069	0.015872	0.0086862
2/87	22/18670	0.004667069	0.015872	0.0086862
2/87	22/18670	0.004667069	0.015872	0.0086862
2/87	22/18670	0.004667069	0.015872	0.0086862
2/87	22/18670	0.004667069	0.015872	0.0086862
2/87	22/18670	0.004667069	0.015872	0.0086862
2/87	22/18670	0.004667069	0.015872	0.0086862
2/87	22/18670	0.004667069	0.015872	0.0086862
5/87	233/18670	0.004674395	0.0158802	0.0086907
4/87	145/18670	0.004719547	0.0160168	0.0087654
7/87	445/18670	0.004735927	0.0160556	0.0087867
3/87	73/18670	0.004805397	0.0162571	0.0088969
3/87	73/18670	0.004805397	0.0162571	0.0088969
4/87	146/18670	0.004835509	0.0163419	0.0089433
4/87	147/18670	0.004953373	0.0167228	0.0091518
3/87	74/18670	0.004991764	0.0168174	0.0092035
3/87	74/18670	0.004991764	0.0168174	0.0092035
5/87	237/18670	0.005020405	0.0168613	0.0092276
5/87	237/18670	0.005020405	0.0168613	0.0092276
5/87	237/18670	0.005020405	0.0168613	0.0092276
2/87	23/18670	0.005096142	0.0170802	0.0093474
2/87	23/18670	0.005096142	0.0170802	0.0093474
5/87	238/18670	0.005109675	0.0171079	0.0093626
3/87	75/18670	0.005182443	0.0173336	0.0094861
5/87	239/18670	0.005200067	0.0173747	0.0095085
6/87	343/18670	0.005340453	0.0178253	0.0097552
6/87	344/18670	0.005415334	0.0180567	0.0098818
2/87	24/18670	0.005542674	0.0183117	0.0100214
2/87	24/18670	0.005542674	0.0183117	0.0100214
2/87	24/18670	0.005542674	0.0183117	0.0100214
2/87	24/18670	0.005542674	0.0183117	0.0100214
2/87	24/18670	0.005542674	0.0183117	0.0100214
2/87	24/18670	0.005542674	0.0183117	0.0100214
2/87	24/18670	0.005542674	0.0183117	0.0100214
2/87	24/18670	0.005542674	0.0183117	0.0100214
2/87	24/18670	0.005542674	0.0183117	0.0100214
4/87	152/18670	0.005571719	0.0183312	0.010032
3/87	77/18670	0.005576844	0.0183312	0.010032
3/87	77/18670	0.005576844	0.0183312	0.010032
3/87	77/18670	0.005576844	0.0183312	0.010032

3/87	77/18670	0.005576844	0.0183312	0.010032
5/87	244/18670	0.00566912	0.0186156	0.0101877
3/87	78/18670	0.00578062	0.0188861	0.0103357
3/87	78/18670	0.00578062	0.0188861	0.0103357
3/87	78/18670	0.00578062	0.0188861	0.0103357
3/87	78/18670	0.00578062	0.0188861	0.0103357
3/87	78/18670	0.00578062	0.0188861	0.0103357
7/87	462/18670	0.005790343	0.0188988	0.0103426
7/87	463/18670	0.005857431	0.0190985	0.0104519
7/87	464/18670	0.0059251	0.0192997	0.0105621
3/87	79/18670	0.005988813	0.0194088	0.0106218
3/87	79/18670	0.005988813	0.0194088	0.0106218
2/87	25/18670	0.006006496	0.0194088	0.0106218
2/87	25/18670	0.006006496	0.0194088	0.0106218
2/87	25/18670	0.006006496	0.0194088	0.0106218
2/87	25/18670	0.006006496	0.0194088	0.0106218
2/87	25/18670	0.006006496	0.0194088	0.0106218
2/87	25/18670	0.006006496	0.0194088	0.0106218
6/87	352/18670	0.006042432	0.0195055	0.0106747
5/87	248/18670	0.006065324	0.0195599	0.0107044
3/87	80/18670	0.006201449	0.0199776	0.010933
6/87	354/18670	0.006207168	0.0199776	0.010933
4/87	157/18670	0.006239697	0.0200624	0.0109794
5/87	250/18670	0.006270577	0.0201217	0.0110119
5/87	250/18670	0.006270577	0.0201217	0.0110119
6/87	355/18670	0.006290756	0.0201466	0.0110255
6/87	355/18670	0.006290756	0.0201466	0.0110255
5/87	251/18670	0.006375017	0.0203766	0.0111514
6/87	356/18670	0.006375163	0.0203766	0.0111514
3/87	81/18670	0.006418552	0.0204548	0.0111942
3/87	81/18670	0.006418552	0.0204548	0.0111942
3/87	81/18670	0.006418552	0.0204548	0.0111942
2/87	26/18670	0.006487441	0.0205531	0.011248
2/87	26/18670	0.006487441	0.0205531	0.011248
2/87	26/18670	0.006487441	0.0205531	0.011248
2/87	26/18670	0.006487441	0.0205531	0.011248
2/87	26/18670	0.006487441	0.0205531	0.011248
2/87	26/18670	0.006487441	0.0205531	0.011248
3/87	82/18670	0.006640145	0.0209754	0.0114791
3/87	82/18670	0.006640145	0.0209754	0.0114791
3/87	82/18670	0.006640145	0.0209754	0.0114791
6/87	361/18670	0.006809645	0.0214313	0.0117286
6/87	361/18670	0.006809645	0.0214313	0.0117286
4/87	161/18670	0.00681094	0.0214313	0.0117286
4/87	161/18670	0.00681094	0.0214313	0.0117286
7/87	477/18670	0.006859196	0.0215426	0.0117895
3/87	83/18670	0.006866252	0.0215426	0.0117895

3/87	83/18670	0.006866252	0.0215426	0.0117895
2/87	27/18670	0.006985344	0.0217394	0.0118972
2/87	27/18670	0.006985344	0.0217394	0.0118972
2/87	27/18670	0.006985344	0.0217394	0.0118972
2/87	27/18670	0.006985344	0.0217394	0.0118972
2/87	27/18670	0.006985344	0.0217394	0.0118972
2/87	27/18670	0.006985344	0.0217394	0.0118972
2/87	27/18670	0.006985344	0.0217394	0.0118972
2/87	27/18670	0.006985344	0.0217394	0.0118972
6/87	363/18670	0.006989332	0.0217394	0.0118972
7/87	479/18670	0.007012118	0.0217893	0.0119245
3/87	84/18670	0.007096894	0.0219895	0.0120341
3/87	84/18670	0.007096894	0.0219895	0.0120341
3/87	84/18670	0.007096894	0.0219895	0.0120341
4/87	163/18670	0.007109134	0.0220064	0.0120433
3/87	85/18670	0.007332094	0.0226749	0.0124092
5/87	260/18670	0.007370702	0.0227509	0.0124508
5/87	260/18670	0.007370702	0.0227509	0.0124508
7/87	484/18670	0.007405536	0.0228367	0.0124977
7/87	485/18670	0.007486149	0.0229101	0.0125379
2/87	28/18670	0.00750004	0.0229101	0.0125379
2/87	28/18670	0.00750004	0.0229101	0.0125379
2/87	28/18670	0.00750004	0.0229101	0.0125379
2/87	28/18670	0.00750004	0.0229101	0.0125379
2/87	28/18670	0.00750004	0.0229101	0.0125379
2/87	28/18670	0.00750004	0.0229101	0.0125379
2/87	28/18670	0.00750004	0.0229101	0.0125379
2/87	28/18670	0.00750004	0.0229101	0.0125379
2/87	28/18670	0.00750004	0.0229101	0.0125379
3/87	86/18670	0.007571872	0.023066	0.0126232
3/87	86/18670	0.007571872	0.023066	0.0126232
4/87	166/18670	0.007572426	0.023066	0.0126232
5/87	262/18670	0.007605905	0.0231462	0.0126671
6/87	370/18670	0.007645434	0.0232447	0.012721
3/87	87/18670	0.007816251	0.0237195	0.0129809
3/87	87/18670	0.007816251	0.0237195	0.0129809
4/87	168/18670	0.0078921	0.0239273	0.0130946
2/87	29/18670	0.008031364	0.0242256	0.0132578
2/87	29/18670	0.008031364	0.0242256	0.0132578
2/87	29/18670	0.008031364	0.0242256	0.0132578
2/87	29/18670	0.008031364	0.0242256	0.0132578
4/87	169/18670	0.008055217	0.0242256	0.0132578
3/87	88/18670	0.008065248	0.0242256	0.0132578
3/87	88/18670	0.008065248	0.0242256	0.0132578
3/87	88/18670	0.008065248	0.0242256	0.0132578
3/87	88/18670	0.008065248	0.0242256	0.0132578
3/87	88/18670	0.008065248	0.0242256	0.0132578

5/87	266/18670	0.008091949	0.0242833	0.0132894
3/87	89/18670	0.008318884	0.0248722	0.0136117
3/87	89/18670	0.008318884	0.0248722	0.0136117
3/87	89/18670	0.008318884	0.0248722	0.0136117
3/87	89/18670	0.008318884	0.0248722	0.0136117
4/87	171/18670	0.008388072	0.025056	0.0137122
3/87	90/18670	0.008577179	0.0253927	0.0138965
3/87	90/18670	0.008577179	0.0253927	0.0138965
2/87	30/18670	0.008579155	0.0253927	0.0138965
2/87	30/18670	0.008579155	0.0253927	0.0138965
2/87	30/18670	0.008579155	0.0253927	0.0138965
2/87	30/18670	0.008579155	0.0253927	0.0138965
2/87	30/18670	0.008579155	0.0253927	0.0138965
2/87	30/18670	0.008579155	0.0253927	0.0138965
2/87	30/18670	0.008579155	0.0253927	0.0138965
2/87	30/18670	0.008579155	0.0253927	0.0138965
7/87	499/18670	0.008684485	0.025681	0.0140543
4/87	173/18670	0.008729833	0.0257916	0.0141148
3/87	91/18670	0.008840149	0.0260937	0.0142802
5/87	273/18670	0.00899387	0.0264992	0.0145021
5/87	273/18670	0.00899387	0.0264992	0.0145021
4/87	175/18670	0.009080594	0.0265769	0.0145446
4/87	175/18670	0.009080594	0.0265769	0.0145446
3/87	92/18670	0.009107813	0.0265769	0.0145446
5/87	274/18670	0.009128161	0.0265769	0.0145446
2/87	31/18670	0.009143252	0.0265769	0.0145446
2/87	31/18670	0.009143252	0.0265769	0.0145446
2/87	31/18670	0.009143252	0.0265769	0.0145446
2/87	31/18670	0.009143252	0.0265769	0.0145446
2/87	31/18670	0.009143252	0.0265769	0.0145446
2/87	31/18670	0.009143252	0.0265769	0.0145446
2/87	31/18670	0.009143252	0.0265769	0.0145446
2/87	31/18670	0.009143252	0.0265769	0.0145446
2/87	31/18670	0.009143252	0.0265769	0.0145446
2/87	31/18670	0.009143252	0.0265769	0.0145446
2/87	31/18670	0.009143252	0.0265769	0.0145446
3/87	93/18670	0.009380188	0.0272168	0.0148948
3/87	93/18670	0.009380188	0.0272168	0.0148948
4/87	177/18670	0.009440445	0.0273183	0.0149503
4/87	177/18670	0.009440445	0.0273183	0.0149503
4/87	177/18670	0.009440445	0.0273183	0.0149503
2/87	32/18670	0.009723493	0.0280123	0.0153301
2/87	32/18670	0.009723493	0.0280123	0.0153301
2/87	32/18670	0.009723493	0.0280123	0.0153301
2/87	32/18670	0.009723493	0.0280123	0.0153301
2/87	32/18670	0.009723493	0.0280123	0.0153301
4/87	179/18670	0.009809477	0.0282099	0.0154383

4/87	179/18670	0.009809477	0.0282099	0.0154383
5/87	279/18670	0.009820485	0.0282165	0.0154419
3/87	95/18670	0.009939136	0.0285069	0.0156008
3/87	95/18670	0.009939136	0.0285069	0.0156008
4/87	181/18670	0.010187778	0.0291942	0.0159769
3/87	96/18670	0.010225742	0.0292107	0.015986
3/87	96/18670	0.010225742	0.0292107	0.015986
3/87	96/18670	0.010225742	0.0292107	0.015986
5/87	282/18670	0.010252815	0.0292107	0.015986
2/87	33/18670	0.010319721	0.0292107	0.015986
2/87	33/18670	0.010319721	0.0292107	0.015986
2/87	33/18670	0.010319721	0.0292107	0.015986
2/87	33/18670	0.010319721	0.0292107	0.015986
2/87	33/18670	0.010319721	0.0292107	0.015986
2/87	33/18670	0.010319721	0.0292107	0.015986
2/87	33/18670	0.010319721	0.0292107	0.015986
2/87	33/18670	0.010319721	0.0292107	0.015986
2/87	33/18670	0.010319721	0.0292107	0.015986
2/87	33/18670	0.010319721	0.0292107	0.015986
4/87	182/18670	0.010380431	0.0293569	0.016066
3/87	97/18670	0.010517121	0.0296658	0.016235
3/87	97/18670	0.010517121	0.0296658	0.016235
3/87	97/18670	0.010517121	0.0296658	0.016235
4/87	183/18670	0.010575434	0.0298043	0.0163109
3/87	98/18670	0.01081329	0.0304482	0.0166632
2/87	34/18670	0.010931776	0.0307551	0.0168312
6/87	400/18670	0.010968989	0.0308163	0.0168646
4/87	185/18670	0.010972529	0.0308163	0.0168646
5/87	287/18670	0.011002165	0.0308727	0.0168956
2/87	35/18670	0.011559502	0.0323247	0.0176902
2/87	35/18670	0.011559502	0.0323247	0.0176902
2/87	35/18670	0.011559502	0.0323247	0.0176902
2/87	35/18670	0.011559502	0.0323247	0.0176902
5/87	291/18670	0.011628036	0.0324883	0.0177797
3/87	101/18670	0.011730665	0.0327187	0.0179058
3/87	101/18670	0.011730665	0.0327187	0.0179058
6/87	408/18670	0.012005504	0.0334322	0.0182962
4/87	190/18670	0.012007107	0.0334322	0.0182962
3/87	102/18670	0.012046123	0.0334833	0.0183242
3/87	102/18670	0.012046123	0.0334833	0.0183242
6/87	409/18670	0.01213977	0.0335955	0.0183857
2/87	36/18670	0.012202743	0.0335955	0.0183857
2/87	36/18670	0.012202743	0.0335955	0.0183857
2/87	36/18670	0.012202743	0.0335955	0.0183857
2/87	36/18670	0.012202743	0.0335955	0.0183857
2/87	36/18670	0.012202743	0.0335955	0.0183857
2/87	36/18670	0.012202743	0.0335955	0.0183857

2/87	36/18670	0.012202743	0.0335955	0.0183857
2/87	36/18670	0.012202743	0.0335955	0.0183857
2/87	36/18670	0.012202743	0.0335955	0.0183857
2/87	36/18670	0.012202743	0.0335955	0.0183857
4/87	191/18670	0.012221275	0.0335955	0.0183857
4/87	191/18670	0.012221275	0.0335955	0.0183857
5/87	295/18670	0.012277807	0.0336938	0.0184394
5/87	295/18670	0.012277807	0.0336938	0.0184394
3/87	103/18670	0.012366434	0.0338796	0.0185411
3/87	103/18670	0.012366434	0.0338796	0.0185411
4/87	192/18670	0.012437883	0.0340466	0.0186325
3/87	104/18670	0.012691611	0.0347118	0.0189965
6/87	414/18670	0.012827062	0.0349402	0.0191215
2/87	37/18670	0.012861344	0.0349402	0.0191215
2/87	37/18670	0.012861344	0.0349402	0.0191215
2/87	37/18670	0.012861344	0.0349402	0.0191215
2/87	37/18670	0.012861344	0.0349402	0.0191215
2/87	37/18670	0.012861344	0.0349402	0.0191215
2/87	37/18670	0.012861344	0.0349402	0.0191215
2/87	37/18670	0.012861344	0.0349402	0.0191215
3/87	105/18670	0.013021663	0.0352575	0.0192952
3/87	105/18670	0.013021663	0.0352575	0.0192952
3/87	105/18670	0.013021663	0.0352575	0.0192952
3/87	105/18670	0.013021663	0.0352575	0.0192952
3/87	106/18670	0.013356602	0.036074	0.019742
3/87	106/18670	0.013356602	0.036074	0.019742
3/87	106/18670	0.013356602	0.036074	0.019742
2/87	38/18670	0.013535152	0.0363141	0.0198734
2/87	38/18670	0.013535152	0.0363141	0.0198734
2/87	38/18670	0.013535152	0.0363141	0.0198734
2/87	38/18670	0.013535152	0.0363141	0.0198734
2/87	38/18670	0.013535152	0.0363141	0.0198734
2/87	38/18670	0.013535152	0.0363141	0.0198734
2/87	38/18670	0.013535152	0.0363141	0.0198734
2/87	38/18670	0.013535152	0.0363141	0.0198734
4/87	197/18670	0.013557859	0.0363449	0.0198903
3/87	107/18670	0.013696437	0.0366255	0.0200439
3/87	107/18670	0.013696437	0.0366255	0.0200439
3/87	107/18670	0.013696437	0.0366255	0.0200439
4/87	199/18670	0.014023257	0.0374239	0.0204808
4/87	199/18670	0.014023257	0.0374239	0.0204808
3/87	108/18670	0.014041178	0.0374239	0.0204808
3/87	108/18670	0.014041178	0.0374239	0.0204808
2/87	39/18670	0.014224014	0.0377869	0.0206794
2/87	39/18670	0.014224014	0.0377869	0.0206794
2/87	39/18670	0.014224014	0.0377869	0.0206794
2/87	39/18670	0.014224014	0.0377869	0.0206794

3/87	109/18670	0.014390834	0.0381675	0.0208877
3/87	109/18670	0.014390834	0.0381675	0.0208877
3/87	110/18670	0.014745414	0.039076	0.0213849
2/87	40/18670	0.014927779	0.0392066	0.0214564
2/87	40/18670	0.014927779	0.0392066	0.0214564
2/87	40/18670	0.014927779	0.0392066	0.0214564
2/87	40/18670	0.014927779	0.0392066	0.0214564
2/87	40/18670	0.014927779	0.0392066	0.0214564
2/87	40/18670	0.014927779	0.0392066	0.0214564
2/87	40/18670	0.014927779	0.0392066	0.0214564
2/87	40/18670	0.014927779	0.0392066	0.0214564
2/87	40/18670	0.014927779	0.0392066	0.0214564
2/87	40/18670	0.014927779	0.0392066	0.0214564
2/87	40/18670	0.014927779	0.0392066	0.0214564
3/87	111/18670	0.015104926	0.0396077	0.0216759
3/87	111/18670	0.015104926	0.0396077	0.0216759
4/87	204/18670	0.015230887	0.0398734	0.0218213
4/87	204/18670	0.015230887	0.0398734	0.0218213
3/87	112/18670	0.015469377	0.0403303	0.0220713
3/87	112/18670	0.015469377	0.0403303	0.0220713
3/87	112/18670	0.015469377	0.0403303	0.0220713
3/87	112/18670	0.015469377	0.0403303	0.0220713
3/87	112/18670	0.015469377	0.0403303	0.0220713
4/87	205/18670	0.015480047	0.0403303	0.0220713
2/87	41/18670	0.015646295	0.0406327	0.0222368
2/87	41/18670	0.015646295	0.0406327	0.0222368
2/87	41/18670	0.015646295	0.0406327	0.0222368
2/87	41/18670	0.015646295	0.0406327	0.0222368
5/87	314/18670	0.015702678	0.0407465	0.0222991
3/87	113/18670	0.015838775	0.0409685	0.0224206
3/87	113/18670	0.015838775	0.0409685	0.0224206
3/87	113/18670	0.015838775	0.0409685	0.0224206
3/87	113/18670	0.015838775	0.0409685	0.0224206
4/87	207/18670	0.015986066	0.0413165	0.022611
3/87	114/18670	0.016213127	0.0418366	0.0228957
3/87	114/18670	0.016213127	0.0418366	0.0228957
4/87	208/18670	0.016242941	0.0418802	0.0229195
2/87	42/18670	0.016379414	0.0421315	0.0230571
2/87	42/18670	0.016379414	0.0421315	0.0230571
2/87	42/18670	0.016379414	0.0421315	0.0230571
4/87	209/18670	0.016502404	0.0424142	0.0232118
3/87	115/18670	0.016592438	0.0425781	0.0233015
3/87	115/18670	0.016592438	0.0425781	0.0233015
3/87	116/18670	0.016976715	0.0434953	0.0238034
3/87	116/18670	0.016976715	0.0434953	0.0238034
2/87	43/18670	0.017126987	0.0436731	0.0239008
2/87	43/18670	0.017126987	0.0436731	0.0239008

2/87	43/18670	0.017126987	0.0436731	0.0239008
2/87	43/18670	0.017126987	0.0436731	0.0239008
2/87	43/18670	0.017126987	0.0436731	0.0239008
2/87	43/18670	0.017126987	0.0436731	0.0239008
4/87	212/18670	0.017296392	0.0440704	0.0241182
3/87	117/18670	0.017365964	0.0441782	0.0241772
3/87	117/18670	0.017365964	0.0441782	0.0241772
4/87	213/18670	0.01756628	0.0446528	0.0244369
4/87	214/18670	0.017838792	0.0451893	0.0247305
4/87	214/18670	0.017838792	0.0451893	0.0247305
2/87	44/18670	0.017888867	0.0451893	0.0247305
2/87	44/18670	0.017888867	0.0451893	0.0247305
2/87	44/18670	0.017888867	0.0451893	0.0247305
2/87	44/18670	0.017888867	0.0451893	0.0247305
2/87	44/18670	0.017888867	0.0451893	0.0247305
2/87	44/18670	0.017888867	0.0451893	0.0247305
6/87	448/18670	0.018242289	0.0460462	0.0251994
3/87	120/18670	0.018563588	0.0468207	0.0256233
2/87	45/18670	0.018664907	0.0469848	0.0257131
4/87	217/18670	0.018672146	0.0469848	0.0257131
4/87	217/18670	0.018672146	0.0469848	0.0257131
3/87	121/18670	0.01897277	0.0476672	0.0260866
3/87	121/18670	0.01897277	0.0476672	0.0260866
3/87	122/18670	0.019386944	0.0484654	0.0265234
3/87	122/18670	0.019386944	0.0484654	0.0265234
2/87	46/18670	0.019454963	0.0484654	0.0265234
2/87	46/18670	0.019454963	0.0484654	0.0265234
2/87	46/18670	0.019454963	0.0484654	0.0265234
2/87	46/18670	0.019454963	0.0484654	0.0265234
2/87	46/18670	0.019454963	0.0484654	0.0265234
2/87	46/18670	0.019454963	0.0484654	0.0265234
2/87	46/18670	0.019454963	0.0484654	0.0265234
2/87	46/18670	0.019454963	0.0484654	0.0265234
2/87	46/18670	0.019454963	0.0484654	0.0265234
5/87	332/18670	0.019489225	0.0485135	0.0265497
4/87	220/18670	0.019529369	0.0485761	0.026584
5/87	334/18670	0.01994389	0.0495691	0.0271274

geneID
MAPKAPK2/AKT2/EGFR/CDK2/MAPK9/MAPK14/PIK3CA/SYK/AKT1/APP/PRKDC/MAPK1/IKBKB/MARK
MAPKAPK2/AKT2/EGFR/CDK2/MAPK9/MAPK14/PIK3CA/SYK/AKT1/APP/PRKDC/MAPK1/IKBKB/MARK
NTRK1/MAPKAPK2/PIK3CB/MAPK10/JAK2/EGFR/MAPK11/MAPK14/PIK3CA/SYK/AKT1/PSEN1/MAPK1
NTRK1/MAPKAPK2/PIK3CB/MAPK10/JAK2/EGFR/PDE5A/PPP2CA/MAPK11/MAPK14/PIK3CG/SYK/AKT
HCRTR2/PIK3CB/TACR1/PTGER1/HCRTR1/JAK2/TRPV1/CRHR1/VDR/PIK3CG/S1PR1/PSEN1/APP/CACN
HCRTR2/PIK3CB/TACR1/PTGER1/HCRTR1/JAK2/TRPV1/CRHR1/VDR/PIK3CG/S1PR1/PSEN1/APP/CACN
HCRTR2/PIK3CB/TACR1/PTGER1/HCRTR1/JAK2/TRPV1/CRHR1/VDR/PIK3CG/S1PR1/PSEN1/APP/CACN
NTRK1/CHRM1/PTGER1/NCSTN/CCNA2/TRPV1/HTR1A/MDM2/EGFR/CDK2/MAPK1/PDE4B/REN/P2RX7
CHRM1/JAK2/ITGB2/MDM2/AKT2/EGFR/MAPK11/MAPK14/SYK/PSEN1/CSF1R/SMO/MAPK1/MAPK8/P2
NCSTN/APH1A/PSEN1/PSEN2/APH1B/PSENEN
CHRM1/JAK2/ITGB2/MDM2/AKT2/EGFR/MAPK11/MAPK14/SYK/PSEN1/CSF1R/SMO/MAPK1/MAPK8/P2
CHRM1/NCSTN/AKT2/EGFR/IL6ST/AKT1/PSEN1/CSF1R/APP/SMO/F2/MAPK1/ADORA2A/PPP1CC/MTOR
NTRK1/HCRTR2/MAPK10/EGFR/MAPK9/MTNR1A/MTNR1B/PRKDC/MAPK8/ADORA2A/PPP1CC/MTOR/
NTRK1/MAPKAPK2/PIK3CB/MAPK10/JAK2/EGFR/PDE5A/MAPK11/MAPK14/PIK3CG/SYK/PSEN1/MAPK
NTRK1/MAPK10/CRHR1/MDM2/AKT2/EGFR/CHEK1/MAPK14/AKT1/APP/PRKDC/MAPK8/PLK3/PPP1CC
CHRM1/ITGB2/MDM2/AKT2/EGFR/MAPK14/AKT1/PSEN1/SMO/F2/MAPK1/MAPK8/PLK3/PLK1/PDPK1
NTRK1/MAPKAPK2/PIK3CB/MAPK10/JAK2/EGFR/PDE5A/PPP2CA/MAPK11/MAPK14/PIK3CG/SYK/PSEN
JAK2/ITGB2/TRPV1/EGFR/MAPK14/SYK/AKT1/F2/MTOR/ICAM1
NTRK1/PIK3CB/JAK2/EGFR/PIK3CG/PIK3CA/AKT1/CSF1R/PTK2/F2/PIK3CD/MAPK1
NTRK1/PIK3CB/JAK2/EGFR/PIK3CG/PIK3CA/AKT1/CSF1R/PTK2/F2/PIK3CD/MAPK1
NTRK1/MAPKAPK2/JAK2/EGFR/TNK2/SYK/AKT1/CSF1R/PTK2/MARK2/PIM1/MTOR/PDPK1
CRHR1/AKT2/ABCB1/PIK3CG/AKT1/PSEN1/APP/CACNA2D1/MTNR1B/F2/PSEN2/PDE4B/P2RX7/KCNH2/
NTRK1/HCRTR2/MAPK10/EGFR/MAPK9/MTNR1A/NPY5R/MTNR1B/PRKDC/MAPK8/ADORA2A/PPP1CC
NTRK1/PIK3CB/JAK2/EGFR/PIK3CG/PIK3CA/AKT1/PTK2/F2/PIK3CD/MAPK1
JAK2/TRPV1/MDM2/EGFR/PDE5A/PIK3CG/AKT1/CACNA2D1/PDE4B/KCNH2/DRD2/AVPR2/ICAM1/CHR
HCRTR2/TACR1/PTGER1/HCRTR1/JAK2/TRPV1/CRHR1/PIK3CG/S1PR1/CACNA2D1/MTNR1B/F2/P2RX7
NTRK1/JAK2/ITGB2/EGFR/PPP2CA/TNK2/SYK/IL6ST/JAK1/PSEN1/CSF1R/APP/PTK2/MTOR/ICAM1
HCRTR2/MAPK10/MAPK9/MTNR1B/PRKDC/MAPK8/ADORA2A/PPP1CC/MTOR/DRD2
NTRK1/JAK2/ITGB2/EGFR/PPP2CA/TNK2/SYK/IL6ST/JAK1/PSEN1/CSF1R/APP/PTK2/MTOR/ICAM1
NTRK1/MAPK10/JAK2/ITGB2/MAPK11/MAPK9/MAPK14/SYK/AKT1/APP/SMO/MAPK1/IKBKB/MAPK8/P
NCSTN/APH1A/PSEN1/PSEN2/APH1B/PSENEN
JAK2/ITGB2/TRPV1/EGFR/PSEN1/APP/SMO/ADORA2A
JAK2/CCNA2/CRHR1/MDM2/AKT2/PIK3CA/AKT1/PSEN1/APP/CACNA2D1/PTK2/PRKDC/PDE3B/PDPK1/
NTRK1/PIK3CB/JAK2/EGFR/PIK3CG/PIK3CA/PTK2/F2/PIK3CD/MAPK1
CHEK1/AKT1/APP/MAPK1/MARK2/MAPK8/PLK1/MTOR/PDPK1/CDC42BPA
NCSTN/PDE5A/ITGAL/PIK3CG/PIK3CA/SYK/IL6ST/AKT1/PSEN1/PRKDC/PIK3CD/P2RX7/ADORA2A/MT
NCSTN/AKT2/EGFR/IL6ST/AKT1/PSEN1/APP/SMO/F2/MAPK1/ADORA2A/MTOR
AKT2/ABCB1/NPY5R/SYK/AKT1/PSEN1/P2RX7/ADORA2A/MTOR
CHEK1/AKT1/APP/MAPK1/MARK2/MAPK8/PLK1/MTOR/PDPK1/CDC42BPA
CHRM1/ITGB2/CRHR1/MDM2/AKT2/MAPK14/SYK/PSEN1/SMO/MAPK1/MAPK8/PLK3
NTRK1/JAK2/NCSTN/ITGB2/CRHR1/AKT2/PIK3CA/AKT1/PSEN1/APP/MTNR1B/ADORA2A/MTOR/PDPK
CYP2D6/CCNA2/CRHR1/MDM2/EGFR/CNR2/CYP3A4/CYP2C19/CYP2C9/ADORA2A/MTOR/DRD2/ICAM1
HCRTR2/HCRTR1/CRHR1/NPY5R/APP/REN/ADORA2A/MTOR/DRD2
PIK3CB/CYP19A1/ITGB2/ITGAL/MAPK14/CNR2/PIK3CG/S1PR1/PIK3CA/SYK/AKT1/APP/F2/PIK3CD/PDE
JAK2/MDM2/EGFR/MAPK11/MAPK14/NPY5R/S1PR1/AKT1/PRKDC/MAPK1/PIM1/MTOR

CYP19A1/JAK2/CRHR1/AKT2/EGFR/VDR/PIK3CA/AKT1/PSEN1/CSF1R/SMO/PRKDC/MAPK1/FASN/DRD
CHRM1/ITGB2/CRHR1/MDM2/AKT2/MAPK14/SYK/PSEN1/SMO/MAPK1/MAPK8/PLK3/PPP1CC/PDPK1
CHRM1/PTGER1/NCSTN/HTR1A/APP/MAPK1/PDE4B/DRD2/CHRM2
CHRM1/PTGER1/NCSTN/HTR1A/APP/MAPK1/PDE4B/DRD2/CHRM2
NCSTN/APH1A/PSEN1/APP/PSEN2/REN/PSENEN
JAK2/ITGB2/TRPV1/EGFR/PSEN1/APP/SMO/ADORA2A
CHRM1/PTGER1/HTR1A/CRHR1/MTNR1A/CNR2/S1PR1/MTNR1B/ADORA2A/DRD2/AVPR2/CHRM2
JAK2/ITGB2/EGFR/PPP2CA/TNK2/SYK/IL6ST/PSEN1/CSF1R/APP/MTOR/ICAM1
CHRM1/PTGER1/NCSTN/HTR1A/APP/MAPK1/PDE4B/DRD2/CHRM2
CHRM1/PTGER1/NCSTN/HTR1A/APP/MAPK1/PDE4B/DRD2/CHRM2
CRHR1/PIK3CG/AKT1/CACNA2D1/MTNR1B/F2/PSEN2/PDE4B/P2RX7/ADORA2A/KCNH2/DRD2/PDPK1/I
EGFR/IL6ST/PSEN1/APP/SMO/F2/MAPK1/ADORA2A
NTRK1/NCSTN/APH1A/PSEN1/PTK2/PSEN2/APH1B/PSENEN
NTRK1/PIK3CB/JAK2/PIK3CG/PIK3CA/PTK2/F2/PIK3CD
JAK2/TRPV1/MDM2/PDE5A/PIK3CG/PIK3CA/CACNA2D1/PDE4B/KCNH2/MTOR/DRD2/CHRM2
MAPKAPK2/MAPK10/EGFR/MAPK11/MAPK9/MAPK14/SYK/AKT1/APP/MAPK1/IKBKB/MAPK8
CHRM1/ITGB2/MDM2/AKT2/MAPK14/PSEN1/SMO/MAPK1/MAPK8/PLK3
AKT2/NPY5R/SYK/AKT1/PSEN1/P2RX7/ADORA2A
NCSTN/APH1A/PSEN1/PSEN2/APH1B/P2RX7/PSENEN
JAK2/TRPV1/MDM2/PDE5A/PIK3CG/PIK3CA/CACNA2D1/PDE4B/KCNH2/MTOR/DRD2/CHRM2
TRPV1/CRHR1/VDR/PIK3CG/PSEN1/CACNA2D1/F2/PSEN2/PDE4B/P2RX7/ADORA2A/DRD2/PDPK1/ICAM
JAK2/CCNA2/TRPV1/CRHR1/MDM2/AKT2/PIK3CA/AKT1/PTK2/PRKDC/PDE3B/MTOR/PDPK1/ICAM1
NTRK1/JAK2/NCSTN/CRHR1/EGFR/NPY5R/CNR2/PSEN1/APP/MAPK1/ADORA2A/MTOR/DRD2/CHRM2
NTRK1/JAK2/NCSTN/CRHR1/EGFR/NPY5R/CNR2/PSEN1/APP/MAPK1/ADORA2A/MTOR/DRD2/CHRM2
PTGER1/CRHR1/EGFR/PDE5A/PDE10A/PPP2CA/SYK/MTNR1B/P2RX7/PDE3B/ADORA2A/MTOR/DRD2/P
HCRTR2/TACR1/CHRM1/CRHR1/S1PR1/F2/DRD2/CHRM2
CYP19A1/JAK2/AKT2/VDR/AKT1/CSF1R/SMO/MAPK1/FASN
NCSTN/EGFR/APH1A/IL6ST/AKT1/PSEN1/APP/PSEN2/APH1B/PSENEN
MAPK10/CRHR1/MDM2/AKT2/EGFR/CHEK1/AKT1/APP/MAPK8/PPP1CC/MTOR/DRD2
JAK2/ITGB2/TRPV1/EGFR/MAPK14/SYK/AKT1/F2/MTOR/ICAM1
MAPKAPK2/MAPK10/EGFR/MAPK11/MAPK9/MAPK14/SYK/AKT1/APP/MAPK1/IKBKB/MAPK8
CRHR1/PIK3CG/CACNA2D1/F2/PSEN2/PDE4B/P2RX7/ADORA2A/DRD2/PDPK1/ICAM1
TACR1/PTGER1/JAK2/TRPV1/CRHR1/PIK3CG/S1PR1/CACNA2D1/F2/P2RX7/DRD2/PDPK1
MAPKAPK2/JAK2/SYK/PSEN1/APP
NTRK1/ITGB2/CHEK1/MAPK14/NPY5R/AKT1/PSEN1/APP/PRKDC/MAPK1/MTOR/ICAM1
AKT2/ABCB1/MAPK14/AKT1/PSEN1/CACNA2D1/F2/P2RX7/KCNH2/PDPK1
CYP19A1/MAPK11/MAPK14/S1PR1/AKT1/SMO/PTK2/MAPK1/PIM1/MTOR
HCRTR2/HTR1A/CRHR1/MTNR1B/ADORA2A/MTOR/DRD2
NCSTN/APH1A/PSEN1/PSEN2/P2RX7/PSENEN
MAPKAPK2/PTGER1/JAK2/MAPK14/CNR2/AKT1/MAPK1/MAPK8/PDE4B/REN/P2RX7/ICAM1
JAK2/AKT2/VDR/AKT1/CSF1R/SMO/MAPK1
TRPV1/CRHR1/VDR/PIK3CG/PSEN1/CACNA2D1/F2/PSEN2/PDE4B/P2RX7/ADORA2A/DRD2/PDPK1/ICAM
JAK2/ITGB2/TRPV1/AKT1/MTOR/ICAM1
CRHR1/AKT2/NPY5R/AKT1/MTNR1B/KCNH2/MTOR/DRD2/ICAM1
CCNA2/TRPV1/CRHR1/MDM2/ADORA2A/MTOR/DRD2/ICAM1
TRPV1/CRHR1/VDR/PIK3CG/PSEN1/CACNA2D1/F2/PSEN2/PDE4B/P2RX7/ADORA2A/DRD2/PDPK1/ICAM
MAPK14/AKT1/PSEN1/SMO/F2/MAPK1/PLK1

NCSTN/APH1A/PSEN1/APP/PSEN2/PSENEN
JAK2/ITGB2/TRPV1/AKT1/MTOR/ICAM1
MAPK11/MAPK14/AKT1/SMO/PTK2/MAPK1/PIM1/MTOR
TRPV1/MDM2/EGFR/CHEK1/VDR/AKT1/MAPK1/MAPK8/P2RX7/PIM1/MTOR/ICAM1
CHRM1/ABCB1/AKT1/PSEN1/CACNA2D1/F2/P2RX7/ADORA2A/KCNH2/DRD2/PDPK1
MAPKAPK2/PTGER1/JAK2/MAPK14/CNR2/AKT1/MAPK1/MAPK8/PDE4B/REN/P2RX7/ICAM1
XIAP/NCSTN/MDM2/APH1A/PSEN1/PSEN2/APH1B/REN/P2RX7/PSENEN
NCSTN/AKT2/EGFR/AKT1/PSEN1/APP/SMO/ADORA2A
MAPK11/MAPK14/PIK3CA/AKT1/APP/SMO/PTK2/PRKDC/MAPK1/PIM1/MTOR/DRD2
CHRM1/PTGER1/HTR1A/CRHR1/MTNR1A/S1PR1/ADORA2A/DRD2/AVPR2/CHRM2
JAK2/ITGB2/TRPV1/EGFR/MAPK14/SYK/AKT1/F2/P2RX7/MTOR/ICAM1
CYP19A1/JAK2/ITGB2/TRPV1/CRHR1/EGFR/MAPK11/MAPK14/SYK/CSF1R/P2RX7/ADORA2A/DRD2
JAK2/ITGB2/TRPV1/HTR1A/AKT1/PSEN1/P2RX7/ADORA2A/MTOR/DRD2/ICAM1/CHRM2
PIK3CB/MAPK10/MAPK9/PIK3CA/SYK/MAPK1/IKBKB/MAPK8/PDPK1
CHRM1/TRPV1/AKT2/CNR2/AKT1/PSEN1/APP/CACNA2D1/MTNR1B/P2RX7/ADORA2A/KCNH2/DRD2
NCSTN/MDM2/EGFR/PPP2CA/MAPK9/AKT1/APP/MAPK1/MAPK8/P2RX7/DRD2/ICAM1
AKT2/ABCB1/AKT1/PSEN1/MTOR
CCNA2/CDK2/CNR2/ADORA2A/DRD2/ICAM1
EGFR/PSEN1/APP/SMO/ADORA2A
CYP19A1/JAK2/XIAP/EGFR/MAPK14/NPY5R/CNR2/PIK3CG/IL6ST/F2/PLA2G2A/ADORA2A
NTRK1/JAK2/NCSTN/AKT2/PIK3CA/PSEN1/APP/MTNR1B/ADORA2A/PDPK1
PIK3CB/MAPK10/MAPK9/PIK3CA/SYK/PTK2/MAPK1/IKBKB/MAPK8/PDPK1
NTRK1/JAK2/ITGB2/CRHR1/PIK3CA/AKT1/PSEN1/MTNR1B/ADORA2A/MTOR/PDPK1
MAPKAPK2/JAK2/MAPK11/MAPK14/SYK/IL6ST/PSEN1/CSF1R/APP/PRKDC/PDE4B/P2RX7/DRD2
CHRM1/ITGB2/MDM2/AKT2/MAPK14/PSEN1/SMO/MAPK1/MAPK8/PLK3
NTRK1/HCRTR1/EGFR/NPY5R/SYK/CSF1R/APP/MAPK1/PLA2G2A/DRD2/ICAM1
CHRM1/PTGER1/NCSTN/HTR1A/MAPK1/DRD2/CHRM2
JAK2/ITGB2/TRPV1/AKT1/MTOR/ICAM1
JAK2/TRPV1/MDM2/PDE5A/PIK3CG/CACNA2D1/PDE4B/KCNH2/DRD2/CHRM2
TRPV1/CRHR1/PIK3CG/PSEN1/CACNA2D1/F2/PSEN2/PDE4B/P2RX7/DRD2/PDPK1
TACR1/TRPV1/PDE5A/PIK3CG/PIK3CA/IL6ST/CACNA2D1/PTK2/PDE4B/KCNH2/MTOR/DRD2/CHRM2
CHRM1/PTGER1/NCSTN/HTR1A/MAPK1/DRD2/CHRM2
JAK2/CCNA2/CRHR1/MDM2/AKT2/PIK3CA/AKT1/PTK2/PRKDC/PDE3B/PDPK1
MDM2/MAPK9/AKT1/PSEN1/PTK2/PLK3/PLK1
EGFR/PIK3CA/AKT1/PSEN1/APP/PTK2/MAPK1/PDPK1
JAK2/ITGB2/PDE5A/MAPK14/PIK3CA/SYK/IL6ST/AKT1/PTK2/ADORA2A/PDPK1/ICAM1
CRHR1/CNR2/PIK3CG/SYK/PIK3CD/PDPK1
CYP19A1/JAK2/ITGB2/CRHR1/EGFR/MAPK11/MAPK14/SYK/CSF1R/P2RX7/ADORA2A/DRD2
PIK3CB/PIK3CG/PIK3CA/PIK3CD
JAK2/MDM2/EGFR/NPY5R/S1PR1/AKT1/MTOR
NCSTN/APH1A/PSEN1/APP/PSEN2/PSENEN
MAPK14/AKT1/SMO/MAPK1/PIM1/MTOR
NCSTN/APH1A/PSEN1/APP/PSENEN
MAPKAPK2/JAK2/SYK/PSEN1/APP
MAPKAPK2/JAK2/SYK/PSEN1/APP
PIK3CB/EGFR/PIK3CG/PIK3CA/AKT1/PTK2/PIK3CD/PLK3/MTOR/DRD2
EGFR/MAPK14/AKT1/SMO/F2/PRKDC/MAPK1/PIM1/MTOR/DRD2

CRHR1/AKT2/AKT1/MTNR1B/KCNH2/MTOR/DRD2
PIK3CB/JAK2/PIK3CG/PIK3CA/SYK/F2/MAPK1/P2RX7/ADORA2A/PDPK1/AVPR2
PIK3CB/JAK2/PIK3CG/PIK3CA/SYK/PSEN1/F2/MAPK1/P2RX7/ADORA2A/PDPK1
CRHR1/PIK3CG/APP/CACNA2D1/MTNR1B/F2/PSEN2/PDE4B/KCNH2/DRD2/PDPK1
NTRK1/JAK2/CRHR1/PIK3CA/AKT1/PSEN1/MTNR1B/ADORA2A/PDPK1
MAPKAPK2/PIK3CB/MAPK10/MAPK11/MAPK14/SYK/MAPK1/P2RX7
CRHR1/PIK3CG/CACNA2D1/F2/PSEN2/PDE4B/DRD2/PDPK1
NTRK1/EGFR/CHEK1/MAPK9/MAPK14/AKT1/MAPK8/P2RX7/DRD2
JAK2/ITGB2/TRPV1/AKT1/MTOR/ICAM1
NTRK1/HCRTR1/EGFR/NPY5R/CSF1R/APP/PLA2G2A/DRD2/ICAM1
NCSTN/CCNA2/EGFR/CDK2/MAPK9/AKT1/APP/MAPK1/MAPK8
ITGB2/PIK3CG/SYK/PTK2/PDE3B/ICAM1
EGFR/MAPK9/AKT1/MAPK1/MAPK8
XIAP/NCSTN/MDM2/APH1A/PSEN1/PSEN2/APH1B/REN/P2RX7/PSENEN
JAK2/MDM2/MAPK9/MAPK14/SYK/AKT1/PSEN1/APP/PTK2/PLK3/PLK1
PIK3CB/MDM2/AKT2/MAPK9/AKT1/PSEN1/APP/PTK2/P2RX7/PLK3/PLK1
TACR1/TRPV1/PDE5A/PIK3CG/PIK3CA/CACNA2D1/PDE4B/KCNH2/MTOR/DRD2/CHRM2
CYP19A1/ITGB2/MAPK14/CNR2/PIK3CG/S1PR1/SYK/PIK3CD/PDE4B
EGFR/S1PR1/SYK/CSF1R/P2RX7
MDM2/CDK2/CHEK1/PRKDC/PLK3/PLK1
MDM2/CDK2/CHEK1/PRKDC/PLK3/PLK1
MAPK14/AKT1/PSEN1/SMO/F2/MAPK1/PLK1
CCNA2/MDM2/EGFR/CDK2/MAPK9/AKT1/MAPK1/MAPK8
NTRK1/NCSTN/CRHR1/EGFR/APP/MAPK1/ADORA2A/DRD2
JAK2/CCNA2/MDM2/EGFR/CDK2/MAPK9/AKT1/PSEN1/APP/MAPK1/MAPK8/PLK3
NTRK1/CHRM1/NCSTN/CRHR1/EGFR/PSEN1/APP/MAPK1/MTOR/DRD2
JAK2/MDM2/EGFR/NPY5R/S1PR1/AKT1/PRKDC/MTOR
MDM2/CDK2/CHEK1/PRKDC/PLK3/PLK1
NTRK1/HCRTR1/EGFR/NPY5R/SYK/CSF1R/APP/PLA2G2A/DRD2/ICAM1
JAK2/MDM2/EGFR/NPY5R/S1PR1/AKT1/PRKDC/MTOR
CCNA2/MDM2/EGFR/CDK2/MAPK9/AKT1/MAPK1/MAPK8/PLK3
EGFR/PIK3CA/AKT1/PSEN1/APP/PTK2/PDPK1
MDM2/MAPK9/AKT1/PSEN1/PTK2/PLK3/PLK1
JAK2/ITGB2/TRPV1/AKT1/MTOR/ICAM1
TRPV1/MDM2/VDR/MAPK1/MAPK8/P2RX7/PIM1/MTOR/ICAM1
PIK3CB/AKT2/MAPK14/PIK3CG/PIK3CA/AKT1/CYP3A4/PIK3CD/MTOR
MAPKAPK2/EGFR/MAPK14/PIK3CG/PIK3CA/AKT1/PSEN1/PTK2/PDE3B/MTOR
NTRK1/PIK3CB/PIK3CA/PSEN1/CSF1R/APP/SMO/PTK2/PIK3CD/MAPK1/MARK2/DRD2
TRPV1/NPY5R/SYK/P2RX7/PLA2G2A/ADORA2A/DRD2
EGFR/PSEN1/APP/SMO/ADORA2A
CYP19A1/CRHR1/AKT2/SYK/AKT1/REN/P2RX7
PIK3CB/EGFR/PIK3CG/PIK3CA/AKT1/PTK2/PIK3CD/MTOR/DRD2
MDM2/MAPK9/AKT1/PSEN1/PLK3/PLK1
PIK3CB/JAK2/NCSTN/MAPK14/AKT1/SMO/PIK3CD/PDE4B/P2RX7
CRHR1/AKT2/AKT1/MTNR1B/KCNH2/MTOR/DRD2
JAK2/ITGB2/TRPV1/AKT1/MTOR/ICAM1
MAPK14/AKT1/SMO/PRKDC/MAPK1/PIM1/MTOR/DRD2

HCRTR2/MTNR1B/ADORA2A/MTOR/DRD2
HCRTR2/MTNR1B/ADORA2A/DRD2
HCRTR2/MTNR1B/ADORA2A/MTOR/DRD2
CRHR1/PIK3CG/SYK/PIK3CD/PDPK1
JAK2/ITGB2/TRPV1/AKT1/MTOR/ICAM1
CRHR1/PIK3CG/SYK/PIK3CD/PDPK1
EGFR/S1PR1/SYK/CSF1R/P2RX7
MDM2/CDK2/CHEK1/MAPK14/PRKDC/PLK3/PLK1
NCSTN/CRHR1/APP/MAPK1/ADORA2A/DRD2
NTRK1/NCSTN/CRHR1/EGFR/PSEN1/APP/MAPK1/MTOR/DRD2
CRHR1/PIK3CG/SYK/PIK3CD/PDPK1
NTRK1/TRPV1/AKT1/APP/PDPK1
TRPV1/AKT2/NPY5R/SYK/AKT1/PSEN1/P2RX7/PLA2G2A/ADORA2A/DRD2
NTRK1/TRPV1/MDM2/EGFR/VDR/AKT1/MAPK1/MAPK8/P2RX7/PIM1/MTOR/ICAM1
TRPV1/AKT2/NPY5R/SYK/AKT1/PSEN1/P2RX7/PLA2G2A/ADORA2A/DRD2
EGFR/VDR/S1PR1/SYK/CSF1R/P2RX7
MDM2/MAPK14/SYK/AKT1/PSEN1/SMO/F2/MAPK1/PLK1
HCRTR2/MTNR1B/ADORA2A/DRD2
PIK3CB/JAK2/PIK3CG/PIK3CA/SYK/F2/MAPK1/P2RX7/ADORA2A/PDPK1
CHRM1/HTR1A/MTNR1A/S1PR1/DRD2/CHRM2
ITGB2/PDE5A/ITGAL/PIK3CA/SYK/IL6ST/AKT1/ADORA2A/PDPK1/ICAM1
PDE5A/PIK3CG/PIK3CA/CACNA2D1/PDE4B/KCNH2/MTOR
CHRM1/TRPV1/AKT1/APP/P2RX7/ADORA2A/DRD2
MDM2/MAPK9/AKT1/PSEN1/PTK2/PLK3/PLK1
NTRK1/TRPV1/AKT1/APP/PDPK1
CYP2D6/CYP3A4/CYP2C19/CYP2C9
TRPV1/MDM2/VDR/MAPK1/MAPK8/P2RX7/PIM1/MTOR/ICAM1
JAK2/EGFR/MAPK11/MAPK14/SYK/CSF1R/P2RX7/ADORA2A/DRD2
PIK3CB/MDM2/AKT2/MAPK9/AKT1/PSEN1/APP/PTK2/P2RX7/PLK3/PLK1
PIK3CB/PIK3CA/PSEN1/PIK3CD/MAPK1/IKBKB/PDE4B/PDPK1
HTR1A/EGFR/AKT1/MTNR1B/ADORA2A/AVPR2/ICAM1
HTR1A/EGFR/AKT1/MTNR1B/ADORA2A/AVPR2/ICAM1
NTRK1/JAK2/EGFR/PSEN1/MAPK1
CRHR1/NPY5R/PSEN1/P2RX7/ADORA2A/DRD2
HTR1A/EGFR/AKT1/MTNR1B/ADORA2A/AVPR2/ICAM1
MDM2/CDK2/CHEK1/MAPK14/PRKDC/PLK3/PLK1
AKT2/PIK3CG/AKT1/PDE3B/MTOR
MAPK10/JAK2/CCNA2/MDM2/EGFR/CDK2/MAPK9/AKT1/MAPK1/MAPK8
PIK3CB/NCSTN/EGFR/VDR/AKT1/PSEN1/SMO/PRKDC/MAPK1/MTOR/PDPK1
NTRK1/PIK3CB/PIK3CA/CSF1R/APP/SMO/PTK2/PIK3CD/MAPK1
NTRK1/JAK2/PIK3CA/PSEN1/MTNR1B/ADORA2A/PDPK1
CCNA2/CRHR1/MDM2/MTOR/DRD2
MAPKAPK2/PIK3CB/MAPK11/MAPK14/PIK3CA/PTK2
NTRK1/PIK3CB/PIK3CA/CSF1R/APP/SMO/PTK2/PIK3CD/MAPK1
AKT2/SYK/AKT1/P2RX7/PLA2G2A/DRD2
HCRTR2/MTNR1B/ADORA2A/DRD2
MDM2/MAPK9/AKT1/PSEN1/PTK2/PLK3/PLK1

AKT2/SYK/AKT1/P2RX7
CRHR1/NPY5R/PSEN1/P2RX7/ADORA2A/DRD2
TRPV1/AKT1/APP/P2RX7/ADORA2A/DRD2
PIK3CB/PIK3CG/PIK3CA/SYK/F2/MAPK1/PDPK1
JAK2/ITGB2/TRPV1/HTR1A/AKT1/MTOR/ICAM1
JAK2/ITGB2/TRPV1/AKT1/MTOR/ICAM1
JAK2/EGFR/MAPK11/MAPK14/SYK/CSF1R/P2RX7/ADORA2A/DRD2
AKT2/NPY5R/AKT1/MTOR
ABCB1/PSEN1/CACNA2D1/F2/P2RX7/KCNH2/PDPK1
CYP19A1/CRHR1/AKT2/SYK/AKT1/REN/P2RX7
MDM2/CDK2/CHEK1/MAPK14/PRKDC/PLK3/PLK1
AKT2/PIK3CA/AKT1/PSEN1/APP/P2RX7/MTOR
NTRK1/TRPV1/CNR2/MAPK1/P2RX7/MTOR
MDM2/MAPK14/PSEN1/SMO/MAPK1/PPP1CC
MDM2/MAPK9/AKT1/PSEN1/PLK3/PLK1
CYP2D6/JAK2/PPP2CA/AKT1/PSEN1/APP/SMO/MARK2/MAPK8/PLK1
JAK2/TRPV1/PDE5A/PIK3CG
MAPK11/MAPK14/S1PR1/MAPK1/PIM1/MTOR
ITGB2/CRHR1/SYK/ADORA2A/DRD2/PDPK1/CHRM2
JAK2/ITGB2/TRPV1/AKT1/MTOR/ICAM1
EGFR/S1PR1/SYK/CSF1R/P2RX7
MDM2/MAPK14/PSEN1/SMO/MAPK1
CYP19A1/CRHR1/AKT2/ABCB1/SYK/AKT1/REN/P2RX7/PLA2G2A/DRD2
CYP2D6/CYP3A4/CYP2C9/ALOX5
NTRK1/TACR1/TRPV1/CRHR1
EGFR/MAPK9/AKT1/MAPK1/MAPK8
TRPV1/NPY5R/PIK3CG/IL6ST/F2/ICAM1
TRPV1/AKT1/APP/P2RX7/ADORA2A/DRD2
JAK2/CCNA2/MDM2/EGFR/CDK2/MAPK9/AKT1/MAPK1/MAPK8
CYP2D6/AKT2/MAPK14/AKT1/CYP3A4/CYP2C19/CYP2C9/ALOX5/FASN/MTOR
JAK2/EGFR/NPY5R/PIK3CG/SYK/IL6ST/PRKDC/IKBKB/PLA2G2A/PDPK1
CYP19A1/ITGB2/MAPK14/CNR2/PIK3CG/S1PR1/SYK/PIK3CD/PDE4B
ITGB2/PDE5A/PIK3CA/SYK/IL6ST/AKT1/ADORA2A/PDPK1/ICAM1
NPY5R/PSEN1/P2RX7/ADORA2A
MAPKAPK2/CYP2D6/SYK/CYP2C19/CYP2C9/ALOX5/FASN
PIK3CG/S1PR1/APP/PIK3CD/ICAM1
PIK3CG/S1PR1/AKT1/APP/PIK3CD/ICAM1
MAPK11/MAPK14/S1PR1/MAPK1/PIM1/MTOR
TRPV1/NPY5R/PSEN1/P2RX7/ADORA2A
CNR2/ADORA2A/DRD2/ICAM1
CYP2D6/CYP19A1/CYP3A4/CYP2C9
NPY5R/CNR2/ADORA2A/DRD2
EGFR/AKT1/AVPR2/ICAM1
MAPKAPK2/JAK2/SYK/PSEN1/APP
MAPKAPK2/CYP2D6/SYK/CYP2C19/CYP2C9/ALOX5
JAK2/TRPV1/EGFR/S1PR1/PIK3CA/SYK/CSF1R/SMO/P2RX7/DRD2/AVPR2
JAK2/IL6ST/JAK1

JAK2/IL6ST/JAK1
EGFR/MAPK14/S1PR1/IL6ST/AKT1/SMO/PTK2/MAPK1/P2RX7/FASN
PIK3CB/PIK3CA/SYK/PSEN1/PIK3CD/MAPK1/IKBKB/PDE4B/PDPK1
AKT2/SYK/AKT1/PLA2G2A/DRD2
HTR1A/EGFR/AKT1/MTNR1B/ADORA2A/AVPR2/ICAM1
PIK3CB/EGFR/PIK3CG/PIK3CA/PTK2/PIK3CD/MTOR
TRPV1/CACNA2D1/F2/PSEN2/P2RX7/DRD2/PDPK1
PIK3CA/AKT1/PTK2/MTOR
TRPV1/AKT2/ABCB1/NPY5R/SYK/AKT1/PSEN1/P2RX7/PLA2G2A/ADORA2A/DRD2
NTRK1/EGFR/PSEN1/ADORA2A/DRD2
PDE5A/PIK3CG/PIK3CA/CACNA2D1/PDE4B/KCNH2/MTOR
MDM2/EGFR/VDR/S1PR1/SYK/CSF1R/P2RX7
CYP2D6/EGFR/LSS/CYP3A4/CYP2C19/CYP2C9
TRPV1/AKT2/AKT1/PSEN1/CACNA2D1/KCNH2
PSEN1/P2RX7/ADORA2A/DRD2
CCNA2/TRPV1/MDM2/ICAM1
CRHR1/CACNA2D1/PDE4B/DRD2
AKT2/LSS/VDR/PIK3CG/AKT1/PTK2/F2/PDE3B/FASN/MTOR
CYP2D6/CCNA2/EGFR/CYP3A4/CYP2C19/CYP2C9/ICAM1
MDM2/CDK2/MAPK11/CHEK1/MAPK14/AKT1/PLK3
TRPV1/AKT2/AKT1/P2RX7/ADORA2A
MDM2/MAPK9/AKT1/PSEN1/PLK3/PLK1
JAK2/ITGB2/TRPV1/AKT1/MTOR/ICAM1
ITGB2/AKT2/EGFR/AKT1/MAPK8/PDPK1
NTRK1/TACR1/TRPV1/HTR1A/CRHR1
CYP19A1/CRHR1/AKT2/ABCB1/SYK/AKT1/REN/P2RX7/PLA2G2A/DRD2
ITGB2/MAPK14/PIK3CG/SYK/PIK3CD/PDE4B
MAPKAPK2/MAPK14/PIK3CG/PIK3CA/AKT1/PSEN1/PTK2/MTOR
CYP2D6/CYP19A1/LSS/VDR/CYP3A4/APP/CYP2C19/CYP2C9/FASN
MAPK10/TRPV1/MDM2/AKT2/EGFR/CHEK1/MAPK14/AKT1/MAPK8
MAPK10/TRPV1/MDM2/AKT2/EGFR/CHEK1/MAPK14/AKT1/MAPK8
AKT2/IL6ST/AKT1/PPP1CC/MTOR
TRPV1/MDM2/VDR/P2RX7/PIM1
CRHR1/PIK3CG/SYK/PIK3CD/PDPK1
NTRK1/EGFR/TNK2/PTK2
MAPK14/PSEN1/SMO/MAPK1
AKT2/IL6ST/AKT1/PPP1CC/MTOR
AKT2/IL6ST/AKT1/PPP1CC/MTOR
NCSTN/CRHR1/PSEN1/APP/MAPK1/ADORA2A/DRD2
AKT2/AKT1/MTOR
NCSTN/EGFR/MAPK9/AKT1/APP/MAPK1/MAPK8
HTR1A/EGFR/AKT1/AVPR2/ICAM1
MDM2/NPY5R/S1PR1/PSEN1/SMO/PTK2/PIM1/MTOR
TACR1/PDE5A/PIK3CG/IL6ST/PTK2/PDE4B/MTOR/CHRM2
CRHR1/AKT1/SMO/MAPK1/DRD2/PDPK1
JAK2/CCNA2/CDK2/CHEK1
PSEN1/P2RX7/ADORA2A/DRD2

JAK2/TRPV1/PDE5A/PIK3CG
NTRK1/JAK2/ITGB2/AKT1/APP/SMO/IKBKB/ICAM1
CYP19A1/JAK2/HTR1A/CRHR1/EGFR/MTNR1B/REN/DRD2
PPP2CA/AKT1/PSEN1/PRKDC/P2RX7/PLK1/ADORA2A/MTOR/DRD2/PDPK1
TRPV1/AKT2/AKT1/PSEN1/CACNA2D1/KCNH2/DRD2
MAPK11/MAPK14/MAPK1/PIM1/MTOR
CCNA2/CRHR1/MDM2/MTOR/DRD2
NTRK1/CYP19A1/EGFR/MAPK14/VDR/AKT1/PTK2/MAPK1/REN/ICAM1
JAK2/TNK2/SYK/IL6ST/CSF1R/MTOR/ICAM1
MAPK14/PSEN1/SMO/MAPK1
AKT2/AKT1/PSEN1/APP/P2RX7
NTRK1/CYP19A1/EGFR/MAPK14/VDR/AKT1/PTK2/MAPK1/REN/ICAM1
PDE5A/PDE10A/PDE4B
NTRK1/TRPV1/CRHR1
JAK2/MAPK11/MAPK14/MAPK1/PIM1
CYP19A1/CRHR1/REN/P2RX7
AKT2/PIK3CA/AKT1/PRKDC/PDE3B/MTOR/PDPK1/ICAM1
MAPK10/EGFR/ABCB1/PLK3/PDPK1
PDE5A/IL6ST/PTK2/MTOR
CRHR1/CNR2/SYK/PDPK1
CCNA2/CRHR1/MDM2/AKT1/PSEN2/PLK3/MTOR/DRD2/ICAM1
MAPK11/MAPK14/MAPK1/PIM1/MTOR
HTR1A/EGFR/AKT1/AVPR2/ICAM1
MAPK14/PSEN1/CSF1R/APP/PIK3CD/FASN/MTOR
XIAP/MAPK9/AKT1/PSEN1/PLK1/MTOR/AVPR2
CYP2D6/EGFR/LSS/CYP3A4/CYP2C19/CYP2C9
MAPK11/MAPK14/SYK/CSF1R/P2RX7/DRD2
HTR1A/PSEN1/P2RX7/ADORA2A/DRD2/CHRM2
PDE5A/IL6ST/PTK2/MTOR
PSEN1/APP/CACNA2D1/ICAM1
TRPV1/CACNA2D1/F2/P2RX7/DRD2/PDPK1
PIK3CB/AKT1/PIK3CD/PDE4B/P2RX7
MAPKAPK2/JAK2/SYK/PSEN1/APP
ITGB2/MAPK14/PIK3CG/SYK/PIK3CD/PDE4B
TRPV1/NPY5R/P2RX7/ADORA2A
PIK3CB/PIK3CD/PDE4B
NPY5R/P2RX7/ADORA2A
MDM2/PSEN1/APP
AKT2/IL6ST/AKT1/PPP1CC/MTOR
PIK3CB/ITGB2/ITGAL/PIK3CA/SYK/PTK2/MAPK1/P2RX7/ADORA2A
MAPK14/SYK/AKT1/PSEN1/SMO/MAPK1
NTRK1/JAK2/PIK3CA/PSEN1/MTNR1B/ADORA2A/PDPK1
CYP19A1/ITGB2/MAPK14/PIK3CG/SYK/PIK3CD/PDE4B
CCNA2/CRHR1/MDM2/AKT1/PSEN2/PLK3/MTOR/DRD2/ICAM1
MAPKAPK2/JAK2/SYK/PSEN1/APP
SYK/P2RX7/PLA2G2A/DRD2
NPY5R/CNR2/ADORA2A/DRD2

NCSTN/APP/ADORA2A/DRD2
PTGER1/CRHR1/PDE10A/MTNR1B/PDE3B/ADORA2A/DRD2
CNR2/ADORA2A/DRD2/ICAM1
PIK3CG/SYK/PTK2/PDE3B
ITGB2/CRHR1/SYK/PDPK1
JAK2/IL6ST/JAK1/ICAM1
HTR1A/CRHR1/SYK/ADORA2A/DRD2
EGFR/S1PR1/SYK/CSF1R/P2RX7
MAPK10/EGFR/MAPK9/SYK/AKT1/APP/MAPK8
MDM2/MAPK9/AKT1/PSEN1/PTK2/PLK3/PLK1
MDM2/MAPK9/AKT1/PSEN1/PTK2/PLK3/PLK1
MTNR1B/ADORA2A/DRD2
AKT2/AKT1/SMO
PPP2CA/AKT1/PSEN1/PRKDC/P2RX7/PLK1/ADORA2A/MTOR/DRD2/PDPK1
JAK2/TRPV1/MAPK14/SYK/AKT1/MAPK1/IKBKB/ICAM1
PIK3CB/JAK2/NCSTN/MAPK14/PIK3CD/PDE4B
MDM2/CDK2/CHEK1/MAPK14/PRKDC/PLK3/PLK1
EGFR/AKT1/MAPK1/PDPK1
JAK2/EGFR/MAPK11/MAPK14/SYK/CSF1R/MTNR1B/P2RX7/ADORA2A/DRD2
ITGB2/CRHR1/SYK/ADORA2A/DRD2/PDPK1/CHRM2
PPP2CA/AKT1/PSEN1/APP/MARK2/MAPK8/PLK1
PIK3CB/PIK3CA/SYK/PSEN1/PTK2/PIK3CD/MAPK1/IKBKB/PDE4B/PDPK1
PIK3CB/PIK3CA/SYK/PSEN1/PTK2/PIK3CD/MAPK1/IKBKB/PDE4B/PDPK1
TRPV1/PIK3CG/APP/REN/P2RX7/ADORA2A
ITGB2/PIK3CA/SYK/IL6ST/AKT1/PDPK1/ICAM1
ITGB2/CSF1R/PTK2/F2/MARK2/ICAM1
JAK2/ITGB2/TRPV1/APP
JAK2/ITGB2/TRPV1/APP
CRHR1/APP/MTOR/DRD2
MAPK14/MAPK1/PIM1/MTOR
TRPV1/MDM2/EGFR/VDR/P2RX7/PIM1/MTOR
NTRK1/EGFR/PSEN1/ADORA2A/DRD2
CCNA2/MDM2/EGFR/CDK2/CCNA1/AKT1/PRKDC/PLK3
AKT2/AKT1/MTOR
PSEN1/P2RX7/ADORA2A
EGFR/MAPK14/VDR/AKT1/PTK2/MAPK1
SYK/P2RX7/PLA2G2A/DRD2
SYK/P2RX7/PLA2G2A/DRD2
JAK2/EGFR/NPY5R/PIK3CG/IL6ST/PLA2G2A
JAK2/ITGB2/TRPV1/SYK/APP
TRPV1/NPY5R/PSEN1/P2RX7/ADORA2A
CHRM1/CSF1R/PPP1CC/MTOR
PIK3CB/PIK3CG/PIK3CA/PIK3CD
JAK2/IL6ST/JAK1/ICAM1
ABCB1/PSEN1/P2RX7/ADORA2A
JAK2/XIAP/SYK/JAK1/PRKDC/IKBKB/DRD2/PDPK1
ITGB2/EGFR/SYK

CYP2D6/CYP3A4/CYP2C9
CCNA2/CRHR1/MDM2/AKT1/PSEN2/PLK3/MTOR/DRD2/ICAM1
CYP19A1/JAK2/HTR1A/CRHR1/EGFR/MTNR1B/REN/DRD2
TRPV1/CACNA2D1/F2/P2RX7/DRD2/PDPK1
MAPK14/MAPK1/PIM1/MTOR
XIAP/MAPK9/AKT1/PSEN1/PLK1/MTOR/AVPR2
PIK3CB/PIK3CA/AKT1/PSEN1/PTK2/MARK2/MAPK8/PLK3/MTOR/DRD2
PIK3CB/PIK3CA/AKT1/PSEN1/PTK2/MARK2/MAPK8/PLK3/MTOR/DRD2
JAK2/TRPV1/MAPK14/SYK/AKT1/MAPK1/IKBKB/ICAM1
MAPK11/MAPK14/MAPK1/PIM1/MTOR
MTNR1B/ADORA2A/DRD2
CRHR1/APP/MTOR/DRD2
TRPV1/MDM2/CACNA2D1/KCNH2/DRD2
JAK2/EGFR/MAPK11/MAPK14/SYK/CSF1R/MTNR1B/P2RX7/ADORA2A/DRD2
AKT2/SYK/AKT1/P2RX7/PLA2G2A/DRD2
MAPK14/SYK/AKT1/PSEN1/SMO/MAPK1
CCNA2/EGFR/CCNA1/AKT1/PLK1
CYP19A1/JAK2/HTR1A/CRHR1/EGFR/MTNR1B/REN/DRD2
PSEN1/APP/CACNA2D1/ICAM1
MAPK14/SYK/AKT1/MAPK1/MAPK8/PDE4B/ICAM1
AKT2/IL6ST/AKT1/PPP1CC/MTOR
AKT2/NPY5R/AKT1
ADORA2A/DRD2/AVPR2
APP/ADORA2A/DRD2
MAPK14/PSEN1/SMO/MAPK1
ITGB2/PIK3CG/SYK/PIK3CD/PDE4B
CCNA2/EGFR/CCNA1/AKT1/PLK1
CYP2D6/JAK2/AKT1/PSEN1/SMO/MAPK8
ITGB2/EGFR/SYK
ADORA2A/DRD2/AVPR2
ITGB2/CRHR1/SYK/PDPK1
MAPK11/MAPK14/MAPK1/PIM1
MDM2/CDK2/PRKDC/PLK3
MDM2/CDK2/PRKDC/PLK3
EGFR/AKT1/AVPR2/ICAM1
MAPK14/PSEN1/SMO/MAPK1
MDM2/MAPK9/AKT1/PSEN1/PTK2/PLK3/PLK1
CYP2D6/CYP3A4/CYP2C19/CYP2C9/ALOX5
ADORA2A/DRD2/AVPR2
PSEN1/APP/P2RX7
PIK3CG/AKT1/PDE3B
CYP19A1/AKT2/PIK3CG/AKT1/CYP3A4/PDE3B/PLA2G2A/MTOR
PIK3CB/PIK3CG/PIK3CA/CSF1R/PIK3CD/PLA2G2A
MDM2/CDK2/PRKDC/PLK3
MDM2/CDK2/PRKDC/PLK3
MDM2/CDK2/PRKDC/PLK3
JAK2/PIK3CG/MTOR/PDPK1/ICAM1

AKT2/LSS/VDR/AKT1/PSEN1/APP/P2RX7/FASN/MTOR
ITGB2/CRHR1/SYK
ADORA2A/DRD2/AVPR2
ITGB2/PIK3CG/PIK3CD/ICAM1
ITGB2/PIK3CA/SYK/IL6ST/AKT1/PDPK1/ICAM1
AKT2/IL6ST/AKT1/PPP1CC/MTOR
MAPKAPK2/JAK2/SYK/PSEN1/APP
CRHR1/APP/CACNA2D1/MTNR1B/PDE4B/DRD2
MDM2/MAPK14/SYK/AKT1/PSEN1/SMO/MAPK1/PPP1CC
MAPK14/MAPK1/PIM1/MTOR
CRHR1/NPY5R/DRD2
CHEK1/MAPK14/PRKDC/MTOR/ICAM1
MDM2/MAPK14/SYK/AKT1/PSEN1/SMO/MAPK1/PPP1CC
CRHR1/ABCB1/APP/CACNA2D1/MTNR1B/PDE4B/DRD2
TRPV1/ADORA2A/DRD2/AVPR2
MDM2/CDK2/PRKDC/PLK3
MDM2/CDK2/PRKDC/PLK3
MDM2/MAPK9/AKT1/PSEN1/PLK3/PLK1
PTGER1/CRHR1/PDE10A/PDE3B/ADORA2A/DRD2
NCSTN/PDE5A/PIK3CG/SYK/IL6ST/P2RX7
MDM2/EGFR/CDK2/AKT1/PRKDC/PLK3
CYP19A1/HTR1A/CRHR1/SYK/REN/ADORA2A/DRD2
MDM2/CDK2/PRKDC/PLK3
PIK3CB/PIK3CG/PIK3CA/PIK3CD
VDR/AKT1/MAPK1/MTOR
ITGB2/PIK3CG/SYK/PIK3CD/PDE4B
EGFR/VDR/CNR2/AKT1
ITGB2/AKT2/EGFR/AKT1/MAPK8/PDPK1
CCNA2/CDK2/CHEK1/ABCB1/APP/PLK3/PLK1
TRPV1/REN/ADORA2A/DRD2/AVPR2
PSEN1/P2RX7/ADORA2A/DRD2/CHRM2
MDM2/CDK2/MAPK11/CHEK1/MAPK14/AKT1/PLK3
PIK3CA/APP/SMO/DRD2
CRHR1/ABCB1/APP/CACNA2D1/MTNR1B/PDE4B/DRD2
CRHR1/MDM2/REN
HTR1A/SYK/PSEN1/P2RX7/ADORA2A/DRD2/CHRM2
MAPK11/MAPK14/MAPK1/PIM1
MAPKAPK2/JAK2/SYK/PSEN1/APP
MAPK14/SYK/AKT1/PSEN1/SMO/MAPK1
CHRM1/IL6ST/F2/PPP1CC/MTOR
MAPKAPK2/JAK2/SYK/PSEN1/APP
TRPV1/F2/P2RX7/DRD2/PDPK1
MTNR1B/ADORA2A/MTOR
PIK3CB/PIK3CD/P2RX7
ITGB2/CRHR1/MAPK14/SYK/F2/PRKDC/P2RX7/PDPK1/ICAM1
TRPV1/F2/P2RX7/DRD2/PDPK1
MDM2/EGFR/CDK2/CCNA1/AKT1/PRKDC/PLK3

CRHR1/APP/APH1B/ADORA2A/MTOR/DRD2
AKT2/AKT1/MTOR
APP/MAPK1/PLK1
AKT2/AKT1/MTOR
JAK2/IL6ST/JAK1
TRPV1/F2/P2RX7/DRD2/PDPK1
AKT2/AKT1/PSEN1/APP/P2RX7/MTOR
CRHR1/ABCB1/APP/CACNA2D1/MTNR1B/PDE4B/DRD2
MAPK14/SYK/F2/ALOX5
MDM2/LSS/SMO/PRKDC/MAPK1/PLK1/PIM1
AKT2/AKT1/MTOR
MDM2/EGFR/CDK2/AKT1/PRKDC/PLK3
TRPV1/F2/P2RX7/DRD2/PDPK1
MAPK14/S1PR1/IL6ST/PTK2/MAPK1/P2RX7
EGFR/AKT1/AVPR2/ICAM1
ITGB2/CRHR1/SYK/P2RX7/PDPK1/ICAM1
MAPK14/AKT1/MAPK1/MAPK8/PDE4B/ICAM1
MDM2/EGFR/CDK2/CHEK1/AKT1/APP/PRKDC/PLK3/PLK1
NPY5R/PIK3CG/IL6ST
MAPKAPK2/SYK/ALOX5
PDE5A/PDE10A/PDE4B
MTNR1B/ADORA2A/DRD2
CRHR1/SYK/PDPK1
SYK/PLA2G2A/DRD2
SYK/PLA2G2A/DRD2
CRHR1/EGFR/PSEN1/CSF1R/APP/SMO/DRD2/AVPR2
MDM2/EGFR/MAPK9/AKT1/PSEN1/PTK2/PLK3/PLK1
TRPV1/PIK3CG/APP/REN/P2RX7
NTRK1/SMO/IKBKB/FASN/PDPK1/ICAM1
CCNA2/MDM2/AKT1/PLK3/MTOR/ICAM1
PDE5A/IL6ST/PTK2/MTOR
PDE5A/PDE10A/PDE4B
CRHR1/SYK/PDPK1
NTRK1/EGFR/ADORA2A
ITGB2/SYK/AKT1
MDM2/VDR/PIM1
MAPK11/MAPK14/SYK/CSF1R/P2RX7/DRD2
JAK2/PPP2CA/IL6ST/CSF1R/F2
MDM2/EGFR/CDK2/CHEK1/AKT1/APP/PLK3/DRD2
MAPK14/AKT1/MAPK1/MAPK8/PDE4B/ICAM1
MAPK10/PSEN1/P2RX7/ADORA2A/DRD2/CHRM2
ITGB2/CRHR1/SYK
MDM2/MTOR/DRD2
PIK3CA/AKT1/MTOR
NPY5R/ADORA2A/MTOR
MDM2/MTOR/DRD2
PSEN1/ADORA2A/DRD2

PIK3CB/PIK3CA/SYK/PTK2/MAPK1
PIK3CB/PIK3CA/SYK/PTK2/MAPK1
CRHR1/APP/MTOR/DRD2
PDE5A/IL6ST/PTK2/MTOR
NCSTN/PDE5A/PIK3CG/SYK/IL6ST/CSF1R/P2RX7
MDM2/EGFR/CDK2/AKT1/APP/PLK3/DRD2
JAK2/ITGB2/CRHR1/PIK3CA/SYK/IL6ST/AKT1/PDPK1
JAK2/XIAP/MDM2/SYK/AKT1/ADORA2A
EGFR/CHEK1/AKT1/MAPK8
TRPV1/NPY5R/PSEN1/P2RX7/ADORA2A
MDM2/EGFR/CHEK1/AKT1/MAPK8
AKT2/AKT1/PSEN1/APP/P2RX7
AKT2/PIK3CA/AKT1/PRKDC/PDE3B/PDPK1
NCSTN/ITGB2/ITGAL/MAPK14/SYK/PSEN1/F2/MAPK1/ALOX5
AKT1/MTOR/PDPK1
PSEN1/P2RX7/ADORA2A
ITGB2/SYK/AKT1
CHRM1/HTR1A/CHRM2
CCNA2/MDM2/AKT1/PLK3/MTOR/ICAM1
PIK3CB/PIK3CA/SYK/PTK2/MAPK1
JAK2/XIAP/SYK/JAK1/PRKDC/IKBKB/DRD2/PDPK1
ITGB2/CRHR1/MAPK14/SYK/P2RX7/PDPK1
TRPV1/AKT1/F2/P2RX7/DRD2/PDPK1
CRHR1/PIK3CG/SYK/PSEN1/PTK2/PIK3CD/PLK1/PDPK1
VDR/AKT1/MAPK1
MAPK11/MAPK14/SYK
CHRM1/PPP1CC/MTOR
AKT2/AKT1/MTOR
ADORA2A/DRD2/AVPR2
JAK2/ITGB2/PIK3CA/SYK/IL6ST/AKT1/PDPK1/ICAM1
PIK3CB/PIK3CA/SYK/PTK2/MAPK1
PIK3CA/APP/SMO/MTOR/DRD2
AKT2/AKT1/PTK2/F2/MTOR
JAK2/PPP2CA/IL6ST/CSF1R/F2
JAK2/MDM2/MAPK9/AKT1/PSEN1/MAPK8/ADORA2A/ICAM1
JAK2/PPP2CA/IL6ST/CSF1R
ITGB2/EGFR/SYK
ITGB2/EGFR/SYK
PIK3CA/AKT1/PTK2/MTOR
XIAP/MDM2/AKT1/ADORA2A
PDE5A/PIK3CA/SYK/IL6ST/AKT1/ADORA2A/PDPK1
NTRK1/ITGB2/APP/IKBKB/ICAM1
AKT2/AKT1/MTOR
CHRM1/JAK2/CHRM2
JAK2/PPP2CA/IL6ST/CSF1R
EGFR/AKT1/PSEN1/APP
EGFR/S1PR1/SYK/CSF1R/SMO/P2RX7

JAK2/MAPK14/PSEN1/CSF1R/APP/PIK3CD/FASN/MTOR
MAPK10/AKT2/EGFR/AKT1/IKBKB/PLK1/PDPK1
MAPK11/MAPK14/MAPK1/PIM1/MTOR
MAPK14/MAPK1/PIM1/MTOR
MAPK14/MAPK1/PIM1/MTOR
AKT2/MAPK14/AKT1
MAPK14/MAPK1/PIM1
JAK2/AKT1/PTK2
MAPK14/MAPK1/PIM1/MTOR
JAK2/MTOR/PDPK1/ICAM1
MAPK1/ADORA2A/KCNH2/DRD2/AVPR2
CYP2D6/CYP19A1/VDR/CYP3A4/CYP2C9/REN
MAPK11/MAPK14/S1PR1/MAPK1/PIM1/MTOR
CCNA2/MDM2/AKT1/PLK3/MTOR/ICAM1
MAPK11/MAPK14/MAPK1/PIM1/MTOR
CHRM1/HTR1A/CHRM2
ITGB2/HTR1A/DRD2
JAK2/IL6ST/CSF1R/F2
CRHR1/CACNA2D1/PDE4B/DRD2
PPP2CA/AKT1/PSEN1/PLK1/ADORA2A/PDPK1
MAPK11/MAPK14/MAPK1/PIM1/MTOR
PIK3CB/PIK3CA/AKT1/PTK2/MAPK8/PLK3/MTOR
PDE5A/PDE10A/PDE4B
CACNA2D1/P2RX7/ADORA2A/KCNH2
MDM2/EGFR/AKT1/APP
CRHR1/MTNR1B/KCNH2/DRD2
CYP19A1/NPY5R/CNR2/F2/ADORA2A/DRD2
JAK2/XIAP/MDM2/SYK/AKT1/ADORA2A
JAK2/PPP2CA/IL6ST/CSF1R/F2
MAPK11/MAPK14/SYK/CSF1R/P2RX7/DRD2
JAK2/IL6ST/CSF1R/F2
XIAP/MDM2/AKT1/ADORA2A
MAPKAPK2/JAK2/SYK/PSEN1/APP
IKBKB/FASN/ICAM1
TRPV1/REN/DRD2/AVPR2
MDM2/EGFR/VDR/PIM1
EGFR/AKT1/PSEN1/APP
HTR1A/SYK
ITGAL/ICAM1
EGFR/PDE5A
AKT2/AKT1
P2RX7/ADORA2A
REN/DRD2
AKT1/MTOR
PDE5A/PIK3CG
CRHR1/DRD2
MAPK11/MAPK14

FASN/ICAM1
PPP2CA/MARK2
PSEN1/APP
CYP19A1/PDE5A/NPY5R/CNR2/AKT1/PRKDC/ADORA2A/DRD2
NTRK1/MAPKAPK2/TRPV1/AKT1/MAPK1/MTOR
MAPKAPK2/JAK2/SYK/PSEN1/APP
MAPKAPK2/JAK2/SYK/PSEN1/APP
EGFR/S1PR1/SYK/CSF1R/SMO/PRKDC/MAPK1/P2RX7
MDM2/EGFR/CDK2/CHEK1/PRKDC/PLK3/PLK1
CYP2D6/CYP3A4/CYP2C9/ALOX5/FASN
CCNA2/CDK2/ABCB1/APP/PLK3/PLK1
MDM2/CDK2/PRKDC/PLK3/PLK1
CRHR1/MAPK14/MAPK8
AKT2/AKT1/MTOR
MAPK1/MAPK8/MTOR
NTRK1/CRHR1/P2RX7
EGFR/AKT1/PDPK1
EGFR/PSEN1/CSF1R/SMO/DRD2/AVPR2
MDM2/EGFR/CDK2/AKT1/APP/PRKDC/PLK3/PLK1
MDM2/CDK2/PRKDC/PLK3
PDE5A/PDE10A/PDE4B
PTGER1/NCSTN/DRD2
AKT2/MAPK14/AKT1
EGFR/VDR/SYK
MAPKAPK2/JAK2/SYK/PSEN1/APP
PIK3CG/PIK3CD
AKT1/ALOX5
ITGB2/SYK
CYP19A1/NPY5R/CNR2/F2/ADORA2A
JAK2/PPP2CA/IL6ST/CSF1R/F2
AKT2/MAPK14/AKT1/MTOR
SYK/CYP2C9/ALOX5/FASN
PSEN1/P2RX7/ADORA2A/DRD2/CHRM2
PSEN1/P2RX7/ADORA2A/DRD2/CHRM2
AKT1/SMO/PDPK1
MAPKAPK2/MAPK14/MTOR
PSEN1/PRKDC/MAPK1
JAK2/XIAP/MDM2/MAPK14/SYK/AKT1/APP/ADORA2A
TACR1/PDE5A/PIK3CG/PDE4B/CHRM2
CHRM1/PTGER1/TRPV1/VDR
EGFR/CDK2/CHEK1/PIK3CA/AKT1/PRKDC/MAPK1
AKT2/MAPK14/AKT1/MTOR
PIK3CB/TNK2/SYK/DRD2
PPP2CA/AKT1/PSEN1/PLK1/ADORA2A/PDPK1
NPY5R/PIK3CG/IL6ST
AKT2/AKT1/MTOR
AKT2/AKT1/MTOR

MDM2/PRKDC/PLK1
PSEN1/APP/P2RX7
PSEN1/APP/P2RX7
MTOR/PDPK1/ICAM1
MAPK1/MAPK8/MTOR
APP/MAPK1/PLK1
PSEN1/APP/P2RX7
ITGB2/ITGAL/SYK/PTK2
NTRK1/PRKDC
SYK/JAK1
VDR/CYP3A4
MTNR1B/DRD2
MAPKAPK2/TRPV1/AKT1/MAPK1/MTOR
XIAP/MDM2/AKT1/PSEN1/APP/F2/ADORA2A
HCRTR2/HCRTR1/CRHR1/NPY5R
PIK3CB/AKT1/PIK3CD/P2RX7
CRHR1/PDE10A/PDE3B
EGFR/PIK3CA/AKT1/APP
ITGAL/PSEN1/MTOR/ICAM1
PDE5A/IL6ST/PTK2/MTOR
MDM2/CDK2/PRKDC/PLK3
MDM2/EGFR/AKT1/APP
JAK2/PDE5A/AKT1/PTK2/ADORA2A
NTRK1/MAPK1/DRD2
MDM2/CDK2/CHEK1/PRKDC/PLK3/PLK1
PDE5A/IL6ST/PTK2/MTOR
EGFR/VDR/CNR2/AKT1
PIK3CG/PIK3CD
EGFR/SMO
JAK2/APP
SYK/JAK1
PIK3CG/PIK3CD
TRPV1/CACNA2D1
CCNA2/EGFR/CDK2/PPP2CA
MDM2/AKT2/CDK2/PLK3
MAPKAPK2/MAPK14/MTOR
MDM2/EGFR/AKT1
NTRK1/IL6ST/PSEN1/SMO/MARK2/PPP1CC/MTOR/DRD2
PSEN1/CSF1R/SMO/PTK2/DRD2
PDE5A/IL6ST/PTK2/MTOR
NCSTN/PDE5A/PIK3CG/SYK/IL6ST/P2RX7
CYP2D6/CYP2C19/CYP2C9
CCNA2/EGFR/PRKDC
EGFR/CNR2/AKT1
PDE5A/PDE10A/PDE4B
CRHR1/MTNR1B/DRD2
TACR1/TRPV1/DRD2/CHRM2

CYP2D6/CYP2C19/CYP2C9/ALOX5
NCSTN/PDE5A/PIK3CG/SYK/IL6ST/P2RX7
PDE5A/IL6ST/PTK2/MTOR
CSF1R/MTOR/ICAM1
JAK2/APP/P2RX7
ADORA2A/DRD2/AVPR2
MAPKAPK2/MAPK14/MTOR
PDE10A/MTNR1B
ADORA2A/DRD2
ADORA2A/DRD2
MAPK1/PLK3
MAPK1/PLK3
JAK2/APP
NTRK1/ICAM1
SYK/JAK1
CHEK1/PLK1
CRHR1/DRD2
TRPV1/CACNA2D1
NCSTN/ITGB2/ITGAL/MAPK14/SYK/PSEN1/MAPK1/ALOX5
EGFR/VDR/AKT1/SMO/PRKDC/MTOR/PDPK1
EGFR/PSEN1/DRD2
ITGB2/CRHR1/SYK
NCSTN/ITGB2/ITGAL/MAPK14/SYK/PSEN1/MAPK1/ALOX5
ITGB2/CRHR1/PIK3CA/SYK/IL6ST/AKT1/PDPK1
S1PR1/IKBKB/FASN/ICAM1
EGFR/CDK2/AKT1/PRKDC/MAPK1
CRHR1/VDR/AKT1/MAPK1/MTOR
JAK2/ITGB2/SYK/PSEN1/DRD2
ITGB2/HTR1A/DRD2
ITGB2/HTR1A/DRD2
F2/P2RX7/PDPK1
MAPK11/MAPK14/SYK
JAK2/PSEN1/SMO
EGFR/AKT1/PLK1/PDPK1
PSEN1/P2RX7/ADORA2A/DRD2/CHRM2
PIK3CB/PIK3CG/PIK3CA/PIK3CD
SYK/CYP2C9/ALOX5
CNR2/CACNA2D1/MTNR1B
PPP2CA/AKT1/PSEN1/PLK1/ADORA2A/PDPK1
NTRK1/MAPK10
CRHR1/SYK
CRHR1/DRD2
VDR/CYP3A4
CRHR1/SYK
AKT2/AKT1
JAK2/APP
SYK/APP

AKT2/AKT1
NTRK1/TRPV1
NTRK1/TRPV1
ADORA2A/DRD2
AKT2/AKT1
CACNA2D1/PDE4B
CRHR1/PSEN1/MTOR/DRD2
XIAP/MAPK9/PLK1/AVPR2
JAK2/IKBKB/MTOR/DRD2
AKT2/PIK3CA/IL6ST/AKT1/PPP1CC/MTOR
CYP19A1/MAPK14/AKT1/APP/ICAM1
CYP19A1/LSS/VDR/CYP3A4/FASN
NCSTN/ITGB2/ITGAL/MAPK14/SYK/PSEN1/MAPK1/ALOX5
MDM2/CDK2/PLK3
EGFR/PSEN1/DRD2
PIK3CA/AKT1/PDPK1
MAPK11/MAPK14/SYK
EGFR/AKT1/PDPK1
MAPKAPK2/XIAP/ITGB2/IKBKB/PDPK1
MAPK11/MAPK14/S1PR1/SMO/MAPK1/PIM1/MTOR
JAK2/PDE5A/AKT1/PTK2/PDE3B/ADORA2A
LSS/VDR/AKT1/FASN/MTOR
PIK3CA/AKT1/PDPK1
ABCB1/SMO/PIM1/DRD2
MAPK1/MTOR
HTR1A/SYK
JAK2/APP
AKT2/AKT1
PDE5A/PDE4B
CCNA2/CDK2
MAPK14/MTOR
PIK3CB/TNK2
CRHR1/REN
MAPK14/AKT1/MAPK1
SYK/CSF1R/APP
PIK3CA/SYK/IL6ST/AKT1/PDPK1
PDE5A/PDE10A/PSEN1/APP/PDE4B/P2RX7/FASN
CACNA2D1/F2/P2RX7/PDPK1
IKBKB/FASN/ICAM1
JAK2/APP/P2RX7
PIK3CB/PIK3CG/SYK
EGFR/MTNR1B/ADORA2A
MAPKAPK2/MAPK14/MTOR
AKT2/MAPK14/PIK3CA/AKT1/MTOR
JAK2/PSEN1/SMO/MAPK8
AKT2/MAPK14/AKT1
MDM2/EGFR/AKT1

EGFR/AKT1/SMO/MTOR/PDPK1
CYP2D6/CYP3A4/CYP2C19/CYP2C9
MDM2/CDK2/PRKDC/PLK3
NTRK1/TRPV1
MAPK9/PLA2G2A
CRHR1/REN
MTNR1B/ADORA2A
AKT2/AKT1
PDE5A/PDE10A
CRHR1/DRD2
SYK/P2RX7
XIAP/APP
ITGB2/ICAM1
PIK3CA/PTK2
CYP19A1/JAK2/EGFR/DRD2
MAPK11/MAPK14/S1PR1/SMO/MAPK1/PIM1/MTOR
MAPK11/MAPK14/S1PR1/SMO/MAPK1/PIM1/MTOR
SYK/F2/P2RX7
CRHR1/ADORA2A/DRD2
CYP2D6/CYP19A1/CYP3A4/CYP2C9
AKT1/IKBKB
EGFR/SYK
EGFR/SYK
AKT2/AKT1
TRPV1/P2RX7
AKT2/AKT1
EGFR/AKT1/PDPK1
PDE5A/PDE10A/PSEN1/APP/PDE4B/P2RX7/FASN
PDE5A/IL6ST/PTK2/MTOR
MDM2/CDK2/PRKDC/PLK3
CRHR1/ADORA2A/DRD2
S1PR1/IKBKB/FASN/ICAM1
NCSTN/AKT2/AKT1/MTOR
MDM2/CDK2/MAPK9/AKT1/PSEN1/PLK3/PLK1
PIK3CB/PIK3CG/PIK3CA/PIK3CD/PLA2G2A
PDE5A/PDE10A/PDE4B
CNR2/CACNA2D1/MTNR1B/KCNH2
CHRM1/CHRM2
NTRK1/TRPV1
SYK/P2RX7
PPP1CC/MTOR
AKT1/MAPK1
CCNA2/CDK2
CCNA2/CDK2
CRHR1/REN
EGFR/SYK/P2RX7/MTOR/PDPK1
NTRK1/MAPK14/APP/PTK2/DRD2

TRPV1/AKT1/F2/P2RX7/DRD2/PDPK1
NCSTN/AKT2/AKT1/MTOR
NCSTN/AKT2/AKT1/MTOR
TRPV1/MDM2/PSEN1/APP/PTK2/F2
CRHR1/DRD2/ICAM1
PDE5A/PDE10A/PSEN1/APP/PDE4B/P2RX7/FASN
XIAP/MAPK9/PLK1/AVPR2
JAK2/XIAP/MDM2/SYK/AKT1/APP/ADORA2A
ITGB2/CRHR1/SYK/P2RX7
PIK3CB/PIK3CG/PIK3CA/CSF1R/PIK3CD/PLA2G2A
CRHR1/VDR/AKT1/MAPK1/MTOR
MAPKAPK2/TRPV1/MAPK1/MTOR
SMO/PDPK1
SYK/ALOX5
CYP2C19/CYP2C9
CRHR1/SYK
ITGB2/HTR1A
ITGB2/HTR1A
CRHR1/DRD2
PSEN1/DRD2
NTRK1/ICAM1
JAK2/PPP2CA/IKBKB/PPP1CC/MTOR/DRD2
PIK3CB/PIK3CG/PIK3CA/CSF1R/PTK2/PIK3CD/PLA2G2A
MAPKAPK2/MAPK14/AKT1
AKT2/MAPK14/AKT1
JAK2/AKT1/P2RX7/PDPK1/ICAM1
PDE5A/PDE10A/PSEN1/APP/PDE4B/P2RX7/FASN
IL6ST/AKT1/SMO/PTK2/FASN
PIK3CB/ITGB2/ITGAL/PTK2/PDPK1
PTGER1/CRHR1/ADORA2A/DRD2
CHRM1/PTGER1/TRPV1/VDR
EGFR/PIK3CA/AKT1/APP
JAK2/IKBKB/MTOR/DRD2
CYP2D6/CYP3A4/CYP2C19/CYP2C9
NTRK1/MAPK14/APP/PTK2/DRD2
JAK2/CRHR1/MDM2/JAK1/DRD2/ICAM1
NPY5R/ICAM1
MDM2/SMO
JAK2/MTOR
NTRK1/TRPV1
PIK3CG/PIK3CA
CYP2D6/CYP3A4/CYP2C9
SMO/PIM1/DRD2
SYK/PRKDC/IKBKB/PDPK1
AKT2/AKT1/MTOR
JAK2/IL6ST/CSF1R
PDE5A/PDE10A/PDE4B

CACNA2D1/F2/PDPK1
NCSTN/CRHR1/APP/DRD2
CACNA2D1/F2/KCNH2/PDPK1
AKT2/AKT1/MTOR
NPY5R/CNR2/DRD2
SMO/DRD2
PIK3CB/PIK3CD
MTNR1A/APP
MAPK14/MAPK8
PSEN1/APP
CRHR1/REN
VDR/CYP3A4
PDE10A/PDE4B
MAPKAPK2/MAPK14
ITGB2/SYK
CRHR1/AKT1/SMO/DRD2/ICAM1
CRHR1/APP/MTOR/DRD2
AKT2/PPP2CA/AKT1/MAPK1/P2RX7/PLK3/PLK1
ITGB2/EGFR/SYK
IL6ST/F2/MTOR
MAPKAPK2/ITGB2/IKBKB/PDPK1
MDM2/MAPK14/PRKDC/ICAM1
IL6ST/PPP1CC/MTOR
CACNA2D1/PDE4B/KCNH2
MDM2/AKT2/CDK2/PLK3/MTOR
EGFR/SYK/AKT1/APP/MAPK1
CYP2D6/CYP3A4/CYP2C9/ALOX5/FASN
NTRK1/TRPV1
VDR/PIM1
CRHR1/NPY5R/MTNR1B/DRD2/AVPR2
CRHR1/SMO/DRD2
EGFR/SYK/AKT1/APP/MAPK1
NTRK1/MAPK10/EGFR/AKT1/MTOR/ICAM1
TRPV1/MDM2/PSEN1/APP/PTK2/F2
SYK/DRD2
VDR/CYP3A4
PPP2CA/APP
PSEN1/DRD2
PSEN1/SMO
PIK3CG/PIK3CA
VDR/MAPK1
JAK2/PTK2
PIK3CA/PTK2
MAPKAPK2/SYK/APP/P2RX7
PSEN1/APP/P2RX7
AKT1/PSEN1/MTOR
NTRK1/JAK2/DRD2

CRHR1/ADORA2A/DRD2
AKT2/MAPK14/PIK3CA/AKT1/MTOR
AKT2/AKT1/MTOR
AKT2/MAPK14/AKT1
AKT1/SMO/PDPK1
CRHR1/MTNR1B/DRD2
ADORA2A/DRD2/CHRM2
ITGB2/PIK3CA/AKT1/SMO/PTK2/MTOR/PDPK1
XIAP/MDM2/TNK2/AKT1/APP/IKBKB/ADORA2A
PDE5A/PDE10A/PSEN1/APP/PDE4B/P2RX7/FASN
MAPKAPK2/MAPK1/MTOR
MAPKAPK2/MAPK14/MTOR
SMO/PDPK1
SMO/PDPK1
PDE10A/PDE3B
JAK2/JAK1
JAK2/JAK1
JAK2/PTK2
ITGB2/PIK3CA/AKT1/PTK2/MTOR/PDPK1
MDM2/CDK2/PRKDC/PLK3/PLK1
TRPV1/CACNA2D1/PSEN2
PIK3CB/JAK2/ITGB2/ITGAL/PTK2/PDPK1
PSEN1/PTK2/MARK2/DRD2
XIAP/MDM2/AKT1/APP/ADORA2A
JAK2/CRHR1/EGFR/MTNR1B/DRD2
CYP2D6/SYK/CYP3A4/CYP2C9/ALOX5/FASN
ITGB2/PIK3CA/AKT1/PTK2/MTOR/PDPK1
PIK3CB/PIK3CG/PIK3CA/PIK3CD/PLA2G2A
CYP2D6/SYK/CYP3A4/CYP2C9/ALOX5/FASN
PIK3CB/PIK3CG/SYK
VDR/SMO/PIM1
LSS/VDR/FASN
PRKDC/MTOR
CYP19A1/MAPK14
AKT2/AKT1
AKT2/AKT1
HTR1A/CRHR1
APP/DRD2
SYK/CSF1R/APP
PDE5A/PIK3CG/PDE4B
MDM2/CDK2/PLK3
MDM2/CDK2/CHEK1/PRKDC/PLK3/PLK1
ITGB2/PIK3CA/AKT1/PTK2/MTOR/PDPK1
MAPKAPK2/SYK/APP/P2RX7
LSS/CYP3A4/APP/FASN
MDM2/CDK2/MAPK9/AKT1/PSEN1/PLK3/PLK1
CCNA2/EGFR/PRKDC

PIK3CB/PIK3CD/P2RX7
ADORA2A/DRD2
CYP19A1/CYP3A4
PSEN1/APP
SMO/PDPK1
PDE5A/PIK3CG
PSEN1/F2
CRHR1/MTOR
CACNA2D1/KCNH2
XIAP/EGFR/PPP2CA/MAPK14/PSEN1/APP
NTRK1/AKT2/TNK2/S1PR1/PTK2/MTOR/ICAM1
CCNA2/EGFR/PRKDC
SYK/PSEN1/F2
CRHR1/REN/AVPR2
MDM2/EGFR/AKT1/APP
AKT1/P2RX7/DRD2
PIK3CB/PIK3CG/PIK3CA/PIK3CD/PLA2G2A
AKT2/EGFR/AKT1/IKBKB/PDPK1
ITGB2/PSEN1/CSF1R/PTK2/F2/MARK2/ICAM1
PDE5A/PIK3CA/SYK/IL6ST/AKT1/ADORA2A/PDPK1
MAPK10/PDPK1
CHRM1/ADORA2A
CYP19A1/CYP3A4
PIK3CG/PIK3CD
CCNA2/ICAM1
SYK/APP
F2/DRD2
CHRM1/CHRM2
ITGB2/ICAM1
AKT2/AKT1/MTOR
CYP19A1/VDR/CYP3A4
CYP19A1/LSS/APP/FASN
XIAP/MDM2/AKT1/APP/ADORA2A
CYP2D6/LSS/VDR/CYP3A4/APP/FASN
EGFR/AKT1/MAPK1
PSEN1/APP/P2RX7
SYK/F2/P2RX7/ICAM1
EGFR/PDE5A
AKT2/AKT1
PIK3CB/PIK3CD
SYK/APP
EGFR/AKT1/F2/MTOR
ITGB2/CRHR1/SYK
PSEN1/CSF1R/APP
TRPV1/CACNA2D1/KCNH2
TRPV1/CACNA2D1/KCNH2
AKT1/PSEN1/MTOR

CYP19A1/LSS/VDR/CYP3A4/FASN
CRHR1/MTNR1B/DRD2
PLK3/PLK1/DRD2
SYK/CSF1R/APP
PSEN1/SMO/PRKDC
PIK3CA/AKT1/MAPK8/MTOR
JAK2/APP/P2RX7
AKT2/AKT1/MTOR
MAPK9/PLA2G2A
MTNR1A/APP
SMO/P2RX7
EGFR/ICAM1
ITGB2/AKT2
PTK2/IKBKB
CHRM1/CHRM2
CHRM1/CHRM2
NTRK1/MDM2/AKT1/PSEN1/PTK2/MARK2/MTOR
JAK2/TRPV1/SYK/DRD2
JAK2/JAK1/ICAM1
CCNA2/EGFR/CDK2/PPP2CA/CHEK1
PIK3CB/ITGB2/ITGAL/MAPK14/ICAM1
JAK2/IKBKB/MTOR/DRD2
LSS/VDR/CYP3A4/FASN
PIK3CG/AKT1/PDE3B
ITGB2/PIK3CA/AKT1/PTK2/PDPK1
ADORA2A/DRD2
SYK/F2
CHEK1/PLK1
VDR/AKT1
TRPV1/P2RX7
SMO/DRD2
EGFR/AKT1
IL6ST/F2
CACNA2D1/KCNH2
PDE5A/PDE4B
CACNA2D1/KCNH2
PPP2CA/PSEN1/APP
EGFR/AKT1/ICAM1
JAK2/SYK/JAK1/IKBKB
NTRK1/APP/PTK2/DRD2
MAPKAPK2/MAPK14/AKT1/MTOR
MTOR/DRD2
P2RX7/ADORA2A
EGFR/PIK3CA
AKT1/MAPK1
CACNA2D1/KCNH2
JAK2/PPP2CA/PSEN1/APP

JAK2/MAPK9/MAPK8/ADORA2A
MDM2/PPP2CA/SMO/MAPK1/MTOR
LSS/VDR/FASN
EGFR/AKT1/PDPK1
ITGAL/PSEN1/MTOR/ICAM1
AKT2/AKT1/MTOR
SYK/APP/P2RX7
JAK2/EGFR/DRD2
AKT2/IL6ST/AKT1/PPP1CC/MTOR
MDM2/DRD2
XIAP/MDM2
PDE10A/MTNR1B
PIK3CB/PIK3CD
PSEN1/SMO
VDR/CSF1R
MTNR1B/KCNH2
SMO/DRD2
XIAP/MDM2
PDPK1/ICAM1
TRPV1/REN/DRD2/AVPR2
PDE5A/PIK3CG/PDE4B
MAPK14/CSF1R/MTOR
PIK3CG/APP/REN
MAPKAPK2/MAPK14/AKT1/MTOR
SYK/P2RX7/ICAM1
JAK2/MTOR
CYP19A1/NPY5R/CNR2/F2/ADORA2A/DRD2
EGFR/SYK/AKT1/APP
AKT2/MAPK14/PIK3CA/AKT1/MTOR
MDM2/SMO
PSEN1/ADORA2A
EGFR/AKT1
SMO/MTOR
AKT2/ABCB1/AKT1/PSEN1/MTOR
S1PR1/CSF1R/CDC42BPA
JAK2/APP/P2RX7
NTRK1/MAPK14/PSEN1/APP/PTK2/DRD2
JAK2/SYK/JAK1/IKBKB
F2/P2RX7/PDPK1
NCSTN/PSEN1/SMO
NTRK1/APP/PTK2/IKBKB/DRD2/PDPK1
MAPK1/MTOR
MAPK9/PLA2G2A
CSF1R/FASN
EGFR/CDK2
CCNA2/EGFR
MAPK9/PLA2G2A

PTK2/F2
ITGB2/AKT2
CRHR1/DRD2
CSF1R/FASN
PIK3CB/AKT1/MTOR/PDPK1
MDM2/AKT2/CHEK1/MAPK14
PIK3CA/AKT1/PSEN1/MAPK8/MTOR
MDM2/PSEN1/APP/PTK2/F2
ITGB2/HTR1A/DRD2
JAK2/APP/P2RX7
NTRK1/APP/PTK2/IKBKB
ADORA2A/DRD2/CHRM2
PIK3CB/PIK3CG/PIK3CA/CSF1R/PIK3CD/PLA2G2A
AKT2/AKT1
AKT1/PIM1
VDR/PIM1
AKT1/P2RX7
AKT1/P2RX7
PIK3CG/PIK3CD
CACNA2D1/KCNH2
AKT2/AKT1/P2RX7
EGFR/IL6ST/AKT1
PDE5A/PDE10A/PDE4B
MTNR1B/KCNH2/DRD2
AKT1/SMO/MTOR
NTRK1/APP/PTK2
PSEN1/ADORA2A/DRD2
CRHR1/AVPR2
JAK2/PDPK1
MAPK14/MTOR
AKT2/AKT1
AKT1/MTOR
MTNR1B/DRD2
CYP19A1/MAPK14
CACNA2D1/KCNH2
JAK2/MAPK14/SYK/APP
F2/P2RX7/ICAM1
MDM2/CDK2/PLK3
APP/ADORA2A/DRD2
PDE5A/CNR2/F2/ADORA2A
MAPKAPK2/MAPK14/AKT1/MTOR
AKT2/AKT1/MTOR
AKT2/MAPK14/AKT1
CACNA2D1/KCNH2
CYP19A1/MAPK14
SMO/DRD2
SMO/PIM1

CRHR1/SMO/DRD2
MAPK11/MAPK14/MTOR
CYP2D6/EGFR/CYP3A4
REN/AVPR2
MTNR1A/APP
F2/P2RX7
PSEN1/SMO
EGFR/PSEN1
HTR1A/CRHR1
PTGER1/TRPV1
MAPK11/PTK2
F2/PDPK1
EGFR/ICAM1
PIK3CB/TNK2
NCSTN/PSEN1/SMO
ITGB2/SYK/DRD2
S1PR1/CACNA2D1/MTNR1B/KCNH2
PSEN1/APP/MTOR/ICAM1
AKT1/MTOR/PDPK1
AKT2/MAPK14/AKT1
AKT2/AKT1/MTOR
MAPK14/SMO/MTOR
ADORA2A/DRD2/CHRM2
PIK3CB/TNK2/SYK/DRD2
JAK2/PDPK1
CYP2D6/CYP3A4
MTNR1B/KCNH2
MAPK14/MTOR
PIK3CB/ITGB2/TNK2/SYK/DRD2
SYK/IKBKB/PDPK1
PSEN1/APP/P2RX7
EGFR/PSEN1/SMO
EGFR/MTOR/ICAM1
MAPK11/APP/IKBKB/ICAM1
PSEN1/APP/P2RX7
AKT2/MAPK14/AKT1
JAK2/EGFR/MTNR1B/DRD2
AKT1/MTOR
APP/ICAM1
CACNA2D1/KCNH2
JAK2/IKBKB/MTOR/DRD2
JAK2/APP/P2RX7
PSEN1/APP/P2RX7
SYK/IKBKB/PDPK1
AKT2/MAPK14/AKT1
VDR/CYP3A4
JAK2/AKT1

ABCB1/P2RX7
IL6ST/MTOR
EGFR/AKT1
CRHR1/DRD2
CRHR1/NPY5R/MTNR1B/DRD2
SYK/IKBKB/PDPK1
PSEN1/APP/P2RX7
CDK2/CHEK1/APP/PLK1
SYK/PRKDC/IKBKB/PDPK1
MDM2/EGFR/CHEK1/PRKDC
CSF1R/APP
ABCB1/P2RX7
JAK2/MTOR
CRHR1/REN
PSEN1/MTOR
ITGB2/ICAM1
NTRK1/JAK2/CCNA2/CDK2/MAPK11/MAPK14
EGFR/VDR/CSF1R
MTNR1B/DRD2
NTRK1/CYP19A1/REN/ICAM1
AKT2/PIK3CG/AKT1/MTOR
CACNA2D1/PDE4B/KCNH2
PSEN1/APP/P2RX7
EGFR/CHEK1/PRKDC
PSEN1/APP/P2RX7
PSEN1/DRD2
SMO/PDPK1
NPY5R/ICAM1
ITGAL/ICAM1
AKT2/AKT1
TRPV1/DRD2
VDR/CSF1R
AKT1/MAPK1
MAPK14/F2
MAPK10/ITGB2/AKT2/EGFR/MAPK8
PPP2CA/SMO/MAPK1/MTOR
PIK3CA/SYK/IL6ST/AKT1/PDPK1

Count
18
18
18
17
19
19
19
18
18
6
18
15
13
14
17
15
15
10
12
12
13
17
14
11
14
15
15
10
15
16
6
8
15
10
10
16
12
9
10
12
14
13
9
16
12

15
14
9
9
7
8
12
12
9
9
14
8
8
8
12
12
10
7
7
12
14
14
14
14
14
8
9
10
12
10
12
11
12
5
12
10
10
7
6
12
7
14
6
9
8
14
7

6
6
8
12
11
12
10
8
12
10
11
13
12
9
13
12
5
6
5
12
10
10
11
13
10
11
7
6
10
11
13
7
11
7
8
12
6
12
4
7
6
6
5
5
5
10
10

7
11
11
11
9
8
8
9
6
9
9
6
5
10
11
11
11
9
5
6
6
7
8
8
12
10
8
6
10
8
9
7
7
6
9
9
10
12
7
5
7
9
6
9
7
6
8

5
4
5
5
6
5
5
7
6
9
5
5
10
12
10
6
9
4
10
6
10
7
7
7
5
4
9
9
11
8
7
7
5
6
7
7
5
10
11
9
7
5
6
9
6
4
7

4
6
6
7
7
6
9
4
7
7
7
7
6
6
6
10
4
6
7
6
5
5
10
4
4
5
6
6
9
10
10
9
9
4
7
5
6
6
5
4
4
4
4
5
6
11
3

3
10
9
5
7
7
7
4
11
5
7
7
6
6
4
4
4
10
7
7
5
6
6
6
5
10
6
8
9
9
9
5
5
5
4
4
5
5
7
3
7
5
8
8
6
4
4

4
8
8
10
7
5
5
10
7
4
5
10
3
3
5
4
8
5
4
4
9
5
5
7
7
6
6
6
4
4
6
5
5
6
4
3
3
3
5
9
6
7
7
9
5
4
4

4
7
4
4
4
4
5
5
7
7
7
3
3
10
8
6
7
4
10
7
7
10
10
6
7
6
4
4
4
4
7
5
8
3
3
6
4
4
6
5
5
4
4
4
4
8
3

3
9
8
6
4
7
10
10
8
5
3
4
5
10
6
6
5
8
4
7
5
3
3
3
4
5
5
6
3
3
4
4
4
4
4
4
7
5
3
3
3
8
6
4
4
4
5

9
3
3
4
7
5
5
6
8
4
3
5
8
7
4
4
4
6
6
6
6
7
4
4
4
5
4
6
7
5
5
7
4
7
3
7
4
5
6
5
5
5
3
3
9
5
7

6
3
3
3
3
5
6
7
4
7
3
6
5
6
4
6
6
9
3
3
3
3
3
3
3
8
8
5
6
6
4
3
3
3
3
3
6
5
8
6
6
3
3
3
3
3
3

5
5
4
4
7
7
8
6
4
5
5
5
6
9
3
3
3
3
6
5
8
6
6
8
3
3
3
3
3
8
5
5
5
5
8
4
3
3
4
4
7
5
3
3
4
4
6

8
7
5
4
4
3
3
3
4
4
5
6
6
6
5
3
3
4
4
6
5
7
3
4
4
4
6
6
5
6
4
4
5
3
4
4
4
2
2
2
2
2
2
2
2
2
2

2
2
2
8
6
5
5
8
7
5
6
5
3
3
3
3
3
6
8
4
3
3
3
3
5
2
2
2
5
5
4
4
5
5
3
3
3
8
5
4
7
4
4
6
3
3
3

3
3
3
3
3
3
3
4
2
2
2
2
5
7
4
4
3
4
4
4
4
4
5
3
6
4
4
2
2
2
2
2
2
4
4
3
3
8
5
4
6
3
3
3
3
3
4

4
6
4
3
3
3
3
2
2
2
2
2
2
2
2
2
2
2
8
7
3
3
8
7
4
5
5
5
3
3
3
3
3
4
5
4
3
3
6
2
2
2
2
2
2
2
2

2
2
2
2
2
2
4
4
4
6
5
5
8
3
3
3
3
3
5
7
6
5
3
4
2
2
2
2
2
2
2
2
2
3
3
5
7
4
3
3
3
3
3
5
4
3
3

5
4
4
2
2
2
2
2
2
2
2
2
2
2
4
7
7
3
3
4
2
2
2
2
2
2
3
7
4
4
3
4
4
7
5
3
4
2
2
2
2
2
2
2
2
5
5

6
4
4
6
3
7
4
7
4
6
5
4
2
2
2
2
2
2
2
2
2
6
7
3
3
5
7
5
5
4
4
4
4
4
5
6
2
2
2
2
2
3
3
4
3
3
3

3
4
4
3
3
2
2
2
2
2
2
2
2
2
2
5
4
7
3
3
4
4
3
3
5
5
5
2
2
5
3
5
6
6
2
2
2
2
2
2
2
2
2
4
3
3
3

3
5
3
3
3
3
3
7
7
7
3
3
2
2
2
2
2
2
6
5
3
6
4
5
5
6
6
5
6
3
3
3
2
2
2
2
2
2
3
3
3
6
6
4
4
7
3

3
2
2
2
2
2
2
2
2
6
7
3
3
3
4
3
5
5
7
7
2
2
2
2
2
2
2
2
2
3
3
4
5
6
3
3
4
2
2
2
2
4
3
3
3
3
3

5
3
3
3
3
4
3
3
2
2
2
2
2
2
2
2
7
4
3
5
5
4
4
3
5
2
2
2
2
2
2
2
2
2
2
2
3
3
4
4
4
2
2
2
2
2
4

4
5
3
3
4
3
3
3
5
2
2
2
2
2
2
2
2
2
2
4
3
3
3
4
3
2
6
4
5
2
2
2
2
5
3
3
6
4
3
3
6
2
2
2
2
2
2

2
2
2
2
4
4
5
5
3
3
4
3
6
2
2
2
2
2
2
2
3
3
3
3
3
3
3
2
2
2
2
2
2
2
2
4
3
3
3
4
4
3
3
2
2
2
2

3
3
3
2
2
2
2
2
2
2
2
2
2
2
3
3
4
4
3
3
3
3
3
4
2
2
2
2
5
3
3
3
3
4
3
3
4
2
2
2
4
3
3
3
3
2
2

2
2
2
2
4
3
3
4
4
4
2
2
2
2
2
2
6
3
2
4
4
3
3
3
3
2
2
2
2
2
2
2
2
2
5
4
5

Table. S4 The results of the GO and KEGG pathway enrichment

Table. S4-2 The results of cellular component in GO analysis

ID	Description	GeneRatio
GO:0043025	neuronal cell body	18/87
GO:0099056	integral component of presynaptic membrane	8/87
GO:0098889	intrinsic component of presynaptic membrane	8/87
GO:0045121	membrane raft	11/87
GO:0098857	membrane microdomain	11/87
GO:0098589	membrane region	11/87
GO:0099699	integral component of synaptic membrane	8/87
GO:0042734	presynaptic membrane	8/87
GO:0099240	intrinsic component of synaptic membrane	8/87
GO:0098793	presynapse	12/87
GO:0043204	perikaryon	7/87
GO:0005942	phosphatidylinositol 3-kinase complex	4/87
GO:0005925	focal adhesion	10/87
GO:0030055	cell-substrate junction	10/87
GO:0061695	transferase complex, transferring phosphorus-containing groups	8/87
GO:0008021	synaptic vesicle	7/87
GO:0098978	glutamatergic synapse	9/87
GO:0005769	early endosome	9/87
GO:0070382	exocytic vesicle	7/87
GO:0097060	synaptic membrane	9/87
GO:0098687	chromosomal region	8/87
GO:0030133	transport vesicle	8/87
GO:0032279	asymmetric synapse	7/87
GO:1902554	serine/threonine protein kinase complex	4/87
GO:0043197	dendritic spine	5/87
GO:0044309	neuron spine	5/87
GO:0000307	cyclin-dependent protein kinase holoenzyme complex	3/87
GO:0035253	ciliary rootlet	2/87
GO:0098981	cholinergic synapse	2/87
GO:0005819	spindle	7/87
GO:0098984	neuron to neuron synapse	7/87
GO:0044853	plasma membrane raft	4/87
GO:1902911	protein kinase complex	4/87
GO:0045177	apical part of cell	7/87
GO:0000775	chromosome, centromeric region	5/87
GO:0009897	external side of plasma membrane	7/87
GO:0099055	integral component of postsynaptic membrane	4/87
GO:0031252	cell leading edge	7/87
GO:0098936	intrinsic component of postsynaptic membrane	4/87
GO:0000776	kinetochore	4/87
GO:0014069	postsynaptic density	6/87
GO:0042629	mast cell granule	2/87
GO:0099572	postsynaptic specialization	6/87
GO:0031594	neuromuscular junction	3/87
GO:0010008	endosome membrane	7/87

GO:0000781	chromosome, telomeric region	4/87
GO:0005901	caveola	3/87
GO:0000922	spindle pole	4/87
GO:0008305	integrin complex	2/87
GO:0150034	distal axon	5/87
GO:0030135	coated vesicle	5/87
GO:0030136	clathrin-coated vesicle	4/87
GO:0005790	smooth endoplasmic reticulum	2/87
GO:0031904	endosome lumen	2/87
GO:0051233	spindle midzone	2/87
GO:0098636	protein complex involved in cell adhesion	2/87
GO:0019898	extrinsic component of membrane	5/87
GO:0098802	plasma membrane signaling receptor complex	5/87
GO:0031965	nuclear membrane	5/87
GO:0005911	cell-cell junction	6/87
GO:0030027	lamellipodium	4/87
GO:0030139	endocytic vesicle	5/87
GO:0043198	dendritic shaft	2/87

BgRatio	pvalue	p.adjust	qvalue
497/19717	4.80E-12	1.23E-09	7.54E-10
73/19717	1.10E-09	1.40E-07	8.63E-08
82/19717	2.83E-09	2.40E-07	1.48E-07
315/19717	1.38E-07	7.28E-06	4.48E-06
316/19717	1.43E-07	7.28E-06	4.48E-06
328/19717	2.08E-07	8.82E-06	5.42E-06
152/19717	3.62E-07	1.32E-05	8.11E-06
161/19717	5.61E-07	1.79E-05	1.10E-05
164/19717	6.46E-07	1.83E-05	1.13E-05
491/19717	1.62E-06	4.14E-05	2.54E-05
134/19717	2.13E-06	4.93E-05	3.03E-05
27/19717	5.74E-06	0.000122027	7.51E-05
405/19717	1.17E-05	0.000229668	0.000141261
412/19717	1.36E-05	0.000247342	0.000152132
259/19717	1.90E-05	0.000322848	0.000198573
191/19717	2.18E-05	0.000339645	0.000208905
349/19717	2.34E-05	0.000339645	0.000208905
350/19717	2.40E-05	0.000339645	0.000208905
207/19717	3.65E-05	0.000489453	0.000301046
432/19717	0.0001213	0.001546334	0.000951099
347/19717	0.0001481	0.001798789	0.001106376
392/19717	0.0003379	0.00391645	0.002408879
328/19717	0.000619	0.00660629	0.004063312
88/19717	0.0006218	0.00660629	0.004063312
159/19717	0.0006948	0.007087023	0.004358994
161/19717	0.0007351	0.007209965	0.004434612
42/19717	0.0008412	0.007322744	0.004503979
10/19717	0.0008464	0.007322744	0.004503979
10/19717	0.0008464	0.007322744	0.004503979
347/19717	0.0008615	0.007322744	0.004503979
350/19717	0.0009058	0.007451222	0.004583001
109/19717	0.0013829	0.010685806	0.006572487
109/19717	0.0013829	0.010685806	0.006572487
384/19717	0.0015455	0.011591447	0.007129517
193/19717	0.0016476	0.012003691	0.007383075
393/19717	0.001763	0.012366256	0.007606077
117/19717	0.0017943	0.012366256	0.007606077
403/19717	0.0020319	0.013635264	0.008386602
122/19717	0.0020907	0.013669895	0.008407902
135/19717	0.0030164	0.019229319	0.011827321
324/19717	0.0031124	0.019357889	0.0119064
22/19717	0.004198	0.025488029	0.015676847
348/19717	0.0044098	0.026151299	0.016084803
76/19717	0.004625	0.026803802	0.016486136
479/19717	0.0052536	0.028958146	0.017811202

158/19717	0.0052777	0.028958146	0.017811202
80/19717	0.0053374	0.028958146	0.017811202
164/19717	0.006014	0.031949522	0.019651099
31/19717	0.0082361	0.042861529	0.026362715
285/19717	0.0085914	0.043281231	0.02662086
289/19717	0.0090914	0.043281231	0.02662086
188/19717	0.0096377	0.043281231	0.02662086
34/19717	0.0098519	0.043281231	0.02662086
34/19717	0.0098519	0.043281231	0.02662086
34/19717	0.0098519	0.043281231	0.02662086
34/19717	0.0098519	0.043281231	0.02662086
295/19717	0.0098785	0.043281231	0.02662086
295/19717	0.0098785	0.043281231	0.02662086
296/19717	0.0100141	0.043281231	0.02662086
416/19717	0.010234	0.043494431	0.026751993
193/19717	0.0105381	0.044052519	0.027095254
303/19717	0.0109994	0.044527025	0.027387107
36/19717	0.0110008	0.044527025	0.027387107

geneID
NTRK1/MAPK10/TRPV1/CRHR1/PDE10A/CNR2/IL6ST/PSEN1/APP/PSEN2/MAPK1/P2RX7/PLK3/ADORA2
CHRM1/NCSTN/APH1A/PSEN1/PSEN2/ADORA2A/DRD2/CHRM2
CHRM1/NCSTN/APH1A/PSEN1/PSEN2/ADORA2A/DRD2/CHRM2
JAK2/ITGB2/EGFR/PPP2CA/S1PR1/PSEN1/APP/SMO/MAPK1/IKBKB/ICAM1
JAK2/ITGB2/EGFR/PPP2CA/S1PR1/PSEN1/APP/SMO/MAPK1/IKBKB/ICAM1
JAK2/ITGB2/EGFR/PPP2CA/S1PR1/PSEN1/APP/SMO/MAPK1/IKBKB/ICAM1
CHRM1/NCSTN/APH1A/PSEN1/PSEN2/ADORA2A/DRD2/CHRM2
CHRM1/NCSTN/APH1A/PSEN1/PSEN2/ADORA2A/DRD2/CHRM2
CHRM1/NCSTN/APH1A/PSEN1/PSEN2/ADORA2A/DRD2/CHRM2
CHRM1/NCSTN/APH1A/PSEN1/APP/PSEN2/PDE4B/P2RX7/ADORA2A/PPP1CC/DRD2/CHRM2
MAPK10/PDE10A/CNR2/APP/MAPK1/DRD2/PDPK1
PIK3CB/PIK3CG/PIK3CA/PIK3CD
JAK2/NCSTN/ITGB2/EGFR/JAK1/PTK2/MAPK1/PPP1CC/PDPK1/ICAM1
JAK2/NCSTN/ITGB2/EGFR/JAK1/PTK2/MAPK1/PPP1CC/PDPK1/ICAM1
PIK3CB/CCNA2/CDK2/CCNA1/PIK3CG/PIK3CA/PIK3CD/IKBKB
NCSTN/APH1A/PSEN1/APP/PSEN2/PDE4B/DRD2
MAPK10/CHRM1/JAK2/MAPK14/ADORA2A/PPP1CC/MTOR/DRD2/CHRM2
NTRK1/NCSTN/AKT2/EGFR/APH1A/PSEN1/APP/PSEN2/MAPK1
NCSTN/APH1A/PSEN1/APP/PSEN2/PDE4B/DRD2
CHRM1/NCSTN/TRPV1/APH1A/PSEN1/PSEN2/ADORA2A/DRD2/CHRM2
CDK2/PPP2CA/CHEK1/PSEN1/PRKDC/PSEN2/PLK1/PPP1CC
NCSTN/APH1A/PSEN1/APP/PSEN2/PDE4B/APH1B/DRD2
CHRM1/MAPK1/PDE4B/ADORA2A/DRD2/PDPK1/CHRM2
CCNA2/CDK2/CCNA1/IKBKB
APP/PTK2/PDE4B/PPP1CC/DRD2
APP/PTK2/PDE4B/PPP1CC/DRD2
CCNA2/CDK2/CCNA1
PSEN1/APP
CHRM1/CHRM2
PPP2CA/MAPK14/AKT1/APP/MAPK1/PLK3/PLK1
CHRM1/MAPK1/PDE4B/ADORA2A/DRD2/PDPK1/CHRM2
JAK2/ITGB2/SMO/MAPK1
CCNA2/CDK2/CCNA1/IKBKB
CRHR1/EGFR/ABCB1/APP/PTK2/PSEN2/REN
PPP2CA/PSEN1/PSEN2/PLK1/PPP1CC
ITGB2/TRPV1/S1PR1/IL6ST/F2/P2RX7/ICAM1
CHRM1/ADORA2A/DRD2/CHRM2
TRPV1/AKT2/PIK3CA/APP/PTK2/ADORA2A/CDC42BPA
CHRM1/ADORA2A/DRD2/CHRM2
PSEN1/PSEN2/PLK1/PPP1CC
CHRM1/MAPK1/PDE4B/ADORA2A/DRD2/PDPK1
PIK3CG/PIK3CD
CHRM1/MAPK1/PDE4B/ADORA2A/DRD2/PDPK1
PSEN1/APP/P2RX7
NTRK1/NCSTN/EGFR/APH1A/PSEN1/APH1B/PSENEN

CDK2/CHEK1/PRKDC/PPP1CC
JAK2/SMO/MAPK1
PPP2CA/MAPK14/PLK3/PLK1
ITGB2/ITGAL
CHRM1/PSEN1/APP/DRD2/CHRM2
EGFR/TNK2/APP/AVPR2/CHRM2
EGFR/TNK2/AVPR2/CHRM2
PSEN1/APP
JAK2/APP
APP/PLK1
ITGB2/ITGAL
PIK3CB/CNR2/PIK3CG/PIK3CA/PIK3CD
ITGB2/ITGAL/SYK/IL6ST/IKBKB
EGFR/PSEN1/PSEN2/P2RX7/ALOX5
TNK2/AKT1/APP/PTK2/P2RX7/CDC42BPA
PIK3CA/APP/PTK2/CDC42BPA
MDM2/EGFR/SYK/SMO/DRD2
PSEN1/APP

Count
18
8
8
11
11
11
8
8
8
12
7
4
10
10
8
7
9
9
7
9
8
8
7
4
5
5
3
2
2
7
7
4
4
7
5
7
4
7
4
4
6
2
6
3
7

4
3
4
2
5
5
4
2
2
2
2
5
5
5
6
4
5
2

Table. S4 The res
Table. S4-3 The r

**ID**
GO:0004674
GO:0004707
GO:0004712
GO:0016303
GO:0035004
GO:0004713
GO:0019902
GO:0016712
GO:0016307
GO:0052742
GO:0008395
GO:0004715
GO:0019903
GO:0004114
GO:0042562
GO:0004112
GO:0043560
GO:0020037
GO:0004708
GO:0035173
GO:0046906
GO:0008081
GO:0001653
GO:0005506
GO:0004497
GO:0008227
GO:0004190
GO:0070001
GO:0004993
GO:0099589
GO:0019825
GO:0030551
GO:0042169
GO:0051219
GO:0008528
GO:0099528
GO:0016705
GO:0004115
GO:0017046
GO:0016725
GO:0008188
GO:0042578
GO:0030553
GO:0030594
GO:0008392

GO:0097110
GO:0008391
GO:0050839
GO:0047555
GO:0004714
GO:0005178
GO:0070851
GO:0098960
GO:0004175
GO:0015464
GO:0030552
GO:0019199
GO:0051019
GO:0097472

ults of the GO and KEGG pathway enrichment
esults of molecular function in GO analysis

Description	GeneRatio
protein serine/threonine kinase activity	25/87
MAP kinase activity	6/87
protein serine/threonine/tyrosine kinase activity	6/87
1-phosphatidylinositol-3-kinase activity	4/87
phosphatidylinositol 3-kinase activity	4/87
protein tyrosine kinase activity	8/87
phosphatase binding	9/87
oxidoreductase activity, acting on paired donors, with incorporation or reduction of molecular oxygen,	5/87
phosphatidylinositol phosphate kinase activity	4/87
phosphatidylinositol kinase activity	4/87
steroid hydroxylase activity	5/87
non-membrane spanning protein tyrosine kinase activity	5/87
protein phosphatase binding	7/87
3',5'-cyclic-nucleotide phosphodiesterase activity	4/87
hormone binding	6/87
cyclic-nucleotide phosphodiesterase activity	4/87
insulin receptor substrate binding	3/87
heme binding	6/87
MAP kinase kinase activity	3/87
histone kinase activity	3/87
tetrapyrrole binding	6/87
phosphoric diester hydrolase activity	5/87
peptide receptor activity	6/87
iron ion binding	6/87
monooxygenase activity	5/87
G protein-coupled amine receptor activity	4/87
aspartic-type endopeptidase activity	3/87
aspartic-type peptidase activity	3/87
G protein-coupled serotonin receptor activity	3/87
serotonin receptor activity	3/87
oxygen binding	3/87
cyclic nucleotide binding	3/87
SH2 domain binding	3/87
phosphoprotein binding	4/87
G protein-coupled peptide receptor activity	5/87
G protein-coupled neurotransmitter receptor activity	3/87
oxidoreductase activity, acting on paired donors, with incorporation or reduction of molecular oxygen	5/87
3',5'-cyclic-AMP phosphodiesterase activity	2/87
peptide hormone binding	3/87
oxidoreductase activity, acting on CH or CH2 groups	2/87
neuropeptide receptor activity	3/87
phosphoric ester hydrolase activity	7/87
cGMP binding	2/87
neurotransmitter receptor activity	4/87
arachidonic acid epoxygenase activity	2/87

scaffold protein binding	3/87
arachidonic acid monooxygenase activity	2/87
cell adhesion molecule binding	8/87
3',5'-cyclic-GMP phosphodiesterase activity	2/87
transmembrane receptor protein tyrosine kinase activity	3/87
integrin binding	4/87
growth factor receptor binding	4/87
postsynaptic neurotransmitter receptor activity	3/87
endopeptidase activity	7/87
acetylcholine receptor activity	2/87
cAMP binding	2/87
transmembrane receptor protein kinase activity	3/87
mitogen-activated protein kinase binding	2/87
cyclin-dependent protein kinase activity	2/87

BgRatio	pvalue	p.adjust	qvalue
439/17696	3.90E-20	1.16E-17	8.37E-18
14/17696	3.45E-11	5.12E-09	3.70E-09
43/17696	6.25E-08	6.18E-06	4.47E-06
10/17696	1.12E-07	8.31E-06	6.01E-06
11/17696	1.75E-07	1.04E-05	7.52E-06
134/17696	3.10E-07	1.32E-05	9.58E-06
185/17696	3.12E-07	1.32E-05	9.58E-06
32/17696	4.64E-07	1.72E-05	1.24E-05
16/17696	9.48E-07	2.82E-05	2.04E-05
16/17696	9.48E-07	2.82E-05	2.04E-05
38/17696	1.13E-06	3.05E-05	2.21E-05
46/17696	2.99E-06	7.40E-05	5.35E-05
140/17696	5.75E-06	0.000131458	9.50E-05
28/17696	1.02E-05	0.000216308	0.00015639
102/17696	1.09E-05	0.000216737	0.00015671
29/17696	1.18E-05	0.000218731	0.00015815
11/17696	1.84E-05	0.000321582	0.00023251
135/17696	5.37E-05	0.000885426	0.00064018
16/17696	6.14E-05	0.000959313	0.0006936
17/17696	7.43E-05	0.001102705	0.00079728
145/17696	7.99E-05	0.001129372	0.00081656
94/17696	9.95E-05	0.001282125	0.000927
152/17696	0.00010361	0.001282125	0.000927
152/17696	0.00010361	0.001282125	0.000927
99/17696	0.00012723	0.001511542	0.00109287
59/17696	0.00020181	0.002305342	0.00166681
25/17696	0.00024412	0.002685305	0.00194153
26/17696	0.00027498	0.002916771	0.00210888
34/17696	0.00061517	0.006090158	0.0044033
34/17696	0.00061517	0.006090158	0.0044033
36/17696	0.00072882	0.006501533	0.00470074
36/17696	0.00072882	0.006501533	0.00470074
36/17696	0.00072882	0.006501533	0.00470074
83/17696	0.00074505	0.006501533	0.00470074
146/17696	0.00076617	0.006501533	0.00470074
41/17696	0.00106902	0.008819451	0.00637664
159/17696	0.00112307	0.009014928	0.00651797
12/17696	0.00152731	0.011937144	0.00863079
49/17696	0.00179607	0.01335939	0.0096591
13/17696	0.00179924	0.01335939	0.0096591
50/17696	0.00190397	0.013792175	0.00997201
367/17696	0.00219796	0.015542699	0.01123768
15/17696	0.00240664	0.016622573	0.01201845
117/17696	0.00264891	0.017880141	0.01292769
16/17696	0.00274167	0.018095042	0.01308307

59/17696	0.00305919	0.019465242	0.01407375
17/17696	0.00309733	0.019465242	0.01407375
499/17696	0.0031459	0.019465242	0.01407375
18/17696	0.0034734	0.020917843	0.01512401
62/17696	0.00352152	0.020917843	0.01512401
132/17696	0.00408372	0.023781677	0.01719462
134/17696	0.00430789	0.024604655	0.01778965
70/17696	0.00495537	0.027768763	0.02007736
427/17696	0.00505326	0.027792915	0.02009482
23/17696	0.0056528	0.02998001	0.02167614
23/17696	0.0056528	0.02998001	0.02167614
79/17696	0.00693569	0.036138594	0.02612891
26/17696	0.00719251	0.036830596	0.02662925
30/17696	0.00950529	0.047848678	0.03459554

geneID
MAPKAPK2/MAPK10/AKT2/EGFR/CDK2/MAPK11/CHEK1/MAPK9/MAPK14/TNK2/PIK3CG/PIK3CA/SYK
MAPK10/MAPK11/MAPK9/MAPK14/MAPK1/MAPK8
MAPK10/MAPK9/MAPK14/TNK2/AKT1/MAPK1
PIK3CB/PIK3CG/PIK3CA/PIK3CD
PIK3CB/PIK3CG/PIK3CA/PIK3CD
NTRK1/JAK2/EGFR/TNK2/SYK/JAK1/CSF1R/PTK2
EGFR/MAPK14/SYK/JAK1/AKT1/CSF1R/PTK2/MAPK1/PPP1CC
CYP2D6/CYP19A1/CYP3A4/CYP2C19/CYP2C9
PIK3CB/PIK3CG/PIK3CA/PIK3CD
PIK3CB/PIK3CG/PIK3CA/PIK3CD
CYP2D6/CYP19A1/CYP3A4/CYP2C19/CYP2C9
JAK2/TNK2/SYK/JAK1/PTK2
EGFR/MAPK14/JAK1/AKT1/CSF1R/PTK2/PPP1CC
PDE5A/PDE10A/PDE4B/PDE3B
HCRTR2/HCRTR1/CRHR1/EGFR/MTNR1A/VDR
PDE5A/PDE10A/PDE4B/PDE3B
PIK3CB/JAK2/PIK3CA
CYP2D6/CYP19A1/JAK2/CYP3A4/CYP2C19/CYP2C9
MAPK10/MAPK14/MAPK1
JAK2/CDK2/CHEK1
CYP2D6/CYP19A1/JAK2/CYP3A4/CYP2C19/CYP2C9
CHRM1/PDE5A/PDE10A/PDE4B/PDE3B
HCRTR2/TACR1/HCRTR1/CRHR1/NPY5R/AVPR2
CYP2D6/CYP19A1/CYP3A4/CYP2C19/CYP2C9/ALOX5
CYP2D6/CYP19A1/CYP3A4/CYP2C19/CYP2C9
CHRM1/HTR1A/DRD2/CHRM2
PSEN1/PSEN2/REN
PSEN1/PSEN2/REN
CHRM1/HTR1A/CHRM2
CHRM1/HTR1A/CHRM2
CYP19A1/CYP3A4/CYP2C19
PDE5A/PDE10A/PDE4B
JAK2/SYK/PTK2
TRPV1/SYK/MAPK1/MTOR
HCRTR2/TACR1/HCRTR1/NPY5R/AVPR2
CHRM1/HTR1A/CHRM2
CYP2D6/CYP19A1/CYP3A4/CYP2C19/CYP2C9
PDE4B/PDE3B
HCRTR2/HCRTR1/CRHR1
CYP3A4/CYP2C9
HCRTR2/TACR1/NPY5R
CHRM1/PDE5A/PDE10A/PPP2CA/PDE4B/PDE3B/PPP1CC
PDE5A/PDE10A
CHRM1/HTR1A/DRD2/CHRM2
CYP2C19/CYP2C9

MDM2/IKBKB/KCNH2
CYP2C19/CYP2C9
ITGB2/EGFR/ITGAL/SYK/PSEN1/MARK2/FASN/ICAM1
PDE5A/PDE10A
NTRK1/EGFR/CSF1R
ITGB2/EGFR/SYK/ICAM1
JAK2/TNK2/IL6ST/APP
CHRM1/DRD2/CHRM2
NCSTN/APH1A/PSEN1/F2/PSEN2/APH1B/REN
CHRM1/CHRM2
PDE10A/PDE4B
NTRK1/EGFR/CSF1R
MAPKAPK2/MAPK14
CCNA2/CDK2

Count
25
6
6
4
4
8
9
5
4
4
5
5
7
4
6
4
3
6
3
3
6
5
6
6
5
4
3
3
3
3
3
3
3
4
5
3
5
2
3
2
3
7
2
4
2

3
2
8
2
3
4
4
3
7
2
2
3
2
2

Table. S4 The results of the GO and KEGG pathway enrichment

Table. S4-4 The results of KEGG pathway enrichment analysis

GO	Description	LogP	Enrichme	Z-score	#TotalGe
hsa04068	FoxO signaling pathway	-25	40	27	24156
hsa04722	Neurotrophin signaling pathway	-22	40	25	24156
hsa04914	Progesterone-mediated oocyte maturation	-22	46	27	24156
hsa01522	Endocrine resistance	-22	46	27	24156
hsa05169	Epstein-Barr virus infection	-21	26	22	24156
hsa04664	Fc epsilon RI signaling pathway	-21	57	28	24156
hsa05160	Hepatitis C	-20	34	23	24156
hsa05200	Pathways in cancer	-20	15	17	24156
hsa04151	PI3K-Akt signaling pathway	-20	17	18	24156
hsa05145	Toxoplasmosis	-19	37	23	24156
hsa04080	Neuroactive ligand-receptor interaction	-19	19	18	24156
hsa04024	cAMP signaling pathway	-18	24	19	24156
hsa04933	AGE-RAGE signaling pathway in diabetic complicatior	-18	39	23	24156
hsa04380	Osteoclast differentiation	-18	32	21	24156
hsa04910	Insulin signaling pathway	-18	30	21	24156
hsa05212	Pancreatic cancer	-17	52	25	24156
hsa04071	Sphingolipid signaling pathway	-17	33	21	24156
hsa04917	Prolactin signaling pathway	-17	48	23	24156
hsa05142	Chagas disease (American trypanosomiasis)	-16	35	21	24156
hsa05164	Influenza A	-16	24	18	24156
hsa04668	TNF signaling pathway	-16	33	20	24156
hsa05161	Hepatitis B	-16	27	19	24156
hsa04012	ErbB signaling pathway	-16	39	21	24156
hsa05215	Prostate cancer	-16	38	21	24156
hsa05205	Proteoglycans in cancer	-15	21	17	24156
hsa05231	Choline metabolism in cancer	-15	34	20	24156
hsa04210	Apoptosis	-15	26	18	24156
hsa04620	Toll-like receptor signaling pathway	-15	32	19	24156
hsa05152	Tuberculosis	-15	22	17	24156
hsa05418	Fluid shear stress and atherosclerosis	-14	25	18	24156
hsa04931	Insulin resistance	-14	31	19	24156
hsa05221	Acute myeloid leukemia	-14	50	22	24156
hsa04370	VEGF signaling pathway	-14	47	21	24156
hsa04510	Focal adhesion	-14	20	16	24156
hsa04015	Rap1 signaling pathway	-14	19	15	24156
hsa04140	Autophagy - animal	-13	26	17	24156
hsa04750	Inflammatory mediator regulation of TRP channels	-13	31	18	24156
hsa04930	Type II diabetes mellitus	-13	54	22	24156
hsa04660	T cell receptor signaling pathway	-13	30	18	24156
hsa04659	Th17 cell differentiation	-13	29	17	24156
hsa01521	EGFR tyrosine kinase inhibitor resistance	-13	35	18	24156
hsa05203	Viral carcinogenesis	-12	18	15	24156
hsa04152	AMPK signaling pathway	-12	25	16	24156
hsa04611	Platelet activation	-12	25	16	24156
hsa05210	Colorectal cancer	-12	42	19	24156

hsa05214	Glioma	-12	39	18	24156
hsa04014	Ras signaling pathway	-12	16	14	24156
hsa05230	Central carbon metabolism in cancer	-12	38	18	24156
hsa04550	Signaling pathways regulating pluripotency of stem cell	-12	22	15	24156
hsa04072	Phospholipase D signaling pathway	-11	21	15	24156
hsa01524	Platinum drug resistance	-11	34	17	24156
hsa04010	MAPK signaling pathway	-11	14	13	24156
hsa05166	HTLV-I infection	-11	14	13	24156
hsa04725	Cholinergic synapse	-11	25	15	24156
hsa05213	Endometrial cancer	-11	44	18	24156
hsa04810	Regulation of actin cytoskeleton	-11	16	13	24156
hsa05222	Small cell lung cancer	-11	30	16	24156
hsa05223	Non-small cell lung cancer	-10	38	17	24156
hsa04728	Dopaminergic synapse	-10	21	14	24156
hsa04658	Th1 and Th2 cell differentiation	-10	27	15	24156
hsa04920	Adipocytokine signaling pathway	-9.8	32	16	24156
hsa05218	Melanoma	-9.8	32	16	24156
hsa05220	Chronic myeloid leukemia	-9.7	31	15	24156
hsa04662	B cell receptor signaling pathway	-9.7	31	15	24156
hsa04630	Jak-STAT signaling pathway	-9.6	18	13	24156
hsa04670	Leukocyte transendothelial migration	-9.5	22	13	24156
hsa04919	Thyroid hormone signaling pathway	-9.4	22	13	24156
hsa04621	NOD-like receptor signaling pathway	-9.2	16	12	24156
hsa05162	Measles	-8.9	19	12	24156
hsa04261	Adrenergic signaling in cardiomyocytes	-8.6	17	12	24156
hsa04066	HIF-1 signaling pathway	-8.5	22	13	24156
hsa04932	Non-alcoholic fatty liver disease (NAFLD)	-8.5	17	12	24156
hsa05131	Shigellosis	-8.4	30	14	24156
hsa04150	mTOR signaling pathway	-8.4	17	12	24156
hsa05120	Epithelial cell signaling in Helicobacter pylori infection	-8.3	29	14	24156
hsa05133	Pertussis	-8	26	13	24156
hsa04062	Chemokine signaling pathway	-7.7	14	10	24156
hsa04330	Notch signaling pathway	-7.7	35	14	24156
hsa04650	Natural killer cell mediated cytotoxicity	-7.6	17	11	24156
hsa04666	Fc gamma R-mediated phagocytosis	-7.4	21	12	24156
hsa04923	Regulation of lipolysis in adipocytes	-7.4	31	13	24156
hsa04912	GnRH signaling pathway	-7.4	21	12	24156
hsa04657	IL-17 signaling pathway	-7.3	21	12	24156
hsa05224	Breast cancer	-7.3	15	10	24156
hsa04915	Estrogen signaling pathway	-7.2	20	11	24156
hsa04213	Longevity regulating pathway - multiple species	-7	27	12	24156
hsa05211	Renal cell carcinoma	-6.9	26	12	24156
hsa04726	Serotonergic synapse	-6.8	17	10	24156
hsa05010	Alzheimer's disease	-6.7	13	9.5	24156
hsa04622	RIG-I-like receptor signaling pathway	-6.7	24	11	24156
hsa04960	Aldosterone-regulated sodium reabsorption	-6.7	38	13	24156
hsa05140	Leishmaniasis	-6.6	23	11	24156

hsa04110	Cell cycle	-6.5	16	9.8	24156
hsa05168	Herpes simplex infection	-6.5	12	9	24156
hsa04973	Carbohydrate digestion and absorption	-6.3	32	12	24156
hsa05132	Salmonella infection	-6.2	19	10	24156
hsa04211	Longevity regulating pathway	-6.1	19	10	24156
hsa04723	Retrograde endocannabinoid signaling	-5.8	16	9.4	24156
hsa04022	cGMP-PKG signaling pathway	-5.7	12	8.4	24156
hsa00591	Linoleic acid metabolism	-5.5	38	12	24156
hsa04020	Calcium signaling pathway	-5.4	11	7.9	24156
hsa04215	Apoptosis - multiple species	-5.2	34	11	24156
hsa04371	Apelin signaling pathway	-5	12	7.8	24156
hsa05206	MicroRNAs in cancer	-4.9	7.4	6.7	24156
hsa05146	Amoebiasis	-4.6	14	7.9	24156
hsa04360	Axon guidance	-4.4	9.5	6.8	24156
hsa00590	Arachidonic acid metabolism	-4.1	18	8	24156
hsa04114	Oocyte meiosis	-4.1	11	6.8	24156
hsa00982	Drug metabolism - cytochrome P450	-3.9	16	7.5	24156
hsa00562	Inositol phosphate metabolism	-3.9	16	7.4	24156
hsa05100	Bacterial invasion of epithelial cells	-3.8	15	7.1	24156
hsa04921	Oxytocin signaling pathway	-3.6	9.1	6	24156
hsa04064	NF-kappa B signaling pathway	-3.4	12	6.3	24156
hsa05219	Bladder cancer	-3.4	20	7.4	24156
hsa05202	Transcriptional misregulation in cancer	-3.3	7.7	5.4	24156
hsa05144	Malaria	-3.1	17	6.7	24156
hsa04270	Vascular smooth muscle contraction	-3	9.2	5.4	24156
hsa05150	Staphylococcus aureus infection	-3	15	6.2	24156
hsa05416	Viral myocarditis	-2.9	14	6.1	24156
hsa04730	Long-term depression	-2.9	14	6	24156
hsa04137	Mitophagy - animal	-2.8	13	5.7	24156
hsa04310	Wnt signaling pathway	-2.7	7.8	4.9	24156
hsa04115	p53 signaling pathway	-2.7	12	5.5	24156
hsa00980	Metabolism of xenobiotics by cytochrome P450	-2.6	11	5.3	24156
hsa05204	Chemical carcinogenesis	-2.5	10	5	24156
hsa04530	Tight junction	-2.5	6.5	4.4	24156
hsa00230	Purine metabolism	-2.4	6.4	4.3	24156
hsa04540	Gap junction	-2.4	9.5	4.8	24156
hsa05034	Alcoholism	-2.4	6.2	4.2	24156
hsa05323	Rheumatoid arthritis	-2.4	9.3	4.7	24156
hsa05032	Morphine addiction	-2.4	9.2	4.7	24156
hsa04713	Circadian entrainment	-2.3	8.7	4.5	24156
hsa04070	Phosphatidylinositol signaling system	-2.3	8.6	4.5	24156
hsa04922	Glucagon signaling pathway	-2.2	8.1	4.3	24156

#GeneInG	#GeneInH	#GeneInG	%InGO	STDV %I	GeneID
132	87	19	22	4.4	207|208|1017|1263|1432|1901|1956|3551|4193|5170|5290|52
119	87	17	20	4.3	207|208|1432|3551|4914|5170|5290|5291|5293|5594|5599|56
96	87	16	18	4.2	207|208|890|1017|1432|5140|5290|5291|5293|5347|5594|559
96	87	16	18	4.2	207|208|1432|1565|1956|2475|4193|5290|5291|5293|5594|55
201	87	19	22	4.4	207|208|890|1017|1432|3383|3551|3683|3716|4193|5290|529
68	87	14	16	3.9	207|208|240|1432|5170|5290|5291|5293|5594|5599|5600|560
131	87	16	18	4.2	207|208|1432|1956|3551|3716|5170|5290|5291|5293|5515|55
395	87	22	25	4.7	207|208|331|1017|1436|1956|2475|3551|3716|4193|4914|529
342	87	21	24	4.6	207|208|1017|1128|1129|1436|1956|2475|3551|3716|3717|41
113	87	15	17	4	207|208|240|331|1432|3551|3716|3717|5170|5294|5594|5599
277	87	19	22	4.4	135|554|1128|1129|1269|1394|1813|1901|2147|3061|3062|33
198	87	17	20	4.3	135|207|208|1128|1129|1813|3350|5140|5142|5290|5291|529
99	87	14	16	3.9	207|208|1432|3383|3717|5290|5291|5292|5293|5594|5599|56
130	87	15	17	4	207|208|1432|1436|3551|3716|5290|5291|5293|5594|5599|56
138	87	15	17	4	207|208|2194|2475|3551|5140|5170|5290|5291|5293|5501|55
64	87	12	14	3.7	207|208|1956|3551|3716|5290|5291|5293|5594|5599|5601|56
118	87	14	16	3.9	207|208|1432|1901|5170|5290|5291|5293|5515|5594|5599|56
70	87	12	14	3.7	207|208|1432|3717|5290|5291|5293|5594|5599|5600|5601|56
102	87	13	15	3.8	207|208|1432|3551|5290|5291|5293|5515|5594|5599|5600|56
173	87	15	17	4	207|208|1432|3383|3551|3716|3717|5290|5291|5293|5594|55
108	87	13	15	3.8	207|208|1432|3383|3551|5290|5291|5293|5594|5599|5600|56
144	87	14	16	3.9	207|208|890|1017|3551|3716|5290|5291|5293|5594|5599|560
86	87	12	14	3.7	207|208|1956|2475|5290|5291|5293|5594|5599|5601|5602|57
87	87	12	14	3.7	207|208|1017|1956|2475|3551|4193|5170|5290|5291|5293|55
203	87	15	17	4	207|208|1432|1956|2475|4193|5170|5290|5291|5293|5501|55
99	87	12	14	3.7	207|208|1956|2475|5170|5290|5291|5293|5594|5599|5601|56
138	87	13	15	3.8	207|208|331|3551|4914|5170|5290|5291|5293|5594|5599|560
104	87	12	14	3.7	207|208|1432|3551|5290|5291|5293|5594|5599|5600|5601|56
179	87	14	16	3.9	207|208|1263|1432|3689|3716|3717|5594|5599|5600|5601|56
142	87	13	15	3.8	207|208|1432|3383|3551|5290|5291|5293|5599|5600|5601|56
107	87	12	14	3.7	207|208|2475|3551|5170|5290|5291|5293|5501|5599|5601|56
55	87	10	11	3.4	207|208|2475|3551|5290|5291|5292|5293|5594|8900
59	87	10	11	3.4	207|208|1432|5290|5291|5293|5594|5600|5747|9261
199	87	14	16	3.9	207|208|331|1956|5170|5290|5291|5293|5501|5594|5599|560
210	87	14	16	3.9	135|207|208|1432|1436|1813|1956|3683|3689|5290|5291|529
128	87	12	14	3.7	207|208|2475|5170|5290|5291|5293|5515|5594|5599|5601|56
97	87	11	13	3.6	1432|4914|5290|5291|5293|5501|5599|5600|5601|5602|7442
46	87	9	10	3.3	2475|3551|5290|5291|5293|5594|5599|5601|5602
103	87	11	13	3.6	207|208|1432|3551|5170|5290|5291|5293|5594|5600|5601
107	87	11	13	3.6	1432|2475|3551|3572|3716|3717|5594|5599|5600|5601|5602
79	87	10	11	3.4	207|208|1956|2475|3716|3717|5290|5291|5293|5594
201	87	13	15	3.8	890|1017|1111|3572|3716|4193|5290|5291|5293|5594|6850|8
120	87	11	13	3.6	207|208|890|2194|2475|5170|5290|5291|5293|5515|8900
123	87	11	13	3.6	207|208|1432|5290|5291|5293|5294|5501|5594|5600|6850
60	87	9	10	3.3	207|208|5290|5291|5293|5594|5599|5601|5602

64	87	9	10	3.3	207|208|1956|2475|4193|5290|5291|5293|5594
227	87	13	15	3.8	207|208|1436|1956|3551|5290|5291|5293|5320|5594|5599|56[illegible]
65	87	9	10	3.3	207|208|1956|2475|4914|5290|5291|5293|5594
139	87	11	13	3.6	207|208|1432|3572|3716|3717|5290|5291|5293|5594|5600
146	87	11	13	3.6	207|208|554|1956|2475|5290|5291|5293|5294|5594|6850
73	87	9	10	3.3	207|208|331|4193|5170|5290|5291|5293|5594
255	87	13	15	3.8	207|208|781|1432|1956|3551|4914|5594|5599|5600|5601|56[illegible]
256	87	13	15	3.8	207|208|331|1111|3383|3551|3683|3689|3716|5290|5291|52[illegible]
112	87	10	11	3.4	207|208|1128|1129|3717|5290|5291|5293|5294|5594
50	87	8	9.2	3.1	207|208|1956|5170|5290|5291|5293|5594
212	87	12	14	3.7	1128|1129|1956|2147|3683|3689|5290|5291|5293|5501|5594
84	87	9	10	3.3	207|208|331|1017|3551|5290|5291|5293|5747
58	87	8	9.2	3.1	207|208|1956|5170|5290|5291|5293|5594
130	87	10	11	3.4	207|208|1432|1813|5501|5515|5599|5600|5601|5602
92	87	9	10	3.3	1432|3551|3716|3717|5594|5599|5600|5601|5602
69	87	8	9.2	3.1	207|208|2475|3551|3717|5599|5601|5602
69	87	8	9.2	3.1	207|208|1956|4193|5290|5291|5293|5594
71	87	8	9.2	3.1	207|208|3551|4193|5290|5291|5293|5594
71	87	8	9.2	3.1	207|208|3551|5290|5291|5293|5594|6850
156	87	10	11	3.4	207|208|2475|3572|3716|3717|5290|5291|5292|5293
114	87	9	10	3.3	1432|3383|3683|3689|5290|5291|5293|5600|5747
116	87	9	10	3.3	207|208|2475|4193|5170|5290|5291|5293|5594
170	87	10	11	3.4	331|1432|3551|3716|5027|5594|5599|5600|5601|5602
134	87	9	10	3.3	207|208|1017|3716|3717|5290|5291|5293|6869
144	87	9	10	3.3	207|208|781|1432|5294|5501|5515|5594|5600
101	87	8	9.2	3.1	207|208|1956|2475|5290|5291|5293|5594
149	87	9	10	3.3	207|208|3551|5290|5291|5293|5599|5601|5602
65	87	7	8	2.9	1432|3551|5594|5599|5600|5601|5602
151	87	9	10	3.3	207|208|2475|3551|5170|5290|5291|5293|5594
68	87	7	8	2.9	1432|1956|3551|5599|5600|5601|5602
76	87	7	8	2.9	1432|3689|5594|5599|5600|5601|5602
182	87	9	10	3.3	207|208|3551|3717|5290|5291|5293|5594|5747
48	87	6	6.9	2.7	5663|5664|23385|51107|55851|83464
133	87	8	9.2	3.1	3383|3683|3689|5290|5291|5293|5594|6850
91	87	7	8	2.9	207|208|5290|5291|5293|5594|6850
54	87	6	6.9	2.7	207|208|5140|5290|5291|5293
92	87	7	8	2.9	1432|1956|5594|5599|5600|5601|5602
93	87	7	8	2.9	1432|3551|5594|5599|5600|5601|5602
144	87	8	9.2	3.1	207|208|1956|2475|5290|5291|5293|5594
98	87	7	8	2.9	207|208|1956|5290|5291|5293|5594
62	87	6	6.9	2.7	207|208|2475|5290|5291|5293
65	87	6	6.9	2.7	207|208|5290|5291|5293|5594
113	87	7	8	2.9	240|351|1557|1559|1565|3350|5594
171	87	8	9.2	3.1	351|5594|5663|5664|23385|51107|55851|83464
70	87	6	6.9	2.7	1432|3551|5599|5600|5601|5602
37	87	5	5.7	2.5	5170|5290|5291|5293|5594
73	87	6	6.9	2.7	1432|3689|3716|3717|5594|5600

124	87	7	8	2.9	890|1017|1111|4193|5347|5591|8900
185	87	8	9.2	3.1	1017|3551|3716|3717|5501|5599|5601|5602
44	87	5	5.7	2.5	207|208|5290|5291|5293
86	87	6	6.9	2.7	1432|5594|5599|5600|5601|5602
89	87	6	6.9	2.7	207|208|2475|5290|5291|5293
101	87	6	6.9	2.7	1432|5594|5599|5600|5601|5602
163	87	7	8	2.9	207|208|5140|5294|5501|5594|8654
29	87	4	4.6	2.2	1557|1559|1576|5320
182	87	7	8	2.9	135|1128|1129|1956|5027|5731|6869
33	87	4	4.6	2.2	331|5599|5601|5602
138	87	6	6.9	2.7	207|208|2475|5140|5294|5594
299	87	8	9.2	3.1	1956|2475|3551|4193|5243|5290|5292|5594
96	87	5	5.7	2.5	3689|5290|5291|5293|5747
175	87	6	6.9	2.7	5290|5291|5293|5594|5747|6608
62	87	4	4.6	2.2	240|1557|1559|5320
124	87	5	5.7	2.5	1017|5347|5501|5515|5594
70	87	4	4.6	2.2	1557|1559|1565|1576
71	87	4	4.6	2.2	5290|5291|5293|5294
76	87	4	4.6	2.2	5290|5291|5293|5747
152	87	5	5.7	2.5	781|1956|5294|5501|5594
95	87	4	4.6	2.2	331|3383|3551|6850
41	87	3	3.4	2	1956|4193|5594
180	87	5	5.7	2.5	1436|4193|4914|5747|8900
49	87	3	3.4	2	3383|3683|3689
121	87	4	4.6	2.2	135|5320|5501|5594
56	87	3	3.4	2	3383|3683|3689
59	87	3	3.4	2	3383|3683|3689
60	87	3	3.4	2	1394|5515|5594
65	87	3	3.4	2	5599|5601|5602
143	87	4	4.6	2.2	5599|5601|5602|5663
69	87	3	3.4	2	1017|1111|4193
74	87	3	3.4	2	1559|1565|1576
82	87	3	3.4	2	1557|1559|1576
170	87	4	4.6	2.2	5515|5599|5601|5602
174	87	4	4.6	2.2	5140|5142|8654|10846
88	87	3	3.4	2	1813|1956|5594
180	87	4	4.6	2.2	135|1813|5501|5594
90	87	3	3.4	2	3383|3683|3689
91	87	3	3.4	2	5140|5142|10846
96	87	3	3.4	2	4543|4544|5594
97	87	3	3.4	2	5290|5291|5293
103	87	3	3.4	2	207|208|5140

Hits
AKT1|AKT2|CDK2|PLK3|MAPK14|S1PR1|EGFR|IKBKB|MDM2|PDPK1|PIK3CA|PIK3CB|PIK3CD|PLK1|MAPK
AKT1|AKT2|MAPK14|IKBKB|NTRK1|PDPK1|PIK3CA|PIK3CB|PIK3CD|MAPK1|MAPK8|MAPK11|MAPK9|M
AKT1|AKT2|CCNA2|CDK2|MAPK14|PDE3B|PIK3CA|PIK3CB|PIK3CD|PLK1|MAPK1|MAPK8|MAPK11|MAPK
AKT1|AKT2|MAPK14|CYP2D6|EGFR|MTOR|MDM2|PIK3CA|PIK3CB|PIK3CD|MAPK1|MAPK8|MAPK11|MA
AKT1|AKT2|CCNA2|CDK2|MAPK14|ICAM1|IKBKB|ITGAL|JAK1|MDM2|PIK3CA|PIK3CB|PIK3CD|MAPK8|M
AKT1|AKT2|ALOX5|MAPK14|PDPK1|PIK3CA|PIK3CB|PIK3CD|MAPK1|MAPK8|MAPK11|MAPK9|MAPK10|S
AKT1|AKT2|MAPK14|EGFR|IKBKB|JAK1|PDPK1|PIK3CA|PIK3CB|PIK3CD|PPP2CA|MAPK1|MAPK8|MAPK1
AKT1|AKT2|XIAP|CDK2|CSF1R|EGFR|MTOR|IKBKB|JAK1|MDM2|NTRK1|PIK3CA|PIK3CB|PIK3CD|MAPK1
AKT1|AKT2|CDK2|CHRM1|CHRM2|CSF1R|EGFR|MTOR|IKBKB|JAK1|JAK2|MDM2|PDPK1|PIK3CA|PIK3CB
AKT1|AKT2|ALOX5|XIAP|MAPK14|IKBKB|JAK1|JAK2|PDPK1|PIK3CG|MAPK1|MAPK8|MAPK11|MAPK9|M
ADORA2A|AVPR2|CHRM1|CHRM2|CNR2|CRHR1|DRD2|S1PR1|F2|HCRTR1|HCRTR2|HTR1A|MTNR1A|MT
ADORA2A|AKT1|AKT2|CHRM1|CHRM2|DRD2|HTR1A|PDE3B|PDE4B|PIK3CA|PIK3CB|PIK3CD|PPP1CC|M
AKT1|AKT2|MAPK14|ICAM1|JAK2|PIK3CA|PIK3CB|PIM1|PIK3CD|MAPK1|MAPK8|MAPK11|MAPK9|MAPK
AKT1|AKT2|MAPK14|CSF1R|IKBKB|JAK1|PIK3CA|PIK3CB|PIK3CD|MAPK1|MAPK8|MAPK11|MAPK9|MAP
AKT1|AKT2|FASN|MTOR|IKBKB|PDE3B|PDPK1|PIK3CA|PIK3CB|PIK3CD|PPP1CC|MAPK1|MAPK8|MAPK9|
AKT1|AKT2|EGFR|IKBKB|JAK1|PIK3CA|PIK3CB|PIK3CD|MAPK1|MAPK8|MAPK9|MAPK10
AKT1|AKT2|MAPK14|S1PR1|PDPK1|PIK3CA|PIK3CB|PIK3CD|PPP2CA|MAPK1|MAPK8|MAPK11|MAPK9|M
AKT1|AKT2|MAPK14|JAK2|PIK3CA|PIK3CB|PIK3CD|MAPK1|MAPK8|MAPK11|MAPK9|MAPK10
AKT1|AKT2|MAPK14|IKBKB|PIK3CA|PIK3CB|PIK3CD|PPP2CA|MAPK1|MAPK8|MAPK11|MAPK9|MAPK10
AKT1|AKT2|MAPK14|ICAM1|IKBKB|JAK1|JAK2|PIK3CA|PIK3CB|PIK3CD|MAPK1|MAPK8|MAPK11|MAPK
AKT1|AKT2|MAPK14|ICAM1|IKBKB|PIK3CA|PIK3CB|PIK3CD|MAPK1|MAPK8|MAPK11|MAPK9|MAPK10
AKT1|AKT2|CCNA2|CDK2|IKBKB|JAK1|PIK3CA|PIK3CB|PIK3CD|MAPK1|MAPK8|MAPK9|MAPK10|CCNA1
AKT1|AKT2|EGFR|MTOR|PIK3CA|PIK3CB|PIK3CD|MAPK1|MAPK8|MAPK9|MAPK10|PTK2
AKT1|AKT2|CDK2|EGFR|MTOR|IKBKB|MDM2|PDPK1|PIK3CA|PIK3CB|PIK3CD|MAPK1
AKT1|AKT2|MAPK14|EGFR|MTOR|MDM2|PDPK1|PIK3CA|PIK3CB|PIK3CD|PPP1CC|MAPK1|MAPK11|PTK
AKT1|AKT2|EGFR|MTOR|PDPK1|PIK3CA|PIK3CB|PIK3CD|MAPK1|MAPK8|MAPK9|MAPK10
AKT1|AKT2|XIAP|IKBKB|NTRK1|PDPK1|PIK3CA|PIK3CB|PIK3CD|MAPK1|MAPK8|MAPK9|MAPK10
AKT1|AKT2|MAPK14|IKBKB|PIK3CA|PIK3CB|PIK3CD|MAPK1|MAPK8|MAPK11|MAPK9|MAPK10
AKT1|AKT2|PLK3|MAPK14|ITGB2|JAK1|JAK2|MAPK1|MAPK8|MAPK11|MAPK9|MAPK10|SYK|VDR
AKT1|AKT2|MAPK14|ICAM1|IKBKB|PIK3CA|PIK3CB|PIK3CD|MAPK8|MAPK11|MAPK9|MAPK10|PTK2
AKT1|AKT2|MTOR|IKBKB|PDPK1|PIK3CA|PIK3CB|PIK3CD|PPP1CC|MAPK8|MAPK9|MAPK10
AKT1|AKT2|MTOR|IKBKB|PIK3CA|PIK3CB|PIM1|PIK3CD|MAPK1|CCNA1
AKT1|AKT2|MAPK14|PIK3CA|PIK3CB|PIK3CD|MAPK1|MAPK11|PTK2|MAPKAPK2
AKT1|AKT2|XIAP|EGFR|PDPK1|PIK3CA|PIK3CB|PIK3CD|PPP1CC|MAPK1|MAPK8|MAPK9|MAPK10|PTK2
ADORA2A|AKT1|AKT2|MAPK14|CSF1R|DRD2|EGFR|ITGAL|ITGB2|PIK3CA|PIK3CB|PIK3CD|MAPK1|MAPK
AKT1|AKT2|MTOR|PDPK1|PIK3CA|PIK3CB|PIK3CD|PPP2CA|MAPK1|MAPK8|MAPK9|MAPK10
MAPK14|NTRK1|PIK3CA|PIK3CB|PIK3CD|PPP1CC|MAPK8|MAPK11|MAPK9|MAPK10|TRPV1
MTOR|IKBKB|PIK3CA|PIK3CB|PIK3CD|MAPK1|MAPK8|MAPK9|MAPK10
AKT1|AKT2|MAPK14|IKBKB|PDPK1|PIK3CA|PIK3CB|PIK3CD|MAPK1|MAPK11|MAPK9
MAPK14|MTOR|IKBKB|IL6ST|JAK1|JAK2|MAPK1|MAPK8|MAPK11|MAPK9|MAPK10
AKT1|AKT2|EGFR|MTOR|JAK1|JAK2|PIK3CA|PIK3CB|PIK3CD|MAPK1
CCNA2|CDK2|CHEK1|IL6ST|JAK1|MDM2|PIK3CA|PIK3CB|PIK3CD|MAPK1|SYK|CCNA1|MAPKAPK2
AKT1|AKT2|CCNA2|FASN|MTOR|PDPK1|PIK3CA|PIK3CB|PIK3CD|PPP2CA|CCNA1
AKT1|AKT2|MAPK14|PIK3CA|PIK3CB|PIK3CD|PIK3CG|PPP1CC|MAPK1|MAPK11|SYK
AKT1|AKT2|PIK3CA|PIK3CB|PIK3CD|MAPK1|MAPK8|MAPK9|MAPK10

AKT1|AKT2|EGFR|MTOR|MDM2|PIK3CA|PIK3CB|PIK3CD|MAPK1
AKT1|AKT2|CSF1R|EGFR|IKBKB|PIK3CA|PIK3CB|PIK3CD|PLA2G2A|MAPK1|MAPK8|MAPK9|MAPK10
AKT1|AKT2|EGFR|MTOR|NTRK1|PIK3CA|PIK3CB|PIK3CD|MAPK1
AKT1|AKT2|MAPK14|IL6ST|JAK1|JAK2|PIK3CA|PIK3CB|PIK3CD|MAPK1|MAPK11
AKT1|AKT2|AVPR2|EGFR|MTOR|PIK3CA|PIK3CB|PIK3CD|PIK3CG|MAPK1|SYK
AKT1|AKT2|XIAP|MDM2|PDPK1|PIK3CA|PIK3CB|PIK3CD|MAPK1
AKT1|AKT2|CACNA2D1|MAPK14|EGFR|IKBKB|NTRK1|MAPK1|MAPK8|MAPK11|MAPK9|MAPK10|MAPK[illegible]
AKT1|AKT2|XIAP|CHEK1|ICAM1|IKBKB|ITGAL|ITGB2|JAK1|PIK3CA|PIK3CB|PIK3CD|MAPK8
AKT1|AKT2|CHRM1|CHRM2|JAK2|PIK3CA|PIK3CB|PIK3CD|PIK3CG|MAPK1
AKT1|AKT2|EGFR|PDPK1|PIK3CA|PIK3CB|PIK3CD|MAPK1
CHRM1|CHRM2|EGFR|F2|ITGAL|ITGB2|PIK3CA|PIK3CB|PIK3CD|PPP1CC|MAPK1|PTK2
AKT1|AKT2|XIAP|CDK2|IKBKB|PIK3CA|PIK3CB|PIK3CD|PTK2
AKT1|AKT2|EGFR|PDPK1|PIK3CA|PIK3CB|PIK3CD|MAPK1
AKT1|AKT2|MAPK14|DRD2|PPP1CC|PPP2CA|MAPK8|MAPK11|MAPK9|MAPK10
MAPK14|IKBKB|JAK1|JAK2|MAPK1|MAPK8|MAPK11|MAPK9|MAPK10
AKT1|AKT2|MTOR|IKBKB|JAK2|MAPK8|MAPK9|MAPK10
AKT1|AKT2|EGFR|MDM2|PIK3CA|PIK3CB|PIK3CD|MAPK1
AKT1|AKT2|IKBKB|MDM2|PIK3CA|PIK3CB|PIK3CD|MAPK1
AKT1|AKT2|IKBKB|PIK3CA|PIK3CB|PIK3CD|MAPK1|SYK
AKT1|AKT2|MTOR|IL6ST|JAK1|JAK2|PIK3CA|PIK3CB|PIM1|PIK3CD
MAPK14|ICAM1|ITGAL|ITGB2|PIK3CA|PIK3CB|PIK3CD|MAPK11|PTK2
AKT1|AKT2|MTOR|MDM2|PDPK1|PIK3CA|PIK3CB|PIK3CD|MAPK1
XIAP|MAPK14|IKBKB|JAK1|P2RX7|MAPK1|MAPK8|MAPK11|MAPK9|MAPK10
AKT1|AKT2|CDK2|JAK1|JAK2|PIK3CA|PIK3CB|PIK3CD|TACR1
AKT1|AKT2|CACNA2D1|MAPK14|PIK3CG|PPP1CC|PPP2CA|MAPK1|MAPK11
AKT1|AKT2|EGFR|MTOR|PIK3CA|PIK3CB|PIK3CD|MAPK1
AKT1|AKT2|IKBKB|PIK3CA|PIK3CB|PIK3CD|MAPK8|MAPK9|MAPK10
MAPK14|IKBKB|MAPK1|MAPK8|MAPK11|MAPK9|MAPK10
AKT1|AKT2|MTOR|IKBKB|PDPK1|PIK3CA|PIK3CB|PIK3CD|MAPK1
MAPK14|EGFR|IKBKB|MAPK8|MAPK11|MAPK9|MAPK10
MAPK14|ITGB2|MAPK1|MAPK8|MAPK11|MAPK9|MAPK10
AKT1|AKT2|IKBKB|JAK2|PIK3CA|PIK3CB|PIK3CD|MAPK1|PTK2
PSEN1|PSEN2|NCSTN|APH1A|PSENEN|APH1B
ICAM1|ITGAL|ITGB2|PIK3CA|PIK3CB|PIK3CD|MAPK1|SYK
AKT1|AKT2|PIK3CA|PIK3CB|PIK3CD|MAPK1|SYK
AKT1|AKT2|PDE3B|PIK3CA|PIK3CB|PIK3CD
MAPK14|EGFR|MAPK1|MAPK8|MAPK11|MAPK9|MAPK10
MAPK14|IKBKB|MAPK1|MAPK8|MAPK11|MAPK9|MAPK10
AKT1|AKT2|EGFR|MTOR|PIK3CA|PIK3CB|PIK3CD|MAPK1
AKT1|AKT2|EGFR|PIK3CA|PIK3CB|PIK3CD|MAPK1
AKT1|AKT2|MTOR|PIK3CA|PIK3CB|PIK3CD
AKT1|AKT2|PIK3CA|PIK3CB|PIK3CD|MAPK1
ALOX5|APP|CYP2C19|CYP2C9|CYP2D6|HTR1A|MAPK1
APP|MAPK1|PSEN1|PSEN2|NCSTN|APH1A|PSENEN|APH1B
MAPK14|IKBKB|MAPK8|MAPK11|MAPK9|MAPK10
PDPK1|PIK3CA|PIK3CB|PIK3CD|MAPK1
MAPK14|ITGB2|JAK1|JAK2|MAPK1|MAPK11

CCNA2|CDK2|CHEK1|MDM2|PLK1|PRKDC|CCNA1
CDK2|IKBKB|JAK1|JAK2|PPP1CC|MAPK8|MAPK9|MAPK10
AKT1|AKT2|PIK3CA|PIK3CB|PIK3CD
MAPK14|MAPK1|MAPK8|MAPK11|MAPK9|MAPK10
AKT1|AKT2|MTOR|PIK3CA|PIK3CB|PIK3CD
MAPK14|MAPK1|MAPK8|MAPK11|MAPK9|MAPK10
AKT1|AKT2|PDE3B|PIK3CG|PPP1CC|MAPK1|PDE5A
CYP2C19|CYP2C9|CYP3A4|PLA2G2A
ADORA2A|CHRM1|CHRM2|EGFR|P2RX7|PTGER1|TACR1
XIAP|MAPK8|MAPK9|MAPK10
AKT1|AKT2|MTOR|PDE3B|PIK3CG|MAPK1
EGFR|MTOR|IKBKB|MDM2|ABCB1|PIK3CA|PIM1|MAPK1
ITGB2|PIK3CA|PIK3CB|PIK3CD|PTK2
PIK3CA|PIK3CB|PIK3CD|MAPK1|PTK2|SMO
ALOX5|CYP2C19|CYP2C9|PLA2G2A
CDK2|PLK1|PPP1CC|PPP2CA|MAPK1
CYP2C19|CYP2C9|CYP2D6|CYP3A4
PIK3CA|PIK3CB|PIK3CD|PIK3CG
PIK3CA|PIK3CB|PIK3CD|PTK2
CACNA2D1|EGFR|PIK3CG|PPP1CC|MAPK1
XIAP|ICAM1|IKBKB|SYK
EGFR|MDM2|MAPK1
CSF1R|MDM2|NTRK1|PTK2|CCNA1
ICAM1|ITGAL|ITGB2
ADORA2A|PLA2G2A|PPP1CC|MAPK1
ICAM1|ITGAL|ITGB2
ICAM1|ITGAL|ITGB2
CRHR1|PPP2CA|MAPK1
MAPK8|MAPK9|MAPK10
MAPK8|MAPK9|MAPK10|PSEN1
CDK2|CHEK1|MDM2
CYP2C9|CYP2D6|CYP3A4
CYP2C19|CYP2C9|CYP3A4
PPP2CA|MAPK8|MAPK9|MAPK10
PDE3B|PDE4B|PDE5A|PDE10A
DRD2|EGFR|MAPK1
ADORA2A|DRD2|PPP1CC|MAPK1
ICAM1|ITGAL|ITGB2
PDE3B|PDE4B|PDE10A
MTNR1A|MTNR1B|MAPK1
PIK3CA|PIK3CB|PIK3CD
AKT1|AKT2|PDE3B

Log(q-value)
-22
-20
-20
-20
-19
-19
-18
-18
-18
-17
-17
-17
-17
-17
-16
-16
-16
-15
-15
-15
-15
-15
-14
-14
-14
-14
-13
-13
-13
-13
-13
-13
-13
-13
-12
-12
-12
-12
-12
-12
-12
-11
-11
-11
-11

-11
-11
-11
-11
-10
-10
-10
-10
-10
-10
-9.9
-9.8
-9.5
-9.5
-9.4
-8.9
-8.9
-8.9
-8.9
-8.7
-8.6
-8.6
-8.4
-8
-7.7
-7.7
-7.6
-7.6
-7.6
-7.5
-7.1
-6.9
-6.9
-6.8
-6.6
-6.6
-6.6
-6.6
-6.5
-6.4
-6.3
-6.1
-6
-6
-6
-5.9
-5.9

-5.8
-5.7
-5.6
-5.5
-5.4
-5.1
-5
-4.8
-4.7
-4.5
-4.3
-4.2
-3.9
-3.7
-3.5
-3.4
-3.3
-3.2
-3.1
-3
-2.8
-2.7
-2.7
-2.5
-2.4
-2.3
-2.3
-2.3
-2.2
-2.1
-2.1
-2
-1.9
-1.9
-1.8
-1.8
-1.8
-1.8
-1.8
-1.7
-1.7
-1.6

Table S5 The topological properties of potential targets in PPI network

node_name	Degree	Closeness	Betweenness
APP	26	45.16667	1366.07941
AKT1	22	43.11667	720.92392
PIK3CA	21	43.45	574.53531
MAPK1	19	43.75	786.76902
EGFR	16	42.08333	893.61809
AKT2	16	38.36667	174.25427
PIK3CD	13	37.2	57.30491
JAK1	13	37.66667	142.26043
PIK3CB	13	37.2	57.30491
MTOR	13	37.75	195.07892
CHRM2	12	36.28333	138.09165
MAPK14	12	37	119.40054
PTK2	11	36	194.2585
S1PR1	11	37.86667	271.99224
MAPK8	11	38.41667	352.35192
MDM2	11	34.61667	148.82284
MAPK11	11	35.91667	94.01061
PPP2CA	11	35.5	202.37087
CHRM1	10	35.1	175.70202
TACR1	10	35.78333	64.0372
CNR2	9	31.96667	38.25056
SYK	9	33.28333	68.39869
JAK2	9	34.5	60.38602
PIK3CG	9	33.6	28.32464
PDPK1	9	33.85	13.98443
NPY5R	8	31.46667	0
MTNR1B	8	31.46667	0
MTNR1A	8	31.46667	0
HTR1A	8	31.46667	0
F2	8	35.03333	29.93762
DRD2	8	31.46667	0
CCNA2	8	31.05	35.42725
HCRTR2	7	32.93333	0
PTGER1	7	32.93333	0
HCRTR1	7	32.93333	0
NTRK1	7	34.61667	150.89892
MAPK9	7	34.08333	50.56179
CHEK1	7	28.58333	11.37218
MAPKAPK2	7	31.58333	331.036
MAPK10	6	32.91667	39.17241
IKBKB	6	30.6	17.50248
PLK1	6	26.11667	7.2
NCSTN	6	29.55	26.8
APH1A	6	29.55	26.8
PSEN2	6	29.55	26.8
PSEN1	6	29.55	26.8

PSENEN	6	29.55	26.8
ABCB1	5	30.66667	571.4134
CYP3A4	5	23.95	173.19666
CYP2C19	5	24.28333	51.37938
PRKDC	5	29.55	17.80495
CCNA1	5	25.95	1.87051
CDK2	5	30.11667	34.12744
ICAM1	5	32.5	204.66762
ITGB2	5	27.3	169.46508
APH1B	5	22.56667	0
AVPR2	4	30.03333	142
ALOX5	4	24.46667	227.36456
XIAP	3	28.46667	1.07495
CYP2D6	3	22.95	0
PDE3B	3	27.48333	0
PPP1CC	3	28.78333	22.44345
CYP2C9	3	20.23333	5.33333
TRPV1	2	25.11667	2.2381
FASN	2	27.55	0
IL6ST	2	25.05	0
ITGAL	2	23.41667	0
MARK2	1	23.11667	0
CSF1R	1	19.5	0
PLA2G2A	1	18.01667	0
CRHR1	1	21.1	0
SMO	1	26.15	0
CYP19A1	1	17.65	0
